# Supplementary material for: Nucleosomal DNA has topological memory
Source: Nat Commun. 2024 May 28;15:4526. doi: 10.1038/s41467-024-49023-4 (PMC11133463; doi:10.1038/s41467-024-49023-4)
Supplement: Supplementary file 1 — Supplementary Information [file 41467_2024_49023_MOESM1_ESM.pdf]

Supplementary Information for

Nucleosomal DNA has topological memory

Joana Segura[1,3], Ofelia Díaz-Ingelmo[1], Belén Martínez-García[1], Alba Ayats-Fraile[1], Christoforos Nikolaou[2] & Joaquim Roca[1]*

[1] DNA Topology Lab. Molecular Biology Institute of Barcelona (IBMB-CSIC), Barcelona 08028, Spain.

[2] Computational Genomics Group. BSRC Alexander Fleming, Athens 16672, Greece.

[3] Present address: Centro de Biología Molecular Severo Ochoa (CSIC/UAM), Madrid 28049, Spain

* Correspondence: joaquim.roca@ibmb.csic.es

Supplementary Fig. 1

Supplementary Fig. 2

Supplementary Fig. 3

Supplementary Fig. 4

Supplementary Fig. 5

Supplementary Fig. 6

Supplementary Fig. 7

Supplementary Fig. 8

Supplementary Fig. 9

Supplementary Fig. 10

Supplementary Fig. 11

Supplementary Fig. 12

Supplementary Table 1

a

Adaptor with BamH1 cohesive-end ligatable but not recutable

```
5'    *GGACGATTACAGCTACGTG       3'
3'    TCCTGCTAATGTCGATGCACCTAG   5'
```

Adaptor with Asc1 cohesive-end ligatable but not recutable

```
5'    *CGTTCGGATCCGTTTAAACGTGAAGAGGTAACTCAT     3'
3'    TGCAAGCCTAGGCAAATTTGCACTTCTCCATTGAGTAGCGC 5'
```

b

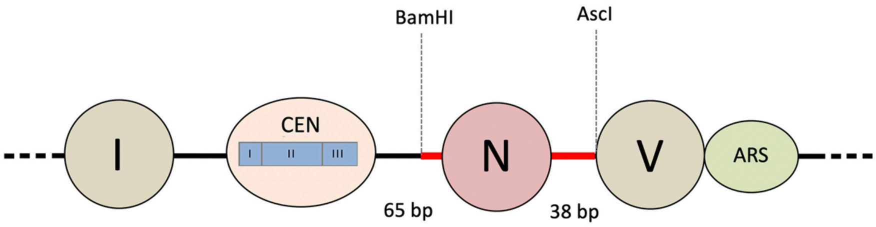

Supplementary Fig. 1. Insertion of the nucleosome library into YCp1.3. a, DNA adaptors ligated to A-tailed nucleosome DNA fragments to generate *BamHI* and *AscI* cohesive ends. **b,** Position of the nucleosome library between nucleosome V and CEN2 of YCp1.3. These two well-structured and positioned elements minimize plausible interferences of the nucleosome library with the functional units of the minichromosome. The lengths (bp) of the resulting linker DNA segments are indicated.

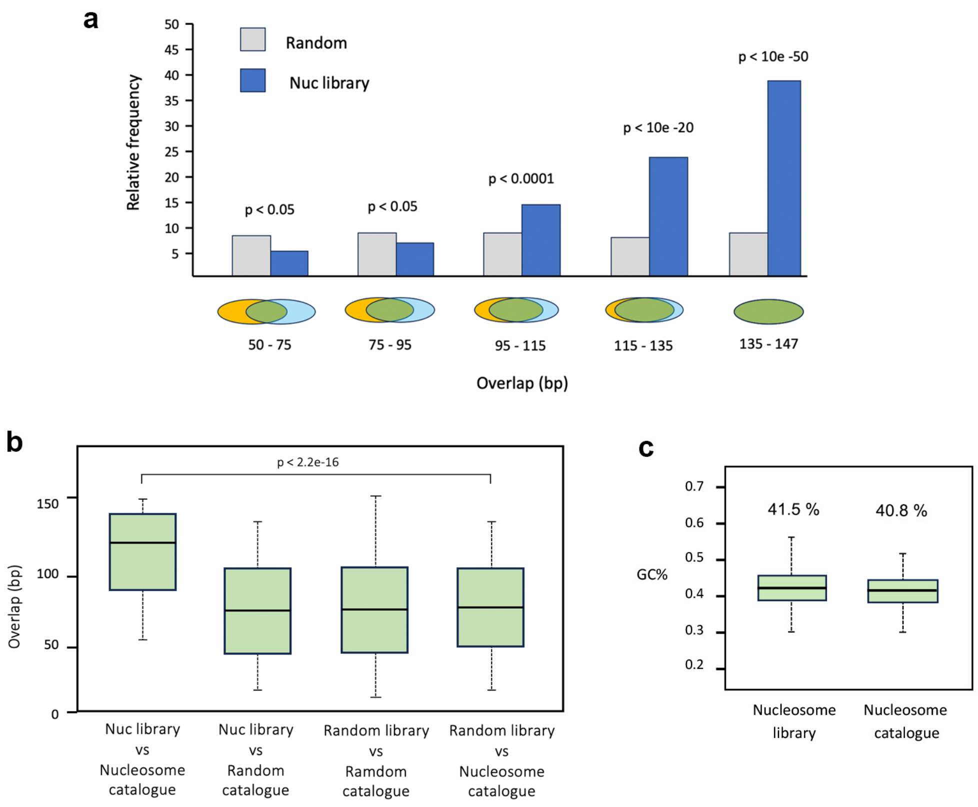

Supplementary Fig. 2. Correspondence of the nucleosome library coordinates with previously referenced nucleosomes. a, Degree of overlap (number of bp) of the nucleosomal DNA library coordinates with those of the catalogue of Jiang and Pugh (2009), which compiled six sets of nucleosome maps generated from different laboratories using different technologies. As a negative control, the overlap of the same number of fragments with randomized coordinates is shown. The indicated *p*-value was determined by an unpaired two-tailed *t*-test. **b**, Comparison of the overlapping (number of bp) of the nucleosome library with the reference catalogue of nucleosomes, and with randomized coordinates in the genome (n=8369). As negative controls, the overlap of a random library of coordinates with randomized coordinates in the genome and with the reference catalogue of nucleosomes are shown. The indicated *p*-value was determined by an unpaired two-tailed *t*-test. **c**, GC-content (%) of the nucleosomal DNA library and that of the full catalogue of nucleosomes (n=8369). In the box plots (b, c), the centre line denotes the median value, while the box contains the 25th (upper limit) to 75th (lower limit) percentiles of the dataset. The black whiskers mark the 5th and 95th percentiles. Source data are provided as a Source Data file.

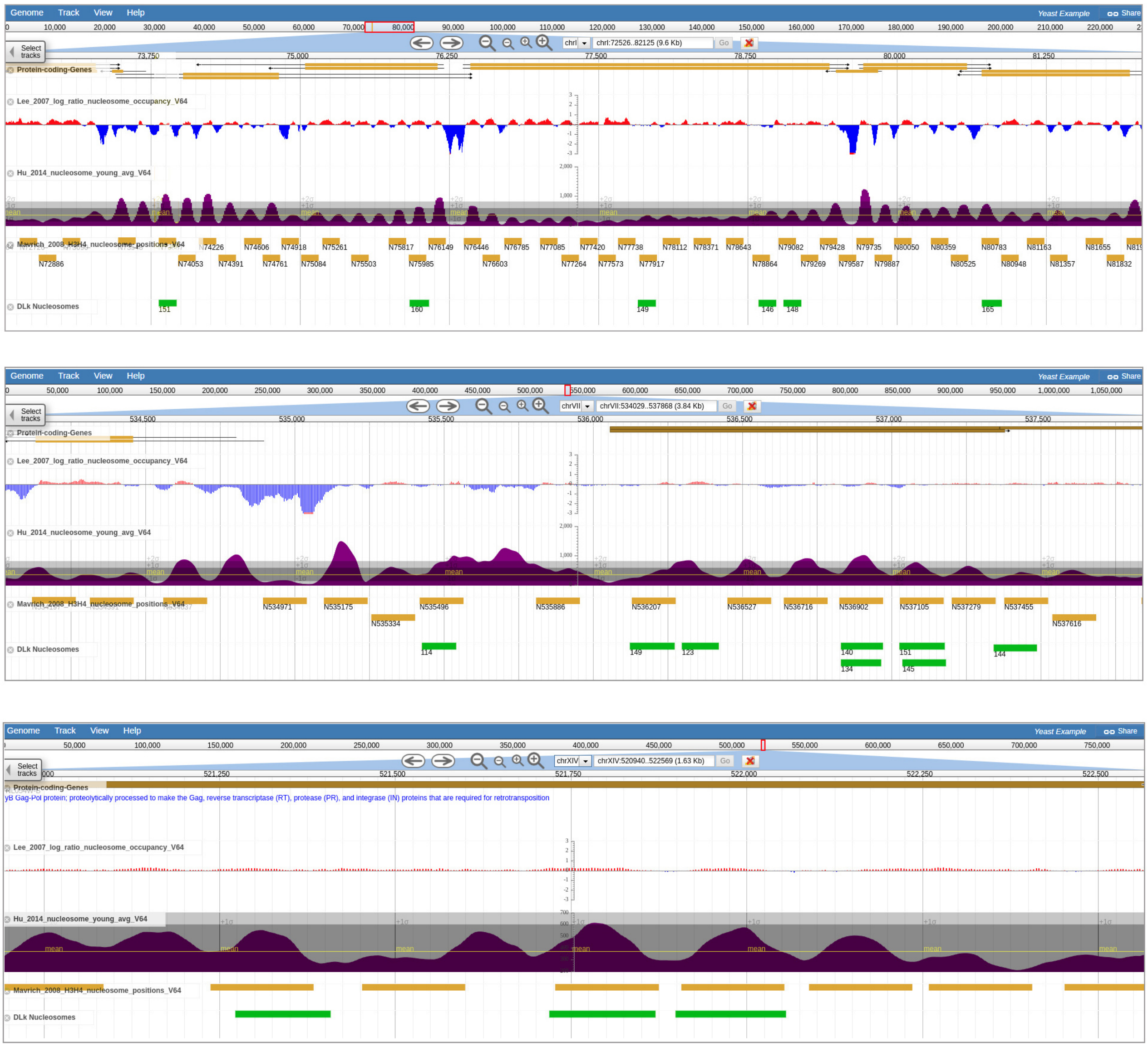

Supplementary Fig. 3. Comparison of the nucleosomal DNA library with previously reported nucleosome occupancy maps. The dataset of nucleosome coordinates (Supplementary Data 1) was uploaded to the Yeast Genome Browser (https://browse.yeastgenome.org/). The 3 snapshots show particular chromosomal regions with a relatively high density of the uploaded nucleosomes (green tracks). For comparison, the snapshots include three different published nucleosomal maps (Lee 2007; Hu 2014, Mavrich 2008) obtained by micrococcal nuclease digestion.

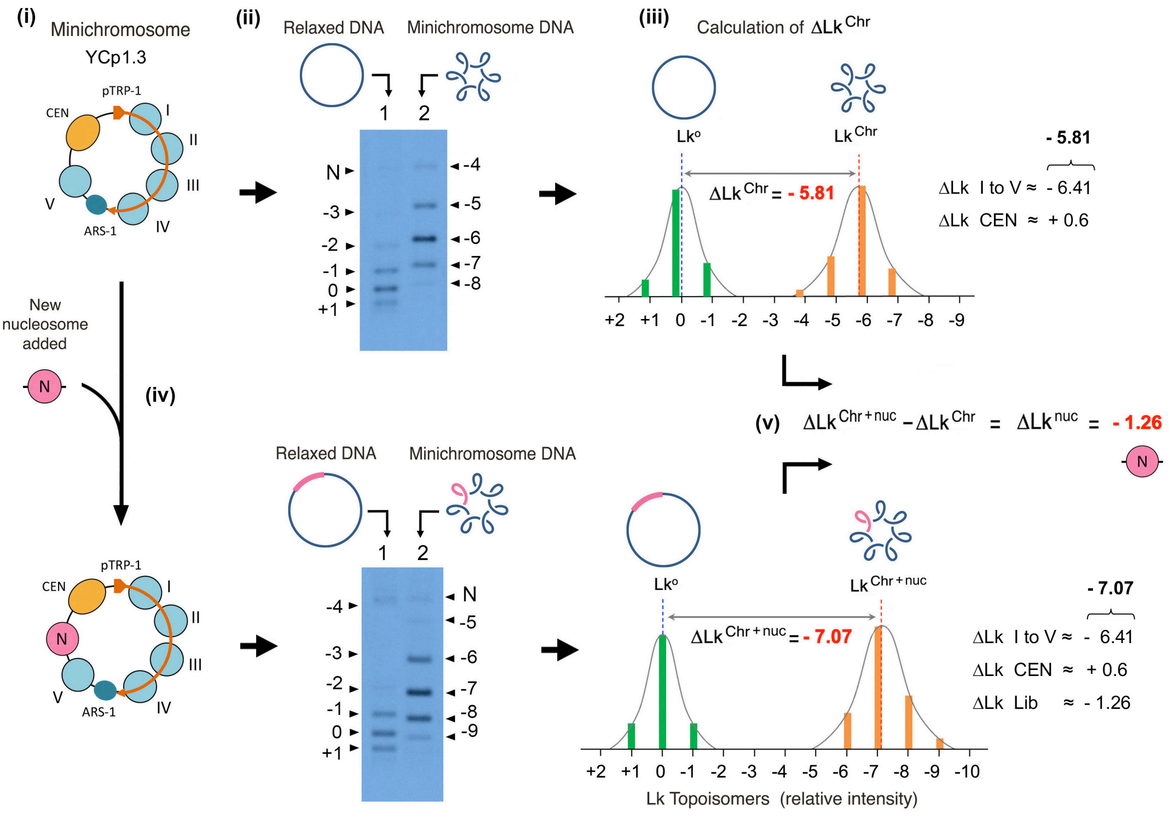

Supplementary Fig. 4. Experimental layout to calculate the ΔLk restrained by nucleosomes *in vivo*. In a circular minichromosome **(i)**, the DNA linking number difference constrained by its chromatin elements (ΔLk^{Chr}) is calculated by comparing, via gel electrophoresis **(ii)**, the Gaussian distribution of Lk topoisomers of the DNA relaxed *in vitro* (lane 1) with that of the minichromosome DNA fixed *in vivo* (lane 2). The gel position of the Lk mean of the relaxed DNA (Lk^0) and that of the minichromosome DNA (Lk^{Chr}) is determined by plotting the intensities of their corresponding Lk topoisomers along a scale of ΔLk units **(iii)**. ΔLk^{Chr} is the distance in Lk units between the two means (Lk^0-Lk^{Chr}=ΔLk^{Chr}). In the YCp1.3 minichromosome, the ΔLk^{Chr} value of -5.81 results from the ΔLk restrained by the point centromere (+0.6) and the ΔLk restrained by the segment I to V (-6.41). Upon adding a new nucleosome (≈150 bp) **(iv)**, the absolute Lk of the DNA circle increases by about 14 units (≈150 bp/10,5 bp per helical turn). The gel position of the Lk mean (Lk^0) of this larger DNA and the corresponding minichromosome DNA ($Lk^{Chr+nuc}$) are again determined to obtain $\Delta Lk^{Chr+nuc}$. The resulting difference between ΔLk^{Chr} and $\Delta Lk^{Chr+nuc}$ equals ΔLk^{nuc}, the ΔLk restrained by the added nucleosome **(v)**. ΔLk^{nuc} is about -1.26 for most nucleosomes, as previously reported by Segura *et al* (2018). Source data are provided as a Source Data file.

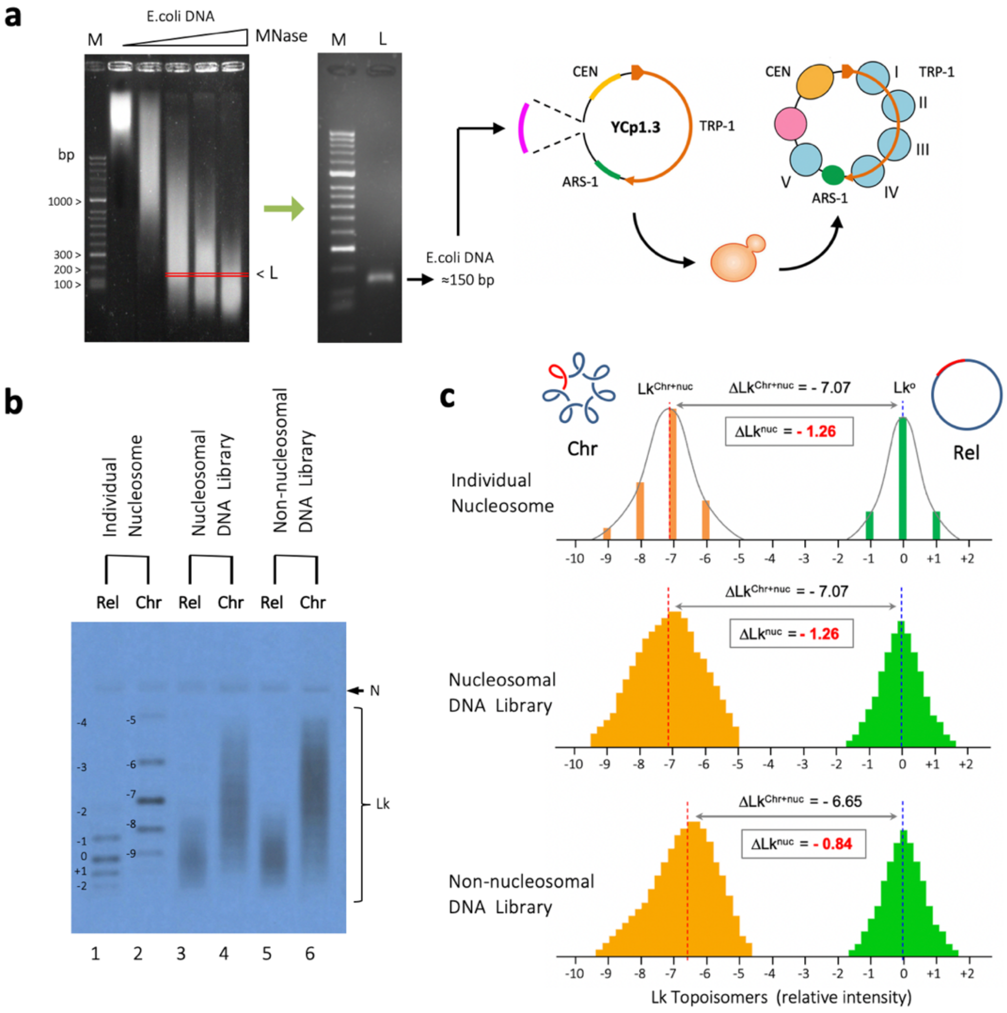

Supplementary Fig. 5. Comparison of the ΔLk restrained by nucleosomal and non-nucleosomal DNA libraries. **a,** MNase digestion of E.coli genomic DNA and gel purification of DNA fragments of ≈150 bp in length (L). Marker DNAs (M). This collection of non-nucleosomal DNA fragments was processed and inserted in the YCp1.3 minichromosome as described for the nucleosomal DNA library. **b**, Gel electrophoresis of the Lk distributions of the YCp1.3 minichromosome after adding one individual nucleosome of ΔLk^{nuc} -1.26 (lanes 1 and 2), the nucleosomal DNA library (lanes 3 and 4), and the non-nucleosomal DNA library (lanes 5 and 6). As explained in Supplementary Fig. 4, the Lk distribution constrained by the minichromosomes (Chr) (lanes 2, 4 and 6) is compared to that of the relaxed DNAs (Rel) (Lanes 1, 3, and 5). **c**, Plots to calculate the DNA linking number difference ($\Delta Lk^{Chr+nuc}$) constrained by the minichromosomes described above. As explained in Supplementary Fig. 4, $\Delta Lk^{Chr+nuc}$ is the distance in Lk units from $Lk^{Chr+nuc}$ (Chr) to Lk^{0} (Rel). The value of ΔLk^{nuc} (or the inserted element) equals $Lk^{Chr+nuc}$ - 5.81, which is the Lk difference constrained by the empty YCp1.3 minichromosome. In agreement with our previous study (Segura *et al* 2018), the nucleosomal DNA library produced an average ΔLk^{nuc} of about -1.26. This single experiment indicated that the non-nucleosomal DNA library produced an average ΔLk^{nuc} of about -0.84, which demonstrated that prokaryotic DNA is less suited than eukaryotic DNA to assemble nucleosomes. Source data are provided as a Source Data file.

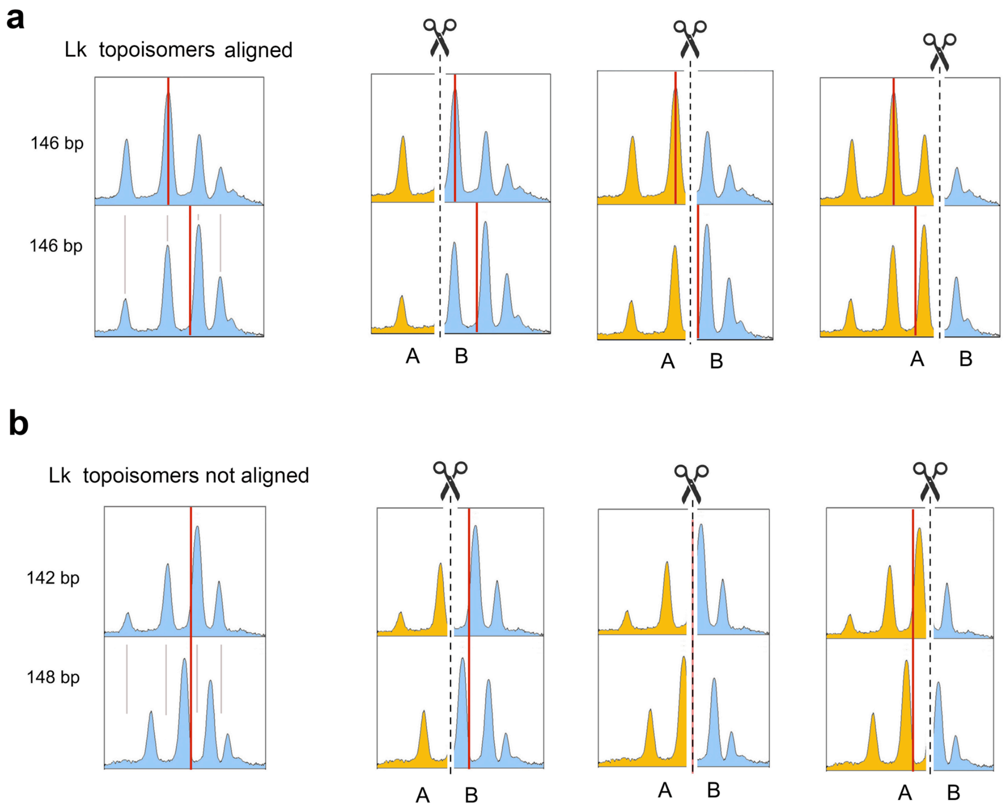

Supplementary Fig. 6. Effect of aligned and non-aligned Lk distributions when conducting Topo-seq. a, Example of two Lk distributions with different Lk mean (red lines), but with aligned topoisomers, since the DNA fragments of the nucleosome library have equal length (146 bp). Note that, whatever the position of a splitting cut (3 examples depicted), the partition of DNA intensities (coloured areas A and B) always denotes that the two distributions have different Lk mean. **b**, Example of two Lk distributions with equal Lk mean (red lines) but with not aligned topoisomers because the DNA fragments of the nucleosome library have different lengths (142 and 148 bp). In this case, depending on the position of the splitting cut, the partition of DNA intensities does not always denote that the two distributions have equal Lk mean.

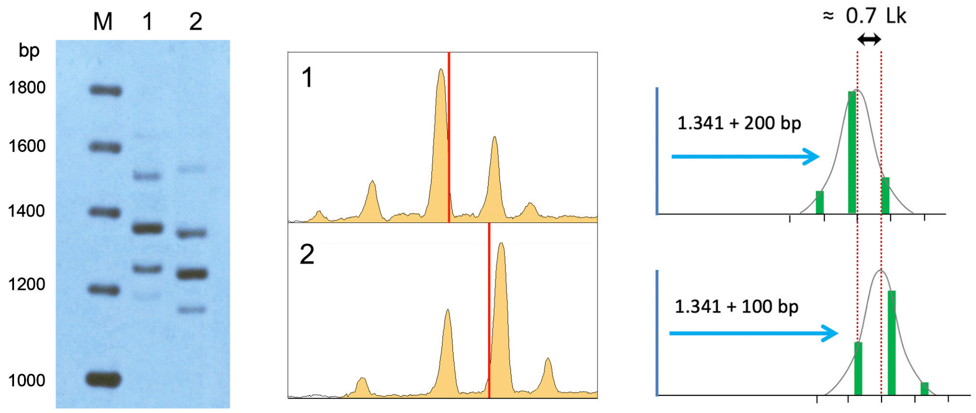

Supplementary Fig. 7. Correlation of DNA length with the electrophoretic velocity of Lk distributions. Left, the gel shows a marker of linear DNAs (M) and the Lk distributions of two relaxed DNA molecules differing in 100 bp in length. These molecules are the YCp1.3 minichromosome DNA (1.341 bp) with an insert of 100 bp (lane 1) and 200 bp (lane 2). Note that the size of these inserts encloses the length variability of the nucleosome library (144±33bp). Middle, densitometry of lanes 1 and 2 indicating the position of the Lk means (red lines). Right, the distance of the Lk means of these DNAs differing in 100 bp was equivalent to 0.7 Lk units, thus 0.007 Lk/bp. Source data are provided as a Source Data file.

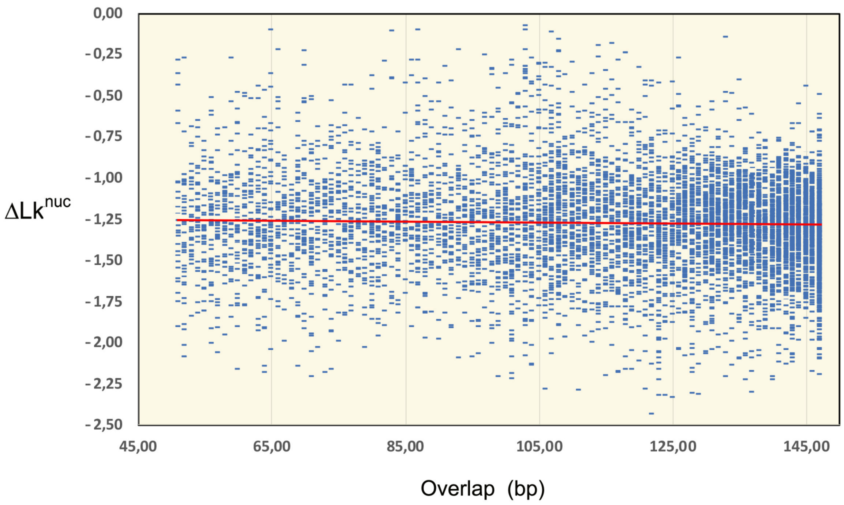

Supplementary Fig. 8. Correlation of ΔLk^{nuc} and nucleosome overlapping with the reference catalogue. The plot shows ΔLk^{nuc} values obtained via Topo-seq against the extent of overlap (bp) of the nucleosomal DNA fragments with previously referenced nucleosomes in Jiang and Puhg (2009). The trend line of the average ΔLk^{nuc} value is depicted (red). Source data are provided as a Source Data file.

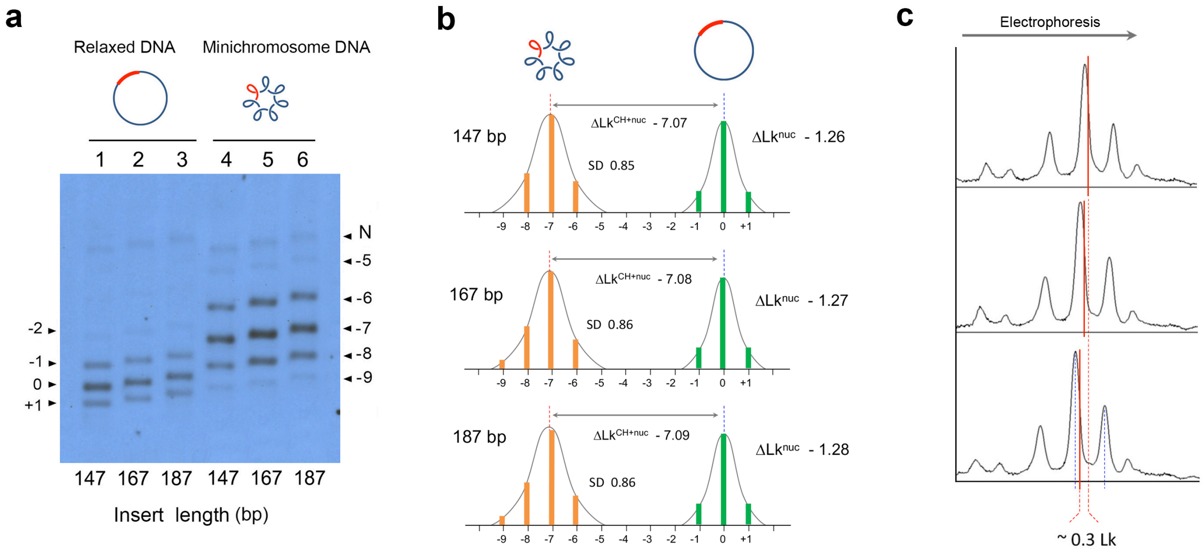

Supplementary Fig. 9. Effect of the linker DNA length on Lk^{nuc}. a, Lk distributions of YCp1.3 hosting a nucleosomal DNA insert of 147 bp plus 0, 10 or 20 bp of extra linker DNA at each side (147, 167, 187 bp inserts). Nucleosome coordinates are described in Supplementary Table 1. Lanes 1-3 show the relaxed DNA Lk distributions of these constructs, and lanes 4-6 show those of the corresponding minichromosomes. **b,** Topoisomer intensities, $\Delta Lk^{Chr+nuc}$ values and standard deviation (SD) of the minichromosome Lk distributions. ΔLk^{nuc} produced by each insert (147, 167, 187 bp) was obtained as the difference between the $\Delta Lk^{Chr+nuc}$ values and -5.81, which is the ΔLk^{Chr} of the empty YCp1.3 minichromosome. Lk^{nuc} was -1.26 for the 147bp insert, -1.27 for the 167bp insert, and -1.28 for the 187bp insert. **c**, Gel densitometry of lanes 4-6 and position of the Lk means (red lines). The Lk mean of the 167 and 187 bp constructs (lanes 5 and 6) was retarded about a distance equivalent to +0.15 and +0.30 Lk units relative to that of the 147 bp construct (lane 4). These values are in good agreement with the 0.007 Lk unit/bp correction used in our study. Source data are provided as a Source Data file.

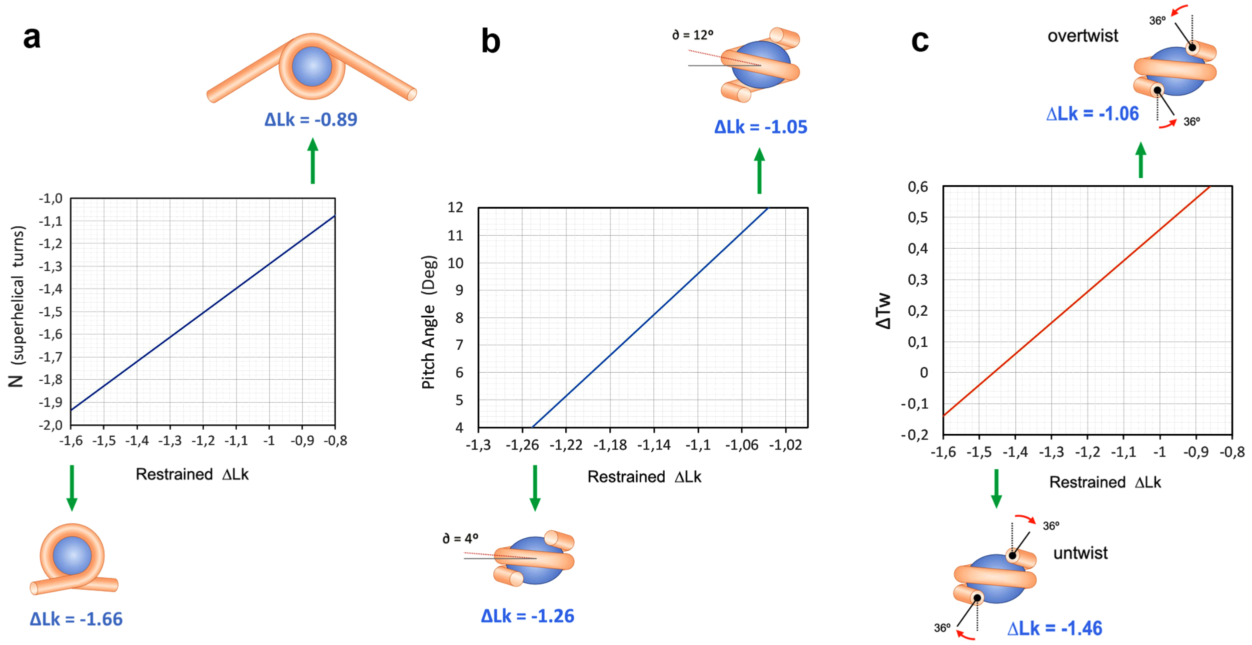

Supplementary Fig. 10. Effect of nucleosome DNA wrapping (ΔWr) and twisting (ΔTw) on the restrained ΔLk. a, Plot of restrained ΔLk as a function of the number of DNA super-helical turns (N) wrapped in the nucleosome. ΔLk values are calculated as ΔTw+ΔWr, where ΔTw=+0.2 and ΔWr=N(1–sin 4°). **b,** Plot of restrained ΔLk as a function of the pitch angle (∂) of the super-helical turns wrapped in the nucleosome. ΔLk values are calculated considering ΔTw=+0.2 and ΔWr=1.56(1–sin ∂). **c,** Plot of restrained ΔLk as a function of the DNA twist (ΔTw) restrained by the nucleosome. ΔLk values are calculated considering ΔWr=1.56(1–sin 4°). Nucleosome DNA geometries are modelled for the indicated ΔLk.

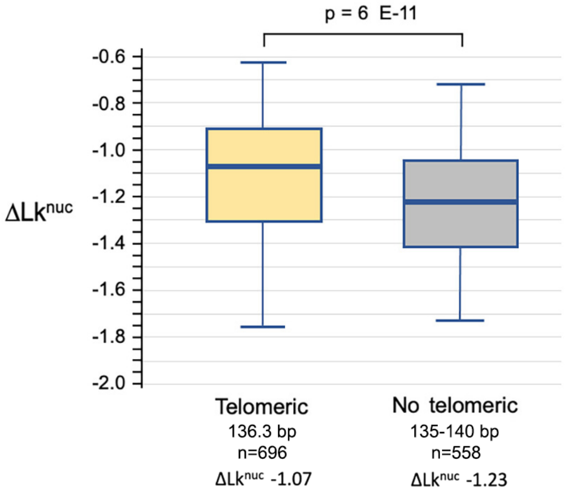

Supplementary Fig. 11. Lk^{nuc} of telomeric and short no-telomeric nucleosomes. ΔLk^{nuc} values restrained by telomeric (n=696) and non-telomeric (n=558) nucleosomal DNAs with fragment lengths 135 to 140 bp. In the box plots, the centre line denotes the median value, while the box contains the 25th (upper limit) to 75th (lower limit) percentiles of the dataset. The black whiskers mark the 5th and 95th percentiles. The indicated *p*-value was determined by an unpaired two-sided *t*-test. Source data are provided as a Source Data file.

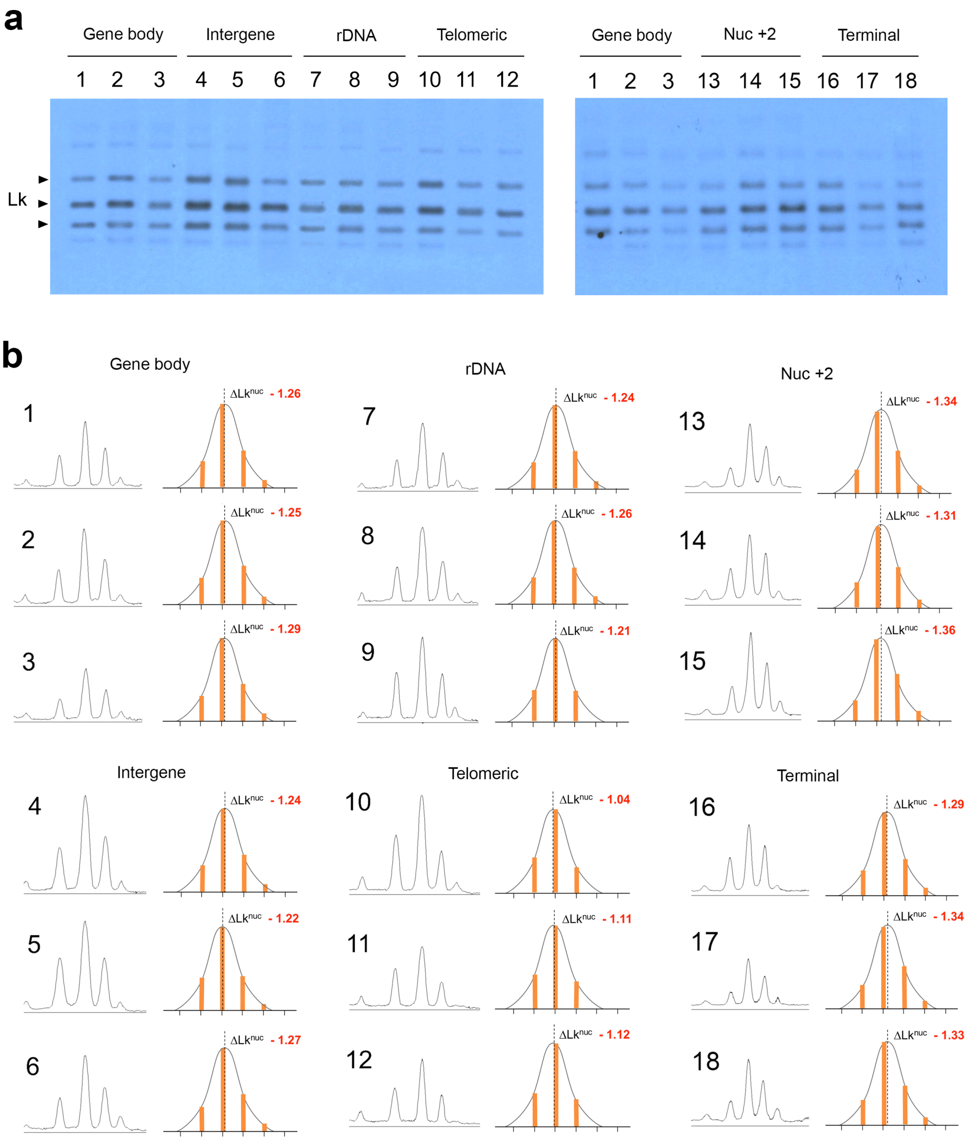

Supplementary Fig. 12. Validation of the ΔLk^{nuc} dependence on the genomic origin of nucleosomes. a, Gel electrophoresis of Lk distributions of the YCp1.3 minichromosome hosting 18 nucleosomal DNA sequences of 147 bp, whose ΔLk^{nuc} determined via Topo-seq was representative of the mean ΔLk^{nuc} value of their corresponding allocations (gene body, intergenic, rDNA, telomeric, nuc +2, terminal nucleosome). The chromosomal coordinates and PCR primers for cloning these 18 nucleosomal DNA sequences into YCp1.3 are described in Supplementary Table 1. Electrophoresis conditions are described in Methods. Arrowheads indicate Lk topoisomers. **b**, Gel densitometry and relative topoisomer intensities of the previous Lk distributions (lanes 1-18). The ΔLk^{nuc} restrained by each of the 18 nucleosomes was calculated relative to the reference nucleosome in line 1 (ΔLK^{nuc} -1.26), which is the same nucleosome used to test the effect of the linker DNA length on Lk^{nuc} (Supplementary Fig. 9). Source data are provided as a Source Data file.

Supplementary Table 1. Coordinates and PCR primers to validate the Lk^{nuc} of individual nucleosome DNAs								
Chrm	Start	End	Nucleosome ID	Location	Forward PCR primer	Reverse PCR primer	ΔLk^{nuc} Topo-seq	ΔLk^{nuc} Individual
chrVIII	261335	261482	+3:YHR079C;	Gene body	TTCAATAAGTTAGGAGTTGGATATG	AGTGGAAGATGAAGTTGCCTCCACT	-1,27	-1,26
chrIV	994735	994883	+7:YDR263C;	Gene body	TACTGAAACAAAGTTTTTCTGTCCG	GTAATCTAGTTTGTTTAGCAGGGTG	-1,27	-1,25
chrXIV	526651	526800	+5:YNL054W;	Gene body	AACAGCGAGATCGGCGAATATTCAT	AGAGGTAAAAGAGGAGCTGGAGGTG	-1,27	-1,29
chrII	224435	224582		Intergene	ACACATGAGTCGTCGTACGCCAGTA	CTCCTGTTCGCTTGTCTGCGGAAC	-1,23	-1,24
chrIII	5166	5314		Intergene	TGAGAGGACACAACTTTTTGAGGCT	GGAGTTTTTCCAGACACCAGTAGC	-1,23	-1,22
chrXVI	678051	678201		Intergene	TGTACACTCTGAATCAGGGAGAACC	CCACAGGTACAAGAAAATTGCGCCA	-1,23	-1,27
chrXII	462258	462407		rDNA	TACCAAGGCCGTCTACAACAAGGCA	GATCCGTAACTTCGGGATAAGGATT	-1,24	-1,24
chrXII	466289	466442		rDNA	ACCGCGGCTGCTGGCACCAGACTTG	GCGCAAATTACCCAATCCTAATTCA	-1,24	-1,26
chrXII	468401	468554		rDNA	AAATCTCTTCCCGTCATTATCGCCC	AGTTTCAAGGTGACAGGTTATGAAG	-1,24	-1,21
chrVIII	558986	559129	+7:YHR218W;	Telomeric	AATCTTCTCTTTATTAATAAATATG	GAGTTTGTGAGTAGTCAGATTTTC	-1,06	-1,04
chrXV	1090465	1090616	+32:YOR396W;	Telomeric	GTACGTCAAGCTGCAGTTTAGCAGG	ATCAGCATCGACAGGAATGCCGTCC	-1,08	-1,11
chrVIII	559259	559411	+9:YHR218W;	Telomeric	GAAGAAGGTTGCGATGGCGTTACTG	AATGAGGTAACCCAATTTTACGTTG	-1,08	-1,12
chrVI	269103	269252	+2:YFR057W;	Nuc +2	AGTCAAGCGGTATCATTAAAGACAC	GACATATTGAATCATATAGATTATG	-1,33	-1,34
chrX	684490	684642	+2:YJR138W;	Nuc +2	AACAACGAATTGTTTCTGTAACTAC	TGCTAGTCCTTGGAGTTTGTGAATTG	-1,33	-1,31
chrVIII	234383	234536	+2:YHR069C;	Nuc +2	GACACGGGATACAGTACCAGCAACA	AACTCAGCTTCTAAAAGTGAGTCTG	-1,33	-1,36
chrIV	1121801	1121948	+21:YDR326C;	Terminal	TAGTTGGCAGATTAATAATTTCTTC	AGGGAACATTTGATGGTGTTGCGG	-1,32	-1,29
chrVIII	522442	522595	+3:SUT174;	Terminal	CCATAATTATACGCAAATGCTGCAGT	ACTAAACTATTGCTGGCCAGCACCC	-1,32	-1,34
chrXV	626417	626570	+12:YOR154W;	Terminal	CTTTGAAGATTTCGAAGAGAGCAAAA	CCTCATATATGGCGGTGGATTTTTTC	-1,32	-1,33
chrVIII	261335	261482	* +3:YHR079C;	Gene body	TTCAATAAGTTAGGAGTTGGATATG	AGTGGAAGATGAAGTTGCCTCCACT	-1,27	-1,26
			* +20 bp linker	Gene body	ATCAGCAGTATTCAATAAGTTAGGA	GGCGGCAGATAGTGGAAGATGAAGT		-1,27
			* +40 bp linker	Gene body	CACGTCGATTATCAGCAGTATTCAA	CGCACCTCAAGGCGGCAGATAGTGG		-1,28

* The same nucleosome DNA (147 bp) with increassing linker DNA lengths
