# Supplementary material for: Nucleosomal DNA has topological memory
Source: Nat Commun. 2024 May 28;15:4526. doi: 10.1038/s41467-024-49023-4 (PMC11133463; doi:10.1038/s41467-024-49023-4)
Supplement: Supplementary file 3 — Description of Additional Supplementary Files [file 41467_2024_49023_MOESM3_ESM.pdf]

Description of Additional Supplementary Files

File Name: Supplementary Data 1
Description: Coordinates of the nucleosome DNA library and their nucleosome ID.

File Name: Supplementary Data 2
Description: Calculation of the ΔLk restrained by individual nucleosomes.

File Name: Supplementary Data 3
Description: ΔLk values and genomic context of nucleosomes.
