# Supplementary material for: Nucleosomal DNA has topological memory
Source: Nat Commun. 2024 May 28;15:4526. doi: 10.1038/s41467-024-49023-4 (PMC11133463; doi:10.1038/s41467-024-49023-4)
Supplement: Supplementary file 4 — Supplementary Data 1 [file 41467_2024_49023_MOESM4_ESM.pdf]

Supplementary Data 1. Coordinates of the nucleosome DNA library and their nucleosome ID.

The four first columns indicate the chromosomal coordinates of the nucleosome library. The last column (nucleosome ID) tags those library sequences that overlapped >50 bp with the previously referenced nucleosomes compiled by Jiang and Pugh (2009). 91% of the library sequences matched with 4276 previously referenced nucleosomes.

Chr	Start	End	Length (bp)	Nucleosome_ID
chrI	375	485	110	N1:382
chrI	1992	2143	151	N1:2081
chrI	3900	4046	146	
chrI	7310	7448	138	N1:7314
chrI	19698	19849	151	N1:19743
chrI	24106	24256	150	
chrI	24106	24267	161	
chrI	24156	24264	108	
chrI	24156	24265	109	
chrI	24156	24266	110	
chrI	24156	24267	111	
chrI	25658	25790	132	
chrI	25658	25797	139	
chrI	25671	25797	126	
chrI	25885	26057	172	
chrI	26200	26329	129	
chrI	26200	26330	130	
chrI	26200	26337	137	
chrI	26204	26330	126	
chrI	26211	26330	119	
chrI	26268	26418	150	N1:26380
chrI	26290	26465	175	N1:26380
chrI	26425	26573	148	N1:26590
chrI	26560	26708	148	N1:26590
chrI	26695	26843	148	N1:26787
chrI	27347	27495	148	N1:27437
chrI	28545	28653	108	N1:28676
chrI	28558	28705	147	N1:28676
chrI	28865	28996	131	N1:28942
chrI	31670	31815	145	N1:31784
chrI	32825	32968	143	N1:32915
chrI	38266	38455	189	N1:38289
chrI	38266	38455	189	N1:38459
chrI	39356	39504	148	N1:39417
chrI	44014	44169	155	N1:44128
chrI	48679	48833	154	N1:48747
chrI	52107	52211	104	N1:52114
chrI	52108	52200	92	N1:52114
chrI	52108	52202	94	N1:52114
chrI	52108	52203	95	N1:52114
chrI	52108	52204	96	N1:52114
chrI	52108	52205	97	N1:52114
chrI	52108	52206	98	N1:52114
chrI	52108	52207	99	N1:52114
chrI	52108	52208	100	N1:52114
chrI	52108	52209	101	N1:52114
chrI	52108	52210	102	N1:52114
chrI	52108	52211	103	N1:52114
chrI	52112	52211	99	N1:52114
chrI	54178	54332	154	N1:54248
chrI	56067	56218	151	N1:56143
chrI	57573	57723	150	N1:57726
chrI	57573	57723	150	N1:57555
chrI	57985	58142	157	N1:58076
chrI	58357	58529	172	N1:58432
chrI	59835	59985	150	N1:59903
chrI	60956	61114	158	N1:61025
chrI	61139	61253	114	
chrI	62731	62859	128	N1:62815
chrI	73815	73966	151	N1:73878
chrI	75929	76089	160	N1:75990
chrI	77820	77969	149	N1:77885
chrI	78867	79013	146	N1:78863
chrI	79075	79223	148	N1:79100
chrI	79820	79919	99	N1:79895
chrI	80712	80877	165	N1:80770
chrI	83877	84031	154	N1:83953
chrI	87561	87716	155	N1:87641
chrI	88852	89013	161	N1:89028
chrI	88852	89013	161	N1:88849
chrI	88889	89053	164	N1:89028
chrI	91036	91130	94	N1:91042
chrI	94271	94416	145	
chrI	97984	98151	167	N1:98096
chrI	100299	100401	102	N1:100335
chrI	100430	100592	162	N1:100502
chrI	100744	100901	157	N1:100813
chrI	100903	101028	125	N1:100972
chrI	101774	101913	139	N1:101784
chrI	102759	102918	159	N1:102829
chrI	110352	110498	146	N1:110405
chrI	110959	111107	148	N1:110988
chrI	111829	111987	158	N1:111914
chrI	111829	111989	160	N1:111914
chrI	111835	111987	152	N1:111914
chrI	111835	111989	154	N1:111914
chrI	113198	113345	147	N1:113359
chrI	113198	113345	147	N1:113192

Chr	Start	End	Length (bp)	Nucleosome_ID
chrI	113272	113431	159	N1:113359
chrI	114916	115064	148	N1:115021
chrI	116310	116474	164	N1:116396
chrI	119258	119408	150	N1:119265
chrI	121555	121709	154	N1:121654
chrI	128610	128759	149	
chrI	128749	128913	164	N1:128845
chrI	132160	132339	179	N1:132234
chrI	132322	132449	127	N1:132398
chrI	133311	133456	145	N1:133382
chrI	133444	133595	151	N1:133558
chrI	134936	135082	146	
chrI	139478	139611	133	N1:139570
chrI	144452	144595	143	N1:144447
chrI	144452	144595	143	N1:144596
chrI	145995	146131	136	N1:145995
chrI	145995	146131	136	N1:146151
chrI	157171	157325	154	N1:157317
chrI	158225	158386	161	N1:158305
chrI	158476	158631	155	N1:158473
chrI	158476	158631	155	N1:158641
chrI	159246	159353	107	N1:159237
chrI	161173	161287	114	N1:161301
chrI	163265	163422	157	N1:163329
chrI	164304	164458	154	N1:164322
chrI	164304	164458	154	N1:164474
chrI	164952	165092	140	N1:165025
chrI	164958	165092	134	N1:165025
chrI	165370	165525	155	N1:165400
chrI	165501	165609	108	N1:165556
chrI	165531	165649	118	N1:165556
chrI	165650	165799	149	N1:165722
chrI	170185	170316	131	
chrI	170746	170892	146	N1:170868
chrI	173251	173422	171	
chrI	174403	174559	156	N1:174467
chrI	177062	177157	95	
chrI	178178	178314	136	N1:178222
chrI	181632	181781	149	N1:181719
chrI	184868	185010	142	N1:184881
chrI	188940	189093	153	N1:189025
chrI	189954	190061	107	N1:190031
chrI	189954	190062	108	N1:190031
chrI	189954	190063	109	N1:190031
chrI	189954	190077	123	N1:190031
chrI	191415	191573	158	N1:191409
chrI	191722	191874	152	N1:191782
chrI	192889	193041	152	N1:192956
chrI	193467	193622	155	N1:193586
chrI	194126	194279	153	
chrI	194141	194279	138	
chrI	194290	194442	152	N1:194358
chrI	204527	204675	148	N1:204558
chrI	204662	204810	148	N1:204754
chrI	204797	204945	148	
chrI	205040	205170	130	
chrI	205573	205699	126	N1:205618
chrI	205573	205705	132	N1:205618
chrI	205573	205712	139	N1:205618
chrI	205580	205712	132	N1:205618
chrI	206390	206543	153	N1:206455
chrI	207406	207531	125	N1:207477
chrI	215442	215555	113	N1:215495
chrI	217878	218015	137	N1:217966
chrI	217916	218076	160	N1:217966
chrI	218711	218856	145	N1:218780
chrI	218738	218886	148	N1:218780
chrI	222387	222554	167	N1:222504
chrI	222751	222855	104	N1:222820
chrI	223257	223420	163	N1:223335
chrI	224065	224173	108	N1:224083
chrI	224877	224997	120	N1:224931
chrI	226540	226687	147	N1:226556
chrI	229861	230005	144	N1:229980
chrI	230119	230218	99	N1:230156
chrI	230124	230218	94	N1:230156
chrII	163	311	148	N2:255
chrII	526	677	151	N2:589
chrII	1089	1240	151	N2:1150
chrII	1727	1858	131	N2:1853
chrII	1727	1870	143	N2:1853
chrII	1727	1914	187	N2:1853
chrII	1931	2076	145	
chrII	1936	2043	107	
chrII	1937	2034	97	
chrII	1937	2042	105	
chrII	1937	2046	109	
chrII	1937	2051	114	
chrII	1937	2054	117	
chrII	1937	2057	120	
chrII	1937	2062	125	
chrII	1937	2071	134	
chrII	1937	2072	135	
chrII	1937	2073	136	
chrII	1937	2075	138	
chrII	1937	2076	139	
chrII	1937	2082	145	
chrII	1937	2086	149	

Chr	Start	End	Length (bp)	Nucleosome_ID
chrII	1938	2076	138	
chrII	1939	2076	137	
chrII	1940	2076	136	
chrII	1941	2076	135	
chrII	1942	2076	134	
chrII	1944	2076	132	
chrII	1947	2076	129	
chrII	1948	2076	128	
chrII	1952	2076	124	
chrII	1953	2076	123	
chrII	1964	2076	112	
chrII	1966	2076	110	
chrII	2124	2286	162	N2:2264
chrII	2124	2286	162	N2:2110
chrII	2191	2286	95	N2:2264
chrII	2267	2374	107	N2:2264
chrII	2267	2414	147	N2:2264
chrII	2522	2620	98	
chrII	2522	2673	151	
chrII	3354	3498	144	N2:3449
chrII	3419	3571	152	N2:3449
chrII	3701	3844	143	N2:3787
chrII	3992	4129	137	N2:4071
chrII	7980	8135	155	
chrII	10712	10874	162	N2:10765
chrII	20981	21152	171	N2:21084
chrII	21442	21596	154	N2:21498
chrII	24248	24399	151	N2:24353
chrII	24299	24437	138	N2:24353
chrII	25280	25416	136	N2:25382
chrII	26126	26261	135	N2:26202
chrII	30232	30334	102	
chrII	31655	31803	148	
chrII	32970	33121	151	N2:32989
chrII	35988	36136	148	N2:36067
chrII	48768	48912	144	N2:48843
chrII	54394	54539	145	N2:54546
chrII	54394	54539	145	N2:54380
chrII	54551	54716	165	N2:54732
chrII	54551	54716	165	N2:54546
chrII	62389	62546	157	N2:62463
chrII	62885	63033	148	N2:62915
chrII	64104	64264	160	
chrII	64653	64794	141	
chrII	65899	66050	151	N2:65948
chrII	67731	67881	150	N2:67884
chrII	69118	69263	145	N2:69156
chrII	79393	79540	147	N2:79471
chrII	81527	81678	151	N2:81676
chrII	81527	81678	151	N2:81506
chrII	86475	86605	130	N2:86602
chrII	87052	87211	159	N2:87114
chrII	94866	95023	157	N2:94979
chrII	97063	97217	154	N2:97213
chrII	97108	97247	139	N2:97213
chrII	98068	98229	161	N2:98203
chrII	98405	98563	158	N2:98532
chrII	102188	102336	148	N2:102248
chrII	105450	105577	127	N2:105509
chrII	105532	105684	152	N2:105680
chrII	105532	105684	152	N2:105509
chrII	105754	105910	156	N2:105836
chrII	106088	106233	145	
chrII	112631	112737	106	N2:112714
chrII	112631	112749	118	N2:112714
chrII	117607	117737	130	N2:117637
chrII	118185	118336	151	N2:118300
chrII	118430	118588	158	N2:118451
chrII	118430	118588	158	N2:118611
chrII	118604	118737	133	N2:118611
chrII	118662	118820	158	N2:118790
chrII	120690	120830	140	N2:120678
chrII	130330	130481	151	N2:130370
chrII	133172	133320	148	N2:133293
chrII	134044	134208	164	N2:134212
chrII	134044	134208	164	N2:134052
chrII	146223	146376	153	N2:146325
chrII	147729	147892	163	
chrII	157306	157457	151	N2:157445
chrII	158794	158975	181	N2:158865
chrII	159579	159667	88	N2:159667
chrII	159579	159734	155	N2:159667
chrII	161150	161286	136	N2:161276
chrII	161650	161786	136	N2:161723
chrII	164812	164963	151	N2:164880
chrII	169155	169305	150	N2:169306
chrII	171571	171720	149	N2:171641
chrII	171661	171810	149	N2:171641
chrII	171661	171810	149	N2:171803
chrII	174305	174447	142	N2:174374
chrII	186048	186192	144	N2:186122
chrII	187191	187351	160	N2:187294
chrII	188175	188332	157	N2:188328
chrII	189372	189539	167	
chrII	192490	192638	148	N2:192564
chrII	195265	195410	145	N2:195403
chrII	203726	203877	151	N2:203822
chrII	204265	204408	143	N2:204305

Chr	Start	End	Length (bp)	Nucleosome_ID
chrII	213654	213809	155	N2:213693
chrII	214589	214739	150	N2:214660
chrII	214869	215017	148	N2:214983
chrII	215320	215464	144	N2:215386
chrII	216639	216771	132	N2:216655
chrII	217546	217694	148	N2:217574
chrII	222615	222772	157	N2:222719
chrII	222619	222772	153	N2:222719
chrII	223146	223281	135	N2:223185
chrII	223772	223929	157	N2:223866
chrII	223872	224016	144	N2:223866
chrII	223872	224016	144	N2:224033
chrII	224435	224582	147	N2:224426
chrII	224435	224582	147	N2:224599
chrII	225175	225325	150	N2:225274
chrII	226374	226481	107	N2:226487
chrII	226832	226946	114	N2:226921
chrII	228528	228693	165	
chrII	233542	233703	161	N2:233579
chrII	234707	234798	91	N2:234705
chrII	239108	239251	143	N2:239202
chrII	249586	249752	166	N2:249650
chrII	252919	253070	151	
chrII	253969	254127	158	N2:254057
chrII	255566	255720	154	N2:255613
chrII	260109	260232	123	
chrII	260209	260364	155	N2:260319
chrII	260642	260776	134	N2:260707
chrII	260642	260782	140	N2:260707
chrII	261685	261820	135	N2:261719
chrII	262132	262282	150	N2:262199
chrII	262311	262468	157	N2:262403
chrII	262974	263121	147	N2:262967
chrII	263717	263867	150	N2:263815
chrII	264916	265023	107	
chrII	269730	269883	153	N2:269845
chrII	269730	269885	155	N2:269845
chrII	269781	269930	149	N2:269845
chrII	272917	273071	154	N2:273057
chrII	279942	280075	133	N2:280032
chrII	282915	283035	120	N2:282943
chrII	288899	289053	154	N2:288983
chrII	289452	289559	107	N2:289558
chrII	289452	289573	121	N2:289558
chrII	292636	292792	156	N2:292683
chrII	295986	296116	130	N2:296031
chrII	296940	297047	107	N2:296987
chrII	296940	297056	116	N2:296987
chrII	298361	298510	149	N2:298428
chrII	298515	298656	141	N2:298593
chrII	300341	300475	134	N2:300360
chrII	303103	303264	161	N2:303172
chrII	304167	304319	152	N2:304247
chrII	306192	306322	130	N2:306304
chrII	310844	311002	158	N2:310950
chrII	314561	314691	130	N2:314625
chrII	316179	316338	159	N2:316235
chrII	320829	320984	155	N2:320918
chrII	324261	324383	122	N2:324329
chrII	324593	324738	145	N2:324676
chrII	333410	333578	168	N2:333441
chrII	334171	334335	164	N2:334243
chrII	341568	341673	105	
chrII	344002	344150	148	N2:344104
chrII	345574	345727	153	N2:345699
chrII	348428	348599	171	N2:348504
chrII	348488	348624	136	N2:348504
chrII	352601	352731	130	N2:352692
chrII	354955	355107	152	N2:355010
chrII	360789	360938	149	N2:360839
chrII	369124	369272	148	N2:369186
chrII	369614	369763	149	N2:369688
chrII	372782	372926	144	N2:372886
chrII	379733	379878	145	N2:379800
chrII	380438	380593	155	N2:380439
chrII	380438	380593	155	N2:380588
chrII	383232	383381	149	N2:383330
chrII	384056	384165	109	
chrII	384888	385039	151	N2:384899
chrII	385956	386120	164	N2:386082
chrII	386724	386859	135	N2:386790
chrII	402057	402202	145	N2:402105
chrII	405294	405447	153	N2:405333
chrII	411086	411218	132	N2:411221
chrII	412922	413067	145	N2:413068
chrII	417276	417442	166	N2:417375
chrII	418844	418980	136	N2:418877
chrII	420492	420630	138	N2:420545
chrII	432763	432911	148	N2:432818
chrII	435614	435764	150	N2:435690
chrII	441246	441413	167	N2:441290
chrII	442313	442462	149	
chrII	447274	447421	147	N2:447332
chrII	448183	448311	128	N2:448253
chrII	448687	448853	166	
chrII	450522	450683	161	N2:450575
chrII	456967	457110	143	N2:456997
chrII	459507	459619	112	N2:459525

Chr	Start	End	Length (bp)	Nucleosome_ID
chrII	463510	463659	149	N2:463671
chrII	464402	464557	155	N2:464478
chrII	467973	468117	144	N2:468080
chrII	469937	470099	162	N2:469962
chrII	478544	478678	134	N2:478563
chrII	479993	480154	161	N2:480057
chrII	480073	480226	153	N2:480057
chrII	483614	483775	161	N2:483752
chrII	490237	490391	154	N2:490316
chrII	496459	496596	137	N2:496548
chrII	498698	498864	166	N2:498780
chrII	499848	499990	142	N2:499949
chrII	501137	501288	151	N2:501227
chrII	503704	503871	167	N2:503875
chrII	503704	503871	167	N2:503696
chrII	505974	506148	174	N2:506017
chrII	506025	506148	123	N2:506017
chrII	506025	506179	154	N2:506017
chrII	506025	506179	154	N2:506177
chrII	509772	509912	140	N2:509886
chrII	511646	511795	149	N2:511651
chrII	512273	512423	150	N2:512342
chrII	519148	519306	158	N2:519220
chrII	523260	523407	147	N2:523344
chrII	523774	523925	151	
chrII	525378	525528	150	N2:525423
chrII	527770	527902	132	N2:527832
chrII	528914	529061	147	N2:528946
chrII	529231	529384	153	N2:529279
chrII	533755	533907	152	N2:533826
chrII	543934	544097	163	N2:543989
chrII	545512	545645	133	
chrII	550054	550200	146	N2:550101
chrII	551769	551916	147	N2:551862
chrII	551821	551946	125	N2:551862
chrII	552584	552756	172	N2:552697
chrII	560316	560442	126	N2:560343
chrII	568857	569009	152	N2:568953
chrII	570513	570675	162	N2:570686
chrII	570513	570675	162	N2:570530
chrII	575360	575489	129	N2:575420
chrII	575363	575489	126	N2:575420
chrII	575635	575789	154	N2:575736
chrII	577662	577794	132	N2:577744
chrII	581371	581525	154	N2:581425
chrII	583028	583186	158	N2:583091
chrII	583277	583426	149	
chrII	589093	589249	156	N2:589146
chrII	598510	598657	147	N2:598568
chrII	599865	600024	159	N2:599938
chrII	601519	601677	158	N2:601690
chrII	601519	601677	158	N2:601539
chrII	608166	608308	142	N2:608176
chrII	608166	608308	142	N2:608330
chrII	610440	610604	164	N2:610482
chrII	615980	616137	157	N2:616096
chrII	618802	618957	155	N2:618851
chrII	621368	621503	135	N2:621487
chrII	624538	624687	149	N2:624638
chrII	626717	626881	164	N2:626851
chrII	633744	633889	145	N2:633802
chrII	633747	633889	142	N2:633802
chrII	634590	634750	160	N2:634645
chrII	635204	635350	146	N2:635289
chrII	636694	636853	159	N2:636766
chrII	636695	636853	158	N2:636766
chrII	636699	636853	154	N2:636766
chrII	641881	642011	130	N2:641909
chrII	643000	643147	147	N2:643066
chrII	643625	643773	148	N2:643703
chrII	645958	646066	108	N2:646057
chrII	652842	653006	164	
chrII	667311	667462	151	N2:667376
chrII	670468	670600	132	N2:670509
chrII	672951	673112	161	N2:673046
chrII	673886	673964	78	N2:673878
chrII	673886	673989	103	N2:673878
chrII	674406	674512	106	N2:674454
chrII	674406	674523	117	N2:674454
chrII	678764	678912	148	N2:678851
chrII	682036	682166	130	N2:682093
chrII	683947	684082	135	N2:684039
chrII	684506	684665	159	N2:684541
chrII	685114	685267	153	N2:685155
chrII	688568	688714	146	N2:688658
chrII	693669	693820	151	N2:693836
chrII	696303	696437	134	
chrII	698169	698308	139	N2:698202
chrII	698728	698854	126	N2:698800
chrII	698730	698836	106	N2:698800
chrII	699474	699629	155	N2:699534
chrII	699802	699945	143	N2:699870
chrII	699802	699953	151	N2:699870
chrII	699834	699945	111	N2:699870
chrII	699834	699953	119	N2:699870
chrII	702899	703031	132	N2:702974
chrII	706525	706696	171	N2:706600
chrII	708493	708657	164	N2:708624

Chr	Start	End	Length (bp)	Nucleosome_ID
chrII	708881	709054	173	N2:708943
chrII	716866	717023	157	N2:716955
chrII	716874	717023	149	N2:716955
chrII	718549	718695	146	N2:718618
chrII	721490	721639	149	N2:721551
chrII	723357	723507	150	N2:723426
chrII	727858	728011	153	N2:727936
chrII	733512	733676	164	N2:733639
chrII	735258	735391	133	N2:735332
chrII	736781	736927	146	N2:736768
chrII	736912	737033	121	
chrII	740252	740411	159	N2:740348
chrII	743678	743781	103	N2:743723
chrII	746216	746373	157	N2:746234
chrII	747192	747321	129	N2:747277
chrII	748930	749090	160	N2:749008
chrII	749254	749401	147	N2:749334
chrII	750045	750206	161	N2:750083
chrII	760513	760620	107	
chrII	760513	760634	121	
chrII	771359	771518	159	
chrII	771799	771953	154	
chrII	774249	774405	156	N2:774294
chrII	775701	775861	160	
chrII	777331	777484	153	N2:777349
chrII	779629	779798	169	N2:779707
chrII	780145	780251	106	N2:780192
chrII	780145	780260	115	N2:780192
chrII	780582	780757	175	N2:780666
chrII	781915	782083	168	N2:782014
chrII	786436	786542	106	N2:786464
chrII	786436	786560	124	N2:786464
chrII	788442	788582	140	N2:788514
chrII	788604	788759	155	N2:788689
chrII	789546	789709	163	N2:789650
chrII	791029	791155	126	N2:791088
chrII	791029	791156	127	N2:791088
chrII	791031	791136	105	N2:791088
chrII	793238	793400	162	N2:793321
chrII	795412	795559	147	N2:795519
chrII	809074	809203	129	N2:809118
chrII	812955	813098	143	N2:812953
chrIII	2	145	143	
chrIII	2	176	174	
chrIII	4	145	141	
chrIII	10	145	135	
chrIII	10	146	136	
chrIII	14	145	131	
chrIII	19	145	126	
chrIII	26	145	119	
chrIII	37	145	108	
chrIII	41	145	104	
chrIII	50	145	95	
chrIII	50	176	126	
chrIII	50	185	135	
chrIII	50	201	151	
chrIII	50	220	170	
chrIII	73	263	190	
chrIII	88	231	143	
chrIII	97	212	115	
chrIII	110	263	153	
chrIII	110	280	170	N3:298
chrIII	110	283	173	N3:298
chrIII	115	280	165	N3:298
chrIII	115	283	168	N3:298
chrIII	118	284	166	N3:298
chrIII	129	252	123	
chrIII	129	260	131	
chrIII	129	263	134	
chrIII	129	280	151	N3:298
chrIII	129	283	154	N3:298
chrIII	129	284	155	N3:298
chrIII	129	301	172	N3:298
chrIII	129	309	180	N3:298
chrIII	129	326	197	N3:298
chrIII	143	284	141	N3:298
chrIII	164	284	120	N3:298
chrIII	164	341	177	N3:298
chrIII	186	284	98	N3:298
chrIII	186	320	134	N3:298
chrIII	186	337	151	N3:298
chrIII	186	341	155	N3:298
chrIII	186	352	166	N3:298
chrIII	186	353	167	N3:298
chrIII	186	355	169	N3:298
chrIII	192	329	137	N3:298
chrIII	246	341	95	N3:298
chrIII	246	352	106	N3:298
chrIII	5166	5314	148	N3:5206
chrIII	5882	5994	112	N3:5973
chrIII	7560	7714	154	N3:7557
chrIII	9830	9940	110	N3:9914
chrIII	9831	9940	109	N3:9914
chrIII	9831	9941	110	N3:9914
chrIII	9832	9955	123	N3:9914
chrIII	11462	11613	151	N3:11616
chrIII	17946	18093	147	N3:18008
chrIII	23277	23427	150	N3:23351

Chr	Start	End	Length (bp)	Nucleosome_ID
chrIII	27235	27381	146	N3:27325
chrIII	32929	33083	154	N3:33061
chrIII	34797	34957	160	N3:34875
chrIII	36971	37125	154	
chrIII	37274	37421	147	N3:37360
chrIII	41105	41261	156	N3:41160
chrIII	42270	42419	149	N3:42308
chrIII	56437	56565	128	N3:56555
chrIII	62417	62568	151	N3:62518
chrIII	62420	62568	148	N3:62518
chrIII	62779	62946	167	N3:62871
chrIII	62988	63143	155	N3:63052
chrIII	68315	68492	177	N3:68474
chrIII	68580	68746	166	N3:68637
chrIII	74254	74383	129	
chrIII	75106	75269	163	N3:75209
chrIII	82256	82435	179	N3:82326
chrIII	86659	86788	129	
chrIII	86884	87013	129	N3:86885
chrIII	88349	88492	143	N3:88396
chrIII	89624	89790	166	
chrIII	90428	90577	149	N3:90522
chrIII	90432	90582	150	N3:90522
chrIII	92227	92379	152	N3:92268
chrIII	93544	93697	153	N3:93669
chrIII	94513	94671	158	N3:94604
chrIII	95867	96028	161	N3:95947
chrIII	95874	96028	154	N3:95947
chrIII	97469	97602	133	N3:97480
chrIII	100878	101025	147	N3:100936
chrIII	100878	101048	170	N3:100936
chrIII	101513	101652	139	N3:101599
chrIII	101547	101689	142	N3:101599
chrIII	101696	101853	157	N3:101768
chrIII	103033	103165	132	N3:103166
chrIII	103103	103249	146	N3:103166
chrIII	103103	103251	148	N3:103166
chrIII	104725	104877	152	N3:104791
chrIII	106067	106230	163	N3:106142
chrIII	116514	116644	130	N3:116605
chrIII	117818	117976	158	N3:117863
chrIII	132816	132923	107	
chrIII	132817	132923	106	
chrIII	133268	133417	149	N3:133250
chrIII	134878	135010	132	N3:134986
chrIII	136592	136744	152	N3:136655
chrIII	137946	138097	151	N3:138059
chrIII	138189	138339	150	N3:138298
chrIII	146706	146857	151	N3:146686
chrIII	154254	154394	140	N3:154383
chrIII	154256	154394	138	N3:154383
chrIII	156400	156550	150	N3:156457
chrIII	156437	156550	113	N3:156457
chrIII	156484	156645	161	N3:156660
chrIII	158189	158338	149	
chrIII	159911	160084	173	N3:160082
chrIII	159911	160084	173	N3:159912
chrIII	161801	161938	137	N3:161850
chrIII	175934	176084	150	N3:176001
chrIII	186417	186569	152	N3:186495
chrIII	188100	188260	160	N3:188165
chrIII	191522	191646	124	N3:191559
chrIII	195429	195578	149	
chrIII	201078	201230	152	
chrIII	208738	208879	141	N3:208806
chrIII	209585	209734	149	N3:209637
chrIII	210916	211072	156	N3:210951
chrIII	213468	213602	134	N3:213471
chrIII	215653	215790	137	N3:215679
chrIII	217960	218102	142	N3:218083
chrIII	228591	228741	150	N3:228733
chrIII	231011	231166	155	N3:231100
chrIII	234251	234402	151	N3:234286
chrIII	234921	235052	131	
chrIII	236307	236469	162	N3:236392
chrIII	238120	238291	171	N3:238307
chrIII	238120	238291	171	N3:238147
chrIII	240298	240452	154	N3:240369
chrIII	240452	240614	162	N3:240532
chrIII	242569	242719	150	N3:242631
chrIII	246007	246188	181	N3:246094
chrIII	246926	247057	131	N3:246993
chrIII	246926	247058	132	N3:246993
chrIII	248934	249085	151	N3:249001
chrIII	251728	251875	147	
chrIII	252213	252361	148	N3:252282
chrIII	252238	252397	159	N3:252282
chrIII	254663	254821	158	N3:254734
chrIII	257083	257228	145	N3:257179
chrIII	257806	257969	163	N3:257802
chrIII	258183	258325	142	N3:258264
chrIII	259144	259292	148	N3:259206
chrIII	262204	262356	152	N3:262264
chrIII	269289	269448	159	N3:269317
chrIII	272784	272929	145	N3:272830
chrIII	276672	276825	153	N3:276740
chrIII	280552	280680	128	N3:280622
chrIII	281034	281170	136	N3:281080

Chr	Start	End	Length (bp)	Nucleosome_ID
chrIII	286830	286938	108	N3:286909
chrIII	286830	286939	109	N3:286909
chrIII	286830	286940	110	N3:286909
chrIII	286830	286941	111	N3:286909
chrIII	286832	286938	106	N3:286909
chrIII	286832	286939	107	N3:286909
chrIII	286832	286955	123	N3:286909
chrIII	288517	288666	149	N3:288593
chrIII	303804	303963	159	N3:303873
chrIII	307204	307345	141	N3:307293
chrIII	307633	307788	155	
chrIII	308042	308191	149	N3:308087
chrIII	310321	310472	151	N3:310383
chrIII	310654	310803	149	N3:310728
chrIII	311798	311947	149	N3:311793
chrIII	314554	314707	153	N3:314702
chrIV	0	126	126	
chrIV	0	145	145	
chrIV	0	155	155	
chrIV	5	126	121	
chrIV	14	126	112	
chrIV	14	134	120	
chrIV	14	155	141	
chrIV	44	126	82	
chrIV	44	134	90	
chrIV	44	145	101	
chrIV	44	155	111	
chrIV	57	155	98	
chrIV	1765	1923	158	N4:1847
chrIV	6558	6652	94	N4:6650
chrIV	8786	8917	131	N4:8900
chrIV	10468	10622	154	N4:10539
chrIV	11706	11865	159	N4:11735
chrIV	12342	12498	156	N4:12435
chrIV	12922	13062	140	
chrIV	13187	13340	153	N4:13269
chrIV	15846	16000	154	N4:15932
chrIV	16215	16372	157	N4:16282
chrIV	16517	16676	159	N4:16612
chrIV	22332	22485	153	N4:22409
chrIV	22780	22921	141	N4:22851
chrIV	24904	25025	121	N4:25029
chrIV	26118	26260	142	N4:26130
chrIV	28132	28284	152	N4:28244
chrIV	28565	28649	84	
chrIV	28606	28762	156	N4:28689
chrIV	29713	29864	151	N4:29784
chrIV	30032	30191	159	N4:30113
chrIV	31789	31954	165	N4:31846
chrIV	32229	32385	156	N4:32309
chrIV	33584	33743	159	N4:33663
chrIV	34942	35098	156	N4:35008
chrIV	43249	43399	150	N4:43358
chrIV	43986	44132	146	N4:44075
chrIV	51938	52044	106	N4:51987
chrIV	55412	55541	129	
chrIV	56586	56757	171	N4:56663
chrIV	60333	60467	134	N4:60396
chrIV	61937	62077	140	N4:62069
chrIV	77124	77282	158	N4:77237
chrIV	78906	79062	156	N4:78931
chrIV	86969	87132	163	N4:87040
chrIV	90699	90847	148	N4:90745
chrIV	94580	94722	142	N4:94705
chrIV	97197	97345	148	N4:97289
chrIV	101729	101892	163	N4:101825
chrIV	103149	103279	130	N4:103192
chrIV	109515	109652	137	N4:109593
chrIV	109515	109656	141	N4:109593
chrIV	109515	109666	151	N4:109593
chrIV	109518	109656	138	N4:109593
chrIV	110479	110614	135	N4:110476
chrIV	113056	113240	184	N4:113034
chrIV	118783	118936	153	N4:118787
chrIV	121491	121639	148	N4:121562
chrIV	122619	122777	158	N4:122683
chrIV	124114	124238	124	N4:124182
chrIV	125012	125174	162	N4:125123
chrIV	126907	127064	157	N4:126899
chrIV	126907	127064	157	N4:127056
chrIV	127133	127254	121	N4:127204
chrIV	130391	130540	149	N4:130459
chrIV	134454	134627	173	N4:134518
chrIV	137744	137906	162	N4:137857
chrIV	147424	147558	134	
chrIV	148138	148259	121	N4:148212
chrIV	148138	148287	149	N4:148212
chrIV	148147	148256	109	N4:148212
chrIV	148147	148259	112	N4:148212
chrIV	148147	148287	140	N4:148212
chrIV	148150	148259	109	N4:148212
chrIV	148662	148839	177	N4:148802
chrIV	150319	150438	119	
chrIV	150791	150954	163	N4:150914
chrIV	151146	151295	149	N4:151233
chrIV	154011	154153	142	N4:154052
chrIV	156456	156616	160	N4:156533
chrIV	156781	156930	149	

Chr	Start	End	Length (bp)	Nucleosome_ID
chrIV	158096	158254	158	N4:158248
chrIV	158096	158254	158	N4:158093
chrIV	158496	158646	150	N4:158568
chrIV	162837	162965	128	N4:162831
chrIV	163802	163934	132	N4:163873
chrIV	163887	164037	150	N4:163873
chrIV	163887	164037	150	N4:164037
chrIV	164671	164820	149	N4:164814
chrIV	168971	169122	151	N4:169057
chrIV	180947	181107	160	N4:181019
chrIV	185030	185179	149	N4:185100
chrIV	185042	185156	114	N4:185100
chrIV	196778	196946	168	N4:196824
chrIV	198969	199115	146	N4:199010
chrIV	200831	200925	94	N4:200946
chrIV	202938	203097	159	N4:203012
chrIV	204891	205041	150	N4:204992
chrIV	205789	205917	128	N4:205848
chrIV	214844	215003	159	
chrIV	224519	224676	157	N4:224539
chrIV	224519	224676	157	N4:224699
chrIV	228430	228579	149	N4:228507
chrIV	230300	230458	158	N4:230409
chrIV	235048	235188	140	N4:235136
chrIV	235556	235704	148	N4:235550
chrIV	235556	235704	148	N4:235716
chrIV	240743	240895	152	N4:240819
chrIV	241083	241236	153	N4:241147
chrIV	243252	243404	152	N4:243309
chrIV	247954	248116	162	N4:247969
chrIV	249148	249302	154	
chrIV	249297	249444	147	
chrIV	253799	253940	141	
chrIV	262881	263029	148	N4:262904
chrIV	268349	268491	142	N4:268409
chrIV	269754	269886	132	N4:269794
chrIV	275399	275562	163	N4:275425
chrIV	277085	277203	118	N4:277148
chrIV	281092	281244	152	N4:281168
chrIV	282751	282862	111	N4:282817
chrIV	287334	287498	164	N4:287398
chrIV	290503	290642	139	N4:290580
chrIV	291728	291875	147	N4:291860
chrIV	294716	294818	102	N4:294818
chrIV	303242	303392	150	N4:303310
chrIV	305394	305543	149	N4:305450
chrIV	307871	308025	154	N4:307996
chrIV	308997	309152	155	N4:309056
chrIV	311358	311498	140	N4:311442
chrIV	313115	313272	157	N4:313232
chrIV	315132	315251	119	N4:315205
chrIV	316257	316381	124	N4:316392
chrIV	317932	318025	93	
chrIV	320773	320934	161	N4:320798
chrIV	324041	324187	146	N4:324118
chrIV	326087	326232	145	N4:326162
chrIV	331066	331232	166	N4:331089
chrIV	331066	331232	166	N4:331253
chrIV	336500	336644	144	N4:336586
chrIV	339003	339155	152	N4:339069
chrIV	339626	339796	170	N4:339709
chrIV	343754	343879	125	N4:343821
chrIV	344625	344793	168	N4:344682
chrIV	344904	345057	153	N4:345036
chrIV	346054	346184	130	N4:346140
chrIV	346695	346849	154	
chrIV	354236	354370	134	N4:354255
chrIV	357373	357527	154	N4:357441
chrIV	357519	357616	97	N4:357598
chrIV	367261	367414	153	N4:367365
chrIV	369734	369884	150	N4:369786
chrIV	372733	372859	126	N4:372737
chrIV	377302	377457	155	N4:377352
chrIV	377961	378102	141	N4:378096
chrIV	379551	379707	156	N4:379708
chrIV	380490	380611	121	N4:380618
chrIV	380895	381031	136	N4:380935
chrIV	383984	384136	152	N4:384003
chrIV	384406	384547	141	
chrIV	386414	386562	148	N4:386473
chrIV	389563	389664	101	N4:389614
chrIV	394898	395059	161	N4:395016
chrIV	395659	395798	139	N4:395816
chrIV	405838	405992	154	N4:405924
chrIV	406138	406284	146	
chrIV	407097	407258	161	N4:407198
chrIV	407639	407774	135	
chrIV	410001	410147	146	N4:410035
chrIV	412510	412666	156	N4:412554
chrIV	414505	414611	106	
chrIV	414505	414627	122	
chrIV	417857	418015	158	N4:418030
chrIV	418210	418363	153	N4:418242
chrIV	418328	418478	150	
chrIV	419977	420127	150	
chrIV	430775	430925	150	
chrIV	431218	431366	148	N4:431307
chrIV	433624	433778	154	N4:433704

Chr	Start	End	Length (bp)	Nucleosome_ID
chrIV	444592	444742	150	N4:444681
chrIV	447153	447303	150	N4:447172
chrIV	452119	452270	151	N4:452201
chrIV	456027	456181	154	N4:456128
chrIV	464196	464345	149	N4:464243
chrIV	465523	465668	145	N4:465537
chrIV	472028	472162	134	N4:472039
chrIV	480768	480918	150	N4:480858
chrIV	481714	481864	150	N4:481812
chrIV	481903	482061	158	N4:481994
chrIV	487395	487546	151	N4:487410
chrIV	488401	488552	151	N4:488452
chrIV	488508	488615	107	N4:488621
chrIV	488508	488632	124	N4:488621
chrIV	491144	491308	164	N4:491134
chrIV	491144	491308	164	N4:491316
chrIV	492176	492332	156	N4:492275
chrIV	494125	494275	150	N4:494221
chrIV	499197	499355	158	N4:499232
chrIV	499814	499967	153	N4:499892
chrIV	503596	503736	140	N4:503693
chrIV	503768	503924	156	
chrIV	508653	508795	142	N4:508667
chrIV	513880	514029	149	N4:513900
chrIV	517444	517573	129	
chrIV	518819	518929	110	N4:518890
chrIV	521357	521482	125	N4:521409
chrIV	522483	522574	91	N4:522532
chrIV	522691	522836	145	N4:522707
chrIV	524988	525151	163	N4:525064
chrIV	525488	525645	157	
chrIV	527030	527163	133	N4:527071
chrIV	527878	528014	136	N4:527977
chrIV	528421	528544	123	N4:528503
chrIV	528421	528548	127	N4:528503
chrIV	529656	529804	148	
chrIV	529731	529886	155	
chrIV	530359	530519	160	N4:530430
chrIV	531763	531899	136	N4:531865
chrIV	532306	532429	123	N4:532381
chrIV	532306	532433	127	N4:532381
chrIV	533541	533689	148	
chrIV	533616	533771	155	
chrIV	534244	534404	160	N4:534319
chrIV	535648	535784	136	N4:535758
chrIV	536109	536229	120	
chrIV	537426	537574	148	N4:537413
chrIV	537501	537656	155	
chrIV	540906	541052	146	N4:541006
chrIV	541731	541872	141	N4:541799
chrIV	545183	545329	146	N4:545271
chrIV	549706	549856	150	N4:549849
chrIV	549706	549856	150	N4:549694
chrIV	552005	552154	149	N4:552080
chrIV	557304	557456	152	N4:557386
chrIV	560465	560600	135	N4:560548
chrIV	570638	570778	140	N4:570621
chrIV	570638	570778	140	N4:570783
chrIV	574209	574359	150	N4:574313
chrIV	575376	575525	149	N4:575491
chrIV	585684	585843	159	N4:585698
chrIV	587983	588137	154	N4:588040
chrIV	601658	601795	137	N4:601720
chrIV	607967	608120	153	N4:608009
chrIV	615631	615766	135	N4:615640
chrIV	618044	618203	159	N4:618139
chrIV	625810	625961	151	N4:625892
chrIV	640754	640908	154	N4:640848
chrIV	643481	643635	154	N4:643559
chrIV	645695	645844	149	N4:645718
chrIV	646491	646637	146	N4:646571
chrIV	647130	647280	150	N4:647179
chrIV	648526	648683	157	N4:648585
chrIV	648994	649155	161	N4:649119
chrIV	649174	649309	135	N4:649272
chrIV	650212	650352	140	N4:650289
chrIV	650218	650352	134	N4:650289
chrIV	650630	650785	155	N4:650651
chrIV	650761	650869	108	N4:650859
chrIV	654067	654214	147	N4:654056
chrIV	654067	654214	147	N4:654214
chrIV	656839	656998	159	N4:656949
chrIV	658487	658641	154	N4:658664
chrIV	658600	658747	147	N4:658664
chrIV	662245	662401	156	N4:662311
chrIV	665084	665248	164	N4:665151
chrIV	665597	665732	135	N4:665678
chrIV	666941	667094	153	N4:667026
chrIV	666948	667109	161	N4:667026
chrIV	669497	669656	159	N4:669574
chrIV	669741	669906	165	N4:669781
chrIV	677555	677687	132	N4:677564
chrIV	682440	682595	155	N4:682517
chrIV	682928	683076	148	N4:683045
chrIV	684905	685053	148	N4:684976
chrIV	685367	685521	154	N4:685462
chrIV	685409	685539	130	N4:685462
chrIV	692976	693125	149	N4:693039

Chr	Start	End	Length (bp)	Nucleosome_ID
chrIV	699427	699543	116	N4:699492
chrIV	699429	699572	143	N4:699492
chrIV	701924	702080	156	N4:702005
chrIV	705337	705438	101	N4:705421
chrIV	705533	705696	163	
chrIV	709396	709543	147	N4:709492
chrIV	710394	710553	159	N4:710486
chrIV	710648	710758	110	N4:710780
chrIV	711680	711827	147	N4:711712
chrIV	712887	713032	145	N4:712928
chrIV	717311	717466	155	N4:717376
chrIV	723685	723835	150	
chrIV	724692	724830	138	N4:724752
chrIV	727870	728018	148	N4:727949
chrIV	729848	729954	106	
chrIV	729848	729970	122	N4:729980
chrIV	730903	730997	94	N4:730925
chrIV	734743	734893	150	N4:734827
chrIV	736895	737045	150	
chrIV	737420	737548	128	N4:737479
chrIV	737638	737793	155	N4:737705
chrIV	745330	745443	113	N4:745385
chrIV	747582	747734	152	N4:747675
chrIV	750682	750850	168	N4:750748
chrIV	760658	760811	153	N4:760826
chrIV	761833	761982	149	N4:761970
chrIV	762005	762157	152	N4:762143
chrIV	764493	764644	151	N4:764585
chrIV	766598	766749	151	N4:766690
chrIV	768253	768411	158	N4:768405
chrIV	771149	771299	150	
chrIV	773973	774101	128	
chrIV	779473	779647	174	N4:779537
chrIV	779988	780110	122	N4:779996
chrIV	785291	785441	150	N4:785366
chrIV	787217	787366	149	N4:787358
chrIV	787217	787366	149	N4:787195
chrIV	789145	789305	160	N4:789230
chrIV	800500	800661	161	N4:800519
chrIV	803067	803219	152	N4:803114
chrIV	805417	805524	107	N4:805529
chrIV	805417	805530	113	N4:805529
chrIV	810559	810694	135	N4:810661
chrIV	815236	815393	157	N4:815317
chrIV	815520	815678	158	N4:815624
chrIV	826418	826583	165	N4:826491
chrIV	827506	827652	146	N4:827588
chrIV	835382	835525	143	N4:835504
chrIV	835382	835528	146	N4:835504
chrIV	835383	835528	145	N4:835504
chrIV	835384	835528	144	N4:835504
chrIV	835385	835528	143	N4:835504
chrIV	835386	835528	142	N4:835504
chrIV	835932	836065	133	N4:835972
chrIV	836520	836671	151	N4:836563
chrIV	837322	837477	155	N4:837482
chrIV	838477	838608	131	N4:838553
chrIV	843960	844136	176	N4:844047
chrIV	844482	844623	141	N4:844544
chrIV	844482	844649	167	N4:844544
chrIV	850466	850631	165	N4:850636
chrIV	850466	850631	165	N4:850477
chrIV	857181	857346	165	
chrIV	861657	861796	139	N4:861762
chrIV	863651	863788	137	N4:863721
chrIV	866143	866295	152	N4:866178
chrIV	867460	867598	138	N4:867531
chrIV	870210	870347	137	N4:870239
chrIV	872409	872511	102	N4:872520
chrIV	873832	873980	148	
chrIV	874621	874762	141	
chrIV	875359	875502	143	N4:875420
chrIV	876645	876791	146	N4:876701
chrIV	877438	877587	149	N4:877571
chrIV	878298	878451	153	N4:878280
chrIV	878496	878645	149	N4:878510
chrIV	879292	879438	146	N4:879370
chrIV	881327	881484	157	N4:881391
chrIV	881795	881956	161	
chrIV	881975	882110	135	N4:882066
chrIV	883013	883153	140	N4:883091
chrIV	883019	883153	134	N4:883091
chrIV	883431	883586	155	N4:883463
chrIV	883562	883670	108	
chrIV	883696	883848	152	N4:883720
chrIV	884819	884969	150	N4:884882
chrIV	888390	888567	177	N4:888453
chrIV	889668	889827	159	N4:889734
chrIV	890834	890988	154	N4:890967
chrIV	891389	891533	144	N4:891510
chrIV	891986	892153	167	N4:892055
chrIV	896334	896482	148	N4:896422
chrIV	897629	897776	147	N4:897659
chrIV	899028	899177	149	N4:899049
chrIV	906466	906593	127	N4:906545
chrIV	907937	908075	138	
chrIV	908538	908667	129	N4:908599
chrIV	909620	909773	153	N4:909630

Chr	Start	End	Length (bp)	Nucleosome_ID
chrIV	915796	915980	184	N4:915793
chrIV	917617	917765	148	N4:917653
chrIV	924934	925100	166	N4:925073
chrIV	926425	926570	145	N4:926519
chrIV	927161	927289	128	N4:927242
chrIV	928961	929115	154	N4:929074
chrIV	930238	930383	145	N4:930295
chrIV	931111	931260	149	N4:931170
chrIV	933440	933600	160	N4:933517
chrIV	944365	944524	159	N4:944467
chrIV	944403	944557	154	N4:944467
chrIV	948002	948171	169	N4:948157
chrIV	949233	949387	154	N4:949302
chrIV	955015	955163	148	N4:955072
chrIV	957192	957317	125	N4:957237
chrIV	957192	957321	129	N4:957237
chrIV	957196	957298	102	N4:957237
chrIV	962120	962274	154	N4:962102
chrIV	962406	962555	149	N4:962480
chrIV	965842	965991	149	N4:965942
chrIV	968475	968604	129	N4:968597
chrIV	971192	971329	137	N4:971253
chrIV	975387	975508	121	N4:975430
chrIV	979606	979759	153	N4:979673
chrIV	980738	980889	151	N4:980809
chrIV	981759	981861	102	N4:981871
chrIV	983182	983330	148	
chrIV	984709	984852	143	N4:984756
chrIV	985995	986141	146	
chrIV	986788	986937	149	N4:986912
chrIV	987343	987492	149	N4:987353
chrIV	987657	987771	114	N4:987788
chrIV	988353	988503	150	N4:988403
chrIV	989662	989806	144	N4:989808
chrIV	989749	989906	157	N4:989808
chrIV	990217	990378	161	N4:990348
chrIV	990397	990532	135	N4:990499
chrIV	991435	991575	140	N4:991512
chrIV	991441	991575	134	N4:991512
chrIV	991853	992008	155	N4:991879
chrIV	991984	992092	108	N4:992082
chrIV	992133	992282	149	
chrIV	994735	994883	148	N4:994783
chrIV	1001383	1001529	146	N4:1001542
chrIV	1001383	1001529	146	N4:1001379
chrIV	1005517	1005664	147	N4:1005589
chrIV	1005876	1006022	146	N4:1005930
chrIV	1012282	1012417	135	N4:1012368
chrIV	1016514	1016662	148	N4:1016582
chrIV	1019255	1019404	149	N4:1019326
chrIV	1021134	1021284	150	N4:1021147
chrIV	1021134	1021284	150	N4:1021302
chrIV	1025226	1025374	148	N4:1025251
chrIV	1028806	1028950	144	N4:1028803
chrIV	1039896	1040063	167	N4:1039922
chrIV	1042109	1042252	143	N4:1042173
chrIV	1052004	1052154	150	N4:1052080
chrIV	1055603	1055756	153	N4:1055666
chrIV	1058574	1058755	181	N4:1058759
chrIV	1058574	1058755	181	N4:1058587
chrIV	1063264	1063420	156	N4:1063318
chrIV	1066695	1066823	128	N4:1066763
chrIV	1075797	1075953	156	N4:1075869
chrIV	1079066	1079219	153	N4:1079155
chrIV	1079249	1079403	154	N4:1079306
chrIV	1080074	1080246	172	N4:1080115
chrIV	1090868	1091040	172	N4:1090903
chrIV	1091309	1091450	141	
chrIV	1096304	1096427	123	
chrIV	1096404	1096559	155	N4:1096525
chrIV	1096837	1096971	134	N4:1096900
chrIV	1096837	1096977	140	N4:1096900
chrIV	1097880	1098015	135	N4:1097914
chrIV	1098034	1098195	161	N4:1098134
chrIV	1098506	1098663	157	N4:1098582
chrIV	1098997	1099126	129	
chrIV	1099909	1100059	150	N4:1100005
chrIV	1100669	1100827	158	N4:1100752
chrIV	1102545	1102694	149	N4:1102665
chrIV	1102953	1103100	147	N4:1102998
chrIV	1107156	1107303	147	N4:1107223
chrIV	1109551	1109723	172	N4:1109646
chrIV	1112644	1112796	152	N4:1112718
chrIV	1117237	1117387	150	N4:1117306
chrIV	1121058	1121205	147	N4:1121186
chrIV	1121801	1121948	147	N4:1121812
chrIV	1124473	1124641	168	N4:1124564
chrIV	1128248	1128395	147	N4:1128331
chrIV	1137826	1137974	148	N4:1137842
chrIV	1140412	1140528	116	N4:1140541
chrIV	1144636	1144783	147	N4:1144674
chrIV	1154940	1155065	125	N4:1155020
chrIV	1155027	1155183	156	N4:1155020
chrIV	1155195	1155331	136	N4:1155235
chrIV	1158507	1158654	147	N4:1158572
chrIV	1160332	1160457	125	N4:1160332
chrIV	1160419	1160575	156	N4:1160485
chrIV	1160587	1160723	136	N4:1160638

Chr	Start	End	Length (bp)	Nucleosome_ID
chrIV	1164215	1164366	151	N4:1164258
chrIV	1166734	1166868	134	N4:1166854
chrIV	1169216	1169370	154	N4:1169282
chrIV	1170479	1170639	160	N4:1170579
chrIV	1170874	1171037	163	N4:1171049
chrIV	1170874	1171037	163	N4:1170900
chrIV	1171736	1171886	150	N4:1171844
chrIV	1175589	1175742	153	N4:1175671
chrIV	1176500	1176647	147	N4:1176576
chrIV	1179339	1179509	170	N4:1179429
chrIV	1185766	1185913	147	N4:1185835
chrIV	1186635	1186765	130	N4:1186749
chrIV	1187842	1187997	155	N4:1187892
chrIV	1199637	1199781	144	N4:1199716
chrIV	1199946	1200093	147	N4:1200030
chrIV	1202429	1202587	158	N4:1202473
chrIV	1205211	1205359	148	N4:1205237
chrIV	1207063	1207212	149	N4:1207202
chrIV	1207237	1207360	123	
chrIV	1207337	1207492	155	N4:1207459
chrIV	1207770	1207904	134	N4:1207827
chrIV	1207770	1207910	140	N4:1207827
chrIV	1207966	1208117	151	N4:1208017
chrIV	1207976	1208121	145	N4:1208017
chrIV	1207994	1208145	151	N4:1208017
chrIV	1207996	1208145	149	N4:1208017
chrIV	1208248	1208402	154	N4:1208380
chrIV	1208282	1208426	144	N4:1208380
chrIV	1208813	1208948	135	N4:1208846
chrIV	1208967	1209128	161	N4:1209000
chrIV	1209439	1209596	157	N4:1209520
chrIV	1209930	1210059	129	N4:1210072
chrIV	1210842	1210992	150	N4:1210937
chrIV	1211574	1211688	114	N4:1211673
chrIV	1215599	1215745	146	N4:1215661
chrIV	1216176	1216327	151	
chrIV	1217346	1217492	146	N4:1217374
chrIV	1218508	1218653	145	N4:1218588
chrIV	1221088	1221239	151	N4:1221154
chrIV	1225710	1225871	161	N4:1225704
chrIV	1225924	1226071	147	N4:1225999
chrIV	1227651	1227806	155	N4:1227756
chrIV	1238397	1238557	160	N4:1238463
chrIV	1240257	1240406	149	N4:1240306
chrIV	1240385	1240526	141	N4:1240462
chrIV	1244524	1244687	163	N4:1244612
chrIV	1245862	1245987	125	N4:1245846
chrIV	1251539	1251695	156	N4:1251612
chrIV	1259546	1259668	122	N4:1259621
chrIV	1265859	1266020	161	N4:1265947
chrIV	1266398	1266553	155	N4:1266476
chrIV	1267550	1267699	149	N4:1267615
chrIV	1269091	1269198	107	N4:1269188
chrIV	1269091	1269209	118	N4:1269188
chrIV	1272800	1272981	181	N4:1272869
chrIV	1277856	1277982	126	N4:1277949
chrIV	1278160	1278320	160	N4:1278264
chrIV	1287754	1287911	157	N4:1287812
chrIV	1288372	1288504	132	
chrIV	1291651	1291799	148	N4:1291819
chrIV	1293635	1293774	139	N4:1293718
chrIV	1298059	1298223	164	N4:1298152
chrIV	1300809	1300966	157	N4:1300924
chrIV	1300809	1300970	161	N4:1300924
chrIV	1300843	1301002	159	N4:1300924
chrIV	1300844	1300970	126	N4:1300924
chrIV	1300844	1300987	143	N4:1300924
chrIV	1302267	1302373	106	N4:1302245
chrIV	1302267	1302383	116	N4:1302245
chrIV	1308150	1308260	110	N4:1308222
chrIV	1308150	1308309	159	N4:1308222
chrIV	1308153	1308309	156	N4:1308222
chrIV	1308185	1308314	129	N4:1308222
chrIV	1308191	1308309	118	N4:1308222
chrIV	1308196	1308309	113	N4:1308222
chrIV	1308234	1308344	110	N4:1308222
chrIV	1308280	1308442	162	N4:1308370
chrIV	1308280	1308477	197	N4:1308370
chrIV	1308478	1308605	127	N4:1308546
chrIV	1308858	1308982	124	N4:1308860
chrIV	1312653	1312780	127	N4:1312676
chrIV	1312778	1312924	146	N4:1312823
chrIV	1324502	1324680	178	N4:1324612
chrIV	1324730	1324882	152	N4:1324791
chrIV	1329033	1329189	156	
chrIV	1331269	1331431	162	N4:1331328
chrIV	1332261	1332409	148	N4:1332317
chrIV	1334150	1334293	143	N4:1334177
chrIV	1334858	1335004	146	N4:1334915
chrIV	1335220	1335376	156	N4:1335248
chrIV	1335220	1335376	156	N4:1335399
chrIV	1337771	1337882	111	N4:1337823
chrIV	1341919	1342069	150	N4:1342003
chrIV	1354730	1354858	128	N4:1354868
chrIV	1356736	1356889	153	
chrIV	1359819	1359977	158	
chrIV	1360674	1360822	148	N4:1360796
chrIV	1361305	1361412	107	N4:1361296

Chr	Start	End	Length (bp)	Nucleosome_ID
chrIV	1361305	1361420	115	N4:1361443
chrIV	1361305	1361420	115	N4:1361296
chrIV	1364336	1364488	152	N4:1364372
chrIV	1364512	1364671	159	N4:1364657
chrIV	1365728	1365875	147	N4:1365799
chrIV	1365746	1365909	163	N4:1365799
chrIV	1367419	1367567	148	N4:1367482
chrIV	1367927	1368075	148	N4:1367994
chrIV	1372245	1372378	133	N4:1372326
chrIV	1376748	1376898	150	N4:1376802
chrIV	1382369	1382521	152	N4:1382446
chrIV	1387683	1387833	150	N4:1387776
chrIV	1390839	1391009	170	N4:1390850
chrIV	1391435	1391580	145	N4:1391591
chrIV	1391940	1392060	120	
chrIV	1397500	1397655	155	
chrIV	1398087	1398256	169	N4:1398124
chrIV	1406033	1406179	146	N4:1406110
chrIV	1407803	1407943	140	N4:1407944
chrIV	1407803	1407943	140	N4:1407780
chrIV	1408060	1408202	142	N4:1408111
chrIV	1408065	1408226	161	N4:1408111
chrIV	1408340	1408489	149	
chrIV	1409678	1409825	147	N4:1409695
chrIV	1413171	1413310	139	N4:1413232
chrIV	1420451	1420608	157	N4:1420526
chrIV	1420630	1420804	174	N4:1420827
chrIV	1420630	1420804	174	N4:1420679
chrIV	1420634	1420804	170	N4:1420827
chrIV	1420634	1420804	170	N4:1420679
chrIV	1424831	1424973	142	N4:1424916
chrIV	1428254	1428414	160	N4:1428344
chrIV	1430994	1431130	136	N4:1431036
chrIV	1433662	1433813	151	N4:1433787
chrIV	1447757	1447907	150	N4:1447835
chrIV	1454001	1454153	152	N4:1454073
chrIV	1460987	1461146	159	N4:1461050
chrIV	1461681	1461828	147	N4:1461801
chrIV	1462856	1462994	138	
chrIV	1462967	1463131	164	N4:1463035
chrIV	1464532	1464669	137	N4:1464622
chrIV	1465805	1465959	154	N4:1465878
chrIV	1466131	1466277	146	N4:1466195
chrIV	1473970	1474117	147	N4:1474065
chrIV	1477488	1477647	159	N4:1477526
chrIV	1478568	1478697	129	N4:1478612
chrIV	1479228	1479388	160	N4:1479283
chrIV	1479727	1479884	157	N4:1479790
chrIV	1481305	1481467	162	N4:1481450
chrIV	1489675	1489832	157	N4:1489795
chrIV	1495628	1495753	125	N4:1495663
chrIV	1496435	1496575	140	N4:1496536
chrIV	1497627	1497776	149	N4:1497727
chrIV	1498743	1498886	143	N4:1498864
chrIV	1499148	1499307	159	N4:1499221
chrIV	1503439	1503593	154	N4:1503589
chrIV	1505128	1505252	124	N4:1505200
chrIV	1507157	1507304	147	N4:1507240
chrIV	1512418	1512571	153	N4:1512551
chrIV	1512829	1512960	131	N4:1512870
chrIV	1526039	1526194	155	N4:1526079
chrIV	1527054	1527225	171	N4:1527142
chrIV	1527204	1527351	147	
chrIV	1527360	1527490	130	N4:1527407
chrIV	1527741	1527877	136	N4:1527764
chrIV	1528122	1528284	162	N4:1528239
chrIV	1528223	1528378	155	N4:1528239
chrIV	1528357	1528494	137	N4:1528410
chrIV	1528652	1528795	143	N4:1528689
chrIV	1528700	1528825	125	N4:1528689
chrIV	1528925	1529077	152	N4:1529021
chrIV	1528998	1529142	144	N4:1529021
chrIV	1529095	1529230	135	N4:1529177
chrIV	1529095	1529282	187	N4:1529177
chrIV	1529138	1529230	92	N4:1529177
chrIV	1529138	1529282	144	N4:1529177
chrIV	1529477	1529629	152	N4:1529532
chrIV	1529778	1529908	130	N4:1529917
chrIV	1529916	1530077	161	N4:1529917
chrIV	1530973	1531125	152	N4:1531041
chrIV	1531313	1531464	151	N4:1531365
chrIV	1531313	1531484	171	N4:1531365
chrIX	1597	1749	152	N9:1629
chrIX	1686	1802	116	N9:1790
chrIX	1686	1821	135	N9:1790
chrIX	1686	1838	152	N9:1790
chrIX	1710	1802	92	N9:1790
chrIX	1710	1811	101	N9:1790
chrIX	1710	1821	111	N9:1790
chrIX	1710	1838	128	N9:1790
chrIX	1710	1857	147	N9:1790
chrIX	1746	1838	92	N9:1790
chrIX	2215	2352	137	N9:2320
chrIX	2382	2529	147	N9:2535
chrIX	2382	2530	148	N9:2535
chrIX	2382	2592	210	N9:2535
chrIX	2383	2530	147	N9:2535
chrIX	2385	2530	145	N9:2535

Chr	Start	End	Length (bp)	Nucleosome_ID
chrIX	2397	2530	133	N9:2535
chrIX	2437	2530	93	N9:2535
chrIX	2437	2592	155	N9:2535
chrIX	2558	2688	130	N9:2535
chrIX	2879	3032	153	N9:2921
chrIX	3324	3468	144	N9:3423
chrIX	3531	3658	127	N9:3591
chrIX	4088	4243	155	N9:4203
chrIX	4805	4968	163	N9:4855
chrIX	5216	5367	151	
chrIX	5429	5588	159	N9:5453
chrIX	9290	9441	151	N9:9376
chrIX	10228	10371	143	N9:10309
chrIX	11460	11611	151	N9:11554
chrIX	12345	12490	145	
chrIX	12910	13059	149	
chrIX	14208	14357	149	N9:14236
chrIX	16078	16185	107	N9:16064
chrIX	25656	25806	150	N9:25676
chrIX	32330	32492	162	N9:32393
chrIX	33763	33917	154	N9:33762
chrIX	35803	35935	132	N9:35809
chrIX	41284	41431	147	N9:41356
chrIX	48410	48570	160	N9:48477
chrIX	55336	55486	150	N9:55401
chrIX	55497	55655	158	N9:55554
chrIX	55498	55655	157	N9:55554
chrIX	55500	55655	155	N9:55554
chrIX	56537	56665	128	N9:56598
chrIX	56786	56893	107	N9:56789
chrIX	56786	56905	119	N9:56789
chrIX	56791	56905	114	N9:56789
chrIX	59003	59166	163	N9:59158
chrIX	59003	59166	163	N9:58990
chrIX	59591	59741	150	N9:59671
chrIX	60731	60881	150	N9:60802
chrIX	62677	62823	146	N9:62723
chrIX	70968	71118	150	N9:71141
chrIX	77046	77204	158	N9:77127
chrIX	78123	78268	145	N9:78189
chrIX	80413	80548	135	N9:80482
chrIX	89142	89281	139	N9:89221
chrIX	91580	91733	153	N9:91642
chrIX	93328	93477	149	N9:93388
chrIX	96377	96489	112	N9:96391
chrIX	97100	97255	155	N9:97083
chrIX	101159	101314	155	N9:101310
chrIX	101159	101314	155	N9:101155
chrIX	109382	109534	152	N9:109439
chrIX	122918	123072	154	N9:122984
chrIX	123420	123580	160	N9:123402
chrIX	123420	123580	160	N9:123567
chrIX	123668	123818	150	N9:123750
chrIX	127761	127867	106	N9:127744
chrIX	127761	127880	119	N9:127744
chrIX	129250	129396	146	N9:129359
chrIX	132115	132265	150	N9:132195
chrIX	140470	140605	135	N9:140507
chrIX	140794	140945	151	N9:140849
chrIX	145476	145609	133	
chrIX	145755	145902	147	N9:145791
chrIX	149396	149568	172	N9:149494
chrIX	150158	150305	147	N9:150234
chrIX	154400	154561	161	N9:154551
chrIX	157942	158056	114	N9:157996
chrIX	158500	158651	151	
chrIX	159528	159680	152	N9:159539
chrIX	160370	160515	145	N9:160446
chrIX	164945	165092	147	N9:165040
chrIX	167654	167783	129	
chrIX	170117	170264	147	N9:170186
chrIX	175448	175623	175	N9:175501
chrIX	175932	176081	149	N9:175976
chrIX	176147	176286	139	N9:176155
chrIX	178557	178730	173	N9:178691
chrIX	182864	183017	153	N9:182946
chrIX	190132	190312	180	N9:190192
chrIX	190978	191126	148	N9:191107
chrIX	192456	192591	135	N9:192542
chrIX	192983	193089	106	N9:193029
chrIX	192983	193098	115	N9:193029
chrIX	193702	193858	156	N9:193815
chrIX	196790	196938	148	N9:196869
chrIX	200251	200397	146	N9:200295
chrIX	205441	205561	120	
chrIX	208655	208802	147	N9:208742
chrIX	212863	213023	160	N9:212982
chrIX	213578	213684	106	
chrIX	213578	213686	108	
chrIX	213578	213701	123	
chrIX	213866	214017	151	N9:213871
chrIX	213866	214017	151	N9:214029
chrIX	217876	218011	135	N9:217888
chrIX	218740	218900	160	N9:218850
chrIX	223366	223481	115	N9:223420
chrIX	224421	224561	140	N9:224566
chrIX	236168	236319	151	N9:236230
chrIX	240288	240448	160	N9:240358

Chr	Start	End	Length (bp)	Nucleosome_ID
chrIX	241031	241216	185	N9:241153
chrIX	241349	241481	132	
chrIX	244655	244803	148	N9:244726
chrIX	244820	244976	156	N9:244898
chrIX	244822	244983	161	N9:244898
chrIX	247146	247297	151	N9:247208
chrIX	249643	249773	130	N9:249728
chrIX	254780	254919	139	N9:254874
chrIX	255472	255626	154	N9:255553
chrIX	255629	255772	143	N9:255778
chrIX	255629	255776	147	N9:255778
chrIX	258137	258291	154	N9:258298
chrIX	258137	258291	154	N9:258138
chrIX	263024	263196	172	N9:263062
chrIX	265122	265273	151	N9:265235
chrIX	265238	265355	117	N9:265235
chrIX	270073	270226	153	N9:270153
chrIX	271691	271837	146	N9:271766
chrIX	280995	281156	161	N9:281073
chrIX	282582	282731	149	N9:282678
chrIX	283375	283539	164	N9:283458
chrIX	285840	285993	153	N9:285843
chrIX	285840	285993	153	N9:286010
chrIX	286233	286373	140	
chrIX	287987	288121	134	N9:288045
chrIX	289697	289831	134	N9:289737
chrIX	289822	289971	149	N9:289899
chrIX	292476	292604	128	N9:292512
chrIX	296896	297052	156	N9:297004
chrIX	299139	299300	161	N9:299210
chrIX	301522	301672	150	N9:301595
chrIX	301692	301851	159	N9:301782
chrIX	301709	301857	148	N9:301782
chrIX	306823	306944	121	N9:306927
chrIX	309339	309491	152	N9:309417
chrIX	309860	310002	142	N9:309904
chrIX	314424	314578	154	
chrIX	320445	320583	138	N9:320481
chrIX	331176	331345	169	N9:331307
chrIX	332149	332296	147	N9:332195
chrIX	333705	333871	166	N9:333780
chrIX	338938	339077	139	N9:338970
chrIX	339456	339607	151	N9:339523
chrIX	343521	343669	148	N9:343647
chrIX	345865	346018	153	N9:345939
chrIX	346400	346566	166	N9:346455
chrIX	352082	352233	151	N9:352150
chrIX	360987	361138	151	N9:361056
chrIX	361485	361638	153	N9:361563
chrIX	364237	364386	149	N9:364329
chrIX	365450	365589	139	N9:365504
chrIX	371073	371217	144	N9:371134
chrIX	377621	377774	153	N9:377728
chrIX	378406	378536	130	N9:378506
chrIX	380201	380307	106	N9:380285
chrIX	380201	380310	109	N9:380285
chrIX	381638	381781	143	N9:381710
chrIX	382286	382451	165	N9:382299
chrIX	386066	386219	153	N9:386093
chrIX	386767	386910	143	N9:386861
chrIX	387904	388014	110	N9:387935
chrIX	388900	389051	151	N9:389018
chrIX	390177	390342	165	N9:390256
chrIX	390386	390532	146	N9:390414
chrIX	392788	392939	151	N9:392855
chrIX	396135	396302	167	N9:396197
chrIX	396795	396922	127	N9:396865
chrIX	405278	405390	112	N9:405412
chrIX	407378	407518	140	N9:407408
chrIX	407520	407679	159	N9:407581
chrIX	409036	409181	145	N9:409099
chrIX	420519	420625	106	N9:420590
chrIX	425922	426069	147	N9:426001
chrIX	433552	433722	170	N9:433692
chrIX	438504	438651	147	N9:438544
chrIX	438570	438711	141	
chrIX	439579	439688	109	N9:439641
chrIX	439796	439887	91	N9:439845
chrV	1165	1320	155	
chrV	1854	1981	127	N5:1841
chrV	3000	3144	144	N5:3094
chrV	3648	3785	137	N5:3709
chrV	3764	3919	155	N5:3872
chrV	4024	4184	160	N5:4031
chrV	4831	4982	151	N5:4948
chrV	7115	7272	157	N5:7242
chrV	9760	9930	170	
chrV	11061	11170	109	N5:11123
chrV	23046	23205	159	N5:23195
chrV	26697	26869	172	N5:26684
chrV	26697	26869	172	N5:26878
chrV	27053	27184	131	N5:27040
chrV	28903	29024	121	N5:29008
chrV	32832	32992	160	N5:32875
chrV	41648	41754	106	N5:41658
chrV	41648	41774	126	N5:41658
chrV	41648	41782	134	N5:41658
chrV	41648	41785	137	N5:41658

Chr	Start	End	Length (bp)	Nucleosome_ID
chrV	41648	41795	147	N5:41813
chrV	41648	41795	147	N5:41658
chrV	41648	41797	149	N5:41813
chrV	41648	41797	149	N5:41658
chrV	41648	41798	150	N5:41813
chrV	41648	41798	150	N5:41658
chrV	41648	41799	151	N5:41813
chrV	41648	41799	151	N5:41658
chrV	41648	41800	152	N5:41813
chrV	41648	41800	152	N5:41658
chrV	41648	41801	153	N5:41813
chrV	41648	41801	153	N5:41658
chrV	41648	41802	154	N5:41813
chrV	41648	41802	154	N5:41658
chrV	41649	41802	153	N5:41813
chrV	41649	41802	153	N5:41658
chrV	41650	41802	152	N5:41813
chrV	41650	41802	152	N5:41658
chrV	41651	41802	151	N5:41813
chrV	41651	41802	151	N5:41658
chrV	41652	41802	150	N5:41813
chrV	41652	41802	150	N5:41658
chrV	41653	41802	149	N5:41813
chrV	41653	41802	149	N5:41658
chrV	41654	41802	148	N5:41813
chrV	41654	41802	148	N5:41658
chrV	41658	41802	144	N5:41813
chrV	41658	41802	144	N5:41658
chrV	42773	42909	136	N5:42882
chrV	44013	44174	161	N5:44097
chrV	51922	52070	148	N5:52074
chrV	56549	56680	131	N5:56569
chrV	58655	58795	140	N5:58725
chrV	63504	63668	164	N5:63566
chrV	64024	64155	131	N5:64053
chrV	65475	65628	153	N5:65581
chrV	69437	69601	164	N5:69487
chrV	71549	71648	99	N5:71615
chrV	72028	72188	160	N5:72094
chrV	78159	78308	149	N5:78154
chrV	78159	78308	149	N5:78324
chrV	78447	78544	97	N5:78484
chrV	78782	78922	140	N5:78847
chrV	79095	79249	154	N5:79182
chrV	83616	83770	154	N5:83673
chrV	84397	84539	142	N5:84467
chrV	87005	87158	153	N5:87089
chrV	92533	92664	131	N5:92614
chrV	94783	94926	143	N5:94888
chrV	96167	96341	174	N5:96189
chrV	105359	105516	157	N5:105459
chrV	105504	105653	149	N5:105613
chrV	106502	106638	136	N5:106562
chrV	112707	112854	147	N5:112767
chrV	114004	114167	163	N5:114084
chrV	116119	116272	153	N5:116132
chrV	116657	116785	128	N5:116788
chrV	119860	120019	159	N5:119994
chrV	121418	121572	154	N5:121505
chrV	127589	127741	152	N5:127639
chrV	129771	129919	148	N5:129920
chrV	132213	132348	135	N5:132209
chrV	134881	135033	152	N5:134908
chrV	137057	137219	162	N5:137135
chrV	137099	137245	146	N5:137135
chrV	143216	143369	153	N5:143330
chrV	146039	146192	153	N5:146118
chrV	147368	147525	157	N5:147443
chrV	147862	148011	149	N5:147935
chrV	150905	151028	123	N5:150967
chrV	154668	154823	155	N5:154737
chrV	154964	155109	145	N5:155071
chrV	156735	156886	151	N5:156804
chrV	157741	157900	159	N5:157806
chrV	158361	158517	156	N5:158435
chrV	158999	159169	170	N5:159105
chrV	161114	161267	153	N5:161151
chrV	162828	162978	150	N5:162902
chrV	162990	163135	145	N5:163066
chrV	163162	163326	164	N5:163230
chrV	164105	164236	131	N5:164174
chrV	165644	165751	107	N5:165694
chrV	165645	165751	106	N5:165694
chrV	165645	165753	108	N5:165694
chrV	165645	165770	125	N5:165694
chrV	169801	169967	166	N5:169892
chrV	170735	170894	159	N5:170840
chrV	175783	175962	179	N5:175893
chrV	177369	177538	169	N5:177535
chrV	177369	177538	169	N5:177381
chrV	177766	177914	148	N5:177821
chrV	179498	179607	109	N5:179488
chrV	179498	179665	167	N5:179488
chrV	179498	179665	167	N5:179670
chrV	179498	179666	168	N5:179488
chrV	179498	179666	168	N5:179670
chrV	179498	179667	169	N5:179488
chrV	179498	179667	169	N5:179670

Chr	Start	End	Length (bp)	Nucleosome_ID
chrV	179498	179669	171	N5:179488
chrV	179498	179669	171	N5:179670
chrV	179498	179670	172	N5:179488
chrV	179498	179670	172	N5:179670
chrV	179499	179670	171	N5:179488
chrV	179499	179670	171	N5:179670
chrV	179502	179670	168	N5:179488
chrV	179502	179670	168	N5:179670
chrV	180478	180630	152	N5:180475
chrV	180478	180630	152	N5:180645
chrV	182865	183029	164	N5:182948
chrV	186698	186836	138	N5:186791
chrV	189568	189720	152	N5:189634
chrV	190625	190773	148	N5:190729
chrV	193660	193792	132	N5:193731
chrV	194009	194135	126	
chrV	194030	194163	133	N5:194161
chrV	195885	196040	155	N5:195969
chrV	198265	198403	138	N5:198244
chrV	201379	201531	152	N5:201422
chrV	201380	201531	151	N5:201422
chrV	201382	201531	149	N5:201422
chrV	201383	201531	148	N5:201422
chrV	206462	206572	110	
chrV	207496	207646	150	N5:207609
chrV	212628	212767	139	
chrV	213547	213709	162	N5:213639
chrV	215300	215450	150	N5:215374
chrV	220704	220853	149	N5:220767
chrV	226712	226861	149	N5:226775
chrV	227455	227599	144	N5:227578
chrV	234231	234385	154	N5:234301
chrV	238011	238157	146	N5:238087
chrV	239144	239298	154	N5:239207
chrV	250567	250727	160	N5:250698
chrV	250628	250757	129	N5:250698
chrV	253010	253142	132	N5:253162
chrV	255896	256035	139	N5:256029
chrV	258267	258412	145	N5:258341
chrV	263170	263306	136	
chrV	268138	268266	128	N5:268214
chrV	271754	271916	162	N5:271765
chrV	273268	273395	127	N5:273323
chrV	274395	274545	150	N5:274471
chrV	279045	279197	152	N5:279105
chrV	282333	282495	162	N5:282372
chrV	286305	286436	131	N5:286328
chrV	293237	293387	150	N5:293318
chrV	293855	294005	150	N5:293929
chrV	298879	299018	139	N5:298928
chrV	299205	299328	123	N5:299273
chrV	299678	299831	153	N5:299784
chrV	301505	301647	142	N5:301602
chrV	314667	314817	150	N5:314812
chrV	315905	316063	158	N5:315980
chrV	320077	320234	157	N5:320131
chrV	321656	321815	159	N5:321825
chrV	321656	321815	159	N5:321662
chrV	323513	323661	148	N5:323606
chrV	324743	324895	152	N5:324823
chrV	324904	325052	148	N5:324975
chrV	325348	325499	151	N5:325472
chrV	328966	329128	162	N5:329104
chrV	331009	331174	165	N5:331055
chrV	333088	333236	148	N5:333122
chrV	340240	340377	137	N5:340278
chrV	340798	340946	148	N5:340808
chrV	341934	342080	146	
chrV	349486	349586	100	
chrV	351515	351672	157	N5:351572
chrV	352323	352478	155	N5:352405
chrV	360556	360703	147	N5:360635
chrV	360556	360709	153	N5:360635
chrV	360596	360757	161	N5:360635
chrV	361101	361246	145	N5:361124
chrV	364550	364707	157	N5:364627
chrV	369752	369900	148	
chrV	370967	371076	109	N5:371022
chrV	372915	373052	137	N5:373029
chrV	373805	373958	153	N5:373887
chrV	376017	376164	147	N5:376149
chrV	376371	376519	148	
chrV	376832	376976	144	N5:376938
chrV	377517	377651	134	N5:377578
chrV	378722	378880	158	N5:378804
chrV	389589	389739	150	N5:389641
chrV	392068	392208	140	N5:392146
chrV	408769	408926	157	N5:408837
chrV	411385	411539	154	N5:411472
chrV	421211	421362	151	N5:421329
chrV	426798	426949	151	
chrV	426821	426975	154	
chrV	428076	428230	154	N5:428133
chrV	429069	429212	143	
chrV	434847	434995	148	N5:434827
chrV	440738	440898	160	N5:440906
chrV	444332	444446	114	N5:444468
chrV	445028	445178	150	N5:445075

Chr	Start	End	Length (bp)	Nucleosome_ID
chrV	446200	446349	149	N5:446313
chrV	446424	446581	157	N5:446484
chrV	446610	446760	150	N5:446692
chrV	446614	446760	146	N5:446692
chrV	447072	447207	135	N5:447121
chrV	447594	447738	144	N5:447622
chrV	447618	447772	154	N5:447622
chrV	447875	448026	151	N5:447970
chrV	447899	448044	145	N5:447970
chrV	447903	448054	151	N5:447970
chrV	448110	448250	140	N5:448190
chrV	448116	448250	134	N5:448190
chrV	448528	448683	155	N5:448518
chrV	448659	448767	108	N5:448761
chrV	448808	448957	149	
chrV	450553	450702	149	N5:450631
chrV	451474	451643	169	N5:451553
chrV	456731	456859	128	N5:456821
chrV	458282	458446	164	
chrV	458920	459063	143	N5:458935
chrV	458920	459063	143	N5:459085
chrV	460202	460309	107	N5:460198
chrV	460202	460330	128	N5:460198
chrV	460206	460309	103	N5:460198
chrV	465104	465256	152	N5:465150
chrV	466895	467059	164	N5:466980
chrV	471982	472140	158	N5:472119
chrV	472402	472551	149	N5:472474
chrV	472672	472832	160	N5:472741
chrV	472989	473145	156	N5:473075
chrV	473919	474069	150	N5:473967
chrV	481969	482118	149	
chrV	488812	488981	169	N5:488949
chrV	489802	489965	163	N5:489855
chrV	493537	493686	149	N5:493561
chrV	495710	495860	150	N5:495765
chrV	496172	496307	135	N5:496222
chrV	497210	497350	140	N5:497289
chrV	497216	497350	134	N5:497289
chrV	497628	497783	155	N5:497650
chrV	497759	497867	108	N5:497866
chrV	497893	498045	152	
chrV	501813	501975	162	N5:501881
chrV	502108	502246	138	N5:502208
chrV	503589	503721	132	N5:503607
chrV	504504	504656	152	N5:504574
chrV	506128	506280	152	N5:506233
chrV	508434	508572	138	
chrV	508812	508941	129	N5:508869
chrV	517240	517402	162	N5:517367
chrV	522575	522735	160	N5:522650
chrV	525612	525757	145	N5:525693
chrV	528789	528933	144	N5:528845
chrV	536553	536715	162	N5:536626
chrV	538015	538173	158	N5:538060
chrV	540818	540968	150	N5:540902
chrV	541050	541210	160	N5:541230
chrV	541050	541210	160	N5:541076
chrV	541616	541757	141	N5:541694
chrV	541626	541757	131	N5:541694
chrV	541626	541777	151	N5:541694
chrV	542716	542874	158	N5:542824
chrV	544584	544725	141	N5:544632
chrV	560594	560752	158	N5:560681
chrV	560787	560934	147	N5:560849
chrV	561033	561174	141	N5:561031
chrV	561033	561174	141	N5:561188
chrV	571735	571876	141	N5:571819
chrV	572939	573101	162	N5:573065
chrV	573040	573195	155	N5:573065
chrV	573174	573311	137	N5:573237
chrV	573461	573612	151	N5:573516
chrV	573469	573612	143	N5:573516
chrV	573517	573642	125	N5:573516
chrV	573742	573894	152	N5:573853
chrV	573815	573959	144	N5:573853
chrV	573912	574047	135	N5:574037
chrV	573912	574099	187	N5:574037
chrV	573955	574047	92	N5:574037
chrV	573955	574099	144	N5:574037
chrV	574294	574446	152	N5:574369
chrV	574595	574725	130	N5:574672
chrV	574965	575116	151	N5:575085
chrV	574970	575104	134	N5:575085
chrV	574983	575079	96	N5:575085
chrV	575037	575152	115	N5:575085
chrV	576130	576281	151	N5:576205
chrVI	257	431	174	N6:337
chrVI	377	525	148	
chrVI	2507	2632	125	N6:2616
chrVI	2537	2680	143	N6:2616
chrVI	2537	2688	151	N6:2616
chrVI	2828	2965	137	N6:2902
chrVI	3919	4067	148	N6:4059
chrVI	4714	4814	100	N6:4769
chrVI	6022	6182	160	N6:6092
chrVI	13717	13867	150	N6:13829
chrVI	15894	16044	150	N6:15963

Chr	Start	End	Length (bp)	Nucleosome_ID
chrVI	24066	24172	106	N6:24173
chrVI	24067	24172	105	N6:24173
chrVI	29981	30130	149	N6:30055
chrVI	36134	36286	152	N6:36241
chrVI	36777	36932	155	N6:36827
chrVI	41762	41909	147	N6:41884
chrVI	48029	48199	170	N6:48100
chrVI	49950	50105	155	N6:50008
chrVI	55917	56077	160	N6:55979
chrVI	60687	60850	163	N6:60777
chrVI	65236	65388	152	N6:65325
chrVI	65445	65606	161	N6:65510
chrVI	68129	68276	147	N6:68162
chrVI	68203	68367	164	
chrVI	71963	72118	155	N6:72001
chrVI	72978	73128	150	
chrVI	76566	76713	147	N6:76715
chrVI	77258	77379	121	
chrVI	79934	80100	166	N6:80017
chrVI	81318	81445	127	N6:81386
chrVI	82718	82885	167	
chrVI	84294	84451	157	N6:84401
chrVI	91095	91248	153	N6:91173
chrVI	94045	94152	107	N6:94050
chrVI	94173	94319	146	N6:94222
chrVI	94286	94435	149	N6:94398
chrVI	97720	97865	145	N6:97797
chrVI	97725	97874	149	N6:97797
chrVI	100985	101138	153	N6:101049
chrVI	108232	108382	150	N6:108293
chrVI	108406	108531	125	N6:108481
chrVI	110912	111064	152	N6:110937
chrVI	110912	111064	152	N6:111087
chrVI	111983	112114	131	N6:112065
chrVI	115465	115634	169	N6:115552
chrVI	123008	123146	138	N6:123085
chrVI	130462	130613	151	N6:130530
chrVI	131462	131615	153	N6:131556
chrVI	139986	140115	129	N6:139988
chrVI	140063	140227	164	N6:140160
chrVI	141451	141594	143	N6:141487
chrVI	142726	142892	166	N6:142759
chrVI	143530	143679	149	N6:143622
chrVI	148326	148498	172	N6:148376
chrVI	151570	151741	171	N6:151682
chrVI	151602	151755	153	N6:151682
chrVI	151830	151995	165	N6:151925
chrVI	152009	152167	158	N6:152083
chrVI	152552	152696	144	N6:152640
chrVI	155423	155586	163	N6:155517
chrVI	162535	162694	159	N6:162635
chrVI	174443	174598	155	N6:174527
chrVI	175285	175426	141	N6:175340
chrVI	176188	176314	126	N6:176274
chrVI	177968	178139	171	N6:178030
chrVI	180563	180715	152	N6:180613
chrVI	182163	182304	141	N6:182222
chrVI	183274	183405	131	
chrVI	183755	183882	127	N6:183852
chrVI	185980	186131	151	N6:186127
chrVI	186486	186602	116	N6:186517
chrVI	192368	192528	160	N6:192442
chrVI	193031	193186	155	N6:193102
chrVI	195001	195152	151	N6:195090
chrVI	197175	197337	162	N6:197229
chrVI	197468	197595	127	
chrVI	198399	198562	163	N6:198429
chrVI	202836	202999	163	N6:202902
chrVI	204046	204202	156	N6:204111
chrVI	208087	208238	151	N6:208144
chrVI	208444	208571	127	N6:208478
chrVI	208746	208898	152	N6:208819
chrVI	210302	210398	96	N6:210340
chrVI	210302	210400	98	N6:210340
chrVI	210302	210402	100	N6:210340
chrVI	210302	210403	101	N6:210340
chrVI	210302	210404	102	N6:210340
chrVI	210302	210405	103	N6:210340
chrVI	210302	210406	104	N6:210340
chrVI	210302	210407	105	N6:210340
chrVI	210302	210409	107	N6:210340
chrVI	214215	214366	151	N6:214204
chrVI	214215	214366	151	N6:214374
chrVI	224528	224665	137	N6:224600
chrVI	225246	225383	137	
chrVI	225640	225800	160	N6:225764
chrVI	228483	228624	141	N6:228495
chrVI	232870	233015	145	N6:232930
chrVI	236135	236288	153	N6:236220
chrVI	236706	236866	160	N6:236786
chrVI	237607	237756	149	N6:237708
chrVI	237976	238116	140	N6:238042
chrVI	239069	239219	150	N6:239120
chrVI	242117	242275	158	N6:242181
chrVI	242831	242985	154	N6:242929
chrVI	242895	243045	150	N6:242929
chrVI	244023	244165	142	N6:244096
chrVI	244610	244760	150	N6:244637

Chr	Start	End	Length (bp)	Nucleosome_ID
chrVI	247013	247149	136	N6:247082
chrVI	258453	258604	151	N6:258521
chrVI	260977	261105	128	N6:261029
chrVI	264647	264774	127	
chrVI	265600	265745	145	N6:265749
chrVI	267454	267607	153	N6:267517
chrVI	269103	269252	149	N6:269131
chrVI	270018	270158	140	N6:270163
chrVII	6501	6604	103	N7:6569
chrVII	7179	7332	153	N7:7156
chrVII	7179	7332	153	N7:7319
chrVII	8743	8898	155	N7:8851
chrVII	8747	8898	151	N7:8851
chrVII	9683	9793	110	N7:9667
chrVII	15630	15761	131	N7:15706
chrVII	15633	15761	128	N7:15706
chrVII	15791	15935	144	
chrVII	20349	20487	138	N7:20424
chrVII	21325	21484	159	N7:21457
chrVII	22410	22546	136	N7:22504
chrVII	23209	23365	156	N7:23217
chrVII	32814	32961	147	N7:32833
chrVII	33222	33355	133	N7:33236
chrVII	36941	37083	142	N7:36962
chrVII	41750	41885	135	N7:41812
chrVII	52996	53149	153	N7:53078
chrVII	53375	53486	111	N7:53412
chrVII	62645	62823	178	N7:62782
chrVII	69139	69303	164	N7:69217
chrVII	72210	72368	158	N7:72277
chrVII	73049	73197	148	N7:73145
chrVII	78222	78356	134	N7:78264
chrVII	83125	83232	107	N7:83164
chrVII	83125	83247	122	N7:83164
chrVII	84927	85059	132	N7:84990
chrVII	88176	88368	192	N7:88188
chrVII	88303	88443	140	
chrVII	88402	88555	153	N7:88473
chrVII	89045	89177	132	
chrVII	90038	90161	123	N7:90075
chrVII	91402	91556	154	N7:91484
chrVII	92970	93138	168	N7:93036
chrVII	97437	97599	162	N7:97514
chrVII	102150	102299	149	N7:102228
chrVII	102520	102673	153	N7:102600
chrVII	103288	103440	152	N7:103447
chrVII	103852	103984	132	N7:103952
chrVII	108983	109147	164	N7:109010
chrVII	116027	116173	146	N7:116126
chrVII	119268	119414	146	N7:119279
chrVII	125888	126050	162	N7:125970
chrVII	138978	139125	147	N7:139099
chrVII	147769	147920	151	N7:147908
chrVII	152057	152208	151	N7:152137
chrVII	156139	156292	153	N7:156241
chrVII	167824	167963	139	N7:167816
chrVII	167824	167963	139	N7:167983
chrVII	168448	168554	106	N7:168455
chrVII	168635	168789	154	N7:168761
chrVII	171388	171548	160	N7:171452
chrVII	174752	174849	97	N7:174743
chrVII	174828	174980	152	N7:174904
chrVII	178188	178320	132	N7:178266
chrVII	183143	183279	136	
chrVII	184850	184994	144	N7:184898
chrVII	196178	196334	156	N7:196334
chrVII	196178	196334	156	N7:196162
chrVII	204030	204183	153	N7:204104
chrVII	204995	205129	134	N7:205048
chrVII	209064	209201	137	N7:209110
chrVII	214611	214774	163	N7:214691
chrVII	217469	217614	145	N7:217533
chrVII	221203	221378	175	N7:221318
chrVII	223475	223638	163	N7:223563
chrVII	226620	226777	157	N7:226722
chrVII	226621	226770	149	N7:226722
chrVII	233772	233924	152	N7:233838
chrVII	238049	238197	148	N7:238126
chrVII	243163	243328	165	N7:243257
chrVII	249506	249649	143	N7:249567
chrVII	251482	251633	151	
chrVII	266552	266693	141	N7:266631
chrVII	267377	267528	151	N7:267455
chrVII	268853	268959	106	N7:268920
chrVII	268853	268977	124	N7:268920
chrVII	270004	270159	155	N7:270127
chrVII	273536	273656	120	N7:273600
chrVII	275567	275723	156	N7:275555
chrVII	275567	275723	156	N7:275720
chrVII	279938	280099	161	N7:280010
chrVII	281760	281908	148	N7:281898
chrVII	281764	281908	144	N7:281898
chrVII	284331	284485	154	N7:284426
chrVII	289603	289760	157	N7:289668
chrVII	293723	293871	148	N7:293806
chrVII	294019	294183	164	N7:294135
chrVII	297177	297313	136	N7:297182
chrVII	299926	300073	147	N7:300004

Chr	Start	End	Length (bp)	Nucleosome_ID
chrVII	300091	300244	153	N7:300166
chrVII	300595	300755	160	
chrVII	313487	313646	159	N7:313555
chrVII	314575	314719	144	N7:314647
chrVII	320068	320221	153	N7:320063
chrVII	320068	320221	153	N7:320242
chrVII	323178	323326	148	
chrVII	324915	325051	136	N7:325012
chrVII	327547	327717	170	N7:327548
chrVII	327547	327717	170	N7:327719
chrVII	328882	329034	152	N7:329029
chrVII	329271	329388	117	N7:329338
chrVII	330372	330517	145	N7:330463
chrVII	331632	331780	148	N7:331795
chrVII	331632	331780	148	N7:331642
chrVII	341547	341699	152	N7:341572
chrVII	342190	342339	149	
chrVII	342310	342453	143	N7:342424
chrVII	343265	343403	138	N7:343315
chrVII	352278	352440	162	N7:352305
chrVII	354451	354624	173	N7:354512
chrVII	356531	356676	145	N7:356589
chrVII	364639	364801	162	N7:364708
chrVII	367967	368120	153	N7:368054
chrVII	370607	370760	153	N7:370672
chrVII	382677	382828	151	N7:382840
chrVII	382677	382828	151	N7:382673
chrVII	383386	383517	131	N7:383447
chrVII	383588	383739	151	N7:383638
chrVII	384835	384978	143	N7:384900
chrVII	384841	384978	137	N7:384900
chrVII	386231	386378	147	N7:386322
chrVII	386588	386752	164	N7:386632
chrVII	398118	398266	148	
chrVII	398740	398893	153	N7:398821
chrVII	399113	399273	160	N7:399182
chrVII	402184	402330	146	N7:402298
chrVII	406230	406375	145	N7:406270
chrVII	412759	412909	150	N7:412835
chrVII	413027	413197	170	N7:413154
chrVII	416964	417114	150	N7:417040
chrVII	419105	419256	151	N7:419189
chrVII	422885	423015	130	N7:422958
chrVII	424432	424587	155	N7:424573
chrVII	426688	426828	140	N7:426744
chrVII	430629	430785	156	N7:430704
chrVII	438337	438504	167	N7:438393
chrVII	445398	445550	152	N7:445477
chrVII	445405	445562	157	N7:445477
chrVII	448295	448446	151	N7:448359
chrVII	449063	449214	151	N7:449131
chrVII	449866	450017	151	N7:449940
chrVII	450289	450475	186	N7:450357
chrVII	450564	450727	163	
chrVII	451657	451811	154	N7:451765
chrVII	452015	452160	145	N7:452093
chrVII	454120	454220	100	N7:454191
chrVII	457180	457330	150	N7:457208
chrVII	457820	457954	134	N7:457886
chrVII	458030	458181	151	N7:458116
chrVII	464168	464326	158	N7:464210
chrVII	468550	468701	151	N7:468622
chrVII	470833	470993	160	
chrVII	471018	471094	76	N7:471038
chrVII	471018	471123	105	N7:471038
chrVII	471173	471322	149	N7:471209
chrVII	475774	475931	157	N7:475867
chrVII	476346	476454	108	
chrVII	476346	476465	119	
chrVII	478282	478437	155	N7:478357
chrVII	482579	482701	122	N7:482600
chrVII	483653	483754	101	N7:483643
chrVII	495041	495196	155	N7:495024
chrVII	501090	501258	168	N7:501148
chrVII	502391	502522	131	N7:502483
chrVII	502870	503027	157	N7:502941
chrVII	504629	504778	149	N7:504726
chrVII	505104	505255	151	N7:505190
chrVII	509103	509251	148	N7:509169
chrVII	509261	509410	149	N7:509325
chrVII	510438	510590	152	N7:510527
chrVII	512325	512500	175	N7:512399
chrVII	515194	515319	125	N7:515214
chrVII	516112	516262	150	N7:516125
chrVII	516112	516262	150	N7:516278
chrVII	517734	517882	148	N7:517806
chrVII	517736	517882	146	N7:517806
chrVII	518337	518484	147	N7:518402
chrVII	519407	519563	156	N7:519436
chrVII	519674	519834	160	
chrVII	519935	520102	167	N7:520057
chrVII	520743	520902	159	N7:520845
chrVII	520956	521107	151	N7:521078
chrVII	526220	526384	164	N7:526309
chrVII	529947	530056	109	N7:529978
chrVII	535422	535536	114	N7:535504
chrVII	536123	536272	149	N7:536209
chrVII	536273	536379	106	

Chr	Start	End	Length (bp)	Nucleosome_ID
chrVII	536273	536391	118	
chrVII	536297	536420	123	
chrVII	536397	536552	155	N7:536510
chrVII	536830	536964	134	N7:536902
chrVII	536830	536970	140	N7:536902
chrVII	537026	537177	151	N7:537109
chrVII	537036	537181	145	N7:537109
chrVII	537308	537462	154	N7:537445
chrVII	537342	537486	144	N7:537445
chrVII	537873	538008	135	N7:537917
chrVII	538055	538187	132	
chrVII	538144	538282	138	N7:538222
chrVII	538499	538656	157	N7:538585
chrVII	538599	538743	144	N7:538585
chrVII	538990	539119	129	
chrVII	539470	539601	131	N7:539492
chrVII	539902	540052	150	N7:539986
chrVII	540853	541004	151	N7:540903
chrVII	542644	542795	151	N7:542763
chrVII	544012	544140	128	N7:544085
chrVII	545414	545580	166	N7:545553
chrVII	546693	546840	147	N7:546767
chrVII	548258	548409	151	N7:548239
chrVII	554460	554622	162	N7:554535
chrVII	554946	555089	143	N7:555041
chrVII	562268	562415	147	N7:562318
chrVII	562775	562889	114	
chrVII	563471	563621	150	N7:563527
chrVII	564404	564533	129	
chrVII	564867	565024	157	N7:564949
chrVII	565335	565496	161	N7:565396
chrVII	565515	565650	135	N7:565587
chrVII	566553	566693	140	N7:566644
chrVII	566559	566693	134	N7:566644
chrVII	566971	567126	155	N7:567004
chrVII	567102	567210	108	N7:567215
chrVII	568106	568272	166	N7:568185
chrVII	568932	569081	149	N7:568958
chrVII	569719	569885	166	N7:569812
chrVII	571017	571160	143	N7:571098
chrVII	572385	572549	164	N7:572444
chrVII	572497	572626	129	N7:572607
chrVII	573872	573982	110	N7:573948
chrVII	575134	575270	136	N7:575205
chrVII	575584	575763	179	N7:575570
chrVII	575584	575763	179	N7:575745
chrVII	578432	578606	174	N7:578577
chrVII	578432	578606	174	N7:578412
chrVII	578979	579129	150	N7:579094
chrVII	579144	579294	150	N7:579259
chrVII	583828	583997	169	N7:583861
chrVII	584107	584258	151	N7:584192
chrVII	584200	584336	136	N7:584192
chrVII	584954	585099	145	N7:584996
chrVII	594298	594445	147	N7:594404
chrVII	595048	595213	165	N7:595207
chrVII	595048	595213	165	N7:595043
chrVII	595281	595432	151	N7:595369
chrVII	610980	611160	180	N7:610959
chrVII	610980	611160	180	N7:611131
chrVII	613266	613414	148	N7:613372
chrVII	637713	637869	156	N7:637824
chrVII	640000	640148	148	N7:640070
chrVII	640002	640148	146	N7:640070
chrVII	640719	640865	146	N7:640822
chrVII	652407	652570	163	N7:652507
chrVII	652780	652925	145	N7:652859
chrVII	654255	654401	146	N7:654248
chrVII	654255	654401	146	N7:654398
chrVII	668831	668991	160	N7:668917
chrVII	671142	671294	152	
chrVII	671676	671849	173	N7:671743
chrVII	674557	674713	156	N7:674628
chrVII	676654	676817	163	N7:676774
chrVII	680899	681051	152	N7:680980
chrVII	681312	681478	166	N7:681411
chrVII	682534	682696	162	N7:682605
chrVII	687608	687758	150	N7:687651
chrVII	689536	689689	153	N7:689603
chrVII	692091	692197	106	N7:692094
chrVII	692091	692218	127	N7:692094
chrVII	692091	692219	128	N7:692094
chrVII	692091	692220	129	N7:692094
chrVII	692091	692221	130	N7:692094
chrVII	692091	692222	131	N7:692094
chrVII	692091	692223	132	N7:692094
chrVII	692092	692223	131	N7:692094
chrVII	692095	692223	128	N7:692094
chrVII	692097	692197	100	N7:692094
chrVII	693772	693921	149	N7:693854
chrVII	697506	697652	146	N7:697541
chrVII	697827	697977	150	N7:697877
chrVII	701841	701992	151	
chrVII	707416	707536	120	N7:707505
chrVII	709744	709890	146	N7:709832
chrVII	712418	712569	151	N7:712512
chrVII	714004	714140	136	N7:714042
chrVII	716031	716189	158	

Chr	Start	End	Length (bp)	Nucleosome_ID
chrVII	716536	716689	153	N7:716629
chrVII	726352	726496	144	N7:726431
chrVII	731531	731680	149	N7:731570
chrVII	740022	740127	105	N7:740078
chrVII	746153	746303	150	N7:746206
chrVII	747978	748102	124	N7:748002
chrVII	748361	748501	140	N7:748439
chrVII	751512	751624	112	N7:751606
chrVII	758738	758894	156	N7:758814
chrVII	760806	760962	156	N7:760938
chrVII	760828	760978	150	N7:760938
chrVII	762415	762565	150	N7:762428
chrVII	766213	766368	155	N7:766304
chrVII	769383	769497	114	N7:769444
chrVII	773391	773557	166	N7:773500
chrVII	776840	777001	161	N7:777019
chrVII	776982	777149	167	N7:777019
chrVII	780856	781004	148	N7:780944
chrVII	781219	781380	161	N7:781318
chrVII	782373	782522	149	
chrVII	782985	783136	151	N7:783055
chrVII	811114	811251	137	N7:811183
chrVII	813520	813649	129	N7:813530
chrVII	813597	813761	164	N7:813691
chrVII	814985	815128	143	N7:815042
chrVII	816260	816426	166	N7:816238
chrVII	817064	817213	149	N7:817170
chrVII	817068	817218	150	N7:817170
chrVII	817863	817968	105	N7:817860
chrVII	817863	817970	107	N7:817860
chrVII	819019	819169	150	N7:819103
chrVII	819762	819909	147	N7:819930
chrVII	820415	820572	157	N7:820493
chrVII	820601	820751	150	N7:820670
chrVII	820792	820937	145	
chrVII	821063	821198	135	N7:821136
chrVII	821345	821513	168	N7:821422
chrVII	822101	822241	140	N7:822182
chrVII	822107	822241	134	N7:822182
chrVII	822519	822674	155	N7:822552
chrVII	822650	822758	108	N7:822752
chrVII	825754	825897	143	N7:825833
chrVII	831865	832015	150	N7:831995
chrVII	841785	841939	154	N7:841891
chrVII	842909	843085	176	N7:843080
chrVII	842909	843085	176	N7:842921
chrVII	843924	844090	166	N7:844011
chrVII	844983	845124	141	N7:845017
chrVII	847489	847638	149	
chrVII	853632	853775	143	N7:853718
chrVII	855743	855883	140	N7:855791
chrVII	855860	856013	153	N7:855962
chrVII	861234	861372	138	N7:861384
chrVII	869483	869631	148	N7:869530
chrVII	873303	873436	133	N7:873338
chrVII	873927	874079	152	N7:873992
chrVII	878924	879054	130	N7:878962
chrVII	879023	879172	149	
chrVII	879984	880088	104	
chrVII	882915	883023	108	
chrVII	882915	883031	116	N7:883049
chrVII	887398	887557	159	
chrVII	889980	890139	159	N7:890158
chrVII	889980	890139	159	N7:890006
chrVII	892462	892609	147	N7:892520
chrVII	895680	895828	148	N7:895781
chrVII	911520	911682	162	N7:911624
chrVII	914384	914518	134	N7:914490
chrVII	920349	920433	84	N7:920408
chrVII	922436	922538	102	N7:922490
chrVII	925873	926024	151	
chrVII	928464	928607	143	
chrVII	928560	928710	150	N7:928631
chrVII	929745	929899	154	N7:929875
chrVII	938659	938812	153	N7:938652
chrVII	943256	943409	153	N7:943358
chrVII	943702	943854	152	N7:943837
chrVII	948590	948746	156	N7:948665
chrVII	949733	949881	148	N7:949820
chrVII	960612	960754	142	N7:960724
chrVII	961680	961814	134	N7:961727
chrVII	961681	961788	107	N7:961727
chrVII	961681	961791	110	N7:961727
chrVII	961681	961794	113	N7:961727
chrVII	961681	961795	114	N7:961727
chrVII	961681	961796	115	N7:961727
chrVII	961681	961797	116	N7:961727
chrVII	961681	961798	117	N7:961727
chrVII	961681	961799	118	N7:961727
chrVII	961681	961800	119	N7:961727
chrVII	961681	961801	120	N7:961727
chrVII	961681	961802	121	N7:961727
chrVII	961681	961803	122	N7:961727
chrVII	961681	961804	123	N7:961727
chrVII	961681	961805	124	N7:961727
chrVII	961681	961806	125	N7:961727
chrVII	961681	961807	126	N7:961727
chrVII	961681	961808	127	N7:961727

Chr	Start	End	Length (bp)	Nucleosome_ID
chrVII	961681	961809	128	N7:961727
chrVII	961681	961810	129	N7:961727
chrVII	961681	961811	130	N7:961727
chrVII	961681	961812	131	N7:961727
chrVII	961681	961813	132	N7:961727
chrVII	961681	961814	133	N7:961727
chrVII	961682	961814	132	N7:961727
chrVII	961683	961814	131	N7:961727
chrVII	961684	961814	130	N7:961727
chrVII	961685	961814	129	N7:961727
chrVII	961686	961814	128	N7:961727
chrVII	961687	961814	127	N7:961727
chrVII	961694	961814	120	N7:961727
chrVII	961695	961814	119	N7:961727
chrVII	961696	961814	118	N7:961727
chrVII	961708	961806	98	N7:961727
chrVII	962925	963072	147	N7:963033
chrVII	966527	966637	110	N7:966590
chrVII	974170	974327	157	N7:974249
chrVII	975022	975129	107	N7:975032
chrVII	975022	975130	108	N7:975032
chrVII	975022	975131	109	N7:975032
chrVII	975022	975142	120	N7:975032
chrVII	979943	980105	162	N7:980040
chrVII	982482	982630	148	N7:982592
chrVII	987451	987604	153	N7:987557
chrVII	987451	987606	155	N7:987557
chrVII	987451	987608	157	N7:987557
chrVII	987451	987609	158	N7:987557
chrVII	988814	988967	153	N7:988944
chrVII	988881	989019	138	N7:988944
chrVII	989319	989475	156	N7:989398
chrVII	989319	989477	158	N7:989398
chrVII	991949	992103	154	N7:992011
chrVII	999326	999473	147	N7:999420
chrVII	1001929	1002078	149	N7:1001976
chrVII	1012079	1012238	159	N7:1012073
chrVII	1012603	1012726	123	N7:1012698
chrVII	1016668	1016789	121	N7:1016735
chrVII	1018529	1018686	157	N7:1018544
chrVII	1018639	1018770	131	N7:1018717
chrVII	1024687	1024847	160	
chrVII	1030700	1030865	165	N7:1030777
chrVII	1030899	1031060	161	N7:1030983
chrVII	1037731	1037891	160	N7:1037843
chrVII	1039928	1040095	167	N7:1040010
chrVII	1043906	1044058	152	N7:1044003
chrVII	1044184	1044344	160	N7:1044171
chrVII	1045755	1045901	146	N7:1045865
chrVII	1049209	1049360	151	N7:1049248
chrVII	1051642	1051789	147	N7:1051693
chrVII	1052012	1052165	153	N7:1052015
chrVII	1065087	1065242	155	N7:1065159
chrVII	1065864	1066016	152	N7:1065932
chrVII	1065866	1066016	150	N7:1065932
chrVII	1065867	1066016	149	N7:1065932
chrVII	1067431	1067584	153	N7:1067518
chrVII	1071145	1071303	158	N7:1071240
chrVII	1079049	1079187	138	N7:1079141
chrVII	1079684	1079846	162	N7:1079686
chrVII	1080323	1080452	129	N7:1080409
chrVII	1085801	1085942	141	N7:1085920
chrVII	1087005	1087167	162	N7:1086993
chrVII	1087005	1087167	162	N7:1087148
chrVII	1087106	1087261	155	N7:1087148
chrVII	1087240	1087377	137	N7:1087315
chrVII	1087527	1087678	151	N7:1087597
chrVII	1087535	1087678	143	N7:1087597
chrVII	1087583	1087708	125	N7:1087597
chrVII	1087808	1087960	152	N7:1087922
chrVII	1087881	1088025	144	N7:1087922
chrVII	1087978	1088113	135	N7:1088072
chrVII	1087978	1088165	187	N7:1088072
chrVII	1088021	1088113	92	N7:1088072
chrVII	1088021	1088165	144	N7:1088072
chrVII	1088360	1088512	152	N7:1088436
chrVII	1088661	1088791	130	N7:1088758
chrVII	1089031	1089182	151	N7:1089163
chrVII	1089036	1089170	134	N7:1089163
chrVII	1089049	1089145	96	N7:1089163
chrVII	1090196	1090347	151	N7:1090283
chrVIII	691	842	151	N8:754
chrVIII	1289	1405	116	N8:1300
chrVIII	1346	1501	155	
chrVIII	1712	1899	187	N8:1845
chrVIII	1733	1899	166	N8:1845
chrVIII	1748	1846	98	N8:1845
chrVIII	1748	1855	107	N8:1845
chrVIII	1748	1878	130	N8:1845
chrVIII	1748	1879	131	N8:1845
chrVIII	1748	1880	132	N8:1845
chrVIII	1748	1889	141	N8:1845
chrVIII	1748	1891	143	N8:1845
chrVIII	1748	1894	146	N8:1845
chrVIII	1748	1895	147	N8:1845
chrVIII	1748	1896	148	N8:1845
chrVIII	1748	1897	149	N8:1845
chrVIII	1748	1898	150	N8:1845

Chr	Start	End	Length (bp)	Nucleosome_ID
chrVIII	1748	1899	151	N8:1845
chrVIII	1748	1909	161	N8:1845
chrVIII	1748	1932	184	N8:1845
chrVIII	1749	1899	150	N8:1845
chrVIII	1750	1899	149	N8:1845
chrVIII	1751	1899	148	N8:1845
chrVIII	1752	1899	147	N8:1845
chrVIII	1753	1899	146	N8:1845
chrVIII	1754	1899	145	N8:1845
chrVIII	1755	1899	144	N8:1845
chrVIII	1756	1899	143	N8:1845
chrVIII	1757	1899	142	N8:1845
chrVIII	1758	1899	141	N8:1845
chrVIII	1759	1899	140	N8:1845
chrVIII	1760	1899	139	N8:1845
chrVIII	1761	1899	138	N8:1845
chrVIII	1762	1899	137	N8:1845
chrVIII	1764	1899	135	N8:1845
chrVIII	1765	1899	134	N8:1845
chrVIII	1766	1899	133	N8:1845
chrVIII	1769	1899	130	N8:1845
chrVIII	1771	1899	128	N8:1845
chrVIII	1773	1899	126	N8:1845
chrVIII	1784	1899	115	N8:1845
chrVIII	1821	1968	147	N8:1845
chrVIII	2051	2222	171	
chrVIII	2441	2594	153	N8:2474
chrVIII	2951	3103	152	N8:2994
chrVIII	3093	3220	127	N8:3153
chrVIII	3233	3376	143	N8:3313
chrVIII	3524	3661	137	N8:3599
chrVIII	4667	4822	155	N8:4741
chrVIII	9299	9453	154	N8:9352
chrVIII	10577	10683	106	N8:10600
chrVIII	10578	10683	105	N8:10600
chrVIII	10864	11003	139	N8:10940
chrVIII	12729	12877	148	N8:12794
chrVIII	14994	15154	160	N8:15064
chrVIII	15294	15466	172	N8:15381
chrVIII	17876	18017	141	N8:18009
chrVIII	18350	18522	172	N8:18473
chrVIII	23920	24090	170	N8:24051
chrVIII	26981	27134	153	N8:27018
chrVIII	32520	32666	146	N8:32599
chrVIII	33329	33481	152	N8:33481
chrVIII	33329	33481	152	N8:33328
chrVIII	33632	33782	150	N8:33647
chrVIII	39986	40150	164	N8:40093
chrVIII	46044	46208	164	N8:46074
chrVIII	48223	48365	142	N8:48283
chrVIII	49771	49922	151	N8:49832
chrVIII	50265	50415	150	N8:50342
chrVIII	51501	51665	164	N8:51521
chrVIII	52096	52204	108	N8:52145
chrVIII	52096	52213	117	N8:52145
chrVIII	52629	52803	174	N8:52786
chrVIII	53713	53863	150	N8:53785
chrVIII	58605	58733	128	N8:58676
chrVIII	59349	59502	153	N8:59376
chrVIII	61087	61247	160	N8:61111
chrVIII	62149	62261	112	N8:62222
chrVIII	65254	65423	169	N8:65391
chrVIII	66426	66576	150	N8:66523
chrVIII	66660	66810	150	N8:66683
chrVIII	66812	66936	124	N8:66852
chrVIII	66813	66902	89	N8:66852
chrVIII	66813	66904	91	N8:66852
chrVIII	66813	66911	98	N8:66852
chrVIII	66813	66912	99	N8:66852
chrVIII	66813	66924	111	N8:66852
chrVIII	66813	66925	112	N8:66852
chrVIII	66813	66926	113	N8:66852
chrVIII	66813	66928	115	N8:66852
chrVIII	66813	66929	116	N8:66852
chrVIII	66813	66930	117	N8:66852
chrVIII	66813	66931	118	N8:66852
chrVIII	66813	66932	119	N8:66852
chrVIII	66813	66933	120	N8:66852
chrVIII	66813	66934	121	N8:66852
chrVIII	66813	66935	122	N8:66852
chrVIII	66813	66936	123	N8:66852
chrVIII	66815	66936	121	N8:66852
chrVIII	66816	66936	120	N8:66852
chrVIII	66817	66936	119	N8:66852
chrVIII	66818	66936	118	N8:66852
chrVIII	66820	66936	116	N8:66852
chrVIII	66821	66936	115	N8:66852
chrVIII	66822	66936	114	N8:66852
chrVIII	66823	66936	113	N8:66852
chrVIII	66830	66936	106	N8:66852
chrVIII	68261	68399	138	N8:68264
chrVIII	69975	70096	121	N8:70043
chrVIII	69975	70122	147	N8:70043
chrVIII	70402	70553	151	N8:70464
chrVIII	72065	72199	134	N8:72067
chrVIII	77481	77612	131	N8:77523
chrVIII	86148	86310	162	N8:86173
chrVIII	86176	86324	148	N8:86173

Chr	Start	End	Length (bp)	Nucleosome_ID
chrVIII	86476	86623	147	N8:86566
chrVIII	88963	89116	153	N8:89119
chrVIII	91372	91534	162	N8:91357
chrVIII	91372	91534	162	N8:91547
chrVIII	94804	94953	149	N8:94867
chrVIII	94985	95141	156	N8:95070
chrVIII	96353	96506	153	N8:96448
chrVIII	100385	100534	149	N8:100544
chrVIII	107252	107412	160	N8:107297
chrVIII	108345	108493	148	N8:108419
chrVIII	109527	109701	174	N8:109636
chrVIII	114779	114932	153	N8:114850
chrVIII	115531	115709	178	N8:115711
chrVIII	121476	121637	161	N8:121495
chrVIII	122292	122451	159	N8:122379
chrVIII	123820	123978	158	N8:123911
chrVIII	125638	125758	120	N8:125733
chrVIII	130844	131002	158	N8:130922
chrVIII	131196	131345	149	N8:131260
chrVIII	142630	142776	146	N8:142760
chrVIII	144193	144334	141	N8:144264
chrVIII	152201	152353	152	N8:152258
chrVIII	153827	153949	122	N8:153924
chrVIII	157804	157963	159	N8:157803
chrVIII	158390	158542	152	N8:158513
chrVIII	162439	162593	154	N8:162524
chrVIII	162949	163087	138	
chrVIII	165932	166076	144	N8:165989
chrVIII	167959	168110	151	N8:168035
chrVIII	171020	171171	151	N8:171084
chrVIII	171703	171855	152	N8:171826
chrVIII	174446	174586	140	
chrVIII	174447	174533	86	
chrVIII	174447	174535	88	
chrVIII	174447	174538	91	
chrVIII	174447	174540	93	
chrVIII	174447	174541	94	
chrVIII	174447	174544	97	
chrVIII	174447	174545	98	
chrVIII	174447	174548	101	
chrVIII	174447	174550	103	
chrVIII	174447	174551	104	
chrVIII	174447	174552	105	
chrVIII	174447	174553	106	
chrVIII	174447	174555	108	
chrVIII	174447	174561	114	
chrVIII	174447	174565	118	
chrVIII	174447	174566	119	
chrVIII	174447	174567	120	
chrVIII	174447	174568	121	
chrVIII	174447	174569	122	
chrVIII	174447	174570	123	
chrVIII	174447	174571	124	
chrVIII	174447	174572	125	
chrVIII	174447	174573	126	
chrVIII	174447	174574	127	
chrVIII	174447	174575	128	
chrVIII	174447	174576	129	
chrVIII	174447	174577	130	
chrVIII	174447	174578	131	
chrVIII	174447	174579	132	
chrVIII	174447	174580	133	
chrVIII	174447	174581	134	
chrVIII	174447	174582	135	
chrVIII	174447	174583	136	
chrVIII	174447	174584	137	
chrVIII	174447	174585	138	
chrVIII	174447	174586	139	
chrVIII	174447	174587	140	
chrVIII	174448	174586	138	
chrVIII	174449	174586	137	
chrVIII	174450	174586	136	
chrVIII	174451	174586	135	
chrVIII	174452	174586	134	
chrVIII	174453	174586	133	
chrVIII	174454	174586	132	
chrVIII	174455	174586	131	
chrVIII	174456	174586	130	
chrVIII	174457	174586	129	
chrVIII	174458	174586	128	
chrVIII	174459	174586	127	
chrVIII	174460	174586	126	
chrVIII	174461	174586	125	
chrVIII	174462	174553	91	
chrVIII	174467	174586	119	
chrVIII	174928	175084	156	N8:175011
chrVIII	176211	176366	155	N8:176283
chrVIII	177719	177867	148	N8:177804
chrVIII	177797	177947	150	N8:177804
chrVIII	178837	178985	148	N8:178874
chrVIII	180375	180509	134	N8:180443
chrVIII	190177	190319	142	N8:190199
chrVIII	194784	194941	157	
chrVIII	194987	195122	135	N8:195028
chrVIII	205654	205804	150	N8:205739
chrVIII	207083	207225	142	N8:207213
chrVIII	212526	212683	157	N8:212615
chrVIII	212729	212874	145	N8:212883

Chr	Start	End	Length (bp)	Nucleosome_ID
chrVIII	212963	213069	106	N8:213049
chrVIII	212963	213076	113	N8:213049
chrVIII	213025	213187	162	N8:213049
chrVIII	214079	214248	169	N8:214186
chrVIII	214176	214329	153	N8:214186
chrVIII	214524	214681	157	N8:214628
chrVIII	214727	214872	145	N8:214799
chrVIII	214961	215067	106	N8:215040
chrVIII	214961	215074	113	N8:215040
chrVIII	215023	215185	162	N8:215040
chrVIII	216077	216246	169	N8:216180
chrVIII	217808	217962	154	N8:217835
chrVIII	218430	218569	139	N8:218465
chrVIII	219279	219430	151	N8:219365
chrVIII	223655	223760	105	N8:223731
chrVIII	226108	226256	148	N8:226185
chrVIII	227838	227999	161	N8:228012
chrVIII	227838	227999	161	N8:227836
chrVIII	228803	228946	143	N8:228877
chrVIII	234383	234536	153	N8:234466
chrVIII	234544	234693	149	N8:234634
chrVIII	240775	240923	148	N8:240835
chrVIII	250570	250724	154	N8:250736
chrVIII	256594	256759	165	N8:256675
chrVIII	258714	258856	142	N8:258740
chrVIII	261335	261482	147	N8:261454
chrVIII	264809	264956	147	N8:264814
chrVIII	270516	270670	154	N8:270646
chrVIII	272692	272812	120	N8:272823
chrVIII	275159	275317	158	
chrVIII	281596	281745	149	N8:281674
chrVIII	288944	289050	106	N8:288960
chrVIII	291607	291756	149	N8:291704
chrVIII	291938	292090	152	N8:292062
chrVIII	294718	294863	145	N8:294786
chrVIII	297474	297608	134	
chrVIII	297952	298079	127	
chrVIII	299058	299223	165	N8:299169
chrVIII	299773	299910	137	N8:299791
chrVIII	310121	310260	139	N8:310259
chrVIII	310839	310998	159	N8:310900
chrVIII	311969	312130	161	N8:312117
chrVIII	333005	333157	152	N8:333100
chrVIII	334511	334659	148	N8:334641
chrVIII	341021	341173	152	N8:341133
chrVIII	341021	341178	157	N8:341133
chrVIII	342001	342158	157	N8:342074
chrVIII	342217	342368	151	N8:342346
chrVIII	343838	343999	161	N8:344006
chrVIII	345951	346111	160	N8:346037
chrVIII	346434	346582	148	N8:346511
chrVIII	350191	350346	155	N8:350266
chrVIII	353263	353415	152	
chrVIII	354917	355076	159	N8:355010
chrVIII	355941	356089	148	N8:356016
chrVIII	357549	357703	154	N8:357581
chrVIII	360834	360982	148	N8:360921
chrVIII	364130	364290	160	N8:364200
chrVIII	370648	370829	181	N8:370765
chrVIII	371736	371883	147	N8:371818
chrVIII	374061	374221	160	N8:374205
chrVIII	374061	374221	160	N8:374049
chrVIII	374061	374255	194	N8:374205
chrVIII	374061	374255	194	N8:374049
chrVIII	374122	374209	87	N8:374205
chrVIII	374122	374221	99	N8:374205
chrVIII	374122	374255	133	N8:374205
chrVIII	376459	376622	163	N8:376456
chrVIII	376459	376622	163	N8:376622
chrVIII	381490	381641	151	N8:381642
chrVIII	382542	382702	160	N8:382584
chrVIII	382976	383151	175	N8:383155
chrVIII	399609	399757	148	N8:399693
chrVIII	402529	402689	160	N8:402659
chrVIII	402529	402689	160	N8:402507
chrVIII	403584	403737	153	N8:403661
chrVIII	404097	404230	133	N8:404184
chrVIII	414157	414333	176	N8:414190
chrVIII	414157	414333	176	N8:414356
chrVIII	416537	416675	138	N8:416611
chrVIII	424973	425127	154	N8:425133
chrVIII	424973	425127	154	N8:424954
chrVIII	427961	428122	161	N8:428089
chrVIII	431042	431168	126	N8:431037
chrVIII	433338	433488	150	N8:433377
chrVIII	439467	439624	157	N8:439505
chrVIII	444830	444988	158	N8:444909
chrVIII	447873	448000	127	N8:448007
chrVIII	456058	456162	104	N8:456183
chrVIII	465568	465728	160	N8:465665
chrVIII	469779	469934	155	N8:469909
chrVIII	469928	470081	153	N8:469909
chrVIII	469928	470081	153	N8:470061
chrVIII	470995	471124	129	N8:471041
chrVIII	482780	482943	163	N8:482852
chrVIII	485160	485318	158	N8:485166
chrVIII	485160	485318	158	N8:485325
chrVIII	495830	495995	165	N8:495933

Chr	Start	End	Length (bp)	Nucleosome_ID
chrVIII	497750	497909	159	N8:497804
chrVIII	501432	501582	150	N8:501512
chrVIII	503721	503876	155	N8:503793
chrVIII	510274	510423	149	N8:510320
chrVIII	518475	518638	163	N8:518487
chrVIII	518475	518638	163	N8:518641
chrVIII	521779	521913	134	N8:521848
chrVIII	522442	522595	153	N8:522525
chrVIII	523030	523182	152	N8:523103
chrVIII	523197	523325	128	N8:523263
chrVIII	525186	525342	156	N8:525241
chrVIII	526030	526198	168	N8:526058
chrVIII	526169	526320	151	N8:526224
chrVIII	526293	526441	148	N8:526381
chrVIII	526293	526481	188	N8:526381
chrVIII	526600	526706	106	N8:526683
chrVIII	526600	526743	143	N8:526683
chrVIII	526600	526749	149	N8:526683
chrVIII	526600	526751	151	N8:526683
chrVIII	526624	526751	127	N8:526683
chrVIII	526651	526751	100	N8:526683
chrVIII	527864	528038	174	N8:527985
chrVIII	536044	536157	113	N8:536107
chrVIII	538480	538617	137	N8:538580
chrVIII	539313	539458	145	N8:539402
chrVIII	539340	539488	148	N8:539402
chrVIII	542782	542926	144	N8:542760
chrVIII	542782	542926	144	N8:542922
chrVIII	542989	543156	167	
chrVIII	543353	543457	104	N8:543426
chrVIII	543780	543928	148	N8:543820
chrVIII	544540	544654	114	
chrVIII	546632	546789	157	N8:546708
chrVIII	546818	546968	150	N8:546888
chrVIII	546822	546968	146	N8:546888
chrVIII	547280	547415	135	N8:547333
chrVIII	548318	548458	140	N8:548393
chrVIII	548324	548458	134	N8:548393
chrVIII	548850	549005	155	N8:548872
chrVIII	548981	549089	108	N8:549081
chrVIII	549893	550056	163	N8:549980
chrVIII	550702	550810	108	N8:550729
chrVIII	551514	551634	120	N8:551574
chrVIII	553179	553326	147	N8:553215
chrVIII	553630	553778	148	N8:553708
chrVIII	556401	556536	135	N8:556476
chrVIII	556826	556854	28	
chrVIII	556826	556859	33	
chrVIII	556826	556865	39	
chrVIII	556826	556871	45	
chrVIII	556826	556873	47	
chrVIII	556826	556876	50	
chrVIII	556826	556884	58	N8:556907
chrVIII	556826	556910	84	N8:556907
chrVIII	556826	556914	88	N8:556907
chrVIII	556826	556918	92	N8:556907
chrVIII	556826	556919	93	N8:556907
chrVIII	556826	556921	95	N8:556907
chrVIII	556826	556923	97	N8:556907
chrVIII	556826	556926	100	N8:556907
chrVIII	556826	556930	104	N8:556907
chrVIII	556826	556935	109	N8:556907
chrVIII	556826	556936	110	N8:556907
chrVIII	556826	556939	113	N8:556907
chrVIII	556826	556942	116	N8:556907
chrVIII	556826	556944	118	N8:556907
chrVIII	556826	556950	124	N8:556907
chrVIII	556826	556954	128	N8:556907
chrVIII	556826	556956	130	N8:556907
chrVIII	556826	556961	135	N8:556907
chrVIII	556826	556963	137	N8:556907
chrVIII	556826	556965	139	N8:556907
chrVIII	556826	556967	141	N8:556907
chrVIII	556826	556969	143	N8:556907
chrVIII	556826	556970	144	N8:556907
chrVIII	556826	556971	145	N8:556907
chrVIII	556826	556972	146	N8:556907
chrVIII	556826	556973	147	N8:556907
chrVIII	556826	556975	149	N8:556907
chrVIII	556826	556976	150	N8:556907
chrVIII	556826	556977	151	N8:556907
chrVIII	556826	556979	153	N8:556907
chrVIII	556826	556981	155	N8:556907
chrVIII	556826	556982	156	N8:556907
chrVIII	556826	556983	157	N8:556907
chrVIII	556826	556984	158	N8:556907
chrVIII	556826	556985	159	N8:556907
chrVIII	556826	556986	160	N8:556907
chrVIII	556826	556987	161	N8:556907
chrVIII	556826	556989	163	N8:556907
chrVIII	556833	556986	153	N8:556907
chrVIII	556840	556986	146	N8:556907
chrVIII	556843	556989	146	N8:556907
chrVIII	556853	556986	133	N8:556907
chrVIII	556853	556989	136	N8:556907
chrVIII	556858	556975	117	N8:556907
chrVIII	556858	556989	131	N8:556907
chrVIII	556859	556963	104	N8:556907

Chr	Start	End	Length (bp)	Nucleosome_ID
chrVIII	556859	556989	130	N8:556907
chrVIII	556863	556986	123	N8:556907
chrVIII	556869	556986	117	N8:556907
chrVIII	556878	556973	95	N8:556907
chrVIII	556878	556975	97	N8:556907
chrVIII	556878	556983	105	N8:556907
chrVIII	556878	556989	111	N8:556907
chrVIII	556883	556989	106	N8:556907
chrVIII	556887	556986	99	N8:556907
chrVIII	556893	556975	82	N8:556907
chrVIII	556893	556989	96	N8:556907
chrVIII	556901	556986	85	N8:556907
chrVIII	556904	556989	85	N8:556907
chrVIII	558701	558838	137	N8:558769
chrVIII	558986	559129	143	N8:559040
chrVIII	559259	559411	152	N8:559370
chrVIII	559332	559476	144	N8:559370
chrVIII	560157	560308	151	
chrVIII	560416	560563	147	N8:560523
chrVIII	560448	560563	115	N8:560523
chrVIII	560502	560671	169	N8:560523
chrVIII	560544	560634	90	N8:560523
chrVIII	560544	560639	95	N8:560523
chrVIII	560784	560911	127	N8:560813
chrVIII	561208	561356	148	
chrVIII	561302	561476	174	N8:561386
chrVIII	561649	561800	151	N8:561716
chrVIII	562455	562594	139	N8:562514
chrVIII	562455	562640	185	N8:562514
chrVIII	562469	562594	125	N8:562514
chrVIII	562469	562614	145	N8:562514
chrVIII	562469	562632	163	N8:562514
chrVIII	562469	562636	167	N8:562514
chrVIII	562469	562640	171	N8:562514
chrVIII	562500	562636	136	N8:562514
chrVIII	562506	562632	126	N8:562514
chrVIII	562506	562636	130	N8:562514
chrVIII	562506	562640	134	N8:562514
chrVIII	562520	562621	101	N8:562514
chrVIII	562520	562640	120	N8:562514
chrVIII	562523	562640	117	N8:562514
chrVIII	562552	562640	88	
chrX	1669	1785	116	N10:1770
chrX	1669	1804	135	N10:1770
chrX	1669	1821	152	N10:1770
chrX	1693	1785	92	N10:1770
chrX	1693	1794	101	N10:1770
chrX	1693	1804	111	N10:1770
chrX	1693	1821	128	N10:1770
chrX	1698	1821	123	N10:1770
chrX	1980	2120	140	N10:2021
chrX	2198	2335	137	N10:2294
chrX	2365	2509	144	N10:2503
chrX	2365	2512	147	N10:2503
chrX	2365	2513	148	N10:2503
chrX	2365	2575	210	N10:2503
chrX	2370	2513	143	N10:2503
chrX	2380	2513	133	N10:2503
chrX	2420	2513	93	N10:2503
chrX	2420	2575	155	N10:2503
chrX	2862	3015	153	N10:2906
chrX	3307	3451	144	N10:3406
chrX	3518	3609	91	N10:3573
chrX	3518	3627	109	N10:3573
chrX	3518	3628	110	N10:3573
chrX	3518	3629	111	N10:3573
chrX	3518	3644	126	N10:3573
chrX	3520	3627	107	N10:3573
chrX	3520	3644	124	N10:3573
chrX	4071	4226	155	N10:4185
chrX	4165	4327	162	N10:4343
chrX	4165	4327	162	N10:4185
chrX	4788	4951	163	N10:4836
chrX	5199	5350	151	
chrX	5412	5571	159	N10:5402
chrX	9273	9424	151	N10:9358
chrX	10211	10354	143	N10:10296
chrX	11443	11594	151	N10:11535
chrX	12328	12473	145	N10:12354
chrX	12893	13042	149	
chrX	14191	14340	149	N10:14274
chrX	16003	16132	129	N10:16047
chrX	16061	16168	107	N10:16047
chrX	21416	21535	119	N10:21469
chrX	28854	29005	151	N10:28867
chrX	28854	29005	151	N10:29020
chrX	30398	30549	151	N10:30469
chrX	31626	31734	108	N10:31614
chrX	32694	32839	145	N10:32766
chrX	33933	34077	144	N10:33990
chrX	37483	37615	132	N10:37503
chrX	38720	38876	156	N10:38782
chrX	40633	40803	170	N10:40707
chrX	40783	40953	170	N10:40870
chrX	46039	46190	151	N10:46072
chrX	53460	53598	138	N10:53518
chrX	55658	55819	161	N10:55711
chrX	59858	59998	140	N10:59955

Chr	Start	End	Length (bp)	Nucleosome_ID
chrX	64405	64545	140	
chrX	86256	86397	141	N10:86335
chrX	88120	88277	157	N10:88190
chrX	90912	91092	180	N10:91046
chrX	94290	94441	151	N10:94365
chrX	94366	94450	84	N10:94365
chrX	94724	94862	138	N10:94769
chrX	100058	100217	159	N10:100203
chrX	100062	100217	155	N10:100203
chrX	100066	100217	151	N10:100203
chrX	105547	105698	151	N10:105607
chrX	106971	107136	165	
chrX	108096	108187	91	N10:108130
chrX	108096	108204	108	N10:108130
chrX	108096	108205	109	N10:108130
chrX	108096	108206	110	N10:108130
chrX	108096	108207	111	N10:108130
chrX	108096	108208	112	N10:108130
chrX	108096	108220	124	N10:108130
chrX	108097	108205	108	N10:108130
chrX	108098	108200	102	N10:108130
chrX	108098	108205	107	N10:108130
chrX	108098	108206	108	N10:108130
chrX	108098	108220	122	N10:108130
chrX	108135	108220	85	N10:108130
chrX	117567	117726	159	
chrX	118042	118180	138	N10:118106
chrX	118045	118180	135	N10:118106
chrX	118063	118213	150	N10:118106
chrX	118208	118364	156	N10:118386
chrX	122371	122478	107	N10:122443
chrX	126969	127111	142	N10:126996
chrX	127866	128026	160	N10:127919
chrX	131244	131407	163	N10:131345
chrX	139844	139996	152	N10:139830
chrX	141168	141318	150	N10:141239
chrX	142696	142846	150	N10:142702
chrX	145224	145374	150	N10:145222
chrX	145224	145374	150	N10:145384
chrX	148124	148271	147	N10:148257
chrX	149558	149726	168	N10:149585
chrX	154966	155110	144	N10:155017
chrX	162602	162770	168	N10:162588
chrX	163009	163162	153	N10:163000
chrX	169091	169251	160	N10:169161
chrX	173679	173830	151	N10:173775
chrX	173874	174016	142	N10:173949
chrX	178426	178580	154	N10:178551
chrX	182961	183077	116	N10:183051
chrX	182964	183077	113	N10:183051
chrX	182966	183077	111	N10:183051
chrX	185927	186084	157	N10:186100
chrX	185927	186084	157	N10:185938
chrX	189658	189821	163	N10:189737
chrX	190658	190805	147	N10:190730
chrX	191225	191359	134	N10:191216
chrX	192006	192112	106	
chrX	192006	192128	122	
chrX	192132	192293	161	N10:192220
chrX	192599	192736	137	N10:192656
chrX	194181	194318	137	N10:194236
chrX	197629	197777	148	N10:197770
chrX	198154	198316	162	N10:198291
chrX	198182	198330	148	N10:198291
chrX	199707	199858	151	N10:199867
chrX	203379	203541	162	N10:203536
chrX	203482	203630	148	N10:203536
chrX	206401	206560	159	N10:206482
chrX	209002	209147	145	N10:209025
chrX	210689	210857	168	N10:210876
chrX	210689	210857	168	N10:210724
chrX	212042	212183	141	N10:212201
chrX	212042	212183	141	N10:212021
chrX	215649	215789	140	N10:215728
chrX	218927	219076	149	
chrX	219865	220016	151	N10:219873
chrX	219865	220016	151	N10:220031
chrX	220580	220715	135	N10:220634
chrX	221358	221508	150	N10:221437
chrX	225766	225921	155	N10:225854
chrX	226052	226204	152	N10:226103
chrX	226472	226633	161	N10:226484
chrX	227406	227571	165	N10:227419
chrX	227406	227571	165	N10:227588
chrX	229005	229137	132	N10:229146
chrX	229384	229535	151	N10:229378
chrX	233516	233665	149	
chrX	235494	235644	150	N10:235627
chrX	237835	237975	140	N10:237857
chrX	240265	240414	149	N10:240350
chrX	240554	240707	153	N10:240699
chrX	240601	240747	146	N10:240699
chrX	242702	242868	166	
chrX	243519	243619	100	N10:243533
chrX	245287	245435	148	N10:245271
chrX	247930	248081	151	N10:247910
chrX	248270	248435	165	N10:248400
chrX	251069	251224	155	N10:251097

Chr	Start	End	Length (bp)	Nucleosome_ID
chrX	251916	252080	164	N10:251970
chrX	253721	253885	164	N10:253709
chrX	254829	255000	171	N10:254961
chrX	255572	255735	163	N10:255685
chrX	271510	271677	167	N10:271629
chrX	271664	271795	131	
chrX	272130	272276	146	N10:272201
chrX	273881	274032	151	N10:273955
chrX	275971	276080	109	N10:276085
chrX	276361	276455	94	
chrX	276361	276463	102	
chrX	276481	276614	133	N10:276566
chrX	278918	279067	149	N10:278918
chrX	283812	283977	165	N10:283889
chrX	287746	287904	158	N10:287749
chrX	292191	292322	131	N10:292221
chrX	292971	293126	155	
chrX	293774	293933	159	N10:293870
chrX	301109	301275	166	N10:301192
chrX	303727	303887	160	N10:303776
chrX	309182	309321	139	N10:309163
chrX	310987	311146	159	N10:310995
chrX	310987	311146	159	N10:311155
chrX	321769	321919	150	N10:321795
chrX	322337	322492	155	N10:322482
chrX	325234	325383	149	N10:325322
chrX	326985	327142	157	N10:327050
chrX	327633	327785	152	N10:327706
chrX	328569	328730	161	
chrX	329259	329407	148	N10:329425
chrX	329259	329407	148	N10:329251
chrX	331899	332052	153	
chrX	333305	333466	161	N10:333312
chrX	333305	333466	161	N10:333478
chrX	333361	333513	152	N10:333478
chrX	337451	337612	161	
chrX	338776	338907	131	N10:338772
chrX	343385	343533	148	N10:343380
chrX	344740	344900	160	
chrX	350347	350517	170	N10:350502
chrX	350347	350517	170	N10:350327
chrX	357774	357934	160	N10:357775
chrX	358769	358924	155	N10:358940
chrX	359603	359757	154	N10:359674
chrX	362162	362320	158	N10:362260
chrX	365711	365851	140	N10:365757
chrX	366070	366219	149	N10:366182
chrX	366353	366502	149	N10:366336
chrX	366353	366502	149	N10:366486
chrX	366520	366671	151	N10:366641
chrX	367696	367845	149	N10:367708
chrX	367696	367845	149	N10:367868
chrX	369411	369539	128	N10:369398
chrX	369776	369927	151	N10:369846
chrX	374096	374246	150	N10:374118
chrX	380580	380750	170	N10:380752
chrX	380580	380750	170	N10:380571
chrX	380923	381086	163	N10:380993
chrX	385357	385485	128	
chrX	386997	387161	164	N10:387073
chrX	392484	392621	137	N10:392516
chrX	392991	393143	152	N10:393070
chrX	394180	394326	146	N10:394214
chrX	394276	394372	96	N10:394374
chrX	398245	398394	149	N10:398328
chrX	399541	399701	160	N10:399582
chrX	407672	407824	152	N10:407809
chrX	410284	410430	146	N10:410379
chrX	410723	410889	166	N10:410731
chrX	412052	412213	161	
chrX	425378	425524	146	
chrX	429176	429332	156	N10:429178
chrX	429194	429332	138	N10:429178
chrX	429194	429339	145	N10:429178
chrX	435614	435721	107	N10:435670
chrX	435614	435731	117	N10:435670
chrX	439795	439950	155	N10:439773
chrX	447409	447538	129	
chrX	448364	448517	153	N10:448344
chrX	456180	456328	148	N10:456302
chrX	459326	459480	154	N10:459411
chrX	465469	465620	151	N10:465481
chrX	466504	466659	155	N10:466505
chrX	467197	467335	138	N10:467195
chrX	467559	467705	146	N10:467683
chrX	470836	470984	148	N10:470847
chrX	472163	472315	152	N10:472226
chrX	472826	472975	149	N10:472972
chrX	473000	473123	123	N10:473146
chrX	473100	473255	155	N10:473146
chrX	473533	473667	134	N10:473586
chrX	473533	473673	140	N10:473586
chrX	473729	473880	151	N10:473881
chrX	473739	473884	145	N10:473881
chrX	473757	473908	151	N10:473881
chrX	474011	474165	154	N10:474051
chrX	474045	474189	144	N10:474051
chrX	474045	474189	144	N10:474202

Chr	Start	End	Length (bp)	Nucleosome_ID
chrX	474576	474711	135	
chrX	475023	475169	146	
chrX	475023	475173	150	
chrX	475693	475822	129	N10:475768
chrX	477337	477451	114	
chrX	477657	477804	147	N10:477753
chrX	478238	478390	152	N10:478237
chrX	478410	478559	149	N10:478482
chrX	478560	478666	106	
chrX	478560	478678	118	
chrX	478584	478707	123	N10:478712
chrX	478684	478839	155	N10:478712
chrX	479117	479251	134	
chrX	479117	479257	140	N10:479275
chrX	480160	480295	135	N10:480180
chrX	480607	480757	150	N10:480589
chrX	480786	480943	157	N10:480816
chrX	481277	481406	129	N10:481374
chrX	481757	481888	131	N10:481781
chrX	482189	482339	150	N10:482287
chrX	483140	483291	151	N10:483274
chrX	495866	496005	139	N10:495880
chrX	496369	496517	148	N10:496351
chrX	498437	498584	147	N10:498554
chrX	503072	503238	166	N10:503242
chrX	503072	503238	166	N10:503073
chrX	503922	504069	147	N10:504008
chrX	512701	512853	152	N10:512703
chrX	516708	516897	189	N10:516725
chrX	519161	519304	143	N10:519222
chrX	519732	519838	106	N10:519795
chrX	519732	519858	126	N10:519795
chrX	523978	524108	130	N10:523988
chrX	526346	526498	152	N10:526454
chrX	527259	527387	128	N10:527394
chrX	530647	530795	148	N10:530763
chrX	531614	531753	139	N10:531763
chrX	533693	533845	152	N10:533743
chrX	537746	537899	153	N10:537764
chrX	542575	542723	148	
chrX	544311	544443	132	N10:544344
chrX	547133	547290	157	N10:547288
chrX	554602	554737	135	N10:554644
chrX	557805	557945	140	N10:557963
chrX	557805	557945	140	N10:557804
chrX	557934	558083	149	N10:557963
chrX	558445	558574	129	N10:558562
chrX	561483	561587	104	
chrX	561700	561836	136	N10:561697
chrX	563670	563822	152	N10:563737
chrX	567134	567269	135	N10:567226
chrX	569137	569291	154	N10:569134
chrX	572775	572903	128	
chrX	573799	573959	160	N10:573951
chrX	582808	582958	150	
chrX	586031	586180	149	N10:586162
chrX	590874	591029	155	N10:591040
chrX	591957	592113	156	N10:592071
chrX	594961	595112	151	N10:594945
chrX	595496	595660	164	N10:595504
chrX	598239	598403	164	N10:598323
chrX	598789	598947	158	N10:598769
chrX	600553	600699	146	N10:600632
chrX	606604	606728	124	N10:606660
chrX	608387	608544	157	N10:608402
chrX	608709	608811	102	N10:608690
chrX	610999	611149	150	N10:610987
chrX	623512	623664	152	N10:623582
chrX	624211	624342	131	N10:624345
chrX	624949	625089	140	N10:625080
chrX	632590	632738	148	N10:632669
chrX	635827	635966	139	N10:635846
chrX	636181	636337	156	N10:636350
chrX	636181	636337	156	N10:636186
chrX	636714	636864	150	N10:636770
chrX	637154	637305	151	N10:637147
chrX	637154	637305	151	N10:637320
chrX	638977	639126	149	N10:638974
chrX	653069	653214	145	N10:653126
chrX	661255	661420	165	N10:661267
chrX	662029	662161	132	N10:662110
chrX	662034	662161	127	N10:662110
chrX	662037	662135	98	N10:662110
chrX	671104	671223	119	
chrX	671901	672050	149	
chrX	673361	673549	188	
chrX	673385	673539	154	
chrX	674527	674676	149	N10:674605
chrX	684490	684642	152	N10:684478
chrX	684490	684642	152	N10:684641
chrX	685942	686069	127	N10:686043
chrX	687031	687188	157	N10:687180
chrX	687681	687842	161	N10:687764
chrX	689548	689698	150	N10:689678
chrX	695637	695789	152	N10:695637
chrX	696260	696391	131	N10:696342
chrX	697538	697692	154	N10:697604
chrX	699421	699528	107	N10:699480

Chr	Start	End	Length (bp)	Nucleosome_ID
chrX	699421	699559	138	N10:699480
chrX	699421	699560	139	N10:699480
chrX	699421	699561	140	N10:699480
chrX	699421	699563	142	N10:699480
chrX	699421	699564	143	N10:699480
chrX	700845	700991	146	N10:700894
chrX	701322	701470	148	N10:701322
chrX	705757	705926	169	N10:705760
chrX	705757	705926	169	N10:705934
chrX	706905	707066	161	N10:707000
chrX	712931	713070	139	N10:712913
chrX	714988	715121	133	N10:715105
chrX	720830	720969	139	N10:720969
chrX	721017	721124	107	
chrX	721017	721148	131	
chrX	721017	721149	132	
chrX	721017	721153	136	
chrX	721018	721153	135	
chrX	721019	721153	134	
chrX	721020	721153	133	
chrX	723086	723238	152	N10:723143
chrX	723301	723431	130	N10:723416
chrX	723442	723594	152	N10:723587
chrX	725588	725741	153	N10:725606
chrX	729427	729584	157	N10:729606
chrX	729427	729584	157	N10:729424
chrX	729799	729953	154	
chrX	732737	732877	140	N10:732736
chrX	735177	735331	154	N10:735172
chrX	735177	735331	154	N10:735346
chrX	736882	737013	131	N10:736967
chrX	739148	739242	94	N10:739259
chrX	745644	745739	95	
chrX	745644	745742	98	
chrXI	6938	7102	164	N11:6990
chrXI	10271	10420	149	N11:10428
chrXI	17984	18132	148	N11:18117
chrXI	19266	19389	123	N11:19336
chrXI	19267	19389	122	N11:19336
chrXI	19267	19414	147	N11:19336
chrXI	20703	20861	158	N11:20761
chrXI	31510	31641	131	
chrXI	32617	32778	161	N11:32717
chrXI	38219	38379	160	
chrXI	39078	39236	158	N11:39170
chrXI	41766	41916	150	
chrXI	42476	42618	142	N11:42545
chrXI	42479	42618	139	N11:42545
chrXI	44129	44285	156	N11:44262
chrXI	45474	45591	117	
chrXI	52845	53010	165	N11:52908
chrXI	53086	53185	99	N11:53095
chrXI	55429	55585	156	N11:55522
chrXI	55463	55623	160	N11:55522
chrXI	55924	56074	150	N11:55980
chrXI	55924	56080	156	N11:55980
chrXI	56846	56978	132	N11:56861
chrXI	61848	62032	184	N11:61896
chrXI	63877	64030	153	N11:63947
chrXI	65154	65316	162	N11:65282
chrXI	65672	65838	166	N11:65746
chrXI	68614	68763	149	N11:68734
chrXI	73102	73261	159	N11:73185
chrXI	86463	86611	148	N11:86556
chrXI	86483	86645	162	N11:86556
chrXI	88336	88484	148	N11:88371
chrXI	89789	89957	168	N11:89946
chrXI	89789	89957	168	N11:89782
chrXI	91032	91184	152	N11:91137
chrXI	97596	97707	111	N11:97729
chrXI	97601	97707	106	N11:97729
chrXI	99816	99946	130	N11:99812
chrXI	99816	99946	130	N11:99961
chrXI	103049	103197	148	N11:103034
chrXI	103049	103197	148	N11:103200
chrXI	103372	103526	154	N11:103384
chrXI	105256	105405	149	N11:105284
chrXI	105988	106134	146	N11:106069
chrXI	110658	110811	153	N11:110802
chrXI	115225	115382	157	N11:115291
chrXI	116126	116262	136	N11:116268
chrXI	116126	116262	136	N11:116112
chrXI	116231	116308	77	N11:116268
chrXI	116232	116308	76	N11:116268
chrXI	116232	116357	125	N11:116268
chrXI	117579	117716	137	N11:117661
chrXI	120342	120499	157	N11:120460
chrXI	120705	120857	152	N11:120781
chrXI	121556	121713	157	N11:121624
chrXI	130706	130855	149	N11:130776
chrXI	130929	131079	150	N11:130940
chrXI	132985	133151	166	N11:133074
chrXI	134033	134163	130	N11:134102
chrXI	136517	136676	159	
chrXI	140618	140768	150	N11:140692
chrXI	142672	142804	132	N11:142756
chrXI	145250	145428	178	N11:145382
chrXI	146568	146719	151	N11:146644

Chr	Start	End	Length (bp)	Nucleosome_ID
chrXI	147356	147520	164	N11:147421
chrXI	149408	149568	160	N11:149508
chrXI	149964	150108	144	N11:149984
chrXI	151755	151890	135	N11:151768
chrXI	161235	161384	149	N11:161325
chrXI	161799	161928	129	N11:161887
chrXI	162802	162952	150	N11:162881
chrXI	170837	170984	147	N11:170954
chrXI	171758	171891	133	N11:171815
chrXI	172320	172456	136	N11:172446
chrXI	178039	178227	188	N11:178152
chrXI	178982	179138	156	N11:179070
chrXI	179058	179209	151	N11:179232
chrXI	179058	179209	151	N11:179070
chrXI	182120	182298	178	N11:182189
chrXI	183536	183690	154	N11:183621
chrXI	183964	184081	117	N11:183955
chrXI	187208	187352	144	N11:187276
chrXI	188029	188179	150	N11:188071
chrXI	190331	190479	148	N11:190473
chrXI	191024	191171	147	N11:191119
chrXI	195322	195449	127	N11:195406
chrXI	196316	196481	165	N11:196493
chrXI	196620	196752	132	N11:196660
chrXI	201937	202099	162	N11:202016
chrXI	204817	204983	166	N11:204909
chrXI	207618	207770	152	
chrXI	209750	209876	126	N11:209839
chrXI	214868	215018	150	N11:214868
chrXI	214868	215019	151	N11:214868
chrXI	214886	215018	132	N11:214868
chrXI	214886	215019	133	N11:214868
chrXI	216300	216452	152	N11:216342
chrXI	216511	216657	146	N11:216610
chrXI	226833	226983	150	N11:226966
chrXI	226930	227062	132	N11:226966
chrXI	227206	227326	120	
chrXI	229813	229973	160	N11:229834
chrXI	233634	233784	150	N11:233638
chrXI	233634	233784	150	N11:233802
chrXI	236594	236764	170	N11:236682
chrXI	241460	241610	150	N11:241499
chrXI	245174	245347	173	N11:245177
chrXI	245189	245339	150	N11:245177
chrXI	245551	245725	174	N11:245572
chrXI	247204	247369	165	N11:247366
chrXI	247204	247369	165	N11:247197
chrXI	249638	249773	135	N11:249715
chrXI	251126	251263	137	N11:251198
chrXI	253186	253299	113	N11:253297
chrXI	253541	253688	147	N11:253657
chrXI	257352	257460	108	
chrXI	257353	257460	107	
chrXI	257353	257478	125	N11:257490
chrXI	257617	257767	150	N11:257744
chrXI	258177	258336	159	N11:258256
chrXI	261407	261543	136	N11:261504
chrXI	269387	269539	152	N11:269548
chrXI	269387	269539	152	N11:269374
chrXI	270529	270668	139	N11:270660
chrXI	275707	275869	162	N11:275750
chrXI	275877	276030	153	N11:276025
chrXI	277324	277474	150	N11:277389
chrXI	278497	278637	140	N11:278487
chrXI	278499	278637	138	N11:278487
chrXI	278501	278637	136	N11:278487
chrXI	278502	278637	135	N11:278487
chrXI	282993	283156	163	N11:283075
chrXI	286651	286824	173	N11:286733
chrXI	287603	287693	90	N11:287704
chrXI	288326	288474	148	
chrXI	290076	290228	152	N11:290108
chrXI	292965	293112	147	N11:293063
chrXI	297006	297125	119	N11:297036
chrXI	297006	297156	150	N11:297036
chrXI	298702	298832	130	N11:298779
chrXI	298875	299007	132	
chrXI	300199	300305	106	N11:300189
chrXI	300199	300317	118	N11:300189
chrXI	303921	304068	147	N11:304040
chrXI	305787	305949	162	
chrXI	318927	319072	145	
chrXI	319054	319202	148	N11:319121
chrXI	322500	322670	170	N11:322551
chrXI	329617	329749	132	
chrXI	329629	329749	120	
chrXI	329629	329775	146	
chrXI	337370	337525	155	N11:337362
chrXI	337447	337600	153	
chrXI	337450	337600	150	
chrXI	337452	337600	148	
chrXI	337462	337600	138	
chrXI	337968	338118	150	
chrXI	340261	340401	140	N11:340305
chrXI	341684	341830	146	N11:341720
chrXI	347279	347450	171	N11:347369
chrXI	347320	347467	147	N11:347369
chrXI	347479	347608	129	N11:347544

Chr	Start	End	Length (bp)	Nucleosome_ID
chrXI	351244	351421	177	
chrXI	351885	352030	145	N11:351957
chrXI	352745	352882	137	N11:352864
chrXI	353147	353287	140	N11:353269
chrXI	355288	355448	160	
chrXI	357460	357614	154	N11:357564
chrXI	357462	357614	152	N11:357564
chrXI	362917	363070	153	
chrXI	364062	364210	148	
chrXI	364602	364723	121	N11:364623
chrXI	368064	368220	156	
chrXI	368389	368522	133	N11:368440
chrXI	368971	369078	107	N11:368963
chrXI	368971	369121	150	N11:368963
chrXI	368971	369122	151	N11:368963
chrXI	372978	373087	109	N11:372980
chrXI	373360	373511	151	N11:373499
chrXI	377591	377751	160	N11:377588
chrXI	379254	379407	153	N11:379329
chrXI	385681	385837	156	N11:385749
chrXI	394646	394790	144	N11:394794
chrXI	397867	398011	144	N11:398025
chrXI	397867	398011	144	N11:397862
chrXI	399301	399460	159	
chrXI	400364	400506	142	N11:400496
chrXI	401469	401625	156	N11:401550
chrXI	401694	401852	158	N11:401711
chrXI	403288	403463	175	N11:403356
chrXI	406796	406955	159	N11:406832
chrXI	407916	408065	149	
chrXI	416183	416327	144	N11:416221
chrXI	417925	418084	159	N11:417916
chrXI	417925	418084	159	N11:418102
chrXI	434342	434496	154	N11:434396
chrXI	434529	434684	155	N11:434609
chrXI	434561	434692	131	N11:434609
chrXI	434662	434792	130	
chrXI	439672	439806	134	N11:439814
chrXI	439682	439780	98	
chrXI	447937	448094	157	N11:447938
chrXI	448453	448602	149	N11:448490
chrXI	450755	450913	158	N11:450835
chrXI	454604	454760	156	N11:454730
chrXI	455031	455138	107	
chrXI	455031	455148	117	N11:455162
chrXI	456736	456888	152	N11:456824
chrXI	459229	459336	107	N11:459206
chrXI	459229	459341	112	N11:459206
chrXI	462337	462501	164	N11:462401
chrXI	468677	468825	148	
chrXI	470132	470274	142	
chrXI	473335	473488	153	N11:473478
chrXI	479959	480086	127	N11:479984
chrXI	483328	483476	148	N11:483388
chrXI	484474	484623	149	N11:484589
chrXI	488055	488207	152	N11:488122
chrXI	488418	488598	180	N11:488497
chrXI	496967	497107	140	N11:497088
chrXI	501192	501346	154	N11:501317
chrXI	506644	506790	146	N11:506685
chrXI	509826	509992	166	N11:509894
chrXI	514096	514200	104	
chrXI	516175	516336	161	N11:516248
chrXI	517302	517453	151	N11:517465
chrXI	517302	517453	151	N11:517303
chrXI	517458	517572	114	N11:517465
chrXI	519553	519695	142	N11:519598
chrXI	527505	527640	135	N11:527634
chrXI	527506	527600	94	
chrXI	527506	527630	124	N11:527634
chrXI	527506	527632	126	N11:527634
chrXI	527506	527635	129	N11:527634
chrXI	527506	527636	130	N11:527634
chrXI	527506	527637	131	N11:527634
chrXI	527506	527638	132	N11:527634
chrXI	527506	527639	133	N11:527634
chrXI	527506	527640	134	N11:527634
chrXI	527506	527641	135	N11:527634
chrXI	527507	527640	133	N11:527634
chrXI	527508	527640	132	N11:527634
chrXI	527509	527640	131	N11:527634
chrXI	527510	527640	130	N11:527634
chrXI	527511	527640	129	N11:527634
chrXI	527513	527640	127	N11:527634
chrXI	527515	527640	125	N11:527634
chrXI	527516	527640	124	N11:527634
chrXI	529083	529220	137	N11:529204
chrXI	529403	529549	146	N11:529570
chrXI	532781	532933	152	N11:532891
chrXI	533171	533320	149	N11:533271
chrXI	540415	540570	155	N11:540451
chrXI	540630	540787	157	N11:540698
chrXI	540646	540797	151	N11:540698
chrXI	541723	541874	151	N11:541853
chrXI	542290	542416	126	N11:542354
chrXI	542290	542440	150	N11:542354
chrXI	544676	544823	147	
chrXI	545722	545853	131	N11:545860

Chr	Start	End	Length (bp)	Nucleosome_ID
chrXI	547817	547975	158	N11:547848
chrXI	548333	548481	148	N11:548344
chrXI	548769	548931	162	N11:548799
chrXI	548769	548931	162	N11:548953
chrXI	549757	549907	150	N11:549905
chrXI	550284	550422	138	N11:550349
chrXI	554400	554551	151	N11:554437
chrXI	558933	559085	152	N11:558954
chrXI	562000	562173	173	N11:562186
chrXI	571010	571152	142	N11:571147
chrXI	573165	573306	141	N11:573269
chrXI	574030	574178	148	N11:574099
chrXI	574402	574555	153	N11:574493
chrXI	575707	575877	170	
chrXI	577254	577388	134	N11:577250
chrXI	577351	577483	132	
chrXI	577690	577817	127	N11:577711
chrXI	578476	578613	137	N11:578514
chrXI	579137	579295	158	N11:579194
chrXI	590716	590822	106	N11:590776
chrXI	590716	590835	119	N11:590776
chrXI	600361	600514	153	N11:600370
chrXI	600361	600514	153	N11:600524
chrXI	601868	602039	171	N11:601908
chrXI	602067	602222	155	N11:602124
chrXI	603803	603944	141	N11:603837
chrXI	604749	604856	107	
chrXI	604749	604865	116	
chrXI	607498	607646	148	N11:607543
chrXI	607998	608143	145	N11:608050
chrXI	613167	613294	127	N11:613186
chrXI	614331	614461	130	N11:614435
chrXI	618644	618800	156	N11:618766
chrXI	621479	621632	153	N11:621620
chrXI	624990	625141	151	N11:625071
chrXI	632085	632240	155	N11:632159
chrXI	632617	632753	136	N11:632714
chrXI	638182	638313	131	N11:638195
chrXI	638182	638316	134	N11:638195
chrXI	643087	643244	157	N11:643162
chrXI	646189	646315	126	N11:646259
chrXI	647330	647482	152	N11:647430
chrXI	648395	648512	117	
chrXI	650461	650612	151	N11:650494
chrXI	651142	651292	150	N11:651252
chrXI	654048	654197	149	
chrXI	655704	655870	166	N11:655848
chrXI	655763	655920	157	N11:655848
chrXI	657176	657328	152	N11:657284
chrXI	658054	658203	149	N11:658207
chrXI	659842	659965	123	N11:659867
chrXI	661850	662006	156	N11:662001
chrXI	663739	663846	107	N11:663807
chrXI	663739	663851	112	N11:663807
chrXI	664373	664519	146	N11:664394
chrXI	666612	666748	136	
chrXI	666628	666815	187	
chrXI	666656	666815	159	
chrXI	666681	666771	90	
chrXI	666681	666810	129	
chrXI	666693	666810	117	
chrXI	666703	666728	25	
chrXI	666703	666733	30	
chrXI	666703	666737	34	
chrXI	666703	666739	36	
chrXI	666709	666792	83	
chrXI	666709	666810	101	
chrXI	666709	666815	106	
chrXI	666714	666815	101	
chrXII	723	874	151	N12:805
chrXII	1167	1315	148	
chrXII	1996	2147	151	N12:2014
chrXII	2008	2142	134	N12:2014
chrXII	2033	2129	96	N12:2014
chrXII	2033	2193	160	N12:2177
chrXII	2033	2193	160	N12:2014
chrXII	2211	2359	148	N12:2350
chrXII	2226	2355	129	N12:2350
chrXII	2226	2359	133	N12:2350
chrXII	2226	2421	195	N12:2350
chrXII	2387	2517	130	
chrXII	2666	2818	152	N12:2754
chrXII	3013	3157	144	N12:3070
chrXII	3013	3200	187	N12:3070
chrXII	3065	3157	92	N12:3070
chrXII	3065	3200	135	N12:3070
chrXII	3153	3297	144	N12:3257
chrXII	3218	3370	152	N12:3257
chrXII	3470	3595	125	N12:3583
chrXII	3500	3643	143	N12:3583
chrXII	3500	3651	151	N12:3583
chrXII	3791	3928	137	N12:3868
chrXII	6346	6517	171	N12:6439
chrXII	6366	6517	151	N12:6439
chrXII	6690	6864	174	N12:6762
chrXII	6705	6857	152	N12:6762
chrXII	7495	7682	187	N12:7546
chrXII	7531	7682	151	N12:7546

Chr	Start	End	Length (bp)	Nucleosome_ID
chrXII	7543	7677	134	N12:7546
chrXII	7568	7664	96	N12:7546
chrXII	7746	7894	148	N12:7816
chrXII	7761	7894	133	N12:7816
chrXII	7761	7956	195	N12:7816
chrXII	7922	8052	130	
chrXII	8201	8353	152	N12:8284
chrXII	8548	8692	144	N12:8607
chrXII	8548	8735	187	N12:8607
chrXII	8600	8692	92	N12:8607
chrXII	8600	8735	135	N12:8607
chrXII	8688	8832	144	N12:8786
chrXII	8753	8905	152	N12:8786
chrXII	9005	9130	125	N12:9112
chrXII	9035	9178	143	N12:9112
chrXII	9326	9463	137	N12:9402
chrXII	11195	11357	162	N12:11275
chrXII	11198	11354	156	N12:11275
chrXII	11205	11354	149	N12:11275
chrXII	11205	11357	152	N12:11275
chrXII	11207	11357	150	N12:11275
chrXII	11208	11354	146	N12:11275
chrXII	11211	11357	146	N12:11275
chrXII	11213	11310	97	N12:11275
chrXII	11213	11346	133	N12:11275
chrXII	11213	11352	139	N12:11275
chrXII	11213	11355	142	N12:11275
chrXII	11213	11357	144	N12:11275
chrXII	11213	11358	145	N12:11275
chrXII	11218	11354	136	N12:11275
chrXII	11222	11354	132	N12:11275
chrXII	11222	11357	135	N12:11275
chrXII	11226	11354	128	N12:11275
chrXII	11226	11357	131	N12:11275
chrXII	11226	11358	132	N12:11275
chrXII	11227	11357	130	N12:11275
chrXII	11228	11357	129	N12:11275
chrXII	11229	11357	128	N12:11275
chrXII	11230	11357	127	N12:11275
chrXII	11231	11354	123	N12:11275
chrXII	11231	11357	126	N12:11275
chrXII	11231	11358	127	N12:11275
chrXII	11233	11357	124	N12:11275
chrXII	11235	11357	122	N12:11275
chrXII	11252	11354	102	N12:11275
chrXII	11252	11357	105	N12:11275
chrXII	11259	11357	98	N12:11275
chrXII	11262	11357	95	N12:11275
chrXII	11273	11357	84	N12:11275
chrXII	13256	13420	164	N12:13360
chrXII	13298	13427	129	N12:13360
chrXII	13457	13612	155	N12:13509
chrXII	15085	15257	172	N12:15154
chrXII	15201	15361	160	N12:15316
chrXII	16724	16865	141	N12:16731
chrXII	18847	18996	149	N12:18970
chrXII	25117	25257	140	N12:25169
chrXII	27714	27865	151	N12:27788
chrXII	35557	35688	131	N12:35640
chrXII	39250	39399	149	N12:39315
chrXII	48577	48723	146	N12:48603
chrXII	51782	51932	150	N12:51881
chrXII	56756	56881	125	N12:56813
chrXII	57933	58079	146	N12:58069
chrXII	60326	60478	152	N12:60341
chrXII	62223	62381	158	
chrXII	67309	67460	151	
chrXII	70032	70180	148	N12:70102
chrXII	70780	70917	137	N12:70939
chrXII	70780	70917	137	N12:70773
chrXII	71343	71501	158	N12:71413
chrXII	73489	73643	154	N12:73587
chrXII	79801	79962	161	
chrXII	99031	99175	144	N12:99103
chrXII	104748	104900	152	N12:104799
chrXII	104994	105164	170	N12:104983
chrXII	106767	106921	154	N12:106904
chrXII	107154	107242	88	N12:107195
chrXII	107154	107250	96	N12:107195
chrXII	107154	107252	98	N12:107195
chrXII	107154	107253	99	N12:107195
chrXII	107154	107255	101	N12:107195
chrXII	107154	107257	103	N12:107195
chrXII	107154	107258	104	N12:107195
chrXII	107154	107259	105	N12:107195
chrXII	107154	107260	106	N12:107195
chrXII	107154	107261	107	N12:107195
chrXII	107154	107262	108	N12:107195
chrXII	107154	107265	111	N12:107195
chrXII	107154	107267	113	N12:107195
chrXII	107154	107269	115	N12:107195
chrXII	107154	107270	116	N12:107195
chrXII	107154	107271	117	N12:107195
chrXII	107154	107273	119	N12:107195
chrXII	107154	107274	120	N12:107195
chrXII	107154	107285	131	N12:107195
chrXII	107271	107423	152	N12:107365
chrXII	110633	110799	166	N12:110737

Chr	Start	End	Length (bp)	Nucleosome_ID
chrXII	112506	112634	128	N12:112551
chrXII	113051	113206	155	N12:113139
chrXII	118248	118400	152	N12:118377
chrXII	119597	119738	141	N12:119672
chrXII	121717	121889	172	N12:121848
chrXII	126647	126799	152	N12:126629
chrXII	132281	132417	136	
chrXII	132310	132466	156	
chrXII	133301	133451	150	N12:133308
chrXII	133762	133946	184	N12:133881
chrXII	136653	136779	126	N12:136749
chrXII	138426	138582	156	N12:138554
chrXII	142345	142492	147	N12:142429
chrXII	143973	144111	138	N12:144010
chrXII	144765	144914	149	N12:144832
chrXII	145430	145576	146	N12:145533
chrXII	155097	155220	123	N12:155205
chrXII	160945	161106	161	N12:161017
chrXII	163470	163588	118	N12:163522
chrXII	171732	171883	151	N12:171897
chrXII	177830	177964	134	N12:177819
chrXII	177830	177964	134	N12:177986
chrXII	178588	178741	153	
chrXII	179076	179226	150	N12:179114
chrXII	180676	180836	160	N12:180728
chrXII	183315	183461	146	N12:183389
chrXII	192705	192836	131	N12:192762
chrXII	193019	193125	106	N12:193086
chrXII	193019	193144	125	N12:193086
chrXII	194389	194550	161	N12:194429
chrXII	197350	197482	132	N12:197391
chrXII	197802	197965	163	
chrXII	199062	199223	161	N12:199130
chrXII	199271	199406	135	N12:199299
chrXII	199721	199871	150	N12:199806
chrXII	207917	208073	156	N12:207961
chrXII	208414	208561	147	N12:208481
chrXII	209818	209960	142	N12:209848
chrXII	211059	211185	126	N12:211093
chrXII	213217	213369	152	N12:213269
chrXII	214356	214519	163	N12:214440
chrXII	216121	216270	149	N12:216153
chrXII	217884	218033	149	N12:218006
chrXII	218108	218265	157	N12:218186
chrXII	218294	218444	150	N12:218356
chrXII	218298	218444	146	N12:218356
chrXII	218756	218891	135	N12:218862
chrXII	219794	219934	140	N12:219876
chrXII	219800	219934	134	N12:219876
chrXII	220212	220367	155	N12:220242
chrXII	220343	220451	108	N12:220455
chrXII	223444	223638	194	N12:223614
chrXII	223444	223638	194	N12:223443
chrXII	230481	230653	172	N12:230555
chrXII	230739	230894	155	N12:230860
chrXII	232813	232957	144	N12:232966
chrXII	232813	232957	144	N12:232814
chrXII	235469	235619	150	N12:235506
chrXII	235483	235631	148	N12:235506
chrXII	237484	237634	150	N12:237551
chrXII	238295	238446	151	N12:238404
chrXII	239615	239753	138	N12:239689
chrXII	255888	256021	133	
chrXII	256324	256484	160	N12:256416
chrXII	259071	259235	164	N12:259139
chrXII	260642	260797	155	N12:260719
chrXII	262571	262718	147	N12:262667
chrXII	270658	270826	168	N12:270763
chrXII	270673	270826	153	N12:270763
chrXII	271752	271896	144	N12:271899
chrXII	273309	273417	108	N12:273383
chrXII	273309	273418	109	N12:273383
chrXII	273309	273419	110	N12:273383
chrXII	273309	273420	111	N12:273383
chrXII	274415	274568	153	N12:274467
chrXII	275945	276112	167	N12:276130
chrXII	275945	276112	167	N12:275964
chrXII	280170	280332	162	N12:280290
chrXII	280378	280532	154	N12:280450
chrXII	281608	281761	153	N12:281710
chrXII	285125	285277	152	N12:285193
chrXII	285128	285277	149	N12:285193
chrXII	286283	286443	160	N12:286398
chrXII	292173	292332	159	N12:292269
chrXII	293330	293472	142	N12:293400
chrXII	295117	295270	153	N12:295096
chrXII	295472	295620	148	N12:295545
chrXII	296601	296740	139	N12:296667
chrXII	300804	300959	155	N12:300827
chrXII	301895	302037	142	N12:301967
chrXII	302498	302646	148	N12:302567
chrXII	304308	304455	147	N12:304426
chrXII	304635	304774	139	N12:304748
chrXII	310453	310583	130	N12:310571
chrXII	311744	311903	159	
chrXII	312700	312835	135	N12:312689
chrXII	314498	314655	157	N12:314563
chrXII	319179	319325	146	N12:319307

Chr	Start	End	Length (bp)	Nucleosome_ID
chrXII	320329	320487	158	N12:320461
chrXII	320335	320487	152	N12:320461
chrXII	320336	320487	151	N12:320461
chrXII	321520	321655	135	N12:321554
chrXII	322116	322262	146	
chrXII	327876	328044	168	N12:327934
chrXII	338236	338387	151	N12:338317
chrXII	340076	340209	133	N12:340159
chrXII	340597	340761	164	N12:340639
chrXII	340875	341034	159	N12:340984
chrXII	347768	347916	148	N12:347855
chrXII	347831	348011	180	N12:348007
chrXII	347831	348011	180	N12:347855
chrXII	348735	348938	203	N12:348883
chrXII	348736	348938	202	N12:348883
chrXII	350679	350830	151	
chrXII	355881	356007	126	N12:355867
chrXII	355881	356007	126	N12:356021
chrXII	358183	358323	140	N12:358217
chrXII	359562	359714	152	N12:359697
chrXII	360814	360966	152	
chrXII	367954	368092	138	N12:368026
chrXII	372826	372968	142	N12:372913
chrXII	375291	375434	143	N12:375295
chrXII	375584	375750	166	N12:375684
chrXII	388796	388957	161	N12:388850
chrXII	388880	389024	144	N12:389034
chrXII	391687	391826	139	
chrXII	395857	396006	149	N12:396018
chrXII	395857	396006	149	N12:395841
chrXII	397064	397220	156	N12:397207
chrXII	398081	398231	150	N12:398173
chrXII	400245	400397	152	
chrXII	401669	401819	150	N12:401676
chrXII	401669	401819	150	N12:401833
chrXII	405414	405520	106	N12:405442
chrXII	405414	405538	124	N12:405442
chrXII	406427	406580	153	N12:406529
chrXII	406621	406768	147	N12:406710
chrXII	408412	408522	110	N12:408439
chrXII	408412	408523	111	N12:408439
chrXII	408415	408537	122	N12:408439
chrXII	411908	412040	132	N12:411988
chrXII	412316	412427	111	N12:412343
chrXII	412319	412427	108	N12:412343
chrXII	412320	412427	107	N12:412343
chrXII	419068	419220	152	N12:419117
chrXII	419734	419893	159	N12:419779
chrXII	421131	421283	152	N12:421250
chrXII	423648	423793	145	N12:423723
chrXII	424263	424441	178	N12:424247
chrXII	425377	425494	117	N12:425416
chrXII	428829	428982	153	N12:428985
chrXII	433448	433586	138	N12:433530
chrXII	433602	433749	147	N12:433706
chrXII	434337	434490	153	N12:434430
chrXII	443464	443629	165	N12:443445
chrXII	446788	446962	174	N12:446975
chrXII	446788	446962	174	N12:446824
chrXII	447291	447454	163	N12:447462
chrXII	447291	447454	163	N12:447286
chrXII	450323	450477	154	N12:450413
chrXII	451465	451626	161	N12:451492
chrXII	451590	451738	148	
chrXII	451599	451733	134	
chrXII	451709	451842	133	N12:451807
chrXII	451726	451885	159	N12:451807
chrXII	451734	451869	135	N12:451807
chrXII	451757	451842	85	N12:451807
chrXII	451757	451908	151	N12:451807
chrXII	451769	451932	163	N12:451807
chrXII	451790	451908	118	N12:451807
chrXII	451790	451932	142	N12:451807
chrXII	451790	451960	170	N12:451807
chrXII	451790	451960	170	N12:451976
chrXII	451800	451934	134	N12:451807
chrXII	451800	451961	161	N12:451807
chrXII	451800	451961	161	N12:451976
chrXII	451851	451934	83	
chrXII	451851	451958	107	N12:451976
chrXII	451851	451959	108	N12:451976
chrXII	451851	451960	109	N12:451976
chrXII	451851	451961	110	N12:451976
chrXII	451876	451960	84	N12:451976
chrXII	451876	452009	133	N12:451976
chrXII	451919	452075	156	N12:451976
chrXII	451919	452077	158	N12:451976
chrXII	451919	452078	159	N12:451976
chrXII	451919	452092	173	N12:451976
chrXII	451923	452075	152	N12:451976
chrXII	451923	452077	154	N12:451976
chrXII	451923	452092	169	N12:451976
chrXII	451931	452062	131	N12:451976
chrXII	451931	452077	146	N12:451976
chrXII	451931	452109	178	N12:451976
chrXII	451932	452062	130	N12:451976
chrXII	451932	452075	143	N12:451976
chrXII	451932	452077	145	N12:451976

Chr	Start	End	Length (bp)	Nucleosome_ID
chrXII	451932	452109	177	N12:451976
chrXII	451945	452051	106	N12:451976
chrXII	451945	452062	117	N12:451976
chrXII	451945	452075	130	N12:451976
chrXII	451945	452077	132	N12:451976
chrXII	451945	452078	133	N12:451976
chrXII	451945	452092	147	N12:451976
chrXII	451945	452109	164	N12:451976
chrXII	451958	452109	151	N12:451976
chrXII	452041	452196	155	N12:452173
chrXII	452059	452215	156	N12:452173
chrXII	452081	452227	146	N12:452173
chrXII	452081	452236	155	N12:452173
chrXII	452081	452247	166	N12:452173
chrXII	452081	452251	170	N12:452173
chrXII	452104	452247	143	N12:452173
chrXII	452104	452251	147	N12:452173
chrXII	452115	452271	156	N12:452173
chrXII	452190	452344	154	N12:452173
chrXII	452190	452344	154	N12:452361
chrXII	452260	452414	154	N12:452361
chrXII	452293	452400	107	N12:452361
chrXII	452294	452455	161	N12:452361
chrXII	452295	452455	160	N12:452361
chrXII	452299	452455	156	N12:452361
chrXII	452323	452455	132	N12:452361
chrXII	452323	452485	162	N12:452361
chrXII	452323	452486	163	N12:452361
chrXII	452334	452441	107	N12:452361
chrXII	452334	452455	121	N12:452361
chrXII	452334	452485	151	N12:452361
chrXII	452334	452486	152	N12:452361
chrXII	452346	452496	150	N12:452361
chrXII	452423	452584	161	N12:452551
chrXII	452469	452584	115	N12:452551
chrXII	452469	452620	151	N12:452551
chrXII	452469	452621	152	N12:452551
chrXII	452469	452629	160	N12:452551
chrXII	452469	452630	161	N12:452551
chrXII	452491	452621	130	N12:452551
chrXII	452491	452629	138	N12:452551
chrXII	452491	452663	172	N12:452551
chrXII	452492	452584	92	N12:452551
chrXII	452492	452620	128	N12:452551
chrXII	452492	452621	129	N12:452551
chrXII	452492	452629	137	N12:452551
chrXII	452492	452663	171	N12:452551
chrXII	452494	452621	127	N12:452551
chrXII	452525	452695	170	N12:452551
chrXII	452539	452673	134	N12:452551
chrXII	452550	452672	122	N12:452551
chrXII	452550	452702	152	N12:452551
chrXII	452668	452796	128	N12:452761
chrXII	452684	452796	112	N12:452761
chrXII	452684	452835	151	N12:452761
chrXII	452686	452814	128	N12:452761
chrXII	452686	452884	198	N12:452761
chrXII	452709	452864	155	N12:452761
chrXII	452709	452884	175	N12:452761
chrXII	452709	452900	191	N12:452761
chrXII	452723	452814	91	N12:452761
chrXII	452723	452864	141	N12:452761
chrXII	452723	452884	161	N12:452761
chrXII	452723	452900	177	N12:452761
chrXII	452765	452864	99	N12:452761
chrXII	452765	452871	106	N12:452761
chrXII	452765	452884	119	N12:452761
chrXII	452765	452900	135	N12:452761
chrXII	452765	452925	160	N12:452761
chrXII	452765	452925	160	N12:452927
chrXII	452773	452864	91	N12:452761
chrXII	452773	452879	106	N12:452761
chrXII	452773	452884	111	N12:452761
chrXII	452773	452900	127	N12:452761
chrXII	452773	452925	152	N12:452761
chrXII	452773	452925	152	N12:452927
chrXII	452783	452962	179	N12:452761
chrXII	452783	452962	179	N12:452927
chrXII	452788	452923	135	N12:452927
chrXII	452788	452953	165	N12:452927
chrXII	452801	452923	122	N12:452927
chrXII	452801	452953	152	N12:452927
chrXII	452801	452999	198	N12:452927
chrXII	452840	452953	113	N12:452927
chrXII	452840	452999	159	N12:452927
chrXII	452840	453023	183	N12:452927
chrXII	452847	452953	106	N12:452927
chrXII	452847	452996	149	N12:452927
chrXII	452876	453034	158	N12:452927
chrXII	452885	453034	149	N12:452927
chrXII	452893	452999	106	N12:452927
chrXII	452895	453023	128	N12:452927
chrXII	452922	453029	107	N12:452927
chrXII	452922	453031	109	N12:452927
chrXII	452922	453034	112	N12:452927
chrXII	452922	453056	134	N12:452927
chrXII	452922	453056	134	N12:453077
chrXII	452922	453075	153	N12:452927

Chr	Start	End	Length (bp)	Nucleosome_ID
chrXII	452922	453075	153	N12:453077
chrXII	452926	452997	71	N12:452927
chrXII	452926	453033	107	N12:452927
chrXII	452926	453034	108	N12:452927
chrXII	452926	453056	130	N12:452927
chrXII	452926	453056	130	N12:453077
chrXII	452926	453070	144	N12:452927
chrXII	452926	453070	144	N12:453077
chrXII	452926	453075	149	N12:452927
chrXII	452926	453075	149	N12:453077
chrXII	452976	453138	162	N12:453077
chrXII	452976	453163	187	N12:453077
chrXII	452991	453141	150	N12:453077
chrXII	453033	453163	130	N12:453077
chrXII	453033	453187	154	N12:453077
chrXII	453033	453193	160	N12:453077
chrXII	453039	453163	124	N12:453077
chrXII	453039	453187	148	N12:453077
chrXII	453039	453193	154	N12:453077
chrXII	453042	453141	99	N12:453077
chrXII	453042	453212	170	N12:453077
chrXII	453042	453218	176	N12:453077
chrXII	453043	453163	120	N12:453077
chrXII	453043	453187	144	N12:453077
chrXII	453043	453190	147	N12:453077
chrXII	453043	453193	150	N12:453077
chrXII	453091	453199	108	N12:453077
chrXII	453092	453199	107	N12:453077
chrXII	453092	453212	120	N12:453077
chrXII	453092	453236	144	N12:453077
chrXII	453092	453245	153	N12:453077
chrXII	453092	453270	178	N12:453077
chrXII	453092	453282	190	N12:453077
chrXII	453092	453286	194	N12:453077
chrXII	453092	453288	196	N12:453077
chrXII	453094	453236	142	N12:453077
chrXII	453095	453201	106	N12:453077
chrXII	453095	453212	117	N12:453077
chrXII	453095	453218	123	N12:453077
chrXII	453095	453236	141	N12:453077
chrXII	453095	453245	150	N12:453077
chrXII	453095	453270	175	N12:453077
chrXII	453095	453286	191	N12:453077
chrXII	453095	453288	193	N12:453077
chrXII	453099	453236	137	N12:453077
chrXII	453099	453287	188	N12:453077
chrXII	453099	453288	189	N12:453077
chrXII	453100	453238	138	N12:453077
chrXII	453100	453245	145	N12:453077
chrXII	453113	453270	157	
chrXII	453113	453286	173	
chrXII	453113	453288	175	
chrXII	453114	453236	122	
chrXII	453114	453269	155	
chrXII	453114	453270	156	
chrXII	453114	453286	172	
chrXII	453114	453287	173	
chrXII	453114	453288	174	
chrXII	453114	453302	188	
chrXII	453114	453309	195	
chrXII	453116	453269	153	
chrXII	453116	453270	154	
chrXII	453116	453288	172	
chrXII	453119	453236	117	
chrXII	453119	453245	126	
chrXII	453119	453269	150	
chrXII	453119	453270	151	
chrXII	453119	453282	163	
chrXII	453119	453286	167	
chrXII	453119	453287	168	
chrXII	453119	453288	169	
chrXII	453119	453302	183	
chrXII	453119	453309	190	
chrXII	453120	453228	108	
chrXII	453121	453270	149	
chrXII	453121	453282	161	
chrXII	453121	453286	165	
chrXII	453121	453288	167	
chrXII	453125	453231	106	
chrXII	453125	453236	111	
chrXII	453125	453270	145	
chrXII	453125	453286	161	
chrXII	453125	453287	162	
chrXII	453125	453288	163	
chrXII	453125	453302	177	
chrXII	453126	453248	122	
chrXII	453126	453270	144	
chrXII	453126	453286	160	
chrXII	453126	453287	161	
chrXII	453126	453288	162	
chrXII	453135	453270	135	
chrXII	453135	453282	147	
chrXII	453135	453286	151	
chrXII	453135	453288	153	
chrXII	453138	453286	148	
chrXII	453138	453288	150	
chrXII	453140	453245	105	
chrXII	453140	453246	106	

Chr	Start	End	Length (bp)	Nucleosome_ID
chrXII	453140	453251	111	
chrXII	453140	453270	130	
chrXII	453140	453282	142	
chrXII	453140	453286	146	
chrXII	453140	453287	147	
chrXII	453140	453288	148	
chrXII	453140	453302	162	
chrXII	453140	453309	169	
chrXII	453140	453323	183	
chrXII	453141	453236	95	
chrXII	453141	453245	104	
chrXII	453141	453251	110	
chrXII	453141	453269	128	
chrXII	453141	453270	129	
chrXII	453141	453281	140	
chrXII	453141	453282	141	
chrXII	453141	453286	145	
chrXII	453141	453287	146	
chrXII	453141	453288	147	
chrXII	453141	453302	161	
chrXII	453141	453309	168	
chrXII	453141	453323	182	
chrXII	453141	453324	183	
chrXII	453144	453288	144	
chrXII	453150	453286	136	
chrXII	453150	453287	137	
chrXII	453150	453288	138	
chrXII	453150	453295	145	
chrXII	453150	453302	152	
chrXII	453152	453240	88	
chrXII	453152	453270	118	
chrXII	453152	453286	134	
chrXII	453152	453287	135	
chrXII	453152	453288	136	
chrXII	453152	453302	150	
chrXII	453152	453309	157	
chrXII	453153	453286	133	
chrXII	453153	453288	135	
chrXII	453159	453270	111	
chrXII	453159	453286	127	
chrXII	453159	453287	128	
chrXII	453159	453288	129	
chrXII	453159	453302	143	
chrXII	453159	453309	150	
chrXII	453159	453335	176	
chrXII	453159	453342	183	
chrXII	453161	453245	84	
chrXII	453161	453268	107	
chrXII	453161	453270	109	
chrXII	453161	453282	121	
chrXII	453161	453286	125	
chrXII	453161	453288	127	
chrXII	453161	453309	148	
chrXII	453161	453323	162	
chrXII	453161	453324	163	
chrXII	453162	453269	107	
chrXII	453162	453286	124	
chrXII	453162	453288	126	
chrXII	453162	453323	161	
chrXII	453162	453324	162	
chrXII	453165	453286	121	
chrXII	453165	453287	122	
chrXII	453165	453288	123	
chrXII	453165	453302	137	
chrXII	453165	453342	177	
chrXII	453180	453286	106	
chrXII	453180	453288	108	
chrXII	453180	453340	160	
chrXII	453181	453270	89	
chrXII	453181	453282	101	
chrXII	453181	453286	105	
chrXII	453181	453288	107	
chrXII	453181	453323	142	
chrXII	453181	453324	143	
chrXII	453286	453432	146	N12:453378
chrXII	453299	453425	126	N12:453378
chrXII	453299	453440	141	N12:453378
chrXII	453299	453460	161	N12:453378
chrXII	453300	453410	110	N12:453378
chrXII	453300	453414	114	N12:453378
chrXII	453300	453440	140	N12:453378
chrXII	453302	453440	138	N12:453378
chrXII	453310	453440	130	N12:453378
chrXII	453310	453460	150	N12:453378
chrXII	453322	453481	159	N12:453378
chrXII	453458	453595	137	N12:453535
chrXII	453458	453610	152	N12:453535
chrXII	453458	453612	154	N12:453535
chrXII	453460	453612	152	N12:453535
chrXII	453487	453595	108	N12:453535
chrXII	453487	453610	123	N12:453535
chrXII	453487	453612	125	N12:453535
chrXII	453500	453652	152	N12:453535
chrXII	453574	453727	153	N12:453693
chrXII	453587	453737	150	N12:453693
chrXII	453606	453774	168	N12:453693
chrXII	453627	453776	149	N12:453693
chrXII	453627	453779	152	N12:453693

Chr	Start	End	Length (bp)	Nucleosome_ID
chrXII	453657	453814	157	N12:453693
chrXII	453659	453776	117	N12:453693
chrXII	453659	453779	120	N12:453693
chrXII	453734	453853	119	N12:453841
chrXII	453748	453910	162	N12:453841
chrXII	453748	453913	165	N12:453841
chrXII	453748	453919	171	N12:453841
chrXII	453748	453922	174	N12:453841
chrXII	453748	453962	214	N12:453841
chrXII	453752	453853	101	N12:453841
chrXII	453752	453859	107	N12:453841
chrXII	453752	453908	156	N12:453841
chrXII	453752	453911	159	N12:453841
chrXII	453752	453914	162	N12:453841
chrXII	453752	453916	164	N12:453841
chrXII	453752	453922	170	N12:453841
chrXII	453752	453924	172	N12:453841
chrXII	453753	453913	160	N12:453841
chrXII	453753	453919	166	N12:453841
chrXII	453753	453922	169	N12:453841
chrXII	453754	453910	156	N12:453841
chrXII	453754	453924	170	N12:453841
chrXII	453774	453910	136	N12:453841
chrXII	453774	453913	139	N12:453841
chrXII	453774	453914	140	N12:453841
chrXII	453774	453916	142	N12:453841
chrXII	453774	453919	145	N12:453841
chrXII	453774	453922	148	N12:453841
chrXII	453774	453925	151	N12:453841
chrXII	453774	453962	188	N12:453841
chrXII	453775	453908	133	N12:453841
chrXII	453775	453911	136	N12:453841
chrXII	453775	453914	139	N12:453841
chrXII	453775	453916	141	N12:453841
chrXII	453775	453919	144	N12:453841
chrXII	453775	453922	147	N12:453841
chrXII	453781	453908	127	N12:453841
chrXII	453781	453911	130	N12:453841
chrXII	453781	453914	133	N12:453841
chrXII	453781	453916	135	N12:453841
chrXII	453781	453922	141	N12:453841
chrXII	453782	453908	126	N12:453841
chrXII	453782	453911	129	N12:453841
chrXII	453782	453913	131	N12:453841
chrXII	453782	453914	132	N12:453841
chrXII	453782	453919	137	N12:453841
chrXII	453782	453922	140	N12:453841
chrXII	453782	453925	143	N12:453841
chrXII	453782	453929	147	N12:453841
chrXII	453782	453937	155	N12:453841
chrXII	453782	453962	180	N12:453841
chrXII	453783	453911	128	N12:453841
chrXII	453783	453913	130	N12:453841
chrXII	453783	453914	131	N12:453841
chrXII	453812	453913	101	N12:453841
chrXII	453812	453919	107	N12:453841
chrXII	453812	453962	150	N12:453841
chrXII	453812	454001	189	N12:453841
chrXII	453812	454001	189	N12:454007
chrXII	453851	453958	107	N12:453841
chrXII	453851	453961	110	N12:453841
chrXII	453851	453962	111	N12:453841
chrXII	453851	454001	150	N12:453841
chrXII	453851	454001	150	N12:454007
chrXII	453909	454072	163	N12:454007
chrXII	453918	454080	162	N12:454007
chrXII	453922	454079	157	N12:454007
chrXII	453929	454079	150	N12:454007
chrXII	453929	454097	168	N12:454007
chrXII	453929	454108	179	N12:454007
chrXII	453959	454097	138	N12:454007
chrXII	453959	454108	149	N12:454007
chrXII	453959	454112	153	N12:454007
chrXII	453960	454116	156	N12:454007
chrXII	453964	454097	133	N12:454007
chrXII	453964	454108	144	N12:454007
chrXII	453971	454123	152	N12:454007
chrXII	454059	454216	157	N12:454198
chrXII	454111	454268	157	N12:454198
chrXII	454120	454267	147	N12:454198
chrXII	454120	454277	157	N12:454198
chrXII	454124	454277	153	N12:454198
chrXII	454131	454277	146	N12:454198
chrXII	454141	454277	136	N12:454198
chrXII	454196	454346	150	N12:454198
chrXII	454196	454346	150	N12:454367
chrXII	454221	454392	171	N12:454198
chrXII	454221	454392	171	N12:454367
chrXII	454244	454392	148	N12:454367
chrXII	454244	454402	158	N12:454367
chrXII	454248	454374	126	N12:454367
chrXII	454248	454392	144	N12:454367
chrXII	454248	454398	150	N12:454367
chrXII	454273	454436	163	N12:454367
chrXII	454277	454398	121	N12:454367
chrXII	454277	454402	125	N12:454367
chrXII	454277	454438	161	N12:454367
chrXII	454277	454446	169	N12:454367

Chr	Start	End	Length (bp)	Nucleosome_ID
chrXII	454398	454551	153	
chrXII	454398	454552	154	
chrXII	454398	454555	157	
chrXII	454398	454567	169	
chrXII	454398	454573	175	
chrXII	454398	454575	177	
chrXII	454404	454552	148	
chrXII	454404	454555	151	
chrXII	454422	454552	130	
chrXII	454422	454555	133	
chrXII	454422	454567	145	
chrXII	454422	454573	151	
chrXII	454422	454575	153	
chrXII	454521	454678	157	N12:454654
chrXII	454553	454712	159	N12:454654
chrXII	454553	454715	162	N12:454654
chrXII	454553	454733	180	N12:454654
chrXII	454555	454703	148	N12:454654
chrXII	454560	454678	118	N12:454654
chrXII	454560	454712	152	N12:454654
chrXII	454560	454715	155	N12:454654
chrXII	454560	454724	164	N12:454654
chrXII	454560	454733	173	N12:454654
chrXII	454562	454712	150	N12:454654
chrXII	454570	454712	142	N12:454654
chrXII	454570	454715	145	N12:454654
chrXII	454570	454724	154	N12:454654
chrXII	454570	454733	163	N12:454654
chrXII	454576	454712	136	N12:454654
chrXII	454576	454715	139	N12:454654
chrXII	454576	454724	148	N12:454654
chrXII	454576	454733	157	N12:454654
chrXII	454576	454772	196	N12:454654
chrXII	454587	454746	159	N12:454654
chrXII	454592	454742	150	N12:454654
chrXII	454626	454724	98	N12:454654
chrXII	454626	454733	107	N12:454654
chrXII	454626	454772	146	N12:454654
chrXII	454649	454741	92	N12:454654
chrXII	454677	454828	151	N12:454654
chrXII	454677	454828	151	N12:454834
chrXII	454723	454854	131	N12:454834
chrXII	454725	454863	138	N12:454834
chrXII	454725	454886	161	N12:454834
chrXII	454733	454863	130	N12:454834
chrXII	454733	454886	153	N12:454834
chrXII	454741	454896	155	N12:454834
chrXII	454747	454853	106	N12:454834
chrXII	454747	454886	139	N12:454834
chrXII	454825	454972	147	N12:454834
chrXII	454825	454972	147	N12:454994
chrXII	454837	454991	154	N12:454834
chrXII	454837	454991	154	N12:454994
chrXII	454864	455021	157	N12:454994
chrXII	454876	455023	147	N12:454994
chrXII	454876	455029	153	N12:454994
chrXII	454901	455029	128	N12:454994
chrXII	454901	455074	173	N12:454994
chrXII	454920	455087	167	N12:454994
chrXII	454932	455073	141	N12:454994
chrXII	454933	455023	90	N12:454994
chrXII	454933	455024	91	N12:454994
chrXII	454933	455025	92	N12:454994
chrXII	454933	455027	94	N12:454994
chrXII	454933	455029	96	N12:454994
chrXII	454933	455073	140	N12:454994
chrXII	454933	455074	141	N12:454994
chrXII	454933	455076	143	N12:454994
chrXII	454933	455132	199	N12:454994
chrXII	454964	455132	168	N12:454994
chrXII	454995	455164	169	N12:454994
chrXII	455000	455132	132	N12:454994
chrXII	455015	455166	151	N12:454994
chrXII	455058	455208	150	
chrXII	455068	455223	155	
chrXII	455095	455269	174	
chrXII	455096	455223	127	
chrXII	455096	455240	144	
chrXII	455096	455243	147	
chrXII	455096	455245	149	
chrXII	455096	455249	153	
chrXII	455097	455240	143	
chrXII	455097	455243	146	
chrXII	455098	455243	145	
chrXII	455098	455249	151	
chrXII	455129	455293	164	N12:455294
chrXII	455138	455289	151	N12:455294
chrXII	455182	455338	156	N12:455294
chrXII	455217	455366	149	N12:455294
chrXII	455221	455395	174	N12:455294
chrXII	455247	455381	134	N12:455294
chrXII	455254	455401	147	N12:455294
chrXII	455255	455400	145	N12:455294
chrXII	455278	455423	145	N12:455294
chrXII	455297	455447	150	N12:455294
chrXII	455367	455512	145	N12:455488
chrXII	455367	455531	164	N12:455488
chrXII	455367	455549	182	N12:455488

Chr	Start	End	Length (bp)	Nucleosome_ID
chrXII	455377	455512	135	N12:455488
chrXII	455377	455531	154	N12:455488
chrXII	455377	455549	172	N12:455488
chrXII	455397	455503	106	N12:455488
chrXII	455397	455512	115	N12:455488
chrXII	455397	455531	134	N12:455488
chrXII	455397	455549	152	N12:455488
chrXII	455397	455568	171	N12:455488
chrXII	455410	455512	102	N12:455488
chrXII	455410	455549	139	N12:455488
chrXII	455410	455560	150	N12:455488
chrXII	455410	455568	158	N12:455488
chrXII	455410	455569	159	N12:455488
chrXII	455416	455571	155	N12:455488
chrXII	455422	455569	147	N12:455488
chrXII	455591	455751	160	N12:455656
chrXII	455701	455870	169	N12:455829
chrXII	455747	455895	148	N12:455829
chrXII	455749	455889	140	N12:455829
chrXII	455783	455958	175	N12:455829
chrXII	455869	456021	152	N12:456042
chrXII	455903	456067	164	N12:456042
chrXII	455918	456039	121	N12:456042
chrXII	455926	456086	160	N12:456042
chrXII	456022	456173	151	N12:456042
chrXII	456022	456205	183	N12:456042
chrXII	456022	456205	183	N12:456220
chrXII	456022	456211	189	N12:456042
chrXII	456022	456211	189	N12:456220
chrXII	456022	456220	198	N12:456042
chrXII	456022	456220	198	N12:456220
chrXII	456025	456173	148	N12:456042
chrXII	456025	456182	157	N12:456042
chrXII	456042	456173	131	N12:456042
chrXII	456045	456173	128	N12:456042
chrXII	456045	456175	130	N12:456042
chrXII	456045	456205	160	N12:456042
chrXII	456045	456205	160	N12:456220
chrXII	456056	456173	117	N12:456042
chrXII	456056	456175	119	N12:456042
chrXII	456056	456205	149	N12:456042
chrXII	456056	456205	149	N12:456220
chrXII	456056	456211	155	N12:456042
chrXII	456056	456211	155	N12:456220
chrXII	456056	456220	164	N12:456042
chrXII	456056	456220	164	N12:456220
chrXII	456063	456215	152	N12:456042
chrXII	456063	456215	152	N12:456220
chrXII	456064	456214	150	N12:456042
chrXII	456064	456214	150	N12:456220
chrXII	456066	456173	107	
chrXII	456067	456205	138	N12:456220
chrXII	456067	456211	144	N12:456220
chrXII	456067	456215	148	N12:456220
chrXII	456067	456220	153	N12:456220
chrXII	456067	456266	199	N12:456220
chrXII	456080	456173	93	
chrXII	456080	456205	125	N12:456220
chrXII	456080	456211	131	N12:456220
chrXII	456080	456220	140	N12:456220
chrXII	456084	456215	131	N12:456220
chrXII	456084	456243	159	N12:456220
chrXII	456084	456266	182	N12:456220
chrXII	456115	456215	100	N12:456220
chrXII	456115	456243	128	N12:456220
chrXII	456115	456266	151	N12:456220
chrXII	456115	456281	166	N12:456220
chrXII	456120	456290	170	N12:456220
chrXII	456124	456215	91	N12:456220
chrXII	456124	456266	142	N12:456220
chrXII	456124	456281	157	N12:456220
chrXII	456124	456333	209	N12:456220
chrXII	456181	456266	85	N12:456220
chrXII	456181	456281	100	N12:456220
chrXII	456181	456287	106	N12:456220
chrXII	456181	456306	125	N12:456220
chrXII	456181	456310	129	N12:456220
chrXII	456181	456329	148	N12:456220
chrXII	456181	456332	151	N12:456220
chrXII	456181	456333	152	N12:456220
chrXII	456181	456336	155	N12:456220
chrXII	456181	456349	168	N12:456220
chrXII	456181	456387	206	N12:456220
chrXII	456186	456333	147	N12:456220
chrXII	456187	456333	146	N12:456220
chrXII	456187	456349	162	N12:456220
chrXII	456194	456333	139	N12:456220
chrXII	456194	456336	142	N12:456220
chrXII	456213	456319	106	N12:456220
chrXII	456213	456320	107	N12:456220
chrXII	456213	456321	108	N12:456220
chrXII	456213	456333	120	N12:456220
chrXII	456213	456336	123	N12:456220
chrXII	456213	456349	136	N12:456220
chrXII	456213	456352	139	N12:456220
chrXII	456213	456387	174	N12:456220
chrXII	456213	456419	206	N12:456220
chrXII	456213	456419	206	N12:456421

Chr	Start	End	Length (bp)	Nucleosome_ID
chrXII	456221	456386	165	N12:456220
chrXII	456240	456333	93	N12:456220
chrXII	456240	456386	146	N12:456220
chrXII	456240	456387	147	N12:456220
chrXII	456240	456419	179	N12:456220
chrXII	456240	456419	179	N12:456421
chrXII	456240	456447	207	N12:456220
chrXII	456240	456447	207	N12:456421
chrXII	456288	456387	99	
chrXII	456288	456395	107	
chrXII	456288	456419	131	N12:456421
chrXII	456288	456447	159	N12:456421
chrXII	456288	456457	169	N12:456421
chrXII	456288	456471	183	N12:456421
chrXII	456290	456419	129	N12:456421
chrXII	456290	456447	157	N12:456421
chrXII	456290	456471	181	N12:456421
chrXII	456298	456447	149	N12:456421
chrXII	456310	456417	107	N12:456421
chrXII	456310	456419	109	N12:456421
chrXII	456310	456447	137	N12:456421
chrXII	456310	456471	161	N12:456421
chrXII	456361	456447	86	N12:456421
chrXII	456361	456466	105	N12:456421
chrXII	456361	456468	107	N12:456421
chrXII	456361	456470	109	N12:456421
chrXII	456361	456471	110	N12:456421
chrXII	456388	456471	83	N12:456421
chrXII	456388	456551	163	N12:456421
chrXII	456388	456551	163	N12:456568
chrXII	456444	456594	150	N12:456421
chrXII	456444	456594	150	N12:456568
chrXII	456444	456595	151	N12:456421
chrXII	456444	456595	151	N12:456568
chrXII	456444	456619	175	N12:456421
chrXII	456444	456619	175	N12:456568
chrXII	456445	456595	150	N12:456568
chrXII	456447	456594	147	N12:456568
chrXII	456447	456595	148	N12:456568
chrXII	456447	456608	161	N12:456568
chrXII	456447	456619	172	N12:456568
chrXII	456479	456585	106	N12:456568
chrXII	456479	456594	115	N12:456568
chrXII	456479	456608	129	N12:456568
chrXII	456479	456609	130	N12:456568
chrXII	456479	456653	174	N12:456568
chrXII	456483	456594	111	N12:456568
chrXII	456483	456608	125	N12:456568
chrXII	456483	456609	126	N12:456568
chrXII	456493	456599	106	N12:456568
chrXII	456493	456608	115	N12:456568
chrXII	456493	456653	160	N12:456568
chrXII	456508	456594	86	N12:456568
chrXII	456508	456608	100	N12:456568
chrXII	456508	456642	134	N12:456568
chrXII	456508	456653	145	N12:456568
chrXII	456508	456670	162	N12:456568
chrXII	456524	456695	171	N12:456568
chrXII	456587	456738	151	N12:456568
chrXII	456587	456738	151	N12:456730
chrXII	456588	456751	163	N12:456568
chrXII	456588	456751	163	N12:456730
chrXII	456594	456751	157	N12:456730
chrXII	456616	456772	156	N12:456730
chrXII	456634	456751	117	N12:456730
chrXII	456634	456762	128	N12:456730
chrXII	456634	456781	147	N12:456730
chrXII	456649	456803	154	N12:456730
chrXII	456653	456762	109	N12:456730
chrXII	456653	456803	150	N12:456730
chrXII	456733	456869	136	N12:456730
chrXII	456733	456869	136	N12:456891
chrXII	456766	456920	154	N12:456891
chrXII	456780	456887	107	N12:456891
chrXII	456780	456890	110	N12:456891
chrXII	456780	456920	140	N12:456891
chrXII	456794	456903	109	N12:456891
chrXII	456795	456903	108	N12:456891
chrXII	456795	456904	109	N12:456891
chrXII	456795	456905	110	N12:456891
chrXII	456797	456890	93	N12:456891
chrXII	456797	456920	123	N12:456891
chrXII	456803	456959	156	N12:456891
chrXII	456804	456911	107	N12:456891
chrXII	456804	456920	116	N12:456891
chrXII	456804	456960	156	N12:456891
chrXII	456817	456920	103	N12:456891
chrXII	456817	456971	154	N12:456891
chrXII	456938	457104	166	N12:457054
chrXII	456984	457118	134	N12:457054
chrXII	456984	457127	143	N12:457054
chrXII	456984	457130	146	N12:457054
chrXII	456984	457140	156	N12:457054
chrXII	456990	457127	137	N12:457054
chrXII	456990	457140	150	N12:457054
chrXII	457005	457166	161	N12:457054
chrXII	457021	457127	106	N12:457054
chrXII	457022	457205	183	N12:457054

Chr	Start	End	Length (bp)	Nucleosome_ID
chrXII	457022	457205	183	N12:457213
chrXII	457047	457207	160	N12:457054
chrXII	457047	457207	160	N12:457213
chrXII	457070	457200	130	N12:457054
chrXII	457070	457200	130	N12:457213
chrXII	457083	457217	134	N12:457213
chrXII	457115	457248	133	N12:457213
chrXII	457140	457305	165	N12:457213
chrXII	457140	457312	172	N12:457213
chrXII	457143	457248	105	N12:457213
chrXII	457143	457279	136	N12:457213
chrXII	457143	457316	173	N12:457213
chrXII	457152	457305	153	N12:457213
chrXII	457152	457312	160	N12:457213
chrXII	457153	457248	95	N12:457213
chrXII	457153	457279	126	N12:457213
chrXII	457153	457312	159	N12:457213
chrXII	457153	457316	163	N12:457213
chrXII	457153	457317	164	N12:457213
chrXII	457154	457312	158	N12:457213
chrXII	457154	457316	162	N12:457213
chrXII	457187	457316	129	N12:457213
chrXII	457187	457317	130	N12:457213
chrXII	457198	457346	148	N12:457213
chrXII	457198	457365	167	N12:457213
chrXII	457212	457365	153	N12:457213
chrXII	457214	457363	149	N12:457213
chrXII	457219	457312	93	N12:457213
chrXII	457219	457346	127	N12:457213
chrXII	457219	457363	144	N12:457213
chrXII	457219	457365	146	N12:457213
chrXII	457219	457422	203	N12:457213
chrXII	457227	457316	89	N12:457213
chrXII	457227	457363	136	N12:457213
chrXII	457227	457395	168	N12:457213
chrXII	457230	457395	165	N12:457213
chrXII	457230	457401	171	N12:457213
chrXII	457248	457395	147	
chrXII	457248	457401	153	
chrXII	457255	457365	110	
chrXII	457255	457407	152	
chrXII	457255	457422	167	
chrXII	457266	457365	99	
chrXII	457266	457407	141	
chrXII	457266	457422	156	
chrXII	457295	457378	83	
chrXII	457295	457384	89	
chrXII	457295	457390	95	
chrXII	457295	457391	96	
chrXII	457295	457393	98	
chrXII	457295	457394	99	
chrXII	457295	457395	100	
chrXII	457295	457397	102	
chrXII	457295	457401	106	
chrXII	457295	457402	107	
chrXII	457337	457476	139	N12:457494
chrXII	457337	457481	144	N12:457494
chrXII	457360	457476	116	N12:457494
chrXII	457360	457481	121	N12:457494
chrXII	457360	457530	170	N12:457494
chrXII	457370	457476	106	N12:457494
chrXII	457372	457476	104	N12:457494
chrXII	457420	457563	143	N12:457494
chrXII	457420	457567	147	N12:457494
chrXII	457420	457571	151	N12:457494
chrXII	457420	457572	152	N12:457494
chrXII	457437	457592	155	N12:457494
chrXII	457440	457593	153	N12:457494
chrXII	457540	457654	114	
chrXII	457553	457706	153	N12:457682
chrXII	457569	457736	167	N12:457682
chrXII	457569	457741	172	N12:457682
chrXII	457600	457736	136	N12:457682
chrXII	457600	457739	139	N12:457682
chrXII	457600	457741	141	N12:457682
chrXII	457606	457763	157	N12:457682
chrXII	457620	457773	153	N12:457682
chrXII	457620	457787	167	N12:457682
chrXII	457622	457771	149	N12:457682
chrXII	457634	457757	123	N12:457682
chrXII	457650	457805	155	N12:457682
chrXII	457667	457819	152	N12:457682
chrXII	457803	457959	156	N12:457885
chrXII	457816	457974	158	N12:457885
chrXII	457820	457953	133	N12:457885
chrXII	457872	458023	151	N12:457885
chrXII	457945	458104	159	
chrXII	457949	458059	110	
chrXII	457949	458101	152	
chrXII	457950	458101	151	
chrXII	457950	458103	153	
chrXII	457959	458110	151	
chrXII	457976	458059	83	
chrXII	457976	458119	143	
chrXII	457983	458089	106	
chrXII	457983	458101	118	
chrXII	457983	458103	120	
chrXII	457983	458137	154	N12:458145

Chr	Start	End	Length (bp)	Nucleosome_ID
chrXII	457986	458117	131	
chrXII	458025	458167	142	N12:458145
chrXII	458055	458137	82	N12:458145
chrXII	458055	458206	151	N12:458145
chrXII	458079	458227	148	N12:458145
chrXII	458087	458231	144	N12:458145
chrXII	458127	458277	150	N12:458145
chrXII	458130	458293	163	N12:458145
chrXII	458134	458272	138	N12:458145
chrXII	458134	458277	143	N12:458145
chrXII	458136	458295	159	N12:458145
chrXII	458143	458291	148	N12:458145
chrXII	458166	458321	155	N12:458145
chrXII	458166	458321	155	N12:458325
chrXII	458166	458322	156	N12:458145
chrXII	458166	458322	156	N12:458325
chrXII	458167	458322	155	N12:458145
chrXII	458167	458322	155	N12:458325
chrXII	458177	458322	145	N12:458325
chrXII	458177	458327	150	N12:458325
chrXII	458177	458350	173	N12:458325
chrXII	458179	458327	148	N12:458325
chrXII	458191	458321	130	N12:458325
chrXII	458191	458352	161	N12:458325
chrXII	458195	458322	127	N12:458325
chrXII	458195	458327	132	N12:458325
chrXII	458195	458350	155	N12:458325
chrXII	458204	458350	146	N12:458325
chrXII	458204	458380	176	N12:458325
chrXII	458291	458451	160	N12:458325
chrXII	458297	458451	154	N12:458325
chrXII	458298	458451	153	N12:458325
chrXII	458319	458472	153	N12:458325
chrXII	458338	458492	154	N12:458325
chrXII	458356	458493	137	
chrXII	458356	458508	152	N12:458524
chrXII	458359	458523	164	N12:458524
chrXII	458365	458493	128	
chrXII	458373	458493	120	
chrXII	458373	458530	157	N12:458524
chrXII	458374	458532	158	N12:458524
chrXII	458395	458501	106	N12:458524
chrXII	458395	458519	124	N12:458524
chrXII	458419	458587	168	N12:458524
chrXII	458508	458658	150	N12:458524
chrXII	458508	458658	150	N12:458681
chrXII	458508	458659	151	N12:458524
chrXII	458508	458659	151	N12:458681
chrXII	458746	458901	155	N12:458830
chrXII	458746	458912	166	N12:458830
chrXII	458750	458912	162	N12:458830
chrXII	458768	458900	132	N12:458830
chrXII	458768	458901	133	N12:458830
chrXII	458768	458912	144	N12:458830
chrXII	458769	458900	131	N12:458830
chrXII	458769	458901	132	N12:458830
chrXII	458769	458912	143	N12:458830
chrXII	458769	458913	144	N12:458830
chrXII	458770	458900	130	N12:458830
chrXII	458770	458901	131	N12:458830
chrXII	458770	458912	142	N12:458830
chrXII	458846	459002	156	N12:458830
chrXII	458846	459002	156	N12:458985
chrXII	458902	459048	146	N12:458985
chrXII	458908	459044	136	N12:458985
chrXII	458908	459048	140	N12:458985
chrXII	458910	459043	133	N12:458985
chrXII	458910	459066	156	N12:458985
chrXII	458913	459002	89	N12:458985
chrXII	458913	459038	125	N12:458985
chrXII	458913	459043	130	N12:458985
chrXII	458913	459044	131	N12:458985
chrXII	458913	459047	134	N12:458985
chrXII	458913	459048	135	N12:458985
chrXII	458913	459066	153	N12:458985
chrXII	458932	459038	106	N12:458985
chrXII	458933	459044	111	N12:458985
chrXII	458933	459047	114	N12:458985
chrXII	458933	459066	133	N12:458985
chrXII	458957	459044	87	N12:458985
chrXII	459050	459146	96	
chrXII	459089	459262	173	N12:459173
chrXII	459089	459265	176	N12:459173
chrXII	459095	459262	167	N12:459173
chrXII	459095	459265	170	N12:459173
chrXII	459098	459233	135	N12:459173
chrXII	459098	459262	164	N12:459173
chrXII	459166	459328	162	N12:459173
chrXII	459185	459328	143	N12:459173
chrXII	459204	459353	149	
chrXII	459204	459417	213	
chrXII	459204	459419	215	
chrXII	459227	459353	126	
chrXII	459227	459376	149	
chrXII	459227	459419	192	
chrXII	459227	459432	205	
chrXII	459235	459395	160	
chrXII	459236	459328	92	

Chr	Start	End	Length (bp)	Nucleosome_ID
chrXII	459236	459393	157	
chrXII	459236	459417	181	
chrXII	459236	459419	183	
chrXII	459264	459353	89	
chrXII	459264	459376	112	
chrXII	459264	459417	153	
chrXII	459264	459419	155	
chrXII	459264	459432	168	
chrXII	459264	459441	177	
chrXII	459265	459376	111	
chrXII	459265	459393	128	
chrXII	459265	459416	151	
chrXII	459265	459417	152	
chrXII	459265	459419	154	
chrXII	459265	459422	157	
chrXII	459265	459432	167	
chrXII	459265	459441	176	
chrXII	459265	459467	202	N12:459485
chrXII	459265	459469	204	N12:459485
chrXII	459267	459393	126	
chrXII	459267	459417	150	
chrXII	459267	459419	152	
chrXII	459267	459422	155	
chrXII	459267	459432	165	
chrXII	459267	459441	174	
chrXII	459267	459469	202	N12:459485
chrXII	459268	459374	106	
chrXII	459268	459376	108	
chrXII	459268	459393	125	
chrXII	459268	459416	148	
chrXII	459268	459417	149	
chrXII	459268	459419	151	
chrXII	459268	459422	154	
chrXII	459268	459431	163	
chrXII	459268	459432	164	
chrXII	459268	459441	173	
chrXII	459268	459467	199	N12:459485
chrXII	459268	459469	201	N12:459485
chrXII	459269	459376	107	
chrXII	459269	459393	124	
chrXII	459269	459413	144	
chrXII	459269	459417	148	
chrXII	459269	459419	150	
chrXII	459269	459422	153	
chrXII	459269	459432	163	
chrXII	459269	459467	198	N12:459485
chrXII	459269	459469	200	N12:459485
chrXII	459271	459419	148	
chrXII	459308	459414	106	
chrXII	459308	459419	111	
chrXII	459308	459467	159	N12:459485
chrXII	459308	459469	161	N12:459485
chrXII	459325	459466	141	N12:459485
chrXII	459326	459417	91	
chrXII	459326	459419	93	
chrXII	459326	459467	141	N12:459485
chrXII	459326	459469	143	N12:459485
chrXII	459408	459559	151	N12:459485
chrXII	459429	459558	129	N12:459485
chrXII	459429	459559	130	N12:459485
chrXII	459429	459579	150	N12:459485
chrXII	459430	459558	128	N12:459485
chrXII	459430	459559	129	N12:459485
chrXII	459430	459573	143	N12:459485
chrXII	459430	459579	149	N12:459485
chrXII	459519	459673	154	
chrXII	459521	459673	152	
chrXII	459602	459756	154	N12:459720
chrXII	459602	459759	157	N12:459720
chrXII	459604	459722	118	N12:459720
chrXII	459604	459756	152	N12:459720
chrXII	459604	459759	155	N12:459720
chrXII	459604	459774	170	N12:459720
chrXII	459604	459796	192	N12:459720
chrXII	459604	459798	194	N12:459720
chrXII	459604	459804	200	N12:459720
chrXII	459604	459806	202	N12:459720
chrXII	459618	459756	138	N12:459720
chrXII	459618	459774	156	N12:459720
chrXII	459618	459796	178	N12:459720
chrXII	459621	459756	135	N12:459720
chrXII	459621	459774	153	N12:459720
chrXII	459625	459756	131	N12:459720
chrXII	459625	459774	149	N12:459720
chrXII	459625	459796	171	N12:459720
chrXII	459625	459798	173	N12:459720
chrXII	459625	459804	179	N12:459720
chrXII	459625	459806	181	N12:459720
chrXII	459628	459756	128	N12:459720
chrXII	459628	459774	146	N12:459720
chrXII	459628	459796	168	N12:459720
chrXII	459628	459798	170	N12:459720
chrXII	459628	459804	176	N12:459720
chrXII	459628	459806	178	N12:459720
chrXII	459633	459756	123	N12:459720
chrXII	459633	459774	141	N12:459720
chrXII	459633	459796	163	N12:459720
chrXII	459633	459798	165	N12:459720

Chr	Start	End	Length (bp)	Nucleosome_ID
chrXII	459633	459804	171	N12:459720
chrXII	459633	459806	173	N12:459720
chrXII	459648	459796	148	N12:459720
chrXII	459673	459804	131	N12:459720
chrXII	459673	459806	133	N12:459720
chrXII	459686	459793	107	N12:459720
chrXII	459686	459796	110	N12:459720
chrXII	459686	459798	112	N12:459720
chrXII	459686	459799	113	N12:459720
chrXII	459686	459801	115	N12:459720
chrXII	459686	459804	118	N12:459720
chrXII	459686	459806	120	N12:459720
chrXII	459686	459856	170	N12:459720
chrXII	459688	459793	105	N12:459720
chrXII	459688	459851	163	N12:459720
chrXII	459698	459804	106	N12:459720
chrXII	459698	459806	108	N12:459720
chrXII	459698	459851	153	N12:459720
chrXII	459698	459869	171	N12:459720
chrXII	459818	459980	162	N12:459899
chrXII	459834	459969	135	N12:459899
chrXII	459846	459969	123	N12:459899
chrXII	459846	460002	156	N12:459899
chrXII	459899	460068	169	N12:459899
chrXII	459899	460068	169	N12:460071
chrXII	459930	460057	127	N12:460071
chrXII	460017	460128	111	N12:460071
chrXII	460017	460159	142	N12:460071
chrXII	460032	460159	127	N12:460071
chrXII	460102	460251	149	
chrXII	460102	460266	164	
chrXII	460112	460251	139	
chrXII	460112	460266	154	
chrXII	460188	460366	178	N12:460347
chrXII	460243	460384	141	N12:460347
chrXII	460256	460415	159	N12:460347
chrXII	460262	460436	174	N12:460347
chrXII	460302	460477	175	N12:460347
chrXII	460314	460446	132	N12:460347
chrXII	460402	460571	169	
chrXII	460461	460595	134	N12:460603
chrXII	460487	460616	129	N12:460603
chrXII	460493	460595	102	N12:460603
chrXII	460493	460659	166	N12:460603
chrXII	460512	460595	83	N12:460603
chrXII	460512	460639	127	N12:460603
chrXII	460521	460659	138	N12:460603
chrXII	460602	460763	161	N12:460603
chrXII	460846	460979	133	N12:460826
chrXII	460863	461022	159	
chrXII	460871	461006	135	
chrXII	460894	460979	85	
chrXII	460894	461045	151	
chrXII	460894	461097	203	
chrXII	460906	461069	163	
chrXII	460927	461045	118	
chrXII	460927	461097	170	
chrXII	460937	461071	134	
chrXII	460937	461098	161	
chrXII	460988	461071	83	
chrXII	460988	461095	107	
chrXII	460988	461096	108	
chrXII	460988	461098	110	
chrXII	461013	461146	133	N12:461125
chrXII	461056	461146	90	N12:461125
chrXII	461056	461148	92	N12:461125
chrXII	461056	461199	143	N12:461125
chrXII	461056	461212	156	N12:461125
chrXII	461056	461214	158	N12:461125
chrXII	461056	461215	159	N12:461125
chrXII	461056	461229	173	N12:461125
chrXII	461060	461146	86	N12:461125
chrXII	461060	461212	152	N12:461125
chrXII	461060	461214	154	N12:461125
chrXII	461060	461215	155	N12:461125
chrXII	461060	461229	169	N12:461125
chrXII	461068	461199	131	N12:461125
chrXII	461068	461214	146	N12:461125
chrXII	461068	461246	178	N12:461125
chrXII	461069	461199	130	N12:461125
chrXII	461069	461214	145	N12:461125
chrXII	461069	461246	177	N12:461125
chrXII	461082	461188	106	N12:461125
chrXII	461082	461199	117	N12:461125
chrXII	461082	461212	130	N12:461125
chrXII	461082	461214	132	N12:461125
chrXII	461082	461215	133	N12:461125
chrXII	461082	461229	147	N12:461125
chrXII	461082	461246	164	N12:461125
chrXII	461095	461246	151	N12:461125
chrXII	461178	461333	155	N12:461308
chrXII	461196	461352	156	N12:461308
chrXII	461218	461364	146	N12:461308
chrXII	461218	461384	166	N12:461308
chrXII	461218	461388	170	N12:461308
chrXII	461241	461384	143	N12:461308
chrXII	461241	461388	147	N12:461308
chrXII	461252	461408	156	N12:461308

Chr	Start	End	Length (bp)	Nucleosome_ID
chrXII	461260	461367	107	N12:461308
chrXII	461327	461481	154	N12:461308
chrXII	461397	461551	154	N12:461507
chrXII	461430	461537	107	N12:461507
chrXII	461431	461592	161	N12:461507
chrXII	461432	461592	160	N12:461507
chrXII	461436	461592	156	N12:461507
chrXII	461460	461622	162	N12:461507
chrXII	461460	461623	163	N12:461507
chrXII	461471	461578	107	N12:461507
chrXII	461471	461592	121	N12:461507
chrXII	461471	461622	151	N12:461507
chrXII	461471	461623	152	N12:461507
chrXII	461483	461633	150	N12:461507
chrXII	461536	461685	149	N12:461692
chrXII	461560	461721	161	N12:461692
chrXII	461606	461757	151	N12:461692
chrXII	461606	461758	152	N12:461692
chrXII	461606	461766	160	N12:461692
chrXII	461606	461767	161	N12:461692
chrXII	461628	461758	130	N12:461692
chrXII	461628	461766	138	N12:461692
chrXII	461628	461800	172	N12:461692
chrXII	461629	461721	92	N12:461692
chrXII	461629	461757	128	N12:461692
chrXII	461629	461758	129	N12:461692
chrXII	461629	461766	137	N12:461692
chrXII	461629	461800	171	N12:461692
chrXII	461662	461758	96	N12:461692
chrXII	461662	461832	170	N12:461692
chrXII	461676	461810	134	N12:461692
chrXII	461687	461800	113	N12:461692
chrXII	461687	461809	122	N12:461692
chrXII	461687	461839	152	N12:461692
chrXII	461805	461933	128	N12:461891
chrXII	461805	461951	146	N12:461891
chrXII	461821	461933	112	N12:461891
chrXII	461821	461972	151	N12:461891
chrXII	461823	461951	128	N12:461891
chrXII	461823	462001	178	N12:461891
chrXII	461846	461952	106	N12:461891
chrXII	461846	462001	155	N12:461891
chrXII	461846	462021	175	N12:461891
chrXII	461846	462037	191	N12:461891
chrXII	461860	461951	91	N12:461891
chrXII	461860	462001	141	N12:461891
chrXII	461860	462021	161	N12:461891
chrXII	461860	462037	177	N12:461891
chrXII	461902	462001	99	N12:461891
chrXII	461902	462008	106	N12:461891
chrXII	461902	462021	119	N12:461891
chrXII	461902	462037	135	N12:461891
chrXII	461902	462062	160	N12:461891
chrXII	461902	462062	160	N12:462072
chrXII	461910	462001	91	N12:461891
chrXII	461910	462016	106	N12:461891
chrXII	461910	462021	111	N12:461891
chrXII	461910	462037	127	N12:461891
chrXII	461910	462062	152	N12:461891
chrXII	461910	462062	152	N12:462072
chrXII	461920	462099	179	N12:462072
chrXII	461925	462060	135	N12:462072
chrXII	461925	462090	165	N12:462072
chrXII	461938	462060	122	N12:462072
chrXII	461938	462090	152	N12:462072
chrXII	461938	462136	198	N12:462072
chrXII	461977	462090	113	N12:462072
chrXII	461977	462136	159	N12:462072
chrXII	461977	462160	183	N12:462072
chrXII	461984	462090	106	N12:462072
chrXII	461984	462133	149	N12:462072
chrXII	462013	462171	158	N12:462072
chrXII	462022	462171	149	N12:462072
chrXII	462030	462136	106	N12:462072
chrXII	462032	462160	128	N12:462072
chrXII	462059	462166	107	N12:462072
chrXII	462059	462167	108	N12:462072
chrXII	462059	462171	112	N12:462072
chrXII	462059	462193	134	N12:462072
chrXII	462059	462212	153	N12:462072
chrXII	462063	462134	71	N12:462072
chrXII	462063	462170	107	N12:462072
chrXII	462063	462171	108	N12:462072
chrXII	462063	462193	130	N12:462072
chrXII	462063	462207	144	N12:462072
chrXII	462063	462212	149	N12:462072
chrXII	462113	462275	162	N12:462253
chrXII	462128	462278	150	N12:462253
chrXII	462168	462275	107	N12:462253
chrXII	462170	462300	130	N12:462253
chrXII	462170	462324	154	N12:462253
chrXII	462170	462330	160	N12:462253
chrXII	462176	462300	124	N12:462253
chrXII	462176	462324	148	N12:462253
chrXII	462176	462330	154	N12:462253
chrXII	462179	462349	170	N12:462253
chrXII	462179	462355	176	N12:462253
chrXII	462180	462300	120	N12:462253

Chr	Start	End	Length (bp)	Nucleosome_ID
chrXII	462180	462324	144	N12:462253
chrXII	462180	462327	147	N12:462253
chrXII	462180	462330	150	N12:462253
chrXII	462228	462336	108	N12:462253
chrXII	462229	462336	107	N12:462253
chrXII	462229	462337	108	N12:462253
chrXII	462229	462349	120	N12:462253
chrXII	462229	462373	144	N12:462253
chrXII	462229	462382	153	N12:462253
chrXII	462229	462388	159	N12:462253
chrXII	462229	462407	178	N12:462253
chrXII	462229	462423	194	N12:462253
chrXII	462229	462425	196	N12:462253
chrXII	462232	462338	106	N12:462253
chrXII	462232	462349	117	N12:462253
chrXII	462232	462355	123	N12:462253
chrXII	462232	462373	141	N12:462253
chrXII	462232	462382	150	N12:462253
chrXII	462232	462407	175	N12:462253
chrXII	462232	462419	187	N12:462253
chrXII	462232	462423	191	N12:462253
chrXII	462232	462425	193	N12:462253
chrXII	462236	462373	137	N12:462253
chrXII	462236	462425	189	N12:462253
chrXII	462237	462375	138	N12:462253
chrXII	462237	462382	145	N12:462253
chrXII	462250	462382	132	N12:462253
chrXII	462250	462407	157	N12:462253
chrXII	462250	462423	173	N12:462253
chrXII	462250	462425	175	N12:462253
chrXII	462251	462373	122	N12:462253
chrXII	462251	462406	155	N12:462253
chrXII	462251	462407	156	N12:462253
chrXII	462251	462423	172	N12:462253
chrXII	462251	462424	173	N12:462253
chrXII	462251	462425	174	N12:462253
chrXII	462251	462439	188	N12:462253
chrXII	462253	462406	153	N12:462253
chrXII	462253	462407	154	N12:462253
chrXII	462253	462424	171	N12:462253
chrXII	462253	462425	172	N12:462253
chrXII	462256	462373	117	N12:462253
chrXII	462256	462388	132	N12:462253
chrXII	462256	462406	150	N12:462253
chrXII	462256	462407	151	N12:462253
chrXII	462256	462419	163	N12:462253
chrXII	462256	462423	167	N12:462253
chrXII	462256	462424	168	N12:462253
chrXII	462256	462425	169	N12:462253
chrXII	462256	462439	183	N12:462253
chrXII	462256	462446	190	N12:462253
chrXII	462257	462365	108	N12:462253
chrXII	462258	462407	149	N12:462253
chrXII	462258	462423	165	N12:462253
chrXII	462258	462425	167	N12:462253
chrXII	462262	462368	106	N12:462253
chrXII	462262	462373	111	N12:462253
chrXII	462262	462407	145	N12:462253
chrXII	462262	462419	157	N12:462253
chrXII	462262	462423	161	N12:462253
chrXII	462262	462424	162	N12:462253
chrXII	462262	462425	163	N12:462253
chrXII	462262	462439	177	N12:462253
chrXII	462263	462385	122	N12:462253
chrXII	462263	462407	144	N12:462253
chrXII	462263	462423	160	N12:462253
chrXII	462263	462424	161	N12:462253
chrXII	462263	462425	162	N12:462253
chrXII	462272	462407	135	N12:462253
chrXII	462272	462419	147	N12:462253
chrXII	462272	462423	151	N12:462253
chrXII	462272	462425	153	N12:462253
chrXII	462275	462423	148	N12:462253
chrXII	462275	462425	150	N12:462253
chrXII	462277	462373	96	
chrXII	462277	462382	105	
chrXII	462277	462383	106	
chrXII	462277	462388	111	
chrXII	462277	462407	130	
chrXII	462277	462419	142	
chrXII	462277	462423	146	
chrXII	462277	462424	147	
chrXII	462277	462425	148	
chrXII	462277	462439	162	
chrXII	462277	462460	183	
chrXII	462278	462373	95	
chrXII	462278	462382	104	
chrXII	462278	462406	128	
chrXII	462278	462407	129	
chrXII	462278	462418	140	
chrXII	462278	462419	141	
chrXII	462278	462420	142	
chrXII	462278	462423	145	
chrXII	462278	462424	146	
chrXII	462278	462425	147	
chrXII	462278	462439	161	
chrXII	462278	462446	168	
chrXII	462278	462460	182	

Chr	Start	End	Length (bp)	Nucleosome_ID
chrXII	462278	462461	183	
chrXII	462278	462479	201	
chrXII	462281	462425	144	
chrXII	462287	462423	136	
chrXII	462287	462425	138	
chrXII	462287	462432	145	
chrXII	462287	462439	152	
chrXII	462289	462377	88	
chrXII	462289	462407	118	
chrXII	462289	462423	134	
chrXII	462289	462424	135	
chrXII	462289	462425	136	
chrXII	462289	462439	150	
chrXII	462290	462423	133	
chrXII	462290	462425	135	
chrXII	462296	462407	111	
chrXII	462296	462423	127	
chrXII	462296	462424	128	
chrXII	462296	462425	129	
chrXII	462296	462439	143	
chrXII	462296	462446	150	
chrXII	462296	462472	176	
chrXII	462296	462479	183	
chrXII	462298	462382	84	
chrXII	462298	462405	107	
chrXII	462298	462407	109	
chrXII	462298	462419	121	
chrXII	462298	462423	125	
chrXII	462298	462425	127	
chrXII	462298	462446	148	
chrXII	462298	462460	162	
chrXII	462298	462461	163	
chrXII	462299	462406	107	
chrXII	462299	462423	124	
chrXII	462299	462425	126	
chrXII	462299	462460	161	
chrXII	462299	462461	162	
chrXII	462302	462424	122	
chrXII	462302	462425	123	
chrXII	462302	462439	137	
chrXII	462302	462479	177	
chrXII	462308	462479	171	
chrXII	462317	462425	108	
chrXII	462317	462477	160	
chrXII	462318	462407	89	
chrXII	462318	462419	101	
chrXII	462318	462423	105	
chrXII	462318	462425	107	
chrXII	462318	462460	142	
chrXII	462318	462461	143	
chrXII	462423	462569	146	N12:462515
chrXII	462436	462577	141	N12:462515
chrXII	462436	462597	161	N12:462515
chrXII	462437	462547	110	N12:462515
chrXII	462437	462577	140	N12:462515
chrXII	462439	462577	138	N12:462515
chrXII	462440	462577	137	N12:462515
chrXII	462447	462577	130	N12:462515
chrXII	462447	462597	150	N12:462515
chrXII	462459	462618	159	N12:462515
chrXII	462595	462732	137	N12:462669
chrXII	462595	462747	152	N12:462669
chrXII	462595	462749	154	N12:462669
chrXII	462597	462749	152	N12:462669
chrXII	462624	462732	108	N12:462669
chrXII	462624	462747	123	N12:462669
chrXII	462624	462749	125	N12:462669
chrXII	462637	462789	152	N12:462669
chrXII	462711	462864	153	N12:462833
chrXII	462724	462874	150	N12:462833
chrXII	462764	462913	149	N12:462833
chrXII	462764	462916	152	N12:462833
chrXII	462794	462951	157	N12:462833
chrXII	462796	462913	117	N12:462833
chrXII	462796	462916	120	N12:462833
chrXII	462871	462990	119	N12:462990
chrXII	462885	463047	162	N12:462990
chrXII	462885	463050	165	N12:462990
chrXII	462885	463056	171	N12:462990
chrXII	462885	463059	174	N12:462990
chrXII	462885	463099	214	N12:462990
chrXII	462889	462996	107	N12:462990
chrXII	462889	463045	156	N12:462990
chrXII	462889	463048	159	N12:462990
chrXII	462889	463051	162	N12:462990
chrXII	462889	463053	164	N12:462990
chrXII	462889	463059	170	N12:462990
chrXII	462889	463061	172	N12:462990
chrXII	462889	463066	177	N12:462990
chrXII	462890	463050	160	N12:462990
chrXII	462890	463059	169	N12:462990
chrXII	462891	463047	156	N12:462990
chrXII	462891	463056	165	N12:462990
chrXII	462891	463061	170	N12:462990
chrXII	462911	463047	136	N12:462990
chrXII	462911	463050	139	N12:462990
chrXII	462911	463051	140	N12:462990
chrXII	462911	463053	142	N12:462990

Chr	Start	End	Length (bp)	Nucleosome_ID
chrXII	462911	463056	145	N12:462990
chrXII	462911	463062	151	N12:462990
chrXII	462911	463099	188	N12:462990
chrXII	462912	463045	133	N12:462990
chrXII	462912	463048	136	N12:462990
chrXII	462912	463051	139	N12:462990
chrXII	462912	463053	141	N12:462990
chrXII	462912	463059	147	N12:462990
chrXII	462918	463045	127	N12:462990
chrXII	462918	463048	130	N12:462990
chrXII	462918	463051	133	N12:462990
chrXII	462918	463053	135	N12:462990
chrXII	462918	463059	141	N12:462990
chrXII	462919	463048	129	N12:462990
chrXII	462919	463050	131	N12:462990
chrXII	462919	463051	132	N12:462990
chrXII	462919	463056	137	N12:462990
chrXII	462919	463059	140	N12:462990
chrXII	462919	463062	143	N12:462990
chrXII	462919	463066	147	N12:462990
chrXII	462919	463074	155	N12:462990
chrXII	462919	463099	180	N12:462990
chrXII	462920	463045	125	N12:462990
chrXII	462920	463048	128	N12:462990
chrXII	462920	463050	130	N12:462990
chrXII	462920	463051	131	N12:462990
chrXII	462949	463050	101	N12:462990
chrXII	462949	463056	107	N12:462990
chrXII	462949	463099	150	N12:462990
chrXII	462949	463138	189	N12:463152
chrXII	462949	463138	189	N12:462990
chrXII	462988	463095	107	N12:462990
chrXII	462988	463099	111	N12:462990
chrXII	462988	463138	150	N12:463152
chrXII	462988	463138	150	N12:462990
chrXII	463046	463209	163	N12:463152
chrXII	463055	463217	162	N12:463152
chrXII	463059	463216	157	N12:463152
chrXII	463059	463234	175	N12:463152
chrXII	463066	463216	150	N12:463152
chrXII	463066	463234	168	N12:463152
chrXII	463066	463245	179	N12:463152
chrXII	463096	463234	138	N12:463152
chrXII	463096	463245	149	N12:463152
chrXII	463096	463249	153	N12:463152
chrXII	463097	463253	156	N12:463152
chrXII	463101	463234	133	N12:463152
chrXII	463101	463245	144	N12:463152
chrXII	463108	463245	137	N12:463152
chrXII	463108	463260	152	N12:463152
chrXII	463196	463353	157	N12:463325
chrXII	463248	463405	157	N12:463325
chrXII	463257	463404	147	N12:463325
chrXII	463257	463414	157	N12:463325
chrXII	463261	463414	153	N12:463325
chrXII	463268	463414	146	N12:463325
chrXII	463278	463414	136	N12:463325
chrXII	463333	463483	150	N12:463325
chrXII	463333	463483	150	N12:463504
chrXII	463358	463511	153	N12:463504
chrXII	463358	463529	171	N12:463504
chrXII	463381	463529	148	N12:463504
chrXII	463381	463539	158	N12:463504
chrXII	463385	463511	126	N12:463504
chrXII	463385	463529	144	N12:463504
chrXII	463385	463535	150	N12:463504
chrXII	463385	463539	154	N12:463504
chrXII	463410	463573	163	N12:463504
chrXII	463414	463521	107	N12:463504
chrXII	463414	463535	121	N12:463504
chrXII	463414	463539	125	N12:463504
chrXII	463414	463575	161	N12:463504
chrXII	463414	463583	169	N12:463504
chrXII	463463	463603	140	N12:463504
chrXII	463471	463608	137	N12:463504
chrXII	463535	463688	153	
chrXII	463535	463689	154	
chrXII	463535	463690	155	
chrXII	463535	463692	157	
chrXII	463535	463704	169	
chrXII	463535	463710	175	
chrXII	463535	463712	177	
chrXII	463541	463689	148	
chrXII	463541	463692	151	
chrXII	463559	463689	130	
chrXII	463559	463704	145	
chrXII	463559	463710	151	
chrXII	463559	463712	153	
chrXII	463658	463815	157	N12:463786
chrXII	463658	463852	194	N12:463786
chrXII	463658	463870	212	N12:463786
chrXII	463690	463849	159	N12:463786
chrXII	463690	463870	180	N12:463786
chrXII	463692	463840	148	N12:463786
chrXII	463697	463815	118	N12:463786
chrXII	463697	463849	152	N12:463786
chrXII	463697	463852	155	N12:463786
chrXII	463697	463861	164	N12:463786

Chr	Start	End	Length (bp)	Nucleosome_ID
chrXII	463697	463870	173	N12:463786
chrXII	463699	463849	150	N12:463786
chrXII	463707	463849	142	N12:463786
chrXII	463707	463852	145	N12:463786
chrXII	463707	463861	154	N12:463786
chrXII	463707	463870	163	N12:463786
chrXII	463713	463849	136	N12:463786
chrXII	463713	463852	139	N12:463786
chrXII	463713	463861	148	N12:463786
chrXII	463713	463870	157	N12:463786
chrXII	463713	463909	196	N12:463786
chrXII	463724	463883	159	N12:463786
chrXII	463729	463879	150	N12:463786
chrXII	463763	463849	86	N12:463786
chrXII	463763	463870	107	N12:463786
chrXII	463763	463909	146	N12:463786
chrXII	463860	463991	131	N12:463954
chrXII	463862	464000	138	N12:463954
chrXII	463862	464023	161	N12:463954
chrXII	463870	464023	153	N12:463954
chrXII	463878	464033	155	N12:463954
chrXII	463884	464023	139	N12:463954
chrXII	463974	464128	154	N12:463954
chrXII	463974	464128	154	N12:464114
chrXII	464001	464158	157	N12:464114
chrXII	464013	464160	147	N12:464114
chrXII	464013	464166	153	N12:464114
chrXII	464038	464166	128	N12:464114
chrXII	464038	464211	173	N12:464114
chrXII	464057	464224	167	N12:464114
chrXII	464069	464210	141	N12:464114
chrXII	464070	464160	90	N12:464114
chrXII	464070	464166	96	N12:464114
chrXII	464070	464210	140	N12:464114
chrXII	464070	464211	141	N12:464114
chrXII	464070	464213	143	N12:464114
chrXII	464101	464269	168	N12:464114
chrXII	464101	464269	168	N12:464279
chrXII	464132	464301	169	N12:464114
chrXII	464132	464301	169	N12:464279
chrXII	464137	464269	132	N12:464114
chrXII	464137	464269	132	N12:464279
chrXII	464152	464303	151	N12:464279
chrXII	464195	464345	150	N12:464279
chrXII	464205	464360	155	N12:464279
chrXII	464232	464406	174	N12:464279
chrXII	464232	464406	174	N12:464427
chrXII	464233	464360	127	N12:464279
chrXII	464233	464377	144	N12:464279
chrXII	464233	464380	147	N12:464279
chrXII	464233	464382	149	N12:464279
chrXII	464233	464386	153	N12:464279
chrXII	464234	464377	143	N12:464279
chrXII	464234	464380	146	N12:464279
chrXII	464234	464382	148	N12:464279
chrXII	464235	464377	142	N12:464279
chrXII	464235	464380	145	N12:464279
chrXII	464235	464382	147	N12:464279
chrXII	464235	464386	151	N12:464279
chrXII	464266	464430	164	N12:464279
chrXII	464266	464430	164	N12:464427
chrXII	464275	464426	151	N12:464279
chrXII	464275	464426	151	N12:464427
chrXII	464319	464475	156	N12:464427
chrXII	464354	464503	149	N12:464427
chrXII	464358	464532	174	N12:464427
chrXII	464384	464518	134	N12:464427
chrXII	464391	464538	147	N12:464427
chrXII	464392	464537	145	N12:464427
chrXII	464415	464560	145	N12:464427
chrXII	464434	464584	150	N12:464427
chrXII	464504	464649	145	N12:464616
chrXII	464504	464668	164	N12:464616
chrXII	464504	464686	182	N12:464616
chrXII	464514	464649	135	N12:464616
chrXII	464514	464668	154	N12:464616
chrXII	464514	464686	172	N12:464616
chrXII	464534	464640	106	N12:464616
chrXII	464534	464649	115	N12:464616
chrXII	464534	464668	134	N12:464616
chrXII	464534	464686	152	N12:464616
chrXII	464547	464649	102	N12:464616
chrXII	464547	464686	139	N12:464616
chrXII	464547	464697	150	N12:464616
chrXII	464547	464705	158	N12:464616
chrXII	464547	464706	159	N12:464616
chrXII	464553	464708	155	N12:464616
chrXII	464559	464706	147	N12:464616
chrXII	464728	464888	160	N12:464796
chrXII	464838	465007	169	N12:464962
chrXII	464884	465032	148	N12:464962
chrXII	464886	465026	140	N12:464962
chrXII	464920	465095	175	N12:464962
chrXII	465006	465158	152	N12:465169
chrXII	465040	465204	164	N12:465169
chrXII	465055	465176	121	N12:465169
chrXII	465063	465223	160	N12:465169
chrXII	465159	465310	151	N12:465169

Chr	Start	End	Length (bp)	Nucleosome_ID
chrXII	465159	465342	183	N12:465169
chrXII	465159	465342	183	N12:465356
chrXII	465159	465348	189	N12:465169
chrXII	465159	465348	189	N12:465356
chrXII	465159	465357	198	N12:465169
chrXII	465159	465357	198	N12:465356
chrXII	465179	465310	131	N12:465169
chrXII	465182	465312	130	N12:465169
chrXII	465193	465310	117	
chrXII	465193	465312	119	
chrXII	465193	465342	149	N12:465356
chrXII	465193	465348	155	N12:465356
chrXII	465193	465357	164	N12:465356
chrXII	465200	465352	152	N12:465356
chrXII	465201	465342	141	N12:465356
chrXII	465201	465345	144	N12:465356
chrXII	465201	465351	150	N12:465356
chrXII	465203	465310	107	
chrXII	465204	465342	138	N12:465356
chrXII	465204	465348	144	N12:465356
chrXII	465204	465352	148	N12:465356
chrXII	465204	465357	153	N12:465356
chrXII	465204	465403	199	N12:465356
chrXII	465204	465418	214	N12:465356
chrXII	465217	465310	93	
chrXII	465217	465342	125	N12:465356
chrXII	465217	465348	131	N12:465356
chrXII	465217	465357	140	N12:465356
chrXII	465221	465352	131	N12:465356
chrXII	465221	465375	154	N12:465356
chrXII	465221	465380	159	N12:465356
chrXII	465221	465403	182	N12:465356
chrXII	465252	465352	100	N12:465356
chrXII	465252	465380	128	N12:465356
chrXII	465252	465403	151	N12:465356
chrXII	465252	465418	166	N12:465356
chrXII	465257	465427	170	N12:465356
chrXII	465261	465352	91	N12:465356
chrXII	465261	465403	142	N12:465356
chrXII	465261	465418	157	N12:465356
chrXII	465261	465470	209	N12:465356
chrXII	465318	465403	85	N12:465356
chrXII	465318	465418	100	N12:465356
chrXII	465318	465447	129	N12:465356
chrXII	465318	465469	151	N12:465356
chrXII	465318	465470	152	N12:465356
chrXII	465318	465473	155	N12:465356
chrXII	465318	465486	168	N12:465356
chrXII	465318	465524	206	N12:465356
chrXII	465318	465524	206	N12:465541
chrXII	465323	465470	147	N12:465356
chrXII	465324	465470	146	N12:465356
chrXII	465324	465486	162	N12:465356
chrXII	465331	465470	139	N12:465356
chrXII	465331	465473	142	N12:465356
chrXII	465350	465456	106	N12:465356
chrXII	465350	465457	107	N12:465356
chrXII	465350	465458	108	N12:465356
chrXII	465350	465470	120	N12:465356
chrXII	465350	465473	123	N12:465356
chrXII	465350	465486	136	N12:465356
chrXII	465350	465524	174	N12:465356
chrXII	465350	465524	174	N12:465541
chrXII	465350	465556	206	N12:465356
chrXII	465350	465556	206	N12:465541
chrXII	465358	465523	165	N12:465356
chrXII	465358	465523	165	N12:465541
chrXII	465377	465470	93	N12:465356
chrXII	465377	465484	107	N12:465356
chrXII	465377	465523	146	N12:465356
chrXII	465377	465523	146	N12:465541
chrXII	465377	465524	147	N12:465356
chrXII	465377	465524	147	N12:465541
chrXII	465377	465556	179	N12:465356
chrXII	465377	465556	179	N12:465541
chrXII	465377	465584	207	N12:465356
chrXII	465377	465584	207	N12:465541
chrXII	465425	465524	99	N12:465541
chrXII	465425	465532	107	N12:465541
chrXII	465425	465556	131	N12:465541
chrXII	465425	465584	159	N12:465541
chrXII	465425	465594	169	N12:465541
chrXII	465425	465608	183	N12:465541
chrXII	465427	465524	97	N12:465541
chrXII	465427	465556	129	N12:465541
chrXII	465427	465584	157	N12:465541
chrXII	465427	465608	181	N12:465541
chrXII	465435	465584	149	N12:465541
chrXII	465447	465556	109	N12:465541
chrXII	465447	465584	137	N12:465541
chrXII	465447	465608	161	N12:465541
chrXII	465498	465584	86	N12:465541
chrXII	465498	465603	105	N12:465541
chrXII	465498	465605	107	N12:465541
chrXII	465498	465606	108	N12:465541
chrXII	465498	465607	109	N12:465541
chrXII	465498	465608	110	N12:465541
chrXII	465525	465608	83	N12:465541

Chr	Start	End	Length (bp)	Nucleosome_ID
chrXII	465525	465688	163	N12:465541
chrXII	465525	465688	163	N12:465711
chrXII	465562	465739	177	N12:465541
chrXII	465562	465739	177	N12:465711
chrXII	465581	465731	150	N12:465711
chrXII	465581	465732	151	N12:465711
chrXII	465581	465756	175	N12:465711
chrXII	465582	465732	150	N12:465711
chrXII	465584	465731	147	N12:465711
chrXII	465584	465732	148	N12:465711
chrXII	465584	465745	161	N12:465711
chrXII	465584	465756	172	N12:465711
chrXII	465616	465722	106	N12:465711
chrXII	465616	465731	115	N12:465711
chrXII	465616	465745	129	N12:465711
chrXII	465616	465746	130	N12:465711
chrXII	465616	465790	174	N12:465711
chrXII	465620	465731	111	N12:465711
chrXII	465620	465745	125	N12:465711
chrXII	465620	465746	126	N12:465711
chrXII	465630	465736	106	N12:465711
chrXII	465630	465745	115	N12:465711
chrXII	465630	465790	160	N12:465711
chrXII	465645	465731	86	N12:465711
chrXII	465645	465745	100	N12:465711
chrXII	465645	465779	134	N12:465711
chrXII	465645	465790	145	N12:465711
chrXII	465645	465807	162	N12:465711
chrXII	465661	465832	171	N12:465711
chrXII	465724	465875	151	N12:465711
chrXII	465724	465875	151	N12:465875
chrXII	465725	465888	163	N12:465711
chrXII	465725	465888	163	N12:465875
chrXII	465731	465888	157	N12:465711
chrXII	465731	465888	157	N12:465875
chrXII	465753	465909	156	N12:465875
chrXII	465771	465899	128	N12:465875
chrXII	465771	465918	147	N12:465875
chrXII	465786	465940	154	N12:465875
chrXII	465790	465899	109	N12:465875
chrXII	465790	465940	150	N12:465875
chrXII	465903	466057	154	N12:466028
chrXII	465917	466024	107	N12:466028
chrXII	465917	466027	110	N12:466028
chrXII	465917	466057	140	N12:466028
chrXII	465931	466040	109	N12:466028
chrXII	465932	466040	108	N12:466028
chrXII	465932	466041	109	N12:466028
chrXII	465934	466027	93	N12:466028
chrXII	465934	466057	123	N12:466028
chrXII	465940	466096	156	N12:466028
chrXII	465941	466048	107	N12:466028
chrXII	465941	466057	116	N12:466028
chrXII	465941	466097	156	N12:466028
chrXII	465954	466057	103	N12:466028
chrXII	465954	466108	154	N12:466028
chrXII	466075	466241	166	N12:466190
chrXII	466105	466258	153	N12:466190
chrXII	466121	466255	134	N12:466190
chrXII	466121	466264	143	N12:466190
chrXII	466121	466267	146	N12:466190
chrXII	466121	466277	156	N12:466190
chrXII	466127	466264	137	N12:466190
chrXII	466127	466277	150	N12:466190
chrXII	466142	466303	161	N12:466190
chrXII	466158	466264	106	N12:466190
chrXII	466159	466264	105	N12:466190
chrXII	466159	466342	183	N12:466190
chrXII	466159	466342	183	N12:466349
chrXII	466184	466344	160	N12:466190
chrXII	466184	466344	160	N12:466349
chrXII	466207	466337	130	N12:466190
chrXII	466207	466337	130	N12:466349
chrXII	466220	466354	134	N12:466349
chrXII	466252	466385	133	N12:466349
chrXII	466277	466442	165	N12:466349
chrXII	466277	466449	172	N12:466349
chrXII	466280	466385	105	N12:466349
chrXII	466280	466416	136	N12:466349
chrXII	466280	466453	173	N12:466349
chrXII	466289	466442	153	N12:466349
chrXII	466289	466449	160	N12:466349
chrXII	466290	466385	95	N12:466349
chrXII	466290	466416	126	N12:466349
chrXII	466290	466449	159	N12:466349
chrXII	466290	466453	163	N12:466349
chrXII	466290	466454	164	N12:466349
chrXII	466291	466449	158	N12:466349
chrXII	466291	466453	162	N12:466349
chrXII	466324	466453	129	N12:466349
chrXII	466324	466454	130	N12:466349
chrXII	466335	466483	148	N12:466349
chrXII	466349	466502	153	N12:466349
chrXII	466351	466500	149	N12:466349
chrXII	466356	466449	93	N12:466349
chrXII	466356	466483	127	N12:466349
chrXII	466356	466500	144	N12:466349
chrXII	466356	466502	146	N12:466349

Chr	Start	End	Length (bp)	Nucleosome_ID
chrXII	466364	466500	136	N12:466349
chrXII	466367	466532	165	N12:466349
chrXII	466367	466538	171	N12:466349
chrXII	466385	466532	147	
chrXII	466385	466538	153	
chrXII	466392	466502	110	
chrXII	466392	466544	152	
chrXII	466403	466500	97	
chrXII	466403	466502	99	
chrXII	466403	466544	141	
chrXII	466403	466559	156	
chrXII	466432	466521	89	
chrXII	466432	466523	91	
chrXII	466432	466527	95	
chrXII	466432	466528	96	
chrXII	466432	466530	98	
chrXII	466432	466531	99	
chrXII	466432	466532	100	
chrXII	466432	466534	102	
chrXII	466432	466538	106	
chrXII	466432	466539	107	
chrXII	466474	466613	139	N12:466630
chrXII	466474	466618	144	N12:466630
chrXII	466497	466613	116	N12:466630
chrXII	466497	466618	121	N12:466630
chrXII	466497	466667	170	N12:466630
chrXII	466507	466613	106	N12:466630
chrXII	466509	466613	104	N12:466630
chrXII	466509	466618	109	N12:466630
chrXII	466557	466700	143	N12:466630
chrXII	466557	466704	147	N12:466630
chrXII	466557	466708	151	N12:466630
chrXII	466557	466709	152	N12:466630
chrXII	466574	466729	155	N12:466630
chrXII	466577	466730	153	N12:466630
chrXII	466611	466736	125	N12:466630
chrXII	466690	466843	153	N12:466825
chrXII	466706	466873	167	N12:466825
chrXII	466706	466878	172	N12:466825
chrXII	466737	466873	136	N12:466825
chrXII	466737	466876	139	N12:466825
chrXII	466737	466878	141	N12:466825
chrXII	466743	466900	157	N12:466825
chrXII	466757	466910	153	N12:466825
chrXII	466757	466924	167	N12:466825
chrXII	466771	466894	123	N12:466825
chrXII	466804	466956	152	N12:466825
chrXII	466940	467096	156	N12:467036
chrXII	466947	467098	151	N12:467036
chrXII	466953	467111	158	N12:467036
chrXII	466957	467090	133	N12:467036
chrXII	467009	467160	151	N12:467036
chrXII	467063	467189	126	
chrXII	467082	467240	158	
chrXII	467082	467241	159	
chrXII	467086	467196	110	
chrXII	467086	467238	152	
chrXII	467087	467238	151	
chrXII	467087	467240	153	
chrXII	467096	467196	100	
chrXII	467096	467247	151	
chrXII	467113	467196	83	
chrXII	467113	467256	143	
chrXII	467120	467226	106	
chrXII	467120	467240	120	
chrXII	467120	467274	154	
chrXII	467123	467254	131	
chrXII	467162	467304	142	N12:467327
chrXII	467192	467274	82	
chrXII	467192	467343	151	N12:467327
chrXII	467216	467364	148	N12:467327
chrXII	467224	467368	144	N12:467327
chrXII	467264	467414	150	N12:467327
chrXII	467267	467430	163	N12:467327
chrXII	467271	467409	138	N12:467327
chrXII	467271	467414	143	N12:467327
chrXII	467273	467432	159	N12:467327
chrXII	467280	467428	148	N12:467327
chrXII	467303	467458	155	N12:467327
chrXII	467303	467459	156	N12:467327
chrXII	467304	467459	155	N12:467327
chrXII	467314	467414	100	N12:467327
chrXII	467314	467459	145	N12:467327
chrXII	467314	467464	150	N12:467327
chrXII	467314	467487	173	N12:467327
chrXII	467316	467464	148	N12:467327
chrXII	467328	467458	130	N12:467327
chrXII	467328	467489	161	N12:467327
chrXII	467332	467459	127	N12:467327
chrXII	467332	467487	155	N12:467327
chrXII	467341	467487	146	N12:467327
chrXII	467341	467517	176	N12:467327
chrXII	467428	467588	160	N12:467582
chrXII	467434	467588	154	N12:467582
chrXII	467435	467598	163	N12:467582
chrXII	467456	467609	153	N12:467582
chrXII	467475	467629	154	N12:467582
chrXII	467493	467630	137	N12:467582

Chr	Start	End	Length (bp)	Nucleosome_ID
chrXII	467493	467645	152	N12:467582
chrXII	467496	467660	164	N12:467582
chrXII	467502	467630	128	N12:467582
chrXII	467510	467630	120	N12:467582
chrXII	467510	467645	135	N12:467582
chrXII	467510	467667	157	N12:467582
chrXII	467511	467669	158	N12:467582
chrXII	467515	467649	134	N12:467582
chrXII	467532	467638	106	N12:467582
chrXII	467556	467724	168	N12:467582
chrXII	467645	467795	150	
chrXII	467645	467796	151	
chrXII	467883	468037	154	N12:467959
chrXII	467883	468038	155	N12:467959
chrXII	467883	468049	166	N12:467959
chrXII	467887	468049	162	N12:467959
chrXII	467905	468037	132	N12:467959
chrXII	467905	468038	133	N12:467959
chrXII	467905	468049	144	N12:467959
chrXII	467906	468037	131	N12:467959
chrXII	467906	468038	132	N12:467959
chrXII	467906	468049	143	N12:467959
chrXII	467906	468050	144	N12:467959
chrXII	467907	468037	130	N12:467959
chrXII	467907	468038	131	N12:467959
chrXII	467907	468049	142	N12:467959
chrXII	467983	468139	156	N12:468113
chrXII	468039	468185	146	N12:468113
chrXII	468045	468181	136	N12:468113
chrXII	468045	468185	140	N12:468113
chrXII	468047	468203	156	N12:468113
chrXII	468050	468139	89	N12:468113
chrXII	468050	468175	125	N12:468113
chrXII	468050	468176	126	N12:468113
chrXII	468050	468180	130	N12:468113
chrXII	468050	468181	131	N12:468113
chrXII	468050	468184	134	N12:468113
chrXII	468050	468185	135	N12:468113
chrXII	468050	468203	153	N12:468113
chrXII	468070	468181	111	N12:468113
chrXII	468070	468184	114	N12:468113
chrXII	468070	468203	133	N12:468113
chrXII	468187	468283	96	N12:468275
chrXII	468226	468399	173	N12:468275
chrXII	468226	468402	176	N12:468275
chrXII	468232	468399	167	N12:468275
chrXII	468232	468402	170	N12:468275
chrXII	468235	468370	135	N12:468275
chrXII	468235	468399	164	N12:468275
chrXII	468303	468465	162	N12:468484
chrXII	468322	468465	143	N12:468484
chrXII	468341	468490	149	N12:468484
chrXII	468341	468513	172	N12:468484
chrXII	468341	468556	215	N12:468484
chrXII	468364	468513	149	N12:468484
chrXII	468364	468554	190	N12:468484
chrXII	468364	468556	192	N12:468484
chrXII	468364	468569	205	N12:468484
chrXII	468372	468532	160	N12:468484
chrXII	468373	468530	157	N12:468484
chrXII	468373	468554	181	N12:468484
chrXII	468373	468556	183	N12:468484
chrXII	468401	468490	89	N12:468484
chrXII	468401	468513	112	N12:468484
chrXII	468401	468532	131	N12:468484
chrXII	468401	468554	153	N12:468484
chrXII	468401	468556	155	N12:468484
chrXII	468401	468569	168	N12:468484
chrXII	468401	468578	177	N12:468484
chrXII	468402	468513	111	N12:468484
chrXII	468402	468530	128	N12:468484
chrXII	468402	468550	148	N12:468484
chrXII	468402	468553	151	N12:468484
chrXII	468402	468554	152	N12:468484
chrXII	468402	468556	154	N12:468484
chrXII	468402	468559	157	N12:468484
chrXII	468402	468569	167	N12:468484
chrXII	468402	468578	176	N12:468484
chrXII	468402	468604	202	N12:468484
chrXII	468402	468606	204	N12:468484
chrXII	468404	468530	126	N12:468484
chrXII	468404	468554	150	N12:468484
chrXII	468404	468556	152	N12:468484
chrXII	468404	468559	155	N12:468484
chrXII	468404	468569	165	N12:468484
chrXII	468404	468578	174	N12:468484
chrXII	468404	468606	202	N12:468484
chrXII	468405	468513	108	N12:468484
chrXII	468405	468530	125	N12:468484
chrXII	468405	468532	127	N12:468484
chrXII	468405	468550	145	N12:468484
chrXII	468405	468553	148	N12:468484
chrXII	468405	468554	149	N12:468484
chrXII	468405	468556	151	N12:468484
chrXII	468405	468559	154	N12:468484
chrXII	468405	468568	163	N12:468484
chrXII	468405	468569	164	N12:468484
chrXII	468405	468578	173	N12:468484

Chr	Start	End	Length (bp)	Nucleosome_ID
chrXII	468405	468604	199	N12:468484
chrXII	468405	468606	201	N12:468484
chrXII	468406	468513	107	N12:468484
chrXII	468406	468530	124	N12:468484
chrXII	468406	468550	144	N12:468484
chrXII	468406	468554	148	N12:468484
chrXII	468406	468556	150	N12:468484
chrXII	468406	468559	153	N12:468484
chrXII	468406	468568	162	N12:468484
chrXII	468406	468604	198	N12:468484
chrXII	468406	468606	200	N12:468484
chrXII	468445	468551	106	N12:468484
chrXII	468445	468554	109	N12:468484
chrXII	468445	468556	111	N12:468484
chrXII	468445	468604	159	N12:468484
chrXII	468445	468606	161	N12:468484
chrXII	468462	468603	141	N12:468484
chrXII	468463	468554	91	N12:468484
chrXII	468463	468556	93	N12:468484
chrXII	468463	468604	141	N12:468484
chrXII	468463	468606	143	N12:468484
chrXII	468545	468696	151	N12:468638
chrXII	468566	468665	99	N12:468638
chrXII	468566	468695	129	N12:468638
chrXII	468566	468710	144	N12:468638
chrXII	468566	468716	150	N12:468638
chrXII	468567	468695	128	N12:468638
chrXII	468567	468710	143	N12:468638
chrXII	468567	468716	149	N12:468638
chrXII	468656	468810	154	N12:468638
chrXII	468658	468810	152	N12:468638
chrXII	468703	468859	156	N12:468850
chrXII	468739	468893	154	N12:468850
chrXII	468739	468896	157	N12:468850
chrXII	468741	468859	118	N12:468850
chrXII	468741	468893	152	N12:468850
chrXII	468741	468896	155	N12:468850
chrXII	468741	468911	170	N12:468850
chrXII	468755	468893	138	N12:468850
chrXII	468755	468911	156	N12:468850
chrXII	468758	468893	135	N12:468850
chrXII	468762	468893	131	N12:468850
chrXII	468762	468911	149	N12:468850
chrXII	468765	468893	128	N12:468850
chrXII	468765	468911	146	N12:468850
chrXII	468770	468893	123	N12:468850
chrXII	468770	468911	141	N12:468850
chrXII	472060	472213	153	N12:472134
chrXII	472461	472545	84	N12:472515
chrXII	472461	472563	102	N12:472515
chrXII	475711	475864	153	N12:475777
chrXII	476909	477023	114	
chrXII	477605	477755	150	N12:477669
chrXII	478538	478667	129	N12:478520
chrXII	479001	479158	157	N12:479064
chrXII	479187	479337	150	N12:479289
chrXII	479469	479630	161	N12:479454
chrXII	480195	480349	154	N12:480208
chrXII	480476	480621	145	N12:480542
chrXII	480480	480631	151	N12:480542
chrXII	480687	480827	140	N12:480768
chrXII	480693	480827	134	N12:480768
chrXII	481105	481260	155	N12:481138
chrXII	481236	481344	108	
chrXII	481385	481534	149	N12:481399
chrXII	482041	482125	84	N12:482076
chrXII	482041	482143	102	N12:482076
chrXII	482885	483037	152	N12:482962
chrXII	485292	485445	153	N12:485368
chrXII	485693	485777	84	N12:485732
chrXII	488944	489097	153	N12:489014
chrXII	489345	489429	84	N12:489399
chrXII	489345	489447	102	N12:489399
chrXII	489345	489466	121	N12:489399
chrXII	489345	489469	124	N12:489399
chrXII	489345	489471	126	N12:489399
chrXII	489359	489469	110	N12:489399
chrXII	489359	489471	112	N12:489399
chrXII	489361	489466	105	N12:489399
chrXII	489556	489702	146	N12:489601
chrXII	489926	490077	151	N12:489932
chrXII	489933	490080	147	N12:489932
chrXII	490009	490164	155	N12:490150
chrXII	490027	490183	156	N12:490150
chrXII	490049	490195	146	N12:490150
chrXII	490049	490215	166	N12:490150
chrXII	490049	490219	170	N12:490150
chrXII	490072	490215	143	N12:490150
chrXII	490072	490219	147	N12:490150
chrXII	490083	490239	156	N12:490150
chrXII	490158	490312	154	N12:490324
chrXII	490158	490312	154	N12:490150
chrXII	490228	490334	106	N12:490324
chrXII	490228	490337	109	N12:490324
chrXII	490228	490382	154	N12:490324
chrXII	490261	490368	107	N12:490324
chrXII	490262	490423	161	N12:490324
chrXII	490263	490423	160	N12:490324

Chr	Start	End	Length (bp)	Nucleosome_ID
chrXII	490267	490423	156	N12:490324
chrXII	490291	490423	132	N12:490324
chrXII	490302	490409	107	N12:490324
chrXII	490302	490423	121	N12:490324
chrXII	494931	495079	148	N12:495031
chrXII	496357	496507	150	
chrXII	508585	508725	140	N12:508668
chrXII	514529	514679	150	N12:514599
chrXII	518369	518501	132	N12:518444
chrXII	522434	522581	147	N12:522432
chrXII	528715	528875	160	N12:528770
chrXII	529249	529419	170	N12:529380
chrXII	537872	537976	104	N12:537943
chrXII	542120	542273	153	N12:542188
chrXII	544133	544261	128	N12:544269
chrXII	545204	545351	147	N12:545241
chrXII	546037	546189	152	N12:546112
chrXII	546038	546189	151	N12:546112
chrXII	546040	546189	149	N12:546112
chrXII	547890	548039	149	N12:547946
chrXII	553542	553689	147	N12:553631
chrXII	555694	555847	153	N12:555721
chrXII	559139	559286	147	N12:559216
chrXII	559606	559755	149	N12:559682
chrXII	560049	560194	145	N12:560179
chrXII	570252	570403	151	N12:570424
chrXII	573533	573669	136	N12:573595
chrXII	576803	576966	163	N12:576902
chrXII	586445	586571	126	N12:586538
chrXII	587477	587583	106	N12:587489
chrXII	588088	588237	149	N12:588110
chrXII	593332	593484	152	N12:593448
chrXII	593504	593653	149	N12:593653
chrXII	593678	593801	123	
chrXII	593778	593933	155	N12:593897
chrXII	594211	594345	134	N12:594281
chrXII	594211	594351	140	N12:594281
chrXII	594407	594558	151	N12:594490
chrXII	594417	594562	145	N12:594490
chrXII	594435	594586	151	N12:594490
chrXII	594689	594843	154	N12:594825
chrXII	594723	594867	144	N12:594825
chrXII	595254	595389	135	N12:595293
chrXII	595436	595568	132	N12:595587
chrXII	595525	595663	138	N12:595587
chrXII	598015	598129	114	N12:598127
chrXII	598489	598636	147	N12:598658
chrXII	603237	603400	163	N12:603419
chrXII	607039	607198	159	N12:607110
chrXII	608189	608339	150	N12:608276
chrXII	610208	610357	149	N12:610297
chrXII	611103	611232	129	N12:611216
chrXII	614828	614977	149	N12:614901
chrXII	614828	614997	169	N12:614901
chrXII	614830	614977	147	N12:614901
chrXII	625233	625381	148	N12:625336
chrXII	628912	629085	173	N12:629078
chrXII	631988	632127	139	N12:632062
chrXII	635940	636108	168	N12:635980
chrXII	640543	640689	146	N12:640632
chrXII	649877	650024	147	N12:649854
chrXII	649877	650024	147	N12:650003
chrXII	651183	651332	149	N12:651337
chrXII	651357	651480	123	N12:651337
chrXII	651457	651612	155	N12:651585
chrXII	651890	652024	134	N12:651960
chrXII	651890	652030	140	N12:651960
chrXII	652086	652237	151	N12:652161
chrXII	652096	652241	145	N12:652161
chrXII	652114	652265	151	N12:652161
chrXII	652116	652265	149	N12:652161
chrXII	652368	652522	154	N12:652504
chrXII	652618	652786	168	N12:652671
chrXII	652933	653068	135	N12:652970
chrXII	653087	653248	161	N12:653246
chrXII	653194	653339	145	N12:653246
chrXII	653561	653718	157	N12:653642
chrXII	654052	654181	129	
chrXII	654964	655114	150	N12:655066
chrXII	656400	656549	149	N12:656524
chrXII	657729	657887	158	N12:657828
chrXII	664370	664478	108	N12:664374
chrXII	664370	664482	112	N12:664374
chrXII	665850	666010	160	N12:665933
chrXII	675650	675803	153	N12:675721
chrXII	678419	678585	166	N12:678473
chrXII	684089	684224	135	N12:684178
chrXII	686656	686810	154	N12:686728
chrXII	686971	687118	147	N12:687063
chrXII	691050	691199	149	N12:691100
chrXII	695440	695604	164	N12:695602
chrXII	698814	698965	151	N12:698805
chrXII	700732	700860	128	N12:700798
chrXII	700732	700861	129	N12:700798
chrXII	704633	704781	148	N12:704801
chrXII	704633	704781	148	N12:704613
chrXII	704633	704785	152	N12:704801
chrXII	704633	704785	152	N12:704613

Chr	Start	End	Length (bp)	Nucleosome_ID
chrXII	704637	704785	148	N12:704801
chrXII	704738	704878	140	N12:704801
chrXII	707321	707471	150	N12:707390
chrXII	707855	708017	162	N12:707899
chrXII	710806	710956	150	N12:710883
chrXII	713802	713939	137	N12:713872
chrXII	714204	714311	107	N12:714214
chrXII	714204	714312	108	N12:714214
chrXII	714794	714945	151	N12:714880
chrXII	721307	721447	140	N12:721455
chrXII	723297	723432	135	N12:723451
chrXII	726306	726432	126	N12:726432
chrXII	727018	727148	130	N12:727116
chrXII	731448	731612	164	N12:731474
chrXII	731448	731612	164	N12:731633
chrXII	733723	733874	151	N12:733718
chrXII	733723	733874	151	N12:733875
chrXII	735532	735635	103	N12:735568
chrXII	736027	736189	162	N12:736034
chrXII	738785	738957	172	N12:738797
chrXII	740597	740703	106	N12:740592
chrXII	745846	745998	152	N12:745926
chrXII	751027	751192	165	N12:751096
chrXII	752922	753058	136	N12:753013
chrXII	753617	753790	173	N12:753609
chrXII	753617	753790	173	N12:753760
chrXII	759427	759579	152	N12:759504
chrXII	761633	761798	165	
chrXII	762105	762269	164	N12:762185
chrXII	770786	770938	152	N12:770870
chrXII	779526	779669	143	N12:779529
chrXII	779740	779903	163	N12:779869
chrXII	781130	781282	152	N12:781211
chrXII	782565	782713	148	N12:782593
chrXII	783914	784060	146	N12:784073
chrXII	783914	784060	146	N12:783921
chrXII	785535	785685	150	N12:785589
chrXII	786502	786666	164	N12:786585
chrXII	792530	792669	139	N12:792622
chrXII	797667	797817	150	N12:797755
chrXII	799286	799439	153	N12:799357
chrXII	803374	803523	149	N12:803394
chrXII	804062	804197	135	N12:804104
chrXII	804515	804661	146	N12:804614
chrXII	806849	807003	154	N12:806937
chrXII	807887	808014	127	N12:807864
chrXII	807887	808014	127	N12:808034
chrXII	827684	827838	154	N12:827706
chrXII	830101	830255	154	N12:830181
chrXII	830655	830774	119	N12:830722
chrXII	830666	830794	128	N12:830722
chrXII	831008	831187	179	N12:831140
chrXII	831542	831700	158	N12:831639
chrXII	839642	839772	130	N12:839733
chrXII	840366	840517	151	
chrXII	841812	841967	155	N12:841835
chrXII	842219	842368	149	N12:842300
chrXII	846202	846357	155	N12:846233
chrXII	864284	864432	148	N12:864280
chrXII	868597	868734	137	N12:868681
chrXII	871320	871444	124	N12:871354
chrXII	871320	871449	129	N12:871354
chrXII	871322	871449	127	N12:871354
chrXII	874679	874837	158	N12:874753
chrXII	878567	878705	138	N12:878691
chrXII	884555	884713	158	N12:884629
chrXII	889517	889681	164	N12:889511
chrXII	893166	893327	161	N12:893245
chrXII	896277	896446	169	N12:896387
chrXII	896668	896822	154	N12:896735
chrXII	896811	896958	147	N12:896895
chrXII	903630	903761	131	N12:903719
chrXII	903784	903945	161	N12:903890
chrXII	903961	904117	156	N12:904050
chrXII	905180	905335	155	
chrXII	908649	908820	171	N12:908748
chrXII	912499	912653	154	N12:912578
chrXII	913527	913695	168	N12:913636
chrXII	922406	922556	150	N12:922523
chrXII	922509	922654	145	N12:922523
chrXII	924375	924524	149	N12:924433
chrXII	926149	926303	154	N12:926312
chrXII	926358	926515	157	N12:926512
chrXII	929019	929172	153	N12:929097
chrXII	931573	931698	125	
chrXII	937960	938108	148	N12:938021
chrXII	938740	938880	140	N12:938790
chrXII	939726	939894	168	N12:939769
chrXII	940022	940161	139	N12:940088
chrXII	941909	942015	106	N12:941949
chrXII	941909	942019	110	N12:941949
chrXII	943265	943394	129	N12:943276
chrXII	944730	944873	143	N12:944794
chrXII	946105	946219	114	N12:946210
chrXII	946809	946958	149	N12:946920
chrXII	947096	947245	149	N12:947106
chrXII	947588	947739	151	N12:947709
chrXII	952012	952159	147	N12:952105

Chr	Start	End	Length (bp)	Nucleosome_ID
chrXII	955881	956039	158	N12:955940
chrXII	956586	956713	127	N12:956629
chrXII	962105	962271	166	N12:962110
chrXII	962105	962271	166	N12:962293
chrXII	966042	966188	146	N12:966156
chrXII	967180	967328	148	N12:967232
chrXII	967209	967341	132	N12:967232
chrXII	974145	974297	152	N12:974223
chrXII	975018	975184	166	N12:975031
chrXII	976449	976598	149	N12:976465
chrXII	978018	978161	143	N12:978103
chrXII	978601	978756	155	N12:978687
chrXII	979540	979688	148	
chrXII	981009	981111	102	
chrXII	981009	981139	130	
chrXII	981073	981227	154	N12:981187
chrXII	981080	981248	168	N12:981187
chrXII	982426	982590	164	N12:982511
chrXII	987388	987536	148	N12:987440
chrXII	988804	988961	157	N12:988850
chrXII	988850	988961	111	N12:988850
chrXII	988957	989104	147	N12:989040
chrXII	990093	990242	149	N12:990189
chrXII	990292	990442	150	N12:990360
chrXII	997620	997777	157	N12:997691
chrXII	998925	999073	148	N12:999015
chrXII	1001567	1001716	149	N12:1001641
chrXII	1005509	1005668	159	N12:1005574
chrXII	1005994	1006142	148	N12:1006065
chrXII	1012419	1012577	158	N12:1012484
chrXII	1012549	1012696	147	N12:1012647
chrXII	1012550	1012632	82	N12:1012647
chrXII	1012550	1012633	83	N12:1012647
chrXII	1012550	1012634	84	N12:1012647
chrXII	1012550	1012635	85	N12:1012647
chrXII	1012550	1012636	86	N12:1012647
chrXII	1012550	1012637	87	N12:1012647
chrXII	1012550	1012638	88	N12:1012647
chrXII	1012550	1012639	89	N12:1012647
chrXII	1012550	1012640	90	N12:1012647
chrXII	1012550	1012641	91	N12:1012647
chrXII	1012550	1012642	92	N12:1012647
chrXII	1012550	1012643	93	N12:1012647
chrXII	1012550	1012644	94	N12:1012647
chrXII	1012550	1012645	95	N12:1012647
chrXII	1012550	1012646	96	N12:1012647
chrXII	1012550	1012648	98	N12:1012647
chrXII	1012550	1012649	99	N12:1012647
chrXII	1012550	1012650	100	N12:1012647
chrXII	1012550	1012651	101	N12:1012647
chrXII	1012550	1012652	102	N12:1012647
chrXII	1012550	1012653	103	N12:1012647
chrXII	1012550	1012654	104	N12:1012647
chrXII	1012550	1012655	105	N12:1012647
chrXII	1012550	1012656	106	N12:1012647
chrXII	1012550	1012657	107	N12:1012647
chrXII	1012550	1012659	109	N12:1012647
chrXII	1012550	1012660	110	N12:1012647
chrXII	1012550	1012661	111	N12:1012647
chrXII	1012550	1012662	112	N12:1012647
chrXII	1012550	1012663	113	N12:1012647
chrXII	1012550	1012664	114	N12:1012647
chrXII	1012550	1012665	115	N12:1012647
chrXII	1012550	1012666	116	N12:1012647
chrXII	1012550	1012667	117	N12:1012647
chrXII	1012550	1012668	118	N12:1012647
chrXII	1012550	1012669	119	N12:1012647
chrXII	1012550	1012670	120	N12:1012647
chrXII	1012550	1012671	121	N12:1012647
chrXII	1012550	1012672	122	N12:1012647
chrXII	1012550	1012673	123	N12:1012647
chrXII	1012550	1012674	124	N12:1012647
chrXII	1012550	1012675	125	N12:1012647
chrXII	1012550	1012676	126	N12:1012647
chrXII	1012550	1012677	127	N12:1012647
chrXII	1012550	1012678	128	N12:1012647
chrXII	1012550	1012679	129	N12:1012647
chrXII	1012550	1012680	130	N12:1012647
chrXII	1012550	1012681	131	N12:1012647
chrXII	1012550	1012682	132	N12:1012647
chrXII	1012550	1012683	133	N12:1012647
chrXII	1012550	1012684	134	N12:1012647
chrXII	1012550	1012685	135	N12:1012647
chrXII	1012550	1012686	136	N12:1012647
chrXII	1012550	1012687	137	N12:1012647
chrXII	1012550	1012688	138	N12:1012647
chrXII	1012550	1012689	139	N12:1012647
chrXII	1012550	1012690	140	N12:1012647
chrXII	1012550	1012691	141	N12:1012647
chrXII	1012550	1012692	142	N12:1012647
chrXII	1012550	1012693	143	N12:1012647
chrXII	1012550	1012694	144	N12:1012647
chrXII	1012550	1012695	145	N12:1012647
chrXII	1012550	1012696	146	N12:1012647
chrXII	1012550	1012698	148	N12:1012647
chrXII	1012551	1012693	142	N12:1012647
chrXII	1012551	1012696	145	N12:1012647
chrXII	1012552	1012696	144	N12:1012647

Chr	Start	End	Length (bp)	Nucleosome_ID
chrXII	1012553	1012693	140	N12:1012647
chrXII	1012553	1012696	143	N12:1012647
chrXII	1012554	1012696	142	N12:1012647
chrXII	1012555	1012696	141	N12:1012647
chrXII	1012556	1012696	140	N12:1012647
chrXII	1012557	1012696	139	N12:1012647
chrXII	1012558	1012696	138	N12:1012647
chrXII	1012559	1012696	137	N12:1012647
chrXII	1012560	1012696	136	N12:1012647
chrXII	1012561	1012696	135	N12:1012647
chrXII	1012562	1012696	134	N12:1012647
chrXII	1012563	1012696	133	N12:1012647
chrXII	1012564	1012696	132	N12:1012647
chrXII	1012565	1012696	131	N12:1012647
chrXII	1012566	1012696	130	N12:1012647
chrXII	1012567	1012696	129	N12:1012647
chrXII	1012568	1012696	128	N12:1012647
chrXII	1012569	1012696	127	N12:1012647
chrXII	1012570	1012696	126	N12:1012647
chrXII	1012571	1012696	125	N12:1012647
chrXII	1012572	1012696	124	N12:1012647
chrXII	1012573	1012696	123	N12:1012647
chrXII	1012574	1012696	122	N12:1012647
chrXII	1012575	1012696	121	N12:1012647
chrXII	1012576	1012696	120	N12:1012647
chrXII	1012578	1012696	118	N12:1012647
chrXII	1012579	1012696	117	N12:1012647
chrXII	1012580	1012696	116	N12:1012647
chrXII	1012581	1012696	115	N12:1012647
chrXII	1012583	1012696	113	N12:1012647
chrXII	1012584	1012696	112	N12:1012647
chrXII	1012586	1012696	110	N12:1012647
chrXII	1012590	1012674	84	N12:1012647
chrXII	1012610	1012696	86	N12:1012647
chrXII	1013868	1014024	156	N12:1013902
chrXII	1017667	1017816	149	N12:1017757
chrXII	1023621	1023767	146	N12:1023663
chrXII	1024132	1024289	157	N12:1024222
chrXII	1031294	1031445	151	N12:1031333
chrXII	1032438	1032586	148	N12:1032481
chrXII	1050400	1050528	128	N12:1050503
chrXII	1050732	1050875	143	N12:1050868
chrXII	1059679	1059820	141	N12:1059812
chrXII	1065029	1065154	125	N12:1065098
chrXII	1065029	1065156	127	N12:1065098
chrXII	1066703	1066854	151	
chrXII	1067646	1067808	162	N12:1067769
chrXII	1067747	1067902	155	N12:1067769
chrXII	1067881	1068018	137	N12:1067950
chrXII	1068176	1068319	143	N12:1068230
chrXII	1068224	1068349	125	N12:1068230
chrXII	1068449	1068601	152	
chrXII	1068522	1068666	144	
chrXII	1068619	1068754	135	N12:1068716
chrXII	1068619	1068806	187	N12:1068716
chrXII	1068662	1068754	92	N12:1068716
chrXII	1068662	1068806	144	N12:1068716
chrXII	1069001	1069153	152	N12:1069071
chrXII	1069302	1069432	130	N12:1069283
chrXII	1069440	1069601	161	
chrXII	1070837	1070988	151	N12:1070915
chrXII	1070837	1071008	171	N12:1070915
chrXII	1072226	1072381	155	N12:1072335
chrXII	1073391	1073538	147	
chrXII	1073547	1073677	130	N12:1073613
chrXII	1073928	1074064	136	N12:1073964
chrXII	1074309	1074471	162	N12:1074439
chrXII	1074410	1074565	155	N12:1074439
chrXII	1074544	1074681	137	N12:1074612
chrXII	1074831	1074982	151	N12:1074890
chrXII	1074839	1074982	143	N12:1074890
chrXII	1074887	1075012	125	N12:1074890
chrXII	1075112	1075264	152	
chrXII	1075185	1075329	144	
chrXII	1075282	1075417	135	N12:1075369
chrXII	1075282	1075469	187	N12:1075369
chrXII	1075325	1075417	92	N12:1075369
chrXII	1075325	1075469	144	N12:1075369
chrXII	1075664	1075816	152	N12:1075734
chrXII	1075965	1076095	130	N12:1076061
chrXII	1076103	1076264	161	
chrXII	1077160	1077312	152	N12:1077229
chrXII	1077500	1077651	151	N12:1077576
chrXIII	687	858	171	N13:774
chrXIII	707	858	151	N13:774
chrXIII	1046	1198	152	N13:1107
chrXIII	1151	1299	148	
chrXIII	1836	2023	187	N13:1892
chrXIII	1872	2023	151	N13:1892
chrXIII	1884	2018	134	N13:1892
chrXIII	1909	2005	96	N13:1892
chrXIII	2542	2694	152	N13:2630
chrXIII	2889	3033	144	N13:2945
chrXIII	2889	3076	187	N13:2945
chrXIII	2941	3033	92	N13:2945
chrXIII	2941	3076	135	N13:2945
chrXIII	3029	3173	144	N13:3125
chrXIII	3094	3246	152	N13:3125

Chr	Start	End	Length (bp)	Nucleosome_ID
chrXIII	3346	3471	125	N13:3450
chrXIII	3376	3519	143	N13:3450
chrXIII	3376	3527	151	N13:3450
chrXIII	5624	5767	143	N13:5763
chrXIII	9070	9207	137	N13:9142
chrXIII	13123	13267	144	N13:13226
chrXIII	14333	14505	172	N13:14398
chrXIII	16025	16179	154	N13:16099
chrXIII	18573	18744	171	N13:18675
chrXIII	19858	19999	141	N13:19950
chrXIII	20186	20333	147	N13:20274
chrXIII	21593	21740	147	N13:21676
chrXIII	23095	23242	147	N13:23173
chrXIII	32413	32558	145	N13:32440
chrXIII	33232	33384	152	N13:33387
chrXIII	38992	39143	151	N13:39050
chrXIII	40472	40624	152	N13:40521
chrXIII	42972	43133	161	N13:43054
chrXIII	47095	47254	159	N13:47157
chrXIII	54675	54757	82	
chrXIII	54913	55020	107	N13:54961
chrXIII	54913	55032	119	N13:54961
chrXIII	59231	59396	165	N13:59272
chrXIII	63883	64021	138	N13:63895
chrXIII	64235	64393	158	N13:64363
chrXIII	64871	65018	147	N13:64980
chrXIII	66681	66829	148	N13:66758
chrXIII	75788	75939	151	N13:75859
chrXIII	76732	76885	153	N13:76800
chrXIII	77591	77728	137	N13:77626
chrXIII	83978	84137	159	
chrXIII	93745	93871	126	N13:93877
chrXIII	98827	98976	149	N13:98894
chrXIII	102493	102629	136	N13:102572
chrXIII	104088	104231	143	N13:104162
chrXIII	104601	104708	107	N13:104679
chrXIII	104601	104720	119	N13:104679
chrXIII	107860	108019	159	N13:107958
chrXIII	109020	109176	156	N13:109082
chrXIII	112712	112852	140	N13:112775
chrXIII	113210	113365	155	N13:113351
chrXIII	113210	113365	155	N13:113196
chrXIII	116680	116830	150	N13:116697
chrXIII	116680	116833	153	N13:116697
chrXIII	116680	116835	155	N13:116697
chrXIII	116684	116835	151	N13:116697
chrXIII	116783	116928	145	
chrXIII	117349	117491	142	N13:117410
chrXIII	117514	117618	104	N13:117572
chrXIII	119216	119393	177	N13:119273
chrXIII	120074	120243	169	N13:120213
chrXIII	120074	120243	169	N13:120063
chrXIII	123583	123746	163	N13:123684
chrXIII	124934	125083	149	N13:125011
chrXIII	125456	125612	156	N13:125539
chrXIII	126339	126468	129	
chrXIII	129825	129930	105	N13:129823
chrXIII	129825	129932	107	N13:129823
chrXIII	129843	129930	87	N13:129823
chrXIII	129843	130004	161	N13:129987
chrXIII	129843	130004	161	N13:129823
chrXIII	130886	131027	141	N13:130956
chrXIII	131028	131190	162	N13:131120
chrXIII	131602	131755	153	N13:131595
chrXIII	133367	133536	169	N13:133467
chrXIII	134348	134447	99	N13:134415
chrXIII	135575	135725	150	N13:135649
chrXIII	139632	139738	106	N13:139678
chrXIII	139632	139741	109	N13:139678
chrXIII	142450	142583	133	N13:142508
chrXIII	147977	148129	152	
chrXIII	148322	148434	112	
chrXIII	154231	154388	157	
chrXIII	155621	155784	163	
chrXIII	163164	163259	95	
chrXIII	169237	169345	108	N13:169314
chrXIII	170327	170483	156	N13:170398
chrXIII	170574	170680	106	N13:170557
chrXIII	170574	170696	122	N13:170557
chrXIII	170574	170696	122	N13:170707
chrXIII	171072	171220	148	N13:171141
chrXIII	175762	175915	153	N13:175792
chrXIII	179143	179321	178	
chrXIII	180747	180905	158	N13:180820
chrXIII	181711	181851	140	N13:181781
chrXIII	181848	181977	129	
chrXIII	184182	184330	148	N13:184276
chrXIII	184701	184824	123	
chrXIII	184801	184956	155	N13:184876
chrXIII	185234	185368	134	N13:185305
chrXIII	185234	185374	140	N13:185305
chrXIII	186277	186412	135	N13:186311
chrXIII	186724	186874	150	N13:186799
chrXIII	186903	187060	157	N13:186999
chrXIII	187135	187284	149	N13:187179
chrXIII	187394	187523	129	
chrXIII	188306	188456	150	N13:188399
chrXIII	195171	195314	143	N13:195288

Chr	Start	End	Length (bp)	Nucleosome_ID
chrXIII	196534	196661	127	N13:196631
chrXIII	196694	196843	149	N13:196842
chrXIII	196868	196991	123	
chrXIII	196968	197123	155	N13:197081
chrXIII	197401	197535	134	N13:197469
chrXIII	197401	197541	140	N13:197469
chrXIII	197597	197748	151	N13:197630
chrXIII	197607	197752	145	N13:197630
chrXIII	197625	197776	151	N13:197630
chrXIII	197879	198033	154	N13:198018
chrXIII	197913	198057	144	N13:198018
chrXIII	198891	199037	146	N13:198960
chrXIII	198891	199041	150	N13:198960
chrXIII	199070	199227	157	N13:199146
chrXIII	199302	199451	149	N13:199440
chrXIII	199561	199690	129	N13:199711
chrXIII	200473	200623	150	N13:200564
chrXIII	203778	203929	151	N13:203861
chrXIII	204102	204256	154	N13:204195
chrXIII	208554	208676	122	N13:208664
chrXIII	211138	211313	175	N13:211187
chrXIII	218290	218401	111	N13:218320
chrXIII	220837	220983	146	N13:220846
chrXIII	228866	229000	134	N13:228934
chrXIII	230938	231091	153	N13:231007
chrXIII	231257	231408	151	N13:231330
chrXIII	233569	233719	150	N13:233649
chrXIII	240347	240506	159	N13:240446
chrXIII	242945	243091	146	N13:243011
chrXIII	243502	243662	160	N13:243575
chrXIII	243825	243980	155	N13:243892
chrXIII	244965	245130	165	N13:244990
chrXIII	244965	245130	165	N13:245141
chrXIII	245559	245710	151	N13:245618
chrXIII	246032	246179	147	N13:246115
chrXIII	246042	246200	158	N13:246115
chrXIII	246354	246509	155	N13:246437
chrXIII	246617	246737	120	N13:246744
chrXIII	250435	250548	113	N13:250485
chrXIII	250507	250631	124	N13:250485
chrXIII	250507	250662	155	N13:250485
chrXIII	250507	250664	157	N13:250485
chrXIII	250507	250665	158	N13:250485
chrXIII	250507	250666	159	N13:250485
chrXIII	250507	250667	160	N13:250485
chrXIII	250507	250668	161	N13:250485
chrXIII	250507	250669	162	N13:250485
chrXIII	250507	250670	163	N13:250485
chrXIII	250507	250671	164	N13:250485
chrXIII	250507	250673	166	N13:250485
chrXIII	250507	250674	167	N13:250485
chrXIII	250507	250675	168	N13:250485
chrXIII	250507	250676	169	N13:250485
chrXIII	250507	250677	170	N13:250485
chrXIII	250507	250678	171	N13:250485
chrXIII	250507	250718	211	N13:250485
chrXIII	250507	250718	211	N13:250740
chrXIII	250508	250678	170	N13:250485
chrXIII	250509	250678	169	
chrXIII	250510	250678	168	
chrXIII	250511	250678	167	
chrXIII	250514	250678	164	
chrXIII	250515	250678	163	
chrXIII	250566	250718	152	N13:250740
chrXIII	253372	253532	160	N13:253539
chrXIII	256009	256161	152	N13:256158
chrXIII	260961	261102	141	N13:261093
chrXIII	261845	261977	132	N13:261963
chrXIII	262094	262251	157	N13:262151
chrXIII	266226	266377	151	N13:266295
chrXIII	271419	271566	147	N13:271513
chrXIII	273047	273197	150	N13:273120
chrXIII	276628	276785	157	N13:276642
chrXIII	276628	276785	157	N13:276801
chrXIII	277879	278014	135	N13:277912
chrXIII	279195	279369	174	N13:279270
chrXIII	283987	284078	91	N13:284101
chrXIII	283987	284079	92	N13:284101
chrXIII	289854	289961	107	N13:289883
chrXIII	289854	289977	123	N13:289883
chrXIII	303791	303918	127	N13:303847
chrXIII	304476	304622	146	N13:304602
chrXIII	310201	310351	150	N13:310222
chrXIII	313026	313180	154	
chrXIII	324504	324669	165	N13:324559
chrXIII	325807	325958	151	N13:325840
chrXIII	333017	333169	152	N13:333098
chrXIII	335127	335272	145	N13:335202
chrXIII	335472	335613	141	N13:335545
chrXIII	336952	337113	161	N13:337009
chrXIII	340438	340585	147	N13:340517
chrXIII	342150	342302	152	N13:342208
chrXIII	348582	348731	149	N13:348736
chrXIII	351853	352022	169	N13:351943
chrXIII	351863	352007	144	N13:351943
chrXIII	357010	357124	114	
chrXIII	357475	357580	105	N13:357470
chrXIII	358631	358781	150	N13:358713

Chr	Start	End	Length (bp)	Nucleosome_ID
chrXIII	358907	359055	148	N13:358983
chrXIII	360027	360184	157	N13:360111
chrXIII	360404	360549	145	N13:360557
chrXIII	360675	360810	135	N13:360775
chrXIII	362078	362238	160	N13:362147
chrXIII	362592	362706	114	N13:362628
chrXIII	374780	374911	131	N13:374845
chrXIII	375262	375391	129	
chrXIII	375501	375650	149	N13:375616
chrXIII	375725	375882	157	N13:375785
chrXIII	375911	376061	150	N13:376003
chrXIII	376373	376508	135	N13:376424
chrXIII	376763	376917	154	N13:376925
chrXIII	377411	377551	140	N13:377486
chrXIII	377417	377551	134	N13:377486
chrXIII	377829	377984	155	N13:377857
chrXIII	377960	378068	108	
chrXIII	379716	379822	106	N13:379764
chrXIII	379716	379838	122	N13:379764
chrXIII	381135	381283	148	N13:381244
chrXIII	383859	384012	153	N13:383963
chrXIII	384143	384283	140	N13:384129
chrXIII	384143	384283	140	N13:384300
chrXIII	384959	385107	148	
chrXIII	386345	386477	132	N13:386478
chrXIII	386345	386477	132	N13:386323
chrXIII	387854	388024	170	N13:387911
chrXIII	395268	395412	144	N13:395361
chrXIII	400158	400292	134	N13:400231
chrXIII	402458	402607	149	N13:402522
chrXIII	406280	406447	167	N13:406339
chrXIII	408429	408536	107	N13:408500
chrXIII	411105	411252	147	N13:411102
chrXIII	412475	412581	106	N13:412535
chrXIII	412475	412589	114	N13:412535
chrXIII	413837	414003	166	N13:413996
chrXIII	422824	422985	161	N13:422941
chrXIII	431294	431455	161	N13:431429
chrXIII	431358	431515	157	N13:431429
chrXIII	435010	435159	149	N13:435081
chrXIII	436980	437142	162	N13:437057
chrXIII	439283	439457	174	N13:439388
chrXIII	443760	443869	109	N13:443884
chrXIII	445137	445296	159	N13:445171
chrXIII	448884	449018	134	N13:448865
chrXIII	448987	449140	153	N13:449042
chrXIII	452811	452953	142	N13:452872
chrXIII	455777	455925	148	N13:455844
chrXIII	456607	456760	153	N13:456671
chrXIII	458537	458683	146	
chrXIII	460130	460281	151	N13:460189
chrXIII	460228	460362	134	N13:460349
chrXIII	460442	460567	125	N13:460520
chrXIII	460442	460595	153	N13:460520
chrXIII	460442	460598	156	N13:460520
chrXIII	460442	460600	158	N13:460520
chrXIII	461194	461345	151	N13:461301
chrXIII	464378	464520	142	N13:464473
chrXIII	467950	468100	150	
chrXIII	470902	471051	149	N13:470916
chrXIII	473040	473146	106	N13:473098
chrXIII	473040	473162	122	N13:473098
chrXIII	473911	474081	170	N13:474025
chrXIII	484473	484603	130	N13:484551
chrXIII	488956	489063	107	N13:489016
chrXIII	488956	489073	117	N13:489016
chrXIII	491261	491409	148	N13:491313
chrXIII	492970	493101	131	N13:492992
chrXIII	495547	495700	153	N13:495639
chrXIII	496801	496973	172	N13:496955
chrXIII	496801	496973	172	N13:496786
chrXIII	499783	499912	129	N13:499808
chrXIII	503855	504003	148	N13:503924
chrXIII	505412	505581	169	N13:505521
chrXIII	512141	512286	145	N13:512212
chrXIII	514521	514669	148	N13:514629
chrXIII	514553	514703	150	N13:514629
chrXIII	515323	515459	136	N13:515420
chrXIII	520223	520378	155	N13:520207
chrXIII	520223	520378	155	N13:520395
chrXIII	520376	520508	132	N13:520395
chrXIII	521730	521901	171	N13:521801
chrXIII	522712	522817	105	N13:522725
chrXIII	528716	528857	141	N13:528767
chrXIII	533451	533610	159	N13:533576
chrXIII	535081	535246	165	N13:535146
chrXIII	536826	536976	150	N13:536905
chrXIII	537368	537507	139	N13:537514
chrXIII	539637	539760	123	N13:539719
chrXIII	540611	540759	148	N13:540749
chrXIII	541727	541880	153	N13:541835
chrXIII	549153	549302	149	N13:549269
chrXIII	549525	549679	154	N13:549554
chrXIII	549713	549857	144	N13:549747
chrXIII	555791	555929	138	N13:555792
chrXIII	562267	562429	162	N13:562366
chrXIII	562901	563062	161	N13:562920
chrXIII	571585	571738	153	N13:571681

Chr	Start	End	Length (bp)	Nucleosome_ID
chrXIII	571590	571738	148	N13:571681
chrXIII	574736	574904	168	N13:574834
chrXIII	580311	580467	156	N13:580401
chrXIII	580644	580772	128	N13:580704
chrXIII	581583	581719	136	N13:581638
chrXIII	582011	582174	163	N13:582099
chrXIII	582013	582174	161	N13:582099
chrXIII	583110	583268	158	
chrXIII	588471	588586	115	
chrXIII	595003	595166	163	N13:595084
chrXIII	596303	596450	147	N13:596386
chrXIII	598087	598214	127	N13:598137
chrXIII	598887	599027	140	N13:598952
chrXIII	600432	600562	130	N13:600483
chrXIII	600662	600821	159	N13:600688
chrXIII	602872	603031	159	N13:602904
chrXIII	608199	608341	142	N13:608209
chrXIII	614091	614242	151	N13:614214
chrXIII	617621	617773	152	N13:617702
chrXIII	617962	618111	149	N13:618058
chrXIII	622293	622443	150	N13:622398
chrXIII	627780	627908	128	N13:627847
chrXIII	627788	627919	131	N13:627847
chrXIII	634568	634713	145	N13:634666
chrXIII	637620	637765	145	N13:637702
chrXIII	641481	641611	130	N13:641533
chrXIII	642381	642525	144	N13:642458
chrXIII	642389	642541	152	N13:642458
chrXIII	649168	649320	152	N13:649263
chrXIII	652798	652958	160	N13:652939
chrXIII	657660	657789	129	N13:657675
chrXIII	658250	658404	154	N13:658346
chrXIII	658827	658990	163	N13:658863
chrXIII	658970	659105	135	N13:659025
chrXIII	660778	660927	149	N13:660944
chrXIII	662900	663052	152	N13:662956
chrXIII	663803	663949	146	N13:663870
chrXIII	663837	663944	107	N13:663870
chrXIII	663837	663953	116	N13:663870
chrXIII	667634	667789	155	N13:667703
chrXIII	671983	672128	145	N13:672090
chrXIII	675888	676037	149	N13:675911
chrXIII	675888	676037	149	N13:676060
chrXIII	675950	676037	87	N13:676060
chrXIII	675950	676105	155	N13:676060
chrXIII	677204	677344	140	N13:677258
chrXIII	677920	678066	146	N13:677969
chrXIII	678554	678723	169	N13:678626
chrXIII	679422	679570	148	
chrXIII	680831	680955	124	N13:680907
chrXIII	684362	684526	164	N13:684432
chrXIII	686491	686656	165	N13:686622
chrXIII	692898	693052	154	N13:692964
chrXIII	693627	693771	144	N13:693719
chrXIII	693831	693958	127	N13:693886
chrXIII	694160	694314	154	N13:694234
chrXIII	694285	694446	161	
chrXIII	694710	694842	132	N13:694758
chrXIII	711631	711787	156	N13:711674
chrXIII	713556	713705	149	N13:713622
chrXIII	714522	714675	153	N13:714616
chrXIII	715186	715332	146	N13:715254
chrXIII	718847	718995	148	N13:719006
chrXIII	718847	718995	148	N13:718849
chrXIII	719890	720048	158	N13:719976
chrXIII	723055	723205	150	
chrXIII	723972	724126	154	N13:724061
chrXIII	730943	731091	148	N13:731008
chrXIII	737903	738069	166	N13:737964
chrXIII	748521	748674	153	N13:748606
chrXIII	750522	750675	153	N13:750581
chrXIII	757851	758017	166	N13:757933
chrXIII	758839	758989	150	N13:759009
chrXIII	758839	758989	150	N13:758860
chrXIII	759376	759557	181	N13:759498
chrXIII	765908	766066	158	N13:766027
chrXIII	772941	773097	156	N13:773019
chrXIII	773665	773803	138	N13:773793
chrXIII	773665	773807	142	N13:773793
chrXIII	774153	774312	159	N13:774317
chrXIII	774153	774312	159	N13:774158
chrXIII	778663	778795	132	N13:778773
chrXIII	779035	779188	153	N13:779109
chrXIII	780072	780218	146	N13:780137
chrXIII	780801	780937	136	N13:780898
chrXIII	790225	790394	169	N13:790301
chrXIII	790368	790515	147	N13:790486
chrXIII	790640	790805	165	N13:790824
chrXIII	790640	790805	165	N13:790646
chrXIII	791775	791919	144	N13:791864
chrXIII	792265	792408	143	N13:792303
chrXIII	792607	792777	170	N13:792794
chrXIII	792607	792777	170	N13:792641
chrXIII	792935	793098	163	N13:793117
chrXIII	792935	793098	163	N13:792956
chrXIII	793020	793160	140	N13:793117
chrXIII	793713	793841	128	N13:793740
chrXIII	795911	796064	153	N13:795966

Chr	Start	End	Length (bp)	Nucleosome_ID
chrXIII	797737	797897	160	N13:797874
chrXIII	797737	797940	203	N13:797874
chrXIII	797778	797885	107	N13:797874
chrXIII	797778	797897	119	N13:797874
chrXIII	797778	797940	162	N13:797874
chrXIII	799674	799836	162	
chrXIII	800670	800819	149	
chrXIII	801962	802127	165	N13:802061
chrXIII	804104	804264	160	N13:804203
chrXIII	806854	806962	108	N13:806905
chrXIII	806855	806962	107	N13:806905
chrXIII	808568	808720	152	
chrXIII	811849	811978	129	N13:811868
chrXIII	816433	816597	164	N13:816517
chrXIII	819869	819975	106	N13:819933
chrXIII	819869	819988	119	N13:819933
chrXIII	820608	820710	102	N13:820698
chrXIII	820608	820711	103	N13:820698
chrXIII	820608	820715	107	N13:820698
chrXIII	820608	820717	109	N13:820698
chrXIII	820608	820733	125	N13:820698
chrXIII	820608	820737	129	N13:820698
chrXIII	820608	820738	130	N13:820698
chrXIII	820608	820740	132	N13:820698
chrXIII	820608	820741	133	N13:820698
chrXIII	820608	820742	134	N13:820698
chrXIII	820608	820743	135	N13:820698
chrXIII	820608	820744	136	N13:820698
chrXIII	820608	820745	137	N13:820698
chrXIII	820608	820746	138	N13:820698
chrXIII	820608	820747	139	N13:820698
chrXIII	820608	820748	140	N13:820698
chrXIII	820608	820749	141	N13:820698
chrXIII	820608	820750	142	N13:820698
chrXIII	820608	820751	143	N13:820698
chrXIII	820609	820751	142	N13:820698
chrXIII	820610	820751	141	N13:820698
chrXIII	820611	820751	140	N13:820698
chrXIII	820612	820751	139	N13:820698
chrXIII	820613	820751	138	N13:820698
chrXIII	820614	820751	137	N13:820698
chrXIII	820615	820751	136	N13:820698
chrXIII	820616	820751	135	N13:820698
chrXIII	820617	820751	134	N13:820698
chrXIII	820619	820751	132	N13:820698
chrXIII	820620	820751	131	N13:820698
chrXIII	820621	820751	130	N13:820698
chrXIII	820624	820751	127	N13:820698
chrXIII	820626	820715	89	N13:820698
chrXIII	828572	828720	148	N13:828670
chrXIII	835990	836099	109	
chrXIII	836333	836463	130	N13:836461
chrXIII	842098	842232	134	N13:842139
chrXIII	843431	843583	152	N13:843529
chrXIII	847888	848017	129	N13:847969
chrXIII	847897	848050	153	N13:847969
chrXIII	851484	851633	149	N13:851582
chrXIII	864286	864433	147	N13:864337
chrXIII	864644	864799	155	N13:864820
chrXIII	864644	864799	155	N13:864653
chrXIII	866005	866155	150	
chrXIII	868732	868890	158	N13:868810
chrXIII	871472	871623	151	N13:871631
chrXIII	871472	871623	151	N13:871471
chrXIII	873640	873761	121	N13:873682
chrXIII	873640	873789	149	N13:873682
chrXIII	873640	873790	150	N13:873682
chrXIII	873648	873761	113	N13:873682
chrXIII	873648	873789	141	N13:873682
chrXIII	873648	873790	142	N13:873682
chrXIII	878188	878341	153	N13:878290
chrXIII	879147	879302	155	N13:879224
chrXIII	884345	884496	151	N13:884479
chrXIII	886126	886284	158	
chrXIII	889335	889469	134	N13:889367
chrXIII	890068	890170	102	N13:890188
chrXIII	891024	891149	125	N13:891052
chrXIII	891955	892105	150	N13:892008
chrXIII	895348	895499	151	N13:895429
chrXIII	902827	902965	138	N13:902909
chrXIII	904201	904335	134	N13:904278
chrXIII	904428	904582	154	N13:904475
chrXIII	910695	910845	150	N13:910757
chrXIII	911000	911154	154	N13:911157
chrXIII	917204	917355	151	N13:917298
chrXIII	921093	921253	160	N13:921174
chrXIII	923830	923976	146	N13:923958
chrXIII	924304	924426	122	N13:924361
chrXIII	924311	924426	115	N13:924361
chrXIII	924321	924426	105	N13:924361
chrXIV	614	765	151	N14:689
chrXIV	953	1105	152	N14:1005
chrXIV	1779	1930	151	N14:1793
chrXIV	1791	1925	134	N14:1793
chrXIV	1816	1912	96	N14:1793
chrXIV	2170	2300	130	N14:2318
chrXIV	2449	2601	152	N14:2530
chrXIV	2796	2940	144	N14:2849

Chr	Start	End	Length (bp)	Nucleosome_ID
chrXIV	2796	2983	187	N14:2849
chrXIV	2848	2940	92	N14:2849
chrXIV	2848	2983	135	N14:2849
chrXIV	2936	3080	144	N14:3031
chrXIV	3001	3153	152	N14:3031
chrXIV	3253	3378	125	N14:3371
chrXIV	3283	3426	143	N14:3371
chrXIV	3584	3721	137	N14:3651
chrXIV	3700	3855	155	N14:3808
chrXIV	3794	3956	162	N14:3967
chrXIV	3794	3956	162	N14:3808
chrXIV	5019	5160	141	N14:5067
chrXIV	15620	15770	150	N14:15736
chrXIV	18901	19054	153	N14:18957
chrXIV	22418	22569	151	N14:22459
chrXIV	24850	25019	169	N14:24952
chrXIV	25836	25986	150	N14:25940
chrXIV	26530	26680	150	N14:26673
chrXIV	27268	27422	154	
chrXIV	30249	30400	151	N14:30360
chrXIV	32059	32210	151	N14:32102
chrXIV	33454	33599	145	N14:33540
chrXIV	33454	33606	152	N14:33540
chrXIV	33459	33599	140	N14:33540
chrXIV	33461	33599	138	N14:33540
chrXIV	33461	33603	142	N14:33540
chrXIV	33461	33606	145	N14:33540
chrXIV	33464	33606	142	N14:33540
chrXIV	33466	33606	140	N14:33540
chrXIV	41403	41548	145	N14:41462
chrXIV	45261	45368	107	N14:45343
chrXIV	45261	45374	113	N14:45343
chrXIV	45783	45937	154	N14:45851
chrXIV	49049	49199	150	N14:49135
chrXIV	53101	53263	162	N14:53189
chrXIV	55301	55450	149	N14:55396
chrXIV	58407	58536	129	N14:58525
chrXIV	60113	60210	97	
chrXIV	61184	61331	147	N14:61254
chrXIV	63570	63733	163	N14:63662
chrXIV	65407	65535	128	N14:65439
chrXIV	73936	74081	145	N14:73929
chrXIV	77741	77895	154	N14:77801
chrXIV	79985	80135	150	N14:80056
chrXIV	81420	81583	163	N14:81459
chrXIV	91093	91242	149	N14:91178
chrXIV	93267	93403	136	N14:93374
chrXIV	97284	97435	151	N14:97317
chrXIV	97941	98103	162	N14:98029
chrXIV	99408	99557	149	N14:99525
chrXIV	99632	99789	157	N14:99713
chrXIV	99818	99968	150	N14:99892
chrXIV	99822	99968	146	N14:99892
chrXIV	100280	100415	135	N14:100384
chrXIV	100932	101089	157	N14:101020
chrXIV	102362	102510	148	N14:102397
chrXIV	103973	104137	164	N14:104026
chrXIV	107707	107834	127	N14:107700
chrXIV	110079	110230	151	N14:110172
chrXIV	111620	111775	155	
chrXIV	112009	112129	120	N14:112032
chrXIV	116452	116578	126	N14:116519
chrXIV	119252	119378	126	N14:119284
chrXIV	121333	121464	131	N14:121346
chrXIV	124849	124975	126	N14:124938
chrXIV	129691	129843	152	N14:129772
chrXIV	129719	129870	151	N14:129772
chrXIV	131962	132135	173	N14:131992
chrXIV	132312	132463	151	N14:132390
chrXIV	133490	133654	164	N14:133593
chrXIV	134176	134325	149	N14:134291
chrXIV	135289	135440	151	N14:135423
chrXIV	135829	135979	150	N14:135901
chrXIV	139766	139921	155	N14:139942
chrXIV	140110	140269	159	N14:140187
chrXIV	141911	142060	149	N14:142074
chrXIV	150518	150667	149	N14:150605
chrXIV	153107	153271	164	N14:153137
chrXIV	153107	153271	164	N14:153294
chrXIV	155351	155502	151	N14:155389
chrXIV	163862	164000	138	N14:164012
chrXIV	163862	164000	138	N14:163855
chrXIV	165154	165280	126	N14:165235
chrXIV	167446	167585	139	N14:167470
chrXIV	174863	175007	144	N14:174931
chrXIV	177654	177807	153	N14:177732
chrXIV	178329	178461	132	N14:178391
chrXIV	183769	183934	165	N14:183818
chrXIV	187776	187960	184	N14:187755
chrXIV	188930	189078	148	
chrXIV	188930	189079	149	
chrXIV	188930	189080	150	
chrXIV	188930	189081	151	
chrXIV	188930	189083	153	
chrXIV	188931	189083	152	
chrXIV	188933	189083	150	
chrXIV	188934	189083	149	
chrXIV	188935	189083	148	

Chr	Start	End	Length (bp)	Nucleosome_ID
chrXIV	190891	191040	149	N14:190930
chrXIV	192308	192455	147	N14:192383
chrXIV	192813	192954	141	N14:192825
chrXIV	193504	193657	153	N14:193627
chrXIV	196760	196910	150	N14:196922
chrXIV	202136	202292	156	N14:202215
chrXIV	203542	203662	120	N14:203649
chrXIV	206857	207023	166	N14:206949
chrXIV	210139	210288	149	N14:210220
chrXIV	216142	216308	166	N14:216232
chrXIV	219506	219633	127	N14:219552
chrXIV	224826	224979	153	N14:224898
chrXIV	227320	227476	156	N14:227397
chrXIV	235082	235232	150	
chrXIV	238200	238369	169	N14:238254
chrXIV	246737	246897	160	N14:246814
chrXIV	247839	247988	149	N14:247931
chrXIV	254524	254675	151	N14:254603
chrXIV	257454	257590	136	N14:257486
chrXIV	257743	257893	150	N14:257796
chrXIV	259132	259283	151	N14:259110
chrXIV	259132	259283	151	N14:259278
chrXIV	265572	265700	128	N14:265642
chrXIV	271513	271667	154	N14:271563
chrXIV	273060	273209	149	N14:273068
chrXIV	273060	273209	149	N14:273226
chrXIV	275916	276056	140	N14:276038
chrXIV	281515	281622	107	N14:281537
chrXIV	281515	281631	116	N14:281537
chrXIV	281832	281976	144	N14:281889
chrXIV	283804	283989	185	N14:283836
chrXIV	287747	287882	135	N14:287841
chrXIV	291077	291208	131	N14:291175
chrXIV	292461	292633	172	N14:292551
chrXIV	292477	292630	153	N14:292551
chrXIV	296854	297025	171	N14:296864
chrXIV	297410	297562	152	N14:297470
chrXIV	298390	298549	159	N14:298469
chrXIV	302946	303074	128	N14:302995
chrXIV	303880	304030	150	N14:303933
chrXIV	307042	307199	157	N14:307110
chrXIV	308271	308446	175	N14:308444
chrXIV	308271	308446	175	N14:308284
chrXIV	309709	309860	151	N14:309803
chrXIV	310999	311146	147	N14:311066
chrXIV	314726	314877	151	N14:314793
chrXIV	317936	318050	114	N14:318021
chrXIV	324620	324765	145	N14:324681
chrXIV	324977	325117	140	N14:325028
chrXIV	336864	337018	154	N14:336949
chrXIV	339979	340132	153	N14:340063
chrXIV	340143	340297	154	
chrXIV	343939	344095	156	N14:344007
chrXIV	348424	348579	155	N14:348518
chrXIV	349341	349497	156	N14:349432
chrXIV	357765	357910	145	N14:357862
chrXIV	357767	357910	143	N14:357862
chrXIV	358288	358439	151	N14:358379
chrXIV	360600	360759	159	N14:360625
chrXIV	360762	360912	150	N14:360803
chrXIV	361214	361352	138	N14:361265
chrXIV	364273	364404	131	N14:364350
chrXIV	365246	365404	158	N14:365272
chrXIV	366145	366271	126	
chrXIV	368092	368240	148	N14:368205
chrXIV	369049	369214	165	N14:369138
chrXIV	376927	377089	162	N14:377019
chrXIV	384387	384546	159	N14:384456
chrXIV	388430	388576	146	N14:388511
chrXIV	388719	388880	161	N14:388701
chrXIV	388719	388880	161	N14:388900
chrXIV	396710	396865	155	N14:396752
chrXIV	403433	403582	149	N14:403451
chrXIV	403995	404145	150	
chrXIV	410286	410440	154	N14:410457
chrXIV	410286	410440	154	N14:410304
chrXIV	410317	410470	153	N14:410457
chrXIV	410317	410470	153	N14:410304
chrXIV	410968	411126	158	N14:411083
chrXIV	411008	411145	137	N14:411083
chrXIV	416136	416292	156	N14:416212
chrXIV	418446	418610	164	N14:418509
chrXIV	419242	419381	139	N14:419334
chrXIV	420269	420420	151	N14:420290
chrXIV	421794	421931	137	N14:421859
chrXIV	421911	422067	156	N14:422017
chrXIV	423473	423612	139	N14:423543
chrXIV	423497	423650	153	N14:423543
chrXIV	426375	426492	117	N14:426395
chrXIV	430126	430279	153	N14:430203
chrXIV	430126	430286	160	N14:430203
chrXIV	439717	439834	117	N14:439718
chrXIV	459511	459626	115	N14:459588
chrXIV	460164	460311	147	N14:460238
chrXIV	464412	464538	126	N14:464472
chrXIV	466515	466659	144	N14:466599
chrXIV	467295	467445	150	
chrXIV	473999	474106	107	N14:474082

Chr	Start	End	Length (bp)	Nucleosome_ID
chrXIV	475462	475606	144	N14:475517
chrXIV	477594	477739	145	N14:477658
chrXIV	478141	478275	134	N14:478165
chrXIV	483192	483305	113	
chrXIV	483272	483422	150	N14:483343
chrXIV	483529	483666	137	N14:483509
chrXIV	483529	483666	137	N14:483683
chrXIV	483752	483901	149	N14:483855
chrXIV	484694	484843	149	
chrXIV	490422	490573	151	N14:490508
chrXIV	490584	490699	115	N14:490670
chrXIV	490584	490718	134	N14:490670
chrXIV	490584	490722	138	N14:490670
chrXIV	500789	500918	129	N14:500830
chrXIV	502692	502828	136	N14:502692
chrXIV	511107	511261	154	
chrXIV	515776	515877	101	N14:515831
chrXIV	516941	517091	150	N14:517027
chrXIV	517457	517589	132	N14:517452
chrXIV	517457	517589	132	N14:517609
chrXIV	520229	520363	134	N14:520294
chrXIV	520229	520369	140	N14:520294
chrXIV	521272	521407	135	N14:521305
chrXIV	521719	521865	146	N14:521781
chrXIV	521719	521869	150	N14:521781
chrXIV	521898	522055	157	N14:521995
chrXIV	524015	524129	114	
chrXIV	524719	524868	149	N14:524853
chrXIV	524979	525110	131	N14:525025
chrXIV	526013	526193	180	N14:526078
chrXIV	526651	526800	149	N14:526736
chrXIV	527097	527235	138	N14:527172
chrXIV	527709	527871	162	N14:527797
chrXIV	529282	529444	162	N14:529327
chrXIV	529934	530079	145	N14:530020
chrXIV	530913	531056	143	
chrXIV	532041	532181	140	N14:532069
chrXIV	532139	532286	147	N14:532293
chrXIV	536984	537144	160	N14:537118
chrXIV	538071	538217	146	N14:538181
chrXIV	538102	538265	163	N14:538181
chrXIV	538210	538368	158	N14:538353
chrXIV	540636	540777	141	N14:540797
chrXIV	541287	541432	145	N14:541365
chrXIV	541780	541943	163	N14:541870
chrXIV	541920	542078	158	
chrXIV	542491	542640	149	N14:542475
chrXIV	544884	545027	143	N14:544952
chrXIV	544927	545097	170	N14:544952
chrXIV	546338	546446	108	N14:546423
chrXIV	546358	546520	162	N14:546423
chrXIV	551772	551902	130	N14:551848
chrXIV	552571	552722	151	N14:552590
chrXIV	553534	553684	150	N14:553556
chrXIV	554534	554685	151	N14:554609
chrXIV	555938	556085	147	N14:555999
chrXIV	557972	558127	155	N14:558022
chrXIV	560057	560198	141	N14:560145
chrXIV	561823	561978	155	N14:561825
chrXIV	562224	562373	149	N14:562251
chrXIV	563011	563177	166	N14:563098
chrXIV	564309	564452	143	N14:564390
chrXIV	565788	565917	129	N14:565899
chrXIV	567163	567273	110	N14:567239
chrXIV	573304	573437	133	N14:573369
chrXIV	575453	575613	160	N14:575635
chrXIV	575453	575613	160	N14:575487
chrXIV	576714	576844	130	N14:576799
chrXIV	576714	576864	150	N14:576799
chrXIV	578365	578517	152	N14:578459
chrXIV	578706	578859	153	N14:578787
chrXIV	585214	585366	152	N14:585310
chrXIV	589016	589141	125	N14:589151
chrXIV	589016	589141	125	N14:589003
chrXIV	593245	593393	148	N14:593327
chrXIV	597466	597618	152	N14:597483
chrXIV	598791	598924	133	N14:598861
chrXIV	602237	602346	109	N14:602324
chrXIV	612323	612470	147	N14:612426
chrXIV	615779	615947	168	N14:615899
chrXIV	620267	620398	131	N14:620245
chrXIV	620267	620398	131	N14:620397
chrXIV	620785	620892	107	N14:620770
chrXIV	620785	620946	161	N14:620770
chrXIV	620785	620946	161	N14:620937
chrXIV	625000	625108	108	N14:625110
chrXIV	625453	625609	156	N14:625501
chrXIV	630331	630483	152	N14:630418
chrXIV	630745	630896	151	N14:630914
chrXIV	630745	630896	151	N14:630752
chrXIV	635632	635775	143	N14:635681
chrXIV	640348	640505	157	N14:640377
chrXIV	640382	640540	158	N14:640377
chrXIV	640382	640540	158	N14:640553
chrXIV	641911	642059	148	N14:641943
chrXIV	645323	645472	149	N14:645480
chrXIV	645323	645472	149	N14:645332
chrXIV	646222	646370	148	N14:646249

Chr	Start	End	Length (bp)	Nucleosome_ID
chrXIV	649139	649287	148	N14:649302
chrXIV	649139	649287	148	N14:649141
chrXIV	659033	659204	171	N14:659107
chrXIV	660122	660273	151	
chrXIV	660694	660857	163	N14:660797
chrXIV	669731	669882	151	N14:669825
chrXIV	673304	673429	125	N14:673428
chrXIV	673304	673441	137	N14:673428
chrXIV	673304	673449	145	N14:673428
chrXIV	673304	673450	146	N14:673428
chrXIV	673304	673451	147	N14:673428
chrXIV	673304	673452	148	N14:673428
chrXIV	673304	673453	149	N14:673428
chrXIV	673304	673454	150	N14:673428
chrXIV	673304	673455	151	N14:673428
chrXIV	673305	673455	150	N14:673428
chrXIV	673306	673455	149	N14:673428
chrXIV	673307	673455	148	N14:673428
chrXIV	673308	673455	147	N14:673428
chrXIV	673309	673455	146	N14:673428
chrXIV	673310	673455	145	N14:673428
chrXIV	673311	673455	144	N14:673428
chrXIV	673313	673455	142	N14:673428
chrXIV	673314	673455	141	N14:673428
chrXIV	673321	673455	134	N14:673428
chrXIV	673329	673455	126	N14:673428
chrXIV	673357	673455	98	N14:673428
chrXIV	675190	675330	140	N14:675271
chrXIV	678978	679124	146	N14:679095
chrXIV	679561	679717	156	N14:679584
chrXIV	681968	682096	128	N14:682040
chrXIV	681972	682075	103	N14:682040
chrXIV	682856	682998	142	N14:682935
chrXIV	689540	689646	106	N14:689607
chrXIV	689540	689701	161	N14:689607
chrXIV	689540	689704	164	N14:689607
chrXIV	689540	689705	165	N14:689607
chrXIV	689541	689705	164	N14:689607
chrXIV	689543	689705	162	N14:689607
chrXIV	689545	689705	160	N14:689607
chrXIV	689550	689705	155	N14:689607
chrXIV	689554	689705	151	N14:689607
chrXIV	694543	694689	146	N14:694619
chrXIV	694549	694699	150	N14:694619
chrXIV	698880	699032	152	N14:698920
chrXIV	704009	704157	148	N14:704120
chrXIV	704660	704785	125	N14:704768
chrXIV	705096	705262	166	N14:705073
chrXIV	705096	705262	166	N14:705244
chrXIV	705533	705682	149	N14:705572
chrXIV	705820	705974	154	N14:705901
chrXIV	706013	706167	154	N14:706150
chrXIV	706675	706825	150	N14:706652
chrXIV	706675	706825	150	N14:706826
chrXIV	707449	707602	153	N14:707511
chrXIV	723355	723513	158	N14:723439
chrXIV	724785	724950	165	N14:724864
chrXIV	727236	727386	150	N14:727324
chrXIV	727633	727771	138	N14:727696
chrXIV	731977	732139	162	N14:732086
chrXIV	735524	735689	165	N14:735604
chrXIV	735878	736030	152	N14:735951
chrXIV	737786	737917	131	N14:737841
chrXIV	749149	749259	110	N14:749152
chrXIV	749725	749855	130	N14:749877
chrXIV	749823	749962	139	N14:749877
chrXIV	750208	750335	127	
chrXIV	753363	753527	164	N14:753511
chrXIV	754387	754526	139	N14:754466
chrXIV	761541	761643	102	N14:761639
chrXIV	763360	763525	165	N14:763380
chrXIV	763360	763525	165	N14:763542
chrXIV	767084	767240	156	N14:767184
chrXIV	773470	773620	150	N14:773461
chrXIV	778474	778625	151	N14:778523
chrXIV	779862	780020	158	N14:779923
chrXIV	781645	781793	148	N14:781689
chrXIV	781935	782064	129	N14:781999
chrXIV	781942	782106	164	N14:781999
chrXIV	783376	783501	125	N14:783457
chrXIV	784039	784210	171	N14:784097
chrXIV	784040	784199	159	N14:784097
chrXIV	784044	784183	139	N14:784097
chrXIV	784044	784199	155	N14:784097
chrXIV	784049	784198	149	N14:784097
chrXIV	784049	784203	154	N14:784097
chrXIV	784063	784183	120	N14:784097
chrXIV	784063	784195	132	N14:784097
chrXIV	784063	784199	136	N14:784097
chrXIV	784063	784202	139	N14:784097
chrXIV	784063	784210	147	N14:784097
chrXIV	784063	784233	170	N14:784097
chrXIV	784063	784239	176	N14:784097
chrXIV	784063	784239	176	N14:784261
chrXIV	784063	784275	212	N14:784097
chrXIV	784063	784275	212	N14:784261
chrXIV	784064	784183	119	N14:784097
chrXIV	784064	784199	135	N14:784097

Chr	Start	End	Length (bp)	Nucleosome_ID
chrXIV	784101	784203	102	N14:784097
chrXIV	784101	784210	109	N14:784097
chrXIV	784106	784198	92	N14:784097
chrXIV	784106	784203	97	N14:784097
chrXIV	784106	784210	104	N14:784097
chrXIV	784106	784238	132	N14:784097
chrXIV	784106	784238	132	N14:784261
chrXIV	784106	784265	159	N14:784097
chrXIV	784106	784265	159	N14:784261
chrXIV	784106	784274	168	N14:784097
chrXIV	784106	784274	168	N14:784261
chrXIV	784106	784275	169	N14:784097
chrXIV	784106	784275	169	N14:784261
chrXIV	784106	784277	171	N14:784097
chrXIV	784106	784277	171	N14:784261
chrXIV	784111	784233	122	N14:784097
chrXIV	784111	784286	175	N14:784097
chrXIV	784111	784286	175	N14:784261
chrXIV	784130	784238	108	N14:784261
chrXIV	784130	784274	144	N14:784261
chrXIV	784130	784275	145	N14:784261
chrXIV	784130	784277	147	N14:784261
chrXIV	784130	784329	199	N14:784261
chrXIV	784132	784275	143	N14:784261
chrXIV	784135	784274	139	N14:784261
chrXIV	784139	784275	136	N14:784261
chrXIV	784139	784296	157	N14:784261
chrXIV	784140	784275	135	N14:784261
chrXIV	784145	784286	141	N14:784261
chrXIV	784164	784275	111	N14:784261
chrXIV	784169	784274	105	N14:784261
chrXIV	784169	784275	106	N14:784261
chrXIV	784169	784277	108	N14:784261
chrXIV	784172	784265	93	N14:784261
chrXIV	784172	784267	95	N14:784261
chrXIV	784172	784271	99	N14:784261
chrXIV	784172	784274	102	N14:784261
chrXIV	784172	784275	103	N14:784261
chrXIV	784172	784277	105	N14:784261
chrXIV	784177	784271	94	N14:784261
chrXIV	784177	784275	98	N14:784261
chrXV	838	992	154	N15:915
chrXV	6743	6894	151	N15:6849
chrXV	8892	9048	156	N15:8985
chrXV	10875	10982	107	N15:10900
chrXV	23820	23972	152	N15:23919
chrXV	29238	29397	159	N15:29410
chrXV	29238	29397	159	N15:29253
chrXV	36163	36311	148	N15:36242
chrXV	39246	39406	160	N15:39349
chrXV	43767	43929	162	N15:43844
chrXV	44442	44591	149	N15:44533
chrXV	49181	49338	157	N15:49259
chrXV	51344	51469	125	
chrXV	53593	53699	106	
chrXV	53593	53708	115	
chrXV	53860	54011	151	N15:53913
chrXV	53996	54153	157	N15:54073
chrXV	63125	63255	130	N15:63178
chrXV	64118	64281	163	N15:64218
chrXV	66159	66329	170	N15:66265
chrXV	72755	72904	149	N15:72830
chrXV	74613	74750	137	N15:74669
chrXV	74660	74806	146	N15:74669
chrXV	74660	74806	146	N15:74825
chrXV	75646	75807	161	N15:75770
chrXV	80807	80978	171	N15:80842
chrXV	101110	101258	148	N15:101210
chrXV	108670	108830	160	N15:108728
chrXV	110950	111091	141	N15:111017
chrXV	112040	112197	157	N15:112099
chrXV	113588	113739	151	N15:113650
chrXV	116654	116808	154	N15:116777
chrXV	118023	118177	154	N15:118099
chrXV	118066	118215	149	N15:118099
chrXV	118240	118363	123	N15:118282
chrXV	118340	118495	155	N15:118453
chrXV	118969	119120	151	N15:119046
chrXV	118979	119124	145	N15:119046
chrXV	118997	119148	151	N15:119046
chrXV	119251	119405	154	N15:119384
chrXV	119285	119429	144	N15:119384
chrXV	119816	119951	135	N15:119853
chrXV	120263	120409	146	N15:120330
chrXV	120263	120413	150	N15:120330
chrXV	120442	120599	157	N15:120533
chrXV	120674	120823	149	N15:120708
chrXV	120933	121062	129	
chrXV	121845	121995	150	N15:121935
chrXV	122824	122966	142	N15:122896
chrXV	132108	132259	151	N15:132177
chrXV	132333	132482	149	N15:132411
chrXV	138290	138437	147	N15:138366
chrXV	141232	141379	147	N15:141343
chrXV	142140	142290	150	N15:142219
chrXV	143684	143828	144	N15:143766
chrXV	147632	147784	152	N15:147706
chrXV	152975	153131	156	N15:153029

Chr	Start	End	Length (bp)	Nucleosome_ID
chrXV	160615	160744	129	N15:160677
chrXV	162501	162638	137	N15:162550
chrXV	163008	163166	158	N15:163097
chrXV	163425	163564	139	N15:163509
chrXV	164146	164288	142	
chrXV	169600	169748	148	N15:169700
chrXV	171569	171743	174	N15:171764
chrXV	173781	173933	152	
chrXV	177092	177238	146	N15:177172
chrXV	177093	177211	118	N15:177172
chrXV	177093	177214	121	N15:177172
chrXV	177093	177217	124	N15:177172
chrXV	177093	177220	127	N15:177172
chrXV	177093	177221	128	N15:177172
chrXV	177093	177222	129	N15:177172
chrXV	177093	177223	130	N15:177172
chrXV	177093	177224	131	N15:177172
chrXV	177093	177225	132	N15:177172
chrXV	177093	177226	133	N15:177172
chrXV	177093	177227	134	N15:177172
chrXV	177093	177228	135	N15:177172
chrXV	177093	177229	136	N15:177172
chrXV	177093	177230	137	N15:177172
chrXV	177093	177231	138	N15:177172
chrXV	177093	177232	139	N15:177172
chrXV	177093	177233	140	N15:177172
chrXV	177093	177234	141	N15:177172
chrXV	177093	177235	142	N15:177172
chrXV	177093	177236	143	N15:177172
chrXV	177093	177237	144	N15:177172
chrXV	177093	177238	145	N15:177172
chrXV	177093	177239	146	N15:177172
chrXV	177094	177238	144	N15:177172
chrXV	177095	177238	143	N15:177172
chrXV	177096	177238	142	N15:177172
chrXV	177097	177238	141	N15:177172
chrXV	177098	177238	140	N15:177172
chrXV	177099	177238	139	N15:177172
chrXV	177100	177238	138	N15:177172
chrXV	177101	177238	137	N15:177172
chrXV	177102	177238	136	N15:177172
chrXV	177103	177238	135	N15:177172
chrXV	177104	177238	134	N15:177172
chrXV	177105	177238	133	N15:177172
chrXV	177106	177238	132	N15:177172
chrXV	177107	177238	131	N15:177172
chrXV	177109	177238	129	N15:177172
chrXV	177110	177238	128	N15:177172
chrXV	177111	177238	127	N15:177172
chrXV	177112	177238	126	N15:177172
chrXV	177113	177238	125	N15:177172
chrXV	177115	177238	123	N15:177172
chrXV	177117	177238	121	N15:177172
chrXV	177118	177238	120	N15:177172
chrXV	177119	177238	119	N15:177172
chrXV	177120	177238	118	N15:177172
chrXV	177121	177238	117	N15:177172
chrXV	177124	177238	114	N15:177172
chrXV	177125	177238	113	N15:177172
chrXV	177128	177238	110	N15:177172
chrXV	177130	177238	108	N15:177172
chrXV	177132	177217	85	N15:177172
chrXV	177132	177238	106	N15:177172
chrXV	177733	177893	160	N15:177858
chrXV	178523	178704	181	N15:178709
chrXV	185749	185889	140	N15:185774
chrXV	187300	187456	156	N15:187379
chrXV	189704	189862	158	N15:189795
chrXV	192332	192491	159	N15:192321
chrXV	196562	196707	145	N15:196728
chrXV	196562	196707	145	N15:196546
chrXV	198924	199072	148	N15:198961
chrXV	202849	203002	153	N15:202912
chrXV	205781	205911	130	N15:205866
chrXV	208753	208900	147	N15:208837
chrXV	209390	209535	145	N15:209458
chrXV	212639	212808	169	N15:212754
chrXV	214778	214916	138	N15:214872
chrXV	215741	215894	153	N15:215837
chrXV	218336	218507	171	N15:218350
chrXV	220428	220557	129	N15:220513
chrXV	222406	222534	128	N15:222459
chrXV	225244	225389	145	N15:225300
chrXV	227790	227919	129	N15:227846
chrXV	229356	229535	179	N15:229431
chrXV	229736	229903	167	N15:229797
chrXV	233701	233860	159	N15:233870
chrXV	233701	233860	159	N15:233712
chrXV	237293	237436	143	N15:237339
chrXV	242082	242240	158	N15:242059
chrXV	242082	242240	158	N15:242224
chrXV	243486	243636	150	N15:243571
chrXV	244606	244758	152	N15:244626
chrXV	244606	244758	152	N15:244776
chrXV	248190	248334	144	N15:248279
chrXV	248556	248722	166	
chrXV	255239	255421	182	N15:255344
chrXV	256726	256872	146	N15:256740

Chr	Start	End	Length (bp)	Nucleosome_ID
chrXV	260668	260810	142	
chrXV	261423	261595	172	N15:261549
chrXV	263580	263731	151	N15:263648
chrXV	265679	265840	161	N15:265718
chrXV	266859	267005	146	N15:266903
chrXV	268238	268379	141	N15:268361
chrXV	270968	271134	166	N15:271050
chrXV	271150	271308	158	N15:271220
chrXV	273050	273212	162	N15:273212
chrXV	276074	276211	137	N15:276066
chrXV	276074	276211	137	N15:276231
chrXV	276839	276983	144	N15:276892
chrXV	279591	279744	153	N15:279667
chrXV	280089	280217	128	
chrXV	282501	282650	149	N15:282573
chrXV	284484	284582	98	
chrXV	284650	284789	139	N15:284768
chrXV	284999	285154	155	N15:285080
chrXV	285195	285326	131	N15:285245
chrXV	292850	293005	155	N15:292974
chrXV	292905	293050	145	N15:292974
chrXV	293213	293372	159	N15:293294
chrXV	294193	294362	169	N15:294360
chrXV	298993	299142	149	N15:299031
chrXV	306450	306602	152	N15:306552
chrXV	311787	311943	156	N15:311928
chrXV	319552	319692	140	N15:319612
chrXV	320812	320963	151	N15:320792
chrXV	321565	321713	148	N15:321561
chrXV	327427	327560	133	N15:327498
chrXV	328438	328583	145	N15:328423
chrXV	328438	328583	145	N15:328597
chrXV	329812	329980	168	N15:329910
chrXV	338001	338161	160	N15:338135
chrXV	341700	341803	103	N15:341798
chrXV	348865	348972	107	N15:348916
chrXV	349258	349411	153	N15:349279
chrXV	351942	352092	150	N15:351997
chrXV	353769	353922	153	N15:353843
chrXV	356685	356815	130	N15:356719
chrXV	357881	358029	148	N15:357967
chrXV	359149	359277	128	
chrXV	359963	360110	147	N15:360012
chrXV	363241	363404	163	N15:363313
chrXV	363647	363809	162	N15:363630
chrXV	365392	365542	150	N15:365381
chrXV	365392	365542	150	N15:365558
chrXV	369266	369425	159	N15:369250
chrXV	369347	369504	157	N15:369518
chrXV	371267	371398	131	N15:371332
chrXV	371602	371749	147	N15:371676
chrXV	377796	377936	140	N15:377801
chrXV	384219	384368	149	N15:384312
chrXV	387959	388110	151	N15:388058
chrXV	389816	389942	126	N15:389861
chrXV	392262	392373	111	N15:392325
chrXV	392576	392737	161	N15:392661
chrXV	401254	401398	144	N15:401349
chrXV	403569	403709	140	
chrXV	405315	405460	145	N15:405385
chrXV	405735	405887	152	N15:405829
chrXV	408309	408462	153	N15:408405
chrXV	413175	413325	150	N15:413244
chrXV	413525	413650	125	N15:413578
chrXV	419166	419317	151	N15:419244
chrXV	420271	420429	158	N15:420382
chrXV	423240	423391	151	N15:423319
chrXV	423241	423391	150	N15:423319
chrXV	423242	423391	149	N15:423319
chrXV	423243	423391	148	N15:423319
chrXV	423245	423391	146	N15:423319
chrXV	433044	433202	158	N15:433178
chrXV	433171	433320	149	N15:433178
chrXV	433171	433320	149	N15:433331
chrXV	435682	435849	167	N15:435774
chrXV	441363	441514	151	N15:441436
chrXV	443421	443578	157	N15:443539
chrXV	445187	445337	150	N15:445171
chrXV	445187	445337	150	N15:445329
chrXV	446874	447012	138	N15:446940
chrXV	450011	450165	154	N15:450081
chrXV	456168	456329	161	N15:456318
chrXV	457433	457588	155	N15:457449
chrXV	457539	457691	152	N15:457612
chrXV	459473	459604	131	
chrXV	465612	465776	164	N15:465716
chrXV	469152	469262	110	N15:469219
chrXV	469152	469277	125	N15:469219
chrXV	480195	480360	165	N15:480344
chrXV	480209	480363	154	N15:480344
chrXV	480628	480755	127	N15:480718
chrXV	480628	480787	159	N15:480718
chrXV	480629	480736	107	N15:480718
chrXV	480629	480737	108	N15:480718
chrXV	480629	480738	109	N15:480718
chrXV	480629	480739	110	N15:480718
chrXV	480629	480741	112	N15:480718
chrXV	480629	480742	113	N15:480718

Chr	Start	End	Length (bp)	Nucleosome_ID
chrXV	480629	480743	114	N15:480718
chrXV	480629	480744	115	N15:480718
chrXV	480629	480745	116	N15:480718
chrXV	480629	480746	117	N15:480718
chrXV	480629	480747	118	N15:480718
chrXV	480629	480748	119	N15:480718
chrXV	480629	480749	120	N15:480718
chrXV	480629	480750	121	N15:480718
chrXV	480629	480751	122	N15:480718
chrXV	480629	480752	123	N15:480718
chrXV	480629	480753	124	N15:480718
chrXV	480629	480754	125	N15:480718
chrXV	480629	480755	126	N15:480718
chrXV	480629	480756	127	N15:480718
chrXV	480629	480757	128	N15:480718
chrXV	480629	480758	129	N15:480718
chrXV	480629	480759	130	N15:480718
chrXV	480629	480760	131	N15:480718
chrXV	480629	480761	132	N15:480718
chrXV	480629	480762	133	N15:480718
chrXV	480629	480763	134	N15:480718
chrXV	480629	480764	135	N15:480718
chrXV	480629	480765	136	N15:480718
chrXV	480629	480766	137	N15:480718
chrXV	480629	480767	138	N15:480718
chrXV	480629	480768	139	N15:480718
chrXV	480629	480769	140	N15:480718
chrXV	480629	480770	141	N15:480718
chrXV	480629	480771	142	N15:480718
chrXV	480629	480772	143	N15:480718
chrXV	480629	480773	144	N15:480718
chrXV	480629	480774	145	N15:480718
chrXV	480629	480775	146	N15:480718
chrXV	480629	480776	147	N15:480718
chrXV	480629	480777	148	N15:480718
chrXV	480629	480778	149	N15:480718
chrXV	480629	480779	150	N15:480718
chrXV	480629	480780	151	N15:480718
chrXV	480629	480781	152	N15:480718
chrXV	480629	480782	153	N15:480718
chrXV	480629	480783	154	N15:480718
chrXV	480629	480784	155	N15:480718
chrXV	480629	480785	156	N15:480718
chrXV	480629	480786	157	N15:480718
chrXV	480629	480787	158	N15:480718
chrXV	480629	480788	159	N15:480718
chrXV	480629	480789	160	N15:480718
chrXV	480630	480787	157	N15:480718
chrXV	480631	480787	156	N15:480718
chrXV	480632	480787	155	N15:480718
chrXV	480633	480787	154	N15:480718
chrXV	480634	480787	153	N15:480718
chrXV	480635	480787	152	N15:480718
chrXV	480636	480787	151	N15:480718
chrXV	480637	480787	150	N15:480718
chrXV	480638	480787	149	N15:480718
chrXV	480639	480787	148	N15:480718
chrXV	480640	480787	147	N15:480718
chrXV	480641	480787	146	N15:480718
chrXV	480642	480787	145	N15:480718
chrXV	480643	480787	144	N15:480718
chrXV	480644	480787	143	N15:480718
chrXV	480645	480787	142	N15:480718
chrXV	480646	480787	141	N15:480718
chrXV	480647	480787	140	N15:480718
chrXV	480648	480787	139	N15:480718
chrXV	480649	480787	138	N15:480718
chrXV	480650	480787	137	N15:480718
chrXV	480651	480787	136	N15:480718
chrXV	480652	480787	135	N15:480718
chrXV	480653	480787	134	N15:480718
chrXV	480654	480787	133	N15:480718
chrXV	480655	480787	132	N15:480718
chrXV	480656	480787	131	N15:480718
chrXV	480657	480787	130	N15:480718
chrXV	480658	480787	129	N15:480718
chrXV	480659	480787	128	N15:480718
chrXV	480660	480787	127	N15:480718
chrXV	480661	480787	126	N15:480718
chrXV	480662	480787	125	N15:480718
chrXV	480663	480787	124	N15:480718
chrXV	480664	480787	123	N15:480718
chrXV	480665	480787	122	N15:480718
chrXV	480666	480787	121	N15:480718
chrXV	480667	480787	120	N15:480718
chrXV	480668	480787	119	N15:480718
chrXV	480669	480787	118	N15:480718
chrXV	480670	480787	117	N15:480718
chrXV	480671	480787	116	N15:480718
chrXV	480672	480787	115	N15:480718
chrXV	480673	480787	114	N15:480718
chrXV	480674	480787	113	N15:480718
chrXV	480675	480787	112	N15:480718
chrXV	480676	480787	111	N15:480718
chrXV	480677	480787	110	N15:480718
chrXV	480678	480787	109	N15:480718
chrXV	480679	480787	108	N15:480718
chrXV	480680	480755	75	N15:480718

Chr	Start	End	Length (bp)	Nucleosome_ID
chrXV	480680	480787	107	N15:480718
chrXV	480681	480787	106	N15:480718
chrXV	480682	480787	105	N15:480718
chrXV	480683	480787	104	N15:480718
chrXV	480684	480787	103	N15:480718
chrXV	480685	480787	102	N15:480718
chrXV	480686	480787	101	N15:480718
chrXV	480687	480787	100	N15:480718
chrXV	480688	480787	99	N15:480718
chrXV	480689	480787	98	N15:480718
chrXV	480690	480787	97	N15:480718
chrXV	480691	480787	96	N15:480718
chrXV	480692	480787	95	N15:480718
chrXV	480693	480787	94	N15:480718
chrXV	480695	480787	92	N15:480718
chrXV	480696	480787	91	N15:480718
chrXV	480697	480787	90	N15:480718
chrXV	480698	480787	89	N15:480718
chrXV	480700	480787	87	N15:480718
chrXV	480701	480787	86	N15:480718
chrXV	480703	480787	84	N15:480718
chrXV	480704	480787	83	N15:480718
chrXV	482098	482249	151	N15:482205
chrXV	483506	483656	150	N15:483559
chrXV	490437	490586	149	
chrXV	492152	492296	144	
chrXV	492636	492766	130	N15:492727
chrXV	499981	500137	156	N15:499985
chrXV	500251	500392	141	N15:500266
chrXV	500870	501031	161	N15:500992
chrXV	504656	504829	173	N15:504757
chrXV	504839	504995	156	N15:504924
chrXV	505651	505827	176	N15:505793
chrXV	508541	508687	146	N15:508522
chrXV	512017	512169	152	N15:512077
chrXV	520467	520627	160	N15:520577
chrXV	521328	521487	159	N15:521406
chrXV	524434	524584	150	N15:524552
chrXV	526545	526704	159	N15:526589
chrXV	527282	527433	151	N15:527364
chrXV	528345	528493	148	N15:528465
chrXV	529088	529208	120	
chrXV	534111	534263	152	N15:534188
chrXV	535122	535279	157	
chrXV	539793	539942	149	N15:539889
chrXV	540123	540277	154	N15:540228
chrXV	542672	542801	129	N15:542768
chrXV	545231	545391	160	N15:545321
chrXV	545588	545715	127	N15:545625
chrXV	550006	550155	149	N15:550070
chrXV	550413	550558	145	N15:550502
chrXV	550604	550752	148	N15:550670
chrXV	559497	559585	88	N15:559589
chrXV	559497	559598	101	N15:559589
chrXV	559497	559601	104	N15:559589
chrXV	559497	559654	157	N15:559589
chrXV	559919	560068	149	N15:559990
chrXV	559929	560086	157	N15:559990
chrXV	565053	565199	146	N15:565222
chrXV	565053	565201	148	N15:565222
chrXV	565053	565202	149	N15:565222
chrXV	566775	566926	151	N15:566870
chrXV	576392	576555	163	N15:576489
chrXV	590114	590263	149	N15:590176
chrXV	590721	590873	152	N15:590856
chrXV	592017	592170	153	N15:592096
chrXV	592017	592179	162	N15:592096
chrXV	592023	592179	156	N15:592096
chrXV	592044	592126	82	N15:592096
chrXV	595352	595475	123	
chrXV	595452	595607	155	N15:595551
chrXV	595885	596019	134	N15:595950
chrXV	595885	596025	140	N15:595950
chrXV	596928	597063	135	N15:596968
chrXV	597189	597334	145	N15:597178
chrXV	597375	597525	150	N15:597445
chrXV	597554	597711	157	N15:597645
chrXV	598045	598174	129	
chrXV	598957	599107	150	N15:599055
chrXV	600156	600263	107	
chrXV	601179	601328	149	N15:601265
chrXV	603463	603616	153	N15:603547
chrXV	605449	605600	151	N15:605543
chrXV	606728	606884	156	N15:606775
chrXV	608095	608251	156	N15:608159
chrXV	610131	610285	154	N15:610149
chrXV	610432	610587	155	N15:610582
chrXV	610432	610587	155	N15:610423
chrXV	613403	613549	146	N15:613529
chrXV	617632	617793	161	N15:617725
chrXV	621001	621151	150	N15:621077
chrXV	622311	622441	130	
chrXV	624028	624159	131	N15:624066
chrXV	625834	625959	125	N15:625885
chrXV	626417	626570	153	N15:626508
chrXV	632144	632251	107	N15:632173
chrXV	632144	632253	109	N15:632173
chrXV	633485	633638	153	N15:633575

Chr	Start	End	Length (bp)	Nucleosome_ID
chrXV	635429	635578	149	N15:635550
chrXV	637512	637669	157	N15:637581
chrXV	641886	642041	155	N15:641956
chrXV	646176	646338	162	N15:646189
chrXV	647481	647636	155	N15:647630
chrXV	650796	650922	126	
chrXV	650923	651080	157	N15:650997
chrXV	651588	651740	152	N15:651687
chrXV	671862	672011	149	N15:671896
chrXV	674697	674849	152	N15:674786
chrXV	674884	675018	134	N15:674983
chrXV	677635	677796	161	N15:677669
chrXV	679372	679525	153	N15:679447
chrXV	680555	680689	134	N15:680616
chrXV	682390	682545	155	N15:682487
chrXV	682421	682570	149	N15:682487
chrXV	682600	682746	146	N15:682669
chrXV	683178	683329	151	N15:683179
chrXV	683178	683329	151	N15:683348
chrXV	683542	683705	163	N15:683602
chrXV	698388	698544	156	N15:698448
chrXV	699187	699320	133	N15:699226
chrXV	704256	704405	149	N15:704277
chrXV	705052	705198	146	N15:705134
chrXV	707863	708011	148	
chrXV	709269	709434	165	N15:709269
chrXV	709269	709434	165	N15:709445
chrXV	709269	709462	193	N15:709269
chrXV	709269	709462	193	N15:709445
chrXV	709332	709434	102	N15:709445
chrXV	709332	709462	130	N15:709445
chrXV	709396	709550	154	N15:709445
chrXV	709396	709571	175	N15:709445
chrXV	709403	709571	168	N15:709445
chrXV	712148	712278	130	N15:712206
chrXV	713754	713908	154	
chrXV	716082	716234	152	N15:716164
chrXV	717294	717452	158	N15:717288
chrXV	717294	717452	158	N15:717452
chrXV	728130	728275	145	N15:728119
chrXV	728130	728275	145	N15:728288
chrXV	729241	729409	168	N15:729229
chrXV	730369	730527	158	N15:730492
chrXV	730626	730772	146	N15:730666
chrXV	732904	733026	122	N15:732962
chrXV	736114	736267	153	N15:736157
chrXV	749719	749841	122	N15:749778
chrXV	751696	751803	107	N15:751682
chrXV	751696	751804	108	N15:751682
chrXV	751696	751805	109	N15:751682
chrXV	751696	751808	112	N15:751682
chrXV	759323	759474	151	N15:759342
chrXV	759886	760039	153	N15:759976
chrXV	762029	762161	132	N15:762082
chrXV	762283	762425	142	N15:762373
chrXV	763377	763524	147	N15:763477
chrXV	765404	765530	126	N15:765450
chrXV	766078	766228	150	N15:766247
chrXV	767013	767155	142	N15:767164
chrXV	767013	767155	142	N15:767010
chrXV	768735	768888	153	N15:768713
chrXV	770268	770418	150	
chrXV	773124	773277	153	N15:773165
chrXV	775176	775297	121	N15:775265
chrXV	777968	778119	151	N15:778110
chrXV	783192	783285	93	N15:783252
chrXV	789013	789147	134	N15:789066
chrXV	790833	790987	154	N15:790822
chrXV	794701	794852	151	
chrXV	796032	796168	136	N15:796058
chrXV	801709	801857	148	N15:801792
chrXV	802168	802313	145	N15:802287
chrXV	804681	804839	158	N15:804764
chrXV	805157	805300	143	N15:805287
chrXV	806800	806942	142	N15:806847
chrXV	806939	807090	151	N15:807004
chrXV	810091	810222	131	N15:810121
chrXV	815641	815767	126	N15:815726
chrXV	816418	816570	152	N15:816506
chrXV	816891	817023	132	N15:817009
chrXV	823212	823372	160	N15:823302
chrXV	823715	823880	165	N15:823900
chrXV	824770	824918	148	N15:824827
chrXV	824898	825045	147	N15:824984
chrXV	826750	826899	149	N15:826822
chrXV	828443	828590	147	N15:828514
chrXV	829595	829749	154	N15:829756
chrXV	832011	832158	147	N15:832060
chrXV	838047	838199	152	N15:838035
chrXV	838047	838199	152	N15:838195
chrXV	838749	838897	148	N15:838866
chrXV	840989	841143	154	N15:841051
chrXV	845931	846091	160	N15:845986
chrXV	851758	851911	153	N15:851815
chrXV	855604	855755	151	N15:855699
chrXV	856174	856339	165	N15:856207
chrXV	856222	856364	142	N15:856207
chrXV	860271	860423	152	N15:860352

Chr	Start	End	Length (bp)	Nucleosome_ID
chrXV	863117	863234	117	N15:863177
chrXV	866318	866474	156	N15:866417
chrXV	866636	866779	143	
chrXV	873282	873420	138	N15:873312
chrXV	873426	873558	132	N15:873537
chrXV	873491	873643	152	N15:873537
chrXV	883077	883230	153	N15:883079
chrXV	883077	883230	153	N15:883239
chrXV	884043	884200	157	N15:884168
chrXV	895782	895933	151	N15:895865
chrXV	900646	900802	156	N15:900717
chrXV	902699	902828	129	N15:902765
chrXV	904931	905079	148	N15:904922
chrXV	917190	917343	153	N15:917323
chrXV	920557	920665	108	N15:920598
chrXV	928849	929014	165	N15:928971
chrXV	932648	932807	159	N15:932684
chrXV	932960	933112	152	N15:933085
chrXV	937286	937429	143	N15:937309
chrXV	941867	942022	155	N15:941903
chrXV	943142	943285	143	N15:943207
chrXV	956493	956644	151	N15:956543
chrXV	958715	958860	145	N15:958840
chrXV	961158	961313	155	N15:961224
chrXV	963324	963474	150	N15:963309
chrXV	967141	967307	166	N15:967161
chrXV	967454	967605	151	N15:967556
chrXV	970738	970906	168	N15:970789
chrXV	970759	970913	154	N15:970789
chrXV	970847	970977	130	
chrXV	970875	970977	102	
chrXV	972298	972446	148	
chrXV	975111	975257	146	
chrXV	975228	975386	158	N15:975305
chrXV	975904	976053	149	N15:975989
chrXV	975908	976058	150	N15:975989
chrXV	977285	977436	151	N15:977376
chrXV	977616	977761	145	
chrXV	981987	982146	159	N15:982019
chrXV	986417	986558	141	N15:986505
chrXV	986699	986864	165	N15:986780
chrXV	987920	988058	138	N15:987920
chrXV	988983	989114	131	N15:989106
chrXV	989889	990033	144	N15:989965
chrXV	996318	996470	152	N15:996407
chrXV	997102	997264	162	N15:997152
chrXV	1000108	1000248	140	
chrXV	1000780	1000917	137	N15:1000816
chrXV	1001402	1001553	151	N15:1001454
chrXV	1002082	1002260	178	N15:1002190
chrXV	1004460	1004618	158	N15:1004637
chrXV	1004460	1004618	158	N15:1004454
chrXV	1005856	1006001	145	
chrXV	1008258	1008393	135	N15:1008325
chrXV	1019659	1019809	150	N15:1019734
chrXV	1019667	1019830	163	N15:1019734
chrXV	1023929	1024082	153	N15:1024013
chrXV	1035603	1035747	144	N15:1035709
chrXV	1036929	1037060	131	N15:1036972
chrXV	1041309	1041484	175	N15:1041351
chrXV	1042260	1042368	108	N15:1042335
chrXV	1042260	1042387	127	N15:1042335
chrXV	1042260	1042389	129	N15:1042335
chrXV	1042260	1042392	132	N15:1042335
chrXV	1042265	1042392	127	N15:1042335
chrXV	1042267	1042368	101	N15:1042335
chrXV	1043663	1043770	107	
chrXV	1043663	1043771	108	
chrXV	1043663	1043772	109	
chrXV	1047470	1047621	151	N15:1047478
chrXV	1047470	1047621	151	N15:1047635
chrXV	1050132	1050280	148	N15:1050219
chrXV	1050137	1050280	143	N15:1050219
chrXV	1050608	1050773	165	N15:1050703
chrXV	1050861	1050999	138	N15:1050887
chrXV	1052062	1052210	148	N15:1052087
chrXV	1056315	1056470	155	N15:1056388
chrXV	1059723	1059876	153	N15:1059763
chrXV	1060927	1061054	127	N15:1060979
chrXV	1061685	1061838	153	N15:1061757
chrXV	1062032	1062164	132	N15:1062167
chrXV	1062744	1062896	152	N15:1062785
chrXV	1071888	1072059	171	N15:1071953
chrXV	1073330	1073480	150	N15:1073396
chrXV	1080435	1080579	144	N15:1080493
chrXV	1085191	1085346	155	N15:1085246
chrXV	1086206	1086377	171	N15:1086303
chrXV	1086356	1086503	147	
chrXV	1086512	1086642	130	N15:1086572
chrXV	1086893	1087029	136	N15:1086930
chrXV	1087274	1087436	162	N15:1087405
chrXV	1087375	1087530	155	N15:1087405
chrXV	1087509	1087646	137	N15:1087575
chrXV	1087804	1087947	143	N15:1087853
chrXV	1087852	1087977	125	N15:1087853
chrXV	1088077	1088229	152	N15:1088185
chrXV	1088150	1088294	144	N15:1088185
chrXV	1088247	1088382	135	N15:1088344

Chr	Start	End	Length (bp)	Nucleosome_ID
chrXV	1088247	1088434	187	N15:1088344
chrXV	1088290	1088382	92	N15:1088344
chrXV	1088290	1088434	144	N15:1088344
chrXV	1088629	1088781	152	N15:1088692
chrXV	1088930	1089060	130	N15:1088919
chrXV	1089068	1089229	161	N15:1089094
chrXV	1090465	1090616	151	N15:1090543
chrXVI	523	674	151	N16:600
chrXVI	847	1021	174	N16:925
chrXVI	1688	1839	151	N16:1728
chrXVI	1700	1834	134	N16:1728
chrXVI	1725	1821	96	N16:1728
chrXVI	1725	1834	109	N16:1728
chrXVI	2079	2209	130	N16:2222
chrXVI	2079	2209	130	N16:2056
chrXVI	2358	2510	152	N16:2443
chrXVI	2705	2849	144	N16:2758
chrXVI	2705	2892	187	N16:2758
chrXVI	2757	2849	92	N16:2758
chrXVI	2757	2892	135	N16:2758
chrXVI	2845	2989	144	N16:2943
chrXVI	2910	3062	152	N16:2943
chrXVI	3162	3287	125	N16:3271
chrXVI	3192	3335	143	N16:3271
chrXVI	3192	3343	151	N16:3271
chrXVI	3493	3630	137	N16:3559
chrXVI	3609	3764	155	N16:3722
chrXVI	3703	3865	162	N16:3880
chrXVI	3703	3865	162	N16:3722
chrXVI	4928	5069	141	N16:4975
chrXVI	8461	8628	167	N16:8544
chrXVI	10566	10710	144	N16:10653
chrXVI	23283	23434	151	N16:23371
chrXVI	26164	26341	177	N16:26333
chrXVI	26440	26591	151	
chrXVI	30175	30330	155	N16:30253
chrXVI	30411	30566	155	N16:30492
chrXVI	37895	38053	158	N16:37928
chrXVI	41612	41762	150	N16:41620
chrXVI	41938	42057	119	
chrXVI	44256	44374	118	N16:44326
chrXVI	54075	54252	177	N16:54126
chrXVI	55410	55560	150	N16:55405
chrXVI	56814	56963	149	N16:56959
chrXVI	56988	57111	123	
chrXVI	57088	57243	155	N16:57189
chrXVI	57521	57655	134	N16:57591
chrXVI	57521	57661	140	N16:57591
chrXVI	57717	57868	151	N16:57746
chrXVI	57727	57872	145	N16:57746
chrXVI	57747	57896	149	N16:57746
chrXVI	57999	58153	154	N16:58141
chrXVI	58564	58699	135	N16:58596
chrXVI	58718	58879	161	N16:58834
chrXVI	59011	59161	150	N16:59079
chrXVI	59190	59347	157	N16:59296
chrXVI	59681	59810	129	N16:59829
chrXVI	60593	60743	150	N16:60686
chrXVI	61572	61714	142	N16:61694
chrXVI	66888	67040	152	N16:66990
chrXVI	67654	67814	160	N16:67736
chrXVI	69456	69617	161	N16:69502
chrXVI	73482	73631	149	N16:73588
chrXVI	74222	74371	149	N16:74321
chrXVI	85235	85390	155	N16:85298
chrXVI	86229	86396	167	N16:86219
chrXVI	93799	93933	134	N16:93856
chrXVI	97906	98054	148	N16:97910
chrXVI	100090	100196	106	N16:100168
chrXVI	101988	102144	156	N16:102072
chrXVI	111563	111694	131	N16:111614
chrXVI	111839	111996	157	N16:111919
chrXVI	112816	112964	148	N16:112902
chrXVI	113851	114002	151	N16:113884
chrXVI	115900	116007	107	N16:115995
chrXVI	115900	116008	108	N16:115995
chrXVI	115900	116009	109	N16:115995
chrXVI	115900	116013	113	N16:115995
chrXVI	117285	117438	153	
chrXVI	119500	119650	150	N16:119550
chrXVI	119632	119770	138	N16:119717
chrXVI	124878	125016	138	N16:124969
chrXVI	127061	127209	148	N16:127222
chrXVI	127061	127209	148	N16:127054
chrXVI	127345	127506	161	N16:127392
chrXVI	128019	128185	166	N16:128056
chrXVI	128210	128316	106	N16:128260
chrXVI	128210	128332	122	N16:128260
chrXVI	130172	130324	152	N16:130248
chrXVI	131123	131279	156	N16:131253
chrXVI	136763	136914	151	N16:136878
chrXVI	136805	136963	158	N16:136878
chrXVI	139377	139534	157	N16:139490
chrXVI	142443	142561	118	N16:142521
chrXVI	144178	144336	158	N16:144285
chrXVI	149028	149196	168	N16:149123
chrXVI	152572	152722	150	N16:152635
chrXVI	161247	161414	167	N16:161357

Chr	Start	End	Length (bp)	Nucleosome_ID
chrXVI	164847	164957	110	N16:164935
chrXVI	165348	165502	154	N16:165511
chrXVI	167030	167184	154	N16:167113
chrXVI	169266	169418	152	N16:169376
chrXVI	173412	173549	137	N16:173465
chrXVI	174194	174363	169	N16:174232
chrXVI	175111	175256	145	N16:175175
chrXVI	177466	177621	155	N16:177552
chrXVI	177625	177797	172	N16:177780
chrXVI	189167	189337	170	N16:189180
chrXVI	190409	190560	151	N16:190473
chrXVI	191083	191228	145	N16:191166
chrXVI	193727	193886	159	N16:193851
chrXVI	194794	194946	152	N16:194885
chrXVI	198714	198866	152	N16:198810
chrXVI	200785	200926	141	
chrXVI	203076	203237	161	N16:203255
chrXVI	203882	204036	154	N16:203940
chrXVI	205158	205313	155	N16:205222
chrXVI	211347	211529	182	N16:211407
chrXVI	212567	212715	148	N16:212620
chrXVI	213085	213234	149	N16:213180
chrXVI	220298	220444	146	N16:220437
chrXVI	227791	227944	153	
chrXVI	228023	228173	150	N16:228073
chrXVI	235024	235161	137	N16:235068
chrXVI	238735	238888	153	N16:238814
chrXVI	239149	239288	139	N16:239177
chrXVI	240071	240209	138	N16:240084
chrXVI	240947	241098	151	N16:241005
chrXVI	242739	242891	152	N16:242817
chrXVI	242995	243131	136	N16:243075
chrXVI	244964	245114	150	N16:245006
chrXVI	246169	246331	162	N16:246246
chrXVI	247500	247655	155	
chrXVI	247870	248025	155	N16:248033
chrXVI	248518	248667	149	N16:248596
chrXVI	251350	251499	149	N16:251383
chrXVI	251647	251802	155	N16:251733
chrXVI	254749	254901	152	N16:254752
chrXVI	254749	254901	152	N16:254920
chrXVI	257303	257455	152	N16:257371
chrXVI	258470	258614	144	N16:258522
chrXVI	269441	269592	151	N16:269499
chrXVI	272088	272225	137	N16:272113
chrXVI	273849	274002	153	N16:273925
chrXVI	275518	275683	165	N16:275553
chrXVI	278767	278916	149	N16:278752
chrXVI	284968	285121	153	N16:285013
chrXVI	287278	287435	157	N16:287376
chrXVI	287516	287643	127	N16:287547
chrXVI	295119	295280	161	N16:295214
chrXVI	296343	296490	147	N16:296416
chrXVI	301254	301382	128	N16:301333
chrXVI	305851	306015	164	N16:305962
chrXVI	306387	306542	155	N16:306463
chrXVI	317763	317907	144	N16:317793
chrXVI	318699	318846	147	
chrXVI	322025	322158	133	N16:322082
chrXVI	323166	323321	155	N16:323256
chrXVI	332594	332745	151	
chrXVI	332948	333099	151	N16:333032
chrXVI	336537	336643	106	N16:336620
chrXVI	336537	336650	113	N16:336620
chrXVI	341289	341447	158	N16:341297
chrXVI	344751	344913	162	N16:344736
chrXVI	344751	344913	162	N16:344907
chrXVI	350439	350593	154	N16:350549
chrXVI	351311	351471	160	N16:351332
chrXVI	354968	355128	160	N16:355047
chrXVI	361977	362073	96	N16:362047
chrXVI	364336	364488	152	N16:364416
chrXVI	366545	366652	107	N16:366569
chrXVI	366545	366653	108	N16:366569
chrXVI	367044	367180	136	N16:367128
chrXVI	370599	370734	135	N16:370630
chrXVI	370905	371054	149	N16:370964
chrXVI	373474	373616	142	N16:373635
chrXVI	379313	379470	157	N16:379337
chrXVI	379794	379947	153	
chrXVI	379982	380135	153	N16:379982
chrXVI	379982	380135	153	N16:380145
chrXVI	383644	383787	143	N16:383784
chrXVI	385233	385386	153	N16:385330
chrXVI	389294	389391	97	N16:389336
chrXVI	390081	390232	151	N16:390091
chrXVI	390081	390232	151	N16:390239
chrXVI	390545	390691	146	N16:390526
chrXVI	392582	392731	149	N16:392651
chrXVI	392582	392736	154	N16:392651
chrXVI	394420	394573	153	N16:394533
chrXVI	407155	407307	152	N16:407273
chrXVI	407160	407307	147	N16:407273
chrXVI	411818	411982	164	N16:411890
chrXVI	411838	411991	153	N16:411890
chrXVI	412957	413111	154	
chrXVI	415881	416027	146	N16:415956
chrXVI	417433	417565	132	N16:417568

Chr	Start	End	Length (bp)	Nucleosome_ID
chrXVI	418493	418641	148	
chrXVI	418678	418846	168	N16:418685
chrXVI	418678	418846	168	N16:418866
chrXVI	432250	432440	190	N16:432391
chrXVI	432594	432737	143	N16:432613
chrXVI	432908	433068	160	N16:433087
chrXVI	433481	433638	157	N16:433601
chrXVI	437116	437278	162	N16:437280
chrXVI	439535	439688	153	
chrXVI	442027	442174	147	N16:442080
chrXVI	442326	442474	148	N16:442468
chrXVI	442340	442502	162	N16:442468
chrXVI	446281	446447	166	N16:446378
chrXVI	448877	449010	133	N16:448925
chrXVI	454839	454945	106	N16:454945
chrXVI	462929	463059	130	N16:462975
chrXVI	465016	465172	156	N16:465100
chrXVI	466157	466315	158	N16:466243
chrXVI	474538	474690	152	N16:474587
chrXVI	484126	484288	162	N16:484123
chrXVI	484126	484288	162	N16:484296
chrXVI	484807	484963	156	
chrXVI	487013	487184	171	N16:487139
chrXVI	493217	493377	160	N16:493271
chrXVI	497274	497428	154	N16:497348
chrXVI	501076	501229	153	N16:501190
chrXVI	501841	501999	158	N16:501884
chrXVI	505233	505380	147	N16:505303
chrXVI	506320	506478	158	N16:506395
chrXVI	506594	506750	156	N16:506676
chrXVI	509864	510024	160	N16:509922
chrXVI	511391	511535	144	N16:511439
chrXVI	517443	517595	152	N16:517508
chrXVI	519049	519173	124	N16:519058
chrXVI	520082	520209	127	N16:520178
chrXVI	522508	522654	146	N16:522677
chrXVI	522574	522734	160	N16:522677
chrXVI	525954	526108	154	N16:526036
chrXVI	536923	537076	153	N16:537021
chrXVI	538001	538163	162	N16:538105
chrXVI	541695	541831	136	N16:541839
chrXVI	547021	547160	139	N16:547005
chrXVI	547636	547771	135	N16:547723
chrXVI	547851	548005	154	N16:547889
chrXVI	550617	550788	171	N16:550762
chrXVI	553457	553564	107	N16:553491
chrXVI	553457	553565	108	N16:553491
chrXVI	560771	560926	155	N16:560761
chrXVI	560771	560926	155	N16:560933
chrXVI	564313	564461	148	N16:564375
chrXVI	564317	564438	121	N16:564375
chrXVI	566468	566620	152	N16:566631
chrXVI	566468	566620	152	N16:566462
chrXVI	568077	568211	134	N16:568157
chrXVI	573444	573609	165	N16:573552
chrXVI	573688	573839	151	N16:573733
chrXVI	578898	579048	150	N16:579014
chrXVI	580106	580256	150	N16:580137
chrXVI	581758	581907	149	N16:581737
chrXVI	581758	581907	149	N16:581900
chrXVI	582967	583116	149	N16:583058
chrXVI	584868	585014	146	N16:584868
chrXVI	591560	591713	153	N16:591636
chrXVI	592210	592352	142	N16:592274
chrXVI	593528	593683	155	N16:593589
chrXVI	594996	595138	142	N16:595089
chrXVI	599046	599200	154	N16:599073
chrXVI	600831	600997	166	N16:600995
chrXVI	600831	600997	166	N16:600844
chrXVI	601108	601231	123	N16:601151
chrXVI	603139	603315	176	N16:603211
chrXVI	604400	604533	133	N16:604513
chrXVI	611112	611263	151	
chrXVI	611769	611929	160	N16:611782
chrXVI	611769	611929	160	N16:611938
chrXVI	614264	614371	107	N16:614344
chrXVI	614264	614381	117	N16:614344
chrXVI	616962	617122	160	N16:617008
chrXVI	617750	617867	117	N16:617790
chrXVI	617888	618049	161	N16:617954
chrXVI	623817	623987	170	N16:623864
chrXVI	627166	627316	150	N16:627249
chrXVI	630544	630672	128	
chrXVI	630787	630947	160	N16:630889
chrXVI	639745	639881	136	N16:639847
chrXVI	644095	644255	160	N16:644162
chrXVI	647938	648089	151	N16:647982
chrXVI	651006	651170	164	N16:651112
chrXVI	651448	651577	129	N16:651501
chrXVI	651814	651966	152	
chrXVI	655669	655818	149	N16:655729
chrXVI	661336	661465	129	N16:661468
chrXVI	664409	664571	162	N16:664522
chrXVI	665020	665144	124	N16:665145
chrXVI	670319	670426	107	N16:670368
chrXVI	670319	670427	108	N16:670368
chrXVI	670635	670782	147	N16:670711
chrXVI	672000	672158	158	N16:672118

Chr	Start	End	Length (bp)	Nucleosome_ID
chrXVI	678051	678201	150	N16:678147
chrXVI	679094	679205	111	N16:679146
chrXVI	679094	679206	112	N16:679146
chrXVI	679094	679207	113	N16:679146
chrXVI	679098	679205	107	N16:679146
chrXVI	679098	679220	122	N16:679146
chrXVI	681181	681332	151	N16:681246
chrXVI	685561	685687	126	N16:685673
chrXVI	685866	686024	158	N16:685893
chrXVI	689048	689183	135	N16:689086
chrXVI	695989	696136	147	N16:696027
chrXVI	697045	697195	150	N16:697144
chrXVI	702075	702202	127	N16:702218
chrXVI	706433	706576	143	N16:706496
chrXVI	719549	719656	107	N16:719618
chrXVI	719549	719694	145	N16:719618
chrXVI	721766	721909	143	N16:721871
chrXVI	722565	722701	136	N16:722569
chrXVI	726383	726544	161	N16:726563
chrXVI	726865	727012	147	N16:726909
chrXVI	730093	730221	128	N16:730129
chrXVI	732571	732734	163	N16:732631
chrXVI	733908	734067	159	N16:733903
chrXVI	734318	734467	149	N16:734451
chrXVI	735641	735790	149	N16:735692
chrXVI	735832	735978	146	N16:735865
chrXVI	736889	737030	141	N16:736965
chrXVI	737626	737736	110	N16:737632
chrXVI	739761	739921	160	N16:739810
chrXVI	740972	741124	152	N16:741035
chrXVI	742321	742473	152	N16:742396
chrXVI	745613	745766	153	N16:745699
chrXVI	747443	747595	152	N16:747452
chrXVI	748166	748325	159	N16:748291
chrXVI	751513	751665	152	N16:751585
chrXVI	753264	753415	151	N16:753426
chrXVI	753264	753415	151	N16:753263
chrXVI	753454	753608	154	N16:753612
chrXVI	759400	759547	147	N16:759472
chrXVI	761447	761589	142	N16:761432
chrXVI	761447	761589	142	N16:761585
chrXVI	763063	763215	152	N16:763092
chrXVI	763430	763578	148	N16:763564
chrXVI	764397	764544	147	N16:764439
chrXVI	764502	764659	157	N16:764652
chrXVI	770323	770455	132	N16:770351
chrXVI	778559	778687	128	N16:778565
chrXVI	786310	786475	165	
chrXVI	788541	788654	113	
chrXVI	788648	788776	128	N16:788711
chrXVI	790762	790920	158	N16:790928
chrXVI	791267	791414	147	N16:791367
chrXVI	791438	791595	157	N16:791540
chrXVI	791737	791890	153	N16:791867
chrXVI	792774	792924	150	N16:792865
chrXVI	794619	794743	124	N16:794744
chrXVI	796842	796998	156	N16:796924
chrXVI	798394	798554	160	N16:798384
chrXVI	798628	798777	149	N16:798655
chrXVI	805070	805217	147	N16:805107
chrXVI	805577	805691	114	
chrXVI	807669	807826	157	N16:807711
chrXVI	808317	808452	135	N16:808366
chrXVI	809355	809495	140	N16:809434
chrXVI	809361	809495	134	N16:809434
chrXVI	809773	809928	155	N16:809806
chrXVI	809904	810012	108	N16:809957
chrXVI	809934	810052	118	N16:809957
chrXVI	810053	810202	149	N16:810107
chrXVI	813679	813806	127	N16:813742
chrXVI	822438	822593	155	
chrXVI	822870	822974	104	N16:822924
chrXVI	827045	827170	125	N16:827139
chrXVI	827247	827389	142	N16:827298
chrXVI	827869	828021	152	N16:827957
chrXVI	830012	830156	144	N16:830081
chrXVI	839224	839390	166	N16:839304
chrXVI	841656	841805	149	
chrXVI	843827	843986	159	N16:843902
chrXVI	844949	845072	123	
chrXVI	845049	845204	155	N16:845158
chrXVI	845482	845616	134	N16:845543
chrXVI	845482	845622	140	N16:845543
chrXVI	846525	846660	135	N16:846560
chrXVI	847154	847311	157	N16:847244
chrXVI	848557	848707	150	N16:848653
chrXVI	849289	849403	114	N16:849394
chrXVI	851012	851164	152	N16:851100
chrXVI	851565	851679	114	N16:851699
chrXVI	853843	853993	150	N16:853920
chrXVI	853847	853993	146	N16:853920
chrXVI	854031	854169	138	N16:854079
chrXVI	854126	854258	132	N16:854239
chrXVI	854305	854440	135	N16:854397
chrXVI	854851	855005	154	N16:854861
chrXVI	855108	855259	151	N16:855198
chrXVI	855132	855277	145	N16:855198
chrXVI	855136	855287	151	N16:855198

Chr	Start	End	Length (bp)	Nucleosome_ID
chrXVI	855343	855483	140	N16:855418
chrXVI	855349	855483	134	N16:855418
chrXVI	855892	856000	108	N16:855993
chrXVI	856112	856214	102	
chrXVI	858667	858810	143	N16:858736
chrXVI	863879	864033	154	N16:863878
chrXVI	863879	864033	154	N16:864033
chrXVI	864255	864385	130	N16:864244
chrXVI	867145	867288	143	
chrXVI	868632	868780	148	N16:868711
chrXVI	888008	888137	129	N16:888074
chrXVI	891517	891679	162	N16:891562
chrXVI	893449	893599	150	N16:893574
chrXVI	897488	897648	160	N16:897619
chrXVI	897990	898142	152	N16:898110
chrXVI	901200	901349	149	N16:901238
chrXVI	905702	905842	140	N16:905808
chrXVI	910317	910457	140	N16:910477
chrXVI	910317	910457	140	N16:910305
chrXVI	922519	922676	157	N16:922581
chrXVI	922519	922681	162	N16:922581
chrXVI	922523	922681	158	N16:922581
chrXVI	927439	927574	135	N16:927563
chrXVI	930833	931008	175	N16:930869
chrXVI	931493	931642	149	N16:931566
chrXVI	940363	940515	152	N16:940386
chrXVI	943076	943229	153	N16:943200
chrXVI	943085	943229	144	N16:943200
chrXVI	944252	944389	137	N16:944314
chrXVI	944537	944680	143	N16:944589
chrXVI	944585	944710	125	N16:944589
chrXVI	944810	944962	152	N16:944922
chrXVI	944883	945027	144	N16:944922
chrXVI	944980	945115	135	N16:945080
chrXVI	944980	945167	187	N16:945080
chrXVI	945023	945115	92	N16:945080
chrXVI	945023	945167	144	N16:945080
chrXVI	945362	945514	152	N16:945422
chrXVI	945663	945793	130	N16:945658
chrXVI	945663	945793	130	N16:945816
chrXVI	945759	945954	195	N16:945816
chrXVI	945821	945954	133	N16:945816
chrXVI	945821	945969	148	N16:945816
chrXVI	946033	946184	151	N16:946078
chrXVI	946038	946172	134	N16:946078
chrXVI	946051	946147	96	N16:946078
chrXVI	946159	946281	122	
chrXVI	946966	947118	152	N16:947053
chrXVI	947306	947457	151	N16:947384
