# Supplementary material for: Nucleosomal DNA has topological memory
Source: Nat Commun. 2024 May 28;15:4526. doi: 10.1038/s41467-024-49023-4 (PMC11133463; doi:10.1038/s41467-024-49023-4)
Supplement: Supplementary file 5 — Supplementary Data 2 [file 41467_2024_49023_MOESM5_ESM.pdf]

Supplementary Data 2. Calculation of the ΔLk restrained by individual nucleosomes

The four first columns indicate the chromosomal coordinates of the nucleosome library. The next two columns indicate the relative abundance (number of reads relative to total reads) of each nucleosome sequence in sections A and B of the gel electrophoresis. Column A/A+B indicates the partition probability of each sequence in section A. Column Z indicates the corresponding Z-scores of the previous partition probabilities in a normal distribution of mean 0 and standard deviation 1. The next column, multiplies Z-scores by 0.85 (mean standard deviation of the Lk distributions of the library) to yield approximate ΔLk deviations within a normal distribution of mean 0. The next column, Phase correction, subtracts from the previous ΔLk values the average ΔLk of all the sequences of the same length. The next column, Length correction, adjusts the previous ΔLk value by adding or subtracting 0.007 for each bp difference from 144 bp (the average length of the library of nucleosome DNA sequences). The last column adds -1.26 to obtain the ΔLk restrained by each nucleosome.

Note that, in some cases, a small positional offset in the nucleosome coordinates is accompanied by a significant difference in the obtained ΔLknuc value. They are however rare. Indicatively, only 90 out of the 3404 overlapping pairs (2.6%) nucleosomes have ΔLknuc values that are >1.5-fold different (greater or smaller) than one of their neighbours and only 152 (4.4%) have values >1.3-fold. Such discrepancies are expected given: a) the inherent variability in nucleosome positioning that exists naturally in cellular populations and b) the process of our calculation steps, which unavoidably incorporates some degree of noise in the final values. This is precisely why this study primarily focused on the presentation, interpretation and discussion of mean values, which are by definition more representative of the populations and more descriptive of general trends that can be meaningfully associated with underlying molecular and biophysical properties of chromatin.

Chrom	Start	End	Length	Section A	Section B	A/A+B	Z-score	Z * 0.85	Phase correction	Length correction	ΔLKnuc
chrI	375	485	110	0,324849907	2,657108488	0,108938444	-1,232193295	-1,047364301	-0,453273061	-0,215273061	**-1,475273061**
chrI	1992	2143	151	1,929152515	0,952408482	0,669481756	0,438482589	0,372710201	0,163375138	0,114375138	**-1,145624862**
chrI	3900	4046	146	0,185221438	0,159352392	0,537537741	0,094232436	0,080097571	-0,15491971	-0,16891971	**-1,42891971**
chrI	7310	7448	138	0,088336378	0,059293913	0,598362147	0,249109985	0,211743487	0,118785994	0,160785994	**-1,099214006**
chrI	19698	19849	151	0,153876272	0,081529131	0,653664997	0,395234266	0,335949126	0,138279312	0,089279312	**-1,170720688**
chrI	24106	24256	150	0,373292436	0,133411305	0,73670748	0,633227577	0,53824344	0,299199857	0,257199857	**-1,002800143**
chrI	24106	24267	161	0,062690333	0,066705652	0,484484373	-0,038901718	-0,03306646	0,007591221	-0,111408779	**-1,371408779**
chrI	24156	24264	108	3,769968652	21,72380747	0,147878001	-1,045577803	-0,888741132	-0,21236164	0,03963836	**-1,22036164**
chrI	24156	24265	109	0,085486818	0,422469132	0,16829573	-0,960921856	-0,816783577	-0,184955994	0,060044006	**-1,199955994**
chrI	24156	24266	110	0,051292091	0,604056741	0,078266853	-1,416826344	-1,204302393	-0,642918922	-0,404918922	**-1,664918922**
chrI	24156	24267	111	4,513703964	28,94284141	0,134912434	-1,103465956	-0,937946063	-0,405214917	-0,174214917	**-1,434214917**
chrI	25658	25790	132	0,330549028	0,233469783	0,586060289	0,217422085	0,184808773	0,27821694	0,36221694	**-0,89778306**
chrI	25658	25797	139	2,68428607	1,474936092	0,645381748	0,372881685	0,316949432	0,206470586	0,241470586	**-1,018529414**
chrI	25671	25797	126	0,094035499	0,114881957	0,450108387	-0,125387512	-0,106579385	0,227304304	0,353304304	**-0,906695696**
chrI	25885	26057	172	0,062690333	0,037058696	0,628480635	0,327831948	0,278657156	0,447151435	0,251151435	**-1,008848565**
chrI	26200	26329	129	0,028495606	0,100058479	0,221662392	-0,766590907	-0,651602271	-0,419518143	-0,314518143	**-1,574518143**
chrI	26200	26330	130	14,93454702	19,2594042	0,436759909	-0,159189193	-0,135310814	0,067390633	0,165390633	**-1,094609367**
chrI	26200	26337	137	0,772230918	0,61517435	0,556600826	0,142356591	0,121003103	0,041760198	0,090760198	**-1,169239802**
chrI	26204	26330	126	0,079787696	0,122293696	0,394829506	-0,266753431	-0,226740416	0,087246837	0,213246837	**-1,046753163**
chrI	26211	26330	119	0,225115286	0,285351958	0,440998495	-0,148438156	-0,126172432	0,30764548	0,48264548	**-0,77735452**
chrI	26268	26418	150	0,416035845	0,237175653	0,636908331	0,350207021	0,297675968	0,072283311	0,030283311	**-1,229716689**
chrI	26290	26465	175	0,034194727	0,092646739	0,269586342	-0,614064532	-0,521954852	-0,350207551	-0,567207551	**-1,827207551**
chrI	26425	26573	148	0,467327936	0,203822827	0,696308433	0,513812434	0,436740568	0,196075677	0,168075677	**-1,091924323**
chrI	26560	26708	148	0,3903898	0,222352175	0,637119401	0,35076961	0,298154169	0,036820934	0,008820934	**-1,251179066**
chrI	26695	26843	148	0,404637603	0,166764131	0,708149064	0,54798548	0,465787658	0,197216135	0,169216135	**-1,090783865**
chrI	27347	27495	148	0,447381012	0,307587175	0,592582601	0,234193452	0,199064434	-0,069301342	-0,097301342	**-1,357301342**
chrI	28545	28653	108	0,219416165	0,548468698	0,285740969	-0,565870321	-0,480989773	0,192229317	0,444229317	**-0,815770683**
chrI	28558	28705	147	0,29350474	0,329822392	0,470867903	-0,073088357	-0,062125103	-0,304279852	-0,325279852	**-1,585279852**
chrI	28865	28996	131	0,769381358	0,763409133	0,501948154	0,004883318	0,00415082	0,164044397	0,255044397	**-1,004955603**
chrI	31670	31815	145	0,572761677	0,478057176	0,54506224	0,113195553	0,09621622	-0,115405277	-0,122405277	**-1,382405277**
chrI	32825	32968	143	0,131079787	0,08894087	0,595761274	0,242390685	0,206032083	0,022965057	0,029965057	**-1,230034943**
chrI	38266	38455	189	0,447381012	0,70040935	0,389775892	-0,279903186	-0,237917708	-0,058713958	-0,373713958	**-1,633713958**
chrI	38266	38455	189	0,447381012	0,70040935	0,389775892	-0,279903186	-0,237917708	-0,058713958	-0,373713958	**-1,633713958**
chrI	39356	39504	148	0,068389454	0,040764565	0,626540868	0,322705529	0,274299699	0,00693267	-0,02106733	**-1,28106733**
chrI	44014	44169	155	2,396480451	2,01969892	0,54265922	0,107135403	0,091065093	0,007886483	-0,069113517	**-1,329113517**
chrI	48679	48833	154	1,493169746	1,3155837	0,531612964	0,079325064	0,067426304	-0,034356893	-0,104356893	**-1,364356893**
chrI	52107	52211	104	0,071239015	0,314998914	0,184443343	-0,898560728	-0,763776618	-0,017609421	0,262390579	**-0,997609421**
chrI	52108	52200	92	0,039893848	0,314998914	0,112410994	-1,213805546	-1,031734714	-0,298494905	0,065505095	**-1,194494905**
chrI	52108	52202	94	0,028495606	0,188999349	0,131017319	-1,121595095	-0,953355831	-0,205050422	0,144949578	**-1,115050422**
chrI	52108	52203	95	0,042743409	0,326116523	0,115879783	-1,195838559	-1,016462775	-0,286469923	0,056530077	**-1,203469923**
chrI	52108	52204	96	0,039893848	0,270528479	0,128514751	-1,133440052	-0,963424045	-0,250333328	0,085666672	**-1,174333328**
chrI	52108	52205	97	0,042743409	0,203822827	0,173354671	-0,940991251	-0,799842563	-0,077989827	0,251010173	**-1,008989827**
chrI	52108	52206	98	0,031345166	0,240881523	0,11514362	-1,199619268	-1,019676378	-0,294433255	0,027566745	**-1,232433255**
chrI	52108	52207	99	0,037044288	0,555880437	0,062477219	-1,53430581	-1,304159938	-0,588375011	-0,273375011	**-1,533375011**
chrI	52108	52208	100	0,085486818	0,326116523	0,207692235	-0,814454777	-0,692286561	0,069901083	0,377901083	**-0,882098917**
chrI	52108	52209	101	0,136778908	0,874585221	0,135242001	-1,101948639	-0,936656343	-0,168128482	0,132871518	**-1,127128482**
chrI	52108	52210	102	0,037044288	0,363175219	0,092559925	-1,325154688	-1,126381485	-0,354587152	-0,060587152	**-1,320587152**
chrI	52108	52211	103	35,59956037	163,3992015	0,178893376	-0,919590578	-0,781651991	-0,03332145	0,25367855	**-1,00632145**
chrI	52112	52211	99	0,042743409	0,389116306	0,098975217	-1,287412835	-1,09430091	-0,367463337	-0,052463337	**-1,312463337**
chrI	54178	54332	154	0,29065518	0,181587609	0,61547828	0,293626349	0,249582396	0,150968964	0,080968964	**-1,179031036**
chrI	56067	56218	151	1,484621064	1,226642831	0,547575272	0,119537596	0,101606956	-0,082434877	-0,131434877	**-1,391434877**
chrI	57573	57723	150	0,262159574	0,133411305	0,662737294	0,419945303	0,356953508	0,125523607	0,083523607	**-1,176476393**
chrI	57573	57723	150	0,262159574	0,133411305	0,662737294	0,419945303	0,356953508	0,125523607	0,083523607	**-1,176476393**
chrI	57985	58142	157	0,108283302	0,133411305	0,448017039	-0,130672891	-0,111071958	-0,140896053	-0,231896053	**-1,491896053**
chrI	58357	58529	172	0,225115286	0,285351958	0,440998495	-0,148438156	-0,126172432	0,060582422	-0,135417578	**-1,395417578**
chrI	59835	59985	150	0,267858695	0,26682261	0,500968881	0,002428627	0,002064333	-0,223981569	-0,265981569	**-1,525981569**
chrI	60956	61114	158	6,844644522	4,895453713	0,583014246	0,209610726	0,178169117	0,156739962	0,058739962	**-1,201260038**
chrI	61139	61253	114	0,253610892	1,775111528	0,125010149	-1,15030008	-0,977755068	-0,464954291	-0,254954291	**-1,514954291**
chrI	62731	62859	128	2,034586257	3,053636533	0,399861865	-0,253704663	-0,215648964	0,029545139	0,141545139	**-1,118454861**
chrI	73815	73966	151	0,142478029	0,133411305	0,516431814	0,041200102	0,035020087	-0,147101359	-0,196101359	**-1,456101359**
chrI	75929	76089	160	0,151026711	0,037058696	0,802968787	0,852273293	0,724432299	0,736809571	0,624809571	**-0,635190429**
chrI	77820	77969	149	0,353345512	0,237175653	0,598362147	0,249109985	0,211743487	-0,034929447	-0,069929447	**-1,329929447**
chrI	78867	79013	146	0,082637257	0,077823261	0,515000562	0,037609696	0,031968242	-0,202014857	-0,216014857	**-1,476014857**
chrI	79075	79223	148	0,270708255	0,222352175	0,549036667	0,123227859	0,104743681	-0,157857802	-0,185857802	**-1,445857802**
chrI	79820	79919	99	0,051292091	0,185293479	0,216801433	-0,783041289	-0,665585096	0,076654568	0,391654568	**-0,868345432**
chrI	80712	80877	165	0,074088575	0,051882174	0,588141101	0,222765786	0,189350918	0,304193553	0,157193553	**-1,102806447**
chrI	83877	84031	154	0,786478721	0,35205761	0,690780522	0,498063968	0,423354373	0,315267959	0,245267959	**-1,014732041**
chrI	87561	87716	155	0,102584181	0,140823044	0,421450846	-0,198183249	-0,168455762	-0,231954094	-0,308954094	**-1,568954094**

Chrom	Start	End	Length	Section A	Section B	A/A+B	Z-score	Z * 0.85	Phase correction	Length correction	ΔLKnuc
chrI	88852	89013	161	1,46467414	1,526818266	0,48961319	-0,026038814	-0,022132992	0,029070756	-0,089929244	**-1,349929244**
chrI	88852	89013	161	1,46467414	1,526818266	0,48961319	-0,026038814	-0,022132992	0,029070756	-0,089929244	**-1,349929244**
chrI	88889	89053	164	0,111132863	0,226058044	0,3295844	-0,441061051	-0,374901893	-0,31832009	-0,45832009	**-1,71832009**
chrI	91036	91130	94	0,028495606	0,055588044	0,338895921	-0,415478239	-0,353156503	0,380938839	0,730938839	**-0,529061161**
chrI	94271	94416	145	0,253610892	0,129705435	0,661622983	0,41689661	0,354362118	0,139529776	0,132529776	**-1,127470224**
chrI	97984	98151	167	0,156725832	0,203822827	0,434687048	-0,164453569	-0,139785534	-0,018823481	-0,179823481	**-1,439823481**
chrI	100299	100401	102	0,071239015	0,037058696	0,657807209	0,406485945	0,345513053	1,111125764	1,405125764	**0,145125764**
chrI	100430	100592	162	0,34194727	0,17417587	0,662530399	0,419378955	0,356472112	0,413429344	0,287429344	**-0,972570656**
chrI	100744	100901	157	0,219416165	0,151940653	0,590849971	0,229731961	0,195272167	0,173217738	0,082217738	**-1,177782262**
chrI	100903	101028	125	0,082637257	0,074117392	0,527175798	0,068172392	0,057946533	0,445541957	0,578541957	**-0,681458043**
chrI	101774	101913	139	0,424584527	0,363175219	0,538977181	0,097857261	0,083178671	-0,043887199	-0,008887199	**-1,268887199**
chrI	102759	102918	159	0,319150785	0,34835174	0,478126708	-0,054855711	-0,046627354	-0,039046068	-0,144046068	**-1,404046068**
chrI	110352	110498	146	4,00648218	2,497756096	0,615980229	0,294940225	0,250699191	0,011903264	-0,002096736	**-1,262096736**
chrI	110959	111107	148	0,324849907	0,229763914	0,585722704	0,216555726	0,184072367	-0,077708219	-0,105708219	**-1,365708219**
chrI	111829	111987	158	0,034194727	0,214940436	0,137253717	-1,092741227	-0,928830043	-0,946517965	-1,044517965	**-2,304517965**
chrI	111829	111989	160	7,66816753	5,996096978	0,561184067	0,153971933	0,130876143	0,151324568	0,039324568	**-1,220675432**
chrI	111835	111987	152	4,339880769	3,146283272	0,579720234	0,201177822	0,171001149	-0,005773661	-0,061773661	**-1,321773661**
chrI	111835	111989	154	0,091185939	0,218646305	0,294307453	-0,540844298	-0,459717653	-0,573586461	-0,643586461	**-1,903586461**
chrI	113198	113345	147	0,09973462	0,066705652	0,599221683	0,251333038	0,213633083	-0,032354286	-0,053354286	**-1,313354286**
chrI	113198	113345	147	0,09973462	0,066705652	0,599221683	0,251333038	0,213633083	-0,032354286	-0,053354286	**-1,313354286**
chrI	113272	113431	159	1,877860425	1,663935441	0,530200072	0,075772802	0,064406881	0,069150553	-0,035849447	**-1,295849447**
chrI	114916	115064	148	0,179522317	0,062999783	0,740230754	0,644056972	0,547448426	0,287356404	0,259356404	**-1,000643596**
chrI	116310	116474	164	2,185612968	1,697288267	0,562881422	0,158278745	0,134536933	0,213769775	0,073769775	**-1,186230225**
chrI	119258	119408	150	2,34518836	0,229763914	0,910769642	1,345509621	1,143683178	0,925706228	0,883706228	**-0,376293772**
chrI	121555	121709	154	0,555664314	0,396528045	0,583563089	0,211017235	0,17936465	0,082446566	0,012446566	**-1,247553434**
chrI	128610	128759	149	0,116831984	0,081529131	0,588986325	0,224938202	0,191197471	-0,043333574	-0,078333574	**-1,338333574**
chrI	128749	128913	164	0,752283994	0,956114351	0,440344605	-0,150095577	-0,127581241	-0,02014932	-0,16014932	**-1,42014932**
chrI	132160	132339	179	0,843469933	1,463818484	0,365567619	-0,343615804	-0,292073433	-0,120001105	-0,365001105	**-1,625001105**
chrI	132322	132449	127	0,045592969	0,074117392	0,380860679	-0,303221116	-0,257737948	0,01200283	0,13100283	**-1,12899717**
chrI	133311	133456	145	4,274340875	2,90169588	0,595640884	0,242079926	0,205767937	-0,012779418	-0,019779418	**-1,279779418**
chrI	133444	133595	151	0,775080479	0,559586306	0,580729578	0,203760266	0,173196226	-0,003572395	-0,052572395	**-1,312572395**
chrI	134936	135082	146	0,612655525	0,363175219	0,627829702	0,326110708	0,277194102	0,040400686	0,026400686	**-1,233599314**
chrI	139478	139611	133	13,67504124	8,486441335	0,617063466	0,297777395	0,253110785	0,245477013	0,322477013	**-0,937522987**
chrI	144452	144595	143	0,302053422	0,192705218	0,610506614	0,280639687	0,238543734	0,049475346	0,056475346	**-1,203524654**
chrI	144452	144595	143	0,302053422	0,192705218	0,610506614	0,280639687	0,238543734	0,049475346	0,056475346	**-1,203524654**
chrI	145995	146131	136	0,507221784	0,226058044	0,691716538	0,500721804	0,425613534	0,379375359	0,435375359	**-0,824624641**
chrI	145995	146131	136	0,507221784	0,226058044	0,691716538	0,500721804	0,425613534	0,379375359	0,435375359	**-0,824624641**
chrI	157171	157325	154	0,09973462	0,100058479	0,499189516	-0,002031583	-0,001726846	-0,090376368	-0,160376368	**-1,420376368**
chrI	158225	158386	161	0,085486818	0,062999783	0,575720754	0,190958009	0,162314307	0,216969523	0,097969523	**-1,162030477**
chrI	158476	158631	155	0,265009134	0,307587175	0,462820193	-0,093331276	-0,079331585	-0,129764872	-0,206764872	**-1,466764872**
chrI	158476	158631	155	0,265009134	0,307587175	0,462820193	-0,093331276	-0,079331585	-0,129764872	-0,206764872	**-1,466764872**
chrI	159246	159353	107	0,48727486	4,006045014	0,108444285	-1,234843997	-1,049617397	-0,32764883	-0,06864883	**-1,32864883**
chrI	161173	161287	114	0,068389454	0,389116306	0,149483263	-1,038652184	-0,882854356	-0,39116562	-0,18116562	**-1,44116562**
chrI	163265	163422	157	0,059840772	0,059293913	0,502295129	0,005753068	0,004890107	-0,009421984	-0,100421984	**-1,360421984**
chrI	164304	164458	154	0,04844253	0,048176305	0,50137771	0,003453413	0,002935401	-0,076236959	-0,146236959	**-1,406236959**
chrI	164304	164458	154	0,04844253	0,048176305	0,50137771	0,003453413	0,002935401	-0,076236959	-0,146236959	**-1,406236959**
chrI	164952	165092	140	0,159575393	0,122293696	0,566132999	0,166537466	0,141556846	-0,007994795	0,020005205	**-1,239994795**
chrI	164958	165092	134	0,136778908	0,137117174	0,499382491	-0,001547865	-0,001315685	-0,033023256	0,036976744	**-1,223023256**
chrI	165370	165525	155	0,102584181	0,081529131	0,557179598	0,143822286	0,122248943	0,089586931	0,012586931	**-1,247413069**
chrI	165501	165609	108	0,045592969	0,155646522	0,226560746	-0,750221201	-0,637688021	0,033894185	0,285894185	**-0,974105815**
chrI	165531	165649	118	1,704037229	4,873218496	0,259080276	-0,646183457	-0,549255938	-0,08731138	0,09468862	**-1,16531138**
chrI	165650	165799	149	0,085486818	0,044470435	0,657807209	0,406485945	0,345513053	0,110095556	0,075095556	**-1,184904444**
chrI	170185	170316	131	0,367593315	0,333528262	0,524293257	0,060931847	0,05179207	0,177713203	0,268713203	**-0,991286797**
chrI	170746	170892	146	0,296354301	0,251999131	0,540443961	0,101552255	0,086319417	-0,145404324	-0,159404324	**-1,419404324**
chrI	173251	173422	171	0,170973635	0,159352392	0,517590565	0,044107306	0,03749121	0,201163341	0,012163341	**-1,247836659**
chrI	174403	174559	156	1,020142689	0,837526525	0,54915196	0,123519062	0,104991202	0,075570974	-0,008429026	**-1,268429026**
chrI	177062	177157	95	0,723788388	3,016577837	0,193507359	-0,865043858	-0,735287279	-0,008986621	0,334013379	**-0,925986621**
chrI	178178	178314	136	0,265009134	0,218646305	0,547929606	0,120432196	0,102367367	0,046707401	0,102707401	**-1,157292599**
chrI	181632	181781	149	0,119681545	0,144528914	0,452978074	-0,118140734	-0,100419624	-0,3358092	-0,3708092	**-1,6308092**
chrI	184868	185010	142	1,048638295	0,685585872	0,604672865	0,265461103	0,225641937	0,065722461	0,079722461	**-1,180277539**
chrI	188940	189093	153	0,085486818	0,051882174	0,622315244	0,311567137	0,264832067	0,11250467	0,04950467	**-1,21049533**
chrI	189954	190061	107	1,894957788	4,335867407	0,304126296	-0,512569358	-0,435683954	0,261864949	0,520864949	**-0,739135051**
chrI	189954	190062	108	0,04844253	0,100058479	0,326210107	-0,450402539	-0,382842158	0,269771927	0,521771927	**-0,738228073**
chrI	189954	190063	109	0,045592969	0,107470218	0,297870247	-0,530535801	-0,450955431	0,164258858	0,409258858	**-0,850741142**
chrI	189954	190077	123	1,85506394	5,666274586	0,246640134	-0,685100876	-0,582335745	-0,172620828	-0,025620828	**-1,285620828**
chrI	191415	191573	158	0,062690333	0,085235	0,423797138	-0,192188854	-0,163360526	-0,171273336	-0,269273336	**-1,529273336**
chrI	191722	191874	152	0,153876272	0,096352609	0,614942093	0,292223409	0,248389898	0,078993697	0,022993697	**-1,237006303**
chrI	192889	193041	152	1,062886098	1,693582397	0,385597042	-0,290813338	-0,247191337	-0,407783132	-0,463783132	**-1,723783132**
chrI	193467	193622	155	2,883755311	2,575579357	0,52822468	0,070807907	0,060186721	0,026701459	-0,050298541	**-1,310298541**
chrI	194126	194279	153	2,658640024	2,212404138	0,545814952	0,115069422	0,097809009	-0,02265981	-0,08565981	**-1,34565981**
chrI	194141	194279	138	0,512920905	0,259410871	0,664119905	0,423733486	0,360173463	0,26050144	0,30250144	**-0,95749856**
chrI	194290	194442	152	0,125380666	0,055588044	0,692830634	0,503889924	0,428306435	0,272098034	0,216098034	**-1,043901966**
chrI	204527	204675	148	0,327699467	0,233469783	0,583958346	0,212030411	0,180225849	-0,078287583	-0,106287583	**-1,366287583**
chrI	204662	204810	148	0,424584527	0,226058044	0,652561861	0,392246244	0,333409307	0,083481064	0,055481064	**-1,204518936**
chrI	204797	204945	148	0,398938482	0,207528696	0,657807209	0,406485945	0,345513053	0,093736202	0,065736202	**-1,194263798**
chrI	205040	205170	130	0,125380666	0,17417587	0,418554265	-0,205593405	-0,174754394	0,002376662	0,100376662	**-1,159623338**
chrI	205573	205699	126	0,102584181	0,081529131	0,557179598	0,143822286	0,122248943	0,406093932	0,532093932	**-0,727906068**
chrI	205573	205705	132	0,039893848	0,051882174	0,434687048	-0,164453569	-0,139785534	-0,078685071	0,005314929	**-1,254685071**
chrI	205573	205712	139	2,778321569	1,448995005	0,657230543	0,404916468	0,344178997	0,212715496	0,247715496	**-1,012284504**
chrI	205580	205712	132	0,330549028	0,248293262	0,571051967	0,179053024	0,152195071	0,17803092	0,26203092	**-0,99796908**
chrI	206390	206543	153	0,133929347	0,289057827	0,316627443	-0,477150599	-0,40557801	-0,513820478	-0,576820478	**-1,836820478**
chrI	207406	207531	125	10,97365781	12,16636983	0,47422838	-0,064644869	-0,054948139	0,310469572	0,443469572	**-0,816530428**
chrI	215442	215555	113	0,085486818	1,007996525	0,078178436	-1,417431283	-1,20481659	-0,693345291	-0,476345291	**-1,736345291**
chrI	217878	218015	137	5,109262126	3,894868927	0,567435336	0,169848443	0,144371176	0,048411892	0,097411892	**-1,162588108**
chrI	217916	218076	160	0,031345166	0,040764565	0,434687048	-0,164453569	-0,139785534	-0,107307354	-0,219307354	**-1,479307354**
chrI	218711	218856	145	2,473418586	1,248878048	0,664487232	0,424740939	0,361029798	0,130349978	0,123349978	**-1,136650022**
chrI	218738	218886	148	0,404637603	0,218646305	0,649202711	0,383168843	0,325693517	0,072440063	0,044440063	**-1,215559937**
chrI	222387	222554	167	0,435982769	0,489174784	0,471252456	-0,072121883	-0,0613036	0,071391448	-0,089608552	**-1,349608552**
chrI	222751	222855	104	0,156725832	0,663350655	0,191111237	-0,873808639	-0,742737344	-0,000770953	0,279229047	**-0,980770953**
chrI	223257	223420	163	0,14817715	0,196411088	0,430012212	-0,176343075	-0,149891614	-0,090544312	-0,223544312	**-1,483544312**
chrI	224065	224173	108	0,04844253	0,318704784	0,131943032	-1,117253234	-0,949665249	-0,310042122	-0,058042122	**-1,318042122**
chrI	224877	224997	120	0,208017923	0,752291524	0,216615512	-0,783674679	-0,666123477	-0,234308076	-0,066308076	**-1,326308076**
chrI	226540	226687	147	1,213912809	0,655938915	0,649202711	0,383168843	0,325693517	0,076075897	0,055075897	**-1,204924103**
chrI	229861	230005	144	4,63908463	2,575579357	0,643007718	0,366509985	0,311533487	0,11349443	0,11349443	**-1,14650557**
chrI	230119	230218	99	0,119681545	0,140823044	0,459422021	-0,101889932	-0,086606442	0,661749047	0,976749047	**-0,283250953**
chrI	230124	230218	94	0,387540239	0,34835174	0,526626529	0,066792441	0,056773575	0,797592648	1,147592648	**-0,112407352**

Chrom	Start	End	Length	Section A	Section B	A/A+B	Z-score	Z * 0.85	Phase correction	Length correction	ΔLKnuc
chrII	163	311	148	0,059840772	0,055588044	0,518421434	0,046192108	0,039263292	-0,051618443	-0,079618443	-1,339618443
chrII	526	677	151	0,564212996	0,318704784	0,639032318	0,355873419	0,302492406	0,186299094	0,137299094	-1,122700906
chrII	1089	1240	151	0,823523009	0,492880654	0,625585474	0,320183734	0,272156174	0,155357044	0,106357044	-1,153642956
chrII	1727	1858	131	2,162816483	2,01969892	0,51710903	0,042899133	0,036464263	0,206368447	0,297368447	-0,962631553
chrII	1727	1870	143	0,359044634	0,292763697	0,550843886	0,12779371	0,108624653	0,044015112	0,051015112	-1,208984888
chrII	1727	1914	187	0,14532759	0,111176087	0,566571175	0,16765125	0,142503563	0,537148593	0,236148593	-1,023851407
chrII	1931	2076	145	0,592708601	0,674468263	0,467739443	-0,080953558	-0,068810524	-0,131920977	-0,138920977	-1,398920977
chrII	1936	2043	107	0,119681545	0,166764131	0,417815854	-0,20748423	-0,176361595	0,366147208	0,625147208	-0,634852792
chrII	1937	2034	97	0,188070999	0,244587392	0,434687048	-0,164453569	-0,139785534	0,168480379	0,497480379	-0,762519621
chrII	1937	2042	105	0,031345166	0,233469783	0,118366302	-1,183193156	-1,005714183	-0,631967553	-0,358967553	-1,618967553
chrII	1937	2046	109	0,142478029	0,237175653	0,375284202	-0,317889966	-0,270206471	0,334933722	0,579933722	-0,680066278
chrII	1937	2051	114	0,042743409	0,166764131	0,204018475	-0,827353109	-0,703250143	-0,092580445	0,117419555	-1,142580445
chrII	1937	2054	117	0,056991212	0,285351958	0,166473927	-0,968193274	-0,822964283	-0,211598983	-0,022598983	-1,282598983
chrII	1937	2057	120	0,042743409	0,151940653	0,219552687	-0,773704813	-0,657649091	-0,037549176	0,130450824	-1,129549176
chrII	1937	2062	125	0,065539893	0,774526742	0,078017494	-1,418533767	-1,205753702	-0,711064879	-0,578064879	-1,838064879
chrII	1937	2071	134	0,059840772	0,055588044	0,518421434	0,046192108	0,039263292	0,105040379	0,175040379	-1,084959621
chrII	1937	2072	135	0,054141651	0,070411522	0,434687048	-0,164453569	-0,139785534	-0,029959369	0,033040631	-1,226959369
chrII	1937	2073	136	0,042743409	0,200116957	0,175899936	-0,930717198	-0,791109618	-0,753126123	-0,697126123	-1,957126123
chrII	1937	2075	138	0,045592969	0,096352609	0,321200349	-0,464344846	-0,394693119	-0,378758786	-0,336758786	-1,596758786
chrII	1937	2076	139	34,02375337	28,62413663	0,543094961	0,108233996	0,091998897	0,119744817	0,154744817	-1,105255183
chrII	1937	2082	145	0,031345166	0,511410002	0,057751945	-1,573928894	-1,337839559	-1,412580974	-1,419580974	-2,679580974
chrII	1937	2086	149	0,287805619	0,418763262	0,407328467	-0,234422577	-0,19925919	-0,33674152	-0,37174152	-1,63174152
chrII	1938	2076	138	0,102584181	0,107470218	0,488369592	-0,029157241	-0,024783655	-0,006450881	0,035549119	-1,224450881
chrII	1939	2076	137	0,056991212	0,103764348	0,354520937	-0,373143192	-0,317171714	-0,307387315	-0,258387315	-1,518387315
chrII	1940	2076	136	0,071239015	0,100058479	0,415878909	-0,212447646	-0,180580499	-0,155600303	-0,099600303	-1,359600303
chrII	1941	2076	135	0,071239015	0,062999783	0,530688713	0,07700122	0,065451037	0,167857361	0,230857361	-1,029142639
chrII	1942	2076	134	0,054141651	0,185293479	0,22612242	-0,751677806	-0,638926135	-0,536837644	-0,466837644	-1,726837644
chrII	1944	2076	132	0,692443222	0,937585003	0,424804437	-0,1896175	-0,161174875	-0,005981789	0,078018211	-1,181981789
chrII	1947	2076	129	0,031345166	0,125999566	0,19921332	-0,84443452	-0,717769342	-0,362897595	-0,257897595	-1,517897595
chrII	1948	2076	128	0,028495606	0,085235	0,250553539	-0,67274886	-0,571836531	-0,207921231	-0,095921231	-1,355921231
chrII	1952	2076	124	0,042743409	0,08894087	0,324590066	-0,454901459	-0,386666241	0,137892569	0,277892569	-0,982107431
chrII	1953	2076	123	0,076938136	0,081529131	0,485514374	-0,036318061	-0,030870352	0,528799631	0,675799631	-0,584200369
chrII	1964	2076	112	0,09688506	0,233469783	0,293275736	-0,543840157	-0,462264134	0,138170855	0,362170855	-0,897829145
chrII	1966	2076	110	0,102584181	0,166764131	0,380860679	-0,303221116	-0,257737948	0,358155933	0,596155933	-0,663844067
chrII	2124	2286	162	0,037044288	0,048176305	0,434687048	-0,164453569	-0,139785534	-0,103886698	-0,229886698	-1,489886698
chrII	2124	2286	162	0,037044288	0,048176305	0,434687048	-0,164453569	-0,139785534	-0,103886698	-0,229886698	-1,489886698
chrII	2191	2286	95	0,039893848	0,062999783	0,387719315	-0,285268256	-0,242478018	0,053315126	0,396315126	-0,863684874
chrII	2267	2374	107	0,071239015	0,133411305	0,348101165	-0,390452017	-0,331884215	0,215051727	0,474051727	-0,785948273
chrII	2267	2414	147	0,079787696	0,114881957	0,409862015	-0,227899939	-0,193714948	-0,278119201	-0,299119201	-1,559119201
chrII	2522	2620	98	0,031345166	0,037058696	0,458236793	-0,104876779	-0,089145262	0,218747595	0,540747595	-0,719252405
chrII	2522	2673	151	0,795027403	0,415057393	0,657001398	0,404293094	0,34364913	0,232079266	0,183079266	-1,076920734
chrII	3354	3498	144	0,128230226	0,059293913	0,683806504	0,478369846	0,406614369	0,307662419	0,307662419	-0,952337581
chrII	3419	3571	152	0,116831984	0,085235	0,578184429	0,197250975	0,167663329	0,065281468	0,009281468	-1,250718532
chrII	3701	3844	143	0,373292436	0,448410219	0,454291384	-0,114826344	-0,097602392	-0,171193058	-0,164193058	-1,424193058
chrII	3992	4129	137	0,444531451	0,474351306	0,483773852	-0,040684142	-0,03458152	-0,043485891	0,005514109	-1,254485891
chrII	7980	8135	155	0,159575393	0,074117392	0,682842618	0,475662609	0,404313218	0,319117223	0,242117223	-1,017882777
chrII	10712	10874	162	0,065539893	0,103764348	0,387113121	-0,28685122	-0,243823537	-0,207400301	-0,333400301	-1,593400301
chrII	20981	21152	171	0,153876272	0,452116089	0,253924441	-0,662190904	-0,562862269	-0,266983406	-0,455983406	-1,715983406
chrII	21442	21596	154	0,091185939	0,118587827	0,434687048	-0,164453569	-0,139785534	-0,206203893	-0,276203893	-1,536203893
chrII	24248	24399	151	0,267858695	0,129705435	0,673749653	0,450290909	0,382747273	0,277943929	0,228943929	-1,031056071
chrII	24299	24437	138	0,703841464	0,659644785	0,516207233	0,04063669	0,034541187	0,059825685	0,101825685	-1,158174315
chrII	25280	25416	136	0,185221438	0,170470001	0,520736284	0,052001583	0,044201345	0,060620037	0,116620037	-1,143379963
chrII	26126	26261	135	0,498673102	0,459527828	0,520426444	0,051223893	0,043540309	0,142174601	0,205174601	-1,054825399
chrII	30232	30334	102	0,139628469	0,140823044	0,497870264	-0,005338482	-0,00453771	0,314168167	0,608168167	-0,651831833
chrII	31655	31803	148	0,09688506	0,055588044	0,635423938	0,346253615	0,294315573	0,195989109	0,167989109	-1,092010891
chrII	32970	33121	151	0,270708255	0,148234783	0,646169599	0,37499955	0,318749618	0,205206515	0,156206515	-1,103793485
chrII	35988	36136	148	0,162424953	0,122293696	0,570475287	0,177584338	0,150946687	0,043798437	0,015798437	-1,244201563
chrII	48768	48912	144	0,222265726	0,17788174	0,555459536	0,139467258	0,118547169	0,020446528	0,020446528	-1,239553472
chrII	54394	54539	145	0,151026711	0,077823261	0,659937642	0,412292949	0,350449007	0,280783299	0,273783299	-0,986216701
chrII	54394	54539	145	0,151026711	0,077823261	0,659937642	0,412292949	0,350449007	0,280783299	0,273783299	-0,986216701
chrII	54551	54716	165	0,239363089	0,185293479	0,56366275	0,160262244	0,136222907	0,267326947	0,120326947	-1,139673053
chrII	54551	54716	165	0,239363089	0,185293479	0,56366275	0,160262244	0,136222907	0,267326947	0,120326947	-1,139673053
chrII	62389	62546	157	0,370442876	0,374292827	0,497415223	-0,006479121	-0,005507253	-0,031861839	-0,122861839	-1,382861839
chrII	62885	63033	148	0,159575393	0,125999566	0,558786364	0,147892925	0,125708986	0,024882618	-0,003117382	-1,263117382
chrII	64104	64264	160	0,056991212	0,051882174	0,523463207	0,058847485	0,050020363	0,053194984	-0,058805016	-1,318805016
chrII	64653	64794	141	0,105433742	0,133411305	0,441431561	-0,147340685	-0,125239582	-0,159025619	-0,138025619	-1,398025619
chrII	65899	66050	151	0,190920559	0,218646305	0,466152357	-0,084945506	-0,07220368	-0,186715774	-0,235715774	-1,495715774
chrII	67731	67881	150	0,370442876	0,326116523	0,531818071	0,079840821	0,067864697	-0,07433213	-0,11633213	-1,37633213
chrII	69118	69263	145	1,057186976	0,752291524	0,584249537	0,212776972	0,180860426	0,107846031	0,100846031	-1,159153969
chrII	79393	79540	147	0,074088575	0,144528914	0,338895921	-0,415478239	-0,353156503	-0,438609071	-0,459609071	-1,719609071
chrII	81527	81678	151	0,256460453	0,233469783	0,523463207	0,058847485	0,050020363	-0,065821732	-0,114821732	-1,374821732
chrII	81527	81678	151	0,256460453	0,233469783	0,523463207	0,058847485	0,050020363	-0,065821732	-0,114821732	-1,374821732
chrII	86475	86605	130	1,094231264	1,382289353	0,441842178	-0,146300265	-0,124355225	0,146709204	0,244709204	-1,015290796
chrII	87052	87211	159	0,054141651	0,085235	0,388455674	-0,283346352	-0,2408444	-0,248149953	-0,353149953	-1,613149953
chrII	94866	95023	157	0,076938136	0,096352609	0,443982948	-0,140878539	-0,119746758	-0,145880406	-0,236880406	-1,496880406
chrII	97063	97217	154	0,823523009	0,655938915	0,556636839	0,142447782	0,121080615	0,056095479	-0,013904521	-1,273904521
chrII	97108	97247	139	0,122531105	0,070411522	0,635064977	0,345298405	0,293503645	0,312172713	0,347172713	-0,912827287
chrII	98068	98229	161	0,14532759	0,17788174	0,449639217	-0,126572917	-0,10758698	-0,084100968	-0,203100968	-1,463100968
chrII	98405	98563	158	0,644000692	0,689291742	0,483015335	-0,04258711	-0,036199044	-0,042718636	-0,140718636	-1,400718636
chrII	102188	102336	148	0,04844253	0,059293913	0,449639217	-0,126572917	-0,10758698	-0,19655441	-0,22455441	-1,48455441
chrII	105450	105577	127	1,262355338	2,308756747	0,35349082	-0,375912914	-0,319525977	0,072226215	0,191226215	-1,068773785
chrII	105532	105684	152	0,133929347	0,140823044	0,487454711	-0,031451562	-0,026733828	-0,12757567	-0,18357567	-1,44357567
chrII	105532	105684	152	0,133929347	0,140823044	0,487454711	-0,031451562	-0,026733828	-0,12757567	-0,18357567	-1,44357567
chrII	105754	105910	156	0,125380666	0,040764565	0,754644987	0,68917996	0,585802966	0,528953837	0,444953837	-0,815046163
chrII	106088	106233	145	0,444531451	0,292763697	0,602921981	0,260917618	0,221779975	0,155157764	0,148157764	-1,111842236
chrII	112631	112737	106	1,820869213	5,54027502	0,247362252	-0,682813808	-0,580391737	-0,144361425	0,121638575	-1,138361425
chrII	112631	112749	118	1,877860425	7,263504374	0,205424514	-0,82240044	-0,699040374	-0,087103148	0,094896852	-1,165103148
chrII	117607	117737	130	1,225311051	1,211819352	0,502767948	0,006938271	0,005897531	0,265325358	0,363325358	-0,896674642
chrII	118185	118336	151	0,230814407	0,196411088	0,540263655	0,101097969	0,085933273	-0,02133991	-0,07033991	-1,33033991
chrII	118430	118588	158	0,102584181	0,070411522	0,592986873	0,235235114	0,199949847	0,162333021	0,064333021	-1,195666979
chrII	118430	118588	158	0,102584181	0,070411522	0,592986873	0,235235114	0,199949847	0,162333021	0,064333021	-1,195666979
chrII	118604	118737	133	11,30990596	7,600738506	0,59807089	0,248356974	0,211103428	0,317086836	0,394086836	-0,865913164
chrII	118662	118820	158	1,250957096	1,445289136	0,463962483	-0,090455864	-0,076887484	-0,10819699	-0,20619699	-1,46619699
chrII	120690	120830	140	0,256460453	0,207528696	0,552729419	0,13256026	0,112676221	0,12126134	0,14926134	-1,11073866
chrII	130330	130481	151	2,789719811	2,471815009	0,530210273	0,075798443	0,064428677	-0,045184127	-0,094184127	-1,354184127
chrII	133172	133320	148	0,054141651	0,081529131	0,399066404	-0,255764342	-0,217399691	-0,320611205	-0,348611205	-1,608611205

Chrom	Start	End	Length	Section A	Section B	A/A+B	Z-score	Z * 0.85	Phase correction	Length correction	ΔLKnuc
chrII	134044	134208	164	0,606956404	0,629997828	0,490686226	-0,02334829	-0,019846047	0,056070417	-0,083929583	**-1,343929583**
chrII	134044	134208	164	0,606956404	0,629997828	0,490686226	-0,02334829	-0,019846047	0,056070417	-0,083929583	**-1,343929583**
chrII	146223	146376	153	0,108283302	0,096352609	0,529151025	0,07313593	0,062165541	0,001766017	-0,061233983	**-1,321233983**
chrII	147729	147892	163	0,159575393	0,289057827	0,355692324	-0,369997104	-0,314497538	-0,25596304	-0,38896304	**-1,64896304**
chrII	157306	157457	151	0,122531105	0,107470218	0,532740871	0,082161538	0,069837308	-0,03377469	-0,08277469	**-1,34277469**
chrII	158794	158975	181	0,353345512	0,70411522	0,33414528	-0,428495293	-0,364220999	0,029190346	-0,229809654	**-1,489809654**
chrII	159579	159667	88	0,039893848	0,044470435	0,472876041	-0,06804215	-0,057835827	0,195231863	0,587231863	**-0,672768137**
chrII	159579	159734	155	4,015030862	3,965280449	0,503117072	0,007813421	0,006641408	-0,07816889	-0,15516889	**-1,41516889**
chrII	161150	161286	136	0,729487509	0,863467612	0,457946052	-0,105609604	-0,089768163	-0,073479554	-0,017479554	**-1,277479554**
chrII	161650	161786	136	2,718480797	2,664520227	0,505012127	0,01256387	0,010679289	0,02354348	0,07954348	**-1,18045652**
chrII	164812	164963	151	0,153876272	0,111176087	0,58055047	0,203301913	0,172806626	0,071079698	0,022079698	**-1,237920302**
chrII	169155	169305	150	0,695292782	0,537351089	0,564066231	0,161286776	0,137093759	-0,00391113	-0,04591113	**-1,30591113**
chrII	171571	171720	149	0,806425645	0,489174784	0,622433913	0,311879406	0,265097495	0,133053285	0,098053285	**-1,161946715**
chrII	171661	171810	149	0,63260245	0,444704349	0,58720733	0,220367006	0,187311955	0,054779123	0,019779123	**-1,240220877**
chrII	171661	171810	149	0,63260245	0,444704349	0,58720733	0,220367006	0,187311955	0,054779123	0,019779123	**-1,240220877**
chrII	174305	174447	142	0,273557816	0,203822827	0,573039188	0,18411706	0,156499501	0,154470237	0,168470237	**-1,091529763**
chrII	186048	186192	144	0,558513874	0,326116523	0,631352796	0,335438395	0,285122636	0,197541935	0,197541935	**-1,062458065**
chrII	187191	187351	160	0,108283302	0,17788174	0,378394585	-0,309699913	-0,263244926	-0,255421457	-0,367421457	**-1,627421457**
chrII	188175	188332	157	0,424584527	0,392822175	0,519428732	0,048719876	0,041411895	0,007410682	-0,083589318	**-1,343589318**
chrII	189372	189539	167	0,131079787	0,218646305	0,374806999	-0,319148382	-0,271276125	-0,099501284	-0,260501284	**-1,520501284**
chrII	192490	192638	148	1,894957788	1,14140783	0,624087487	0,316233837	0,268798762	0,159912897	0,131912897	**-1,128087103**
chrII	195265	195410	145	0,957452356	0,70782109	0,574952035	0,188996027	0,160646623	0,095612534	0,088612534	**-1,171387466**
chrII	203726	203877	151	1,470373261	1,082113917	0,576055102	0,191811584	0,163039847	0,058560082	0,009560082	**-1,250439918**
chrII	204265	204408	143	0,376141997	0,589233263	0,389632936	-0,280275857	-0,238234478	-0,318212415	-0,311212415	**-1,571212415**
chrII	213654	213809	155	0,262159574	0,059293913	0,815544346	0,898514518	0,763737341	0,678396211	0,601396211	**-0,658603789**
chrII	214589	214739	150	0,222265726	0,240881523	0,479902939	-0,050397187	-0,042837609	-0,187830875	-0,229830875	**-1,489830875**
chrII	214869	215017	148	0,210867483	0,137117174	0,605967759	0,268824834	0,228501109	0,111380536	0,083380536	**-1,176619464**
chrII	215320	215464	144	1,476072382	0,937585003	0,611550086	0,283361383	0,240857176	0,15608066	0,15608066	**-1,10391934**
chrII	216639	216771	132	0,04844253	0,185293479	0,207253175	-0,815989135	-0,693590765	-0,547397887	-0,463397887	**-1,723397887**
chrII	217546	217694	148	1,307948308	0,767115003	0,630317302	0,332693846	0,282789769	0,16136999	0,13336999	**-1,12663001**
chrII	222615	222772	157	2,299595391	1,441583266	0,614671365	0,291515265	0,247787976	0,208974001	0,117974001	**-1,142025999**
chrII	222619	222772	153	0,059840772	0,066705652	0,472876041	-0,06804215	-0,057835827	-0,123041905	-0,186041905	**-1,446041905**
chrII	223146	223281	135	0,062690333	0,077823261	0,446151373	-0,135390993	-0,115082344	-0,017836418	0,045163582	**-1,214836418**
chrII	223772	223929	157	0,062690333	0,074117392	0,458236793	-0,104876779	-0,089145262	-0,132106003	-0,223106003	**-1,483106003**
chrII	223872	224016	144	0,054141651	0,037058696	0,593656197	0,236960278	0,201416237	0,1242995	0,1242995	**-1,1357005**
chrII	223872	224016	144	0,054141651	0,037058696	0,593656197	0,236960278	0,201416237	0,1242995	0,1242995	**-1,1357005**
chrII	224435	224582	147	0,247911771	0,192705218	0,562646873	0,157683434	0,134030919	0,048101653	0,027101653	**-1,232898347**
chrII	224435	224582	147	0,247911771	0,192705218	0,562646873	0,157683434	0,134030919	0,048101653	0,027101653	**-1,232898347**
chrII	225175	225325	150	0,478726178	0,43729261	0,522616112	0,056720586	0,048212498	-0,108601247	-0,150601247	**-1,410601247**
chrII	226374	226481	107	0,056991212	0,585527393	0,088699706	-1,348805608	-1,146484767	-0,595265069	-0,336265069	**-1,596265069**
chrII	226832	226946	114	0,091185939	0,566998046	0,138541716	-1,086894512	-0,923860335	-0,299868328	-0,089868328	**-1,349868328**
chrII	228528	228693	165	0,062690333	0,155646522	0,287126664	-0,56179848	-0,477528708	-0,347544703	-0,494544703	**-1,754544703**
chrII	233542	233703	161	0,122531105	0,159352392	0,434687048	-0,164453569	-0,139785534	-0,109641967	-0,228641967	**-1,488641967**
chrII	234707	234798	91	2,105825271	9,772378079	0,177284831	-0,925762034	-0,786897729	-0,510796163	-0,139796163	**-1,399796163**
chrII	239108	239251	143	0,638301571	0,459527828	0,581421459	0,205531254	0,174701566	0,092067678	0,099067678	**-1,160932322**
chrII	249586	249752	166	0,119681545	0,170470001	0,412479432	-0,221171546	-0,187995814	-0,046240793	-0,200240793	**-1,460240793**
chrII	252919	253070	151	0,515770466	0,448410219	0,53493134	0,087672069	0,074521258	-0,029288891	-0,078288891	**-1,338288891**
chrII	253969	254127	158	0,262159574	0,340940001	0,434687048	-0,164453569	-0,139785534	-0,159313475	-0,257313475	**-1,517313475**
chrII	255566	255720	154	0,34194727	0,35205761	0,492715945	-0,018259434	-0,015520519	-0,100021544	-0,170021544	**-1,430021544**
chrII	260109	260232	123	0,088336378	0,226058044	0,280973108	-0,579953143	-0,492960172	0,059807211	0,206807211	**-1,053192789**
chrII	260209	260364	155	0,094035499	0,111176087	0,458236793	-0,104876779	-0,089145262	-0,172131457	-0,249131457	**-1,509131457**
chrII	260642	260776	134	0,142478029	0,055588044	0,719345959	0,58089966	0,493764711	0,603929933	0,673929933	**-0,586070067**
chrII	260642	260782	140	0,176672756	0,107470218	0,62177415	0,310143677	0,263622125	0,268242464	0,296242464	**-0,963757536**
chrII	261685	261820	135	0,059840772	0,048176305	0,553993628	0,135757811	0,11539414	0,201273103	0,264273103	**-0,995726897**
chrII	262132	262282	150	1,060036537	0,552174567	0,657504798	0,405662767	0,344813352	0,19221466	0,15021466	**-1,10978534**
chrII	262311	262468	157	0,04844253	0,055588044	0,465656665	-0,08619258	-0,073263693	-0,111281745	-0,202281745	**-1,462281745**
chrII	262974	263121	147	0,222265726	0,125999566	0,638208088	0,35367318	0,300622203	0,222279176	0,201279176	**-1,058720824**
chrII	263717	263867	150	0,492973981	0,429880871	0,534183658	0,085790846	0,072922219	-0,082266737	-0,124266737	**-1,384266737**
chrII	264916	265023	107	0,088336378	0,678174133	0,115244836	-1,199098437	-1,019233671	-0,457520689	-0,198520689	**-1,458520689**
chrII	269730	269883	153	3,801313818	2,175345443	0,636026525	0,347857838	0,295679162	0,221615763	0,158615763	**-1,101384237**
chrII	269730	269885	155	0,105433742	0,52623348	0,166913428	-0,966434402	-0,821469241	-0,896425246	-0,973425246	**-2,233425246**
chrII	269781	269930	149	0,14817715	0,144528914	0,506231912	0,015621722	0,013278464	-0,115364781	-0,150364781	**-1,410364781**
chrII	272917	273071	154	0,065539893	0,059293913	0,525017182	0,062749932	0,053337442	-0,051071069	-0,121071069	**-1,381071069**
chrII	279942	280075	133	1,22246149	1,178466526	0,509162075	0,022967935	0,019522745	0,106456886	0,183456886	**-1,076543114**
chrII	282915	283035	120	0,786478721	2,501461966	0,239201006	-0,708875061	-0,602543802	-0,00349185	0,16450815	**-1,09549185**
chrII	288899	289053	154	1,139824233	0,922761525	0,552619075	0,132281233	0,112439048	-0,000754737	-0,070754737	**-1,330754737**
chrII	289452	289559	107	0,119681545	0,559586306	0,176191975	-0,92997515	-0,790478878	-0,215533893	0,043466107	**-1,216533893**
chrII	289452	289573	121	0,142478029	0,648527176	0,180122745	-0,914897409	-0,777662797	-0,186147454	-0,025147454	**-1,285147454**
chrII	292636	292792	156	1,285151823	1,137701961	0,530428964	0,07634821	0,064895979	0,011153476	-0,072846524	**-1,332846524**
chrII	295986	296116	130	0,116831984	0,111176087	0,512402843	0,031094327	0,026430178	0,301059399	0,399059399	**-0,860940601**
chrII	296940	297047	107	0,424584527	2,660814357	0,137610903	-1,091116084	-0,927448672	-0,334508013	-0,075508013	**-1,335508013**
chrII	296940	297056	116	0,435982769	3,531693708	0,109883649	-1,227147126	-1,043075057	-0,434188266	-0,238188266	**-1,498188266**
chrII	298361	298510	149	0,361894194	0,233469783	0,607853696	0,273729366	0,232669961	0,101731233	0,066731233	**-1,193268767**
chrII	298515	298656	141	2,194161649	1,460112614	0,600437047	0,254478509	0,216306733	0,179726446	0,200726446	**-1,059273554**
chrII	300341	300475	134	1,689789426	1,241466309	0,576472877	0,192878343	0,163946591	0,275637106	0,345637106	**-0,914362894**
chrII	303103	303264	161	0,367593315	0,448410219	0,450480053	-0,124448587	-0,105781299	-0,082900746	-0,201900746	**-1,461900746**
chrII	304167	304319	152	0,168124074	0,125999566	0,571610206	0,180475116	0,153403848	0,048789963	-0,007210037	**-1,267210037**
chrII	306192	306322	130	0,225115286	0,452116089	0,332405282	-0,433281104	-0,368288938	-0,118327839	-0,020327839	**-1,280327839**
chrII	310844	311002	158	0,185221438	0,185293479	0,499902783	-0,000243688	-0,000207135	-0,010252604	-0,108252604	**-1,368252604**
chrII	314561	314691	130	0,350495952	0,381704567	0,478688478	-0,053445496	-0,045428672	0,199479088	0,297479088	**-0,962520912**
chrII	316179	316338	159	0,059840772	0,092646739	0,392430643	-0,272989502	-0,232041076	-0,239946668	-0,344946668	**-1,604946668**
chrII	320829	320984	155	0,963151477	0,733762177	0,567590151	0,170242159	0,144705835	0,07510426	-0,00189574	**-1,26189574**
chrII	324261	324383	122	0,188070999	0,422469132	0,308040355	-0,501412691	-0,426200787	0,130540583	0,284540583	**-0,975459417**
chrII	324593	324738	145	0,216566604	0,200116957	0,519738776	0,049497979	0,042073282	-0,02086413	-0,02786413	**-1,28786413**
chrII	333410	333578	168	0,367593315	0,581821524	0,387178818	-0,28667963	-0,243677685	0,019902012	-0,148097988	**-1,408097988**
chrII	334171	334335	164	0,170973635	0,314998914	0,351817475	-0,380418277	-0,323355536	-0,239948638	-0,379948638	**-1,639948638**
chrII	341568	341673	105	0,085486818	0,133411305	0,390532439	-0,277931585	-0,236241848	0,152424809	0,425424809	**-0,834575191**
chrII	344002	344150	148	3,536304684	2,334697835	0,602334043	0,259393151	0,220484178	0,086011167	0,058011167	**-1,201988833**
chrII	345574	345727	153	0,071239015	0,059293913	0,545755127	0,114943701	0,097702146	0,025621043	-0,037378957	**-1,297378957**
chrII	348428	348599	171	0,265009134	0,259410871	0,505337576	0,013379719	0,011372761	0,31746519	0,12846519	**-1,13153481**
chrII	348488	348624	136	0,549965193	0,485468915	0,531144559	0,078147301	0,066425206	0,071222097	0,127222097	**-1,132777903**
chrII	352601	352731	130	0,170973635	0,214940436	0,443035505	-0,14327752	-0,121785892	0,106689233	0,204689233	**-1,055310767**
chrII	354955	355107	152	0,464478375	0,340940001	0,576692051	0,193438075	0,164422364	0,061765223	0,005765223	**-1,254234777**
chrII	360789	360938	149	0,037044288	0,040764565	0,47609348	-0,059960669	-0,050966568	-0,179616729	-0,214616729	**-1,474616729**
chrII	369124	369272	148	0,205168362	0,203822827	0,501644944	0,004123275	0,003504784	-0,141493757	-0,169493757	**-1,429493757**
chrII	369614	369763	149	0,176672756	0,08894087	0,665149446	0,426558255	0,362574517	0,231627205	0,196627205	**-1,063372795**

Chrom	Start	End	Length	Section A	Section B	A/A+B	Z-score	Z * 0.85	Phase correction	Length correction	ΔLKnuc
chrII	372782	372926	144	0,7807796	0,43729261	0,640996152	0,361122739	0,306954328	0,245257057	0,245257057	**-1,014742943**
chrII	379733	379878	145	0,19661968	0,129705435	0,602526961	0,259893303	0,220909308	0,154047949	0,147047949	**-1,112952051**
chrII	380438	380593	155	1,775276244	0,948702612	0,651721741	0,389972994	0,331477045	0,259634452	0,182634452	**-1,077365548**
chrII	380438	380593	155	1,775276244	0,948702612	0,651721741	0,389972994	0,331477045	0,259634452	0,182634452	**-1,077365548**
chrII	383232	383381	149	0,914708947	0,400233914	0,695626383	0,511862517	0,435083139	0,298313505	0,263313505	**-0,996686495**
chrII	384056	384165	109	0,219416165	2,63487327	0,07687243	-1,426427917	-1,21246373	-0,608780409	-0,363780409	**-1,623780409**
chrII	384888	385039	151	0,501522663	0,415057393	0,547167331	0,118507771	0,100731606	-0,010140026	-0,059140026	**-1,319140026**
chrII	385956	386120	164	0,364743755	0,300175436	0,548553508	0,12200762	0,103706477	0,188306369	0,048306369	**-1,211693631**
chrII	386724	386859	135	5,587988304	4,787983496	0,538550838	0,096783504	0,082265978	0,143856833	0,206856833	**-1,053143167**
chrII	402057	402202	145	0,267858695	0,237175653	0,530377183	0,076218036	0,064785331	-0,009781246	-0,016781246	**-1,276781246**
chrII	405294	405447	153	0,250761331	0,155646522	0,617018911	0,297660652	0,253011555	0,163447736	0,100447736	**-1,159552264**
chrII	411086	411218	132	0,689593661	0,507704132	0,57595835	0,191564566	0,162829881	0,298959502	0,382959502	**-0,877040498**
chrII	412922	413067	145	0,367593315	0,314998914	0,538525491	0,09671967	0,082211719	0,000707699	-0,006292301	**-1,266292301**
chrII	417276	417442	166	0,037044288	0,037058696	0,499902783	-0,000243688	-0,000207135	0,1552076	0,0012076	**-1,2587924**
chrII	418844	418980	136	0,034194727	1,041349352	0,031792957	-1,855072217	-1,576811384	-1,564708743	-1,508708743	**-2,768708743**
chrII	420492	420630	138	2,826764099	2,438462183	0,536874191	0,092561893	0,078677609	0,108551358	0,150551358	**-1,109448642**
chrII	432763	432911	148	0,031345166	0,066705652	0,319682862	-0,468585806	-0,398297935	-0,543373047	-0,571373047	**-1,831373047**
chrII	435614	435764	150	5,884342605	4,917688931	0,544744068	0,112392927	0,095533988	-0,067178642	-0,109178642	**-1,369178642**
chrII	441246	441413	167	0,042743409	0,074117392	0,365763444	-0,34309514	-0,291630869	-0,083828399	-0,244828399	**-1,504828399**
chrII	442313	442462	149	0,817823887	0,726350437	0,529618887	0,074311878	0,063165096	-0,074837877	-0,109837877	**-1,369837877**
chrII	447274	447421	147	0,133929347	0,070411522	0,655421247	0,399998657	0,339998858	0,278840004	0,257840004	**-1,002159996**
chrII	448183	448311	128	0,427434088	0,759703264	0,360054451	-0,358313251	-0,304566264	0,055054216	0,167054216	**-1,092945784**
chrII	448687	448853	166	0,43883233	0,718938698	0,379032053	-0,308023953	-0,26182036	-0,095032771	-0,249032771	**-1,509032771**
chrII	450522	450683	161	0,151026711	0,137117174	0,524136442	0,060538046	0,051457339	0,064698676	-0,054301324	**-1,314301324**
chrII	456967	457110	143	0,128230226	0,181587609	0,413889104	-0,217551974	-0,184919178	-0,279475687	-0,272475687	**-1,532475687**
chrII	459507	459619	112	0,091185939	0,900526308	0,091947981	-1,328854548	-1,129526366	-0,543094464	-0,319094464	**-1,579094464**
chrII	463510	463659	149	0,458779254	0,429880871	0,516259525	0,040767875	0,034652694	-0,109145294	-0,144145294	**-1,404145294**
chrII	464402	464557	155	0,347646391	0,207528696	0,626192347	0,32178536	0,273517556	0,209587693	0,132587693	**-1,127412307**
chrII	467973	468117	144	0,812124766	0,470645436	0,6331023	0,340081173	0,289068997	0,227761572	0,227761572	**-1,032238428**
chrII	469937	470099	162	1,057186976	1,096937395	0,490773416	-0,023129678	-0,019660227	0,01445662	-0,11154338	**-1,37154338**
chrII	478544	478678	134	0,111132863	0,066705652	0,624908854	0,318399007	0,270639156	0,374158766	0,444158766	**-0,815841234**
chrII	479993	480154	161	0,071239015	0,059293913	0,545755127	0,114943701	0,097702146	0,119670466	0,000670466	**-1,259329534**
chrII	480073	480226	153	1,818019652	1,300760222	0,582926569	0,209386076	0,177978165	0,09238891	0,02938891	**-1,23061109**
chrII	483614	483775	161	3,71012788	2,834990228	0,566854229	0,168370847	0,14311522	0,164034151	0,045034151	**-1,214965849**
chrII	490237	490391	154	0,219416165	0,092646739	0,703115179	0,533381326	0,453374127	0,348466602	0,278466602	**-0,981533398**
chrII	496459	496596	137	0,219416165	0,237175653	0,480552117	-0,048767936	-0,041452746	-0,035572939	0,013427061	**-1,246572939**
chrII	498698	498864	166	0,176672756	0,229763914	0,434687048	-0,164453569	-0,139785534	0,034590256	-0,119409744	**-1,379409744**
chrII	499848	499990	142	1,165470279	0,622586089	0,651808466	0,390207566	0,331676431	0,318641738	0,332641738	**-0,927358262**
chrII	501137	501288	151	1,929152515	1,004290656	0,657641005	0,406033495	0,345128471	0,228495722	0,179495722	**-1,080504278**
chrII	503704	503871	167	1,601453048	2,645990879	0,377039244	-0,313266119	-0,266276202	-0,031303465	-0,192303465	**-1,452303465**
chrII	503704	503871	167	1,601453048	2,645990879	0,377039244	-0,313266119	-0,266276202	-0,031303465	-0,192303465	**-1,452303465**
chrII	505974	506148	174	0,401788042	0,659644785	0,37853365	-0,309334224	-0,26293409	0,075731346	-0,134268654	**-1,394268654**
chrII	506025	506148	123	0,065539893	0,070411522	0,4820832	-0,044925865	-0,038186985	0,48360805	0,63060805	**-0,62939195**
chrII	506025	506179	154	0,715239706	0,496586524	0,590216393	0,228101654	0,193886406	0,088906439	0,018906439	**-1,241093561**
chrII	506025	506179	154	0,715239706	0,496586524	0,590216393	0,228101654	0,193886406	0,088906439	0,018906439	**-1,241093561**
chrII	509772	509912	140	0,726637949	0,548468698	0,569864451	0,176029078	0,149624716	0,149331902	0,177331902	**-1,082668098**
chrII	511646	511795	149	0,233663968	0,229763914	0,504207833	0,010547668	0,008965518	-0,139658486	-0,174658486	**-1,434658486**
chrII	512273	512423	150	0,407487163	0,415057393	0,495398286	-0,011535042	-0,009804786	-0,14506331	-0,18706331	**-1,44706331**
chrII	519148	519306	158	0,464478375	0,389116306	0,544143943	0,110879232	0,094247347	0,086944464	-0,011055536	**-1,271055536**
chrII	523260	523407	147	0,307752543	0,300175436	0,506231912	0,015621722	0,013278464	-0,056333998	-0,077333998	**-1,337333998**
chrII	523774	523925	151	0,453080133	0,374292827	0,547612932	0,119632674	0,101687773	-0,007681204	-0,056681204	**-1,316681204**
chrII	525378	525528	150	0,766531797	0,589233263	0,565386895	0,164641441	0,139945225	0,014631912	-0,027368088	**-1,287368088**
chrII	527770	527902	132	0,236513528	0,240881523	0,495425179	-0,011467628	-0,009747484	0,109722164	0,193722164	**-1,066277836**
chrII	528914	529061	147	0,076938136	0,044470435	0,633712557	0,341702373	0,290447017	0,181350619	0,160350619	**-1,099649381**
chrII	529231	529384	153	0,045592969	0,037058696	0,551627959	0,129775456	0,110309137	0,049576779	-0,013423221	**-1,273423221**
chrII	533755	533907	152	0,105433742	0,048176305	0,686372697	0,485594627	0,412755433	0,326773409	0,270773409	**-0,989226591**
chrII	543934	544097	163	0,153876272	0,159352392	0,49125859	-0,021913219	-0,018626236	0,04763648	-0,08536352	**-1,34536352**
chrII	545512	545645	133	2,581701889	2,538520661	0,504216733	0,01056998	0,008984483	0,094236569	0,171236569	**-1,088763431**
chrII	550054	550200	146	0,225115286	0,085235	0,725358719	0,598835536	0,509010206	0,416119188	0,402119188	**-0,857880812**
chrII	551769	551916	147	0,136778908	0,118587827	0,535617563	0,08939893	0,07598909	-0,019364602	-0,040364602	**-1,300364602**
chrII	551821	551946	125	0,031345166	0,114881957	0,214359455	-0,791385773	-0,672677907	-0,199670932	-0,066670932	**-1,326670932**
chrII	552584	552756	172	0,153876272	0,274234349	0,359431101	-0,359979851	-0,305982873	0,015374615	-0,180625385	**-1,440625385**
chrII	560316	560442	126	0,601257283	1,704700006	0,260740858	-0,641063054	-0,544903596	-0,087672782	0,038327218	**-1,221672782**
chrII	568857	569009	152	0,29350474	0,255705001	0,534412845	0,086367464	0,073412344	0,00253359	-0,05346641	**-1,31346641**
chrII	570513	570675	162	0,501522663	0,455821958	0,523868471	0,059865123	0,050885354	0,096410325	-0,029589675	**-1,289589675**
chrII	570513	570675	162	0,501522663	0,455821958	0,523868471	0,059865123	0,050885354	0,096410325	-0,029589675	**-1,289589675**
chrII	575360	575489	129	8,354911631	11,22507895	0,426706621	-0,184765151	-0,157050378	0,177480188	0,282480188	**-0,977519812**
chrII	575363	575489	126	0,028495606	0,070411522	0,288104674	-0,558930214	-0,475090682	-0,041867983	0,084132017	**-1,175867983**
chrII	575635	575789	154	0,034194727	0,062999783	0,351817475	-0,380418277	-0,323355536	-0,424362726	-0,494362726	**-1,754362726**
chrII	577662	577794	132	0,162424953	0,485468915	0,250696852	-0,672298469	-0,571453699	-0,460094452	-0,376094452	**-1,636094452**
chrII	581371	581525	154	0,034194727	0,051882174	0,397257877	-0,260451199	-0,221383519	-0,315286481	-0,385286481	**-1,645286481**
chrII	583028	583186	158	3,068976748	2,657108488	0,53596421	0,090271358	0,076730654	0,068094189	-0,029905811	**-1,289905811**
chrII	583277	583426	149	0,336248149	0,196411088	0,631263153	0,335200699	0,284920594	0,135412008	0,100412008	**-1,159587992**
chrII	589093	589249	156	0,307752543	0,418763262	0,423600616	-0,192690667	-0,163787067	-0,21610912	-0,30010912	**-1,56010912**
chrII	598510	598657	147	0,185221438	0,140823044	0,568086406	0,171504378	0,145778722	0,048319102	0,027319102	**-1,232680898**
chrII	599865	600024	159	0,09973462	0,070411522	0,58617033	0,217704522	0,185048844	0,182642937	0,077642937	**-1,182357063**
chrII	601519	601677	158	0,051292091	0,066705652	0,434687048	-0,164453569	-0,139785534	-0,153310659	-0,251310659	**-1,511310659**
chrII	601519	601677	158	0,051292091	0,066705652	0,434687048	-0,164453569	-0,139785534	-0,153310659	-0,251310659	**-1,511310659**
chrII	608166	608308	142	0,384690679	0,259410871	0,597251597	0,246239543	0,209303612	0,19201439	0,20601439	**-1,05398561**
chrII	608166	608308	142	0,384690679	0,259410871	0,597251597	0,246239543	0,209303612	0,19201439	0,20601439	**-1,05398561**
chrII	610440	610604	164	0,094035499	0,129705435	0,420287416	-0,201158254	-0,170984516	-0,084272313	-0,224272313	**-1,484272313**
chrII	615980	616137	157	0,202318801	0,111176087	0,64536555	0,372838161	0,316912436	0,287751705	0,196751705	**-1,063248295**
chrII	618802	618957	155	0,230814407	0,170470001	0,575189075	0,18960094	0,161160799	0,107692003	0,030692003	**-1,229307997**
chrII	621368	621503	135	0,059840772	0,059293913	0,502295129	0,005753068	0,004890107	0,067734804	0,130734804	**-1,129265196**
chrII	624538	624687	149	0,666797177	0,455821958	0,593965625	0,23775806	0,202094351	0,045900264	0,010900264	**-1,249099736**
chrII	626717	626881	164	0,353345512	0,400233914	0,468889542	-0,078061561	-0,066352326	0,038569952	-0,101430048	**-1,361430048**
chrII	633744	633889	145	11,37829541	6,262919588	0,64498366	0,371812199	0,316040369	0,231545998	0,224545998	**-1,035454002**
chrII	633747	633889	142	0,056991212	0,044470435	0,561702018	0,155285859	0,13199298	0,110819889	0,124819889	**-1,135180111**
chrII	634590	634750	160	0,265009134	0,237175653	0,52771239	0,06952062	0,059092527	0,075334957	-0,036665043	**-1,296665043**
chrII	635204	635350	146	0,170973635	0,500292393	0,254703244	-0,659762131	-0,560797812	-0,664915442	-0,678915442	**-1,938915442**
chrII	636694	636853	159	1,943400318	1,975228485	0,495938864	-0,010179934	-0,008652944	-0,010705026	-0,115705026	**-1,375705026**
chrII	636695	636853	158	0,83207169	0,837526525	0,498366423	-0,004094782	-0,003480564	-0,006353674	-0,104353674	**-1,364353674**
chrII	636699	636853	154	0,039893848	0,048176305	0,452978074	-0,118140734	-0,100419624	-0,185038997	-0,255038997	**-1,515038997**
chrII	641881	642011	130	1,322196111	2,167933703	0,378838662	-0,308532302	-0,262252457	-0,047245845	0,050754155	**-1,209245845**
chrII	643000	643147	147	1,484621064	1,356348266	0,522575534	0,056618707	0,048125901	-0,048472018	-0,069472018	**-1,329472018**
chrII	643625	643773	148	0,190920559	0,114881957	0,624326319	0,316863258	0,269333769	0,132845152	0,104845152	**-1,155154848**

Chrom	Start	End	Length	Section A	Section B	A/A+B	Z-score	Z * 0.85	Phase correction	Length correction	ΔLKnuc
chrII	645958	646066	108	0,387540239	0,392822175	0,496615716	-0,008483244	-0,007210757	0,590769055	0,842769055	**-0,417230945**
chrII	652842	653006	164	0,09973462	0,066705652	0,599221683	0,251333038	0,213633083	0,324402012	0,184402012	**-1,075597988**
chrII	667311	667462	151	0,042743409	0,218646305	0,163523683	-0,98007851	-0,833066733	-0,937118016	-0,986118016	**-2,246118016**
chrII	670468	670600	132	0,034194727	0,059293913	0,365763444	-0,34309514	-0,291630869	-0,188029643	-0,104029643	**-1,364029643**
chrII	672951	673112	161	0,205168362	0,151940653	0,574525855	0,187908614	0,159722322	0,194497737	0,075497737	**-1,184502263**
chrII	673886	673964	78	0,09688506	0,111176087	0,465656665	-0,08619258	-0,073263693		0,462	**-0,798**
chrII	673886	673989	103	0,079787696	0,08894087	0,472876041	-0,06804215	-0,057835827	0,300093445	0,587093445	**-0,672906555**
chrII	674406	674512	106	0,131079787	1,063584569	0,109721016	-1,228013149	-1,043811177	-0,588178995	-0,322178995	**-1,582178995**
chrII	674406	674523	117	0,185221438	1,348936527	0,12073166	-1,171337061	-0,995636502	-0,383474226	-0,194474226	**-1,454474226**
chrII	678764	678912	148	0,116831984	0,074117392	0,61184795	0,284138687	0,241517884	0,112323926	0,084323926	**-1,175676074**
chrII	682036	682166	130	0,763682236	0,874585221	0,466152357	-0,084945506	-0,07220368	0,116153727	0,214153727	**-1,045846273**
chrII	683947	684082	135	0,413186285	0,377998697	0,52223727	0,055769465	0,047404045	0,103414501	0,166414501	**-1,093585499**
chrII	684506	684665	159	0,24221265	0,255705001	0,486451222	-0,033968281	-0,028873039	-0,035190924	-0,140190924	**-1,400190924**
chrII	685114	685267	153	1,501718428	0,956114351	0,61099292	0,281907864	0,239621685	0,179347677	0,116347677	**-1,143652323**
chrII	688568	688714	146	0,082637257	0,074117392	0,527175798	0,068172392	0,057946533	-0,039717319	-0,053717319	**-1,313717319**
chrII	693669	693820	151	0,09973462	0,148234783	0,402205349	-0,247642923	-0,210496484	-0,315186433	-0,364186433	**-1,624186433**
chrII	696303	696437	134	0,53856695	0,452116089	0,543631948	0,10958803	0,093149826	0,205401047	0,275401047	**-0,984598953**
chrII	698169	698308	139	0,413186285	0,244587392	0,628158741	0,326980653	0,277933555	0,290761886	0,325761886	**-0,934238114**
chrII	698728	698854	126	7,762203029	13,34483635	0,367754231	-0,33780723	-0,287136145	0,137998008	0,263998008	**-0,996001992**
chrII	698730	698836	106	0,059840772	0,085235	0,412479432	-0,221171546	-0,187995814	0,278221695	0,544221695	**-0,715778305**
chrII	699474	699629	155	0,418885406	0,385410436	0,52081011	0,052186888	0,044358855	-0,0077482	-0,0847482	**-1,3447482**
chrII	699802	699945	143	0,068389454	0,125999566	0,351817475	-0,380418277	-0,323355536	-0,426432646	-0,419432646	**-1,679432646**
chrII	699802	699953	151	0,29350474	0,503998263	0,368029636	-0,337076446	-0,286514979	-0,391062035	-0,440062035	**-1,700062035**
chrII	699834	699945	111	0,646850252	3,817045666	0,144907109	-1,058529261	-0,899749872	-0,284347211	-0,053347211	**-1,313347211**
chrII	699834	699953	119	0,039893848	0,096352609	0,292806499	-0,545204323	-0,463423675	0,150677432	0,325677432	**-0,934322568**
chrII	702899	703031	132	0,156725832	0,381704567	0,291079093	-0,550235021	-0,467699768	-0,358616715	-0,274616715	**-1,534616715**
chrII	706525	706696	171	0,133929347	0,163058261	0,450959378	-0,123237847	-0,10475217	0,206150217	0,017150217	**-1,242849783**
chrII	708493	708657	164	0,176672756	0,111176087	0,613769206	0,28915654	0,245783059	0,359630702	0,219630702	**-1,040369298**
chrII	708881	709054	173	0,512920905	0,978349569	0,343948944	-0,401709424	-0,34145301	-0,018569108	-0,221569108	**-1,481569108**
chrII	716866	717023	157	0,205168362	0,203822827	0,501644944	0,004123275	0,003504784	-0,018908789	-0,109908789	**-1,369908789**
chrII	716874	717023	149	0,378991558	0,200116957	0,654439622	0,397334573	0,337734387	0,186111937	0,151111937	**-1,108888063**
chrII	718549	718695	146	0,612655525	0,778232612	0,440477929	-0,149757607	-0,127293966	-0,209357466	-0,223357466	**-1,483357466**
chrII	721490	721639	149	0,213717044	0,114881957	0,650388599	0,386369815	0,328414343	0,180893704	0,145893704	**-1,114106296**
chrII	723357	723507	150	0,735186631	0,52993935	0,581117329	0,204752703	0,174039797	0,05622132	0,01422132	**-1,24577868**
chrII	727858	728011	153	0,626903328	0,370586958	0,628480635	0,327831948	0,278657156	0,211959253	0,148959253	**-1,111040747**
chrII	733512	733676	164	0,128230226	0,248293262	0,340563684	-0,410925221	-0,349286438	-0,228574356	-0,368574356	**-1,628574356**
chrII	735258	735391	133	0,125380666	0,048176305	0,722417922	0,590039516	0,501533589	0,581374356	0,658374356	**-0,601625644**
chrII	736781	736927	146	2,556055843	1,871464137	0,577310967	0,195019011	0,165766159	0,079946368	0,065946368	**-1,194053632**
chrII	736912	737033	121	0,435982769	1,256289787	0,257631531	-0,650664535	-0,553064855	0,022614777	0,183614777	**-1,076385223**
chrII	740252	740411	159	0,284956058	0,251999131	0,530688713	0,07700122	0,065451037	0,056331496	-0,048668504	**-1,308668504**
chrII	743678	743781	103	0,054141651	0,385410436	0,123174597	-1,159262522	-0,985373144	-0,624747713	-0,337747713	**-1,597747713**
chrII	746216	746373	157	3,527756002	4,009750884	0,468026903	-0,08023066	-0,068196061	-0,072993514	-0,163993514	**-1,423993514**
chrII	747192	747321	129	0,88051422	1,297054353	0,404356598	-0,242086426	-0,205773462	0,108827465	0,213827465	**-1,046172535**
chrII	748930	749090	160	0,453080133	0,470645436	0,490492142	-0,023834923	-0,020259685	0,004154254	-0,107845746	**-1,367845746**
chrII	749254	749401	147	5,992625907	3,631752187	0,622650716	0,312449984	0,265582486	0,172668771	0,151668771	**-1,108331229**
chrII	750045	750206	161	0,111132863	0,070411522	0,612152576	0,284933816	0,242193743	0,278174415	0,159174415	**-1,100825585**
chrII	760513	760620	107	0,364743755	1,007996525	0,265704853	-0,625855528	-0,531977199	0,055117639	0,314117639	**-0,945882361**
chrII	760513	760634	121	0,319150785	1,182172396	0,212579669	-0,79750235	-0,677876997	-0,120239247	0,040760753	**-1,219239247**
chrII	771359	771518	159	0,413186285	0,478057176	0,463606526	-0,091351809	-0,077649038	-0,072711441	-0,177711441	**-1,437711441**
chrII	771799	771953	154	0,125380666	0,100058479	0,556161913	0,141245282	0,12005849	0,031079725	-0,038920275	**-1,298920275**
chrII	774249	774405	156	0,276407377	0,289057827	0,488814121	-0,028042515	-0,023836138	-0,067326104	-0,151326104	**-1,411326104**
chrII	775701	775861	160	0,082637257	0,037058696	0,690393076	0,496964836	0,42242011	0,442536486	0,330536486	**-0,929463514**
chrII	777331	777484	153	0,102584181	0,092646739	0,525450481	0,063838228	0,054262494	-0,016755057	-0,079755057	**-1,339755057**
chrII	779629	779798	169	0,059840772	0,118587827	0,335376575	-0,425114581	-0,361347394	-0,07207337	-0,24707337	**-1,50707337**
chrII	780145	780251	106	0,094035499	0,270528479	0,257939634	-0,649710456	-0,552253888	-0,059022222	0,206977778	**-1,053022222**
chrII	780145	780260	115	0,133929347	0,337234132	0,284252395	-0,570254951	-0,484716709	0,138388095	0,341388095	**-0,918611905**
chrII	780582	780757	175	0,088336378	0,181587609	0,327263905	-0,447480992	-0,380358843	-0,023281704	-0,240281704	**-1,500281704**
chrII	781915	782083	168	0,094035499	0,103764348	0,47540734	-0,061683752	-0,052431189	0,229523645	0,061523645	**-1,198476355**
chrII	786436	786542	106	0,185221438	0,344645871	0,349561928	-0,38650344	-0,328527924	0,192244278	0,458244278	**-0,801755722**
chrII	786436	786560	124	0,179522317	0,418763262	0,300061247	-0,524224369	-0,445590714	0,057610066	0,197610066	**-1,062389934**
chrII	788442	788582	140	0,416035845	0,270528479	0,605967759	0,268824834	0,228501109	0,221719537	0,249719537	**-1,010280463**
chrII	788604	788759	155	0,094035499	0,103764348	0,47540734	-0,061683752	-0,052431189	-0,110389067	-0,187389067	**-1,447389067**
chrII	789546	789709	163	0,202318801	0,329822392	0,380197594	-0,304961878	-0,259217596	-0,193585326	-0,326585326	**-1,586585326**
chrII	791029	791155	126	0,039893848	0,122293696	0,245973563	-0,687215201	-0,584132921	-0,167735875	-0,041735875	**-1,301735875**
chrII	791029	791156	127	8,753850113	14,37136223	0,378541394	-0,309313861	-0,262916782	0,104188184	0,223188184	**-1,036811816**
chrII	791031	791136	105	0,074088575	0,096352609	0,434687048	-0,164453569	-0,139785534	0,273929751	0,546929751	**-0,713070249**
chrII	793238	793400	162	0,598407723	0,448410219	0,571644504	0,1805625	0,153478125	0,206077544	0,080077544	**-1,179922456**
chrII	795412	795559	147	0,082637257	0,055588044	0,597844653	0,247772164	0,210606339	0,112927708	0,091927708	**-1,168072292**
chrII	809074	809203	129	0,142478029	0,366881088	0,27972019	-0,583672934	-0,496121994	-0,1955723	-0,0905723	**-1,3505723**
chrII	812955	813098	143	0,359044634	0,270528479	0,57029855	0,177134301	0,150564156	0,035625321	0,042625321	**-1,217374679**
chrIII	2	145	143	0,344796831	0,159352392	0,683918204	0,478683799	0,406881229	0,298320548	0,305320548	**-0,954679452**
chrIII	2	176	174	0,669646737	0,074117392	0,900348257	1,283538485	1,091007713	1,240915948	1,030915948	**-0,229084052**
chrIII	4	145	141	0,210867483	0,270528479	0,438033344	-0,155957289	-0,132563695	-0,167102945	-0,146102945	**-1,406102945**
chrIII	10	145	135	0,675345858	0,874585221	0,43572638	-0,161813451	-0,137541434	-0,12229281	-0,05929281	**-1,31929281**
chrIII	10	146	136	0,074088575	0,051882174	0,588141101	0,222765786	0,189350918	0,208974256	0,264974256	**-0,995025744**
chrIII	14	145	131	0,056991212	0,048176305	0,541908887	0,105243965	0,08945737	0,27623444	0,36723444	**-0,89276556**
chrIII	19	145	126	0,065539893	0,051882174	0,558156527	0,146296985	0,124352437	0,457895969	0,583895969	**-0,676104031**
chrIII	26	145	119	0,059840772	0,133411305	0,309651379	-0,496838761	-0,422312947	-0,021431779	0,153568221	**-1,106431779**
chrIII	37	145	108	0,074088575	0,133411305	0,357053581	-0,366345661	-0,311393812	0,096984747	0,348984747	**-0,911015253**
chrIII	41	145	104	0,079787696	0,185293479	0,300993446	-0,521545394	-0,443313585	-0,036883833	0,243116167	**-1,016883833**
chrIII	50	145	95	0,199469241	0,370586958	0,349911537	-0,385559308	-0,327725412	0,076768639	0,419768639	**-0,840231361**
chrIII	50	176	126	2,151418241	0,296469566	0,8788876	1,16944398	0,994027383	1,290676152	1,416676152	**0,156676152**
chrIII	50	185	135	0,14532759	0,062999783	0,69759239	0,517488464	0,439865194	0,450177698	0,513177698	**-0,746822302**
chrIII	50	201	151	0,44168189	0,08894087	0,83238399	0,963628885	0,819084552	0,630061678	0,581061678	**-0,678938322**
chrIII	50	220	170	0,606956404	0,148234783	0,803712245	0,854955993	0,726712594	0,738073111	0,556073111	**-0,703926889**
chrIII	73	263	190	0,119681545	0,118587827	0,502295129	0,005753068	0,004890107	0,269206811	-0,052793189	**-1,312793189**
chrIII	88	231	143	0,04844253	0,040764565	0,543034495	0,108081541	0,09186931	-0,014464587	-0,007464587	**-1,267464587**
chrIII	97	212	115	0,034194727	0,055588044	0,380860679	-0,303221116	-0,257737948	0,125668629	0,328668629	**-0,931331371**
chrIII	110	263	153	0,367593315	0,26311674	0,582824568	0,209124736	0,177756026	0,053369477	-0,009630523	**-1,269630523**
chrIII	110	280	170	0,672496298	0,248293262	0,73034744	0,613864123	0,521784505	0,55339614	0,37139614	**-0,88860386**
chrIII	110	283	173	0,455929693	0,289057827	0,611996417	0,284526191	0,241847262	0,346609158	0,143609158	**-1,116390842**
chrIII	115	280	165	0,296354301	0,100058479	0,747590179	0,666925625	0,566886781	0,483721517	0,336721517	**-0,923278483**
chrIII	115	283	168	0,108283302	0,062999783	0,632189116	0,337656888	0,287008354	0,280775739	0,112775739	**-1,147224261**
chrIII	118	284	166	0,168124074	0,055588044	0,751519747	0,679279938	0,577387947	0,484847373	0,330847373	**-0,929152627**
chrIII	129	252	123	0,076938136	0,111176087	0,408996909	-0,230126057	-0,195607148	0,193894175	0,340894175	**-0,919105825**
chrIII	129	260	131	0,071239015	0,048176305	0,596565123	0,24446623	0,207796296	0,386187095	0,477187095	**-0,782812905**

Chrom	Start	End	Length	Section A	Section B	A/A+B	Z-score	Z * 0.85	Phase correction	Length correction	ΔLKnuc
chrIII	129	263	134	3,336835443	1,641700223	0,670244358	0,440588012	0,37449981	0,46923419	0,53923419	**-0,72076581**
chrIII	129	280	151	3,522056881	1,211819352	0,744011188	0,65576145	0,557397233	0,365105029	0,316105029	**-0,943894971**
chrIII	129	283	154	2,769772887	1,130290221	0,710186684	0,553930177	0,470840651	0,374478112	0,304478112	**-0,955521888**
chrIII	129	284	155	0,054141651	0,103764348	0,342872667	-0,404635672	-0,343940321	-0,386242825	-0,463242825	**-1,723242825**
chrIII	129	301	172	0,076938136	0,144528914	0,34740218	-0,39234359	-0,333492051	-0,239454046	-0,435454046	**-1,695454046**
chrIII	129	309	180	0,159575393	0,129705435	0,551627959	0,129775456	0,110309137	0,323534988	0,071534988	**-1,188465012**
chrIII	129	326	197	0,760832676	0,396528045	0,657385949	0,405339328	0,344538429	0,626714483	0,255714483	**-1,004285517**
chrIII	143	284	141	0,222265726	0,389116306	0,363546382	-0,348995399	-0,296646089	-0,334492119	-0,313492119	**-1,573492119**
chrIII	164	284	120	0,105433742	0,133411305	0,441431561	-0,147340685	-0,125239582	0,263478412	0,431478412	**-0,828521588**
chrIII	164	341	177	0,151026711	0,044470435	0,772526424	0,747192861	0,635113932	0,814217871	0,583217871	**-0,676782129**
chrIII	186	284	98	0,675345858	1,007996525	0,401193402	-0,250259331	-0,212720432	0,18882432	0,51082432	**-0,74917568**
chrIII	186	320	134	0,219416165	0,507704132	0,301760473	-0,519343898	-0,441442313	-0,361770143	-0,291770143	**-1,551770143**
chrIII	186	337	151	0,034194727	0,048176305	0,415130494	-0,214366854	-0,182211826	-0,362725041	-0,411725041	**-1,671725041**
chrIII	186	341	155	0,119681545	0,107470218	0,526879225	0,067427283	0,057313191	0,009551777	-0,067448223	**-1,327448223**
chrIII	186	352	166	1,114178188	1,011702395	0,524101964	0,060451462	0,051383743	-0,012052459	-0,166052459	**-1,426052459**
chrIII	186	353	167	0,14532759	0,170470001	0,460192206	-0,099949507	-0,084957081	-0,111908001	-0,272908001	**-1,532908001**
chrIII	186	355	169	0,202318801	0,055588044	0,784464644	0,787360758	0,669256644	0,662770011	0,487770011	**-0,772229989**
chrIII	192	329	137	0,113982423	0,070411522	0,618146236	0,300615741	0,25552338	0,284763137	0,333763137	**-0,926236863**
chrIII	246	341	95	0,156725832	0,326116523	0,324590066	-0,454901459	-0,386666241	0,016447417	0,359447417	**-0,900552583**
chrIII	246	352	106	0,042743409	0,055588044	0,434687048	-0,164453569	-0,139785534	0,28829251	0,55429251	**-0,70570749**
chrIII	5166	5314	148	3,171560929	2,167933703	0,593981481	0,237798943	0,202129102	0,055999786	0,027999786	**-1,232000214**
chrIII	5882	5994	112	0,202318801	2,401403487	0,077703679	-1,420688427	-1,207585163	-0,799019833	-0,575019833	**-1,835019833**
chrIII	7560	7714	154	0,062690333	0,08894087	0,413439528	-0,218706025	-0,185900121	-0,259499231	-0,329499231	**-1,589499231**
chrIII	9830	9940	110	0,079787696	0,144528914	0,355692324	-0,369997104	-0,314497538	0,101456424	0,339456424	**-0,920543576**
chrIII	9831	9940	109	2,641542661	5,959038282	0,307135376	-0,503986659	-0,42838866	-0,016392141	0,228607859	**-1,031392141**
chrIII	9831	9941	110	0,059840772	0,092646739	0,392430643	-0,272989502	-0,232041076	0,187696999	0,425696999	**-0,834303001**
chrIII	9832	9955	123	0,045592969	0,448410219	0,092292865	-1,326767123	-1,127752055	-0,752931237	-0,605931237	**-1,865931237**
chrIII	11462	11613	151	0,056991212	0,044470435	0,561702018	0,155285859	0,13199298	-0,049056487	-0,098056487	**-1,358056487**
chrIII	17946	18093	147	0,205168362	0,200116957	0,506231912	0,015621722	0,013278464	-0,100815646	-0,121815646	**-1,381815646**
chrIII	23277	23427	150	0,795027403	0,548468698	0,591760112	0,232074982	0,197263735	-0,002688533	-0,044688533	**-1,304688533**
chrIII	27235	27381	146	0,165274514	0,137117174	0,546557727	0,116969093	0,099423729	-0,013126281	-0,027126281	**-1,287126281**
chrIII	32929	33083	154	1,265204899	0,904232177	0,58319502	0,210073946	0,178562854	0,124326924	0,054326924	**-1,205673076**
chrIII	34797	34957	160	0,458779254	0,544762828	0,457159956	-0,107591283	-0,091452591	-0,182230317	-0,294230317	**-1,554230317**
chrIII	36971	37125	154	1,168319839	1,026525873	0,532301579	0,081056724	0,068898216	0,016647479	-0,053352521	**-1,313352521**
chrIII	37274	37421	147	0,29065518	0,163058261	0,640613994	0,360100456	0,306085387	0,193926136	0,172926136	**-1,087073864**
chrIII	41105	41261	156	0,512920905	0,463233697	0,525450481	0,063838228	0,054262494	-0,031512355	-0,115512355	**-1,375512355**
chrIII	42270	42419	149	0,199469241	0,096352609	0,674288397	0,45178593	0,38401804	0,174343281	0,139343281	**-1,120656719**
chrIII	56437	56565	128	0,185221438	0,314998914	0,370279692	-0,331112667	-0,281445767	-0,014300779	0,097699221	**-1,162300779**
chrIII	62417	62568	151	13,98279378	10,67661026	0,567036971	0,168835471	0,14351015	-0,029661321	-0,078661321	**-1,338661321**
chrIII	62420	62568	148	0,039893848	0,037058696	0,518421434	0,046192108	0,039263292	-0,107111568	-0,135111568	**-1,395111568**
chrIII	62779	62946	167	0,230814407	0,270528479	0,460392305	-0,099445434	-0,084528619	-0,10512384	-0,26612384	**-1,52612384**
chrIII	62988	63143	155	0,159575393	0,181587609	0,467739443	-0,080953558	-0,068810524	-0,123107083	-0,200107083	**-1,460107083**
chrIII	68315	68492	177	0,037044288	0,085235	0,302948179	-0,51593994	-0,438548949	-0,235689326	-0,466689326	**-1,726689326**
chrIII	68580	68746	166	4,029278665	3,583575882	0,529273039	0,073442598	0,062426208	0,000224316	-0,153775684	**-1,413775684**
chrIII	74254	74383	129	1,005894886	1,167348917	0,462854137	-0,093245819	-0,079258946	0,145353208	0,250353208	**-1,009646792**
chrIII	75106	75269	163	1,70688679	1,537935875	0,526033921	0,065303749	0,055508186	-0,032221364	-0,165221364	**-1,425221364**
chrIII	82256	82435	179	0,028495606	0,118587827	0,1937377	-0,864204794	-0,734574075	-0,522682426	-0,767682426	**-2,027682426**
chrIII	86659	86788	129	3,020534219	5,573627846	0,351463493	-0,381372335	-0,324166485	-0,096349554	0,008650446	**-1,251349554**
chrIII	86884	87013	129	0,176672756	0,366881088	0,325032668	-0,453671427	-0,385620713	-0,176965765	-0,071965765	**-1,331965765**
chrIII	88349	88492	143	0,188070999	0,092646739	0,669964783	0,439815924	0,373843536	0,247127492	0,254127492	**-1,005872508**
chrIII	89624	89790	166	0,042743409	0,074117392	0,365763444	-0,34309514	-0,291630869	-0,357992996	-0,511992996	**-1,771992996**
chrIII	90428	90577	149	0,119681545	0,051882174	0,69759239	0,517488464	0,439865194	0,229648339	0,194648339	**-1,065351661**
chrIII	90432	90582	150	0,056991212	0,040764565	0,582995843	0,209563572	0,178129036	-0,018665557	-0,060665557	**-1,320665557**
chrIII	92227	92379	152	3,997933499	2,164227834	0,648787541	0,382049137	0,324741767	0,203568862	0,147568862	**-1,112431138**
chrIII	93544	93697	153	1,681240744	1,267407396	0,570173403	0,176815652	0,150293304	0,029672552	-0,033327448	**-1,293327448**
chrIII	94513	94671	158	1,344992595	1,471230223	0,477587422	-0,056209587	-0,047778149	-0,107807665	-0,205807665	**-1,465807665**
chrIII	95867	96028	161	0,076938136	0,125999566	0,379121943	-0,307787693	-0,261619539	-0,331905784	-0,450905784	**-1,710905784**
chrIII	95874	96028	154	0,213717044	0,244587392	0,466321133	-0,084520926	-0,071842787	-0,117836567	-0,187836567	**-1,447836567**
chrIII	97469	97602	133	0,028495606	0,062999783	0,311443082	-0,491764035	-0,41799943	-0,312463613	-0,235463613	**-1,495463613**
chrIII	100878	101025	147	0,094035499	0,074117392	0,559226183	0,149007602	0,126656462	-0,00886259	-0,02986259	**-1,28986259**
chrIII	100878	101048	170	0,085486818	0,077823261	0,523463207	0,058847485	0,050020363	0,106177271	-0,075822729	**-1,335822729**
chrIII	101513	101652	139	0,119681545	0,081529131	0,594807131	0,239928434	0,203939169	0,179601212	0,214601212	**-1,045398788**
chrIII	101547	101689	142	1,045788734	0,755997394	0,580417796	0,202962418	0,172518055	0,10153868	0,11553868	**-1,14446132**
chrIII	101696	101853	157	0,065539893	0,037058696	0,638799168	0,355250862	0,301963233	0,232088092	0,141088092	**-1,118911908**
chrIII	103033	103165	132	10,5405246	6,470448284	0,619630909	0,304511568	0,258834833	0,401752809	0,485752809	**-0,774247191**
chrIII	103103	103249	146	0,056991212	0,037058696	0,605967759	0,268824834	0,228501109	0,117216263	0,103216263	**-1,156783737**
chrIII	103103	103251	148	17,08881482	8,983027859	0,655450979	0,400079394	0,340067485	0,185477499	0,157477499	**-1,102522501**
chrIII	104725	104877	152	6,280431526	3,379753056	0,650135768	0,385687039	0,327833983	0,195910235	0,139910235	**-1,120089765**
chrIII	106067	106230	163	0,227964847	0,159352392	0,588573975	0,223878237	0,190296502	0,078090595	-0,054909405	**-1,314909405**
chrIII	116514	116644	130	0,370442876	0,600350872	0,38158762	-0,301313772	-0,256116706	-0,056378718	0,041621282	**-1,218378718**
chrIII	117818	117976	158	0,125380666	0,103764348	0,547167331	0,118507771	0,100731606	0,049370399	-0,048629601	**-1,308629601**
chrIII	132816	132923	107	0,817823887	5,89233263	0,121878511	-1,16564743	-0,990800316	-0,551858369	-0,292858369	**-1,552858369**
chrIII	132817	132923	106	0,04844253	1,082113917	0,042848396	-1,718547538	-1,460765408	-1,011595077	-0,745595077	**-2,005595077**
chrIII	133268	133417	149	0,125380666	0,070411522	0,640376244	0,359464657	0,305544959	0,096053301	0,061053301	**-1,198946699**
chrIII	134878	135010	132	0,037044288	0,107470218	0,25633612	-0,65468243	-0,556480065	-0,426285663	-0,342285663	**-1,602285663**
chrIII	136592	136744	152	0,322000346	0,144528914	0,690203968	0,496428581	0,421964293	0,290980715	0,234980715	**-1,025019285**
chrIII	137946	138097	151	0,29065518	0,192705218	0,601321873	0,256770098	0,218254584	0,057875812	0,008875812	**-1,251124188**
chrIII	138189	138339	150	2,325241436	1,363760005	0,630317302	0,332693846	0,282789769	0,079782755	0,037782755	**-1,222217245**
chrIII	146706	146857	151	0,128230226	0,17417587	0,424033205	-0,191586128	-0,162848208	-0,308003251	-0,357003251	**-1,617003251**
chrIII	154254	154394	140	0,567062556	0,466939567	0,548415273	0,121658533	0,103409753	0,067615651	0,095615651	**-1,164384349**
chrIII	154256	154394	138	0,153876272	0,207528696	0,42577243	-0,18714766	-0,159075511	-0,163052849	-0,121052849	**-1,381052849**
chrIII	156400	156550	150	6,454254722	3,968986319	0,61921764	0,303426675	0,257912674	0,055700303	0,013700303	**-1,246299697**
chrIII	156437	156550	113	0,97454972	2,075286964	0,319541609	-0,468981	-0,39863385	0,000349124	0,217349124	**-1,042650876**
chrIII	156484	156645	161	0,359044634	0,448410219	0,444662178	-0,13915919	-0,118285311	-0,175770183	-0,294770183	**-1,554770183**
chrIII	158189	158338	149	0,239363089	0,26682261	0,472876041	-0,06804215	-0,057835827	-0,269439488	-0,304439488	**-1,564439488**
chrIII	159911	160084	173	0,239363089	0,541056958	0,306710585	-0,505196015	-0,429416612	-0,306715977	-0,509715977	**-1,769715977**
chrIII	159911	160084	173	0,239363089	0,541056958	0,306710585	-0,505196015	-0,429416612	-0,306715977	-0,509715977	**-1,769715977**
chrIII	161801	161938	137	0,028495606	0,100058479	0,221662392	-0,766590907	-0,651602271	-0,634675426	-0,585675426	**-1,845675426**
chrIII	175934	176084	150	0,142478029	0,074117392	0,657807209	0,406485945	0,345513053	0,142023193	0,100023193	**-1,159976807**
chrIII	186417	186569	152	0,296354301	0,244587392	0,54784888	0,120228376	0,10219412	-0,029774075	-0,085774075	**-1,345774075**
chrIII	188100	188260	160	0,253610892	0,381704567	0,39918892	-0,255447043	-0,217129987	-0,297387205	-0,409387205	**-1,669387205**
chrIII	191522	191646	124	2,045984499	3,457576317	0,371756499	-0,327204791	-0,278124073	0,088500058	0,228500058	**-1,031499942**
chrIII	195429	195578	149	0,222265726	0,129705435	0,631488458	0,335798151	0,285428429	0,08556642	0,05056642	**-1,20943358**
chrIII	201078	201230	152	0,139628469	0,085235	0,620947766	0,307970909	0,261775272	0,12718959	0,07118959	**-1,18881041**
chrIII	208738	208879	141	0,236513528	0,718938698	0,247540925	-0,68224847	-0,579911199	-0,643906559	-0,622906559	**-1,882906559**
chrIII	209585	209734	149	0,188070999	0,092646739	0,669964783	0,439815924	0,373843536	0,18593129	0,15093129	**-1,10906871**

Chrom	Start	End	Length	Section A	Section B	A/A+B	Z-score	Z * 0.85	Phase correction	Length correction	ΔLKnuc
chrIII	210916	211072	156	0,227964847	0,163058261	0,582995843	0,209563572	0,178129036	0,099265444	0,015265444	**-1,244734556**
chrIII	213468	213602	134	0,547115632	0,385410436	0,586702775	0,219071373	0,186210667	0,235392304	0,305392304	**-0,954607696**
chrIII	215653	215790	137	5,562342259	2,935048706	0,654594132	0,397753717	0,338090659	0,345383895	0,394383895	**-0,865616105**
chrIII	217960	218102	142	0,567062556	0,44099848	0,562527998	0,157381739	0,133774478	0,037886471	0,051886471	**-1,208113529**
chrIII	228591	228741	150	1,307948308	0,874585221	0,599279824	0,251483454	0,213760936	0,017233845	-0,024766155	**-1,284766155**
chrIII	231011	231166	155	0,042743409	0,070411522	0,377742344	-0,311415611	-0,264703269	-0,322060501	-0,399060501	**-1,659060501**
chrIII	234251	234402	151	0,094035499	0,107470218	0,466664175	-0,083658001	-0,071109301	-0,209111731	-0,258111731	**-1,518111731**
chrIII	234921	235052	131	0,105433742	0,463233697	0,185404921	-0,894957435	-0,76071382	-0,59644927	-0,50544927	**-1,76544927**
chrIII	236307	236469	162	0,247911771	0,222352175	0,527175798	0,068172392	0,057946533	-0,003862065	-0,129862065	**-1,389862065**
chrIII	238120	238291	171	0,142478029	0,43729261	0,245748956	-0,687928336	-0,584739086	-0,513163652	-0,702163652	**-1,962163652**
chrIII	238120	238291	171	0,142478029	0,43729261	0,245748956	-0,687928336	-0,584739086	-0,513163652	-0,702163652	**-1,962163652**
chrIII	240298	240452	154	0,031345166	0,055588044	0,360566076	-0,356946107	-0,303404191	-0,34725888	-0,41725888	**-1,67725888**
chrIII	240452	240614	162	0,661098055	0,492880654	0,572885834	0,183726101	0,156167186	0,103297933	-0,022702067	**-1,282702067**
chrIII	242569	242719	150	0,185221438	0,081529131	0,694361923	0,508252677	0,432014775	0,242769543	0,200769543	**-1,059230457**
chrIII	246007	246188	181	0,04844253	0,166764131	0,225097726	-0,755089219	-0,641825836	-0,410933178	-0,669933178	**-1,929933178**
chrIII	246926	247057	131	0,04844253	0,048176305	0,50137771	0,003453413	0,002935401	0,161545227	0,252545227	**-1,007454773**
chrIII	246926	247058	132	11,87411895	8,808851988	0,574101225	0,186825376	0,158801569	0,278239571	0,362239571	**-0,897760429**
chrIII	248934	249085	151	0,265009134	0,285351958	0,481518658	-0,046342437	-0,039391072	-0,171690621	-0,220690621	**-1,480690621**
chrIII	251728	251875	147	0,393239361	0,17417587	0,693036315	0,504475364	0,428804059	0,295289457	0,274289457	**-0,985710543**
chrIII	252213	252361	148	14,90605141	8,701381771	0,631413475	0,335599302	0,285259406	0,106878214	0,078878214	**-1,181121786**
chrIII	252238	252397	159	1,843665698	1,712111745	0,51849862	0,046385793	0,039427924	-0,005693883	-0,110693883	**-1,370693883**
chrIII	254663	254821	158	1,097080825	0,896820438	0,550218231	0,126212725	0,107280816	0,064890317	-0,033109683	**-1,293109683**
chrIII	257083	257228	145	0,108283302	0,125999566	0,462190442	-0,094916836	-0,080679311	-0,196182799	-0,203182799	**-1,463182799**
chrIII	257806	257969	163	0,108283302	0,092646739	0,538910465	0,097689227	0,083035843	-0,01677351	-0,14977351	**-1,40977351**
chrIII	258183	258325	142	1,296550065	0,726350437	0,640936153	0,360962213	0,306817881	0,20726129	0,22126129	**-1,03873871**
chrIII	259144	259292	148	0,492973981	0,366881088	0,573322178	0,184838583	0,157112796	-0,04261948	-0,07061948	**-1,33061948**
chrIII	262204	262356	152	0,68389454	0,407645654	0,626540868	0,322705529	0,274299699	0,145361696	0,089361696	**-1,170638304**
chrIII	269289	269448	159	0,153876272	0,181587609	0,458696987	-0,10371695	-0,088159408	-0,146255299	-0,251255299	**-1,511255299**
chrIII	272784	272929	145	0,062690333	0,100058479	0,38519687	-0,291859905	-0,248080919	-0,369330826	-0,376330826	**-1,636330826**
chrIII	276672	276825	153	0,125380666	0,148234783	0,458236793	-0,104876779	-0,089145262	-0,202113886	-0,265113886	**-1,525113886**
chrIII	280552	280680	128	0,606956404	1,511994788	0,286441899	-0,563809481	-0,479238059	-0,240452975	-0,128452975	**-1,388452975**
chrIII	281034	281170	136	0,686744101	0,585527393	0,539777951	0,099874329	0,084893179	0,126881609	0,182881609	**-1,077118391**
chrIII	286830	286938	108	7,719459621	20,97522181	0,269020572	-0,615777856	-0,523411177	-0,104362447	0,147637553	**-1,112362447**
chrIII	286830	286939	109	2,652940903	7,100446113	0,272002013	-0,606769297	-0,515753903	-0,109724778	0,135275222	**-1,124724778**
chrIII	286830	286940	110	0,165274514	0,637409568	0,205902319	-0,820721997	-0,697613698	-0,287761628	-0,049761628	**-1,309761628**
chrIII	286830	286941	111	0,065539893	0,17788174	0,269244325	-0,615100047	-0,52283504	-0,126714204	0,104285796	**-1,155714204**
chrIII	286832	286938	106	0,176672756	1,40823044	0,111472269	-1,218735659	-1,03592531	-0,586583781	-0,320583781	**-1,580583781**
chrIII	286832	286939	107	0,071239015	0,585527393	0,108469334	-1,234709424	-1,049503011	-0,642068654	-0,383068654	**-1,643068654**
chrIII	286832	286955	123	0,404637603	2,868343054	0,123629696	-1,157031743	-0,983476982	-0,608702946	-0,461702946	**-1,721702946**
chrIII	288517	288666	149	0,065539893	0,107470218	0,378821174	-0,308578276	-0,262291535	-0,446922271	-0,481922271	**-1,741922271**
chrIII	303804	303963	159	0,359044634	0,314998914	0,532672755	0,081990221	0,069691688	-0,00880065	-0,11380065	**-1,37380065**
chrIII	307204	307345	141	0,045592969	0,037058696	0,551627959	0,129775456	0,110309137	0,042459342	0,063459342	**-1,196540658**
chrIII	307633	307788	155	0,190920559	0,070411522	0,730566865	0,614528311	0,522349064	0,44289037	0,36589037	**-0,89410963**
chrIII	308042	308191	149	2,78117113	2,338403704	0,543242597	0,108606247	0,09231531	-0,075040044	-0,110040044	**-1,370040044**
chrIII	310321	310472	151	0,056991212	0,103764348	0,354520937	-0,373143192	-0,317171714	-0,438404911	-0,487404911	**-1,747404911**
chrIII	310654	310803	149	0,04844253	0,040764565	0,543034495	0,108081541	0,09186931	-0,085621571	-0,120621571	**-1,380621571**
chrIII	311798	311947	149	0,601257283	0,463233697	0,564830792	0,16322864	0,138744344	-0,05314441	-0,08814441	**-1,34814441**
chrIII	314554	314707	153	0,270708255	0,255705001	0,514250453	0,035728188	0,030368959	-0,075051656	-0,138051656	**-1,398051656**
chrIV	0	126	126	0,170973635	0,292763697	0,368686373	-0,335334533	-0,285034353	0,03016491	0,15616491	**-1,10383509**
chrIV	0	145	145	0,108283302	0,196411088	0,355383315	-0,370826679	-0,315202677	-0,490917866	-0,497917866	**-1,757917866**
chrIV	0	155	155	0,119681545	0,044470435	0,729089865	0,610062706	0,5185533	0,407184851	0,330184851	**-0,929815149**
chrIV	5	126	121	0,051292091	0,062999783	0,448781606	-0,128740216	-0,109429183	0,44863893	0,60963893	**-0,65036107**
chrIV	14	126	112	0,056991212	0,044470435	0,561702018	0,155285859	0,13199298	0,694521463	0,918521463	**-0,341478537**
chrIV	14	134	120	0,364743755	0,125999566	0,743247518	0,653389875	0,555381394	1,101244849	1,269244849	**0,009244849**
chrIV	14	155	141	0,624053768	0,070411522	0,898610452	1,273673656	1,082622608	0,918480777	0,939480777	**-0,320519223**
chrIV	44	126	82	0,031345166	0,43729261	0,066885701	-1,499394179	-1,274485052	-0,971422563	-0,537422563	**-1,797422563**
chrIV	44	134	90	0,071239015	0,125999566	0,361181948	-0,355301282	-0,302006089	0,015467902	0,393467902	**-0,866532098**
chrIV	44	145	101	0,065539893	0,337234132	0,162721251	-0,983335176	-0,8358349	-0,376249574	-0,075249574	**-1,335249574**
chrIV	44	155	111	0,059840772	0,077823261	0,434687048	-0,164453569	-0,139785534	0,401249498	0,632249498	**-0,627750502**
chrIV	57	155	98	0,037044288	0,140823044	0,208269204	-0,812441383	-0,690575176	-0,237481081	0,084518919	**-1,175481081**
chrIV	1765	1923	158	0,19377012	0,200116957	0,491943329	-0,020196453	-0,017166985	0,00297484	-0,09502516	**-1,35502516**
chrIV	6558	6652	94	1,980444606	11,64754809	0,145321813	-1,056710731	-0,898204121	-0,504337508	-0,154337508	**-1,414337508**
chrIV	8786	8917	131	0,219416165	0,311293045	0,413439528	-0,218706025	-0,185900121	-0,076132351	0,014867649	**-1,245132351**
chrIV	10468	10622	154	0,059840772	0,066705652	0,472876041	-0,06804215	-0,057835827	-0,143432049	-0,213432049	**-1,473432049**
chrIV	11706	11865	159	0,054141651	0,037058696	0,593656197	0,236960278	0,201416237	0,214856707	0,109856707	**-1,150143293**
chrIV	12342	12498	156	0,028495606	0,051882174	0,354520937	-0,373143192	-0,317171714	-0,270260576	-0,354260576	**-1,614260576**
chrIV	12922	13062	140	2,191312089	1,378583483	0,613830866	0,289317699	0,245920045	0,134244554	0,162244554	**-1,097755446**
chrIV	13187	13340	153	0,09688506	0,051882174	0,65125268	0,388704654	0,330398956	0,255320579	0,192320579	**-1,067679421**
chrIV	15846	16000	154	0,524319147	0,452116089	0,536972784	0,092810095	0,07888858	-0,012552837	-0,082552837	**-1,342552837**
chrIV	16215	16372	157	0,789328282	0,496586524	0,613826265	0,289305674	0,245909823	0,281892997	0,190892997	**-1,069107003**
chrIV	16517	16676	159	4,827155628	3,60210523	0,572666537	0,183167078	0,155692016	0,172945287	0,067945287	**-1,192054713**
chrIV	22332	22485	153	0,552814753	0,496586524	0,526790624	0,067204689	0,057123986	-0,020674134	-0,083674134	**-1,343674134**
chrIV	22780	22921	141	0,216566604	0,103764348	0,676071427	0,456741101	0,388229936	0,222885276	0,243885276	**-1,016114724**
chrIV	24904	25025	121	0,068389454	0,374292827	0,154488799	-1,017369684	-0,864764232	-0,290808288	-0,129808288	**-1,389808288**
chrIV	26118	26260	142	0,113982423	0,096352609	0,541908887	0,105243965	0,08945737	-0,068219095	-0,054219095	**-1,314219095**
chrIV	28132	28284	152	0,427434088	0,452116089	0,485968964	-0,035177847	-0,02990117	-0,144268241	-0,200268241	**-1,460268241**
chrIV	28565	28649	84	0,205168362	0,188999349	0,520510322	0,051434423	0,043719259	0,494520691	0,914520691	**-0,345479309**
chrIV	28606	28762	156	0,510071344	0,322410653	0,612711561	0,286393336	0,243434336	0,28800125	0,20400125	**-1,05599875**
chrIV	29713	29864	151	0,062690333	0,074117392	0,458236793	-0,104876779	-0,089145262	-0,150773062	-0,199773062	**-1,459773062**
chrIV	30032	30191	159	0,074088575	0,085235	0,465019536	-0,087795679	-0,074626327	-0,057734494	-0,162734494	**-1,422734494**
chrIV	31789	31954	165	0,401788043	0,670762394	0,374609329	-0,319668215	-0,271717983	-0,186088039	-0,333088039	**-1,593088039**
chrIV	32229	32385	156	0,108283302	0,192705218	0,359758911	-0,359103289	-0,305237796	-0,26634737	-0,35034737	**-1,61034737**
chrIV	33584	33743	159	0,421734966	0,463233697	0,476553559	-0,058805386	-0,049984578	-0,026758771	-0,131758771	**-1,391758771**
chrIV	34942	35098	156	0,247911771	0,289057827	0,461686791	-0,096185077	-0,081757315	-0,046390195	-0,130390195	**-1,390390195**
chrIV	43249	43399	150	0,199469241	0,137117174	0,592624158	0,234300517	0,19915544	0,088461441	0,046461441	**-1,213538559**
chrIV	43986	44132	146	0,173823196	0,148234783	0,539726407	0,09974448	0,084782808	-0,111102157	-0,125102157	**-1,385102157**
chrIV	51938	52044	106	0,076938136	0,337234132	0,18576361	-0,893616302	-0,759573857	-0,222172823	0,043827177	**-1,216172823**
chrIV	55412	55541	129	2,060232302	3,472399795	0,37237833	-0,325560819	-0,276726696	-0,041002235	0,063997765	**-1,196002235**
chrIV	56586	56757	171	0,190920559	0,188999349	0,502528442	0,006337906	0,00538722	0,221133588	0,032133588	**-1,227866412**
chrIV	60333	60467	134	0,547115632	0,515115871	0,515062517	0,037765106	0,03210034	0,092544827	0,162544827	**-1,097455173**
chrIV	61937	62077	140	3,376729291	1,84922892	0,646145483	0,374934296	0,318694492	0,203565359	0,231565359	**-1,028434641**
chrIV	77124	77282	158	0,054141651	0,081529131	0,399066404	-0,255764342	-0,217399691	-0,193926047	-0,291926047	**-1,551926047**
chrIV	78906	79062	156	0,273557816	0,240881523	0,531759132	0,079692611	0,067738719	0,111806886	0,027806886	**-1,232193114**
chrIV	86969	87132	163	0,273557816	0,185293479	0,596179675	0,243470869	0,206950239	0,234734919	0,101734919	**-1,158265081**
chrIV	90699	90847	148	0,381841118	0,34835174	0,522931872	0,057513369	0,048886364	-0,122873582	-0,150873582	**-1,410873582**
chrIV	94580	94722	142	0,353345512	0,363175219	0,493140669	-0,017194641	-0,014615445	-0,17929725	-0,16529725	**-1,42529725**

Chrom	Start	End	Length	Section A	Section B	A/A+B	Z-score	Z * 0.85	Phase correction	Length correction	ΔLKnuc
chrIV	97197	97345	148	0,629752889	0,218646305	0,742283695	0,650401964	0,552841669	0,376156247	0,348156247	**-0,911843753**
chrIV	101729	101892	163	0,102584181	0,070411522	0,592986873	0,235235114	0,199949847	0,224554779	0,091554779	**-1,168445221**
chrIV	103149	103279	130	0,105433742	0,040764565	0,721169375	0,586318873	0,498371042	0,632787552	0,730787552	**-0,529212448**
chrIV	109515	109652	137	0,042743409	0,062999783	0,404219015	-0,242441566	-0,206075331	-0,232348703	-0,183348703	**-1,443348703**
chrIV	109515	109656	141	8,169690193	5,821921108	0,583899168	0,211878705	0,180096899	0,025168222	0,046168222	**-1,213831778**
chrIV	109515	109666	151	1,929152515	0,989467177	0,660981121	0,415142268	0,352870928	0,293994135	0,244994135	**-1,015005865**
chrIV	109518	109656	138	0,034194727	0,044470435	0,434687048	-0,164453569	-0,139785534	-0,204156087	-0,162156087	**-1,422156087**
chrIV	110479	110614	135	1,242408414	1,215525222	0,505468657	0,01370832	0,011652072	0,008085083	0,071085083	**-1,188914917**
chrIV	113056	113240	184	0,094035499	0,203822827	0,315705458	-0,47974192	-0,407780632	-0,007311362	-0,287311362	**-1,547311362**
chrIV	118783	118936	153	0,544266071	0,455821958	0,544218164	0,111066427	0,094406463	0,031868623	-0,031131377	**-1,291131377**
chrIV	121491	121639	148	0,629752889	0,396528045	0,613626219	0,288782843	0,245465417	0,068811191	0,040811191	**-1,219188809**
chrIV	122619	122777	158	0,356195073	0,407645654	0,466321133	-0,084520926	-0,071842787	-0,080248176	-0,178248176	**-1,438248176**
chrIV	124114	124238	124	5,796006227	10,83966852	0,348408244	-0,38962145	-0,331178233	0,095008169	0,235008169	**-1,024991831**
chrIV	125012	125174	162	0,122531105	0,151940653	0,446425184	-0,134698361	-0,114493607	-0,106641519	-0,232641519	**-1,492641519**
chrIV	126907	127064	157	5,308731367	3,876339579	0,57797391	0,196712944	0,167206002	0,189707939	0,098707939	**-1,161292061**
chrIV	126907	127064	157	5,308731367	3,876339579	0,57797391	0,196712944	0,167206002	0,189707939	0,098707939	**-1,161292061**
chrIV	127133	127254	121	0,059840772	0,285351958	0,173354671	-0,940991251	-0,799842563	-0,239539227	-0,078539227	**-1,338539227**
chrIV	130391	130540	149	0,139628469	0,114881957	0,54861591	0,122165207	0,103840426	-0,076628326	-0,111628326	**-1,371628326**
chrIV	134454	134627	173	1,060036537	1,108055004	0,488926098	-0,027761722	-0,023597463	0,239164791	0,036164791	**-1,223835209**
chrIV	137744	137906	162	0,512920905	0,533645219	0,490098899	-0,024820929	-0,021097789	-0,012042036	-0,138042036	**-1,398042036**
chrIV	147424	147558	134	2,570303646	1,130290221	0,69456518	0,508832496	0,432507621	0,484370843	0,554370843	**-0,705629157**
chrIV	148138	148259	121	0,837770812	1,360054135	0,381181774	-0,302378494	-0,25702172	0,285254248	0,446254248	**-0,813745752**
chrIV	148138	148287	149	7,619725	4,57674893	0,624748189	0,317975367	0,270279062	0,086153719	0,051153719	**-1,208846281**
chrIV	148147	148256	109	0,065539893	0,251999131	0,206399492	-0,818977968	-0,696131272	-0,107393819	0,137606181	**-1,122393819**
chrIV	148147	148259	112	11,95105709	41,96526711	0,221659345	-0,766601154	-0,651610981	-0,105336115	0,118663885	**-1,141336115**
chrIV	148147	148287	140	1,31934655	1,93075805	0,40593972	-0,238002134	-0,202301814	-0,333186748	-0,305186748	**-1,565186748**
chrIV	148150	148259	109	0,028495606	0,08894087	0,242646977	-0,69781331	-0,593141313	-0,009588753	0,235411247	**-1,024588753**
chrIV	148662	148839	177	0,139628469	0,17788174	0,439760565	-0,151576297	-0,128839852	0,204776147	-0,026223853	**-1,286223853**
chrIV	150319	150438	119	2,288197149	11,37331374	0,167492246	-0,964122541	-0,81950416	-0,268528407	-0,093528407	**-1,353528407**
chrIV	150791	150954	163	0,692443222	0,96352609	0,418149791	-0,206629036	-0,175634681	-0,145821231	-0,278821231	**-1,538821231**
chrIV	151146	151295	149	1,305098747	0,70411522	0,649556876	0,384124399	0,326505739	0,145355341	0,110355341	**-1,149644659**
chrIV	154011	154153	142	0,042743409	0,081529131	0,343948944	-0,401709424	-0,34145301	-0,503524939	-0,489524939	**-1,749524939**
chrIV	156456	156616	160	0,125380666	0,151940653	0,452113333	-0,120323781	-0,102275214	-0,100583844	-0,212583844	**-1,472583844**
chrIV	156781	156930	149	0,658248495	0,52623348	0,555726899	0,140144018	0,119122416	-0,065907042	-0,100907042	**-1,360907042**
chrIV	158096	158254	158	0,567062556	0,544762828	0,510028431	0,025140196	0,021369167	0,014869717	-0,083130283	**-1,343130283**
chrIV	158096	158254	158	0,567062556	0,544762828	0,510028431	0,025140196	0,021369167	0,014869717	-0,083130283	**-1,343130283**
chrIV	158496	158646	150	0,105433742	0,059293913	0,640048823	0,358589298	0,304800903	0,195796353	0,153796353	**-1,106203647**
chrIV	162837	162965	128	1,211063248	1,93816979	0,384558155	-0,293530996	-0,249501347	0,012581614	0,124581614	**-1,135418386**
chrIV	163802	163934	132	1,236709293	1,14140783	0,520037168	0,050246866	0,042709836	0,141690088	0,225690088	**-1,034309912**
chrIV	163887	164037	150	0,53571739	0,26682261	0,667527338	0,433095594	0,368131255	0,272521266	0,230521266	**-1,029478734**
chrIV	163887	164037	150	0,53571739	0,26682261	0,667527338	0,433095594	0,368131255	0,272521266	0,230521266	**-1,029478734**
chrIV	164671	164820	149	0,920408068	0,552174567	0,625029826	0,318718021	0,270910318	0,087536287	0,052536287	**-1,207463713**
chrIV	168971	169122	151	0,450230572	0,229763914	0,662109151	0,418226261	0,355492322	0,303711889	0,254711889	**-1,005288111**
chrIV	180947	181107	160	0,042743409	0,037058696	0,535617563	0,08939893	0,07598909	0,078506457	-0,033493543	**-1,293493543**
chrIV	185030	185179	149	3,453667427	2,327286095	0,59742176	0,246679232	0,209677347	0,031572531	-0,003427469	**-1,263427469**
chrIV	185042	185156	114	0,09973462	0,448410219	0,181949392	-0,90796108	-0,771766918	-0,230167402	-0,020167402	**-1,280167402**
chrIV	196778	196946	168	0,065539893	0,044470435	0,595761274	0,242390685	0,206032083	0,364632468	0,196632468	**-1,063367532**
chrIV	198969	199115	146	0,834921251	0,400233914	0,675964668	0,456444093	0,387977479	0,192389934	0,178389934	**-1,081610066**
chrIV	200831	200925	94	0,159575393	0,96723196	0,14161728	-1,073081478	-0,912119256	-0,491682972	-0,141682972	**-1,401682972**
chrIV	202938	203097	159	0,108283302	0,163058261	0,399066404	-0,255764342	-0,217399691	-0,196079247	-0,301079247	**-1,561079247**
chrIV	204891	205041	150	1,70688679	1,104349134	0,607165971	0,271940125	0,231149106	0,137165497	0,095165497	**-1,164834503**
chrIV	205789	205917	128	0,387540239	0,396528045	0,49426848	-0,014367283	-0,012212191	0,233289463	0,345289463	**-0,914710537**
chrIV	214844	215003	159	0,04844253	0,055588044	0,465656665	-0,08619258	-0,073263693	-0,061304769	-0,166304769	**-1,426304769**
chrIV	224519	224676	157	0,558513874	0,515115871	0,52021088	0,050682852	0,043080425	0,059131226	-0,031868774	**-1,291868774**
chrIV	224519	224676	157	0,558513874	0,515115871	0,52021088	0,050682852	0,043080425	0,059131226	-0,031868774	**-1,291868774**
chrIV	228430	228579	149	0,606956404	0,578115654	0,512168353	0,030506268	0,025930328	-0,147284036	-0,182284036	**-1,442284036**
chrIV	230300	230458	158	0,04844253	0,051882174	0,482857442	-0,042983253	-0,036535765	-0,045873142	-0,143873142	**-1,403873142**
chrIV	235048	235188	140	1,079983461	0,637409568	0,628850498	0,328810397	0,279488837	0,147889203	0,175889203	**-1,084110797**
chrIV	235556	235704	148	1,131275552	0,804173699	0,584502824	0,213426448	0,181412481	0,005979333	-0,022020667	**-1,282020667**
chrIV	235556	235704	148	1,131275552	0,804173699	0,584502824	0,213426448	0,181412481	0,005979333	-0,022020667	**-1,282020667**
chrIV	240743	240895	152	0,14532759	0,037058696	0,796812048	0,83028812	0,705744902	0,587609955	0,531609955	**-0,728390045**
chrIV	241083	241236	153	0,142478029	0,17788174	0,444743825	-0,138952542	-0,118109661	-0,185388003	-0,248388003	**-1,508388003**
chrIV	243252	243404	152	0,327699467	0,481763045	0,404835878	-0,240849509	-0,204722082	-0,319774479	-0,375774479	**-1,635774479**
chrIV	247954	248116	162	0,276407377	0,544762828	0,336601809	-0,421755324	-0,358492025	-0,343018586	-0,469018586	**-1,729018586**
chrIV	249148	249302	154	0,079787696	0,066705652	0,544650641	0,112157259	0,09533367	0,004636672	-0,065363328	**-1,325363328**
chrIV	249297	249444	147	0,071239015	0,051882174	0,578608892	0,198335964	0,168585569	-0,002329213	-0,023329213	**-1,283329213**
chrIV	253799	253940	141	0,139628469	0,081529131	0,631352796	0,335438395	0,285122636	0,125831948	0,146831948	**-1,113168052**
chrIV	262881	263029	148	0,054141651	0,048176305	0,529151025	0,07313593	0,062165541	-0,12841368	-0,15641368	**-1,41641368**
chrIV	268349	268491	142	1,951949	0,893114568	0,686082737	0,484777019	0,412060466	0,248688773	0,262688773	**-0,997311227**
chrIV	269754	269886	132	0,567062556	0,503998263	0,529440108	0,073862515	0,062783138	0,176711988	0,260711988	**-0,999288012**
chrIV	275399	275562	163	0,606956404	0,681880002	0,470933628	-0,072923171	-0,061984696	-0,029105141	-0,162105141	**-1,422105141**
chrIV	277085	277203	118	0,042743409	0,322410653	0,117055822	-1,189834	-1,0113589	-0,464351584	-0,282351584	**-1,542351584**
chrIV	281092	281244	152	0,227964847	0,244587392	0,482411949	-0,044100996	-0,037485847	-0,157127037	-0,213127037	**-1,473127037**
chrIV	282751	282862	111	0,034194727	0,303881305	0,101145079	-1,275053919	-1,083795831	-0,560919947	-0,329919947	**-1,589919947**
chrIV	287334	287498	164	0,846319493	0,893114568	0,486548764	-0,033723639	-0,028665093	0,026443501	-0,113556499	**-1,373556499**
chrIV	290503	290642	139	1,399134247	0,922761525	0,60258271	0,260037847	0,22103217	0,12500996	0,16000996	**-1,09999004**
chrIV	291728	291875	147	0,082637257	0,037058696	0,690393076	0,496964836	0,42242011	0,257577313	0,236577313	**-1,023422687**
chrIV	294716	294818	102	0,122531105	0,715232829	0,146259704	-1,052610794	-0,894719174	-0,374369689	-0,080369689	**-1,340369689**
chrIV	303242	303392	150	0,336248149	0,274234349	0,550790809	0,127659576	0,10851064	0,026362858	-0,015637142	**-1,275637142**
chrIV	305394	305543	149	0,63545201	0,785644351	0,447156173	-0,132849576	-0,112922139	-0,275453476	-0,310453476	**-1,570453476**
chrIV	307871	308025	154	0,381841118	0,392822175	0,492912368	-0,017766994	-0,015101945	-0,108235216	-0,178235216	**-1,438235216**
chrIV	308997	309152	155	0,889062902	0,774526742	0,534424403	0,086396544	0,073437062	-0,022995753	-0,099995753	**-1,359995753**
chrIV	311358	311498	140	0,658248495	0,607762611	0,519938958	0,050000381	0,042500324	-0,092086032	-0,064086032	**-1,324086032**
chrIV	313115	313272	157	0,04844253	0,037058696	0,566571175	0,16765125	0,142503563	0,16365864	0,07265864	**-1,18734136**
chrIV	315132	315251	119	0,085486818	0,125999566	0,404219015	-0,242441566	-0,206075331	0,334562048	0,509562048	**-0,750437952**
chrIV	316257	316381	124	0,136778908	0,218646305	0,384831753	-0,292815072	-0,248892811	0,162674824	0,302674824	**-0,957325176**
chrIV	317932	318025	93	2,225506816	8,186265899	0,213749078	-0,793480106	-0,67445809	-0,311586684	0,045413316	**-1,214586684**
chrIV	320773	320934	161	0,327699467	0,329822392	0,498385665	-0,004046557	-0,003439573	0,01191987	-0,10708013	**-1,36708013**
chrIV	324041	324187	146	0,361894194	0,255705001	0,585969342	0,217188668	0,184610368	-0,011155009	-0,025155009	**-1,285155009**
chrIV	326087	326232	145	0,071239015	0,055588044	0,561702018	0,155285859	0,13199298	-0,038473147	-0,045473147	**-1,305473147**
chrIV	331066	331232	166	0,034194727	0,044470435	0,434687048	-0,164453569	-0,139785534	-0,021411356	-0,175411356	**-1,435411356**
chrIV	331066	331232	166	0,034194727	0,044470435	0,434687048	-0,164453569	-0,139785534	-0,021411356	-0,175411356	**-1,435411356**
chrIV	336500	336644	144	0,738036191	0,585527393	0,557612947	0,144919911	0,123181924	-0,009088495	-0,009088495	**-1,269088495**
chrIV	339003	339155	152	0,051292091	0,051882174	0,497140355	-0,007168129	-0,00609291	-0,113959585	-0,169959585	**-1,429959585**
chrIV	339626	339796	170	0,159575393	0,244587392	0,394829506	-0,266753431	-0,226740416	-0,015911274	-0,197911274	**-1,457911274**
chrIV	343754	343879	125	0,53856695	1,500877179	0,264075364	-0,630831465	-0,536206745	-0,136432816	-0,003432816	**-1,263432816**

Chrom	Start	End	Length	Section A	Section B	A/A+B	Z-score	Z * 0.85	Phase correction	Length correction	ΔLKnuc
chrIV	344625	344793	168	0,034194727	0,040764565	0,456177292	-0,110069087	-0,093558724	0,074357169	-0,093642831	**-1,353642831**
chrIV	344904	345057	153	1,932002076	1,133996091	0,63013804	0,332218971	0,282386125	0,213629633	0,150629633	**-1,109370367**
chrIV	346054	346184	130	0,498673102	0,629997828	0,441823288	-0,146348124	-0,124395906	0,005861123	0,103861123	**-1,156138877**
chrIV	346695	346849	154	0,304902982	0,292763697	0,510155565	0,025458977	0,02164013	-0,073788246	-0,143788246	**-1,403788246**
chrIV	354236	354370	134	0,233663968	0,214940436	0,520868645	0,052333815	0,044483743	0,083318708	0,153318708	**-1,106681292**
chrIV	357373	357527	154	0,398938482	0,244587392	0,619926094	0,305286692	0,259493688	0,148148738	0,078148738	**-1,181851262**
chrIV	357519	357616	97	0,14532759	1,389701092	0,094674185	-1,31250924	-1,115632854	-0,665323564	-0,336323564	**-1,596323564**
chrIV	367261	367414	153	2,348037921	2,097522181	0,528175948	0,070685446	0,060082629	-0,015313528	-0,078313528	**-1,338313528**
chrIV	369734	369884	150	0,572761677	0,78935022	0,420495319	-0,200626496	-0,170532521	-0,257769134	-0,299769134	**-1,559769134**
chrIV	372733	372859	126	0,125380666	0,552174567	0,185048627	-0,896291207	-0,761847526	-0,457174564	-0,331174564	**-1,591174564**
chrIV	377302	377457	155	0,393239361	0,555880437	0,414320049	-0,216446021	-0,183979117	-0,265995297	-0,342995297	**-1,602995297**
chrIV	377961	378102	141	0,102584181	0,074117392	0,58055047	0,203301913	0,172806626	0,002616803	0,023616803	**-1,236383197**
chrIV	379551	379707	156	0,373292436	0,407645654	0,47800516	-0,055160849	-0,046886722	-0,006401121	-0,090401121	**-1,350401121**
chrIV	380490	380611	121	0,142478029	0,548468698	0,206206967	-0,819653028	-0,696705073	-0,163833261	-0,002833261	**-1,262833261**
chrIV	380895	381031	136	0,287805619	0,251999131	0,533166147	0,083231199	0,070746519	0,057012071	0,113012071	**-1,146987929**
chrIV	383984	384136	152	0,589859041	0,270528479	0,685573683	0,483342407	0,410841046	0,304802025	0,248802025	**-1,011197975**
chrIV	384406	384547	141	1,17401896	1,115466743	0,512787199	0,032058244	0,027249508	-0,144858974	-0,123858974	**-1,383858974**
chrIV	386414	386562	148	0,105433742	0,040764565	0,721169375	0,586318873	0,498371042	0,306521717	0,278521717	**-0,981478283**
chrIV	389563	389664	101	0,054141651	0,426175002	0,112720745	-1,212185213	-1,030357431	-0,544448267	-0,243448267	**-1,503448267**
chrIV	394898	395059	161	0,282106498	0,303881305	0,481420426	-0,046588934	-0,039600594	-0,021132814	-0,140132814	**-1,400132814**
chrIV	395659	395798	139	0,612655525	0,511410002	0,545035419	0,113127892	0,096158708	-0,007800588	0,027199412	**-1,232800588**
chrIV	405838	405992	154	2,037435817	1,523112397	0,572225313	0,182042499	0,154736124	0,048614901	-0,021385099	**-1,281385099**
chrIV	406138	406284	146	0,82922213	0,311293045	0,72705927	0,603943118	0,51335165	0,321241964	0,307241964	**-0,952758036**
chrIV	407097	407258	161	0,159575393	0,100058479	0,614617006	0,291373097	0,247667133	0,259288758	0,140288758	**-1,119711242**
chrIV	407639	407774	135	3,040481143	2,664520227	0,532950116	0,082687823	0,070284649	0,063855797	0,126855797	**-1,133144203**
chrIV	410001	410147	146	0,085486818	0,100058479	0,460732874	-0,09858756	-0,083799426	-0,283684068	-0,297684068	**-1,557684068**
chrIV	412510	412666	156	0,421734966	0,88199696	0,323482886	-0,457981444	-0,389284228	-0,345284093	-0,429284093	**-1,689284093**
chrIV	414505	414611	106	0,108283302	0,389116306	0,217698809	-0,779988545	-0,662990263	-0,1449347	0,1210653	**-1,1389347**
chrIV	414505	414627	122	0,108283302	0,43729261	0,198475225	-0,847080146	-0,720018124	-0,203091331	-0,049091331	**-1,309091331**
chrIV	417857	418015	158	0,58700948	0,655938915	0,472271803	-0,069560337	-0,059126287	-0,054643102	-0,152643102	**-1,412643102**
chrIV	418210	418363	153	0,270708255	0,237175653	0,533012074	0,082843661	0,070417112	0,001423578	-0,061576422	**-1,321576422**
chrIV	418328	418478	150	0,076938136	0,100058479	0,434687048	-0,164453569	-0,139785534	-0,239852845	-0,281852845	**-1,541852845**
chrIV	419977	420127	150	0,056991212	0,062999783	0,474962408	-0,062801193	-0,053381014	-0,148435292	-0,190435292	**-1,450435292**
chrIV	430775	430925	150	0,079787696	0,066705652	0,544650641	0,112157259	0,09533367	-0,003814201	-0,045814201	**-1,305814201**
chrIV	431218	431366	148	1,265204899	0,989467177	0,561148077	0,153880643	0,130798547	-0,06736047	-0,09536047	**-1,35536047**
chrIV	433624	433778	154	5,154855096	3,980103927	0,56429975	0,161879814	0,137597842	0,037578162	-0,032421838	**-1,292421838**
chrIV	444592	444742	150	0,179522317	0,114881957	0,609781626	0,278749922	0,236937434	0,141476159	0,099476159	**-1,160523841**
chrIV	447153	447303	150	1,860763061	1,252583918	0,597672882	0,2473282	0,21022897	0,130187425	0,088187425	**-1,171812575**
chrIV	452119	452270	151	0,247911771	0,281646088	0,468148601	-0,079924628	-0,067935934	-0,118081433	-0,167081433	**-1,427081433**
chrIV	456027	456181	154	1,114178188	0,678174133	0,621629004	0,309761946	0,263297654	0,164999118	0,094999118	**-1,165000882**
chrIV	464196	464345	149	0,350495952	0,277940218	0,55772721	0,145209355	0,123427951	-0,040150598	-0,075150598	**-1,335150598**
chrIV	465523	465668	145	0,287805619	0,200116957	0,589859197	0,227182794	0,193105375	0,028778533	0,021778533	**-1,238221467**
chrIV	472028	472162	134	1,649895578	1,222936961	0,574309695	0,187357158	0,159253584	0,184583951	0,254583951	**-1,005416049**
chrIV	480768	480918	150	0,356195073	0,292763697	0,548871653	0,122811089	0,104389426	0,019671842	-0,022328158	**-1,282328158**
chrIV	481714	481864	150	0,960301917	0,559586306	0,631824039	0,336688251	0,286185013	0,207850393	0,165850393	**-1,094149607**
chrIV	481903	482061	158	0,059840772	0,055588044	0,518421434	0,046192108	0,039262392	0,051683921	-0,046316079	**-1,306316079**
chrIV	487395	487546	151	0,247911771	0,222352175	0,527175798	0,068172392	0,057946533	0,00069642	-0,04830358	**-1,30830358**
chrIV	488401	488552	151	0,478726178	0,318704784	0,600335579	0,254215803	0,216083433	0,143503378	0,094503378	**-1,165496622**
chrIV	488508	488615	107	0,054141651	0,140823044	0,277699771	-0,589688448	-0,501235181	0,056980382	0,315980382	**-0,944019618**
chrIV	488508	488632	124	0,054141651	0,203822827	0,209880258	-0,806836798	-0,685811279	-0,283800432	-0,143800432	**-1,403800432**
chrIV	491144	491308	164	0,159575393	0,185293479	0,462713239	-0,093600543	-0,079560461	-0,022663446	-0,162663446	**-1,422663446**
chrIV	491144	491308	164	0,159575393	0,185293479	0,462713239	-0,093600543	-0,079560461	-0,022663446	-0,162663446	**-1,422663446**
chrIV	492176	492332	156	1,12272687	0,930173264	0,546897948	0,117827796	0,100153627	0,154909575	0,070909575	**-1,189090425**
chrIV	494125	494275	150	0,19661968	0,111176087	0,638799168	0,355250862	0,301963233	0,213833903	0,171833903	**-1,088166097**
chrIV	499197	499355	158	0,162424953	0,077823261	0,676071427	0,456741101	0,388229936	0,395689628	0,297689628	**-0,962310372**
chrIV	499814	499967	153	0,102584181	0,118587827	0,463820816	-0,090812431	-0,077190567	-0,142156544	-0,205156544	**-1,465156544**
chrIV	503596	503736	140	0,185221438	0,107470218	0,63282104	0,339334281	0,288434139	0,164015832	0,192015832	**-1,067984168**
chrIV	503768	503924	156	0,034194727	0,040764565	0,456177292	-0,110069087	-0,093558724	-0,042951835	-0,126951835	**-1,386951835**
chrIV	508653	508795	142	0,669646737	0,363175219	0,648366094	0,380912991	0,323776043	0,167412466	0,181412466	**-1,078587534**
chrIV	513880	514029	149	0,091185939	0,051882174	0,637360324	0,351411905	0,298700119	0,141571865	0,106571865	**-1,153428135**
chrIV	517444	517573	129	0,208017923	0,340940001	0,378932362	-0,308285993	-0,262043094	-0,02776644	0,07723356	**-1,18276644**
chrIV	518819	518929	110	0,151026711	0,737468046	0,169980419	-0,954242636	-0,81110624	-0,219534086	0,018465914	**-1,241534086**
chrIV	521357	521482	125	1,581506124	4,13204458	0,276799175	-0,592376721	-0,503520213	-0,112691432	0,020308568	**-1,239691432**
chrIV	522483	522574	91	0,407487163	1,004290656	0,28863406	-0,557379572	-0,473772636	-0,145891693	0,225108307	**-1,034891693**
chrIV	522691	522836	145	0,359044634	0,166764131	0,682842618	0,475662609	0,404313218	0,23553621	0,22853621	**-1,03146379**
chrIV	524988	525151	163	0,09973462	0,062999783	0,61286746	0,286800501	0,243780426	0,28443055	0,15143055	**-1,10856945**
chrIV	525488	525645	157	0,510071344	0,559586306	0,476854762	-0,058049093	-0,049341729	-0,045523345	-0,136523345	**-1,396523345**
chrIV	527030	527163	133	1,450426337	1,371171744	0,514044274	0,035211049	0,029929391	0,123047456	0,200047456	**-1,059952544**
chrIV	527878	528014	136	0,056991212	0,059293913	0,490098899	-0,024820929	-0,021097789	-0,036376702	0,019623298	**-1,240376702**
chrIV	528421	528544	123	0,039893848	0,044470435	0,472876041	-0,06804215	-0,057835827	0,429844903	0,576844903	**-0,683155097**
chrIV	528421	528548	127	6,39726351	8,26408916	0,43633515	-0,160267576	-0,13622744	0,163004468	0,282004468	**-0,977995532**
chrIV	529656	529804	148	0,227964847	0,159352392	0,588573975	0,223878237	0,190296502	-0,015964923	-0,043964923	**-1,303964923**
chrIV	529731	529886	155	0,085486818	0,085235	0,500737507	0,001848658	0,001571359	-0,082662276	-0,159662276	**-1,419662276**
chrIV	530359	530519	160	0,056991212	0,040764565	0,582995843	0,209563572	0,178129036	0,188408754	0,076408754	**-1,183591246**
chrIV	531763	531899	136	0,071239015	0,066705652	0,516431814	0,041200102	0,035020087	0,0180789	0,0740789	**-1,1859211**
chrIV	532306	532429	123	0,042743409	0,062999783	0,404219015	-0,242441566	-0,206075331	0,266195855	0,413195855	**-0,846804145**
chrIV	532306	532433	127	6,414360873	8,293736117	0,436110863	-0,160837072	-0,136711511	0,166145066	0,285145066	**-0,974854934**
chrIV	533541	533689	148	0,225115286	0,200116957	0,529393736	0,07374596	0,062684066	-0,140567679	-0,168567679	**-1,428567679**
chrIV	533616	533771	155	0,136778908	0,077823261	0,637360324	0,351411905	0,298700119	0,225537375	0,148537375	**-1,111462625**
chrIV	534244	534404	160	0,065539893	0,055588044	0,541079911	0,103154718	0,08768151	0,101896699	-0,010103301	**-1,270103301**
chrIV	535648	535784	136	0,065539893	0,051882174	0,558156527	0,146296985	0,124352437	0,107449203	0,163449203	**-1,096550797**
chrIV	536109	536229	120	1,66129382	9,805730905	0,144875751	-1,058666911	-0,899866875	-0,341892143	-0,173892143	**-1,433892143**
chrIV	537426	537574	148	0,205168362	0,129705435	0,612673681	0,286294411	0,24335025	0,040540711	0,012540711	**-1,247459289**
chrIV	537501	537656	155	0,111132863	0,070411522	0,612152576	0,284933816	0,242193743	0,177657373	0,100657373	**-1,159342627**
chrIV	540906	541052	146	0,051292091	0,066705652	0,434687048	-0,164453569	-0,139785534	-0,337192237	-0,351192237	**-1,611192237**
chrIV	541731	541872	141	0,364743755	0,166764131	0,686243355	0,485229878	0,412445397	0,245874374	0,266874374	**-0,993125626**
chrIV	545183	545329	146	3,61609238	1,830699572	0,663893979	0,423114063	0,359646953	0,162085357	0,148085357	**-1,111914643**
chrIV	549706	549856	150	0,24506221	0,292763697	0,455653413	-0,111390332	-0,094681782	-0,182719952	-0,224719952	**-1,484719952**
chrIV	549706	549856	150	0,24506221	0,292763697	0,455653413	-0,111390332	-0,094681782	-0,182719952	-0,224719952	**-1,484719952**
chrIV	552005	552154	149	0,279256937	0,207528696	0,573675388	0,185739279	0,157878387	-0,002012773	-0,037012773	**-1,297012773**
chrIV	557304	557456	152	0,136778908	0,077823261	0,637360324	0,351411905	0,298700119	0,189460635	0,133460635	**-1,126539365**
chrIV	560465	560600	135	0,435982769	0,344645871	0,558502144	0,147172696	0,125096791	0,088646014	0,151646014	**-1,108353986**
chrIV	570638	570778	140	0,131079787	0,100058479	0,567105523	0,169009772	0,143658307	0,019324095	0,047324095	**-1,212675905**
chrIV	570638	570778	140	0,131079787	0,100058479	0,567105523	0,169009772	0,143658307	0,019324095	0,047324095	**-1,212675905**
chrIV	574209	574359	150	0,170973635	0,107470218	0,614032715	0,289845327	0,246368528	0,16875484	0,12675484	**-1,13324516**
chrIV	575376	575525	149	0,381841118	0,344645871	0,52559939	0,064212254	0,054580416	-0,102404726	-0,137404726	**-1,397404726**

Chrom	Start	End	Length	Section A	Section B	A/A+B	Z-score	Z * 0.85	Phase correction	Length correction	ΔLKnuc
chrIV	585684	585843	159	0,19377012	0,100058479	0,659466508	0,41100757	0,349356434	0,35877657	0,25377657	**-1,00622343**
chrIV	587983	588137	154	1,000195765	0,696703481	0,589425546	0,226067518	0,19215739	0,084461587	0,014461587	**-1,245538413**
chrIV	601658	601795	137	0,444531451	0,17417587	0,718484226	0,578344513	0,491592836	0,460089359	0,509089359	**-0,750910641**
chrIV	607967	608120	153	0,059840772	0,077823261	0,434687048	-0,164453569	-0,139785534	-0,212272632	-0,275272632	**-1,535272632**
chrIV	615631	615766	135	0,034194727	0,092646739	0,269586342	-0,614064532	-0,521954852	-0,570681315	-0,507681315	**-1,767681315**
chrIV	618044	618203	159	0,601257283	0,563292176	0,516300341	0,040870272	0,034739731	0,043236421	-0,061763579	**-1,321763579**
chrIV	625810	625961	151	0,136778908	0,140823044	0,492715945	-0,018259434	-0,015520519	-0,084621247	-0,133621247	**-1,393621247**
chrIV	640754	640908	154	7,152397065	4,154279798	0,632581717	0,338698905	0,287894069	0,170648784	0,100648784	**-1,159351216**
chrIV	643481	643635	154	2,672887827	2,012287181	0,570499036	0,177644815	0,150998092	0,03058229	-0,03941771	**-1,29941771**
chrIV	645695	645844	149	0,09973462	0,062999783	0,61286746	0,286800501	0,243780426	0,095451835	0,060451835	**-1,199548165**
chrIV	646491	646637	146	0,074088575	0,062999783	0,540443961	0,101552255	0,086319417	-0,102936315	-0,116936315	**-1,376936315**
chrIV	647130	647280	150	0,447381012	0,400233914	0,527811625	0,069769969	0,059304474	-0,026296514	-0,068296514	**-1,328296514**
chrIV	648526	648683	157	0,076938136	0,037058696	0,674914686	0,453525163	0,385496389	0,393714289	0,302714289	**-0,957285711**
chrIV	648994	649155	161	0,071239015	0,074117392	0,490098899	-0,024820929	-0,021097789	-0,018407214	-0,137407214	**-1,397407214**
chrIV	649174	649309	135	0,105433742	0,066705652	0,612490489	0,28581604	0,242943634	0,192964386	0,255964386	**-1,004035614**
chrIV	650212	650352	140	0,165274514	0,122293696	0,574731518	0,188433343	0,160168342	0,035811504	0,063811504	**-1,196188496**
chrIV	650218	650352	134	0,125380666	0,118587827	0,51392155	0,034903236	0,029667751	0,044452902	0,114452902	**-1,145547098**
chrIV	650630	650785	155	0,108283302	0,077823261	0,581834946	0,206589951	0,175601459	0,114175556	0,037175556	**-1,222824444**
chrIV	650761	650869	108	0,088336378	0,166764131	0,346280681	-0,395381497	-0,336074272	0,261766643	0,513766643	**-0,746233357**
chrIV	654067	654214	147	1,883559546	0,97093783	0,659856815	0,412072382	0,350261525	0,185407766	0,164407766	**-1,095592234**
chrIV	654067	654214	147	1,883559546	0,97093783	0,659856815	0,412072382	0,350261525	0,185407766	0,164407766	**-1,095592234**
chrIV	656839	656998	159	0,302053422	0,214940436	0,584249537	0,212776972	0,180860426	0,187677433	0,082677433	**-1,177322567**
chrIV	658487	658641	154	0,333398588	0,389116306	0,461441821	-0,096801992	-0,082281694	-0,209803086	-0,279803086	**-1,539803086**
chrIV	658600	658747	147	0,444531451	0,359469349	0,552899264	0,13298977	0,113041304	-0,040267512	-0,061267512	**-1,321267512**
chrIV	662245	662401	156	0,34194727	0,281646088	0,548349763	0,121493107	0,103269141	0,142831041	0,058831041	**-1,201168959**
chrIV	665084	665248	164	0,04844253	0,085235	0,36238349	-0,352095051	-0,299280793	-0,237505983	-0,377505983	**-1,637505983**
chrIV	665597	665732	135	0,649699813	0,670762394	0,492024542	-0,019992839	-0,016993913	-0,06501557	-0,00201557	**-1,26201557**
chrIV	666941	667094	153	1,22246149	1,115466743	0,522882385	0,057389119	0,048780751	-0,021306476	-0,084306476	**-1,344306476**
chrIV	666948	667109	161	0,094035499	0,140823044	0,400392074	-0,252332398	-0,214482538	-0,210752762	-0,329752762	**-1,589752762**
chrIV	669497	669656	159	0,492973981	0,52623348	0,483683646	-0,040910443	-0,034773876	-0,028388218	-0,133388218	**-1,393388218**
chrIV	669741	669906	165	0,48727486	0,930173264	0,343769096	-0,402198167	-0,341868442	-0,256900623	-0,403900623	**-1,663900623**
chrIV	677555	677687	132	0,581310359	1,167348917	0,332432033	-0,43320745	-0,368226333	-0,264701538	-0,180701538	**-1,440701538**
chrIV	682440	682595	155	0,076938136	0,044470435	0,633712557	0,341702373	0,290447017	0,237390633	0,160390633	**-1,099609367**
chrIV	682928	683076	148	0,646850252	0,452116089	0,588598784	0,223942004	0,190350703	-0,011709395	-0,039709395	**-1,299709395**
chrIV	684905	685053	148	0,259310013	0,207528696	0,555459536	0,139467258	0,118547169	-0,08843622	-0,11643622	**-1,37643622**
chrIV	685367	685521	154	0,253610892	0,122293696	0,674668254	0,452840646	0,384914549	0,25646396	0,18646396	**-1,07353604**
chrIV	685409	685539	130	0,282106498	0,359469349	0,439708725	-0,151707742	-0,128951581	-0,012834812	0,085165188	**-1,174834812**
chrIV	692976	693125	149	0,062690333	0,037058696	0,628480635	0,327831948	0,278657156	0,133180597	0,098180597	**-1,161819403**
chrIV	699427	699543	116	0,262159574	1,015408265	0,205202077	-0,823182615	-0,699705223	-0,142881546	0,053118454	**-1,206881546**
chrIV	699429	699572	143	0,14817715	0,070411522	0,677881195	0,461782069	0,392514759	0,235146202	0,242146202	**-1,017853798**
chrIV	701924	702080	156	0,113982423	0,148234783	0,434687048	-0,164453569	-0,139785534	-0,101347585	-0,185347585	**-1,445347585**
chrIV	705337	705438	101	0,14532759	1,096937395	0,116985982	-1,190189384	-1,011660976	-0,535147377	-0,234147377	**-1,494147377**
chrIV	705533	705696	163	0,131079787	0,111176087	0,541079911	0,103154718	0,08768151	0,123220117	-0,009779883	**-1,269779883**
chrIV	709396	709543	147	0,407487163	0,389116306	0,511530742	0,028907309	0,024571212	-0,133813573	-0,154813573	**-1,414813573**
chrIV	710394	710553	159	1,250957096	1,267407396	0,496733932	-0,00818691	-0,006958874	-0,005386623	-0,110386623	**-1,370386623**
chrIV	710648	710758	110	0,418885406	0,463233697	0,47486264	-0,063051771	-0,053594005	0,534241165	0,772241165	**-0,487758835**
chrIV	711680	711827	147	0,262159574	0,255705001	0,506231912	0,015621722	0,013278464	-0,154110026	-0,175110026	**-1,435110026**
chrIV	712887	713032	145	2,159966922	1,337818918	0,617524063	0,298984497	0,254136822	0,068779988	0,061779988	**-1,198220012**
chrIV	717311	717466	155	0,14532759	0,218646305	0,399280256	-0,255210514	-0,216928937	-0,256478262	-0,333478262	**-1,593478262**
chrIV	723685	723835	150	0,094035499	0,074117392	0,559226183	0,149007602	0,126656462	0,034925894	-0,007074106	**-1,267074106**
chrIV	724692	724830	138	0,324849907	0,370586958	0,467116317	-0,082520731	-0,070142622	-0,139829463	-0,097829463	**-1,357829463**
chrIV	727870	728018	148	0,87766466	0,663350655	0,569536624	0,175194569	0,148915383	-0,056269525	-0,084269525	**-1,344269525**
chrIV	729848	729954	106	0,037044288	0,163058261	0,185126515	-0,8959995	-0,761599575	-0,227131484	0,038868516	**-1,221131484**
chrIV	729848	729970	122	0,071239015	0,270528479	0,208442921	-0,811835827	-0,690060453	-0,19237542	-0,03837542	**-1,29837542**
chrIV	730903	730997	94	0,558513874	2,20128653	0,202374735	-0,833168919	-0,708193581	-0,267649955	0,082350045	**-1,177649955**
chrIV	734743	734893	150	0,079787696	0,144528914	0,355692324	-0,369997104	-0,314497538	-0,401921558	-0,443921558	**-1,703921558**
chrIV	736895	737045	150	0,24221265	0,200116957	0,547584077	0,119559826	0,101625852	0,01731623	-0,02468377	**-1,28468377**
chrIV	737420	737548	128	0,359044634	0,522527611	0,407277606	-0,234553618	-0,199370575	0,035744442	0,147744442	**-1,112255558**
chrIV	737638	737793	155	0,133929347	0,200116957	0,400930486	-0,250939386	-0,213298478	-0,257408687	-0,334408687	**-1,594408687**
chrIV	745330	745443	113	2,020338454	17,69923311	0,102453466	-1,267694866	-1,077540653	-0,526221438	-0,309221438	**-1,569221438**
chrIV	747582	747734	152	0,749434433	0,804173699	0,482383181	-0,044173177	-0,0375472	-0,147098449	-0,203098449	**-1,463098449**
chrIV	750682	750850	168	0,079787696	0,148234783	0,349911537	-0,385559308	-0,327725412	-0,15250291	-0,32050291	**-1,58050291**
chrIV	760658	760811	153	0,284956058	0,281646088	0,502920895	0,007321663	0,006223413	-0,074555805	-0,137555805	**-1,397555805**
chrIV	761833	761982	149	0,78362916	0,515115871	0,603374135	0,262090424	0,22277686	0,076154591	0,041154591	**-1,218845409**
chrIV	762005	762157	152	1,461824579	0,848644133	0,632696115	0,339002603	0,288152213	0,185932814	0,129932814	**-1,130067186**
chrIV	764493	764644	151	0,199469241	0,229763914	0,464710702	-0,088572826	-0,075286902	-0,146314455	-0,195314455	**-1,455314455**
chrIV	766598	766749	151	0,182371877	0,129705435	0,58438044	0,213112623	0,181145729	0,105787675	0,056787675	**-1,203212325**
chrIV	768253	768411	158	0,079787696	0,129705435	0,380860679	-0,303221116	-0,257737948	-0,249347271	-0,347347271	**-1,607347271**
chrIV	771149	771299	150	0,424584527	0,285351958	0,598059877	0,248328506	0,21107923	0,123772928	0,081772928	**-1,178227072**
chrIV	773973	774101	128	0,14817715	0,340940001	0,302948179	-0,51593994	-0,438548949	-0,197804847	-0,085804847	**-1,345804847**
chrIV	779473	779647	174	0,034194727	0,125999566	0,213457836	-0,794480647	-0,67530855	-0,379471933	-0,589471933	**-1,849471933**
chrIV	779988	780110	122	0,92895675	3,331576751	0,218037659	-0,778837715	-0,662012058	-0,179242314	-0,025242314	**-1,285242314**
chrIV	785291	785441	150	0,153876272	0,277940218	0,356346446	-0,368241871	-0,31300559	-0,394944994	-0,436944994	**-1,696944994**
chrIV	787217	787366	149	1,191116324	0,822703046	0,591471282	0,231331294	0,1966316	0,047899321	0,012899321	**-1,247100679**
chrIV	787217	787366	149	1,191116324	0,822703046	0,591471282	0,231331294	0,1966316	0,047899321	0,012899321	**-1,247100679**
chrIV	789145	789305	160	0,128230226	0,122293696	0,51184823	0,029703476	0,025247955	0,0383882	-0,0736118	**-1,3336118**
chrIV	800500	800661	161	0,051292091	0,059293913	0,463820816	-0,090812431	-0,077190567	-0,066438319	-0,185438319	**-1,445438319**
chrIV	803067	803219	152	0,139628469	0,181587609	0,434687048	-0,164453569	-0,139785534	-0,238898307	-0,294898307	**-1,554898307**
chrIV	805417	805524	107	0,173823196	0,489174784	0,262177564	-0,636646501	-0,541149525	0,021629282	0,280629282	**-0,979370718**
chrIV	805417	805530	113	0,09973462	0,478057176	0,172613424	-0,943888071	-0,80230486	-0,25002957	-0,03302957	**-1,29302957**
chrIV	810559	810694	135	0,227964847	0,277940218	0,450607955	-0,124125498	-0,105506674	-0,16535258	-0,10235258	**-1,36235258**
chrIV	815236	815393	157	0,071239015	0,066705652	0,516431814	0,041200102	0,035020087	0,052672634	-0,038327366	**-1,298327366**
chrIV	815520	815678	158	0,284956058	0,511410002	0,357820445	-0,364290771	-0,309647155	-0,301962576	-0,399962576	**-1,659962576**
chrIV	826418	826583	165	0,265009134	0,329822392	0,445519651	-0,136989237	-0,116440852	-0,02680278	-0,17380278	**-1,43380278**
chrIV	827506	827652	146	0,97739928	0,400233914	0,709477155	0,551857937	0,469079246	0,282469943	0,268469943	**-0,991530057**
chrIV	835382	835525	143	0,088336378	0,070411522	0,556456986	0,141992377	0,12069352	-0,039362561	-0,032362561	**-1,292362561**
chrIV	835382	835528	146	21,76494374	15,62765201	0,582065602	0,207180626	0,176103532	-0,0059835	-0,0199835	**-1,2799835**
chrIV	835383	835528	145	0,04844253	0,044470435	0,521375354	0,053605728	0,045564869	-0,133809352	-0,140809352	**-1,400809352**
chrIV	835384	835528	144	0,105433742	0,077823261	0,575332675	0,189967434	0,161472318	0,024917168	0,024917168	**-1,235082832**
chrIV	835385	835528	143	0,051292091	0,074117392	0,408996909	-0,230126057	-0,195607148	-0,3334253	-0,3264253	**-1,5864253**
chrIV	835386	835528	142	0,028495606	0,070411522	0,288104674	-0,558930214	-0,475090682	-0,634812245	-0,620812245	**-1,880812245**
chrIV	835932	836065	133	5,018076188	3,409400012	0,595442345	0,241567496	0,205332371	0,274766364	0,351766364	**-0,908233636**
chrIV	836520	836671	151	0,111132863	0,040764565	0,731630971	0,617753167	0,525090192	0,443146629	0,394146629	**-0,865853371**
chrIV	837322	837477	155	3,413773579	2,731225879	0,55553684	0,139662925	0,118713486	0,08656511	0,00956511	**-1,25043489**
chrIV	838477	838608	131	0,302053422	5,870097412	0,048938114	-1,655238008	-1,406952307	-1,302460103	-1,211460103	**-2,471460103**
chrIV	843960	844136	176	0,421734966	0,993173047	0,298065289	-0,529973104	-0,450477139	-0,133694818	-0,357694818	**-1,617694818**

Chrom	Start	End	Length	Section A	Section B	A/A+B	Z-score	Z * 0.85	Phase correction	Length correction	ΔLKnuc
chrIV	844482	844623	141	9,451992456	5,707039151	0,623522181	0,314744522	0,267532844	0,10053779	0,12153779	**-1,13846221**
chrIV	844482	844649	167	0,185221438	0,285351958	0,393607968	-0,269927623	-0,229438479	-0,10155302	-0,26255302	**-1,52255302**
chrIV	850466	850631	165	0,470177496	0,463233697	0,503719582	0,009323745	0,007925183	0,113646055	-0,033353945	**-1,293353945**
chrIV	850466	850631	165	0,470177496	0,463233697	0,503719582	0,009323745	0,007925183	0,113646055	-0,033353945	**-1,293353945**
chrIV	857181	857346	165	0,085486818	0,081529131	0,51184823	0,029703476	0,025247955	0,146205924	-0,000794076	**-1,260794076**
chrIV	861657	861796	139	0,498673102	0,433586741	0,534907844	0,087612945	0,074471003	-0,03451816	0,00048184	**-1,25951816**
chrIV	863651	863788	137	3,476463912	2,920225228	0,54347864	0,109201437	0,092821221	0,063382656	0,112382656	**-1,147617344**
chrIV	866143	866295	152	0,313451664	0,148234783	0,678927584	0,464702061	0,394996752	0,292782287	0,236782287	**-1,023217713**
chrIV	867460	867598	138	0,062690333	0,122293696	0,338895921	-0,415478239	-0,353156503	-0,425394345	-0,383394345	**-1,643394345**
chrIV	870210	870347	137	0,136778908	0,140823044	0,492715945	-0,018259434	-0,015520519	-0,065218809	-0,016218809	**-1,276218809**
chrIV	872409	872511	102	0,094035499	0,129705435	0,420287416	-0,201158254	-0,170984516	0,377162022	0,671162022	**-0,588837978**
chrIV	873832	873980	148	0,082637257	0,085235	0,492262738	-0,019395657	-0,016486308	-0,228638041	-0,256638041	**-1,516638041**
chrIV	874621	874762	141	0,170973635	0,185293479	0,479902939	-0,050397187	-0,042837609	-0,200702297	-0,179702297	**-1,439702297**
chrIV	875359	875502	143	0,185221438	0,111176087	0,624908854	0,318399007	0,270639156	0,132418923	0,139418923	**-1,120581077**
chrIV	876645	876791	146	0,068389454	0,051882174	0,568624996	0,172874585	0,146943398	-0,033156374	-0,047156374	**-1,307156374**
chrIV	877438	877587	149	0,085486818	0,077823261	0,523463207	0,058847485	0,050020363	-0,102636453	-0,137636453	**-1,397636453**
chrIV	878298	878451	153	0,247911771	0,400233914	0,3824939	-0,298937414	-0,254096802	-0,328776053	-0,391776053	**-1,651776053**
chrIV	878496	878645	149	0,065539893	0,100058479	0,395776194	-0,264295292	-0,224650998	-0,380764605	-0,415764605	**-1,675764605**
chrIV	879292	879438	146	0,091185939	0,051882174	0,637360324	0,351411905	0,298700119	0,12084834	0,10684834	**-1,15315166**
chrIV	881327	881484	157	0,045592969	0,059293913	0,434687048	-0,164453569	-0,139785534	-0,124286737	-0,215286737	**-1,475286737**
chrIV	881795	881956	161	0,085486818	0,085235	0,500737507	0,001848658	0,001571359	0,013345779	-0,105654221	**-1,365654221**
chrIV	881975	882110	135	0,076938136	0,062999783	0,549801916	0,125160904	0,106386768	0,05340794	0,11640794	**-1,14359206**
chrIV	883013	883153	140	0,136778908	0,077823261	0,637360324	0,351411905	0,298700119	0,185498198	0,213498198	**-1,046501802**
chrIV	883019	883153	134	0,136778908	0,118587827	0,535617563	0,08939893	0,07598909	0,103760962	0,173760962	**-1,086239038**
chrIV	883431	883586	155	0,079787696	0,08894087	0,472876041	-0,06804215	-0,057835827	-0,07585025	-0,15285025	**-1,41285025**
chrIV	883562	883670	108	0,082637257	0,140823044	0,369807329	-0,332363684	-0,282509132	0,305213328	0,557213328	**-0,702786672**
chrIV	883696	883848	152	0,039893848	0,037058696	0,518421434	0,046192108	0,039263292	-0,058578115	-0,114578115	**-1,374578115**
chrIV	884819	884969	150	3,630340183	2,834990228	0,561508841	0,154795779	0,131576412	0,059139204	0,017139204	**-1,242860796**
chrIV	888390	888567	177	0,416035845	0,61146848	0,404899361	-0,2406857	-0,204582845	0,162128822	-0,068871178	**-1,328871178**
chrIV	889668	889827	159	0,649699813	1,159937178	0,359022177	-0,361073698	-0,306912643	-0,304720174	-0,409720174	**-1,669720174**
chrIV	890834	890988	154	0,14817715	0,040764565	0,784247935	0,786620371	0,668627316	0,538218417	0,468218417	**-0,791781583**
chrIV	891389	891533	144	0,572761677	0,192705218	0,748251402	0,668997305	0,568647709	0,424514894	0,424514894	**-0,835485106**
chrIV	891986	892153	167	0,125380666	0,237175653	0,345823971	-0,396619676	-0,337126724	-0,187359805	-0,348359805	**-1,608359805**
chrIV	896334	896482	148	0,227964847	0,070411522	0,764017766	0,71928641	0,611393448	0,40732014	0,37932014	**-0,88067986**
chrIV	897629	897776	147	0,113982423	0,085235	0,572150875	0,181852797	0,154574878	-0,005044597	-0,026044597	**-1,286044597**
chrIV	899028	899177	149	0,199469241	0,26311674	0,431204682	-0,173307964	-0,147311769	-0,297654491	-0,332654491	**-1,592654491**
chrIV	906466	906593	127	0,378991558	0,96352609	0,282299125	-0,576025049	-0,489621292	-0,213777757	-0,094777757	**-1,354777757**
chrIV	907937	908075	138	0,056991212	0,125999566	0,311443082	-0,491764035	-0,41799943	-0,49183835	-0,44983835	**-1,70983835**
chrIV	908538	908667	129	0,062690333	0,326116523	0,161237725	-0,989383693	-0,840976139	-0,620390989	-0,515390989	**-1,775390989**
chrIV	909620	909773	153	0,042743409	0,074117392	0,365763444	-0,34309514	-0,291630869	-0,363271496	-0,426271496	**-1,686271496**
chrIV	915796	915980	184	0,179522317	0,359469349	0,333070673	-0,431449801	-0,366732331	0,053933553	-0,226066447	**-1,486066447**
chrIV	917617	917765	148	0,082637257	0,048176305	0,631717813	0,336406466	0,285945496	0,07692569	0,04892569	**-1,21107431**
chrIV	924934	925100	166	0,233663968	0,114881957	0,670396499	0,441008282	0,37485704	0,496978806	0,342978806	**-0,917021194**
chrIV	926425	926570	145	0,393239361	0,240881523	0,620133118	0,305830423	0,259955859	0,08751488	0,08051488	**-1,17948512**
chrIV	927161	927289	128	0,162424953	0,074117392	0,686663326	0,486414449	0,413452282	0,675965023	0,787965023	**-0,472034977**
chrIV	928961	929115	154	0,054141651	0,048176305	0,529151025	0,07313593	0,062165541	-0,070149499	-0,140149499	**-1,400149499**
chrIV	930238	930383	145	0,760832676	0,585527393	0,565103417	0,163921215	0,139333033	-0,033991281	-0,040991281	**-1,300991281**
chrIV	931111	931260	149	0,923257629	0,478057176	0,658850978	0,409329261	0,347929872	0,213172003	0,178172003	**-1,081827997**
chrIV	933440	933600	160	0,037044288	0,074117392	0,333246923	-0,430964963	-0,366320218	-0,354468871	-0,466468871	**-1,726468871**
chrIV	944365	944524	159	0,142478029	0,188999349	0,429827309	-0,17681384	-0,150291764	-0,148022674	-0,253022674	**-1,513022674**
chrIV	944403	944557	154	0,364743755	0,314998914	0,536590936	0,091848853	0,078071525	-0,049890931	-0,119890931	**-1,379890931**
chrIV	948002	948171	169	0,034194727	0,100058479	0,254703244	-0,659762131	-0,560797812	-0,379066687	-0,554066687	**-1,814066687**
chrIV	949233	949387	154	0,188070999	0,055588044	0,771861355	0,744990787	0,633242169	0,510635993	0,440635993	**-0,819364007**
chrIV	955015	955163	148	1,188266763	0,685585872	0,634130316	0,342812703	0,291390798	0,088821498	0,060821498	**-1,199178502**
chrIV	957192	957317	125	0,045592969	0,055588044	0,450607955	-0,124125498	-0,105506674	0,272250071	0,405250071	**-0,854749929**
chrIV	957192	957321	129	9,614417409	9,609319817	0,500132586	0,000332343	0,000282492	0,212384766	0,317384766	**-0,942615234**
chrIV	957196	957298	102	0,062690333	0,059293913	0,51392155	0,034903236	0,029667751	0,570345204	0,864345204	**-0,395654796**
chrIV	962120	962274	154	0,347646391	0,381704567	0,476651724	-0,0585589	-0,049775065	-0,173690862	-0,243690862	**-1,503690862**
chrIV	962406	962555	149	0,302053422	0,203822827	0,59708955	0,245820872	0,208947741	0,078621791	0,043621791	**-1,216378209**
chrIV	965842	965991	149	0,065539893	0,051882174	0,558156527	0,146296985	0,124352437	-0,002042977	-0,037042977	**-1,297042977**
chrIV	968475	968604	129	4,339880769	7,20791633	0,375818932	-0,316480448	-0,269008381	-0,062294172	0,042705828	**-1,217294172**
chrIV	971192	971329	137	0,108283302	0,08894087	0,549036667	0,123227859	0,104743681	0,057527524	0,106527524	**-1,153472476**
chrIV	975387	975508	121	0,475876617	1,634288484	0,225516296	-0,753694656	-0,640640457	-0,112154132	0,048845868	**-1,211154132**
chrIV	979606	979759	153	0,284956058	0,26311674	0,519923739	0,049962185	0,042467857	-0,038501555	-0,101501555	**-1,361501555**
chrIV	980738	980889	151	2,14856868	1,819581963	0,541453406	0,10409597	0,088481575	-0,005028889	-0,054028889	**-1,314028889**
chrIV	981759	981861	102	0,131079787	0,170470001	0,434687048	-0,164453569	-0,139785534	0,409622463	0,703622463	**-0,556377537**
chrIV	983182	983330	148	0,074088575	0,074117392	0,499902783	-0,000243688	-0,000207135	-0,201235912	-0,229235912	**-1,489235912**
chrIV	984709	984852	143	0,176672756	0,066705652	0,725917953	0,600513459	0,51043644	0,370365209	0,377365209	**-0,882634791**
chrIV	985995	986141	146	0,108283302	0,044470435	0,708874979	0,550101085	0,467585923	0,292387819	0,278387819	**-0,981612181**
chrIV	986788	986937	149	0,085486818	0,044470435	0,657807209	0,406485945	0,345513053	0,223491274	0,188491274	**-1,071508726**
chrIV	987343	987492	149	0,094035499	0,085235	0,524545307	0,06156483	0,052330105	-0,067452466	-0,102452466	**-1,362452466**
chrIV	987657	987771	114	0,088336378	0,403939784	0,179444761	-0,917483145	-0,779860673	-0,235773884	-0,025773884	**-1,285773884**
chrIV	988353	988503	150	0,111132863	0,248293262	0,309195284	-0,498132622	-0,423412729	-0,491175299	-0,533175299	**-1,793175299**
chrIV	989662	989806	144	0,039893848	0,037058696	0,518421434	0,046192108	0,039263292	-0,107248348	-0,107248348	**-1,367248348**
chrIV	989749	989906	157	0,074088575	0,081529131	0,47609348	-0,059960669	-0,050966568	-0,035005362	-0,126005362	**-1,386005362**
chrIV	990217	990378	161	0,059840772	0,096352609	0,38311977	-0,297297305	-0,252702709	-0,244091229	-0,363091229	**-1,623091229**
chrIV	990397	990532	135	0,074088575	0,085235	0,465019536	-0,087795679	-0,074626327	-0,130630588	-0,067630588	**-1,327630588**
chrIV	991435	991575	140	0,170973635	0,114881957	0,598111913	0,248463027	0,211193573	0,086786656	0,114786656	**-1,145213344**
chrIV	991441	991575	134	0,119681545	0,081529131	0,594807131	0,239928434	0,203939169	0,227725641	0,297725641	**-0,962274359**
chrIV	991853	992008	155	0,094035499	0,085235	0,524545307	0,06156483	0,052330105	0,03936446	-0,03763554	**-1,29763554**
chrIV	991984	992092	108	0,079787696	0,151940653	0,344315647	-0,400713197	-0,340606218	0,241805891	0,493805891	**-0,766194109**
chrIV	992133	992282	149	0,062690333	0,070411522	0,470995186	-0,072768458	-0,061853189	-0,183913425	-0,218913425	**-1,478913425**
chrIV	994735	994883	148	0,376141997	0,248293262	0,60237149	0,259490228	0,220566694	0,013359261	-0,014640739	**-1,274640739**
chrIV	1001383	1001529	146	0,113982423	0,107470218	0,514703382	0,036864261	0,031334622	-0,136974783	-0,150974783	**-1,410974783**
chrIV	1001383	1001529	146	0,113982423	0,107470218	0,514703382	0,036864261	0,031334622	-0,136974783	-0,150974783	**-1,410974783**
chrIV	1005517	1005664	147	0,53571739	0,322410653	0,624286077	0,316757196	0,269243616	0,097961085	0,076961085	**-1,183038915**
chrIV	1005876	1006022	146	0,327699467	0,211234566	0,608051166	0,274243282	0,23310679	0,067203845	0,053203845	**-1,206796155**
chrIV	1012282	1012417	135	0,450230572	0,329822392	0,57717949	0,194683129	0,16548066	0,118104347	0,181104347	**-1,078895653**
chrIV	1016514	1016662	148	0,213717044	0,077823261	0,733061742	0,62209939	0,528784481	0,320611072	0,292611072	**-0,967388928**
chrIV	1019255	1019404	149	0,233663968	0,34835174	0,401473645	-0,249534587	-0,212104399	-0,330001612	-0,365001612	**-1,625001612**
chrIV	1021134	1021284	150	0,042743409	0,044470435	0,490098899	-0,024820929	-0,021097789	-0,096208887	-0,138208887	**-1,398208887**
chrIV	1021134	1021284	150	0,042743409	0,044470435	0,490098899	-0,024820929	-0,021097789	-0,096208887	-0,138208887	**-1,398208887**
chrIV	1025226	1025374	148	0,324849907	0,218646305	0,597704086	0,247408847	0,21029752	0,006268667	-0,021731333	**-1,281731333**
chrIV	1028806	1028950	144	0,453080133	0,303881305	0,598551141	0,249598683	0,21215888	0,052448063	0,052448063	**-1,207551937**
chrIV	1039896	1040063	167	0,136778908	0,285351958	0,324020154	-0,456486317	-0,38801337	-0,239125295	-0,400125295	**-1,660125295**
chrIV	1042109	1042252	143	1,017293128	0,785644351	0,564242044	0,16173326	0,137473271	0,003390984	0,010390984	**-1,249609016**

Chrom	Start	End	Length	Section A	Section B	A/A+B	Z-score	Z * 0.85	Phase correction	Length correction	ΔLKnuc
chrIV	1052004	1052154	150	0,111132863	0,055588044	0,666580246	0,43048963	0,365916185	0,291305814	0,249305814	**-1,010694186**
chrIV	1055603	1055756	153	2,402179572	1,93075805	0,554399759	0,136785326	0,116267527	0,043268067	-0,019731933	**-1,279731933**
chrIV	1058574	1058755	181	0,091185939	0,140823044	0,393027622	-0,271436616	-0,230721123	0,150923081	-0,108076919	**-1,368076919**
chrIV	1058574	1058755	181	0,091185939	0,140823044	0,393027622	-0,271436616	-0,230721123	0,150923081	-0,108076919	**-1,368076919**
chrIV	1063264	1063420	156	0,14817715	0,062999783	0,701672991	0,529218308	0,449835562	0,478301603	0,394301603	**-0,865698397**
chrIV	1066695	1066823	128	0,14532759	0,125999566	0,535617563	0,08939893	0,07598909	0,325899691	0,437899691	**-0,822100309**
chrIV	1075797	1075953	156	0,09973462	0,166764131	0,374240479	-0,320642986	-0,272546538	-0,24149808	-0,32549808	**-1,58549808**
chrIV	1079066	1079219	153	0,122531105	0,074117392	0,623097085	0,313625053	0,266581295	0,197740224	0,134740224	**-1,125259776**
chrIV	1079249	1079403	154	1,826568334	1,1451137	0,614658067	0,291480486	0,247758413	0,123957043	0,053957043	**-1,206042957**
chrIV	1080074	1080246	172	0,168124074	0,281646088	0,373799973	-0,321805635	-0,273534789	-0,043505391	-0,239505391	**-1,499505391**
chrIV	1090868	1091040	172	0,165274514	0,17417587	0,486888575	-0,032871387	-0,027940679	0,210997989	0,014997989	**-1,245002011**
chrIV	1091309	1091450	141	0,119681545	0,140823044	0,459422021	-0,101889932	-0,086606442	-0,24521628	-0,22421628	**-1,48421628**
chrIV	1096304	1096427	123	0,065539893	0,200116957	0,24670884	-0,684883118	-0,582150651	-0,129179707	0,017820293	**-1,242179707**
chrIV	1096404	1096559	155	0,09973462	0,103764348	0,490098899	-0,024820929	-0,021097789	-0,043357536	-0,120357536	**-1,380357536**
chrIV	1096837	1096971	134	0,108283302	0,125999566	0,462190442	-0,094916836	-0,080679311	-0,063225439	0,006774561	**-1,253225439**
chrIV	1096837	1096977	140	0,168124074	0,129705435	0,564497704	0,162382575	0,138025189	0,009297538	0,037297538	**-1,222702462**
chrIV	1097880	1098015	135	0,076938136	0,074117392	0,509336779	0,023405971	0,019895076	-0,024031054	0,038968946	**-1,221031054**
chrIV	1098034	1098195	161	0,062690333	0,096352609	0,394172367	-0,268460684	-0,228191582	-0,219648121	-0,338648121	**-1,598648121**
chrIV	1098506	1098663	157	0,056991212	0,037058696	0,605967759	0,268824834	0,228501109	0,245984574	0,154984574	**-1,105015426**
chrIV	1098997	1099126	129	0,068389454	0,125999566	0,351817475	-0,380418277	-0,323355536	-0,125463803	-0,020463803	**-1,280463803**
chrIV	1099909	1100059	150	0,464478375	0,35205761	0,568840056	0,173421802	0,147408532	0,064320209	0,022320209	**-1,237679791**
chrIV	1100669	1100827	158	0,039893848	0,037058696	0,518424134	0,046192108	0,039263292	0,05365371	-0,04434629	**-1,30434629**
chrIV	1102545	1102694	149	0,162424953	0,114881957	0,585722704	0,216555726	0,184072367	0,064262463	0,029262463	**-1,230737537**
chrIV	1102953	1103100	147	0,068389454	0,144528914	0,321200349	-0,464344846	-0,394693119	-0,560385305	-0,581385305	**-1,841385305**
chrIV	1107156	1107303	147	0,216566604	0,200116957	0,519738776	0,049497979	0,042073282	-0,123751627	-0,144751627	**-1,404751627**
chrIV	1109551	1109723	172	1,376337762	1,941875659	0,414782772	-0,215258806	-0,182969985	0,065552071	-0,130447929	**-1,390447929**
chrIV	1112644	1112796	152	0,333398588	0,326116523	0,505520772	0,013838965	0,011763121	-0,066598612	-0,122598612	**-1,382598612**
chrIV	1117237	1117387	150	0,307752543	0,151940653	0,669473784	0,43846059	0,372691501	0,288203525	0,246203525	**-1,013796475**
chrIV	1121058	1121205	147	0,139628469	0,118587827	0,540742282	0,102303929	0,08695834	-0,076747123	-0,097747123	**-1,357747123**
chrIV	1121801	1121948	147	0,467327936	0,366881088	0,560204843	0,151488587	0,128765299	-0,039931937	-0,060931937	**-1,320931937**
chrIV	1124473	1124641	168	0,336248149	0,696703481	0,325521679	-0,452313214	-0,384466232	-0,209973632	-0,377973632	**-1,637973632**
chrIV	1128248	1128395	147	0,398938482	0,296469566	0,573675388	0,185739279	0,157878387	-0,002399164	-0,023399164	**-1,283399164**
chrIV	1137826	1137974	148	0,578460798	0,433586741	0,57157473	0,180384731	0,153327021	-0,055574184	-0,083574184	**-1,343574184**
chrIV	1140412	1140528	116	2,459170784	16,59488397	0,129062859	-1,130832215	-0,961207383	-0,394879409	-0,198879409	**-1,458879409**
chrIV	1144636	1144783	147	0,763682236	0,607762611	0,556845022	0,142974962	0,121528717	-0,042722881	-0,063722881	**-1,323722881**
chrIV	1154940	1155065	125	1,347842156	2,645990879	0,337480847	-0,419348174	-0,356445948	-0,002208036	0,130791964	**-1,129208036**
chrIV	1155027	1155183	156	0,592708601	0,455821958	0,565275467	0,164358328	0,139704579	0,188745118	0,104745118	**-1,155254882**
chrIV	1155195	1155331	136	0,58700948	0,389116306	0,601366636	0,256886065	0,218353155	0,205030227	0,261030227	**-0,998969773**
chrIV	1158507	1158654	147	0,960301917	0,544762828	0,638046915	0,353243137	0,300256667	0,140265636	0,119265636	**-1,140734364**
chrIV	1160332	1160457	125	1,242408414	2,790519793	0,308066088	-0,50133955	-0,426138618	-0,087566306	0,045433694	**-1,214566306**
chrIV	1160419	1160575	156	0,661098055	0,444704349	0,597844653	0,247772164	0,210606339	0,250750814	0,166750814	**-1,093249186**
chrIV	1160587	1160723	136	0,615505086	0,366881088	0,626540868	0,322705529	0,274299699	0,257359042	0,313359042	**-0,946640958**
chrIV	1164215	1164366	151	0,498673102	0,363175219	0,578608892	0,198335964	0,168585569	0,074253583	0,025253583	**-1,234746417**
chrIV	1166734	1166868	134	0,549965193	0,592939133	0,481199677	-0,047142876	-0,040071445	-0,040065171	0,029934829	**-1,230065171**
chrIV	1169216	1169370	154	1,02584181	0,859761742	0,544038968	0,11061448	0,094022308	-0,029826604	-0,099826604	**-1,359826604**
chrIV	1170479	1170639	160	0,350495952	0,244587392	0,588986325	0,224938202	0,191197471	0,199334089	0,087334089	**-1,172665911**
chrIV	1170874	1171037	163	0,151026711	0,207528696	0,42120885	-0,198801911	-0,168981625	-0,137421618	-0,270421618	**-1,530421618**
chrIV	1170874	1171037	163	0,151026711	0,207528696	0,42120885	-0,198801911	-0,168981625	-0,137421618	-0,270421618	**-1,530421618**
chrIV	1171736	1171886	150	0,564212996	0,589233263	0,489154125	-0,027189926	-0,023111437	-0,107341001	-0,149341001	**-1,409341001**
chrIV	1175589	1175742	153	0,461628815	0,52993935	0,465554292	-0,086450148	-0,073482626	-0,143943524	-0,206943524	**-1,466943524**
chrIV	1176500	1176647	147	0,208017923	0,163058261	0,560580095	0,152440128	0,129574109	-0,032180512	-0,053180512	**-1,313180512**
chrIV	1179339	1179509	170	0,111132863	0,226058044	0,3295844	-0,441061051	-0,374901893	-0,156893454	-0,338893454	**-1,598893454**
chrIV	1185766	1185913	147	0,504372223	0,35205761	0,588924164	0,224778397	0,191061638	0,025305653	0,004305653	**-1,255694347**
chrIV	1186635	1186765	130	0,136778908	0,340940001	0,286316713	-0,564177371	-0,479550765	-0,354892523	-0,256892523	**-1,516892523**
chrIV	1187842	1187997	155	0,105433742	0,17417587	0,377074811	-0,313172484	-0,266196611	-0,26581164	-0,34281164	**-1,60281164**
chrIV	1199637	1199781	144	0,299203861	0,289057827	0,508623742	0,021618199	0,01837547	-0,147776687	-0,147776687	**-1,407776687**
chrIV	1199946	1200093	147	0,259310013	0,26311674	0,496356688	-0,009132555	-0,007762672	-0,173907531	-0,194907531	**-1,454907531**
chrIV	1202429	1202587	158	0,051292091	0,111176087	0,315705458	-0,47974192	-0,407780632	-0,39463873	-0,49263873	**-1,75263873**
chrIV	1205211	1205359	148	2,026037575	1,337818918	0,602296079	0,259294733	0,220400523	0,018353932	-0,009646068	**-1,269646068**
chrIV	1207063	1207212	149	0,082637257	0,044470435	0,650135768	0,385687039	0,327833983	0,201123255	0,166123255	**-1,093876745**
chrIV	1207237	1207360	123	0,059840772	0,233469783	0,204018475	-0,827353109	-0,703250143	-0,272145384	-0,125145384	**-1,385145384**
chrIV	1207337	1207492	155	0,091185939	0,111176087	0,450607955	-0,124125498	-0,105506674	-0,109858693	-0,186858693	**-1,446858693**
chrIV	1207770	1207904	134	0,122531105	0,118587827	0,508177041	0,020498237	0,017423501	0,010008307	0,080008307	**-1,179991693**
chrIV	1207770	1207910	140	0,159575393	0,096352609	0,623516738	0,314730183	0,267520656	0,147175634	0,175175634	**-1,084824366**
chrIV	1207966	1208117	151	0,085486818	0,100058479	0,460732874	-0,09858756	-0,083799426	-0,193669143	-0,242669143	**-1,502669143**
chrIV	1207976	1208121	145	0,09688506	0,092646739	0,511181027	0,028030349	0,023825797	-0,154192634	-0,161192634	**-1,421192634**
chrIV	1207994	1208145	151	0,042743409	0,048176305	0,470122564	-0,074961771	-0,063717505	-0,175614536	-0,224614536	**-1,484614536**
chrIV	1207996	1208145	149	0,051292091	0,048176305	0,515662191	0,039269381	0,033378974	-0,093787762	-0,128787762	**-1,388787762**
chrIV	1208248	1208402	154	0,222265726	0,125999566	0,638208088	0,35367318	0,300622203	0,183411283	0,113411283	**-1,146588717**
chrIV	1208282	1208426	144	0,071239015	0,037058696	0,657807209	0,406485945	0,345513053	0,193157046	0,193157046	**-1,066842954**
chrIV	1208813	1208948	135	0,076938136	0,074117392	0,509336779	0,023405971	0,019895076	-0,014399633	0,048600367	**-1,211399633**
chrIV	1208967	1209128	161	0,071239015	0,070411522	0,502920895	0,007321663	0,006223413	0,016480924	-0,102519076	**-1,362519076**
chrIV	1209439	1209596	157	0,065539893	0,048176305	0,576346155	0,192554744	0,163671533	0,173780624	0,082780624	**-1,177219376**
chrIV	1209930	1210059	129	0,045592969	0,118587827	0,277699771	-0,589688448	-0,501235181	-0,323192145	-0,218192145	**-1,478192145**
chrIV	1210842	1210992	150	0,131079787	0,218646305	0,374806999	-0,319148382	-0,271276125	-0,347101236	-0,389101236	**-1,649101236**
chrIV	1211574	1211688	114	0,031345166	0,17788174	0,149814223	-1,037230501	-0,881645926	-0,335958414	-0,125958414	**-1,385958414**
chrIV	1215599	1215745	146	0,076938136	0,055588044	0,58055047	0,203301913	0,172806626	0,005004586	-0,008995414	**-1,268995414**
chrIV	1216176	1216327	151	0,054141651	0,085235	0,388455674	-0,283346352	-0,2408444	-0,343756105	-0,392756105	**-1,652756105**
chrIV	1217346	1217492	146	0,302053422	0,285351958	0,514216302	0,035642529	0,03029615	-0,138513411	-0,152513411	**-1,412513411**
chrIV	1218508	1218653	145	0,735186631	0,552174567	0,571080309	0,179125217	0,152256434	-0,021764769	-0,028764769	**-1,288764769**
chrIV	1221088	1221239	151	0,091185939	0,122293696	0,427141159	-0,183657287	-0,156108694	-0,261973339	-0,310973339	**-1,570973339**
chrIV	1225710	1225871	161	0,253610892	0,311293045	0,448945167	-0,12832683	-0,109077806	-0,102847672	-0,221847672	**-1,481847672**
chrIV	1225924	1226071	147	0,153876272	0,055588044	0,734618073	0,626840405	0,532814345	0,375707512	0,354707512	**-0,905292488**
chrIV	1227651	1227806	155	0,034194727	0,062999783	0,351817475	-0,380418277	-0,323355536	-0,326778754	-0,403778754	**-1,663778754**
chrIV	1238397	1238557	160	0,313451664	0,322410653	0,492955244	-0,017659501	-0,015010576	-0,00584321	-0,11784321	**-1,37784321**
chrIV	1240257	1240406	149	0,250761331	0,203822827	0,551627959	0,129775456	0,110309137	-0,019931113	-0,054931113	**-1,314931113**
chrIV	1240385	1240526	141	0,430283648	0,366881088	0,539767539	0,099848099	0,084870884	-0,069831282	-0,048831282	**-1,308831282**
chrIV	1244524	1244687	163	0,173823196	0,155646522	0,52758474	0,069199879	0,058819897	0,09286825	-0,04013175	**-1,30013175**
chrIV	1245862	1245987	125	1,384886444	2,482932618	0,358053575	-0,363666381	-0,309116424	0,029948512	0,162948512	**-1,097051488**
chrIV	1251539	1251695	156	0,940354993	0,685585872	0,578345137	0,197661741	0,16801248	0,206817998	0,122817998	**-1,137182002**
chrIV	1259546	1259668	122	0,561363435	2,015993051	0,217805895	-0,77962474	-0,662681029	-0,173201745	-0,019201745	**-1,279201745**
chrIV	1265859	1266020	161	0,09973462	0,100058479	0,499189516	-0,002031583	-0,001726846	0,001910915	-0,117089085	**-1,377089085**
chrIV	1266398	1266553	155	0,236513528	0,274234349	0,463072954	-0,092694951	-0,078790708	-0,08406368	-0,16106368	**-1,42106368**
chrIV	1267550	1267699	149	0,302053422	0,248293262	0,548842086	0,122736414	0,104325951	-0,019929408	-0,054929408	**-1,314929408**
chrIV	1269091	1269198	107	0,082637257	0,292763697	0,220130653	-0,771752035	-0,65598923	-0,085760565	0,173239435	**-1,086760565**
chrIV	1269091	1269209	118	0,139628469	0,52623348	0,209695822	-0,807477133	-0,686355563	-0,139623014	0,042376986	**-1,217623014**

Chrom	Start	End	Length	Section A	Section B	A/A+B	Z-score	Z * 0.85	Phase correction	Length correction	ΔLKnuc
chrIV	1272800	1272981	181	0,068389454	0,218646305	0,238261094	-0,711907289	-0,605121196	-0,207560067	-0,466560067	**-1,726560067**
chrIV	1277856	1277982	126	0,273557816	0,915349786	0,230091738	-0,738544765	-0,62776305	-0,331556501	-0,205556501	**-1,465556501**
chrIV	1278160	1278320	160	2,137170438	1,945581529	0,523463207	0,058847485	0,050020363	0,068449084	-0,043550916	**-1,303550916**
chrIV	1287754	1287911	157	0,826372569	0,433586741	0,655872426	0,401224087	0,341040474	0,349838993	0,258838993	**-1,001161007**
chrIV	1288372	1288504	132	0,302053422	0,192705218	0,610506614	0,280639687	0,238543734	0,33557521	0,41957521	**-0,84042479**
chrIV	1291651	1291799	148	1,108479067	0,744879785	0,598091981	0,248411498	0,211149773	0,009670395	-0,018329605	**-1,278329605**
chrIV	1293635	1293774	139	2,105825271	1,878875876	0,528477593	0,07144347	0,060726949	-0,054514777	-0,019514777	**-1,279514777**
chrIV	1298059	1298223	164	0,065539893	0,048176305	0,576346155	0,192554744	0,163671533	0,234858175	0,094858175	**-1,165141825**
chrIV	1300809	1300966	157	0,037044288	0,040764565	0,47609348	-0,059960669	-0,050966568	-0,043797029	-0,134797029	**-1,394797029**
chrIV	1300809	1300970	161	2,818215417	2,99063675	0,485158743	-0,037210099	-0,031628584	-0,02282097	-0,14182097	**-1,40182097**
chrIV	1300843	1301002	159	0,467327936	0,296469566	0,61184795	0,284138687	0,241517884	0,236117138	0,131117138	**-1,128882862**
chrIV	1300844	1300970	126	0,059840772	0,114881957	0,342489913	-0,405677162	-0,344825587	-0,052655185	0,073344815	**-1,186655185**
chrIV	1300844	1300987	143	0,168124074	0,226058044	0,426513701	-0,185257077	-0,157468516	-0,293729534	-0,286729534	**-1,546729534**
chrIV	1302267	1302373	106	0,182371877	1,030231743	0,150396943	-1,034732432	-0,879522567	-0,309502499	-0,043502499	**-1,303502499**
chrIV	1302267	1302383	116	0,210867483	1,267407396	0,142644299	-1,06851427	-0,908237129	-0,348978415	-0,152978415	**-1,412978415**
chrIV	1308150	1308260	110	0,071239015	0,255705001	0,217893619	-0,779326791	-0,662427772	-0,099372724	0,138627276	**-1,121372724**
chrIV	1308150	1308309	159	20,13784464	16,52447245	0,549279103	0,12384021	0,105264178	0,101372459	-0,003627541	**-1,263627541**
chrIV	1308153	1308309	156	0,031345166	0,285351958	0,098975217	-1,287412835	-1,09430091	-1,063150106	-1,147150106	**-2,407150106**
chrIV	1308185	1308314	129	0,116831984	0,222352175	0,344450001	-0,400348293	-0,340296049	-0,18875978	-0,08375978	**-1,34375978**
chrIV	1308191	1308309	118	0,082637257	0,08894087	0,481630488	-0,046061821	-0,039152548	0,504883598	0,686883598	**-0,573116402**
chrIV	1308196	1308309	113	0,176672756	0,444704349	0,284324534	-0,570042211	-0,48453588	0,052176889	0,269176889	**-0,990823111**
chrIV	1308234	1308344	110	0,091185939	0,226058044	0,287431578	-0,560903746	-0,476768184	0,081482944	0,319482944	**-0,940517056**
chrIV	1308280	1308442	162	1,242408414	0,670762394	0,649397539	0,383694456	0,326140288	0,351410947	0,225410947	**-1,034589053**
chrIV	1308280	1308477	197	0,042743409	0,066705652	0,390532439	-0,277931585	-0,236241848	0,197801403	-0,173198597	**-1,433198597**
chrIV	1308478	1308605	127	0,136778908	0,048176305	0,739524483	0,641880105	0,545598089	0,806970267	0,925970267	**-0,334029733**
chrIV	1308858	1308982	124	4,741668811	15,75365158	0,23135373	-0,734395942	-0,624236551	-0,221140054	-0,081140054	**-1,341140054**
chrIV	1312653	1312780	127	0,14532759	0,333528262	0,303489222	-0,514391296	-0,437232602	-0,180916089	-0,061916089	**-1,321916089**
chrIV	1312778	1312924	146	0,287805619	0,237175653	0,548220735	0,121167293	0,102992199	-0,065573388	-0,079573388	**-1,339573388**
chrIV	1324502	1324680	178	0,04844253	0,062999783	0,434687048	-0,164453569	-0,139785534	0,240834911	0,002834911	**-1,257165089**
chrIV	1324730	1324882	152	0,265009134	0,037058696	0,877316642	1,161676939	0,987425398	0,918566477	0,862566477	**-0,397433523**
chrIV	1329033	1329189	156	0,552814753	0,511410002	0,519453011	0,048780806	0,041463685	0,076079714	-0,007920286	**-1,267920286**
chrIV	1331269	1331431	162	0,076938136	0,040764565	0,653664997	0,395234266	0,335949126	0,359127163	0,233127163	**-1,026872837**
chrIV	1332261	1332409	148	0,136778908	0,114881957	0,543504879	0,109267603	0,092877463	-0,108982393	-0,136982393	**-1,396982393**
chrIV	1334150	1334293	143	0,766531797	0,466939567	0,621442718	0,309272085	0,262881272	0,123818166	0,130818166	**-1,129181834**
chrIV	1334858	1335004	146	0,176672756	0,066705652	0,725917953	0,600513459	0,51043644	0,337689956	0,323689956	**-0,936310044**
chrIV	1335220	1335376	156	0,159575393	0,200116957	0,443644111	-0,141736401	-0,120475941	-0,082561302	-0,166561302	**-1,426561302**
chrIV	1335220	1335376	156	0,159575393	0,200116957	0,443644111	-0,141736401	-0,120475941	-0,082561302	-0,166561302	**-1,426561302**
chrIV	1337771	1337882	111	0,299203861	0,096352609	0,756412507	0,694809033	0,590587678	1,136701928	1,367701928	**0,107701928**
chrIV	1341919	1342069	150	1,117027749	0,711526959	0,610880136	0,281613708	0,239371652	0,173372693	0,131372693	**-1,128627307**
chrIV	1354730	1354858	128	0,481575739	0,667056524	0,419260153	-0,203786546	-0,173218564	0,072986624	0,184986624	**-1,075013376**
chrIV	1356736	1356889	153	0,153876272	0,251999131	0,379121943	-0,307787693	-0,261619539	-0,345536338	-0,408536338	**-1,668536338**
chrIV	1359819	1359977	158	0,239363089	0,222352175	0,518421434	0,046192108	0,039263292	0,051421046	-0,046578954	**-1,306578954**
chrIV	1360674	1360822	148	0,359044634	0,188999349	0,655138356	0,399230614	0,339346022	0,137789796	0,109789796	**-1,150210204**
chrIV	1361305	1361412	107	0,29065518	0,867173481	0,251034708	-0,671237229	-0,570551645	0,022109575	0,281109575	**-0,978890425**
chrIV	1361305	1361420	115	0,564212996	1,445289136	0,280772529	-0,580548103	-0,493465887	0,055209345	0,258209345	**-1,001790655**
chrIV	1361305	1361420	115	0,564212996	1,445289136	0,280772529	-0,580548103	-0,493465887	0,055209345	0,258209345	**-1,001790655**
chrIV	1364336	1364488	152	0,056991212	0,037058696	0,605967759	0,268824834	0,228501109	0,162795131	0,106795131	**-1,153204869**
chrIV	1364512	1364671	159	0,612655525	0,637409568	0,490098899	-0,024820929	-0,021097789	-0,009921696	-0,114921696	**-1,374921696**
chrIV	1365728	1365875	147	0,045592969	0,062999783	0,419852784	-0,202270107	-0,171929591	-0,324996813	-0,345996813	**-1,605996813**
chrIV	1365746	1365909	163	0,176672756	0,125999566	0,583709654	0,211392905	0,179683969	0,223499563	0,090499563	**-1,169500437**
chrIV	1367419	1367567	148	0,113982423	0,048176305	0,702906497	0,532778373	0,452861617	0,269380942	0,241380942	**-1,018619058**
chrIV	1367927	1368075	148	0,170973635	0,159352392	0,517590565	0,044107306	0,03749121	-0,139036708	-0,167036708	**-1,427036708**
chrIV	1372245	1372378	133	0,185221438	0,163058261	0,531818071	0,079840821	0,067864697	0,13917141	0,21617141	**-1,04382859**
chrIV	1376748	1376898	150	0,042743409	0,077823261	0,354520937	-0,373143192	-0,317171714	-0,374420309	-0,416420309	**-1,676420309**
chrIV	1382369	1382521	152	0,604106844	0,589233263	0,506231912	0,015621722	0,013278464	-0,057429188	-0,113429188	**-1,373429188**
chrIV	1387683	1387833	150	0,530018269	0,377998697	0,583709654	0,211392905	0,179683969	0,120248556	0,078248556	**-1,181751444**
chrIV	1390839	1391009	170	0,045592969	0,092646739	0,329810947	-0,440435256	-0,374369967	-0,171104723	-0,353104723	**-1,613104723**
chrIV	1391435	1391580	145	0,074088575	0,051882174	0,588141101	0,222765786	0,189350918	0,0014058	-0,0055942	**-1,2655942**
chrIV	1391940	1392060	120	1,843665698	8,727322858	0,174408069	-0,936888068	-0,796354858	-0,266456605	-0,098456605	**-1,358456605**
chrIV	1397500	1397655	155	0,282106498	0,251999131	0,528184843	0,070707798	0,060101628	0,089701734	0,012701734	**-1,247298266**
chrIV	1398087	1398256	169	0,04844253	0,055588044	0,465656665	-0,08619258	-0,073263693	0,128087561	-0,046912439	**-1,306912439**
chrIV	1406033	1406179	146	0,58415992	0,444704349	0,567771607	0,170703659	0,14509811	-0,023647896	-0,037647896	**-1,297647896**
chrIV	1407803	1407943	140	0,672496298	0,574409785	0,539331957	0,09875086	0,083938231	-0,041460181	-0,013460181	**-1,273460181**
chrIV	1407803	1407943	140	0,672496298	0,574409785	0,539331957	0,09875086	0,083938231	-0,041460181	-0,013460181	**-1,273460181**
chrIV	1408060	1408202	142	0,071239015	0,055588044	0,561702018	0,155285859	0,13199298	-0,030788755	-0,016788755	**-1,276788755**
chrIV	1408065	1408226	161	0,170973635	0,170470001	0,500737507	0,001848658	0,001571359	0,014560023	-0,104439977	**-1,364439977**
chrIV	1408340	1408489	149	0,470177496	0,333528262	0,585011979	0,214732295	0,182522451	0,059047639	0,024047639	**-1,235952361**
chrIV	1409678	1409825	147	0,467327936	0,35576348	0,567771607	0,170703659	0,14509811	-0,02047182	-0,04147182	**-1,30147182**
chrIV	1413171	1413310	139	0,125380666	0,111176087	0,530023616	0,075329227	0,064029843	-0,052423238	-0,017423238	**-1,277423238**
chrIV	1420451	1420608	157	0,082637257	0,34835174	0,19173867	-0,871507085	-0,740781022	-0,72111598	-0,81211598	**-2,07211598**
chrIV	1420630	1420804	174	13,03958923	16,98029441	0,434365082	-0,165271659	-0,14048091	0,159807867	-0,050192133	**-1,310192133**
chrIV	1420630	1420804	174	13,03958923	16,98029441	0,434365082	-0,165271659	-0,14048091	0,159807867	-0,050192133	**-1,310192133**
chrIV	1420634	1420804	170	0,028495606	0,059293913	0,324590066	-0,454901459	-0,386666241	-0,173403162	-0,355403162	**-1,615403162**
chrIV	1420634	1420804	170	0,028495606	0,059293913	0,324590066	-0,454901459	-0,386666241	-0,173403162	-0,355403162	**-1,615403162**
chrIV	1424831	1424973	142	0,777930039	0,374292827	0,675155876	0,454195325	0,386066026	0,218718947	0,232718947	**-1,027281053**
chrIV	1428254	1428414	160	0,165274514	0,240881523	0,406923692	-0,235465589	-0,200145751	-0,171886236	-0,283886236	**-1,543886236**
chrIV	1430994	1431130	136	0,102584181	0,203822827	0,334797111	-0,426704981	-0,362699234	-0,386789679	-0,330789679	**-1,590789679**
chrIV	1433662	1433813	151	0,062690333	0,081529131	0,434687048	-0,164453569	-0,139785534	-0,253571203	-0,302571203	**-1,562571203**
chrIV	1447757	1447907	150	0,359044634	0,255705001	0,584050178	0,21226584	0,180425964	0,128892706	0,086892706	**-1,173107294**
chrIV	1454001	1454153	152	1,541612276	1,289642613	0,544497877	0,111771928	0,095006139	0,016041017	-0,039958983	**-1,299958983**
chrIV	1460987	1461146	159	2,060232302	1,652817832	0,554862506	0,137956258	0,117262819	0,123925227	0,018925227	**-1,241074773**
chrIV	1461681	1461828	147	0,09973462	0,070411522	0,58617033	0,217704522	0,185048844	0,016544915	-0,004455085	**-1,264455085**
chrIV	1462856	1462994	138	1,108479067	0,96723196	0,534023789	0,085388642	0,072580346	-0,001373496	0,040626504	**-1,219373496**
chrIV	1462967	1463131	164	1,105629506	0,844939264	0,566824451	0,16829514	0,143050869	0,221876497	0,081876497	**-1,178123503**
chrIV	1464532	1464669	137	0,273557816	0,292763697	0,483043306	-0,042516934	-0,036139394	-0,087784607	-0,038784607	**-1,298784607**
chrIV	1465805	1465959	154	0,113982423	0,085235	0,572150875	0,181852797	0,154574878	0,04130594	-0,02869406	**-1,28869406**
chrIV	1466131	1466277	146	0,310602104	0,251999131	0,552082158	0,130923676	0,111285125	-0,066047467	-0,080047467	**-1,340047467**
chrIV	1473970	1474117	147	0,424584527	0,385410436	0,524181688	0,060651668	0,051553918	-0,113674278	-0,134674278	**-1,394674278**
chrIV	1477488	1477647	159	0,852018614	0,726350437	0,539809504	0,099953814	0,084960742	0,083842688	-0,021157312	**-1,281157312**
chrIV	1478568	1478697	129	0,709540585	1,378583483	0,339798097	-0,413014221	-0,351062088	-0,209258552	-0,104258552	**-1,364258552**
chrIV	1479228	1479388	160	0,088336378	0,070411522	0,556456986	0,141992377	0,12069352	0,145321039	0,033321039	**-1,226678961**
chrIV	1479727	1479884	157	0,618354647	0,522527611	0,5419969	0,105465808	0,089645937	0,107932308	0,016932308	**-1,243067692**
chrIV	1481305	1481467	162	0,179522317	0,229763914	0,438622908	-0,154461562	-0,131292328	-0,105295083	-0,231295083	**-1,491295083**
chrIV	1489675	1489832	157	0,561363435	0,61888022	0,475633512	-0,061115751	-0,051948389	-0,02833781	-0,11933781	**-1,37933781**
chrIV	1495628	1495753	125	0,082637257	0,148234783	0,357935317	-0,363983093	-0,309385629	0,018812758	0,151812758	**-1,108187242**
chrIV	1496435	1496575	140	0,182371877	0,17417587	0,511493562	0,028814074	0,024491963	-0,119387013	-0,091387013	**-1,351387013**

Chrom	Start	End	Length	Section A	Section B	A/A+B	Z-score	Z * 0.85	Phase correction	Length correction	ΔLKnuc
chrIV	1497627	1497776	149	0,190920559	0,159352392	0,54506224	0,113195553	0,09621622	-0,018031642	-0,053031642	-1,313031642
chrIV	1498743	1498886	143	0,903310705	0,985761308	0,478176956	-0,054729569	-0,046520133	-0,175867749	-0,168867749	-1,428867749
chrIV	1499148	1499307	159	0,079787696	0,096352609	0,452978074	-0,118140734	-0,100419624	-0,094238528	-0,199238528	-1,459238528
chrIV	1503439	1503593	154	0,34194727	0,240881523	0,586702775	0,219071373	0,186210667	0,073822531	0,003822531	-1,256177469
chrIV	1505128	1505252	124	0,059840772	0,055588044	0,518421434	0,046192108	0,039263292	0,456549629	0,596549629	-0,663450371
chrIV	1507157	1507304	147	0,273557816	0,218646305	0,555781238	0,140281573	0,119239337	-0,037227142	-0,058227142	-1,318227142
chrIV	1512418	1512571	153	0,530018269	0,648527176	0,449722385	-0,126362771	-0,107408355	-0,188941655	-0,251941655	-1,511941655
chrIV	1512829	1512960	131	1,376337762	1,374877614	0,500265364	0,000665169	0,000565394	0,092336758	0,183336758	-1,076663242
chrIV	1526039	1526194	155	0,3903898	0,433586741	0,473787518	-0,065752297	-0,055889452	-0,023461567	-0,100461567	-1,360461567
chrIV	1527054	1527225	171	0,031345166	0,08894087	0,260588572	-0,641531928	-0,545302139	-0,320058663	-0,509058663	-1,769058663
chrIV	1527204	1527351	147	1,649895578	1,089525656	0,602278889	0,259250173	0,220362647	0,061208033	0,040208033	-1,219791967
chrIV	1527360	1527490	130	0,259310013	0,270528479	0,489413315	-0,026539999	-0,022558999	0,093185411	0,191185411	-1,068814589
chrIV	1527741	1527877	136	0,495823542	0,566998046	0,46651625	-0,084030099	-0,071425584	-0,103047804	-0,047047804	-1,307047804
chrIV	1528122	1528284	162	0,062690333	0,066705652	0,484484373	-0,038901718	-0,03306646	-0,007786404	-0,133786404	-1,393786404
chrIV	1528223	1528378	155	0,062690333	0,188999349	0,249077882	-0,677394378	-0,575785221	-0,53569017	-0,61269017	-1,87269017
chrIV	1528357	1528494	137	0,162424953	0,122293696	0,570475287	0,177584338	0,150946687	0,089196304	0,138196304	-1,121803696
chrIV	1528652	1528795	143	0,313451664	0,433586741	0,419592436	-0,202936235	-0,1724958	-0,298961505	-0,291961505	-1,551961505
chrIV	1528700	1528825	125	0,210867483	0,214940436	0,495217383	-0,01198853	-0,010190251	0,323016528	0,456016528	-0,803983472
chrIV	1528925	1529077	152	0,09688506	0,08894087	0,521375354	0,053605728	0,045564869	-0,037584297	-0,093584297	-1,353584297
chrIV	1528998	1529142	144	0,09688506	0,059293913	0,620346375	0,30639062	0,260432027	0,107179247	0,107179247	-1,152820753
chrIV	1529095	1529230	135	1,279452702	0,87829109	0,592958583	0,235162213	0,199887881	0,186855848	0,249855848	-1,010144152
chrIV	1529095	1529282	187	0,128230226	0,074117392	0,633712557	0,341702373	0,290447017	0,705715081	0,404715081	-0,855284919
chrIV	1529138	1529230	92	0,170973635	0,137117174	0,554945587	0,138166505	0,117441529	0,456737995	0,820737995	-0,439262005
chrIV	1529138	1529282	144	1,439028095	0,61146848	0,701794927	0,529569933	0,450134443	0,282846411	0,282846411	-0,977153589
chrIV	1529477	1529629	152	0,165274514	0,140823044	0,539940648	0,100284197	0,085241567	0,004730172	-0,051269828	-1,311269828
chrIV	1529778	1529908	130	0,327699467	0,485468915	0,402990911	-0,245613003	-0,208771053	-0,098262391	-0,000262391	-1,260262391
chrIV	1529916	1530077	161	0,182371877	0,207528696	0,467739443	-0,080953558	-0,068810524	-0,047708642	-0,166708642	-1,426708642
chrIV	1530973	1531125	152	0,037044288	0,048176305	0,434687048	-0,164453569	-0,139785534	-0,221106359	-0,277106359	-1,537106359
chrIV	1531313	1531464	151	0,515770466	0,359469349	0,589290452	0,225720135	0,191862115	0,081929697	0,032929697	-1,227070303
chrIV	1531313	1531484	171	0,028495606	0,051882174	0,354520937	-0,373143192	-0,317171714	-0,098343973	-0,287343973	-1,547343973
chrIX	1597	1749	152	0,062690333	0,040764565	0,605967759	0,268824834	0,228501109	0,12314995	0,06714995	-1,19285005
chrIX	1686	1802	116	0,119681545	0,114881957	0,510230892	0,025647854	0,021800676	0,549404827	0,745404827	-0,514595173
chrIX	1686	1821	135	2,322391876	1,222936961	0,655056832	0,399009321	0,339157923	0,416188243	0,479188243	-0,780811757
chrIX	1686	1838	152	0,333398588	0,259410871	0,562404299	0,157067817	0,133507644	0,045231069	-0,010768931	-1,270768931
chrIX	1710	1802	92	0,116831984	0,137117174	0,460060528	-0,100281233	-0,085239048	0,384362519	0,748362519	-0,511637481
chrIX	1710	1811	101	0,09973462	0,196411088	0,336775505	-0,421279483	-0,358087561	0,129205877	0,430205877	-0,829794123
chrIX	1710	1821	111	0,410336724	0,418763262	0,494918262	-0,012738373	-0,010827617	0,556370722	0,787370722	-0,472629278
chrIX	1710	1838	128	7,195140473	7,82309068	0,479093736	-0,052428241	-0,044564005	0,314144122	0,426144122	-0,833855878
chrIX	1710	1857	147	0,071239015	0,044470435	0,615671537	0,294132147	0,250012325	0,077120251	0,056120251	-1,203879749
chrIX	1746	1838	92	0,042743409	0,125999566	0,25330482	-0,664126048	-0,564507141	-0,086758461	0,277241539	-0,982758461
chrIX	2215	2352	137	0,128230226	0,100058479	0,561702018	0,155285859	0,13199298	0,11547547	0,16447547	-1,09552453
chrIX	2382	2529	147	0,062690333	0,040764565	0,605967759	0,268824834	0,228501109	0,061943972	0,040943972	-1,219056028
chrIX	2382	2530	148	17,53334627	11,31772569	0,607719058	0,273379012	0,23237216	0,053883019	0,025883019	-1,234116981
chrIX	2382	2592	210	0,091185939	0,122293696	0,427141159	-0,183657287	-0,156108694	0,217349873	-0,244650127	-1,504650127
chrIX	2383	2530	147	0,051292091	0,08894087	0,365763444	-0,34309514	-0,291630869	-0,463892777	-0,484892777	-1,744892777
chrIX	2385	2530	145	0,028495606	0,037058696	0,434687048	-0,164453569	-0,139785534	-0,285370664	-0,292370664	-1,552370664
chrIX	2397	2530	133	1,162620718	1,078408047	0,518788842	0,047114067	0,040046957	0,208947731	0,285947731	-0,974052269
chrIX	2437	2530	93	0,210867483	0,407645654	0,340926442	-0,409936016	-0,348445614	0,146231211	0,503231211	-0,756768789
chrIX	2437	2592	155	0,900461144	0,718938698	0,55604621	0,140952356	0,119809503	0,082881379	0,005881379	-1,254118621
chrIX	2558	2688	130	0,3903898	0,444704349	0,467479984	-0,081606077	-0,069365165	0,209137097	0,307137097	-0,952862903
chrIX	2879	3032	153	0,823523009	0,685585872	0,545701519	0,114808437	0,097587171	0,029805292	-0,033194708	-1,293194708
chrIX	3324	3468	144	0,09973462	0,066705652	0,599221683	0,251333038	0,213633083	0,093621403	0,093621403	-1,166378597
chrIX	3531	3658	127	0,567062556	1,137701961	0,332633951	-0,432651594	-0,367753855	0,03588107	0,15488107	-1,10511893
chrIX	4088	4243	155	0,088336378	0,196411088	0,310227091	-0,495206755	-0,420925742	-0,451390823	-0,528390823	-1,788390823
chrIX	4805	4968	163	0,213717044	0,329822392	0,393195101	-0,271001078	-0,230350917	-0,158739927	-0,291739927	-1,551739927
chrIX	5216	5367	151	0,205168362	0,118587827	0,633712557	0,341702373	0,290447017	0,138095623	0,089095623	-1,170904377
chrIX	5429	5588	159	0,530018269	0,470645436	0,529666726	0,074432125	0,063267306	0,088734685	-0,016265315	-1,276265315
chrIX	9290	9441	151	0,589859041	0,470645436	0,556206083	0,14135711	0,120153544	-0,031792373	-0,080792373	-1,340792373
chrIX	10228	10371	143	0,102584181	0,059293913	0,633712557	0,341702373	0,290447017	0,1800709	0,1870709	-1,0729291
chrIX	11460	11611	151	0,319150785	0,289057827	0,52473901	0,0620513	0,052743605	-0,09506829	-0,14406829	-1,40406829
chrIX	12345	12490	145	0,236513528	0,137117174	0,633014168	0,339847115	0,288870048	0,140066543	0,133066543	-1,126933457
chrIX	12910	13059	149	0,068389454	0,055588044	0,551627959	0,129775456	0,110309137	-0,065544233	-0,100544233	-1,360544233
chrIX	14208	14357	149	0,122531105	0,055588044	0,687916521	0,489953281	0,416460289	0,249257829	0,214257829	-1,045742171
chrIX	16078	16185	107	0,168124074	1,615759136	0,094246122	-1,315052545	-1,117794663	-0,554620992	-0,295620992	-1,555620992
chrIX	25656	25806	150	1,467523701	1,363760005	0,518324496	0,045948864	0,039056534	-0,132893897	-0,174893897	-1,434893897
chrIX	32330	32492	162	0,076938136	0,055588044	0,58055047	0,203301913	0,172806626	0,235027004	0,109027004	-1,150972996
chrIX	33763	33917	154	0,188070999	0,211234566	0,470995186	-0,072768458	-0,061853189	-0,117263707	-0,187263707	-1,447263707
chrIX	35803	35935	132	0,373292436	0,300175436	0,554283956	0,136492329	0,11601848	0,321260624	0,405260624	-0,854739376
chrIX	41284	41431	147	0,723788388	0,485468915	0,598539605	0,24956885	0,212133523	0,037046296	0,016046296	-1,243953704
chrIX	48410	48570	160	0,706691025	0,407645654	0,634180888	0,342947146	0,291505074	0,356834562	0,244834562	-1,015165438
chrIX	55336	55486	150	0,199469241	0,074117392	0,729089865	0,610062706	0,5185533	0,35763216	0,31563216	-0,94436784
chrIX	55497	55655	158	10,26981634	9,631555035	0,516035611	0,040206146	0,034175224	0,051419363	-0,046580637	-1,306580637
chrIX	55498	55655	157	0,031345166	0,037058696	0,458236793	-0,104876779	-0,089145262	-0,07861383	-0,16961383	-1,42961383
chrIX	55500	55655	155	0,034194727	0,062999783	0,351817475	-0,380418277	-0,323355536	-0,352362206	-0,429362206	-1,689362206
chrIX	56537	56665	128	0,173823196	0,622586089	0,218258625	-0,778087807	-0,661374636	-0,310850015	-0,198850015	-1,458850015
chrIX	56786	56893	107	0,985947962	3,450164578	0,222254948	-0,764599787	-0,649909819	-0,080763297	0,178236703	-1,081763297
chrIX	56786	56905	119	1,191116324	4,754630669	0,200330812	-0,840440188	-0,71437416	-0,198552102	-0,023552102	-1,283552102
chrIX	56791	56905	114	0,054141651	0,148234783	0,267529425	-0,620302195	-0,527256866	0,042055219	0,252055219	-1,007944781
chrIX	59003	59166	163	0,059840772	0,085235	0,412479432	-0,221171546	-0,187995814	-0,097248005	-0,230248005	-1,490248005
chrIX	59003	59166	163	0,059840772	0,085235	0,412479432	-0,221171546	-0,187995814	-0,097248005	-0,230248005	-1,490248005
chrIX	59591	59741	150	0,230814407	0,140823044	0,621074131	0,308303061	0,262057602	0,09893919	0,05693919	-1,20306081
chrIX	60731	60881	150	0,29065518	0,274234349	0,51453455	0,036440779	0,030974662	-0,129779436	-0,171779436	-1,431779436
chrIX	62677	62823	146	0,413186285	0,311293045	0,570321703	0,177193254	0,150614266	-0,003875339	-0,017875339	-1,277875339
chrIX	70968	71118	150	0,162424953	0,17417587	0,482544729	-0,043767845	-0,037202668	-0,192228197	-0,234228197	-1,494228197
chrIX	77046	77204	158	0,869115978	0,826408916	0,512594053	0,031573855	0,026837777	0,057249924	-0,040750076	-1,300750076
chrIX	78123	78268	145	2,427825617	1,560171093	0,608783255	0,276149178	0,234726801	0,091962418	0,084962418	-1,175037582
chrIX	80413	80548	135	0,119681545	0,122293696	0,49460244	-0,013530088	-0,011500575	0,047001473	0,110001473	-1,149998527
chrIX	89142	89281	139	0,202318801	0,218646305	0,480607058	-0,048630057	-0,041335549	-0,089422241	-0,054422241	-1,314422241
chrIX	91580	91733	153	0,296354301	0,211234566	0,583847125	0,211745295	0,1799835	0,113408267	0,050408267	-1,209591733
chrIX	93328	93477	149	0,530018269	0,222352175	0,704464501	0,537284661	0,456691962	0,284259101	0,249259101	-1,010740899
chrIX	96377	96489	112	0,424584527	4,157985667	0,092652051	-1,324599259	-1,12590937	-0,56614521	-0,34214521	-1,60214521
chrIX	97100	97255	155	0,042743409	0,074117392	0,365763444	-0,34309514	-0,291630869	-0,320090971	-0,397090971	-1,657090971
chrIX	101159	101314	155	0,156725832	0,200116957	0,439201342	-0,152994409	-0,130045247	-0,159745126	-0,236745126	-1,496745126
chrIX	101159	101314	155	0,156725832	0,200116957	0,439201342	-0,152994409	-0,130045247	-0,159745126	-0,236745126	-1,496745126
chrIX	109382	109534	152	0,284956058	0,226058044	0,55762856	0,144959461	0,123215542	0,043797221	-0,012202779	-1,272202779
chrIX	122918	123072	154	0,122531105	0,159352392	0,434687048	-0,164453569	-0,139785534	-0,182403055	-0,252403055	-1,512403055

Chrom	Start	End	Length	Section A	Section B	A/A+B	Z-score	Z * 0.85	Phase correction	Length correction	ΔLKnuc
chrIX	123420	123580	160	0,042743409	0,059293913	0,418899751	-0,204708983	-0,174002636	-0,097104963	-0,209104963	**-1,469104963**
chrIX	123420	123580	160	0,042743409	0,059293913	0,418899751	-0,204708983	-0,174002636	-0,097104963	-0,209104963	**-1,469104963**
chrIX	123668	123818	150	0,102584181	0,096352609	0,515662191	0,039269381	0,033378974	-0,120071678	-0,162071678	**-1,422071678**
chrIX	127761	127867	106	0,299203861	1,182172396	0,201976952	-0,834580573	-0,709393487	-0,187987067	0,078012933	**-1,181987067**
chrIX	127761	127880	119	0,376141997	1,423053918	0,209061167	-0,8096831	-0,688230635	-0,198622363	-0,023622363	**-1,283622363**
chrIX	129250	129396	146	0,581310359	0,26682261	0,685400026	0,482853237	0,410425251	0,250406189	0,236406189	**-1,023593811**
chrIX	132115	132265	150	0,307752543	0,244587392	0,557179598	0,143822286	0,122248943	-0,035879065	-0,077879065	**-1,337879065**
chrIX	140470	140605	135	0,074088575	0,137117174	0,350788628	-0,383192206	-0,325713375	-0,28975792	-0,22675792	**-1,48675792**
chrIX	140794	140945	151	0,128230226	0,125999566	0,504387095	0,010997038	0,009347483	-0,136811521	-0,185811521	**-1,445811521**
chrIX	145476	145609	133	1,347842156	0,993173047	0,575751133	0,19103556	0,162380226	0,304864899	0,381864899	**-0,878135101**
chrIX	145755	145902	147	0,210867483	0,203822827	0,508493876	0,021292599	0,018098709	-0,154251811	-0,175251811	**-1,435251811**
chrIX	149396	149568	172	0,663947616	1,096937395	0,377053363	-0,313228948	-0,266244606	-0,024252043	-0,220252043	**-1,480252043**
chrIX	150158	150305	147	0,470177496	0,385410436	0,549537316	0,124492463	0,105818594	-0,062166142	-0,083166142	**-1,343166142**
chrIX	154400	154561	161	0,128230226	0,140823044	0,47659791	-0,058694023	-0,04988992	0,015001562	-0,103998438	**-1,363998438**
chrIX	157942	158056	114	0,344796831	1,104349134	0,237931057	-0,712973568	-0,606027533	-0,038522929	0,171477071	**-1,088522929**
chrIX	158500	158651	151	0,478726178	0,329822392	0,592080916	0,232901146	0,197965974	0,057280364	0,008280364	**-1,251719636**
chrIX	159528	159680	152	1,966196803	1,226642831	0,615814456	0,294506251	0,250330313	0,177272698	0,121272698	**-1,138727302**
chrIX	160370	160515	145	0,074088575	0,051882174	0,588141101	0,222765786	0,189350918	0,049174709	0,042174709	**-1,217825291**
chrIX	164945	165092	147	0,666797177	0,400233914	0,624908854	0,318399007	0,270639156	0,098542471	0,077542471	**-1,182457529**
chrIX	167654	167783	129	1,615700851	2,445873922	0,397801577	-0,259041586	-0,220185348	0,090794051	0,195794051	**-1,064205949**
chrIX	170117	170264	147	0,968850598	0,596645002	0,61887788	0,302535022	0,257154768	0,081088001	0,060088001	**-1,199911999**
chrIX	175448	175623	175	0,971700159	1,045055221	0,481813595	-0,045602359	-0,038762005	0,29007498	0,07307498	**-1,18692502**
chrIX	175932	176081	149	0,435982769	0,296469566	0,595237052	0,241037703	0,204882048	0,049475732	0,014475732	**-1,245524268**
chrIX	176147	176286	139	1,225311051	0,978349569	0,556034373	0,14092239	0,119784031	0,064799404	0,099799404	**-1,160200596**
chrIX	178557	178730	173	0,889062902	0,818997177	0,520510322	0,051434423	0,043719259	0,351253929	0,148253929	**-1,111746071**
chrIX	182864	183017	153	0,108283302	0,077823261	0,581834946	0,206589951	0,175601459	0,116675414	0,053675414	**-1,206324586**
chrIX	190132	190312	180	0,059840772	0,148234783	0,287591554	-0,560434497	-0,476369322	-0,12557736	-0,37757736	**-1,63757736**
chrIX	190978	191126	148	0,316301225	0,218646305	0,591275232	0,230826573	0,196202587	0,018654704	-0,009345296	**-1,269345296**
chrIX	192456	192591	135	0,322000346	0,289057827	0,526955305	0,067618422	0,057475659	0,073133909	0,136133909	**-1,123866091**
chrIX	192983	193089	106	0,068389454	0,448410219	0,132332619	-1,115432247	-0,94811741	-0,413425886	-0,147425886	**-1,407425886**
chrIX	192983	193098	115	0,085486818	0,474351306	0,152699171	-1,024925498	-0,871186673	-0,322278188	-0,119278188	**-1,379278188**
chrIX	193702	193858	156	1,12557643	0,978349569	0,534988603	0,087816159	0,074643735	0,056960887	-0,027039113	**-1,287039113**
chrIX	196790	196938	148	0,162424953	0,085235	0,655838584	0,40113215	0,340962327	0,161937104	0,133937104	**-1,126062896**
chrIX	200251	200397	146	0,199469241	0,244587392	0,449197751	-0,127688487	-0,108535214	-0,279507125	-0,293507125	**-1,553507125**
chrIX	205441	205561	120	0,190920559	0,607762611	0,239044175	-0,709380558	-0,602973475	-0,136780623	0,031219377	**-1,228780623**
chrIX	208655	208802	147	0,518620026	0,281646088	0,64805946	0,380086672	0,323073671	0,134711989	0,113711989	**-1,146288011**
chrIX	212863	213023	160	0,897611584	0,837526525	0,517314201	0,043413899	0,036901814	0,102378238	-0,009621762	**-1,269621762**
chrIX	213578	213684	106	1,151222476	2,494050226	0,315812443	-0,479441066	-0,407524906	0,143782447	0,409782447	**-0,850217553**
chrIX	213578	213686	108	0,031345166	0,062999783	0,332240005	-0,433736208	-0,368675777	0,207129567	0,459129567	**-0,800870433**
chrIX	213578	213701	123	1,179718082	3,161106751	0,271772791	-0,607460147	-0,516341125	-0,078552165	0,068447835	**-1,191552165**
chrIX	213866	214017	151	0,14532759	0,203822827	0,416232039	-0,211542365	-0,17981101	-0,333303779	-0,382303779	**-1,642303779**
chrIX	213866	214017	151	0,14532759	0,203822827	0,416232039	-0,211542365	-0,17981101	-0,333303779	-0,382303779	**-1,642303779**
chrIX	217876	218011	135	0,105433742	0,059293913	0,640048823	0,358589298	0,304800903	0,308413673	0,371413673	**-0,888586327**
chrIX	218740	218900	160	2,689985191	2,875754793	0,483311329	-0,041844502	-0,035567826	0,025320978	-0,086679022	**-1,346679022**
chrIX	223366	223481	115	0,068389454	0,496586524	0,121048428	-1,16976176	-0,994297496	-0,448521161	-0,245521161	**-1,505521161**
chrIX	224421	224561	140	0,225115286	0,233469783	0,490891006	-0,022834846	-0,019409619	-0,113669602	-0,085669602	**-1,345669602**
chrIX	236168	236319	151	0,230814407	0,096352609	0,705494123	0,540268681	0,459228379	0,300224443	0,251224443	**-1,008775557**
chrIX	240288	240448	160	0,541416511	0,522527611	0,508876829	0,022252747	0,018914835	0,084427963	-0,027572037	**-1,287572037**
chrIX	241031	241216	185	0,068389454	0,055588044	0,551627959	0,129775456	0,110309137	0,484910274	0,197910274	**-1,062089726**
chrIX	241349	241481	132	1,846515258	1,552759353	0,543208616	0,108520566	0,092242481	0,278590676	0,362590676	**-0,897409324**
chrIX	244655	244803	148	1,322196111	0,904232177	0,593864225	0,23749661	0,201872118	0,023608413	-0,004391587	**-1,264391587**
chrIX	244820	244976	156	0,481575739	0,396528045	0,548426903	0,121687903	0,103434718	0,088756425	0,004756425	**-1,255243575**
chrIX	244822	244983	161	0,435982769	0,370586958	0,540539466	0,101792891	0,086523957	0,154595562	0,035595562	**-1,224404438**
chrIX	247146	247297	151	0,031345166	0,074117392	0,297216064	-0,532424343	-0,452560692	-0,599432857	-0,648432857	**-1,908432857**
chrIX	249643	249773	130	0,510071344	0,659644785	0,436064214	-0,160955528	-0,136812198	0,138956314	0,236956314	**-1,023043686**
chrIX	254780	254919	139	0,142478029	0,222352175	0,390532439	-0,277931585	-0,236241848	-0,311526198	-0,276526198	**-1,536526198**
chrIX	255472	255626	154	1,473222822	1,119172613	0,568286305	0,172012896	0,146210962	0,111460975	0,041460975	**-1,218539025**
chrIX	255629	255772	143	0,700991904	0,600350872	0,538668149	0,097078943	0,082517102	-0,024251977	-0,017251977	**-1,277251977**
chrIX	255629	255776	147	1,627099093	0,818997177	0,665181953	0,4266475	0,362650375	0,17307808	0,15207808	**-1,10792192**
chrIX	258137	258291	154	0,031345166	0,081529131	0,277699771	-0,589688448	-0,501235181	-0,540358384	-0,610358384	**-1,870358384**
chrIX	258137	258291	154	0,031345166	0,081529131	0,277699771	-0,589688448	-0,501235181	-0,540358384	-0,610358384	**-1,870358384**
chrIX	263024	263196	172	0,125380666	0,059293913	0,678927584	0,464702061	0,394996752	0,669741742	0,473741742	**-0,786258258**
chrIX	265122	265273	151	1,607152169	0,848644133	0,654432197	0,397314432	0,337717267	0,197350332	0,148350332	**-1,111649668**
chrIX	265238	265355	117	0,056991212	1,067290439	0,050691223	-1,638188175	-1,392459948	-0,865674668	-0,676674668	**-1,936674668**
chrIX	270073	270226	153	3,630340183	2,81275501	0,563446616	0,159713497	0,135756473	0,082062898	0,019062898	**-1,240937102**
chrIX	271691	271837	146	0,381841118	0,344645871	0,52559939	0,064212254	0,054580416	-0,111880416	-0,125880416	**-1,385880416**
chrIX	280995	281156	161	0,34194727	0,248293262	0,57933546	0,200193718	0,17016466	0,245134221	0,126134221	**-1,133865779**
chrIX	282582	282731	149	0,042743409	0,037058696	0,535617563	0,08939893	0,07598909	-0,095150929	-0,130150929	**-1,390150929**
chrIX	283375	283539	164	0,113982423	0,207528696	0,354520937	-0,373143192	-0,317171714	-0,210878615	-0,350878615	**-1,610878615**
chrIX	285840	285993	153	0,287805619	0,277940218	0,508718933	0,021856865	0,018578335	-0,03465103	-0,09765103	**-1,35765103**
chrIX	285840	285993	153	0,287805619	0,277940218	0,508718933	0,021856865	0,018578335	-0,03465103	-0,09765103	**-1,35765103**
chrIX	286233	286373	140	0,310602104	0,085235	0,784671524	0,788067966	0,669857771	0,568021848	0,596021848	**-0,663978152**
chrIX	287987	288121	134	0,287805619	0,522527611	0,355169464	-0,371400936	-0,315690796	-0,205324736	-0,135324736	**-1,395324736**
chrIX	289697	289831	134	1,538762715	1,32299544	0,537698377	0,094636892	0,080441358	0,189289507	0,259289507	**-1,000710493**
chrIX	289822	289971	149	0,552814753	0,444704349	0,554189641	0,136253708	0,115815652	-0,058811109	-0,093811109	**-1,353811109**
chrIX	292476	292604	128	0,347646391	0,429880871	0,447117944	-0,132946251	-0,113004313	0,208997126	0,320997126	**-0,939002874**
chrIX	296896	297052	156	0,136778908	0,140823044	0,492715945	-0,018259434	-0,015520519	-0,01982984	-0,10382984	**-1,36382984**
chrIX	299139	299300	161	0,681044979	0,552174567	0,552249582	0,131346972	0,111644926	0,175505743	0,056505743	**-1,203494257**
chrIX	301522	301672	150	0,182371877	0,137117174	0,570823558	0,178471268	0,151700578	-0,00402044	-0,04602044	**-1,30602044**
chrIX	301692	301851	159	0,757983115	0,900526308	0,457026716	-0,107927211	-0,091738129	-0,044464246	-0,149464246	**-1,409464246**
chrIX	301709	301857	148	0,504372223	0,322410653	0,610041932	0,279428325	0,237514076	0,068192412	0,040192412	**-1,219807588**
chrIX	306823	306944	121	0,726637949	2,401403487	0,232298057	-0,731299698	-0,621604744	-0,15745718	0,00354282	**-1,25645718**
chrIX	309339	309491	152	0,652549374	0,741173916	0,468205833	-0,079780711	-0,067813604	-0,136785614	-0,192785614	**-1,452785614**
chrIX	309860	310002	142	3,693030516	2,605226314	0,586357561	0,218185122	0,185457353	0,0793834	0,0933834	**-1,1666166**
chrIX	314424	314578	154	0,336248149	0,214940436	0,610041932	0,279428325	0,237514076	0,203602018	0,133602018	**-1,126397982**
chrIX	320445	320583	138	0,210867483	0,255705001	0,451950105	-0,120735916	-0,102625529	-0,134205055	-0,092205055	**-1,352205055**
chrIX	331176	331345	169	0,122531105	0,185293479	0,398054968	-0,258384812	-0,21962709	0,007193209	-0,167806791	**-1,427806791**
chrIX	332149	332296	147	0,068389454	0,048176305	0,586702775	0,219071373	0,186210667	0,001086874	-0,019913126	**-1,279913126**
chrIX	333705	333871	166	0,213717044	0,448410219	0,322773364	-0,459957499	-0,390963874	-0,230484936	-0,384484936	**-1,644484936**
chrIX	338938	339077	139	0,131079787	0,059293913	0,688539366	0,491714385	0,417957228	0,329587138	0,364587138	**-0,895412862**
chrIX	339456	339607	151	0,367593315	0,188999349	0,660435071	0,413650807	0,351603186	0,217904632	0,168904632	**-1,091095368**
chrIX	343521	343669	148	0,612655525	0,400233914	0,604859229	0,265945033	0,226053278	0,053784546	0,025784546	**-1,234215454**
chrIX	345865	346018	153	0,170973635	0,040764565	0,807476566	0,868634885	0,738339652	0,68467303	0,62167303	**-0,63832697**
chrIX	346400	346566	166	0,24506221	0,163058261	0,60046537	0,254551841	0,216369065	0,384382689	0,230382689	**-1,029617311**
chrIX	352082	352233	151	0,09973462	0,148234783	0,402205349	-0,247642923	-0,210496484	-0,338800673	-0,387800673	**-1,647800673**
chrIX	360987	361138	151	5,265987958	3,43163523	0,60545138	0,267483079	0,227360617	0,098296546	0,049296546	**-1,210703454**

Chrom	Start	End	Length	Section A	Section B	A/A+B	Z-score	Z * 0.85	Phase correction	Length correction	ΔLKnuc
chrIX	361485	361638	153	1,191116324	0,637409568	0,651407962	0,389124465	0,330755795	0,280456639	0,217456639	**-1,042543361**
chrIX	364237	364386	149	0,082637257	0,051882174	0,614314648	0,290582424	0,24699506	0,078083179	0,043083179	**-1,216916821**
chrIX	365450	365589	139	1,27090402	1,05617283	0,546137537	0,115908669	0,098522369	0,007361026	0,042361026	**-1,217638974**
chrIX	371073	371217	144	0,82922213	0,559586306	0,59707452	0,24578204	0,208914734	0,080538698	0,080538698	**-1,179461302**
chrIX	377621	377774	153	0,068389454	0,077823261	0,467739443	-0,080953558	-0,068810524	-0,121311502	-0,184311502	**-1,444311502**
chrIX	378406	378536	130	0,225115286	0,322410653	0,411149993	-0,224587763	-0,190899598	0,05720642	0,15520642	**-1,10479358**
chrIX	380201	380307	106	0,037044288	0,270528479	0,120440727	-1,172786449	-0,996868482	-0,447369505	-0,181369505	**-1,441369505**
chrIX	380201	380310	109	0,039893848	0,307587175	0,11480871	-1,20134495	-1,021143207	-0,447718401	-0,202718401	**-1,462718401**
chrIX	381638	381781	143	0,076938136	0,096352609	0,443982948	-0,140878539	-0,119746758	-0,237053572	-0,230053572	**-1,490053572**
chrIX	382286	382451	165	0,09973462	0,218646305	0,313255639	-0,486643094	-0,41364663	-0,29619655	-0,44319655	**-1,70319655**
chrIX	386066	386219	153	0,273557816	0,188999349	0,591403262	0,231156174	0,196482748	0,159673874	0,096673874	**-1,163326126**
chrIX	386767	386910	143	2,014639333	1,915934572	0,512556024	0,031478483	0,02675671	-0,095305049	-0,088305049	**-1,348305049**
chrIX	387904	388014	110	0,373292436	2,116051529	0,14995615	-1,036621478	-0,881128257	-0,309240035	-0,071240035	**-1,331240035**
chrIX	388900	389051	151	0,190920559	0,26311674	0,420495319	-0,200626496	-0,170532521	-0,280890837	-0,329890837	**-1,589890837**
chrIX	390177	390342	165	3,171560929	3,776281101	0,456481439	-0,109302103	-0,092906788	0,055295516	-0,091704484	**-1,351704484**
chrIX	390386	390532	146	0,547115632	0,400233914	0,577522451	0,195559327	0,166225428	-0,003423677	-0,017423677	**-1,277423677**
chrIX	392788	392939	151	3,17441049	1,652817832	0,657605209	0,40593606	0,345045651	0,229725897	0,180725897	**-1,079274103**
chrIX	396135	396302	167	0,259310013	0,333528262	0,437404304	-0,15755355	-0,133920518	0,070293696	-0,090706304	**-1,350706304**
chrIX	396795	396922	127	0,074088575	0,181587609	0,289775034	-0,554042049	-0,470935742	-0,098545508	0,020454492	**-1,239545508**
chrIX	405278	405390	112	2,604498373	21,17533877	0,109525492	-1,229055535	-1,044697205	-0,474251564	-0,250251564	**-1,510251564**
chrIX	407378	407518	140	1,139824233	0,741173916	0,605967759	0,268824834	0,228501109	0,115642388	0,143642388	**-1,116357612**
chrIX	407520	407679	159	0,051292091	0,070411522	0,421450846	-0,198183249	-0,168455762	-0,107422306	-0,212422306	**-1,472422306**
chrIX	409036	409181	145	2,641542661	1,708405876	0,607258371	0,272180469	0,231353399	0,082802247	0,075802247	**-1,184197753**
chrIX	420519	420625	106	3,536304684	29,85819119	0,105894837	-1,248659409	-1,061360498	-0,499833426	-0,233833426	**-1,493833426**
chrIX	425922	426069	147	0,763682236	0,678174133	0,529652088	0,07439533	0,063236031	-0,124452334	-0,145452334	**-1,405452334**
chrIX	433552	433722	170	0,678195419	1,067290439	0,388542489	-0,283119835	-0,24065186	-0,008340168	-0,190340168	**-1,450340168**
chrIX	438504	438651	147	0,473027057	0,448410219	0,513357848	0,033489418	0,028466005	-0,152835083	-0,173835083	**-1,433835083**
chrIX	438570	438711	141	0,09688506	0,081529131	0,543034495	0,108081541	0,09186931	-0,023801477	-0,002801477	**-1,262801477**
chrIX	439579	439688	109	1,988993287	7,356151114	0,212837084	-0,796615851	-0,677123473	-0,09293233	0,15206767	**-1,10793233**
chrIX	439796	439887	91	0,170973635	0,600350872	0,221662392	-0,766590907	-0,651602271	-0,197286073	0,173713927	**-1,086286073**
chrV	1165	1320	155	0,547115632	0,518821741	0,513271836	0,033273697	0,028282642	-0,068729016	-0,145729016	**-1,405729016**
chrV	1854	1981	127	0,265009134	0,166764131	0,613769206	0,28915654	0,245783059	0,646234699	0,765234699	**-0,494765301**
chrV	3000	3144	144	0,091185939	0,037058696	0,711031218	0,556399818	0,472939845	0,300529854	0,300529854	**-0,959470146**
chrV	3648	3785	137	0,151026711	0,163058261	0,480846663	-0,048028754	-0,040824441	-0,085180049	-0,036180049	**-1,296180049**
chrV	3764	3919	155	0,062690333	0,185293479	0,252800102	-0,665704177	-0,56584855	-0,666651267	-0,743651267	**-2,003651267**
chrV	4024	4184	160	0,58700948	0,492880654	0,543582594	0,109463573	0,093044037	0,089543487	-0,022456513	**-1,282456513**
chrV	4831	4982	151	0,236513528	0,159352392	0,597458676	0,246774626	0,209758432	0,098465856	0,049465856	**-1,210534144**
chrV	7115	7272	157	0,173823196	0,237175653	0,422928668	-0,194406838	-0,165245812	-0,214806674	-0,305806674	**-1,565806674**
chrV	9760	9930	170	0,071239015	0,037058696	0,657807209	0,406485945	0,345513053	0,743886826	0,561886826	**-0,698113174**
chrV	11061	11170	109	0,324849907	2,490344357	0,115391648	-1,198343556	-1,018592022	-0,53523065	-0,29023065	**-1,55023065**
chrV	23046	23205	159	0,236513528	0,17788174	0,570743796	0,178268128	0,151527909	0,14142897	0,03642897	**-1,22357103**
chrV	26697	26869	172	0,116831984	0,159352392	0,423021699	-0,194169201	-0,165043821	0,306567242	0,110567242	**-1,149432758**
chrV	26697	26869	172	0,116831984	0,159352392	0,423021699	-0,194169201	-0,165043821	0,306567242	0,110567242	**-1,149432758**
chrV	27053	27184	131	7,545636425	7,011505243	0,518346019	0,046002869	0,039102439	0,263834413	0,354834413	**-0,905165587**
chrV	28903	29024	121	0,866266417	2,186463051	0,28376783	-0,571684613	-0,485931921	0,016509854	0,177509854	**-1,082490146**
chrV	32832	32992	160	0,128230226	0,188999349	0,404219015	-0,242441566	-0,206075331	-0,202941414	-0,314941414	**-1,574941414**
chrV	41648	41754	106	0,071239015	0,926467395	0,071402783	-1,465422925	-1,245609486	-0,786909181	-0,520909181	**-1,780909181**
chrV	41648	41774	126	0,031345166	0,059293913	0,345823971	-0,396619676	-0,337126724	0,086350864	0,212350864	**-1,047649136**
chrV	41648	41782	134	0,028495606	0,192705218	0,128822331	-1,131975672	-0,962179321	-0,897017223	-0,827017223	**-2,087017223**
chrV	41648	41785	137	0,037044288	0,044470435	0,454449042	-0,114428548	-0,097264266	-0,151538346	-0,102538346	**-1,362538346**
chrV	41648	41795	147	0,037044288	0,040764565	0,47609348	-0,059960669	-0,050966568	-0,226225129	-0,247225129	**-1,507225129**
chrV	41648	41795	147	0,037044288	0,040764565	0,47609348	-0,059960669	-0,050966568	-0,226225129	-0,247225129	**-1,507225129**
chrV	41648	41797	149	0,09973462	0,055588044	0,642112476	0,3641111	0,309494435	0,1538125	0,1188125	**-1,1411875**
chrV	41648	41797	149	0,09973462	0,055588044	0,642112476	0,3641111	0,309494435	0,1538125	0,1188125	**-1,1411875**
chrV	41648	41798	150	0,153876272	0,100058479	0,605967759	0,268824834	0,228501109	0,096458292	0,054458292	**-1,205541708**
chrV	41648	41798	150	0,153876272	0,100058479	0,605967759	0,268824834	0,228501109	0,096458292	0,054458292	**-1,205541708**
chrV	41648	41799	151	0,076938136	0,044470435	0,633712557	0,341702373	0,290447017	0,179985154	0,130985154	**-1,129014846**
chrV	41648	41799	151	0,076938136	0,044470435	0,633712557	0,341702373	0,290447017	0,179985154	0,130985154	**-1,129014846**
chrV	41648	41800	152	0,031345166	0,048176305	0,394172367	-0,268460684	-0,228191582	-0,328917889	-0,384917889	**-1,644917889**
chrV	41648	41800	152	0,031345166	0,048176305	0,394172367	-0,268460684	-0,228191582	-0,328917889	-0,384917889	**-1,644917889**
chrV	41648	41801	153	0,076938136	0,107470218	0,417216109	-0,209020522	-0,177667444	-0,322272097	-0,385272097	**-1,645272097**
chrV	41648	41801	153	0,076938136	0,107470218	0,417216109	-0,209020522	-0,177667444	-0,322272097	-0,385272097	**-1,645272097**
chrV	41648	41802	154	47,4537324	27,73472793	0,631130524	0,334849059	0,2846217	0,173558409	0,103558409	**-1,156441591**
chrV	41648	41802	154	47,4537324	27,73472793	0,631130524	0,334849059	0,2846217	0,173558409	0,103558409	**-1,156441591**
chrV	41649	41802	153	0,051292091	0,081529131	0,386173911	-0,289305214	-0,245909432	-0,390590505	-0,453590505	**-1,713590505**
chrV	41649	41802	153	0,051292091	0,081529131	0,386173911	-0,289305214	-0,245909432	-0,390590505	-0,453590505	**-1,713590505**
chrV	41650	41802	152	0,045592969	0,08894087	0,338895921	-0,415478239	-0,353156503	-0,465225545	-0,521225545	**-1,781225545**
chrV	41650	41802	152	0,045592969	0,08894087	0,338895921	-0,415478239	-0,353156503	-0,465225545	-0,521225545	**-1,781225545**
chrV	41651	41802	151	0,076938136	0,092646739	0,453685128	-0,116356189	-0,098902761	-0,209929443	-0,258929443	**-1,518929443**
chrV	41651	41802	151	0,076938136	0,092646739	0,453685128	-0,116356189	-0,098902761	-0,209929443	-0,258929443	**-1,518929443**
chrV	41652	41802	150	0,09688506	0,077823261	0,554553208	0,137173592	0,116597553	-0,007938999	-0,049938999	**-1,309938999**
chrV	41652	41802	150	0,09688506	0,077823261	0,554553208	0,137173592	0,116597553	-0,007938999	-0,049938999	**-1,309938999**
chrV	41653	41802	149	0,065539893	0,081529131	0,445640364	-0,13668381	-0,116181238	-0,262833551	-0,297833551	**-1,557833551**
chrV	41653	41802	149	0,065539893	0,081529131	0,445640364	-0,13668381	-0,116181238	-0,262833551	-0,297833551	**-1,557833551**
chrV	41654	41802	148	0,076938136	0,100058479	0,434687048	-0,164453569	-0,139785534	-0,327267201	-0,355267201	**-1,615267201**
chrV	41654	41802	148	0,076938136	0,100058479	0,434687048	-0,164453569	-0,139785534	-0,327267201	-0,355267201	**-1,615267201**
chrV	41658	41802	144	0,028495606	0,062999783	0,311443082	-0,491764035	-0,41799943	-0,597621742	-0,597621742	**-1,857621742**
chrV	41658	41802	144	0,028495606	0,062999783	0,311443082	-0,491764035	-0,41799943	-0,597621742	-0,597621742	**-1,857621742**
chrV	42773	42909	136	0,045592969	0,040764565	0,527955893	0,070132481	0,059612609	0,038546852	0,094546852	**-1,165453148**
chrV	44013	44174	161	0,119681545	0,085235	0,584050178	0,21226584	0,180425964	0,191151844	0,072151844	**-1,187848156**
chrV	51922	52070	148	0,233663968	0,114881957	0,670396499	0,441008282	0,37485704	0,191077972	0,163077972	**-1,096922028**
chrV	56549	56680	131	2,741277281	3,261165229	0,456693634	-0,108767039	-0,092451983	0,110409905	0,201409905	**-1,058590095**
chrV	58655	58795	140	1,000195765	0,600350872	0,624908854	0,318399007	0,270639156	0,163878157	0,191878157	**-1,068121843**
chrV	63504	63668	164	0,094035499	0,118587827	0,442263326	-0,145233329	-0,12344833	0,021092963	-0,118907037	**-1,378907037**
chrV	64024	64155	131	0,162424953	0,314998914	0,340211213	-0,411886758	-0,350103745	-0,16730923	-0,07630923	**-1,33630923**
chrV	65475	65628	153	0,530018269	0,34835174	0,603411163	0,262186483	0,22285851	0,085660374	0,022660374	**-1,237339626**
chrV	69437	69601	164	1,347842156	1,133996091	0,543082192	0,108201802	0,091971532	0,240350819	0,100350819	**-1,159649181**
chrV	71549	71648	99	0,367593315	2,149404356	0,146044361	-1,05355059	-0,895518001	-0,434388459	-0,119388459	**-1,379388459**
chrV	72028	72188	160	0,09973462	0,107470218	0,481333454	-0,046807179	-0,039786102	-0,037697122	-0,149697122	**-1,409697122**
chrV	78159	78308	149	0,076938136	0,040764565	0,653664997	0,395234266	0,335949126	0,190857919	0,155857919	**-1,104142081**
chrV	78159	78308	149	0,076938136	0,040764565	0,653664997	0,395234266	0,335949126	0,190857919	0,155857919	**-1,104142081**
chrV	78447	78544	97	0,133929347	0,485468915	0,216224932	-0,785006317	-0,667255369	-0,222851563	0,106148437	**-1,153851563**
chrV	78782	78922	140	0,125380666	0,081529131	0,605967759	0,268824834	0,228501109	0,124749033	0,152749033	**-1,107250967**
chrV	79095	79249	154	0,524319147	0,322410653	0,619228409	0,30345494	0,257936699	0,153993948	0,083993948	**-1,176006052**
chrV	83616	83770	154	2,205559892	1,356348266	0,619207401	0,303399799	0,257889829	0,160306321	0,090306321	**-1,169693679**
chrV	84397	84539	142	0,624053768	0,318704784	0,661944425	0,417775659	0,35510931	0,20199085	0,21599085	**-1,04400915**

Chrom	Start	End	Length	Section A	Section B	A/A+B	Z-score	Z * 0.85	Phase correction	Length correction	ΔLKnuc
chrV	87005	87158	153	0,270708255	0,118587827	0,695378834	0,511155276	0,434481985	0,320577967	0,257577967	-1,002422033
chrV	92533	92664	131	2,023188014	2,701578923	0,428209061	-0,180935604	-0,153795263	0,007108844	0,098108844	-1,161891156
chrV	94783	94926	143	1,097080825	0,592939133	0,649152585	0,38303363	0,325578585	0,161088409	0,168088409	-1,091911591
chrV	96167	96341	174	0,262159574	0,251999131	0,509880648	0,024769644	0,021054198	0,504189345	0,294189345	-0,965810655
chrV	105359	105516	157	0,09688506	0,166764131	0,367477175	-0,338542574	-0,287761188	-0,325054623	-0,416054623	-1,676054623
chrV	105504	105653	149	0,190920559	0,059293913	0,763027643	0,716075528	0,608664198	0,450475331	0,415475331	-0,844524669
chrV	106502	106638	136	0,054141651	0,066705652	0,448017039	-0,130672891	-0,111071958	-0,135012408	-0,079012408	-1,339012408
chrV	112707	112854	147	0,09688506	0,055588044	0,635423938	0,346253615	0,294315573	0,12058787	0,09958787	-1,16041213
chrV	114004	114167	163	1,179718082	1,478641962	0,443776638	-0,14140086	-0,120190731	0,012290536	-0,120709464	-1,380709464
chrV	116119	116272	153	6,59103363	5,247511323	0,556743557	0,142718019	0,121310316	0,019072973	-0,043927027	-1,303927027
chrV	116657	116785	128	0,287805619	0,870879351	0,248389879	-0,679565285	-0,577630492	-0,196624347	-0,084624347	-1,344624347
chrV	119860	120019	159	0,045592969	0,044470435	0,506231912	0,015621722	0,013278464	-0,000154346	-0,105154346	-1,365154346
chrV	121418	121572	154	0,492973981	0,329822392	0,599144572	0,251133552	0,213463519	0,118194078	0,048194078	-1,211805922
chrV	127589	127741	152	0,09973462	0,062999783	0,61286746	0,286800501	0,243780426	0,120464269	0,064464269	-1,195535731
chrV	129771	129919	148	0,284956058	0,166764131	0,630824269	0,334037235	0,283931649	0,09111919	0,06311919	-1,19688081
chrV	132213	132348	135	0,43883233	0,600350872	0,422285819	-0,196049223	-0,16664184	-0,158209347	-0,095209347	-1,355209347
chrV	134881	135033	152	0,24221265	0,274234349	0,468998078	-0,077788674	-0,066120373	-0,182693151	-0,238693151	-1,498693151
chrV	137057	137219	162	0,492973981	0,470645436	0,511585769	0,0290453	0,024688505	0,074786651	-0,051213349	-1,311213349
chrV	137099	137245	146	0,236513528	0,151940653	0,608858238	0,276344444	0,234892777	0,036372228	0,022372228	-1,237627772
chrV	143216	143369	153	0,310602104	0,118587827	0,723693828	0,59385015	0,504772627	0,400696281	0,337696281	-0,922303719
chrV	146039	146192	153	0,159575393	0,103764348	0,605967759	0,268824834	0,228501109	0,120440237	0,057440237	-1,202559763
chrV	147368	147525	157	0,447381012	0,511410002	0,466609517	-0,08379549	-0,071226167	-0,101076532	-0,192076532	-1,452076532
chrV	147862	148011	149	0,125380666	0,096352609	0,56545715	0,164819952	0,140096959	-0,0120106	-0,0470106	-1,3070106
chrV	150905	151028	123	0,142478029	0,770820872	0,156003723	-1,011018769	-0,859365954	-0,366539424	-0,219539424	-1,479539424
chrV	154668	154823	155	0,227964847	0,274234349	0,453933118	-0,11573037	-0,098370815	-0,192104692	-0,269104692	-1,529104692
chrV	154964	155109	145	0,284956058	0,370586958	0,434687048	-0,164453569	-0,139785534	-0,338098809	-0,345098809	-1,605098809
chrV	156735	156886	151	0,068389454	0,114881957	0,373159423	-0,323497043	-0,274972487	-0,384406271	-0,433406271	-1,693406271
chrV	157741	157900	159	0,125380666	0,044470435	0,738179884	0,637744162	0,542082538	0,544873574	0,439873574	-0,820126426
chrV	158361	158517	156	0,113982423	0,125999566	0,474962408	-0,062801193	-0,053381014	-0,113691927	-0,197691927	-1,457691927
chrV	158999	159169	170	0,074088575	0,159352392	0,317376063	-0,47504889	-0,403791556	0,008831741	-0,173168259	-1,433168259
chrV	161114	161267	153	1,091381703	0,885702829	0,552015701	0,130755661	0,111142312	-0,02070096	-0,08370096	-1,34370096
chrV	162828	162978	150	0,190920559	0,17417587	0,522931872	0,057513369	0,048886364	-0,085541174	-0,127541174	-1,387541174
chrV	162990	163135	145	7,750804787	4,154279798	0,651049955	0,38815668	0,329933178	0,134311071	0,127311071	-1,132688929
chrV	163162	163326	164	0,743735312	0,685585872	0,520341628	0,051011015	0,043359362	0,190217642	0,050217642	-1,209782358
chrV	164105	164236	131	0,09688506	0,062999783	0,605967759	0,268824834	0,228501109	0,390321148	0,481321148	-0,778678852
chrV	165644	165751	107	0,094035499	0,155646522	0,376621026	-0,314367347	-0,267212245	0,180257713	0,439257713	-0,820742287
chrV	165645	165751	106	1,228160611	3,735516535	0,247429592	-0,682600712	-0,580210605	-0,137996043	0,128003957	-1,131996043
chrV	165645	165753	108	0,031345166	0,163058261	0,161237725	-0,989383693	-0,840976139	-0,373562961	-0,121562961	-1,381562961
chrV	165645	165770	125	1,598603487	4,117221102	0,279680292	-0,583791251	-0,496222793	-0,021063114	0,111936886	-1,148063114
chrV	169801	169967	166	0,051292091	0,155646522	0,247861382	-0,681235065	-0,579049806	-0,38064542	-0,53464542	-1,79464542
chrV	170735	170894	159	0,039893848	0,048176305	0,452978074	-0,118140734	-0,100419624	-0,091763496	-0,196763496	-1,456763496
chrV	175783	175962	179	0,68389454	1,738052832	0,282373824	-0,575804033	-0,489433428	-0,011190621	-0,256190621	-1,516190621
chrV	177369	177538	169	0,079787696	0,133411305	0,374240479	-0,320642986	-0,272546538	0,028469708	-0,146530292	-1,406530292
chrV	177369	177538	169	0,079787696	0,133411305	0,374240479	-0,320642986	-0,272546538	0,028469708	-0,146530292	-1,406530292
chrV	177766	177914	148	0,159575393	0,159352392	0,50034961	0,000876343	0,000744891	-0,194333537	-0,222333537	-1,482333537
chrV	179498	179607	109	0,034194727	0,815291307	0,04025343	-1,747752689	-1,485589785	-1,000610091	-0,755610091	-2,015610091
chrV	179498	179665	167	0,034194727	0,166764131	0,170157849	-0,953541662	-0,810510413	-0,583173751	-0,744173751	-2,004173751
chrV	179498	179665	167	0,034194727	0,166764131	0,170157849	-0,953541662	-0,810510413	-0,583173751	-0,744173751	-2,004173751
chrV	179498	179666	168	0,039893848	0,096352609	0,292806499	-0,545204323	-0,463423675	-0,21057675	-0,37857675	-1,63857675
chrV	179498	179666	168	0,039893848	0,096352609	0,292806499	-0,545204323	-0,463423675	-0,21057675	-0,37857675	-1,63857675
chrV	179498	179667	169	0,085486818	0,255705001	0,250553539	-0,67274886	-0,571836531	-0,245425085	-0,420425085	-1,680425085
chrV	179498	179667	169	0,085486818	0,255705001	0,250553539	-0,67274886	-0,571836531	-0,245425085	-0,420425085	-1,680425085
chrV	179498	179669	171	0,034194727	0,144528914	0,191327386	-0,873015237	-0,742062952	-0,31083774	-0,49983774	-1,75983774
chrV	179498	179669	171	0,034194727	0,144528914	0,191327386	-0,873015237	-0,742062952	-0,31083774	-0,49983774	-1,75983774
chrV	179498	179670	172	15,48166265	30,57712989	0,336128279	-0,423053049	-0,359595091	0,113381321	-0,082618679	-1,342618679
chrV	179498	179670	172	15,48166265	30,57712989	0,336128279	-0,423053049	-0,359595091	0,113381321	-0,082618679	-1,342618679
chrV	179499	179670	171	0,059840772	0,140823044	0,298214065	-0,529544001	-0,450112401	0,003907434	-0,185092566	-1,445092566
chrV	179499	179670	171	0,059840772	0,140823044	0,298214065	-0,529544001	-0,450112401	0,003907434	-0,185092566	-1,445092566
chrV	179502	179670	168	0,031345166	0,118587827	0,209061167	-0,8096831	-0,688230635	-0,413093399	-0,581093399	-1,841093399
chrV	179502	179670	168	0,031345166	0,118587827	0,209061167	-0,8096831	-0,688230635	-0,413093399	-0,581093399	-1,841093399
chrV	180478	180630	152	1,022992249	0,785644351	0,565615143	0,16522141	0,140438199	0,026604359	-0,029395641	-1,289395641
chrV	180478	180630	152	1,022992249	0,785644351	0,565615143	0,16522141	0,140438199	0,026604359	-0,029395641	-1,289395641
chrV	182865	183029	164	0,284956058	0,478057176	0,373461489	-0,322699306	-0,27429441	-0,109791068	-0,249791068	-1,509791068
chrV	186698	186836	138	9,155638155	5,996096978	0,604263345	0,264397925	0,224738237	0,167258922	0,209258922	-1,050741078
chrV	189568	189720	152	0,19661968	0,096352609	0,671120401	0,443009037	0,376557681	0,268755198	0,212755198	-1,047244802
chrV	190625	190773	148	1,014443568	0,767115003	0,569413537	0,174881273	0,148649082	-0,033681522	-0,061681522	-1,321681522
chrV	193660	193792	132	0,219416165	0,081529131	0,729089865	0,610062706	0,5185533	0,623434496	0,707434496	-0,552565504
chrV	194009	194135	126	0,068389454	0,092646739	0,424683747	-0,189925521	-0,161436693	0,248227019	0,374227019	-0,885772981
chrV	194030	194163	133	9,192682442	8,760675684	0,512031364	0,030162729	0,02563832	0,088255212	0,165255212	-1,094744788
chrV	195885	196040	155	0,316301225	0,17788174	0,640048823	0,358589298	0,304800903	0,23311419	0,15611419	-1,10388581
chrV	198265	198403	138	0,094035499	0,044470435	0,678927584	0,464702061	0,394996752	0,327236775	0,369236775	-0,890763225
chrV	201379	201531	152	10,68015307	8,53091177	0,555937589	0,140677372	0,119575766	0,00493926	-0,05106074	-1,31106074
chrV	201380	201531	151	0,056991212	0,051882174	0,523463207	0,058847485	0,050020363	-0,056803429	-0,105803429	-1,365803429
chrV	201382	201531	149	0,088336378	0,066705652	0,569757619	0,175757115	0,149393547	0,003451775	-0,031548225	-1,291548225
chrV	201383	201531	148	0,045592969	0,040764565	0,527955893	0,070132481	0,059612609	-0,114498629	-0,142498629	-1,402498629
chrV	206462	206572	110	0,088336378	0,537351089	0,141182911	-1,075019892	-0,913766908	-0,416113353	-0,178113353	-1,438113353
chrV	207496	207646	150	0,105433742	0,122293696	0,462982163	-0,092923514	-0,078984987	-0,21106086	-0,25306086	-1,51306086
chrV	212628	212767	139	0,113982423	0,081529131	0,582995843	0,209563572	0,178129036	0,087573978	0,122573978	-1,137426022
chrV	213547	213709	162	2,54180804	3,031401315	0,456076181	-0,110324078	-0,093775466	-0,03126739	-0,15726739	-1,41726739
chrV	215300	215450	150	0,065539893	0,070411522	0,4820832	-0,044925865	-0,038186985	-0,162001951	-0,204001951	-1,464001951
chrV	220704	220853	149	0,088336378	0,122293696	0,419391098	-0,203451441	-0,172933725	-0,324077269	-0,359077269	-1,619077269
chrV	226712	226861	149	0,173823196	0,122293696	0,587008713	0,219856939	0,186878398	0,037830098	0,002830098	-1,257169902
chrV	227455	227599	144	0,085486818	0,059293913	0,590457149	0,22872109	0,194412926	0,010215692	0,010215692	-1,249784308
chrV	234231	234385	154	0,997346204	0,541056958	0,648299632	0,380733867	0,323623787	0,218628317	0,148628317	-1,111371683
chrV	238011	238157	146	0,424584527	0,274234349	0,607574497	0,273002874	0,232052443	0,04056343	0,02656343	-1,23343657
chrV	239144	239298	154	0,068389454	0,048176305	0,586702775	0,219071373	0,186210667	0,082089419	0,012089419	-1,247910581
chrV	250567	250727	160	0,122531105	0,081529131	0,60046537	0,254551841	0,216369065	0,208964727	0,096964727	-1,163035273
chrV	250628	250757	129	1,473222822	2,245756965	0,396136281	-0,263360721	-0,223856613	0,082996262	0,187996262	-1,072003738
chrV	253010	253142	132	0,054141651	0,055588044	0,493409293	-0,016521205	-0,014043024	0,075990049	0,159990049	-1,100009951
chrV	255896	256035	139	0,549965193	0,581821524	0,485926531	-0,035284276	-0,029991635	-0,127552539	-0,092552539	-1,352552539
chrV	258267	258412	145	0,09973462	0,062999783	0,61286746	0,286800501	0,243780426	0,041710092	0,034710092	-1,225289908
chrV	263170	263306	136	0,119681545	0,133411305	0,472876041	-0,06804215	-0,057835827	-0,088138703	-0,032138703	-1,292138703
chrV	268138	268266	128	0,159575393	0,337234132	0,321200349	-0,464344846	-0,394693119	-0,022559319	0,089440681	-1,170559319
chrV	271754	271916	162	1,296550065	1,208113483	0,517654384	0,044267431	0,037627316	0,114984481	-0,011015519	-1,271015519
chrV	273268	273395	127	0,353345512	0,626291959	0,360690074	-0,356614863	-0,303122634	0,090243252	0,209243252	-1,050756748
chrV	274395	274545	150	0,085486818	0,051882174	0,622315244	0,311567137	0,264832067	0,136318916	0,094318916	-1,165681084

Chrom	Start	End	Length	Section A	Section B	A/A+B	Z-score	Z * 0.85	Phase correction	Length correction	ΔLKnuc
chrV	279045	279197	152	0,518620026	0,552174567	0,484331943	-0,039284096	-0,033391482	-0,149899569	-0,205899569	-1,465899569
chrV	282333	282495	162	0,122531105	0,181587609	0,402905508	-0,245833639	-0,208958593	-0,126285142	-0,252285142	-1,512285142
chrV	286305	286436	131	0,116831984	0,081529131	0,588986325	0,224938202	0,191197471	0,343300819	0,434300819	-0,825699181
chrV	293237	293387	150	0,575611238	0,626291959	0,478916471	-0,052873194	-0,044942215	-0,177895789	-0,219895789	-1,479895789
chrV	293855	294005	150	0,29065518	0,196411088	0,596746683	0,244935165	0,20819489	0,073830772	0,031830772	-1,228169228
chrV	298879	299018	139	0,527168708	0,470645436	0,528323547	0,071056352	0,060397899	-0,043345527	-0,008345527	-1,268345527
chrV	299205	299328	123	0,039893848	0,389116306	0,092990452	-1,322562523	-1,124178145	-0,635545952	-0,488545952	-1,748545952
chrV	299678	299831	153	0,649699813	0,481763045	0,574212232	0,187108535	0,159042255	0,029346533	-0,033653467	-1,293653467
chrV	301505	301647	142	0,125380666	0,08894087	0,585011979	0,214732295	0,182522451	0,021100065	0,035100065	-1,224899935
chrV	314667	314817	150	0,227964847	0,092646739	0,711031218	0,556399818	0,472939845	0,326463792	0,284463792	-0,975536208
chrV	315905	316063	158	0,085486818	0,066705652	0,561702018	0,155285859	0,13199298	0,120302862	0,022302862	-1,237697138
chrV	320077	320234	157	0,082637257	0,037058696	0,690393076	0,496964836	0,42242011	0,393607764	0,302607764	-0,957392236
chrV	321656	321815	159	0,09688506	0,148234783	0,395255882	-0,265646117	-0,2257992	-0,230969993	-0,335969993	-1,595969993
chrV	321656	321815	159	0,09688506	0,148234783	0,395255882	-0,265646117	-0,2257992	-0,230969993	-0,335969993	-1,595969993
chrV	323513	323661	148	0,852018614	0,403939784	0,678381239	0,46317697	0,393700425	0,212650082	0,184650082	-1,075349918
chrV	324743	324895	152	0,63260245	0,607762611	0,510013116	0,025101796	0,021336526	-0,094358804	-0,150358804	-1,410358804
chrV	324904	325052	148	0,629752889	0,381704567	0,622619256	0,312367184	0,265512106	0,091073817	0,063073817	-1,196926183
chrV	325348	325499	151	0,302053422	0,322410653	0,483700238	-0,040868818	-0,034738495	-0,136431373	-0,185431373	-1,445431373
chrV	328966	329128	162	0,074088575	0,070411522	0,512723359	0,03189814	0,027113419	0,112897669	-0,013102331	-1,273102331
chrV	331009	331174	165	0,142478029	0,188999349	0,429827309	-0,17681384	-0,150291764	0,058186504	-0,088813496	-1,348813496
chrV	333088	333236	148	0,071239015	0,096352609	0,425075031	-0,188926962	-0,160587917	-0,334644224	-0,362644224	-1,622644224
chrV	340240	340377	137	0,937505432	0,763409133	0,551177261	0,128636255	0,109340817	0,051901532	0,100901532	-1,159098468
chrV	340798	340946	148	0,179522317	0,163058261	0,524029464	0,060269401	0,051228991	-0,125410293	-0,153410293	-1,413410293
chrV	341934	342080	146	0,14817715	0,081529131	0,645072262	0,372050197	0,316242667	0,125567135	0,111567135	-1,148432865
chrV	349486	349586	100	9,993408966	28,46107836	0,259876276	-0,643726886	-0,547167853	-0,088302446	0,219697554	-1,040302446
chrV	351515	351672	157	0,125380666	0,17417587	0,418554265	-0,205593405	-0,174754394	-0,201705746	-0,292705746	-1,552705746
chrV	352323	352478	155	0,113982423	0,129705435	0,467739443	-0,080953558	-0,068810524	-0,139046162	-0,216046162	-1,476046162
chrV	360556	360703	147	0,042743409	0,040764565	0,51184823	0,029703476	0,025247955	-0,165553627	-0,186553627	-1,446553627
chrV	360556	360709	153	0,065539893	0,048176305	0,576346155	0,192554744	0,163671533	0,045315248	-0,017684752	-1,277684752
chrV	360596	360757	161	0,370442876	0,340940001	0,520736284	0,052001583	0,044201345	0,070736906	-0,048263094	-1,308263094
chrV	361101	361246	145	0,695292782	0,678174133	0,506231912	0,015621722	0,013278464	-0,191577418	-0,198577418	-1,458577418
chrV	364550	364707	157	0,3903898	0,429880871	0,475928024	-0,060376156	-0,051319733	-0,071709887	-0,162709887	-1,422709887
chrV	369752	369900	148	0,091185939	0,037058696	0,711031218	0,556399818	0,472939845	0,305120146	0,277120146	-0,982879854
chrV	370967	371076	109	0,133929347	0,659644785	0,16876728	-0,959048011	-0,81519081	-0,317652675	-0,072652675	-1,332652675
chrV	372915	373052	137	0,641151131	0,941290873	0,405165642	-0,239998674	-0,203998873	-0,261221282	-0,212221282	-1,472221282
chrV	373805	373958	153	0,638301571	0,496586524	0,562435692	0,157147484	0,133575361	0,020064557	-0,042935443	-1,302935443
chrV	376017	376164	147	0,948903674	0,685585872	0,58055047	0,203301913	0,172806626	-0,009673883	-0,030673883	-1,290673883
chrV	376371	376519	148	0,353345512	0,307587175	0,534616488	0,086879841	0,073847865	-0,087841574	-0,115841574	-1,375841574
chrV	376832	376976	144	0,418885406	0,222352175	0,653245253	0,394096909	0,334982372	0,146428127	0,146428127	-1,113571873
chrV	377517	377651	134	0,914708947	1,074702178	0,459788797	-0,10096582	-0,085820947	-0,014259516	0,055740484	-1,204259516
chrV	378722	378880	158	0,287805619	0,270528479	0,515472044	0,038792391	0,032973532	0,019199567	-0,078800433	-1,338800433
chrV	389589	389739	150	1,456125458	1,32299544	0,523951822	0,060074429	0,051063265	-0,09279508	-0,13479508	-1,39479508
chrV	392068	392208	140	0,04844253	0,077823261	0,383655221	-0,295894775	-0,251510559	-0,370776899	-0,342776899	-1,602776899
chrV	408769	408926	157	0,267858695	0,200116957	0,572377417	0,18243015	0,155065628	0,145743206	0,054743206	-1,205256794
chrV	411385	411539	154	2,05168362	1,374877614	0,598758779	0,250135657	0,212615308	0,11768312	0,04768312	-1,21231688
chrV	421211	421362	151	0,230814407	0,148234783	0,608929957	0,276531218	0,235051535	0,131165119	0,082165119	-1,177834881
chrV	426798	426949	151	0,034194727	0,037058696	0,479902939	-0,050397187	-0,042837609	-0,148116882	-0,197116882	-1,457116882
chrV	426821	426975	154	0,359044634	0,374292827	0,489603563	-0,026062954	-0,022153511	-0,116654544	-0,186654544	-1,446654544
chrV	428076	428230	154	0,082637257	0,08894087	0,481630488	-0,046061821	-0,039152548	-0,12927449	-0,19927449	-1,45927449
chrV	429069	429212	143	0,102584181	0,040764565	0,715626635	0,569898223	0,48441349	0,306917275	0,313917275	-0,946082725
chrV	434847	434995	148	0,569912117	0,429880871	0,57003012	0,176450848	0,149983221	-0,004879224	-0,032879224	-1,292879224
chrV	440738	440898	160	0,433133209	0,333528262	0,564960188	0,163557348	0,139023746	0,12875477	0,01675477	-1,24324523
chrV	444332	444446	114	0,04844253	0,307587175	0,136063169	-1,098178991	-0,933452143	-0,434456338	-0,224456338	-1,484456338
chrV	445028	445178	150	0,119681545	0,181587609	0,397257877	-0,260451199	-0,221383519	-0,344320469	-0,386320469	-1,646320469
chrV	446200	446349	149	0,09688506	0,037058696	0,723326439	0,592752021	0,503839218	0,341173135	0,306173135	-0,953826865
chrV	446424	446581	157	0,065539893	0,059293913	0,525017182	0,062749932	0,053337442	0,046711811	-0,044288189	-1,304288189
chrV	446610	446760	150	8,944770672	4,346985016	0,672956296	0,448091159	0,380877485	0,270271871	0,228271871	-1,031728129
chrV	446614	446760	146	0,074088575	0,044470435	0,624908854	0,318399007	0,270639156	0,080365727	0,066365727	-1,193634273
chrV	447072	447207	135	0,076938136	0,044470435	0,633712557	0,341702373	0,290447017	0,287223637	0,350223637	-0,909776363
chrV	447594	447738	144	0,04844253	0,037058696	0,566571175	0,16765125	0,142503563	-0,043305836	-0,043305836	-1,303305836
chrV	447618	447772	154	0,213717044	0,129705435	0,622315244	0,311567137	0,264832067	0,173086433	0,103086433	-1,156913567
chrV	447875	448026	151	0,04844253	0,037058696	0,566571175	0,16765125	0,142503563	0,032733921	-0,016266079	-1,276266079
chrV	447899	448044	145	0,111132863	0,051882174	0,681733814	0,472552647	0,40166975	0,204879692	0,197879692	-1,062120308
chrV	447903	448054	151	0,139628469	0,085235	0,620947766	0,307970909	0,261775272	0,148354874	0,099354874	-1,160645126
chrV	448110	448250	140	0,162424953	0,118587827	0,577998458	0,196775678	0,167259327	0,044908485	0,072908485	-1,187091515
chrV	448116	448250	134	0,131079787	0,111176087	0,541079911	0,103154718	0,08768151	0,147211649	0,217211649	-1,042788351
chrV	448528	448683	155	0,116831984	0,037058696	0,759188173	0,703693523	0,598139495	0,536734974	0,459734974	-0,800265026
chrV	448659	448767	108	0,074088575	0,133411305	0,357053581	-0,366345661	-0,311393812	0,171934774	0,423934774	-0,836065226
chrV	448808	448957	149	0,074088575	0,055588044	0,571333335	0,179769754	0,152804291	0,003004272	-0,031995728	-1,291995728
chrV	450553	450702	149	0,136778908	0,151940653	0,473743129	-0,065863805	-0,055984234	-0,203929923	-0,238929923	-1,498929923
chrV	451474	451643	169	0,336248149	0,511410002	0,396678954	-0,2619527	-0,222659795	0,129226717	-0,045773283	-1,305773283
chrV	456731	456859	128	0,082637257	0,370586958	0,182331955	-0,906513904	-0,770536818	-0,434477679	-0,322477679	-1,582477679
chrV	458282	458446	164	0,170973635	0,148234783	0,535617563	0,08939893	0,07598909	0,23683996	0,09683996	-1,16316004
chrV	458920	459063	143	4,391172859	2,067875225	0,6798483	0,467274637	0,397183441	0,215363801	0,222363801	-1,037636199
chrV	458920	459063	143	4,391172859	2,067875225	0,6798483	0,467274637	0,397183441	0,215363801	0,222363801	-1,037636199
chrV	460202	460309	107	0,028495606	0,574409785	0,04726381	-1,671983276	-1,421185784	-0,96904916	-0,71004916	-1,97004916
chrV	460202	460330	128	8,209584041	12,84824983	0,389858904	-0,279686797	-0,237733777	0,072737897	0,184737897	-1,075262103
chrV	460206	460309	103	0,04844253	0,092646739	0,343346664	-0,403346515	-0,342844538	0,119749143	0,406749143	-0,853250857
chrV	465104	465256	152	0,051292091	0,048176305	0,515662191	0,039269381	0,033378974	-0,091236171	-0,147236171	-1,407236171
chrV	466895	467059	164	0,056991212	0,074117392	0,434687048	-0,164453569	-0,139785534	0,050733324	-0,089266676	-1,349266676
chrV	471982	472140	158	0,971700159	1,159937178	0,455846847	-0,110902461	-0,094267092	-0,102037468	-0,200037468	-1,460037468
chrV	472402	472551	149	0,421734966	0,359469349	0,539852325	0,100061691	0,085052437	-0,070135805	-0,105135805	-1,365135805
chrV	472672	472832	160	2,473418586	2,204992399	0,528687752	0,071971616	0,061175874	0,068324761	-0,043675239	-1,303675239
chrV	472989	473145	156	0,376141997	0,222352175	0,628480635	0,327831948	0,278657156	0,228083011	0,144083011	-1,115916989
chrV	473919	474069	150	0,854868175	0,515115871	0,623998635	0,315999708	0,268599752	0,155634466	0,113634466	-1,146365534
chrV	481969	482118	149	0,350495952	0,270528479	0,564383516	0,162092556	0,137778672	-0,011220774	-0,046220774	-1,306220774
chrV	488812	488981	169	0,082637257	0,114881957	0,418375789	-0,206050355	-0,175142801	0,201633446	0,026633446	-1,233366554
chrV	489802	489965	163	0,056991212	0,055588044	0,506231912	0,015621722	0,013278464	0,15136756	0,01836756	-1,24163244
chrV	493537	493686	149	0,071239015	0,062999783	0,530688713	0,07700122	0,065451037	-0,075183782	-0,110183782	-1,370183782
chrV	495710	495860	150	9,138540791	4,239514798	0,683099329	0,476383282	0,40492579	0,28929017	0,24729017	-1,01270983
chrV	496172	496307	135	0,045592969	0,077823261	0,36942442	-0,33337817	-0,283371444	-0,285903475	-0,222903475	-1,482903475
chrV	497210	497350	140	0,133929347	0,103764348	0,563453511	0,159731003	0,135771353	0,009830864	0,037830864	-1,222169136
chrV	497216	497350	134	0,119681545	0,114881957	0,510230892	0,025647854	0,021800676	0,067639843	0,137639843	-1,122360157
chrV	497628	497783	155	0,09973462	0,074117392	0,573675388	0,185739279	0,157878387	0,101854433	0,024854433	-1,235145567
chrV	497759	497867	108	0,062690333	0,181587609	0,256635258	-0,653753674	-0,555690623	-0,071909369	0,180090631	-1,079909369
chrV	497893	498045	152	0,034194727	0,044470435	0,434687048	-0,164453569	-0,139785534	-0,276611579	-0,332611579	-1,592611579

Chrom	Start	End	Length	Section A	Section B	A/A+B	Z-score	Z * 0.85	Phase correction	Length correction	ΔLKnuc
chrV	501813	501975	162	1,769577122	1,567582832	0,5302644	0,075934513	0,064544336	0,157476764	0,031476764	**-1,228523236**
chrV	502108	502246	138	0,054141651	0,08894087	0,378394585	-0,309699913	-0,263244926	-0,329012948	-0,287012948	**-1,547012948**
chrV	503589	503721	132	2,10012615	2,078992834	0,502528442	0,006337906	0,00538722	0,086307199	0,170307199	**-1,089692801**
chrV	504504	504656	152	0,074088575	0,040764565	0,645072262	0,372050197	0,316242667	0,172576537	0,116576537	**-1,143423463**
chrV	506128	506280	152	1,21961193	0,88199696	0,580322978	0,202719804	0,172311833	0,023121974	-0,032878026	**-1,292878026**
chrV	508434	508572	138	0,210867483	0,214940436	0,495217383	-0,01198853	-0,010190251	-0,087140811	-0,045140811	**-1,305140811**
chrV	508812	508941	129	0,091185939	0,170470001	0,348495581	-0,389385276	-0,330977484	-0,04527805	0,05972195	**-1,20027805**
chrV	517240	517402	162	1,812320531	1,993757833	0,476164797	-0,059781583	-0,050814346	0,05028485	-0,07571515	**-1,33571515**
chrV	522575	522735	160	0,484425299	0,785644351	0,381416326	-0,301763114	-0,256498647	-0,255618347	-0,367618347	**-1,627618347**
chrV	525612	525757	145	0,227964847	0,166764131	0,577522451	0,195559327	0,166225428	-0,026545217	-0,033545217	**-1,293545217**
chrV	528789	528933	144	0,054141651	0,048176305	0,529151025	0,07313593	0,062165541	-0,131505336	-0,131505336	**-1,391505336**
chrV	536553	536715	162	0,407487163	0,470645436	0,464038305	-0,090265028	-0,076725274	0,016068058	-0,109931942	**-1,369931942**
chrV	538015	538173	158	0,122531105	0,137117174	0,47191187	-0,070464771	-0,059895055	-0,064613978	-0,162613978	**-1,422613978**
chrV	540818	540968	150	0,056991212	0,074117392	0,434687048	-0,164453569	-0,139785534	-0,254260148	-0,296260148	**-1,556260148**
chrV	541050	541210	160	0,108283302	0,08894087	0,549036667	0,123227859	0,104743681	0,107835625	-0,004164375	**-1,264164375**
chrV	541050	541210	160	0,108283302	0,08894087	0,549036667	0,123227859	0,104743681	0,107835625	-0,004164375	**-1,264164375**
chrV	541616	541757	141	1,490320185	1,115466743	0,571927109	0,181282576	0,15409019	0,025348572	0,046348572	**-1,213651428**
chrV	541626	541757	131	0,119681545	0,070411522	0,629594476	0,330779497	0,281162572	0,409048251	0,500048251	**-0,759951749**
chrV	541626	541777	151	0,530018269	0,285351958	0,650033876	0,385411926	0,327600137	0,21766675	0,16866675	**-1,09133325**
chrV	542716	542874	158	0,056991212	0,133411305	0,299319635	-0,52635832	-0,447404572	-0,467210392	-0,565210392	**-1,825210392**
chrV	544584	544725	141	2,6386931	1,945581529	0,575596646	0,190641201	0,162045021	0,028699208	0,049699208	**-1,210300792**
chrV	560594	560752	158	0,530018269	0,470645436	0,529666726	0,074432125	0,063267306	0,04622618	-0,05177382	**-1,31177382**
chrV	560787	560934	147	0,108283302	0,055588044	0,660782406	0,414599399	0,352409489	0,155744156	0,134744156	**-1,125255844**
chrV	561033	561174	141	0,718089267	0,481763045	0,59848138	0,249418287	0,212005544	0,07555097	0,09655097	**-1,16344903**
chrV	561033	561174	141	0,718089267	0,481763045	0,59848138	0,249418287	0,212005544	0,07555097	0,09655097	**-1,16344903**
chrV	571735	571876	141	0,105433742	0,066705652	0,612490489	0,28581604	0,242943634	0,095001421	0,116001421	**-1,143998579**
chrV	572939	573101	162	0,051292091	0,048176305	0,515662191	0,039269381	0,033378974	0,149819157	0,023819157	**-1,236180843**
chrV	573040	573195	155	0,076938136	0,207528696	0,270464346	-0,611409232	-0,519697847	-0,586495307	-0,663495307	**-1,923495307**
chrV	573174	573311	137	0,142478029	0,118587827	0,545755127	0,114943701	0,097702146	0,039369336	0,088369336	**-1,171630664**
chrV	573461	573612	151	0,074088575	0,037058696	0,666580246	0,43048963	0,365916185	0,268751335	0,219751335	**-1,040248665**
chrV	573469	573612	143	0,393239361	0,452116089	0,465176348	-0,087401099	-0,074290934	-0,249217954	-0,242217954	**-1,502217954**
chrV	573517	573642	125	0,173823196	0,226058044	0,434687048	-0,164453569	-0,139785534	0,305728929	0,438728929	**-0,821271071**
chrV	573742	573894	152	0,091185939	0,059293913	0,605967759	0,268824834	0,228501109	0,079872155	0,023872155	**-1,236127845**
chrV	573815	573959	144	0,105433742	0,044470435	0,703340921	0,534033788	0,453928719	0,25992553	0,25992553	**-1,00007447**
chrV	573912	574047	135	1,262355338	0,837526525	0,601155408	0,256338873	0,217888042	0,20290009	0,26590009	**-0,99409991**
chrV	573912	574099	187	0,139628469	0,081529131	0,631352796	0,335438395	0,285122636	0,776387644	0,475387644	**-0,784612356**
chrV	573955	574047	92	0,151026711	0,144528914	0,510992512	0,027557628	0,023423984	0,411193949	0,775193949	**-0,484806051**
chrV	573955	574099	144	1,373488201	0,626291959	0,686819596	0,486855398	0,413827089	0,225517805	0,225517805	**-1,034482195**
chrV	574294	574446	152	0,168124074	0,148234783	0,5314347	0,07887682	0,067045297	-0,082100759	-0,138100759	**-1,398100759**
chrV	574595	574725	130	0,384690679	0,470645436	0,449753812	-0,126283363	-0,107340859	0,159978061	0,257978061	**-1,002021939**
chrV	574965	575116	151	0,498673102	0,248293262	0,667597801	0,433289593	0,368296154	0,264436286	0,215436286	**-1,044563714**
chrV	574970	575104	134	0,626903328	0,444704349	0,585011979	0,214732295	0,182522451	0,216984343	0,286984343	**-0,973015657**
chrV	574983	575079	96	0,068389454	0,111176087	0,380860679	-0,303221116	-0,257737948	0,155770281	0,491770281	**-0,768229719**
chrV	575037	575152	115	0,113982423	0,114881957	0,498034789	-0,004926073	-0,004187162	0,491652003	0,694652003	**-0,565347997**
chrV	576130	576281	151	0,549965193	0,35576348	0,607207445	0,272048002	0,231240802	0,121723756	0,072723756	**-1,187276244**
chrVI	257	431	174	0,037044288	0,040764565	0,47609348	-0,059960669	-0,050966568	0,269885654	0,059885654	**-1,200114346**
chrVI	377	525	148	0,056991212	0,062999783	0,474962408	-0,062801193	-0,053381014	-0,19273657	-0,22073657	**-1,48073657**
chrVI	2507	2632	125	0,202318801	0,218646305	0,480607058	-0,048630057	-0,041335549	0,368938868	0,501938868	**-0,758061132**
chrVI	2537	2680	143	0,307752543	0,422469132	0,421450846	-0,198183249	-0,168455762	-0,185728731	-0,178728731	**-1,438728731**
chrVI	2537	2688	151	0,039893848	0,037058696	0,518421434	0,046192108	0,039263292	-0,077433233	-0,126433233	**-1,386433233**
chrVI	2828	2965	137	0,558513874	0,518821741	0,518421434	0,046192108	0,039263292	0,200811074	0,249811074	**-1,010188926**
chrVI	3919	4067	148	0,14532759	0,077823261	0,65125268	0,388704654	0,330398956	0,189217026	0,161217026	**-1,098782974**
chrVI	4714	4814	100	0,527168708	0,200116957	0,724844079	0,597292902	0,507698967	1,014255146	1,322255146	**0,062255146**
chrVI	6022	6182	160	0,478726178	0,329822392	0,592080916	0,232901146	0,197965974	0,162385959	0,050385959	**-1,209614041**
chrVI	13717	13867	150	0,062690333	0,044470435	0,585011979	0,214732295	0,182522451	0,051743114	0,009743114	**-1,250256886**
chrVI	15894	16044	150	3,439419624	1,93446392	0,640024964	0,358525523	0,304746694	0,17766544	0,13566544	**-1,12433456**
chrVI	24066	24172	106	0,507221784	2,416226965	0,173501172	-0,940419656	-0,799356708	-0,338111344	-0,072111344	**-1,332111344**
chrVI	24067	24172	105	0,034194727	0,426175002	0,074276663	-1,444660304	-1,227961258	-0,74528138	-0,47228138	**-1,73228138**
chrVI	29981	30130	149	0,507221784	0,592939133	0,461043268	-0,097805758	-0,083134894	-0,217408731	-0,252408731	**-1,512408731**
chrVI	36134	36286	152	0,04844253	0,048176305	0,50137771	0,003453413	0,002935401	-0,098020516	-0,154020516	**-1,414020516**
chrVI	36777	36932	155	0,216566604	0,08894087	0,708874979	0,550101085	0,467585923	0,367772466	0,290772466	**-0,969227534**
chrVI	41762	41909	147	0,091185939	0,040764565	0,691061693	0,498861988	0,42403269	0,329548098	0,308548098	**-0,951451902**
chrVI	48029	48199	170	2,704232994	3,368635447	0,445297477	-0,137551417	-0,116918705	0,110350476	-0,071649524	**-1,331649524**
chrVI	49950	50105	155	0,14817715	0,037058696	0,799937774	0,841398987	0,715189139	0,620358902	0,543358902	**-0,716641098**
chrVI	55917	56077	160	0,162424953	0,166764131	0,493409293	-0,016521205	-0,014043024	-0,041934322	-0,153934322	**-1,413934322**
chrVI	60687	60850	163	1,538762715	1,593523919	0,49125859	-0,021913219	-0,018626236	-0,021413713	-0,154413713	**-1,414413713**
chrVI	65236	65388	152	0,43883233	0,233469783	0,65273085	0,392703751	0,333798188	0,223734835	0,167734835	**-1,092265165**
chrVI	65445	65606	161	0,353345512	0,218646305	0,617745747	0,299565626	0,254630782	0,240376832	0,121376832	**-1,138623168**
chrVI	68129	68276	147	0,250761331	0,233469783	0,517854644	0,044769906	0,03805442	-0,066365696	-0,087365696	**-1,347365696**
chrVI	68203	68367	164	0,119681545	0,103764348	0,535617563	0,08939893	0,07598909	0,141372297	0,001372297	**-1,258627703**
chrVI	71963	72118	155	0,202318801	0,140823044	0,589606905	0,226533908	0,192553821	0,096805439	0,019805439	**-1,240194561**
chrVI	72978	73128	150	0,230814407	0,17788174	0,564757972	0,163043664	0,138587115	0,010207619	-0,031792381	**-1,291792381**
chrVI	76566	76713	147	0,444531451	0,196411088	0,693558976	0,505963822	0,430069249	0,292919404	0,271919404	**-0,988080596**
chrVI	77258	77379	121	0,039893848	0,200116957	0,166216884	-0,969223341	-0,82383934	-0,399582826	-0,238582826	**-1,498582826**
chrVI	79934	80100	166	0,04844253	0,188999349	0,204018475	-0,827353109	-0,703250143	-0,55583285	-0,70983285	**-1,96983285**
chrVI	81318	81445	127	0,282106498	0,70040935	0,287126664	-0,56179848	-0,477528708	-0,093917645	0,025082355	**-1,234917645**
chrVI	82718	82885	167	0,267858695	0,311293045	0,462501753	-0,094133	-0,08001305	0,090036949	-0,070963051	**-1,330963051**
chrVI	84294	84451	157	0,176672756	0,192705218	0,478298027	-0,054425638	-0,046261792	-0,097113915	-0,188113915	**-1,448113915**
chrVI	91095	91248	153	0,655398934	0,589233263	0,526580411	0,066676582	0,056675095	-0,060248162	-0,123248162	**-1,383248162**
chrVI	94045	94152	107	0,153876272	0,507704132	0,23258892	-0,730347436	-0,62079532	-0,172735745	0,086264255	**-1,173735745**
chrVI	94173	94319	146	3,188658293	2,694167184	0,542028368	0,105545127	0,089713358	0,010993383	-0,003006617	**-1,263006617**
chrVI	94286	94435	149	0,088336378	0,062999783	0,583709654	0,211392905	0,179683969	0,054143965	0,019143965	**-1,240856035**
chrVI	97720	97865	145	0,079787696	0,062999783	0,558786364	0,147892925	0,125708986	0,072146015	0,065146015	**-1,194853985**
chrVI	97725	97874	149	0,034194727	0,118587827	0,223813035	-0,759378669	-0,645471868	-0,77690461	-0,81190461	**-2,07190461**
chrVI	100985	101138	153	2,14856868	1,797346746	0,544504493	0,111788617	0,095020325	-0,018002939	-0,081002939	**-1,341002939**
chrVI	108232	108382	150	0,324849907	0,196411088	0,623200105	0,313896315	0,266811868	0,130671474	0,088671474	**-1,171328526**
chrVI	108406	108531	125	2,065931423	5,684803934	0,266546505	-0,623291457	-0,529797739	-0,129091324	0,003908676	**-1,256091324**
chrVI	110912	111064	152	0,467327936	0,489174784	0,488579829	-0,028630034	-0,024335529	-0,133062316	-0,189062316	**-1,449062316**
chrVI	110912	111064	152	0,467327936	0,489174784	0,488579829	-0,028630034	-0,024335529	-0,133062316	-0,189062316	**-1,449062316**
chrVI	111983	112114	131	2,843861462	4,684219147	0,377767137	-0,311350375	-0,264647819	-0,006445116	0,084554884	**-1,175445116**
chrVI	115465	115634	169	0,054141651	0,044470435	0,549036667	0,123227859	0,104743681	0,298760557	0,123760557	**-1,136239443**
chrVI	123008	123146	138	0,24506221	0,203822827	0,545935351	0,115398462	0,098088692	0,17989455	0,22189455	**-1,03810545**
chrVI	130462	130613	151	0,772230918	0,533645219	0,591350815	0,231021151	0,196367979	0,06944386	0,02044386	**-1,23955614**
chrVI	131462	131615	153	0,208017923	0,155646522	0,572005115	0,181481351	0,154259148	0,044607726	-0,018392274	**-1,278392274**
chrVI	139986	140115	129	0,162424953	0,370586958	0,304730438	-0,510843172	-0,434216696	-0,149365929	-0,044365929	**-1,304365929**
chrVI	140063	140227	164	0,53856695	0,541056958	0,498846817	-0,002890606	-0,002457015	0,089193282	-0,050806718	**-1,310806718**

Chrom	Start	End	Length	Section A	Section B	A/A+B	Z-score	Z * 0.85	Phase correction	Length correction	ΔLKnuc
chrVI	141451	141594	143	0,202318801	0,114881957	0,637825718	0,352653046	0,299755089	0,267079225	0,274079225	**-0,985920775**
chrVI	142726	142892	166	0,059840772	0,066705652	0,472876041	-0,06804215	-0,057835827	0,112685298	-0,041314702	**-1,301314702**
chrVI	143530	143679	149	0,09688506	0,070411522	0,579121574	0,199646764	0,16969975	0,040860939	0,005860939	**-1,254139061**
chrVI	148326	148498	172	0,056991212	0,077823261	0,422738082	-0,194893703	-0,165659647	0,129779351	-0,066220649	**-1,326220649**
chrVI	151570	151741	171	0,695292782	1,278525005	0,352257836	-0,37923189	-0,322347107	-0,058539224	-0,247539224	**-1,507539224**
chrVI	151602	151755	153	0,866266417	0,663350655	0,566328942	0,167035499	0,141980174	0,03358165	-0,02941835	**-1,28941835**
chrVI	151830	151995	165	0,797876963	1,1451137	0,410643745	-0,225889337	-0,192005937	-0,066101052	-0,213101052	**-1,473101052**
chrVI	152009	152167	158	1,453275898	1,523112397	0,488268248	-0,029411381	-0,024999674	-0,071043515	-0,169043515	**-1,429043515**
chrVI	152552	152696	144	0,09973462	0,051882174	0,657807209	0,406485945	0,345513053	0,306475138	0,306475138	**-0,953524862**
chrVI	155423	155586	163	0,515770466	0,644821307	0,444403	-0,139815204	-0,118842923	-0,104475844	-0,237475844	**-1,497475844**
chrVI	162535	162694	159	0,347646391	0,274234349	0,559024213	0,148495706	0,12622135	0,074237703	-0,030762297	**-1,290762297**
chrVI	174443	174598	155	1,521665352	1,541641745	0,496739407	-0,008173185	-0,006947207	-0,097724964	-0,174724964	**-1,434724964**
chrVI	175285	175426	141	0,541416511	0,44099848	0,551107746	0,128460559	0,109191475	0,140399459	0,161399459	**-1,098600541**
chrVI	176188	176314	126	0,133929347	0,077823261	0,632480272	0,33842962	0,287665177	0,673308123	0,799308123	**-0,460691877**
chrVI	177968	178139	171	0,142478029	0,26682261	0,348101165	-0,390452017	-0,331884215	-0,053795237	-0,242795237	**-1,502795237**
chrVI	180563	180715	152	1,823718773	1,367465874	0,571486446	0,180159812	0,15313584	0,023323106	-0,032676894	**-1,292676894**
chrVI	182163	182304	141	4,399721541	2,920225228	0,601059226	0,256089736	0,217676275	0,222190028	0,243190028	**-1,016809972**
chrVI	183274	183405	131	0,088336378	1,000584786	0,081122841	-1,397558522	-1,187924744	-0,961995957	-0,870995957	**-2,130995957**
chrVI	183755	183882	127	1,065735658	1,041349352	0,50578674	0,014505715	0,012329858	0,387916479	0,506916479	**-0,753083521**
chrVI	185980	186131	151	1,940550757	1,511994788	0,562063768	0,156203695	0,13277314	0,00327801	-0,04572199	**-1,30572199**
chrVI	186486	186602	116	0,085486818	0,481763045	0,15070399	-1,033418742	-0,87840593	-0,436241078	-0,240241078	**-1,500241078**
chrVI	192368	192528	160	0,24506221	0,289057827	0,45881486	-0,103419898	-0,087906914	-0,107828325	-0,219828325	**-1,479828325**
chrVI	193031	193186	155	1,216762369	1,222936961	0,498734559	-0,003171996	-0,002696196	-0,085354477	-0,162354477	**-1,422354477**
chrVI	195001	195152	151	0,139628469	0,111176087	0,556722218	0,142663981	0,121264384	0,002543751	-0,046456249	**-1,306456249**
chrVI	197175	197337	162	0,447381012	0,492880654	0,475804798	-0,060685606	-0,051582765	-0,062677025	-0,188677025	**-1,448677025**
chrVI	197468	197595	127	0,772230918	1,990051964	0,279562576	-0,584141446	-0,496520229	-0,121299419	-0,002299419	**-1,262299419**
chrVI	198399	198562	163	2,692834751	1,752876311	0,605715197	0,268168519	0,227943241	0,250926202	0,117926202	**-1,142073798**
chrVI	202836	202999	163	0,934655871	0,959820221	0,493358494	-0,016648556	-0,014151273	0,036852612	-0,096147388	**-1,356147388**
chrVI	204046	204202	156	0,168124074	0,137117174	0,550790809	0,127659576	0,10851064	0,046465223	-0,037534777	**-1,297534777**
chrVI	208087	208238	151	2,661489585	1,271113265	0,676775583	0,458701085	0,389895922	0,272194055	0,223194055	**-1,036805945**
chrVI	208444	208571	127	0,105433742	0,255705001	0,291948022	-0,54770272	-0,465547312	-0,127102919	-0,008102919	**-1,268102919**
chrVI	208746	208898	152	5,662076879	4,120926971	0,578766703	0,198739409	0,168928498	0,043709333	-0,012290667	**-1,272290667**
chrVI	210302	210398	96	0,059840772	0,585527393	0,092723465	-1,324168984	-1,125543636	-0,641444235	-0,305444235	**-1,565444235**
chrVI	210302	210400	98	0,19661968	1,367465874	0,125709031	-1,14691183	-0,974875055	-0,476462921	-0,154462921	**-1,414462921**
chrVI	210302	210402	100	0,398938482	2,786813923	0,125225828	-1,149253041	-0,976865085	-0,467983737	-0,159983737	**-1,419983737**
chrVI	210302	210403	101	0,031345166	0,218646305	0,125384943	-1,148481403	-0,976209192	-0,477289577	-0,176289577	**-1,436289577**
chrVI	210302	210404	102	0,675345858	3,713281318	0,153885448	-1,019910529	-0,866923949	-0,377411677	-0,083411677	**-1,343411677**
chrVI	210302	210405	103	0,034194727	0,337234132	0,092062655	-1,328159833	-1,128935858	-0,644392982	-0,357392982	**-1,617392982**
chrVI	210302	210406	104	0,176672756	1,237760439	0,124907105	-1,150800763	-0,978180649	-0,48114542	-0,20114542	**-1,46114542**
chrVI	210302	210407	105	0,031345166	0,214940436	0,127271615	-1,13938351	-0,968475983	-0,5010736	-0,2280736	**-1,4880736**
chrVI	210302	210409	107	1,56440876	7,797149593	0,167109866	-0,965649231	-0,820801847	-0,366556663	-0,107556663	**-1,367556663**
chrVI	214215	214366	151	0,453080133	0,366881088	0,552562879	0,132139134	0,112318264	0,002655362	-0,046344638	**-1,306344638**
chrVI	214215	214366	151	0,453080133	0,366881088	0,552562879	0,132139134	0,112318264	0,002655362	-0,046344638	**-1,306344638**
chrVI	224528	224665	137	0,592708601	0,566998046	0,511084939	0,027789399	0,023620989	0,155804265	0,204804265	**-1,055195735**
chrVI	225246	225383	137	11,46093267	5,884920891	0,660730395	0,41445733	0,35228873	0,452000133	0,501000133	**-0,758999867**
chrVI	225640	225800	160	1,085682582	1,204407613	0,474078525	-0,065021289	-0,055268095	-0,06969213	-0,18169213	**-1,44169213**
chrVI	228483	228624	141	0,210867483	0,133411305	0,612490489	0,28581604	0,242943634	0,243905708	0,264905708	**-0,995094292**
chrVI	232870	233015	145	3,556251608	2,145698486	0,623690413	0,31518766	0,267909511	0,205397532	0,198397532	**-1,061602468**
chrVI	236135	236288	153	0,53571739	0,70040935	0,433383869	-0,167765533	-0,142600703	-0,246244981	-0,309244981	**-1,569244981**
chrVI	236706	236866	160	0,182371877	0,166764131	0,522351957	0,056057394	0,047648785	0,020579733	-0,091420267	**-1,351420267**
chrVI	237607	237756	149	0,592708601	0,374292827	0,612934566	0,286975777	0,243929411	0,110618469	0,075618469	**-1,184381531**
chrVI	237976	238116	140	0,886213341	0,763409133	0,537221913	0,093437281	0,079421689	0,141215564	0,169215564	**-1,090784436**
chrVI	239069	239219	150	0,629752889	0,537351089	0,539585933	0,099390615	0,084482023	-0,040599476	-0,082599476	**-1,342599476**
chrVI	242117	242275	158	0,359044634	0,211234566	0,629594476	0,330779497	0,281162572	0,226182572	0,128182572	**-1,131817428**
chrVI	242831	242985	154	0,179522317	0,163058261	0,524029464	0,060269401	0,051228991	-0,04019621	-0,11019621	**-1,37019621**
chrVI	242895	243045	150	0,04844253	0,059293913	0,449639217	-0,126572917	-0,10758698	-0,221153594	-0,263153594	**-1,523153594**
chrVI	244023	244165	142	0,678195419	0,559586306	0,547911966	0,120387658	0,10232951	0,085483579	0,099483579	**-1,160516421**
chrVI	244610	244760	150	1,233859733	0,870879351	0,586229306	0,217855901	0,185177516	0,073472902	0,031472902	**-1,228527098**
chrVI	247013	247149	136	0,504372223	0,285351958	0,638668836	0,35490291	0,301667474	0,498421504	0,554421504	**-0,705578496**
chrVI	258453	258604	151	0,059840772	0,096352609	0,38311977	-0,297297305	-0,252702709	-0,362543592	-0,411543592	**-1,671543592**
chrVI	260977	261105	128	0,498673102	0,70040935	0,415878909	-0,212447646	-0,180580499	0,136344029	0,248344029	**-1,011655971**
chrVI	264647	264774	127	2,043134938	3,231518273	0,387349624	-0,286233551	-0,243298518	0,067631273	0,186631273	**-1,073368727**
chrVI	265600	265745	145	0,14532759	0,081529131	0,640613994	0,360100456	0,306085387	0,232861507	0,225861507	**-1,034138493**
chrVI	267454	267607	153	0,125380666	0,155646522	0,446151373	-0,135390993	-0,115082344	-0,211590669	-0,274590669	**-1,534590669**
chrVI	269103	269252	149	0,094035499	0,077823261	0,547167331	0,118507771	0,100731606	-0,034072761	-0,069072761	**-1,329072761**
chrVI	270018	270158	140	0,48727486	0,511410002	0,487916538	-0,030293381	-0,025749374	0,031661252	0,059661252	**-1,200338748**
chrVII	6501	6604	103	0,122531105	0,674468263	0,15374053	-1,020521792	-0,867443523	-0,371732916	-0,084732916	**-1,344732916**
chrVII	7179	7332	153	0,111132863	0,137117174	0,44766504	-0,13156284	-0,111828414	-0,219523593	-0,282523593	**-1,542523593**
chrVII	7179	7332	153	0,111132863	0,137117174	0,44766504	-0,13156284	-0,111828414	-0,219523593	-0,282523593	**-1,542523593**
chrVII	8743	8898	155	8,232380526	6,659447633	0,552811947	0,132768958	0,112853614	0,049528237	-0,027471763	**-1,287471763**
chrVII	8747	8898	151	0,054141651	0,044470435	0,549036667	0,123227859	0,104743681	-0,016453083	-0,065453083	**-1,325453083**
chrVII	9683	9793	110	0,29350474	1,57870044	0,156769537	-1,007823765	-0,8566502	-0,174384333	0,063615667	**-1,196384333**
chrVII	15630	15761	131	9,825284892	9,331379599	0,512891213	0,032319105	0,027471239	0,226185837	0,317185837	**-0,942814163**
chrVII	15633	15761	128	0,045592969	0,085235	0,348495581	-0,389385276	-0,330977484	0,07335771	0,18535771	**-1,07464229**
chrVII	15791	15935	144	0,302053422	0,340940001	0,469761293	-0,075869923	-0,064489434	-0,239304451	-0,239304451	**-1,499304451**
chrVII	20349	20487	138	3,829809424	3,012871967	0,559694249	0,150194073	0,127664962	0,022793831	0,064793831	**-1,195206169**
chrVII	21325	21484	159	0,256460453	0,277940218	0,479902939	-0,050397187	-0,042837609	-0,030544131	-0,135544131	**-1,395544131**
chrVII	22410	22546	136	0,364743755	0,244587392	0,598596931	0,249717095	0,21225953	0,15362908	0,20962908	**-1,05037092**
chrVII	23209	23365	156	0,042743409	0,044470435	0,490098899	-0,024820929	-0,021097789	-0,082702114	-0,166702114	**-1,426702114**
chrVII	32814	32961	147	0,125380666	0,122293696	0,506231912	0,015621722	0,013278464	-0,175570695	-0,196570695	**-1,456570695**
chrVII	33222	33355	133	0,199469241	0,270528479	0,424404699	-0,190637769	-0,162042103	-0,123549984	-0,046549984	**-1,306549984**
chrVII	36941	37083	142	0,381841118	0,311293045	0,550890634	0,127911851	0,108725073	-0,013313104	0,000686896	**-1,259313104**
chrVII	41750	41885	135	0,182371877	0,35205761	0,341245911	-0,409065189	-0,34770541	-0,377749961	-0,314749961	**-1,574749961**
chrVII	52996	53149	153	4,473810116	3,023989576	0,596683067	0,24477085	0,208055222	0,094797651	0,031797651	**-1,228202349**
chrVII	53375	53486	111	0,131079787	0,941290873	0,122233656	-1,163893157	-0,989309184	-0,335857664	-0,104857664	**-1,364857664**
chrVII	62645	62823	178	0,04844253	0,188999349	0,204018475	-0,827353109	-0,703250143	-0,263732769	-0,501732769	**-1,761732769**
chrVII	69139	69303	164	0,564212996	0,559586306	0,502058503	0,005159925	0,004385937	0,069410458	-0,070589542	**-1,330589542**
chrVII	72210	72368	158	0,43883233	0,481763045	0,476683179	-0,058479919	-0,049707931	-0,046539502	-0,144539502	**-1,404539502**
chrVII	73049	73197	148	0,076938136	0,066705652	0,535617563	0,08939893	0,07598909	-0,105409235	-0,133409235	**-1,393409235**
chrVII	78222	78356	134	0,302053422	0,233469783	0,564034236	0,161205527	0,137024698	0,14029456	0,21029456	**-1,04970544**
chrVII	83125	83232	107	0,151026711	0,61888022	0,196162296	-0,85540931	-0,727097914	-0,092800065	0,166199935	**-1,093800065**
chrVII	83125	83247	122	0,185221438	0,715232829	0,205697774	-0,821440246	-0,698224209	-0,208671883	-0,054671883	**-1,314671883**
chrVII	84927	85059	132	0,498673102	0,511410002	0,493695123	-0,015804641	-0,013433945	0,105423188	0,189423188	**-1,070576812**
chrVII	88176	88368	192	0,136778908	0,196411088	0,41051325	-0,226224907	-0,192291171	0,270843567	-0,065156433	**-1,325156433**
chrVII	88303	88443	140	0,475876617	0,285351958	0,625142872	0,319016163	0,271163738	0,139169989	0,167169989	**-1,092830011**
chrVII	88402	88555	153	0,24506221	0,137117174	0,641222996	0,361729731	0,307470272	0,203403199	0,140403199	**-1,119596801**

Chrom	Start	End	Length	Section A	Section B	A/A+B	Z-score	Z * 0.85	Phase correction	Length correction	ΔLKnuc
chrVII	89045	89177	132	0,19377012	0,218646305	0,469840938	-0,07566971	-0,064319253	0,054802347	0,138802347	**-1,121197653**
chrVII	90038	90161	123	0,037044288	0,103764348	0,263082498	-0,633871027	-0,538790373	-0,062779921	0,084220079	**-1,175779921**
chrVII	91402	91556	154	0,208017923	0,133411305	0,609256343	0,277381333	0,235774133	0,173593639	0,103593639	**-1,156406361**
chrVII	92970	93138	168	2,843861462	3,212988925	0,469528101	-0,076456145	-0,064987724	0,161782507	-0,006217493	**-1,266217493**
chrVII	97437	97599	162	0,296354301	0,344645871	0,462331079	-0,094562726	-0,080378317	-0,041513424	-0,167513424	**-1,427513424**
chrVII	102150	102299	149	0,076938136	0,066705652	0,535617563	0,08939893	0,07598909	-0,110788269	-0,145788269	**-1,405788269**
chrVII	102520	102673	153	0,042743409	0,059293913	0,418899751	-0,204708983	-0,174002636	-0,280581125	-0,343581125	**-1,603581125**
chrVII	103288	103440	152	0,233663968	0,129705435	0,643048	0,366617972	0,311625276	0,179971599	0,123971599	**-1,136028401**
chrVII	103852	103984	132	0,581310359	0,678174133	0,461546262	-0,096538972	-0,082058126	0,026244755	0,110244755	**-1,149755245**
chrVII	108983	109147	164	0,757983115	0,889408699	0,460111012	-0,100154051	-0,085130943	-0,010889484	-0,150889484	**-1,410889484**
chrVII	116027	116173	146	0,376141997	0,207528696	0,644442151	0,370358087	0,314804374	0,153355084	0,139355084	**-1,120644916**
chrVII	119268	119414	146	0,396088921	0,237175653	0,625471465	0,319882942	0,271900501	0,10447816	0,09047816	**-1,16952184**
chrVII	125888	126050	162	0,78362916	0,837526525	0,48337687	-0,041680073	-0,035428062	0,001570111	-0,124429889	**-1,384429889**
chrVII	138978	139125	147	0,435982769	0,337234132	0,563855716	0,160752208	0,136639377	-0,047937718	-0,068937718	**-1,328937718**
chrVII	147769	147920	151	0,19377012	0,181587609	0,516227867	0,040688453	0,034585185	-0,086131641	-0,135131641	**-1,395131641**
chrVII	152057	152208	151	0,094035499	0,125999566	0,427365971	-0,183084213	-0,155621581	-0,282722759	-0,331722759	**-1,591722759**
chrVII	156139	156292	153	0,076938136	0,111176087	0,408996909	-0,230126057	-0,195607148	-0,301930912	-0,364930912	**-1,624930912**
chrVII	167824	167963	139	0,521469587	0,277940218	0,652318227	0,391586796	0,332848776	0,20671779	0,24171779	**-1,01828221**
chrVII	167824	167963	139	0,521469587	0,277940218	0,652318227	0,391586796	0,332848776	0,20671779	0,24171779	**-1,01828221**
chrVII	168448	168554	106	0,051292091	0,570703915	0,082463698	-1,38868882	-1,180385497	-0,602342667	-0,336342667	**-1,596342667**
chrVII	168635	168789	154	0,042743409	0,074117392	0,365763444	-0,34309514	-0,291630869	-0,356079628	-0,426079628	**-1,686079628**
chrVII	171388	171548	160	0,125380666	0,125999566	0,498768996	-0,003085674	-0,002622823	0,042768107	-0,069231893	**-1,329231893**
chrVII	174752	174849	97	1,393435125	7,063387417	0,164770529	-0,975038711	-0,828782904	-0,394810284	-0,065810284	**-1,325810284**
chrVII	174828	174980	152	0,273557816	0,163058261	0,626540868	0,322705529	0,274299699	0,139848201	0,083848201	**-1,176151799**
chrVII	178188	178320	132	0,051292091	0,044470435	0,535617563	0,08939893	0,07598909	0,171276535	0,255276535	**-1,004723465**
chrVII	183143	183279	136	0,757983115	0,926467395	0,449988356	-0,125690766	-0,106837151	-0,168240676	-0,112240676	**-1,372240676**
chrVII	184850	184994	144	0,678195419	0,329822392	0,672801027	0,447660898	0,380511763	0,208784636	0,208784636	**-1,051215364**
chrVII	196178	196334	156	2,140019998	1,812170224	0,541476973	0,104155366	0,088532061	0,041015225	-0,042984775	**-1,302984775**
chrVII	196178	196334	156	2,140019998	1,812170224	0,541476973	0,104155366	0,088532061	0,041015225	-0,042984775	**-1,302984775**
chrVII	204030	204183	153	0,48727486	0,337234132	0,590987927	0,230087027	0,195573973	0,089649878	0,026649878	**-1,233350122**
chrVII	204995	205129	134	1,339293474	0,974643699	0,578794226	0,198809775	0,168988309	0,169336543	0,239336543	**-1,020663457**
chrVII	209064	209201	137	0,353345512	0,337234132	0,511665114	0,029244273	0,024857632	-0,088980566	-0,039980566	**-1,299980566**
chrVII	214611	214774	163	0,661098055	0,752291524	0,467739443	-0,080953558	-0,068810524	-0,01160981	-0,14460981	**-1,40460981**
chrVII	217469	217614	145	0,236513528	0,148234783	0,61472272	0,291649583	0,247902146	0,122466618	0,115466618	**-1,144533382**
chrVII	221203	221378	175	0,501522663	0,503998263	0,498768996	-0,003085674	-0,002622823	0,378803359	0,161803359	**-1,098196641**
chrVII	223475	223638	163	0,843469933	0,874585221	0,490944619	-0,022700423	-0,01929536	0,048443371	-0,084556629	**-1,344556629**
chrVII	226620	226777	157	0,330549028	0,08894087	0,787978518	0,799426819	0,679512796	0,64924189	0,55824189	**-0,70175811**
chrVII	226621	226770	149	4,439615389	2,972107402	0,598999115	0,250757281	0,213143689	0,034999523	-4,77E-07	**-1,260000477**
chrVII	233772	233924	152	0,219416165	0,17788174	0,552271135	0,131401466	0,111691246	-0,025728485	-0,081728485	**-1,341728485**
chrVII	238049	238197	148	0,350495952	0,237175653	0,596414645	0,244077615	0,207465972	0,015443579	-0,012556421	**-1,272556421**
chrVII	243163	243328	165	0,73233707	0,61146848	0,5449725	0,112969166	0,096023791	0,201988332	0,054988332	**-1,205011668**
chrVII	249506	249649	143	0,034194727	0,070411522	0,326889907	-0,448517424	-0,381239811	-0,532763635	-0,525763635	**-1,785763635**
chrVII	251482	251633	151	0,09688506	0,044470435	0,685400026	0,482853237	0,410425251	0,28058505	0,23158505	**-1,02841495**
chrVII	266552	266693	141	0,113982423	0,074117392	0,605967759	0,268824834	0,228501109	0,101885783	0,122885783	**-1,137114217**
chrVII	267377	267528	151	1,478921943	0,889408699	0,624457547	0,317209152	0,269627779	0,137615653	0,088615653	**-1,171384347**
chrVII	268853	268959	106	0,068389454	0,159352392	0,300293755	-0,523555831	-0,445022456	0,151624118	0,417624118	**-0,842375882**
chrVII	268853	268977	124	0,071239015	0,214940436	0,248931272	-0,677856719	-0,576178211	-0,117224761	0,022775239	**-1,237224761**
chrVII	270004	270159	155	0,678195419	0,648527176	0,511181027	0,028030349	0,023825797	-0,03256371	-0,10956371	**-1,36956371**
chrVII	273536	273656	120	0,076938136	0,251999131	0,233899115	-0,726066131	-0,617156212	-0,029368652	0,138631348	**-1,121368652**
chrVII	275567	275723	156	1,718285032	1,371171744	0,556177075	0,141283669	0,120091118	0,058662709	-0,025337291	**-1,285337291**
chrVII	275567	275723	156	1,718285032	1,371171744	0,556177075	0,141283669	0,120091118	0,058662709	-0,025337291	**-1,285337291**
chrVII	279938	280099	161	1,182567642	0,763409133	0,607698744	0,273326155	0,232327231	0,253661488	0,134661488	**-1,125338512**
chrVII	281760	281908	148	13,45277551	8,697675901	0,607336404	0,272383457	0,231525938	0,034874121	0,006874121	**-1,253125879**
chrVII	281764	281908	144	0,082637257	0,077823261	0,515000562	0,037609696	0,031968242	-0,128605836	-0,128605836	**-1,388605836**
chrVII	284331	284485	154	0,44168189	0,237175653	0,650625296	0,387009186	0,328957808	0,267497128	0,197497128	**-1,062502872**
chrVII	289603	289760	157	0,071239015	0,085235	0,455276965	-0,11233987	-0,09548889	-0,111679938	-0,202679938	**-1,462679938**
chrVII	293723	293871	148	1,031540931	0,433586741	0,704062145	0,536119867	0,455701887	0,246833074	0,218833074	**-1,041166926**
chrVII	294019	294183	164	0,068389454	0,163058261	0,295485544	-0,537429332	-0,456814932	-0,380793194	-0,520793194	**-1,780793194**
chrVII	297177	297313	136	1,849364819	1,460112614	0,558808711	0,147949557	0,125757123	0,05325725	0,10925725	**-1,15074275**
chrVII	299926	300073	147	0,265009134	0,151940653	0,635590046	0,346695744	0,294691382	0,110323283	0,089323283	**-1,170676717**
chrVII	300091	300244	153	0,527168708	0,340940001	0,607261167	0,272187743	0,231359582	0,132294679	0,069294679	**-1,190705321**
chrVII	300595	300755	160	0,076938136	0,096352609	0,443982948	-0,140878539	-0,119746758	-0,079002598	-0,191002598	**-1,451002598**
chrVII	313487	313646	159	0,202318801	0,26311674	0,434687048	-0,164453569	-0,139785534	-0,122474576	-0,227474576	**-1,487474576**
chrVII	314575	314719	144	0,142478029	0,144528914	0,496427117	-0,008956009	-0,007612608	-0,152057745	-0,152057745	**-1,412057745**
chrVII	320068	320221	153	0,720938828	0,452116089	0,614582333	0,291282418	0,247590055	0,163719947	0,100719947	**-1,159280053**
chrVII	320068	320221	153	0,720938828	0,452116089	0,614582333	0,291282418	0,247590055	0,163719947	0,100719947	**-1,159280053**
chrVII	323178	323326	148	0,416035845	0,411351523	0,502830791	0,007095801	0,006031431	-0,202382885	-0,230382885	**-1,490382885**
chrVII	324915	325051	136	0,284956058	0,359469349	0,442186256	-0,145428565	-0,123614281	-0,207869503	-0,151869503	**-1,411869503**
chrVII	327547	327717	170	0,199469241	0,248293262	0,445480003	-0,137089559	-0,116526125	0,194020159	0,012020159	**-1,247979841**
chrVII	327547	327717	170	0,199469241	0,248293262	0,445480003	-0,137089559	-0,116526125	0,194020159	0,012020159	**-1,247979841**
chrVII	328882	329034	152	0,131079787	0,129705435	0,502635026	0,006605078	0,005614317	-0,131105949	-0,187105949	**-1,447105949**
chrVII	329271	329388	117	0,028495606	0,400233914	0,066465229	-1,502645598	-1,277248758	-0,707571749	-0,518571749	**-1,778571749**
chrVII	330372	330517	145	0,393239361	0,259410871	0,602526961	0,259893303	0,220909308	0,099839043	0,092839043	**-1,167160957**
chrVII	331632	331780	148	1,048638295	0,678174133	0,607268212	0,272206067	0,231375157	0,012582381	-0,015417619	**-1,275417619**
chrVII	331632	331780	148	1,048638295	0,678174133	0,607268212	0,272206067	0,231375157	0,012582381	-0,015417619	**-1,275417619**
chrVII	341547	341699	152	0,208017923	0,237175653	0,46725275	-0,082177582	-0,069850944	-0,212194936	-0,268194936	**-1,528194936**
chrVII	342190	342339	149	0,427434088	0,289057827	0,596565123	0,24446623	0,207796296	0,029450745	-0,005549255	**-1,265549255**
chrVII	342310	342453	143	2,111524392	1,208113483	0,636070702	0,347975482	0,29577916	0,142117775	0,149117775	**-1,110882225**
chrVII	343265	343403	138	2,350887481	1,745464572	0,573897812	0,186306545	0,158360563	0,048613243	0,090613243	**-1,169386757**
chrVII	352278	352440	162	0,29350474	0,363175219	0,446952486	-0,133364686	-0,113359983	-0,080163356	-0,206163356	**-1,466163356**
chrVII	354451	354624	173	0,079787696	0,151940653	0,344315647	-0,400713197	-0,340606218	0,001447473	-0,201552527	**-1,461552527**
chrVII	356531	356676	145	0,04844253	0,037058696	0,566571175	0,16765125	0,142503563	0,004535019	-0,002464981	**-1,262464981**
chrVII	364639	364801	162	0,578460798	0,637409568	0,475758613	-0,060801588	-0,05168135	-0,018407366	-0,144407366	**-1,404407366**
chrVII	367967	368120	153	0,133929347	0,062999783	0,680089062	0,46794786	0,397755681	0,311627336	0,248627336	**-1,011372664**
chrVII	370607	370760	153	0,267858695	0,311293045	0,462501753	-0,094133	-0,08001305	-0,168822377	-0,231822377	**-1,491822377**
chrVII	382677	382828	151	0,216566604	0,211234566	0,506231912	0,015621722	0,013278464	-0,119412347	-0,168412347	**-1,428412347**
chrVII	382677	382828	151	0,216566604	0,211234566	0,506231912	0,015621722	0,013278464	-0,119412347	-0,168412347	**-1,428412347**
chrVII	383386	383517	131	0,122531105	0,100058479	0,55047996	0,126874057	0,107842948	0,294874491	0,385857491	**-0,874142509**
chrVII	383588	383739	151	0,185221438	0,051882174	0,781183536	0,776196573	0,659767087	0,51947176	0,47047176	**-0,78952824**
chrVII	384835	384978	143	0,088336378	0,196411088	0,310227091	-0,495206755	-0,420925742	-0,574958092	-0,567958092	**-1,827958092**
chrVII	384841	384978	137	0,076938136	0,107470218	0,417216109	-0,209020522	-0,177667444	-0,290809551	-0,241809551	**-1,501809551**
chrVII	386231	386378	147	3,718676561	1,804758485	0,67325433	0,448917266	0,381579676	0,198113304	0,177113304	**-1,082886696**
chrVII	386588	386752	164	0,387540239	0,589233263	0,39675548	-0,26175419	-0,222491061	-0,134194436	-0,274194436	**-1,534194436**
chrVII	398118	398266	148	5,462607639	2,860931315	0,656284264	0,402343192	0,341991714	0,12232298	0,09432298	**-1,16567702**
chrVII	398740	398893	153	0,24221265	0,188999349	0,561702018	0,155285859	0,13199298	0,040841734	-0,022158266	**-1,282158266**
chrVII	399113	399273	160	2,407878693	2,234639356	0,518657907	0,046785499	0,039767674	0,07442469	-0,03757531	**-1,29757531**

Chrom	Start	End	Length	Section A	Section B	A/A+B	Z-score	Z * 0.85	Phase correction	Length correction	ΔLKnuc
chrVII	402184	402330	146	0,088336378	0,081529131	0,520037168	0,050246866	0,042709836	-0,128219053	-0,142219053	**-1,402219053**
chrVII	406230	406375	145	0,071239015	0,037058696	0,657807209	0,406485945	0,345513053	0,205399496	0,198399496	**-1,061600504**
chrVII	412759	412909	150	0,843469933	0,741173916	0,53227729	0,080995639	0,068846293	-0,071005527	-0,113005527	**-1,373005527**
chrVII	413027	413197	170	0,034194727	0,155646522	0,180122745	-0,914897409	-0,777662797	-0,438692813	-0,620692813	**-1,880692813**
chrVII	416964	417114	150	7,659618848	6,900329155	0,526074602	0,065405938	0,055595047	-0,083567264	-0,125567264	**-1,385567264**
chrVII	419105	419256	151	0,210867483	0,218646305	0,490944619	-0,022700423	-0,01929536	-0,162173429	-0,211173429	**-1,471173429**
chrVII	422885	423015	130	0,481575739	0,748585655	0,391473624	-0,275480315	-0,234158267	-0,000825096	0,097174904	**-1,162825096**
chrVII	424432	424587	155	0,091185939	0,103764348	0,467739443	-0,080953558	-0,068810524	-0,122672603	-0,199672603	**-1,459672603**
chrVII	426688	426828	140	0,547115632	0,44099848	0,55369681	0,13500695	0,114755908	-0,009124711	0,018875289	**-1,241124711**
chrVII	430629	430785	156	0,202318801	0,192705218	0,512168353	0,030506268	0,025930328	-0,026031438	-0,110031438	**-1,370031438**
chrVII	438337	438504	167	3,1231184	2,98693088	0,511144552	0,027938883	0,02374805	0,195631873	0,034631873	**-1,225368127**
chrVII	445398	445550	152	0,14532759	0,114881957	0,558502144	0,147172696	0,125096791	-0,01424666	-0,07024666	**-1,33024666**
chrVII	445405	445562	157	0,082637257	0,048176305	0,631717813	0,336406466	0,285945496	0,284646424	0,193646424	**-1,066353576**
chrVII	448295	448446	151	0,889062902	0,496586524	0,641621817	0,362797223	0,30837764	0,16622849	0,11722849	**-1,14277151**
chrVII	449063	449214	151	7,762203029	6,633506546	0,53920253	0,098424855	0,083661127	-0,063267789	-0,112267789	**-1,372267789**
chrVII	449866	450017	151	3,530605563	2,857225445	0,552708041	0,132506202	0,112630272	-0,040950154	-0,089950154	**-1,349950154**
chrVII	450289	450475	186	0,059840772	0,059293913	0,502295129	0,005753068	0,004890107	0,435588056	0,141588056	**-1,118411944**
chrVII	450564	450727	163	0,176672756	0,188999349	0,483145293	-0,042261061	-0,035921902	0,022609663	-0,110390337	**-1,370390337**
chrVII	451657	451811	154	1,362089959	0,952408482	0,588503295	0,223696577	0,19014209	0,133365889	0,063365889	**-1,196634111**
chrVII	452015	452160	145	0,131079787	0,140823044	0,4820832	-0,044925865	-0,038186985	-0,191813534	-0,198813534	**-1,458813534**
chrVII	454120	454220	100	0,074088575	0,637409568	0,104130385	-1,258362274	-1,069607933	-0,611903427	-0,303903427	**-1,563903427**
chrVII	457180	457330	150	0,119681545	0,144528914	0,452978074	-0,118140734	-0,100419624	-0,237786381	-0,279786381	**-1,539786381**
chrVII	457820	457954	134	0,367593315	0,374292827	0,495484811	-0,011318142	-0,009620421	-0,007197554	0,062802446	**-1,197197554**
chrVII	458030	458181	151	0,966001038	0,574409785	0,627106109	0,324198469	0,275568699	0,120546827	0,071546827	**-1,188453173**
chrVII	464168	464326	158	0,116831984	0,17417587	0,401473645	-0,249534587	-0,212104399	-0,205938962	-0,303938962	**-1,563938962**
chrVII	468550	468701	151	0,410336724	0,296469566	0,58055047	0,203301913	0,172806626	0,011497835	-0,037502165	**-1,297502165**
chrVII	470833	470993	160	0,122531105	0,111176087	0,524293257	0,060931847	0,05179207	0,082832195	-0,029167805	**-1,289167805**
chrVII	471018	471094	76	0,119681545	0,040764565	0,745929861	0,661736237	0,562475801		0,476	**-0,784**
chrVII	471018	471123	105	0,108283302	0,085235	0,559550703	0,149830185	0,127355657	0,646562227	0,919562227	**-0,340437773**
chrVII	471173	471322	149	0,239363089	0,125999566	0,655138356	0,399230614	0,339346022	0,155064634	0,120064634	**-1,139935366**
chrVII	475774	475931	157	0,188070999	0,200116957	0,484484373	-0,038901718	-0,03306646	-0,022988807	-0,113988807	**-1,373988807**
chrVII	476346	476454	108	0,088336378	0,581821524	0,131814275	-1,117855882	-0,950177499	-0,297185286	-0,045185286	**-1,305185286**
chrVII	476346	476465	119	0,071239015	0,741173916	0,087688184	-1,35512929	-1,151859897	-0,535063938	-0,360063938	**-1,620063938**
chrVII	478282	478437	155	0,475876617	0,185293479	0,719749154	0,582096486	0,494782013	0,439899592	0,362899592	**-0,897100408**
chrVII	482579	482701	122	0,122531105	0,400233914	0,234390406	-0,724464178	-0,615794551	-0,135825791	0,018174209	**-1,241825791**
chrVII	483653	483754	101	0,056991212	0,107470218	0,346532387	-0,394699362	-0,335494458	0,135109524	0,436109524	**-0,823890476**
chrVII	495041	495196	155	0,156725832	0,081529131	0,657807209	0,406485945	0,345513053	0,278376586	0,201376586	**-1,058623414**
chrVII	501090	501258	168	0,421734966	0,781938481	0,350373241	-0,38431299	-0,326666042	-0,071675317	-0,239675317	**-1,499675317**
chrVII	502391	502522	131	0,837770812	1,259995657	0,399363239	-0,254995625	-0,216746281	-0,040325931	0,050674069	**-1,209325931**
chrVII	502870	503027	157	0,430283648	0,489174784	0,467975096	-0,08036094	-0,068306799	-0,055405847	-0,146405847	**-1,406405847**
chrVII	504629	504778	149	1,079983461	0,88199696	0,550455779	0,126812955	0,107791012	-0,066011428	-0,101011428	**-1,361011428**
chrVII	505104	505255	151	0,407487163	0,377998697	0,518770845	0,047068905	0,040008569	-0,115476987	-0,164476987	**-1,424476987**
chrVII	509103	509251	148	0,376141997	0,229763914	0,620792751	0,307563498	0,261428973	0,043608396	0,015608396	**-1,244391604**
chrVII	509261	509410	149	0,116831984	0,129705435	0,473891486	-0,065491126	-0,055667457	-0,228709292	-0,263709292	**-1,523709292**
chrVII	510438	510590	152	0,119681545	0,111176087	0,518421434	0,046192108	0,039263292	-0,110014425	-0,166014425	**-1,426014425**
chrVII	512325	512500	175	0,83207169	1,293348483	0,39148574	-0,275448768	-0,234131453	0,193783244	-0,023216756	**-1,283216756**
chrVII	515194	515319	125	0,541416511	1,026525873	0,34530383	-0,398030558	-0,338325975	0,09761383	0,23061383	**-1,02938617**
chrVII	516112	516262	150	0,581310359	0,596645002	0,493490991	-0,016316389	-0,013868931	-0,157946635	-0,199946635	**-1,459946635**
chrVII	516112	516262	150	0,581310359	0,596645002	0,493490991	-0,016316389	-0,013868931	-0,157946635	-0,199946635	**-1,459946635**
chrVII	517734	517882	148	10,99930385	7,004093504	0,610957123	0,281814497	0,239542323	0,022014422	-0,005985578	**-1,265985578**
chrVII	517736	517882	146	0,028495606	0,051882174	0,354520937	-0,373143192	-0,317171714	-0,499840711	-0,513840711	**-1,773840711**
chrVII	518337	518484	147	2,895153553	2,312462617	0,555946033	0,140698749	0,119593937	-0,06490264	-0,08590264	**-1,34590264**
chrVII	519407	519563	156	0,364743755	0,296469566	0,551627959	0,129775456	0,110309137	0,068403533	-0,015596467	**-1,275596467**
chrVII	519674	519834	160	3,596145456	3,231518273	0,526702193	0,066982525	0,056935146	0,087109296	-0,024890704	**-1,284890704**
chrVII	519935	520102	167	0,524319147	0,600350872	0,466198208	-0,08483016	-0,072105636	0,09191371	-0,06908629	**-1,32908629**
chrVII	520743	520902	159	0,133929347	0,111176087	0,54641525	0,116609512	0,099118085	0,112786991	0,007786991	**-1,252213009**
chrVII	520956	521107	151	0,116831984	0,192705218	0,377440848	-0,312208996	-0,265377647	-0,413404401	-0,462404401	**-1,722404401**
chrVII	526220	526384	164	0,119681545	0,255705001	0,318822147	-0,470995015	-0,400345763	-0,308989585	-0,448989585	**-1,708989585**
chrVII	529947	530056	109	0,170973635	0,17417587	0,49536109	-0,011628286	-0,009884043	0,665803302	0,910803302	**-0,349196698**
chrVII	535422	535536	114	0,085486818	0,570703915	0,130277392	-1,125080828	-0,956318704	-0,339281652	-0,129281652	**-1,389281652**
chrVII	536123	536272	149	0,062690333	0,040764565	0,605967759	0,268824834	0,228501109	0,055229877	0,020229877	**-1,239770123**
chrVII	536273	536379	106	0,840620372	2,164227834	0,279754688	-0,583570406	-0,496034845	0,101769946	0,367769946	**-0,892230054**
chrVII	536273	536391	118	0,837770812	2,479226748	0,252569017	-0,666427276	-0,566463185	0,014833424	0,196833424	**-1,063166576**
chrVII	536297	536420	123	0,076938136	0,155646522	0,330796263	-0,437715508	-0,372058182	0,124498511	0,271498511	**-0,988501489**
chrVII	536397	536552	155	0,125380666	0,08894087	0,585011979	0,214732295	0,182522451	0,110216743	0,033216743	**-1,226783257**
chrVII	536830	536964	134	0,159575393	0,155646522	0,506231912	0,015621722	0,013278464	0,014724359	0,084724359	**-1,175275641**
chrVII	536830	536970	140	0,136778908	0,100058479	0,577522451	0,195559327	0,166225428	0,052443065	0,080443065	**-1,179556935**
chrVII	537026	537177	151	0,151026711	0,085235	0,639234814	0,356414232	0,302952097	0,155744882	0,106744882	**-1,153255118**
chrVII	537036	537181	145	0,116831984	0,062999783	0,649673781	0,384439894	0,326773909	0,151391116	0,144391116	**-1,115608884**
chrVII	537308	537462	154	0,230814407	0,170470001	0,575189075	0,18960094	0,161160799	0,099386273	0,029386273	**-1,230613727**
chrVII	537342	537486	144	0,062690333	0,037058696	0,628480635	0,327831948	0,278657156	0,145485296	0,145485296	**-1,114514704**
chrVII	537873	538008	135	0,079787696	0,074117392	0,518421434	0,046192108	0,039263292	-0,003992722	0,059007278	**-1,200992722**
chrVII	538055	538187	132	0,159575393	0,211234566	0,430342791	-0,175501512	-0,149176285	-0,063847503	0,020152497	**-1,239847503**
chrVII	538144	538282	138	0,561363435	0,52623348	0,516150264	0,040493772	0,034419707	-0,05356563	-0,01156563	**-1,27156563**
chrVII	538499	538656	157	0,045592969	0,059293913	0,434687048	-0,164453569	-0,139785534	-0,114812911	-0,205812911	**-1,465812911**
chrVII	538599	538743	144	0,037044288	0,037058696	0,499902783	-0,000243688	-0,000207135	-0,13422548	-0,13422548	**-1,39422548**
chrVII	538990	539119	129	0,045592969	0,100058479	0,313027917	-0,487285766	-0,414192901	-0,078675531	0,026324469	**-1,233675531**
chrVII	539470	539601	131	0,065539893	0,155646522	0,296310663	-0,535041262	-0,454785073	-0,285414983	-0,194414983	**-1,454414983**
chrVII	539902	540052	150	0,125380666	0,192705218	0,394172367	-0,268460684	-0,228191582	-0,369066088	-0,411066088	**-1,671066088**
chrVII	540853	541004	151	0,085486818	0,059293913	0,590457149	0,22872109	0,194412926	0,049038511	3,8511E-05	**-1,259961489**
chrVII	542644	542795	151	0,689593661	0,541056958	0,560348851	0,151853737	0,129075677	-0,007033793	-0,056033793	**-1,316033793**
chrVII	544012	544140	128	0,304902982	0,492880654	0,382187561	-0,299740475	-0,254779404	0,132788472	0,244788472	**-1,015211528**
chrVII	545414	545580	166	0,128230226	0,066705652	0,657807209	0,406485945	0,345513053	0,467670817	0,313670817	**-0,946329183**
chrVII	546693	546840	147	0,378991558	0,077823261	0,829639368	0,9527411	0,809829935	0,622368144	0,601368144	**-0,658631856**
chrVII	548258	548409	151	2,481967268	1,637994354	0,602424851	0,259628569	0,220684284	0,079659318	0,030659318	**-1,229340682**
chrVII	554460	554622	162	0,142478029	0,137117174	0,509586815	0,024032894	0,02042796	0,060820549	-0,065179451	**-1,325179451**
chrVII	554946	555089	143	0,3903898	0,43729261	0,471666179	-0,07108217	-0,060419845	-0,219131007	-0,212131007	**-1,472131007**
chrVII	562268	562415	147	0,062690333	0,044470435	0,585011979	0,214732295	0,182522451	-0,007448153	-0,028448153	**-1,288448153**
chrVII	562775	562889	114	0,051292091	0,377998697	0,119480995	-1,177585257	-1,000947468	-0,375104861	-0,165104861	**-1,425104861**
chrVII	563471	563621	150	0,515770466	0,407645654	0,558546093	0,147284059	0,12519145	-0,014115291	-0,056115291	**-1,316115291**
chrVII	564404	564533	129	0,054141651	0,066705652	0,448017039	-0,130672891	-0,111071958	0,211288898	0,316288898	**-0,943711102**
chrVII	564867	565024	157	0,065539893	0,066705652	0,495592445	-0,011048326	-0,009391077	0,00943547	-0,08156453	**-1,34156453**
chrVII	565335	565496	161	0,071239015	0,070411522	0,502920895	0,007321663	0,006223413	0,022145412	-0,096854588	**-1,356854588**
chrVII	565515	565650	135	0,085486818	0,066705652	0,561702018	0,155285859	0,13199298	0,095212873	0,158212873	**-1,101787127**
chrVII	566553	566693	140	0,165274514	0,129705435	0,560290672	0,151706213	0,128950281	0,022086642	0,050086642	**-1,209913358**
chrVII	566559	566693	134	0,14817715	0,074117392	0,666580246	0,43048963	0,365916185	0,364031075	0,434031075	**-0,825968925**

Chrom	Start	End	Length	Section A	Section B	A/A+B	Z-score	Z * 0.85	Phase correction	Length correction	ΔLKnuc
chrVII	566971	567126	155	0,113982423	0,092646739	0,551627959	0,129775456	0,110309137	0,029917646	-0,047082354	**-1,307082354**
chrVII	567102	567210	108	0,062690333	0,192705218	0,245463684	-0,688834586	-0,585509398	0,082988296	0,334988296	**-0,925011704**
chrVII	568106	568272	166	0,14532759	0,151940653	0,488876943	-0,027884981	-0,023702234	0,096483813	-0,057516187	**-1,317516187**
chrVII	568932	569081	149	0,094035499	0,066705652	0,585011979	0,214732295	0,182522451	0,018534006	-0,016465994	**-1,276465994**
chrVII	569719	569885	166	0,031345166	0,107470218	0,225804702	-0,752734617	-0,639824425	-0,509964984	-0,663964984	**-1,923964984**
chrVII	571017	571160	143	0,185221438	0,103764348	0,640936153	0,360962213	0,306817881	0,149921887	0,156921887	**-1,103078113**
chrVII	572385	572549	164	0,527168708	0,496586524	0,514936277	0,037448446	0,03183118	0,118336157	-0,021663843	**-1,281663843**
chrVII	572497	572626	129	0,19377012	0,366881088	0,345616164	-0,397183258	-0,337605769	-0,019394484	0,085605516	**-1,174394484**
chrVII	573872	573982	110	0,116831984	0,818997177	0,124843282	-1,151111022	-0,978444369	-0,320307143	-0,082307143	**-1,342307143**
chrVII	575134	575270	136	0,461628815	0,463233697	0,499132367	-0,002174836	-0,00184861	-0,094923694	-0,038923694	**-1,298923694**
chrVII	575584	575763	179	0,094035499	0,192705218	0,327946098	-0,445591717	-0,378752959	0,063727262	-0,181272738	**-1,441272738**
chrVII	575584	575763	179	0,094035499	0,192705218	0,327946098	-0,445591717	-0,378752959	0,063727262	-0,181272738	**-1,441272738**
chrVII	578432	578606	174	0,039893848	0,100058479	0,285053125	-0,567895024	-0,48271077	-0,100721487	-0,310721487	**-1,570721487**
chrVII	578432	578606	174	0,039893848	0,100058479	0,285053125	-0,567895024	-0,48271077	-0,100721487	-0,310721487	**-1,570721487**
chrVII	578979	579129	150	1,524514912	0,989467177	0,606414389	0,269985742	0,229487881	0,079015555	0,037015555	**-1,222984445**
chrVII	579144	579294	150	0,216566604	0,222352175	0,493409293	-0,016521205	-0,014043024	-0,158838059	-0,200838059	**-1,460838059**
chrVII	583828	583997	169	0,102584181	0,092646739	0,525450481	0,063838228	0,054262494	0,345712178	0,170712178	**-1,089287822**
chrVII	584107	584258	151	0,256460453	0,181587609	0,585461904	0,215886537	0,183503557	0,037173223	-0,011826777	**-1,271826777**
chrVII	584200	584336	136	0,125380666	0,037058696	0,771861355	0,744990787	0,633242169	0,529479713	0,585479713	**-0,674520287**
chrVII	584954	585099	145	0,139628469	0,155646522	0,472876041	-0,06804215	-0,057835827	-0,230111235	-0,237111235	**-1,497111235**
chrVII	594298	594445	147	0,903310705	0,403939784	0,691000472	0,498688204	0,423884973	0,244178703	0,223178703	**-1,036821297**
chrVII	595048	595213	165	0,028495606	0,055588044	0,338895921	-0,415478239	-0,353156503	-0,241510807	-0,388510807	**-1,648510807**
chrVII	595048	595213	165	0,028495606	0,055588044	0,338895921	-0,415478239	-0,353156503	-0,241510807	-0,388510807	**-1,648510807**
chrVII	595281	595432	151	0,230814407	0,155646522	0,597251597	0,246239543	0,209303612	0,059859581	0,010859581	**-1,249140419**
chrVII	610980	611160	180	0,324849907	0,452116089	0,418100546	-0,206755141	-0,17574187	0,257034573	0,005034573	**-1,254965427**
chrVII	610980	611160	180	0,324849907	0,452116089	0,418100546	-0,206755141	-0,17574187	0,257034573	0,005034573	**-1,254965427**
chrVII	613266	613414	148	3,804163379	2,686755444	0,586074712	0,217459102	0,184840237	-0,038462944	-0,066462944	**-1,326462944**
chrVII	637713	637869	156	0,213717044	0,185293479	0,535617563	0,08939893	0,07598909	0,046151127	-0,037848873	**-1,297848873**
chrVII	640000	640148	148	12,27305743	8,686558292	0,585557369	0,216131479	0,183711757	-0,039519137	-0,067519137	**-1,327519137**
chrVII	640002	640148	146	0,054141651	0,085235	0,388455674	-0,283346352	-0,2408444	-0,41590033	-0,42990033	**-1,68990033**
chrVII	640719	640865	146	0,735186631	1,200701743	0,379767057	-0,306092637	-0,260178741	-0,439622115	-0,453622115	**-1,713622115**
chrVII	652407	652570	163	0,076938136	0,114881957	0,40109529	-0,250513093	-0,212936129	-0,145933903	-0,278933903	**-1,538933903**
chrVII	652780	652925	145	2,288197149	1,245172178	0,64759637	0,37883922	0,322013337	0,150137435	0,143137435	**-1,116862565**
chrVII	654255	654401	146	0,63260245	0,433586741	0,593330391	0,236120435	0,20070237	0,015280533	0,001280533	**-1,258719467**
chrVII	654255	654401	146	0,63260245	0,433586741	0,593330391	0,236120435	0,20070237	0,015280533	0,001280533	**-1,258719467**
chrVII	668831	668991	160	0,202318801	0,26311674	0,434687048	-0,164453569	-0,139785534	-0,113326114	-0,225326114	**-1,485326114**
chrVII	671142	671294	152	0,04844253	0,055588044	0,465656665	-0,08619258	-0,073263693	-0,21970138	-0,27570138	**-1,53570138**
chrVII	671676	671849	173	0,14532759	0,137117174	0,51453455	0,036440779	0,030974662	0,399705709	0,196705709	**-1,063294291**
chrVII	674557	674713	156	1,065735658	1,033937612	0,507572141	0,018981684	0,016134431	-0,010891259	-0,094891259	**-1,354891259**
chrVII	676654	676817	163	0,24506221	0,144528914	0,629024111	0,329269787	0,279879319	0,342444717	0,209444717	**-1,050555283**
chrVII	680899	681051	152	0,803576085	0,444704349	0,643746439	0,368491045	0,313217388	0,179196486	0,123196486	**-1,136803514**
chrVII	681312	681478	166	0,039893848	0,074117392	0,349911537	-0,385559308	-0,327725412	-0,185713766	-0,339713766	**-1,599713766**
chrVII	682534	682696	162	0,43883233	0,511410002	0,461810967	-0,095872377	-0,08149152	-0,048187556	-0,174187556	**-1,434187556**
chrVII	687608	687758	150	0,14532759	0,133411305	0,521375354	0,053605728	0,045564869	-0,098455956	-0,140455956	**-1,400455956**
chrVII	689536	689689	153	0,074088575	0,051882174	0,588141101	0,222765786	0,189350918	0,103047355	0,040047355	**-1,219952645**
chrVII	692091	692197	106	0,102584181	0,807879568	0,11267245	-1,212437641	-1,030571995	-0,417156342	-0,151156342	**-1,411156342**
chrVII	692091	692218	127	0,039893848	0,055588044	0,417815854	-0,20748423	-0,176361595	0,244765772	0,363765772	**-0,896234228**
chrVII	692091	692219	128	0,051292091	0,08894087	0,365763444	-0,34309514	-0,291630869	0,072034507	0,184034507	**-1,075965493**
chrVII	692091	692220	129	0,031345166	0,059293913	0,345823971	-0,396619676	-0,337126724	-0,036786782	0,068213218	**-1,191786782**
chrVII	692091	692221	130	0,028495606	0,085235	0,250553539	-0,67274886	-0,571836531	-0,371516088	-0,273516088	**-1,533516088**
chrVII	692091	692222	131	0,056991212	0,111176087	0,338895921	-0,415478239	-0,353156503	-0,189763356	-0,098763356	**-1,358763356**
chrVII	692091	692223	132	25,52351415	23,00603834	0,525937554	0,065061679	0,055302427	0,124318559	0,208318559	**-1,051681441**
chrVII	692092	692223	131	0,039893848	0,051882174	0,434687048	-0,164453569	-0,139785534	0,007618017	0,098618017	**-1,161381983**
chrVII	692095	692223	128	0,04844253	0,074117392	0,395255882	-0,265646117	-0,2257992	0,13385168	0,24585168	**-1,01414832**
chrVII	692097	692197	100	0,04844253	0,059293913	0,449639217	-0,126572917	-0,10758698	0,346591594	0,654591594	**-0,605408406**
chrVII	693772	693921	149	0,270708255	0,085235	0,760537673	0,708033178	0,601828201	0,44326871	0,40826871	**-0,85173129**
chrVII	697506	697652	146	0,24221265	0,170470001	0,586922298	0,219635036	0,18668978	-0,003158175	-0,017158175	**-1,277158175**
chrVII	697827	697977	150	0,752283994	0,500292393	0,600589315	0,254872767	0,216641852	0,073224274	0,031224274	**-1,228775726**
chrVII	701841	701992	151	0,068389454	0,037058696	0,648560019	0,381435717	0,324220359	0,169255456	0,120255456	**-1,139744544**
chrVII	707416	707536	120	0,168124074	0,641115437	0,207755642	-0,814233348	-0,692098346	-0,104073719	0,063926281	**-1,196073719**
chrVII	709744	709890	146	0,202318801	0,159352392	0,559399823	0,149447726	0,127030567	-0,062973352	-0,076973352	**-1,336973352**
chrVII	712418	712569	151	0,156725832	0,114881957	0,577029962	0,194301161	0,165155987	0,003351975	-0,045648025	**-1,305648025**
chrVII	714004	714140	136	0,082637257	0,062999783	0,56741923	0,169807485	0,144336362	0,03758359	0,09358359	**-1,16641641**
chrVII	716031	716189	158	0,04844253	0,08894087	0,352608321	-0,378288023	-0,32154482	-0,320539305	-0,418539305	**-1,678539305**
chrVII	716536	716689	153	0,173823196	0,307587175	0,361070733	-0,355598233	-0,302258498	-0,399950029	-0,462950029	**-1,722950029**
chrVII	726352	726496	144	0,270708255	0,166764131	0,618800783	0,302332726	0,256982817	0,124009755	0,124009755	**-1,135990245**
chrVII	731531	731680	149	0,723788388	0,533645219	0,575607638	0,190669259	0,16206887	-0,002052363	-0,037052363	**-1,297052363**
chrVII	740022	740127	105	4,798660023	35,6801123	0,118547568	-1,182278695	-1,00493689	-0,477218768	-0,204218768	**-1,464218768**
chrVII	746153	746303	150	0,188070999	0,244587392	0,434687048	-0,164453569	-0,139785534	-0,287410555	-0,329410555	**-1,589410555**
chrVII	747978	748102	124	0,675345858	2,116051529	0,241938271	-0,700081285	-0,595069093	-0,140440085	-0,000440085	**-1,260440085**
chrVII	748361	748501	140	0,319150785	0,148234783	0,682842618	0,475662609	0,404313218	0,285265856	0,313265856	**-0,946734144**
chrVII	751512	751624	112	2,846711023	15,45718201	0,155524894	-1,013021717	-0,86106846	-0,234813929	-0,010813929	**-1,270813929**
chrVII	758738	758894	156	0,738036191	0,389116306	0,654779361	0,398256287	0,338517844	0,302455459	0,218455459	**-1,041544541**
chrVII	760806	760962	156	0,427434088	0,340940001	0,556283839	0,141553976	0,120320879	0,086265624	0,002265624	**-1,257734376**
chrVII	760828	760978	150	1,963347242	1,252583918	0,610506614	0,280639687	0,238543734	0,089742023	0,047742023	**-1,212257977**
chrVII	762415	762565	150	0,205168362	0,133411305	0,605967759	0,268824834	0,228501109	0,079942294	0,037942294	**-1,222057706**
chrVII	766213	766368	155	1,584355684	0,796761959	0,665383203	0,427200092	0,363120078	0,278020661	0,201020661	**-1,058979339**
chrVII	769383	769497	114	0,065539893	0,211234566	0,236798921	-0,71663743	-0,609141816	0,000369645	0,210369645	**-1,049630355**
chrVII	773391	773557	166	0,051292091	0,166764131	0,235224155	-0,721749813	-0,613487341	-0,453772188	-0,607772188	**-1,867772188**
chrVII	776840	777001	161	0,176672756	0,129705435	0,576649256	0,193328781	0,164329464	0,174341115	0,055341115	**-1,204658885**
chrVII	776982	777149	167	0,567062556	0,681880002	0,454034136	-0,115475457	-0,098154138	0,065349084	-0,095650916	**-1,355650916**
chrVII	780856	781004	148	1,048638295	0,433586741	0,707475768	0,546025419	0,464121606	0,242424011	0,214424011	**-1,045575989**
chrVII	781219	781380	161	1,162620718	1,041349352	0,527512027	0,069017177	0,058664601	0,078215792	-0,040784208	**-1,300784208**
chrVII	782373	782522	149	0,113982423	0,085235	0,572150875	0,181852797	0,154574878	-0,007708936	-0,042708936	**-1,302708936**
chrVII	782985	783136	151	0,202318801	0,207528696	0,493644105	-0,015932539	-0,013542658	-0,184305693	-0,233305693	**-1,493305693**
chrVII	811114	811251	137	0,04844253	0,08894087	0,352608321	-0,378288023	-0,32154482	-0,437645565	-0,388645565	**-1,648645565**
chrVII	813520	813649	129	0,188070999	0,396528045	0,321709385	-0,462924104	-0,393485489	-0,113882154	-0,008882154	**-1,268882154**
chrVII	813597	813761	164	0,504372223	0,492880654	0,505761613	0,014442723	0,012276315	0,099684987	-0,040315013	**-1,300315013**
chrVII	814985	815128	143	0,239363089	0,103764348	0,69759239	0,517488464	0,439865194	0,273929894	0,280929894	**-0,979070106**
chrVII	816260	816426	166	0,042743409	0,085235	0,333989218	-0,428924135	-0,364585514	-0,194770339	-0,348770339	**-1,608770339**
chrVII	817064	817213	149	0,085486818	0,085235	0,500737507	0,001848658	0,001571359	-0,154977044	-0,189977044	**-1,449977044**
chrVII	817068	817218	150	0,059840772	0,044470435	0,573675388	0,185739279	0,157878387	0,023365063	-0,018634937	**-1,278634937**
chrVII	817863	817968	105	0,028495606	0,485468915	0,055442749	-1,594225676	-1,355091824	-0,804400316	-0,531400316	**-1,791400316**
chrVII	817863	817970	107	0,028495606	0,311293045	0,083862736	-1,379549252	-1,172616864	-0,524364634	-0,265364634	**-1,525364634**
chrVII	819019	819169	150	0,53571739	0,433586741	0,552682458	0,13244151	0,112575284	-0,011251878	-0,053251878	**-1,313251878**
chrVII	819762	819909	147	0,19661968	0,163058261	0,546654819	0,117214141	0,09963202	-0,085069831	-0,106069831	**-1,366069831**

Chrom	Start	End	Length	Section A	Section B	A/A+B	Z-score	Z * 0.85	Phase correction	Length correction	ΔLKnuc
chrVII	820415	820572	157	0,054141651	0,081529131	0,399066404	-0,255764342	-0,217399691	-0,206311394	-0,297311394	**-1,557311394**
chrVII	820601	820751	150	1,091381703	0,622586089	0,636757416	0,349804839	0,297334113	0,169797521	0,127797521	**-1,132202479**
chrVII	820792	820937	145	0,113982423	0,051882174	0,68720164	0,487933817	0,414743744	0,247060269	0,240060269	**-1,019939731**
chrVII	821063	821198	135	0,113982423	0,077823261	0,594259882	0,238516864	0,202739335	0,155586555	0,218586555	**-1,041413445**
chrVII	821345	821513	168	0,042743409	0,048176305	0,470122564	-0,074961771	-0,063717505	0,207632583	0,039632583	**-1,220367417**
chrVII	822101	822241	140	0,153876272	0,107470218	0,588782623	0,224414545	0,190752364	0,074049724	0,102049724	**-1,157950276**
chrVII	822107	822241	134	0,125380666	0,103764348	0,547167331	0,118507771	0,100731606	0,08663241	0,15663241	**-1,10336759**
chrVII	822519	822674	155	0,085486818	0,125999566	0,404219015	-0,242441566	-0,206075331	-0,284234488	-0,361234488	**-1,621234488**
chrVII	822650	822758	108	0,062690333	0,163058261	0,277699771	-0,589688448	-0,501235181	0,178474159	0,430474159	**-0,829525841**
chrVII	825754	825897	143	0,601257283	0,363175219	0,62343117	0,314504815	0,267329093	0,115715303	0,122715303	**-1,137284697**
chrVII	831865	832015	150	0,29065518	0,233469783	0,554553208	0,137173592	0,116597553	-0,015760078	-0,057760078	**-1,317760078**
chrVII	841785	841939	154	0,151026711	0,140823044	0,517481027	0,043832468	0,037257598	-0,032164835	-0,102164835	**-1,362164835**
chrVII	842909	843085	176	0,170973635	0,307587175	0,357266269	-0,365775588	-0,310909249	0,11531991	-0,10868009	**-1,36868009**
chrVII	842909	843085	176	0,170973635	0,307587175	0,357266269	-0,365775588	-0,310909249	0,11531991	-0,10868009	**-1,36868009**
chrVII	843924	844090	166	0,091185939	0,059293913	0,605967759	0,268824834	0,228501109	0,400989568	0,246989568	**-1,013010432**
chrVII	844983	845124	141	0,370442876	0,166764131	0,689571936	0,494637357	0,420441753	0,30966266	0,33066266	**-0,92933734**
chrVII	847489	847638	149	0,170973635	0,085235	0,667321907	0,432530091	0,367650578	0,210204546	0,175204546	**-1,084795454**
chrVII	853632	853775	143	0,504372223	0,251999131	0,666831471	0,431180602	0,366503512	0,198755499	0,205755499	**-1,054244501**
chrVII	855743	855883	140	1,359240398	0,993173047	0,577806763	0,196285802	0,166842931	0,04724025	0,07524025	**-1,18475975**
chrVII	855860	856013	153	0,113982423	0,122293696	0,482411949	-0,044100996	-0,037485847	-0,145646796	-0,208646796	**-1,468646796**
chrVII	861234	861372	138	0,874815099	0,933879134	0,483672189	-0,040939185	-0,034798307	-0,134465861	-0,092465861	**-1,352465861**
chrVII	869483	869631	148	0,054141651	0,048176305	0,529151025	0,07313593	0,062165541	-0,162254733	-0,190254733	**-1,450254733**
chrVII	873303	873436	133	0,410336724	0,670762394	0,379555137	-0,306649365	-0,260651961	-0,225824646	-0,148824646	**-1,408824646**
chrVII	873927	874079	152	0,142478029	0,077823261	0,646741692	0,376538476	0,320057705	0,190200841	0,134200841	**-1,125799159**
chrVII	878924	879054	130	1,250957096	1,767699789	0,414408508	-0,216219036	-0,18378618	0,024604983	0,122604983	**-1,137395017**
chrVII	879023	879172	149	0,552814753	0,318704784	0,634311372	0,343294051	0,291799943	0,137427531	0,102427531	**-1,157572469**
chrVII	879984	880088	104	0,364743755	2,28281566	0,137766032	-1,09041117	-0,926849494	-0,41452278	-0,13452278	**-1,39452278**
chrVII	882915	883023	108	0,09688506	0,048176305	0,667890174	0,43409473	0,36898052	1,042251293	1,294251293	**0,034251293**
chrVII	882915	883031	116	0,088336378	0,040764565	0,6842427	0,479596127	0,407656708	0,993893655	1,189893655	**-0,070106345**
chrVII	887398	887557	159	0,544266071	0,61888022	0,467925725	-0,080485095	-0,068412331	-0,04758926	-0,15258926	**-1,41258926**
chrVII	889980	890139	159	0,159575393	0,255705001	0,384259395	-0,294312935	-0,250165995	-0,21106307	-0,31606307	**-1,57606307**
chrVII	889980	890139	159	0,159575393	0,255705001	0,384259395	-0,294312935	-0,250165995	-0,21106307	-0,31606307	**-1,57606307**
chrVII	892462	892609	147	0,53571739	0,429880871	0,554803598	0,137807188	0,11713611	-0,0694593	-0,0904593	**-1,3504593**
chrVII	895680	895828	148	0,085486818	0,100058479	0,460732874	-0,09858756	-0,083799426	-0,289695109	-0,317695109	**-1,577695109**
chrVII	911520	911682	162	0,074088575	0,055588044	0,571333335	0,179769754	0,152804291	0,170656766	0,044656766	**-1,215343234**
chrVII	914384	914518	134	0,304902982	0,129705435	0,701557931	0,528886572	0,449553586	0,428768128	0,498768128	**-0,761231872**
chrVII	920349	920433	84	0,208017923	0,459527828	0,311615979	-0,491275002	-0,417583751	-0,052818035	0,367181965	**-0,892818035**
chrVII	922436	922538	102	0,267858695	2,960989793	0,082957963	-1,385446676	-1,177629675	-0,704878864	-0,410878864	**-1,670878864**
chrVII	925873	926024	151	0,170973635	0,166764131	0,506231912	0,015621722	0,013278464	-0,148316922	-0,197316922	**-1,457316922**
chrVII	928464	928607	143	0,085486818	0,107470218	0,443035505	-0,14327752	-0,121785892	-0,291581278	-0,284581278	**-1,544581278**
chrVII	928560	928710	150	0,24506221	0,200116957	0,55047996	0,126874057	0,107842948	-0,01855031	-0,06055031	**-1,32055031**
chrVII	929745	929899	154	0,076938136	0,144528914	0,34740218	-0,39234359	-0,333492051	-0,399924862	-0,469924862	**-1,729924862**
chrVII	938659	938812	153	0,034194727	0,051882174	0,397257877	-0,260451199	-0,221383519	-0,326277095	-0,389277095	**-1,649277095**
chrVII	943256	943409	153	2,231205937	1,504583049	0,597251597	0,246239543	0,209303612	0,10965375	0,04665375	**-1,21334625**
chrVII	943702	943854	152	0,83207169	0,61146848	0,576410485	0,192719017	0,163811164	0,045005996	-0,010994004	**-1,270994004**
chrVII	948590	948746	156	0,105433742	0,148234783	0,415635884	-0,213070763	-0,181110149	-0,211273123	-0,295273123	**-1,555273123**
chrVII	949733	949881	148	2,3423388	1,793640876	0,56633228	0,167043985	0,141987387	-0,06073511	-0,08873511	**-1,34873511**
chrVII	960612	960754	142	1,134125112	0,629997828	0,642883263	0,366176369	0,311249914	0,173962263	0,187962263	**-1,072037737**
chrVII	961680	961814	134	0,159575393	0,43729261	0,267354577	-0,620833537	-0,527708506	-0,552766225	-0,482766225	**-1,742766225**
chrVII	961681	961788	107	0,028495606	0,185293479	0,133288404	-1,11098035	-0,944333298	-0,28136431	-0,02236431	**-1,28236431**
chrVII	961681	961791	110	0,031345166	0,066705652	0,319682862	-0,468585806	-0,398297935	0,244361751	0,482361751	**-0,777638249**
chrVII	961681	961794	113	0,031345166	0,062999783	0,332240005	-0,433736208	-0,368675777	0,2434063	0,4604063	**-0,7995937**
chrVII	961681	961795	114	0,074088575	0,077823261	0,487707719	-0,030817056	-0,026194498	0,58259512	0,79259512	**-0,46740488**
chrVII	961681	961796	115	0,031345166	0,066705652	0,319682862	-0,468585806	-0,398297935	0,196571125	0,399571125	**-0,860428875**
chrVII	961681	961797	116	0,045592969	0,222352175	0,170157849	-0,953541662	-0,810510413	-0,226380421	-0,030380421	**-1,290380421**
chrVII	961681	961798	117	0,045592969	0,066705652	0,405997586	-0,23785292	-0,202174982	0,370580049	0,559580049	**-0,700419951**
chrVII	961681	961799	118	0,111132863	0,43729261	0,202639863	-0,83222895	-0,707394607	-0,102469031	0,079530969	**-1,180469031**
chrVII	961681	961800	119	0,071239015	0,059293913	0,545755127	0,114943701	0,097702146	0,699935031	0,874935031	**-0,385064969**
chrVII	961681	961801	120	0,125380666	0,207528696	0,376621026	-0,314367347	-0,267212245	0,300247709	0,468247709	**-0,791752291**
chrVII	961681	961802	121	0,045592969	0,240881523	0,159151934	-0,997949458	-0,84825704	-0,323433316	-0,162433316	**-1,422433316**
chrVII	961681	961803	122	0,039893848	0,125999566	0,240478794	-0,704763239	-0,599048753	-0,112893163	0,041106837	**-1,218893163**
chrVII	961681	961804	123	0,085486818	0,337234132	0,202229905	-0,833682698	-0,708630293	-0,229559562	-0,082559562	**-1,342559562**
chrVII	961681	961805	124	0,045592969	0,133411305	0,254703244	-0,659762131	-0,560797812	-0,114138058	0,025861942	**-1,234138058**
chrVII	961681	961806	125	0,111132863	1,515700658	0,068312376	-1,488478487	-1,265206714	-0,848143821	-0,715143821	**-1,975143821**
chrVII	961681	961807	126	0,068389454	0,148234783	0,315705458	-0,47974192	-0,407780632	0,024180985	0,150180985	**-1,109819015**
chrVII	961681	961808	127	0,04844253	0,044470435	0,521375354	0,053605728	0,045564869	0,456144939	0,575144939	**-0,684855061**
chrVII	961681	961809	128	0,14817715	0,248293262	0,373740753	-0,321961969	-0,273667674	0,079897227	0,191897227	**-1,068102773**
chrVII	961681	961810	129	0,094035499	0,196411088	0,323761764	-0,457205247	-0,38862446	-0,1218176	-0,0168176	**-1,2768176**
chrVII	961681	961811	130	0,071239015	0,118587827	0,375284202	-0,317889966	-0,270206471	-0,070362305	0,027637695	**-1,232362305**
chrVII	961681	961812	131	0,267858695	0,274234349	0,49411941	-0,014740987	-0,012529839	0,132543111	0,223543111	**-1,036456889**
chrVII	961681	961813	132	0,094035499	0,170470001	0,355514344	-0,370474883	-0,31490365	-0,257047964	-0,173047964	**-1,433047964**
chrVII	961681	961814	133	96,44907707	78,60519963	0,5509667	0,128104088	0,108888475	0,12810508	0,20510508	**-1,05489492**
chrVII	961682	961814	132	0,373292436	0,422469132	0,46910086	-0,077530259	-0,06590072	-0,019670018	0,064329982	**-1,195670018**
chrVII	961683	961814	131	0,085486818	0,125999566	0,404219015	-0,242441566	-0,206075331	-0,082596042	0,008403958	**-1,251596042**
chrVII	961684	961814	130	0,065539893	0,218646305	0,230623069	-0,736796456	-0,626276987	-0,426790809	-0,328790809	**-1,588790809**
chrVII	961685	961814	129	0,128230226	0,185293479	0,408996909	-0,230126057	-0,195607148	0,058374438	0,163374438	**-1,096625562**
chrVII	961686	961814	128	0,435982769	0,429880871	0,503523591	0,008832447	0,00750758	0,35450653	0,46650653	**-0,79349347**
chrVII	961687	961814	127	0,062690333	0,096352609	0,394172367	-0,268460684	-0,228191582	0,191365107	0,310365107	**-0,949634893**
chrVII	961694	961814	120	0,039893848	0,129705435	0,235224155	-0,721749813	-0,613487341	-0,071899238	0,096100762	**-1,163899238**
chrVII	961695	961814	119	0,045592969	0,081529131	0,358654941	-0,362056402	-0,307747942	0,304698856	0,479698856	**-0,780301144**
chrVII	961696	961814	118	0,051292091	0,170470001	0,231293321	-0,734594249	-0,624405111	-0,021381997	0,160618003	**-1,099381997**
chrVII	961708	961806	98	0,091185939	0,218646305	0,294307453	-0,540844298	-0,459717653	-0,011572954	0,310427046	**-0,949572954**
chrVII	962925	963072	147	0,133929347	0,107470218	0,554803598	0,137807188	0,11713611	-0,060990433	-0,081990433	**-1,341990433**
chrVII	966527	966637	110	1,31934655	9,679731339	0,119950651	-1,175233524	-0,998948496	-0,337676012	-0,099676012	**-1,359676012**
chrVII	974170	974327	157	0,054141651	0,062999783	0,462190442	-0,094916836	-0,080679311	-0,068815787	-0,159815787	**-1,419815787**
chrVII	975022	975129	107	2,809666735	9,312850251	0,231772555	-0,733021838	-0,623068562	0,03941828	0,29841828	**-0,96158172**
chrVII	975022	975130	108	0,059840772	0,218646305	0,214878093	-0,789608938	-0,671167597	-0,010475442	0,241524558	**-1,018475442**
chrVII	975022	975131	109	0,045592969	0,281646088	0,139326185	-1,083351646	-0,920848899	-0,237938788	0,007061212	**-1,252938788**
chrVII	975022	975142	120	3,604694138	12,44431004	0,224605471	-0,756731162	-0,643221488	-0,117455307	0,050544693	**-1,209455307**
chrVII	979943	980105	162	1,020142689	0,733762177	0,581640834	0,206092917	0,175178979	0,193558439	0,067558439	**-1,192441561**
chrVII	982482	982630	148	0,133929347	0,062999783	0,680089062	0,46794786	0,397755681	0,208140798	0,180140798	**-1,079859202**
chrVII	987451	987604	153	0,039893848	0,055588044	0,417815854	-0,20748423	-0,176361595	-0,28099418	-0,34399418	**-1,60399418**
chrVII	987451	987606	155	0,034194727	0,055588044	0,380860679	-0,303221116	-0,257737948	-0,336811507	-0,413811507	**-1,673811507**
chrVII	987451	987608	157	0,039893848	0,074117392	0,349911537	-0,385559308	-0,327725412	-0,323417946	-0,414417946	**-1,674417946**
chrVII	987451	987609	158	10,96225956	12,62589765	0,464735734	-0,088509834	-0,075233359	-0,071625702	-0,169625702	**-1,429625702**
chrVII	988814	988967	153	0,051292091	0,037058696	0,58055047	0,203301913	0,172806626	0,08146539	0,01846539	**-1,24153461**

Chrom	Start	End	Length	Section A	Section B	A/A+B	Z-score	Z * 0.85	Phase correction	Length correction	ΔLKnuc
chrVII	988881	989019	138	1,020142689	0,381704567	0,727713155	0,605911247	0,51502456	0,414284484	0,456284484	**-0,803715516**
chrVII	989319	989475	156	0,051292091	0,037058696	0,58055047	0,203301913	0,172806626	0,146733935	0,062733935	**-1,197266065**
chrVII	989319	989477	158	5,932785135	5,62921589	0,513127885	0,032912669	0,027975769	0,029374917	-0,068625083	**-1,328625083**
chrVII	991949	992103	154	0,997346204	0,681880002	0,593932015	0,237671396	0,202020687	0,140968505	0,070968505	**-1,189031495**
chrVII	999326	999473	147	0,116831984	0,118587827	0,496270827	-0,009347788	-0,007945619	-0,183530646	-0,204530646	**-1,464530646**
chrVII	1001929	1002078	149	0,373292436	0,203822827	0,646824751	0,376761979	0,320247683	0,167800202	0,132800202	**-1,127199798**
chrVII	1012079	1012238	159	0,09973462	0,166764131	0,374240479	-0,320642986	-0,272546538	-0,236341534	-0,341341534	**-1,601341534**
chrVII	1012603	1012726	123	0,347646391	1,815876094	0,160685361	-0,991645016	-0,842898264	-0,387318607	-0,240318607	**-1,500318607**
chrVII	1016668	1016789	121	0,059840772	0,122293696	0,328552705	-0,443913104	-0,377326138	0,129959103	0,290959103	**-0,969040897**
chrVII	1018529	1018686	157	0,170973635	0,103764348	0,622315244	0,311567137	0,264832067	0,259547725	0,168547725	**-1,091452275**
chrVII	1018639	1018770	131	0,142478029	0,140823044	0,502920895	0,007321663	0,006223413	0,140118615	0,231118615	**-1,028881385**
chrVII	1024687	1024847	160	0,088336378	0,044470435	0,665149446	0,426558255	0,362574517	0,387499338	0,275499338	**-0,984500662**
chrVII	1030700	1030865	165	0,102584181	0,096352609	0,515662191	0,039269381	0,033378974	0,14295827	-0,00404173	**-1,26404173**
chrVII	1030899	1031060	161	0,034194727	0,044470435	0,434687048	-0,164453569	-0,139785534	-0,113972672	-0,232972672	**-1,492972672**
chrVII	1037731	1037891	160	0,037044288	0,059293913	0,384523349	-0,293622086	-0,249578773	-0,23194904	-0,34394904	**-1,60394904**
chrVII	1039928	1040095	167	0,883363781	1,14140783	0,436278233	-0,16041209	-0,136350277	0,067492627	-0,093507373	**-1,353507373**
chrVII	1043906	1044058	152	0,219416165	0,118587827	0,649152585	0,38303363	0,325578585	0,206128415	0,150128415	**-1,109871585**
chrVII	1044184	1044344	160	0,726637949	0,726350437	0,500098938	0,000248001	0,000210801	0,029784739	-0,082215261	**-1,342215261**
chrVII	1045755	1045901	146	1,094231264	0,711526959	0,605967759	0,268824834	0,228501109	0,039137381	0,025137381	**-1,234862619**
chrVII	1049209	1049360	151	0,059840772	0,051882174	0,535617563	0,08939893	0,07598909	-0,07606989	-0,12506989	**-1,38506989**
chrVII	1051642	1051789	147	0,735186631	0,340940001	0,683178549	0,47660573	0,40511487	0,231132449	0,210132449	**-1,049867551**
chrVII	1052012	1052165	153	0,29065518	0,222352175	0,566571175	0,16765125	0,142503563	0,065309513	0,002309513	**-1,257690487**
chrVII	1065087	1065242	155	0,384690679	0,326116523	0,541202562	0,103463804	0,087944233	0,013425617	-0,063574383	**-1,323574383**
chrVII	1065864	1066016	152	13,65509432	7,630385462	0,641521566	0,36252885	0,308149523	0,198340983	0,142340983	**-1,117659017**
chrVII	1065866	1066016	150	0,039893848	0,037058696	0,518421434	0,046192108	0,039263292	-0,085352075	-0,127352075	**-1,387352075**
chrVII	1065867	1066016	149	0,056991212	0,037058696	0,605967759	0,268824834	0,228501109	0,079664841	0,044664841	**-1,215335159**
chrVII	1067431	1067584	153	0,179522317	0,155646522	0,535617563	0,08939893	0,07598909	0,001664918	-0,061335082	**-1,321335082**
chrVII	1071145	1071303	158	0,53571739	0,426175002	0,556941083	0,143218227	0,121735493	0,122071387	0,024071387	**-1,235928613**
chrVII	1079049	1079187	138	0,034194727	0,062999783	0,351817475	-0,380418277	-0,323355536	-0,443784877	-0,401784877	**-1,661784877**
chrVII	1079684	1079846	162	0,119681545	0,118587827	0,502295129	0,005753068	0,004890107	0,036271509	-0,089728491	**-1,349728491**
chrVII	1080323	1080452	129	0,162424953	0,344645871	0,320320053	-0,466804011	-0,396783409	-0,158721496	-0,053721496	**-1,313721496**
chrVII	1085801	1085942	141	0,111132863	0,085235	0,565942212	0,166052574	0,141144688	0,027549433	0,048549433	**-1,211450567**
chrVII	1087005	1087167	162	0,042743409	0,040764565	0,51184823	0,029703476	0,025247955	0,065816684	-0,060183316	**-1,320183316**
chrVII	1087005	1087167	162	0,042743409	0,040764565	0,51184823	0,029703476	0,025247955	0,065816684	-0,060183316	**-1,320183316**
chrVII	1087106	1087261	155	0,091185939	0,211234566	0,301520357	-0,520032799	-0,442027879	-0,507548914	-0,584548914	**-1,844548914**
chrVII	1087240	1087377	137	0,14817715	0,114881957	0,563284624	0,159302245	0,135406908	0,011856877	0,060856877	**-1,199143123**
chrVII	1087527	1087678	151	0,062690333	0,040764565	0,605967759	0,268824834	0,228501109	0,07157271	0,02257271	**-1,23742729**
chrVII	1087535	1087678	143	0,356195073	0,470645436	0,430790544	-0,174361858	-0,148207579	-0,319093981	-0,312093981	**-1,572093981**
chrVII	1087583	1087708	125	0,188070999	0,185293479	0,503719582	0,009323745	0,007925183	0,433284586	0,566284586	**-0,693715414**
chrVII	1087808	1087960	152	0,113982423	0,077823261	0,594259882	0,238516864	0,202739335	0,096716138	0,040716138	**-1,219283862**
chrVII	1087881	1088025	144	0,088336378	0,044470435	0,665149446	0,426558255	0,362574517	0,236936735	0,236936735	**-1,023063265**
chrVII	1087978	1088113	135	1,188266763	0,733762177	0,618235625	0,30085017	0,255722644	0,203690499	0,266690499	**-0,993309501**
chrVII	1087978	1088165	187	0,125380666	0,074117392	0,628480635	0,327831948	0,278657156	0,722103776	0,421103776	**-0,838896224**
chrVII	1088021	1088113	92	0,105433742	0,100058479	0,513078994	0,032790051	0,027871543	0,417436812	0,781436812	**-0,478563188**
chrVII	1088021	1088165	144	1,347842156	0,592939133	0,694484311	0,508601784	0,432311517	0,304489824	0,304489824	**-0,955510176**
chrVII	1088360	1088512	152	0,136778908	0,125999566	0,520510322	0,051434423	0,043719259	-0,061737626	-0,117737626	**-1,377737626**
chrVII	1088661	1088791	130	0,444531451	0,448410219	0,497828096	-0,005444184	-0,004627556	0,202514043	0,300514043	**-0,959485957**
chrVII	1089031	1089182	151	0,416035845	0,285351958	0,593160935	0,235683687	0,200331134	0,052210155	0,003210155	**-1,256789845**
chrVII	1089036	1089170	134	0,700991904	0,426175002	0,621906038	0,310490576	0,263916989	0,232846895	0,302846895	**-0,957153105**
chrVII	1089049	1089145	96	0,085486818	0,122293696	0,411428464	-0,223871969	-0,190291174	0,221216578	0,557216578	**-0,702783422**
chrVII	1090196	1090347	151	0,558513874	0,400233914	0,58254515	0,20840891	0,177147573	0,032894276	-0,016105724	**-1,276105724**
chrVIII	691	842	151	0,58415992	0,392822175	0,597922851	0,247974292	0,210778148	0,094444938	0,045444938	**-1,214555062**
chrVIII	1289	1405	116	0,156725832	0,229763914	0,405510971	-0,239107867	-0,203241687	0,269748559	0,465748559	**-0,794251441**
chrVIII	1346	1501	155	0,53856695	0,522527611	0,507557922	0,018946034	0,016104129	0,026227512	-0,050772488	**-1,310772488**
chrVIII	1712	1899	187	0,076938136	0,122293696	0,386173911	-0,289305214	-0,245909432	0,052637638	-0,248362362	**-1,508362362**
chrVIII	1733	1899	166	0,102584181	0,741173916	0,121580085	-1,167124315	-0,992055668	-0,83701612	-0,99101612	**-2,25101612**
chrVIII	1748	1846	98	0,028495606	0,077823261	0,268020217	-0,618811668	-0,525989918	0,110862216	0,432862216	**-0,827137784**
chrVIII	1748	1855	107	0,624053768	2,797931532	0,182365999	-0,90638521	-0,770427429	-0,083236509	0,175763491	**-1,084236509**
chrVIII	1748	1878	130	0,042743409	0,233469783	0,15474789	-1,016280608	-0,863838517	-0,406753508	-0,308753508	**-1,568753508**
chrVIII	1748	1879	131	0,273557816	0,444704349	0,380860679	-0,303221211	-0,257737948	0,153635608	0,244635608	**-1,015364392**
chrVIII	1748	1880	132	0,059840772	0,122293696	0,328552705	-0,443913104	-0,377326138	-0,009239046	0,074760954	**-1,185239046**
chrVIII	1748	1889	141	0,056991212	0,100058479	0,362886495	-0,350753893	-0,298140809	-0,177915625	-0,156915625	**-1,416915625**
chrVIII	1748	1891	143	0,259310013	0,366881088	0,414106832	-0,216993175	-0,184444199	-0,098785569	-0,091785569	**-1,351785569**
chrVIII	1748	1894	146	0,239363089	0,151940653	0,611706619	0,28376985	0,241204373	0,246443036	0,232443036	**-1,027556964**
chrVIII	1748	1895	147	0,24221265	0,277940218	0,465656665	-0,08619258	-0,073263693	-0,110704254	-0,131704254	**-1,391704254**
chrVIII	1748	1896	148	0,225115286	0,144528914	0,609005325	0,276727507	0,235218381	0,159351768	0,131351768	**-1,128648232**
chrVIII	1748	1897	149	0,199469241	0,196411088	0,503862472	0,009681932	0,008229642	-0,120258873	-0,155258873	**-1,415258873**
chrVIII	1748	1898	150	0,116831984	0,211234566	0,356122817	-0,368841816	-0,313515544	-0,417025954	-0,459025954	**-1,719025954**
chrVIII	1748	1899	151	133,1257713	80,91766225	0,621956811	0,310624134	0,264030513	0,15163894	0,10263894	**-1,15736106**
chrVIII	1748	1909	161	0,122531105	0,604056741	0,168639079	-0,959557122	-0,815623554	-0,73715602	-0,85615602	**-2,11615602**
chrVIII	1748	1932	184	0,151026711	0,537351089	0,219395092	-0,77423779	-0,658102121	-0,364963662	-0,644963662	**-1,904963662**
chrVIII	1749	1899	150	0,170973635	0,233469783	0,422738082	-0,194893703	-0,165659647	-0,273487647	-0,315487647	**-1,575487647**
chrVIII	1750	1899	149	0,319150785	0,422469132	0,430342791	-0,175501512	-0,149176285	-0,279888675	-0,314888675	**-1,574888675**
chrVIII	1751	1899	148	0,111132863	0,185293479	0,374908863	-0,318879716	-0,271047759	-0,346679602	-0,374679602	**-1,634679602**
chrVIII	1752	1899	147	1,003045325	0,867173481	0,53632512	0,091179756	0,077502792	0,017650706	-0,003349294	**-1,263349294**
chrVIII	1753	1899	146	0,159575393	0,133411305	0,544650641	0,112157259	0,09533367	0,111183624	0,097183624	**-1,162816376**
chrVIII	1754	1899	145	0,190920559	0,214940436	0,47040874	-0,074242436	-0,063106071	-0,042440533	-0,049440533	**-1,309440533**
chrVIII	1755	1899	144	0,051292091	0,211234566	0,1953786	-0,858244988	-0,72950824	-0,617474038	-0,617474038	**-1,877474038**
chrVIII	1756	1899	143	0,051292091	0,133411305	0,277699771	-0,589688448	-0,501235181	-0,413952	-0,406952	**-1,666952**
chrVIII	1757	1899	142	0,14817715	0,207528696	0,416572153	-0,210670615	-0,179070023	-0,062406001	-0,048406001	**-1,308406001**
chrVIII	1758	1899	141	0,262159574	0,503998263	0,342174368	-0,406536101	-0,345555686	-0,200200269	-0,179200269	**-1,439200269**
chrVIII	1759	1899	140	0,04844253	0,062999783	0,434687048	-0,164453569	-0,139785534	-0,004253829	0,023746171	**-1,236253829**
chrVIII	1760	1899	139	0,133929347	0,270528479	0,331133034	-0,436786668	-0,371268668	-0,213682121	-0,178682121	**-1,438682121**
chrVIII	1761	1899	138	0,04844253	0,248293262	0,163251388	-0,981182451	-0,834005084	-0,667350855	-0,625350855	**-1,885350855**
chrVIII	1762	1899	137	0,079787696	0,307587175	0,205970243	-0,820483581	-0,697411043	-0,527108503	-0,478108503	**-1,738108503**
chrVIII	1764	1899	135	0,111132863	0,192705218	0,365763444	-0,34309514	-0,291630869	-0,0436654	0,0193346	**-1,2406654**
chrVIII	1765	1899	134	0,059840772	0,203822827	0,226958793	-0,748899824	-0,63656485	-0,356702362	-0,286702362	**-1,546702362**
chrVIII	1766	1899	133	0,042743409	0,055588044	0,434687048	-0,164453569	-0,139785534	0,206191624	0,283191624	**-0,976808376**
chrVIII	1769	1899	130	0,142478029	0,166764131	0,460732874	-0,09858756	-0,083799426	0,361700525	0,459700525	**-0,800299475**
chrVIII	1771	1899	128	0,042743409	0,259410871	0,141462199	-1,073773081	-0,912707119	-0,401747623	-0,289747623	**-1,549747623**
chrVIII	1773	1899	126	0,054141651	0,26682261	0,168684361	-0,959377271	-0,81547068	-0,267502018	-0,141502018	**-1,401502018**
chrVIII	1784	1899	115	0,136778908	0,170470001	0,445172966	-0,137866493	-0,117186519	0,367194985	0,570194985	**-0,689805015**
chrVIII	1821	1968	147	0,199469241	0,051882174	0,793587102	0,818930974	0,696091328	0,644736724	0,623736724	**-0,636263276**
chrVIII	2051	2222	171	0,108283302	0,055588044	0,660782406	0,414599399	0,352409489	0,545568832	0,356568832	**-0,903431168**
chrVIII	2441	2594	153	0,803576085	0,500292393	0,616301489	0,295781409	0,251414198	0,181389708	0,118389708	**-1,141610292**
chrVIII	2951	3103	152	0,091185939	0,081529131	0,527955893	0,070132481	0,059612609	-0,007567952	-0,063567952	**-1,323567952**

Chrom	Start	End	Length	Section A	Section B	A/A+B	Z-score	Z * 0.85	Phase correction	Length correction	ΔLKnuc
chrVIII	3093	3220	127	0,555664314	1,174760656	0,321114364	-0,464584928	-0,394897188	0,144799093	0,263799093	**-0,996200907**
chrVIII	3233	3376	143	0,373292436	0,392822175	0,487254036	-0,031954832	-0,027161607	0,056225957	0,063225957	**-1,196774043**
chrVIII	3524	3661	137	0,470177496	0,533645219	0,468386986	-0,079325188	-0,06742641	0,059508263	0,108508263	**-1,151491737**
chrVIII	4667	4822	155	0,316301225	0,500292393	0,387342269	-0,286252759	-0,243314845	-0,224009173	-0,301009173	**-1,561009173**
chrVIII	9299	9453	154	0,53571739	0,629997828	0,459561119	-0,101539457	-0,086308538	-0,093690734	-0,163690734	**-1,423690734**
chrVIII	10577	10683	106	0,418885406	2,697873053	0,134397776	-1,105840522	-0,939964444	-0,316720016	-0,050720016	**-1,310720016**
chrVIII	10578	10683	105	0,059840772	0,885702829	0,063287163	-1,527751185	-1,298588507	-0,650540864	-0,377540864	**-1,637540864**
chrVIII	10864	11003	139	0,310602104	0,229763914	0,574799476	0,188606742	0,160315731	0,316148488	0,351148488	**-0,908851512**
chrVIII	12729	12877	148	0,028495606	0,044470435	0,390532439	-0,277931585	-0,236241848	-0,314577493	-0,342577493	**-1,602577493**
chrVIII	14994	15154	160	2,19701121	2,02340479	0,520567454	0,05157782	0,043841147	0,137831864	0,025831864	**-1,234168136**
chrVIII	15294	15466	172	0,034194727	0,055588044	0,380860679	-0,303221116	-0,257737948	-0,041801652	-0,237801652	**-1,497801652**
chrVIII	17876	18017	141	0,065539893	0,085235	0,434687048	-0,164453569	-0,139785534	-0,003839156	0,017160844	**-1,242839156**
chrVIII	18350	18522	172	0,322000346	0,500292393	0,391588458	-0,275181347	-0,233904145	-0,013435581	-0,209435581	**-1,469435581**
chrVIII	23920	24090	170	0,173823196	0,400233914	0,30279774	-0,516370765	-0,438915151	-0,260762371	-0,442762371	**-1,702762371**
chrVIII	26981	27134	153	0,698142343	0,433586741	0,616881154	0,297299724	0,252704765	0,190740619	0,127740619	**-1,132259381**
chrVIII	32520	32666	146	0,210867483	0,203822827	0,508493876	0,021292599	0,018098709	0,035243604	0,021243604	**-1,238756396**
chrVIII	33329	33481	152	2,097276589	1,645406093	0,560367193	0,151900247	0,12911521	0,062739327	0,006739327	**-1,253260673**
chrVIII	33329	33481	152	2,097276589	1,645406093	0,560367193	0,151900247	0,12911521	0,062739327	0,006739327	**-1,253260673**
chrVIII	33632	33782	150	0,302053422	0,196411088	0,605967759	0,268824834	0,228501109	0,129956915	0,087956915	**-1,172043085**
chrVIII	39986	40150	164	0,071239015	0,100058479	0,415878909	-0,212447646	-0,180580499	-0,03506912	-0,17506912	**-1,43506912**
chrVIII	46044	46208	164	0,943204553	0,926467395	0,504475961	0,011219806	0,009536836	0,162822848	0,022822848	**-1,237177152**
chrVIII	48223	48365	142	0,279256937	0,185293479	0,601133757	0,25628279	0,217840372	0,336995803	0,350995803	**-0,909004197**
chrVIII	49771	49922	151	0,44168189	0,314998914	0,583709654	0,211392905	0,179683969	0,078215958	0,029215958	**-1,230784042**
chrVIII	50265	50415	150	0,19377012	0,059293913	0,765696008	0,724745812	0,61603394	0,506953564	0,464953564	**-0,795046436**
chrVIII	51501	51665	164	0,604106844	0,563292176	0,517481027	0,043832468	0,037257598	0,199471971	0,059471971	**-1,200528029**
chrVIII	52096	52204	108	0,031345166	0,188999349	0,142255261	-1,070241716	-0,909705459	-0,247071045	0,004928955	**-1,255071045**
chrVIII	52096	52213	117	0,04844253	0,26311674	0,155484155	-1,013192315	-0,861213468	-0,340502374	-0,151502374	**-1,411502374**
chrVIII	52629	52803	174	1,46467414	2,038228268	0,418131586	-0,206675654	-0,175674306	0,051816378	-0,158183622	**-1,418183622**
chrVIII	53713	53863	150	0,504372223	0,307587175	0,621179118	0,308579045	0,262292188	0,153381421	0,111381421	**-1,148618579**
chrVIII	58605	58733	128	0,373292436	0,289057827	0,563587662	0,160071596	0,136060856	0,633409479	0,745409479	**-0,514590521**
chrVIII	59349	59502	153	6,804750673	5,340158063	0,560296567	0,151721162	0,128962988	0,077061847	0,014061847	**-1,245938153**
chrVIII	61087	61247	160	0,031345166	0,096352609	0,245463684	-0,688834586	-0,585509398	-0,50021545	-0,61221545	**-1,87221545**
chrVIII	62149	62261	112	1,382036883	7,400621548	0,157359744	-1,005368404	-0,854563143	-0,254683303	-0,030683303	**-1,290683303**
chrVIII	65254	65423	169	0,082637257	0,074117392	0,527175798	0,068172392	0,057946533	0,220764326	0,045764326	**-1,214235674**
chrVIII	66426	66576	150	6,129404815	2,783108054	0,687730263	0,489426929	0,41601289	0,324511449	0,282511449	**-0,977488551**
chrVIII	66660	66810	150	0,897611584	0,796761959	0,529760151	0,074666958	0,063466914	-0,024633142	-0,066633142	**-1,326633142**
chrVIII	66812	66936	124	0,042743409	0,240881523	0,15070399	-1,033418742	-0,87840593	-0,31438553	-0,17438553	**-1,43438553**
chrVIII	66813	66902	89	0,037044288	0,062999783	0,370279692	-0,331112667	-0,281445767	0,390429391	0,775429391	**-0,484570609**
chrVIII	66813	66904	91	0,028495606	0,125999566	0,184443343	-0,898560728	-0,763776618	-0,086525697	0,284474303	**-0,975525697**
chrVIII	66813	66911	98	0,034194727	0,125999566	0,213457836	-0,794480647	-0,67530855	-0,013864323	0,308135677	**-0,951864323**
chrVIII	66813	66912	99	0,045592969	0,137117174	0,249537154	-0,67594698	-0,574554933	0,105360341	0,420360341	**-0,839639659**
chrVIII	66813	66924	111	0,059840772	0,062999783	0,487141826	-0,032236246	-0,027400809	0,564809056	0,795809056	**-0,464190944**
chrVIII	66813	66925	112	0,065539893	0,092646739	0,414320049	-0,216446021	-0,183979117	0,407903066	0,631903066	**-0,628096934**
chrVIII	66813	66926	113	0,028495606	0,085235	0,250553539	-0,67274886	-0,571836531	-0,010373951	0,206626049	**-1,053373951**
chrVIII	66813	66928	115	0,037044288	0,092646739	0,285634931	-0,566182298	-0,481254953	0,021266584	0,224266584	**-1,035733416**
chrVIII	66813	66929	116	0,074088575	0,140823044	0,344739737	-0,399561558	-0,339627325	0,143939247	0,339939247	**-0,920060753**
chrVIII	66813	66930	117	0,042743409	0,159352392	0,211500727	-0,801224908	-0,681041172	-0,15646726	0,03253274	**-1,22746726**
chrVIII	66813	66931	118	0,128230226	0,403939784	0,240957258	-0,703226647	-0,59774265	-0,079170762	0,102829238	**-1,157170762**
chrVIII	66813	66932	119	0,139628469	0,289057827	0,325712462	-0,451783548	-0,384016016	0,109174674	0,284174674	**-0,975825326**
chrVIII	66813	66933	120	0,176672756	0,333528262	0,346280681	-0,395381497	-0,336074272	0,152539665	0,320539665	**-0,939460335**
chrVIII	66813	66934	121	0,133929347	0,159352392	0,456657642	-0,108857794	-0,092529125	0,458432272	0,619432272	**-0,640567728**
chrVIII	66813	66935	122	0,065539893	0,181587609	0,265206797	-0,627374783	-0,533268565	0,02761144	0,18161144	**-1,07838856**
chrVIII	66813	66936	123	49,87870845	96,96407752	0,339674218	-0,413352411	-0,35134955	0,242906556	0,389906556	**-0,870093444**
chrVIII	66815	66936	121	0,065539893	0,244587392	0,211332238	-0,801807229	-0,681536144	-0,151420064	0,009579936	**-1,250420064**
chrVIII	66816	66936	120	0,037044288	0,181587609	0,169436794	-0,956393263	-0,812934274	-0,313470966	-0,145470966	**-1,405470966**
chrVIII	66817	66936	119	0,185221438	0,533645219	0,257657573	-0,65058387	-0,55299629	-0,04502513	0,12997487	**-1,13002513**
chrVIII	66818	66936	118	0,085486818	0,118587827	0,418899751	-0,204708983	-0,174002636	0,339622688	0,521622688	**-0,738377312**
chrVIII	66820	66936	116	0,088336378	0,34835174	0,202287111	-0,833479734	-0,708457774	-0,216546637	-0,020546637	**-1,280546637**
chrVIII	66821	66936	115	0,054141651	0,051882174	0,510655515	0,026712592	0,022705704	0,510886461	0,713886461	**-0,546113539**
chrVIII	66822	66936	114	0,051292091	0,066705652	0,434687048	-0,164453569	-0,139785534	0,316777536	0,526777536	**-0,733222464**
chrVIII	66823	66936	113	0,031345166	0,103764348	0,231998216	-0,73228205	-0,622439743	-0,063581507	0,153418493	**-1,106581507**
chrVIII	66830	66936	106	0,105433742	0,274234349	0,277699771	-0,589688448	-0,501235181	0,124420492	0,390420492	**-0,869579508**
chrVIII	68261	68399	138	0,153876272	0,125999566	0,549801916	0,125160904	0,106386768	0,28068908	0,32268908	**-0,93731092**
chrVIII	69975	70096	121	0,170973635	0,122293696	0,582995843	0,209563572	0,178129036	0,701091517	0,862091517	**-0,397908483**
chrVIII	69975	70122	147	0,316301225	0,170470001	0,649794417	0,384765495	0,327050671	0,275247812	0,254247812	**-1,005752188**
chrVIII	70402	70553	151	0,455929693	0,385410436	0,541908887	0,105243965	0,08945737	-0,003551993	-0,052551993	**-1,312551993**
chrVIII	72065	72199	134	0,53856695	0,848644133	0,388237202	-0,283916456	-0,241328988	0,053873791	0,123873791	**-1,136126209**
chrVIII	77481	77612	131	0,082637257	0,066705652	0,553339005	0,13410191	0,113986624	0,525574091	0,616574091	**-0,643425909**
chrVIII	86148	86310	162	0,039893848	0,092646739	0,300993446	-0,521545394	-0,443313585	-0,298499431	-0,424499431	**-1,684499431**
chrVIII	86176	86324	148	0,284956058	0,181587609	0,610781108	0,281355451	0,239152133	0,172357835	0,144357835	**-1,115642165**
chrVIII	86476	86623	147	0,102584181	0,114881957	0,471724849	-0,070934736	-0,060294526	-0,130195094	-0,151195094	**-1,411195094**
chrVIII	88963	89116	153	0,159575393	0,448410219	0,262465739	-0,635762125	-0,540397806	-0,603932052	-0,666932052	**-1,926932052**
chrVIII	91372	91534	162	0,113982423	0,218646305	0,342671614	-0,405182689	-0,344405286	-0,207772132	-0,333772132	**-1,593772132**
chrVIII	91372	91534	162	0,113982423	0,218646305	0,342671614	-0,405182689	-0,344405286	-0,207772132	-0,333772132	**-1,593772132**
chrVIII	94804	94953	149	0,19377012	0,100058479	0,659466508	0,41100757	0,349356434	0,229059008	0,194059008	**-1,065940992**
chrVIII	94985	95141	156	0,076938136	0,085235	0,474419732	-0,064164224	-0,05453959	-0,053346819	-0,137346819	**-1,397346819**
chrVIII	96353	96506	153	0,752283994	0,466939567	0,617018911	0,297660652	0,253011555	0,186925542	0,123925542	**-1,136074458**
chrVIII	100385	100534	149	0,037044288	0,074117392	0,333246923	-0,430964963	-0,366320218	-0,503941269	-0,538941269	**-1,798941269**
chrVIII	107252	107412	160	0,028495606	0,044470435	0,390532439	-0,277931585	-0,236241848	-0,146043361	-0,258043361	**-1,518043361**
chrVIII	108345	108493	148	0,09688506	0,140823044	0,407579962	-0,23377466	-0,198708461	-0,274329229	-0,302329229	**-1,562329229**
chrVIII	109527	109701	174	0,24506221	0,470645436	0,342405466	-0,405907004	-0,345020953	-0,096944703	-0,306944703	**-1,566944703**
chrVIII	114779	114932	153	1,638497336	1,093231526	0,59980233	0,252835492	0,214910168	0,16095662	0,09795662	**-1,16204338**
chrVIII	115531	115709	178	0,082637257	0,129705435	0,3891693	-0,28148478	-0,239262063	0,043980934	-0,194019066	**-1,454019066**
chrVIII	121476	121637	161	0,812124766	0,570703915	0,587292394	0,220585474	0,187497653	0,278590182	0,159590182	**-1,100409818**
chrVIII	122292	122451	159	0,091185939	0,037058696	0,711031218	0,556399818	0,472939845	0,573935886	0,468935886	**-0,791064114**
chrVIII	123820	123978	158	0,453080133	0,563292176	0,445781658	-0,136326323	-0,115877374	-0,052352592	-0,150352592	**-1,410352592**
chrVIII	125638	125758	120	0,247911771	0,374292827	0,398440917	-0,257384672	-0,218776971	0,314988238	0,482988238	**-0,777011762**
chrVIII	130844	131002	158	2,122922635	2,045640007	0,509269697	0,023237776	0,01975211	0,082472461	-0,015527539	**-1,275527539**
chrVIII	131196	131345	149	0,681044979	0,337234132	0,668819553	0,436655929	0,37115754	0,252654129	0,217654129	**-1,042345871**
chrVIII	142630	142776	146	0,869115978	0,667056524	0,565767176	0,165607749	0,140766587	0,139401799	0,125401799	**-1,134598201**
chrVIII	144193	144334	141	2,564604525	1,801052615	0,587449825	0,220989833	0,187841358	0,326286935	0,347286935	**-0,912713065**
chrVIII	152201	152353	152	0,082637257	0,096352609	0,461686791	-0,096185077	-0,081757315	-0,146538567	-0,202538567	**-1,462538567**
chrVIII	153827	153949	122	0,387540239	2,334697835	0,142360892	-1,069772367	-0,909306512	-0,310468334	-0,156468334	**-1,416468334**
chrVIII	157804	157963	159	0,162424953	0,107470218	0,601807556	0,258028531	0,219324251	0,325196433	0,220196433	**-1,039803567**
chrVIII	158390	158542	152	0,250761331	0,163058261	0,605967759	0,268824834	0,228501109	0,172034719	0,116034719	**-1,143965281**
chrVIII	162439	162593	154	1,647046017	1,100643265	0,599429502	0,251870714	0,214090107	0,207284304	0,137284304	**-1,122715696**

Chrom	Start	End	Length	Section A	Section B	A/A+B	Z-score	Z * 0.85	Phase correction	Length correction	ΔLKnuc
chrVIII	162949	163087	138	0,401788042	0,240881523	0,625186043	0,319130028	0,271260524	0,445857169	0,487857169	**-0,772142831**
chrVIII	165932	166076	144	0,453080133	0,381704567	0,542750883	0,107366495	0,091261521	0,170537298	0,170537298	**-1,089462702**
chrVIII	167959	168110	151	1,159771157	1,111760874	0,51056782	0,026492695	0,022518791	-0,067459633	-0,116459633	**-1,376459633**
chrVIII	171020	171171	151	0,105433742	0,055588044	0,654779361	0,398256287	0,338517844	0,244739505	0,195739505	**-1,064260495**
chrVIII	171703	171855	152	0,393239361	0,333528262	0,541079911	0,103154718	0,08768151	0,036875132	-0,019124868	**-1,279124868**
chrVIII	174446	174586	140	0,108283302	1,982640225	0,051787309	-1,627765505	-1,383600679	-1,251796618	-1,223796618	**-2,483796618**
chrVIII	174447	174533	86	0,037044288	0,952408482	0,037439167	-1,781208876	-1,514027544	-0,903935898	-0,497935898	**-1,757935898**
chrVIII	174447	174535	88	0,045592969	0,496586524	0,084092021	-1,378062339	-1,171352988	-0,535734994	-0,143734994	**-1,403734994**
chrVIII	174447	174538	91	0,09688506	0,607762611	0,137494331	-1,091646154	-0,927899231	-0,246103149	0,124896851	**-1,135103149**
chrVIII	174447	174540	93	0,045592969	0,396528045	0,10312328	-1,263953936	-1,074360846	-0,431705523	-0,074705523	**-1,334705523**
chrVIII	174447	174541	94	0,04844253	0,97093783	0,047521545	-1,669374972	-1,418968726	-0,778759971	-0,428759971	**-1,688759971**
chrVIII	174447	174544	97	0,031345166	0,87829109	0,034459012	-1,818956785	-1,546113267	-0,917430106	-0,588430106	**-1,848430106**
chrVIII	174447	174545	98	0,039893848	1,271113265	0,030429925	-1,874512288	-1,593335445	-0,948644163	-0,626644163	**-1,886644163**
chrVIII	174447	174548	101	0,034194727	0,314998914	0,097924827	-1,293466814	-1,099446792	-0,408309311	-0,107309311	**-1,367309311**
chrVIII	174447	174550	103	0,031345166	0,270528479	0,103835386	-1,259996094	-1,07099668	-0,364354548	-0,077354548	**-1,337354548**
chrVIII	174447	174551	104	0,034194727	0,359469349	0,086862706	-1,360330337	-1,156280786	-0,449594893	-0,169594893	**-1,429594893**
chrVIII	174447	174552	105	0,059840772	0,44099848	0,119480995	-1,177585257	-1,000947468	-0,35218531	-0,07918531	**-1,33918531**
chrVIII	174447	174553	106	0,142478029	9,661201991	0,014533117	-2,182587053	-1,855198995	-1,218746809	-0,952746809	**-2,212746809**
chrVIII	174447	174555	108	0,039893848	0,34835174	0,102754157	-1,266013318	-1,076111321	-0,385899438	-0,133899438	**-1,393899438**
chrVIII	174447	174561	114	0,042743409	0,444704349	0,087688184	-1,35512929	-1,151859897	-0,700334161	-0,490334161	**-1,750334161**
chrVIII	174447	174565	118	0,051292091	0,570703915	0,082463698	-1,38868882	-1,180385497	-0,677634194	-0,495634194	**-1,755634194**
chrVIII	174447	174566	119	0,056991212	0,418763262	0,119791226	-1,176031092	-0,999626428	-0,498491617	-0,323491617	**-1,583491617**
chrVIII	174447	174567	120	0,085486818	0,574409785	0,129545776	-1,128540904	-0,959259768	-0,427140194	-0,259140194	**-1,519140194**
chrVIII	174447	174568	121	0,065539893	0,43729261	0,130341402	-1,124778745	-0,956061933	-0,40579122	-0,24479122	**-1,50479122**
chrVIII	174447	174569	122	0,088336378	0,318704784	0,21702075	-0,782294532	-0,664950353	-0,066773356	0,087226644	**-1,172773356**
chrVIII	174447	174570	123	0,085486818	0,377998697	0,184443343	-0,898560728	-0,763776618	-0,192586702	-0,045586702	**-1,305586702**
chrVIII	174447	174571	124	0,182371877	0,507704132	0,264277956	-0,630211965	-0,53568017	0,033754057	0,173754057	**-1,086245943**
chrVIII	174447	174572	125	0,111132863	0,678174133	0,140798021	-1,076740885	-0,915229752	-0,389021409	-0,256021409	**-1,516021409**
chrVIII	174447	174573	126	0,065539893	0,270528479	0,195019522	-0,85954656	-0,730614576	-0,205498373	-0,079498373	**-1,339498373**
chrVIII	174447	174574	127	0,094035499	0,61888022	0,131902687	-1,117442025	-0,949825721	-0,40227693	-0,28327693	**-1,54327693**
chrVIII	174447	174575	128	0,09688506	0,433586741	0,182639416	-0,905352197	-0,769549367	-0,281042701	-0,169042701	**-1,429042701**
chrVIII	174447	174576	129	0,250761331	0,733762177	0,254703244	-0,659762131	-0,560797812	-0,078539464	0,026460536	**-1,233539464**
chrVIII	174447	174577	130	0,444531451	1,096937395	0,288381729	-0,558118516	-0,474400738	-0,034052957	0,063947043	**-1,196052957**
chrVIII	174447	174578	131	0,239363089	0,604056741	0,283800642	-0,571587769	-0,485849604	-0,089911953	0,001088047	**-1,258911953**
chrVIII	174447	174579	132	0,273557816	0,641115437	0,299077091	-0,527056746	-0,447998234	-0,066932972	0,017067028	**-1,242932972**
chrVIII	174447	174580	133	0,284956058	0,778232612	0,268020217	-0,618811668	-0,525989918	-0,189127814	-0,112127814	**-1,372127814**
chrVIII	174447	174581	134	0,521469587	1,304466092	0,285590338	-0,566313514	-0,481366487	-0,196338066	-0,126338066	**-1,386338066**
chrVIII	174447	174582	135	0,834921251	1,40452457	0,372824939	-0,324380637	-0,275723541	-0,041892414	0,021107586	**-1,238892414**
chrVIII	174447	174583	136	0,481575739	0,959820221	0,334103711	-0,428609513	-0,364318086	-0,174172993	-0,118172993	**-1,378172993**
chrVIII	174447	174584	137	0,624053768	1,58240631	0,282830301	-0,574454027	-0,488285923	-0,363889836	-0,314889836	**-1,574889836**
chrVIII	174447	174585	138	0,367593315	1,430465657	0,204438965	-0,825869842	-0,701989366	-0,529809058	-0,487809058	**-1,747809058**
chrVIII	174447	174586	139	409,06582	529,931938	0,435640891	-0,162030568	-0,137725983	0,01363651	0,04863651	**-1,21136349**
chrVIII	174447	174587	140	0,128230226	0,496586524	0,205228535	-0,823089551	-0,699626119	-0,561791499	-0,533791499	**-1,793791499**
chrVIII	174448	174586	138	0,435982769	1,356348266	0,243249021	-0,695889487	-0,591506064	-0,434348415	-0,392348415	**-1,652348415**
chrVIII	174449	174586	137	0,53571739	1,489759571	0,264489501	-0,629565348	-0,535130546	-0,388178604	-0,339178604	**-1,599178604**
chrVIII	174450	174586	136	0,624053768	1,808464354	0,2565464	-0,654029499	-0,555925074	-0,383297859	-0,327297859	**-1,587297859**
chrVIII	174451	174586	135	0,97739928	2,01969892	0,3261152	-0,450665847	-0,38306597	-0,161499898	-0,098499898	**-1,358499898**
chrVIII	174452	174586	134	0,638301571	1,927052181	0,248816199	-0,678219707	-0,576486751	-0,296654464	-0,226654464	**-1,486654464**
chrVIII	174453	174586	133	0,265009134	1,371171744	0,161968116	-0,986401291	-0,838441097	-0,515119925	-0,438119925	**-1,698119925**
chrVIII	174454	174586	132	0,213717044	1,715817615	0,110760925	-1,22249141	-1,039117699	-0,67614847	-0,59214847	**-1,85214847**
chrVIII	174455	174586	131	0,304902982	0,730056307	0,294603841	-0,539984556	-0,458986873	-0,075822515	0,015177485	**-1,244822515**
chrVIII	174456	174586	130	0,19661968	0,755997394	0,206399492	-0,818977968	-0,696131272	-0,252295965	-0,154295965	**-1,414295965**
chrVIII	174457	174586	129	0,227964847	1,026525873	0,181719038	-0,908833395	-0,772508386	-0,303023269	-0,198023269	**-1,458023269**
chrVIII	174458	174586	128	0,230814407	0,581821524	0,284031752	-0,570905793	-0,485269924	-0,00752345	0,10447655	**-1,15552345**
chrVIII	174459	174586	127	0,14817715	0,900526308	0,141295568	-1,074516764	-0,91333925	-0,359069553	-0,240069553	**-1,500069553**
chrVIII	174460	174586	126	0,199469241	1,041349352	0,160756167	-0,99135486	-0,842651631	-0,306462389	-0,180462389	**-1,440462389**
chrVIII	174461	174586	125	0,131079787	2,20128653	0,056200343	-1,587494526	-1,349370347	-0,808941559	-0,675941559	**-1,935941559**
chrVIII	174462	174553	91	1,148372915	1,374877614	0,455116486	-0,112744686	-0,095832983	0,591042643	0,962042643	**-0,297957357**
chrVIII	174467	174586	119	0,037044288	0,907938047	0,039201037	-1,760033809	-1,496028738	-1,006322845	-0,831322845	**-2,091322845**
chrVIII	174928	175084	156	0,028495606	0,059293913	0,324590006	-0,454901459	-0,386666241	-0,381251745	-0,465251745	**-1,725251745**
chrVIII	176211	176366	155	11,64330454	5,566216107	0,676561816	0,458105889	0,389390006	0,414519494	0,337519494	**-0,922480506**
chrVIII	177719	177867	148	0,97454972	0,670762394	0,592319057	0,233514533	0,198487353	0,12204331	0,09404331	**-1,16595669**
chrVIII	177797	177947	150	2,00039153	1,208113483	0,623465297	0,314594696	0,267405492	0,167756456	0,125756456	**-1,134243544**
chrVIII	178837	178985	148	2,04883406	1,05246696	0,660636954	0,414202117	0,352071799	0,272900181	0,244900181	**-1,015099819**
chrVIII	180375	180509	134	1,5615592	1,14140783	0,577720402	0,196065121	0,166655353	0,434974991	0,504974991	**-0,755025009**
chrVIII	190177	190319	142	0,404637603	0,485468915	0,454594585	-0,114061338	-0,096952138	-0,002374651	0,011625349	**-1,248374651**
chrVIII	194784	194941	157	0,065539893	0,133411305	0,329426985	-0,441495981	-0,375271584	-0,354812859	-0,445812859	**-1,705812859**
chrVIII	194987	195122	135	0,105433742	0,114881957	0,478557553	-0,053774149	-0,045708026	0,183974627	0,246974627	**-1,013025373**
chrVIII	205654	205804	150	0,256460453	0,214940436	0,544038968	0,11061448	0,094022308	-0,00516269	-0,04716269	**-1,30716269**
chrVIII	207083	207225	142	1,65844426	1,078408047	0,605967759	0,268824834	0,228501109	0,320200177	0,334200177	**-0,925799823**
chrVIII	212526	212683	157	1,188266763	1,05246696	0,53030253	0,076030366	0,064625811	0,073793991	-0,017206009	**-1,277206009**
chrVIII	212729	212874	145	0,054141651	0,059293913	0,477289917	-0,056956516	-0,048413039	-0,033209905	-0,040209905	**-1,300209905**
chrVIII	212963	213069	106	0,056991212	0,459527828	0,110337098	-1,224737354	-1,041026751	-0,416910171	-0,150910171	**-1,410910171**
chrVIII	212963	213076	113	0,054141651	0,507704132	0,0963639	-1,302551864	-1,107169084	-0,604861438	-0,387861438	**-1,647861438**
chrVIII	213025	213187	162	0,19661968	0,218646305	0,473478896	-0,066527591	-0,056548453	0,081124089	-0,044875911	**-1,304875911**
chrVIII	214079	214248	169	0,037044288	0,040764565	0,47609348	-0,059960669	-0,050966568	0,10129583	-0,07370417	**-1,33370417**
chrVIII	214176	214329	153	0,265009134	0,374292827	0,414528893	-0,21591015	-0,183523627	-0,23838711	-0,30138711	**-1,56138711**
chrVIII	214524	214681	157	1,22246149	1,115466743	0,522882385	0,057389119	0,048780751	0,070428953	-0,020571047	**-1,280571047**
chrVIII	214727	214872	145	0,079787696	0,055588044	0,589379577	0,225949308	0,192056912	0,2059234	0,1989234	**-1,0610766**
chrVIII	214961	215067	106	0,04844253	0,411351523	0,105357017	-1,251604445	-1,063863778	-0,419393278	-0,153393278	**-1,413393278**
chrVIII	214961	215074	113	0,062690333	0,578115654	0,097830442	-1,294013136	-1,099911165	-0,595190833	-0,378190833	**-1,638190833**
chrVIII	215023	215185	162	0,179522317	0,188999349	0,487141826	-0,032236246	-0,027400809	0,110499756	-0,015500244	**-1,275500244**
chrVIII	216077	216246	169	0,034194727	0,062999783	0,351817475	-0,380418277	-0,323355536	-0,176889619	-0,351889619	**-1,611889619**
chrVIII	217808	217962	154	0,302053422	0,259410871	0,537974411	0,095331934	0,081032144	0,077450723	0,007450723	**-1,252549277**
chrVIII	218430	218569	139	0,14817715	0,34835174	0,298426039	-0,528932784	-0,449592866	-0,294807445	-0,259807445	**-1,519807445**
chrVIII	219279	219430	151	0,569912117	0,422469132	0,57428747	0,187300463	0,159205394	0,063875473	0,014875473	**-1,245124527**
chrVIII	223655	223760	105	1,162620718	10,10961221	0,103140232	-1,263859491	-1,074280567	-0,442807162	-0,169807162	**-1,429807162**
chrVIII	226108	226256	148	0,267858695	0,044470435	0,857616755	1,069673076	0,909222114	0,798670411	0,770670411	**-0,489329589**
chrVIII	227838	227999	161	0,113982423	0,17417587	0,395554894	-0,264869768	-0,225139303	-0,136272845	-0,255272845	**-1,515272845**
chrVIII	227838	227999	161	0,113982423	0,17417587	0,395554894	-0,264869768	-0,225139303	-0,136272845	-0,255272845	**-1,515272845**
chrVIII	228803	228946	143	0,225115286	0,17788174	0,558602847	0,147427874	0,125313693	0,193942271	0,200942271	**-1,059057729**
chrVIII	234383	234536	153	0,219416165	0,196411088	0,52766182	0,069393554	0,058984521	-0,011466065	-0,074466065	**-1,334466065**
chrVIII	234544	234693	149	0,678195419	0,489174784	0,580960022	0,204350056	0,173697548	0,055903487	0,020903487	**-1,239096513**
chrVIII	240775	240923	148	1,057186976	0,730056307	0,591518226	0,231452159	0,196734335	0,090268591	0,062268591	**-1,197731409**
chrVIII	250570	250724	154	0,501522663	0,26311674	0,655894348	0,401283645	0,341091098	0,342704377	0,272704377	**-0,987295623**
chrVIII	256594	256759	165	0,034194727	0,070411522	0,326889907	-0,448517424	-0,381239811	-0,220989876	-0,367989876	**-1,627989876**

Chrom	Start	End	Length	Section A	Section B	A/A+B	Z-score	Z * 0.85	Phase correction	Length correction	ΔLKnuc
chrVIII	258714	258856	142	0,504372223	0,377998697	0,571610206	0,180475116	0,153403848	0,254374304	0,268374304	**-0,991625696**
chrVIII	261335	261482	147	0,279256937	0,240881523	0,536889615	0,092600721	0,078710613	0,011523758	-0,009476242	**-1,269476242**
chrVIII	264809	264956	147	0,356195073	0,226058044	0,611752969	0,283890806	0,241307185	0,16766423	0,14666423	**-1,11333577**
chrVIII	270516	270670	154	0,219416165	0,248293262	0,469129234	-0,077458923	-0,065840085	-0,07332622	-0,14332622	**-1,40332622**
chrVIII	272692	272812	120	9,480488061	21,07898616	0,310230732	-0,495196439	-0,420916973	0,126508326	0,294508326	**-0,965491674**
chrVIII	275159	275317	158	0,182371877	0,144528914	0,55788142	0,145600009	0,123760008	0,191190839	0,093190839	**-1,166809161**
chrVIII	281596	281745	149	1,36778908	0,941290873	0,592352412	0,233600453	0,198560385	0,06919785	0,03419785	**-1,22580215**
chrVIII	288944	289050	106	0,031345166	0,096352609	0,245463684	-0,688834586	-0,585509398	0,061082463	0,327082463	**-0,932917537**
chrVIII	291607	291756	149	0,202318801	0,096352609	0,677395942	0,460429288	0,391364895	0,262472444	0,227472444	**-1,032527556**
chrVIII	291938	292090	152	1,470373261	1,05246696	0,582824568	0,209124736	0,177756026	0,139654792	0,083654792	**-1,176345208**
chrVIII	294718	294863	145	0,250761331	0,181587609	0,579997562	0,201887241	0,171604155	0,181380176	0,174380176	**-1,085619824**
chrVIII	297474	297608	134	0,860567296	1,030231743	0,455134194	-0,112700017	-0,095795015	0,167780066	0,237780066	**-1,022219934**
chrVIII	297952	298079	127	0,227964847	0,329822392	0,408694984	-0,230903248	-0,196267761	0,348948915	0,467948915	**-0,792051085**
chrVIII	299058	299223	165	0,185221438	0,211234566	0,46719292	-0,082328063	-0,069978854	0,071191272	-0,075808728	**-1,335808728**
chrVIII	299773	299910	137	2,348037921	1,949287398	0,546395199	0,116558907	0,099075071	0,249105497	0,298105497	**-0,961894503**
chrVIII	310121	310260	139	0,091185939	0,040764565	0,691061693	0,498861988	0,42403269	0,565599023	0,600599023	**-0,659400977**
chrVIII	310839	310998	159	0,333398588	0,422469132	0,441080601	-0,14823007	-0,125995559	-0,039089165	-0,144089165	**-1,404089165**
chrVIII	311969	312130	161	0,393239361	0,411351523	0,488744489	-0,028217126	-0,023984557	0,072509865	-0,046490135	**-1,306490135**
chrVIII	333005	333157	152	0,592708601	0,396528045	0,599157546	0,251167115	0,213492048	0,145014874	0,089014874	**-1,170985126**
chrVIII	334511	334659	148	0,461628815	0,337234132	0,577857337	0,196415039	0,166952783	0,049415472	0,021415472	**-1,238584528**
chrVIII	341021	341173	152	0,054141651	0,08894087	0,378394585	-0,309699913	-0,263244926	-0,328478449	-0,384478449	**-1,644478449**
chrVIII	341021	341178	157	3,382428413	3,346400229	0,502677151	0,006710673	0,005704072	0,038610573	-0,052389427	**-1,312389427**
chrVIII	342001	342158	157	2,852410144	2,360638922	0,547167331	0,118507771	0,100731606	0,142700054	0,051700054	**-1,208299946**
chrVIII	342217	342368	151	0,151026711	0,17417587	0,464408094	-0,089334359	-0,075934205	-0,171422533	-0,220422533	**-1,480422533**
chrVIII	343838	343999	161	0,433133209	0,496586524	0,465875031	-0,085643193	-0,072796714	0,048423088	-0,070576912	**-1,330576912**
chrVIII	345951	346111	160	0,037044288	0,062999783	0,370279692	-0,331112667	-0,281445767	-0,192221048	-0,304221048	**-1,564221048**
chrVIII	346434	346582	148	0,09973462	0,159352392	0,38494643	-0,292515043	-0,248637787	-0,374165903	-0,402165903	**-1,662165903**
chrVIII	350191	350346	155	0,786478721	0,904232177	0,465176348	-0,087401099	-0,074290934	-0,049031371	-0,126031371	**-1,386031371**
chrVIII	353263	353415	152	0,085486818	0,111176087	0,434687048	-0,164453569	-0,139785534	-0,206945572	-0,262945572	**-1,522945572**
chrVIII	354917	355076	159	0,051292091	0,085235	0,375691668	-0,31681585	-0,269293473	-0,183652808	-0,288652808	**-1,548652808**
chrVIII	355941	356089	148	3,915296242	2,712696532	0,59072126	0,229400715	0,194990608	0,07923795	0,05123795	**-1,20876205**
chrVIII	357549	357703	154	2,040285378	1,1451137	0,6405117	0,35982688	0,305852848	0,290343432	0,220343432	**-1,039656568**
chrVIII	360834	360982	148	0,453080133	0,522527611	0,464408094	-0,089334359	-0,075934205	-0,170143089	-0,198143089	**-1,458143089**
chrVIII	364130	364290	160	0,045592969	0,044470435	0,506231912	0,015621722	0,013278464	0,100653733	-0,011346267	**-1,271346267**
chrVIII	370648	370829	181	0,074088575	0,107470218	0,408069331	-0,232514197	-0,197637067	0,095693482	-0,163306518	**-1,423306518**
chrVIII	371736	371883	147	0,031345166	0,040764565	0,434687048	-0,164453569	-0,139785534	-0,223276641	-0,244276641	**-1,504276641**
chrVIII	374061	374221	160	6,132254376	5,506922194	0,526863248	0,067387144	0,057279073	0,147601807	0,035601807	**-1,224398193**
chrVIII	374061	374221	160	6,132254376	5,506922194	0,526863248	0,067387144	0,057279073	0,147601807	0,035601807	**-1,224398193**
chrVIII	374061	374255	194	0,056991212	0,070411522	0,447331152	-0,132407093	-0,112546029	0,237900122	-0,112099878	**-1,372099878**
chrVIII	374061	374255	194	0,056991212	0,070411522	0,447331152	-0,132407093	-0,112546029	0,237900122	-0,112099878	**-1,372099878**
chrVIII	374122	374209	87	0,068389454	0,062999783	0,520510322	0,051434423	0,043719259	0,672040791	1,071040791	**-0,188959209**
chrVIII	374122	374221	99	0,262159574	0,274234349	0,488744489	-0,028217126	-0,023984557	0,67632298	0,99132298	**-0,26867702**
chrVIII	374122	374255	133	1,396284686	1,652817832	0,457933007	-0,105642485	-0,089796113	0,228613066	0,305613066	**-0,954386934**
chrVIII	376459	376622	163	0,185221438	0,237175653	0,438500742	-0,154771469	-0,131555749	0,025805197	-0,107194803	**-1,367194803**
chrVIII	376459	376622	163	0,185221438	0,237175653	0,438500742	-0,154771469	-0,131555749	0,025805197	-0,107194803	**-1,367194803**
chrVIII	381490	381641	151	0,039893848	0,055588044	0,417815854	-0,20748423	-0,176361595	-0,282911886	-0,331911886	**-1,591911886**
chrVIII	382542	382702	160	0,128230226	0,170470001	0,429294037	-0,178171777	-0,15144601	-0,064106299	-0,176106299	**-1,436106299**
chrVIII	382976	383151	175	0,094035499	0,103764348	0,47540734	-0,061683752	-0,052431189	0,230660011	0,013660011	**-1,246339989**
chrVIII	399609	399757	148	0,19377012	0,118587827	0,620346375	0,30639062	0,260432027	0,168686882	0,140686882	**-1,119313118**
chrVIII	402529	402689	160	0,173823196	0,185293479	0,484029865	-0,040041889	-0,034035606	0,053157064	-0,058842936	**-1,318842936**
chrVIII	402529	402689	160	0,173823196	0,185293479	0,484029865	-0,040041889	-0,034035606	0,053157064	-0,058842936	**-1,318842936**
chrVIII	403584	403737	153	0,421734966	0,389116306	0,520113837	0,050439292	0,042873398	-0,002040433	-0,065040433	**-1,325040433**
chrVIII	404097	404230	133	0,302053422	0,377998697	0,444162166	-0,140424842	-0,119361116	0,19363348	0,27063348	**-0,98936652**
chrVIII	414157	414333	176	0,085486818	0,166764131	0,338895921	-0,415478239	-0,353156503	-0,075454807	-0,299454807	**-1,559454807**
chrVIII	414157	414333	176	0,085486818	0,166764131	0,338895921	-0,415478239	-0,353156503	-0,075454807	-0,299454807	**-1,559454807**
chrVIII	416537	416675	138	5,864395681	3,794810448	0,607130193	0,271847066	0,231070006	0,389508758	0,431508758	**-0,828491242**
chrVIII	424973	425127	154	0,182371877	0,133411305	0,577522451	0,195559327	0,166225428	0,159062132	0,089062132	**-1,170937868**
chrVIII	424973	425127	154	0,182371877	0,133411305	0,577522451	0,195559327	0,166225428	0,159062132	0,089062132	**-1,170937868**
chrVIII	427961	428122	161	0,056991212	0,062999783	0,474962408	-0,062801193	-0,053381014	0,081986683	-0,037013317	**-1,297013317**
chrVIII	431042	431168	126	0,082637257	0,100058479	0,452321762	-0,11979755	-0,101827917	0,422001132	0,548001132	**-0,711998868**
chrVIII	433338	433488	150	0,068389454	0,059293913	0,535617563	0,08939893	0,07598909	-0,026091826	-0,068091826	**-1,328091826**
chrVIII	439467	439624	157	0,364743755	0,259410871	0,58438044	0,213112623	0,181145729	0,231309493	0,140309493	**-1,119690507**
chrVIII	444830	444988	158	1,003045325	1,352642396	0,425797238	-0,187084379	-0,159021722	-0,061465552	-0,159465552	**-1,419465552**
chrVIII	447873	448000	127	0,344796831	1,030231743	0,25075612	-0,672112246	-0,571295409	-0,032966999	0,086033001	**-1,173966999**
chrVIII	456058	456162	104	0,094035499	1,371171744	0,064178975	-1,520609146	-1,292517774	-0,579025913	-0,299025913	**-1,559025913**
chrVIII	465568	465728	160	0,746584873	0,681880002	0,522648394	0,056801634	0,048281389	0,130321915	0,018321915	**-1,241678085**
chrVIII	469779	469934	155	0,062690333	0,081529131	0,434687048	-0,164453569	-0,139785534	-0,124060373	-0,201060373	**-1,461060373**
chrVIII	469928	470081	153	0,225115286	0,144528914	0,609005325	0,276727507	0,235218381	0,197310974	0,134310974	**-1,125689026**
chrVIII	469928	470081	153	0,225115286	0,144528914	0,609005325	0,276727507	0,235218381	0,197310974	0,134310974	**-1,125689026**
chrVIII	470995	471124	129	1,433328974	1,560171093	0,478813744	-0,053131056	-0,045161397	0,42932635	0,53432635	**-0,72567365**
chrVIII	482780	482943	163	2,023188014	1,715817615	0,541103228	0,103213476	0,087731455	0,241427544	0,108427544	**-1,151572456**
chrVIII	485160	485318	158	0,652549374	0,418763262	0,609111992	0,277005326	0,235454527	0,326465982	0,228465982	**-1,031534018**
chrVIII	485160	485318	158	0,652549374	0,418763262	0,609111992	0,277005326	0,235454527	0,326465982	0,228465982	**-1,031534018**
chrVIII	495830	495995	165	0,253610892	0,289057827	0,467340171	-0,081957711	-0,069664054	0,085022425	-0,061977575	**-1,321977575**
chrVIII	497750	497909	159	0,131079787	0,133411305	0,495592445	-0,011048326	-0,009391077	0,077665771	-0,027334229	**-1,287334229**
chrVIII	501432	501582	150	0,139628469	0,196411088	0,415512001	-0,213388432	-0,181380167	-0,281211409	-0,323211409	**-1,583211409**
chrVIII	503721	503876	155	0,92895675	0,537351089	0,6335346	0,341229522	0,290045094	0,30620918	0,22920918	**-1,03079082**
chrVIII	510274	510423	149	0,14817715	0,111176087	0,571333335	0,179769754	0,152804291	0,030477201	-0,004522799	**-1,264522799**
chrVIII	518475	518638	163	2,883755311	2,482932618	0,537343581	0,093743597	0,079682057	0,218661863	0,085661863	**-1,174338137**
chrVIII	518475	518638	163	2,883755311	2,482932618	0,537343581	0,093743597	0,079682057	0,218661863	0,085661863	**-1,174338137**
chrVIII	521779	521913	134	1,134125112	1,66764131	0,404789315	-0,240969661	-0,204824212	0,038768173	0,108768173	**-1,151231827**
chrVIII	522442	522595	153	0,333398588	0,314998914	0,514188576	0,035572986	0,030237038	0,003486603	-0,059513397	**-1,319513397**
chrVIII	523030	523182	152	0,48727486	0,292763697	0,62468048	0,31779685	0,270127323	0,209105386	0,153105386	**-1,106894614**
chrVIII	523197	523325	128	0,917558508	1,560171093	0,370322293	-0,330999866	-0,281349886	0,208982526	0,320982526	**-0,939017474**
chrVIII	525186	525342	156	0,142478029	0,133411305	0,516431814	0,041200102	0,035020087	0,053047378	-0,030952622	**-1,290952622**
chrVIII	526030	526198	168	0,168124074	0,118587827	0,586386801	0,218260181	0,185521153	0,342288972	0,174288972	**-1,085711028**
chrVIII	526169	526320	151	2,849560584	1,482347831	0,657807209	0,406485945	0,345513053	0,248331216	0,199331216	**-1,060668784**
chrVIII	526293	526441	148	5,200448065	3,60210523	0,590788592	0,229573995	0,195137895	0,086484539	0,058484539	**-1,201515461**
chrVIII	526293	526481	188	0,042743409	0,044470435	0,490098899	-0,024820929	-0,021097789	0,313288137	0,005288137	**-1,254711863**
chrVIII	526600	526706	106	0,054141651	0,092646739	0,368841506	-0,334923211	-0,284684729	0,356354326	0,622354326	**-0,637645674**
chrVIII	526600	526743	143	0,076938136	0,096352609	0,443982948	-0,140878539	-0,119746758	-0,042753797	-0,035753797	**-1,295753797**
chrVIII	526600	526749	149	0,074088575	0,051882174	0,588141101	0,222765786	0,189350918	0,089191411	0,054191411	**-1,205808589**
chrVIII	526600	526751	151	4,830005189	2,99434262	0,61730451	0,298409053	0,253647695	0,155853097	0,106853097	**-1,153146903**
chrVIII	526624	526751	127	0,09688506	0,100058479	0,491943329	-0,020196453	-0,017166985	0,495579096	0,614579096	**-0,645420904**
chrVIII	526651	526751	100	0,111132863	0,092646739	0,54535813	0,113942039	0,096850733	0,824320311	1,132320311	**-0,127679689**
chrVIII	527864	528038	174	0,49012442	0,87829109	0,358169296	-0,3633565	-0,308853025	-0,048700927	-0,258700927	**-1,518700927**

Chrom	Start	End	Length	Section A	Section B	A/A+B	Z-score	Z * 0.85	Phase correction	Length correction	ΔLKnuc
chrVIII	536044	536157	113	0,054141651	1,004290656	0,051152682	-1,633778522	-1,388711744	-0,92602132	-0,70902132	-1,96902132
chrVIII	538480	538617	137	5,089315202	3,457576317	0,595458032	0,241607982	0,205366785	0,358582012	0,407582012	-0,852417988
chrVIII	539313	539458	145	2,305294512	1,356348266	0,629579304	0,33073933	0,28112843	0,27798331	0,27098331	-0,98901669
chrVIII	539340	539488	148	0,427434088	0,222352175	0,657807209	0,406485945	0,345513053	0,232270157	0,204270157	-1,055729843
chrVIII	542782	542926	144	1,923453394	1,378583483	0,582505122	0,208306373	0,177060417	0,236907971	0,236907971	-1,023092029
chrVIII	542782	542926	144	1,923453394	1,378583483	0,582505122	0,208306373	0,177060417	0,236907971	0,236907971	-1,023092029
chrVIII	542989	543156	167	0,473027057	0,563292176	0,456449173	-0,109383467	-0,092975947	0,066404914	-0,094595086	-1,354595086
chrVIII	543353	543457	104	0,142478029	0,715232829	0,166114289	-0,969634765	-0,82418955	-0,132766739	0,147233261	-1,112766739
chrVIII	543780	543928	148	0,028495606	0,044470435	0,390532439	-0,277931585	-0,236241848	-0,353852386	-0,381852386	-1,641852386
chrVIII	544540	544654	114	0,065539893	0,474351306	0,121394632	-1,168043388	-0,99283688	-0,534603356	-0,324603356	-1,584603356
chrVIII	546632	546789	157	0,042743409	0,048176305	0,470122564	-0,074961771	-0,063717505	-0,01663172	-0,10763172	-1,36763172
chrVIII	546818	546968	150	8,981814959	4,031986102	0,690176138	0,496349676	0,421897225	0,312624047	0,270624047	-0,989375953
chrVIII	546822	546968	146	0,056991212	0,048176305	0,541908887	0,105243965	0,08945737	0,092381253	0,078381253	-1,181618747
chrVIII	547280	547415	135	0,085486818	0,059293913	0,590457149	0,22872109	0,194412926	0,427683275	0,490683275	-0,769316725
chrVIII	548318	548458	140	0,139628469	0,129705435	0,518421434	0,046192108	0,039263292	0,16143811	0,18943811	-1,07056189
chrVIII	548324	548458	134	0,139628469	0,125999566	0,525654112	0,064349707	0,054697251	0,292030714	0,362030714	-0,897969286
chrVIII	548850	549005	155	0,102584181	0,081529131	0,557179598	0,143822286	0,122248943	0,143861795	0,066861795	-1,193138205
chrVIII	548981	549089	108	0,065539893	0,192705218	0,253789483	-0,662612181	-0,563220354	0,09964897	0,35164897	-0,90835103
chrVIII	549893	550056	163	0,19377012	0,200116957	0,491943329	-0,020196453	-0,017166985	0,113683695	-0,019316305	-1,279316305
chrVIII	550702	550810	108	0,09973462	0,277940218	0,264075364	-0,630831465	-0,536206745	0,096643465	0,348643465	-0,911356535
chrVIII	551514	551634	120	0,24221265	0,733762177	0,2481751	-0,680243648	-0,578207101	-0,021007121	0,146992879	-1,113007121
chrVIII	553179	553326	147	1,333594353	0,70411522	0,65445752	0,397383121	0,337775653	0,264992015	0,243992015	-1,016007985
chrVIII	553630	553778	148	0,347646391	0,300175436	0,536638898	0,091969585	0,078174148	-0,044517702	-0,072517702	-1,332517702
chrVIII	556401	556536	135	0,840620372	0,718938698	0,539011563	0,097943858	0,083252279	0,284767935	0,347767935	-0,912232065
chrVIII	556826	556854	28	0,034194727	0,552174567	0,058316026	-1,569068185	-1,333707957		0,812	-0,448
chrVIII	556826	556859	33	0,068389454	0,415057393	0,141462199	-1,073773081	-0,912707119		0,777	-0,483
chrVIII	556826	556865	39	0,042743409	0,515115871	0,076620414	-1,428177319	-1,213950721		0,735	-0,525
chrVIII	556826	556871	45	0,051292091	0,485468915	0,095558526	-1,307281714	-1,111189457		0,693	-0,567
chrVIII	556826	556873	47	0,045592969	0,35576348	0,113597201	-1,207617557	-1,026474924		0,679	-0,581
chrVIII	556826	556876	50	0,04844253	0,518821741	0,085396758	-1,369658539	-1,164209758		0,658	-0,602
chrVIII	556826	556884	58	0,04844253	0,452116089	0,096776937	-1,300137421	-1,105116808		0,602	-0,658
chrVIII	556826	556910	84	0,094035499	0,648527176	0,126636447	-1,142435854	-0,971070476	-0,386387911	0,033612089	-1,226387911
chrVIII	556826	556914	88	0,139628469	0,326116523	0,299795964	-0,524987432	-0,446239318	0,198488658	0,590488658	-0,669511342
chrVIII	556826	556918	92	0,051292091	0,34835174	0,128344507	-1,134251626	-0,964113882	-0,283094785	0,080905215	-1,179094785
chrVIII	556826	556919	93	0,575611238	0,996878917	0,366050774	-0,342331347	-0,290981645	0,348099827	0,705099827	-0,554900173
chrVIII	556826	556921	95	0,068389454	0,188999349	0,265704853	-0,625855528	-0,531977199	0,114882628	0,457882628	-0,802117372
chrVIII	556826	556923	97	0,056991212	0,111176087	0,338895921	-0,415478239	-0,353156503	0,278463196	0,607463196	-0,652536804
chrVIII	556826	556926	100	0,14532759	0,307587175	0,320871831	-0,465262255	-0,395472916	0,320019364	0,628019364	-0,631980636
chrVIII	556826	556930	104	0,068389454	0,300175436	0,185556074	-0,894392081	-0,760233269	-0,079931527	0,200068473	-1,059931527
chrVIII	556826	556935	109	0,151026711	0,17788174	0,459175526	-0,102511037	-0,087134382	0,518483926	0,763483926	-0,496516074
chrVIII	556826	556936	110	0,558513874	0,415057393	0,573675388	0,185739279	0,157878387	0,75138264	0,98938264	-0,27061736
chrVIII	556826	556939	113	0,302053422	0,370586958	0,449056332	-0,12804588	-0,108838998	0,357335394	0,574335394	-0,685664606
chrVIII	556826	556942	116	0,188070999	0,474351306	0,283914049	-0,571253086	-0,485565123	0,024748151	0,220748151	-1,039251849
chrVIII	556826	556944	118	0,059840772	0,222352175	0,212056229	-0,799306935	-0,679410895	-0,202593646	-0,020593646	-1,280593646
chrVIII	556826	556950	124	0,413186285	0,474351306	0,465542292	-0,086480339	-0,073508288	0,483067027	0,623067027	-0,636932973
chrVIII	556826	556954	128	0,04844253	0,289057827	0,14353327	-1,064578883	-0,90489205	-0,412902706	-0,300902706	-1,560902706
chrVIII	556826	556956	130	0,028495606	0,207528696	0,12073166	-1,171337061	-0,995636502	-0,540452052	-0,442452052	-1,702452052
chrVIII	556826	556961	135	0,09688506	0,192705218	0,33455909	-0,427358568	-0,363254783	-0,155952049	-0,092952049	-1,352952049
chrVIII	556826	556963	137	0,168124074	0,170470001	0,496535784	-0,008683612	-0,00738107	0,150892802	0,199892802	-1,060107198
chrVIII	556826	556965	139	0,105433742	0,207528696	0,336889444	-0,4209674	-0,35782229	-0,215694378	-0,180694378	-1,440694378
chrVIII	556826	556967	141	0,125380666	0,222352175	0,360566076	-0,356946107	-0,303404191	-0,184598416	-0,163598416	-1,423598416
chrVIII	556826	556969	143	0,122531105	0,133411305	0,478744828	-0,053304046	-0,045308439	0,044334928	0,051334928	-1,208665072
chrVIII	556826	556970	144	0,051292091	0,244587392	0,173354671	-0,940991251	-0,799842563	-0,75641571	-0,75641571	-2,01641571
chrVIII	556826	556971	145	0,059840772	0,148234783	0,287591554	-0,560434497	-0,476369322	-0,468863071	-0,475863071	-1,735863071
chrVIII	556826	556972	146	0,042743409	0,233469783	0,15474789	-1,016280608	-0,863838517	-0,862743975	-0,876743975	-2,136743975
chrVIII	556826	556973	147	0,826372569	1,222936961	0,403244389	-0,244958225	-0,208214491	-0,278662379	-0,299662379	-1,559662379
chrVIII	556826	556975	149	1,02584181	0,644821307	0,614032715	0,289845327	0,246368528	0,142664434	0,107664434	-1,152335566
chrVIII	556826	556976	150	0,034194727	0,251999131	0,119480995	-1,177585257	-1,000947468	-1,103678773	-1,145678773	-2,405678773
chrVIII	556826	556977	151	0,059840772	0,155646522	0,277699771	-0,589688448	-0,501235181	-0,589001685	-0,638001685	-1,898001685
chrVIII	556826	556979	153	0,265009134	0,429880871	0,381368464	-0,301888676	-0,256605374	-0,281003431	-0,344003431	-1,604003431
chrVIII	556826	556981	155	0,059840772	0,292763697	0,16971076	-0,955308878	-0,812012546	-0,806811282	-0,883811282	-2,143811282
chrVIII	556826	556982	156	0,473027057	0,637409568	0,425982939	-0,186610698	-0,158619094	-0,139601099	-0,223601099	-1,483601099
chrVIII	556826	556983	157	0,455929693	0,948702612	0,324590066	-0,454901459	-0,386666241	-0,328147984	-0,419147984	-1,679147984
chrVIII	556826	556984	158	0,213717044	0,214940436	0,498572996	-0,003576976	-0,00304043	0,09010487	-0,00789513	-1,26789513
chrVIII	556826	556985	159	0,14532759	0,26311674	0,355807583	-0,369687743	-0,314234581	-0,217692098	-0,322692098	-1,582692098
chrVIII	556826	556986	160	4,912642446	11,15466743	0,305753887	-0,507922388	-0,431734029	-0,352828859	-0,464828859	-1,724828859
chrVIII	556826	556987	161	1,592904366	1,604641528	0,498164024	-0,004600529	-0,00391045	0,148691623	0,029691623	-1,230308377
chrVIII	556826	556989	163	22,00430683	12,47766287	0,638139498	0,35349016	0,300466636	0,438092727	0,305092727	-0,954907273
chrVIII	556833	556986	153	0,042743409	0,092646739	0,315705458	-0,47974192	-0,407780632	-0,430257145	-0,493257145	-1,753257145
chrVIII	556840	556986	146	0,074088575	0,040764565	0,645072262	0,372050197	0,316242667	0,305541745	0,291541745	-0,968458255
chrVIII	556843	556989	146	0,071239015	0,114881957	0,382756516	-0,298249123	-0,253511755	-0,288075941	-0,302075941	-1,562075941
chrVIII	556853	556986	133	0,031345166	0,051882174	0,376621026	-0,314367347	-0,267212245	0,040958895	0,117958895	-1,142041105
chrVIII	556853	556989	136	0,259310013	0,196411088	0,569010328	0,173855096	0,147776831	0,329398965	0,385398965	-0,874601035
chrVIII	556858	556975	117	0,04844253	0,037058696	0,566571175	0,16765125	0,142503563	0,662751799	0,851751799	-0,408248201
chrVIII	556858	556989	131	0,105433742	0,159352392	0,398184528	-0,258049045	-0,219341688	0,164669993	0,255669993	-1,004330007
chrVIII	556859	556963	104	0,102584181	0,062999783	0,619529685	0,304245806	0,258608935	0,912651864	1,192651864	-0,067348136
chrVIII	556859	556989	130	0,09688506	0,114881957	0,45750779	-0,106714373	-0,090707217	0,369016972	0,467016972	-0,792983028
chrVIII	556863	556986	123	0,04844253	0,200116957	0,194893104	-0,860005143	-0,731004372	-0,153982049	-0,006982049	-1,266982049
chrVIII	556869	556986	117	0,059840772	0,151940653	0,282559116	-0,575255914	-0,488967527	0,022228005	0,211228005	-1,048771995
chrVIII	556878	556973	95	0,042743409	0,248293262	0,146866059	-1,04996954	-0,892474109	-0,254114788	0,088885212	-1,171114788
chrVIII	556878	556975	97	0,031345166	0,129705435	0,194629303	-0,860962664	-0,731818264	-0,071943975	0,257056025	-1,002943975
chrVIII	556878	556983	105	0,028495606	0,08894087	0,242646977	-0,69781331	-0,593141313	0,034102657	0,307102657	-0,952897343
chrVIII	556878	556989	111	0,641151131	0,570703915	0,529065859	0,072921881	0,061983599	0,672797816	0,903797816	-0,356202184
chrVIII	556883	556989	106	0,521469587	0,478057176	0,521716483	0,054462061	0,046292752	0,718549573	0,984549573	-0,275450427
chrVIII	556887	556986	99	0,168124074	0,333528262	0,335140619	-0,425762057	-0,361897749	0,357970238	0,672970238	-0,587029762
chrVIII	556893	556975	82	0,054141651	0,066705652	0,448017039	-0,130672891	-0,111071958	0,486371872	0,920371872	-0,339628128
chrVIII	556893	556989	96	0,105433742	0,08894087	0,542425478	0,106546147	0,090564225	0,711059436	1,047059436	-0,212940564
chrVIII	556901	556986	85	0,059840772	0,340940001	0,149310486	-1,039395206	-0,883485925	-0,30958262	0,10341738	-1,15658262
chrVIII	556904	556989	85	0,407487163	0,366881088	0,52621884	0,065768269	0,055903029	0,669233531	1,082233531	-0,177766469
chrVIII	558701	558838	137	0,504372223	0,470645436	0,517295475	0,043366916	0,036861878	0,198654037	0,247654037	-1,012345963
chrVIII	558986	559129	143	0,401788042	0,344645871	0,538276779	0,096039338	0,081679337	0,190870383	0,197870383	-1,062129617
chrVIII	559259	559411	152	0,105433742	0,059293913	0,640048823	0,358589298	0,304800903	0,23836908	0,18236908	-1,07763092
chrVIII	559332	559476	144	0,09688506	0,048176305	0,667890174	0,43409473	0,36898052	0,396777586	0,396777586	-0,863222414
chrVIII	560157	560308	151	0,698142343	0,470645436	0,597321734	0,246420767	0,209457652	0,132836544	0,083836544	-1,176163456
chrVIII	560416	560563	147	0,094035499	0,096352609	0,493914773	-0,015253994	-0,012965895	-0,076717533	-0,097717533	-1,357717533
chrVIII	560448	560563	115	0,116831984	0,107470218	0,520868645	0,052333815	0,044483743	0,526085074	0,729085074	-0,530914926

Chrom	Start	End	Length	Section A	Section B	A/A+B	Z-score	Z * 0.85	Phase correction	Length correction	ΔLKnuc
chrVIII	560502	560671	169	0,071239015	0,296469566	0,1937377	-0,864204794	-0,734574075	-0,581890925	-0,756890925	**-2,016890925**
chrVIII	560544	560634	90	0,111132863	0,188999349	0,370279692	-0,331112667	-0,281445767	0,386635458	0,764635458	**-0,495364542**
chrVIII	560544	560639	95	0,062690333	0,103764348	0,376621026	-0,314367347	-0,267212245	0,362524987	0,705524987	**-0,554475013**
chrVIII	560784	560911	127	0,034194727	0,055588044	0,380860679	-0,303221116	-0,257737948	0,250530614	0,369530614	**-0,890469386**
chrVIII	561208	561356	148	0,056991212	0,055588044	0,506231912	0,015621722	0,013278464	-0,107654132	-0,135654132	**-1,395654132**
chrVIII	561302	561476	174	0,028495606	0,055588044	0,338895921	-0,415478239	-0,353156503	-0,075644246	-0,285644246	**-1,545644246**
chrVIII	561649	561800	151	0,53856695	0,35205761	0,60470705	0,265549866	0,225717386	0,158021988	0,109021988	**-1,150978012**
chrVIII	562455	562594	139	0,612655525	0,066705652	0,901811209	1,29194099	1,098149841	1,249984111	1,284984111	**0,024984111**
chrVIII	562455	562640	185	0,176672756	0,051882174	0,772999103	0,748760132	0,636446112	0,930183756	0,643183756	**-0,616816244**
chrVIII	562469	562594	125	0,136778908	0,111176087	0,551627959	0,129775456	0,110309137	0,644434514	0,777434514	**-0,482565486**
chrVIII	562469	562614	145	0,028495606	0,037058696	0,434687048	-0,164453569	-0,139785534	-0,134378939	-0,141378939	**-1,401378939**
chrVIII	562469	562632	163	0,039893848	0,044470435	0,472876041	-0,06804215	-0,057835827	0,087069844	-0,045930156	**-1,305930156**
chrVIII	562469	562636	167	0,168124074	0,411351523	0,290131414	-0,553000851	-0,470050723	-0,296037227	-0,457037227	**-1,717037227**
chrVIII	562469	562640	171	0,136778908	0,125999566	0,520510322	0,051434423	0,043719259	0,255977844	0,066977844	**-1,193022156**
chrVIII	562500	562636	136	0,233663968	0,140823044	0,623957468	0,315891237	0,268507552	0,436544841	0,492544841	**-0,767455159**
chrVIII	562506	562632	126	0,059840772	0,062999783	0,487141826	-0,032236246	-0,027400809	0,497205419	0,623205419	**-0,636794581**
chrVIII	562506	562636	130	0,14817715	0,463233697	0,242352832	-0,698754187	-0,593941059	-0,162128897	-0,064128897	**-1,324128897**
chrVIII	562506	562640	134	0,256460453	0,100058479	0,719345959	0,58089966	0,493764711	0,733026293	0,803026293	**-0,456973707**
chrVIII	562520	562621	101	0,367593315	0,111176087	0,767787819	0,731580977	0,62184383	1,329846173	1,630846173	**0,370846173**
chrVIII	562520	562640	120	0,079787696	0,185293479	0,300993446	-0,521545394	-0,443313585	0,117241241	0,285241241	**-0,974758759**
chrVIII	562523	562640	117	0,581310359	0,037058696	0,940070261	1,555363688	1,322059135	1,842860303	2,031860303	**0,771860303**
chrVIII	562552	562640	88	0,162424953	0,248293262	0,395465668	-0,265101417	-0,225336204	0,441643904	0,833643904	**-0,426356096**
chrX	1669	1785	116	0,139628469	0,085235	0,620947766	0,307970909	0,261775272	0,976875124	1,172875124	**-0,087124876**
chrX	1669	1804	135	2,353737042	1,237760439	0,65536369	0,399842372	0,339866016	0,361212062	0,424212062	**-0,835787938**
chrX	1669	1821	152	0,304902982	0,285351958	0,516561509	0,041525478	0,035296656	-0,073767415	-0,129767415	**-1,389767415**
chrX	1693	1785	92	0,156725832	0,17788174	0,468386986	-0,079325188	-0,06742641	0,379492978	0,743492978	**-0,516507022**
chrX	1693	1794	101	0,079787696	0,196411088	0,288877797	-0,556666083	-0,47316617	0,109043226	0,410043226	**-0,849956774**
chrX	1693	1804	111	0,416035845	0,370586958	0,528888615	0,072476421	0,061604958	0,848664579	1,079664579	**-0,180335421**
chrX	1693	1821	128	7,292025533	8,378971117	0,465319832	-0,087040066	-0,073984056	0,361257021	0,473257021	**-0,786742979**
chrX	1698	1821	123	0,056991212	0,074117392	0,434687048	-0,164453569	-0,139785534	0,450048044	0,597048044	**-0,662951956**
chrX	1980	2120	140	0,068389454	0,040764565	0,626540868	0,322705529	0,274299699	0,131516735	0,159516735	**-1,100483265**
chrX	2198	2335	137	0,111132863	0,081529131	0,576828158	0,1937857	0,164717845	0,10435634	0,15335634	**-1,10664366**
chrX	2365	2509	144	0,074088575	0,048176305	0,605967759	0,268824834	0,228501109	0,053138325	0,053138325	**-1,206861675**
chrX	2365	2512	147	0,04844253	0,040764565	0,543034495	0,108081541	0,09186931	-0,029235743	-0,050235743	**-1,310235743**
chrX	2365	2513	148	17,40511604	11,19913787	0,608479987	0,275359539	0,234055608	0,138148361	0,110148361	**-1,149851639**
chrX	2365	2575	210	0,091185939	0,085235	0,516865737	0,042288735	0,035945424	0,273164822	-0,188835178	**-1,448835178**
chrX	2370	2513	143	0,045592969	0,037058696	0,551627959	0,129775456	0,110309137	-0,074709072	-0,067709072	**-1,327709072**
chrX	2380	2513	133	1,088532143	1,171054787	0,48173944	-0,04578843	-0,038920166	0,011656548	0,088656548	**-1,171343452**
chrX	2420	2513	93	0,316301225	0,459527828	0,407694483	-0,233479654	-0,198457706	0,274149528	0,631149528	**-0,628850472**
chrX	2420	2575	155	0,900461144	0,807879568	0,52709693	0,06797424	0,057778104	0,004136618	-0,072863382	**-1,332863382**
chrX	2862	3015	153	0,83207169	0,604056741	0,579385292	0,200321158	0,170272984	0,094062119	0,031062119	**-1,228937881**
chrX	3307	3451	144	0,091185939	0,051882174	0,637360324	0,351411905	0,298700119	0,12560838	0,12560838	**-1,13439162**
chrX	3518	3609	91	0,031345166	0,129705435	0,194629303	-0,860962664	-0,731818264	-0,341099332	0,029900668	**-1,230099332**
chrX	3518	3627	109	14,86045844	43,32902711	0,255380475	-0,657653281	-0,559005289	0,186674252	0,431674252	**-0,828325748**
chrX	3518	3628	110	0,262159574	0,800467829	0,24670884	-0,684883118	-0,582150651	0,14610346	0,38410346	**-0,87589654**
chrX	3518	3629	111	0,168124074	0,885702829	0,159536707	-0,9963638	-0,84690923	-0,076302234	0,154697766	**-1,105302234**
chrX	3518	3644	126	0,034194727	2,093816312	0,016068867	-2,142693289	-1,821289296	-1,323596047	-1,197596047	**-2,457596047**
chrX	3520	3627	107	0,265009134	2,427344574	0,098430282	-1,290547693	-1,096965539	-0,414407206	-0,155407206	**-1,415407206**
chrX	3520	3644	124	0,424584527	4,120926971	0,093407426	-1,320060405	-1,122051344	-0,599221196	-0,459221196	**-1,719221196**
chrX	4071	4226	155	0,074088575	0,200116957	0,270193583	-0,612227627	-0,520393483	-0,573277336	-0,650277336	**-1,910277336**
chrX	4165	4327	162	0,054141651	0,070411522	0,434687048	-0,164453569	-0,139785534	-0,064790461	-0,190790461	**-1,450790461**
chrX	4165	4327	162	0,054141651	0,070411522	0,434687048	-0,164453569	-0,139785534	-0,064790461	-0,190790461	**-1,450790461**
chrX	4788	4951	163	0,253610892	0,329822392	0,434687048	-0,164453569	-0,139785534	-0,050979978	-0,183979978	**-1,443979978**
chrX	5199	5350	151	0,233663968	0,125999566	0,649673781	0,384439894	0,326773909	0,186702566	0,137702566	**-1,122297434**
chrX	5412	5571	159	0,492973981	0,452116089	0,521615872	0,054209495	0,046078071	0,043569487	-0,061430513	**-1,321430513**
chrX	9273	9424	151	0,492973981	0,466939567	0,513560812	0,033998463	0,028898694	-0,090793187	-0,139793187	**-1,399793187**
chrX	10211	10354	143	0,102584181	0,055588044	0,648560019	0,381435717	0,324220359	0,15281907	0,15981907	**-1,10018093**
chrX	11443	11594	151	0,359044634	0,255705001	0,584050178	0,21226584	0,180425964	0,057161732	0,008161732	**-1,251838268**
chrX	12328	12473	145	0,279256937	0,133411305	0,676710512	0,458519889	0,389741906	0,215339177	0,208339177	**-1,051660823**
chrX	12893	13042	149	0,039893848	0,048176305	0,452978074	-0,118140734	-0,100419624	-0,202918187	-0,237918187	**-1,497918187**
chrX	14191	14340	149	0,136778908	0,055588044	0,711031218	0,556399818	0,472939845	0,368803942	0,333803942	**-0,926196058**
chrX	16003	16132	129	0,133929347	0,125999566	0,515253751	0,038244804	0,032508084	0,417341776	0,522341776	**-0,737658224**
chrX	16061	16168	107	0,142478029	1,708405876	0,076978372	-1,425693812	-1,211839741	-0,533038136	-0,274038136	**-1,534038136**
chrX	21416	21535	119	0,065539893	0,500292393	0,115829186	-1,196097859	-1,01668318	-0,375940079	-0,200940079	**-1,460940079**
chrX	28854	29005	151	0,330549028	0,292763697	0,530310091	0,076049373	0,064641967	-0,049456578	-0,098456578	**-1,358456578**
chrX	28854	29005	151	0,330549028	0,292763697	0,530310091	0,076049373	0,064641967	-0,049456578	-0,098456578	**-1,358456578**
chrX	30398	30549	151	0,541416511	0,455821958	0,54291579	0,107782253	0,091614915	-0,027830824	-0,076830824	**-1,336830824**
chrX	31626	31734	108	0,720938828	1,964110877	0,268501111	-0,617352537	-0,524749656	0,171907592	0,423907592	**-0,836092408**
chrX	32694	32839	145	1,353541277	1,030231743	0,567814664	0,170813172	0,145191196	-0,023824729	-0,030824729	**-1,290824729**
chrX	33933	34077	144	1,202514566	0,856055873	0,584150313	0,212522567	0,180644182	0,004374341	0,004374341	**-1,255625659**
chrX	37483	37615	132	0,92895675	1,693582397	0,354220356	-0,373951081	-0,317858419	-0,224493138	-0,140493138	**-1,400493138**
chrX	38720	38876	156	0,404637603	0,326116523	0,55372606	0,13508094	0,114818799	0,09035836	0,00635836	**-1,25364164**
chrX	40633	40803	170	0,250761331	0,500292393	0,333879353	-0,42922608	-0,364842168	-0,264630918	-0,446630918	**-1,706630918**
chrX	40783	40953	170	0,185221438	0,285351958	0,393607968	-0,269927623	-0,229438479	-0,095780227	-0,277780227	**-1,537780227**
chrX	46039	46190	151	0,284956058	0,218646305	0,565835427	0,165781194	0,140914015	0,028632183	-0,020367817	**-1,280367817**
chrX	53460	53598	138	0,14532759	0,125999566	0,535617563	0,08939893	0,07598909	-0,012023216	0,029976784	**-1,230023216**
chrX	55658	55819	161	0,82922213	0,796761959	0,5099817	0,025023022	0,021269569	0,080330804	-0,038669196	**-1,298669196**
chrX	59858	59998	140	3,997933499	2,834990228	0,585098511	0,214954263	0,182711124	0,041734344	0,069734344	**-1,190265656**
chrX	64405	64545	140	0,09688506	0,081529131	0,543034495	0,108081541	0,09186931	-0,053034458	-0,025034458	**-1,285034458**
chrX	86256	86397	141	0,176672756	0,111176087	0,613769206	0,28915654	0,245783059	0,065295833	0,086295833	**-1,173704167**
chrX	88120	88277	157	1,46467414	1,530524136	0,489007406	-0,027557834	-0,023424159	-0,0303057	-0,1213057	**-1,3813057**
chrX	90912	91092	180	0,720938828	1,58240631	0,31299644	-0,487374615	-0,414268423	-0,207804921	-0,459804921	**-1,719804921**
chrX	94290	94441	151	0,213717044	0,055588044	0,793587102	0,818930974	0,696091328	0,591519909	0,542519909	**-0,717480091**
chrX	94366	94450	84	0,216566604	0,385410436	0,359758911	-0,359103289	-0,305237796	0,058599328	0,478599328	**-0,781400672**
chrX	94724	94862	138	0,333398588	0,363175219	0,478626364	-0,053601417	-0,045561205	-0,134699576	-0,092699576	**-1,352699576**
chrX	100058	100217	159	11,01070209	8,693970031	0,558786364	0,147892925	0,125708986	0,135727092	0,030727092	**-1,229272908**
chrX	100062	100217	155	0,039893848	0,059293913	0,402205349	-0,247642923	-0,210496484	-0,249128037	-0,326128037	**-1,586128037**
chrX	100066	100217	151	0,039893848	0,040764565	0,49460244	-0,013530088	-0,011500575	-0,113028956	-0,162028956	**-1,422028956**
chrX	105547	105698	151	1,062886098	0,607762611	0,636211606	0,348350745	0,296098133	0,18935592	0,14035592	**-1,11964408**
chrX	106971	107136	165	0,344796831	0,34835174	0,49743568	-0,006427842	-0,005463666	0,118780462	-0,028219538	**-1,288219538**
chrX	108096	108187	91	0,088336378	0,326116523	0,213139727	-0,795574396	-0,676238236	-0,266196189	0,104803811	**-1,155196189**
chrX	108096	108204	108	0,028495606	0,100058479	0,221662392	-0,766590907	-0,651602271	0,053881696	0,305881696	**-0,954118304**
chrX	108096	108205	109	36,9929955	115,334073	0,242852409	-0,697156566	-0,592583081	0,149300326	0,394300326	**-0,865699674**
chrX	108096	108206	110	0,512920905	1,893699355	0,213129139	-0,795610814	-0,676269192	0,077112619	0,315112619	**-0,944887381**
chrX	108096	108207	111	0,376141997	2,516285444	0,130043711	-1,126184528	-0,957256849	-0,155729181	0,075270819	**-1,184729181**
chrX	108096	108208	112	0,028495606	0,166764131	0,145936926	-1,054019799	-0,895916829	-0,117019389	0,106980611	**-1,153019389**

Chrom	Start	End	Length	Section A	Section B	A/A+B	Z-score	Z * 0.85	Phase correction	Length correction	ΔLKnuc
chrX	108096	108220	124	0,051292091	5,62551002	0,009035385	-2,364164857	-2,009540128	-1,506057229	-1,366057229	**-2,626057229**
chrX	108097	108205	108	0,034194727	0,103764348	0,247861382	-0,681235065	-0,579049806	0,128730357	0,380730357	**-0,879269643**
chrX	108098	108200	102	0,037044288	0,26311674	0,123414715	-1,158084807	-0,984372086	-0,393329665	-0,099329665	**-1,359329665**
chrX	108098	108205	107	0,823523009	8,00467829	0,093283216	-1,32080488	-1,122684148	-0,442181936	-0,183181936	**-1,443181936**
chrX	108098	108206	108	0,031345166	0,277940218	0,101347066	-1,273913333	-1,082826333	-0,35277316	-0,10077316	**-1,36077316**
chrX	108098	108220	122	1,233859733	10,79149221	0,102604875	-1,266847707	-1,076820551	-0,453361863	-0,299361863	**-1,559361863**
chrX	108135	108220	85	0,031345166	0,255705001	0,10919752	-1,230807045	-1,046185988	-0,664526842	-0,251526842	**-1,511526842**
chrX	117567	117726	159	0,253610892	0,207528696	0,54996556	0,125574335	0,106738185	0,122510802	0,017510802	**-1,242489198**
chrX	118042	118180	138	8,870682096	5,518039803	0,616502436	0,29630767	0,25186152	0,150146802	0,192146802	**-1,067853198**
chrX	118045	118180	135	0,056991212	0,037058696	0,605967759	0,268824834	0,228501109	0,255711578	0,318711578	**-0,941288422**
chrX	118063	118213	150	4,69037672	2,979519141	0,611530692	0,283310777	0,24081416	0,106275804	0,064275804	**-1,195724196**
chrX	118208	118364	156	0,29065518	0,392822175	0,425259414	-0,18845648	-0,160188008	-0,179681402	-0,263681402	**-1,523681402**
chrX	122371	122478	107	0,062690333	0,444704349	0,12355339	-1,157405375	-0,983794569	-0,298979864	-0,039979864	**-1,299979864**
chrX	126969	127111	142	0,980248841	0,79305609	0,552780756	0,132690081	0,112786569	-0,07384166	-0,05984166	**-1,31984166**
chrX	127866	128026	160	0,239363089	0,203822827	0,540096335	0,100676421	0,085574958	0,123873309	0,011873309	**-1,248126691**
chrX	131244	131407	163	0,649699813	1,004290656	0,392807471	-0,272009205	-0,231207824	-0,132964617	-0,265964617	**-1,525964617**
chrX	139844	139996	152	0,059840772	0,070411522	0,459422021	-0,101889932	-0,086606442	-0,196267132	-0,252267132	**-1,512267132**
chrX	141168	141318	150	0,062690333	0,048176305	0,56545715	0,164819952	0,140096959	0,005226772	-0,036773228	**-1,296773228**
chrX	142696	142846	150	0,73233707	0,544762828	0,573437576	0,185132835	0,15736291	0,008443327	-0,033556673	**-1,293556673**
chrX	145224	145374	150	0,424584527	0,444704349	0,488427424	-0,029012215	-0,024660382	-0,175802941	-0,217802941	**-1,477802941**
chrX	145224	145374	150	0,424584527	0,444704349	0,488427424	-0,029012215	-0,024660382	-0,175802941	-0,217802941	**-1,477802941**
chrX	148124	148271	147	0,156725832	0,103764348	0,601657352	0,2576393	0,218993405	0,106111371	0,085111371	**-1,174888629**
chrX	149558	149726	168	0,988797522	1,693582397	0,368626948	-0,33549211	-0,285168294	-0,189893786	-0,357893786	**-1,617893786**
chrX	154966	155110	144	0,324849907	0,392822175	0,452643923	-0,118984235	-0,1011366	-0,279786272	-0,279786272	**-1,539786272**
chrX	162602	162770	168	0,48727486	0,652233046	0,427618674	-0,182440113	-0,155074096	-0,05141696	-0,21941696	**-1,47941696**
chrX	163009	163162	153	1,877860425	1,786229137	0,512503964	0,031347923	0,026645735	-0,043731271	-0,106731271	**-1,366731271**
chrX	169091	169251	160	0,094035499	0,148234783	0,388142938	-0,284162468	-0,241538098	-0,19210159	-0,30410159	**-1,56410159**
chrX	173679	173830	151	0,028495606	0,037058696	0,434687048	-0,164453569	-0,139785534	-0,253499413	-0,302499413	**-1,562499413**
chrX	173874	174016	142	0,960301917	0,711526959	0,57440204	0,187592737	0,159453827	-0,026576999	-0,012576999	**-1,272576999**
chrX	178426	178580	154	0,823523009	0,596645002	0,579877171	0,201579264	0,171342374	0,085839665	0,015839665	**-1,244160335**
chrX	182961	183077	116	8,728204067	26,16343922	0,250151705	-0,674012431	-0,572910567	0,130628926	0,326628926	**-0,933371074**
chrX	182964	183077	113	0,042743409	0,203822827	0,173354671	-0,940991251	-0,799842563	-0,040078441	0,176921559	**-1,083078441**
chrX	182966	183077	111	0,039893848	0,096352609	0,292806499	-0,545204323	-0,463423675	0,336325301	0,567325301	**-0,692674699**
chrX	185927	186084	157	0,071239015	0,062999783	0,530688713	0,07700122	0,065451037	0,067124536	-0,023875464	**-1,283875464**
chrX	185927	186084	157	0,071239015	0,062999783	0,530688713	0,07700122	0,065451037	0,067124536	-0,023875464	**-1,283875464**
chrX	189658	189821	163	0,43883233	0,340940001	0,562769814	0,157995466	0,134296146	0,238555579	0,105555579	**-1,154444421**
chrX	190658	190805	147	1,188266763	0,737468046	0,617045897	0,297731361	0,253071657	0,135833629	0,114833629	**-1,145166371**
chrX	191225	191359	134	0,322000346	0,344645871	0,483015335	-0,04258711	-0,036199044	-0,006156856	0,063843144	**-1,196156856**
chrX	192006	192112	106	0,262159574	0,804173699	0,245851443	-0,687602893	-0,584462459	0,052378171	0,318378171	**-0,941621829**
chrX	192006	192128	122	0,190920559	0,893114568	0,17612027	-0,930252163	-0,790714339	-0,176933223	-0,022933223	**-1,282933223**
chrX	192132	192293	161	0,53571739	0,470645436	0,532330265	0,081128865	0,068959535	0,114542894	-0,004457106	**-1,264457106**
chrX	192599	192736	137	0,042743409	0,125999566	0,25330482	-0,664126048	-0,564507141	-0,623692411	-0,574692411	**-1,834692411**
chrX	194181	194318	137	5,303032246	4,065338928	0,566057018	0,166344351	0,141392699	0,071968938	0,120968938	**-1,139031062**
chrX	197629	197777	148	0,151026711	0,066705652	0,693634647	0,506179412	0,4302525	0,331527788	0,303527788	**-0,956472212**
chrX	198154	198316	162	0,037044288	0,077823261	0,322495674	-0,460731366	-0,391621661	-0,309549094	-0,435549094	**-1,695549094**
chrX	198182	198330	148	0,282106498	0,222352175	0,559226183	0,149007602	0,126656462	0,029934556	0,001934556	**-1,258065444**
chrX	199707	199858	151	0,051292091	0,070411522	0,421450846	-0,198183249	-0,168455762	-0,283069346	-0,332069346	**-1,592069346**
chrX	203379	203541	162	0,139628469	0,192705218	0,420145397	-0,201521534	-0,171293304	-0,094224784	-0,220224784	**-1,480224784**
chrX	203482	203630	148	0,136778908	0,08894087	0,605967759	0,268824834	0,228501109	0,127621958	0,099621958	**-1,160378042**
chrX	206401	206560	159	0,168124074	0,314998914	0,347994358	-0,390740963	-0,332129819	-0,310180542	-0,415180542	**-1,675180542**
chrX	209002	209147	145	0,578460798	0,444704349	0,565364056	0,164583414	0,139895902	-0,03003576	-0,03703576	**-1,29703576**
chrX	210689	210857	168	0,92610719	1,267407396	0,422202431	-0,196262308	-0,166822962	-0,065451383	-0,233451383	**-1,493451383**
chrX	210689	210857	168	0,92610719	1,267407396	0,422202431	-0,196262308	-0,166822962	-0,065451383	-0,233451383	**-1,493451383**
chrX	212042	212183	141	3,539154245	2,11234566	0,626232735	0,32189198	0,273608183	0,091915266	0,112915266	**-1,147084734**
chrX	212042	212183	141	3,539154245	2,11234566	0,626232735	0,32189198	0,273608183	0,091915266	0,112915266	**-1,147084734**
chrX	215649	215789	140	0,139628469	0,103764348	0,573675388	0,185739279	0,157878387	0,00753646	0,03553646	**-1,22446354**
chrX	218927	219076	149	0,037044288	0,055588044	0,399906675	-0,253588671	-0,21555037	-0,313594929	-0,348594929	**-1,608594929**
chrX	219865	220016	151	0,373292436	0,244587392	0,604150547	0,264105137	0,224489366	0,109423823	0,060423823	**-1,199576177**
chrX	219865	220016	151	0,373292436	0,244587392	0,604150547	0,264105137	0,224489366	0,109423823	0,060423823	**-1,199576177**
chrX	220580	220715	135	0,327699467	0,70411522	0,317595273	-0,474433869	-0,403268789	-0,388892948	-0,325892948	**-1,585892948**
chrX	221358	221508	150	0,265009134	0,203822827	0,565253985	0,164303749	0,139658187	-0,007063329	-0,049063329	**-1,309063329**
chrX	225766	225921	155	0,823523009	0,685585872	0,545701519	0,114808437	0,097587171	0,060791685	-0,016208315	**-1,276208315**
chrX	226052	226204	152	0,179522317	0,203822827	0,468304659	-0,079532203	-0,067602373	-0,172928758	-0,228928758	**-1,488928758**
chrX	226472	226633	161	0,279256937	0,259410871	0,518421434	0,046192108	0,039263292	0,084475079	-0,034524921	**-1,294524921**
chrX	227406	227571	165	0,359044634	0,463233697	0,436646109	-0,159478092	-0,135556378	-0,003733447	-0,150733447	**-1,410733447**
chrX	227406	227571	165	0,359044634	0,463233697	0,436646109	-0,159478092	-0,135556378	-0,003733447	-0,150733447	**-1,410733447**
chrX	229005	229137	132	0,128230226	0,200116957	0,390532439	-0,277931585	-0,236241848	-0,152275249	-0,068275249	**-1,328275249**
chrX	229384	229535	151	0,205168362	0,203822827	0,501644944	0,004123275	0,003504784	-0,110721107	-0,159721107	**-1,419721107**
chrX	233516	233665	149	0,213717044	0,307587175	0,409966074	-0,227632249	-0,193487411	-0,288696633	-0,323696633	**-1,583696633**
chrX	235494	235644	150	0,512920905	0,481763045	0,515662191	0,039269381	0,033378974	-0,115236663	-0,157236663	**-1,417236663**
chrX	237835	237975	140	1,934851636	1,241466309	0,609149232	0,277102325	0,235536977	0,084796891	0,112796891	**-1,147203109**
chrX	240265	240414	149	0,356195073	0,203822827	0,6360423	0,347899847	0,29571487	0,198447788	0,163447788	**-1,096552212**
chrX	240554	240707	153	0,122531105	0,103764348	0,541465166	0,104125608	0,088506767	0,007812413	-0,055187587	**-1,315187587**
chrX	240601	240747	146	0,814974327	0,626291959	0,56545715	0,164819952	0,140096959	-0,026899591	-0,040899591	**-1,300899591**
chrX	242702	242868	166	0,481575739	0,826408916	0,36818149	-0,336673583	-0,286172546	-0,164505634	-0,318505634	**-1,578505634**
chrX	243519	243619	100	0,034194727	0,059293913	0,365763444	-0,34309514	-0,291630869	0,285432307	0,593432307	**-0,666567693**
chrX	245287	245435	148	0,156725832	0,148234783	0,51392155	0,034903236	0,029667751	-0,070801988	-0,098801988	**-1,358801988**
chrX	247930	248081	151	0,213717044	0,270528479	0,441340257	-0,14757205	-0,125436242	-0,258695434	-0,307695434	**-1,567695434**
chrX	248270	248435	165	0,199469241	0,285351958	0,411428464	-0,223871969	-0,190291174	-0,060112674	-0,207112674	**-1,467112674**
chrX	251069	251224	155	0,076938136	0,077823261	0,497140355	-0,007168129	-0,00609291	-0,035474	-0,112474	**-1,372474**
chrX	251916	252080	164	0,037044288	0,044470435	0,454449042	-0,114428548	-0,097264266	0,020206835	-0,119793165	**-1,379793165**
chrX	253721	253885	164	1,835117016	2,419932835	0,431279792	-0,173116845	-0,147149318	-0,026559303	-0,166559303	**-1,426559303**
chrX	254829	255000	171	0,176672756	0,251999131	0,412139824	-0,222043979	-0,188737382	-0,01692918	-0,20592918	**-1,46592918**
chrX	255572	255735	163	0,142478029	0,303881305	0,31920029	-0,469936228	-0,399445793	-0,297054238	-0,430054238	**-1,690054238**
chrX	271510	271677	167	0,045592969	0,074117392	0,380860679	-0,303221116	-0,257737948	-0,177903556	-0,338903556	**-1,598903556**
chrX	271664	271795	131	0,313451664	0,259410871	0,547167331	0,118507771	0,100731606	0,370516535	0,461516535	**-0,798483465**
chrX	272130	272276	146	3,533455124	2,64228501	0,572150875	0,181852797	0,154574878	-0,000905833	-0,014905833	**-1,274905833**
chrX	273881	274032	151	0,302053422	0,237175653	0,560157892	0,15136954	0,128664109	-0,008405679	-0,057405679	**-1,317405679**
chrX	275971	276080	109	1,723984153	15,91300397	0,097748218	-1,29448938	-1,100315973	-0,357479318	-0,112479318	**-1,372479318**
chrX	276361	276455	94	0,24221265	0,233469783	0,509189814	0,023037485	0,019581862	0,505187217	0,855187217	**-0,404812783**
chrX	276361	276463	102	0,270708255	0,337234132	0,445286036	-0,137580369	-0,116943314	0,49666608	0,79066608	**-0,46933392**
chrX	276481	276614	133	0,262159574	0,544762828	0,324888209	-0,454072817	-0,385961895	-0,338107296	-0,261107296	**-1,521107296**
chrX	278918	279067	149	0,322000346	0,333528262	0,49120716	-0,022042166	-0,018735841	-0,113743397	-0,148743397	**-1,408743397**
chrX	283812	283977	165	0,347646391	0,318704784	0,521716483	0,054462061	0,046292752	0,173543533	0,026543533	**-1,233456467**
chrX	287746	287904	158	0,250761331	0,148234783	0,628480635	0,327831948	0,278657156	0,277120717	0,179120717	**-1,080879283**
chrX	292191	292322	131	0,63260245	0,44099848	0,58923426	0,225575652	0,191739305	0,456759888	0,547759888	**-0,712240112**

Chrom	Start	End	Length	Section A	Section B	A/A+B	Z-score	Z * 0.85	Phase correction	Length correction	ΔLKnuc
chrX	292971	293126	155	0,34194727	0,196411088	0,635166642	0,345568907	0,293733571	0,272592108	0,195592108	**-1,064407892**
chrX	293774	293933	159	0,088336378	0,059293913	0,598362147	0,249109985	0,211743487	0,243399097	0,138399097	**-1,121600903**
chrX	301109	301275	166	0,24506221	0,240881523	0,504301617	0,010782764	0,009165349	0,12453526	-0,02946474	**-1,28946474**
chrX	303727	303887	160	0,256460453	0,233469783	0,523463207	0,058847485	0,050020363	0,098413314	-0,013586686	**-1,273586686**
chrX	309182	309321	139	0,102584181	0,111176087	0,479902939	-0,050397187	-0,042837609	-0,166568691	-0,131568691	**-1,391568691**
chrX	310987	311146	159	1,176868521	0,989467177	0,543253071	0,108632656	0,092337758	0,131146171	0,026146171	**-1,233853829**
chrX	310987	311146	159	1,176868521	0,989467177	0,543253071	0,108632656	0,092337758	0,131146171	0,026146171	**-1,233853829**
chrX	321769	321919	150	0,153876272	0,144528914	0,515662191	0,039269381	0,033378974	-0,115862454	-0,157862454	**-1,417862454**
chrX	322337	322492	155	0,076938136	0,092646739	0,453685128	-0,116356189	-0,098902761	-0,124691398	-0,201691398	**-1,461691398**
chrX	325234	325383	149	0,367593315	0,226058044	0,619207401	0,303399799	0,257889829	0,171138877	0,136138877	**-1,123861123**
chrX	326985	327142	157	1,718285032	1,571288701	0,522342763	0,05603431	0,047629164	0,042486558	-0,048513442	**-1,308513442**
chrX	327633	327785	152	0,09973462	0,096352609	0,508623742	0,021618199	0,01837547	-0,089140593	-0,145140593	**-1,405140593**
chrX	328569	328730	161	0,170973635	0,237175653	0,418899751	-0,204708983	-0,174002636	-0,13263706	-0,25163706	**-1,51163706**
chrX	329259	329407	148	0,430283648	0,644821307	0,400224784	-0,252765321	-0,214850523	-0,308777234	-0,336777234	**-1,596777234**
chrX	329259	329407	148	0,430283648	0,644821307	0,400224784	-0,252765321	-0,214850523	-0,308777234	-0,336777234	**-1,596777234**
chrX	331899	332052	153	0,082637257	0,096352609	0,461686791	-0,096185077	-0,081757315	-0,159096683	-0,222096683	**-1,482096683**
chrX	333305	333466	161	1,473222822	1,715817615	0,461964296	-0,095486273	-0,081163332	-0,031095529	-0,150095529	**-1,410095529**
chrX	333305	333466	161	1,473222822	1,715817615	0,461964296	-0,095486273	-0,081163332	-0,031095529	-0,150095529	**-1,410095529**
chrX	333361	333513	152	0,111132863	0,055588044	0,666580246	0,43048963	0,365916185	0,283731699	0,227731699	**-1,032268301**
chrX	337451	337612	161	0,085486818	0,062999783	0,575720754	0,190958009	0,162314307	0,224105819	0,105105819	**-1,154894181**
chrX	338776	338907	131	0,575611238	1,037643482	0,356801211	-0,367022249	-0,311968912	-0,083496263	0,007503737	**-1,252496263**
chrX	343385	343533	148	0,547115632	0,389116306	0,58438044	0,213112623	0,181145729	0,084033425	0,056033425	**-1,203966575**
chrX	344740	344900	160	0,071239015	0,096352609	0,425075031	-0,188926962	-0,160587917	-0,105610994	-0,217610994	**-1,477610994**
chrX	350347	350517	170	0,401788042	0,566998046	0,414733497	-0,215385217	-0,183077435	-0,046234514	-0,228234514	**-1,488234514**
chrX	350347	350517	170	0,401788042	0,566998046	0,414733497	-0,215385217	-0,183077435	-0,046234514	-0,228234514	**-1,488234514**
chrX	357774	357934	160	0,49012442	0,596645002	0,450992097	-0,123155209	-0,104681928	-0,052926248	-0,164926248	**-1,424926248**
chrX	358769	358924	155	0,116831984	0,107470218	0,520868645	0,052333815	0,044483743	0,013941346	-0,063058654	**-1,323058654**
chrX	359603	359757	154	4,080570756	2,557050009	0,61476407	0,291757738	0,247994077	0,166164504	0,096164504	**-1,163835496**
chrX	362162	362320	158	4,681828039	4,547101973	0,507299116	0,018297192	0,015552613	0,013572689	-0,084427311	**-1,344427311**
chrX	365711	365851	140	1,715435471	1,686170658	0,504301617	0,010782764	0,009165349	-0,153159	-0,125159	**-1,385159**
chrX	366070	366219	149	1,61285129	1,441583266	0,528035962	0,070333681	0,059783629	-0,030857401	-0,065857401	**-1,325857401**
chrX	366353	366502	149	1,159771157	0,578115654	0,667345623	0,43259537	0,367706065	0,277746223	0,242746223	**-1,017253777**
chrX	366353	366502	149	1,159771157	0,578115654	0,667345623	0,43259537	0,367706065	0,277746223	0,242746223	**-1,017253777**
chrX	366520	366671	151	1,162620718	0,559586306	0,675076052	0,453973505	0,385877479	0,25754938	0,20854938	**-1,05145062**
chrX	367696	367845	149	0,364743755	0,326116523	0,527955893	0,070132481	0,059612609	-0,026155084	-0,061155084	**-1,321155084**
chrX	367696	367845	149	0,364743755	0,326116523	0,527955893	0,070132481	0,059612609	-0,026155084	-0,061155084	**-1,321155084**
chrX	369411	369539	128	0,273557816	0,674468263	0,288555159	-0,557610598	-0,473969008	-0,050777534	0,061222466	**-1,198777534**
chrX	369776	369927	151	0,644000692	0,411351523	0,610223471	0,279901524	0,237916295	0,106330376	0,057330376	**-1,202669624**
chrX	374096	374246	150	0,028495606	0,044470435	0,390532439	-0,277931585	-0,236241848	-0,383702638	-0,425702638	**-1,685702638**
chrX	380580	380750	170	0,247911771	0,129705435	0,656516088	0,402973357	0,342527354	0,48555887	0,30355887	**-0,95644113**
chrX	380580	380750	170	0,247911771	0,129705435	0,656516088	0,402973357	0,342527354	0,48555887	0,30355887	**-0,95644113**
chrX	380923	381086	163	1,296550065	1,063584569	0,549354281	0,124030106	0,10542559	0,217790335	0,084790335	**-1,175209665**
chrX	385357	385485	128	0,703841464	1,904816963	0,269809745	-0,613388498	-0,521380224	-0,113501152	-0,001501152	**-1,261501152**
chrX	386997	387161	164	0,031345166	0,055588044	0,360566076	-0,356946107	-0,303404191	-0,186430376	-0,326430376	**-1,586430376**
chrX	392484	392621	137	0,370442876	0,366881088	0,502415348	0,006054416	0,005146254	-0,065479645	-0,016479645	**-1,276479645**
chrX	392991	393143	152	0,179522317	0,111176087	0,617555219	0,299066166	0,254206241	0,172978617	0,116978617	**-1,143021383**
chrX	394180	394326	146	0,450230572	0,322410653	0,582716217	0,208847146	0,177520074	0,024350925	0,010350925	**-1,249649075**
chrX	394276	394372	96	3,36818061	12,65554461	0,210199599	-0,805728874	-0,684869543	-0,147782309	0,188217691	**-1,071782309**
chrX	398245	398394	149	0,364743755	0,366881088	0,498539324	-0,00366138	-0,003112173	-0,094163967	-0,129163967	**-1,389163967**
chrX	399541	399701	160	0,09973462	0,185293479	0,349911537	-0,385559308	-0,327725412	-0,27190185	-0,38390185	**-1,64390185**
chrX	407672	407824	152	1,598603487	1,148819569	0,581855599	0,206642838	0,175646412	0,097537682	0,041537682	**-1,218462318**
chrX	410284	410430	146	0,381841118	0,274234349	0,58200792	0,207032907	0,175977971	0,030037525	0,016037525	**-1,243962475**
chrX	410723	410889	166	0,279256937	0,389116306	0,417815854	-0,20748423	-0,176361595	-0,063927134	-0,217927134	**-1,477927134**
chrX	412052	412213	161	0,718089267	0,648527176	0,525450481	0,063838228	0,054262494	0,115285502	-0,003714498	**-1,263714498**
chrX	425378	425524	146	0,398938482	0,251999131	0,61286746	0,286800501	0,243780426	0,097607643	0,083607643	**-1,176392357**
chrX	429176	429332	156	0,73233707	0,489174784	0,59953333	0,252139371	0,214318465	0,194852405	0,110852405	**-1,149147595**
chrX	429194	429332	138	0,042743409	0,037058696	0,535617563	0,08939893	0,07598909	-0,033419524	0,008580476	**-1,251419524**
chrX	429194	429339	145	0,433133209	0,296469566	0,593656197	0,236960278	0,201416237	0,026624303	0,019624303	**-1,240375697**
chrX	435614	435721	107	0,666797177	3,035107185	0,180122745	-0,914897409	-0,777662797	-0,101078135	0,157921865	**-1,102078135**
chrX	435614	435731	117	0,803576085	3,331576751	0,194328025	-0,862057185	-0,732748607	-0,032064954	0,156935046	**-1,103064954**
chrX	439795	439950	155	0,054141651	0,040764565	0,570475287	0,177584338	0,150946687	0,117971542	0,040971542	**-1,219028458**
chrX	447409	447538	129	0,267858695	0,452116089	0,372038995	-0,32645783	-0,277489155	0,094383077	0,199383077	**-1,060616923**
chrX	448364	448517	153	0,062690333	0,040764565	0,605967759	0,268824834	0,228501109	0,147884486	0,084884486	**-1,175115514**
chrX	456180	456328	148	0,079787696	0,111176087	0,417815854	-0,20748423	-0,176361595	-0,271992869	-0,299992869	**-1,559992869**
chrX	459326	459480	154	0,09688506	0,044470435	0,685400026	0,482853237	0,410425251	0,333071046	0,263071046	**-0,996928954**
chrX	465469	465620	151	0,102584181	0,077823261	0,568624996	0,172874585	0,146943398	0,029981403	-0,019018597	**-1,279018597**
chrX	466504	466659	155	0,133929347	0,074117392	0,643746439	0,368491045	0,313217388	0,286107094	0,209107094	**-1,050892906**
chrX	467197	467335	138	1,541612276	0,996878917	0,607294711	0,272275	0,23143375	0,118358531	0,160358531	**-1,099641469**
chrX	467559	467705	146	0,302053422	0,982055438	0,235224155	-0,721749813	-0,613487341	-0,766973872	-0,780973872	**-2,040973872**
chrX	470836	470984	148	0,666797177	0,485468915	0,578683328	0,198526256	0,168747318	0,063492151	0,035492151	**-1,224507849**
chrX	472163	472315	152	0,302053422	0,274234349	0,524136442	0,060538046	0,051457339	-0,036345881	-0,092345881	**-1,352345881**
chrX	472826	472975	149	0,079787696	0,048176305	0,623516738	0,314730183	0,267520656	0,174960831	0,139960831	**-1,120039169**
chrX	473000	473123	123	0,071239015	0,188999349	0,273745245	-0,601524812	-0,51129609	0,067607002	0,214607002	**-1,045392998**
chrX	473100	473255	155	0,105433742	0,096352609	0,52250185	0,056433715	0,047968658	0,011777119	-0,065222881	**-1,325222881**
chrX	473533	473667	134	0,128230226	0,100058479	0,561702018	0,155285859	0,13199298	0,152615509	0,222615509	**-1,037384491**
chrX	473533	473673	140	0,179522317	0,100058479	0,642112476	0,3641111	0,309494435	0,144406829	0,172406829	**-1,087593171**
chrX	473729	473880	151	0,119681545	0,077823261	0,605967759	0,268824834	0,228501109	0,111840476	0,062840476	**-1,197159524**
chrX	473739	473884	145	0,076938136	0,066705652	0,535617563	0,08939893	0,07598909	-0,095180335	-0,102180335	**-1,362180335**
chrX	473757	473908	151	0,037044288	0,055588044	0,399906675	-0,253588671	-0,21555037	-0,33497204	-0,38397204	**-1,64397204**
chrX	474011	474165	154	0,176672756	0,148234783	0,543763178	0,109918963	0,093431118	0,014492876	-0,055507124	**-1,315507124**
chrX	474045	474189	144	0,065539893	0,040764565	0,616530051	0,29638	0,251923	0,080385137	0,080385137	**-1,179614863**
chrX	474045	474189	144	0,065539893	0,040764565	0,616530051	0,29638	0,251923	0,080385137	0,080385137	**-1,179614863**
chrX	474576	474711	135	0,082637257	0,100058479	0,452321762	-0,11979755	-0,101827917	-0,095907621	-0,032907621	**-1,292907621**
chrX	475023	475169	146	0,088336378	0,048176305	0,64709283	0,377483478	0,320860956	0,163898839	0,149898839	**-1,110101161**
chrX	475023	475173	150	8,864982975	4,235808928	0,676675352	0,458421991	0,389658692	0,241641886	0,199641886	**-1,060358114**
chrX	475693	475822	129	0,062690333	0,092646739	0,403576119	-0,244101464	-0,207486244	0,148617956	0,253617956	**-1,006382044**
chrX	477337	477451	114	0,042743409	0,226058044	0,159014798	-0,998515203	-0,848737923	-0,117420193	0,092579807	**-1,167420193**
chrX	477657	477804	147	0,544266071	0,596645002	0,47704513	-0,057571113	-0,048935446	-0,15626861	-0,17726861	**-1,43726861**
chrX	478238	478390	152	0,037044288	0,037058696	0,499902783	-0,000243688	-0,000207135	-0,10248238	-0,15848238	**-1,41848238**
chrX	478410	478559	149	0,079787696	0,066705652	0,544650641	0,112157259	0,09533367	-0,004871786	-0,039871786	**-1,299871786**
chrX	478560	478666	106	0,749434433	2,308756747	0,245058072	-0,690124109	-0,586605492	0,070880424	0,336880424	**-0,923119576**
chrX	478560	478678	118	0,934655871	2,716402401	0,255995879	-0,655739487	-0,557378564	0,11621819	0,29821819	**-0,96178181**
chrX	478584	478707	123	0,088336378	0,188999349	0,318517845	-0,471847434	-0,401070319	0,151786019	0,298786019	**-0,961213981**
chrX	478684	478839	155	0,09973462	0,081529131	0,550218231	0,126212725	0,107280816	0,066702176	-0,010297824	**-1,270297824**
chrX	479117	479251	134	0,165274514	0,118587827	0,582234733	0,207613792	0,176471723	0,192149877	0,262149877	**-0,997850123**
chrX	479117	479257	140	0,188070999	0,129705435	0,591834317	0,232266067	0,197426157	0,022723659	0,050723659	**-1,209276341**

Chrom	Start	End	Length	Section A	Section B	A/A+B	Z-score	Z * 0.85	Phase correction	Length correction	ΔLKnuc
chrX	480160	480295	135	0,079787696	0,059293913	0,573675388	0,185739279	0,157878387	0,153648194	0,216648194	-1,043351806
chrX	480607	480757	150	8,993213202	4,187632624	0,68229409	0,474123529	0,403005	0,264571345	0,222571345	-1,037428655
chrX	480786	480943	157	0,031345166	0,062999783	0,332240005	-0,433736208	-0,368675777	-0,364180794	-0,455180794	-1,715180794
chrX	481277	481406	129	0,082637257	0,118587827	0,410670755	-0,225819886	-0,191946903	0,140929774	0,245929774	-1,014070226
chrX	481757	481888	131	0,094035499	0,107470218	0,466664175	-0,083658001	-0,071109301	0,101412317	0,192412317	-1,067587683
chrX	482189	482339	150	0,168124074	0,181587609	0,480750522	-0,048270025	-0,041029521	-0,181185188	-0,223185188	-1,483185188
chrX	483140	483291	151	0,059840772	0,096352609	0,38311977	-0,297297305	-0,252702709	-0,36333072	-0,41233072	-1,67233072
chrX	495866	496005	139	0,054141651	0,037058696	0,593656197	0,236960278	0,201416237	0,079341601	0,114341601	-1,145658399
chrX	496369	496517	148	0,495823542	0,340940001	0,592549169	0,23410732	0,198991222	0,09602632	0,06802632	-1,19197368
chrX	498437	498584	147	0,182371877	0,144528914	0,55788142	0,145600009	0,123760008	0,018735614	-0,002264386	-1,262264386
chrX	503072	503238	166	0,97454972	1,248878048	0,438309593	-0,155256401	-0,131967941	-0,027143302	-0,181143302	-1,441143302
chrX	503072	503238	166	0,97454972	1,248878048	0,438309593	-0,155256401	-0,131967941	-0,027143302	-0,181143302	-1,441143302
chrX	503922	504069	147	0,384690679	0,322410653	0,544038968	0,11061448	0,094022308	-0,012096882	-0,033096882	-1,293096882
chrX	512701	512853	152	0,111132863	0,040764565	0,731630971	0,617753167	0,525090192	0,426091403	0,370091403	-0,889908597
chrX	516708	516897	189	0,14817715	0,181587609	0,449341981	-0,127324005	-0,108225404	0,129090868	-0,185909132	-1,445909132
chrX	519161	519304	143	0,530018269	0,400233914	0,569757619	0,175757115	0,149393547	-0,026845374	-0,019845374	-1,279845374
chrX	519732	519838	106	0,042743409	0,340940001	0,111402807	-1,219101651	-1,036236403	-0,357632717	-0,091632717	-1,351632717
chrX	519732	519858	126	1,90635603	5,09927654	0,272117616	-0,606420994	-0,515457845	-0,033615602	0,092384398	-1,167615602
chrX	523978	524108	130	0,085486818	0,107470218	0,443035505	-0,14327752	-0,121785892	0,171987695	0,269987695	-0,990012305
chrX	526346	526498	152	0,814974327	0,289057827	0,738179884	0,637744162	0,542082538	0,434611459	0,378611459	-0,881388541
chrX	527259	527387	128	0,182371877	0,655938915	0,217546856	-0,780504955	-0,663429212	-0,245180911	-0,133180911	-1,393180911
chrX	530647	530795	148	0,367593315	0,229763914	0,615365978	0,29333246	0,249332591	0,146270521	0,118270521	-1,141729479
chrX	531614	531753	139	0,282106498	0,170470001	0,623334395	0,314249947	0,267112455	0,147930683	0,182930683	-1,077069317
chrX	533693	533845	152	0,327699467	0,296469566	0,525017182	0,062749932	0,053337442	-0,059582729	-0,115582729	-1,375582729
chrX	537746	537899	153	0,028495606	0,051882174	0,354520937	-0,373143192	-0,317171714	-0,403252063	-0,466252063	-1,726252063
chrX	542575	542723	148	0,156725832	1,133996091	0,12142494	-1,167893119	-0,992709151	-1,106643628	-1,134643628	-2,394643628
chrX	544311	544443	132	1,017293128	1,356348266	0,428579115	-0,179992788	-0,15299387	-0,082662035	0,001337965	-1,258662035
chrX	547133	547290	157	0,062690333	0,077823261	0,446151373	-0,135390993	-0,115082344	-0,122099822	-0,213099822	-1,473099822
chrX	554602	554737	135	0,133929347	0,151940653	0,468497385	-0,079047591	-0,067190452	-0,070845257	-0,007845257	-1,267845257
chrX	557805	557945	140	0,618354647	0,396528045	0,609286819	0,277460721	0,235841613	0,059409062	0,087409062	-1,172590938
chrX	557805	557945	140	0,618354647	0,396528045	0,609286819	0,277460721	0,235841613	0,059409062	0,087409062	-1,172590938
chrX	557934	558083	149	0,119681545	0,040764565	0,745929861	0,661736237	0,562475801	0,459046321	0,424046321	-0,835953679
chrX	558445	558574	129	2,271099785	2,872048923	0,441577701	-0,146970376	-0,12492482	0,18771165	0,29271165	-0,96728835
chrX	561483	561587	104	0,14532759	1,901111094	0,071014876	-1,468274214	-1,248033082	-0,603504592	-0,323504592	-1,583504592
chrX	561700	561836	136	0,165274514	0,144528914	0,533481877	0,084025388	0,071421579	0,027655478	0,083655478	-1,176344522
chrX	563670	563822	152	3,348233686	2,831284358	0,541827641	0,105039183	0,089283305	-0,019483118	-0,075483118	-1,335483118
chrX	567134	567269	135	0,464478375	0,370586958	0,556218007	0,1413873	0,120179205	0,111508917	0,174508917	-1,085491083
chrX	569137	569291	154	3,561950729	1,864052398	0,656459395	0,402819234	0,342396349	0,267457629	0,197457629	-1,062542371
chrX	572775	572903	128	0,068389454	0,059293913	0,535617563	0,08939893	0,07598909	0,474791432	0,586791432	-0,673208568
chrX	573799	573959	160	0,122531105	0,137117174	0,47191187	-0,070464771	-0,059895055	0,000797969	-0,111202031	-1,371202031
chrX	582808	582958	150	0,128230226	0,055588044	0,69759239	0,517488464	0,439865194	0,299255242	0,257255242	-1,002744758
chrX	586031	586180	149	0,182371877	0,155646522	0,539532397	0,099255756	0,084367393	-0,031640475	-0,066640475	-1,326640475
chrX	590874	591029	155	0,478726178	0,481763045	0,498419104	-0,003962729	-0,00336832	-0,042331855	-0,119331855	-1,379331855
chrX	591957	592113	156	0,604106844	0,507704132	0,543353912	0,108886928	0,092553889	0,079508363	-0,004491637	-1,264491637
chrX	594961	595112	151	2,422126496	1,57870044	0,605406466	0,267366398	0,227261438	0,117887219	0,068887219	-1,191112781
chrX	595496	595660	164	0,091185939	0,040764565	0,691061693	0,498861988	0,42403269	0,530945945	0,390945945	-0,869054055
chrX	598239	598403	164	0,433133209	0,43729261	0,497610709	-0,005989101	-0,005090736	0,107072693	-0,032927307	-1,292927307
chrX	598789	598947	158	0,128230226	0,129705435	0,497140355	-0,007168129	-0,00609291	-0,011018598	-0,109018598	-1,369018598
chrX	600553	600699	146	0,153876272	0,066705652	0,69759239	0,517488464	0,439865194	0,315826314	0,301826314	-0,958173686
chrX	606604	606728	124	0,037044288	0,114881957	0,243830733	-0,694033076	-0,589928115	-0,095552723	0,044447277	-1,215552723
chrX	608387	608544	157	0,663947616	0,689291742	0,490635757	-0,023474833	-0,019953608	-0,032765238	-0,123765238	-1,383765238
chrX	608709	608811	102	0,071239015	0,255705001	0,217893619	-0,779326791	-0,662427772	-0,050717165	0,243282835	-1,016717165
chrX	610999	611149	150	0,219416165	0,185293479	0,542156996	0,105869357	0,089988953	-0,055078591	-0,097078591	-1,357078591
chrX	623512	623664	152	0,567062556	0,744879785	0,432231309	-0,170696241	-0,145091805	-0,248751633	-0,304751633	-1,564751633
chrX	624211	624342	131	5,063669157	7,55997394	0,401125817	-0,250434134	-0,212869014	-0,045013879	0,045986121	-1,214013879
chrX	624949	625089	140	1,088532143	0,652233046	0,625318193	0,319478604	0,271556814	0,090487835	0,118487835	-1,141512165
chrX	632590	632738	148	0,692443222	0,544762828	0,559683023	0,150165614	0,127640772	0,016729073	-0,011270927	-1,271270927
chrX	635827	635966	139	4,322783405	3,005460228	0,58987987	0,22723597	0,193150575	0,059900737	0,094900737	-1,165099263
chrX	636181	636337	156	0,410336724	0,52993935	0,436400261	-0,160102259	-0,13608692	-0,140780806	-0,224780806	-1,484780806
chrX	636181	636337	156	0,410336724	0,52993935	0,436400261	-0,160102259	-0,13608692	-0,140780806	-0,224780806	-1,484780806
chrX	636714	636864	150	0,307752543	0,259410871	0,542617058	0,107029113	0,090974746	-0,057844187	-0,099844187	-1,359844187
chrX	637154	637305	151	0,444531451	0,555880437	0,444348429	-0,139953338	-0,118960337	-0,231868913	-0,280868913	-1,540868913
chrX	637154	637305	151	0,444531451	0,555880437	0,444348429	-0,139953338	-0,118960337	-0,231868913	-0,280868913	-1,540868913
chrX	638977	639126	149	0,319150785	0,200116957	0,614617006	0,291373097	0,247667133	0,135155481	0,100155481	-1,159844519
chrX	653069	653214	145	0,615505086	0,377998697	0,619529685	0,304245806	0,258608935	0,088693411	0,081693411	-1,178306589
chrX	661255	661420	165	0,19661968	0,244587392	0,445640364	-0,13668381	-0,116181238	0,007280614	-0,139719386	-1,399719386
chrX	662029	662161	132	13,16781946	8,953380902	0,595257908	0,241091522	0,204927794	0,260195779	0,344195779	-0,915804221
chrX	662034	662161	127	0,045592969	0,066705652	0,405997586	-0,23785292	-0,202174982	0,256880714	0,375880714	-0,884119286
chrX	662037	662135	98	0,031345166	0,044470435	0,413439528	-0,218706025	-0,185900121	0,359755844	0,681755844	-0,578244156
chrX	671104	671223	119	0,618354647	2,809049141	0,180414881	-0,913785129	-0,776717359	-0,142155771	0,032844229	-1,227155771
chrX	671901	672050	149	0,225115286	0,314998914	0,416792015	-0,210107168	-0,178591093	-0,290638614	-0,325638614	-1,585638614
chrX	673361	673549	188	0,09688506	0,159352392	0,378106554	-0,310457456	-0,263888837	-0,040961129	-0,348961129	-1,608961129
chrX	673385	673539	154	0,418885406	0,35205761	0,543341592	0,108855862	0,092527483	0,025358056	-0,044641944	-1,304641944
chrX	674527	674676	149	0,307752543	0,211234566	0,592986873	0,235235114	0,199949847	0,092519592	0,057519592	-1,202480408
chrX	684490	684642	152	0,3903898	0,337234132	0,536526883	0,091687619	0,077934477	-0,010344291	-0,066344291	-1,326344291
chrX	684490	684642	152	0,3903898	0,337234132	0,536526883	0,091687619	0,077934477	-0,010344291	-0,066344291	-1,326344291
chrX	685942	686069	127	0,492973981	0,922761525	0,348210509	-0,39015624	-0,331632804	0,117668893	0,236668893	-1,023331107
chrX	687031	687188	157	0,455929693	0,61517435	0,425663311	-0,187426022	-0,159312119	-0,171408057	-0,262408057	-1,522408057
chrX	687681	687842	161	0,450230572	0,641115437	0,41254613	-0,221000223	-0,18785019	-0,124074974	-0,243074974	-1,503074974
chrX	689548	689698	150	0,812124766	0,452116089	0,642381365	0,364831393	0,310106684	0,162263239	0,120263239	-1,139736761
chrX	695637	695789	152	0,108283302	0,074117392	0,593656197	0,236960278	0,201416237	0,115366601	0,059366601	-1,200633399
chrX	696260	696391	131	1,12557643	1,226642831	0,478516799	-0,053876453	-0,045794985	0,079073615	0,170073615	-1,089926385
chrX	697538	697692	154	0,113982423	0,122293696	0,482411949	-0,044100996	-0,037485847	-0,093417057	-0,163417057	-1,423417057
chrX	699421	699528	107	0,034194727	0,255705001	0,117953636	-1,1852787	-1,007486895	-0,321540912	-0,062540912	-1,322540912
chrX	699421	699559	138	0,04844253	0,040764565	0,543034495	0,108081541	0,09186931	-0,020454783	0,021545217	-1,238454783
chrX	699421	699560	139	0,045592969	0,051882174	0,467739443	-0,080953558	-0,068810524	-0,202267091	-0,167267091	-1,427267091
chrX	699421	699561	140	0,062690333	0,037058696	0,628480635	0,327831948	0,278657156	0,098849309	0,126849309	-1,133150691
chrX	699421	699563	142	0,034194727	0,040764565	0,456177292	-0,110069087	-0,093558724	-0,277311187	-0,263311187	-1,523311187
chrX	699421	699564	143	16,10286686	10,44684634	0,606517545	0,270253923	0,229715834	0,054434842	0,061434842	-1,198565158
chrX	700845	700991	146	1,376337762	0,815291307	0,627997585	0,326554541	0,27757136	0,1540763	0,1400763	-1,1199237
chrX	701322	701470	148	3,094622794	2,405109357	0,562686093	0,157782975	0,134115528	0,028180105	0,000180105	-1,259819895
chrX	705757	705926	169	0,350495952	0,678174133	0,340727272	-0,410479083	-0,348907221	-0,237219511	-0,412219511	-1,672219511
chrX	705757	705926	169	0,350495952	0,678174133	0,340727272	-0,410479083	-0,348907221	-0,237219511	-0,412219511	-1,672219511
chrX	706905	707066	161	0,068389454	0,096352609	0,415130494	-0,214366854	-0,182211826	-0,110130344	-0,229130344	-1,489130344
chrX	712931	713070	139	0,324849907	0,255705001	0,559550703	0,149830185	0,127355657	-0,004911679	0,030088321	-1,229911679
chrX	714988	715121	133	0,478726178	0,359469349	0,571139027	0,179274784	0,152383566	0,19317228	0,27017228	-0,98982772

Chrom	Start	End	Length	Section A	Section B	A/A+B	Z-score	Z * 0.85	Phase correction	Length correction	ΔLKnuc
chrX	720830	720969	139	0,948903674	0,381704567	0,713135275	0,562567469	0,478182348	0,336129742	0,371129742	**-0,888870258**
chrX	721017	721124	107	0,034194727	0,485468915	0,065801654	-1,507809432	-1,281638017	-0,576883772	-0,317883772	**-1,577883772**
chrX	721017	721148	131	0,04844253	0,055588044	0,465656665	-0,08619258	-0,073263693	0,034771502	0,125771502	**-1,134228498**
chrX	721017	721149	132	0,085486818	0,118587827	0,418899751	-0,204708983	-0,174002636	-0,139438328	-0,055438328	**-1,315438328**
chrX	721017	721153	136	18,6589227	16,02047419	0,538040577	0,095498544	0,081173762	0,026933837	0,082933837	**-1,177066163**
chrX	721018	721153	135	0,065539893	0,037058696	0,638799168	0,355250862	0,301963233	0,273001084	0,336001084	**-0,923998916**
chrX	721019	721153	134	0,039893848	0,059293913	0,402205349	-0,247642923	-0,210496484	-0,189371996	-0,119371996	**-1,379371996**
chrX	721020	721153	133	0,031345166	0,051882174	0,376621026	-0,314367347	-0,267212245	-0,233510415	-0,156510415	**-1,416510415**
chrX	723086	723238	152	0,760832676	0,822703046	0,480464485	-0,04898786	-0,041639681	-0,108966951	-0,164966951	**-1,424966951**
chrX	723301	723431	130	0,156725832	0,066705652	0,701449182	0,528573085	0,449287122	0,736266246	0,834266246	**-0,425733754**
chrX	723442	723594	152	0,29350474	0,17417587	0,627575173	0,325437929	0,27662224	0,209168622	0,153168622	**-1,106831378**
chrX	725588	725741	153	0,347646391	0,381704567	0,476651724	-0,0585589	-0,049775065	-0,143226462	-0,206226462	**-1,466226462**
chrX	729427	729584	157	0,834921251	0,559586306	0,598721209	0,250038492	0,212532718	0,199302874	0,108302874	**-1,151697126**
chrX	729427	729584	157	0,834921251	0,559586306	0,598721209	0,250038492	0,212532718	0,199302874	0,108302874	**-1,151697126**
chrX	729799	729953	154	0,552814753	0,392822175	0,584595141	0,21366319	0,181613711	0,13041811	0,06041811	**-1,19958189**
chrX	732737	732877	140	2,088727908	1,58611218	0,568386068	0,172266698	0,146426693	-0,033381153	-0,005381153	**-1,265381153**
chrX	735177	735331	154	0,039893848	0,081529131	0,328552705	-0,443913104	-0,377326138	-0,425704053	-0,495704053	**-1,755704053**
chrX	735177	735331	154	0,039893848	0,081529131	0,328552705	-0,443913104	-0,377326138	-0,425704053	-0,495704053	**-1,755704053**
chrX	736882	737013	131	0,205168362	0,385410436	0,34740218	-0,39234359	-0,333492051	-0,22883147	-0,13783147	**-1,39783147**
chrX	739148	739242	94	2,065931423	11,71425374	0,14992044	-1,036774676	-0,881258475	-0,376313551	-0,026313551	**-1,286313551**
chrX	745644	745739	95	0,085486818	0,307587175	0,217482762	-0,780722841	-0,663614415	-0,131020398	0,211979602	**-1,048020398**
chrX	745644	745742	98	0,056991212	0,077823261	0,422738082	-0,194893703	-0,165659647	0,38564365	0,70764365	**-0,55235635**
chrXI	6938	7102	164	3,259897308	2,886872402	0,530343166	0,07613252	0,064712642	0,122198787	-0,017801213	**-1,277801213**
chrXI	10271	10420	149	0,273557816	0,151940653	0,642911399	0,366251789	0,31131402	0,207069374	0,172069374	**-1,087930626**
chrXI	17984	18132	148	0,213717044	0,129705435	0,622315244	0,311567137	0,264832067	0,144482326	0,116482326	**-1,143517674**
chrXI	19266	19389	123	1,929152515	5,89233263	0,246647853	-0,68507641	-0,582314949	0,025296211	0,172296211	**-1,087703789**
chrXI	19267	19389	122	0,065539893	0,207528696	0,240012568	-0,706262136	-0,600322816	0,028135062	0,182135062	**-1,077864938**
chrXI	19267	19414	147	0,09973462	0,118587827	0,456822566	-0,108441943	-0,092175651	-0,165101711	-0,186101711	**-1,446101711**
chrXI	20703	20861	158	0,447381012	0,52993935	0,457762909	-0,106071255	-0,090160566	-0,067894316	-0,165894316	**-1,425894316**
chrXI	31510	31641	131	0,282106498	0,500292393	0,360566076	-0,356946107	-0,303404191	-0,076794249	0,014205751	**-1,245794249**
chrXI	32617	32778	161	0,14817715	0,188999349	0,439464645	-0,152326671	-0,12947767	-0,123199352	-0,242199352	**-1,502199352**
chrXI	38219	38379	160	0,068389454	0,037058696	0,648560019	0,381435717	0,324220359	0,339421729	0,227421729	**-1,032578271**
chrXI	39078	39236	158	0,396088921	0,61146848	0,393117971	-0,271201653	-0,230521405	-0,210674681	-0,308674681	**-1,568674681**
chrXI	41766	41916	150	0,273557816	0,211234566	0,564278289	0,16182531	0,137551513	0,017444382	-0,024555618	**-1,284555618**
chrXI	42476	42618	142	8,534433948	5,770038934	0,596626944	0,244625896	0,207932012	0,102552613	0,116552613	**-1,143447387**
chrXI	42479	42618	139	0,039893848	0,051882174	0,434687048	-0,164453569	-0,139785534	-0,142993666	-0,107993666	**-1,367993666**
chrXI	44129	44285	156	0,153876272	0,211234566	0,421450846	-0,198183249	-0,168455762	-0,179919203	-0,263919203	**-1,523919203**
chrXI	45474	45591	117	0,176672756	0,952408482	0,156474796	-1,009052218	-0,857694386	-0,160710626	0,028289374	**-1,231710626**
chrXI	52845	53010	165	1,344992595	1,66764131	0,446450726	-0,134633754	-0,114438691	-0,033049486	-0,180049486	**-1,440049486**
chrXI	53086	53185	99	0,091185939	1,148819569	0,073536721	-1,449946491	-1,232454517	-0,785695943	-0,470695943	**-1,730695943**
chrXI	55429	55585	156	0,307752543	0,244587392	0,557179598	0,143822286	0,122248943	0,097166489	0,013166489	**-1,246833511**
chrXI	55463	55623	160	0,113982423	0,103764348	0,523463207	0,058847485	0,050020363	0,068429027	-0,043570973	**-1,303570973**
chrXI	55924	56074	150	0,863416857	0,800467829	0,518916283	0,047433872	0,040318791	-0,08374672	-0,12574672	**-1,38574672**
chrXI	55924	56080	156	0,125380666	0,122293696	0,506231912	0,015621722	0,013278464	-0,010459686	-0,094459686	**-1,354459686**
chrXI	56846	56978	132	0,156725832	0,170470001	0,47899703	-0,052670982	-0,044770335	0,136424479	0,220424479	**-1,039575521**
chrXI	61848	62032	184	0,09688506	0,170470001	0,36238349	-0,352095051	-0,299280793	0,011385456	-0,268614544	**-1,528614544**
chrXI	63877	64030	153	0,076938136	0,118587827	0,393493196	-0,270226	-0,2296921	-0,322514067	-0,385514067	**-1,645514067**
chrXI	65154	65316	162	0,210867483	0,155646522	0,575332675	0,189967434	0,161472318	0,169430117	0,043430117	**-1,216569883**
chrXI	65672	65838	166	3,923844923	3,068460011	0,561166162	0,153926517	0,130837539	0,233631803	0,079631803	**-1,180368197**
chrXI	68614	68763	149	1,085682582	0,885702829	0,550720613	0,127482183	0,108359856	0,001605651	-0,033394349	**-1,293394349**
chrXI	73102	73261	159	0,108283302	0,111176087	0,493409293	-0,016521205	-0,014043024	0,010190038	-0,094809962	**-1,354809962**
chrXI	86463	86611	148	0,153876272	0,125999566	0,549801916	0,125160904	0,106386768	-0,006537371	-0,034537371	**-1,294537371**
chrXI	86483	86645	162	1,051487855	1,011702395	0,509641733	0,024170595	0,020545005	0,034350256	-0,091649744	**-1,351649744**
chrXI	88336	88484	148	0,09973462	0,092646739	0,518421434	0,046192108	0,039263292	-0,072316734	-0,100316734	**-1,360316734**
chrXI	89789	89957	168	0,284956058	0,233469783	0,549656355	0,124793175	0,106074199	0,248118621	0,080118621	**-1,179881379**
chrXI	89789	89957	168	0,284956058	0,233469783	0,549656355	0,124793175	0,106074199	0,248118621	0,080118621	**-1,179881379**
chrXI	91032	91184	152	2,481967268	2,116051529	0,539790588	0,099906161	0,084920237	3,61E-05	-0,055963927	**-1,315963927**
chrXI	97596	97707	111	1,70973635	14,67153766	0,104371391	-1,257029976	-1,068475479	-0,378182241	-0,147182241	**-1,407182241**
chrXI	97601	97707	106	0,042743409	0,289057827	0,128822331	-1,131975672	-0,962179321	-0,441094211	-0,175094211	**-1,435094211**
chrXI	99816	99946	130	0,168124074	0,100058479	0,626901611	0,323658255	0,275109516	0,527325968	0,625325968	**-0,634674032**
chrXI	99816	99946	130	0,168124074	0,100058479	0,626901611	0,323658255	0,275109516	0,527325968	0,625325968	**-0,634674032**
chrXI	103049	103197	148	0,347646391	0,274234349	0,559024213	0,148495706	0,12622135	0,005110108	-0,022889892	**-1,282889892**
chrXI	103049	103197	148	0,347646391	0,274234349	0,559024213	0,148495706	0,12622135	0,005110108	-0,022889892	**-1,282889892**
chrXI	103372	103526	154	5,189049823	3,5205761	0,595783317	0,242447586	0,206080448	0,173625353	0,103625353	**-1,156374647**
chrXI	105256	105405	149	0,339097709	0,192705218	0,637637914	0,352152134	0,299329314	0,185161926	0,150161926	**-1,109838074**
chrXI	105988	106134	146	0,276407377	0,100058479	0,734216325	0,625615224	0,531772941	0,441727294	0,427727294	**-0,832272706**
chrXI	110658	110811	153	0,128230226	0,125999566	0,504387095	0,010997038	0,009347483	-0,075077383	-0,138077383	**-1,398077383**
chrXI	115225	115382	157	0,353345512	0,329822392	0,517216207	0,043168034	0,036692829	0,032830238	-0,058169762	**-1,318169762**
chrXI	116126	116262	136	0,031345166	0,070411522	0,308040355	-0,501412691	-0,426200787	-0,320932821	-0,264932821	**-1,524932821**
chrXI	116126	116262	136	0,031345166	0,070411522	0,308040355	-0,501412691	-0,426200787	-0,320932821	-0,264932821	**-1,524932821**
chrXI	116231	116308	77	0,131079787	0,040764565	0,762782048	0,715280226	0,607988192		0,469	**-0,791**
chrXI	116232	116308	76	1,242408414	0,911643916	0,576777266	0,193655717	0,16460736		0,476	**-0,784**
chrXI	116232	116357	125	1,70973635	1,545347614	0,525251075	0,06333738	0,053836773	0,572627202	0,705627202	**-0,554372798**
chrXI	117579	117716	137	0,626903328	0,515115871	0,548942897	0,122991025	0,104542371	0,163653163	0,212653163	**-1,047346837**
chrXI	120342	120499	157	0,44168189	0,363175219	0,548770565	0,122555783	0,104172416	0,097356327	0,006356327	**-1,253643673**
chrXI	120705	120857	152	0,980248841	0,933879134	0,512112489	0,030366173	0,025811247	-0,057594098	-0,113594098	**-1,373594098**
chrXI	121556	121713	157	0,037044288	0,092646739	0,285634931	-0,566182298	-0,481254953	-0,489848996	-0,580848996	**-1,840848996**
chrXI	130706	130855	149	1,441877655	1,156231309	0,554971972	0,138233278	0,117498286	0,000210026	-0,034789974	**-1,294789974**
chrXI	130929	131079	150	0,102584181	0,074117392	0,58055047	0,203301913	0,172806626	0,058158787	0,016158787	**-1,243841213**
chrXI	132985	133151	166	1,179718082	1,100643265	0,517338221	0,043474165	0,03695304	0,148479833	-0,005520167	**-1,265520167**
chrXI	134033	134163	130	0,404637603	1,05246696	0,277699771	-0,589688448	-0,501235181	-0,251200418	-0,153200418	**-1,413200418**
chrXI	136517	136676	159	0,162424953	0,281646088	0,365763444	-0,34309514	-0,291630869	-0,275354503	-0,380354503	**-1,640354503**
chrXI	140618	140768	150	0,316301225	0,285351958	0,525720189	0,064515681	0,054838328	-0,063274442	-0,105274442	**-1,365274442**
chrXI	142672	142804	132	1,695488547	1,285936744	0,568683895	0,173024449	0,147070782	0,302129605	0,386129605	**-0,873870395**
chrXI	145250	145428	178	0,151026711	0,155646522	0,49246786	-0,018881397	-0,016049187	0,279449967	0,041449967	**-1,218550033**
chrXI	146568	146719	151	0,473027057	0,337234132	0,583795773	0,211613657	0,179871608	0,101971149	0,052971149	**-1,207028851**
chrXI	147356	147520	164	0,056991212	0,081529131	0,411428464	-0,223871969	-0,190291174	-0,125104196	-0,265104196	**-1,525104196**
chrXI	149408	149568	160	0,108283302	0,125999566	0,462190442	-0,094916836	-0,080679311	-0,062937275	-0,174937275	**-1,434937275**
chrXI	149964	150108	144	0,316301225	0,307587175	0,506983661	0,017506335	0,014880385	-0,045308002	-0,045308002	**-1,305308002**
chrXI	151755	151890	135	0,054141651	0,137117174	0,283080537	-0,573714411	-0,48765725	-0,37137928	-0,30837928	**-1,56837928**
chrXI	161235	161384	149	0,222265726	0,159352392	0,5824297	0,208113175	0,176896198	0,059452199	0,024452199	**-1,235547801**
chrXI	161799	161928	129	1,8579135	2,616343922	0,415245107	-0,214072894	-0,18196196	0,112655679	0,217655679	**-1,042344321**
chrXI	162802	162952	150	0,498673102	0,522527611	0,488320362	-0,029280693	-0,024888589	-0,147849305	-0,189849305	**-1,449849305**
chrXI	170837	170984	147	0,082637257	0,26311674	0,239005934	-0,709503843	-0,603078266	-0,675666349	-0,696666349	**-1,956666349**
chrXI	171758	171891	133	0,14817715	0,200116957	0,425436857	-0,188003748	-0,159803185	-0,027196837	0,049803163	**-1,210196837**
chrXI	172320	172456	136	1,134125112	1,178466526	0,490413047	-0,02403324	-0,020428254	0,075219961	0,131219961	**-1,128780039**

Chrom	Start	End	Length	Section A	Section B	A/A+B	Z-score	Z * 0.85	Phase correction	Length correction	ΔLKnuc
chrXI	178039	178227	188	0,139628469	0,277940218	0,334384433	-0,427838279	-0,363662537	-0,052970865	-0,360970865	-1,620970865
chrXI	178982	179138	156	0,119681545	0,133411305	0,472876041	-0,06804215	-0,057835827	-0,07586694	-0,15986694	-1,41986694
chrXI	179058	179209	151	0,042743409	0,092646739	0,315705458	-0,47974192	-0,407780632	-0,492591915	-0,541591915	-1,801591915
chrXI	179058	179209	151	0,042743409	0,092646739	0,315705458	-0,47974192	-0,407780632	-0,492591915	-0,541591915	-1,801591915
chrXI	182120	182298	178	0,202318801	0,185293479	0,521961795	0,05507789	0,046816206	0,355868288	0,117868288	-1,142131712
chrXI	183536	183690	154	0,284956058	0,296469566	0,490098899	-0,024820929	-0,021097789	-0,053518065	-0,123518065	-1,383518065
chrXI	183964	184081	117	0,458779254	2,171639573	0,174413006	-0,936868875	-0,796338543	-0,102982643	0,086017357	-1,173982643
chrXI	187208	187352	144	1,208213687	0,844938264	0,588467739	0,223605195	0,190064415	0,117481947	0,117481947	-1,142518053
chrXI	188029	188179	150	0,718089267	0,515115871	0,582295066	0,207768321	0,176603073	0,047590992	0,005590992	-1,254409008
chrXI	190331	190479	148	0,364743755	0,322410653	0,530803194	0,077289038	0,065695682	-0,054896657	-0,082896657	-1,342896657
chrXI	191024	191171	147	0,3903898	0,270528479	0,590677868	0,229289049	0,194895692	0,11557827	0,09457827	-1,16542173
chrXI	195322	195449	127	0,128230226	0,370586958	0,257068582	-0,652409303	-0,554547908	-0,166366987	-0,047366987	-1,307366987
chrXI	196316	196481	165	2,912250916	3,009166098	0,491816555	-0,020514294	-0,017437149	0,064685247	-0,082314753	-1,342314753
chrXI	196620	196752	132	0,527168708	0,678174133	0,437359969	-0,157666069	-0,134016159	0,003751455	0,087751455	-1,172248545
chrXI	201937	202099	162	0,088336378	0,155646522	0,36205971	-0,352958678	-0,300014876	-0,271529265	-0,397529265	-1,657529265
chrXI	204817	204983	166	0,133929347	0,188999349	0,414733497	-0,215385217	-0,183077435	-0,071847362	-0,225847362	-1,485847362
chrXI	207618	207770	152	0,971700159	0,589233263	0,622512239	0,312085531	0,265272701	0,180054768	0,124054768	-1,135945232
chrXI	209750	209876	126	0,595558162	1,84552305	0,243973105	-0,693579092	-0,589542228	-0,123680546	0,002319454	-1,257680546
chrXI	214868	215018	150	0,054141651	0,122293696	0,30686397	-0,50475925	-0,429045363	-0,555026937	-0,597026937	-1,857026937
chrXI	214868	215019	151	0,658248495	0,607762611	0,519938958	0,050000381	0,042500324	-0,040297145	-0,089297145	-1,349297145
chrXI	214886	215018	132	0,492973981	0,807879568	0,378961937	-0,308208251	-0,261977013	-0,111451285	-0,027451285	-1,287451285
chrXI	214886	215019	133	0,153876272	0,181587609	0,458696987	-0,10371695	-0,088159408	0,037749368	0,114749368	-1,145250632
chrXI	216300	216452	152	0,772230918	0,718938698	0,517869268	0,044806601	0,038085611	-0,035795978	-0,091795978	-1,351795978
chrXI	216511	216657	146	1,131275552	0,633703698	0,640956857	0,361017604	0,306864964	0,218566995	0,204566995	-1,055433005
chrXI	226833	226983	150	0,555664314	0,52623348	0,513601485	0,034100475	0,028985404	-0,094144118	-0,136144118	-1,396144118
chrXI	226930	227062	132	7,286326412	8,786616771	0,453328698	-0,117255741	-0,09966738	0,061094685	0,145094685	-1,114905315
chrXI	227206	227326	120	0,236513528	1,389701092	0,145438078	-1,056201522	-0,897771294	-0,215397937	-0,047397937	-1,307397937
chrXI	229813	229973	160	0,3903898	0,381704567	0,505624464	0,014098907	0,011984071	0,016770907	-0,095229093	-1,355229093
chrXI	233634	233784	150	0,359044634	0,289057827	0,553993628	0,135757811	0,11539414	-0,011879396	-0,053879396	-1,313879396
chrXI	233634	233784	150	0,359044634	0,289057827	0,553993628	0,135757811	0,11539414	-0,011879396	-0,053879396	-1,313879396
chrXI	236594	236764	170	0,19377012	0,26311674	0,424109636	-0,191390999	-0,162682349	-0,026111303	-0,208111303	-1,468111303
chrXI	241460	241610	150	0,310602104	0,17788174	0,635849287	0,347385899	0,295278014	0,173541198	0,131541198	-1,128458802
chrXI	245174	245347	173	0,088336378	0,148234783	0,373402986	-0,322853792	-0,274425723	-0,071240295	-0,274240295	-1,534240295
chrXI	245189	245339	150	0,609805965	0,463233697	0,568297693	0,172041869	0,146235588	0,01372297	-0,02827703	-1,28827703
chrXI	245551	245725	174	0,102584181	0,17788174	0,365763444	-0,34309514	-0,291630869	-0,046690766	-0,256690766	-1,516690766
chrXI	247204	247369	165	0,492973981	0,585527393	0,457091658	-0,107763477	-0,091598955	-0,008912496	-0,155912496	-1,415912496
chrXI	247204	247369	165	0,492973981	0,585527393	0,457091658	-0,107763477	-0,091598955	-0,008912496	-0,155912496	-1,415912496
chrXI	249638	249773	135	0,045592969	0,092646739	0,329810947	-0,440435256	-0,374369967	-0,257078261	-0,194078261	-1,454078261
chrXI	251126	251263	137	0,045592969	0,122293696	0,271569926	-0,608071803	-0,516861033	-0,458708437	-0,409708437	-1,669708437
chrXI	253186	253299	113	0,330549028	2,208698269	0,130175989	-1,125559597	-0,956725658	-0,261056574	-0,044056574	-1,304056574
chrXI	253541	253688	147	0,142478029	0,188999349	0,429827309	-0,17681384	-0,150291764	-0,239689262	-0,260689262	-1,520689262
chrXI	257352	257460	108	0,039893848	0,048176305	0,452978074	-0,118140734	-0,100419624	0,566956687	0,818956687	-0,441043313
chrXI	257353	257460	107	0,407487163	0,937585003	0,302948179	-0,51593994	-0,438548949	0,127014847	0,386014847	-0,873985153
chrXI	257353	257478	125	0,755133555	1,682464789	0,309785883	-0,496457358	-0,421988754	0,071761483	0,204761483	-1,055238517
chrXI	257617	257767	150	1,188266763	0,715232829	0,624253753	0,316672003	0,269171202	0,136265898	0,094265898	-1,165734102
chrXI	258177	258336	159	0,324849907	0,314998914	0,507679906	0,019296987	0,016402439	0,032865901	-0,072134099	-1,332134099
chrXI	261407	261543	136	0,091185939	0,066705652	0,577522451	0,195559327	0,166225428	0,243684279	0,299684279	-0,960315721
chrXI	269387	269539	152	0,347646391	0,340940001	0,504869679	0,012206779	0,010375762	-0,068663016	-0,124663016	-1,384663016
chrXI	269387	269539	152	0,347646391	0,340940001	0,504869679	0,012206779	0,010375762	-0,068663016	-0,124663016	-1,384663016
chrXI	270529	270668	139	0,646850252	0,544762828	0,542835811	0,10758061	0,091443518	0,083212133	0,118212133	-1,141787867
chrXI	275707	275869	162	4,943987612	5,784862412	0,460812445	-0,098387135	-0,083629065	-0,043772106	-0,169772106	-1,429772106
chrXI	275877	276030	153	0,102584181	0,059293913	0,633712557	0,341702373	0,290447017	0,205647948	0,142647948	-1,117352052
chrXI	277324	277474	150	0,122531105	0,125999566	0,493022067	-0,017491975	-0,014868179	-0,142413256	-0,184413256	-1,444413256
chrXI	278497	278637	140	20,10934904	14,40471505	0,582642165	0,208657437	0,177358821	0,143641681	0,171641681	-1,088358319
chrXI	278499	278637	138	0,04844253	0,037058696	0,566571175	0,16765125	0,142503563	0,146950165	0,188950165	-1,071049835
chrXI	278501	278637	136	0,056991212	0,051882174	0,523463207	0,058847485	0,050020363	0,12053464	0,17653464	-1,08346536
chrXI	278502	278637	135	0,09688506	0,062999783	0,605967759	0,268824834	0,228501109	0,340239586	0,403239586	-0,856760414
chrXI	282993	283156	163	0,14817715	0,181587609	0,449341981	-0,127324005	-0,108225404	-0,048195826	-0,181195826	-1,441195826
chrXI	286651	286824	173	0,914708947	1,204407613	0,431646359	-0,172184202	-0,146356571	0,066114029	-0,136885971	-1,396885971
chrXI	287603	287693	90	0,028495606	0,070411522	0,288104674	-0,558930214	-0,475090682	-0,110563336	0,267436664	-0,992563336
chrXI	288326	288474	148	0,185221438	0,17788174	0,510106904	0,025336963	0,021536418	-0,095497141	-0,123497141	-1,383497141
chrXI	290076	290228	152	3,66168535	3,650281535	0,500779805	0,001954682	0,00166148	-0,074011365	-0,130011365	-1,390011365
chrXI	292965	293112	147	0,082637257	0,062999783	0,56741923	0,169807485	0,144336362	0,036694373	0,015694373	-1,244305627
chrXI	297006	297125	119	2,131471316	8,834793075	0,194366216	-0,861918383	-0,732630625	-0,072503768	0,102496232	-1,157503768
chrXI	297006	297156	150	0,644000692	0,300175436	0,682076864	0,47351434	0,402487189	0,277234288	0,235234288	-1,024765712
chrXI	298702	298832	130	0,276407377	0,418763262	0,397610833	-0,259536057	-0,220605649	0,010576411	0,108576411	-1,151423589
chrXI	298875	299007	132	0,085486818	0,218646305	0,281083549	-0,579625641	-0,492681795	-0,325964606	-0,241964606	-1,501964606
chrXI	300199	300305	106	0,378991558	1,363760005	0,217467346	-0,780775251	-0,663658963	-0,128868376	0,137131624	-1,122868376
chrXI	300199	300317	118	0,284956058	1,574994571	0,153206249	-1,022778687	-0,869361884	-0,211205404	-0,029205404	-1,289205404
chrXI	303921	304068	147	0,170973635	0,17788174	0,490098899	-0,024820929	-0,021097789	-0,139259895	-0,160259895	-1,420259895
chrXI	305787	305949	162	0,136778908	0,092646739	0,596179675	0,243470869	0,206950239	0,255257197	0,129257197	-1,130742803
chrXI	318927	319072	145	0,113982423	0,166764131	0,405997586	-0,23785292	-0,202174982	-0,280433426	-0,287433426	-1,547433426
chrXI	319054	319202	148	0,339097709	0,359469349	0,485418981	-0,036557337	-0,031073736	-0,13825176	-0,16625176	-1,42625176
chrXI	322500	322670	170	0,225115286	0,544762828	0,2924038	-0,54637586	-0,464419481	-0,32712977	-0,50912977	-1,76912977
chrXI	329617	329749	132	0,208017923	0,237175653	0,46725275	-0,082177582	-0,069850944	0,0872634	0,1712634	-1,0887366
chrXI	329629	329749	120	0,037044288	0,062999783	0,370279692	-0,331112667	-0,281445767	0,390026352	0,558026352	-0,701973648
chrXI	329629	329775	146	0,712390146	0,796761959	0,472046617	-0,070126174	-0,059607248	-0,142694403	-0,156694403	-1,416694403
chrXI	337370	337525	155	1,923453394	2,004875442	0,489636554	-0,025980229	-0,022083195	-0,048232608	-0,125232608	-1,385232608
chrXI	337447	337600	153	8,705407583	6,967034808	0,555459536	0,139467258	0,118547169	0,035492035	-0,027507965	-1,287507965
chrXI	337450	337600	150	0,037044288	0,037058696	0,499902783	-0,000243688	-0,000207135	-0,127669448	-0,169669448	-1,429669448
chrXI	337452	337600	148	0,108283302	0,129705435	0,454993389	-0,113055218	-0,096096936	-0,196611305	-0,224611305	-1,484611305
chrXI	337462	337600	138	0,039893848	0,037058696	0,518421434	0,046192108	0,039263292	0,036098418	0,078098418	-1,181901582
chrXI	337968	338118	150	0,188070999	0,151940653	0,553131041	0,13357593	0,113539541	-0,011410235	-0,053410235	-1,313410235
chrXI	340261	340401	140	0,227964847	0,181587609	0,556619411	0,142403651	0,121043103	0,07029509	0,09829509	-1,16170491
chrXI	341684	341830	146	0,860567296	0,674468263	0,560617173	0,152534155	0,129654032	0,051995378	0,037995378	-1,222004622
chrXI	347279	347450	171	0,051292091	0,066705652	0,434687048	-0,164453569	-0,139785534	0,038800622	-0,150199378	-1,410199378
chrXI	347320	347467	147	0,558513874	0,689291742	0,447596859	-0,131735231	-0,111974946	-0,2327025	-0,2537025	-1,5137025
chrXI	347479	347608	129	0,065539893	0,100058479	0,395776194	-0,264295292	-0,224650998	0,050872762	0,155872762	-1,104127238
chrXI	351244	351421	177	0,176672756	0,322410653	0,353994448	-0,374558427	-0,318374663	-0,05369598	-0,28469598	-1,54469598
chrXI	351885	352030	145	0,803576085	0,541056958	0,597617386	0,247184775	0,210107059	0,131848615	0,124848615	-1,135151385
chrXI	352745	352882	137	0,208017923	0,181587609	0,533919325	0,085125837	0,072356961	0,127933562	0,176933562	-1,083066438
chrXI	353147	353287	140	0,435982769	0,344645871	0,558502144	0,147172696	0,125096791	0,081551487	0,109551487	-1,150448513
chrXI	355288	355448	160	0,552814753	0,463233697	0,544083063	0,11072569	0,094116836	0,098966096	-0,013033904	-1,273033904
chrXI	357460	357614	154	1,587205245	1,641700223	0,491561385	-0,021154048	-0,017980941	-0,052746132	-0,122746132	-1,382746132
chrXI	357462	357614	152	0,031345166	0,055588044	0,360566076	-0,356946107	-0,303404191	-0,376727786	-0,432727786	-1,692727786
chrXI	362917	363070	153	0,262159574	0,303881305	0,463146008	-0,092511045	-0,078634388	-0,15123929	-0,21423929	-1,47423929

Chrom	Start	End	Length	Section A	Section B	A/A+B	Z-score	Z * 0.85	Phase correction	Length correction	ΔLKnuc
chrXI	364062	364210	148	0,364743755	0,466939567	0,438560862	-0,154618956	-0,131426113	-0,23775083	-0,26575083	**-1,52575083**
chrXI	364602	364723	121	2,020338454	4,250632407	0,322173153	-0,461630518	-0,39238594	0,252526211	0,413526211	**-0,846473789**
chrXI	368064	368220	156	0,361894194	0,43729261	0,45282804	-0,118519456	-0,100741538	-0,115457895	-0,199457895	**-1,459457895**
chrXI	368389	368522	133	1,951949	2,594108705	0,429371804	-0,177973727	-0,151277668	-0,022237908	0,054762092	**-1,205237908**
chrXI	368971	369078	107	0,076938136	0,222352175	0,257068582	-0,652409303	-0,554547908	0,030230289	0,289230289	**-0,970769711**
chrXI	368971	369121	150	0,059840772	0,051882174	0,535617563	0,08939893	0,07598909	-0,04670179	-0,08870179	**-1,34870179**
chrXI	368971	369122	151	11,09333935	9,865024818	0,529303683	0,073519618	0,062491675	-0,022130205	-0,071130205	**-1,331130205**
chrXI	372978	373087	109	1,66129382	15,76847506	0,095313589	-1,308726012	-1,11241711	-0,415283221	-0,170283221	**-1,430283221**
chrXI	373360	373511	151	0,279256937	0,203822827	0,578076247	0,196974484	0,167428312	0,080158728	0,031158728	**-1,228841272**
chrXI	377591	377751	160	0,059840772	0,066705652	0,472876041	-0,06804215	-0,057835827	-0,051960104	-0,163960104	**-1,423960104**
chrXI	379254	379407	153	0,424584527	0,503998263	0,457239281	-0,107391293	-0,091282599	-0,157716649	-0,220716649	**-1,480716649**
chrXI	385681	385837	156	0,276407377	0,285351958	0,492038779	-0,019957148	-0,016963575	-0,026355021	-0,110355021	**-1,370355021**
chrXI	394646	394790	144	0,53571739	0,411351523	0,565658298	0,165331072	0,140531411	0,072557241	0,072557241	**-1,187442759**
chrXI	397867	398011	144	0,133929347	0,062999783	0,680089062	0,46794786	0,397755681	0,321993033	0,321993033	**-0,938006967**
chrXI	397867	398011	144	0,133929347	0,062999783	0,680089062	0,46794786	0,397755681	0,321993033	0,321993033	**-0,938006967**
chrXI	399301	399460	159	0,404637603	0,403939784	0,50043151	0,001081636	0,000919391	0,015340948	-0,089659052	**-1,349659052**
chrXI	400364	400506	142	0,552814753	0,459527828	0,546074781	0,115750303	0,098387758	-0,000531614	0,013468386	**-1,246531614**
chrXI	401469	401625	156	0,812124766	0,722644568	0,529151025	0,07313593	0,062165541	0,050081969	-0,033918031	**-1,293918031**
chrXI	401694	401852	158	0,313451664	0,289057827	0,520243529	0,050764798	0,043150079	0,060076725	-0,037923275	**-1,297923275**
chrXI	403288	403463	175	0,373292436	0,696703481	0,348872767	-0,38836555	-0,330110718	-0,069859788	-0,286859788	**-1,546859788**
chrXI	406796	406955	159	0,136778908	0,137117174	0,499382491	-0,001547865	-0,001315685	0,019417289	-0,085582711	**-1,345582711**
chrXI	407916	408065	149	0,076938136	0,059293913	0,564757972	0,163043664	0,138587115	0,008281562	-0,026718438	**-1,286718438**
chrXI	416183	416327	144	0,076938136	0,037058696	0,674914686	0,453525163	0,385496389	0,306335025	0,306335025	**-0,953664975**
chrXI	417925	418084	159	1,094231264	1,067290439	0,506231912	0,015621722	0,013278464	0,028843489	-0,076156511	**-1,336156511**
chrXI	417925	418084	159	1,094231264	1,067290439	0,506231912	0,015621722	0,013278464	0,028843489	-0,076156511	**-1,336156511**
chrXI	434342	434496	154	0,450230572	0,448410219	0,501012837	0,002538808	0,002157987	-0,020534294	-0,090534294	**-1,350534294**
chrXI	434529	434684	155	0,265009134	0,070411522	0,790079947	0,806698677	0,685693876	0,650303421	0,573303421	**-0,686696579**
chrXI	434561	434692	131	0,122531105	0,070411522	0,635064977	0,345298405	0,293503645	0,528537142	0,619537142	**-0,640462858**
chrXI	434662	434792	130	0,062690333	0,085235	0,423797138	-0,192188854	-0,163360526	0,073698243	0,171698243	**-1,088301757**
chrXI	439672	439806	134	8,297920419	7,1263872	0,53797685	0,095338075	0,081037364	0,188557019	0,258557019	**-1,001442981**
chrXI	439682	439780	98	0,045592969	0,040764565	0,527955893	0,070132481	0,059612609	0,491175723	0,813175723	**-0,446824277**
chrXI	447937	448094	157	0,071239015	0,100058479	0,415878909	-0,212447646	-0,180580499	-0,187607083	-0,278607083	**-1,538607083**
chrXI	448453	448602	149	0,188070999	0,155646522	0,547167331	0,118507771	0,100731606	-0,018032092	-0,053032092	**-1,313032092**
chrXI	450755	450913	158	1,61285129	0,889408699	0,644557839	0,370668677	0,315068376	0,335707304	0,237707304	**-1,022292696**
chrXI	454604	454760	156	0,068389454	0,070411522	0,492715945	-0,018259434	-0,015520519	-0,022766752	-0,106766752	**-1,366766752**
chrXI	455031	455138	107	0,997346204	3,272282838	0,233590833	-0,727072301	-0,618011456	-0,012180878	0,246819122	**-1,013180878**
chrXI	455031	455148	117	1,333594353	5,577333716	0,192968924	-0,867007596	-0,736956456	-0,066213638	0,122786362	**-1,137213638**
chrXI	456736	456888	152	0,24221265	0,300175436	0,446567054	-0,134339514	-0,114188587	-0,178743672	-0,234743672	**-1,494743672**
chrXI	459229	459336	107	0,045592969	0,655938915	0,064990587	-1,514176126	-1,287049707	-0,655623344	-0,396623344	**-1,656623344**
chrXI	459229	459341	112	0,09973462	0,830114786	0,107258896	-1,241238134	-1,055052414	-0,347589223	-0,123589223	**-1,383589223**
chrXI	462337	462501	164	0,233663968	0,163058261	0,588986325	0,224938202	0,191197471	0,264660202	0,124660202	**-1,135339798**
chrXI	468677	468825	148	0,14817715	0,170470001	0,465019536	-0,087795679	-0,074626327	-0,179080651	-0,207080651	**-1,467080651**
chrXI	470132	470274	142	0,165274514	0,196411088	0,456956299	-0,108104753	-0,09188904	-0,174480946	-0,160480946	**-1,420480946**
chrXI	473335	473488	153	0,128230226	0,08894087	0,590457149	0,22872109	0,194412926	0,146961619	0,083961619	**-1,176038381**
chrXI	479959	480086	127	0,09973462	0,429880871	0,188315149	-0,88412211	-0,751503794	-0,403628482	-0,284628482	**-1,544628482**
chrXI	483328	483476	148	0,068389454	0,044470435	0,605967759	0,268824834	0,228501109	0,128183725	0,100183725	**-1,159816275**
chrXI	484474	484623	149	1,117027749	0,596645002	0,651832591	0,390272821	0,331731898	0,216750513	0,181750513	**-1,078249487**
chrXI	488055	488207	152	0,082637257	0,226058044	0,267698461	-0,619788682	-0,52682038	-0,603810319	-0,659810319	**-1,919810319**
chrXI	488418	488598	180	0,350495952	0,874585221	0,286100186	-0,564813869	-0,480091788	-0,164439491	-0,416439491	**-1,676439491**
chrXI	496967	497107	140	0,028495606	0,044470435	0,390532439	-0,277931585	-0,236241848	-0,300335667	-0,272335667	**-1,532335667**
chrXI	501192	501346	154	0,190920559	0,085235	0,691351496	0,499684841	0,424732115	0,398969242	0,328969242	**-0,931030758**
chrXI	506644	506790	146	0,473027057	0,363175219	0,565684967	0,165398841	0,140589015	0,069343434	0,055343434	**-1,204656566**
chrXI	509826	509992	166	0,208017923	0,270528479	0,434687048	-0,164453569	-0,139785534	-0,019164296	-0,173164296	**-1,433164296**
chrXI	514096	514200	104	0,082637257	1,815876094	0,043527351	-1,711143087	-1,454471624	-0,961858161	-0,681858161	**-1,941858161**
chrXI	516175	516336	161	0,085486818	0,100058479	0,460732874	-0,09858756	-0,083799426	-0,077654672	-0,196654672	**-1,456654672**
chrXI	517302	517453	151	0,199469241	0,163058261	0,550218231	0,126212725	0,107280816	0,025131321	-0,023868679	**-1,283868679**
chrXI	517302	517453	151	0,199469241	0,163058261	0,550218231	0,126212725	0,107280816	0,025131321	-0,023868679	**-1,283868679**
chrXI	517458	517572	114	0,068389454	0,281646088	0,1953786	-0,858244988	-0,72950824	-0,021799122	0,188200878	**-1,071799122**
chrXI	519553	519695	142	0,495823542	1,637994354	0,232364506	-0,731082091	-0,621419778	-0,708964418	-0,694964418	**-1,954964418**
chrXI	527505	527640	135	0,042743409	0,163058261	0,207692235	-0,814454777	-0,692286561	-0,579897392	-0,516897392	**-1,776897392**
chrXI	527506	527600	94	0,028495606	0,048176305	0,371656395	-0,327469522	-0,278349094	0,128260389	0,478260389	**-0,781739611**
chrXI	527506	527630	124	0,059840772	0,637409568	0,085823941	-1,366927974	-1,161888778	-0,608056172	-0,468056172	**-1,728056172**
chrXI	527506	527632	126	0,037044288	0,059293913	0,384523349	-0,293622086	-0,249578773	0,191353175	0,317353175	**-0,942646825**
chrXI	527506	527635	129	0,059840772	0,085235	0,412479432	-0,221171546	-0,187995814	0,087041228	0,192041228	**-1,067958772**
chrXI	527506	527636	130	0,037044288	0,077823261	0,322495674	-0,460731366	-0,391621661	-0,149221893	-0,051221893	**-1,311221893**
chrXI	527506	527637	131	0,111132863	0,185293479	0,374908863	-0,318879716	-0,271047759	-0,051788904	0,039211096	**-1,220788904**
chrXI	527506	527638	132	0,042743409	0,096352609	0,307294266	-0,503534497	-0,428004322	-0,258465716	-0,174465716	**-1,434465716**
chrXI	527506	527639	133	0,034194727	0,103764348	0,247861382	-0,681235065	-0,579049806	-0,45629572	-0,37929572	**-1,63929572**
chrXI	527506	527640	134	36,15237512	35,83946469	0,502173235	0,00544752	0,004630392	0,115925049	0,185925049	**-1,074074951**
chrXI	527506	527641	135	0,094035499	0,318704784	0,227832133	-0,746005211	-0,63410443	-0,528193084	-0,465193084	**-1,725193084**
chrXI	527507	527640	133	0,054141651	0,166764131	0,245089334	-0,690024678	-0,586520976	-0,46819413	-0,39119413	**-1,65119413**
chrXI	527508	527640	132	0,042743409	0,092646739	0,315705458	-0,47974192	-0,407780632	-0,232614796	-0,148614796	**-1,408614796**
chrXI	527509	527640	131	0,136778908	0,08894087	0,605967759	0,268824834	0,228501109	0,437710722	0,528710722	**-0,731289278**
chrXI	527510	527640	130	0,074088575	0,103764348	0,416572153	-0,210670615	-0,179070023	0,054984012	0,152984012	**-1,107015988**
chrXI	527511	527640	129	0,091185939	0,196411088	0,31706148	-0,47593181	-0,404542039	-0,136822961	-0,031822961	**-1,291822961**
chrXI	527513	527640	127	0,059840772	0,08894087	0,402205349	-0,247642923	-0,210496484	0,143233897	0,262233897	**-0,997766103**
chrXI	527515	527640	125	0,037044288	0,085235	0,302948179	-0,51593994	-0,438548949	0,041073689	0,174073689	**-1,085926311**
chrXI	527516	527640	124	0,037044288	0,037058696	0,499902783	-0,000243688	-0,000207135	0,539444463	0,679444463	**-0,580555537**
chrXI	529083	529220	137	1,886409106	1,423053918	0,570004588	0,176385844	0,149927968	0,196971017	0,245971017	**-1,014028983**
chrXI	529403	529549	146	1,396284686	0,78935022	0,63884626	0,355376596	0,302070107	0,226784138	0,212784138	**-1,047215862**
chrXI	532781	532933	152	0,276407377	0,125999566	0,686885209	0,487040568	0,413984483	0,342205913	0,286205913	**-0,973794087**
chrXI	533171	533320	149	0,581310359	0,415057393	0,58342952	0,210674902	0,179073667	0,064852224	0,029852224	**-1,230147776**
chrXI	540415	540570	155	0,071239015	0,100058479	0,415878909	-0,212447646	-0,180580499	-0,208139632	-0,285139632	**-1,545139632**
chrXI	540630	540787	157	0,344796831	0,415057393	0,453767078	-0,116149378	-0,098726971	-0,105999207	-0,196999207	**-1,456999207**
chrXI	540646	540797	151	0,108283302	0,092646739	0,538910465	0,097689227	0,083035843	0,005067737	-0,043932263	**-1,303932263**
chrXI	541723	541874	151	0,102584181	0,051882174	0,664119905	0,423733486	0,360173463	0,264218048	0,215218048	**-1,044781952**
chrXI	542290	542416	126	0,564212996	1,615759136	0,258816609	-0,64699803	-0,549948325	-0,122873632	0,003126368	**-1,256873632**
chrXI	542290	542440	150	0,202318801	0,103764348	0,660992942	0,415174565	0,35289838	0,238459261	0,196459261	**-1,063540739**
chrXI	544676	544823	147	0,276407377	0,114881957	0,706401511	0,542902438	0,461467072	0,341068724	0,320068724	**-0,939931276**
chrXI	545722	545853	131	0,170973635	0,170470001	0,500737507	0,001848658	0,001571359	0,196877991	0,287877991	**-0,972122009**
chrXI	547817	547975	158	0,954602795	1,037643482	0,479159031	-0,052264347	-0,044424695	-0,020496788	-0,118496788	**-1,378496788**
chrXI	548333	548481	148	0,97739928	0,674468263	0,591693495	0,231903444	0,197117927	0,099937219	0,071937219	**-1,188062781**
chrXI	548769	548931	162	0,14532759	0,08894087	0,620346375	0,30639062	0,260432027	0,317153766	0,191153766	**-1,068846234**
chrXI	548769	548931	162	0,14532759	0,08894087	0,620346375	0,30639062	0,260432027	0,317153766	0,191153766	**-1,068846234**
chrXI	549757	549907	150	0,168124074	0,192705218	0,465937988	-0,085484805	-0,072662084	-0,175039257	-0,217039257	**-1,477039257**
chrXI	550284	550422	138	0,031345166	0,040764565	0,434687048	-0,164453569	-0,139785534	-0,137780273	-0,095780273	**-1,355780273**

Chrom	Start	End	Length	Section A	Section B	A/A+B	Z-score	Z * 0.85	Phase correction	Length correction	ΔLKnuc
chrXI	554400	554551	151	0,253610892	0,192705218	0,568231543	0,171873585	0,146092547	0,047898088	-0,001101912	-1,261101912
chrXI	558933	559085	152	0,233663968	0,251999131	0,481123578	-0,047333843	-0,040233767	-0,124657893	-0,180657893	-1,440657893
chrXI	562000	562173	173	0,344796831	0,492880654	0,411610479	-0,223404172	-0,189893546	0,048865595	-0,154134405	-1,414134405
chrXI	571010	571152	142	0,168124074	0,211234566	0,443179768	-0,142912183	-0,121475355	-0,180668893	-0,166668893	-1,426668893
chrXI	573165	573306	141	0,418885406	0,244587392	0,631352796	0,335438395	0,285122636	0,20166148	0,22266148	-1,03733852
chrXI	574030	574178	148	0,854868175	0,592939133	0,590457149	0,22872109	0,194412926	0,111159116	0,083159116	-1,176840884
chrXI	574402	574555	153	5,117810808	3,446458708	0,597577038	0,2470805	0,210018425	0,164114661	0,101114661	-1,158885339
chrXI	575707	575877	170	0,04844253	0,137117174	0,26106169	-0,640075711	-0,544064354	-0,372578963	-0,554578963	-1,814578963
chrXI	577254	577388	134	0,170973635	0,170470001	0,500737507	0,001848658	0,001571359	0,10510824	0,17510824	-1,08489176
chrXI	577351	577483	132	1,037240052	1,615759136	0,390968854	-0,276794756	-0,235275543	-0,072560245	0,011439755	-1,248560245
chrXI	577690	577817	127	0,44168189	8,871851771	0,047423664	-1,670364202	-1,419809571	-1,091113062	-0,972113062	-2,232113062
chrXI	578476	578613	137	0,646850252	0,507704132	0,560259665	0,151627591	0,128883452	0,157936971	0,206936971	-1,053063029
chrXI	579137	579295	158	0,162424953	0,144528914	0,529151025	0,07313593	0,062165541	0,091240397	-0,006759603	-1,266759603
chrXI	590716	590822	106	0,416035845	1,267407396	0,247133871	-0,68353674	-0,581006229	-0,021979271	0,244020729	-1,015979271
chrXI	590716	590835	119	0,521469587	1,430465657	0,267155167	-0,621439735	-0,528223775	0,156434597	0,331434597	-0,928565403
chrXI	600361	600514	153	0,48727486	0,43729261	0,527030071	0,067806264	0,057635325	0,014242579	-0,048757421	-1,308757421
chrXI	600361	600514	153	0,48727486	0,43729261	0,527030071	0,067806264	0,057635325	0,014242579	-0,048757421	-1,308757421
chrXI	601868	602039	171	0,131079787	0,270528479	0,326387174	-0,449911372	-0,382424667	-0,197568101	-0,386568101	-1,646568101
chrXI	602067	602222	155	0,282106498	0,259410871	0,520955586	0,052552043	0,044669236	0,018358273	-0,058641727	-1,318641727
chrXI	603803	603944	141	2,128621756	1,367465874	0,608858238	0,276344444	0,234892777	0,133224849	0,154224849	-1,105775151
chrXI	604749	604856	107	0,128230226	0,674468263	0,159748932	-0,995490292	-0,846166748	-0,206103495	0,052896505	-1,207103495
chrXI	604749	604865	116	0,09973462	0,774526742	0,114078724	-1,205118789	-1,024350971	-0,336489492	-0,140489492	-1,400489492
chrXI	607498	607646	148	0,720938828	0,359469349	0,667283757	0,432425091	0,367561327	0,281278491	0,253278491	-1,006721509
chrXI	607998	608143	145	0,609805965	0,35205761	0,633983842	0,342423354	0,291059851	0,206151235	0,199151235	-1,060848765
chrXI	613167	613294	127	0,812124766	1,960405007	0,292918321	-0,544879143	-0,463147271	-0,151647269	-0,032647269	-1,292647269
chrXI	614331	614461	130	0,373292436	0,392822175	0,487254036	-0,031954832	-0,027161607	0,199710646	0,297710646	-0,962289354
chrXI	618644	618800	156	0,319150785	0,300175436	0,515319349	0,038409356	0,032647953	0,030685073	-0,053314927	-1,313314927
chrXI	621479	621632	153	0,037044288	0,040764565	0,47609348	-0,059960669	-0,050966568	-0,094530231	-0,157530231	-1,417530231
chrXI	624990	625141	151	0,188070999	0,044470435	0,808763392	0,873348384	0,742346126	0,64900238	0,60000238	-0,65999762
chrXI	632085	632240	155	0,239363089	0,17417587	0,578816297	0,198866205	0,169036274	0,154632923	0,077632923	-1,182367077
chrXI	632617	632753	136	0,151026711	0,151940653	0,498491683	-0,0037808	-0,00321368	0,054140796	0,110140796	-1,149859204
chrXI	638182	638313	131	0,056991212	0,044470435	0,561702018	0,155285859	0,13199298	0,306258774	0,397258774	-0,862741226
chrXI	638182	638316	134	6,246236799	5,27345241	0,542222684	0,106034937	0,090129697	0,202873744	0,272873744	-0,987126256
chrXI	643087	643244	157	0,085486818	0,114881957	0,426647404	-0,184916144	-0,157178722	-0,156185576	-0,247185576	-1,507185576
chrXI	646189	646315	126	0,085486818	0,181587609	0,320086122	-0,467457987	-0,397339289	0,002489349	0,128489349	-1,131510651
chrXI	647330	647482	152	0,088336378	0,055588044	0,613769206	0,28915654	0,245783059	0,155678929	0,099678929	-1,160321071
chrXI	648395	648512	117	0,034194727	0,626291959	0,051772016	-1,627909715	-1,383723258	-0,72710144	-0,53810144	-1,79810144
chrXI	650461	650612	151	0,378991558	0,300175436	0,558024111	0,145961504	0,124067278	0,040387547	-0,008612453	-1,268612453
chrXI	651142	651292	150	0,720938828	0,507704132	0,58677651	0,219260691	0,186371588	0,091582902	0,049582902	-1,210417098
chrXI	654048	654197	149	0,185221438	0,129705435	0,588141101	0,222765786	0,189350918	0,067288598	0,032288598	-1,227711402
chrXI	655704	655870	166	0,131079787	0,181587609	0,419230749	-0,203861798	-0,173282528	-0,041669477	-0,195669477	-1,455669477
chrXI	655763	655920	157	0,094035499	0,077823261	0,547167331	0,118507771	0,100731606	0,102618568	0,011618568	-1,248381432
chrXI	657176	657328	152	0,396088921	0,292763697	0,574998063	0,189113484	0,160746461	0,070588109	0,014588109	-1,245411891
chrXI	658054	658203	149	0,122531105	0,092646739	0,569441084	0,174951387	0,148708679	0,031618445	-0,003381555	-1,263381555
chrXI	659842	659965	123	2,898003113	7,748973289	0,272190245	-0,606202204	-0,515271873	0,073248898	0,220248898	-1,039751102
chrXI	661850	662006	156	0,185221438	0,259410871	0,416572153	-0,210670615	-0,179070023	-0,179923982	-0,263923982	-1,523923982
chrXI	663739	663846	107	0,082637257	1,05617283	0,072564564	-1,456953824	-1,23841075	-0,590157694	-0,331157694	-1,591157694
chrXI	663739	663851	112	0,136778908	1,467524353	0,085257514	-1,370550803	-1,164968182	-0,460994452	-0,236994452	-1,496994452
chrXI	664373	664519	146	1,26805446	0,996878917	0,559863912	0,150624192	0,128030563	0,054160132	0,040160132	-1,219839868
chrXI	666612	666748	136	1,288001384	0,181587609	0,876436466	1,157355694	0,98375234	1,033209526	1,089209526	-0,170790474
chrXI	666628	666815	187	0,045592969	0,051882174	0,467739443	-0,080953558	-0,068810524	0,243475613	-0,057524387	-1,317524387
chrXI	666656	666815	159	0,113982423	0,100058479	0,532526364	0,081622044	0,069378737	0,088620929	-0,016379071	-1,276379071
chrXI	666681	666771	90	0,031345166	0,040764565	0,434687048	-0,164453569	-0,139785534	0,244324492	0,622324492	-0,637675508
chrXI	666681	666810	129	1,02584181	0,285351958	0,782372396	0,780230509	0,663195933	0,928223985	1,033223985	-0,226776015
chrXI	666693	666810	117	0,208017923	0,133411305	0,609256343	0,277381333	0,235774133	0,889668174	1,078668174	-0,181331826
chrXI	666703	666728	25	0,113982423	0,062999783	0,644033238	0,369260555	0,313871472		0,805	-0,455
chrXI	666703	666733	30	0,253610892	0,200116957	0,558949362	0,148306006	0,126060105		0,798	-0,462
chrXI	666703	666737	34	0,188070999	0,166764131	0,530023616	0,075329227	0,064029843		0,77	-0,49
chrXI	666703	666739	36	0,216566604	0,244587392	0,469618839	-0,076228037	-0,064793831		0,756	-0,504
chrXI	666709	666792	83	0,034194727	0,159352392	0,176673914	-0,928115177	-0,788897901	-0,445737534	-0,018737534	-1,278737534
chrXI	666709	666810	101	0,786478721	1,200701743	0,395776194	-0,264295292	-0,224650998	0,231224229	0,532224229	-0,727775771
chrXI	666709	666815	106	0,564212996	0,722644568	0,43844246	-0,154919323	-0,131681425	0,414853959	0,680853959	-0,579146041
chrXI	666714	666815	101	0,045592969	0,096352609	0,321200349	-0,464344846	-0,394693119	0,081054919	0,382054919	-0,877945081
chrXII	723	874	151	0,578460798	0,381704567	0,602459555	0,25971854	0,220760759	0,093970571	0,044970571	-1,215029429
chrXII	1167	1315	148	0,045592969	0,051882174	0,467739443	-0,080953558	-0,068810524	-0,162874716	-0,190874716	-1,450874716
chrXII	1996	2147	151	0,504372223	0,277940218	0,644719675	0,371103224	0,315437741	0,194715863	0,145715863	-1,114284137
chrXII	2008	2142	134	0,592708601	0,418763262	0,585986247	0,217232053	0,184647245	0,171166216	0,241166216	-1,018833784
chrXII	2033	2129	96	0,056991212	0,122293696	0,317880698	-0,47363334	-0,402588339	0,159797715	0,495797715	-0,764202285
chrXII	2033	2193	160	0,037044288	0,044470435	0,454449042	-0,114428548	-0,097264266	-0,106624355	-0,218624355	-1,478624355
chrXII	2033	2193	160	0,037044288	0,044470435	0,454449042	-0,114428548	-0,097264266	-0,106624355	-0,218624355	-1,478624355
chrXII	2211	2359	148	0,775080479	0,522527611	0,597314771	0,246402775	0,209442359	0,114836564	0,086836564	-1,173163436
chrXII	2226	2355	129	0,042743409	0,074117392	0,365763444	-0,34309514	-0,291630869	-0,048857305	0,056142695	-1,203857305
chrXII	2226	2359	133	13,34164265	9,831671992	0,57573303	0,190989346	0,162340944	0,232221032	0,309221032	-0,950778968
chrXII	2226	2421	195	0,076938136	0,066705652	0,535617563	0,08939893	0,07598909	0,149474199	-0,207525801	-1,467525801
chrXII	2387	2517	130	0,350495952	0,463233697	0,430727763	-0,174521638	-0,148343393	-0,018494239	0,079505761	-1,180494239
chrXII	2666	2818	152	0,170973635	0,114881957	0,598111913	0,248463027	0,211193573	0,058948536	0,002948536	-1,257051464
chrXII	3013	3157	144	1,299399626	0,592939133	0,686663326	0,486414449	0,413452282	0,280276974	0,280276974	-0,979723026
chrXII	3013	3200	187	0,131079787	0,062999783	0,67539199	0,45485158	0,386623843	0,441936062	0,140936062	-1,119063938
chrXII	3065	3157	92	0,153876272	0,114881957	0,572545341	0,182858153	0,15542943	0,533587426	0,897587426	-0,362412574
chrXII	3065	3200	135	1,165470279	0,852350003	0,577588742	0,195728705	0,166369399	0,179887046	0,242887046	-1,017112954
chrXII	3153	3297	144	0,105433742	0,037058696	0,739925174	0,64311474	0,546647529	0,410440407	0,410440407	-0,849559593
chrXII	3218	3370	152	0,102584181	0,074117392	0,58055047	0,203301913	0,172806626	0,032133523	-0,023866477	-1,283866477
chrXII	3470	3595	125	0,230814407	0,237175653	0,493203653	-0,01703674	-0,014481229	0,385091451	0,518091451	-0,741908549
chrXII	3500	3643	143	0,330549028	0,400233914	0,452321762	-0,11979755	-0,101827917	-0,164970922	-0,157970922	-1,417970922
chrXII	3500	3651	151	0,045592969	0,037058696	0,551627959	0,129775456	0,110309137	-0,018111307	-0,067111307	-1,327111307
chrXII	3791	3928	137	0,410336724	0,466939567	0,467739443	-0,080953558	-0,068810524	-0,02038484	0,02861516	-1,23138484
chrXII	6346	6517	171	0,034194727	0,059293913	0,365763444	-0,34309514	-0,291630869	-0,13689495	-0,32589495	-1,58589495
chrXII	6366	6517	151	0,626903328	0,43729261	0,589086376	0,225195424	0,191416111	0,068977125	0,019977125	-1,240022875
chrXII	6690	6864	174	0,028495606	0,048176305	0,371656395	-0,327469522	-0,278349094	-0,072909836	-0,282909836	-1,542909836
chrXII	6705	6857	152	0,034194727	0,051882174	0,397257877	-0,260451199	-0,221383519	-0,345251081	-0,401251081	-1,661251081
chrXII	7495	7682	187	0,065539893	0,037058696	0,638799168	0,355250862	0,301963233	0,353522145	0,052522145	-1,207477855
chrXII	7531	7682	151	0,561363435	0,26311674	0,680869537	0,470131702	0,399611946	0,272527021	0,223527021	-1,036472979
chrXII	7543	7677	134	0,652549374	0,444704349	0,594711469	0,23968165	0,203729402	0,210733109	0,280733109	-0,979266891
chrXII	7568	7664	96	0,037044288	0,107470218	0,25633612	-0,65468243	-0,556480065	0,026047505	0,362047505	-0,897952495
chrXII	7746	7894	148	0,675345858	0,670762394	0,501702487	0,004267516	0,003627389	-0,092037904	-0,120037904	-1,380037904
chrXII	7761	7894	133	13,05383703	9,876142427	0,569291266	0,17457007	0,148384559	0,215679755	0,292679755	-0,967320245

Chrom	Start	End	Length	Section A	Section B	A/A+B	Z-score	Z * 0.85	Phase correction	Length correction	ΔLKnuc
chrXII	7761	7956	195	0,082637257	0,081529131	0,503375009	0,008459995	0,007190996	0,079047487	-0,277952513	**-1,537952513**
chrXII	7922	8052	130	0,333398588	0,444704349	0,428476198	-0,180254982	-0,153216735	-0,018881073	0,079118927	**-1,180881073**
chrXII	8201	8353	152	0,176672756	0,092646739	0,655996907	0,401562292	0,341327948	0,222993139	0,166993139	**-1,093006861**
chrXII	8548	8692	144	1,407682928	0,61517435	0,695888407	0,512611386	0,435719678	0,295105628	0,295105628	**-0,964894372**
chrXII	8548	8735	187	0,116831984	0,092646739	0,55772721	0,145209355	0,123427951	0,177653657	-0,123346343	**-1,383346343**
chrXII	8600	8692	92	0,125380666	0,17417587	0,418554265	-0,205593405	-0,174754394	0,183007114	0,547007114	**-0,712992886**
chrXII	8600	8735	135	1,262355338	0,870879351	0,591756427	0,232065494	0,19725567	0,212034861	0,275034861	**-0,984965139**
chrXII	8688	8832	144	0,09688506	0,062999783	0,605967759	0,268824834	0,228501109	0,07798138	0,07798138	**-1,18201862**
chrXII	8753	8905	152	0,09688506	0,077823261	0,554553208	0,137173592	0,116597553	0,004423552	-0,051576448	**-1,311576448**
chrXII	9005	9130	125	0,159575393	0,259410871	0,380860679	-0,303221116	-0,257737948	0,110673859	0,243673859	**-1,016326141**
chrXII	9035	9178	143	0,29350474	0,429880871	0,405737598	-0,238523365	-0,20274486	-0,272598667	-0,265598667	**-1,525598667**
chrXII	9326	9463	137	0,504372223	0,448410219	0,529367672	0,073680449	0,062628381	0,113587303	0,162587303	**-1,097412697**
chrXII	11195	11357	162	0,128230226	0,085235	0,600707798	0,255179578	0,216902641	0,213742641	0,087742641	**-1,172257359**
chrXII	11198	11354	156	0,185221438	0,077823261	0,704144347	0,536357779	0,455904112	0,461386243	0,377386243	**-0,882613757**
chrXII	11205	11354	149	0,039893848	0,059293913	0,402205349	-0,247642923	-0,210496484	-0,353300921	-0,388300921	**-1,648300921**
chrXII	11205	11357	152	0,128230226	0,037058696	0,775794437	0,75806658	0,644356593	0,527511964	0,471511964	**-0,788488036**
chrXII	11207	11357	150	0,125380666	0,081529131	0,605967759	0,268824834	0,228501109	0,131804571	0,089804571	**-1,170195429**
chrXII	11208	11354	146	0,34194727	0,181587609	0,653150885	0,393841275	0,334765084	0,185395834	0,171395834	**-1,088604166**
chrXII	11211	11357	146	0,088336378	0,051882174	0,629990659	0,331828607	0,282054316	0,136111871	0,122111871	**-1,137888129**
chrXII	11213	11310	97	0,094035499	0,077823261	0,547167331	0,118507771	0,100731606	0,655734131	0,984734131	**-0,275265869**
chrXII	11213	11346	133	0,108283302	0,037058696	0,745024175	0,658912981	0,560076034	0,624919884	0,701919884	**-0,558080116**
chrXII	11213	11352	139	0,034194727	0,100058479	0,254703244	-0,659762131	-0,560797812	-0,590797997	-0,555797997	**-1,815797997**
chrXII	11213	11355	142	0,031345166	0,103764348	0,231998216	-0,73228205	-0,622439743	-0,657383612	-0,643383612	**-1,903383612**
chrXII	11213	11357	144	3,724375683	5,658862847	0,396917937	-0,2613328	-0,22213288	-0,378455941	-0,378455941	**-1,638455941**
chrXII	11213	11358	145	0,034194727	0,111176087	0,235224155	-0,721749813	-0,613487341	-0,807981651	-0,814981651	**-2,074981651**
chrXII	11218	11354	136	0,056991212	0,040764565	0,582995843	0,209563572	0,178129036	0,144974149	0,200974149	**-1,059025851**
chrXII	11222	11354	132	0,042743409	0,040764565	0,51184823	0,029703476	0,025247955	0,146256695	0,230256695	**-1,029743305**
chrXII	11222	11357	135	0,056991212	0,074117392	0,434687048	-0,164453569	-0,139785534	-0,134619259	-0,071619259	**-1,331619259**
chrXII	11226	11354	128	0,718089267	0,448410219	0,615593299	0,293927372	0,249838266	0,498784127	0,610784127	**-0,649215873**
chrXII	11226	11357	131	0,934655871	0,641115437	0,593141826	0,235634438	0,200289273	0,272786581	0,363786581	**-0,896213419**
chrXII	11226	11358	132	0,119681545	0,114881957	0,510230892	0,025647854	0,021800676	0,14658318	0,23058318	**-1,02941682**
chrXII	11227	11357	130	0,09973462	0,08894087	0,528604008	0,071761159	0,060996985	0,202152468	0,300152468	**-0,959847532**
chrXII	11228	11357	129	0,039893848	0,118587827	0,251725307	-0,669070333	-0,568709783	-0,340122644	-0,235122644	**-1,495122644**
chrXII	11229	11357	128	0,934655871	0,896820438	0,510329217	0,025894402	0,022010241	0,26797266	0,37997266	**-0,88002734**
chrXII	11230	11357	127	0,031345166	0,085235	0,268872204	-0,616227458	-0,523793339	-0,28934951	-0,17034951	**-1,43034951**
chrXII	11231	11354	123	0,547115632	0,326116523	0,626540868	0,322705529	0,274299699	0,693320613	0,840320613	**-0,419679387**
chrXII	11231	11357	126	1,020142689	0,396528045	0,720098654	0,583134602	0,495664412	0,722730576	0,848730576	**-0,411269424**
chrXII	11231	11358	127	0,404637603	0,159352392	0,717455286	0,575298511	0,489003734	0,719559615	0,838559615	**-0,421440385**
chrXII	11233	11357	124	0,065539893	0,040764565	0,616530051	0,29638	0,251923	0,689774459	0,829774459	**-0,430225541**
chrXII	11235	11357	122	0,216566604	0,251999131	0,462190442	-0,094916836	-0,080679311	0,293098613	0,447098613	**-0,812901387**
chrXII	11252	11354	102	0,076938136	0,066705652	0,535617563	0,08939893	0,07598909	0,569578512	0,863578512	**-0,396421488**
chrXII	11252	11357	105	0,43883233	0,985761308	0,308040355	-0,501412691	-0,426200787	0,155973964	0,428973964	**-0,831026036**
chrXII	11259	11357	98	0,062690333	0,096352609	0,394172367	-0,268460684	-0,228191582	0,317686663	0,639686663	**-0,620313337**
chrXII	11262	11357	95	0,085486818	0,322410653	0,20957918	-0,807882268	-0,686699927	-0,153259517	0,189740483	**-1,070259517**
chrXII	11273	11357	84	0,031345166	0,111176087	0,219933277	-0,772418577	-0,656555791	-0,328520136	0,091479864	**-1,168520136**
chrXII	13256	13420	164	0,125380666	0,051882174	0,707315001	0,545557714	0,463724057	0,455770013	0,315770013	**-0,944229987**
chrXII	13298	13427	129	0,156725832	0,374292827	0,295141855	-0,538424944	-0,457661202	-0,239486236	-0,134486236	**-1,394486236**
chrXII	13457	13612	155	0,14532759	0,059293913	0,710226382	0,554046188	0,47093926	0,355620708	0,278620708	**-0,981379292**
chrXII	15085	15257	172	0,49012442	0,541056958	0,475303793	-0,061943802	-0,052652232	0,079247134	-0,116752866	**-1,376752866**
chrXII	15201	15361	160	0,364743755	0,314998914	0,536590936	0,091848853	0,078071525	0,066694293	-0,045305707	**-1,305305707**
chrXII	16724	16865	141	0,159575393	0,151940653	0,512254168	0,030721476	0,026113255	0,009913435	0,030913435	**-1,229086565**
chrXII	18847	18996	149	0,909009826	0,578115654	0,611252943	0,282586129	0,24019821	0,089176588	0,054176588	**-1,205823412**
chrXII	25117	25257	140	1,743931077	1,23405457	0,585607617	0,216260411	0,183821349	0,210367273	0,238367273	**-1,021632727**
chrXII	27714	27865	151	0,769381358	0,400233914	0,657807209	0,406485945	0,345513053	0,210723624	0,161723624	**-1,098276376**
chrXII	35557	35688	131	0,153876272	0,100058479	0,605967759	0,268824834	0,228501109	0,309415172	0,400415172	**-0,859584828**
chrXII	39250	39399	149	0,532867829	0,70782109	0,429493502	-0,177663815	-0,151014242	-0,304332552	-0,339332552	**-1,599332552**
chrXII	48577	48723	146	0,039893848	0,040764565	0,49460244	-0,013530088	-0,011500575	-0,152204565	-0,166204565	**-1,426204565**
chrXII	51782	51932	150	0,87766466	0,70782109	0,553561998	0,134665941	0,11446605	0,015408087	-0,026591913	**-1,286591913**
chrXII	56756	56881	125	0,062690333	0,103764348	0,376621026	-0,314367347	-0,267212245	0,099125764	0,232125764	**-1,027874236**
chrXII	57933	58079	146	0,133929347	0,085235	0,611090941	0,282163534	0,239839004	0,10553226	0,09153226	**-1,16846774**
chrXII	60326	60478	152	0,074088575	0,066705652	0,52621884	0,065768269	0,055903029	-0,0452987	-0,1012987	**-1,3612987**
chrXII	62223	62381	158	0,250761331	0,248293262	0,502472745	0,006198292	0,005268549	-0,046856717	-0,144856717	**-1,404856717**
chrXII	67309	67460	151	0,088336378	0,166764131	0,346280681	-0,395381497	-0,336074272	-0,466579442	-0,515579442	**-1,775579442**
chrXII	70032	70180	148	0,188070999	0,151940653	0,553131041	0,13357593	0,113539541	0,025729856	-0,002270144	**-1,262270144**
chrXII	70780	70917	137	1,581506124	1,374877614	0,534946159	0,087709357	0,074552953	0,13420713	0,18320713	**-1,07679287**
chrXII	70780	70917	137	1,581506124	1,374877614	0,534946159	0,087709357	0,074552953	0,13420713	0,18320713	**-1,07679287**
chrXII	71343	71501	158	0,299203861	0,429880871	0,410382838	-0,226560288	-0,192576245	-0,233948363	-0,331948363	**-1,591948363**
chrXII	73489	73643	154	0,045592969	0,040764565	0,527955893	0,070132481	0,059612609	-0,121911524	-0,191911524	**-1,451911524**
chrXII	79801	79962	161	0,131079787	0,085235	0,605967759	0,268824834	0,228501109	0,202542856	0,083542856	**-1,176457144**
chrXII	99031	99175	144	2,074480105	1,237760439	0,626307201	0,322088569	0,273775284	0,123978737	0,123978737	**-1,136021263**
chrXII	104748	104900	152	0,595558162	0,370586958	0,616427232	0,296110709	0,251694103	0,157276799	0,101276799	**-1,158723201**
chrXII	104994	105164	170	0,19377012	0,200116957	0,491943329	-0,020196453	-0,017166985	0,157926685	-0,024073315	**-1,284073315**
chrXII	106767	106921	154	0,649699813	0,807879568	0,445738888	-0,136434532	-0,115969352	-0,299492884	-0,369492884	**-1,629492884**
chrXII	107154	107242	88	0,031345166	0,125999566	0,19921332	-0,84443452	-0,717769342	-0,309940147	0,082059853	**-1,177940147**
chrXII	107154	107250	96	0,028495606	0,133411305	0,175999936	-0,930717198	-0,791109618	-0,223943904	0,112056096	**-1,147943904**
chrXII	107154	107252	98	0,028495606	0,062999783	0,311443082	-0,491764035	-0,41799943	0,108189924	0,430189924	**-0,829810076**
chrXII	107154	107253	99	0,028495606	0,066705652	0,299319635	-0,52635832	-0,447404572	0,048964852	0,363964852	**-0,896035148**
chrXII	107154	107255	101	0,04844253	0,129705435	0,271923004	-0,607007389	-0,51595628	-0,081529761	0,219470239	**-1,040529761**
chrXII	107154	107257	103	0,042743409	0,066705652	0,390532439	-0,277931585	-0,236241848	0,335249143	0,622249143	**-0,637750857**
chrXII	107154	107258	104	0,039893848	0,085235	0,318822147	-0,470995015	-0,400345763	0,18420368	0,46420368	**-0,79579632**
chrXII	107154	107259	105	0,037044288	0,103764348	0,263082498	-0,633871027	-0,538790373	0,027955547	0,300955547	**-0,959044453**
chrXII	107154	107260	106	10,13303743	23,328449	0,302826877	-0,516287316	-0,438844218	0,084082013	0,350082013	**-0,909917987**
chrXII	107154	107261	107	0,250761331	0,522527611	0,324278957	-0,455766476	-0,387401505	0,110046441	0,369046441	**-0,890953559**
chrXII	107154	107262	108	0,239363089	0,674468263	0,261933549	-0,637395748	-0,541786386	-0,004194712	0,247805288	**-1,012194712**
chrXII	107154	107265	111	0,039893848	0,151940653	0,207959715	-0,813520966	-0,691492821	-0,092650654	0,138349346	**-1,121650654**
chrXII	107154	107267	113	0,045592969	0,207528696	0,180122745	-0,914897409	-0,777662797	-0,219695093	-0,002695093	**-1,262695093**
chrXII	107154	107269	115	0,054141651	0,259410871	0,172671713	-0,943659989	-0,802110991	-0,129868891	0,073131109	**-1,186868891**
chrXII	107154	107270	116	0,039893848	0,100058479	0,285053125	-0,567895024	-0,48271077	0,118324276	0,314324276	**-0,945675724**
chrXII	107154	107271	117	0,028495606	0,240881523	0,105783316	-1,249269194	-1,061878815	-0,514444505	-0,325444505	**-1,585444505**
chrXII	107154	107273	119	0,051292091	0,170470001	0,231293321	-0,734594249	-0,624405111	-0,220611231	-0,045611231	**-1,305611231**
chrXII	107154	107274	120	11,24721562	28,02749162	0,286372997	-0,564011958	-0,479410164	-0,088463625	0,079536375	**-1,180463625**
chrXII	107154	107285	131	3,359631928	3,546517187	0,486469648	-0,033922068	-0,028833758	0,056012127	0,147012127	**-1,112987873**
chrXII	107271	107423	152	1,504567988	0,885702829	0,62945503	0,330410325	0,280848776	0,181151651	0,125151651	**-1,134848349**
chrXII	110633	110799	166	0,162424953	0,292763697	0,356829972	-0,366945134	-0,311903364	-0,208628817	-0,362628817	**-1,622628817**
chrXII	112506	112634	128	0,094035499	0,35205761	0,210797919	-0,803655711	-0,683107355	-0,441031641	-0,329031641	**-1,589031641**
chrXII	113051	113206	155	0,091185939	0,08894087	0,506231912	0,015621722	0,013278464	-0,103164869	-0,180164869	**-1,440164869**

Chrom	Start	End	Length	Section A	Section B	A/A+B	Z-score	Z * 0.85	Phase correction	Length correction	ΔLKnuc
chrXII	118248	118400	152	0,14817715	0,144528914	0,506231912	0,015621722	0,013278464	-0,083982792	-0,139982792	**-1,399982792**
chrXII	119597	119738	141	0,111132863	0,133411305	0,454449042	-0,114428548	-0,097264266	-0,117877866	-0,096877866	**-1,356877866**
chrXII	121717	121889	172	0,028495606	0,051882174	0,354520937	-0,373143192	-0,317171714	-0,190744401	-0,386744401	**-1,646744401**
chrXII	126647	126799	152	0,122531105	0,107470218	0,532740871	0,082161538	0,069837308	-0,036649175	-0,092649175	**-1,352649175**
chrXII	132281	132417	136	3,151614005	2,779402184	0,531378419	0,078735306	0,066925011	0,041156744	0,097156744	**-1,162843256**
chrXII	132310	132466	156	0,111132863	0,118587827	0,483773852	-0,040684142	-0,03458152	-0,026869368	-0,110869368	**-1,370869368**
chrXII	133301	133451	150	1,179718082	1,015408265	0,53742605	0,093951228	0,079858544	-0,020944539	-0,062944539	**-1,322944539**
chrXII	133762	133946	184	0,059840772	0,103764348	0,365763444	-0,34309514	-0,291630869	-0,246922939	-0,526922939	**-1,786922939**
chrXII	136653	136779	126	0,416035845	1,037643482	0,286195062	-0,564534946	-0,479854704	-0,246410878	-0,120410878	**-1,380410878**
chrXII	138426	138582	156	0,404637603	0,474351306	0,460344378	-0,099566165	-0,08463124	-0,101659153	-0,185659153	**-1,445659153**
chrXII	142345	142492	147	0,039893848	0,040764565	0,49460244	-0,013530088	-0,011500575	-0,174631662	-0,195631662	**-1,455631662**
chrXII	143973	144111	138	0,424584527	0,659644785	0,391600303	-0,275150512	-0,233877935	-0,155961249	-0,113961249	**-1,373961249**
chrXII	144765	144914	149	2,39363089	1,560171093	0,60539979	0,267349056	0,227246697	0,074078304	0,039078304	**-1,220921696**
chrXII	145430	145576	146	0,330549028	0,289057827	0,533481877	0,084025388	0,071421579	-0,053704722	-0,067704722	**-1,327704722**
chrXII	155097	155220	123	0,113982423	0,181587609	0,385639525	-0,290711661	-0,247104912	0,184641063	0,331641063	**-0,928358937**
chrXII	160945	161106	161	0,071239015	0,062999783	0,530688713	0,07700122	0,065451037	0,049858502	-0,069141498	**-1,329141498**
chrXII	163470	163588	118	1,880709985	11,01384439	0,145853042	-1,054386316	-0,896228369	-0,431741427	-0,249741427	**-1,509741427**
chrXII	171732	171883	151	0,09688506	0,118587827	0,449639217	-0,126572917	-0,10758698	-0,239097036	-0,288097036	**-1,548097036**
chrXII	177830	177964	134	0,230814407	0,188999349	0,549801916	0,125160904	0,106386768	0,10180946	0,17180946	**-1,08819054**
chrXII	177830	177964	134	0,230814407	0,188999349	0,549801916	0,125160904	0,106386768	0,10180946	0,17180946	**-1,08819054**
chrXII	178588	178741	153	0,133929347	0,081529131	0,621601659	0,309690034	0,263236529	0,152146299	0,089146299	**-1,170853701**
chrXII	179076	179226	150	0,04844253	0,111176087	0,303489222	-0,514391296	-0,437232602	-0,515080258	-0,557080258	**-1,817080258**
chrXII	180676	180836	160	0,937505432	0,767115003	0,549978994	0,125608276	0,106767034	0,099076995	-0,012923005	**-1,272923005**
chrXII	183315	183461	146	1,393435125	0,767115003	0,644944594	0,37170727	0,315951179	0,184406624	0,170406624	**-1,089593376**
chrXII	192705	192836	131	2,185612968	2,657108488	0,451319158	-0,12232919	-0,103979812	-0,027488888	0,063511112	**-1,196488888**
chrXII	193019	193125	106	0,153876272	0,340940001	0,310976579	-0,493084111	-0,419121495	0,125559079	0,391559079	**-0,868440921**
chrXII	193019	193144	125	0,19661968	0,415057393	0,3214436	-0,463665806	-0,394115935	-0,011759005	0,121240995	**-1,138759005**
chrXII	194389	194550	161	0,091185939	0,125999566	0,419852784	-0,202270107	-0,171929591	-0,171441442	-0,290441442	**-1,550441442**
chrXII	197350	197482	132	1,134125112	7,359856983	0,133521015	-1,109900209	-0,943415178	-0,825958585	-0,741958585	**-2,001958585**
chrXII	197802	197965	163	9,605868727	10,40237591	0,480095526	-0,049913836	-0,042426761	0,030258379	-0,102741621	**-1,362741621**
chrXII	199062	199223	161	1,686939865	1,875170007	0,473578841	-0,066276513	-0,056335036	-0,050848524	-0,169848524	**-1,429848524**
chrXII	199271	199406	135	0,450230572	0,370586958	0,548514811	0,121909896	0,103623411	0,114877818	0,177877818	**-1,082122182**
chrXII	199721	199871	150	19,63632198	19,49287398	0,501833005	0,004594677	0,003905476	-0,081895841	-0,123895841	**-1,383895841**
chrXII	207917	208073	156	0,131079787	0,255705001	0,338895921	-0,415478239	-0,353156503	-0,364440981	-0,448440981	**-1,708440981**
chrXII	208414	208561	147	0,151026711	0,133411305	0,530965281	0,07769655	0,066042068	-0,091723134	-0,112723134	**-1,372723134**
chrXII	209818	209960	142	0,444531451	0,233469783	0,655649914	0,400619655	0,340526707	0,311200063	0,325200063	**-0,934799937**
chrXII	211059	211185	126	0,094035499	0,070411522	0,571828535	0,1810314	0,15387669	0,402331061	0,528331061	**-0,731668939**
chrXII	213217	213369	152	0,156725832	0,096352609	0,619277689	0,303584289	0,258046646	0,152248929	0,096248929	**-1,163751071**
chrXII	214356	214519	163	0,062690333	0,096352609	0,394172367	-0,268460684	-0,228191582	-0,157549705	-0,290549705	**-1,550549705**
chrXII	216121	216270	149	0,076938136	0,044470435	0,633712557	0,341702373	0,290447017	0,136457726	0,101457726	**-1,158542274**
chrXII	217884	218033	149	0,125380666	0,040764565	0,754644987	0,68917996	0,585802966	0,429462213	0,394462213	**-0,865537787**
chrXII	218108	218265	157	0,051292091	0,062999783	0,448781606	-0,128740216	-0,109429183	-0,194823073	-0,285823073	**-1,545823073**
chrXII	218294	218444	150	9,218328488	4,302514581	0,681786516	0,472700362	0,401795308	0,319207357	0,277207357	**-0,982792643**
chrXII	218298	218444	146	0,09688506	0,051882174	0,65125268	0,388704654	0,330398956	0,193906975	0,179906975	**-1,080093025**
chrXII	218756	218891	135	0,088336378	0,08894087	0,498295067	-0,004273647	-0,0036326	0,01444599	0,07744599	**-1,18255401**
chrXII	219794	219934	140	0,14532759	0,125999566	0,535617563	0,08939893	0,07598909	0,133799211	0,161799211	**-1,098200789**
chrXII	219800	219934	134	0,111132863	0,096352609	0,535617563	0,08939893	0,07598909	0,078651916	0,148651916	**-1,111348084**
chrXII	220212	220367	155	0,116831984	0,037058696	0,759188173	0,703693523	0,598139495	0,4751371	0,3981371	**-0,8618629**
chrXII	220343	220451	108	0,042743409	0,155646522	0,215451503	-0,787647358	-0,669500254	-0,116492107	0,135507893	**-1,124492107**
chrXII	223444	223638	194	0,04844253	0,185293479	0,207253175	-0,815989135	-0,693590765	-0,622023883	-0,972023883	**-2,232023883**
chrXII	223444	223638	194	0,04844253	0,185293479	0,207253175	-0,815989135	-0,693590765	-0,622023883	-0,972023883	**-2,232023883**
chrXII	230481	230653	172	0,213717044	0,478057176	0,308940457	-0,498855884	-0,424027501	-0,305350347	-0,501350347	**-1,761350347**
chrXII	230739	230894	155	0,225115286	0,163058261	0,579934639	0,201726272	0,171467332	0,045097264	-0,031902736	**-1,291902736**
chrXII	232813	232957	144	1,054337416	0,985761308	0,516807056	0,042141512	0,035820285	-0,116459762	-0,116459762	**-1,376459762**
chrXII	232813	232957	144	1,054337416	0,985761308	0,516807056	0,042141512	0,035820285	-0,116459762	-0,116459762	**-1,376459762**
chrXII	235469	235619	150	0,116831984	0,044470435	0,72430398	0,595675482	0,506324159	0,420545047	0,378545047	**ΔLKnuc0,881454953**
chrXII	235483	235631	148	0,247911771	0,35576348	0,410670755	-0,225819886	-0,191946903	-0,283333857	-0,311333857	**-1,571333857**
chrXII	237484	237634	150	0,319150785	0,326116523	0,49460244	-0,013530088	-0,011500575	-0,112712146	-0,154712146	**-1,414712146**
chrXII	238295	238446	151	0,051292091	0,048176305	0,515662191	0,039269381	0,033378974	-0,110905244	-0,159905244	**-1,419905244**
chrXII	239615	239753	138	0,34194727	0,392822175	0,465380361	-0,086887768	-0,073854602	-0,001336787	0,040663213	**-1,219336787**
chrXII	255888	256021	133	0,233663968	0,203822827	0,534105191	0,085593433	0,072754418	0,127162215	0,204162215	**-1,055837785**
chrXII	256324	256484	160	0,094035499	0,055588044	0,628480635	0,327831948	0,278657156	0,25246996	0,14046996	**-1,11953004**
chrXII	259071	259235	164	0,29065518	0,307587175	0,485848548	-0,035479871	-0,030157891	-0,045486947	-0,185486947	**-1,445486947**
chrXII	260642	260797	155	0,173823196	0,081529131	0,68071906	0,46971048	0,399253908	0,275607702	0,198607702	**-1,061392298**
chrXII	262571	262718	147	1,105629506	0,659644785	0,62632165	0,322126715	0,273807708	0,12498415	0,10398415	**-1,15601585**
chrXII	270658	270826	168	0,236513528	0,366881088	0,391971559	-0,274184138	-0,233056517	-0,098070813	-0,266070813	**-1,526070813**
chrXII	270673	270826	153	0,173823196	0,048176305	0,782989131	0,782328165	0,66497894	0,550453334	0,487453334	**-0,772546666**
chrXII	271752	271896	144	4,49375704	3,231518273	0,581695391	0,206232607	0,175297716	0,023106802	0,023106802	**-1,236893198**
chrXII	273309	273417	108	2,994888173	15,38306462	0,162960924	-0,982361374	-0,835007168	-0,273740562	-0,021740562	**-1,281740562**
chrXII	273309	273418	109	0,068389454	0,292673697	0,189364135	-0,880241917	-0,74820563	-0,109731612	0,135268388	**-1,124731612**
chrXII	273309	273419	110	0,045592969	0,426175002	0,096642782	-1,300920803	-1,105782682	-0,398347604	-0,160347604	**-1,420347604**
chrXII	273309	273420	111	2,826764099	19,84863746	0,124662141	-1,151992193	-0,979193364	-0,39519264	-0,16419264	**-1,42419264**
chrXII	274415	274568	153	5,411315548	4,258044146	0,559635355	0,150044776	0,127538059	0,011215076	-0,051784924	**-1,311784924**
chrXII	275945	276112	167	0,233663968	0,35205761	0,398933515	-0,256108537	-0,217692257	-0,054374505	-0,215374505	**-1,475374505**
chrXII	275945	276112	167	0,233663968	0,35205761	0,398933515	-0,256108537	-0,217692257	-0,054374505	-0,215374505	**-1,475374505**
chrXII	280170	280332	162	0,256460453	0,148234783	0,633712557	0,341702373	0,290447017	0,295377389	0,169377389	**-1,090622611**
chrXII	280378	280532	154	1,849364819	1,089525656	0,629273134	0,329928838	0,280439513	0,09784117	0,02784117	**-1,23215883**
chrXII	281608	281761	153	0,071239015	0,125999566	0,361181948	-0,355301282	-0,302006089	-0,423857777	-0,486857777	**-1,746857777**
chrXII	285125	285277	152	7,628273682	4,521160886	0,627870675	0,326219022	0,277286169	0,166931155	0,110931155	**-1,149068845**
chrXII	285128	285277	149	0,051292091	0,048176305	0,515662191	0,039269381	0,033378974	-0,123503979	-0,158503979	**-1,418503979**
chrXII	286283	286443	160	0,153876272	0,137117174	0,528796348	0,072244535	0,061407855	0,040409835	-0,071590165	**-1,331590165**
chrXII	292173	292332	159	0,088336378	0,107470218	0,451140973	-0,122779201	-0,104362321	-0,139775814	-0,244775814	**-1,504775814**
chrXII	293330	293472	142	0,227964847	0,274234349	0,453933118	-0,11573037	-0,098370815	-0,118315349	-0,104315349	**-1,364315349**
chrXII	295117	295270	153	0,29350474	0,270528479	0,520367826	0,051076768	0,043415253	-0,081531963	-0,144531963	**-1,404531963**
chrXII	295472	295620	148	1,017293128	0,459527828	0,688839852	0,492564557	0,418679873	0,322988245	0,294988245	**-0,965011755**
chrXII	296601	296740	139	0,262159574	0,170470001	0,605967759	0,268824834	0,228501109	0,202626761	0,237626761	**-1,022373239**
chrXII	300804	300959	155	0,353345512	0,163058261	0,6842427	0,479596127	0,407656708	0,282738181	0,205738181	**-1,054261819**
chrXII	301895	302037	142	0,056991212	0,085235	0,400708216	-0,251514398	-0,213787238	-0,223906682	-0,209906682	**-1,469906682**
chrXII	302498	302646	148	0,903310705	0,61146848	0,596331606	0,243863179	0,207283702	0,104687712	0,076687712	**-1,183312288**
chrXII	304308	304455	147	2,222657255	1,478641962	0,600507315	0,254660445	0,216461378	0,070027915	0,049027915	**-1,210972085**
chrXII	304635	304774	139	0,182371877	0,114881957	0,613522371	0,288511463	0,245234744	0,206879322	0,241879322	**-1,018120678**
chrXII	310453	310583	130	0,074088575	0,114881957	0,392064172	-0,273943107	-0,232851641	-0,091342475	0,006657525	**-1,253342475**
chrXII	311744	311903	159	0,031345166	0,055588044	0,360566076	-0,356946107	-0,303404191	-0,341893012	-0,446893012	**-1,706893012**
chrXII	312700	312835	135	0,111132863	0,17417587	0,389517915	-0,280575734	-0,238489374	-0,221469889	-0,158469889	**-1,418469889**
chrXII	314498	314655	157	0,430283648	0,429880871	0,500234128	0,000586872	0,000498841	-0,088540057	-0,179540057	**-1,439540057**
chrXII	319179	319325	146	0,173823196	0,203822827	0,460280753	-0,099726444	-0,084767477	-0,22371803	-0,23771803	**-1,49771803**

DATA 2

Chrom	Start	End	Length	Section A	Section B	A/A+B	Z-score	Z * 0.85	Phase correction	Length correction	ΔLKnuc
chrXII	320329	320487	158	0,44168189	0,633703698	0,410719555	-0,225694405	-0,191840244	-0,233880671	-0,331880671	-1,591880671
chrXII	320335	320487	152	0,051292091	0,044470435	0,535617563	0,08939893	0,07598909	-0,03533557	-0,09133557	-1,35133557
chrXII	320336	320487	151	0,094035499	0,129705435	0,420287416	-0,201158254	-0,170984516	-0,306866232	-0,355866232	-1,615866232
chrXII	321520	321655	135	0,094035499	0,107470218	0,466664175	-0,083658001	-0,071109301	-0,063570288	-0,000570288	-1,260570288
chrXII	322116	322262	146	0,051292091	0,037058696	0,58055047	0,203301913	0,172806626	0,030913728	0,016913728	-1,243086272
chrXII	327876	328044	168	0,19377012	0,218646305	0,469840938	-0,07566971	-0,064319253	0,067556774	-0,100443226	-1,360443226
chrXII	338236	338387	151	0,105433742	0,081529131	0,563928764	0,160937697	0,136797043	0,010094553	-0,038905447	-1,298905447
chrXII	340076	340209	133	1,5615592	1,49346544	0,511144552	0,027938883	0,02374805	0,078747667	0,155747667	-1,104252333
chrXII	340597	340761	164	1,430479413	1,941875659	0,424178173	-0,191216029	-0,162533625	-0,179950386	-0,319950386	-1,579950386
chrXII	340875	341034	159	0,082637257	0,055588044	0,597844653	0,247772164	0,210606339	0,176205254	0,071205254	-1,188794746
chrXII	347768	347916	148	0,738036191	0,544762828	0,575332675	0,189967434	0,161472318	0,056600327	0,028600327	-1,231399673
chrXII	347831	348011	180	0,202318801	0,452116089	0,30915039	-0,498260025	-0,423521021	-0,267904971	-0,519904971	-1,779904971
chrXII	347831	348011	180	0,202318801	0,452116089	0,30915039	-0,498260025	-0,423521021	-0,267904971	-0,519904971	-1,779904971
chrXII	348735	348938	203	0,088336378	0,125999566	0,412139824	-0,222043979	-0,188737382	-0,152869978	-0,565869978	-1,825869978
chrXII	348736	348938	202	0,079787696	0,096352609	0,452978074	-0,118140734	-0,100419624	-0,077917651	-0,483917651	-1,743917651
chrXII	350679	350830	151	3,453667427	2,190168921	0,61193614	0,284368859	0,241713531	0,135944322	0,086944322	-1,173055678
chrXII	355881	356007	126	0,324849907	1,096937395	0,22847996	-0,743862076	-0,632282764	-0,391486704	-0,265486704	-1,525486704
chrXII	355881	356007	126	0,324849907	1,096937395	0,22847996	-0,743862076	-0,632282764	-0,391486704	-0,265486704	-1,525486704
chrXII	358183	358323	140	1,521665352	1,511994788	0,501593877	0,003995268	0,003395978	0,046140705	0,074140705	-1,185859295
chrXII	359562	359714	152	0,304902982	0,259410871	0,540307456	0,101208326	0,086027077	-0,021600487	-0,077600487	-1,337600487
chrXII	360814	360966	152	0,162424953	0,070411522	0,69759239	0,517488464	0,439865194	0,318432625	0,262432625	-0,997567375
chrXII	367954	368092	138	0,054141651	0,100058479	0,351112876	-0,382317666	-0,324970016	-0,268814766	-0,226814766	-1,486814766
chrXII	372826	372968	142	0,92610719	0,915349786	0,502920895	0,007321663	0,006223413	-0,008508295	0,005491705	-1,254508295
chrXII	375291	375434	143	8,0101148	4,495219799	0,640535824	0,359891395	0,305907686	0,2522374	0,2592374	-1,0007626
chrXII	375584	375750	166	0,085486818	0,207528696	0,291748435	-0,548284062	-0,466041453	-0,36642701	-0,52042701	-1,78042701
chrXII	388796	388957	161	2,741277281	2,612638053	0,512013566	0,030118096	0,025600382	0,02797669	-0,09102331	-1,35102331
chrXII	388880	389024	144	0,102584181	0,059293913	0,633712557	0,341702373	0,290447017	0,148105143	0,148105143	-1,111894857
chrXII	391687	391826	139	0,170973635	0,103764348	0,622315244	0,311567137	0,264832067	0,228981162	0,263981162	-0,996018838
chrXII	395857	396006	149	0,094035499	0,037058696	0,717312458	0,574876116	0,488644698	0,335033822	0,300033822	-0,959966178
chrXII	395857	396006	149	0,094035499	0,037058696	0,717312458	0,574876116	0,488644698	0,335033822	0,300033822	-0,959966178
chrXII	397064	397220	156	0,259310013	0,196411088	0,569010328	0,173855096	0,147776831	0,135868738	0,051868738	-1,208131262
chrXII	398081	398231	150	0,34194727	0,188999349	0,644033238	0,369260555	0,313871472	0,214062848	0,172062848	-1,087937152
chrXII	400245	400397	152	0,068389454	0,055588044	0,551627959	0,129775456	0,110309137	-0,011418445	-0,067418445	-1,327418445
chrXII	401669	401819	150	0,259310013	0,200116957	0,564420528	0,162186559	0,137858575	0,040573111	-0,001426889	-1,261426889
chrXII	401669	401819	150	0,259310013	0,200116957	0,564420528	0,162186559	0,137858575	0,040573111	-0,001426889	-1,261426889
chrXII	405414	405520	106	0,205168362	0,826408916	0,19888802	-0,845599796	-0,718759827	-0,20871227	0,05728773	-1,20271227
chrXII	405414	405538	124	0,247911771	1,282230874	0,162018732	-0,986194937	-0,838265696	-0,410378564	-0,270378564	-1,530378564
chrXII	406427	406580	153	0,809275206	0,837526525	0,491422368	-0,021502592	-0,018277203	-0,1437777	-0,2067777	-1,4667777
chrXII	406621	406768	147	1,798072728	1,411936309	0,56014569	0,151338603	0,128637812	-0,014054924	-0,035054924	-1,295054924
chrXII	408412	408522	110	1,057186976	2,90540175	0,266792001	-0,622544332	-0,529162682	0,165189926	0,403189926	-0,856810074
chrXII	408412	408523	111	0,042743409	0,077823261	0,354520937	-0,373143192	-0,317171714	0,255088896	0,486088896	-0,773911104
chrXII	408415	408537	122	0,028495606	0,300175436	0,086699472	-1,361363185	-1,157158707	-0,765925096	-0,611925096	-1,871925096
chrXII	411908	412040	132	0,87766466	1,248878048	0,412719038	-0,220556114	-0,187472697	-0,070599746	0,013400254	-1,246599746
chrXII	412316	412427	111	6,140803057	36,49910948	0,144015376	-1,062451526	-0,903083797	-0,348712832	-0,117712832	-1,377712832
chrXII	412319	412427	108	0,028495606	0,181587609	0,135639612	-1,100121415	-0,935103203	-0,355948696	-0,103948696	-1,363948696
chrXII	412320	412427	107	0,054141651	0,340940001	0,137039143	-1,093718891	-0,929661058	-0,409672298	-0,150672298	-1,410672298
chrXII	419068	419220	152	0,056991212	0,118587827	0,324590066	-0,454901459	-0,386666241	-0,515397902	-0,571397902	-1,831397902
chrXII	419734	419893	159	2,154267801	1,482347831	0,592382594	0,233678201	0,198626471	0,167895595	0,062895595	-1,197104405
chrXII	421131	421283	152	0,424584527	0,381704567	0,526590934	0,066703018	0,056697565	-0,081536735	-0,137536735	-1,397536735
chrXII	423648	423793	145	0,176672756	0,037058696	0,826619039	0,940857046	0,799728489	0,587352627	0,580352627	-0,679647373
chrXII	424263	424441	178	0,071239015	0,059293913	0,545755127	0,114943701	0,097702146	0,22770264	-0,01029736	-1,27029736
chrXII	425377	425494	117	0,712390146	5,547686759	0,113798945	-1,206569731	-1,025584272	-0,493343258	-0,304343258	-1,564343258
chrXII	428829	428982	153	0,128230226	0,070411522	0,645535127	0,373293861	0,317299782	0,195225716	0,132225716	-1,127774284
chrXII	433448	433586	138	8,517336584	5,729274369	0,597850016	0,247786025	0,210618121	0,268118424	0,310118424	-0,949881576
chrXII	433602	433749	147	0,151026711	0,274234349	0,35513882	-0,371483236	-0,315760751	-0,447650857	-0,468650857	-1,728650857
chrXII	434337	434490	153	2,778321569	1,815876094	0,604745763	0,265650389	0,22580283	0,100153744	0,037153744	-1,222846256
chrXII	443464	443629	165	0,142478029	0,166764131	0,460732784	-0,09858756	-0,083799426	-0,033060345	-0,180060345	-1,440060345
chrXII	446788	446962	174	0,108283302	0,17417587	0,383359129	-0,29667027	-0,252169729	-0,042566972	-0,252566972	-1,512566972
chrXII	446788	446962	174	0,108283302	0,17417587	0,383359129	-0,29667027	-0,252169729	-0,042566972	-0,252566972	-1,512566972
chrXII	447291	447454	163	0,125380666	0,196411088	0,389632936	-0,280275857	-0,238234478	-0,151928781	-0,284928781	-1,544928781
chrXII	447291	447454	163	0,125380666	0,196411088	0,389632936	-0,280275857	-0,238234478	-0,151928781	-0,284928781	-1,544928781
chrXII	450323	450477	154	0,840620372	0,444704349	0,654014008	0,396180352	0,336753299	0,166512966	0,096512966	-1,163487034
chrXII	451465	451626	161	0,062690333	0,103764348	0,376621026	-0,314367347	-0,267212245	-0,260740675	-0,379740675	-1,639740675
chrXII	451590	451738	148	0,04844253	0,059293913	0,449639217	-0,126572917	-0,10758698	-0,206170043	-0,234170043	-1,494170043
chrXII	451599	451733	134	0,045592969	0,044470435	0,506231912	0,015621722	0,013278464	0,025931925	0,095931925	-1,164068075
chrXII	451709	451842	133	0,384690679	0,685585872	0,359431101	-0,359979851	-0,305982873	-0,250511471	-0,173511471	-1,433511471
chrXII	451726	451885	159	0,054141651	0,100058479	0,351112876	-0,382317666	-0,324970016	-0,357956651	-0,462956651	-1,722956651
chrXII	451734	451869	135	0,045592969	0,092646739	0,329810947	-0,440435256	-0,374369967	-0,372225321	-0,309225321	-1,569225321
chrXII	451757	451842	85	0,04844253	0,051882174	0,482857442	-0,042983253	-0,036535765	0,389672539	0,802672539	-0,457327461
chrXII	451757	451908	151	0,464478375	0,511410002	0,475954408	-0,060309901	-0,051263415	-0,153446946	-0,202446946	-1,462446946
chrXII	451769	451932	163	0,088336378	0,144528914	0,379345404	-0,307200444	-0,261120377	-0,172028581	-0,305028581	-1,565028581
chrXII	451790	451908	118	0,054141651	0,155646522	0,258077709	-0,649283084	-0,551890622	-0,087323576	0,094676424	-1,165323576
chrXII	451790	451932	142	0,045592969	0,048176305	0,486225044	-0,034535557	-0,029355224	-0,045093773	-0,031093773	-1,291093773
chrXII	451790	451960	170	1,399134247	1,508288918	0,481228279	-0,047071103	-0,040010437	0,139419652	-0,042580348	-1,302580348
chrXII	451790	451960	170	1,399134247	1,508288918	0,481228279	-0,047071103	-0,040010437	0,139419652	-0,042580348	-1,302580348
chrXII	451800	451934	134	1,21961193	0,974643699	0,555820349	0,140380579	0,119323492	0,125479691	0,195479691	-1,064520309
chrXII	451800	451961	161	0,296354301	0,426175002	0,410162328	-0,227127429	-0,193058315	-0,169953909	-0,288953909	-1,548953909
chrXII	451800	451961	161	0,296354301	0,426175002	0,410162328	-0,227127429	-0,193058315	-0,169953909	-0,288953909	-1,548953909
chrXII	451851	451934	83	0,259310013	0,426175002	0,378286917	-0,309983065	-0,263485605	0,054184397	0,481184397	-0,778815603
chrXII	451851	451958	107	2,288197149	12,388722	0,15590446	-1,011433657	-0,859718608	-0,328802255	-0,069802255	-1,329802255
chrXII	451851	451959	108	0,065539893	0,34835174	0,15835037	-1,001260798	-0,851071678	-0,248939933	0,003060067	-1,256939933
chrXII	451851	451960	109	0,04844253	0,466939567	0,093993428	-1,316557908	-1,119074222	-0,495613919	-0,250613919	-1,510613919
chrXII	451851	451961	110	2,507613313	16,39476702	0,132661245	-1,113899065	-0,946814205	-0,263875966	-0,025875966	-1,285875966
chrXII	451876	451960	84	0,037044288	0,062999783	0,370279692	-0,331112667	-0,281445767	0,048417289	0,468417289	-0,791582711
chrXII	451876	452009	133	0,897611584	1,011702395	0,470122564	-0,074961771	-0,063717505	-0,010600939	0,066399061	-1,193600939
chrXII	451919	452075	156	0,079787696	0,092646739	0,462713239	-0,093600543	-0,079560461	-0,076369664	-0,160369664	-1,420369664
chrXII	451919	452077	158	0,113982423	0,081529131	0,582995843	0,209563572	0,178129036	0,136410237	0,038410237	-1,221589763
chrXII	451919	452078	159	0,889062902	0,674468263	0,568624996	0,172874585	0,146943398	0,121453034	0,016453034	-1,243546966
chrXII	451919	452092	173	0,09973462	0,096352609	0,508623742	0,021618199	0,01837547	0,15710684	-0,04589316	-1,30589316
chrXII	451923	452075	152	1,265204899	0,722644568	0,636469169	0,349036828	0,296681304	0,158237503	0,102237503	-1,157762497
chrXII	451923	452077	154	0,054141651	0,070411522	0,434687048	-0,164453569	-0,139785534	-0,309654166	-0,379654166	-1,639654166
chrXII	451923	452092	169	0,131079787	0,111176087	0,541079911	0,103154718	0,08768151	0,199492153	0,024492153	-1,235507847
chrXII	451931	452062	131	0,364743755	0,485468915	0,429002963	-0,178913112	-0,152076146	-0,085948784	0,005051216	-1,254948784
chrXII	451931	452077	146	2,29389627	1,534230006	0,599221683	0,251333038	0,213633083	0,080212524	0,066212524	-1,193787476
chrXII	451931	452109	178	0,091185939	0,103764348	0,467739443	-0,080953558	-0,068810624	0,060735308	-0,177264692	-1,437264692
chrXII	451932	452062	130	4,747367932	5,647745238	0,45669228	-0,108770453	-0,092454885	0,037504009	0,135504009	-1,124495991

Chrom	Start	End	Length	Section A	Section B	A/A+B	Z-score	Z * 0.85	Phase correction	Length correction	ΔLKnuc
chrXII	451932	452075	143	0,051292091	0,048176305	0,515662191	0,039269381	0,033378974	-0,029064137	-0,022064137	**-1,282064137**
chrXII	451932	452077	145	0,672496298	0,844938264	0,443179768	-0,142912183	-0,121475355	-0,338633967	-0,345633967	**-1,605633967**
chrXII	451932	452109	177	0,125380666	0,125999566	0,498768996	-0,003085674	-0,002622823	0,159689408	-0,071310592	**-1,331310592**
chrXII	451945	452051	106	0,168124074	0,270528479	0,383273899	-0,296893528	-0,252359499	0,257845604	0,523845604	**-0,736154396**
chrXII	451945	452062	117	0,14817715	0,26682261	0,357053581	-0,366345661	-0,311393812	0,202718613	0,391718613	**-0,868281387**
chrXII	451945	452075	130	0,188070999	0,111176087	0,628480635	0,327831948	0,278657156	0,408185349	0,506185349	**-0,753814651**
chrXII	451945	452077	132	3,342534564	2,879460663	0,537212653	0,093413969	0,079401874	0,193593672	0,277593672	**-0,982406328**
chrXII	451945	452078	133	0,156725832	0,125999566	0,554339417	0,136632651	0,116137754	0,155615448	0,232615448	**-1,027384552**
chrXII	451945	452092	147	3,160162687	1,534230006	0,673178171	0,448706136	0,381400215	0,241676572	0,220676572	**-1,039323428**
chrXII	451945	452109	164	0,208017923	0,170470001	0,549602536	0,124657219	0,105958636	0,093266889	-0,046733111	**-1,306733111**
chrXII	451958	452109	151	0,478726178	0,251999131	0,655138356	0,399230614	0,339346022	0,220938216	0,171938216	**-1,088061784**
chrXII	452041	452196	155	0,185221438	0,08894087	0,675590454	0,45540334	0,387092839	0,256637472	0,179637472	**-1,080362528**
chrXII	452059	452215	156	0,253610892	0,229763914	0,524667171	0,06187088	0,052590248	0,049802559	-0,034197441	**-1,294197441**
chrXII	452081	452227	146	0,68389454	0,377998697	0,644033238	0,369260555	0,313871472	0,168453549	0,154453549	**-1,105546451**
chrXII	452081	452236	155	0,074088575	0,070411522	0,512723359	0,03189814	0,027113419	-0,102692328	-0,179692328	**-1,439692328**
chrXII	452081	452247	166	1,461824579	1,174760656	0,554438582	0,136883557	0,116351023	0,218347231	0,064347231	**-1,195652769**
chrXII	452081	452251	170	0,071239015	0,085235	0,455276965	-0,11233987	-0,09548889	0,078196631	-0,103803369	**-1,363803369**
chrXII	452104	452247	143	0,076938136	0,040764565	0,653664997	0,395234266	0,335949126	0,275132834	0,282132834	**-0,977867166**
chrXII	452104	452251	147	0,313451664	0,163058261	0,657807209	0,406485945	0,345513053	0,20586995	0,18486995	**-1,07513005**
chrXII	452115	452271	156	0,567062556	0,448410219	0,558422215	0,146970164	0,124924639	0,125004046	0,041004046	**-1,218995954**
chrXII	452190	452344	154	0,618354647	0,43729261	0,585758777	0,216648296	0,184151051	0,01709395	-0,05290605	**-1,31290605**
chrXII	452190	452344	154	0,618354647	0,43729261	0,585758777	0,216648296	0,184151051	0,01709395	-0,05290605	**-1,31290605**
chrXII	452260	452414	154	0,373292436	0,285351958	0,566758694	0,168127962	0,142908767	-0,024485193	-0,094485193	**-1,354485193**
chrXII	452293	452400	107	0,113982423	0,852350003	0,117953636	-1,1852787	-1,007486895	-0,478635313	-0,219635313	**-1,479635313**
chrXII	452294	452455	161	0,558513874	0,541056958	0,507938059	0,019899076	0,016914214	0,035748986	-0,083251014	**-1,343251014**
chrXII	452295	452455	160	0,267858695	0,270528479	0,497520572	-0,006215044	-0,005282788	-0,023266066	-0,135266066	**-1,395266066**
chrXII	452299	452455	156	0,153876272	0,196411088	0,439285825	-0,152780153	-0,12986313	-0,127744026	-0,211744026	**-1,471744026**
chrXII	452323	452455	132	0,054141651	0,074117392	0,42212736	-0,196454145	-0,166986023	-0,021849248	0,062150752	**-1,197849248**
chrXII	452323	452485	162	0,071239015	0,059293913	0,545755127	0,114943701	0,097702146	0,098982299	-0,027017701	**-1,287017701**
chrXII	452323	452486	163	0,34194727	0,255705001	0,572150875	0,181852797	0,154574878	0,240182565	0,107182565	**-1,152817435**
chrXII	452334	452441	107	0,031345166	0,070411522	0,308040355	-0,501412691	-0,426200787	0,104221579	0,363221579	**-0,896778421**
chrXII	452334	452455	121	0,039893848	0,074117392	0,349911571	-0,385559308	-0,327725412	0,054617055	0,215617055	**-1,044382945**
chrXII	452334	452485	151	0,624053768	0,400233914	0,609256343	0,277381333	0,235774133	0,109101522	0,060101522	**-1,199898478**
chrXII	452334	452486	152	0,051292091	0,066705652	0,434687048	-0,164453569	-0,139785534	-0,280333429	-0,336333429	**-1,596333429**
chrXII	452346	452496	150	0,182371877	0,114881957	0,613522371	0,288511463	0,245234744	0,132855305	0,090855305	**-1,169144695**
chrXII	452423	452584	161	0,14817715	0,207528696	0,416572153	-0,210670615	-0,179070023	-0,155443647	-0,274443647	**-1,534443647**
chrXII	452469	452584	115	0,028495606	0,151940653	0,157926162	-1,003017694	-0,852565039	-0,177893602	0,025106398	**-1,234893602**
chrXII	452469	452620	151	0,282106498	0,326116523	0,463820816	-0,090812431	-0,077190567	-0,20661518	-0,25561518	**-1,51561518**
chrXII	452469	452621	152	0,330549028	0,392822175	0,456956299	-0,108104753	-0,09188904	-0,236052632	-0,292052632	**-1,552052632**
chrXII	452469	452629	160	0,282106498	0,222352175	0,559226183	0,149007602	0,126656462	0,12092135	0,00892135	**-1,25107865**
chrXII	452469	452630	161	0,225115286	0,274234349	0,450816963	-0,123597557	-0,105057923	-0,091757401	-0,210757401	**-1,470757401**
chrXII	452491	452621	130	0,119681545	0,181587609	0,397257877	-0,260451199	-0,221383519	-0,075878987	0,022121013	**-1,237878987**
chrXII	452491	452629	138	0,034194727	0,044470435	0,434687048	-0,164453569	-0,139785534	-0,095026015	-0,053026015	**-1,313026015**
chrXII	452491	452663	172	0,219416165	0,26682261	0,451251888	-0,122499079	-0,104124217	0,005679695	-0,190320305	**-1,450320305**
chrXII	452492	452584	92	0,034194727	0,107470218	0,241377477	-0,701878473	-0,596596702	-0,242468787	0,121531213	**-1,138468787**
chrXII	452492	452620	128	0,116831984	0,296469566	0,282679762	-0,574899121	-0,488664253	-0,262493358	-0,150493358	**-1,410493358**
chrXII	452492	452621	129	1,46467414	3,742928274	0,281256905	-0,579111693	-0,492244939	-0,273985011	-0,168985011	**-1,428985011**
chrXII	452492	452629	137	0,122531105	0,181587609	0,402905508	-0,245833639	-0,208958593	-0,154106135	-0,105106135	**-1,365106135**
chrXII	452492	452663	171	0,039893848	0,133411305	0,230194241	-0,738207309	-0,627476213	-0,467598169	-0,656598169	**-1,916598169**
chrXII	452494	452621	127	0,028495606	0,051882174	0,354520937	-0,373143192	-0,317171714	-0,093550926	0,025449074	**-1,234550926**
chrXII	452525	452695	170	0,04844253	0,085235	0,36238349	-0,352095051	-0,299280793	-0,132892129	-0,314892129	**-1,574892129**
chrXII	452539	452673	134	0,273557816	0,370586958	0,424683747	-0,189925521	-0,161436693	-0,15971257	-0,08971257	**-1,34971257**
chrXII	452550	452672	122	0,29350474	1,115466743	0,208311342	-0,812294469	-0,690450299	-0,299173722	-0,145173722	**-1,405173722**
chrXII	452550	452702	152	0,165274514	0,163058261	0,503375009	0,008459995	0,007190996	-0,137196622	-0,193196622	**-1,453196622**
chrXII	452668	452796	128	0,282106498	0,463233697	0,378493606	-0,309439521	-0,263023593	-0,047558592	0,064441408	**-1,195558592**
chrXII	452684	452796	112	0,034194727	0,044470435	0,434687048	-0,164453569	-0,139785534	0,351605931	0,575605931	**-0,684394069**
chrXII	452684	452835	151	0,592708601	0,333528262	0,6399104	0,358219306	0,30448641	0,175959639	0,126959639	**-1,133040361**
chrXII	452686	452814	128	0,73233707	1,185878265	0,381780427	-0,30080807	-0,25568686	-0,040401963	0,071598037	**-1,188401963**
chrXII	452686	452884	198	0,034194727	0,048176305	0,415130494	-0,214366854	-0,182211826	-0,140198455	-0,518198455	**-1,778198455**
chrXII	452709	452864	155	0,871965539	0,692997611	0,557179598	0,143822286	0,122248943	-0,003921092	-0,080921092	**-1,340921092**
chrXII	452709	452884	175	0,094035499	0,096352609	0,493914773	-0,015253994	-0,012965895	0,242883564	0,025883564	**-1,234116436**
chrXII	452709	452900	191	0,094035499	0,062999783	0,598817654	0,250287928	0,212744739	0,255077275	-0,073922725	**-1,333922725**
chrXII	452723	452814	91	0,04844253	0,092646739	0,343346664	-0,403346515	-0,342844538	0,10127586	0,47227586	**-0,78772414**
chrXII	452723	452864	141	0,076938136	0,155646522	0,330796263	-0,437715508	-0,372058182	-0,398532571	-0,377532571	**-1,637532571**
chrXII	452723	452884	161	0,88051422	0,737468046	0,544205112	0,111033507	0,094378481	0,111132479	-0,007867521	**-1,267867521**
chrXII	452723	452900	177	0,125380666	0,122293696	0,506231912	0,015621722	0,013278464	0,163689555	-0,067310445	**-1,327310445**
chrXII	452765	452864	99	0,122531105	0,133411305	0,478744828	-0,053304046	-0,045308439	0,440643621	0,755643621	**-0,504356379**
chrXII	452765	452871	106	0,091185939	0,188999349	0,325448704	-0,452515846	-0,384638469	0,093033171	0,359033171	**-0,900966829**
chrXII	452765	452884	119	0,088336378	0,107470218	0,451140973	-0,122779201	-0,104362321	0,305468751	0,480468751	**-0,779531249**
chrXII	452765	452900	135	2,405029132	2,02340479	0,543087957	0,108216337	0,091983887	0,102668836	0,165668836	**-1,094331164**
chrXII	452765	452925	160	0,113982423	0,144528914	0,440918471	-0,148640972	-0,126344826	-0,137037703	-0,249037703	**-1,509037703**
chrXII	452765	452925	160	0,113982423	0,144528914	0,440918471	-0,148640972	-0,126344826	-0,137037703	-0,249037703	**-1,509037703**
chrXII	452773	452864	91	0,059840772	0,096352609	0,38311977	-0,297297305	-0,252702709	0,180943827	0,551943827	**-0,708056173**
chrXII	452773	452879	106	0,042743409	0,129705435	0,247861382	-0,681235065	-0,579049806	-0,10208273	0,16391727	**-1,09608273**
chrXII	452773	452884	111	0,028495606	0,070411522	0,288104674	-0,558930214	-0,475090682	0,067993226	0,298993226	**-0,961006774**
chrXII	452773	452900	127	0,170973635	0,259410871	0,397257877	-0,260451199	-0,221383519	0,008317586	0,127317586	**-1,132682414**
chrXII	452773	452925	152	0,752283994	0,622586089	0,547167331	0,118507771	0,100731606	-0,054232757	-0,110232757	**-1,370232757**
chrXII	452773	452925	152	0,752283994	0,622586089	0,547167331	0,118507771	0,100731606	-0,054232757	-0,110232757	**-1,370232757**
chrXII	452783	452962	179	0,082637257	0,148234783	0,357935317	-0,363983093	-0,309385629	-0,169350294	-0,414350294	**-1,674350294**
chrXII	452783	452962	179	0,082637257	0,148234783	0,357935317	-0,363983093	-0,309385629	-0,169350294	-0,414350294	**-1,674350294**
chrXII	452788	452923	135	0,672496298	0,511410002	0,568031691	0,171365199	0,145660419	0,144369235	0,207369235	**-1,052630765**
chrXII	452788	452953	165	0,088336378	0,055588044	0,613769206	0,28915654	0,245783059	0,302799342	0,155799342	**-1,104200658**
chrXII	452801	452923	122	0,116831984	0,103764348	0,529618887	0,074311878	0,063165096	0,441038612	0,595038612	**-0,664961388**
chrXII	452801	452953	152	2,063081862	1,415642179	0,593057063	0,235415993	0,200103594	0,047959902	-0,008040098	**-1,268040098**
chrXII	452801	452999	198	0,062690333	0,066705652	0,484484373	-0,038901718	-0,03306646	0,013493278	-0,364506722	**-1,624506722**
chrXII	452840	452953	113	0,116831984	0,170470001	0,406652199	-0,236165307	-0,200740511	0,351159875	0,568159875	**-0,691840125**
chrXII	452840	452999	159	0,604106844	0,463233697	0,565992596	0,166180623	0,14125353	0,110269708	0,005269708	**-1,254730292**
chrXII	452840	453023	183	0,065539893	0,096352609	0,404835878	-0,240849509	-0,204722082	-0,025460647	-0,298460647	**-1,558460647**
chrXII	452847	452953	106	0,034194727	0,08894087	0,277699771	-0,589688448	-0,501235181	-0,037668425	0,228331575	**-1,031668425**
chrXII	452847	452996	149	0,170973635	0,200116957	0,460732874	-0,09858756	-0,083799426	-0,232444518	-0,267444518	**-1,527444518**
chrXII	452876	453034	158	1,533063594	1,397112831	0,523198392	0,058182555	0,049455172	0,006910067	-0,091089933	**-1,351089933**
chrXII	452885	453034	149	0,324849907	0,144528914	0,692084714	0,501768218	0,426502985	0,277992086	0,242992086	**-1,017007914**
chrXII	452893	452999	106	0,054141651	0,092646739	0,368841506	-0,334923211	-0,284684729	0,184618073	0,450618073	**-0,809381927**
chrXII	452895	453023	128	1,478921943	1,700994137	0,465082067	-0,087638333	-0,074492583	0,141637186	0,253637186	**-1,006362814**
chrXII	452922	453029	107	1,211063248	7,841620028	0,133779479	-1,108701541	-0,94239631	-0,420338671	-0,161338671	**-1,421338671**
chrXII	452922	453031	109	0,031345166	0,163058261	0,161237725	-0,989383693	-0,840976139	-0,210115667	0,034884333	**-1,225115667**

Chrom	Start	End	Length	Section A	Section B	A/A+B	Z-score	Z * 0.85	Phase correction	Length correction	ΔLKnuc
chrXII	452922	453034	112	1,718285032	10,44684634	0,14124673	-1,07473484	-0,913524614	-0,436328186	-0,212328186	-1,472328186
chrXII	452922	453056	134	0,085486818	0,137117174	0,384030928	-0,294911018	-0,250674365	-0,251546027	-0,181546027	-1,441546027
chrXII	452922	453056	134	0,085486818	0,137117174	0,384030928	-0,294911018	-0,250674365	-0,251546027	-0,181546027	-1,441546027
chrXII	452922	453075	153	0,031345166	0,077823261	0,287126664	-0,56179848	-0,477528708	-0,595531986	-0,658531986	-1,918531986
chrXII	452922	453075	153	0,031345166	0,077823261	0,287126664	-0,56179848	-0,477528708	-0,595531986	-0,658531986	-1,918531986
chrXII	452926	452997	71	0,065539893	0,125999566	0,342174368	-0,406536101	-0,345555686		0,511	-0,749
chrXII	452926	453033	107	0,159575393	0,289057827	0,355692324	-0,369997104	-0,314497538	0,223013604	0,482013604	-0,777986396
chrXII	452926	453034	108	0,216566604	0,581821524	0,27125479	-0,609022416	-0,517669054	0,097481871	0,349481871	-0,910518129
chrXII	452926	453056	130	0,58700948	0,503998263	0,538043368	0,095505572	0,081179736	0,221328974	0,319328974	-0,940671026
chrXII	452926	453056	130	0,58700948	0,503998263	0,538043368	0,095505572	0,081179736	0,221328974	0,319328974	-0,940671026
chrXII	452926	453070	144	0,076938136	0,044470435	0,633712557	0,341702373	0,290447017	0,154750695	0,154750695	-1,105249305
chrXII	452926	453070	144	0,076938136	0,044470435	0,633712557	0,341702373	0,290447017	0,154750695	0,154750695	-1,105249305
chrXII	452926	453075	149	0,401788042	0,26311674	0,604279068	0,26443874	0,224772929	0,071574536	0,036574536	-1,223425464
chrXII	452926	453075	149	0,401788042	0,26311674	0,604279068	0,26443874	0,224772929	0,071574536	0,036574536	-1,223425464
chrXII	452976	453138	162	0,239363089	0,35576348	0,402205349	-0,247642923	-0,210496484	-0,204667628	-0,330667628	-1,590667628
chrXII	452976	453163	187	0,039893848	0,040764565	0,49460244	-0,013530088	-0,011500575	0,040879243	-0,260120757	-1,520120757
chrXII	452991	453141	150	0,775080479	0,641115437	0,547297496	0,118836352	0,101010899	-0,021610662	-0,063610662	-1,323610662
chrXII	453033	453163	130	1,305098747	1,767699789	0,424726428	-0,189816588	-0,1613441	-0,021362186	0,076637814	-1,183362186
chrXII	453033	453187	154	0,102584181	0,08894087	0,535617563	0,08939893	0,07598909	-0,081350238	-0,151350238	-1,411350238
chrXII	453033	453193	160	0,076938136	0,103764348	0,42577243	-0,18714766	-0,159075511	-0,158822309	-0,270822309	-1,530822309
chrXII	453039	453163	124	0,102584181	0,137117174	0,427966629	-0,181553355	-0,154320352	0,279806752	0,419806752	-0,840193248
chrXII	453039	453187	148	1,111328628	0,748585655	0,597516046	0,246922881	0,209884448	0,107966427	0,079966427	-1,180033573
chrXII	453039	453193	154	0,094035499	0,096352609	0,493914773	-0,015253994	-0,012965895	-0,173427977	-0,243427977	-1,503427977
chrXII	453042	453141	99	0,028495606	0,074117392	0,277699771	-0,589688448	-0,501235181	-0,030505566	0,284494434	-0,975505566
chrXII	453042	453212	170	0,062690333	0,107470218	0,368418723	-0,336044318	-0,28563767	-0,134007874	-0,316007874	-1,576007874
chrXII	453042	453218	176	0,703841464	0,629997828	0,527680859	0,069441393	0,059025184	0,252519086	0,028519086	-1,231480914
chrXII	453043	453163	120	0,122531105	0,233469783	0,344187638	-0,401060914	-0,340901777	0,066047091	0,234047091	-1,025952909
chrXII	453043	453187	144	0,14532759	0,111176087	0,566571175	0,16765125	0,142503563	0,02833511	0,02833511	-1,23166489
chrXII	453043	453190	147	0,279256937	0,244587392	0,533091458	0,083043332	0,070586832	-0,078773731	-0,099773731	-1,359773731
chrXII	453043	453193	150	1,567258321	1,070996308	0,594051197	0,237978712	0,202281905	0,074416672	0,032416672	-1,227583328
chrXII	453091	453199	108	0,065539893	0,140823044	0,317595273	-0,474433869	-0,403268789	0,222020228	0,474020228	-0,785979772
chrXII	453092	453199	107	1,769577122	2,964695663	0,373780135	-0,321858003	-0,273579302	0,271312997	0,530312997	-0,729687003
chrXII	453092	453212	120	1,393435125	3,357517838	0,293295921	-0,543781498	-0,462214274	-0,064455905	0,103544095	-1,156455905
chrXII	453092	453236	144	0,133929347	0,192705218	0,410028091	-0,227472716	-0,193351809	-0,300901931	-0,300901931	-1,560901931
chrXII	453092	453245	153	0,210867483	0,240881523	0,466780182	-0,083366199	-0,070861269	-0,18892762	-0,25192762	-1,51192762
chrXII	453092	453270	178	0,088336378	0,118587827	0,426902103	-0,184266739	-0,156626728	-0,022025594	-0,260025594	-1,520025594
chrXII	453092	453282	190	0,039893848	0,111176087	0,264075364	-0,630831465	-0,536206745	-0,515441483	-0,837441483	-2,097441483
chrXII	453092	453286	194	0,142478029	0,218646305	0,394540095	-0,267505227	-0,227379443	-0,149000832	-0,499000832	-1,759000832
chrXII	453092	453288	196	0,139628469	0,151940653	0,478886337	-0,052948834	-0,045006509	0,01835609	-0,34564391	-1,60564391
chrXII	453094	453236	142	0,031345166	0,096352609	0,245463684	-0,688834586	-0,585509398	-0,604329181	-0,590329181	-1,850329181
chrXII	453095	453201	106	0,108283302	0,237175653	0,313447663	-0,486101327	-0,413186128	0,052841431	0,318841431	-0,941158569
chrXII	453095	453212	117	0,128230226	0,159352392	0,445890044	-0,136052108	-0,115644291	0,391670966	0,580670966	-0,679329034
chrXII	453095	453218	123	0,079787696	0,051882174	0,605967759	0,268824834	0,228501109	0,654930591	0,801930591	-0,458069409
chrXII	453095	453236	141	0,04844253	0,148234783	0,246304615	-0,686164739	-0,583240028	-0,628564354	-0,607564354	-1,867564354
chrXII	453095	453245	150	2,476268147	1,40823044	0,637474333	0,351715902	0,298958517	0,177339301	0,135339301	-1,124660699
chrXII	453095	453270	175	0,068389454	0,103764348	0,397257877	-0,260451199	-0,221383519	0,025067671	-0,191932329	-1,451932329
chrXII	453095	453286	191	0,179522317	0,185293479	0,492090307	-0,01982796	-0,016853766	0,026046178	-0,302953822	-1,562953822
chrXII	453095	453288	193	0,182371877	0,188999349	0,491077027	-0,022368443	-0,019013176	0,041561199	-0,301438801	-1,561438801
chrXII	453099	453236	137	1,339293474	0,570703915	0,70120173	0,527859957	0,448680963	0,504630723	0,553630723	-0,706369277
chrXII	453099	453287	188	0,076938136	0,066705652	0,535617563	0,08939893	0,07598909	0,119094131	-0,188905869	-1,448905869
chrXII	453099	453288	189	0,139628469	0,137117174	0,504537188	0,01137329	0,009667296	0,045679934	-0,269320066	-1,529320066
chrXII	453100	453238	138	0,045592969	0,051882174	0,467739443	-0,080953558	-0,068810524	-0,030023589	0,011976411	-1,248023589
chrXII	453100	453245	145	0,190920559	0,040764565	0,824051866	0,930917448	0,791279831	0,565833646	0,558833646	-0,701166354
chrXII	453113	453270	157	0,404637603	0,389116306	0,509777147	0,024510127	0,020833608	-0,081507267	-0,172507267	-1,432507267
chrXII	453113	453286	173	0,059840772	0,100058479	0,374240479	-0,320642986	-0,272546538	-0,129096458	-0,332096458	-1,592096458
chrXII	453113	453288	175	0,074088575	0,111176087	0,399906675	-0,253588671	-0,21555037	0,025066481	-0,191933519	-1,451933519
chrXII	453114	453236	122	0,131079787	0,185293479	0,414320049	-0,216446021	-0,183979117	0,191503816	0,345503816	-0,914496184
chrXII	453114	453269	155	0,071239015	0,077823261	0,477914444	-0,055388588	-0,0470803	-0,171105897	-0,248105897	-1,508105897
chrXII	453114	453270	156	3,194357414	1,875170007	0,630109505	0,332143387	0,282321879	0,294506455	0,210506455	-1,049493545
chrXII	453114	453286	172	0,287805619	0,311293045	0,480397698	-0,049155472	-0,041782151	0,067273846	-0,128726154	-1,388726154
chrXII	453114	453287	173	0,396088921	0,444704349	0,471089547	-0,072531304	-0,061651608	0,072354329	-0,130645671	-1,390645671
chrXII	453114	453288	174	0,914708947	1,182172396	0,436223514	-0,160551029	-0,136468375	0,08363206	-0,12636794	-1,38636794
chrXII	453114	453302	188	0,113982423	0,111176087	0,506231912	0,015621722	0,013278464	0,045313105	-0,262686895	-1,522686895
chrXII	453114	453309	195	0,034194727	0,062999783	0,351817475	-0,380418277	-0,323355536	-0,244987022	-0,601987022	-1,861987022
chrXII	453116	453269	153	0,589859041	0,196411088	0,75019897	0,675116015	0,573848613	0,453219102	0,390219102	-0,869780898
chrXII	453116	453270	154	0,142478029	0,040764565	0,77753772	0,763903822	0,649318248	0,486762655	0,416762655	-0,843237345
chrXII	453116	453288	172	0,168124074	0,151940653	0,525281483	0,063413755	0,053901691	0,163908041	-0,032091959	-1,292091959
chrXII	453119	453236	117	0,162424953	0,259410871	0,385043053	-0,292262269	-0,248422928	0,243045293	0,432045293	-0,827954707
chrXII	453119	453245	126	0,071239015	0,100058479	0,415878909	-0,212447646	-0,180580499	0,065962342	0,191962342	-1,068037658
chrXII	453119	453269	150	0,136778908	0,111176087	0,551627959	0,129775456	0,110309137	-0,017742249	-0,059742249	-1,319742249
chrXII	453119	453270	151	1,188266763	0,985761308	0,54657379	0,117009634	0,099458189	-0,026573212	-0,075573212	-1,335573212
chrXII	453119	453282	163	0,028495606	0,303881305	0,085732808	-1,367509647	-1,1623832	-1,079034165	-1,212034165	-2,472034165
chrXII	453119	453286	167	0,720938828	0,893114568	0,446663555	-0,134095435	-0,113981119	0,046085585	-0,114914415	-1,374914415
chrXII	453119	453287	168	1,273753581	1,115466743	0,533125207	0,083128223	0,070658989	0,207152547	0,039152547	-1,220847453
chrXII	453119	453288	169	7,651070167	7,534032853	0,503853688	0,009659915	0,008210928	0,128840312	-0,046159688	-1,306159688
chrXII	453119	453302	183	0,29350474	0,226058044	0,564907166	0,163422653	0,138909255	0,299436496	0,026436496	-1,233563504
chrXII	453119	453309	190	0,079787696	0,170470001	0,318822147	-0,470995015	-0,400345763	-0,375136229	-0,697136229	-1,957136229
chrXII	453120	453228	108	0,059840772	0,074117392	0,446712395	-0,133971905	-0,113876119	0,518112775	0,770112775	-0,489887225
chrXII	453121	453270	149	1,382036883	0,763409133	0,644172295	0,369633736	0,314188676	0,160780882	0,125780882	-1,134219118
chrXII	453121	453282	161	0,056991212	0,092646739	0,380860679	-0,303221116	-0,257737948	-0,250300245	-0,369300245	-1,629300245
chrXII	453121	453286	165	0,250761331	0,300175436	0,455154468	-0,112648873	-0,095751542	-0,039224619	-0,186224619	-1,446224619
chrXII	453121	453288	167	0,159575393	0,26311674	0,377521558	-0,311996587	-0,265197099	-0,106854968	-0,267854968	-1,527854968
chrXII	453125	453231	106	0,296354301	0,870879351	0,253894583	-0,6622841	-0,562941485	-0,089676986	0,176323014	-1,083676986
chrXII	453125	453236	111	0,205168362	0,978349569	0,173354671	-0,940991251	-0,799842563	-0,271868661	-0,040868661	-1,300868661
chrXII	453125	453270	145	0,424584527	0,35576348	0,544096382	0,11075928	0,094145388	-0,122673767	-0,129673767	-1,389673767
chrXII	453125	453286	161	0,378991558	0,448410219	0,45805021	-0,105347063	-0,089545003	-0,080005225	-0,199005225	-1,459005225
chrXII	453125	453287	162	2,78117113	2,193874791	0,559024213	0,148495706	0,12622135	0,14638526	0,02038526	-1,23961474
chrXII	453125	453288	163	1,231010172	1,397112831	0,468398994	-0,079294993	-0,067400744	0,014033736	-0,118966264	-1,378966264
chrXII	453125	453302	177	0,14817715	0,148234783	0,499902783	-0,000243688	-0,000207135	0,143546357	-0,087453643	-1,347453643
chrXII	453126	453248	122	0,136778908	0,696703481	0,164105337	-0,977724354	-0,831065701	-0,488624393	-0,334624393	-1,594624393
chrXII	453126	453270	144	0,168124074	0,170470001	0,496535784	-0,008683612	-0,00738107	-0,110868156	-0,110868156	-1,370868156
chrXII	453126	453286	160	0,572761677	0,61888022	0,48064916	-0,048524398	-0,041245738	-0,042253349	-0,154253349	-1,414253349
chrXII	453126	453287	161	0,151026711	0,140823044	0,517481027	0,043832468	0,037257598	0,044059254	-0,074940746	-1,334940746
chrXII	453126	453288	162	0,484425299	0,770820872	0,385920555	-0,289967488	-0,246472365	-0,226408164	-0,352408164	-1,612408164
chrXII	453135	453270	135	0,119681545	0,144528914	0,452978074	-0,118140734	-0,100419624	-0,091874124	-0,028874124	-1,288874124
chrXII	453135	453282	147	1,601453048	1,32299544	0,547608568	0,119621655	0,101678407	-0,047952715	-0,068952715	-1,328952715

Chrom	Start	End	Length	Section A	Section B	A/A+B	Z-score	Z * 0.85	Phase correction	Length correction	ΔLKnuc
chrXII	453135	453286	151	0,430283648	0,61888022	0,410120536	-0,227234924	-0,193149685	-0,306071406	-0,355071406	**-1,615071406**
chrXII	453135	453288	153	0,310602104	0,426175002	0,421568615	-0,197882202	-0,168199872	-0,297874875	-0,360874875	**-1,620874875**
chrXII	453138	453286	148	0,068389454	0,055588044	0,551627959	0,129775456	0,110309137	0,013630939	-0,014369061	**-1,274369061**
chrXII	453138	453288	150	0,156725832	0,292763697	0,348675157	-0,38889974	-0,330564779	-0,452737165	-0,494737165	**-1,754737165**
chrXII	453140	453245	105	0,108283302	0,077823261	0,581834946	0,206589951	0,175601459	0,748393557	1,021393557	**-0,238606443**
chrXII	453140	453246	106	0,085486818	0,17788174	0,324590066	-0,454901459	-0,386666241	0,078767339	0,344767339	**-0,915232661**
chrXII	453140	453251	111	0,122531105	0,70040935	0,148894254	-1,041187561	-0,885009427	-0,384838737	-0,153838737	**-1,413838737**
chrXII	453140	453270	130	0,387540239	0,429880871	0,47410109	-0,064964608	-0,055219916	0,077825013	0,175825013	**-1,084174987**
chrXII	453140	453282	142	0,259310013	0,389116306	0,399906675	-0,253588671	-0,21555037	-0,232152831	-0,218152831	**-1,478152831**
chrXII	453140	453286	146	1,242408414	1,108055004	0,528580196	0,071701317	0,06094612	-0,084675116	-0,098675116	**-1,358675116**
chrXII	453140	453287	147	1,288001384	1,167348917	0,524569298	0,06162508	0,052381318	-0,099617363	-0,120617363	**-1,380617363**
chrXII	453140	453288	148	5,106412566	3,561340665	0,589127589	0,225301386	0,191506178	0,093680306	0,065680306	**-1,194319694**
chrXII	453140	453302	162	0,065539893	0,114881957	0,36325918	-0,349760614	-0,297296522	-0,280411185	-0,406411185	**-1,666411185**
chrXII	453140	453309	169	0,034194727	0,092646739	0,269586342	-0,614064532	-0,521954852	-0,408043158	-0,583043158	**-1,843043158**
chrXII	453140	453323	183	0,074088575	0,077823261	0,487707719	-0,030817056	-0,026194498	0,124288316	-0,148711684	**-1,408711684**
chrXII	453141	453236	95	0,131079787	0,211234566	0,382922264	-0,297814788	-0,25314257	0,273132047	0,616132047	**-0,643867953**
chrXII	453141	453245	104	0,199469241	0,211234566	0,48567663	-0,035911081	-0,030524419	0,552406369	0,832406369	**-0,427593631**
chrXII	453141	453251	110	0,065539893	0,200116957	0,24670884	-0,684883118	-0,582150651	0,090098053	0,328098053	**-0,931901947**
chrXII	453141	453269	128	0,116831984	0,103764348	0,529618887	0,074311878	0,063165096	0,2756474	0,3876474	**-0,8723526**
chrXII	453141	453270	129	0,988797522	1,011702395	0,494275213	-0,014350406	-0,012197845	0,209114833	0,314114833	**-0,945885167**
chrXII	453141	453281	140	0,054141651	0,062999783	0,462190442	-0,094916836	-0,080679311	-0,04334751	-0,01534751	**-1,27534751**
chrXII	453141	453282	141	0,330549028	0,392822175	0,456956299	-0,108104753	-0,09188904	-0,143524872	-0,122524872	**-1,382524872**
chrXII	453141	453286	145	6,491299009	4,139456319	0,610615032	0,280922379	0,238784023	0,032681886	0,025681886	**-1,234318114**
chrXII	453141	453287	146	1,062886098	1,022820004	0,509604923	0,024078299	0,020466554	-0,124220625	-0,138220625	**-1,398220625**
chrXII	453141	453288	147	7,349016745	5,429098933	0,575125232	0,18943801	0,161022309	0,009564057	-0,011435943	**-1,271435943**
chrXII	453141	453302	161	0,398938482	0,35576348	0,528604008	0,071761159	0,060996985	0,067177375	-0,051822625	**-1,311822625**
chrXII	453141	453309	168	0,746584873	0,863467612	0,463702196	-0,091111	-0,07744435	0,056858772	-0,111141228	**-1,371141228**
chrXII	453141	453323	182	0,065539893	0,081529131	0,445640364	-0,13668381	-0,116181238	0,066974078	-0,199025922	**-1,459025922**
chrXII	453141	453324	183	0,042743409	0,048176305	0,470122564	-0,074961771	-0,063717505	0,087250953	-0,185749047	**-1,445749047**
chrXII	453144	453288	144	0,037044288	0,096352609	0,277699771	-0,589688448	-0,501235181	-0,609085848	-0,609085848	**-1,869085848**
chrXII	453150	453286	136	0,131079787	0,114881957	0,532927539	0,082631037	0,070236381	0,043906171	0,099906171	**-1,160093829**
chrXII	453150	453287	137	0,039893848	0,051882174	0,434687048	-0,164453569	-0,139785534	-0,077786282	-0,028786282	**-1,288786282**
chrXII	453150	453288	138	0,102584181	0,122293696	0,456177292	-0,110069087	-0,093558724	-0,066642731	-0,024642731	**-1,284642731**
chrXII	453150	453295	145	0,094035499	0,044470435	0,678927584	0,464702061	0,394996752	0,204011487	0,197011487	**-1,062988513**
chrXII	453150	453302	152	0,09973462	0,203822827	0,328552705	-0,443913104	-0,377326138	-0,53351621	-0,58951621	**-1,84951621**
chrXII	453152	453240	88	0,102584181	0,137117174	0,427966629	-0,181553355	-0,154320352	0,274284332	0,666284332	**-0,593715668**
chrXII	453152	453270	118	0,190920559	0,170470001	0,528294262	0,070982761	0,060335347	0,513510439	0,695510439	**-0,564489561**
chrXII	453152	453286	134	1,894957788	1,608347397	0,540905713	0,102715748	0,087308386	0,076732939	0,146732939	**-1,113267061**
chrXII	453152	453287	135	0,29350474	0,303881305	0,491315025	-0,021771723	-0,018505964	-0,013451589	0,049548411	**-1,210451589**
chrXII	453152	453288	136	0,703841464	0,770820872	0,477289917	-0,056956516	-0,048413039	-0,083494979	-0,027494979	**-1,287494979**
chrXII	453152	453302	150	0,182371877	0,192705218	0,486225044	-0,034535557	-0,029355224	-0,156554267	-0,198554267	**-1,458554267**
chrXII	453152	453309	157	0,04844253	0,044470435	0,521375354	0,053605728	0,045564869	-0,052027613	-0,143027613	**-1,403027613**
chrXII	453153	453286	133	0,054141651	0,107470218	0,335010364	-0,426119559	-0,362201625	-0,33187596	-0,25487596	**-1,51487596**
chrXII	453153	453288	135	0,088336378	0,077823261	0,531635591	0,07938196	0,067474666	0,067082055	0,130082055	**-1,129917945**
chrXII	453159	453270	111	0,225115286	0,281646088	0,44422345	-0,140269704	-0,119229248	0,381265429	0,612265429	**-0,647734571**
chrXII	453159	453286	127	0,478726178	0,655938915	0,421909673	-0,197010469	-0,167458899	0,070134939	0,189134939	**-1,070865061**
chrXII	453159	453287	128	0,544266071	0,648527176	0,456295399	-0,109771242	-0,093305555	0,116665585	0,228665585	**-1,031334415**
chrXII	453159	453288	129	1,134125112	1,356348266	0,455385359	-0,112066452	-0,095256484	0,129126482	0,234126482	**-1,025873518**
chrXII	453159	453302	143	2,165666043	1,523112397	0,587095722	0,22008038	0,187068323	0,128321638	0,135321638	**-1,124678362**
chrXII	453159	453309	150	0,105433742	0,137117174	0,434687048	-0,164453569	-0,139785534	-0,259989909	-0,301989909	**-1,561989909**
chrXII	453159	453335	176	0,045592969	0,096352609	0,321200349	-0,464344846	-0,394693119	-0,204153227	-0,428153227	**-1,688153227**
chrXII	453159	453342	183	0,028495606	0,048176305	0,371656395	-0,327469522	-0,278349094	-0,133339773	-0,406339773	**-1,666339773**
chrXII	453161	453245	84	0,122531105	0,100058479	0,55047996	0,126874057	0,107842948	0,44136194	0,86136194	**-0,39863806**
chrXII	453161	453268	107	0,265009134	0,581821524	0,3129423	-0,487527441	-0,414398325	0,146960567	0,405960567	**-0,854039433**
chrXII	453161	453270	109	0,079787696	0,185293479	0,300993446	-0,521545394	-0,443313585	0,187194449	0,432194449	**-0,827805551**
chrXII	453161	453282	121	0,131079787	0,163058261	0,445640364	-0,13668381	-0,116181238	0,27406708	0,43506708	**-0,82493292**
chrXII	453161	453286	125	3,90674756	5,907156108	0,398082933	-0,258312336	-0,219565485	0,184542265	0,317542265	**-0,942457735**
chrXII	453161	453288	127	0,666797177	1,015408265	0,396382725	-0,262721233	-0,223313048	0,027655605	0,146655605	**-1,113344395**
chrXII	453161	453309	148	0,339097709	0,366881088	0,480322795	-0,049343454	-0,041941936	-0,139578397	-0,167578397	**-1,427578397**
chrXII	453161	453323	162	0,222265726	0,140823044	0,612152576	0,284933816	0,242193743	0,280547015	0,154547015	**-1,105452985**
chrXII	453161	453324	163	0,09973462	0,066705652	0,599221683	0,251333038	0,213633083	0,291452564	0,158452564	**-1,101547436**
chrXII	453162	453269	107	0,173823196	0,233469783	0,426776803	-0,184586205	-0,156898275	0,406945026	0,665945026	**-0,594054974**
chrXII	453162	453286	124	0,111132863	0,185293479	0,374908863	-0,318879716	-0,271047759	0,16264587	0,30264587	**-0,95735413**
chrXII	453162	453288	126	0,14532759	0,329822392	0,305856246	-0,507630508	-0,431485931	-0,156809458	-0,030809458	**-1,290809458**
chrXII	453162	453323	161	0,222265726	0,214940436	0,508377386	0,021000537	0,017850457	0,022278271	-0,096721729	**-1,356721729**
chrXII	453162	453324	162	0,373292436	0,374292827	0,499330918	-0,001677139	-0,001425569	0,043632421	-0,082367579	**-1,342367579**
chrXII	453165	453286	121	0,059840772	0,107470218	0,357661934	-0,364715392	-0,310008083	0,088920118	0,249920118	**-1,010079882**
chrXII	453165	453287	122	0,045592969	0,074117392	0,380860679	-0,303221116	-0,257737948	0,085000907	0,239000907	**-1,020999093**
chrXII	453165	453288	123	0,156725832	0,292763697	0,348675157	-0,38889974	-0,330564779	0,111176365	0,258176365	**-1,001823635**
chrXII	453165	453302	137	0,045592969	0,059293913	0,434687048	-0,164453569	-0,139785534	-0,071037093	-0,022037093	**-1,282037093**
chrXII	453165	453342	177	0,256460453	0,233469783	0,523463207	0,058847485	0,050020363	0,203049028	-0,027950972	**-1,287950972**
chrXII	453180	453286	106	0,031345166	0,166764131	0,158221582	-1,001793859	-0,85152478	-0,406973842	-0,140973842	**-1,400973842**
chrXII	453180	453288	108	0,062690333	0,214940436	0,225804702	-0,752734617	-0,639824425	0,013653156	0,265653156	**-0,994346844**
chrXII	453180	453340	160	0,111132863	0,125999566	0,468653164	-0,078655895	-0,066857511	-0,068407218	-0,180407218	**-1,440407218**
chrXII	453181	453270	89	0,04844253	0,277940218	0,148422459	-1,04322323	-0,886739745	-0,462550529	-0,077550529	**-1,337550529**
chrXII	453181	453282	101	0,113982423	1,856640659	0,057840804	-1,573160721	-1,337186613	-0,895233757	-0,594233757	**-1,854233757**
chrXII	453181	453286	105	0,279256937	1,045055221	0,21086942	-0,803408194	-0,682896965	-0,130474245	0,142525755	**-1,117474245**
chrXII	453181	453288	107	0,227964847	0,96723196	0,19073415	-0,875194106	-0,74391499	-0,191141437	0,067858563	**-1,192141437**
chrXII	453181	453323	142	0,410336724	0,418763262	0,494918262	-0,012738373	-0,010827617	-0,020947266	-0,006947266	**-1,266947266**
chrXII	453181	453324	143	0,045592969	0,100058479	0,313027917	-0,487285766	-0,414192901	-0,469976	-0,462976	**-1,722976**
chrXII	453286	453432	146	0,53571739	0,214940436	0,713663898	0,564120388	0,47950233	0,329999522	0,315999522	**-0,944000478**
chrXII	453299	453425	126	0,037044288	0,133411305	0,217325152	-0,781258786	-0,664069968	-0,381497291	-0,255497291	**-1,515497291**
chrXII	453299	453440	141	13,33594353	8,542029379	0,609560291	0,278173185	0,236447207	0,171490712	0,192490712	**-1,067509288**
chrXII	453299	453460	161	0,079787696	0,055588044	0,589379577	0,225949308	0,192056912	0,198988413	0,079988413	**-1,180011587**
chrXII	453300	453410	110	0,028495606	0,218646305	0,115300581	-1,198811728	-1,018989969	-0,343285834	-0,105285834	**-1,365285834**
chrXII	453300	453414	114	0,031345166	0,255705001	0,10919752	-1,230807045	-1,046185988	-0,449902166	-0,239902166	**-1,499902166**
chrXII	453300	453440	140	0,031345166	0,144528914	0,178225048	-0,922150465	-0,783827895	-0,746581283	-0,718581283	**-1,978581283**
chrXII	453302	453440	138	0,042743409	0,040764565	0,51184823	0,029703476	0,025247955	0,053743977	0,095743977	**-1,164256023**
chrXII	453310	453440	130	0,116831984	0,077823261	0,600199516	0,25386356	0,215784026	0,344227911	0,442227911	**-0,817772089**
chrXII	453310	453460	150	0,119681545	0,085235	0,584050178	0,21226584	0,180425964	0,065115525	0,023115525	**-1,236884475**
chrXII	453322	453481	159	0,176672756	0,148234783	0,543763178	0,109918963	0,093431118	0,054934533	-0,050065467	**-1,310065467**
chrXII	453458	453595	137	0,094035499	0,237175653	0,283914049	-0,571253086	-0,485565123	-0,422667366	-0,373667366	**-1,633667366**
chrXII	453458	453610	152	0,344796831	0,281646088	0,550404227	0,126682692	0,107680288	-0,038015096	-0,094015096	**-1,354015096**
chrXII	453458	453612	154	0,225115286	0,255705001	0,468190075	-0,079820337	-0,067847286	-0,228180698	-0,298180698	**-1,558180698**
chrXII	453460	453612	152	0,179522317	0,166764131	0,518421434	0,046192108	0,039263292	-0,107694186	-0,163694186	**-1,423694186**
chrXII	453487	453595	108	0,381841118	3,772575231	0,091912097	-1,32907207	-1,129711259	-0,463679521	-0,211679521	**-1,471679521**

Chrom	Start	End	Length	Section A	Section B	A/A+B	Z-score	Z * 0.85	Phase correction	Length correction	ΔLKnuc
chrXII	453487	453610	123	0,04844253	0,188999349	0,204018475	-0,827353109	-0,703250143	-0,264331296	-0,117331296	**-1,377331296**
chrXII	453487	453612	125	0,085486818	0,170470001	0,333989218	-0,428924135	-0,364585514	0,01586215	0,14886215	**-1,11113785**
chrXII	453500	453652	152	0,347646391	0,251999131	0,579753168	0,201262063	0,171072754	0,033024016	-0,022975984	**-1,282975984**
chrXII	453574	453727	153	0,113982423	0,044470435	0,719345959	0,58089966	0,493764711	0,364325495	0,301325495	**-0,958674505**
chrXII	453587	453737	150	0,054141651	0,085235	0,388455674	-0,283346352	-0,2408444	-0,362370448	-0,404370448	**-1,664370448**
chrXII	453606	453774	168	0,062690333	0,044470435	0,585011979	0,214732295	0,182522451	0,313676152	0,145676152	**-1,114323848**
chrXII	453627	453776	149	0,074088575	0,133411305	0,357053581	-0,366345661	-0,311393812	-0,447013422	-0,482013422	**-1,742013422**
chrXII	453627	453779	152	1,376337762	0,667056524	0,673554669	0,449750072	0,382287561	0,246202221	0,190202221	**-1,069797779**
chrXII	453657	453814	157	0,247911771	0,096352609	0,720120307	0,583198937	0,495719096	0,406659632	0,315659632	**-0,944340368**
chrXII	453659	453776	117	1,057186976	5,392040237	0,163924598	-0,978455287	-0,831686994	-0,337011648	-0,148011648	**-1,408011648**
chrXII	453659	453779	120	0,082637257	0,200116957	0,292258268	-0,546799428	-0,464779514	-0,065080791	0,102919209	**-1,157080791**
chrXII	453734	453853	119	0,410336724	0,848644133	0,325927691	-0,451186162	-0,383508238	0,031158634	0,206158634	**-1,053841366**
chrXII	453748	453910	162	0,039893848	0,111176087	0,264075364	-0,630831465	-0,536206745	-0,490176937	-0,616176937	**-1,876176937**
chrXII	453748	453913	165	3,182959172	3,290812186	0,491670001	-0,020881728	-0,017749469	0,039893353	-0,107106647	**-1,367106647**
chrXII	453748	453919	171	0,624053768	0,741173916	0,457106002	-0,107727312	-0,091568216	0,069043188	-0,119956812	**-1,379956812**
chrXII	453748	453922	174	0,031345166	0,077823261	0,287126664	-0,56179848	-0,477528708	-0,248968275	-0,458968275	**-1,718968275**
chrXII	453748	453962	214	0,119681545	0,188999349	0,387719315	-0,285268256	-0,242478018	0,018914742	-0,471085258	**-1,731085258**
chrXII	453752	453853	101	0,056991212	0,163058261	0,25899272	-0,646453904	-0,549485818	-0,066030532	0,234969468	**-1,025030532**
chrXII	453752	453859	107	0,202318801	0,237175653	0,460344378	-0,099566165	-0,08463124	0,46589067	0,72489067	**-0,53510933**
chrXII	453752	453908	156	0,678195419	0,959820221	0,414034764	-0,21717813	-0,18460141	-0,178404847	-0,262404847	**-1,522404847**
chrXII	453752	453911	159	0,63260245	0,667056524	0,486744956	-0,033231584	-0,028246847	-0,062007064	-0,167007064	**-1,427007064**
chrXII	453752	453914	162	1,07428434	1,05617283	0,504250616	0,010654916	0,009056678	0,044899598	-0,081100402	**-1,341100402**
chrXII	453752	453916	164	2,011789772	1,912228703	0,512686111	0,031804726	0,027034017	0,011069017	-0,128930983	**-1,388930983**
chrXII	453752	453922	170	2,63584354	3,25004762	0,447824037	-0,13116084	-0,111486714	0,054166177	-0,127833823	**-1,387833823**
chrXII	453752	453924	172	0,330549028	0,329822392	0,500550172	0,001379076	0,001172215	0,106453431	-0,089546569	**-1,349546569**
chrXII	453753	453913	160	0,037044288	0,062999783	0,370279692	-0,331112667	-0,281445767	-0,273741933	-0,385741933	**-1,645741933**
chrXII	453753	453919	166	0,085486818	0,062999783	0,575720754	0,190958009	0,162314307	0,279053599	0,125053599	**-1,134946401**
chrXII	453753	453922	169	0,102584181	0,122293696	0,456177292	-0,110069087	-0,093558724	0,021229332	-0,153770668	**-1,413770668**
chrXII	453754	453910	156	0,182371877	0,333528262	0,353502284	-0,375882075	-0,319499764	-0,306313296	-0,390313296	**-1,650313296**
chrXII	453754	453924	170	0,045592969	0,096352609	0,321200349	-0,464344846	-0,394693119	-0,231342306	-0,413342306	**-1,673342306**
chrXII	453774	453910	136	0,09688506	0,077823261	0,554553208	0,137173592	0,116597553	0,07479581	0,13079581	**-1,12920419**
chrXII	453774	453913	139	0,797876963	0,733762177	0,520930122	0,052488125	0,044614907	0,004857857	0,039857857	**-1,220142143**
chrXII	453774	453914	140	0,091185939	0,17417587	0,34362872	-0,402579708	-0,342192752	-0,310051489	-0,282051489	**-1,542051489**
chrXII	453774	453916	142	0,076938136	0,081529131	0,485514374	-0,036318061	-0,030870352	-0,051713418	-0,037713418	**-1,297713418**
chrXII	453774	453919	145	9,870877861	5,45504002	0,644064384	0,369344136	0,313942516	0,127412679	0,120412679	**-1,139587321**
chrXII	453774	453922	148	0,04844253	0,155646522	0,237359767	-0,714821199	-0,607598019	-0,706673623	-0,734673623	**-1,994673623**
chrXII	453774	453925	151	0,085486818	0,081529131	0,51184823	0,029703476	0,025247955	-0,099783162	-0,148783162	**-1,408783162**
chrXII	453774	453962	188	0,504372223	0,326116523	0,607319757	0,272340151	0,231489128	0,251958584	-0,056041416	**-1,316041416**
chrXII	453775	453908	133	0,703841464	0,930173264	0,430743647	-0,174481212	-0,14830903	-0,120308731	-0,043308731	**-1,303308731**
chrXII	453775	453911	136	0,176672756	0,155646522	0,531635591	0,07938196	0,067474666	0,034226704	0,090226704	**-1,169773296**
chrXII	453775	453914	139	7,81349512	5,27345241	0,597044888	0,245705487	0,208849664	0,16854541	0,20354541	**-1,05645459**
chrXII	453775	453916	141	0,276407377	0,34835174	0,442422318	-0,144830582	-0,123105995	-0,198427921	-0,177427921	**-1,437427921**
chrXII	453775	453919	144	0,037044288	0,181587609	0,169436794	-0,956393263	-0,812934274	-0,918844761	-0,918844761	**-2,178844761**
chrXII	453775	453922	147	0,447381012	0,503998263	0,470244648	-0,074654896	-0,063456662	-0,220005416	-0,241005416	**-1,501005416**
chrXII	453781	453908	127	6,308927132	6,407448501	0,496126201	-0,009710327	-0,008253778	0,268710526	0,387710526	**-0,872289474**
chrXII	453781	453911	130	0,094035499	0,08894087	0,51392155	0,034903236	0,029667761	0,161371498	0,259371498	**-1,000628502**
chrXII	453781	453914	133	0,777930039	0,796761959	0,494020443	-0,014989087	-0,012740724	0,00089945	0,07789945	**-1,18210055**
chrXII	453781	453916	135	0,24506221	0,200116957	0,55047996	0,126874057	0,107842948	0,109813167	0,172813167	**-1,087186833**
chrXII	453781	453922	141	0,473027057	0,507704132	0,482320805	-0,044329684	-0,037680231	-0,106406678	-0,085406678	**-1,345406678**
chrXII	453782	453908	126	0,082637257	0,096352609	0,461686791	-0,096185077	-0,081757315	0,172933344	0,298933344	**-0,961066656**
chrXII	453782	453911	129	0,085486818	0,17788174	0,324590066	-0,454901459	-0,386666241	-0,157732098	-0,052732098	**-1,312732098**
chrXII	453782	453913	131	0,373292436	0,226058044	0,622828292	0,312917403	0,265979793	0,330479689	0,421479689	**-0,838520311**
chrXII	453782	453914	132	0,088336378	0,096352609	0,478298027	-0,054425638	-0,046261792	0,085987547	0,169987547	**-1,090012453**
chrXII	453782	453919	137	0,173823196	0,196411088	0,469495137	-0,076539017	-0,065058164	-0,006429412	0,042570588	**-1,217429412**
chrXII	453782	453922	140	0,068389454	0,062999783	0,520510322	0,051434423	0,043719259	0,085002832	0,113002832	**-1,146997168**
chrXII	453782	453925	143	0,213717044	0,151940653	0,584472981	0,213349921	0,181347433	0,120070199	0,127070199	**-1,132929801**
chrXII	453782	453929	147	0,190920559	0,151940653	0,556845022	0,142974962	0,121528717	-0,029697855	-0,050697855	**-1,310697855**
chrXII	453782	453937	155	0,085486818	0,100058479	0,460732874	-0,09858756	-0,083799426	-0,20462973	-0,28162973	**-1,54162973**
chrXII	453782	453962	180	0,059840772	0,074117392	0,446712395	-0,133971905	-0,113876119	0,049196035	-0,202803965	**-1,462803965**
chrXII	453783	453911	128	0,059840772	0,070411522	0,459422021	-0,101889932	-0,086606442	0,121313982	0,233313982	**-1,026686018**
chrXII	453783	453913	130	0,059840772	0,059293913	0,502295129	0,005753068	0,004890107	0,137629418	0,235629418	**-1,024370582**
chrXII	453783	453914	131	0,028495606	0,122293696	0,18897631	-0,881674934	-0,749423694	-0,68682847	-0,59582847	**-1,85582847**
chrXII	453812	453913	101	0,156725832	0,222352175	0,413439528	-0,218706025	-0,185900121	0,286590897	0,587590897	**-0,672409103**
chrXII	453812	453919	107	0,413186285	0,411351523	0,5011126	0,002788878	0,002370546	0,540754032	0,799754032	**-0,460245968**
chrXII	453812	453962	150	2,68428607	1,571288701	0,630769335	0,333891639	0,283807893	0,154712147	0,112712147	**-1,147287853**
chrXII	453812	454001	189	0,111132863	0,100058479	0,52621884	0,065768269	0,055903029	0,101547112	-0,213452888	**-1,473452888**
chrXII	453812	454001	189	0,111132863	0,100058479	0,52621884	0,065768269	0,055903029	0,101547112	-0,213452888	**-1,473452888**
chrXII	453851	453958	107	0,179522317	0,218646305	0,450870076	-0,123463404	-0,104943894	0,441364284	0,700364284	**-0,559635716**
chrXII	453851	453961	110	0,028495606	0,133411305	0,175999936	-0,930717198	-0,791109618	-0,118559031	0,119440969	**-1,140559031**
chrXII	453851	453962	111	0,102584181	0,133411305	0,434687048	-0,164453569	-0,139785534	0,37049467	0,60149467	**-0,65850533**
chrXII	453851	454001	150	1,421930731	0,770820872	0,648468677	0,381189491	0,324011068	0,202557613	0,160557613	**-1,099442387**
chrXII	453851	454001	150	1,421930731	0,770820872	0,648468677	0,381189491	0,324011068	0,202557613	0,160557613	**-1,099442387**
chrXII	453909	454072	163	0,094035499	0,129705435	0,420287416	-0,201158254	-0,170984516	-0,107128358	-0,240128358	**-1,500128358**
chrXII	453918	454080	162	0,282106498	0,218646305	0,56336479	0,159505761	0,135579897	0,151011692	0,025011692	**-1,234988308**
chrXII	453922	454079	157	0,666797177	0,548468698	0,548684194	0,122337654	0,103987006	0,004428209	-0,086571791	**-1,346571791**
chrXII	453929	454079	150	0,051292091	0,111176087	0,315705458	-0,47974192	-0,407780632	-0,531161087	-0,573161087	**-1,833161087**
chrXII	453929	454097	168	0,652549374	1,119172613	0,36831364	-0,336323037	-0,285874581	-0,159762446	-0,327762446	**-1,587762446**
chrXII	453929	454108	179	0,108283302	0,148234783	0,42212736	-0,196454145	-0,166986023	-0,022671331	-0,267671331	**-1,527671331**
chrXII	453959	454097	138	0,108283302	0,111176087	0,493409293	-0,016521205	-0,014043024	0,015511747	0,057511747	**-1,202488253**
chrXII	453959	454108	149	0,48727486	0,300175436	0,618800783	0,302332726	0,256982817	0,118025928	0,083025928	**-1,176974072**
chrXII	453959	454112	153	0,156725832	0,077823261	0,668200546	0,434949741	0,36970728	0,238955318	0,175955318	**-1,084044682**
chrXII	453960	454116	156	0,068389454	0,055588044	0,551627959	0,129775456	0,110309137	0,131896953	0,047896953	**-1,212103047**
chrXII	453964	454097	133	0,076938136	0,107470218	0,417216109	-0,209020522	-0,177667444	-0,160610196	-0,083610196	**-1,343610196**
chrXII	453964	454108	144	0,581310359	0,363175219	0,61547828	0,293626349	0,249582396	0,147478582	0,147478582	**-1,112521418**
chrXII	453971	454123	152	0,108283302	0,077823261	0,581834946	0,206589951	0,175601459	0,035613079	-0,020386921	**-1,280386921**
chrXII	454059	454216	157	0,045592969	0,040764565	0,527955893	0,070132481	0,059612609	-0,046576609	-0,137576609	**-1,397576609**
chrXII	454111	454268	157	0,108283302	0,122293696	0,469618839	-0,076228037	-0,064793831	-0,180530374	-0,271530374	**-1,531530374**
chrXII	454120	454267	147	0,059840772	0,081529131	0,423292165	-0,193478389	-0,164456631	-0,309901998	-0,330901998	**-1,590901998**
chrXII	454120	454277	157	0,09688506	0,08894087	0,521375354	0,053605728	0,045564869	-0,073361653	-0,164361653	**-1,424361653**
chrXII	454124	454277	153	0,199469241	0,17788174	0,528604008	0,071761159	0,060996985	-0,076493395	-0,139493395	**-1,399493395**
chrXII	454131	454277	146	0,14532759	0,148234783	0,495048423	-0,012412082	-0,010550269	-0,151230991	-0,165230991	**-1,425230991**
chrXII	454141	454277	136	0,213717044	0,229763914	0,481908051	-0,045365348	-0,038560545	-0,076053521	-0,020053521	**-1,280053521**
chrXII	454196	454346	150	0,105433742	0,037058696	0,739925174	0,64311474	0,546647529	0,420291519	0,378291519	**-0,881708481**
chrXII	454196	454346	150	0,105433742	0,037058696	0,739925174	0,64311474	0,546647529	0,420291519	0,378291519	**-0,881708481**
chrXII	454221	454392	171	0,501522663	0,511410002	0,495119449	-0,012234032	-0,010398928	0,152528067	-0,036471933	**-1,296471933**
chrXII	454221	454392	171	0,501522663	0,511410002	0,495119449	-0,012234032	-0,010398928	0,152528067	-0,036471933	**-1,296471933**

Chrom	Start	End	Length	Section A	Section B	A/A+B	Z-score	Z * 0.85	Phase correction	Length correction	ΔLKnuc
chrXII	454244	454392	148	0,04844253	0,066705652	0,420697304	-0,200109928	-0,170093439	-0,27818022	-0,30618022	**-1,56618022**
chrXII	454244	454402	158	0,410336724	0,244587392	0,626540868	0,322705529	0,274299699	0,237017651	0,139017651	**-1,120982349**
chrXII	454248	454374	126	0,433133209	0,633703698	0,405997586	-0,23785292	-0,202174982	0,055687691	0,181687691	**-1,078312309**
chrXII	454248	454392	144	0,04844253	0,051882174	0,482857442	-0,042983253	-0,036535765	-0,138153008	-0,138153008	**-1,398153008**
chrXII	454248	454398	150	0,444531451	0,307587175	0,591039014	0,230218519	0,195685741	0,05403379	0,01203379	**-1,24796621**
chrXII	454273	454436	163	0,416035845	0,35205761	0,541647429	0,104584969	0,088897224	0,147307966	0,014307966	**-1,245692034**
chrXII	454277	454398	121	0,054141651	0,048176305	0,529151025	0,07313593	0,062165541	0,464726559	0,625726559	**-0,634273441**
chrXII	454277	454402	125	0,037044288	0,133411305	0,217325152	-0,781258786	-0,664069968	-0,295699207	-0,162699207	**-1,422699207**
chrXII	454277	454438	161	0,373292436	0,248293262	0,600548625	0,254767408	0,216552296	0,230628115	0,111628115	**-1,148371885**
chrXII	454277	454446	169	0,954602795	1,600935658	0,373542724	-0,322484804	-0,274112083	-0,151449775	-0,326449775	**-1,586449775**
chrXII	454398	454551	153	0,091185939	0,085235	0,516865737	0,042288735	0,035945424	-0,099643255	-0,162643255	**-1,422643255**
chrXII	454398	454552	154	5,220394989	2,894284141	0,643327346	0,367366967	0,312261922	0,148413881	0,078413881	**-1,181586119**
chrXII	454398	454555	157	0,208017923	0,129705435	0,615941769	0,294839538	0,250613607	0,135507463	0,044507463	**-1,215492537**
chrXII	454398	454567	169	0,176672756	0,125999566	0,583709654	0,211392905	0,179683969	0,297796923	0,122796923	**-1,137203077**
chrXII	454398	454573	175	1,159771157	1,782523267	0,394172367	-0,268460684	-0,228191582	0,013821164	-0,203178836	**-1,463178836**
chrXII	454398	454575	177	0,062690333	0,037058696	0,628480635	0,327831948	0,278657156	0,439752423	0,208752423	**-1,051247577**
chrXII	454404	454552	148	0,336248149	0,211234566	0,61417126	0,290207525	0,246676396	0,143569962	0,115569962	**-1,144430038**
chrXII	454404	454555	151	0,960301917	0,507704132	0,654153924	0,396559731	0,337075771	0,208942116	0,159942116	**-1,100057884**
chrXII	454422	454552	130	0,3903898	0,311293045	0,556362184	0,141752339	0,120489488	0,241473258	0,339473258	**-0,920526742**
chrXII	454422	454555	133	0,088336378	0,044470435	0,665149446	0,426558255	0,362574517	0,38480568	0,46180568	**-0,79819432**
chrXII	454422	454567	145	1,259505778	0,692997611	0,645072262	0,372050197	0,316242667	0,12828311	0,12128311	**-1,13871689**
chrXII	454422	454573	151	0,116831984	0,070411522	0,623957468	0,315891237	0,268507552	0,147845437	0,098845437	**-1,161154563**
chrXII	454422	454575	153	0,703841464	0,452116089	0,608881756	0,276405688	0,234944834	0,112068247	0,049068247	**-1,210931753**
chrXII	454521	454678	157	0,561363435	0,522527611	0,517915004	0,044921358	0,038183155	-0,075562991	-0,166562991	**-1,426562991**
chrXII	454553	454712	159	0,718089267	0,52623348	0,577092453	0,194460792	0,165291673	0,133082602	0,028082602	**-1,231917398**
chrXII	454553	454715	162	0,065539893	0,048176305	0,576346155	0,192554744	0,163671533	0,188931362	0,062931362	**-1,197068638**
chrXII	454553	454733	180	0,074088575	0,125999566	0,370279692	-0,331112667	-0,281445767	-0,120580235	-0,372580235	**-1,632580235**
chrXII	454555	454703	148	0,766531797	0,507704132	0,601561908	0,257391992	0,218783194	0,112447845	0,084447845	**-1,175552155**
chrXII	454560	454678	118	0,051292091	0,08894087	0,365763444	-0,34309514	-0,291630869	0,158964129	0,340964129	**-0,919035871**
chrXII	454560	454712	152	0,381841118	0,366881088	0,509990374	0,025044772	0,021288056	-0,122878957	-0,178878957	**-1,438878957**
chrXII	454560	454715	155	1,117027749	0,919055655	0,54861591	0,122165207	0,103840426	-0,007898696	-0,084898696	**-1,344898696**
chrXII	454560	454724	164	0,142478029	0,074117392	0,657807209	0,406485945	0,345513053	0,331403884	0,191403884	**-1,068596116**
chrXII	454560	454733	173	0,216566604	0,237175653	0,477289917	-0,056956516	-0,048413039	0,08994852	-0,11305148	**-1,37305148**
chrXII	454562	454712	150	0,168124074	0,085235	0,663580235	0,422254138	0,358916017	0,212326297	0,170326297	**-1,089673703**
chrXII	454570	454712	142	0,082637257	0,051882174	0,614314648	0,290582424	0,24699506	0,234180798	0,248180798	**-1,011819202**
chrXII	454570	454715	145	0,059840772	0,048176305	0,553993628	0,135757811	0,11539414	-0,066321638	-0,073321638	**-1,333321638**
chrXII	454570	454724	154	0,797876963	0,43729261	0,645965526	0,374450809	0,318283187	0,156046954	0,086046954	**-1,173953046**
chrXII	454570	454733	163	0,113982423	0,129705435	0,467739443	-0,080953558	-0,068810524	-0,013490175	-0,146490175	**-1,406490175**
chrXII	454576	454712	136	0,094035499	0,144528914	0,394172367	-0,268460684	-0,228191582	-0,272847827	-0,216847827	**-1,476847827**
chrXII	454576	454715	139	0,116831984	0,122293696	0,488579829	-0,028630034	-0,024335529	-0,066344565	-0,031344565	**-1,291344565**
chrXII	454576	454724	148	0,133929347	0,129705435	0,508010916	0,020081738	0,017069477	-0,08348199	-0,11148199	**-1,37148199**
chrXII	454576	454733	157	1,789524046	1,619465006	0,524942738	0,062562961	0,053178517	-0,061750071	-0,152750071	**-1,412750071**
chrXII	454576	454772	196	0,059840772	0,051882174	0,535617563	0,08939893	0,07598909	0,130076926	-0,233923074	**-1,493923074**
chrXII	454587	454746	159	0,085486818	0,085235	0,500737507	0,001848658	0,001571359	-0,030125026	-0,135125026	**-1,395125026**
chrXII	454592	454742	150	0,14532759	0,107470218	0,574876781	0,188803997	0,160483398	0,019809661	-0,022190339	**-1,282190339**
chrXII	454626	454724	98	0,051292091	0,048176305	0,515662191	0,039269381	0,033378974	0,581862749	0,903862749	**-0,356137251**
chrXII	454626	454733	107	0,128230226	0,163058261	0,440217282	-0,150418352	-0,127855599	0,425385357	0,684385357	**-0,575614643**
chrXII	454626	454772	146	1,12557643	0,70411522	0,615172742	0,292826836	0,24890281	0,106705574	0,092705574	**-1,167294426**
chrXII	454649	454741	92	0,031345166	0,037058696	0,458236793	-0,104876779	-0,089145262	0,277034387	0,641034387	**-0,618965613**
chrXII	454677	454828	151	0,04844253	0,048176305	0,50137771	0,003453413	0,002935401	-0,120219436	-0,169219436	**-1,429219436**
chrXII	454677	454828	151	0,04844253	0,048176305	0,50137771	0,003453413	0,002935401	-0,120219436	-0,169219436	**-1,429219436**
chrXII	454723	454854	131	0,262159574	0,259410871	0,502635026	0,006605078	0,005614317	0,051885394	0,142885394	**-1,117114606**
chrXII	454725	454863	138	0,92895675	0,752291524	0,552539898	0,132081025	0,112268872	0,135889795	0,177889795	**-1,082110205**
chrXII	454725	454886	161	0,062690333	0,055588044	0,530023616	0,075329227	0,064029843	0,081234846	-0,037765154	**-1,297765154**
chrXII	454733	454863	130	0,056991212	0,077823261	0,422738082	-0,194893703	-0,165659647	-0,037206879	0,060793121	**-1,199206879**
chrXII	454733	454886	153	0,170973635	0,26311674	0,39386645	-0,269255726	-0,228867367	-0,350674328	-0,413674328	**-1,673674328**
chrXII	454741	454896	155	0,039893848	0,044470435	0,472876841	-0,06804215	-0,057835827	-0,168428713	-0,245428713	**-1,505428713**
chrXII	454747	454853	106	0,045592969	0,037058696	0,551627959	0,129775456	0,110309137	0,550377411	0,816377411	**-0,443622589**
chrXII	454747	454886	139	0,356195073	0,255705001	0,582113139	0,207302373	0,176207017	0,147166905	0,182166905	**-1,077833095**
chrXII	454825	454972	147	0,034194727	0,055588044	0,380860679	-0,303221116	-0,257737948	-0,399443806	-0,420443806	**-1,680443806**
chrXII	454825	454972	147	0,034194727	0,055588044	0,380860679	-0,303221116	-0,257737948	-0,399443806	-0,420443806	**-1,680443806**
chrXII	454837	454991	154	0,307752543	0,111176087	0,734618073	0,626840405	0,532814345	0,371795248	0,301795248	**-0,958204752**
chrXII	454837	454991	154	0,307752543	0,111176087	0,734618073	0,626840405	0,532814345	0,371795248	0,301795248	**-0,958204752**
chrXII	454864	455021	157	0,173823196	0,081529131	0,68071906	0,46971048	0,399253908	0,296990657	0,205990657	**-1,054009343**
chrXII	454876	455023	147	0,812124766	0,411351523	0,663784638	0,422814341	0,35939219	0,215351213	0,194351213	**-1,065648787**
chrXII	454876	455029	153	0,125380666	0,170470001	0,423797138	-0,192188854	-0,163360526	-0,293796046	-0,356796046	**-1,616796046**
chrXII	454901	455029	128	0,094035499	0,248293262	0,27469354	-0,598678825	-0,508877001	-0,303662519	-0,191662519	**-1,451662519**
chrXII	454901	455074	173	0,210867483	0,255705001	0,451950105	-0,120735916	-0,102625529	0,040995719	-0,162004281	**-1,422004281**
chrXII	454920	455087	167	0,09973462	0,103764348	0,490098899	-0,024820929	-0,021097789	0,147009799	-0,013990201	**-1,273990201**
chrXII	454932	455073	141	0,689593661	0,370586958	0,650449224	0,386533562	0,328553528	0,260644273	0,281644273	**-0,978355727**
chrXII	454933	455023	90	0,190920559	0,407645654	0,318963141	-0,470600177	-0,40001015	0,034285231	0,412285231	**-0,847714769**
chrXII	454933	455024	91	0,031345166	0,140823044	0,18206129	-0,907537593	-0,771406954	-0,376537125	-0,005537125	**-1,265537125**
chrXII	454933	455025	92	0,031345166	0,044470435	0,413439528	-0,218706025	-0,185900121	0,20438963	0,56838963	**-0,69161037**
chrXII	454933	455027	94	0,028495606	0,08894087	0,242646977	-0,69781331	-0,593141313	-0,084638715	0,265361285	**-0,994638715**
chrXII	454933	455029	96	5,867245241	28,82054771	0,16914438	-0,957551906	-0,81391912	-0,257792976	0,078207024	**-1,181792976**
chrXII	454933	455073	140	0,074088575	0,037058696	0,666580246	0,43048963	0,365916185	0,401637802	0,429637802	**-0,830362198**
chrXII	454933	455074	141	0,24506221	0,237175653	0,508177041	0,020498237	0,017423501	-0,087709506	-0,066709506	**-1,326709506**
chrXII	454933	455076	143	0,669646737	0,481763045	0,581588542	0,205959029	0,175065175	0,107599857	0,114599857	**-1,145400143**
chrXII	454933	455132	199	0,031345166	0,096352609	0,245463684	-0,688834586	-0,585509398	-0,535476381	-0,920476381	**-2,180476381**
chrXII	454964	455132	168	0,190920559	0,192705218	0,497673958	-0,005830555	-0,004955971	0,114320963	-0,053679037	**-1,313679037**
chrXII	454995	455164	169	0,125380666	0,259410871	0,325840498	-0,451428154	-0,383713931	-0,262455115	-0,437455115	**-1,697455115**
chrXII	455000	455132	132	0,085486818	0,163058261	0,343948944	-0,401709424	-0,34145301	-0,213881427	-0,129881427	**-1,389881427**
chrXII	455015	455166	151	0,481575739	0,318704784	0,601758665	0,257901833	0,219216558	0,093046579	0,044046579	**-1,215953421**
chrXII	455058	455208	150	0,042743409	0,044470435	0,490098899	-0,024820929	-0,021097789	-0,137311083	-0,179311083	**-1,439311083**
chrXII	455068	455223	155	0,162424953	0,118587827	0,577998458	0,196775678	0,167259327	0,055866116	-0,021133884	**-1,281133884**
chrXII	455095	455269	174	0,14817715	0,222352175	0,399906675	-0,253588671	-0,21555037	0,022525549	-0,187474451	**-1,447474451**
chrXII	455096	455223	127	0,054141651	0,096352609	0,359758911	-0,359103289	-0,305237796	-0,048315108	0,070684892	**-1,189315108**
chrXII	455096	455240	144	0,849169054	0,70782109	0,545391413	0,11402601	0,096922109	-0,016225788	-0,016225788	**-1,276225788**
chrXII	455096	455243	147	0,14817715	0,111176087	0,571333335	0,179769754	0,152804291	0,023903006	0,002903006	**-1,257096994**
chrXII	455096	455245	149	0,971700159	0,61146848	0,613769206	0,28915654	0,245783059	0,095716462	0,060716462	**-1,199283538**
chrXII	455096	455249	153	0,056991212	0,037058696	0,605967759	0,268824834	0,228501109	0,111772271	0,048772271	**-1,211227729**
chrXII	455097	455240	143	0,04844253	0,044470435	0,521375354	0,053605728	0,045564869	-0,033935047	-0,026935047	**-1,286935047**
chrXII	455097	455243	146	0,527168708	0,318704784	0,623224056	0,313959381	0,266865474	0,131253075	0,117253075	**-1,142746925**
chrXII	455098	455243	145	0,051292091	0,040764565	0,557179598	0,143822286	0,122248943	-0,059139823	-0,066139823	**-1,326139823**
chrXII	455098	455249	151	0,151026711	0,151940653	0,498491683	-0,0037808	-0,00321368	-0,120443897	-0,169443897	**-1,429443897**
chrXII	455129	455293	164	0,059840772	0,044470435	0,573675388	0,185739279	0,157878387	0,120678445	-0,019321555	**-1,279321555**

Chrom	Start	End	Length	Section A	Section B	A/A+B	Z-score	Z * 0.85	Phase correction	Length correction	ΔLKnuc
chrXII	455138	455289	151	0,091185939	0,081529131	0,527955893	0,070132481	0,059612609	-0,054275748	-0,103275748	**-1,363275748**
chrXII	455182	455338	156	0,034194727	0,044470435	0,434687048	-0,164453569	-0,139785534	-0,112895455	-0,196895455	**-1,456895455**
chrXII	455217	455366	149	0,059840772	0,051882174	0,535617563	0,08939893	0,07598909	-0,076046834	-0,111046834	**-1,371046834**
chrXII	455221	455395	174	0,054141651	0,218646305	0,198475225	-0,847080146	-0,720018124	-0,487052919	-0,697052919	**-1,957052919**
chrXII	455247	455381	134	0,085486818	0,08894087	0,490098899	-0,024820929	-0,021097789	-0,033894193	0,036105807	**-1,223894193**
chrXII	455254	455401	147	0,133929347	0,103764348	0,563453511	0,159731003	0,135771353	0,015886157	-0,005113843	**-1,265113843**
chrXII	455255	455400	145	0,102584181	0,074117392	0,58055047	0,203301913	0,172806626	-0,000794559	-0,007794559	**-1,267794559**
chrXII	455278	455423	145	0,068389454	0,08894087	0,434687048	-0,164453569	-0,139785534	-0,306385181	-0,313385181	**-1,573385181**
chrXII	455297	455447	150	0,265009134	0,26311674	0,501791613	0,004490922	0,003817284	-0,123154852	-0,165154852	**-1,425154852**
chrXII	455367	455512	145	1,410532489	0,763409133	0,648836415	0,382180925	0,324853786	0,155291229	0,148291229	**-1,111708771**
chrXII	455367	455531	164	0,068389454	0,085235	0,445172966	-0,137866493	-0,117186519	-0,155598298	-0,295598298	**-1,555598298**
chrXII	455367	455549	182	0,219416165	0,222352175	0,49667698	-0,008329671	-0,007080221	0,166341286	-0,099658714	**-1,359658714**
chrXII	455377	455512	135	0,062690333	0,037058696	0,628480635	0,327831948	0,278657156	0,283073674	0,346073674	**-0,913926326**
chrXII	455377	455531	154	0,567062556	0,233469783	0,708356837	0,548590764	0,466302149	0,324069805	0,254069805	**-1,005930195**
chrXII	455377	455549	172	0,102584181	0,077823261	0,568624996	0,172874585	0,146943398	0,247276298	0,051276298	**-1,208723702**
chrXII	455397	455503	106	0,085486818	0,103764348	0,451710916	-0,121339881	-0,103138899	0,317438125	0,583438125	**-0,676561875**
chrXII	455397	455512	115	0,079787696	0,081529131	0,49460244	-0,013530088	-0,011500575	0,648722201	0,851722201	**-0,408277799**
chrXII	455397	455531	134	0,105433742	0,100058479	0,513078994	0,032790051	0,027871543	0,014470214	0,084470214	**-1,175529786**
chrXII	455397	455549	152	1,761028441	1,334113048	0,568965408	0,173740784	0,147679667	0,021277837	-0,034722163	**-1,294722163**
chrXII	455397	455568	171	0,034194727	0,059293913	0,365763444	-0,34309514	-0,291630869	-0,132457993	-0,321457993	**-1,581457993**
chrXII	455410	455512	102	0,062690333	0,062999783	0,498768996	-0,003085674	-0,002622823	0,504219582	0,798219582	**-0,461780418**
chrXII	455410	455549	139	0,128230226	0,196411088	0,394990473	-0,266335355	-0,226385052	-0,270215213	-0,235215213	**-1,495215213**
chrXII	455410	455560	150	0,156725832	0,037058696	0,808763392	0,873348384	0,742346126	0,624840622	0,582840622	**-0,677159378**
chrXII	455410	455568	158	0,524319147	0,344645871	0,603383492	0,262114698	0,222797493	0,199292228	0,101292228	**-1,158707772**
chrXII	455410	455569	159	0,29065518	0,237175653	0,550659722	0,127328308	0,108229062	0,083393586	-0,021606414	**-1,281606414**
chrXII	455416	455571	155	0,088336378	0,118587827	0,426902103	-0,184266739	-0,156626728	-0,270926045	-0,347926045	**-1,607926045**
chrXII	455422	455569	147	1,003045325	0,537351089	0,651160517	0,388455518	0,33018719	0,218737231	0,197737231	**-1,062262769**
chrXII	455591	455751	160	0,045592969	0,077823261	0,36942442	-0,33337817	-0,283371444	-0,273431743	-0,385431743	**-1,645431743**
chrXII	455701	455870	169	0,079787696	0,137117174	0,367846494	-0,337562389	-0,286928031	-0,160963139	-0,335963139	**-1,595963139**
chrXII	455747	455895	148	0,960301917	0,544762828	0,638046915	0,353243137	0,300256667	0,200387699	0,172387699	**-1,087612301**
chrXII	455749	455889	140	0,336248149	0,26682261	0,557560028	0,144785863	0,123067983	0,156871321	0,184871321	**-1,075128679**
chrXII	455783	455958	175	0,045592969	0,111176087	0,290828881	-0,550964862	-0,468320133	-0,227204164	-0,444204164	**-1,704204164**
chrXII	455869	456021	152	0,045592969	0,040764565	0,527955893	0,070132481	0,059612609	-0,061701939	-0,117701939	**-1,377701939**
chrXII	455903	456067	164	0,085486818	0,077823261	0,523463207	0,058847485	0,050020363	0,010280425	-0,129719575	**-1,389719575**
chrXII	455918	456039	121	0,287805619	0,874585221	0,247597976	-0,682068004	-0,579757803	-0,181252299	-0,020252299	**-1,280252299**
chrXII	455926	456086	160	0,262159574	0,259410871	0,502635026	0,006605078	0,005614317	0,007330386	-0,104669614	**-1,364669614**
chrXII	456022	456173	151	2,191312089	1,330407179	0,62222793	0,311337397	0,264636787	0,158908992	0,109908992	**-1,150091008**
chrXII	456022	456205	183	0,210867483	0,26311674	0,444882916	-0,138600522	-0,117810443	0,005903191	-0,267096809	**-1,527096809**
chrXII	456022	456205	183	0,210867483	0,26311674	0,444882916	-0,138600522	-0,117810443	0,005903191	-0,267096809	**-1,527096809**
chrXII	456022	456211	189	0,111132863	0,070411522	0,612152576	0,284933816	0,242193743	0,291546318	-0,023453682	**-1,283453682**
chrXII	456022	456211	189	0,111132863	0,070411522	0,612152576	0,284933816	0,242193743	0,291546318	-0,023453682	**-1,283453682**
chrXII	456022	456220	198	0,062690333	0,055588044	0,530023616	0,075329227	0,064029843	0,118059114	-0,259940886	**-1,519940886**
chrXII	456022	456220	198	0,062690333	0,055588044	0,530023616	0,075329227	0,064029843	0,118059114	-0,259940886	**-1,519940886**
chrXII	456025	456173	148	0,085486818	0,037058696	0,69759239	0,517488464	0,439865194	0,306358185	0,278358185	**-0,981641815**
chrXII	456025	456182	157	0,071239015	0,037058696	0,657807209	0,406485945	0,345513053	0,229767352	0,138767352	**-1,121232648**
chrXII	456042	456173	131	0,111132863	0,037058696	0,74992708	0,674260298	0,573121253	0,606712094	0,697712094	**-0,562287906**
chrXII	456045	456173	128	0,091185939	0,081529131	0,527955893	0,070132481	0,059612609	0,254983651	0,366983651	**-0,893016349**
chrXII	456045	456175	130	0,396088921	0,396528045	0,499722991	-0,000694358	-0,000590204	0,134288824	0,232288824	**-1,027711176**
chrXII	456045	456205	160	0,088336378	0,081529131	0,520037168	0,050246866	0,042709836	0,035943381	-0,076056619	**-1,336056619**
chrXII	456045	456205	160	0,088336378	0,081529131	0,520037168	0,050246866	0,042709836	0,035943381	-0,076056619	**-1,336056619**
chrXII	456056	456173	117	0,424584527	0,570703915	0,426594451	-0,185051167	-0,157293492	0,332357375	0,521357375	**-0,738642625**
chrXII	456056	456175	119	0,059840772	0,074117392	0,446712395	-0,133971905	-0,113876119	0,303114708	0,478114708	**-0,781885292**
chrXII	456056	456205	149	4,453863192	3,146283272	0,586023337	0,217327245	0,184728158	0,033871174	-0,001128826	**-1,261128826**
chrXII	456056	456205	149	4,453863192	3,146283272	0,586023337	0,217327245	0,184728158	0,033871174	-0,001128826	**-1,261128826**
chrXII	456056	456211	155	0,250761331	0,26311674	0,487978268	-0,030138575	-0,025617789	-0,140494129	-0,217494129	**-1,477494129**
chrXII	456056	456211	155	0,250761331	0,26311674	0,487978268	-0,030138575	-0,025617789	-0,140494129	-0,217494129	**-1,477494129**
chrXII	456056	456220	164	0,262159574	0,248293262	0,513582363	0,034052516	0,028944639	-0,010412803	-0,150412803	**-1,410412803**
chrXII	456056	456220	164	0,262159574	0,248293262	0,513582363	0,034052516	0,028944639	-0,010412803	-0,150412803	**-1,410412803**
chrXII	456063	456215	152	0,361894194	0,170470001	0,679786878	0,467102923	0,397037484	0,269587555	0,213587555	**-1,046412445**
chrXII	456063	456215	152	0,361894194	0,170470001	0,679786878	0,467102923	0,397037484	0,269587555	0,213587555	**-1,046412445**
chrXII	456064	456214	150	0,122531105	0,070411522	0,635064977	0,345298405	0,293503645	0,176412996	0,134412996	**-1,125587004**
chrXII	456064	456214	150	0,122531105	0,070411522	0,635064977	0,345298405	0,293503645	0,176412996	0,134412996	**-1,125587004**
chrXII	456066	456173	107	0,165274514	0,155646522	0,515000562	0,037609696	0,031968242	0,585141421	0,844141421	**-0,415858579**
chrXII	456067	456205	138	0,373292436	0,333528262	0,528128898	0,070567214	0,059982132	0,080974611	0,122974611	**-1,137025389**
chrXII	456067	456211	144	1,880709985	0,852350003	0,688133445	0,490566465	0,416981495	0,2889066	0,2889066	**-0,9710934**
chrXII	456067	456215	148	1,553010518	1,048761091	0,596905014	0,245344146	0,208542524	0,074794215	0,046794215	**-1,213205785**
chrXII	456067	456220	153	0,14817715	0,074117392	0,666580246	0,43048963	0,365916185	0,241334896	0,178334896	**-1,081665104**
chrXII	456067	456266	199	0,170973635	0,214940436	0,443035505	-0,14327752	-0,121785892	-0,074861336	-0,459861336	**-1,719861336**
chrXII	456080	456173	93	0,205168362	0,196411088	0,510903539	0,027334523	0,023234344	0,422860872	0,779860872	**-0,480139128**
chrXII	456080	456205	125	0,527168708	0,396528045	0,570716208	0,178197868	0,151468187	0,492116465	0,625116465	**-0,634883535**
chrXII	456080	456211	131	0,199469241	0,092646739	0,682842618	0,475662609	0,404313218	0,44614102	0,53714102	**-0,72285898**
chrXII	456080	456220	140	2,137170438	1,367465874	0,609812331	0,278829937	0,237005447	0,275744062	0,303744062	**-0,956255938**
chrXII	456084	456215	131	0,068389454	0,070411522	0,492715945	-0,018259434	-0,015520519	0,030076617	0,121076617	**-1,138923383**
chrXII	456084	456243	159	0,316301225	0,277940218	0,53227729	0,080995639	0,068846293	0,055517876	-0,049482124	**-1,309482124**
chrXII	456084	456266	182	0,082637257	0,103764348	0,443329106	-0,142534017	-0,121153915	0,053155818	-0,212844182	**-1,472844182**
chrXII	456115	456215	100	0,173823196	0,233469783	0,426776803	-0,184586205	-0,156898275	0,258010743	0,566010743	**-0,693989257**
chrXII	456115	456243	128	0,062690333	0,103764348	0,376621026	-0,314367347	-0,267212245	-0,067431524	0,044568476	**-1,215431524**
chrXII	456115	456266	151	7,235034322	4,346985016	0,624678142	0,317790687	0,270122084	0,15948707	0,11048707	**-1,14951293**
chrXII	456115	456281	166	0,373292436	0,548468698	0,40497741	-0,240484318	-0,20441167	-0,083622718	-0,237622718	**-1,497622718**
chrXII	456120	456290	170	0,039893848	0,040764565	0,49460244	-0,013530088	-0,011500575	0,160363943	-0,021636057	**-1,281636057**
chrXII	456124	456215	91	0,059840772	0,100058479	0,374240479	-0,320642986	-0,272546538	0,103798198	0,474798198	**-0,785201802**
chrXII	456124	456266	142	0,427434088	0,448410219	0,488025194	-0,030020896	-0,025517762	-0,033910624	-0,019910624	**-1,279910624**
chrXII	456124	456281	157	1,478921943	1,382289353	0,516886657	0,042341221	0,035990038	-0,078016872	-0,169016872	**-1,429016872**
chrXII	456124	456333	209	0,250761331	0,296469566	0,458236793	-0,104876779	-0,089145262	0,082135938	-0,372864062	**-1,632864062**
chrXII	456181	456266	85	1,071434779	1,385995222	0,435998087	-0,161123447	-0,13695493	0,256336774	0,669336774	**-0,590663226**
chrXII	456181	456281	100	0,578460798	0,867173481	0,40014325	-0,252976337	-0,215029886	0,222190456	0,530190456	**-0,729809544**
chrXII	456181	456287	106	0,09973462	0,344645871	0,224435191	-0,757299614	-0,643704672	-0,208175501	0,057824499	**-1,202175501**
chrXII	456181	456306	125	0,056991212	0,059293913	0,490098899	-0,024820929	-0,021097789	0,313456953	0,446456953	**-0,813543047**
chrXII	456181	456310	129	1,56440876	1,763993919	0,470017877	-0,075224925	-0,063941186	0,18665456	0,29165456	**-0,96834544**
chrXII	456181	456329	148	0,042743409	0,037058696	0,535617563	0,08939893	0,07598909	-0,06181526	-0,08981526	**-1,34981526**
chrXII	456181	456332	151	0,037044288	0,081529131	0,31241646	-0,489012398	-0,415660538	-0,531742954	-0,580742954	**-1,840742954**
chrXII	456181	456333	152	14,91460009	11,08425591	0,573663706	0,185709488	0,157853065	0,018413455	-0,037586545	**-1,297586545**
chrXII	456181	456336	155	0,250761331	0,248293262	0,502472745	0,006198292	0,005268549	-0,11091953	-0,18791953	**-1,44791953**
chrXII	456181	456349	168	0,330549028	0,218646305	0,6018788	0,258213162	0,219481187	0,351279549	0,183279549	**-1,076720451**
chrXII	456181	456387	206	0,239363089	0,163058261	0,594807131	0,239928434	0,203939169	0,280599079	-0,153400921	**-1,413400921**
chrXII	456186	456333	147	0,045592969	0,070411522	0,393027622	-0,271436616	-0,230721123	-0,346256815	-0,367256815	**-1,627256815**

Chrom	Start	End	Length	Section A	Section B	A/A+B	Z-score	Z * 0.85	Phase correction	Length correction	ΔLKnuc
chrXII	456187	456333	146	0,458779254	0,652233046	0,412938051	-0,219993647	-0,1869946	-0,32371293	-0,33771293	**-1,59771293**
chrXII	456187	456349	162	0,495823542	0,459527828	0,518996002	0,047633924	0,040488835	0,069862664	-0,056137336	**-1,316137336**
chrXII	456194	456333	139	0,3903898	0,381704567	0,505624464	0,014098907	0,011984071	-0,03389814	0,00110186	**-1,25889814**
chrXII	456194	456336	142	0,572761677	0,366881088	0,609552586	0,278153109	0,236430142	0,232499925	0,246499925	**-1,013500075**
chrXII	456213	456319	106	3,975137014	8,638381988	0,31514893	-0,481307652	-0,409111504	0,024846884	0,290846884	**-0,969153116**
chrXII	456213	456320	107	0,09688506	0,192705218	0,33455909	-0,427358568	-0,363254783	0,185371853	0,444371853	**-0,815628147**
chrXII	456213	456321	108	0,065539893	0,229763914	0,221940564	-0,76565581	-0,650807439	0,023574618	0,275574618	**-0,984425382**
chrXII	456213	456333	120	4,331332087	10,28749395	0,296284536	-0,535116832	-0,454849307	-0,055941248	0,112058752	**-1,147941248**
chrXII	456213	456336	123	0,068389454	0,125999566	0,351817475	-0,380418277	-0,323355536	0,103082503	0,250082503	**-1,009917497**
chrXII	456213	456349	136	0,122531105	0,222352175	0,355282823	-0,371096517	-0,31543204	-0,365253966	-0,309253966	**-1,569253966**
chrXII	456213	456352	139	0,054141651	0,051882174	0,510655515	0,026712592	0,022705704	-0,029217269	0,005782731	**-1,254217269**
chrXII	456213	456387	174	0,173823196	0,196411088	0,469495137	-0,076539017	-0,065058164	0,160254557	-0,049745443	**-1,309745443**
chrXII	456213	456419	206	0,133929347	0,159352392	0,456657642	-0,108857794	-0,092529125	-0,0088479	-0,4428479	**-1,7028479**
chrXII	456213	456419	206	0,133929347	0,159352392	0,456657642	-0,108857794	-0,092529125	-0,0088479	-0,4428479	**-1,7028479**
chrXII	456221	456386	165	0,615505086	0,377998697	0,619529685	0,304245806	0,258608935	0,321764652	0,174764652	**-1,085235348**
chrXII	456240	456333	93	0,416035845	0,485468915	0,461490459	-0,096679504	-0,082177578	0,32754207	0,68454207	**-0,57545793**
chrXII	456240	456386	146	0,253610892	0,114881957	0,6882383	0,490862927	0,417233488	0,286336317	0,272336317	**-0,987663683**
chrXII	456240	456387	147	2,305294512	1,552759353	0,597527819	0,246953305	0,20991031	0,099791893	0,078791893	**-1,181208107**
chrXII	456240	456419	179	0,29065518	0,311293045	0,482857442	-0,042983253	-0,036535765	0,116910517	-0,128089483	**-1,388089483**
chrXII	456240	456419	179	0,29065518	0,311293045	0,482857442	-0,042983253	-0,036535765	0,116910517	-0,128089483	**-1,388089483**
chrXII	456240	456447	207	0,165274514	0,129705435	0,560290672	0,151706213	0,128950281	0,261007412	-0,179992588	**-1,439992588**
chrXII	456240	456447	207	0,165274514	0,129705435	0,560290672	0,151706213	0,128950281	0,261007412	-0,179992588	**-1,439992588**
chrXII	456288	456387	99	0,330549028	0,403939784	0,450039568	-0,125561379	-0,106727172	0,355074839	0,670074839	**-0,589925161**
chrXII	456288	456395	107	0,04844253	0,274234349	0,150127056	-1,035888609	-0,880505318	-0,354476424	-0,095476424	**-1,355476424**
chrXII	456288	456419	131	6,274732405	6,296272414	0,499143266	-0,002147515	-0,001825388	0,041872249	0,132872249	**-1,127127751**
chrXII	456288	456447	159	2,251152861	1,923346311	0,539262979	0,098577115	0,083790548	0,068411309	-0,036588691	**-1,296588691**
chrXII	456288	456457	169	0,165274514	0,188999349	0,46651625	-0,084030099	-0,071425584	0,066001998	-0,108998002	**-1,368998002**
chrXII	456288	456471	183	0,413186285	1,030231743	0,286255455	-0,564357421	-0,479703807	-0,369593388	-0,642593388	**-1,902593388**
chrXII	456290	456419	129	0,179522317	0,329822392	0,352457409	-0,378694391	-0,321890232	-0,080364681	0,024635319	**-1,235364681**
chrXII	456290	456447	157	0,606956404	0,904232177	0,401641735	-0,249099947	-0,211734955	-0,312206413	-0,403206413	**-1,663206413**
chrXII	456290	456471	181	0,074088575	0,17417587	0,298426039	-0,528932784	-0,449592866	-0,257796652	-0,516796652	**-1,776796652**
chrXII	456298	456447	149	0,931806311	0,563292176	0,623240756	0,314003358	0,266902854	0,101352444	0,066352444	**-1,193647556**
chrXII	456310	456417	107	0,071239015	0,055588044	0,561702018	0,155285859	0,13199298	0,63140811	0,89040811	**-0,36959189**
chrXII	456310	456419	109	0,065539893	0,122293696	0,34892531	-0,388223533	-0,329990003	0,30713624	0,55213624	**-0,70786376**
chrXII	456310	456447	137	0,082637257	0,118587827	0,410670755	-0,225819886	-0,191946903	-0,136510747	-0,087510747	**-1,347510747**
chrXII	456310	456471	161	0,578460798	0,578115654	0,500149209	0,000374013	0,000317911	0,024638021	-0,094361979	**-1,354361979**
chrXII	456361	456447	86	0,128230226	0,70411522	0,154058903	-1,019179398	-0,866302488	-0,474979226	-0,068979226	**-1,328979226**
chrXII	456361	456466	105	0,028495606	0,418763262	0,063711662	-1,524341906	-1,29569062	-0,729721808	-0,456721808	**-1,716721808**
chrXII	456361	456468	107	1,17401896	8,734734597	0,118483011	-1,182604262	-1,005213622	-0,521622032	-0,262622032	**-1,522622032**
chrXII	456361	456470	109	0,042743409	0,34835174	0,109291585	-1,230304312	-1,045758665	-0,406743856	-0,161743856	**-1,421743856**
chrXII	456361	456471	110	1,256656217	14,53812636	0,079561476	-1,408027415	-1,196823303	-0,50994537	-0,27194537	**-1,53194537**
chrXII	456388	456471	83	0,09688506	0,277940218	0,258480592	-0,648036746	-0,550831234	-0,243338967	0,183661033	**-1,076338967**
chrXII	456388	456551	163	0,322000346	0,281646088	0,533425409	0,083883345	0,071300843	0,121990496	-0,011009504	**-1,271009504**
chrXII	456388	456551	163	0,322000346	0,281646088	0,533425409	0,083883345	0,071300843	0,121990496	-0,011009504	**-1,271009504**
chrXII	456444	456594	150	0,473027057	0,281646088	0,626797257	0,323382625	0,274875231	0,164067773	0,122067773	**-1,137932227**
chrXII	456444	456594	150	0,473027057	0,281646088	0,626797257	0,323382625	0,274875231	0,164067773	0,122067773	**-1,137932227**
chrXII	456444	456595	151	0,199469241	0,188999349	0,513475854	0,033785384	0,028717576	-0,095318252	-0,144318252	**-1,404318252**
chrXII	456444	456595	151	0,199469241	0,188999349	0,513475854	0,033785384	0,028717576	-0,095318252	-0,144318252	**-1,404318252**
chrXII	456444	456619	175	0,051292091	0,118587827	0,301931455	-0,518853495	-0,441025471	-0,190758771	-0,407758771	**-1,667758771**
chrXII	456444	456619	175	0,051292091	0,118587827	0,301931455	-0,518853495	-0,441025471	-0,190758771	-0,407758771	**-1,667758771**
chrXII	456445	456595	150	0,071239015	0,103764348	0,407072261	-0,235082731	-0,199820321	-0,316067859	-0,358067859	**-1,618067859**
chrXII	456447	456594	147	1,142673794	0,926467395	0,552245444	0,131336508	0,111636032	-0,001503292	-0,022503292	**-1,282503292**
chrXII	456447	456595	148	0,074088575	0,096352609	0,434687048	-0,164453569	-0,139785534	-0,276730455	-0,304730455	**-1,564730455**
chrXII	456447	456608	161	0,085486818	0,222352175	0,277699771	-0,589688448	-0,501235181	-0,466538213	-0,585538213	**-1,845538213**
chrXII	456447	456619	172	0,58700948	0,944996743	0,383163901	-0,297181688	-0,252604435	-0,146807876	-0,342807876	**-1,602807876**
chrXII	456479	456585	106	0,113982423	0,17788174	0,390532439	-0,277931585	-0,236241848	0,179795974	0,445795974	**-0,814204026**
chrXII	456479	456594	115	0,068389454	0,125999566	0,351817475	-0,380418277	-0,323355536	0,320601485	0,523601485	**-0,736398515**
chrXII	456479	456608	129	1,800922289	1,912228703	0,485011865	-0,037578525	-0,031941746	0,220386243	0,325386243	**-0,934613757**
chrXII	456479	456609	130	0,088336378	0,100058479	0,468889542	-0,078061561	-0,066352326	0,051295948	0,149295948	**-1,110704052**
chrXII	456479	456653	174	0,056991212	0,059293913	0,490098899	-0,024820929	-0,021097789	0,196798578	-0,013201422	**-1,273201422**
chrXII	456483	456594	111	0,031345166	0,111176087	0,219933277	-0,772418577	-0,656555791	-0,142021328	0,088978672	**-1,171021328**
chrXII	456483	456608	125	0,270708255	0,489174784	0,356249898	-0,368500871	-0,31322574	0,012368728	0,145368728	**-1,114631272**
chrXII	456483	456609	126	0,247911771	0,392822175	0,386918427	-0,287359779	-0,244255812	0,002324501	0,128324501	**-1,131675499**
chrXII	456493	456599	106	0,065539893	0,062999783	0,509880648	0,024769644	0,021054198	0,424854448	0,690854448	**-0,569145552**
chrXII	456493	456608	115	0,054141651	0,100058479	0,351112876	-0,382317666	-0,324970016	0,329839263	0,532839263	**-0,727160737**
chrXII	456493	456653	160	0,284956058	0,340940001	0,455276965	-0,11233987	-0,09548889	-0,110686853	-0,222686853	**-1,482686853**
chrXII	456508	456594	86	0,039893848	0,055588044	0,417815854	-0,20748423	-0,176361595	0,220105638	0,626105638	**-0,633894362**
chrXII	456508	456608	100	0,153876272	0,185293479	0,453685128	-0,116356189	-0,098902761	0,359659711	0,667659711	**-0,592340289**
chrXII	456508	456642	134	0,227964847	0,26311674	0,464209722	-0,089833605	-0,076358564	-0,091826631	-0,021826631	**-1,281826631**
chrXII	456508	456653	145	0,09688506	0,055588044	0,635423938	0,346253615	0,294315573	0,12102827	0,11402827	**-1,14597173**
chrXII	456508	456670	162	0,159575393	0,133411305	0,544650641	0,112157259	0,09533367	0,118697713	-0,007302287	**-1,267302287**
chrXII	456524	456695	171	0,199469241	0,359469349	0,356871478	-0,36683385	-0,311808773	-0,160925187	-0,349925187	**-1,609925187**
chrXII	456587	456738	151	0,051292091	0,096352609	0,34740218	-0,39234359	-0,333492051	-0,461478345	-0,510478345	**-1,770478345**
chrXII	456587	456738	151	0,051292091	0,096352609	0,34740218	-0,39234359	-0,333492051	-0,461478345	-0,510478345	**-1,770478345**
chrXII	456588	456751	163	0,068389454	0,037058696	0,648560019	0,381435717	0,324220359	0,375194338	0,242194338	**-1,017805662**
chrXII	456588	456751	163	0,068389454	0,037058696	0,648560019	0,381435717	0,324220359	0,375194338	0,242194338	**-1,017805662**
chrXII	456594	456751	157	0,102584181	0,122293696	0,456177292	-0,110069087	-0,093558724	-0,190495057	-0,281495057	**-1,541495057**
chrXII	456616	456772	156	0,307752543	0,218646305	0,584637569	0,213771999	0,181706199	0,211014995	0,127014995	**-1,132985005**
chrXII	456634	456751	117	0,039893848	0,059293913	0,402205349	-0,247642923	-0,210496484	0,28589714	0,47489714	**-0,78510286**
chrXII	456634	456762	128	0,738036191	1,045055221	0,413908219	-0,217502911	-0,184877475	0,024891251	0,136891251	**-1,123108749**
chrXII	456634	456781	147	0,270708255	0,229763914	0,540905713	0,102715748	0,087308386	-0,019825975	-0,040825975	**-1,300825975**
chrXII	456649	456803	154	0,250761331	0,207528696	0,547167331	0,118507771	0,100731606	-0,036873488	-0,106873488	**-1,366873488**
chrXII	456653	456762	109	0,031345166	0,044470435	0,413439528	-0,218706025	-0,185900121	0,468337565	0,713337565	**-0,546662435**
chrXII	456653	456803	150	0,19377012	0,081529131	0,703852696	0,535513815	0,455186742	0,336987848	0,294987848	**-0,965012152**
chrXII	456733	456869	136	0,045592969	0,037058696	0,551627959	0,129775456	0,110309137	0,066186264	0,122186264	**-1,137813736**
chrXII	456733	456869	136	0,045592969	0,037058696	0,551627959	0,129775456	0,110309137	0,066186264	0,122186264	**-1,137813736**
chrXII	456766	456920	154	0,062690333	0,048176305	0,56545715	0,164819952	0,140096959	-0,00740753	-0,07740753	**-1,33740753**
chrXII	456780	456887	107	0,039893848	0,470645436	0,078140604	-1,417690283	-1,20503674	-0,744208602	-0,485208602	**-1,745208602**
chrXII	456780	456890	110	0,037044288	0,489174784	0,070397083	-1,472840067	-1,251914057	-0,579302824	-0,341302824	**-1,601302824**
chrXII	456780	456920	140	0,045592969	0,314998914	0,126439256	-1,143385642	-0,971877795	-0,941661016	-0,913661016	**-2,173661016**
chrXII	456794	456903	109	0,076938136	0,200116957	0,277699771	-0,589688448	-0,501235181	0,15961202	0,40461202	**-0,85538798**
chrXII	456795	456903	108	2,815365857	8,842204815	0,241505365	-0,701468431	-0,596248166	0,0864811	0,3384811	**-0,9215189**
chrXII	456795	456904	109	0,037044288	0,137117174	0,21270083	-0,797085011	-0,67752226	-0,000424336	0,244575664	**-1,015424336**
chrXII	456795	456905	110	0,031345166	0,185293479	0,144688711	-1,059488371	-0,900565115	-0,212605321	0,025394679	**-1,234605321**
chrXII	456797	456890	93	0,056991212	0,481763045	0,105783316	-1,249269194	-1,061878815	-0,648908122	-0,291908122	**-1,551908122**
chrXII	456797	456920	123	0,051292091	0,596645002	0,079162146	-1,410729832	-1,199120357	-0,772943385	-0,625943385	**-1,885943385**

Chrom	Start	End	Length	Section A	Section B	A/A+B	Z-score	Z * 0.85	Phase correction	Length correction	ΔLKnuc
chrXII	456803	456959	156	0,085486818	0,103764348	0,451710916	-0,121339881	-0,103138899	-0,074588796	-0,158588796	**-1,418588796**
chrXII	456804	456911	107	0,04844253	0,166764131	0,225097726	-0,755089219	-0,641825836	-0,187188098	0,071811902	**-1,188188098**
chrXII	456804	456920	116	0,042743409	0,218646305	0,163523683	-0,98007851	-0,833066733	-0,235414435	-0,039414435	**-1,299414435**
chrXII	456804	456960	156	0,082637257	0,203822827	0,288477389	-0,557838343	-0,474162592	-0,45160905	-0,53560905	**-1,79560905**
chrXII	456817	456920	103	0,051292091	0,192705218	0,210215804	-0,805672675	-0,684821774	-0,100304825	0,186695175	**-1,073304825**
chrXII	456817	456971	154	0,051292091	0,044470435	0,535617563	0,08939893	0,07598909	-0,084587486	-0,154587486	**-1,414587486**
chrXII	456938	457104	166	0,062690333	0,077823261	0,446151373	-0,135390993	-0,115082344	0,019739117	-0,134260883	**-1,394260883**
chrXII	456984	457118	134	0,09688506	0,074117392	0,566571175	0,16765125	0,142503563	0,143635748	0,213635748	**-1,046364252**
chrXII	456984	457127	143	0,492973981	0,559586306	0,468357002	-0,079400583	-0,067490496	-0,154129479	-0,147129479	**-1,407129479**
chrXII	456984	457130	146	0,051292091	0,077823261	0,397257877	-0,260451199	-0,221383519	-0,353737478	-0,367737478	**-1,627737478**
chrXII	456984	457140	156	0,037044288	0,066705652	0,357053581	-0,366345661	-0,311393812	-0,283988076	-0,367988076	**-1,627988076**
chrXII	456990	457127	137	0,105433742	0,155646522	0,403836506	-0,243429088	-0,206914725	-0,146231234	-0,097231234	**-1,357231234**
chrXII	456990	457140	150	0,552814753	0,429880871	0,562549318	0,157435847	0,13382047	0,036565297	-0,005434703	**-1,265434703**
chrXII	457005	457166	161	0,091185939	0,181587609	0,33429172	-0,428092961	-0,363879017	-0,328329845	-0,447329845	**-1,707329845**
chrXII	457021	457127	106	0,065539893	0,670762394	0,089012209	-1,346862817	-1,144833394	-0,735888188	-0,469888188	**-1,729888188**
chrXII	457022	457205	183	0,071239015	0,255705001	0,217893619	-0,779326791	-0,662427772	-0,561253224	-0,834253224	**-2,094253224**
chrXII	457022	457205	183	0,071239015	0,255705001	0,217893619	-0,779326791	-0,662427772	-0,561253224	-0,834253224	**-2,094253224**
chrXII	457047	457207	160	0,094035499	0,103764348	0,47540734	-0,061683752	-0,052431189	-0,068694378	-0,180694378	**-1,440694378**
chrXII	457047	457207	160	0,094035499	0,103764348	0,47540734	-0,061683752	-0,052431189	-0,068694378	-0,180694378	**-1,440694378**
chrXII	457070	457200	130	0,267858695	0,537351089	0,33265703	-0,432588068	-0,367699858	-0,253691373	-0,155691373	**-1,415691373**
chrXII	457070	457200	130	0,267858695	0,537351089	0,33265703	-0,432588068	-0,367699858	-0,253691373	-0,155691373	**-1,415691373**
chrXII	457083	457217	134	0,051292091	0,085235	0,375691668	-0,31681585	-0,269293473	-0,271019882	-0,201019882	**-1,461019882**
chrXII	457115	457248	133	1,065735658	0,919055655	0,536950989	0,092755225	0,078841941	0,091402548	0,168402548	**-1,091597452**
chrXII	457140	457305	165	0,074088575	0,066705652	0,52621884	0,065768269	0,055903029	0,123287051	-0,023712949	**-1,283712949**
chrXII	457140	457312	172	0,777930039	1,397112831	0,357661934	-0,364715392	-0,310008083	-0,204956141	-0,400956141	**-1,660956141**
chrXII	457143	457248	105	0,039893848	0,100058479	0,285053125	-0,567895024	-0,48271077	0,089865288	0,362865288	**-0,897134712**
chrXII	457143	457279	136	0,558513874	0,433586741	0,562960919	0,15848053	0,134708451	0,097578637	0,153578637	**-1,106421363**
chrXII	457143	457316	173	0,059840772	0,074117392	0,446712395	-0,133971905	-0,113876119	0,034504381	-0,168495619	**-1,428495619**
chrXII	457152	457305	153	0,552814753	0,300175436	0,6480904	0,380170037	0,323144532	0,197570353	0,134570353	**-1,125429647**
chrXII	457152	457312	160	0,059840772	0,070411522	0,459422021	-0,101889932	-0,086606442	-0,099274216	-0,211274216	**-1,471274216**
chrXII	457153	457248	95	0,091185939	0,207528696	0,305261035	-0,509328378	-0,432929121	0,094245116	0,437245116	**-0,822754884**
chrXII	457153	457279	126	0,079787696	0,096352609	0,452978074	-0,118140734	-0,100419624	0,151398994	0,277398994	**-0,982601006**
chrXII	457153	457312	159	0,139628469	0,166764131	0,455717497	-0,111228699	-0,094544395	-0,117818599	-0,222818599	**-1,482818599**
chrXII	457153	457316	163	1,735382395	1,589818049	0,521888056	0,054892775	0,046658858	0,092452593	-0,040547407	**-1,300547407**
chrXII	457153	457317	164	0,074088575	0,166764131	0,307609478	-0,502637789	-0,427242121	-0,458981961	-0,598981961	**-1,858981961**
chrXII	457154	457312	158	0,284956058	0,311293045	0,477914444	-0,055388588	-0,0470803	-0,074625836	-0,172625836	**-1,432625836**
chrXII	457154	457316	162	0,079787696	0,081529131	0,49460244	-0,013530088	-0,011500575	0,015251926	-0,110748074	**-1,370748074**
chrXII	457187	457316	129	0,088336378	0,111176087	0,442761197	-0,143972235	-0,122376399	0,120739353	0,225739353	**-1,034260647**
chrXII	457187	457317	130	0,407487163	0,366881088	0,52621884	0,065768269	0,055903029	0,159829042	0,257829042	**-1,002170958**
chrXII	457198	457346	148	0,421734966	0,255705001	0,622542198	0,312164377	0,26533972	0,130367657	0,102367657	**-1,157632343**
chrXII	457198	457365	167	0,054141651	0,037058696	0,593656197	0,236960278	0,201416237	0,360214571	0,199214571	**-1,060785429**
chrXII	457212	457365	153	0,094035499	0,048176305	0,661235543	0,415837502	0,353461877	0,249050769	0,186050769	**-1,073949231**
chrXII	457214	457363	149	0,262159574	0,103764348	0,716431908	0,572274263	0,486433123	0,320715917	0,285715917	**-0,974284083**
chrXII	457219	457312	93	0,051292091	0,129705435	0,283385589	-0,572813203	-0,486891222	-0,067833668	0,289166332	**-0,970833668**
chrXII	457219	457346	127	0,054141651	0,085235	0,388455674	-0,283346352	-0,2408444	0,001903039	0,120903039	**-1,139096961**
chrXII	457219	457363	144	0,045592969	0,044470435	0,506231912	0,015621722	0,013278464	-0,118376444	-0,118376444	**-1,378376444**
chrXII	457219	457365	146	0,869115978	0,681880002	0,560359916	0,151881795	0,129099526	-0,012451175	-0,026451175	**-1,286451175**
chrXII	457219	457422	203	0,028495606	0,048176305	0,371656395	-0,327469522	-0,278349094	-0,242949793	-0,655949793	**-1,915949793**
chrXII	457227	457316	89	0,045592969	0,037058696	0,551627959	0,129775456	0,110309137	0,533144053	0,918144053	**-0,341855947**
chrXII	457227	457363	136	0,473027057	0,311293045	0,603104595	0,261391241	0,222182555	0,189736683	0,245736683	**-1,014263317**
chrXII	457227	457395	168	0,037044288	0,092646739	0,285634931	-0,566182298	-0,481254953	-0,342082218	-0,510082218	**-1,770082218**
chrXII	457230	457395	165	0,054141651	0,192705218	0,219332946	-0,774448026	-0,658280822	-0,589545303	-0,736545303	**-1,996545303**
chrXII	457230	457401	171	0,247911771	0,233469783	0,515000562	0,037609696	0,031968242	0,180262495	-0,008737505	**-1,268737505**
chrXII	457248	457395	147	0,059840772	0,100058479	0,374240479	-0,320642986	-0,272546538	-0,378630334	-0,399630334	**-1,659630334**
chrXII	457248	457401	153	0,210867483	0,092646739	0,694753219	0,509369056	0,432963697	0,341698489	0,278698489	**-0,981301511**
chrXII	457255	457365	110	0,062690333	0,059293913	0,51392155	0,034903236	0,029667751	0,727221901	0,965221901	**-0,294778099**
chrXII	457255	457407	152	0,512920905	0,26311674	0,660948484	0,415053099	0,352795134	0,213627948	0,157627948	**-1,102372052**
chrXII	457255	457422	167	0,037044288	0,051882174	0,416572153	-0,210670615	-0,179070023	-0,033556303	-0,194556303	**-1,454556303**
chrXII	457266	457365	99	0,088336378	0,096352609	0,478298027	-0,054425638	-0,046261792	0,400334672	0,715334672	**-0,544665328**
chrXII	457266	457407	141	0,116831984	0,062999783	0,649673781	0,384439894	0,326773909	0,222597822	0,243597822	**-1,016402178**
chrXII	457266	457422	156	1,159771157	0,804173699	0,590531427	0,228912214	0,194575382	0,214166428	0,130166428	**-1,129833572**
chrXII	457295	457378	83	0,028495606	0,140823044	0,16829573	-0,960921856	-0,816783577	-0,504960809	-0,077960809	**-1,337960809**
chrXII	457295	457384	89	0,076938136	0,229763914	0,250856282	-0,67179759	-0,571027951	-0,14434915	0,24065085	**-1,01934915**
chrXII	457295	457390	95	0,062690333	0,359469349	0,1484991	-1,042892249	-0,886458411	-0,376055282	-0,033055282	**-1,293055282**
chrXII	457295	457391	96	0,091185939	0,889408699	0,092990452	-1,322562523	-1,124178145	-0,555094384	-0,219094384	**-1,479094384**
chrXII	457295	457393	98	0,037044288	0,344645871	0,097053295	-1,298526168	-1,103747243	-0,560740471	-0,238740471	**-1,498740471**
chrXII	457295	457394	99	0,031345166	0,381704567	0,075887148	-1,433292386	-1,218298528	-0,760260463	-0,445260463	**-1,705260463**
chrXII	457295	457395	100	14,8490602	81,00289725	0,154916609	-1,015572055	-0,863236247	-0,409307624	-0,101307624	**-1,361307624**
chrXII	457295	457397	102	0,037044288	0,481763045	0,071402783	-1,465422925	-1,245609486	-0,733109196	-0,439109196	**-1,699109196**
chrXII	457295	457401	106	0,136778908	0,318704784	0,300293755	-0,523555831	-0,445022456	-0,046004191	0,219995809	**-1,040004191**
chrXII	457295	457402	107	0,133929347	0,296469566	0,31117492	-0,492522755	-0,418644342	0,009189281	0,268189281	**-0,991810719**
chrXII	457337	457476	139	0,071239015	0,085235	0,455276965	-0,11233987	-0,09548889	-0,150582036	-0,115582036	**-1,375582036**
chrXII	457337	457481	144	0,649699813	0,277940218	0,700379232	0,525491536	0,446667806	0,300064179	0,300064179	**-0,959935821**
chrXII	457360	457476	116	0,045592969	0,17788174	0,204018475	-0,827353109	-0,703250143	-0,110417981	0,085582019	**-1,174417981**
chrXII	457360	457481	121	0,042743409	0,037058696	0,535617563	0,08939893	0,07598909	0,459366615	0,620366615	**-0,639633385**
chrXII	457360	457530	170	0,165274514	0,237175653	0,410670755	-0,225819886	-0,191946903	-0,019578819	-0,201578819	**-1,461578819**
chrXII	457370	457476	106	0,792177842	4,684219147	0,144653107	-1,05964482	-0,900698097	-0,502843022	-0,236843022	**-1,496843022**
chrXII	457372	457476	104	0,04844253	0,896820438	0,051247676	-1,632874705	-1,387943499	-0,8246605	-0,5446605	**-1,8046605**
chrXII	457420	457563	143	0,105433742	0,092646739	0,53227729	0,080995639	0,068846293	-0,025611526	-0,018611526	**-1,278611526**
chrXII	457420	457567	147	0,076938136	0,059293913	0,564757972	0,163043664	0,138587115	0,03266304	0,01166304	**-1,24833696**
chrXII	457420	457571	151	0,085486818	0,051882174	0,622315244	0,311567137	0,264832067	0,13385782	0,08485782	**-1,17514218**
chrXII	457420	457572	152	0,512920905	0,17417587	0,746504602	0,663530589	0,564001001	0,419854182	0,363854182	**-0,896145818**
chrXII	457437	457592	155	0,045592969	0,037058696	0,551627959	0,129775456	0,110309137	-0,012422117	-0,089422117	**-1,349422117**
chrXII	457440	457593	153	0,045592969	0,044470435	0,506231912	0,015621722	0,013278464	-0,082659838	-0,145659838	**-1,405659838**
chrXII	457540	457654	114	0,04844253	0,415057393	0,104514645	-1,256239112	-1,067803245	-0,459183439	-0,249183439	**-1,509183439**
chrXII	457553	457706	153	0,190920559	0,159352392	0,54506224	0,113195553	0,09621622	-0,005591869	-0,068591869	**-1,328591869**
chrXII	457569	457736	167	0,054141651	0,103764348	0,342872667	-0,404635672	-0,343940321	-0,198046263	-0,359046263	**-1,619046263**
chrXII	457569	457741	172	0,336248149	0,559586306	0,375346301	-0,317726245	-0,270067308	-0,14254435	-0,33854435	**-1,59854435**
chrXII	457600	457736	136	0,769381358	0,755997394	0,504387095	0,010997038	0,009347483	-0,025052112	0,030947888	**-1,229052112**
chrXII	457600	457739	139	0,142478029	0,037058696	0,793587102	0,818930974	0,696091328	0,652400744	0,687400744	**-0,572599256**
chrXII	457600	457741	141	0,076938136	0,125999566	0,379121943	-0,307787693	-0,261619539	-0,357682821	-0,336682821	**-1,596682821**
chrXII	457606	457763	157	0,122531105	0,044470435	0,733712426	0,624079848	0,53046787	0,427229536	0,336229536	**-0,923770464**
chrXII	457620	457773	153	0,507221784	0,470645436	0,518702103	0,046896405	0,039861944	-0,061555884	-0,124555884	**-1,384555884**
chrXII	457620	457787	167	0,079787696	0,129705435	0,380860679	-0,303221116	-0,257737948	-0,104940035	-0,265940035	**-1,525940035**
chrXII	457622	457771	149	0,054141651	0,059293913	0,477289917	-0,056956516	-0,048413039	-0,226147421	-0,261147421	**-1,521147421**
chrXII	457634	457757	123	0,159575393	0,43729261	0,267354577	-0,620833537	-0,527708506	-0,090859799	0,056140201	**-1,203859799**

Chrom	Start	End	Length	Section A	Section B	A/A+B	Z-score	Z * 0.85	Phase correction	Length correction	ΔLKnuc
chrXII	457650	457805	155	0,039893848	0,074117392	0,349911537	-0,385559308	-0,327725412	-0,449824643	-0,526824643	**-1,786824643**
chrXII	457667	457819	152	0,045592969	0,051882174	0,467739443	-0,080953558	-0,068810524	-0,225599981	-0,281599981	**-1,541599981**
chrXII	457803	457959	156	0,239363089	0,159352392	0,600335579	0,254215803	0,216083433	0,227279109	0,143279109	**-1,116720891**
chrXII	457816	457974	158	0,09973462	0,059293913	0,627149217	0,324312357	0,275665503	0,237309414	0,139309414	**-1,120690586**
chrXII	457820	457953	133	0,116831984	0,070411522	0,623957468	0,315891237	0,268507552	0,276763448	0,353763448	**-0,906236552**
chrXII	457872	458023	151	0,276407377	0,214940436	0,562549318	0,157435847	0,13382047	0,000251234	-0,048748766	**-1,308748766**
chrXII	457945	458104	159	0,350495952	0,385410436	0,476277904	-0,059497562	-0,050572928	-0,07431108	-0,17931108	**-1,43931108**
chrXII	457949	458059	110	0,04844253	0,103764348	0,31826768	-0,472548462	-0,401666193	0,281930632	0,519930632	**-0,740069368**
chrXII	457949	458101	152	0,34194727	0,207528696	0,622315244	0,311567137	0,264832067	0,105765228	0,049765228	**-1,210234772**
chrXII	457950	458101	151	0,159575393	0,08894087	0,642112476	0,3641111	0,309494435	0,170696655	0,121696655	**-1,138303345**
chrXII	457950	458103	153	0,527168708	0,370586958	0,58720733	0,220367006	0,187311955	0,099732668	0,036732668	**-1,223267332**
chrXII	457959	458110	151	0,151026711	0,092646739	0,619791408	0,304932997	0,259193047	0,12199394	0,07299394	**-1,18700606**
chrXII	457976	458059	83	0,059840772	0,081529131	0,423292165	-0,193478389	-0,164456631	0,141697137	0,568697137	**-0,691302863**
chrXII	457976	458119	143	0,139628469	0,074117392	0,653245253	0,394096909	0,334982372	0,236524301	0,243524301	**-1,016475699**
chrXII	457983	458089	106	0,062690333	0,062999783	0,498768996	-0,003085674	-0,002622823	0,417907765	0,683907765	**-0,576092235**
chrXII	457983	458101	118	0,031345166	0,040764565	0,434687048	-0,164453569	-0,139785534	0,299375305	0,481375305	**-0,778624695**
chrXII	457983	458103	120	0,039893848	0,070411522	0,361667325	-0,354005648	-0,300904801	0,089769079	0,257769079	**-1,002230921**
chrXII	457983	458137	154	0,743735312	0,678174133	0,523053922	0,057819814	0,049146842	-0,121691684	-0,191691684	**-1,451691684**
chrXII	457986	458117	131	0,091185939	0,040764565	0,691061693	0,498861988	0,42403269	0,475856577	0,566856577	**-0,693143423**
chrXII	458025	458167	142	0,094035499	0,111176087	0,458236793	-0,104876779	-0,089145262	-0,082913323	-0,068913323	**-1,328913323**
chrXII	458055	458137	82	0,056991212	0,048176305	0,541908887	0,105243965	0,08945737		0,434	**-0,826**
chrXII	458055	458206	151	0,931806311	0,52623348	0,639081537	0,35600486	0,302604131	0,164892351	0,115892351	**-1,144107649**
chrXII	458079	458227	148	0,079787696	0,048176305	0,623516738	0,314730183	0,267520656	0,128403373	0,100403373	**-1,159596627**
chrXII	458087	458231	144	0,350495952	0,181587609	0,658723511	0,408981855	0,347634577	0,205192381	0,205192381	**-1,054807619**
chrXII	458127	458277	150	0,079787696	0,085235	0,483495289	-0,041382985	-0,035175537	-0,135385509	-0,177385509	**-1,437385509**
chrXII	458130	458293	163	0,139628469	0,118587827	0,540742282	0,102303929	0,08695834	0,133112844	0,000112844	**-1,259887156**
chrXII	458134	458272	138	0,142478029	0,08894087	0,615671537	0,294132147	0,250012325	0,253683895	0,295683895	**-0,964316105**
chrXII	458134	458277	143	0,336248149	0,233469783	0,590201097	0,228062304	0,193852958	0,086485005	0,093485005	**-1,166514995**
chrXII	458136	458295	159	0,094035499	0,081529131	0,535617563	0,08939893	0,07598909	0,064857272	-0,040142728	**-1,300142728**
chrXII	458143	458291	148	0,168124074	0,092646739	0,644719675	0,371103224	0,315437741	0,169967647	0,141967647	**-1,118032353**
chrXII	458166	458321	155	0,481575739	0,507704132	0,486794236	-0,03310799	-0,028141791	-0,139247261	-0,216247261	**-1,476247261**
chrXII	458166	458321	155	0,481575739	0,507704132	0,486794236	-0,03310799	-0,028141791	-0,139247261	-0,216247261	**-1,476247261**
chrXII	458166	458322	156	0,056991212	0,048176305	0,541908887	0,105243965	0,08945737	0,079166027	-0,004833973	**-1,264833973**
chrXII	458166	458322	156	0,056991212	0,048176305	0,541908887	0,105243965	0,08945737	0,079166027	-0,004833973	**-1,264833973**
chrXII	458167	458322	155	0,170973635	0,103764348	0,622315244	0,311567137	0,264832067	0,156000788	0,079000788	**-1,180999212**
chrXII	458167	458322	155	0,170973635	0,103764348	0,622315244	0,311567137	0,264832067	0,156000788	0,079000788	**-1,180999212**
chrXII	458177	458322	145	0,823523009	0,541056958	0,603499266	0,262415054	0,223052796	0,039020365	0,032020365	**-1,227979635**
chrXII	458177	458327	150	0,037044288	0,044470435	0,454449042	-0,114428548	-0,097264266	-0,209203035	-0,251203035	**-1,511203035**
chrXII	458177	458350	173	0,555664314	0,78935022	0,413128855	-0,219503686	-0,186578133	-0,02623011	-0,22923011	**-1,48923011**
chrXII	458179	458327	148	0,304902982	0,118587827	0,719975442	0,582768555	0,495353272	0,358663308	0,330663308	**-0,929336692**
chrXII	458191	458321	130	0,054141651	0,048176305	0,529151025	0,07313593	0,062165541	0,173347052	0,271347052	**-0,988652948**
chrXII	458191	458352	161	0,626903328	0,444704349	0,585011979	0,214732295	0,182522451	0,216507294	0,097507294	**-1,162492706**
chrXII	458195	458322	127	0,062690333	0,103764348	0,376621026	-0,314367347	-0,267212245	-0,033457517	0,085542483	**-1,174457517**
chrXII	458195	458327	132	0,045592969	0,051882174	0,467739443	-0,080953558	-0,068810524	0,049619481	0,133619481	**-1,126380519**
chrXII	458195	458350	155	0,891912463	0,492880654	0,644076326	0,369376183	0,313969755	0,211489392	0,134489392	**-1,125510608**
chrXII	458204	458350	146	0,062690333	0,066705652	0,484484373	-0,038901718	-0,03306646	-0,171133047	-0,185133047	**-1,445133047**
chrXII	458204	458380	176	0,199469241	0,281646088	0,414597559	-0,215733974	-0,183373878	0,006105358	-0,217894642	**-1,477894642**
chrXII	458291	458451	160	0,433133209	0,255705001	0,628788012	0,328645073	0,279348312	0,267588812	0,155588812	**-1,104411188**
chrXII	458297	458451	154	1,27090402	0,733762177	0,633972889	0,342394241	0,291035105	0,116915377	0,046915377	**-1,213084623**
chrXII	458298	458451	153	0,059840772	0,040764565	0,594807131	0,239928434	0,203939169	0,116906473	0,053906473	**-1,206093527**
chrXII	458319	458472	153	0,208017923	0,107470218	0,65935259	0,410696874	0,349092343	0,240116559	0,177116559	**-1,082883441**
chrXII	458338	458492	154	0,276407377	0,166764131	0,62370295	0,315220686	0,267937583	0,089711611	0,019711611	**-1,240288389**
chrXII	458356	458493	137	0,051292091	0,051882174	0,497140355	-0,007168129	-0,00609291	0,05135137	0,10035137	**-1,15964863**
chrXII	458356	458508	152	0,29065518	0,148234783	0,662250688	0,418613496	0,355821471	0,202040626	0,146040626	**-1,113959374**
chrXII	458359	458523	164	0,071239015	0,092646739	0,434687048	-0,164453569	-0,139785534	-0,159855743	-0,299855743	**-1,559855743**
chrXII	458365	458493	128	0,720938828	1,074702178	0,401493854	-0,249482329	-0,21205998	0,020852559	0,132852559	**-1,127147441**
chrXII	458373	458493	120	0,042743409	0,059293913	0,418899751	-0,204708983	-0,174002636	0,220005997	0,388005997	**-0,871994003**
chrXII	458373	458530	157	0,219416165	0,17788174	0,552271135	0,131401466	0,111691246	0,014875251	-0,076124749	**-1,336124749**
chrXII	458374	458532	158	0,051292091	0,062999783	0,448781606	-0,128740216	-0,109429183	-0,13867944	-0,23667944	**-1,49667944**
chrXII	458395	458501	106	0,051292091	0,055588044	0,479902939	-0,050397187	-0,042837609	0,380376981	0,646376981	**-0,613623019**
chrXII	458395	458519	124	0,04844253	0,074117392	0,395255882	-0,265646117	-0,2257992	0,197359159	0,337359159	**-0,922640841**
chrXII	458419	458587	168	0,074088575	0,100058479	0,425436857	-0,188003748	-0,159803185	-0,018254399	-0,186254399	**-1,446254399**
chrXII	458508	458658	150	0,062690333	0,059293913	0,51392155	0,034903236	0,029667751	-0,091642725	-0,133642725	**-1,393642725**
chrXII	458508	458658	150	0,062690333	0,059293913	0,51392155	0,034903236	0,029667751	-0,091642725	-0,133642725	**-1,393642725**
chrXII	458508	458659	151	0,116831984	0,081529131	0,588986325	0,224938202	0,191197471	0,058288264	0,009288264	**-1,250711736**
chrXII	458508	458659	151	0,116831984	0,081529131	0,588986325	0,224938202	0,191197471	0,058288264	0,009288264	**-1,250711736**
chrXII	458746	458901	155	0,09688506	0,081529131	0,543034495	0,108081541	0,09186931	-0,019935675	-0,096935675	**-1,356935675**
chrXII	458746	458912	166	0,239363089	0,200116957	0,544650641	0,112157259	0,09533367	0,243211807	0,089211807	**-1,170788193**
chrXII	458750	458912	162	0,037044288	0,055588044	0,399906675	-0,253588671	-0,21555037	-0,188860532	-0,314860532	**-1,574860532**
chrXII	458768	458900	132	0,14817715	0,129705435	0,533236547	0,083408278	0,070897036	0,192103715	0,276103715	**-0,983896285**
chrXII	458768	458901	133	0,182371877	0,133411305	0,577522451	0,195559327	0,166225428	0,173072164	0,250072164	**-1,009927836**
chrXII	458768	458912	144	0,712390146	0,466939567	0,604063595	0,263879451	0,224297533	0,09403121	0,09403121	**-1,16596879**
chrXII	458769	458900	131	0,626903328	0,689291742	0,476299709	-0,059442808	-0,050526387	-0,016463585	0,074536415	**-1,185463585**
chrXII	458769	458901	132	1,587205245	1,426759788	0,526617007	0,066768519	0,056753241	0,185562068	0,269562068	**-0,990437932**
chrXII	458769	458912	143	0,803576085	0,61517435	0,566397067	0,167208665	0,142127365	0,027433769	0,034433769	**-1,225566231**
chrXII	458769	458913	144	0,233663968	0,155646522	0,600199516	0,25386356	0,215784026	0,092090529	0,092090529	**-1,167909471**
chrXII	458770	458900	130	0,082637257	0,163058261	0,336340107	-0,422472439	-0,359101573	-0,273229287	-0,175229287	**-1,435229287**
chrXII	458770	458901	131	0,740885752	1,156231309	0,390532439	-0,277931585	-0,236241848	-0,210998286	-0,119998286	**-1,379998286**
chrXII	458770	458912	142	0,14817715	0,122293696	0,54784888	0,120228376	0,10219412	0,096050443	0,110050443	**-1,149949557**
chrXII	458846	459002	156	0,176672756	0,051882174	0,772999103	0,748760132	0,636446112	0,636984729	0,552984729	**-0,707015271**
chrXII	458846	459002	156	0,176672756	0,051882174	0,772999103	0,748760132	0,636446112	0,636984729	0,552984729	**-0,707015271**
chrXII	458902	459048	146	0,094035499	0,085235	0,524545307	0,06156483	0,052330105	-0,086067074	-0,100067074	**-1,360067074**
chrXII	458908	459044	136	0,142478029	0,085235	0,625691158	0,320462592	0,272393203	0,239976702	0,295976702	**-0,964023298**
chrXII	458908	459048	140	0,136778908	0,140823044	0,492715945	-0,018259434	-0,015520519	0,010627022	0,038627022	**-1,221372978**
chrXII	458910	459043	133	0,039893848	0,040764565	0,49460244	-0,013530088	-0,011500575	-0,014184511	0,062815489	**-1,197184511**
chrXII	458910	459066	156	0,074088575	0,107470218	0,408069331	-0,232514197	-0,197637067	-0,213282324	-0,297282324	**-1,557282324**
chrXII	458913	459002	89	0,085486818	0,159352392	0,349154932	-0,387602979	-0,329462532	0,113233966	0,498233966	**-0,761766034**
chrXII	458913	459038	125	1,117027749	1,901111094	0,370104825	-0,331575727	-0,281839368	0,036050174	0,169050174	**-1,090949826**
chrXII	458913	459043	130	0,803576085	1,026525873	0,439088151	-0,153281484	-0,130289261	-0,029761209	0,068238791	**-1,191761209**
chrXII	458913	459044	131	3,701579198	4,947335887	0,42798191	-0,181514414	-0,154287252	-0,111686726	-0,020686726	**-1,280686726**
chrXII	458913	459047	134	1,749630198	1,852934789	0,485662356	-0,035946885	-0,030554852	-0,033438986	0,036561014	**-1,223438986**
chrXII	458913	459048	135	0,19661968	0,192705218	0,505027244	0,012601764	0,0107115	0,01129021	0,07429021	**-1,18570979**
chrXII	458913	459066	153	0,159575393	0,129705435	0,551627959	0,129775456	0,110309137	0,001993428	-0,061006572	**-1,321006572**
chrXII	458932	459038	106	0,039893848	0,044470435	0,472876041	-0,06804215	-0,057835827	0,370968565	0,636968565	**-0,623031435**
chrXII	458933	459044	111	0,136778908	0,218646305	0,384831753	-0,292815072	-0,248892811	0,25839996	0,48939996	**-0,77060004**
chrXII	458933	459047	114	0,059840772	0,092646739	0,392430643	-0,272989502	-0,232041076	0,379349407	0,589349407	**-0,670650593**

Chrom	Start	End	Length	Section A	Section B	A/A+B	Z-score	Z * 0.85	Phase correction	Length correction	ΔLKnuc
chrXII	458933	459066	133	0,330549028	0,26311674	0,556793141	0,142843581	0,121417044	0,115936515	0,192936515	-1,067063485
chrXII	458957	459044	87	0,09688506	0,159352392	0,378106554	-0,310457456	-0,263888837	0,124882335	0,523882335	-0,736117665
chrXII	459050	459146	96	0,182371877	1,030231743	0,150396943	-1,034732432	-0,879522567	-0,305102172	0,030897828	-1,229102172
chrXII	459089	459262	173	0,313451664	0,544762828	0,365236974	-0,344495143	-0,292820872	-0,123410505	-0,326410505	-1,586410505
chrXII	459089	459265	176	0,042743409	0,155646522	0,215451503	-0,787647358	-0,669500254	-0,494183737	-0,718183737	-1,978183737
chrXII	459095	459262	167	0,068389454	0,207528696	0,247861382	-0,681235065	-0,579049806	-0,424112943	-0,585112943	-1,845112943
chrXII	459095	459265	170	0,678195419	1,1451137	0,371958552	-0,326670515	-0,277669938	-0,111855544	-0,293855544	-1,553855544
chrXII	459098	459233	135	1,880709985	1,504583049	0,555553084	0,139704043	0,118748436	0,122672455	0,185672455	-1,074327545
chrXII	459098	459262	164	0,168124074	0,251999131	0,400178024	-0,252886336	-0,214953386	-0,237625524	-0,377625524	-1,637625524
chrXII	459166	459328	162	0,068389454	0,062999783	0,520510322	0,051434423	0,043719259	0,07276317	-0,05323683	-1,31323683
chrXII	459185	459328	143	0,834921251	0,52993935	0,611726392	0,28382145	0,241248232	0,126029327	0,133029327	-1,126970673
chrXII	459204	459353	149	0,886213341	0,422469132	0,677179804	0,459827015	0,390852962	0,211245938	0,176245938	-1,083754062
chrXII	459204	459417	213	0,042743409	0,074117392	0,365763444	-0,34309514	-0,291630869	-0,051668548	-0,534668548	-1,794668548
chrXII	459204	459419	215	0,076938136	0,085235	0,474419732	-0,064164224	-0,05453959	0,248559708	-0,248440292	-1,508440292
chrXII	459227	459353	126	0,045592969	0,066705652	0,405997586	-0,23785292	-0,202174982	0,050053594	0,176053594	-1,083946406
chrXII	459227	459376	149	0,775080479	0,574409785	0,574350553	0,187461389	0,15934218	-0,032600072	-0,067600072	-1,327600072
chrXII	459227	459419	192	0,065539893	0,085235	0,434687048	-0,164453569	-0,139785534	-0,083369252	-0,419369252	-1,679369252
chrXII	459227	459432	205	0,031345166	0,055588044	0,360566076	-0,356946107	-0,303404191	-0,239623666	-0,666623666	-1,926623666
chrXII	459235	459395	160	0,14532759	0,070411522	0,673626532	0,449949388	0,38245698	0,3644137	0,2524137	-1,0075863
chrXII	459236	459328	92	0,028495606	0,051882174	0,354520937	-0,373143192	-0,317171714	0,07019594	0,43419594	-0,82580406
chrXII	459236	459393	157	0,501522663	0,470645436	0,5158806	0,039817281	0,033844689	-0,057383138	-0,148383138	-1,408383138
chrXII	459236	459417	181	0,105433742	0,08894087	0,542425478	0,106546147	0,090564225	0,279099634	0,020099634	-1,239900366
chrXII	459236	459419	183	0,19377012	0,259410871	0,427577775	-0,182544352	-0,155162699	-0,050105474	-0,323105474	-1,583105474
chrXII	459264	459353	89	0,082637257	0,085235	0,492262738	-0,019395657	-0,016486308	0,421551971	0,806551971	-0,453448029
chrXII	459264	459376	112	0,113982423	0,226058044	0,335202525	-0,425592166	-0,361753341	0,100840471	0,324840471	-0,935159529
chrXII	459264	459417	153	2,097276589	1,567582832	0,572266586	0,182147684	0,154825532	0,056156998	-0,006843002	-1,266843002
chrXII	459264	459419	155	2,00039153	1,567582832	0,560651879	0,152622168	0,129728843	0,03403389	-0,04296611	-1,30296611
chrXII	459264	459432	168	0,988797522	0,856055873	0,535976206	0,090301549	0,076756317	0,205214496	0,037214496	-1,222785504
chrXII	459264	459441	177	0,73233707	1,019114134	0,418131586	-0,206675654	-0,175674306	-0,018443008	-0,249443008	-1,509443008
chrXII	459265	459376	111	0,045592969	0,059293913	0,434687048	-0,164453569	-0,139785534	0,36628747	0,59728747	-0,66271253
chrXII	459265	459393	128	0,188070999	0,233469783	0,446151373	-0,135390993	-0,115082344	0,110401581	0,222401581	-1,037598419
chrXII	459265	459416	151	0,054141651	0,044470435	0,549036667	0,123227859	0,104743681	-0,02405579	-0,07305579	-1,33305579
chrXII	459265	459417	152	1,664143381	1,182172396	0,584665761	0,213844301	0,181767656	0,019242725	-0,036757275	-1,296757275
chrXII	459265	459419	154	11,28710947	6,959623069	0,618582502	0,301760039	0,256496033	0,070696014	0,000696014	-1,259303986
chrXII	459265	459422	157	0,236513528	0,226058044	0,511301477	0,028332392	0,024082533	-0,062848697	-0,153848697	-1,413848697
chrXII	459265	459432	167	0,151026711	0,248293262	0,37820976	-0,310185996	-0,263658096	-0,115426888	-0,276426888	-1,536426888
chrXII	459265	459441	176	0,039893848	0,140823044	0,220753288	-0,769651619	-0,654203876	-0,479536502	-0,703536502	-1,963536502
chrXII	459265	459467	202	0,128230226	0,096352609	0,570970734	0,178846117	0,1520192	0,166797533	-0,239202467	-1,499202467
chrXII	459265	459469	204	0,162424953	0,170470001	0,487916538	-0,030293381	-0,025749374	0,034716958	-0,385283042	-1,645283042
chrXII	459267	459393	126	0,042743409	0,077823261	0,354520937	-0,373143192	-0,317171714	-0,082318047	0,043681953	-1,216318047
chrXII	459267	459417	150	0,53571739	0,400233914	0,572377417	0,18243015	0,155065628	0,028609461	-0,013390539	-1,273390539
chrXII	459267	459419	152	2,789719811	1,908522833	0,593779424	0,237277969	0,201686274	0,032628061	-0,023371939	-1,283371939
chrXII	459267	459422	155	0,681044979	0,363175219	0,652204373	0,391278682	0,33258688	0,238652723	0,161652723	-1,098347277
chrXII	459267	459432	165	0,153876272	0,200116957	0,434687048	-0,164453569	-0,139785534	-0,048804089	-0,195804089	-1,455804089
chrXII	459267	459441	174	0,065539893	0,051882174	0,558156527	0,146296985	0,124352437	0,344176444	0,134176444	-1,125823556
chrXII	459267	459469	202	0,04844253	0,070411522	0,407579962	-0,23377466	-0,198708461	-0,181406659	-0,587406659	-1,847406659
chrXII	459268	459374	106	0,04844253	0,248293262	0,163251388	-0,981182451	-0,834005084	-0,411548963	-0,145548963	-1,405548963
chrXII	459268	459376	108	0,042743409	0,185293479	0,187440765	-0,887366654	-0,754261656	-0,075596376	0,176403624	-1,083596376
chrXII	459268	459393	125	0,24221265	0,200116957	0,547584077	0,119559826	0,101625852	0,428546269	0,561546269	-0,698453731
chrXII	459268	459416	148	0,054141651	0,055588044	0,493409293	-0,016521205	-0,014043024	-0,153000592	-0,181000592	-1,441000592
chrXII	459268	459417	149	2,179913846	1,256289787	0,634396002	0,343519071	0,29199121	0,103573735	0,068573735	-1,191426265
chrXII	459268	459419	151	11,13893232	6,099861326	0,646154977	0,374960229	0,318716195	0,193474119	0,144474119	-1,115525881
chrXII	459268	459422	154	0,310602104	0,214940436	0,591012298	0,230149754	0,195627291	0,007455482	-0,062544518	-1,322544518
chrXII	459268	459431	163	0,088336378	0,103764348	0,459844061	-0,100826586	-0,085702598	-0,045496428	-0,178496428	-1,438496428
chrXII	459268	459432	164	1,635647775	1,363760005	0,545323576	0,11385486	0,096776631	0,080652704	-0,059347296	-1,319347296
chrXII	459268	459441	173	0,059840772	0,196411088	0,23352327	-0,727292911	-0,618198974	-0,437103265	-0,640103265	-1,900103265
chrXII	459268	459467	199	0,156725832	0,100058479	0,610340373	0,280206274	0,238175333	0,268028998	-0,116971002	-1,376971002
chrXII	459268	459469	201	0,188070999	0,166764131	0,530023616	0,075329227	0,064029843	0,090624871	-0,308375129	-1,568375129
chrXII	459269	459376	107	0,068389454	0,26682261	0,204018475	-0,827353109	-0,703250143	-0,287326132	-0,028326132	-1,288326132
chrXII	459269	459393	124	0,113982423	0,300175436	0,275214923	-0,597116135	-0,507548715	-0,095014891	0,044985109	-1,215014891
chrXII	459269	459413	144	0,065539893	0,048176305	0,576346155	0,192554744	0,163671533	0,045439062	0,045439062	-1,214560938
chrXII	459269	459417	148	4,963934536	2,542226531	0,661314684	0,416053806	0,353645735	0,214404809	0,186404809	-1,073595191
chrXII	459269	459419	150	3,388127534	2,190168921	0,607376743	0,272488395	0,231615136	0,115909088	0,073909088	-1,186090912
chrXII	459269	459422	153	0,108283302	0,144528914	0,42831515	-0,180665296	-0,153565501	-0,263776623	-0,326776623	-1,586776623
chrXII	459269	459432	163	0,028495606	0,08894087	0,242646977	-0,69781331	-0,593141313	-0,549235017	-0,682235017	-1,942235017
chrXII	459269	459467	198	0,102584181	0,085235	0,54618586	0,116030612	0,09862602	0,146674733	-0,231325267	-1,491325267
chrXII	459269	459469	200	0,136778908	0,133411305	0,506231912	0,015621722	0,013278464	0,034276504	-0,357723496	-1,617723496
chrXII	459271	459419	148	0,045592969	0,040764565	0,527955893	0,070132481	0,059612609	-0,080597729	-0,108597729	-1,368597729
chrXII	459308	459414	106	0,182371877	0,285351958	0,389913585	-0,279544268	-0,237612628	0,179479867	0,445479867	-0,814520133
chrXII	459308	459419	111	0,24506221	0,329822392	0,426280699	-0,185851269	-0,157973579	0,329440195	0,560440195	-0,699559805
chrXII	459308	459467	159	0,088336378	0,077823261	0,531635591	0,07938196	0,067474666	0,046243575	-0,058756425	-1,318756425
chrXII	459308	459469	161	0,988797522	0,581821524	0,629559106	0,330685854	0,281082976	0,315178619	0,196178619	-1,063821381
chrXII	459325	459466	141	0,222265726	0,08894087	0,714206347	0,565715338	0,480858037	0,384192582	0,405192582	-0,854807418
chrXII	459326	459417	91	0,102584181	0,185293479	0,356346446	-0,368241871	-0,31300559	0,061535577	0,432535577	-0,827464423
chrXII	459326	459419	93	0,401788042	0,389116306	0,508010916	0,020081738	0,017069477	0,452410134	0,809410134	-0,450589866
chrXII	459326	459467	141	1,373488201	0,659644785	0,675552564	0,455297989	0,387003291	0,295791692	0,316791692	-0,943208308
chrXII	459326	459469	143	0,19661968	0,107470218	0,646584058	0,376114353	0,3196972	0,18507962	0,19207962	-1,06792038
chrXII	459408	459559	151	0,230814407	0,092646739	0,713576915	0,563864767	0,479285052	0,342247805	0,293247805	-0,966752195
chrXII	459429	459558	129	1,131275552	1,58611218	0,41630995	-0,211342655	-0,179641257	0,088905802	0,193905802	-1,066094198
chrXII	459429	459559	130	0,105433742	0,140823044	0,428145528	-0,181097487	-0,153932864	-0,039653976	0,058346024	-1,201653976
chrXII	459429	459579	150	0,076938136	0,08894087	0,463820816	-0,090812431	-0,077190567	-0,202459762	-0,244459762	-1,504459762
chrXII	459430	459558	128	0,162424953	0,233469783	0,410273081	-0,226842569	-0,192816183	0,028038129	0,140038129	-1,119961871
chrXII	459430	459559	129	0,04844253	0,037058696	0,566571175	0,16765125	0,142503563	0,416198501	0,521198501	-0,738801499
chrXII	459430	459573	143	0,361894194	0,218646305	0,623374587	0,314355796	0,267202426	0,120743519	0,127743519	-1,132256481
chrXII	459430	459579	149	0,644000692	0,43729261	0,595583724	0,24193239	0,205642532	0,015042638	-0,019957362	-1,279957362
chrXII	459519	459673	154	0,199469241	0,133411305	0,599221683	0,251333038	0,213633083	0,032105848	-0,037894152	-1,297894152
chrXII	459521	459673	152	0,19377012	0,125999566	0,605967759	0,268824834	0,228501109	0,051836236	-0,004163764	-1,264163764
chrXII	459602	459756	154	0,056991212	0,037058696	0,605967759	0,268824834	0,228501109	0,049896685	-0,020103315	-1,280103315
chrXII	459602	459759	157	0,076938136	0,051882174	0,597251597	0,246239543	0,209303612	0,115316486	0,024316486	-1,235683514
chrXII	459604	459722	118	0,054141651	0,111176087	0,327500555	-0,446825428	-0,379801614	0,034575182	0,216575182	-1,043424818
chrXII	459604	459756	152	5,852997439	3,461282186	0,628389706	0,32759145	0,278452733	0,091988041	0,035988041	-1,224011959
chrXII	459604	459759	155	0,088336378	0,037058696	0,704464501	0,537284661	0,456691962	0,3653848	0,2883848	-0,9716152
chrXII	459604	459774	170	0,29350474	0,35576348	0,452054684	-0,120471861	-0,102401082	0,060746635	-0,121253365	-1,381253365
chrXII	459604	459796	192	0,615505086	0,659644785	0,482692349	-0,043397467	-0,036887847	0,027819097	-0,308180903	-1,568180903
chrXII	459604	459798	194	0,139628469	0,214940436	0,39379749	-0,269434058	-0,22901895	-0,15635304	-0,50635304	-1,76635304
chrXII	459604	459804	200	0,319150785	0,200116957	0,614617006	0,291373097	0,247667133	0,266221122	-0,125778878	-1,385778878

Chrom	Start	End	Length	Section A	Section B	A/A+B	Z-score	Z * 0.85	Phase correction	Length correction	ΔLKnuc
chrXII	459604	459806	202	0,168124074	0,274234349	0,380063011	-0,305315302	-0,259518007	-0,239480729	-0,645480729	**-1,905480729**
chrXII	459618	459756	138	0,162424953	0,144528914	0,529151025	0,07313593	0,062165541	0,065556212	0,107556212	**-1,152443788**
chrXII	459618	459774	156	0,227964847	0,144528914	0,611996417	0,284526191	0,241847262	0,231376996	0,147376996	**-1,112623004**
chrXII	459618	459796	178	0,09688506	0,081529131	0,543034495	0,108081541	0,09186931	0,230896603	-0,007103397	**-1,267103397**
chrXII	459621	459756	135	0,062690333	0,066705652	0,484484373	-0,038901718	-0,03306646	-0,031611434	0,031388566	**-1,228611434**
chrXII	459621	459774	153	0,04844253	0,040764565	0,543034495	0,108081541	0,09186931	-0,023246386	-0,086246386	**-1,346246386**
chrXII	459625	459756	131	0,074088575	0,155646522	0,322495674	-0,460731366	-0,391621661	-0,352083941	-0,261083941	**-1,521083941**
chrXII	459625	459774	149	0,042743409	0,040764565	0,51184823	0,029703476	0,025247955	-0,169308181	-0,204308181	**-1,464308181**
chrXII	459625	459796	171	0,116831984	0,214940436	0,352144956	-0,379535953	-0,32260556	-0,183843104	-0,372843104	**-1,632843104**
chrXII	459625	459798	173	0,675345858	0,759703264	0,470608182	-0,073741139	-0,062679968	0,112291993	-0,090708007	**-1,350708007**
chrXII	459625	459804	179	0,062690333	0,08894087	0,413439528	-0,218706025	-0,185900121	-0,037441342	-0,282441342	**-1,542441342**
chrXII	459625	459806	181	0,056991212	0,125999566	0,311443082	-0,491764035	-0,41799943	-0,225677107	-0,484677107	**-1,744677107**
chrXII	459628	459756	128	0,339097709	0,344645871	0,495942806	-0,010170054	-0,008644546	0,225212864	0,337212864	**-0,922787136**
chrXII	459628	459774	146	0,176672756	0,188999349	0,483145293	-0,042261061	-0,035921902	-0,179679542	-0,193679542	**-1,453679542**
chrXII	459628	459796	168	3,755720849	3,331576751	0,529922837	0,075075895	0,063814511	0,19542209	0,02742209	**-1,23257791**
chrXII	459628	459798	170	0,131079787	0,203822827	0,391396727	-0,275680526	-0,234328447	-0,05370819	-0,23570819	**-1,49570819**
chrXII	459628	459804	176	0,250761331	0,188999349	0,570222266	0,176940065	0,150399055	0,332024167	0,108024167	**-1,151975833**
chrXII	459628	459806	178	0,173823196	0,374292827	0,31712847	-0,475743762	-0,404382198	-0,27017145	-0,50817145	**-1,76817145**
chrXII	459633	459756	123	0,310602104	0,17788174	0,635849287	0,347385899	0,295278014	0,704687935	0,851687935	**-0,408312065**
chrXII	459633	459774	141	1,940550757	0,907938047	0,681256235	0,471214548	0,400532366	0,333141734	0,354141734	**-0,905858266**
chrXII	459633	459796	163	0,284956058	0,26311674	0,519923739	0,049962185	0,042467857	0,075175677	-0,057824323	**-1,317824323**
chrXII	459633	459798	165	0,065539893	0,092646739	0,414320049	-0,216446021	-0,183979117	-0,085815425	-0,232815425	**-1,492815425**
chrXII	459633	459804	171	0,151026711	0,08894087	0,629362977	0,330166647	0,28064165	0,413188338	0,224188338	**-1,035811662**
chrXII	459633	459806	173	0,108283302	0,140823044	0,434687048	-0,164453569	-0,139785534	0,028962189	-0,174037811	**-1,434037811**
chrXII	459648	459796	148	0,262159574	0,17417587	0,600821174	0,255473185	0,217152207	0,074104443	0,046104443	**-1,213895557**
chrXII	459673	459804	131	3,938092726	2,820166749	0,582708128	0,208826422	0,177502459	0,203789038	0,294789038	**-0,965210962**
chrXII	459673	459806	133	0,740885752	1,030231743	0,418315416	-0,206204936	-0,175274196	-0,178716608	-0,101716608	**-1,361716608**
chrXII	459686	459793	107	0,393239361	0,52623348	0,427679147	-0,182285988	-0,154943089	0,246148572	0,505148572	**-0,754851428**
chrXII	459686	459796	110	0,210867483	0,715232829	0,227693999	-0,746462631	-0,634493236	0,043516857	0,281516857	**-0,978483143**
chrXII	459686	459798	112	0,056991212	0,170470001	0,250553539	-0,67274886	-0,571836531	-0,087389994	0,136610006	**-1,123389994**
chrXII	459686	459799	113	0,43883233	0,767115003	0,363890129	-0,348079796	-0,295867827	0,250356662	0,467356662	**-0,792643338**
chrXII	459686	459801	115	0,028495606	0,096352609	0,228241997	-0,744648908	-0,632951572	-0,012719075	0,190280925	**-1,069719075**
chrXII	459686	459804	118	0,692443222	1,31928957	0,344202383	-0,401020859	-0,34086773	0,058581584	0,240581584	**-1,019418416**
chrXII	459686	459806	120	3,009135976	10,98419743	0,215040683	-0,789052425	-0,670694561	-0,283134601	-0,115134601	**-1,375134601**
chrXII	459686	459856	170	0,051292091	0,133411305	0,277699771	-0,589688448	-0,501235181	-0,321960877	-0,503960877	**-1,763960877**
chrXII	459688	459793	105	0,074088575	0,52623348	0,123414715	-1,158084807	-0,984372086	-0,391871398	-0,118871398	**-1,378871398**
chrXII	459688	459851	163	0,051292091	0,048176305	0,515662191	0,039269381	0,033378974	0,067957472	-0,065042528	**-1,325042528**
chrXII	459698	459804	106	0,051292091	0,181587609	0,220251445	-0,771344283	-0,655642641	-0,248933518	0,017066482	**-1,242933518**
chrXII	459698	459806	108	0,037044288	0,148234783	0,199937788	-0,84184347	-0,715566949	-0,057499049	0,194500951	**-1,065499049**
chrXII	459698	459851	153	0,116831984	0,151940653	0,434687048	-0,164453569	-0,139785534	-0,251371076	-0,314371076	**-1,574371076**
chrXII	459698	459869	171	0,062690333	0,08894087	0,413439528	-0,218706025	-0,185900121	-0,048642081	-0,237642081	**-1,497642081**
chrXII	459818	459980	162	0,04844253	0,077823261	0,383655221	-0,295894775	-0,251510559	-0,224125296	-0,350125296	**-1,610125296**
chrXII	459834	459969	135	0,555664314	0,726350437	0,433430515	-0,167646954	-0,142499911	-0,137389082	-0,074389082	**-1,334389082**
chrXII	459846	459969	123	0,054141651	0,103764348	0,342872667	-0,404635672	-0,343940321	0,056182004	0,203182004	**-1,056817996**
chrXII	459846	460002	156	0,233663968	0,35205761	0,398933515	-0,256108537	-0,217692257	-0,239955114	-0,323955114	**-1,583955114**
chrXII	459899	460068	169	0,119681545	0,200116957	0,374240479	-0,320642986	-0,272546538	-0,137256926	-0,312256926	**-1,572256926**
chrXII	459899	460068	169	0,119681545	0,200116957	0,374240479	-0,320642986	-0,272546538	-0,137256926	-0,312256926	**-1,572256926**
chrXII	459930	460057	127	0,188070999	0,503998263	0,2717517	-0,607523728	-0,516395169	-0,270320036	-0,151320036	**-1,411320036**
chrXII	460017	460128	111	0,054141651	0,070411522	0,434687048	-0,164453569	-0,139785534	0,336307004	0,567307004	**-0,692692996**
chrXII	460017	460159	142	2,00039153	1,360054135	0,595275666	0,241137346	0,204966744	0,20019254	0,21419254	**-1,04580746**
chrXII	460032	460159	127	0,63545201	1,196995874	0,346777671	-0,394034805	-0,334929585	-0,078835518	0,040164482	**-1,219835518**
chrXII	460102	460251	149	1,672692063	0,870879351	0,65761553	0,405964152	0,345069529	0,153040795	0,118040795	**-1,141959205**
chrXII	460102	460266	164	0,136778908	0,092646739	0,596179675	0,243470869	0,206950239	0,185863164	0,045863164	**-1,214136836**
chrXII	460112	460251	139	0,424584527	0,370586958	0,533953411	0,085211589	0,072429851	0,041041141	0,076041141	**-1,183958859**
chrXII	460112	460266	154	1,934851636	1,623170875	0,543799717	0,110011108	0,093509442	-0,089879802	-0,159879802	**-1,419879802**
chrXII	460188	460366	178	0,227964847	0,381704567	0,373915505	-0,321500662	-0,273275563	-0,13467678	-0,37267678	**-1,63267678**
chrXII	460243	460384	141	0,504372223	0,307587175	0,621179118	0,308579045	0,262292188	0,193761091	0,214761091	**-1,045238909**
chrXII	460256	460415	159	0,091185939	0,151940653	0,37505539	-0,318493294	-0,2707193	-0,293160485	-0,398160485	**-1,658160485**
chrXII	460262	460436	174	0,034194727	0,111176087	0,235224155	-0,721749813	-0,613487341	-0,390156586	-0,600156586	**-1,860156586**
chrXII	460302	460477	175	0,108283302	0,277940218	0,280364339	-0,581759513	-0,494495586	-0,247575025	-0,464575025	**-1,724575025**
chrXII	460314	460446	132	0,051292091	0,107470218	0,323074734	-0,459117949	-0,390250257	-0,25320196	-0,16920196	**-1,42920196**
chrXII	460402	460571	169	0,062690333	0,133411305	0,319682862	-0,468585806	-0,398297935	-0,258998877	-0,433998877	**-1,693998877**
chrXII	460461	460595	134	0,575611238	0,463233697	0,554087736	0,135995892	0,115596508	0,120089627	0,190089627	**-1,069910373**
chrXII	460487	460616	129	0,065539893	0,085235	0,434687048	-0,164453569	-0,139785534	0,137388747	0,242388747	**-1,017611253**
chrXII	460493	460595	102	2,236905058	21,52739638	0,094128795	-1,315751121	-1,118388453	-0,590469984	-0,296469984	**-1,556469984**
chrXII	460493	460659	166	0,039893848	0,170470001	0,189642129	-0,879215839	-0,747333463	-0,594001199	-0,748001199	**-2,008001199**
chrXII	460512	460595	83	0,042743409	0,200116957	0,175999936	-0,930717198	-0,791109618	-0,477340248	-0,050340248	**-1,310340248**
chrXII	460512	460639	127	0,812124766	1,57870044	0,339683873	-0,41332605	-0,351327143	-0,088662665	0,030337335	**-1,229662665**
chrXII	460521	460659	138	0,470177496	0,518821741	0,47540734	-0,061683752	-0,052431189	-0,057402451	-0,015402451	**-1,275402451**
chrXII	460602	460763	161	0,065539893	0,103764348	0,387113121	-0,28685122	-0,243823537	-0,211887061	-0,330887061	**-1,590887061**
chrXII	460846	460979	133	0,401788042	0,70411522	0,363312105	-0,349619586	-0,297176648	-0,296111747	-0,219111747	**-1,479111747**
chrXII	460863	461022	159	0,056991212	0,048176305	0,541908887	0,105243965	0,08945737	0,069176183	-0,035823817	**-1,295823817**
chrXII	460871	461006	135	0,051292091	0,08894087	0,365763444	-0,34309514	-0,291630869	-0,286873741	-0,223873741	**-1,483873741**
chrXII	460894	460979	85	0,051292091	0,092646739	0,356346446	-0,368241871	-0,31300559	0,087974672	0,500974672	**-0,759025328**
chrXII	460894	461045	151	0,416035845	0,481763045	0,463395366	-0,091883336	-0,078100835	-0,214354561	-0,263354561	**-1,523354561**
chrXII	460894	461097	203	0,039893848	0,051882174	0,434687048	-0,164453569	-0,139785534	-0,092695745	-0,505695745	**-1,765695745**
chrXII	460906	461069	163	0,108283302	0,118587827	0,477289917	-0,056956516	-0,048413039	-0,006714976	-0,139714976	**-1,399714976**
chrXII	460927	461045	118	0,04844253	0,133411305	0,266381679	-0,623793272	-0,530224281	-0,145043342	0,036956658	**-1,223043342**
chrXII	460927	461097	170	1,350691717	1,697288267	0,443143237	-0,143004694	-0,12155399	0,064906065	-0,117093935	**-1,377093935**
chrXII	460937	461071	134	1,191116324	0,815291307	0,593656197	0,236960278	0,201416237	0,212795203	0,282795203	**-0,977204797**
chrXII	460937	461098	161	0,279256937	0,403939784	0,408750406	-0,230760574	-0,196146488	-0,163901964	-0,282901964	**-1,542901964**
chrXII	460988	461071	83	0,213717044	0,418763262	0,33790308	-0,418192798	-0,355463878	-0,02369586	0,40330414	**-0,85669586**
chrXII	460988	461095	107	2,276798906	12,01813504	0,159273132	-0,997449727	-0,847832268	-0,43776598	-0,17876598	**-1,43876598**
chrXII	460988	461096	108	0,068389454	0,211234566	0,244576464	-0,691656728	-0,587908219	0,062926921	0,314926921	**-0,945073079**
chrXII	460988	461098	110	2,447772541	16,56894289	0,128716894	-1,132477383	-0,962605776	-0,277774635	-0,039774635	**-1,299774635**
chrXII	461013	461146	133	0,917558508	1,033937612	0,470182082	-0,074812165	-0,06359034	-0,084856788	-0,007856788	**-1,267856788**
chrXII	461056	461146	90	0,037044288	0,062999783	0,370279692	-0,331112667	-0,281445767	0,143986723	0,521986723	**-0,738013277**
chrXII	461056	461148	92	0,059840772	0,381704567	0,13552577	-1,100644199	-0,935547569	-0,551851605	-0,187851605	**-1,447851605**
chrXII	461056	461199	143	0,056991212	0,085235	0,400708216	-0,251514398	-0,213787238	-0,36288963	-0,35588963	**-1,61588963**
chrXII	461056	461212	156	0,122531105	0,085235	0,589755027	0,226914861	0,192877632	0,166618636	0,082618636	**-1,177381364**
chrXII	461056	461214	158	0,088336378	0,08894087	0,498295067	-0,004273647	-0,0036326	-0,024805765	-0,122805765	**-1,382805765**
chrXII	461056	461215	159	0,891912463	0,644821307	0,580394913	0,202903865	0,172468285	0,147797059	0,042797059	**-1,217202941**
chrXII	461056	461229	173	0,085486818	0,077823261	0,523463207	0,058847485	0,050020363	0,210306733	0,007306733	**-1,252693267**
chrXII	461060	461146	86	0,045592969	0,062999783	0,419852784	-0,202270107	-0,171929591	0,218427844	0,624427844	**-0,635572156**
chrXII	461060	461212	152	1,142673794	0,748585655	0,604186694	0,264198961	0,224569117	0,040143336	-0,015856664	**-1,275856664**
chrXII	461060	461214	154	0,054141651	0,085235	0,388455674	-0,283346352	-0,2408444	-0,418558927	-0,488558927	**-1,748558927**

Chrom	Start	End	Length	Section A	Section B	A/A+B	Z-score	Z * 0.85	Phase correction	Length correction	ΔLKnuc
chrXII	461060	461215	155	0,056991212	0,055588044	0,506231912	0,015621722	0,013278464	-0,082763076	-0,159763076	-1,419763076
chrXII	461060	461229	169	0,185221438	0,08894087	0,675590454	0,45540334	0,387092839	0,528669881	0,353669881	-0,906330119
chrXII	461068	461199	131	0,401788042	0,474351306	0,458589199	-0,103988596	-0,088390306	-0,039852873	0,051147127	-1,208852873
chrXII	461068	461214	146	2,239754619	1,608347397	0,582041383	0,207118603	0,176050812	0,033841175	0,019841175	-1,240158825
chrXII	461068	461246	178	0,071239015	0,066705652	0,516431814	0,041200102	0,035020087	0,175386258	-0,062613742	-1,322613742
chrXII	461069	461199	130	4,938288491	5,677392194	0,465188115	-0,08737149	-0,074265766	0,042806057	0,140806057	-1,119193943
chrXII	461069	461214	145	0,698142343	0,833820655	0,455717497	-0,111228699	-0,094544395	-0,287933122	-0,294933122	-1,554933122
chrXII	461069	461246	177	0,094035499	0,103764348	0,47540734	-0,061683752	-0,052431189	0,102218864	-0,128781136	-1,388781136
chrXII	461082	461188	106	0,170973635	0,370586958	0,315705458	-0,47974192	-0,407780632	0,010167577	0,276167577	-0,983832423
chrXII	461082	461199	117	0,179522317	0,292763697	0,380113558	-0,305182559	-0,259405175	0,261048571	0,450048571	-0,809951429
chrXII	461082	461212	130	0,173823196	0,159352392	0,521716483	0,054462061	0,046292752	0,15997489	0,25797489	-1,00202511
chrXII	461082	461214	132	3,345384125	3,264871099	0,506090009	0,015265981	0,012976084	0,154760044	0,238760044	-1,021239956
chrXII	461082	461215	133	0,122531105	0,111176087	0,524293257	0,060931847	0,05179207	0,032401436	0,109401436	-1,150598564
chrXII	461082	461229	147	2,766923327	1,348936527	0,672258878	0,446159211	0,37923533	0,263033894	0,242033894	-1,017966106
chrXII	461082	461246	164	0,179522317	0,17417587	0,507557922	0,018946034	0,016104129	0,004248782	-0,135751218	-1,395751218
chrXII	461095	461246	151	0,521469587	0,237175653	0,687369484	0,488407779	0,415146613	0,283029678	0,234029678	-1,025970322
chrXII	461178	461333	155	0,153876272	0,144528914	0,515662191	0,039269381	0,033378974	-0,052661098	-0,129661098	-1,389661098
chrXII	461196	461352	156	0,256460453	0,26311674	0,493594515	-0,016056859	-0,01364833	-0,046413046	-0,130413046	-1,390413046
chrXII	461218	461364	146	0,558513874	0,366881088	0,603541079	0,262523537	0,223145006	0,060113714	0,046113714	-1,213886286
chrXII	461218	461384	166	1,225311051	1,297054353	0,485778567	-0,0356554	-0,03030709	0,112102742	-0,041897258	-1,301897258
chrXII	461218	461388	170	0,105433742	0,077823261	0,575332675	0,189967434	0,161472318	0,335294378	0,153294378	-1,106705622
chrXII	461241	461384	143	0,062690333	0,040764565	0,605967759	0,268824834	0,228501109	0,079641393	0,086641393	-1,173358607
chrXII	461241	461388	147	0,339097709	0,114881957	0,746944708	0,664906054	0,565170146	0,457522133	0,436522133	-0,823477867
chrXII	461252	461408	156	0,558513874	0,374292827	0,598745564	0,250101478	0,212586256	0,175687621	0,091687621	-1,168312379
chrXII	461260	461367	107	0,042743409	0,155646522	0,215451503	-0,787647358	-0,669500254	-0,257905485	0,001094515	-1,258905485
chrXII	461327	461481	154	0,592708601	0,433586741	0,577522451	0,195559327	0,166225428	-0,013883463	-0,083883463	-1,343883463
chrXII	461397	461551	154	0,353345512	0,281646088	0,556456986	0,141992377	0,12069352	-0,064557994	-0,134557994	-1,394557994
chrXII	461430	461537	107	0,113982423	0,893114568	0,113179192	-1,209792869	-1,028323939	-0,592911389	-0,333911389	-1,593911389
chrXII	461431	461592	161	0,572761677	0,518821741	0,524707198	0,061971406	0,052675695	0,074769109	-0,044230891	-1,304230891
chrXII	461432	461592	160	0,313451664	0,233469783	0,573120081	0,184323297	0,156674802	0,132888108	0,020888108	-1,239111892
chrXII	461436	461592	156	0,213717044	0,148234783	0,590457149	0,22872109	0,194412926	0,157208737	0,073208737	-1,186791263
chrXII	461460	461622	162	0,042743409	0,048176305	0,470122564	-0,074961771	-0,063717505	-0,033288148	-0,159288148	-1,419288148
chrXII	461460	461623	163	0,322000346	0,311293045	0,508453666	0,021191784	0,018013016	0,059358846	-0,073641154	-1,333641154
chrXII	461471	461578	107	0,042743409	0,059293913	0,418899751	-0,204708983	-0,174002636	0,270052943	0,529052943	-0,730947057
chrXII	461471	461592	121	0,054141651	0,081529131	0,399066404	-0,255764342	-0,217399691	0,176353223	0,337353223	-0,922646777
chrXII	461471	461622	151	0,624053768	0,403939784	0,607060002	0,271664505	0,23091483	0,099281351	0,050281351	-1,209718649
chrXII	461471	461623	152	0,076938136	0,062999783	0,549801916	0,125160904	0,106386768	-0,093184694	-0,149184694	-1,409184694
chrXII	461483	461633	150	0,19661968	0,137117174	0,589145842	0,225348315	0,191546068	0,053970159	0,011970159	-1,248029841
chrXII	461536	461685	149	0,054141651	0,044470435	0,549036667	0,123227859	0,104743681	-0,078995135	-0,113995135	-1,373995135
chrXII	461560	461721	161	0,14532759	0,188999349	0,434687048	-0,164453569	-0,139785534	-0,123916154	-0,242916154	-1,502916154
chrXII	461606	461757	151	0,279256937	0,381704567	0,422501062	-0,195499251	-0,166174364	-0,308421207	-0,357421207	-1,617421207
chrXII	461606	461758	152	0,356195073	0,433586741	0,4510044	-0,123124135	-0,104655515	-0,307816206	-0,363816206	-1,623816206
chrXII	461606	461766	160	0,267858695	0,311293045	0,462501753	-0,094133	-0,08001305	-0,104822976	-0,216822976	-1,476822976
chrXII	461606	461767	161	0,225115286	0,211234566	0,515905494	0,03987973	0,03389777	0,044849179	-0,074150821	-1,334150821
chrXII	461628	461758	130	0,074088575	0,270528479	0,214988127	-0,789232287	-0,670847444	-0,546770553	-0,448770553	-1,708770553
chrXII	461628	461766	138	0,034194727	0,074117392	0,315705458	-0,47974192	-0,407780632	-0,4071081	-0,3651081	-1,6251081
chrXII	461628	461800	172	0,208017923	0,311293045	0,400565241	-0,251884318	-0,21410167	-0,089659216	-0,285659216	-1,545659216
chrXII	461629	461721	92	0,028495606	0,103764348	0,215451503	-0,787647358	-0,669500254	-0,282017207	0,081982793	-1,178017207
chrXII	461629	461757	128	0,085486818	0,289057827	0,228241997	-0,744648908	-0,632951572	-0,406312406	-0,294312406	-1,554312406
chrXII	461629	461758	129	1,373488201	3,828163275	0,264048487	-0,630913699	-0,536276619	-0,266107881	-0,161107881	-1,421107881
chrXII	461629	461766	137	0,082637257	0,17788174	0,317202423	-0,475536189	-0,40420576	-0,335194707	-0,286194707	-1,546194707
chrXII	461629	461800	171	0,039893848	0,148234783	0,212056229	-0,799306935	-0,679410895	-0,531364364	-0,720364364	-1,980364364
chrXII	461662	461758	96	0,045592969	0,092646739	0,329810947	-0,440435256	-0,374369967	0,197806774	0,533806774	-0,726193226
chrXII	461662	461832	170	0,045592969	0,077823261	0,36942442	-0,33337817	-0,283371444	-0,104249051	-0,286249051	-1,546249051
chrXII	461676	461810	134	0,19661968	0,389116306	0,3356797	-0,424283048	-0,360640591	-0,352380032	-0,282380032	-1,542380032
chrXII	461687	461800	113	0,054141651	0,137117174	0,283080537	-0,573714411	-0,48765725	0,062123162	0,279123162	-0,980876838
chrXII	461687	461809	122	0,24506221	1,304466092	0,158152781	-1,002078746	-0,851766934	-0,496595426	-0,342595426	-1,602595426
chrXII	461687	461839	152	0,168124074	0,114881957	0,594065341	0,238015182	0,202312905	-0,003174046	-0,059174046	-1,319174046
chrXII	461805	461933	128	0,239363089	0,537351089	0,308173966	-0,501032951	-0,425878008	-0,220454211	-0,108454211	-1,368454211
chrXII	461805	461951	146	0,037044288	0,044470435	0,454449042	-0,114428548	-0,097264266	-0,259184236	-0,273184236	-1,533184236
chrXII	461821	461933	112	0,059840772	0,114881957	0,342489913	-0,405677162	-0,344825587	0,16215198	0,38615198	-0,87384802
chrXII	461821	461972	151	0,581310359	0,35205761	0,622809415	0,312867712	0,265937555	0,123803557	0,074803557	-1,185196443
chrXII	461823	461951	128	0,752283994	1,067290439	0,413439528	-0,218706025	-0,185900121	0,048728662	0,160728662	-1,099271338
chrXII	461823	462001	178	0,031345166	0,048176305	0,394172367	-0,268460684	-0,228191582	-0,091564566	-0,329564566	-1,589564566
chrXII	461846	461952	106	0,059840772	0,074117392	0,446712395	-0,133971905	-0,113876119	0,318516123	0,584516123	-0,675483877
chrXII	461846	462001	155	0,806425645	0,722644568	0,527396086	0,068725866	0,058416986	-0,030892209	-0,107892209	-1,367892209
chrXII	461846	462021	175	0,111132863	0,107470218	0,508377386	0,021000537	0,017850457	0,252500839	0,035500839	-1,224499161
chrXII	461846	462037	191	0,079787696	0,074117392	0,518421434	0,046192108	0,039263292	0,086431018	-0,242568982	-1,502568982
chrXII	461860	461951	91	0,039893848	0,051882174	0,434687048	-0,164453569	-0,139785534	0,252830473	0,623830473	-0,636169527
chrXII	461860	462001	141	0,128230226	0,159352392	0,445890044	-0,136052108	-0,115644291	-0,180207735	-0,159207735	-1,419207735
chrXII	461860	462021	161	0,971700159	0,752291524	0,563633902	0,160188997	0,136160647	0,140268931	0,021268931	-1,238731069
chrXII	461860	462037	177	0,131079787	0,129705435	0,502635026	0,006605078	0,005614317	0,157910486	-0,073089514	-1,333089514
chrXII	461902	462001	99	0,09688506	0,148234783	0,395255882	-0,265646117	-0,2257992	0,236616762	0,551616762	-0,708383238
chrXII	461902	462008	106	0,088336378	0,17788174	0,331819558	-0,434894351	-0,369660198	0,078436835	0,344436835	-0,915563165
chrXII	461902	462021	119	0,105433742	0,122293696	0,462982163	-0,092923514	-0,078984987	0,329290222	0,504290222	-0,755709778
chrXII	461902	462037	135	2,396480451	2,015993051	0,543114978	0,108284468	0,092041798	0,071954108	0,134954108	-1,125045892
chrXII	461902	462062	160	0,153876272	0,062999783	0,7095125	0,551961108	0,469166941	0,445668795	0,333668795	-0,926331205
chrXII	461902	462062	160	0,153876272	0,062999783	0,7095125	0,551961108	0,469166941	0,445668795	0,333668795	-0,926331205
chrXII	461910	462001	91	0,04844253	0,096352609	0,33455909	-0,427358568	-0,363254783	0,020030635	0,391030635	-0,868969365
chrXII	461910	462016	106	0,037044288	0,08894087	0,294036919	-0,541629394	-0,460384985	-0,008198979	0,257801021	-1,002198979
chrXII	461910	462021	111	0,056991212	0,092646739	0,380860679	-0,303221116	-0,257737948	0,210131886	0,441131886	-0,818868114
chrXII	461910	462037	127	0,185221438	0,233469783	0,442381948	-0,144932843	-0,123192917	0,147417871	0,266417871	-0,993582129
chrXII	461910	462062	152	0,795027403	0,578115654	0,578983667	0,199294137	0,169400016	-0,039538681	-0,095538681	-1,355538681
chrXII	461910	462062	152	0,795027403	0,578115654	0,578983667	0,199294137	0,169400016	-0,039538681	-0,095538681	-1,355538681
chrXII	461920	462099	179	0,056991212	0,133411305	0,299319635	-0,52635832	-0,447404572	-0,290874783	-0,535874783	-1,795874783
chrXII	461925	462060	135	0,681044979	0,548468698	0,553914114	0,135556657	0,115223159	0,094841478	0,157841478	-1,102158522
chrXII	461925	462090	165	0,094035499	0,100058479	0,484484373	-0,038901718	-0,03306646	0,061915785	-0,085084215	-1,345084215
chrXII	461938	462060	122	0,125380666	0,163058261	0,434687048	-0,164453569	-0,139785534	0,220891379	0,374891379	-0,885108621
chrXII	461938	462090	152	2,142869559	1,371171744	0,609802041	0,278803123	0,236982654	0,037822466	-0,018177534	-1,278177534
chrXII	461938	462136	198	0,04844253	0,055588044	0,465656665	-0,08619258	-0,073263693	-0,032009443	-0,410009443	-1,670009443
chrXII	461977	462090	113	0,153876272	0,229763914	0,40109529	-0,250513093	-0,212936129	0,339026421	0,556026421	-0,703973579
chrXII	461977	462136	159	0,629752889	0,511410002	0,551851882	0,130341515	0,110790288	0,081974808	-0,023025192	-1,283025192
chrXII	461977	462160	183	0,074088575	0,077823261	0,487707719	-0,030817056	-0,026194498	0,068079991	-0,204920009	-1,464920009
chrXII	461984	462090	106	0,031345166	0,055588044	0,360566076	-0,356946107	-0,303404191	0,167267369	0,433267369	-0,826732631
chrXII	461984	462133	149	0,210867483	0,203822827	0,508493876	0,021292599	0,018098709	-0,177566543	-0,212566543	-1,472566543
chrXII	462013	462171	158	1,590054806	1,330407179	0,544453177	0,111659181	0,094910303	0,067212132	-0,030787868	-1,290787868

DATA 2

Chrom	Start	End	Length	Section A	Section B	A/A+B	Z-score	Z * 0.85	Phase correction	Length correction	ΔLKnuc
chrXII	462022	462171	149	0,276407377	0,155646522	0,639752071	0,357796169	0,304126743	0,114965081	0,079965081	**-1,180034919**
chrXII	462030	462136	106	0,065539893	0,096352609	0,404835878	-0,240849509	-0,204722082	0,254346396	0,520346396	**-0,739653604**
chrXII	462032	462160	128	1,493169746	1,752876311	0,459996476	-0,100442599	-0,08537621	0,16694006	0,27894006	**-0,98105994**
chrXII	462059	462166	107	1,339293474	7,960207855	0,144017773	-1,062440962	-0,903074817	-0,463163252	-0,204163252	**-1,464163252**
chrXII	462059	462167	108	0,034194727	0,244587392	0,122657533	-1,161804056	-0,987533448	-0,316770286	-0,064770286	**-1,324770286**
chrXII	462059	462171	112	1,689789426	10,28008221	0,141170221	-1,075076584	-0,913815096	-0,398563087	-0,174563087	**-1,434563087**
chrXII	462059	462193	134	0,076938136	0,100058479	0,434687048	-0,164453569	-0,139785534	-0,123044451	-0,053044451	**-1,313044451**
chrXII	462059	462212	153	0,042743409	0,070411522	0,377742344	-0,311415611	-0,264703269	-0,383287945	-0,446287945	**-1,706287945**
chrXII	462063	462134	71	0,056991212	0,140823044	0,288104674	-0,558930214	-0,475090682		0,511	**-0,749**
chrXII	462063	462170	107	0,119681545	0,274234349	0,303825122	-0,513430458	-0,436415889	0,021477307	0,280477307	**-0,979522693**
chrXII	462063	462171	108	0,29350474	0,470645436	0,384093008	-0,294748492	-0,250536218	0,402970359	0,654970359	**-0,605029641**
chrXII	462063	462193	130	0,578460798	0,592939133	0,493820072	-0,015491401	-0,013167691	0,119210153	0,217210153	**-1,042789847**
chrXII	462063	462207	144	0,074088575	0,040764565	0,645072262	0,372050197	0,316242667	0,197359946	0,197359946	**-1,062640054**
chrXII	462063	462212	149	0,455929693	0,370586958	0,551627959	0,129775456	0,110309137	-0,089053019	-0,124053019	**-1,384053019**
chrXII	462113	462275	162	0,24221265	0,344645871	0,412727499	-0,220534382	-0,187454224	-0,153597824	-0,279597824	**-1,539597824**
chrXII	462128	462278	150	0,889062902	0,61146848	0,592498706	0,233977317	0,198880719	0,059440052	0,017440052	**-1,242559948**
chrXII	462168	462275	107	0,034194727	0,081529131	0,295485544	-0,537429332	-0,456814932	0,007584165	0,266584165	**-0,993415835**
chrXII	462170	462300	130	1,313647429	1,700994137	0,435755761	-0,161738835	-0,137478009	-0,011539009	0,086460991	**-1,173539009**
chrXII	462170	462324	154	0,14817715	0,118587827	0,555459536	0,139467258	0,118547169	-0,072908952	-0,142908952	**-1,402908952**
chrXII	462170	462330	160	0,071239015	0,08894087	0,444743825	-0,138952542	-0,118109661	-0,132258326	-0,244258326	**-1,504258326**
chrXII	462176	462300	124	0,142478029	0,159352392	0,472046617	-0,070126174	-0,059607248	0,328147196	0,468147196	**-0,791852804**
chrXII	462176	462324	148	1,151222476	0,61888022	0,650370444	0,386320781	0,328372663	0,182191959	0,154191959	**-1,105808041**
chrXII	462176	462330	154	0,113982423	0,066705652	0,630824269	0,334037235	0,283931649	0,107726902	0,037726902	**-1,222273098**
chrXII	462179	462349	170	0,04844253	0,103764348	0,31826768	-0,472548462	-0,401666193	-0,214822088	-0,396822088	**-1,656822088**
chrXII	462179	462355	176	0,644000692	0,641115437	0,501122566	0,002813859	0,00239178	0,173083655	-0,050916345	**-1,310916345**
chrXII	462180	462300	120	0,162424953	0,289057827	0,359758911	-0,359103289	-0,305237796	0,084472506	0,252472506	**-1,007527494**
chrXII	462180	462324	144	0,165274514	0,111176087	0,597844653	0,247772164	0,210606339	0,093207335	0,093207335	**-1,166792665**
chrXII	462180	462327	147	0,322000346	0,207528696	0,608088169	0,274339592	0,233188653	0,124070637	0,103070637	**-1,156929363**
chrXII	462180	462330	150	1,51311667	1,089525656	0,581377108	0,205417712	0,174605055	0,020891488	-0,021108512	**-1,281108512**
chrXII	462228	462336	108	0,133929347	0,166764131	0,44540157	-0,137288019	-0,116694816	0,524238864	0,776238864	**-0,483761136**
chrXII	462229	462336	107	1,578656563	2,864637184	0,355289714	-0,371078012	-0,315416311	0,159397885	0,418397885	**-0,841602115**
chrXII	462229	462337	108	0,042743409	0,092646739	0,315705458	-0,47974192	-0,407780632	0,237560225	0,489560225	**-0,770439775**
chrXII	462229	462349	120	1,407682928	3,313047403	0,298191769	-0,529608301	-0,450167056	-0,056835858	0,111164142	**-1,148835858**
chrXII	462229	462373	144	0,09973462	0,148234783	0,402205349	-0,247642923	-0,210496484	-0,33916081	-0,33916081	**-1,59916081**
chrXII	462229	462382	153	0,247911771	0,222352175	0,527175798	0,068172392	0,057946533	-0,061740384	-0,124740384	**-1,384740384**
chrXII	462229	462388	159	0,042743409	0,037058696	0,535617563	0,08939893	0,07598909	0,039720575	-0,065279425	**-1,325279425**
chrXII	462229	462407	178	0,09688506	0,188999349	0,338895921	-0,415478239	-0,353156503	-0,213292995	-0,451292995	**-1,711292995**
chrXII	462229	462423	194	0,159575393	0,222352175	0,417815854	-0,20748423	-0,176361595	-0,10060805	-0,45060805	**-1,71060805**
chrXII	462229	462425	196	0,119681545	0,218646305	0,353744289	-0,375231134	-0,318946464	-0,273176877	-0,637176877	**-1,897176877**
chrXII	462232	462338	106	0,09688506	0,251999131	0,277699771	-0,589688448	-0,501235181	-0,016167448	0,249832552	**-1,010167448**
chrXII	462232	462349	117	0,122531105	0,229763914	0,347808225	-0,391244593	-0,332557904	0,203462798	0,392462798	**-0,867537202**
chrXII	462232	462355	123	0,042743409	0,048176305	0,470122564	-0,074961771	-0,063717505	0,349751273	0,496751273	**-0,763248727**
chrXII	462232	462373	141	0,054141651	0,092646739	0,368841506	-0,334923211	-0,284684729	-0,344144002	-0,323144002	**-1,583144002**
chrXII	462232	462382	150	2,382232648	1,630582615	0,593656197	0,236960278	0,201416237	0,059277754	0,017277754	**-1,242722246**
chrXII	462232	462407	175	0,091185939	0,08894087	0,506231912	0,015621722	0,013278464	0,242545265	0,025545265	**-1,234454735**
chrXII	462232	462419	187	0,059840772	0,062999783	0,487141826	-0,032236246	-0,027400809	0,024745491	-0,276254509	**-1,536254509**
chrXII	462232	462423	191	0,182371877	0,181587609	0,501077411	0,002700672	0,002295572	0,049604939	-0,279395061	**-1,539395061**
chrXII	462232	462425	193	0,14532759	0,192705218	0,429921553	-0,176573888	-0,150087805	-0,090854267	-0,433854267	**-1,693854267**
chrXII	462236	462373	137	1,31934655	0,763409133	0,633461985	0,341036599	0,289881109	0,355526437	0,404526437	**-0,855473563**
chrXII	462236	462425	189	0,156725832	0,111176087	0,585011979	0,214732295	0,182522451	0,227455153	-0,087544847	**-1,347544847**
chrXII	462237	462375	138	0,051292091	0,051882174	0,497140355	-0,007168129	-0,00609291	-0,004113025	0,037886975	**-1,222113025**
chrXII	462237	462382	145	0,176672756	0,077823261	0,694206369	0,507809051	0,431637694	0,237608696	0,230608696	**-1,029391304**
chrXII	462250	462382	132	0,068389454	0,103764348	0,397257877	-0,260451199	-0,221383519	-0,082446751	0,001553249	**-1,258446751**
chrXII	462250	462407	157	0,404637603	0,426175002	0,487038353	-0,032495748	-0,027621386	-0,120150145	-0,211150145	**-1,471150145**
chrXII	462250	462423	173	0,074088575	0,144528914	0,338895921	-0,415478239	-0,353156503	-0,174996903	-0,377996903	**-1,637996903**
chrXII	462250	462425	175	0,04844253	0,08894087	0,352608321	-0,378288023	-0,32154482	-0,111528981	-0,328528981	**-1,588528981**
chrXII	462251	462373	122	0,159575393	0,125999566	0,558786364	0,147892925	0,125708986	0,522424323	0,676424323	**-0,583575677**
chrXII	462251	462406	155	0,076938136	0,059293913	0,564757972	0,163043664	0,138587115	0,052002181	-0,024997819	**-1,284997819**
chrXII	462251	462407	156	3,083224551	2,012287181	0,605086341	0,266534858	0,226554629	0,180591704	0,096591704	**-1,163408296**
chrXII	462251	462423	172	0,304902982	0,251999131	0,547498339	0,119343374	0,101441868	0,212393688	0,016393688	**-1,243606312**
chrXII	462251	462424	173	0,3903898	0,444704349	0,467479984	-0,081606077	-0,069365165	0,117633691	-0,085366309	**-1,345366309**
chrXII	462251	462425	174	0,983098401	1,171054787	0,456373487	-0,109574325	-0,093138177	0,144537921	-0,065462079	**-1,325462079**
chrXII	462251	462439	188	0,09973462	0,118587827	0,456822566	-0,108441943	-0,092175651	-0,075109934	-0,383109934	**-1,643109934**
chrXII	462253	462406	153	0,49012442	0,188999349	0,721701173	0,587902625	0,499717231	0,381870413	0,318870413	**-0,941129587**
chrXII	462253	462407	154	0,102584181	0,066705652	0,605967759	0,268824834	0,228501109	0,046129994	-0,023870006	**-1,283870006**
chrXII	462253	462424	171	0,056991212	0,051882174	0,523463207	0,058847485	0,050020363	0,196335149	0,007335149	**-1,252664851**
chrXII	462253	462425	172	0,14532759	0,08894087	0,620346375	0,30639062	0,260432027	0,371383847	0,175383847	**-1,084616153**
chrXII	462256	462373	117	0,185221438	0,244587392	0,430939117	-0,173983748	-0,147886185	0,382935317	0,571935317	**-0,688064683**
chrXII	462256	462388	132	0,054141651	0,133411305	0,288673942	-0,557262806	-0,473673385	-0,330836606	-0,246836606	**-1,506836606**
chrXII	462256	462406	150	0,156725832	0,114881957	0,577029962	0,194301161	0,165155987	0,013414443	-0,028585557	**-1,288585557**
chrXII	462256	462407	151	1,225311051	1,182172396	0,508959284	0,022459482	0,01909056	-0,120895149	-0,169895149	**-1,429895149**
chrXII	462256	462419	163	0,051292091	0,251999131	0,169118282	-0,957655378	-0,814007071	-0,774397761	-0,907397761	**-2,167397761**
chrXII	462256	462423	167	0,658248495	0,893114568	0,424303318	-0,19089656	-0,162262076	-0,014482441	-0,175482441	**-1,435482441**
chrXII	462256	462424	168	1,031540931	1,237760439	0,454563217	-0,114140479	-0,097019408	0,027285127	-0,140714873	**-1,400714873**
chrXII	462256	462425	169	7,366114108	7,674855897	0,48973664	-0,025729268	-0,021869877	0,1235177	-0,0514823	**-1,3114823**
chrXII	462256	462439	183	0,284956058	0,318704784	0,472046617	-0,070126174	-0,059607248	0,025731811	-0,247268189	**-1,507268189**
chrXII	462256	462446	190	0,113982423	0,17417587	0,395554894	-0,264869768	-0,225139303	-0,206095378	-0,528095378	**-1,788095378**
chrXII	462257	462365	108	0,079787696	0,074117392	0,518421434	0,046192108	0,039263292	0,671846555	0,923846555	**-0,336153445**
chrXII	462258	462407	149	1,168319839	0,726350437	0,616634912	0,296654659	0,25215646	0,057675139	0,022675139	**-1,237324861**
chrXII	462258	462423	165	0,250761331	0,289057827	0,464528403	-0,089031589	-0,075676851	0,02879259	-0,11820741	**-1,37820741**
chrXII	462258	462425	167	0,153876272	0,233469783	0,397257877	-0,260451199	-0,221383519	-0,070431317	-0,231431317	**-1,491431317**
chrXII	462262	462368	106	0,24506221	0,841232394	0,225594612	-0,753433889	-0,640418806	-0,13643658	0,12956342	**-1,13043658**
chrXII	462262	462373	111	0,202318801	0,830114786	0,195963018	-0,856129712	-0,727710255	-0,25956716	-0,02856716	**-1,28856716**
chrXII	462262	462407	145	0,461628815	0,43729261	0,513536336	0,033937075	0,028846514	-0,153251734	-0,160251734	**-1,420251734**
chrXII	462262	462419	157	0,042743409	0,144528914	0,228241997	-0,744648908	-0,632951572	-0,725701185	-0,816701185	**-2,076701185**
chrXII	462262	462423	161	0,401788042	0,418763262	0,48965621	-0,025930941	-0,0220413	-0,025982031	-0,144982031	**-1,404982031**
chrXII	462262	462424	162	2,806817175	2,223521747	0,557977746	0,145844042	0,123967435	0,168951192	0,042951192	**-1,217048808**
chrXII	462262	462425	163	1,339293474	1,308171961	0,505877605	0,014733504	0,012523479	0,037790173	-0,095209827	**-1,355209827**
chrXII	462262	462439	177	0,128230226	0,144528914	0,470122564	-0,074961771	-0,063717505	0,086668945	-0,144331055	**-1,404331055**
chrXII	462263	462385	122	0,165274514	0,718938698	0,186917037	-0,889314512	-0,755917335	-0,359249066	-0,205249066	**-1,465249066**
chrXII	462263	462407	144	0,159575393	0,17417587	0,478126708	-0,054855711	-0,046627354	-0,172089506	-0,172089506	**-1,432089506**
chrXII	462263	462423	160	0,58415992	0,52993935	0,524333814	0,061033699	0,051878644	0,049522866	-0,062477134	**-1,322477134**
chrXII	462263	462424	161	0,139628469	0,155646522	0,472876041	-0,06804215	-0,057835827	-0,066678272	-0,185678272	**-1,445678272**
chrXII	462263	462425	162	0,492973981	0,774526742	0,388933885	-0,282098777	-0,239783961	-0,181583167	-0,307583167	**-1,567583167**
chrXII	462272	462407	135	0,108283302	0,181587609	0,37355698	-0,322447164	-0,274080089	-0,291203503	-0,228203503	**-1,488203503**
chrXII	462272	462419	147	1,678391184	1,385995222	0,547708729	0,119874529	0,10189335	-0,011397848	-0,032397848	**-1,292397848**

Chrom	Start	End	Length	Section A	Section B	A/A+B	Z-score	Z * 0.85	Phase correction	Length correction	ΔLKnuc
chrXII	462272	462423	151	0,384690679	0,500292393	0,434687048	-0,164453569	-0,139785534	-0,270990156	-0,319990156	**-1,579990156**
chrXII	462272	462425	153	0,262159574	0,452116089	0,367028568	-0,339733626	-0,288773582	-0,413374966	-0,476374966	**-1,736374966**
chrXII	462275	462423	148	0,062690333	0,055588044	0,530023616	0,075329227	0,064029843	-0,082781459	-0,110781459	**-1,370781459**
chrXII	462275	462425	150	0,102584181	0,251999131	0,289309106	-0,555404213	-0,472093581	-0,613876347	-0,655876347	**-1,915876347**
chrXII	462277	462373	96	0,068389454	0,103764348	0,397257877	-0,260451199	-0,221383519	0,356878809	0,692878809	**-0,567121191**
chrXII	462277	462382	105	0,111132863	0,092646739	0,54535813	0,113942039	0,096850733	0,684741492	0,957741492	**-0,302258508**
chrXII	462277	462383	106	0,062690333	0,159352392	0,28233455	-0,575920231	-0,489532196	0,023544586	0,289544586	**-0,970455414**
chrXII	462277	462388	111	0,14817715	0,830114786	0,151465166	-1,03016974	-0,875644279	-0,37354736	-0,14254736	**-1,40254736**
chrXII	462277	462407	130	0,413186285	0,426175002	0,492262738	-0,019395657	-0,016486308	0,111542353	0,209542353	**-1,050457647**
chrXII	462277	462419	142	0,276407377	0,292763697	0,485631455	-0,036024391	-0,030620732	-0,014675382	-0,000675382	**-1,260675382**
chrXII	462277	462423	146	1,547311397	1,074702178	0,590123336	0,227862253	0,193682915	0,031090305	0,017090305	**-1,242909695**
chrXII	462277	462424	147	1,296550065	1,189584135	0,521512501	0,053950004	0,045857503	-0,070397919	-0,091397919	**-1,351397919**
chrXII	462277	462425	148	5,095014323	3,683634361	0,580387085	0,202883835	0,17245126	0,016854049	-0,011145951	**-1,271145951**
chrXII	462277	462439	162	0,071239015	0,133411305	0,348101165	-0,390452017	-0,331884215	-0,264712858	-0,390712858	**-1,650712858**
chrXII	462277	462460	183	0,079787696	0,059293913	0,573675388	0,185739279	0,157878387	0,241714469	-0,031285531	**-1,291285531**
chrXII	462278	462373	95	0,168124074	0,159352392	0,513392844	0,03357719	0,028540612	0,527911024	0,870911024	**-0,389088976**
chrXII	462278	462382	104	0,19377012	0,207528696	0,482857442	-0,042983253	-0,036535765	0,530633819	0,810633819	**-0,449366181**
chrXII	462278	462406	128	0,09688506	0,048176305	0,667890174	0,43409473	0,36898052	0,634153169	0,746153169	**-0,513846831**
chrXII	462278	462407	129	0,943204553	0,893114568	0,513638693	0,034193795	0,029064726	0,296966478	0,401966478	**-0,858033522**
chrXII	462278	462418	140	0,042743409	0,051882174	0,451710916	-0,121339881	-0,103138899	-0,080192965	-0,052192965	**-1,312192965**
chrXII	462278	462419	141	0,336248149	0,359469349	0,483311329	-0,041844502	-0,035567826	-0,091812732	-0,070812732	**-1,330812732**
chrXII	462278	462420	142	0,031345166	0,051882174	0,376621026	-0,314367347	-0,267212245	-0,247351734	-0,233351734	**-1,493351734**
chrXII	462278	462423	145	6,39726351	3,87263371	0,622914073	0,313143221	0,266171738	0,095348549	0,088348549	**-1,171651451**
chrXII	462278	462424	146	0,988797522	1,019114134	0,492450711	-0,018924391	-0,016085733	-0,186693145	-0,200693145	**-1,460693145**
chrXII	462278	462425	147	7,528539062	5,492098715	0,578200484	0,197292009	0,167698208	0,046022179	0,025022179	**-1,234977821**
chrXII	462278	462439	161	0,467327936	0,296469566	0,61184795	0,284138687	0,241517884	0,223789437	0,104789437	**-1,155210563**
chrXII	462278	462446	168	0,789328282	0,837526525	0,48518668	-0,037140024	-0,031569021	0,087929701	-0,080070299	**-1,340070299**
chrXII	462278	462460	182	0,059840772	0,070411522	0,459422021	-0,101889932	-0,086606442	0,101125749	-0,164874251	**-1,424874251**
chrXII	462278	462461	183	0,054141651	0,062999783	0,462190442	-0,094916836	-0,080679311	-0,007534897	-0,280534897	**-1,540534897**
chrXII	462278	462479	201	0,045592969	0,048176305	0,486225044	-0,034535557	-0,029355224	-0,014067841	-0,413067841	**-1,673067841**
chrXII	462281	462425	144	0,037044288	0,103764348	0,263082498	-0,633871027	-0,538790373	-0,667457252	-0,667457252	**-1,927457252**
chrXII	462287	462423	136	0,14817715	0,122293696	0,54784888	0,120228376	0,10219412	0,080022787	0,136022787	**-1,123977213**
chrXII	462287	462425	138	0,125380666	0,196411088	0,389632936	-0,280275857	-0,238234478	-0,234089606	-0,192089606	**-1,452089606**
chrXII	462287	462432	145	0,082637257	0,059293913	0,582234733	0,207613792	0,176471723	0,007745309	0,000745309	**-1,259254691**
chrXII	462287	462439	152	0,094035499	0,222352175	0,297216064	-0,532424343	-0,452560692	-0,653162681	-0,709162681	**-1,969162681**
chrXII	462289	462377	88	0,105433742	0,200116957	0,345061366	-0,398688514	-0,338885237	0,096442296	0,488442296	**-0,771557704**
chrXII	462289	462407	118	0,159575393	0,240881523	0,398483299	-0,257274861	-0,218683631	0,166235083	0,348235083	**-0,911764917**
chrXII	462289	462423	134	1,877860425	1,793640876	0,511469361	0,028753386	0,024440378	0,032786959	0,102786959	**-1,157213041**
chrXII	462289	462424	135	0,253610892	0,340940001	0,42655876	-0,185142177	-0,157370851	-0,179744813	-0,116744813	**-1,376744813**
chrXII	462289	462425	136	0,720938828	0,726350437	0,498130433	-0,004686328	-0,003983379	-0,035319998	0,020680002	**-1,239319998**
chrXII	462289	462439	150	0,205168362	0,092646739	0,688911882	0,492768407	0,418853146	0,275027621	0,233027621	**-1,026972379**
chrXII	462290	462423	133	0,085486818	0,074117392	0,535617563	0,08939893	0,07598909	0,060835515	0,137835515	**-1,122164485**
chrXII	462290	462425	135	0,09973462	0,062999783	0,61286746	0,286800501	0,243780426	0,225207375	0,288207375	**-0,971792625**
chrXII	462296	462407	111	0,202318801	0,311293045	0,393913814	-0,26913262	-0,228762727	0,277333998	0,508333998	**-0,751666002**
chrXII	462296	462423	127	0,421734966	0,696703481	0,377074811	-0,313172484	-0,266196611	0,002781853	0,121781853	**-1,138218147**
chrXII	462296	462424	128	0,461628815	0,544762828	0,458696987	-0,10371695	-0,088159408	0,160240504	0,272240504	**-0,987759496**
chrXII	462296	462425	129	1,193965884	1,267407396	0,485081192	-0,037404627	-0,031793933	0,208376178	0,313376178	**-0,946623822**
chrXII	462296	462439	143	2,174214725	1,389701092	0,610063435	0,279484371	0,237561716	0,104254767	0,111254767	**-1,148745233**
chrXII	462296	462446	150	0,108283302	0,122293696	0,469618839	-0,076228037	-0,064793831	-0,209981275	-0,251981275	**-1,511981275**
chrXII	462296	462472	176	0,039893848	0,066705652	0,374240479	-0,320642986	-0,272546538	-0,100458547	-0,324458547	**-1,584458547**
chrXII	462296	462479	183	0,028495606	0,055588044	0,338895921	-0,415478239	-0,353156503	-0,298632027	-0,571632027	**-1,831632027**
chrXII	462298	462382	84	0,151026711	0,133411305	0,530965281	0,07769655	0,066042068	0,417229998	0,837229998	**-0,422770002**
chrXII	462298	462405	107	0,256460453	0,600350872	0,299319635	-0,52635832	-0,447404572	0,049307852	0,308307852	**-0,951692148**
chrXII	462298	462407	109	0,09688506	0,192705218	0,33455909	-0,427358568	-0,363254783	0,310184077	0,555184077	**-0,704815923**
chrXII	462298	462419	121	0,09973462	0,192705218	0,341043207	-0,409617695	-0,348175041	0,041438669	0,202438669	**-1,057561331**
chrXII	462298	462423	125	4,066322953	6,014626326	0,403367068	-0,244641361	-0,207945156	0,117844793	0,250844793	**-1,009155207**
chrXII	462298	462425	127	0,763682236	1,119172613	0,40559804	-0,238883294	-0,2030508	0,060173886	0,179173886	**-1,080826114**
chrXII	462298	462446	148	0,270708255	0,322410653	0,456414812	-0,109470114	-0,093049597	-0,261906994	-0,289906994	**-1,549906994**
chrXII	462298	462460	162	0,190920559	0,207528696	0,479159031	-0,052264347	-0,044424695	0,013619878	-0,112380122	**-1,372380122**
chrXII	462298	462461	163	0,079787696	0,096352609	0,452978074	-0,118140734	-0,100419624	-0,085055751	-0,218055751	**-1,478055751**
chrXII	462299	462406	107	0,139628469	0,188999349	0,424883291	-0,189416258	-0,161003819	0,350349386	0,609349386	**-0,650650614**
chrXII	462299	462423	124	0,119681545	0,218646305	0,353744289	-0,375231134	-0,318946464	0,071272192	0,211272192	**-1,048727808**
chrXII	462299	462425	126	0,162424953	0,307587175	0,345576089	-0,397291956	-0,337698162	-0,101184027	0,024815973	**-1,235184027**
chrXII	462299	462460	161	0,216566604	0,237175653	0,477289917	-0,056956516	-0,048413039	-0,063117698	-0,182117698	**-1,442117698**
chrXII	462299	462461	162	0,410336724	0,400233914	0,506231912	0,015621722	0,013278464	0,10353815	-0,02246185	**-1,28246185**
chrXII	462302	462424	122	0,074088575	0,129705435	0,363546382	-0,348995399	-0,296646089	0,098622083	0,252622083	**-1,007377917**
chrXII	462302	462425	123	0,14817715	0,281646088	0,344739737	-0,399561558	-0,339627325	0,070830501	0,217830501	**-1,042169499**
chrXII	462302	462439	137	0,028495606	0,055588044	0,338895921	-0,415478239	-0,353156503	-0,281231006	-0,232231006	**-1,492231006**
chrXII	462302	462479	177	0,262159574	0,259410871	0,502635026	0,006605078	0,005614317	0,154766359	-0,076233641	**-1,336233641**
chrXII	462308	462479	171	0,071239015	0,085235	0,455276965	-0,11233987	-0,09548889	0,038248195	-0,150751805	**-1,410751805**
chrXII	462317	462425	108	0,062690333	0,285351958	0,180122745	-0,914897409	-0,777662797	-0,152751066	0,099248934	**-1,160751066**
chrXII	462317	462477	160	0,074088575	0,151940653	0,327783163	-0,446042806	-0,379136385	-0,380379822	-0,492379822	**-1,752379822**
chrXII	462318	462407	89	0,051292091	0,244587392	0,173354671	-0,940991251	-0,799842563	-0,343961854	0,041038146	**-1,218961854**
chrXII	462318	462419	101	0,14532759	1,797346746	0,074808004	-1,440889127	-1,224755758	-0,747901076	-0,446901076	**-1,706901076**
chrXII	462318	462423	105	0,270708255	1,05617283	0,204018475	-0,827353109	-0,703250143	-0,146388482	0,126611518	**-1,133388482**
chrXII	462318	462425	107	0,247911771	1,108055004	0,182830269	-0,904631694	-0,76893694	-0,247235465	0,011764535	**-1,248235465**
chrXII	462318	462460	142	0,416035845	0,503998263	0,452196111	-0,120114784	-0,102097566	-0,089013962	-0,075013962	**-1,335013962**
chrXII	462318	462461	143	0,039893848	0,085235	0,318822147	-0,470995015	-0,400345763	-0,537923092	-0,530923092	**-1,790923092**
chrXII	462423	462569	146	0,53856695	0,181587609	0,747849115	0,667736558	0,567576075	0,401576342	0,387576342	**-0,872423658**
chrXII	462436	462577	141	13,86881136	8,549441118	0,618639271	0,301908968	0,256622623	0,210383747	0,231383747	**-1,028616253**
chrXII	462436	462597	161	0,059840772	0,074117392	0,446712395	-0,133971905	-0,113876119	-0,130804872	-0,249804872	**-1,509804872**
chrXII	462437	462547	110	0,031345166	0,211234566	0,129215933	-1,130105279	-0,960589487	-0,294457738	-0,056457738	**-1,316457738**
chrXII	462437	462577	140	0,039893848	0,070411522	0,361667325	-0,354005648	-0,300904801	-0,280851225	-0,252851225	**-1,512851225**
chrXII	462439	462577	138	0,062690333	0,092646739	0,403576119	-0,244101464	-0,207486244	-0,210489747	-0,168489747	**-1,428489747**
chrXII	462440	462577	137	0,039893848	0,066705652	0,374240479	-0,320642986	-0,272546538	-0,201360047	-0,152360047	**-1,412360047**
chrXII	462447	462577	130	0,14817715	0,118587827	0,555459536	0,139467258	0,118547169	0,237534996	0,335534996	**-0,924465004**
chrXII	462447	462597	150	0,139628469	0,111176087	0,556722218	0,142663981	0,121264384	-0,016042782	-0,058042782	**-1,318042782**
chrXII	462459	462618	159	0,156725832	0,144528914	0,520243529	0,050764798	0,043150079	0,008980311	-0,096019689	**-1,356019689**
chrXII	462595	462732	137	0,082637257	0,222352175	0,270951215	-0,609938679	-0,518447877	-0,440212947	-0,391212947	**-1,651212947**
chrXII	462595	462747	152	0,344796831	0,300175436	0,534591716	0,086817511	0,073794884	-0,123696431	-0,179696431	**-1,439696431**
chrXII	462595	462749	154	0,233663968	0,196411088	0,543309743	0,108775554	0,092459221	-0,085710209	-0,155710209	**-1,415710209**
chrXII	462597	462749	152	0,210867483	0,114881957	0,647330301	0,378122765	0,32140435	0,115276419	0,059276419	**-1,200723581**
chrXII	462624	462732	108	0,424584527	3,909692405	0,097959714	-1,293264983	-1,099275236	-0,462986452	-0,210986452	**-1,470986452**
chrXII	462624	462747	123	0,051292091	0,166764131	0,235224155	-0,721749813	-0,613487341	-0,194820989	-0,047820989	**-1,307820989**
chrXII	462624	462749	125	0,065539893	0,218646305	0,230623069	-0,736796456	-0,626276987	-0,302388607	-0,169388607	**-1,429388607**
chrXII	462637	462789	152	0,416035845	0,140823044	0,747111797	0,665428585	0,565614298	0,355941276	0,299941276	**-0,960058724**

Chrom	Start	End	Length	Section A	Section B	A/A+B	Z-score	Z * 0.85	Phase correction	Length correction	ΔLKnuc
chrXII	462711	462864	153	0,119681545	0,062999783	0,655138356	0,399230614	0,339346022	0,209736798	0,146736798	-1,113263202
chrXII	462724	462874	150	0,051292091	0,051882174	0,497140355	-0,007168129	-0,00609291	-0,130615997	-0,172615997	-1,432615997
chrXII	462764	462913	149	0,056991212	0,107470218	0,346532387	-0,394699362	-0,335494458	-0,512936083	-0,547936083	-1,807936083
chrXII	462764	462916	152	1,330744792	0,589233263	0,693104168	0,504668535	0,428968255	0,223057022	0,167057022	-1,092942978
chrXII	462794	462951	157	0,230814407	0,077823261	0,747849115	0,667736558	0,567576075	0,474221815	0,383221815	-0,876778185
chrXII	462796	462913	117	1,114178188	5,18451154	0,17689047	-0,927280453	-0,788188385	-0,25478172	-0,06578172	-1,32578172
chrXII	462796	462916	120	0,059840772	0,240881523	0,198990142	-0,845233853	-0,718448775	-0,320727582	-0,152727582	-1,412727582
chrXII	462871	462990	119	0,367593315	0,982055438	0,272362209	-0,605684292	-0,514831648	-0,074231333	0,100768667	-1,159231333
chrXII	462885	463047	162	0,04844253	0,155646522	0,237359767	-0,714821199	-0,607598019	-0,521815123	-0,647815123	-1,907815123
chrXII	462885	463050	165	3,214304338	3,524281969	0,476999803	-0,057684921	-0,049032183	0,046955711	-0,100044289	-1,360044289
chrXII	462885	463056	171	0,675345858	0,781938481	0,463427651	-0,091802067	-0,078031757	0,046291972	-0,142708028	-1,402708028
chrXII	462885	463059	174	0,056991212	0,070411522	0,447331152	-0,132407093	-0,112546029	0,124494291	-0,085505709	-1,345505709
chrXII	462885	463099	214	0,153876272	0,133411305	0,535617563	0,08939893	0,07598909	0,361860225	-0,128139775	-1,388139775
chrXII	462889	462996	107	0,276407377	0,255705001	0,519453011	0,048780806	0,041463685	0,580701873	0,839701873	-0,420298127
chrXII	462889	463045	156	0,766531797	0,96352609	0,443067138	-0,143197409	-0,121717797	-0,168219097	-0,252219097	-1,512219097
chrXII	462889	463048	159	0,612655525	0,641115437	0,488650275	-0,028453381	-0,024185374	-0,050230859	-0,155230859	-1,415230859
chrXII	462889	463051	162	1,062886098	1,1451137	0,481379617	-0,046691337	-0,039687637	0,033322019	-0,092677981	-1,352677981
chrXII	462889	463053	164	1,997541969	1,804758485	0,525350901	0,063588112	0,054049895	0,051474831	-0,088525169	-1,348525169
chrXII	462889	463059	170	2,613047055	3,25004762	0,445677104	-0,136590852	-0,116102224	0,060058218	-0,121941782	-1,381941782
chrXII	462889	463061	172	0,34194727	0,292763697	0,538744859	0,097272137	0,082681316	0,200074826	0,004074826	-1,255925174
chrXII	462889	463066	177	0,031345166	0,040764565	0,434687048	-0,164453569	-0,139785534	0,010644009	-0,220355991	-1,480355991
chrXII	462890	463050	160	0,068389454	0,040764565	0,626540868	0,322705529	0,274299699	0,277400943	0,165400943	-1,094599057
chrXII	462890	463059	169	0,079787696	0,085235	0,483495289	-0,041382985	-0,035175537	0,116437209	-0,058562791	-1,318562791
chrXII	462891	463047	156	0,151026711	0,277940218	0,352070756	-0,379735842	-0,322775466	-0,371156071	-0,455156071	-1,715156071
chrXII	462891	463056	165	0,039893848	0,08894087	0,309651379	-0,496838761	-0,422312947	-0,323680222	-0,470680222	-1,730680222
chrXII	462891	463061	170	0,045592969	0,070411522	0,393027622	-0,271436616	-0,230721123	-0,054767484	-0,236767484	-1,496767484
chrXII	462911	463047	136	0,09688506	0,107470218	0,47410109	-0,064964608	-0,055219916	-0,084132838	-0,028132838	-1,288132838
chrXII	462911	463050	139	0,729487509	0,752291524	0,49230519	-0,019289223	-0,01639584	-0,048277334	-0,013277334	-1,273277334
chrXII	462911	463051	140	0,062690333	0,17417587	0,264665588	-0,629027313	-0,534673216	-0,515682671	-0,487682671	-1,747682671
chrXII	462911	463053	142	0,068389454	0,081529131	0,456177292	-0,110069087	-0,093558724	-0,080359236	-0,066359236	-1,326359236
chrXII	462911	463056	145	9,736948514	5,440216541	0,641552522	0,362611717	0,308219959	0,117105004	0,110105004	-1,149894996
chrXII	462911	463062	151	0,113982423	0,092646739	0,551627959	0,129775456	0,110309137	-0,02235686	-0,07135686	-1,33135686
chrXII	462911	463099	188	0,521469587	0,392822175	0,570353588	0,177274443	0,150683276	0,170846327	-0,137153673	-1,397153673
chrXII	462912	463045	133	0,769381358	0,915349786	0,456679014	-0,108803903	-0,092483317	-0,09433178	-0,01733178	-1,27733178
chrXII	462912	463048	136	0,14817715	0,192705218	0,434687048	-0,164453569	-0,139785534	-0,162103625	-0,106103625	-1,366103625
chrXII	462912	463051	139	7,719459621	5,421687193	0,587426633	0,220930261	0,187790722	0,157951021	0,192951021	-1,067048979
chrXII	462912	463053	141	0,29350474	0,255705001	0,534412845	0,086367464	0,073412344	0,040365307	0,061365307	-1,198634693
chrXII	462912	463059	147	0,527168708	0,544762828	0,491793263	-0,02057269	-0,017486786	-0,139418388	-0,160418388	-1,420418388
chrXII	462918	463045	127	5,964130301	6,559389154	0,476234362	-0,059606898	-0,050665863	0,212300505	0,331300505	-0,928699495
chrXII	462918	463048	130	0,085486818	0,111176087	0,434687048	-0,164453569	-0,139785534	-0,027890733	0,070109267	-1,189890733
chrXII	462918	463051	133	0,866266417	0,785644351	0,52440267	0,061206617	0,052025624	0,053904986	0,130904986	-1,129095014
chrXII	462918	463053	135	0,287805619	0,277940218	0,508718933	0,021856865	0,018578335	-0,003000469	0,059999531	-1,200000469
chrXII	462918	463059	141	0,464478375	0,52623348	0,468832964	-0,078203815	-0,066473243	-0,093478689	-0,072478689	-1,332478689
chrXII	462919	463048	129	0,142478029	0,218646305	0,394540095	-0,267505227	-0,227379443	0,003586197	0,108586197	-1,151413803
chrXII	462919	463050	131	0,344796831	0,289057827	0,543968284	0,110436217	0,093870785	0,147999292	0,238999292	-1,021000708
chrXII	462919	463051	132	0,071239015	0,118587827	0,375284202	-0,317889966	-0,270206471	-0,127583622	-0,043583622	-1,303583622
chrXII	462919	463056	137	0,156725832	0,159352392	0,495845079	-0,010415029	-0,008852775	0,077813954	0,126813954	-1,133186046
chrXII	462919	463059	140	0,056991212	0,096352609	0,371656395	-0,327469522	-0,278349094	-0,237927096	-0,209927096	-1,469927096
chrXII	462919	463062	143	0,253610892	0,137117174	0,649072626	0,382817954	0,325395261	0,183052955	0,190052955	-1,069947045
chrXII	462919	463066	147	0,188070999	0,166764131	0,530023616	0,075329227	0,064029843	-0,067886903	-0,088886903	-1,348886903
chrXII	462919	463074	155	0,068389454	0,100058479	0,405997586	-0,23785292	-0,202174982	-0,299297045	-0,376297045	-1,636297045
chrXII	462919	463099	180	0,071239015	0,051882174	0,578608892	0,198335964	0,168585569	0,339219731	0,087219731	-1,172780269
chrXII	462920	463045	125	0,028495606	0,040764565	0,411428464	-0,223871969	-0,190291174	0,148708774	0,281708774	-0,978291226
chrXII	462920	463048	128	0,059840772	0,111176087	0,349911537	-0,385559308	-0,327725412	-0,089160695	0,022839305	-1,237160695
chrXII	462920	463050	130	0,045592969	0,070411522	0,393027622	-0,271436616	-0,230721123	-0,112710878	-0,014710878	-1,274710878
chrXII	462920	463051	131	0,062690333	0,122293696	0,338895921	-0,415478239	-0,353156503	-0,30107321	-0,21007321	-1,47007321
chrXII	462949	463050	101	0,151026711	0,248293262	0,37820976	-0,310185996	-0,263658096	0,20979647	0,51079647	-0,74920353
chrXII	462949	463056	107	0,464478375	0,444704349	0,510874616	0,027261996	0,023172697	0,574204694	0,833204694	-0,426795306
chrXII	462949	463099	150	2,746976403	1,504583049	0,646110312	0,374840117	0,3186141	0,19581757	0,15381757	-1,10618243
chrXII	462949	463138	189	0,125380666	0,08894087	0,585011979	0,214732295	0,182522451	0,236241452	-0,078758548	-1,338758548
chrXII	462949	463138	189	0,125380666	0,08894087	0,585011979	0,214732295	0,182522451	0,236241452	-0,078758548	-1,338758548
chrXII	462988	463095	107	0,170973635	0,203822827	0,456177292	-0,110069087	-0,093558724	0,45883822	0,71783822	-0,54216178
chrXII	462988	463099	111	0,09688506	0,133411305	0,420697304	-0,200109928	-0,170093439	0,315641974	0,546641974	-0,713358026
chrXII	462988	463138	150	1,427629852	0,841232394	0,629227206	0,329807278	0,280336186	0,170432647	0,128432647	-1,131567353
chrXII	462988	463138	150	1,427629852	0,841232394	0,629227206	0,329807278	0,280336186	0,170432647	0,128432647	-1,131567353
chrXII	463046	463209	163	0,094035499	0,148234783	0,388142938	-0,284162468	-0,241538098	-0,229772572	-0,362772572	-1,622772572
chrXII	463055	463217	162	0,227964847	0,26311674	0,464209722	-0,089833605	-0,076358564	0,006980548	-0,119019452	-1,379019452
chrXII	463059	463216	157	0,681044979	0,496586524	0,578317562	0,197591256	0,167952568	0,085930515	-0,005069485	-1,265069485
chrXII	463059	463234	175	0,039893848	0,051882174	0,434687048	-0,164453569	-0,139785534	0,061637422	-0,155362578	-1,415362578
chrXII	463066	463216	150	0,037044288	0,103764348	0,263082498	-0,633871027	-0,538790373	-0,651700408	-0,693700408	-1,953700408
chrXII	463066	463234	168	0,772230918	1,096937395	0,413141456	-0,219471329	-0,18655063	-0,064294796	-0,232294796	-1,492294796
chrXII	463066	463245	179	0,085486818	0,144528914	0,371656395	-0,327469522	-0,278349094	-0,116560895	-0,361560895	-1,621560895
chrXII	463096	463234	138	0,059840772	0,111176087	0,349911537	-0,385559308	-0,327725412	-0,326826372	-0,284826372	-1,544826372
chrXII	463096	463245	149	0,467327936	0,277940218	0,627060117	0,324076964	0,27546542	0,105003009	0,070003009	-1,189996991
chrXII	463096	463249	153	0,151026711	0,085235	0,639234814	0,356414232	0,302952097	0,173869539	0,110869539	-1,149130461
chrXII	463097	463253	156	0,085486818	0,062999783	0,575720754	0,190958009	0,162314307	0,105933737	0,021933737	-1,238066263
chrXII	463101	463234	133	0,108283302	0,081529131	0,570475287	0,177584338	0,150946687	0,149857161	0,226857161	-1,033142839
chrXII	463101	463245	144	0,461628815	0,370586958	0,554698469	0,137541158	0,116909984	0,010841464	0,010841464	-1,249158536
chrXII	463108	463245	137	0,062690333	0,048176305	0,56545715	0,164819952	0,140096959	0,224351152	0,273351152	-0,986648848
chrXII	463108	463260	152	0,139628469	0,077823261	0,642112476	0,3641111	0,309494435	0,113817293	0,057817293	-1,202182707
chrXII	463196	463353	157	0,062690333	0,044470435	0,585011979	0,214732295	0,182522451	0,104754633	0,013754633	-1,246245367
chrXII	463248	463405	157	0,094035499	0,107470218	0,466664175	-0,083658001	-0,071109301	-0,14445714	-0,23545714	-1,49545714
chrXII	463257	463404	147	0,065539893	0,059293913	0,525017182	0,062749932	0,053337442	-0,075702689	-0,096702689	-1,356702689
chrXII	463257	463414	157	0,09688506	0,096352609	0,50137771	0,003453413	0,002935401	-0,072163998	-0,163163998	-1,423163998
chrXII	463261	463414	153	0,173823196	0,244587392	0,415436895	-0,213581034	-0,181543879	-0,305062193	-0,368062193	-1,628062193
chrXII	463268	463414	146	0,208017923	0,140823044	0,596311622	0,243811574	0,207239838	0,044929881	0,030929881	-1,229070119
chrXII	463278	463414	136	0,233663968	0,281646088	0,453443447	-0,11696613	-0,099421211	-0,111356249	-0,055356249	-1,315356249
chrXII	463333	463483	150	0,119681545	0,077823261	0,605967759	0,268824834	0,228501109	0,117073577	0,075073577	-1,184926423
chrXII	463333	463483	150	0,119681545	0,077823261	0,605967759	0,268824834	0,228501109	0,117073577	0,075073577	-1,184926423
chrXII	463358	463511	153	0,031345166	0,066705652	0,319682862	-0,468585806	-0,398297935	-0,521061972	-0,584061972	-1,844061972
chrXII	463358	463529	171	0,492973981	0,426175002	0,536337406	0,091210681	0,077529079	0,194017747	0,005017747	-1,254982253
chrXII	463381	463529	148	0,065539893	0,037058696	0,638799168	0,355250862	0,301963233	0,122758989	0,094758989	-1,165241011
chrXII	463381	463539	158	0,364743755	0,311293045	0,539532397	0,099255756	0,084367393	0,054257432	-0,043742568	-1,303742568
chrXII	463385	463511	126	0,455929693	0,652233046	0,411428464	-0,223871969	-0,190291174	0,048920659	0,174920659	-1,085079341
chrXII	463385	463529	144	0,037044288	0,037058696	0,499902783	-0,000243688	-0,000207135	-0,122706278	-0,122706278	-1,382706278
chrXII	463385	463535	150	0,384690679	0,296469566	0,564757972	0,163043664	0,138587115	0,017909114	-0,024090886	-1,284090886

Chrom	Start	End	Length	Section A	Section B	A/A+B	Z-score	Z * 0.85	Phase correction	Length correction	ΔLKnuc
chrXII	463385	463539	154	0,028495606	0,070411522	0,288104674	-0,558930214	-0,475090682	-0,651963002	-0,721963002	**-1,981963002**
chrXII	463410	463573	163	0,381841118	0,359469349	0,515089338	0,037832385	0,032157528	0,046536916	-0,086463084	**-1,346463084**
chrXII	463414	463521	107	0,037044288	0,055588044	0,399906675	-0,253588671	-0,21555037	0,361423191	0,620423191	**-0,639576809**
chrXII	463414	463535	121	0,039893848	0,040764565	0,49460244	-0,013530088	-0,011500575	0,382865359	0,543865359	**-0,716134641**
chrXII	463414	463539	125	0,031345166	0,114881957	0,214359455	-0,791385773	-0,672677907	-0,339607688	-0,206607688	**-1,466607688**
chrXII	463414	463575	161	0,339097709	0,211234566	0,616169039	0,295434579	0,251119392	0,241877373	0,122877373	**-1,137122627**
chrXII	463414	463583	169	0,980248841	1,49717131	0,395673233	-0,264562559	-0,224878175	-0,064229188	-0,239229188	**-1,499229188**
chrXII	463463	463603	140	0,054141651	0,059293913	0,477289917	-0,056956516	-0,048413039	0,006892165	0,034892165	**-1,225107835**
chrXII	463471	463608	137	0,128230226	0,289057827	0,307294266	-0,503534497	-0,428004322	-0,351700146	-0,302700146	**-1,562700146**
chrXII	463535	463688	153	0,062690333	0,077823261	0,446151373	-0,135390993	-0,115082344	-0,232538451	-0,295538451	**-1,555538451**
chrXII	463535	463689	154	5,189049823	3,098106968	0,626155623	0,321688416	0,273435154	0,105846384	0,035846384	**-1,224153616**
chrXII	463535	463690	155	0,054141651	0,062999783	0,462190442	-0,094916836	-0,080679311	-0,175024304	-0,252024304	**-1,512024304**
chrXII	463535	463692	157	0,19661968	0,133411305	0,595761274	0,242390685	0,206032083	0,136886211	0,045886211	**-1,214113789**
chrXII	463535	463704	169	0,159575393	0,151940653	0,512254168	0,030721476	0,026113255	0,191529162	0,016529162	**-1,243470838**
chrXII	463535	463710	175	1,077133901	1,826993702	0,37089758	-0,32947702	-0,280055467	-0,074768098	-0,291768098	**-1,551768098**
chrXII	463535	463712	177	0,076938136	0,074117392	0,509336779	0,023405971	0,019895076	0,167101448	-0,063898552	**-1,323898552**
chrXII	463541	463689	148	0,322000346	0,203822827	0,612373822	0,28551142	0,242684707	0,060246552	0,032246552	**-1,227753448**
chrXII	463541	463692	151	0,994496644	0,511410002	0,660397274	0,413547605	0,351515464	0,211094203	0,162094203	**-1,097905797**
chrXII	463559	463689	130	0,407487163	0,248293262	0,621377442	0,309100452	0,262735385	0,384086819	0,482086819	**-0,777913181**
chrXII	463559	463704	145	1,185417203	0,633703698	0,651642891	0,389759738	0,331295778	0,160461549	0,153461549	**-1,106538451**
chrXII	463559	463710	151	0,094035499	0,096352609	0,493914773	-0,015253994	-0,012965895	-0,169491197	-0,218491197	**-1,478491197**
chrXII	463559	463712	153	0,663947616	0,392822175	0,628280276	0,327302042	0,278206735	0,164665235	0,101665235	**-1,158334765**
chrXII	463658	463815	157	0,629752889	0,552174567	0,532818563	0,082356944	0,070003402	0,011918877	-0,079081123	**-1,339081123**
chrXII	463658	463852	194	0,028495606	0,040764565	0,411428464	-0,223871969	-0,190291174	-0,121037165	-0,471037165	**-1,731037165**
chrXII	463658	463870	212	0,037044288	0,037058696	0,499902783	-0,000243688	-0,000207135	0,20878783	-0,26721217	**-1,52721217**
chrXII	463690	463849	159	0,615505086	0,500292393	0,551627959	0,129775456	0,110309137	0,080116289	-0,024883711	**-1,284883711**
chrXII	463690	463870	180	0,071239015	0,103764348	0,407072261	-0,235082731	-0,199820321	-0,010182911	-0,262182911	**-1,522182911**
chrXII	463692	463840	148	0,760832676	0,463233697	0,621561618	0,309584739	0,263147028	0,082629095	0,054629095	**-1,205370905**
chrXII	463697	463815	118	0,04844253	0,062999783	0,434687048	-0,164453569	-0,139785534	0,246592139	0,428592139	**-0,831407861**
chrXII	463697	463849	152	0,384690679	0,307587175	0,555688264	0,14004622	0,119039287	-0,071924403	-0,127924403	**-1,387924403**
chrXII	463697	463852	155	1,108479067	0,896820438	0,552774817	0,132675062	0,112773803	0,018093444	-0,058906556	**-1,318906556**
chrXII	463697	463861	164	0,088336378	0,066705652	0,569757619	0,175757115	0,149393547	0,140565794	0,000565794	**-1,259434206**
chrXII	463697	463870	173	0,170973635	0,192705218	0,470122564	-0,074961771	-0,063717505	0,128162034	-0,074837966	**-1,334837966**
chrXII	463699	463849	150	0,199469241	0,103764348	0,657807209	0,406485945	0,345513053	0,213749184	0,171749184	**-1,088250816**
chrXII	463707	463849	142	0,085486818	0,062999783	0,575720754	0,190958009	0,162314307	0,174465328	0,188465328	**-1,071534672**
chrXII	463707	463852	145	0,054141651	0,062999783	0,462190442	-0,094916836	-0,080679311	-0,260546399	-0,267546399	**-1,527546399**
chrXII	463707	463861	154	0,800726524	0,489174784	0,620765727	0,307492481	0,261368609	0,106308139	0,036308139	**-1,223691861**
chrXII	463707	463870	163	0,139628469	0,103764348	0,573675388	0,185739279	0,157878387	0,14247458	0,00947458	**-1,25052542**
chrXII	463713	463849	136	0,131079787	0,137117174	0,488744489	-0,028217126	-0,023984557	-0,029972975	0,026027025	**-1,233972975**
chrXII	463713	463852	139	0,085486818	0,125999566	0,404219015	-0,242441566	-0,206075331	-0,240489107	-0,205489107	**-1,465489107**
chrXII	463713	463861	148	0,133929347	0,111176087	0,54641525	0,116609512	0,099118085	-0,079426781	-0,107426781	**-1,367426781**
chrXII	463713	463870	157	1,963347242	1,608347397	0,549696276	0,124894026	0,106159922	0,04457675	-0,04642325	**-1,30642325**
chrXII	463713	463909	196	0,062690333	0,051882174	0,547167331	0,118507771	0,100731606	0,141226838	-0,222773162	**-1,482773162**
chrXII	463724	463883	159	0,116831984	0,066705652	0,636556002	0,349268164	0,296877939	0,271389819	0,166389819	**-1,093610181**
chrXII	463729	463879	150	0,151026711	0,133411305	0,530965281	0,07769655	0,066042068	-0,055274644	-0,097274644	**-1,357274644**
chrXII	463763	463849	86	0,039893848	0,037058696	0,518421434	0,046192108	0,039263292	0,412842017	0,818842017	**-0,441157983**
chrXII	463763	463870	107	0,113982423	0,148234783	0,434687048	-0,164453569	-0,139785534	0,424999413	0,683999413	**-0,576000587**
chrXII	463763	463909	146	1,077133901	0,689291742	0,609781626	0,278749922	0,236937434	0,075629071	0,061629071	**-1,198370929**
chrXII	463860	463991	131	0,296354301	0,318704784	0,481830621	-0,045559635	-0,03872569	0,022539486	0,113539486	**-1,146460514**
chrXII	463862	464000	138	1,031540931	0,778232612	0,56998943	0,176331979	0,149882182	0,153942811	0,195942811	**-1,064057189**
chrXII	463862	464023	161	0,068389454	0,044470435	0,605967759	0,268824834	0,228501109	0,227137764	0,108137764	**-1,151862236**
chrXII	463870	464023	153	0,205168362	0,218646305	0,484099249	-0,039867831	-0,033887657	-0,158560325	-0,221560325	**-1,481560325**
chrXII	463878	464033	155	0,054141651	0,044470435	0,549036667	0,123227859	0,104743681	0,017045594	-0,059954406	**-1,319954406**
chrXII	463884	464023	139	0,361894194	0,244587392	0,596710934	0,244842828	0,208116404	0,170413131	0,205413131	**-1,054586869**
chrXII	463974	464128	154	0,24506221	0,070411522	0,776807021	0,761453978	0,647235881	0,492674682	0,422674682	**-0,837325318**
chrXII	463974	464128	154	0,24506221	0,070411522	0,776807021	0,761453978	0,647235881	0,492674682	0,422674682	**-0,837325318**
chrXII	464001	464158	157	0,159575393	0,100058479	0,614617006	0,291373097	0,247667133	0,186177044	0,095177044	**-1,164822956**
chrXII	464013	464160	147	0,729487509	0,43729261	0,625214209	0,319204319	0,271323671	0,142872019	0,121872019	**-1,138127981**
chrXII	464013	464166	153	0,128230226	0,200116957	0,390532439	-0,277931585	-0,236241848	-0,355143929	-0,418143929	**-1,678143929**
chrXII	464038	464166	128	0,119681545	0,248293262	0,325243855	-0,453084759	-0,385122045	-0,143466261	-0,031466261	**-1,291466261**
chrXII	464038	464211	173	0,202318801	0,292763697	0,408656743	-0,231001693	-0,196351439	0,003503322	-0,199496678	**-1,459496678**
chrXII	464057	464224	167	0,091185939	0,129705435	0,412808961	-0,220325165	-0,18727639	-0,050343759	-0,211343759	**-1,471343759**
chrXII	464069	464210	141	0,649699813	0,429880871	0,601807556	0,258028531	0,219324251	0,190157889	0,211157889	**-1,048842111**
chrXII	464070	464160	90	0,185221438	0,359469349	0,340048781	-0,412330002	-0,350480502	0,096112046	0,474112046	**-0,785887954**
chrXII	464070	464166	96	5,821652272	28,08678554	0,171687422	-0,947518087	-0,805390374	-0,234013946	0,101986054	**-1,158013946**
chrXII	464070	464210	140	0,042743409	0,040764565	0,51184823	0,029703476	0,025247955	0,088256082	0,116256082	**-1,143743918**
chrXII	464070	464211	141	0,236513528	0,248293262	0,487851105	-0,030457471	-0,025888351	-0,063584909	-0,042584909	**-1,302584909**
chrXII	464070	464213	143	0,624053768	0,452116089	0,579884081	0,201596939	0,171357398	0,026326478	0,033326478	**-1,226673522**
chrXII	464101	464269	168	0,227964847	0,207528696	0,523463207	0,058847485	0,050020363	0,176699056	0,008699056	**-1,251300944**
chrXII	464101	464269	168	0,227964847	0,207528696	0,523463207	0,058847485	0,050020363	0,176699056	0,008699056	**-1,251300944**
chrXII	464132	464301	169	0,139628469	0,281646088	0,331442919	-0,435932317	-0,370542469	-0,214425781	-0,389425781	**-1,649425781**
chrXII	464132	464301	169	0,139628469	0,281646088	0,331442919	-0,435932317	-0,370542469	-0,214425781	-0,389425781	**-1,649425781**
chrXII	464137	464269	132	0,111132863	0,118587827	0,483773852	-0,040684142	-0,03458152	0,108072332	0,192072332	**-1,067927668**
chrXII	464137	464269	132	0,111132863	0,118587827	0,483773852	-0,040684142	-0,03458152	0,108072332	0,192072332	**-1,067927668**
chrXII	464152	464303	151	0,498673102	0,307587175	0,61850139	0,301547257	0,256315168	0,091828708	0,042828708	**-1,217171292**
chrXII	464195	464345	150	0,037044288	0,059293913	0,384523349	-0,293622086	-0,249578773	-0,369626346	-0,411626346	**-1,671626346**
chrXII	464205	464360	155	0,153876272	0,111176087	0,58055047	0,203301913	0,172806626	0,089422092	0,012422092	**-1,247577908**
chrXII	464232	464406	174	0,102584181	0,144528914	0,415130494	-0,214366854	-0,182211826	0,051618736	-0,158381264	**-1,418381264**
chrXII	464232	464406	174	0,102584181	0,144528914	0,415130494	-0,214366854	-0,182211826	0,051618736	-0,158381264	**-1,418381264**
chrXII	464233	464360	127	0,034194727	0,074117392	0,315705458	-0,47974192	-0,407780632	-0,158187093	-0,039187093	**-1,299187093**
chrXII	464233	464377	144	0,994496644	0,604056741	0,622122885	0,311061025	0,264401871	0,140105817	0,140105817	**-1,119894183**
chrXII	464233	464380	147	0,136778908	0,077823261	0,637360324	0,351411905	0,298700119	0,161150912	0,140150912	**-1,119849088**
chrXII	464233	464382	149	0,994496644	0,481763045	0,67365969	0,450041356	0,382535153	0,201669305	0,166669305	**-1,093330695**
chrXII	464233	464386	153	0,059840772	0,040764565	0,594807131	0,239928434	0,203939169	0,082924825	0,019924825	**-1,240075175**
chrXII	464234	464377	143	0,045592969	0,062999783	0,419852784	-0,202270107	-0,171929591	-0,329792225	-0,322792225	**-1,582792225**
chrXII	464234	464380	146	0,450230572	0,333528262	0,574450395	0,187716099	0,159558684	-0,00563756	-0,01963756	**-1,27963756**
chrXII	464234	464382	148	0,065539893	0,044470435	0,595761274	0,242390685	0,206032083	0,036630998	0,008630998	**-1,251369002**
chrXII	464235	464377	142	0,042743409	0,040764565	0,51184823	0,029703476	0,025247955	0,020116824	0,034116824	**-1,225883176**
chrXII	464235	464380	145	0,042743409	0,040764565	0,51184823	0,029703476	0,025247955	-0,136622677	-0,143622677	**-1,403622677**
chrXII	464235	464382	147	0,031345166	0,037058696	0,458236793	-0,104876779	-0,089145262	-0,22202983	-0,24302983	**-1,50302983**
chrXII	464235	464386	151	0,227964847	0,118587827	0,657807209	0,406485945	0,345513053	0,172357329	0,123357329	**-1,136642671**
chrXII	464266	464430	164	0,04844253	0,037058696	0,566571175	0,16765125	0,142503563	0,131004069	-0,008995931	**-1,268995931**
chrXII	464266	464430	164	0,04844253	0,037058696	0,566571175	0,16765125	0,142503563	0,131004069	-0,008995931	**-1,268995931**
chrXII	464275	464426	151	0,079787696	0,059293913	0,573675388	0,185739279	0,157878387	-0,014866282	-0,063866282	**-1,323866282**
chrXII	464275	464426	151	0,079787696	0,059293913	0,573675388	0,185739279	0,157878387	-0,014866282	-0,063866282	**-1,323866282**
chrXII	464319	464475	156	0,034194727	0,059293913	0,365763444	-0,34309514	-0,291630869	-0,345774551	-0,429774551	**-1,689774551**

Chrom	Start	End	Length	Section A	Section B	A/A+B	Z-score	Z * 0.85	Phase correction	Length correction	ΔLKnuc
chrXII	464354	464503	149	0,045592969	0,044470435	0,506231912	0,015621722	0,013278464	-0,164627491	-0,199627491	**-1,459627491**
chrXII	464358	464532	174	0,045592969	0,129705435	0,26008776	-0,64307487	-0,54661364	-0,304508688	-0,514508688	**-1,774508688**
chrXII	464384	464518	134	0,068389454	0,163058261	0,295485544	-0,537429332	-0,456814932	-0,445147555	-0,375147555	**-1,635147555**
chrXII	464391	464538	147	0,153876272	0,148234783	0,509336779	0,023405971	0,019895076	-0,121020385	-0,142020385	**-1,402020385**
chrXII	464392	464537	145	0,051292091	0,074117392	0,408996909	-0,230126057	-0,195607148	-0,363022925	-0,370022925	**-1,630022925**
chrXII	464415	464560	145	0,059840772	0,051882174	0,535617563	0,08939893	0,07598909	-0,093717059	-0,100717059	**-1,360717059**
chrXII	464434	464584	150	0,367593315	0,281646088	0,566190705	0,166684133	0,141681513	0,021782757	-0,020217243	**-1,280217243**
chrXII	464504	464649	145	1,473222822	0,800467829	0,647943387	0,379773944	0,322807852	0,165095809	0,158095809	**-1,101904191**
chrXII	464504	464668	164	0,108283302	0,066705652	0,618800783	0,302332726	0,256982817	0,269489425	0,129489425	**-1,130510575**
chrXII	464504	464686	182	0,19377012	0,185293479	0,511181027	0,028030349	0,023825797	0,211022954	-0,054977046	**-1,314977046**
chrXII	464514	464649	135	0,076938136	0,066705652	0,535617563	0,08939893	0,07598909	0,046761185	0,109761185	**-1,150238815**
chrXII	464514	464668	154	0,547115632	0,307587175	0,640123827	0,358789798	0,304971328	0,154200067	0,084200067	**-1,175799933**
chrXII	464514	464686	172	0,105433742	0,081529131	0,563928764	0,160937697	0,136797043	0,253973888	0,057973888	**-1,202026112**
chrXII	464534	464640	106	0,076938136	0,118587827	0,393493196	-0,270226	-0,2296921	0,283811914	0,549811914	**-0,710188086**
chrXII	464534	464649	115	0,09973462	0,100058479	0,499189516	-0,002031583	-0,001726846	0,604326493	0,807326493	**-0,452673507**
chrXII	464534	464668	134	0,125380666	0,044470435	0,738179884	0,637744162	0,542082538	0,556066499	0,626066499	**-0,633933501**
chrXII	464534	464686	152	1,780975365	1,348936527	0,569017732	0,173873936	0,147792846	-0,023918936	-0,079918936	**-1,339918936**
chrXII	464547	464649	102	0,068389454	0,074117392	0,479902939	-0,050397187	-0,042837609	0,490360132	0,784360132	**-0,475639868**
chrXII	464547	464686	139	0,125380666	0,17788174	0,413439528	-0,218706025	-0,185900121	-0,186086837	-0,151086837	**-1,411086837**
chrXII	464547	464697	150	0,168124074	0,037058696	0,819386902	0,913031079	0,776076417	0,653505093	0,611505093	**-0,648494907**
chrXII	464547	464705	158	0,530018269	0,366881088	0,590945087	0,229976764	0,195480249	0,169376284	0,071376284	**-1,188623716**
chrXII	464547	464706	159	0,279256937	0,333528262	0,455717497	-0,111228699	-0,094544395	-0,114829738	-0,219829738	**-1,479829738**
chrXII	464553	464708	155	0,131079787	0,100058479	0,567105523	0,169009772	0,143658307	0,069140507	-0,007859493	**-1,267859493**
chrXII	464559	464706	147	1,042939174	0,489174784	0,68071906	0,46971048	0,399253908	0,254471556	0,233471556	**-1,026528444**
chrXII	464728	464888	160	0,045592969	0,096352609	0,321200349	-0,464344846	-0,394693119	-0,385799016	-0,497799016	**-1,757799016**
chrXII	464838	465007	169	0,059840772	0,122293696	0,328552705	-0,443913104	-0,377326138	-0,21329069	-0,38829069	**-1,64829069**
chrXII	464884	465032	148	0,795027403	0,667056524	0,543763178	0,109918963	0,093431118	-0,070902718	-0,098902718	**-1,358902718**
chrXII	464886	465026	140	0,324849907	0,26682261	0,549036667	0,123227859	0,104743681	0,16863814	0,19663814	**-1,06336186**
chrXII	464920	465095	175	0,031345166	0,085235	0,268872204	-0,616227458	-0,523793339	-0,31408638	-0,53108638	**-1,79108638**
chrXII	465006	465158	152	0,082637257	0,070411522	0,539940648	0,100284197	0,085241567	-0,083748791	-0,139748791	**-1,399748791**
chrXII	465040	465204	164	0,056991212	0,100058479	0,362886495	-0,350753893	-0,298140809	-0,288477357	-0,428477357	**-1,688477357**
chrXII	465055	465176	121	0,256460453	0,922761525	0,217482762	-0,780722841	-0,663614415	-0,251886964	-0,090886964	**-1,350886964**
chrXII	465063	465223	160	0,276407377	0,26311674	0,512316999	0,030879046	0,026247189	0,02962321	-0,08237679	**-1,34237679**
chrXII	465159	465310	151	2,105825271	1,252583918	0,627030583	0,323998943	0,275399102	0,096523512	0,047523512	**-1,212476488**
chrXII	465159	465342	183	0,230814407	0,170470001	0,575189075	0,18960094	0,161160799	0,213304016	-0,059695984	**-1,319695984**
chrXII	465159	465342	183	0,230814407	0,170470001	0,575189075	0,18960094	0,161160799	0,213304016	-0,059695984	**-1,319695984**
chrXII	465159	465348	189	0,091185939	0,070411522	0,564278289	0,16182531	0,137551513	0,179653649	-0,135346351	**-1,395346351**
chrXII	465159	465348	189	0,091185939	0,070411522	0,564278289	0,16182531	0,137551513	0,179653649	-0,135346351	**-1,395346351**
chrXII	465159	465357	198	0,082637257	0,044470435	0,650135768	0,385687039	0,327833983	0,372884471	-0,005115529	**-1,265115529**
chrXII	465159	465357	198	0,082637257	0,044470435	0,650135768	0,385687039	0,327833983	0,372884471	-0,005115529	**-1,265115529**
chrXII	465179	465310	131	0,085486818	0,059293913	0,590457149	0,22872109	0,194412926	0,262547175	0,353547175	**-0,906452825**
chrXII	465182	465312	130	0,339097709	0,374292827	0,475332503	-0,061871698	-0,052590944	0,072684647	0,170684647	**-1,089315353**
chrXII	465193	465310	117	0,467327936	0,555880437	0,456728022	-0,108680331	-0,092378281	0,42473058	0,61373058	**-0,64626942**
chrXII	465193	465312	119	0,113982423	0,096352609	0,541908887	0,105243965	0,08945737	0,528840803	0,703840803	**-0,556159197**
chrXII	465193	465342	149	4,525102207	3,16481262	0,588446336	0,223550187	0,190017659	0,021160812	-0,013839188	**-1,273839188**
chrXII	465193	465348	155	0,302053422	0,226058044	0,57195013	0,181341238	0,154140052	0,083483414	0,006483414	**-1,253516586**
chrXII	465193	465357	164	0,333398588	0,26311674	0,558910346	0,148207125	0,125976056	0,151832088	0,011832088	**-1,248167912**
chrXII	465200	465352	152	0,319150785	0,214940436	0,597558568	0,24703277	0,209977854	0,049201235	-0,006798765	**-1,266798765**
chrXII	465201	465342	141	0,102584181	0,040764565	0,715626635	0,569898223	0,48441349	0,425961554	0,446961554	**-0,813038446**
chrXII	465201	465345	144	0,105433742	0,044470435	0,703340921	0,534033788	0,453928719	0,309666143	0,309666143	**-0,950333857**
chrXII	465201	465351	150	0,102584181	0,048176305	0,680444751	0,468942833	0,398601408	0,266825013	0,224825013	**-1,035174987**
chrXII	465203	465310	107	0,14532759	0,111176087	0,566571175	0,16765125	0,142503563	0,687244311	0,946244311	**-0,313755689**
chrXII	465204	465342	138	0,333398588	0,318704784	0,511266469	0,028244604	0,024007913	0,011373325	0,053373325	**-1,206626675**
chrXII	465204	465348	144	1,803771849	0,830114786	0,684832758	0,481256115	0,409067697	0,249920606	0,249920606	**-1,010079394**
chrXII	465204	465352	148	1,607152169	1,156231309	0,581588542	0,205959029	0,175065175	0,026139418	-0,001860582	**-1,261860582**
chrXII	465204	465357	153	0,142478029	0,066705652	0,681114454	0,470817466	0,400194846	0,286500968	0,223500968	**-1,036499032**
chrXII	465204	465403	199	0,179522317	0,203822827	0,468304659	-0,079532203	-0,067602373	-0,03584809	-0,42084809	**-1,68084809**
chrXII	465204	465418	214	0,031345166	0,059293913	0,345823971	-0,396619676	-0,337126724	-0,02160931	-0,51160931	**-1,77160931**
chrXII	465217	465310	93	0,253610892	0,207528696	0,54996556	0,125574335	0,106738185	0,55334545	0,91034545	**-0,34965455**
chrXII	465217	465342	125	0,433133209	0,400233914	0,519738776	0,049497979	0,042073282	0,36843055	0,50143055	**-0,75856945**
chrXII	465217	465348	131	0,19377012	0,137117174	0,585607617	0,216260411	0,183821349	0,259771079	0,350771079	**-0,909228921**
chrXII	465217	465357	140	2,313843194	1,378583483	0,626645671	0,322982286	0,274534943	0,344765973	0,372765973	**-0,887234027**
chrXII	465221	465352	131	0,082637257	0,044470435	0,650135768	0,385687039	0,327833983	0,40290448	0,49390448	**-0,76609552**
chrXII	465221	465375	154	0,071239015	0,055588044	0,561702018	0,155285859	0,13199298	-0,020621696	-0,090621696	**-1,350621696**
chrXII	465221	465380	159	0,282106498	0,270528479	0,510475286	0,026260665	0,022321566	0,011408144	-0,093591856	**-1,353591856**
chrXII	465221	465403	182	0,065539893	0,133411305	0,329426985	-0,441495981	-0,375271584	-0,18169705	-0,44769705	**-1,70769705**
chrXII	465252	465352	100	0,199469241	0,240881523	0,452978074	-0,118140734	-0,100419624	0,373758045	0,681758045	**-0,578241955**
chrXII	465252	465380	128	0,108283302	0,103764348	0,510655515	0,026712592	0,022705704	0,259567845	0,371567845	**-0,888432155**
chrXII	465252	465403	151	6,878839249	4,243220667	0,618486081	0,301507098	0,256281033	0,089441044	0,040441044	**-1,219558956**
chrXII	465252	465418	166	0,424584527	0,585527393	0,420334142	-0,201038739	-0,170882928	-0,027285825	-0,181285825	**-1,441285825**
chrXII	465257	465427	170	0,034194727	0,037058696	0,479902939	-0,050397187	-0,042837609	0,125044255	-0,056955745	**-1,316955745**
chrXII	465261	465352	91	0,056991212	0,081529131	0,411428464	-0,223871969	-0,190291174	0,170399014	0,541399014	**-0,718600986**
chrXII	465261	465403	142	0,504372223	0,644821307	0,438892328	-0,153778159	-0,130711435	-0,135703957	-0,121703957	**-1,381703957**
chrXII	465261	465418	157	1,478921943	1,348936527	0,522983013	0,057641774	0,048995508	-0,009370725	-0,100370725	**-1,360370725**
chrXII	465261	465470	209	0,276407377	0,281646088	0,495306264	-0,011765723	-0,010000865	0,192675678	-0,262324322	**-1,522324322**
chrXII	465318	465403	85	1,031540931	1,382289353	0,427346089	-0,183134893	-0,155664659	0,239781386	0,652781386	**-0,607218614**
chrXII	465318	465418	100	0,552814753	0,830114786	0,399741807	-0,25401546	-0,215913141	0,259626447	0,567626447	**-0,692373553**
chrXII	465318	465447	129	1,373488201	1,801052615	0,432657282	-0,169612916	-0,144170979	0,088834611	0,193834611	**-1,066165389**
chrXII	465318	465469	151	0,051292091	0,085235	0,375691668	-0,31681585	-0,269293473	-0,436366324	-0,485366324	**-1,745366324**
chrXII	465318	465470	152	15,1169189	10,99160917	0,579003108	0,199343845	0,169442268	0,011466675	-0,044533325	**-1,304533325**
chrXII	465318	465473	155	0,259310013	0,185293479	0,583238813	0,21018617	0,178658244	0,123109539	0,046109539	**-1,213890461**
chrXII	465318	465486	168	0,361894194	0,35205761	0,506888829	0,017268592	0,014678304	0,126997767	-0,041002233	**-1,301002233**
chrXII	465318	465524	206	0,202318801	0,203822827	0,498148398	-0,004641295	-0,003945101	0,094123566	-0,339876434	**-1,599876434**
chrXII	465318	465524	206	0,202318801	0,203822827	0,498148398	-0,004641295	-0,003945101	0,094123566	-0,339876434	**-1,599876434**
chrXII	465323	465470	147	0,031345166	0,048176305	0,394172367	-0,268460684	-0,228191582	-0,333559164	-0,354559164	**-1,614559164**
chrXII	465324	465470	146	0,501522663	0,696703481	0,418554265	-0,205593405	-0,174754394	-0,34265492	-0,35665492	**-1,61665492**
chrXII	465324	465486	162	0,498673102	0,422469132	0,541363846	0,103870259	0,088289721	0,169370763	0,043370763	**-1,216629237**
chrXII	465331	465470	139	0,347646391	0,411351523	0,458033395	-0,105389448	-0,08958103	-0,086337704	-0,051337704	**-1,311337704**
chrXII	465331	465473	142	0,421734966	0,370586958	0,53227729	0,080995639	0,068846293	0,056548177	0,070548177	**-1,189451823**
chrXII	465350	465456	106	4,080570756	8,290030247	0,329860348	-0,440298818	-0,374253995	0,15690388	0,42290388	**-0,83709612**
chrXII	465350	465457	107	0,111132863	0,229763914	0,326001507	-0,450981319	-0,383334121	0,148207884	0,407207884	**-0,852792116**
chrXII	465350	465458	108	0,065539893	0,240881523	0,213888097	-0,793002797	-0,674052378	-0,042623833	0,209376167	**-1,050623833**
chrXII	465350	465470	120	4,234447027	11,00643265	0,277834818	-0,589285701	-0,500892846	-0,137149517	0,030850483	**-1,229149517**
chrXII	465350	465473	123	0,04844253	0,133411305	0,266381679	-0,623793272	-0,530224281	-0,080061194	0,066938806	**-1,193061194**
chrXII	465350	465486	136	0,133929347	0,196411088	0,405428259	-0,239321213	-0,203423031	-0,198680295	-0,142680295	**-1,402680295**
chrXII	465350	465524	174	0,119681545	0,185293479	0,392430643	-0,272989502	-0,232041076	0,009739437	-0,200260563	**-1,460260563**

Chrom	Start	End	Length	Section A	Section B	A/A+B	Z-score	Z * 0.85	Phase correction	Length correction	ΔLKnuc
chrXII	465350	465524	174	0,119681545	0,185293479	0,392430643	-0,272989502	-0,232041076	0,009739437	-0,200260563	-1,460260563
chrXII	465350	465556	206	0,14532759	0,226058044	0,391311824	-0,275901597	-0,234516357	-0,111916871	-0,545916871	-1,805916871
chrXII	465350	465556	206	0,14532759	0,226058044	0,391311824	-0,275901597	-0,234516357	-0,111916871	-0,545916871	-1,805916871
chrXII	465358	465523	165	0,646850252	0,366881088	0,638088443	0,353353936	0,300350846	0,399853267	0,252853267	-1,007146733
chrXII	465358	465523	165	0,646850252	0,366881088	0,638088443	0,353353936	0,300350846	0,399853267	0,252853267	-1,007146733
chrXII	465377	465470	93	0,401788042	0,518821741	0,436436859	-0,160009338	-0,136007937	0,333946502	0,690946502	-0,569053498
chrXII	465377	465484	107	0,045592969	0,100058479	0,313027917	-0,487285766	-0,414192901	0,122878606	0,381878606	-0,878121394
chrXII	465377	465523	146	0,29065518	0,140823044	0,673626532	0,449949388	0,38245698	0,234438031	0,220438031	-1,039561969
chrXII	465377	465523	146	0,29065518	0,140823044	0,673626532	0,449949388	0,38245698	0,234438031	0,220438031	-1,039561969
chrXII	465377	465524	147	2,259701543	1,589818049	0,587008713	0,219856939	0,186878398	0,070833286	0,049833286	-1,210166714
chrXII	465377	465524	147	2,259701543	1,589818049	0,587008713	0,219856939	0,186878398	0,070833286	0,049833286	-1,210166714
chrXII	465377	465556	179	0,282106498	0,277940218	0,503719582	0,009323745	0,007925183	0,164647713	-0,080352287	-1,340352287
chrXII	465377	465556	179	0,282106498	0,277940218	0,503719582	0,009323745	0,007925183	0,164647713	-0,080352287	-1,340352287
chrXII	465377	465584	207	0,102584181	0,140823044	0,421450846	-0,198183249	-0,168455762	-0,011269214	-0,452269214	-1,712269214
chrXII	465377	465584	207	0,102584181	0,140823044	0,421450846	-0,198183249	-0,168455762	-0,011269214	-0,452269214	-1,712269214
chrXII	465425	465524	99	0,330549028	0,452116089	0,422337754	-0,195916517	-0,16652904	0,294999837	0,609999837	-0,650000163
chrXII	465425	465532	107	0,051292091	0,237175653	0,177808756	-0,923748045	-0,785185838	-0,237452803	0,021547197	-1,238452803
chrXII	465425	465556	131	6,337422738	6,062802631	0,51107319	0,027759937	0,023595946	0,093092114	0,184092114	-1,075907886
chrXII	465425	465584	159	2,513312435	1,808464354	0,5815461	0,205850361	0,174972807	0,1704375	0,0654375	-1,1945625
chrXII	465425	465594	169	0,173823196	0,211234566	0,451421093	-0,122071761	-0,103760997	0,063978312	-0,111021688	-1,371021688
chrXII	465425	465608	183	0,455929693	0,952408482	0,323735947	-0,45727709	-0,388685526	-0,344009901	-0,617009901	-1,877009901
chrXII	465427	465524	97	0,039893848	0,077823261	0,338895921	-0,415478239	-0,353156503	0,20057533	0,52957533	-0,73042467
chrXII	465427	465556	129	0,159575393	0,277940218	0,36473074	-0,345841972	-0,293965676	-0,088939783	0,016060217	-1,243939783
chrXII	465427	465584	157	0,615505086	0,956114351	0,391637486	-0,275053712	-0,233795655	-0,289094	-0,380094	-1,640094
chrXII	465427	465608	181	0,065539893	0,248293262	0,208836741	-0,810464113	-0,688894496	-0,510628688	-0,769628688	-2,029628688
chrXII	465435	465584	149	0,97739928	0,696703481	0,583834698	0,211713438	0,179956422	0,011371093	-0,023628907	-1,283628907
chrXII	465447	465556	109	0,062690333	0,129705435	0,325840498	-0,451428154	-0,383713931	0,289572067	0,534572067	-0,725427933
chrXII	465447	465584	137	0,102584181	0,096352609	0,515662191	0,039269381	0,033378974	0,104905919	0,153905919	-1,106094081
chrXII	465447	465608	161	0,569912117	0,61146848	0,482411949	-0,044100996	-0,037485847	-0,040102613	-0,159102613	-1,419102613
chrXII	465498	465584	86	0,182371877	0,667056524	0,214699529	-0,790220412	-0,67168735	-0,287204061	0,118795939	-1,141204061
chrXII	465498	465603	105	0,028495606	0,337234132	0,07791438	-1,419241021	-1,206354868	-0,639306636	-0,366306636	-1,626306636
chrXII	465498	465605	107	1,099930385	8,152913073	0,118874851	-1,180630098	-1,003535583	-0,44340594	-0,18440594	-1,44440594
chrXII	465498	465606	108	0,028495606	0,166764131	0,145936926	-1,054019799	-0,895916829	-0,274178349	-0,022178349	-1,282178349
chrXII	465498	465607	109	0,037044288	0,270528479	0,120440727	-1,172786449	-0,996868482	-0,30673857	-0,06173857	-1,32173857
chrXII	465498	465608	110	1,384886444	14,22683331	0,088708129	-1,348753182	-1,146440205	-0,488691085	-0,250691085	-1,510691085
chrXII	465525	465608	83	0,074088575	0,211234566	0,259665497	-0,644377003	-0,547720452	-0,216290733	0,210709267	-1,049290733
chrXII	465525	465688	163	0,29065518	0,318704784	0,476984372	-0,057723664	-0,049065114	-0,055472887	-0,188472887	-1,448472887
chrXII	465525	465688	163	0,29065518	0,318704784	0,476984372	-0,057723664	-0,049065114	-0,055472887	-0,188472887	-1,448472887
chrXII	465562	465739	177	0,042743409	0,062999783	0,404219015	-0,242441566	-0,206075331	-0,05286528	-0,28386528	-1,54386528
chrXII	465562	465739	177	0,042743409	0,062999783	0,404219015	-0,242441566	-0,206075331	-0,05286528	-0,28386528	-1,54386528
chrXII	465581	465731	150	0,433133209	0,318704784	0,576099124	0,191923981	0,163135384	0,020419368	-0,021580632	-1,281580632
chrXII	465581	465732	151	0,188070999	0,144528914	0,56545715	0,164819952	0,140096959	-0,019742751	-0,068742751	-1,328742751
chrXII	465581	465756	175	0,059840772	0,111176087	0,349911537	-0,385559308	-0,327725412	-0,133495994	-0,350495994	-1,610495994
chrXII	465582	465732	150	0,09973462	0,085235	0,539194599	0,098404877	0,083644145	-0,049958361	-0,091958361	-1,351958361
chrXII	465584	465731	147	1,014443568	0,952408482	0,515770146	0,039540193	0,033609164	-0,075467928	-0,096467928	-1,356467928
chrXII	465584	465732	148	0,09973462	0,114881957	0,464710702	-0,088572826	-0,075286902	-0,228457509	-0,256457509	-1,516457509
chrXII	465584	465745	161	0,105433742	0,192705218	0,353639597	-0,375512712	-0,319185805	-0,316353124	-0,435353124	-1,695353124
chrXII	465584	465756	172	0,749434433	0,841232394	0,471144818	-0,072392395	-0,061533536	0,059174474	-0,136825526	-1,396825526
chrXII	465616	465722	106	0,088336378	0,17788174	0,331819558	-0,434894351	-0,369660198	0,16988647	0,43588647	-0,82411353
chrXII	465616	465731	115	0,088336378	0,159352392	0,356642645	-0,36744744	-0,312330324	0,295189631	0,498189631	-0,761810369
chrXII	465616	465745	129	1,769577122	2,001169572	0,469290903	-0,077052467	-0,065494597	0,13130193	0,23630193	-1,02369807
chrXII	465616	465746	130	0,054141651	0,148234783	0,267529425	-0,620302195	-0,527256866	-0,405618533	-0,307618533	-1,567618533
chrXII	465616	465790	174	0,074088575	0,051882174	0,588141101	0,222765786	0,189350918	0,443694287	0,233694287	-1,026305713
chrXII	465620	465731	111	0,054141651	0,077823261	0,410273081	-0,226842569	-0,192816183	0,291260884	0,522260884	-0,737739116
chrXII	465620	465745	125	0,316301225	0,466939567	0,403836506	-0,243429088	-0,206914725	0,123960413	0,256960413	-1,003039587
chrXII	465620	465746	126	0,24506221	0,289057827	0,45881486	-0,103419898	-0,087906914	0,145612146	0,271612146	-0,988387854
chrXII	465630	465736	106	0,074088575	0,085235	0,465019536	-0,087795679	-0,074626327	0,443138989	0,709138989	-0,550861011
chrXII	465630	465745	115	0,065539893	0,111176087	0,370877003	-0,329531476	-0,280101754	0,328335453	0,531335453	-0,728664547
chrXII	465630	465790	160	0,327699467	0,255705001	0,561702018	0,155285859	0,13199298	0,137835561	0,025835561	-1,234164439
chrXII	465645	465731	86	0,031345166	0,037058696	0,458236793	-0,104876779	-0,089145262	0,279297746	0,685297746	-0,574702254
chrXII	465645	465745	100	0,162424953	0,137117174	0,542244106	0,106088938	0,090175597	0,541180914	0,849180914	-0,410819086
chrXII	465645	465779	134	0,299203861	0,311293045	0,490098899	-0,024820929	-0,021097789	-0,005662104	0,064337896	-1,195662104
chrXII	465645	465790	145	0,065539893	0,055588044	0,541079911	0,103154718	0,08768151	-0,066502136	-0,073502136	-1,333502136
chrXII	465645	465807	162	0,273557816	0,137117174	0,666117544	0,429217551	0,364834918	0,434231055	0,308231055	-0,951768945
chrXII	465661	465832	171	0,262159574	0,311293045	0,457159956	-0,107591283	-0,091452591	0,020545211	-0,168454789	-1,428454789
chrXII	465724	465875	151	0,068389454	0,074117392	0,479902939	-0,050397187	-0,042837609	-0,202976185	-0,251976185	-1,511976185
chrXII	465724	465875	151	0,068389454	0,074117392	0,479902939	-0,050397187	-0,042837609	-0,202976185	-0,251976185	-1,511976185
chrXII	465725	465888	163	0,056991212	0,040764565	0,582995843	0,209563572	0,178129036	0,180556729	0,047556729	-1,212443271
chrXII	465725	465888	163	0,056991212	0,040764565	0,582995843	0,209563572	0,178129036	0,180556729	0,047556729	-1,212443271
chrXII	465731	465888	157	0,139628469	0,129705435	0,518421434	0,046192108	0,039263292	-0,013958294	-0,104958294	-1,364958294
chrXII	465731	465888	157	0,139628469	0,129705435	0,518421434	0,046192108	0,039263292	-0,013958294	-0,104958294	-1,364958294
chrXII	465753	465909	156	0,327699467	0,248293262	0,568929868	0,173650344	0,147602792	0,088825351	0,004825351	-1,255174649
chrXII	465771	465899	128	0,795027403	1,085819787	0,422696436	-0,195000098	-0,165750083	0,077297	0,189297	-1,070703
chrXII	465771	465918	147	0,370442876	0,26682261	0,581300705	0,205222117	0,174438799	0,062916549	0,041916549	-1,218083451
chrXII	465786	465940	154	0,302053422	0,17788174	0,629362977	0,330166647	0,28064165	0,126975925	0,056975925	-1,203024075
chrXII	465790	465899	109	0,028495606	0,070411522	0,288104674	-0,558930214	-0,475090682	0,219662805	0,464662805	-0,795337195
chrXII	465790	465940	150	0,236513528	0,077823261	0,752420767	0,682127291	0,579808197	0,441691229	0,399691229	-0,860308771
chrXII	465903	466057	154	0,088336378	0,044470435	0,665149446	0,426558255	0,362574517	0,218874345	0,148874345	-1,111125655
chrXII	465917	466024	107	0,037044288	0,507704132	0,068002561	-1,490833852	-1,267208774	-0,713368733	-0,454368733	-1,714368733
chrXII	465917	466027	110	0,034194727	0,426175002	0,074276663	-1,444660304	-1,227961258	-0,577447913	-0,339447913	-1,599447913
chrXII	465917	466057	140	0,045592969	0,396528045	0,10312328	-1,263953936	-1,074360846	-1,017467514	-0,989467514	-2,249467514
chrXII	465931	466040	109	0,113982423	0,26682261	0,299319635	-0,52635832	-0,447404572	0,243881771	0,488881771	-0,771118229
chrXII	465932	466040	108	2,701383433	8,79773438	0,234920928	-0,722736385	-0,614325927	0,004138527	0,256138527	-1,003861473
chrXII	465932	466041	109	0,028495606	0,137117174	0,172061636	-0,946049634	-0,804142189	-0,101046151	0,143953849	-1,116046151
chrXII	465934	466027	93	0,065539893	0,455821958	0,125709031	-1,14691183	-0,974875055	-0,502374122	-0,145374122	-1,405374122
chrXII	465934	466057	123	0,039893848	0,722644568	0,052317165	-1,622789879	-1,379371397	-0,931151178	-0,784151178	-2,044151178
chrXII	465940	466096	156	0,074088575	0,08894087	0,454449042	-0,114428548	-0,097264266	-0,16389203	-0,24789203	-1,50789203
chrXII	465941	466048	107	0,04844253	0,159352392	0,23312663	-0,728588752	-0,61930044	-0,063341363	0,195658637	-1,064341363
chrXII	465941	466057	116	0,039893848	0,17417587	0,186359138	-0,891393179	-0,757684202	-0,169141412	0,026858588	-1,233141412
chrXII	465941	466097	156	0,119681545	0,17417587	0,407277606	-0,234553618	-0,199370575	-0,271176487	-0,355176487	-1,615176487
chrXII	465954	466057	103	0,068389454	0,148234783	0,315705458	-0,47974192	-0,407780632	0,195474815	0,482474815	-0,777525185
chrXII	465954	466108	154	0,042743409	0,044470435	0,490098899	-0,024820929	-0,021097789	-0,163198002	-0,233198002	-1,493198002
chrXII	466075	466241	166	0,071239015	0,114881957	0,382756516	-0,298249123	-0,253511755	-0,102575912	-0,256575912	-1,516575912
chrXII	466105	466258	153	0,037044288	0,044470435	0,454449042	-0,114428548	-0,097264266	-0,205878287	-0,268878287	-1,528878287
chrXII	466121	466255	134	0,088336378	0,137117174	0,391816306	-0,274588228	-0,233399994	-0,212794148	-0,142794148	-1,402794148
chrXII	466121	466264	143	0,481575739	0,496586524	0,492327048	-0,019234425	-0,016349262	-0,176706159	-0,169706159	-1,429706159

Chrom	Start	End	Length	Section A	Section B	A/A+B	Z-score	Z * 0.85	Phase correction	Length correction	ΔLKnuc
chrXII	466121	466267	146	0,082637257	0,059293913	0,582234733	0,207613792	0,176471723	0,018654012	0,004654012	-1,255345988
chrXII	466121	466277	156	0,04844253	0,070411522	0,407579962	-0,23377466	-0,198708461	-0,283858752	-0,367858752	-1,627858752
chrXII	466127	466264	137	0,108283302	0,137117174	0,441251393	-0,147797242	-0,125627655	-0,062071282	-0,013071282	-1,273071282
chrXII	466127	466277	150	0,447381012	0,481763045	0,481498007	-0,046394257	-0,039435118	-0,1780357	-0,2200357	-1,4800357
chrXII	466142	466303	161	0,076938136	0,181587609	0,297603381	-0,531305981	-0,451610084	-0,433771488	-0,552771488	-1,812771488
chrXII	466158	466264	106	0,028495606	0,474351306	0,056668551	-1,58337024	-1,345864704	-0,828864991	-0,562864991	-1,822864991
chrXII	466159	466264	105	0,028495606	0,114881957	0,198745224	-0,846111675	-0,719194924	-0,15903945	0,11396055	-1,14603945
chrXII	466159	466342	183	0,062690333	0,329822392	0,159715415	-0,995628195	-0,846283966	-0,806348407	-1,079348407	-2,339348407
chrXII	466159	466342	183	0,062690333	0,329822392	0,159715415	-0,995628195	-0,846283966	-0,806348407	-1,079348407	-2,339348407
chrXII	466184	466344	160	0,09688506	0,111176087	0,465656665	-0,08619258	-0,073263693	-0,066213629	-0,178213629	-1,438213629
chrXII	466184	466344	160	0,09688506	0,111176087	0,465656665	-0,08619258	-0,073263693	-0,066213629	-0,178213629	-1,438213629
chrXII	466207	466337	130	0,19377012	0,507704132	0,27623269	-0,594069873	-0,504959392	-0,368726957	-0,270726957	-1,530726957
chrXII	466207	466337	130	0,19377012	0,507704132	0,27623269	-0,594069873	-0,504959392	-0,368726957	-0,270726957	-1,530726957
chrXII	466220	466354	134	0,076938136	0,048176305	0,614942093	0,292223409	0,248389898	0,27135812	0,34135812	-0,91864188
chrXII	466252	466385	133	1,1711694	0,874585221	0,572487721	0,182711288	0,155304595	0,153297833	0,230297833	-1,029702167
chrXII	466277	466442	165	0,04844253	0,070411522	0,407579962	-0,23377466	-0,198708461	-0,09474518	-0,24174518	-1,50174518
chrXII	466277	466449	172	0,78362916	1,500877179	0,343019035	-0,404237514	-0,343601887	-0,220271405	-0,416271405	-1,676271405
chrXII	466280	466385	105	0,051292091	0,070411522	0,421450846	-0,198183249	-0,168455762	0,387411243	0,660411243	-0,599588757
chrXII	466280	466416	136	0,532867829	0,411351523	0,564347498	0,162001079	0,137700917	0,152069748	0,208069748	-1,051930252
chrXII	466280	466453	173	0,054141651	0,074117392	0,42212736	-0,196454145	-0,166986023	0,044995741	-0,158004259	-1,418004259
chrXII	466289	466442	153	0,464478375	0,326116523	0,587504898	0,221131295	0,187961601	0,085165091	0,022165091	-1,237834909
chrXII	466289	466449	160	0,065539893	0,092646739	0,414320049	-0,216446021	-0,183979117	-0,179531932	-0,291531932	-1,551531932
chrXII	466290	466385	95	0,091185939	0,166764131	0,353502284	-0,375882075	-0,319499764	0,177373527	0,520373527	-0,739626473
chrXII	466290	466416	126	0,111132863	0,111176087	0,499902783	-0,000243688	-0,000207135	0,233502708	0,359502708	-0,900497292
chrXII	466290	466449	159	0,111132863	0,17788174	0,384523349	-0,293622086	-0,249578773	-0,250122146	-0,355122146	-1,615122146
chrXII	466290	466453	163	1,721134592	1,574994571	0,522168431	0,055596643	0,047257147	0,050784061	-0,082215939	-1,342215939
chrXII	466290	466454	164	0,094035499	0,125999566	0,427365971	-0,183084213	-0,155621581	-0,129692835	-0,269692835	-1,529692835
chrXII	466291	466449	158	0,353345512	0,337234132	0,511665114	0,029244273	0,024857632	-0,001058459	-0,099058459	-1,359058459
chrXII	466291	466453	162	0,04844253	0,070411522	0,407579962	-0,23377466	-0,198708461	-0,130868428	-0,256868428	-1,516868428
chrXII	466324	466453	129	0,065539893	0,066705652	0,495592445	-0,011048326	-0,009391077	0,181495176	0,286495176	-0,973504824
chrXII	466324	466454	130	0,401788042	0,318704784	0,557657242	0,145032115	0,123277298	0,259354619	0,357354619	-0,902645381
chrXII	466335	466483	148	0,433133209	0,255705001	0,628788012	0,328645073	0,279348312	0,125193558	0,097193558	-1,162806442
chrXII	466349	466502	153	0,122531105	0,070411522	0,635064977	0,345298405	0,293503645	0,191974548	0,128974548	-1,131025452
chrXII	466351	466500	149	0,256460453	0,159352392	0,616768953	0,297005782	0,252454915	0,08723728	0,05223728	-1,20776272
chrXII	466356	466449	93	0,051292091	0,133411305	0,277699771	-0,589688448	-0,501235181	-0,022541919	0,334458081	-0,925541919
chrXII	466356	466483	127	0,068389454	0,074117392	0,479902939	-0,050397187	-0,042837609	0,219305522	0,338305522	-0,921694478
chrXII	466356	466500	144	0,059840772	0,037058696	0,617555219	0,299066166	0,254206241	0,068766193	0,068766193	-1,191233807
chrXII	466356	466502	146	0,97454972	0,641115437	0,603187929	0,261607395	0,222366286	0,062133318	0,048133318	-1,211866682
chrXII	466364	466500	136	0,384690679	0,333528262	0,535617563	0,08939893	0,07598909	0,092183369	0,148183369	-1,111816631
chrXII	466367	466532	165	0,125380666	0,185293479	0,403576119	-0,244101464	-0,207486244	-0,097521289	-0,244521289	-1,504521289
chrXII	466367	466538	171	0,279256937	0,251999131	0,525654112	0,064349707	0,054697251	0,164102245	-0,024897755	-1,284897755
chrXII	466385	466532	147	0,051292091	0,040764565	0,557179598	0,143822286	0,122248943	0,012818718	-0,008181282	-1,268181282
chrXII	466385	466538	153	0,225115286	0,059293913	0,791519004	0,811703139	0,689947668	0,595188444	0,532188444	-0,727811556
chrXII	466392	466502	110	0,042743409	0,08894087	0,324590066	-0,454901459	-0,386666241	0,247238188	0,485238188	-0,774761812
chrXII	466392	466544	152	0,507221784	0,281646088	0,642974321	0,366420455	0,311457386	0,155507694	0,099507694	-1,160492306
chrXII	466403	466500	97	0,042743409	0,070411522	0,377742344	-0,311415611	-0,264703269	0,286622456	0,615622456	-0,644377544
chrXII	466403	466502	99	0,128230226	0,051882174	0,711945574	0,559077467	0,475215847	0,947228858	1,262228858	0,002228858
chrXII	466403	466544	141	0,094035499	0,040764565	0,69759239	0,517488464	0,439865194	0,379753977	0,400753977	-0,859246023
chrXII	466403	466559	156	1,062886098	0,79305609	0,572693538	0,183235904	0,155750518	0,064435227	-0,019564773	-1,279564773
chrXII	466432	466521	89	0,037044288	0,270528479	0,120440727	-1,172786449	-0,996868482	-0,559349753	-0,174349753	-1,434349753
chrXII	466432	466523	91	0,028495606	0,129705435	0,180122745	-0,914897409	-0,777662797	-0,416883336	-0,045883336	-1,305883336
chrXII	466432	466527	95	0,028495606	0,389116306	0,068234658	-1,489068562	-1,265708277	-0,76579282	-0,42279282	-1,68279282
chrXII	466432	466528	96	0,102584181	0,852350003	0,107425394	-1,240336958	-1,054286414	-0,478346336	-0,142346336	-1,402346336
chrXII	466432	466530	98	0,031345166	0,344645871	0,083366792	-1,382775925	-1,175359536	-0,658597643	-0,336597643	-1,596597643
chrXII	466432	466531	99	0,042743409	0,389116306	0,098975217	-1,287412835	-1,09430091	-0,642053606	-0,327053606	-1,587053606
chrXII	466432	466532	100	15,31068902	81,57360116	0,158030667	-1,002584589	-0,852196901	-0,429212083	-0,121212083	-1,381212083
chrXII	466432	466534	102	0,034194727	0,426175002	0,074276663	-1,444660304	-1,227961258	-0,683766327	-0,389766327	-1,649766327
chrXII	466432	466538	106	0,139628469	0,381704567	0,267829697	-0,619390113	-0,526481596	-0,028076878	0,237923122	-1,022076878
chrXII	466432	466539	107	0,199469241	0,318704784	0,38494643	-0,292515043	-0,248637787	0,299502365	0,558502365	-0,701497635
chrXII	466474	466613	139	0,074088575	0,096352609	0,434687048	-0,164453569	-0,139785534	-0,142357428	-0,107357428	-1,367357428
chrXII	466474	466618	144	0,544266071	0,329822392	0,622667034	0,312492935	0,265618995	0,076543587	0,076543587	-1,183456413
chrXII	466497	466613	116	0,056991212	0,17417587	0,246536882	-0,685428183	-0,582613956	0,013925073	0,209925073	-1,050074927
chrXII	466497	466618	121	0,039893848	0,037058696	0,518421434	0,046192108	0,039263292	0,43661063	0,59761063	-0,66238937
chrXII	466497	466667	170	0,173823196	0,237175653	0,422928668	-0,194406838	-0,165245812	-0,003557855	-0,185557855	-1,445557855
chrXII	466507	466613	106	0,789328282	5,110394149	0,133790749	-1,108649309	-0,942351913	-0,425414891	-0,159414891	-1,419414891
chrXII	466509	466613	104	0,04844253	0,833820655	0,054907119	-1,599028642	-1,359174346	-0,778094708	-0,498094708	-1,758094708
chrXII	466509	466618	109	0,034194727	0,077823261	0,305261035	-0,509328378	-0,432929121	0,278468028	0,523468028	-0,736531972
chrXII	466557	466700	143	0,190920559	0,085235	0,691351496	0,499684841	0,424732115	0,263339397	0,270339397	-0,989660603
chrXII	466557	466704	147	0,079787696	0,066705652	0,544650641	0,112157259	0,09533367	-0,023748707	-0,044748707	-1,304748707
chrXII	466557	466708	151	0,094035499	0,055588044	0,628480635	0,327831948	0,278657156	0,125162267	0,076162267	-1,183837733
chrXII	466557	466709	152	0,464478375	0,181587609	0,718933339	0,579675715	0,492724358	0,337952451	0,281952451	-0,978047549
chrXII	466574	466729	155	0,039893848	0,077823261	0,338895921	-0,415478239	-0,353156503	-0,417345084	-0,494345084	-1,754345084
chrXII	466577	466730	153	0,062690333	0,040764565	0,605967759	0,268824834	0,228501109	0,122891759	0,059891759	-1,200108241
chrXII	466611	466736	125	0,028495606	0,111176087	0,204018475	-0,827353109	-0,703250143	-0,398849683	-0,265849683	-1,525849683
chrXII	466690	466843	153	0,162424953	0,085235	0,655838584	0,40113215	0,340962327	0,229230877	0,166230877	-1,093769123
chrXII	466706	466873	167	0,031345166	0,077823261	0,287126664	-0,56179848	-0,477528708	-0,342604263	-0,503604263	-1,763604263
chrXII	466706	466878	172	0,284956058	0,566998046	0,334473485	-0,427593677	-0,363454625	-0,234582062	-0,430582062	-1,690582062
chrXII	466737	466873	136	0,817823887	0,663350655	0,55214552	0,131083871	0,11142129	0,139476622	0,195476622	-1,064523378
chrXII	466737	466876	139	0,168124074	0,066705652	0,71594034	0,570823457	0,485199939	0,476130719	0,511130719	-0,748869281
chrXII	466737	466878	141	0,076938136	0,103764348	0,42577243	-0,18714766	-0,159075511	-0,219186302	-0,198186302	-1,458186302
chrXII	466743	466900	157	0,09973462	0,040764565	0,709859063	0,552973037	0,470027081	0,404620484	0,313620484	-0,946379516
chrXII	466757	466910	153	0,564212996	0,485468915	0,537508544	0,094158927	0,080035088	-0,031696362	-0,094696362	-1,354696362
chrXII	466757	466924	167	0,059840772	0,114881957	0,342489913	-0,405677162	-0,344825587	-0,214540173	-0,375540173	-1,635540173
chrXII	466771	466894	123	0,173823196	0,52623348	0,248298747	-0,679853078	-0,577875116	-0,132787505	0,014212495	-1,245787505
chrXII	466804	466956	152	0,074088575	0,059293913	0,555459536	0,139467258	0,118547169	-0,036999552	-0,092999552	-1,352999552
chrXII	466940	467096	156	0,19377012	0,118587827	0,620346375	0,30639062	0,260432027	0,172841998	0,088841998	-1,171158002
chrXII	466947	467098	151	0,051292091	0,051882174	0,497140355	-0,007168129	-0,00609291	-0,154342238	-0,203342238	-1,463342238
chrXII	466953	467111	158	0,122531105	0,059293913	0,673895739	0,450696199	0,383091769	0,355690498	0,257690498	-1,002309502
chrXII	466957	467090	133	0,113982423	0,055588044	0,672183225	0,445949741	0,37905728	0,376221453	0,453221453	-0,806778547
chrXII	467009	467160	151	0,250761331	0,181587609	0,579997562	0,201887241	0,171604155	0,030149541	-0,018850459	-1,278850459
chrXII	467063	467189	126	0,054141651	0,048176305	0,529151025	0,07313593	0,062165541	0,270447543	0,396447543	-0,863552457
chrXII	467082	467240	158	0,042743409	0,040764565	0,51184823	0,029703476	0,025247955	-0,004117349	-0,102117349	-1,362117349
chrXII	467082	467241	159	0,307752543	0,35576348	0,463820816	-0,090812431	-0,077190567	-0,078762402	-0,183762402	-1,443762402
chrXII	467086	467196	110	0,034194727	0,048176305	0,415130494	-0,214366854	-0,182211826	0,447312824	0,685312824	-0,574687176
chrXII	467086	467238	152	0,330549028	0,214940436	0,605967759	0,268824834	0,228501109	0,079284507	0,023284507	-1,236715493
chrXII	467087	467238	151	0,14817715	0,092646739	0,615292571	0,293140374	0,249169318	0,109641251	0,060641251	-1,199358749

Chrom	Start	End	Length	Section A	Section B	A/A+B	Z-score	Z * 0.85	Phase correction	Length correction	ΔLKnuc
chrXII	467087	467240	153	0,547115632	0,403939784	0,575272085	0,189812794	0,161340875	0,038388067	-0,024611933	**-1,284611933**
chrXII	467096	467196	100	0,034194727	0,044470435	0,434687048	-0,164453569	-0,139785534	0,262855529	0,570855529	**-0,689144471**
chrXII	467096	467247	151	0,116831984	0,051882174	0,692484764	0,502905845	0,427469968	0,294669253	0,245669253	**-1,014330747**
chrXII	467113	467196	83	0,068389454	0,137117174	0,332784663	-0,432236787	-0,367401269	-0,038964081	0,388035919	**-0,871964081**
chrXII	467113	467256	143	0,128230226	0,085235	0,600707798	0,255179578	0,216902641	0,050708423	0,057708423	**-1,202291577**
chrXII	467120	467226	106	0,085486818	0,085235	0,500737507	0,001848658	0,001571359	0,519553222	0,785553222	**-0,474446778**
chrXII	467120	467240	120	0,028495606	0,077823261	0,268020217	-0,618811668	-0,525989918	-0,175839735	-0,007839735	**-1,267839735**
chrXII	467120	467274	154	0,735186631	0,715232829	0,506878631	0,017243027	0,014656573	-0,123196088	-0,193196088	**-1,453196088**
chrXII	467123	467254	131	0,079787696	0,062999783	0,558786364	0,147892925	0,125708986	0,200435818	0,291435818	**-0,968564182**
chrXII	467162	467304	142	0,091185939	0,144528914	0,386848507	-0,287542436	-0,24441107	-0,265607052	-0,251607052	**-1,511607052**
chrXII	467192	467274	82	0,056991212	0,070411522	0,447331152	-0,132407093	-0,112546029	0,209452455	0,643452455	**-0,616547545**
chrXII	467192	467343	151	0,860567296	0,574409785	0,599708043	0,252591479	0,214702758	0,085859114	0,036859114	**-1,223140886**
chrXII	467216	467364	148	0,054141651	0,048176305	0,529151025	0,07313593	0,062165541	-0,085866419	-0,113866419	**-1,373866419**
chrXII	467224	467368	144	0,364743755	0,192705218	0,654308775	0,396979674	0,337432723	0,139595359	0,139595359	**-1,120404641**
chrXII	467264	467414	150	0,111132863	0,070411522	0,612152576	0,284933816	0,242193743	0,099836118	0,057836118	**-1,202163882**
chrXII	467267	467430	163	0,173823196	0,103764348	0,626192347	0,32178536	0,273517556	0,276847486	0,143847486	**-1,116152514**
chrXII	467271	467409	138	0,190920559	0,081529131	0,700755282	0,526574021	0,447587917	0,427295635	0,469295635	**-0,790704365**
chrXII	467271	467414	143	0,310602104	0,207528696	0,59946659	0,251966677	0,214171675	0,045666761	0,052666761	**-1,207333239**
chrXII	467273	467432	159	0,09973462	0,081529131	0,550218231	0,126212725	0,107280816	0,11080193	0,00580193	**-1,25419807**
chrXII	467280	467428	148	0,088336378	0,077823261	0,531635591	0,07938196	0,067474666	-0,068606866	-0,096606866	**-1,356606866**
chrXII	467303	467458	155	0,547115632	0,515115871	0,515062517	0,037765106	0,03210034	-0,039416837	-0,116416837	**-1,376416837**
chrXII	467303	467459	156	0,054141651	0,051882174	0,510655515	0,026712592	0,022705704	-0,065747579	-0,149747579	**-1,409747579**
chrXII	467304	467459	155	0,128230226	0,085235	0,600707798	0,255179578	0,216902641	0,1467977	0,0697977	**-1,1902023**
chrXII	467314	467414	100	0,034194727	0,048176305	0,415130494	-0,214366854	-0,182211826	0,242851694	0,550851694	**-0,709148306**
chrXII	467314	467459	145	0,846319493	0,581821524	0,592602189	0,234243918	0,19910733	0,047488923	0,040488923	**-1,219511077**
chrXII	467314	467464	150	0,051292091	0,048176305	0,515662191	0,039269381	0,033378974	-0,12353872	-0,16553872	**-1,42553872**
chrXII	467314	467487	173	0,561363435	0,833820655	0,402357968	-0,247248471	-0,2101612	-0,000968457	-0,203968457	**-1,463968457**
chrXII	467316	467464	148	0,299203861	0,170470001	0,637046013	0,350573989	0,297987891	0,163787347	0,135787347	**-1,124212653**
chrXII	467328	467458	130	0,051292091	0,051882174	0,497140355	-0,007168129	-0,00609291	0,113997572	0,211997572	**-1,048002428**
chrXII	467328	467489	161	0,598407723	0,415057393	0,590457149	0,22872109	0,194412926	0,212033052	0,093033052	**-1,166966948**
chrXII	467332	467459	127	0,076938136	0,066705652	0,535617563	0,08939893	0,07598909	0,323849141	0,442849141	**-0,817150859**
chrXII	467332	467487	155	0,963151477	0,463233697	0,675239405	0,454427464	0,386263344	0,331608117	0,254608117	**-1,005391883**
chrXII	467341	467487	146	0,082637257	0,081529131	0,503375009	0,008459995	0,007190996	-0,148135006	-0,162135006	**-1,422135006**
chrXII	467341	467517	176	0,208017923	0,251999131	0,452196111	-0,120114784	-0,102097566	0,068371505	-0,155628495	**-1,415628495**
chrXII	467428	467588	160	0,518620026	0,26682261	0,660290138	0,413255097	0,351266833	0,347152949	0,235152949	**-1,024847051**
chrXII	467434	467588	154	1,205364127	0,796761959	0,602042067	0,258636308	0,219840862	0,083802184	0,013802184	**-1,246197816**
chrXII	467435	467598	163	0,094035499	0,048176305	0,661235543	0,415837502	0,353461877	0,349897787	0,216897787	**-1,043102213**
chrXII	467456	467609	153	0,14817715	0,137117174	0,519383449	0,048606234	0,041315299	-0,083761565	-0,146761565	**-1,406761565**
chrXII	467475	467629	154	0,273557816	0,248293262	0,524206671	0,060714406	0,051607245	-0,090864087	-0,160864087	**-1,420864087**
chrXII	467493	467630	137	0,054141651	0,037058696	0,593656197	0,236960278	0,201416237	0,264984181	0,313984181	**-0,946015819**
chrXII	467493	467645	152	0,202318801	0,125999566	0,616227484	0,295587617	0,251249475	0,101066187	0,045066187	**-1,214933813**
chrXII	467496	467660	164	0,116831984	0,100058479	0,538668149	0,097078943	0,082517102	0,106274428	-0,033725572	**-1,293725572**
chrXII	467502	467630	128	0,820673448	1,030231743	0,443390322	-0,142379007	-0,121022156	0,124402598	0,236402598	**-1,023597402**
chrXII	467510	467630	120	0,054141651	0,085235	0,388455674	-0,283346352	-0,2408444	0,135625693	0,303625693	**-0,956374307**
chrXII	467510	467645	135	0,028495606	0,051882174	0,354520937	-0,373143192	-0,317171714	-0,347213338	-0,284213338	**-1,544213338**
chrXII	467510	467667	157	0,153876272	0,192705218	0,443982948	-0,140878539	-0,119746758	-0,184756594	-0,275756594	**-1,535756594**
chrXII	467511	467669	158	0,074088575	0,081529131	0,47609348	-0,059960669	-0,050966568	-0,08030926	-0,17830926	**-1,43830926**
chrXII	467515	467649	134	0,091185939	0,051882174	0,637360324	0,351411905	0,298700119	0,320313436	0,390313436	**-0,869686564**
chrXII	467532	467638	106	0,04844253	0,066705652	0,420697304	-0,200109929	-0,170093439	0,346383264	0,612383264	**-0,647616736**
chrXII	467556	467724	168	0,065539893	0,085235	0,434687048	-0,164453569	-0,139785534	-0,038857542	-0,206857542	**-1,466857542**
chrXII	467645	467795	150	0,037044288	0,066705652	0,357053581	-0,366345661	-0,311393812	-0,46262558	-0,50462558	**-1,76462558**
chrXII	467645	467796	151	0,094035499	0,111176087	0,458236793	-0,104876779	-0,089145262	-0,226740651	-0,275740651	**-1,535740651**
chrXII	467883	468037	154	0,034194727	0,037058696	0,479902939	-0,050397187	-0,042837609	-0,179928375	-0,249928375	**-1,509928375**
chrXII	467883	468038	155	0,088336378	0,096352609	0,478298027	-0,054425638	-0,046261792	-0,096762196	-0,173762196	**-1,433762196**
chrXII	467883	468049	166	0,225115286	0,159352392	0,585524607	0,216047418	0,183640306	0,342370126	0,188370126	**-1,071629874**
chrXII	467887	468049	162	0,04844253	0,051882174	0,482857442	-0,042983253	-0,036535765	0,026588256	-0,099411744	**-1,359411744**
chrXII	467905	468037	132	0,113982423	0,166764131	0,405997586	-0,23785292	-0,202174982	-0,066353329	0,017646671	**-1,242353329**
chrXII	467905	468038	133	0,133929347	0,170470001	0,439979088	-0,151022236	-0,128368901	-0,131191197	-0,054191197	**-1,314191197**
chrXII	467905	468049	144	0,63545201	0,426175002	0,598564282	0,249632664	0,212187765	0,008867386	0,008867386	**-1,251132614**
chrXII	467906	468037	131	0,681044979	0,678174133	0,501056065	0,002647165	0,00225009	0,067997586	0,158997586	**-1,101002414**
chrXII	467906	468038	132	1,521665352	1,434171527	0,514800178	0,037107059	0,031541	0,157695023	0,241695023	**-1,018304977**
chrXII	467906	468049	143	0,800726524	0,566998046	0,585444279	0,215841315	0,183465118	0,018441326	0,025441326	**-1,234558674**
chrXII	467906	468050	144	0,19377012	0,062999783	0,754644987	0,68917996	0,585802966	0,390022271	0,390022271	**-0,869977729**
chrXII	467907	468037	130	0,091185939	0,107470218	0,459013908	-0,102918298	-0,087480553	0,013309602	0,111309602	**-1,148690398**
chrXII	467907	468038	131	0,703841464	1,037643482	0,40416167	-0,242589598	-0,206201158	-0,140637707	-0,049637707	**-1,309637707**
chrXII	467907	468049	142	0,131079787	0,111176087	0,541079911	0,103154718	0,08768151	0,060609908	0,074609908	**-1,185390092**
chrXII	467983	468139	156	0,216566604	0,066705652	0,764517524	0,720909899	0,612773414	0,525601987	0,441601987	**-0,818398013**
chrXII	468039	468185	146	0,088336378	0,077823261	0,531635591	0,07938196	0,067474666	-0,09506816	-0,10906816	**-1,36906816**
chrXII	468045	468181	136	0,113982423	0,137117174	0,453933118	-0,11573037	-0,098370815	-0,078772836	-0,022772836	**-1,282772836**
chrXII	468045	468185	140	0,173823196	0,085235	0,670981263	0,442624345	0,376230693	0,42950572	0,45750572	**-0,80249428**
chrXII	468047	468203	156	0,051292091	0,08894087	0,365763444	-0,34309514	-0,291630869	-0,364220607	-0,448220607	**-1,708220607**
chrXII	468050	468139	89	0,108283302	0,133411305	0,448017039	-0,130672891	-0,111071958	0,309158069	0,694158069	**-0,565841931**
chrXII	468050	468175	125	1,162620718	1,823287833	0,389369165	-0,280963586	-0,238819048	0,032695602	0,165695602	**-1,094304398**
chrXII	468050	468176	126	0,028495606	0,037058696	0,434687048	-0,164453569	-0,139785534	0,09038191	0,21638191	**-1,04361809**
chrXII	468050	468180	130	0,883363781	1,119172613	0,44112246	-0,148123985	-0,125905388	-0,023329644	0,074670356	**-1,185329644**
chrXII	468050	468181	131	3,744322607	5,028865018	0,42679158	-0,18454853	-0,15686625	-0,071440179	0,019559821	**-1,240440179**
chrXII	468050	468184	134	1,723984153	1,971522616	0,466508184	-0,084050389	-0,071442831	-0,0495425	0,0204575	**-1,2395425**
chrXII	468050	468185	135	0,227964847	0,218646305	0,5104325	0,02615338	0,022230373	-0,011465711	0,051534289	**-1,208465711**
chrXII	468050	468203	153	0,131079787	0,122293696	0,517338221	0,043474165	0,03695304	-0,07753062	-0,14053062	**-1,40053062**
chrXII	468070	468181	111	0,119681545	0,233469783	0,338895921	-0,415478239	-0,353156503	0,135821224	0,366821224	**-0,893178776**
chrXII	468070	468184	114	0,039893848	0,070411522	0,361667325	-0,354005648	-0,300904801	0,325933148	0,535933148	**-0,724066852**
chrXII	468070	468203	133	0,313451664	0,259410871	0,547167331	0,118507771	0,100731606	0,083818863	0,160818863	**-1,099181137**
chrXII	468187	468283	96	0,151026711	0,930173264	0,139684346	-1,081738612	-0,91947782	-0,376706257	-0,040706257	**-1,300706257**
chrXII	468226	468399	173	0,353345512	0,548468698	0,391816306	-0,274588228	-0,233399994	-0,038027388	-0,241027388	**-1,501027388**
chrXII	468226	468402	176	0,039893848	0,062999783	0,387719315	-0,285268256	-0,242478018	-0,079694439	-0,303694439	**-1,563694439**
chrXII	468232	468399	167	0,091185939	0,125999566	0,419852784	-0,202270107	-0,171929591	-0,044487332	-0,205487332	**-1,465487332**
chrXII	468232	468402	170	0,689593661	1,196995874	0,365523951	-0,343731921	-0,292172133	-0,128627036	-0,310627036	**-1,570627036**
chrXII	468235	468370	135	1,940550757	1,467524353	0,569397884	0,174841432	0,148615217	0,125104532	0,188104532	**-1,071895468**
chrXII	468235	468399	164	0,156725832	0,17417587	0,473632595	-0,066141475	-0,056220254	-0,028588157	-0,168588157	**-1,428588157**
chrXII	468303	468465	162	0,062690333	0,055588044	0,530023616	0,075329227	0,064029843	0,130079181	0,004079181	**-1,255920819**
chrXII	468322	468465	143	0,800726524	0,52993935	0,601748748	0,257876135	0,219194715	0,050927602	0,057927602	**-1,202072398**
chrXII	468341	468490	149	0,814974327	0,396528045	0,672697261	0,4473734	0,38026739	0,216774829	0,181774829	**-1,078225171**
chrXII	468341	468513	172	0,062690333	0,059293913	0,51392155	0,034903236	0,029667751	0,153402831	-0,042597169	**-1,302597169**
chrXII	468341	468556	215	0,04844253	0,062999783	0,434687048	-0,164453569	-0,139785534	0,185853124	-0,311146876	**-1,571146876**
chrXII	468364	468513	149	0,757983115	0,633703698	0,544650641	0,112157259	0,09533367	-0,065222575	-0,100222575	**-1,360222575**
chrXII	468364	468554	190	0,037044288	0,066705652	0,357053581	-0,366345661	-0,311393812	-0,284584018	-0,606584018	**-1,866584018**

Chrom	Start	End	Length	Section A	Section B	A/A+B	Z-score	Z * 0.85	Phase correction	Length correction	ΔLKnuc
chrXII	468364	468556	192	0,071239015	0,103764348	0,407072261	-0,235082731	-0,199820321	-0,131382058	-0,467382058	-1,727382058
chrXII	468364	468569	205	0,037044288	0,051882174	0,416572153	-0,210670615	-0,179070023	-0,113549754	-0,540549754	-1,800549754
chrXII	468372	468532	160	0,168124074	0,062999783	0,727419819	0,605028046	0,514273839	0,502629408	0,390629408	-0,869370592
chrXII	468373	468530	157	0,427434088	0,429880871	0,498572996	-0,003576976	-0,00304043	-0,054697754	-0,145697754	-1,405697754
chrXII	468373	468554	181	0,136778908	0,103764348	0,568624996	0,172874585	0,146943398	0,320394299	0,061394299	-1,198605701
chrXII	468373	468556	183	0,259310013	0,340940001	0,432003343	-0,171276081	-0,145584669	-0,112116746	-0,385116746	-1,645116746
chrXII	468401	468490	89	0,116831984	0,092646739	0,55772721	0,145209355	0,123427951	0,554163856	0,939163856	-0,320836144
chrXII	468401	468513	112	0,151026711	0,226058044	0,400511314	-0,252023851	-0,214220273	0,321459698	0,545459698	-0,714540302
chrXII	468401	468532	131	0,039893848	0,040764565	0,49460244	-0,013530088	-0,011500575	0,095875161	0,186875161	-1,073124839
chrXII	468401	468554	153	2,239754619	1,515700658	0,596400291	0,244040545	0,207434463	0,086055611	0,023055611	-1,236944389
chrXII	468401	468556	155	2,137170438	1,767699789	0,547308954	0,118865275	0,101035484	0,054820825	-0,022179175	-1,282179175
chrXII	468401	468569	168	0,87766466	0,770820872	0,53240665	0,081320967	0,069122822	0,162309563	-0,005690437	-1,265690437
chrXII	468401	468578	177	0,812124766	0,959820221	0,458323916	-0,10465719	-0,088958611	0,050360836	-0,180639164	-1,440639164
chrXII	468402	468513	111	0,056991212	0,070411522	0,447331152	-0,132407093	-0,112546029	0,364467781	0,595467781	-0,664532219
chrXII	468402	468530	128	0,202318801	0,181587609	0,52700032	0,067731518	0,05757179	0,303723315	0,415723315	-0,844276685
chrXII	468402	468550	148	0,051292091	0,037058696	0,58055047	0,203301913	0,172806626	0,054765271	0,026765271	-1,233234729
chrXII	468402	468553	151	0,042743409	0,074117392	0,365763444	-0,34309514	-0,291630869	-0,420648055	-0,469648055	-1,729648055
chrXII	468402	468554	152	1,572957442	1,226642831	0,561850725	0,155663143	0,132313672	-0,03183442	-0,08783442	-1,34783442
chrXII	468402	468556	154	11,17312705	7,1263872	0,610569597	0,280803909	0,238683323	0,088976806	0,018976806	-1,241023194
chrXII	468402	468559	157	0,199469241	0,155646522	0,561702018	0,155285859	0,13199298	0,080755179	-0,010244821	-1,270244821
chrXII	468402	468569	167	0,168124074	0,259410871	0,393240544	-0,270882911	-0,230250475	-0,099945279	-0,260945279	-1,520945279
chrXII	468402	468578	176	0,059840772	0,196411088	0,23352327	-0,727292911	-0,618198974	-0,45101063	-0,67501063	-1,93501063
chrXII	468402	468604	202	0,116831984	0,074117392	0,61184795	0,284138687	0,241517884	0,26679045	-0,13920955	-1,39920955
chrXII	468402	468606	204	0,185221438	0,188999349	0,494952297	-0,012653052	-0,010755094	0,051015704	-0,368984296	-1,628984296
chrXII	468404	468530	126	0,054141651	0,074117392	0,42212736	-0,196454145	-0,166986023	0,062766605	0,188766605	-1,071233395
chrXII	468404	468554	150	0,606956404	0,392822175	0,607090827	0,271744676	0,230982975	0,068141063	0,026141063	-1,233858937
chrXII	468404	468556	152	2,823914538	1,574994571	0,641957919	0,363697164	0,309142589	0,139189658	0,083189658	-1,176810342
chrXII	468404	468559	155	0,703841464	0,403939784	0,635361417	0,346087223	0,29417414	0,249887945	0,172887945	-1,087112055
chrXII	468404	468569	165	0,188070999	0,151940653	0,553131041	0,13357593	0,113539541	0,22776447	0,08076447	-1,17923553
chrXII	468404	468578	174	0,037044288	0,070411522	0,344739737	-0,399561558	-0,339627325	-0,074182889	-0,284182889	-1,544182889
chrXII	468404	468606	202	0,056991212	0,044470435	0,561702018	0,155285859	0,13199298	0,156966565	-0,249033435	-1,509033435
chrXII	468405	468513	108	0,037044288	0,122293696	0,232488743	-0,730675332	-0,621074032	-0,013063141	0,238936859	-1,021063141
chrXII	468405	468530	125	0,222265726	0,285351958	0,437860486	-0,156395895	-0,132936511	0,127156126	0,260156126	-0,999843874
chrXII	468405	468532	127	0,04844253	0,081529131	0,372716096	-0,324668217	-0,275967985	-0,047020613	0,071979387	-1,188020613
chrXII	468405	468550	145	0,068389454	0,040764565	0,626540868	0,322705529	0,274299699	0,117719359	0,110719359	-1,149280641
chrXII	468405	468553	148	0,068389454	0,077823261	0,467739443	-0,080953558	-0,068810524	-0,192884123	-0,220884123	-1,480884123
chrXII	468405	468554	149	2,117223514	1,241466309	0,630371849	0,332838358	0,282912604	0,127052079	0,092052079	-1,167947921
chrXII	468405	468556	151	10,80268417	5,866391543	0,648067377	0,380108002	0,323091801	0,190035171	0,141035171	-1,118964829
chrXII	468405	468559	154	0,296354301	0,17417587	0,629830602	0,331404725	0,281694016	0,121679706	0,051679706	-1,208320294
chrXII	468405	468568	163	0,105433742	0,103764348	0,503989982	0,010001569	0,008501334	0,006317162	-0,126682838	-1,386682838
chrXII	468405	468569	164	1,766727562	1,437877396	0,551309002	0,128969231	0,109623846	0,148489617	0,008489617	-1,251510383
chrXII	468405	468578	173	0,071239015	0,144528914	0,330164984	-0,439457641	-0,373538995	-0,168934013	-0,371934013	-1,631934013
chrXII	468405	468604	199	0,173823196	0,066705652	0,722670886	0,590794338	0,502175188	0,532680747	0,147680747	-1,112319253
chrXII	468405	468606	201	0,222265726	0,181587609	0,550362486	0,126577221	0,107590638	0,125964251	-0,273035749	-1,533035749
chrXII	468406	468513	107	0,045592969	0,255705001	0,15132186	-1,030780594	-0,876163505	-0,317675714	-0,058675714	-1,318675714
chrXII	468406	468530	124	0,122531105	0,300175436	0,289872744	-0,55375652	-0,470693042	-0,068335156	0,071664844	-1,188335156
chrXII	468406	468550	144	0,082637257	0,051882174	0,614314648	0,290582424	0,24699506	0,048490104	0,048490104	-1,211509896
chrXII	468406	468554	148	4,963934536	2,694167184	0,648193863	0,380448829	0,323381505	0,200887874	0,172887874	-1,087112126
chrXII	468406	468556	150	3,31973808	2,175345443	0,604128776	0,264048627	0,224441333	0,055993204	0,013993204	-1,246006796
chrXII	468406	468559	153	0,179522317	0,137117174	0,566961234	0,168642904	0,143346468	0,014218311	-0,048781689	-1,308781689
chrXII	468406	468568	162	0,045592969	0,055588044	0,450607955	-0,124125498	-0,105506674	-0,040619073	-0,166619073	-1,426619073
chrXII	468406	468604	198	0,139628469	0,081529131	0,631352796	0,335438395	0,285122636	0,332136344	-0,045863656	-1,305863656
chrXII	468406	468606	200	0,091185939	0,114881957	0,442504343	-0,144622812	-0,12292939	-0,102377046	-0,494377046	-1,754377046
chrXII	468445	468551	106	0,185221438	0,251999131	0,423633861	-0,192605774	-0,163714908	0,334535265	0,600535265	-0,659464735
chrXII	468445	468554	109	0,076938136	0,08894087	0,463820816	-0,090812431	-0,077190567	0,62851509	0,87351509	-0,38648491
chrXII	468445	468556	111	0,188070999	0,333528262	0,360566076	-0,356946107	-0,303404191	0,164728432	0,395728432	-0,864271568
chrXII	468445	468604	159	0,085486818	0,051882174	0,622315244	0,311567137	0,264832067	0,271385118	0,166385118	-1,093614882
chrXII	468445	468606	161	0,886213341	0,696703481	0,559860966	0,150616725	0,128024216	0,129723965	0,010723965	-1,249276035
chrXII	468462	468603	141	0,227964847	0,144528914	0,611996417	0,284526191	0,241847262	0,180887453	0,201887453	-1,058112547
chrXII	468463	468554	91	0,116831984	0,148234783	0,44076436	-0,149031573	-0,126676837	0,223227124	0,594227124	-0,665772876
chrXII	468463	468556	93	0,370442876	0,359469349	0,507517018	0,018843485	0,016016962	0,478510765	0,835510765	-0,424489235
chrXII	468463	468604	141	1,359240398	0,61888022	0,687137269	0,487752073	0,414589262	0,350132594	0,371132594	-0,888867406
chrXII	468463	468606	143	0,156725832	0,085235	0,647732241	0,379205162	0,322324387	0,145108836	0,152108836	-1,107891164
chrXII	468545	468696	151	0,259310013	0,085235	0,752615777	0,682744278	0,580332637	0,438501529	0,389501529	-0,870498471
chrXII	468566	468665	99	0,028495606	0,144528914	0,164691143	-0,975358855	-0,829055027	-0,395735727	-0,080735727	-1,340735727
chrXII	468566	468695	129	1,105629506	1,57870044	0,411882864	-0,222704206	-0,189298575	-0,018024667	0,086975333	-1,173024667
chrXII	468566	468710	144	0,045592969	0,040764565	0,527955893	0,070132481	0,059612609	-0,132045818	-0,132045818	-1,392045818
chrXII	468566	468716	150	0,054141651	0,085235	0,388455674	-0,283346352	-0,2408444	-0,401042994	-0,443042994	-1,703042994
chrXII	468567	468695	128	0,216566604	0,222352175	0,493409293	-0,016521205	-0,014043024	0,234434371	0,346434371	-0,913565629
chrXII	468567	468710	143	0,381841118	0,185293479	0,673281299	0,448992035	0,38164323	0,198887661	0,205887661	-1,054112339
chrXII	468567	468716	149	0,567062556	0,470645436	0,546456768	0,116714293	0,099207149	-0,051445114	-0,086445114	-1,346445114
chrXII	468656	468810	154	0,225115286	0,144528914	0,609005325	0,276727507	0,235218381	0,069378358	-0,000621642	-1,260621642
chrXII	468658	468810	152	0,156725832	0,159352392	0,495845079	-0,010415029	-0,008852775	-0,166197731	-0,222197731	-1,482197731
chrXII	468703	468859	156	0,074088575	0,059293913	0,555459536	0,139467258	0,118547169	0,031035157	-0,052964843	-1,312964843
chrXII	468739	468893	154	0,039893848	0,037058696	0,518421434	0,046192108	0,039263292	-0,13375081	-0,20375081	-1,46375081
chrXII	468739	468896	157	0,051292091	0,037058696	0,58055047	0,203301913	0,172806626	0,120305758	0,029305758	-1,230694242
chrXII	468741	468859	118	0,088336378	0,111176087	0,442761197	-0,143972235	-0,122376399	0,274558064	0,456558064	-0,803441936
chrXII	468741	468893	152	5,693422046	3,668810883	0,608126511	0,274439387	0,233273479	0,079078142	0,023078142	-1,236921858
chrXII	468741	468896	155	0,128230226	0,040764565	0,75878212	0,702390353	0,5970318	0,557937161	0,480937161	-0,779062839
chrXII	468741	468911	170	0,387540239	0,26682261	0,592240589	0,233312412	0,19831555	0,359492955	0,177492955	-1,082507045
chrXII	468755	468893	138	0,122531105	0,137117174	0,47191187	-0,070464771	-0,059895055	-0,081623807	-0,039623807	-1,299623807
chrXII	468755	468911	156	0,265009134	0,148234783	0,641289861	0,361908673	0,307622372	0,217518715	0,133518715	-1,126481285
chrXII	468758	468893	135	0,074088575	0,059293913	0,555459536	0,139467258	0,118547169	0,095920266	0,158920266	-1,101079734
chrXII	468762	468893	131	0,045592969	0,107470218	0,297870247	-0,530535801	-0,450955431	-0,337212546	-0,246212546	-1,506212546
chrXII	468762	468911	149	0,042743409	0,048176305	0,470122564	-0,074961771	-0,063717505	-0,216021843	-0,251021843	-1,511021843
chrXII	468765	468893	128	0,299203861	0,452116089	0,39823761	-0,257911486	-0,219224763	0,012968118	0,124968118	-1,135031882
chrXII	468765	468911	146	0,176672756	0,188999349	0,483145293	-0,042261061	-0,035921902	-0,189343424	-0,203343424	-1,463343424
chrXII	468770	468893	123	0,327699467	0,255705001	0,561702018	0,155285859	0,13199298	0,586666765	0,733666765	-0,526333235
chrXII	468770	468911	141	1,755329319	0,926467395	0,654534816	0,397592802	0,337953881	0,276586883	0,297586883	-0,962413117
chrXII	472060	472213	153	0,14817715	0,085235	0,634830492	0,344674598	0,292973409	0,173115235	0,110115235	-1,149884765
chrXII	472461	472545	84	0,094035499	0,077823261	0,547167331	0,118507771	0,100731606	0,451034737	0,871034737	-0,388965263
chrXII	472461	472563	102	0,071239015	0,074117392	0,490098899	-0,024820929	-0,021097789	0,497126199	0,791126199	-0,468873801
chrXII	475711	475864	153	0,111132863	0,085235	0,565942212	0,166052574	0,141144688	0,00894137	-0,05405863	-1,31405863
chrXII	476909	477023	114	0,059840772	0,433586741	0,121275711	-1,168633262	-0,993338272	-0,374773089	-0,164773089	-1,424773089
chrXII	477605	477755	150	0,159575393	0,185293479	0,462713239	-0,093600543	-0,079560461	-0,229104029	-0,271104029	-1,531104029
chrXII	478538	478667	129	0,062690333	0,092646739	0,403576119	-0,244101464	-0,207486244	-0,04839556	0,05660444	-1,20339556

Chrom	Start	End	Length	Section A	Section B	A/A+B	Z-score	Z * 0.85	Phase correction	Length correction	ΔLKnuc
chrXII	479001	479158	157	0,059840772	0,066705652	0,472876041	-0,06804215	-0,057835827	-0,114621638	-0,205621638	**-1,465621638**
chrXII	479187	479337	150	8,705407583	4,109809363	0,679302397	0,465748954	0,395886611	0,252516974	0,210516974	**-1,049483026**
chrXII	479469	479630	161	0,079787696	0,111176087	0,417815854	-0,20748423	-0,176361595	-0,187850609	-0,306850609	**-1,566850609**
chrXII	480195	480349	154	0,202318801	0,144528914	0,583307292	0,210361658	0,178807409	-0,000920335	-0,070920335	**-1,330920335**
chrXII	480476	480621	145	0,062690333	0,066705652	0,484484373	-0,038901718	-0,03306646	-0,18811423	-0,19511423	**-1,45511423**
chrXII	480480	480631	151	0,125380666	0,081529131	0,605967759	0,268824834	0,228501109	0,085107564	0,036107564	**-1,223892436**
chrXII	480687	480827	140	0,170973635	0,111176087	0,605967759	0,268824834	0,228501109	0,283240276	0,311240276	**-0,948759724**
chrXII	480693	480827	134	0,153876272	0,129705435	0,542617058	0,107029113	0,090974746	0,10187273	0,17187273	**-1,08812727**
chrXII	481105	481260	155	0,094035499	0,096352609	0,493914773	-0,015253994	-0,012965895	-0,037396656	-0,114396656	**-1,374396656**
chrXII	481236	481344	108	0,068389454	0,148234783	0,315705458	-0,47974192	-0,407780632	0,189218296	0,441218296	**-0,818781704**
chrXII	481385	481534	149	0,076938136	0,037058696	0,674914686	0,453525163	0,385496389	0,242830799	0,207830799	**-1,052169201**
chrXII	482041	482125	84	0,091185939	0,081529131	0,527955893	0,070132481	0,059612609	0,442060116	0,862060116	**-0,397939884**
chrXII	482041	482143	102	0,088336378	0,111176087	0,442761197	-0,143972235	-0,122376399	0,410409522	0,704409522	**-0,555590478**
chrXII	482885	483037	152	0,042743409	0,051882174	0,451710916	-0,121339881	-0,103138899	-0,250952739	-0,306952739	**-1,566952739**
chrXII	485292	485445	153	0,131079787	0,077823261	0,627467086	0,325152274	0,276379433	0,126261587	0,063261587	**-1,196738413**
chrXII	485693	485777	84	0,088336378	0,08894087	0,498295067	-0,004273647	-0,0036326	0,387667577	0,807667577	**-0,452332423**
chrXII	488944	489097	153	0,159575393	0,066705652	0,705208837	0,539441393	0,458525184	0,292209921	0,229209921	**-1,030790079**
chrXII	489345	489429	84	0,105433742	0,133411305	0,441431561	-0,147340685	-0,125239582	0,261279712	0,681279712	**-0,578720288**
chrXII	489345	489447	102	0,076938136	0,085235	0,474419732	-0,064164224	-0,05453959	0,505240769	0,799240769	**-0,460759231**
chrXII	489345	489466	121	0,04844253	0,074117392	0,395255882	-0,265646117	-0,2257992	0,173375695	0,334375695	**-0,925624305**
chrXII	489345	489469	124	0,661098055	0,744879785	0,470205174	-0,074754118	-0,063541	0,347035325	0,487035325	**-0,772964675**
chrXII	489345	489471	126	0,216566604	0,26311674	0,451478266	-0,12192738	-0,103638273	0,131176332	0,257176332	**-1,002823668**
chrXII	489359	489469	110	0,202318801	0,663350655	0,233713688	-0,726671238	-0,617670553	0,028117089	0,266117089	**-0,993882911**
chrXII	489359	489471	112	0,04844253	0,185293479	0,207253175	-0,815989135	-0,693590765	-0,141352657	0,082647343	**-1,177352657**
chrXII	489361	489466	105	0,108283302	0,481763045	0,183516605	-0,902044538	-0,766737857	-0,254056183	0,018943817	**-1,241056183**
chrXII	489556	489702	146	0,572761677	0,396528045	0,590908646	0,229882972	0,195400527	0,032418859	0,018418859	**-1,241581141**
chrXII	489926	490077	151	0,450230572	0,233469783	0,658520313	0,408428145	0,347163923	0,218092853	0,169092853	**-1,090907147**
chrXII	489933	490080	147	0,039893848	0,037058696	0,518421434	0,046192108	0,039263292	-0,071555735	-0,092555735	**-1,352555735**
chrXII	490009	490164	155	0,199469241	0,148234783	0,573675388	0,185739279	0,157878387	0,12672154	0,04972154	**-1,21027846**
chrXII	490027	490183	156	0,270708255	0,192705218	0,584161383	0,212550948	0,180668306	0,084222592	0,000222592	**-1,259777408**
chrXII	490049	490195	146	0,621204207	0,35576348	0,635849287	0,347385899	0,295278014	0,137110251	0,123110251	**-1,136889749**
chrXII	490049	490215	166	1,216762369	1,271113265	0,489076864	-0,027383708	-0,023276152	0,137859987	-0,016140013	**-1,276140013**
chrXII	490049	490219	170	0,074088575	0,040764565	0,645072262	0,372050197	0,316242667	0,485424364	0,303424364	**-0,956575636**
chrXII	490072	490215	143	0,085486818	0,062999783	0,575720754	0,190958009	0,162314307	-0,015629292	-0,008629292	**-1,268629292**
chrXII	490072	490219	147	0,279256937	0,129705435	0,682842618	0,475662609	0,404313218	0,292508077	0,271508077	**-0,988491923**
chrXII	490083	490239	156	0,564212996	0,374292827	0,6011822	0,256408274	0,217947033	0,131616633	0,047616633	**-1,212383367**
chrXII	490158	490312	154	0,626903328	0,478057176	0,567353608	0,169640608	0,144194517	-0,029112771	-0,099112771	**-1,359112771**
chrXII	490158	490312	154	0,626903328	0,478057176	0,567353608	0,169640608	0,144194517	-0,029112771	-0,099112771	**-1,359112771**
chrXII	490228	490334	106	0,039893848	0,26311674	0,131658264	-1,118586632	-0,950798637	-0,45529239	-0,18929239	**-1,44929239**
chrXII	490228	490337	109	0,04844253	0,389116306	0,110710894	-1,222756213	-1,039342781	-0,34460868	-0,09960868	**-1,35960868**
chrXII	490228	490382	154	0,410336724	0,248293262	0,623015552	0,313410384	0,266398826	0,095747842	0,025747842	**-1,234252158**
chrXII	490261	490368	107	0,116831984	0,867173481	0,118731032	-1,181354153	-1,00415103	-0,443551773	-0,184551773	**-1,444551773**
chrXII	490262	490423	161	0,521469587	0,492880654	0,514092239	0,035331355	0,030031652	0,022093478	-0,096906522	**-1,356906522**
chrXII	490263	490423	160	0,327699467	0,277940218	0,541079911	0,103154718	0,08768151	0,071376015	-0,040623985	**-1,300623985**
chrXII	490267	490423	156	0,19661968	0,151940653	0,564090809	0,161349191	0,137146813	0,052814173	-0,031185827	**-1,291185827**
chrXII	490291	490423	132	0,056991212	0,051882174	0,523463207	0,058847485	0,050020363	0,165441325	0,249441325	**-1,010558675**
chrXII	490302	490409	107	0,037044288	0,096352609	0,277699771	-0,589688448	-0,501235181	0,054717084	0,313717084	**-0,946282916**
chrXII	490302	490423	121	0,031345166	0,051882174	0,376621026	-0,314367347	-0,267212245	0,109482758	0,270482758	**-0,989517242**
chrXII	494931	495079	148	0,849169054	0,544762828	0,609189778	0,277207939	0,235626748	0,099978359	0,071978359	**-1,188021641**
chrXII	496357	496507	150	0,29065518	0,111176087	0,723326439	0,592752021	0,503839218	0,351274434	0,309274434	**-0,950725566**
chrXII	508585	508725	140	0,159575393	0,151940653	0,512254168	0,030721476	0,026113255	0,072767973	0,100767973	**-1,159232027**
chrXII	514529	514679	150	6,665122205	3,072165881	0,684494712	0,480304941	0,4082592	0,257987018	0,215987018	**-1,044012982**
chrXII	518369	518501	132	0,108283302	0,192705218	0,359758911	-0,359103289	-0,305237796	-0,215154497	-0,131154497	**-1,391154497**
chrXII	522434	522581	147	0,336248149	0,214940436	0,610041932	0,279428325	0,237514076	0,134656097	0,113656097	**-1,146343903**
chrXII	528715	528875	160	0,333398588	0,35576348	0,483773852	-0,040684142	-0,03458152	-0,055233078	-0,167233078	**-1,427233078**
chrXII	529249	529419	170	0,102584181	0,211234566	0,326889907	-0,448517424	-0,381239811	-0,220679256	-0,402679256	**-1,662679256**
chrXII	537872	537976	104	0,153876272	0,837526525	0,155210649	-1,014338418	-0,862187655	-0,272899303	0,007100697	**-1,252899303**
chrXII	542120	542273	153	0,29350474	0,377998697	0,437085983	-0,158361478	-0,134607257	-0,305624905	-0,368624905	**-1,628624905**
chrXII	544133	544261	128	0,498673102	0,919055655	0,351740839	-0,380624799	-0,323531079	-0,086353244	0,025646756	**-1,234353244**
chrXII	545204	545351	147	2,530409798	1,67134718	0,602226595	0,259114613	0,220247421	0,119182351	0,098182351	**-1,161817649**
chrXII	546037	546189	152	17,92088651	7,000387634	0,719099931	0,580169768	0,493144303	0,326083951	0,270083951	**-0,989916049**
chrXII	546038	546189	151	0,045592969	0,044470435	0,506231912	0,015621722	0,013278464	-0,124649953	-0,173649953	**-1,433649953**
chrXII	546040	546189	149	0,14817715	0,103764348	0,588141101	0,222765786	0,189350918	0,053605712	0,018605712	**-1,241394288**
chrXII	547890	548039	149	0,059840772	0,037058696	0,617555219	0,299066166	0,254206241	0,113379706	0,078379706	**-1,181620294**
chrXII	553542	553689	147	2,259701543	1,360054135	0,624269079	0,316712396	0,269205536	0,173377474	0,152377474	**-1,107622526**
chrXII	555694	555847	153	0,458779254	0,363175219	0,558156527	0,146296985	0,124352437	-0,050030693	-0,113030693	**-1,373030693**
chrXII	559139	559286	147	0,772230918	0,559586306	0,579832505	0,201465007	0,171245256	0,071316519	0,050316519	**-1,209683481**
chrXII	559606	559755	149	0,176672756	0,066705652	0,725917953	0,600513459	0,51043644	0,375374992	0,340374992	**-0,919625008**
chrXII	560049	560194	145	0,056991212	0,040764565	0,582995843	0,209563572	0,178129036	0,026889769	0,019889769	**-1,240110231**
chrXII	570252	570403	151	0,076938136	0,048176305	0,614942093	0,292223409	0,248389898	0,113954422	0,064954422	**-1,195045578**
chrXII	573533	573669	136	11,95390665	8,386382856	0,587695993	0,221622182	0,188378855	0,219720685	0,275720685	**-0,984279315**
chrXII	576803	576966	163	0,481575739	0,52993935	0,47609348	-0,059960669	-0,050966568	-0,046396479	-0,179396479	**-1,439396479**
chrXII	586445	586571	126	0,769381358	2,542226531	0,23232864	-0,731199541	-0,62151961	-0,369237124	-0,243237124	**-1,503237124**
chrXII	587477	587583	106	0,091185939	0,496586524	0,155138161	-1,014642398	-0,862446038	-0,384130062	-0,118130062	**-1,378130062**
chrXII	588088	588237	149	0,153876272	0,100058479	0,605967759	0,268824834	0,228501109	0,094061789	0,059061789	**-1,200938211**
chrXII	593332	593484	152	0,054141651	0,037058696	0,593656197	0,236960278	0,201416237	0,030555685	-0,025444315	**-1,285444315**
chrXII	593504	593653	149	0,102584181	0,055588044	0,648560019	0,381435717	0,324220359	0,201164322	0,166164322	**-1,093835678**
chrXII	593678	593801	123	0,076938136	0,192705218	0,285332958	-0,567071042	-0,482010386	-0,039554228	0,107445772	**-1,152554228**
chrXII	593778	593933	155	0,079787696	0,077823261	0,506231912	0,015621722	0,013278464	-0,012428874	-0,089428874	**-1,349428874**
chrXII	594211	594345	134	0,128230226	0,122293696	0,51184823	0,029703476	0,025247955	0,029611292	0,099611292	**-1,160388708**
chrXII	594211	594351	140	0,190920559	0,155646522	0,550890634	0,127911851	0,108725073	0,159903297	0,187903297	**-1,072096703**
chrXII	594407	594558	151	0,136778908	0,066705652	0,672183225	0,445949741	0,37905728	0,237150716	0,188150716	**-1,071849284**
chrXII	594417	594562	145	0,122531105	0,066705652	0,64750161	0,378584033	0,321796428	0,155074964	0,148074964	**-1,111925036**
chrXII	594435	594586	151	0,045592969	0,062999783	0,419852784	-0,202270107	-0,171929591	-0,311286771	-0,360286771	**-1,620286771**
chrXII	594689	594843	154	0,210867483	0,155646522	0,575332675	0,189967434	0,161472318	-0,005690177	-0,075690177	**-1,335690177**
chrXII	594723	594867	144	0,051292091	0,037058696	0,58055047	0,203301913	0,172806626	-0,006666298	-0,006666298	**-1,266666298**
chrXII	595254	595389	135	0,068389454	0,070411522	0,492715945	-0,018259434	-0,015520519	-0,044025624	0,018974376	**-1,241025624**
chrXII	595436	595568	132	0,190920559	0,229763914	0,453833149	-0,115982643	-0,098585246	-0,012026385	0,071973615	**-1,188026385**
chrXII	595525	595663	138	0,555664314	0,455821958	0,549354281	0,124030106	0,10542559	0,07087782	0,11287782	**-1,14712218**
chrXII	598015	598129	114	0,028495606	0,229763914	0,110337098	-1,224737354	-1,041026751	-0,411548698	-0,201548698	**-1,461548698**
chrXII	598489	598636	147	0,076938136	0,040764565	0,653664997	0,395234266	0,335949126	0,237455117	0,216455117	**-1,043544883**
chrXII	603237	603400	163	0,131079787	0,074117392	0,638799168	0,355250862	0,301963233	0,303964955	0,170964955	**-1,089035045**
chrXII	607039	607198	159	0,094035499	0,133411305	0,413439528	-0,218706025	-0,185900121	-0,175087145	-0,280087145	**-1,540087145**
chrXII	608189	608339	150	0,051292091	0,137117174	0,272237623	-0,606059495	-0,515150571	-0,668000913	-0,710000913	**-1,970000913**
chrXII	610208	610357	149	1,632798214	1,05617283	0,607220453	0,272081837	0,231269561	0,115719056	0,080719056	**-1,179280944**
chrXII	611103	611232	129	0,259310013	0,163058261	0,613942923	0,289610604	0,246169013	0,392831321	0,497831321	**-0,762168679**

Chrom	Start	End	Length	Section A	Section B	A/A+B	Z-score	Z * 0.85	Phase correction	Length correction	ΔLKnuc
chrXII	614828	614977	149	0,051292091	0,122293696	0,295485544	-0,537429332	-0,456814932	-0,579652591	-0,614652591	**-1,874652591**
chrXII	614828	614997	169	0,344796831	0,737468046	0,318588211	-0,471650294	-0,40090275	-0,2325472	-0,4075472	**-1,6675472**
chrXII	614830	614977	147	0,663947616	0,655938915	0,503033859	0,007604831	0,006464107	-0,097254727	-0,118254727	**-1,378254727**
chrXII	625233	625381	148	0,378991558	0,251999131	0,600629398	0,254976559	0,216730075	0,085676016	0,057676016	**-1,202323984**
chrXII	628912	629085	173	0,153876272	0,403939784	0,275854863	-0,595200097	-0,505920082	-0,305049895	-0,508049895	**-1,768049895**
chrXII	631988	632127	139	0,849169054	0,830114786	0,505673332	0,014221413	0,012088201	0,02209834	0,05709834	**-1,20290166**
chrXII	635940	636108	168	0,299203861	0,444704349	0,402205349	-0,247642923	-0,210496484	-0,115986029	-0,283986029	**-1,543986029**
chrXII	640543	640689	146	0,803576085	0,548468698	0,594341323	0,238726903	0,202917868	0,040885225	0,026885225	**-1,233114775**
chrXII	649877	650024	147	0,059840772	0,085235	0,412479432	-0,221171546	-0,187995814	-0,289065353	-0,310065353	**-1,570065353**
chrXII	649877	650024	147	0,059840772	0,085235	0,412479432	-0,221171546	-0,187995814	-0,289065353	-0,310065353	**-1,570065353**
chrXII	651183	651332	149	0,071239015	0,066705652	0,516431814	0,041200102	0,035020087	-0,094171765	-0,129171765	**-1,389171765**
chrXII	651357	651480	123	0,079787696	0,17788174	0,309651379	-0,496838761	-0,422312947	0,022579957	0,169579957	**-1,090420043**
chrXII	651457	651612	155	0,105433742	0,051882174	0,670203909	0,440476288	0,374404845	0,358145426	0,281145426	**-0,978854574**
chrXII	651890	652024	134	0,136778908	0,118587827	0,535617563	0,08939893	0,07598909	0,079566427	0,149566427	**-1,110433573**
chrXII	651890	652030	140	0,19377012	0,103764348	0,65125268	0,388704654	0,330398956	0,349960031	0,377960031	**-0,882039969**
chrXII	652086	652237	151	0,116831984	0,070411522	0,623957468	0,315891237	0,268507552	0,129742996	0,080742996	**-1,179257004**
chrXII	652096	652241	145	0,119681545	0,051882174	0,69759239	0,517488464	0,439865194	0,281528831	0,274528831	**-0,985471169**
chrXII	652114	652265	151	0,045592969	0,044470435	0,506231912	0,015621722	0,013278464	-0,126100285	-0,175100285	**-1,435100285**
chrXII	652116	652265	149	0,054141651	0,044470435	0,549036667	0,123227859	0,104743681	-0,032548019	-0,067548019	**-1,327548019**
chrXII	652368	652522	154	0,190920559	0,203822827	0,483657398	-0,040976291	-0,034829847	-0,211774625	-0,281774625	**-1,541774625**
chrXII	652618	652786	168	0,051292091	0,048176305	0,515662191	0,039269381	0,033378974	0,121480336	-0,046519664	**-1,306519664**
chrXII	652933	653068	135	0,094035499	0,070411522	0,571828535	0,1810314	0,15387669	0,119354371	0,182354371	**-1,077645629**
chrXII	653087	653248	161	0,068389454	0,066705652	0,506231912	0,015621722	0,013278464	0,009984022	-0,109015978	**-1,369015978**
chrXII	653194	653339	145	0,113982423	0,081529131	0,582995843	0,209563572	0,178129036	0,020927992	0,013927992	**-1,246072008**
chrXII	653561	653718	157	0,062690333	0,059293913	0,51392155	0,034903236	0,029667751	-0,02250691	-0,11350691	**-1,37350691**
chrXII	654052	654181	129	0,071239015	0,118587827	0,375284202	-0,317889966	-0,270206471	-0,126861347	-0,021861347	**-1,281861347**
chrXII	654964	655114	150	0,156725832	0,163058261	0,490098899	-0,024820929	-0,021097789	-0,16402872	-0,20602872	**-1,46602872**
chrXII	656400	656549	149	0,076938136	0,059293913	0,564757972	0,163043664	0,138587115	0,000335264	-0,034664736	**-1,294664736**
chrXII	657729	657887	158	0,219416165	0,322410653	0,404956266	-0,240538873	-0,204458042	-0,234834728	-0,332834728	**-1,592834728**
chrXII	664370	664478	108	0,561363435	2,253168704	0,199451776	-0,843581058	-0,717043899	-0,104143121	0,147856879	**-1,112143121**
chrXII	664370	664482	112	0,418885406	2,468109139	0,145093937	-1,057709561	-0,899053127	-0,329450233	-0,105450233	**-1,365450233**
chrXII	665850	666010	160	0,04844253	0,051882174	0,482857442	-0,042983253	-0,036535765	-0,061175291	-0,173175291	**-1,433175291**
chrXII	675650	675803	153	0,136778908	0,166764131	0,450607955	-0,124125498	-0,105506674	-0,289298274	-0,352298274	**-1,612298274**
chrXII	678419	678585	166	0,162424953	0,192705218	0,457367372	-0,107068364	-0,09100811	0,074658487	-0,079341513	**-1,339341513**
chrXII	684089	684224	135	1,325045671	0,907938047	0,593396925	0,236291928	0,200848138	0,167778999	0,230778999	**-1,029221001**
chrXII	686656	686810	154	0,159575393	0,137117174	0,537847625	0,095012688	0,080760785	-0,091944125	-0,161944125	**-1,421944125**
chrXII	686971	687118	147	0,800726524	0,655938915	0,549698306	0,124899153	0,10616428	0,021618128	0,000618128	**-1,259381872**
chrXII	691050	691199	149	0,19661968	0,244587392	0,445640364	-0,13668381	-0,116181238	-0,248316174	-0,283316174	**-1,543316174**
chrXII	695440	695604	164	0,299203861	0,26682261	0,528604008	0,071761159	0,060996985	0,100991811	-0,039008189	**-1,299008189**
chrXII	698814	698965	151	0,347646391	0,237175653	0,594448165	0,239002467	0,203152097	0,05670905	0,00770905	**-1,25229095**
chrXII	700732	700860	128	0,031345166	0,366881088	0,078711953	-1,413788855	-1,201720527	-0,957816082	-0,845816082	**-2,105816082**
chrXII	700732	700861	129	4,10051768	47,14236691	0,080021211	-1,404928899	-1,194189564	-1,049084361	-0,944084361	**-2,204084361**
chrXII	704633	704781	148	0,119681545	0,066705652	0,642112476	0,3641111	0,309494435	0,174433576	0,146433576	**-1,113566424**
chrXII	704633	704781	148	0,119681545	0,066705652	0,642112476	0,3641111	0,309494435	0,174433576	0,146433576	**-1,113566424**
chrXII	704633	704785	152	15,72957442	9,883554166	0,614121558	0,290077583	0,246565946	0,095678721	0,039678721	**-1,220321279**
chrXII	704633	704785	152	15,72957442	9,883554166	0,614121558	0,290077583	0,246565946	0,095678721	0,039678721	**-1,220321279**
chrXII	704637	704785	148	0,051292091	0,059293913	0,463820816	-0,090812431	-0,077190567	-0,208555695	-0,236555695	**-1,496555695**
chrXII	704738	704878	140	2,017488893	1,156231309	0,635685809	0,346950665	0,294908066	0,29623924	0,32423924	**-0,93576076**
chrXII	707321	707471	150	0,598407723	0,452116089	0,569627948	0,175427028	0,149112974	-0,000650556	-0,042650556	**-1,302650556**
chrXII	707855	708017	162	0,404637603	0,392822175	0,507408165	0,018570584	0,015784996	0,085198503	-0,040801497	**-1,300801497**
chrXII	710806	710956	150	0,210867483	0,111176087	0,654779361	0,398256287	0,338517844	0,176620492	0,134620492	**-1,125379508**
chrXII	713802	713939	137	0,068389454	0,044470435	0,605967759	0,268824834	0,228501109	0,295243657	0,344243657	**-0,915756343**
chrXII	714204	714311	107	2,68713563	14,98653658	0,152041726	-1,027715972	-0,873558576	-0,326109314	-0,067109314	**-1,327109314**
chrXII	714204	714312	108	0,028495606	0,163058261	0,148760274	-1,041765208	-0,885500426	-0,258675365	-0,006675365	**-1,266675365**
chrXII	714794	714945	151	0,139628469	0,188999349	0,424883291	-0,189416258	-0,161003819	-0,305690376	-0,354690376	**-1,614690376**
chrXII	721307	721447	140	0,381841118	0,340940001	0,528294262	0,070982761	0,060335347	0,052746194	0,080746194	**-1,179253806**
chrXII	723297	723432	135	1,880709985	1,612053267	0,538459501	0,09655235	0,082069498	0,044886603	0,107886603	**-1,152113397**
chrXII	726306	726432	126	0,270708255	0,274234349	0,496764711	-0,008109755	-0,006893292	0,234659473	0,360659473	**-0,899340527**
chrXII	727018	727148	130	0,142478029	0,592939133	0,1937377	-0,864204794	-0,734574075	-0,630398558	-0,532398558	**-1,792398558**
chrXII	731448	731612	164	0,339097709	0,370586958	0,47781462	-0,055639195	-0,047293316	0,001616181	-0,138383819	**-1,398383819**
chrXII	731448	731612	164	0,339097709	0,370586958	0,47781462	-0,055639195	-0,047293316	0,001616181	-0,138383819	**-1,398383819**
chrXII	733723	733874	151	0,213717044	0,17417587	0,550969188	0,128110377	0,10889382	-0,043314842	-0,092314842	**-1,352314842**
chrXII	733723	733874	151	0,213717044	0,17417587	0,550969188	0,128110377	0,10889382	-0,043314842	-0,092314842	**-1,352314842**
chrXII	735532	735635	103	0,031345166	1,371171744	0,022349225	-2,007481199	-1,706359019	-1,098022605	-0,811022605	**-2,071022605**
chrXII	736027	736189	162	0,846319493	0,778232612	0,520955586	0,052552043	0,044669236	0,128623762	0,002623762	**-1,257376238**
chrXII	738785	738957	172	0,065539893	0,08894087	0,424259254	-0,191009043	-0,162357687	-0,031648485	-0,227648485	**-1,487648485**
chrXII	740597	740703	106	0,347646391	2,805343271	0,110259287	-1,225150361	-1,041377806	-0,564174977	-0,298174977	**-1,558174977**
chrXII	745846	745998	152	0,04844253	0,077823261	0,383655221	-0,295894775	-0,251510559	-0,386485895	-0,442485895	**-1,702485895**
chrXII	751027	751192	165	0,162424953	0,133411305	0,549036667	0,123227859	0,104743681	0,21539719	0,06839719	**-1,19160281**
chrXII	752922	753058	136	2,259701543	1,137701961	0,665126041	0,426494002	0,362519902	0,402362262	0,458362262	**-0,801637738**
chrXII	753617	753790	173	0,088336378	0,096352609	0,478298027	-0,054425638	-0,046261792	0,16060237	-0,04239763	**-1,30239763**
chrXII	753617	753790	173	0,088336378	0,096352609	0,478298027	-0,054425638	-0,046261792	0,16060237	-0,04239763	**-1,30239763**
chrXII	759427	759579	152	0,595558162	0,43729261	0,576615885	0,193243554	0,164257021	0,025659677	-0,030340323	**-1,290340323**
chrXII	761633	761798	165	0,039893848	0,081529131	0,328552705	-0,443913104	-0,377326138	-0,266856365	-0,413856365	**-1,673856365**
chrXII	762105	762269	164	0,333398588	0,541056958	0,381264193	-0,302162243	-0,256837906	-0,205670916	-0,345670916	**-1,605670916**
chrXII	770786	770938	152	0,128230226	0,188999349	0,404219015	-0,242441566	-0,206075331	-0,332730318	-0,388730318	**-1,648730318**
chrXII	779526	779669	143	1,162620718	0,982055438	0,542096164	0,105716018	0,089858615	-0,078404865	-0,071404865	**-1,331404865**
chrXII	779740	779903	163	0,284956058	0,459527828	0,382756516	-0,298249123	-0,253511755	-0,252898663	-0,385898663	**-1,645898663**
chrXII	781130	781282	152	0,076938136	0,107470218	0,417216109	-0,209020522	-0,177667444	-0,313690566	-0,369690566	**-1,629690566**
chrXII	782565	782713	148	0,09973462	0,062999783	0,61286746	0,286800501	0,243780426	0,106064005	0,078064005	**-1,181935995**
chrXII	783914	784060	146	0,652549374	0,466939567	0,582899348	0,209316331	0,177918881	0,021305907	0,007305907	**-1,252694093**
chrXII	783914	784060	146	0,652549374	0,466939567	0,582899348	0,209316331	0,177918881	0,021305907	0,007305907	**-1,252694093**
chrXII	785535	785685	150	0,661098055	0,381704567	0,633962786	0,342367387	0,291012279	0,134586729	0,092586729	**-1,167413271**
chrXII	786502	786666	164	0,179522317	0,300175436	0,374240479	-0,320642986	-0,272546538	-0,222967527	-0,362967527	**-1,622967527**
chrXII	792530	792669	139	1,937701197	1,289642613	0,600401231	0,254385777	0,216227911	0,239327645	0,274327645	**-0,985672355**
chrXII	797667	797817	150	0,136778908	0,040764565	0,770396711	0,740153964	0,62913087	0,48449507	0,44249507	**-0,81750493**
chrXII	799286	799439	153	0,159575393	0,159352392	0,50034961	0,000876343	0,000744891	-0,179847957	-0,242847957	**-1,502847957**
chrXII	803374	803523	149	0,82922213	0,733762177	0,53053772	0,076621618	0,065128376	-0,072805467	-0,107805467	**-1,367805467**
chrXII	804062	804197	135	0,051292091	0,055588044	0,479902939	-0,050397187	-0,042837609	-0,075608929	-0,012608929	**-1,272608929**
chrXII	804515	804661	146	0,265009134	0,044470435	0,856305749	1,063867998	0,904287798	0,753220225	0,739220225	**-0,520779775**
chrXII	806849	807003	154	0,239363089	0,244587392	0,49460244	-0,013530088	-0,011500575	-0,176225373	-0,246225373	**-1,506225373**
chrXII	807887	808014	127	0,128230226	0,26682261	0,324590066	-0,454901459	-0,386666241	-0,166191765	-0,047191765	**-1,307191765**
chrXII	807887	808014	127	0,128230226	0,26682261	0,324590066	-0,454901459	-0,386666241	-0,166191765	-0,047191765	**-1,307191765**
chrXII	827684	827838	154	6,069564043	4,580454799	0,569911108	0,176147858	0,14972568	-0,002999705	-0,072999705	**-1,332999705**
chrXII	830101	830255	154	0,202318801	0,170470001	0,542716949	0,107280945	0,091188803	-0,04999663	-0,11999663	**-1,37999663**
chrXII	830655	830774	119	0,863416857	3,390870664	0,202952164	-0,831122683	-0,70645428	-0,28550083	-0,11050083	**-1,37050083**

Chrom	Start	End	Length	Section A	Section B	A/A+B	Z-score	Z * 0.85	Phase correction	Length correction	ΔLKnuc
chrXII	830666	830794	128	1,61000173	1,904816963	0,458061104	-0,105319605	-0,089521664	0,149034225	0,261034225	**-0,998965775**
chrXII	831008	831187	179	0,074088575	0,122293696	0,377267126	-0,312666232	-0,265766297	-0,108248547	-0,353248547	**-1,613248547**
chrXII	831542	831700	158	0,116831984	0,096352609	0,548032024	0,120690792	0,102587173	0,072919538	-0,025080462	**-1,285080462**
chrXII	839642	839772	130	0,185221438	0,722644568	0,204018475	-0,827353109	-0,703250143	-0,618652983	-0,520652983	**-1,780652983**
chrXII	840366	840517	151	0,772230918	0,522527611	0,596428524	0,244113455	0,207496437	0,055322049	0,006322049	**-1,253677951**
chrXII	841812	841967	155	0,208017923	0,300175436	0,409328298	-0,229273181	-0,194882204	-0,210434673	-0,287434673	**-1,547434673**
chrXII	842219	842368	149	0,227964847	0,207528696	0,523463207	0,058847485	0,050020363	-0,085688311	-0,120688311	**-1,380688311**
chrXII	846202	846357	155	0,43883233	0,35205761	0,55485891	0,137947158	0,117255084	0,100142879	0,023142879	**-1,236857121**
chrXII	864284	864432	148	0,324849907	0,159352392	0,670897077	0,442391614	0,376032872	0,230370887	0,202370887	**-1,057629113**
chrXII	868597	868734	137	0,039893848	0,051882174	0,434687048	-0,164453569	-0,139785534	-0,068072435	-0,019072435	**-1,279072435**
chrXII	871320	871444	124	0,039893848	0,422469132	0,086282531	-1,364007955	-1,159406762	-0,751475206	-0,611475206	**-1,871475206**
chrXII	871320	871449	129	15,88914981	16,56894289	0,489528142	-0,02625207	-0,02231426	0,113139043	0,218139043	**-1,041860957**
chrXII	871322	871449	127	0,054141651	0,100058479	0,351112876	-0,382317666	-0,324970016	-0,086968948	0,032031052	**-1,227968948**
chrXII	874679	874837	158	0,042743409	0,111176087	0,277699771	-0,589688448	-0,501235181	-0,532568156	-0,630568156	**-1,890568156**
chrXII	878567	878705	138	0,142478029	0,051882174	0,733061742	0,62209939	0,528784481	0,504654047	0,546654047	**-0,713345953**
chrXII	884555	884713	158	0,156725832	0,148234783	0,51392155	0,034903236	0,029667751	-0,003433238	-0,101433238	**-1,361433238**
chrXII	889517	889681	164	0,233663968	0,418763262	0,35814564	-0,363419845	-0,308906868	-0,261222833	-0,401222833	**-1,661222833**
chrXII	893166	893327	161	1,929152515	1,486053701	0,564871458	0,163331944	0,138832153	0,129729637	0,010729637	**-1,249270363**
chrXII	896277	896446	169	0,626903328	0,863467612	0,42063577	-0,200267293	-0,170227199	0,001603494	-0,173396506	**-1,433396506**
chrXII	896668	896822	154	0,09688506	0,118587827	0,449639217	-0,126572917	-0,10758698	-0,244832143	-0,314832143	**-1,574832143**
chrXII	896811	896958	147	0,307752543	0,222352175	0,58055047	0,203301913	0,172806626	0,099295902	0,078295902	**-1,181704098**
chrXII	903630	903761	131	1,12272687	1,174760656	0,488675937	-0,028389029	-0,024130675	0,090431113	0,181431113	**-1,078568887**
chrXII	903784	903945	161	0,074088575	0,066705652	0,52621884	0,065768269	0,055903029	0,04728561	-0,07171439	**-1,33171439**
chrXII	903961	904117	156	1,117027749	0,737468046	0,602335013	0,259395665	0,220486315	0,128206635	0,044206635	**-1,215793365**
chrXII	905180	905335	155	0,031345166	0,059293913	0,345823971	-0,396619676	-0,337126724	-0,360798567	-0,437798567	**-1,697798567**
chrXII	908649	908820	171	0,037044288	0,081529131	0,31241646	-0,489012398	-0,415660538	-0,3049698	-0,4939698	**-1,7539698**
chrXII	912499	912653	154	0,239363089	0,163058261	0,594807131	0,239928434	0,203939169	0,073578333	0,003578333	**-1,256421667**
chrXII	913527	913695	168	0,071239015	0,114881957	0,382756516	-0,298249123	-0,253511755	-0,161973244	-0,329973244	**-1,589973244**
chrXII	922406	922556	150	0,056991212	0,077823261	0,422738082	-0,194893703	-0,165659647	-0,302816867	-0,344816867	**-1,604816867**
chrXII	922509	922654	145	0,658248495	0,418763262	0,611180417	0,282396933	0,240037393	0,081741032	0,074741032	**-1,185258968**
chrXII	924375	924524	149	2,818215417	1,941875659	0,592050734	0,232823413	0,197899901	0,06163309	0,02663309	**-1,23336691**
chrXII	926149	926303	154	0,649699813	0,552174567	0,540572146	0,101875234	0,086593949	-0,037460252	-0,107460252	**-1,367460252**
chrXII	926358	926515	157	1,812320531	1,589818049	0,532700385	0,082059712	0,069750755	0,007308265	-0,083691735	**-1,343691735**
chrXII	929019	929172	153	1,051487855	0,844938264	0,55445759	0,136931651	0,116391904	-0,055424745	-0,118424745	**-1,378424745**
chrXII	931573	931698	125	1,040089613	2,671931966	0,280194926	-0,582262541	-0,49492316	-0,226750099	-0,093750099	**-1,353750099**
chrXII	937960	938108	148	1,427629852	1,093231526	0,566326203	0,167028538	0,141974257	-0,003993363	-0,031993363	**-1,291993363**
chrXII	938740	938880	140	0,367593315	0,374292827	0,495484811	-0,011318142	-0,009620421	-0,014618974	0,013381026	**-1,246618974**
chrXII	939726	939894	168	0,065539893	0,077823261	0,457159956	-0,107591283	-0,091452591	0,007911093	-0,160088907	**-1,420088907**
chrXII	940022	940161	139	0,396088921	0,237175653	0,625471465	0,319882942	0,271900501	0,302831363	0,337831363	**-0,922168637**
chrXII	941909	942015	106	0,09688506	0,396528045	0,196356884	-0,854706289	-0,726500345	-0,230131786	0,035868214	**-1,224131786**
chrXII	941909	942019	110	0,056991212	0,433586741	0,116171572	-1,194344741	-1,01519303	-0,373497851	-0,135497851	**-1,395497851**
chrXII	943265	943394	129	0,182371877	0,396528045	0,315031788	-0,481637369	-0,409391764	-0,273878182	-0,168878182	**-1,428878182**
chrXII	944730	944873	143	0,225115286	0,081529131	0,734124849	0,625336388	0,53153593	0,367010161	0,374010161	**-0,885989839**
chrXII	946105	946219	114	0,034194727	0,244587392	0,122657533	-1,161804056	-0,987533448	-0,336011908	-0,126011908	**-1,386011908**
chrXII	946809	946958	149	0,09688506	0,048176305	0,667890174	0,43409473	0,36898052	0,231551119	0,196551119	**-1,063448881**
chrXII	947096	947245	149	0,09973462	0,155646522	0,390532439	-0,277931585	-0,236241848	-0,366922292	-0,401922292	**-1,661922292**
chrXII	947588	947739	151	0,09688506	0,129705435	0,427577775	-0,182544352	-0,155162699	-0,309389137	-0,358389137	**-1,618389137**
chrXII	952012	952159	147	0,265009134	0,107470218	0,711473355	0,557694096	0,474039982	0,40182985	0,38082985	**-0,87917015**
chrXII	955881	956039	158	0,453080133	0,314998914	0,589887375	0,227255273	0,193166982	0,153564721	0,055564721	**-1,204435279**
chrXII	956586	956713	127	0,085486818	0,229763914	0,271170878	-0,609275633	-0,517884288	-0,284935455	-0,165935455	**-1,425935455**
chrXII	962105	962271	166	0,136778908	0,155646522	0,467739443	-0,080953558	-0,068810524	0,092124662	-0,061875338	**-1,321875338**
chrXII	962105	962271	166	0,136778908	0,155646522	0,467739443	-0,080953558	-0,068810524	0,092124662	-0,061875338	**-1,321875338**
chrXII	966042	966188	146	0,581310359	0,34835174	0,625292092	0,319409753	0,27149829	0,115847183	0,101847183	**-1,158152817**
chrXII	967180	967328	148	0,062690333	0,070411522	0,470995186	-0,072768458	-0,061853189	-0,193968846	-0,221968846	**-1,481968846**
chrXII	967209	967341	132	0,265009134	0,181587609	0,593396925	0,236291928	0,200848138	0,288239532	0,372239532	**-0,887760468**
chrXII	974145	974297	152	0,085486818	0,096352609	0,470122564	-0,074961771	-0,063717505	-0,205288488	-0,261288488	**-1,521288488**
chrXII	975018	975184	166	0,222265726	0,311293045	0,416572153	-0,210676015	-0,179070023	-0,009018085	-0,163018085	**-1,423018085**
chrXII	976449	976598	149	0,094035499	0,066705652	0,585011979	0,214732295	0,182522451	0,062489467	0,027489467	**-1,232510533**
chrXII	978018	978161	143	0,227964847	0,077823261	0,745499386	0,660393679	0,561334627	0,415370943	0,422370943	**-0,837629057**
chrXII	978601	978756	155	0,267858695	0,270528479	0,497520572	-0,006215044	-0,005282788	-0,023556813	-0,100556813	**-1,360556813**
chrXII	979540	979688	148	0,065539893	0,074117392	0,469290903	-0,077052467	-0,065494597	-0,197456149	-0,225456149	**-1,485456149**
chrXII	981009	981111	102	0,122531105	0,140823044	0,465271216	-0,087162393	-0,074088034	0,500014484	0,794014484	**-0,465985516**
chrXII	981009	981139	130	1,681240744	1,84922892	0,476208806	-0,059671071	-0,05072041	0,031882678	0,129882678	**-1,130117322**
chrXII	981073	981227	154	0,168124074	0,133411305	0,557560028	0,144785863	0,123067983	0,006679472	-0,063320528	**-1,323320528**
chrXII	981080	981248	168	0,823523009	0,978349569	0,457037317	-0,107900483	-0,09171541	0,012008773	-0,155991227	**-1,415991227**
chrXII	982426	982590	164	0,567062556	0,511410002	0,525801563	0,06472008	0,055012068	0,101448203	-0,038551797	**-1,298551797**
chrXII	987388	987536	148	1,22246149	0,781938481	0,609888998	0,279029737	0,237175277	0,1015471	0,0735471	**-1,1864529**
chrXII	988804	988961	157	1,729683274	1,597229788	0,519906364	0,049918579	0,042430792	-0,036466686	-0,127466686	**-1,387466686**
chrXII	988850	988961	111	0,037044288	0,070411522	0,344739737	-0,399561558	-0,339627325	0,133368771	0,364368771	**-0,895631229**
chrXII	988957	989104	147	0,128230226	0,074117392	0,633712557	0,341702373	0,290447017	0,210538674	0,189538674	**-1,070461326**
chrXII	990093	990242	149	0,48727486	0,318704784	0,604574649	0,265206091	0,225425178	0,11319889	0,07819889	**-1,18180111**
chrXII	990292	990442	150	0,464478375	0,237175653	0,661976354	0,417862991	0,355183542	0,226933449	0,184933449	**-1,075066551**
chrXII	997620	997777	157	0,330549028	0,374292827	0,468969068	-0,077861613	-0,066182371	-0,129616283	-0,220616283	**-1,480616283**
chrXII	998925	999073	148	0,085486818	0,185293479	0,315705458	-0,47974192	-0,407780632	-0,540157734	-0,568157734	**-1,828157734**
chrXII	1001567	1001716	149	0,205168362	0,244587392	0,456177292	-0,110069087	-0,093558724	-0,199810195	-0,234810195	**-1,494810195**
chrXII	1005509	1005668	159	0,04844253	0,103764348	0,31826768	-0,472548462	-0,401666193	-0,400200535	-0,505200535	**-1,765200535**
chrXII	1005994	1006142	148	0,088336378	0,055588044	0,613769206	0,28915654	0,245783059	0,111123532	0,083123532	**-1,176876468**
chrXII	1012419	1012577	158	0,373292436	0,307587175	0,548250278	0,121241892	0,103055609	0,065425324	-0,032574676	**-1,292574676**
chrXII	1012549	1012696	147	0,14817715	1,93075805	0,071275502	-1,466357184	-1,246403606	-1,32624131	-1,34724131	**-2,60724131**
chrXII	1012550	1012632	82	0,028495606	0,337234132	0,07791438	-1,419241021	-1,206354868	-0,877987772	-0,443987772	**-1,703987772**
chrXII	1012550	1012633	83	0,068389454	0,552174567	0,110205316	-1,225436951	-1,041621408	-0,722418571	-0,295418571	**-1,555418571**
chrXII	1012550	1012634	84	0,042743409	0,192705218	0,181540276	-0,909510814	-0,773084192	-0,389970034	0,030029966	**-1,229970034**
chrXII	1012550	1012635	85	0,051292091	0,155646522	0,247861382	-0,681235065	-0,579049806	-0,203611192	0,209388808	**-1,050611192**
chrXII	1012550	1012636	86	0,116831984	0,140823044	0,453443447	-0,11696613	-0,099421211	0,259644047	0,665644047	**-0,594355953**
chrXII	1012550	1012637	87	0,034194727	0,581821524	0,055509456	-1,593630085	-1,354585572	-0,955592235	-0,556592235	**-1,816592235**
chrXII	1012550	1012638	88	0,034194727	0,185293479	0,155793004	-1,011899709	-0,860114752	-0,42009307	-0,02809307	**-1,28809307**
chrXII	1012550	1012639	89	0,065539893	0,314998914	0,172229197	-0,945392764	-0,80358385	-0,374917554	0,010082446	**-1,249917554**
chrXII	1012550	1012640	90	0,028495606	0,214940436	0,117055822	-1,189834	-1,0113589	-0,550514376	-0,172514376	**-1,432514376**
chrXII	1012550	1012641	91	0,091185939	0,503998263	0,153206249	-1,022778687	-0,869361884	-0,50332261	-0,13232261	**-1,39232261**
chrXII	1012550	1012642	92	0,128230226	0,344645871	0,271170878	-0,609275633	-0,517884288	-0,117001793	0,246998207	**-1,013001793**
chrXII	1012550	1012643	93	0,062690333	0,333528262	0,158221582	-1,001793859	-0,85152478	-0,365010483	-0,008010483	**-1,268010483**
chrXII	1012550	1012644	94	0,079787696	0,415057393	0,161237725	-0,989383693	-0,840976139	-0,324401513	0,025598487	**-1,234401513**
chrXII	1012550	1012645	95	0,056991212	0,626291959	0,083407896	-1,382507947	-1,175131755	-0,642710548	-0,299710548	**-1,559710548**
chrXII	1012550	1012646	96	0,042743409	0,188999349	0,184443343	-0,898560728	-0,763776618	-0,208476066	0,127523934	**-1,132476066**
chrXII	1012550	1012648	98	0,128230226	0,737468046	0,148123463	-1,044515554	-0,887838221	-0,402709012	-0,080709012	**-1,340709012**
chrXII	1012550	1012649	99	0,074088575	0,251999131	0,227204441	-0,748085011	-0,635872259	-0,223547102	0,091452898	**-1,168547102**

Chrom	Start	End	Length	Section A	Section B	A/A+B	Z-score	Z * 0.85	Phase correction	Length correction	ΔLKnuc
chrXII	1012550	1012650	100	0,102584181	0,244587392	0,295485544	-0,537429332	-0,456814932	-0,026978077	0,281021923	**-0,978978077**
chrXII	1012550	1012651	101	0,065539893	0,08894087	0,424259254	-0,191009043	-0,162357687	0,357675864	0,658675864	**-0,601324136**
chrXII	1012550	1012652	102	0,056991212	0,944996743	0,056878141	-1,581532732	-1,344302822	-0,766895924	-0,472895924	**-1,732895924**
chrXII	1012550	1012653	103	0,094035499	0,429880871	0,179485705	-0,917326814	-0,779727792	-0,20060965	0,08639035	**-1,17360965**
chrXII	1012550	1012654	104	0,04844253	0,218646305	0,181372351	-0,910147548	-0,773625416	-0,174786672	0,105213328	**-1,154786672**
chrXII	1012550	1012655	105	0,082637257	0,103764348	0,443329106	-0,142534017	-0,121153915	0,4006554	0,6736554	**-0,5863446**
chrXII	1012550	1012656	106	0,042743409	0,418763262	0,092617098	-1,324809941	-1,12608845	-0,64226122	-0,37626122	**-1,63626122**
chrXII	1012550	1012657	107	0,037044288	0,140823044	0,208269204	-0,812441383	-0,690575176	-0,140816806	0,118183194	**-1,141816806**
chrXII	1012550	1012659	109	0,074088575	0,270528479	0,214988127	-0,789232287	-0,670847444	0,029133906	0,274133906	**-0,985866094**
chrXII	1012550	1012660	110	0,054141651	0,107470218	0,335010364	-0,426119559	-0,362201625	0,268441436	0,506441436	**-0,753558564**
chrXII	1012550	1012661	111	0,165274514	0,515115871	0,242911301	-0,696968348	-0,592423096	-0,115849445	0,115150555	**-1,144849445**
chrXII	1012550	1012662	112	0,116831984	0,248293262	0,31997783	-0,467760794	-0,397596675	0,168799096	0,392799096	**-0,867200904**
chrXII	1012550	1012663	113	0,133929347	0,444704349	0,231457912	-0,734054005	-0,623945904	-0,061667559	0,155332441	**-1,104667559**
chrXII	1012550	1012664	114	0,162424953	1,385995222	0,104897208	-1,25413094	-1,066011299	-0,396563429	-0,186563429	**-1,446563429**
chrXII	1012550	1012665	115	0,199469241	0,885702829	0,183813467	-0,900927376	-0,765788269	-0,165845338	0,037154662	**-1,222845338**
chrXII	1012550	1012666	116	0,156725832	0,744879785	0,173829698	-0,939138975	-0,798268129	-0,210760103	-0,014760103	**-1,274760103**
chrXII	1012550	1012667	117	0,14532759	0,296469566	0,328946413	-0,442824299	-0,376400654	0,110346368	0,299346368	**-0,960653632**
chrXII	1012550	1012668	118	0,076938136	0,240881523	0,24208111	-0,699623888	-0,594680304	-0,210907071	-0,028907071	**-1,288907071**
chrXII	1012550	1012669	119	0,213717044	0,377998697	0,361181948	-0,355301282	-0,302006089	0,104291854	0,279291854	**-0,980708146**
chrXII	1012550	1012670	120	0,062690333	0,144528914	0,30253142	-0,517133686	-0,439563633	-0,055419989	0,112580011	**-1,147419989**
chrXII	1012550	1012671	121	0,159575393	0,715232829	0,182411858	-0,906211882	-0,7702801	-0,392951922	-0,231951922	**-1,491951922**
chrXII	1012550	1012672	122	0,111132863	0,392822175	0,220521385	-0,770433532	-0,654868502	-0,252985062	-0,098985062	**-1,358985062**
chrXII	1012550	1012673	123	0,119681545	0,396528045	0,231846806	-0,732778376	-0,62286162	-0,188851291	-0,041851291	**-1,301851291**
chrXII	1012550	1012674	124	0,208017923	5,399451976	0,037096574	-1,785420424	-1,517607361	-1,11299362	-0,97299362	**-2,23299362**
chrXII	1012550	1012675	125	0,190920559	0,503998263	0,274737931	-0,598545721	-0,508763863	-0,242276971	-0,109276971	**-1,369276971**
chrXII	1012550	1012676	126	0,205168362	0,544762828	0,273582916	-0,602012476	-0,511710605	-0,256116407	-0,130116407	**-1,390116407**
chrXII	1012550	1012677	127	0,122531105	0,559586306	0,179633452	-0,916762889	-0,779248455	-0,547013805	-0,428013805	**-1,688013805**
chrXII	1012550	1012678	128	0,125380666	0,163058261	0,434687048	-0,164453569	-0,139785534	0,094263704	0,206263704	**-1,053736296**
chrXII	1012550	1012679	129	0,227964847	0,155646522	0,594259882	0,238516864	0,202739335	0,339398156	0,444398156	**-0,815601844**
chrXII	1012550	1012680	130	0,185221438	0,222352175	0,454449042	-0,114428548	-0,097264266	-0,012603453	0,085396547	**-1,174603453**
chrXII	1012550	1012681	131	0,185221438	0,359469349	0,340048781	-0,412330002	-0,350480502	-0,220263522	-0,129263522	**-1,389263522**
chrXII	1012550	1012682	132	0,364743755	0,35205761	0,508849135	0,022183312	0,018855816	0,079626403	0,163626403	**-1,096373597**
chrXII	1012550	1012683	133	0,330549028	0,715232829	0,316078373	-0,478693422	-0,406889409	-0,418493863	-0,341493863	**-1,601493863**
chrXII	1012550	1012684	134	0,484425299	0,359469349	0,574035278	0,186657164	0,15865859	0,162535143	0,232535143	**-1,027464857**
chrXII	1012550	1012685	135	0,626903328	0,496586524	0,557996432	0,14589138	0,124007673	0,091603725	0,154603725	**-1,105396275**
chrXII	1012550	1012686	136	0,319150785	0,392822175	0,448262509	-0,13005234	-0,110544489	-0,058704946	-0,002704946	**-1,262704946**
chrXII	1012550	1012687	137	0,236513528	0,396528045	0,373614528	-0,322295218	-0,273950935	-0,19086849	-0,14186849	**-1,40186849**
chrXII	1012550	1012688	138	0,968850598	0,770820872	0,55691584	0,143154302	0,121681156	0,088853174	0,130853174	**-1,129146826**
chrXII	1012550	1012689	139	0,307752543	0,240881523	0,56094319	0,153360973	0,130356827	0,161771863	0,196771863	**-1,063228137**
chrXII	1012550	1012690	140	0,720938828	0,559586306	0,563002481	0,158586028	0,134798124	0,135767377	0,163767377	**-1,096232623**
chrXII	1012550	1012691	141	0,504372223	0,604056741	0,455033421	-0,112954231	-0,096011096	-0,156176989	-0,135176989	**-1,395176989**
chrXII	1012550	1012692	142	0,492973981	0,359469349	0,578307042	0,197564368	0,167929713	0,132131801	0,146131801	**-1,113868199**
chrXII	1012550	1012693	143	3,881101515	2,316168487	0,626259872	0,321963618	0,273669075	0,156021178	0,163021178	**-1,096978822**
chrXII	1012550	1012694	144	0,626903328	0,61517435	0,504721516	0,011835361	0,010060057	-0,171926774	-0,171926774	**-1,431926774**
chrXII	1012550	1012695	145	0,618354647	0,607762611	0,50431934	0,010827192	0,009203114	-0,146781992	-0,153781992	**-1,413781992**
chrXII	1012550	1012696	146	799,1792126	406,4227167	0,662888133	0,420358291	0,357304547	0,203411957	0,189411957	**-1,070588043**
chrXII	1012550	1012698	148	0,239363089	0,289057827	0,452978074	-0,118140734	-0,100419624	-0,237375093	-0,265375093	**-1,525375093**
chrXII	1012551	1012693	142	0,031345166	0,044470435	0,413439528	-0,218706025	-0,185900121	-0,243015829	-0,229015829	**-1,489015829**
chrXII	1012551	1012696	145	0,666797177	1,434171527	0,317376063	-0,47504889	-0,403791556	-0,559416557	-0,566416557	**-1,826416557**
chrXII	1012552	1012696	144	0,58415992	0,844938264	0,408761222	-0,230732731	-0,196122822	-0,380072941	-0,380072941	**-1,640072941**
chrXII	1012553	1012693	140	0,179522317	0,08894087	0,668703665	0,436336407	0,370885946	0,384897455	0,412897455	**-0,847102545**
chrXII	1012553	1012696	143	1,820869213	2,631167401	0,408996909	-0,230126057	-0,195607148	-0,312814544	-0,305814544	**-1,565814544**
chrXII	1012554	1012696	142	1,111328628	0,559586306	0,665101858	0,426427614	0,362463472	0,305303686	0,319303686	**-0,940696314**
chrXII	1012555	1012696	141	1,205364127	1,096937395	0,523547466	0,059059058	0,050200199	0,00488396	0,02588396	**-1,23411604**
chrXII	1012556	1012696	140	0,259310013	0,289057827	0,472876041	-0,06804215	-0,057835827	-0,067232068	-0,039232068	**-1,299232068**
chrXII	1012557	1012696	139	0,287805619	0,859761742	0,250796275	-0,671986094	-0,57118818	-0,537170494	-0,502170494	**-1,762170494**
chrXII	1012558	1012696	138	0,404637603	0,763409133	0,346422442	-0,394997295	-0,335747701	-0,365648443	-0,323648443	**-1,583648443**
chrXII	1012559	1012696	137	0,606956404	0,433586741	0,583307292	0,210361658	0,178807409	0,264351079	0,313351079	**-0,946648921**
chrXII	1012560	1012696	136	0,515770466	0,759703264	0,404375609	-0,242037356	-0,205731753	-0,15937568	-0,10337568	**-1,36337568**
chrXII	1012561	1012696	135	0,302053422	0,344645871	0,467069356	-0,082638847	-0,07024302	-0,098297759	-0,035297759	**-1,295297759**
chrXII	1012562	1012696	134	0,122531105	0,533645219	0,186735029	-0,88999223	-0,756493395	-0,748357556	-0,678357556	**-1,938357556**
chrXII	1012563	1012696	133	0,094035499	0,148234783	0,388142938	-0,284162468	-0,241538098	-0,249966431	-0,172966431	**-1,432966431**
chrXII	1012564	1012696	132	0,09688506	0,989467177	0,089183836	-1,345797995	-1,143928296	-1,083462671	-0,999462671	**-2,259462671**
chrXII	1012565	1012696	131	0,179522317	0,763409133	0,190387453	-0,876469399	-0,744998989	-0,616833694	-0,525833694	**-1,785833694**
chrXII	1012566	1012696	130	0,102584181	0,270528479	0,274941571	-0,597935244	-0,508244957	-0,421583607	-0,323583607	**-1,583583607**
chrXII	1012567	1012696	129	0,079787696	0,804173699	0,090261517	-1,339146351	-1,138274398	-1,003811696	-0,898811696	**-2,158811696**
chrXII	1012568	1012696	128	0,054141651	0,433586741	0,11100779	-1,221186061	-1,038008152	-0,801151486	-0,689151486	**-1,949151486**
chrXII	1012569	1012696	127	0,039893848	0,403939784	0,089884689	-1,341465454	-1,140245636	-0,911981344	-0,792981344	**-2,052981344**
chrXII	1012570	1012696	126	0,076938136	0,596645002	0,114222182	-1,204375793	-1,023719424	-0,782861355	-0,656861355	**-1,916861355**
chrXII	1012571	1012696	125	0,056991212	0,96352609	0,055845414	-1,590639059	-1,3520432	-1,090038942	-0,957038942	**-2,217038942**
chrXII	1012572	1012696	124	0,054141651	0,244587392	0,181239998	-0,910649661	-0,774052212	-0,367194401	-0,227194401	**-1,487194401**
chrXII	1012573	1012696	123	0,056991212	0,622586089	0,083862736	-1,379549252	-1,172616864	-0,758224647	-0,611224647	**-1,871224647**
chrXII	1012574	1012696	122	0,059840772	0,61888022	0,088166968	-1,352129359	-1,149309955	-0,745573068	-0,591573068	**-1,851573068**
chrXII	1012575	1012696	121	0,071239015	0,111176087	0,390532439	-0,277931585	-0,236241848	0,147413407	0,308413407	**-0,951586593**
chrXII	1012576	1012696	120	0,039893848	0,733762177	0,051565356	-1,629861787	-1,385382519	-1,005664905	-0,837664905	**-2,097664905**
chrXII	1012578	1012696	118	0,031345166	1,004290656	0,030266592	-1,876889917	-1,59535643	-1,204261357	-1,022261357	**-2,282261357**
chrXII	1012579	1012696	117	0,079787696	0,426175002	0,15769482	-1,003977126	-0,853380557	-0,365830513	-0,176830513	**-1,436830513**
chrXII	1012580	1012696	116	0,079787696	1,749170441	0,043624671	-1,710089407	-1,453575996	-0,883610656	-0,687610656	**-1,947610656**
chrXII	1012581	1012696	115	0,059840772	0,874585221	0,064040141	-1,5217159	-1,293458515	-0,679134278	-0,476134278	**-1,736134278**
chrXII	1012583	1012696	113	0,068389454	0,181587609	0,273582916	-0,602012476	-0,511710605	0,080090552	0,297090552	**-0,962909448**
chrXII	1012584	1012696	112	0,039893848	1,100643265	0,034978124	-1,812193861	-1,540364782	-0,972328572	-0,748328572	**-2,008328572**
chrXII	1012586	1012696	110	0,042743409	0,074117392	0,365763444	-0,34309514	-0,291630869	0,315619961	0,553619961	**-0,706380039**
chrXII	1012590	1012674	84	0,151026711	0,333528262	0,311681273	-0,49109035	-0,417426797	-0,010573336	0,409426664	**-0,850573336**
chrXII	1012610	1012696	86	0,045592969	0,515115871	0,081313092	-1,396293324	-1,186849326	-0,8260582	-0,4200582	**-1,6800582**
chrXII	1013868	1014024	156	3,946641408	2,223521747	0,639633233	0,357478614	0,303856822	0,213645184	0,129645184	**-1,130354816**
chrXII	1017667	1017816	149	0,823523009	0,941290873	0,46663448	-0,083732696	-0,071172792	-0,183527849	-0,218527849	**-1,478527849**
chrXII	1023621	1023767	146	2,479117708	1,589818049	0,609279147	0,277440736	0,235824626	0,076106146	0,062106146	**-1,197893854**
chrXII	1024132	1024289	157	0,136778908	0,170470001	0,445172966	-0,137866493	-0,117186519	-0,171310166	-0,262310166	**-1,522310166**
chrXII	1031294	1031445	151	0,726637949	0,552174567	0,568213041	0,171826517	0,14605254	-0,006034471	-0,055034471	**-1,315034471**
chrXII	1032438	1032586	148	0,569912117	0,548468698	0,509586815	0,024032894	0,02042796	-0,13057051	-0,15857051	**-1,41857051**
chrXII	1050400	1050528	128	0,205168362	0,522527611	0,281942418	-0,577080856	-0,490518728	-0,254878192	-0,142878192	**-1,402878192**
chrXII	1050732	1050875	143	0,470177496	0,767115003	0,380005129	-0,305467316	-0,259647219	-0,381285902	-0,374285902	**-1,634285902**
chrXII	1059679	1059820	141	0,051292091	0,062999783	0,448781606	-0,128740216	-0,109429183	-0,158733471	-0,137733471	**-1,397733471**
chrXII	1065029	1065154	125	0,262159574	0,096352609	0,731243138	0,61657707	0,524090509	0,776247047	0,909247047	**-0,350752953**
chrXII	1065029	1065156	127	0,527168708	0,166764131	0,759682607	0,705281957	0,599489663	0,837994918	0,956994918	**-0,303005082**
chrXII	1066703	1066854	151	0,225115286	0,196411088	0,534047927	0,085449369	0,072631964	-0,084222444	-0,133222444	**-1,393222444**

Chrom	Start	End	Length	Section A	Section B	A/A+B	Z-score	Z * 0.85	Phase correction	Length correction	ΔLKnuc
chrXII	1067646	1067808	162	0,051292091	0,040764565	0,557179598	0,143822286	0,122248943	0,199753013	0,073753013	-1,186246987
chrXII	1067747	1067902	155	0,071239015	0,188999349	0,273745245	-0,601524812	-0,51129609	-0,515097459	-0,592097459	-1,852097459
chrXII	1067881	1068018	137	0,14532759	0,137117174	0,51453455	0,036440779	0,030974662	0,118210849	0,167210849	-1,092789151
chrXII	1068176	1068319	143	0,430283648	0,377998697	0,532343247	0,081161514	0,068987287	-0,049361921	-0,042361921	-1,302361921
chrXII	1068224	1068349	125	0,210867483	0,274234349	0,434687048	-0,164453569	-0,139785534	0,105327729	0,238327729	-1,021672271
chrXII	1068449	1068601	152	0,09973462	0,062999783	0,61286746	0,286800501	0,243780426	0,102815557	0,046815557	-1,213184443
chrXII	1068522	1068666	144	0,105433742	0,070411522	0,599582493	0,252266586	0,214426598	0,028991948	0,028991948	-1,231008052
chrXII	1068619	1068754	135	1,077133901	0,778232612	0,58055047	0,203301913	0,172806626	0,147419865	0,210419865	-1,049580135
chrXII	1068619	1068806	187	0,125380666	0,055588044	0,692830634	0,503889924	0,428306435	0,477878216	0,176878216	-1,083121784
chrXII	1068662	1068754	92	0,133929347	0,100058479	0,572377417	0,18243015	0,155065628	0,529950635	0,893950635	-0,366049365
chrXII	1068662	1068806	144	1,310797868	0,518821741	0,716431908	0,572274263	0,486433123	0,311268149	0,311268149	-0,948731851
chrXII	1069001	1069153	152	0,156725832	0,129705435	0,547167331	0,118507771	0,100731606	-0,035882452	-0,091882452	-1,351882452
chrXII	1069302	1069432	130	0,398938482	0,44099848	0,474962408	-0,062801193	-0,053381014	0,044228196	0,142228196	-1,117771804
chrXII	1069440	1069601	161	0,199469241	0,133411305	0,599221683	0,251333038	0,213633083	0,205239575	0,086239575	-1,173760425
chrXII	1070837	1070988	151	0,549965193	0,389116306	0,585641601	0,216347614	0,183895472	0,034970085	-0,014029915	-1,274029915
chrXII	1070837	1071008	171	0,04844253	0,044470435	0,521375354	0,053605728	0,045564869	0,170299862	-0,018700138	-1,278700138
chrXII	1072226	1072381	155	0,324849907	0,455821958	0,416115812	-0,211840305	-0,180064259	-0,171760617	-0,248760617	-1,508760617
chrXII	1073391	1073538	147	1,726833714	1,193290004	0,591356354	0,23103541	0,196380098	0,119017326	0,098017326	-1,161982674
chrXII	1073547	1073677	130	0,29350474	0,274234349	0,516971168	0,042553249	0,036170262	0,112570725	0,210570725	-1,049429275
chrXII	1073928	1074064	136	0,527168708	0,674468263	0,438708795	-0,154243695	-0,131107141	-0,096139293	-0,040139293	-1,300139293
chrXII	1074309	1074471	162	0,04844253	0,040764565	0,543034495	0,108081541	0,09186931	0,169631887	0,043631887	-1,216368113
chrXII	1074410	1074565	155	0,059840772	0,188999349	0,240478794	-0,704763239	-0,599048753	-0,592018038	-0,669018038	-1,929018038
chrXII	1074544	1074681	137	0,136778908	0,129705435	0,513271836	0,033273697	0,028282642	0,111572837	0,160572837	-1,099427163
chrXII	1074831	1074982	151	0,051292091	0,059293913	0,463820816	-0,090812431	-0,077190567	-0,211081729	-0,260081729	-1,520081729
chrXII	1074839	1074982	143	0,427434088	0,43729261	0,494299631	-0,014289193	-0,012145814	-0,134980516	-0,127980516	-1,387980516
chrXII	1074887	1075012	125	0,256460453	0,289057827	0,470122564	-0,074961771	-0,063717505	0,175775758	0,308775758	-0,951224242
chrXII	1075112	1075264	152	0,113982423	0,085235	0,572150875	0,181852797	0,154574878	0,016323256	-0,039676744	-1,299676744
chrXII	1075185	1075329	144	0,076938136	0,070411522	0,522146688	0,055542058	0,047210749	-0,133480009	-0,133480009	-1,393480009
chrXII	1075282	1075417	135	1,205364127	0,770820872	0,609944984	0,279175646	0,237299299	0,210835569	0,273835569	-0,986164431
chrXII	1075282	1075469	187	0,151026711	0,066705652	0,693634647	0,506179412	0,4302525	0,472808164	0,171808164	-1,088191836
chrXII	1075325	1075417	92	0,182371877	0,125999566	0,591403262	0,231156174	0,196482748	0,570784137	0,934784137	-0,325215863
chrXII	1075325	1075469	144	1,353541277	0,61146848	0,688821657	0,492513068	0,418636108	0,231961775	0,231961775	-1,028038225
chrXII	1075664	1075816	152	0,199469241	0,122293696	0,619926094	0,305286692	0,259493688	0,134750185	0,078750185	-1,181249815
chrXII	1075965	1076095	130	0,413186285	0,415057393	0,498870436	-0,0028314	-0,00240669	0,075874338	0,173874338	-1,086125662
chrXII	1076103	1076264	161	0,247911771	0,207528696	0,544334087	0,111358804	0,094654983	0,089054602	-0,029945398	-1,289945398
chrXII	1077160	1077312	152	0,042743409	0,040764565	0,51184823	0,029703476	0,025247955	-0,083093427	-0,139093427	-1,399093427
chrXII	1077500	1077651	151	0,541416511	0,385410436	0,584161383	0,212550948	0,180668306	0,043131073	-0,005868927	-1,265868927
chrXIII	687	858	171	0,045592969	0,048176305	0,486225044	-0,034535557	-0,029355224	0,265977604	0,076977604	-1,183022396
chrXIII	707	858	151	0,58700948	0,340940001	0,632587756	0,338714936	0,287907696	0,143290712	0,094290712	-1,165709288
chrXIII	1046	1198	152	0,045592969	0,051882174	0,467739443	-0,080953558	-0,068810524	-0,164095132	-0,220095132	-1,480095132
chrXIII	1151	1299	148	0,062690333	0,059293913	0,51392155	0,034903236	0,029667751	-0,151779997	-0,179779997	-1,439779997
chrXIII	1836	2023	187	0,071239015	0,040764565	0,6360423	0,347899847	0,29571487	0,650428694	0,349428694	-0,910571306
chrXIII	1872	2023	151	0,473027057	0,289057827	0,620701272	0,307323098	0,261224633	0,12022764	0,07122764	-1,18877236
chrXIII	1884	2018	134	0,572761677	0,489174784	0,539355882	0,098811125	0,083989456	0,15113177	0,22113177	-1,03886823
chrXIII	1909	2005	96	0,039893848	0,103764348	0,277699771	-0,589688448	-0,501235181	-0,033176331	0,302823669	-0,957176331
chrXIII	2542	2694	152	0,165274514	0,107470218	0,605967759	0,268824834	0,228501109	0,139978606	0,083978606	-1,176021394
chrXIII	2889	3033	144	1,342143035	0,626291959	0,681832542	0,472829373	0,401904967	0,256471872	0,256471872	-1,003528128
chrXIII	2889	3076	187	0,119681545	0,081529131	0,594807131	0,239928434	0,203939169	0,557059861	0,256059861	-1,003940139
chrXIII	2941	3033	92	0,208017923	0,137117174	0,602714486	0,260379534	0,221322604	0,624703275	0,988703275	-0,271296725
chrXIII	2941	3076	135	1,199665006	0,711526959	0,627705133	0,325781423	0,27691421	0,337671475	0,400671475	-0,859328525
chrXIII	3029	3173	144	0,059840772	0,048176305	0,553993628	0,135757811	0,11539414	-0,034580427	-0,034580427	-1,294580427
chrXIII	3094	3246	152	0,125380666	0,085235	0,595305506	0,241214353	0,2050322	0,12159943	0,06559943	-1,19440057
chrXIII	3346	3471	125	0,225115286	0,303881305	0,425551487	-0,187711297	-0,159554603	0,299345477	0,432345477	-0,827654523
chrXIII	3376	3519	143	0,376141997	0,43729261	0,46241209	-0,094358752	-0,080204939	-0,196081815	-0,189081815	-1,449081815
chrXIII	3376	3527	151	0,054141651	0,066705652	0,448017039	-0,130672891	-0,111071958	-0,247775214	-0,296775214	-1,556775214
chrXIII	5624	5767	143	0,296354301	0,207528696	0,588141101	0,222765786	0,189350918	0,05900657	0,06600657	-1,19399343
chrXIII	9070	9207	137	0,105433742	0,092646739	0,53227729	0,080995639	0,068842293	0,074698835	0,123698835	-1,136301165
chrXIII	13123	13267	144	0,498673102	0,370586958	0,573675388	0,185739279	0,157878387	0,001693055	0,001693055	-1,258306945
chrXIII	14333	14505	172	0,074088575	0,188999349	0,281611463	-0,578061014	-0,491351862	-0,188543528	-0,384543528	-1,644543528
chrXIII	16025	16179	154	1,008744447	0,781938481	0,56332946	0,159416068	0,135503658	0,046243023	-0,023756977	-1,283756977
chrXIII	18573	18744	171	0,598407723	1,096937395	0,352971036	-0,377311574	-0,320714838	-0,035644976	-0,224644976	-1,484644976
chrXIII	19858	19999	141	1,629948654	1,133996091	0,589718248	0,226820268	0,192797228	0,121333724	0,142333724	-1,117666276
chrXIII	20186	20333	147	0,806425645	0,61888022	0,565791291	0,165669032	0,140818677	-0,039687818	-0,060687818	-1,320687818
chrXIII	21593	21740	147	0,871965539	0,566998046	0,605967759	0,268824834	0,228501109	0,042672996	0,021672996	-1,238327004
chrXIII	23095	23242	147	0,230814407	0,144528914	0,614942093	0,292223409	0,248389898	0,059233146	0,038233146	-1,221766854
chrXIII	32413	32558	145	0,24221265	0,181587609	0,571525487	0,180259276	0,153220385	-0,008997143	-0,015997143	-1,275997143
chrXIII	33232	33384	152	0,09688506	0,070411522	0,579121574	0,199646764	0,16969975	0,072463432	0,016463432	-1,243536568
chrXIII	38992	39143	151	0,125380666	0,100058479	0,556161913	0,141245282	0,12005849	-0,019742332	-0,068742332	-1,328742332
chrXIII	40472	40624	152	1,045788734	0,926467395	0,53024996	0,075898212	0,06451348	-0,027492743	-0,083492743	-1,343492743
chrXIII	42972	43133	161	0,63260245	0,637409568	0,498107452	-0,004743933	-0,004032343	0,069202662	-0,049797338	-1,309797338
chrXIII	47095	47254	159	0,909009826	0,685585872	0,570056615	0,176518304	0,150040559	0,237685581	0,132685581	-1,127314419
chrXIII	54675	54757	82	1,820869213	2,916519358	0,384361381	-0,294045989	-0,249939091	0,072235478	0,506235478	-0,753764522
chrXIII	54913	55020	107	0,074088575	0,403939784	0,154987824	-1,015273133	-0,862982163	-0,172871325	0,086128675	-1,173871325
chrXIII	54913	55032	119	0,082637257	0,455821958	0,153469854	-1,021664524	-0,868414845	-0,239745926	-0,064745926	-1,324745926
chrXIII	59231	59396	165	0,162424953	0,155646522	0,510655515	0,026712592	0,022705704	0,263807945	0,116807945	-1,143192055
chrXIII	63883	64021	138	0,393239361	0,303881305	0,564090809	0,161349191	0,137146813	0,158086866	0,200086866	-1,059913134
chrXIII	64235	64393	158	0,042743409	0,096352609	0,307294266	-0,503534497	-0,428004322	-0,347670368	-0,445670368	-1,705670368
chrXIII	64871	65018	147	0,407487163	0,303881305	0,572821514	0,183562133	0,156027813	-0,027217953	-0,048217953	-1,308217953
chrXIII	66681	66829	148	0,116831984	0,074117392	0,61184795	0,284138687	0,241517884	0,070515368	0,042515368	-1,217484632
chrXIII	75788	75939	151	0,267858695	0,211234566	0,559095101	0,148675368	0,126374063	-0,008183113	-0,057183113	-1,317183113
chrXIII	76732	76885	153	2,19701121	2,093816312	0,512025058	0,030146916	0,025624878	-0,068942299	-0,131942299	-1,391942299
chrXIII	77591	77728	137	0,068389454	0,070411522	0,492715945	-0,018259434	-0,015520519	0,00337707	0,05237707	-1,20762293
chrXIII	83978	84137	159	0,626903328	0,578115654	0,520243529	0,050764798	0,043150079	0,149088721	0,044088721	-1,215911279
chrXIII	93745	93871	126	0,068389454	0,214940436	0,241377477	-0,701878473	-0,596596702	-0,148405246	-0,022405246	-1,282405246
chrXIII	98827	98976	149	0,350495952	0,118587827	0,747192651	0,665681504	0,565829278	0,431325885	0,396325885	-0,863674115
chrXIII	102493	102629	136	0,598407723	0,422469132	0,58617033	0,217704522	0,185048844	0,20021136	0,25621136	-1,00378864
chrXIII	104088	104231	143	1,421930731	0,926467395	0,605489638	0,267582473	0,227445102	0,103169044	0,110169044	-1,149830956
chrXIII	104601	104708	107	0,125380666	0,692997611	0,153206249	-1,022778687	-0,869361884	-0,190806582	0,068193418	-1,191806582
chrXIII	104601	104720	119	0,24221265	1,130290221	0,17647515	-0,92888189	-0,789549606	-0,177640637	-0,002640637	-1,262640637
chrXIII	107860	108019	159	0,504372223	0,422469132	0,544183986	0,110980226	0,094333192	0,193405712	0,088405712	-1,171594288
chrXIII	109020	109176	156	1,12272687	1,030231743	0,521480935	0,053870763	0,045790149	0,090124794	0,006124794	-1,253875206
chrXIII	112712	112852	140	0,911859387	0,937585003	0,493045042	-0,017434378	-0,014819221	-0,012036558	0,015963442	-1,244036558
chrXIII	113210	113365	155	0,068389454	0,044470435	0,605967759	0,268824834	0,228501109	0,164884161	0,087884161	-1,172115839
chrXIII	113210	113365	155	0,068389454	0,044470435	0,605967759	0,268824834	0,228501109	0,164884161	0,087884161	-1,172115839
chrXIII	116680	116830	150	0,034194727	0,051882174	0,397257877	-0,260451199	-0,221383519	-0,374344701	-0,416344701	-1,676344701
chrXIII	116680	116833	153	0,079787696	0,062999783	0,558786364	0,147892925	0,125708986	0,03145303	-0,03154697	-1,29154697

Chrom	Start	End	Length	Section A	Section B	A/A+B	Z-score	Z * 0.85	Phase correction	Length correction	ΔLKnuc
chrXIII	116680	116835	155	16,6784781	10,31714091	0,617821658	0,299764647	0,25479995	0,195429563	0,118429563	**-1,141570437**
chrXIII	116684	116835	151	0,065539893	0,059293913	0,525017182	0,062749932	0,053337442	-0,085129796	-0,134129796	**-1,394129796**
chrXIII	116783	116928	145	0,091185939	0,048176305	0,654308775	0,396979674	0,337432723	0,172376419	0,165376419	**-1,094623581**
chrXIII	117349	117491	142	1,780975365	1,304466092	0,577218978	0,194784006	0,165566405	0,073633234	0,087633234	**-1,172366766**
chrXIII	117514	117618	104	4,505155283	40,30133167	0,100546943	-1,278441255	-1,086675066	-0,552456473	-0,272456473	**-1,532456473**
chrXIII	119216	119393	177	0,24221265	0,455821958	0,346992322	-0,393453388	-0,33443538	0,03299322	-0,19800678	**-1,45800678**
chrXIII	120074	120243	169	0,470177496	1,026525873	0,314142071	-0,484143344	-0,411521842	-0,118893484	-0,293893484	**-1,553893484**
chrXIII	120074	120243	169	0,470177496	1,026525873	0,314142071	-0,484143344	-0,411521842	-0,118893484	-0,293893484	**-1,553893484**
chrXIII	123583	123746	163	0,652549374	0,904232177	0,419165665	-0,20402837	-0,173424114	-0,014894304	-0,147894304	**-1,407894304**
chrXIII	124934	125083	149	0,273557816	0,200116957	0,577522451	0,195559327	0,166225428	0,030863675	-0,004136325	**-1,264136325**
chrXIII	125456	125612	156	1,689789426	1,393406962	0,548064156	0,120771925	0,102656136	0,142102089	0,058102089	**-1,201897911**
chrXIII	126339	126468	129	0,045592969	0,037058696	0,551627959	0,129775456	0,110309137	0,452252663	0,557252663	**-0,702747337**
chrXIII	129825	129930	105	2,154267801	17,96605572	0,107069243	-1,242265865	-1,055925985	-0,489714441	-0,216714441	**-1,476714441**
chrXIII	129825	129932	107	1,307948308	9,509261339	0,120913651	-1,17043166	-0,994866911	-0,28524102	-0,02624102	**-1,28624102**
chrXIII	129843	129930	87	0,068389454	0,233469783	0,226560746	-0,750221201	-0,637688021	-0,305659195	0,093340805	**-1,166659195**
chrXIII	129843	130004	161	0,817823887	0,629997828	0,564865051	0,163315669	0,138818318	0,216212545	0,097212545	**-1,162787455**
chrXIII	129843	130004	161	0,817823887	0,629997828	0,564865051	0,163315669	0,138818318	0,216212545	0,097212545	**-1,162787455**
chrXIII	130886	131027	141	6,288980208	3,761457622	0,625741914	0,320596525	0,272507046	0,203352203	0,224352203	**-1,035647797**
chrXIII	131028	131190	162	0,125380666	0,081529131	0,605967759	0,268824834	0,228501109	0,333154984	0,207154984	**-1,052845016**
chrXIII	131602	131755	153	0,359044634	0,326116523	0,524029464	0,060269401	0,051228991	-0,047976245	-0,110976245	**-1,370976245**
chrXIII	133367	133536	169	0,176672756	0,300175436	0,370501051	-0,330526594	-0,280947605	0,016410295	-0,158589705	**-1,418589705**
chrXIII	134348	134447	99	0,327699467	2,116051529	0,134096914	-1,107231555	-0,941146822	-0,484663408	-0,169663408	**-1,429663408**
chrXIII	135575	135725	150	0,042743409	0,037058696	0,535617563	0,08939893	0,07598909	-0,079659658	-0,121659658	**-1,381659658**
chrXIII	139632	139738	106	0,133929347	1,208113483	0,099795137	-1,282719764	-1,0903118	-0,450777998	-0,184777998	**-1,444777998**
chrXIII	139632	139741	109	0,088336378	1,456406744	0,057185157	-1,578850666	-1,342023066	-0,633734967	-0,388734967	**-1,648734967**
chrXIII	142450	142583	133	1,134125112	10,75813939	0,095366624	-1,308413054	-1,112151096	-1,032482142	-0,955482142	**-2,215482142**
chrXIII	147977	148129	152	0,265009134	0,196411088	0,574333594	0,187418125	0,159305406	0,061820708	0,005820708	**-1,254179292**
chrXIII	148322	148434	112	1,336443914	10,60619873	0,111905208	-1,216458222	-1,033989488	-0,345900204	-0,121900204	**-1,381900204**
chrXIII	154231	154388	157	0,085486818	0,118587827	0,418899751	-0,204708983	-0,174002636	-0,1377113	-0,2287113	**-1,4887113**
chrXIII	155621	155784	163	0,037044288	0,037058696	0,499902783	-0,000243688	-0,000207135	0,157913263	0,024913263	**-1,235086737**
chrXIII	163164	163259	95	0,427434088	1,775111528	0,194063671	-0,863018416	-0,733565653	-0,280794011	0,062205989	**-1,197794011**
chrXIII	169237	169345	108	0,065539893	0,733762177	0,081996401	-1,391767538	-1,183002408	-0,458604463	-0,206604463	**-1,466604463**
chrXIII	170327	170483	156	0,065539893	0,08894087	0,424259254	-0,191009043	-0,162357687	-0,125530991	-0,209530991	**-1,469530991**
chrXIII	170574	170680	106	0,082637257	0,107470218	0,434687048	-0,164453569	-0,139785534	0,514602502	0,780602502	**-0,479397498**
chrXIII	170574	170696	122	0,068389454	0,081529131	0,456177292	-0,110069087	-0,093558724	0,467199798	0,621199798	**-0,638800202**
chrXIII	170574	170696	122	0,068389454	0,081529131	0,456177292	-0,110069087	-0,093558724	0,467199798	0,621199798	**-0,638800202**
chrXIII	171072	171220	148	0,592708601	0,478057176	0,553537117	0,134603005	0,114412554	-0,059544467	-0,087544467	**-1,347544467**
chrXIII	175762	175915	153	1,145523355	0,896820438	0,560886643	0,153217556	0,130234923	0,033738532	-0,029261468	**-1,289261468**
chrXIII	179143	179321	178	1,128425991	2,160521964	0,343096335	-0,404027263	-0,343423174	0,012339021	-0,225660979	**-1,485660979**
chrXIII	180747	180905	158	0,131079787	0,081529131	0,616530051	0,29638	0,251923	0,342010068	0,244010068	**-1,015989932**
chrXIII	181711	181851	140	2,356586603	1,712111745	0,579199145	0,199845122	0,169868354	0,16196119	0,18996119	**-1,07003881**
chrXIII	181848	181977	129	0,04844253	0,092646739	0,343346664	-0,403346515	-0,342844538	0,030213249	0,135213249	**-1,124786751**
chrXIII	184182	184330	148	0,051292091	0,040764565	0,557179598	0,143822286	0,122248943	-0,052931298	-0,080931298	**-1,340931298**
chrXIII	184701	184824	123	0,088336378	0,185293479	0,322831649	-0,459795102	-0,390825837	0,125433804	0,272433804	**-0,987566196**
chrXIII	184801	184956	155	0,076938136	0,100058479	0,434687048	-0,164453569	-0,139785534	-0,201963216	-0,278963216	**-1,538963216**
chrXIII	185234	185368	134	0,153876272	0,081529131	0,653664997	0,395234266	0,335949126	0,404001973	0,474001973	**-0,785998027**
chrXIII	185234	185374	140	0,168124074	0,125999566	0,571610206	0,180475116	0,153403848	0,147504313	0,175504313	**-1,084495687**
chrXIII	186277	186412	135	0,082637257	0,055588044	0,597844653	0,247772164	0,210606339	0,28176275	0,34476275	**-0,91523725**
chrXIII	186724	186874	150	8,810841324	4,124632841	0,68113787	0,470883041	0,400250585	0,240242633	0,198242633	**-1,061757367**
chrXIII	186903	187060	157	0,054141651	0,081529131	0,399066404	-0,255764342	-0,217399691	-0,176474579	-0,267474579	**-1,527474579**
chrXIII	187135	187284	149	0,079787696	0,055588044	0,589379577	0,225949308	0,192056912	0,057636305	0,022636305	**-1,237363695**
chrXIII	187394	187523	129	0,034194727	0,066705652	0,338895921	-0,415478239	-0,353156503	0,008667179	0,113667179	**-1,146332821**
chrXIII	188306	188456	150	0,444531451	0,381704567	0,538019388	0,095446574	0,081129588	-0,071263247	-0,113263247	**-1,373263247**
chrXIII	195171	195314	143	0,210867483	0,148234783	0,58720733	0,220367006	0,187311955	0,046659925	0,053659925	**-1,206340075**
chrXIII	196534	196661	127	0,031345166	0,107470218	0,225804702	-0,752734617	-0,639824425	-0,208408121	-0,089408121	**-1,349408121**
chrXIII	196694	196843	149	0,071239015	0,070411522	0,502920895	0,007321663	0,006223413	-0,141177973	-0,176177973	**-1,436177973**
chrXIII	196868	196991	123	0,076938136	0,233469783	0,247861382	-0,681235065	-0,579049806	-0,054746496	0,092253504	**-1,167746496**
chrXIII	196968	197123	155	0,062690333	0,092646739	0,403576119	-0,244101464	-0,207486244	-0,271269175	-0,348269175	**-1,608269175**
chrXIII	197401	197535	134	0,14817715	0,085235	0,634830492	0,344674598	0,292973409	0,323602132	0,393602132	**-0,866397868**
chrXIII	197401	197541	140	0,179522317	0,081529131	0,687689413	0,48931151	0,415914784	0,402413702	0,430413702	**-0,829586298**
chrXIII	197597	197748	151	0,113982423	0,066705652	0,630824269	0,334037235	0,283931649	0,14708664	0,09808664	**-1,16191336**
chrXIII	197607	197752	145	0,113982423	0,077823261	0,594259882	0,238516864	0,202739335	0,016292599	0,009292599	**-1,250707401**
chrXIII	197625	197776	151	0,059840772	0,059293913	0,502295129	0,005753068	0,004890107	-0,138382794	-0,187382794	**-1,447382794**
chrXIII	197879	198033	154	0,225115286	0,159352392	0,585524607	0,216047418	0,183640306	0,095573711	0,025573711	**-1,234426289**
chrXIII	197913	198057	144	0,062690333	0,048176305	0,56545715	0,164819952	0,140096959	-0,013608373	-0,013608373	**-1,273608373**
chrXIII	198891	199037	146	0,079787696	0,037058696	0,682842618	0,475662609	0,404313218	0,208799295	0,194799295	**-1,065200705**
chrXIII	198891	199041	150	8,882080339	4,328455668	0,672348218	0,446406602	0,379445612	0,233985211	0,191985211	**-1,068014789**
chrXIII	199070	199227	157	0,054141651	0,040764565	0,570475287	0,177584338	0,150946687	0,195883468	0,104883468	**-1,155116532**
chrXIII	199302	199451	149	0,09973462	0,051882174	0,657807209	0,406485945	0,345513053	0,201150587	0,166150587	**-1,093849413**
chrXIII	199561	199690	129	0,04844253	0,085235	0,36238349	-0,352095051	-0,299280793	0,038738439	0,143738439	**-1,116261561**
chrXIII	200473	200623	150	0,165274514	0,196411088	0,456956299	-0,108104753	-0,09188904	-0,235549481	-0,277549481	**-1,537549481**
chrXIII	203778	203929	151	0,031345166	0,059293913	0,345823971	-0,396619676	-0,337126724	-0,477825043	-0,526825043	**-1,786825043**
chrXIII	204102	204256	154	0,823523009	0,61517435	0,572408786	0,182510101	0,155133586	0,062922211	-0,007077789	**-1,267077789**
chrXIII	208554	208676	122	0,085486818	0,062999783	0,575720754	0,190958009	0,162314307	0,720592252	0,874592252	**-0,385407748**
chrXIII	211138	211313	175	4,134712407	3,642869796	0,531619249	0,079340866	0,067439736	0,413215776	0,196215776	**-1,063784224**
chrXIII	218290	218401	111	0,24221265	1,841817181	0,116223216	-1,194080622	-1,014968529	-0,318884584	-0,087884584	**-1,347884584**
chrXIII	220837	220983	146	0,091185939	0,040764565	0,691061693	0,498861988	0,42403269	0,237761285	0,223761285	**-1,036238715**
chrXIII	228866	229000	134	0,350495952	0,314998914	0,526669655	0,066900784	0,056865666	0,089086098	0,159086098	**-1,100913902**
chrXIII	230938	231091	153	0,122531105	0,118587827	0,508177041	0,020498237	0,017423501	-0,078564521	-0,141564521	**-1,401564521**
chrXIII	231257	231408	151	0,190920559	0,17417587	0,522931872	0,057513369	0,048886364	-0,097040535	-0,146040535	**-1,406040535**
chrXIII	233569	233719	150	0,695292782	0,455821958	0,604016922	0,263758316	0,224194569	0,077530362	0,035530362	**-1,224469638**
chrXIII	240347	240506	159	0,071239015	0,185293479	0,277699771	-0,589688448	-0,501235181	-0,410706464	-0,515706464	**-1,775706464**
chrXIII	242945	243091	146	1,285151823	0,837526525	0,605438796	0,267450388	0,22733283	0,050367355	0,036367355	**-1,223632645**
chrXIII	243502	243662	160	0,04844253	0,070411522	0,407579962	-0,23377466	-0,198708461	-0,139479903	-0,251479903	**-1,511479903**
chrXIII	243825	243980	155	2,843861462	2,382874139	0,544098971	0,11076581	0,094150939	0,064556371	-0,012443629	**-1,272443629**
chrXIII	244965	245130	165	0,122531105	0,203822827	0,375454662	-0,317440575	-0,269824489	-0,012863435	-0,159863435	**-1,419863435**
chrXIII	244965	245130	165	0,122531105	0,203822827	0,375454662	-0,317440575	-0,269824489	-0,012863435	-0,159863435	**-1,419863435**
chrXIII	245559	245710	151	0,356195073	0,170470001	0,676321805	0,457437813	0,388822141	0,248959311	0,199959311	**-1,060040689**
chrXIII	246032	246179	147	0,142478029	0,17788174	0,444743825	-0,138952542	-0,118109661	-0,300607109	-0,321607109	**-1,581607109**
chrXIII	246042	246200	158	0,501522663	0,503998263	0,498768996	-0,003085674	-0,002622823	0,094767343	-0,003232657	**-1,263232657**
chrXIII	246354	246509	155	0,29350474	0,100058479	0,745762628	0,661214529	0,56203235	0,52102062	0,44402062	**-0,81597938**
chrXIII	246617	246737	120	0,322000346	8,071383942	0,038363589	-1,769999775	-1,504499809	-0,922758173	-0,754758173	**-2,014758173**
chrXIII	250435	250548	113	0,185221438	0,192705218	0,490098899	-0,024820929	-0,021097789	0,628743846	0,845743846	**-0,414256154**
chrXIII	250507	250631	124	0,037044288	1,093231526	0,032774556	-1,841494599	-1,565270409	-1,064232096	-0,924232096	**-2,184232096**
chrXIII	250507	250662	155	0,039893848	0,114881957	0,257752484	-0,65028992	-0,552746432	-0,585632252	-0,662632252	**-1,922632252**
chrXIII	250507	250664	157	0,028495606	0,251999131	0,101590519	-1,2725408	-1,08165968	-1,027686658	-1,118686658	**-2,378686658**

Chrom	Start	End	Length	Section A	Section B	A/A+B	Z-score	Z * 0.85	Phase correction	Length correction	ΔLKnuc
chrXIII	250507	250665	158	0,042743409	0,188999349	0,184443343	-0,898560728	-0,763776618	-0,671478431	-0,769478431	**-2,029478431**
chrXIII	250507	250666	159	0,04844253	0,307587175	0,136063169	-1,098178991	-0,933452143	-0,83565512	-0,94065512	**-2,20065512**
chrXIII	250507	250667	160	0,059840772	0,111176087	0,349911537	-0,385559308	-0,327725412	-0,280154651	-0,392154651	**-1,652154651**
chrXIII	250507	250668	161	0,054141651	0,188999349	0,222675942	-0,763186995	-0,648708946	-0,567263119	-0,686263119	**-1,946263119**
chrXIII	250507	250669	162	0,059840772	0,144528914	0,292806499	-0,545204323	-0,463423675	-0,353441696	-0,479441696	**-1,739441696**
chrXIII	250507	250670	163	0,037044288	0,26311674	0,123414715	-1,158084807	-0,984372086	-0,815768783	-0,948768783	**-2,208768783**
chrXIII	250507	250671	164	0,054141651	0,251999131	0,176852135	-0,927428169	-0,788313944	-0,572065587	-0,712065587	**-1,972065587**
chrXIII	250507	250673	166	0,082637257	0,385410436	0,176557343	-0,928564773	-0,789280057	-0,496430155	-0,650430155	**-1,910430155**
chrXIII	250507	250674	167	0,065539893	0,125999566	0,342174368	-0,406536101	-0,345555686	-0,061753129	-0,222753129	**-1,482753129**
chrXIII	250507	250675	168	0,074088575	0,255705001	0,224651359	-0,756578012	-0,64309131	-0,346771304	-0,514771304	**-1,774771304**
chrXIII	250507	250676	169	0,031345166	0,144528914	0,178225048	-0,922150465	-0,783827895	-0,497912245	-0,672912245	**-1,932912245**
chrXIII	250507	250677	170	0,031345166	0,218646305	0,125384943	-1,148481403	-0,976209192	-0,706264149	-0,888264149	**-2,148264149**
chrXIII	250507	250678	171	44,76944633	62,04366848	0,419138103	-0,20409891	-0,173484073	0,113141412	-0,075858588	**-1,335858588**
chrXIII	250507	250718	211	0,042743409	0,678174133	0,059290288	-1,560759205	-1,326645324	-0,992214476	-1,461214476	**-2,721214476**
chrXIII	250507	250718	211	0,042743409	0,678174133	0,059290288	-1,560759205	-1,326645324	-0,992214476	-1,461214476	**-2,721214476**
chrXIII	250508	250678	170	0,116831984	0,096352609	0,548032024	0,120690792	0,102587173	0,405013329	0,223013329	**-1,036986671**
chrXIII	250509	250678	169	0,037044288	0,122293696	0,232488743	-0,730675332	-0,621074032	-0,33337862	-0,50837862	**-1,76837862**
chrXIII	250510	250678	168	0,054141651	0,122293696	0,30686397	-0,50475925	-0,429045363	-0,119810362	-0,287810362	**-1,547810362**
chrXIII	250511	250678	167	0,14817715	0,466939567	0,240892738	-0,703433756	-0,597918692	-0,316106909	-0,477106909	**-1,737106909**
chrXIII	250514	250678	164	0,085486818	0,181587609	0,320086122	-0,467457987	-0,397339289	-0,183573622	-0,323573622	**-1,583573622**
chrXIII	250515	250678	163	0,039893848	0,085235	0,318822147	-0,470995015	-0,400345763	-0,22930085	-0,36230085	**-1,62230085**
chrXIII	250566	250718	152	6,109457891	3,668810883	0,624799546	0,318110779	0,270394162	0,167575663	0,111575663	**-1,148424337**
chrXIII	253372	253532	160	0,09973462	0,044470435	0,691616672	0,500438064	0,425372355	0,476347675	0,364347675	**-0,895652325**
chrXIII	256009	256161	152	0,071239015	0,077823261	0,477914444	-0,055388588	-0,0470803	-0,143542201	-0,199542201	**-1,459542201**
chrXIII	260961	261102	141	0,43883233	0,277940218	0,612233729	0,285145669	0,242373819	0,167502451	0,188502451	**-1,071497549**
chrXIII	261845	261977	132	0,205168362	0,474351306	0,301931455	-0,518853495	-0,441025471	-0,290420039	-0,206420039	**-1,466420039**
chrXIII	262094	262251	157	0,079787696	0,062999783	0,558786364	0,147892925	0,125708986	0,197831156	0,106831156	**-1,153168844**
chrXIII	266226	266377	151	0,094035499	0,08894087	0,51392155	0,034903236	0,029667751	-0,120313389	-0,169313389	**-1,429313389**
chrXIII	271419	271566	147	0,236513528	0,122293696	0,659166015	0,4101881	0,348659885	0,172854195	0,151854195	**-1,108145805**
chrXIII	273047	273197	150	0,136778908	0,133411305	0,506231912	0,015621722	0,013278464	-0,126081353	-0,168081353	**-1,428081353**
chrXIII	276628	276785	157	0,131079787	0,185293479	0,414320049	-0,216446021	-0,183979117	-0,100674262	-0,191674262	**-1,451674262**
chrXIII	276628	276785	157	0,131079787	0,185293479	0,414320049	-0,216446021	-0,183979117	-0,100674262	-0,191674262	**-1,451674262**
chrXIII	277879	278014	135	2,348037921	1,686170658	0,582031859	0,207094214	0,176030082	0,24984962	0,31284962	**-0,94715038**
chrXIII	279195	279369	174	0,726637949	0,844938264	0,462362527	-0,094483544	-0,080311013	0,246884606	0,036884606	**-1,223115394**
chrXIII	283987	284078	91	0,059840772	0,159352392	0,273004738	-0,603750588	-0,513188	-0,126905132	0,244094868	**-1,015905132**
chrXIII	283987	284079	92	3,690180956	10,82855091	0,254166892	-0,66143438	-0,562219223	-0,132006025	0,231993975	**-1,028006025**
chrXIII	289854	289961	107	0,581310359	1,897405224	0,234520799	-0,724039316	-0,615433419	0,105027903	0,364027903	**-0,895972097**
chrXIII	289854	289977	123	0,507221784	2,008581312	0,201614262	-0,835869143	-0,710488771	-0,178630713	-0,031630713	**-1,291630713**
chrXIII	303791	303918	127	0,795027403	1,775111528	0,309332462	-0,497743386	-0,423081878	-0,015799892	0,103200108	**-1,156799892**
chrXIII	304476	304622	146	0,407487163	0,329822392	0,552667682	0,132404146	0,112543524	-0,067575043	-0,081575043	**-1,341575043**
chrXIII	310201	310351	150	0,074088575	0,066705652	0,52621884	0,065768269	0,055903029	-0,086518271	-0,128518271	**-1,388518271**
chrXIII	313026	313180	154	0,119681545	0,085235	0,584050178	0,21226584	0,180425964	0,092247352	0,022247352	**-1,237752648**
chrXIII	324504	324669	165	0,054141651	0,096352609	0,359758911	-0,359103289	-0,305237796	-0,049600636	-0,196600636	**-1,456600636**
chrXIII	325807	325958	151	0,205168362	0,137117174	0,599407045	0,251812611	0,214040719	0,055786457	0,006786457	**-1,253213543**
chrXIII	333017	333169	152	1,182567642	0,61888022	0,656453993	0,40280455	0,342383867	0,246147724	0,190147724	**-1,069852276**
chrXIII	335127	335272	145	0,076938136	0,055588044	0,58055047	0,203301913	0,172806626	-0,015986666	-0,022986666	**-1,282986666**
chrXIII	335472	335613	141	0,156725832	0,163058261	0,490098899	-0,024820929	-0,021097789	-0,107087722	-0,086087722	**-1,346087722**
chrXIII	336952	337113	161	2,422126496	2,004875442	0,547125691	0,118402661	0,100642262	0,188959843	0,069959843	**-1,190040157**
chrXIII	340438	340585	147	0,105433742	0,118587827	0,47064103	-0,073658577	-0,06260979	-0,229910328	-0,250910328	**-1,510910328**
chrXIII	342150	342302	152	0,361894194	0,237175653	0,604093489	0,26395704	0,224363484	0,127941461	0,071941461	**-1,188058539**
chrXIII	348582	348731	149	0,527168708	0,389116306	0,575332675	0,189967434	0,161472318	0,017542935	-0,017457065	**-1,277457065**
chrXIII	351853	352022	169	0,14532759	0,159352392	0,476984372	-0,057723664	-0,049065114	0,24778352	0,07278352	**-1,18721648**
chrXIII	351863	352007	144	1,604302609	1,612053267	0,498795118	-0,003020196	-0,002567166	-0,163567584	-0,163567584	**-1,423567584**
chrXIII	357010	357124	114	0,042743409	0,329822392	0,11472714	-1,2017658	-1,02150093	-0,388113404	-0,178113404	**-1,438113404**
chrXIII	357475	357580	105	0,034194727	0,43729261	0,072525229	-1,457238859	-1,23865303	-0,650157197	-0,377157197	**-1,637157197**
chrXIII	358631	358781	150	0,404637603	0,314998914	0,562280531	0,156753732	0,133240672	-0,000298543	-0,042298543	**-1,302298543**
chrXIII	358907	359055	148	1,114178188	0,896820438	0,554042242	0,135880798	0,115498679	-0,058182852	-0,086182852	**-1,346182852**
chrXIII	360027	360184	157	0,04844253	0,081529131	0,372716096	-0,324668217	-0,275967985	-0,184050948	-0,275050948	**-1,535050948**
chrXIII	360404	360549	145	0,122531105	0,059293913	0,673895739	0,450696199	0,383091769	0,202268778	0,195268778	**-1,064731222**
chrXIII	360675	360810	135	0,102584181	0,059293913	0,633712557	0,341702373	0,290447017	0,348176929	0,411176929	**-0,848823071**
chrXIII	362078	362238	160	0,091185939	0,081529131	0,527955893	0,070132481	0,059612609	0,102262385	-0,009737615	**-1,269737615**
chrXIII	362592	362706	114	0,042743409	0,311293045	0,12073166	-1,171337061	-0,995636502	-0,366220804	-0,156220804	**-1,416220804**
chrXIII	374780	374911	131	0,076938136	0,100058479	0,434687048	-0,164453569	-0,139785534	0,047066863	0,138066863	**-1,121933137**
chrXIII	375262	375391	129	0,045592969	0,114881957	0,28411273	-0,570666901	-0,485066866	-0,16465933	-0,05965933	**-1,31965933**
chrXIII	375501	375650	149	0,102584181	0,051882174	0,664119905	0,423733486	0,360173463	0,218772432	0,183772432	**-1,076227568**
chrXIII	375725	375882	157	0,031345166	0,062999783	0,332240005	-0,433736208	-0,368675777	-0,28827456	-0,37927456	**-1,63927456**
chrXIII	375911	376061	150	9,201231124	3,78369284	0,70860878	0,54932499	0,466926241	0,349628586	0,307628586	**-0,952371414**
chrXIII	376373	376508	135	0,074088575	0,051882174	0,588141101	0,222765786	0,189350918	0,245031815	0,308031815	**-0,951968185**
chrXIII	376763	376917	154	0,031345166	0,037058696	0,458236793	-0,104876779	-0,089145262	-0,18158548	-0,25158548	**-1,51158548**
chrXIII	377411	377551	140	0,153876272	0,159352392	0,49125859	-0,021913219	-0,018626236	-0,038495708	-0,010495708	**-1,270495708**
chrXIII	377417	377551	134	0,153876272	0,114881957	0,572545341	0,182858153	0,15542943	0,208079393	0,278079393	**-0,981920607**
chrXIII	377829	377984	155	0,09973462	0,074117392	0,573675388	0,185739279	0,157878387	0,142738506	0,065738506	**-1,194261494**
chrXIII	377960	378068	108	0,065539893	0,140823044	0,317595273	-0,474433869	-0,403268789	0,30193043	0,55393043	**-0,70606957**
chrXIII	379716	379822	106	0,128230226	0,35576348	0,264941929	-0,628183315	-0,533955818	0,129468459	0,395468459	**-0,864531541**
chrXIII	379716	379838	122	0,088336378	0,426175002	0,171689843	-0,94750858	-0,805382293	-0,250189495	-0,096189495	**-1,356189495**
chrXIII	381135	381283	148	0,119681545	0,062999783	0,655138356	0,399230614	0,339346022	0,163368206	0,135368206	**-1,124631794**
chrXIII	383859	384012	153	0,48727486	0,43729261	0,527030071	0,067806264	0,057635325	-0,040344256	-0,103344256	**-1,363344256**
chrXIII	384143	384283	140	0,136778908	0,140823044	0,492715945	-0,018259434	-0,015520519	-0,024686057	0,003313943	**-1,256686057**
chrXIII	384143	384283	140	0,136778908	0,140823044	0,492715945	-0,018259434	-0,015520519	-0,024686057	0,003313943	**-1,256686057**
chrXIII	384959	385107	148	0,24221265	0,155646522	0,60878991	0,276166509	0,234741533	0,051523716	0,023523716	**-1,236476284**
chrXIII	386345	386477	132	0,128230226	0,114881957	0,527452901	0,068868618	0,058538325	0,210376561	0,294376561	**-0,965623439**
chrXIII	386345	386477	132	0,128230226	0,114881957	0,527452901	0,068868618	0,058538325	0,210376561	0,294376561	**-0,965623439**
chrXIII	387854	388024	170	0,475876617	0,648527176	0,423225731	-0,193648063	-0,164600853	0,128647487	-0,053352513	**-1,313352513**
chrXIII	395268	395412	144	1,393435125	1,067290439	0,566270024	0,166885743	0,141852882	-0,021700963	-0,021700963	**-1,281700963**
chrXIII	400158	400292	134	0,045592969	0,114881957	0,28411273	-0,570666901	-0,485066866	-0,442416398	-0,372416398	**-1,632416398**
chrXIII	402458	402607	149	0,327699467	0,181587609	0,643447443	0,36768904	0,312535684	0,1727933	0,1377933	**-1,1222067**
chrXIII	406280	406447	167	0,188070999	0,292763697	0,391134418	-0,276363569	-0,234909033	0,052631556	-0,108368444	**-1,368368444**
chrXIII	408429	408536	107	0,227964847	0,148234783	0,605967759	0,268824834	0,228501109	0,96074776	1,21974776	**-0,04025224**
chrXIII	411105	411252	147	1,439028095	0,692997611	0,674958135	0,453645875	0,385598993	0,214500101	0,193500101	**-1,066499899**
chrXIII	412475	412581	106	0,190920559	1,463818484	0,115378047	-1,198413462	-1,018651443	-0,363510065	-0,097510065	**-1,357510065**
chrXIII	412475	412589	114	0,162424953	1,915934572	0,078150556	-1,41762214	-1,204978819	-0,584297997	-0,374297997	**-1,634297997**
chrXIII	413837	414003	166	0,051292091	0,044470435	0,535617563	0,08939893	0,07598909	0,381213987	0,227213987	**-1,032786013**
chrXIII	422824	422985	161	0,131079787	0,103764348	0,558156527	0,146296985	0,124352437	0,233456619	0,114456619	**-1,145543381**
chrXIII	431294	431455	161	0,034194727	0,044470435	0,434687048	-0,164453569	-0,139785534	-0,01838954	-0,13738954	**-1,39738954**
chrXIII	431358	431515	157	0,04844253	0,092646739	0,343346664	-0,403346515	-0,342844538	-0,256674928	-0,347674928	**-1,607674928**
chrXIII	435010	435159	149	1,478921943	0,804173699	0,647770473	0,379308141	0,32241192	0,182225987	0,147225987	**-1,112774013**

Chrom	Start	End	Length	Section A	Section B	A/A+B	Z-score	Z * 0.85	Phase correction	Length correction	ΔLKnuc
chrXIII	436980	437142	162	0,153876272	0,125999566	0,549801916	0,125160904	0,106386768	0,224068124	0,098068124	**-1,161931876**
chrXIII	439283	439457	174	0,054141651	0,166764131	0,245089334	-0,690024678	-0,586520976	-0,258658448	-0,468658448	**-1,728658448**
chrXIII	443760	443869	109	0,054141651	0,188999349	0,222675942	-0,763186995	-0,648708946	0,07306066	0,31806066	**-0,94193934**
chrXIII	445137	445296	159	0,113982423	0,222352175	0,338895921	-0,415478239	-0,353156503	-0,273215942	-0,378215942	**-1,638215942**
chrXIII	448884	449018	134	4,225898345	4,161691537	0,503827489	0,009594238	0,008155102	0,06471085	0,13471085	**-1,12528915**
chrXIII	448987	449140	153	1,216762369	0,52623348	0,698086785	0,518905805	0,441069934	0,349055763	0,286055763	**-0,973944237**
chrXIII	452811	452953	142	0,723788388	0,581821524	0,554368025	0,136705034	0,116199279	0,021969308	0,035969308	**-1,224030692**
chrXIII	455777	455925	148	0,356195073	0,303881305	0,539627056	0,099494207	0,084570076	-0,087167806	-0,115167806	**-1,375167806**
chrXIII	456607	456760	153	0,094035499	0,111176087	0,458236793	-0,104876779	-0,089145262	-0,17253899	-0,23553899	**-1,49553899**
chrXIII	458537	458683	146	0,510071344	0,229763914	0,689439086	0,494261052	0,420121894	0,241089972	0,227089972	**-1,032910028**
chrXIII	460130	460281	151	0,492973981	0,518821741	0,487226789	-0,032023164	-0,027219689	-0,173220402	-0,222220402	**-1,482220402**
chrXIII	460228	460362	134	0,453080133	0,244587392	0,649421274	0,383758496	0,326194722	0,388423293	0,458423293	**-0,801576707**
chrXIII	460442	460567	125	0,031345166	0,051882174	0,376621026	-0,314367347	-0,267212245	0,207676006	0,340676006	**-0,919323994**
chrXIII	460442	460595	153	0,085486818	0,044470435	0,657807209	0,406485945	0,345513053	0,260649291	0,197649291	**-1,062350709**
chrXIII	460442	460598	156	0,028495606	0,059293913	0,324590066	-0,454901459	-0,386666241	-0,354620617	-0,438620617	**-1,698620617**
chrXIII	460442	460600	158	8,198185799	5,558804368	0,595928739	0,242822993	0,206399544	0,296429082	0,198429082	**-1,061570918**
chrXIII	461194	461345	151	0,108283302	0,077823261	0,581834946	0,206589951	0,175601459	0,046242148	-0,002757852	**-1,262757852**
chrXIII	464378	464520	142	0,216566604	0,17788174	0,549036667	0,123227859	0,104743681	0,00187161	0,01587161	**-1,24412839**
chrXIII	467950	468100	150	0,119681545	0,144528914	0,452978074	-0,118140734	-0,100419624	-0,21087914	-0,25287914	**-1,51287914**
chrXIII	470902	471051	149	0,282106498	0,340940001	0,452785624	-0,118626528	-0,100832548	-0,244207987	-0,279207987	**-1,539207987**
chrXIII	473040	473146	106	0,740885752	3,031401315	0,196402272	-0,854542368	-0,726361013	-0,061787622	0,204212378	**-1,055787622**
chrXIII	473040	473162	122	0,954602795	3,6910461	0,205483199	-0,822194168	-0,698865043	-0,134734	0,019266	**-1,240734**
chrXIII	473911	474081	170	0,082637257	0,070411522	0,539940648	0,100284197	0,085241567	0,380076496	0,198076496	**-1,061923504**
chrXIII	484473	484603	130	0,059840772	0,915349786	0,061363158	-1,543430674	-1,311916073	-1,035076681	-0,937076681	**-2,197076681**
chrXIII	488956	489063	107	0,108283302	1,159937178	0,08538208	-1,369752547	-1,164289665	-0,421093102	-0,162093102	**-1,422093102**
chrXIII	488956	489073	117	0,131079787	1,397112831	0,085774388	-1,367244197	-1,162157567	-0,548386548	-0,359386548	**-1,619386548**
chrXIII	491261	491409	148	0,447381012	0,344645871	0,564855842	0,163292275	0,138798434	-0,039601077	-0,067601077	**-1,327601077**
chrXIII	492970	493101	131	1,627099093	1,986346094	0,450290238	-0,124928095	-0,106188881	0,058771503	0,149771503	**-1,110228497**
chrXIII	495547	495700	153	0,692443222	0,418763262	0,623145411	0,313752298	0,266689453	0,184144699	0,121144699	**-1,138855301**
chrXIII	496801	496973	172	0,715239706	0,982055438	0,421399725	-0,198313936	-0,168566845	0,144107123	-0,051892877	**-1,311892877**
chrXIII	496801	496973	172	0,715239706	0,982055438	0,421399725	-0,198313936	-0,168566845	0,144107123	-0,051892877	**-1,311892877**
chrXIII	499783	499912	129	1,042939174	1,467524353	0,415436895	-0,213581034	-0,181543879	0,132195684	0,237195684	**-1,022804316**
chrXIII	503855	504003	148	0,19377012	0,100058479	0,659466508	0,41100757	0,349356434	0,188809862	0,160809862	**-1,099190138**
chrXIII	505412	505581	169	1,134125112	1,971522616	0,365181505	-0,344642686	-0,292946283	0,007764389	-0,167235611	**-1,427235611**
chrXIII	512141	512286	145	0,039893848	0,037058696	0,518421434	0,046192108	0,039263292	-0,15228132	-0,15928132	**-1,41928132**
chrXIII	514521	514669	148	0,273557816	0,159352392	0,631904286	0,336901138	0,286365968	0,126358879	0,098358879	**-1,161641121**
chrXIII	514553	514703	150	0,062690333	0,085235	0,423797138	-0,192188854	-0,163360526	-0,28606141	-0,32806141	**-1,58806141**
chrXIII	515323	515459	136	0,034194727	0,048176305	0,415130494	-0,214366854	-0,182211826	-0,16289729	-0,10689729	**-1,36689729**
chrXIII	520223	520378	155	0,353345512	0,259410871	0,576649256	0,193328781	0,164329464	0,16239202	0,08539202	**-1,17460798**
chrXIII	520223	520378	155	0,353345512	0,259410871	0,576649256	0,193328781	0,164329464	0,16239202	0,08539202	**-1,17460798**
chrXIII	520376	520508	132	0,082637257	0,040764565	0,66965994	0,438974353	0,3731282	0,521285501	0,605285501	**-0,654714499**
chrXIII	521730	521901	171	0,170973635	0,415057393	0,291748435	-0,548284062	-0,466041453	-0,168508894	-0,357508894	**-1,617508894**
chrXIII	522712	522817	105	0,031345166	0,203822827	0,133288404	-1,11098035	-0,944333298	-0,351817696	-0,078817696	**-1,338817696**
chrXIII	528716	528857	141	0,088336378	0,059293913	0,598362147	0,249109985	0,211743487	0,12005505	0,14105505	**-1,11894495**
chrXIII	533451	533610	159	0,407487163	0,270528479	0,600999649	0,255935423	0,21754511	0,300296435	0,195296435	**-1,064703565**
chrXIII	535081	535246	165	0,190920559	0,392822175	0,327062844	-0,448038116	-0,380832398	-0,112159054	-0,259159054	**-1,519159054**
chrXIII	536826	536976	150	1,496019306	1,093231526	0,577780757	0,196219347	0,166786445	0,041577329	-0,000422671	**-1,260422671**
chrXIII	537368	537507	139	0,418885406	0,426175002	0,495686938	-0,010811454	-0,009189736	0,006568947	0,041568947	**-1,218431053**
chrXIII	539637	539760	123	0,059840772	0,207528696	0,223813035	-0,759378669	-0,645471868	-0,131669314	0,015330686	**-1,244669314**
chrXIII	540611	540759	148	2,481967268	2,053051747	0,547289275	0,118815599	0,100993259	-0,059502544	-0,087502544	**-1,347502544**
chrXIII	541727	541880	153	0,188070999	0,17788174	0,51392155	0,034903236	0,029667751	-0,052830119	-0,115830119	**-1,375830119**
chrXIII	549153	549302	149	0,116831984	0,133411305	0,466873595	-0,083131234	-0,070661549	-0,205657959	-0,240657959	**-1,500657959**
chrXIII	549525	549679	154	0,208017923	0,203822827	0,505093104	0,012766866	0,010851836	-0,077554174	-0,147554174	**-1,407554174**
chrXIII	549713	549857	144	0,83207169	0,674468263	0,552306421	0,131490682	0,11176708	-0,04361384	-0,04361384	**-1,30361384**
chrXIII	555791	555929	138	0,416035845	0,415057393	0,500588654	0,001475537	0,001254206	0,018899254	0,060899254	**-1,199100746**
chrXIII	562267	562429	162	2,855259705	2,657108488	0,517973329	0,045067707	0,038307551	0,168592054	0,042592054	**-1,217407946**
chrXIII	562901	563062	161	0,068389454	0,137117174	0,332784663	-0,432236787	-0,367401269	-0,248779898	-0,367779898	**-1,627779898**
chrXIII	571585	571738	153	8,343513389	8,408618074	0,498056824	-0,00487084	-0,004140214	-0,088203072	-0,151203072	**-1,411203072**
chrXIII	571590	571738	148	0,051292091	0,037058696	0,58055047	0,203301913	0,172806626	0,01298275	-0,01501725	**-1,27501725**
chrXIII	574736	574904	168	0,102584181	0,070411522	0,592986873	0,235235114	0,199949847	0,510278279	0,342278279	**-0,917721721**
chrXIII	580311	580467	156	0,814974327	0,559586306	0,592898056	0,235006242	0,199755306	0,234213633	0,150213633	**-1,109786367**
chrXIII	580644	580772	128	0,800726524	1,637994354	0,328338733	-0,444505066	-0,377829306	-0,00637328	0,10562672	**-1,15437328**
chrXIII	581583	581719	136	0,128230226	0,166764131	0,434687048	-0,164453569	-0,139785534	-0,116981759	-0,060981759	**-1,320981759**
chrXIII	582011	582174	163	3,587596775	3,231518273	0,52610885	0,065491969	0,055668174	0,232853812	0,099853812	**-1,160146188**
chrXIII	582013	582174	161	0,079787696	0,077823261	0,506231912	0,015621722	0,013278464	0,115144654	-0,003855346	**-1,263855346**
chrXIII	583110	583268	158	0,247911771	0,207528696	0,544334087	0,111358804	0,094654983	0,189323913	0,091323913	**-1,168676087**
chrXIII	588471	588586	115	0,051292091	0,329822392	0,134584469	-1,104978423	-0,939231659	-0,294804913	-0,091804913	**-1,351804913**
chrXIII	595003	595166	163	0,994496644	1,23405457	0,446252542	-0,135135069	-0,114864808	0,08155548	-0,05144452	**-1,31144452**
chrXIII	596303	596450	147	0,074088575	0,037058696	0,666580246	0,43048963	0,365916185	0,193878531	0,172878531	**-1,087121469**
chrXIII	598087	598214	127	0,230814407	1,511994788	0,132438139	-1,114939662	-0,947698713	-0,545991525	-0,426991525	**-1,686991525**
chrXIII	598887	599027	140	0,475876617	0,448410219	0,514858157	0,037252492	0,031664618	0,005968723	0,033968723	**-1,226031277**
chrXIII	600432	600562	130	0,435982769	0,544762828	0,444542163	-0,139462957	-0,118543513	0,143068905	0,241068905	**-1,018931095**
chrXIII	600662	600821	159	0,763682236	0,907938047	0,456851502	-0,108368984	-0,092113637	-0,011411267	-0,116411267	**-1,376411267**
chrXIII	602872	603031	159	0,746584873	0,856055873	0,465846681	-0,085714515	-0,072857338	-0,004766695	-0,109766695	**-1,369766695**
chrXIII	608199	608341	142	0,344796831	0,274234349	0,556994287	0,143352968	0,121850023	0,016869505	0,030869505	**-1,229130495**
chrXIII	614091	614242	151	0,755133555	0,563292176	0,572753957	0,183389919	0,155881431	0,031790775	-0,017209225	**-1,277209225**
chrXIII	617621	617773	152	0,190920559	0,107470218	0,639833982	0,358015071	0,30431281	0,206944527	0,150944527	**-1,109055473**
chrXIII	617962	618111	149	0,347646391	0,415057393	0,455807875	-0,111000752	-0,094350639	-0,223148784	-0,258148784	**-1,518148784**
chrXIII	622293	622443	150	2,020338454	1,352642396	0,598977149	0,250700464	0,213095395	0,080176494	0,038176494	**-1,221823506**
chrXIII	627780	627908	128	0,367593315	0,61888022	0,372633732	-0,324885852	-0,276152974	0,102015632	0,214015632	**-1,045984368**
chrXIII	627788	627919	131	0,946054114	0,993173047	0,487851105	-0,030457471	-0,025888851	0,13564691	0,22664691	**-1,03335309**
chrXIII	634568	634713	145	0,29065518	0,251999131	0,535617563	0,08939893	0,07598909	-0,109256504	-0,116256504	**-1,376256504**
chrXIII	637620	637765	145	0,051292091	0,051882174	0,497140355	-0,007168129	-0,00609291	-0,195292351	-0,202292351	**-1,462292351**
chrXIII	641481	641611	130	0,219416165	0,059293913	0,787255941	0,796936145	0,677395723	0,916768888	1,014768888	**-0,245231112**
chrXIII	642381	642525	144	3,650287107	1,952993268	0,651455373	0,389252658	0,330864759	0,170663877	0,170663877	**-1,089336123**
chrXIII	642389	642541	152	4,641934191	3,387164795	0,578138867	0,197134526	0,167564347	0,072769746	0,016769746	**-1,243230254**
chrXIII	649168	649320	152	0,034194727	0,062999783	0,351817475	-0,380418277	-0,323355536	-0,409134006	-0,465134006	**-1,725134006**
chrXIII	652798	652958	160	0,059840772	0,077823261	0,434687048	-0,164453569	-0,139785534	-0,111894571	-0,223894571	**-1,483894571**
chrXIII	657660	657789	129	1,045788734	1,763993919	0,372195598	-0,326043828	-0,277137254	-0,006476411	0,098523589	**-1,161476411**
chrXIII	658250	658404	154	0,698142343	0,711526959	0,495252569	-0,011900326	-0,010115277	-0,099230454	-0,169230454	**-1,429230454**
chrXIII	658827	658990	163	0,042743409	0,040764565	0,51184823	0,029703476	0,025247955	0,22744016	0,09444016	**-1,16555984**
chrXIII	658970	659105	135	3,601844578	3,350106099	0,5181056	0,0453996	0,03858966	0,088965713	0,151965713	**-1,108034287**
chrXIII	660778	660927	149	0,547115632	0,43729261	0,555781238	0,140281573	0,119239337	-0,010258508	-0,045258508	**-1,305258508**
chrXIII	662900	663052	152	0,062690333	0,148234783	0,297216064	-0,532424343	-0,452560692	-0,536514575	-0,592514575	**-1,852514575**
chrXIII	663803	663949	146	0,190920559	0,125999566	0,602424851	0,259628569	0,220684284	0,042098562	0,028098562	**-1,231901438**
chrXIII	663837	663944	107	0,202318801	1,545347614	0,115765114	-1,19642634	-1,016962389	-0,309336811	-0,050336811	**-1,310336811**

Chrom	Start	End	Length	Section A	Section B	A/A+B	Z-score	Z * 0.85	Phase correction	Length correction	ΔLKnuc
chrXIII	663837	663953	116	0,190920559	1,508288918	0,112358459	-1,214080675	-1,031968574	-0,399453801	-0,203453801	**-1,463453801**
chrXIII	667634	667789	155	0,313451664	0,218646305	0,589086376	0,225195424	0,191416111	0,201437372	0,124437372	**-1,135562628**
chrXIII	671983	672128	145	3,573348972	2,697873053	0,569801062	0,175867708	0,149487552	-0,044176996	-0,051176996	**-1,311176996**
chrXIII	675888	676037	149	1,017293128	0,637409568	0,614789068	0,291823124	0,248049656	0,121958337	0,086958337	**-1,173041663**
chrXIII	675888	676037	149	1,017293128	0,637409568	0,614789068	0,291823124	0,248049656	0,121958337	0,086958337	**-1,173041663**
chrXIII	675950	676037	87	0,045592969	0,066705652	0,405997586	-0,23785292	-0,202174982	0,154676396	0,553676396	**-0,706323604**
chrXIII	675950	676105	155	0,464478375	0,496586524	0,483295536	-0,041884125	-0,035601506	-0,01028913	-0,08728913	**-1,34728913**
chrXIII	677204	677344	140	0,076938136	0,111176087	0,408996909	-0,230126057	-0,195607148	-0,234891716	-0,206891716	**-1,466891716**
chrXIII	677920	678066	146	1,028691371	0,96723196	0,515396235	0,038602224	0,03281189	-0,148725477	-0,162725477	**-1,422725477**
chrXIII	678554	678723	169	1,401983807	2,538520661	0,355787899	-0,369740572	-0,314279486	-0,02859263	-0,20359263	**-1,46359263**
chrXIII	679422	679570	148	0,105433742	0,125999566	0,455568573	-0,11160432	-0,094863672	-0,247292214	-0,275292214	**-1,535292214**
chrXIII	680831	680955	124	0,273557816	0,481763045	0,362174316	-0,352652957	-0,299755013	0,175697427	0,315697427	**-0,944302573**
chrXIII	684362	684526	164	0,051292091	0,107470218	0,323074734	-0,459117949	-0,390250257	-0,163087733	-0,303087733	**-1,563087733**
chrXIII	686491	686656	165	0,233663968	0,285351958	0,450205777	-0,125141469	-0,106370249	0,15857449	0,01157449	**-1,24842551**
chrXIII	692898	693052	154	0,094035499	0,122293696	0,434687048	-0,164453569	-0,139785534	-0,230026231	-0,300026231	**-1,560026231**
chrXIII	693627	693771	144	0,359044634	0,222352175	0,617555219	0,299066166	0,254206241	0,10533851	0,10533851	**-1,15466149**
chrXIII	693831	693958	127	2,04883406	4,009750884	0,338170394	-0,417461624	-0,35484238	0,010323253	0,129323253	**-1,130676747**
chrXIII	694160	694314	154	0,09688506	0,062999783	0,605967759	0,268824834	0,228501109	0,153346103	0,083346103	**-1,176653897**
chrXIII	694285	694446	161	0,29065518	0,35205761	0,452231828	-0,120024607	-0,102020916	-0,009551281	-0,128551281	**-1,388551281**
chrXIII	694710	694842	132	3,011985537	3,046224794	0,497174144	-0,007083429	-0,006020915	0,126384619	0,210384619	**-1,049615381**
chrXIII	711631	711787	156	0,567062556	0,392822175	0,590761096	0,229503234	0,195077748	0,227542303	0,143542303	**-1,116457697**
chrXIII	713556	713705	149	0,213717044	0,140823044	0,602800787	0,260603323	0,221512824	0,097966863	0,062966863	**-1,197033137**
chrXIII	714522	714675	153	0,151026711	0,281646088	0,349055248	-0,387872358	-0,329691504	-0,420643898	-0,483643898	**-1,743643898**
chrXIII	715186	715332	146	0,116831984	0,059293913	0,663343584	0,421605719	0,358364861	0,171890165	0,157890165	**-1,102109835**
chrXIII	718847	718995	148	1,176868521	1,085819787	0,520119593	0,050453738	0,042885677	-0,106105952	-0,134105952	**-1,394105952**
chrXIII	718847	718995	148	1,176868521	1,085819787	0,520119593	0,050453738	0,042885677	-0,106105952	-0,134105952	**-1,394105952**
chrXIII	719890	720048	158	0,151026711	0,137117174	0,524136442	0,060538046	0,051457339	0,148115299	0,050115299	**-1,209884701**
chrXIII	723055	723205	150	2,234055497	1,57870044	0,585942435	0,217119612	0,18455167	0,055294245	0,013294245	**-1,246705755**
chrXIII	723972	724126	154	0,316301225	0,333528262	0,486744956	-0,033231584	-0,028246847	-0,095963828	-0,165963828	**-1,425963828**
chrXIII	730943	731091	148	0,116831984	0,081529131	0,588986325	0,224938202	0,191197471	0,044984487	0,016984487	**-1,243015513**
chrXIII	737903	738069	166	0,094035499	0,274234349	0,255344009	-0,657766759	-0,559101745	-0,26034349	-0,41434349	**-1,67434349**
chrXIII	748521	748674	153	0,213717044	0,181587609	0,540638826	0,102043248	0,086736761	-0,002026035	-0,065026035	**-1,325026035**
chrXIII	750522	750675	153	1,627099093	0,948702612	0,631686473	0,336323335	0,285874835	0,206215764	0,143215764	**-1,116784236**
chrXIII	757851	758017	166	1,159771157	1,334113048	0,465046114	-0,087728802	-0,074569482	0,233108496	0,079108496	**-1,180891504**
chrXIII	758839	758989	150	2,718480797	1,897405224	0,588940191	0,224819601	0,191096661	0,063650441	0,021650441	**-1,238349559**
chrXIII	758839	758989	150	2,718480797	1,897405224	0,588940191	0,224819601	0,191096661	0,063650441	0,021650441	**-1,238349559**
chrXIII	759376	759557	181	0,122531105	0,274234349	0,308825035	-0,499183568	-0,424306033	-0,074404033	-0,333404033	**-1,593404033**
chrXIII	765908	766066	158	0,09973462	0,077823261	0,561702018	0,155285859	0,13199298	0,227090165	0,129090165	**-1,130909835**
chrXIII	772941	773097	156	0,757983115	0,648527176	0,538910465	0,097689227	0,083035843	0,11475335	0,03075335	**-1,22924665**
chrXIII	773665	773803	138	0,034194727	0,037058696	0,479902939	-0,050397187	-0,042837609	-0,036895417	0,005104583	**-1,254895417**
chrXIII	773665	773807	142	4,456712753	3,590987622	0,553787113	0,13523538	0,114950073	0,010392358	0,024392358	**-1,235607642**
chrXIII	774153	774312	159	0,267858695	0,125999566	0,680089062	0,46794786	0,397755681	0,468215021	0,363215021	**-0,896784979**
chrXIII	774153	774312	159	0,267858695	0,125999566	0,680089062	0,46794786	0,397755681	0,468215021	0,363215021	**-0,896784979**
chrXIII	778663	778795	132	0,094035499	0,192705218	0,327946098	-0,445591717	-0,378752959	-0,259094604	-0,175094604	**-1,435094604**
chrXIII	779035	779188	153	0,182371877	0,340940001	0,348495581	-0,389385276	-0,330977484	-0,397841578	-0,460841578	**-1,720841578**
chrXIII	780072	780218	146	0,031345166	0,059293913	0,345823971	-0,396619676	-0,337126724	-0,526675782	-0,540675782	**-1,800675782**
chrXIII	780801	780937	136	0,037044288	0,051882174	0,416572153	-0,210670615	-0,179070023	-0,165242476	-0,109242476	**-1,369242476**
chrXIII	790225	790394	169	0,222265726	0,214940436	0,508377386	0,021000537	0,017850457	0,288918023	0,113918023	**-1,146081977**
chrXIII	790368	790515	147	0,82922213	0,663350655	0,555565624	0,139735783	0,118775416	-0,067560293	-0,088560293	**-1,348560293**
chrXIII	790640	790805	165	0,059840772	0,062999783	0,487141826	-0,032236246	-0,027400809	0,239757338	0,092757338	**-1,167242662**
chrXIII	790640	790805	165	0,059840772	0,062999783	0,487141826	-0,032236246	-0,027400809	0,239757338	0,092757338	**-1,167242662**
chrXIII	791775	791919	144	0,675345858	0,522527611	0,563787308	0,160578507	0,136491731	-0,02525158	-0,02525158	**-1,28525158**
chrXIII	792265	792408	143	0,165274514	0,151940653	0,521017061	0,052706352	0,044800399	-0,098420283	-0,091420283	**-1,351420283**
chrXIII	792607	792777	170	0,512920905	1,007996525	0,337244412	-0,419995386	-0,356996078	-0,059527541	-0,241527541	**-1,501527541**
chrXIII	792607	792777	170	0,512920905	1,007996525	0,337244412	-0,419995386	-0,356996078	-0,059527541	-0,241527541	**-1,501527541**
chrXIII	792935	793098	163	0,102584181	0,129705435	0,441621897	-0,146858392	-0,124829633	0,096910313	-0,036089687	**-1,296089687**
chrXIII	792935	793098	163	0,102584181	0,129705435	0,441621897	-0,146858392	-0,124829633	0,096910313	-0,036089687	**-1,296089687**
chrXIII	793020	793160	140	1,798072728	1,05617283	0,629964273	0,331758724	0,281994916	0,246436629	0,274436629	**-0,985563371**
chrXIII	793713	793841	128	0,091185939	0,144528914	0,386848507	-0,287542436	-0,24441107	0,1142702	0,2262702	**-1,0337298**
chrXIII	795911	796064	153	7,517140819	5,766333064	0,565901728	0,165949687	0,141057234	0,076028475	0,013028475	**-1,246971525**
chrXIII	797737	797897	160	3,901048439	3,735516535	0,510838113	0,027170462	0,023094893	0,056151023	-0,055848977	**-1,315848977**
chrXIII	797737	797940	203	0,208017923	0,233469783	0,471174893	-0,072316812	-0,06146929	0,272227782	-0,140772218	**-1,400772218**
chrXIII	797778	797885	107	0,279256937	0,35205761	0,442341996	-0,145034044	-0,123278937	0,595317323	0,854317323	**-0,405682677**
chrXIII	797778	797897	119	0,307752543	0,52993935	0,367381546	-0,338796427	-0,287976963	0,306848795	0,481848795	**-0,778151205**
chrXIII	797778	797940	162	3,02623334	2,434756313	0,554154747	0,136165427	0,115740613	0,241742425	0,115742425	**-1,144257575**
chrXIII	799674	799836	162	0,09688506	0,051882174	0,65125268	0,388704654	0,330398956	0,458562455	0,332562455	**-0,927437545**
chrXIII	800670	800819	149	0,208017923	0,270528479	0,434687048	-0,164453569	-0,139785534	-0,265584081	-0,300584081	**-1,560584081**
chrXIII	801962	802127	165	0,091185939	0,200116957	0,313027917	-0,487285766	-0,414192901	-0,121671835	-0,268671835	**-1,528671835**
chrXIII	804104	804264	160	0,236513528	0,285351958	0,453207838	-0,117560789	-0,099926671	-0,039894864	-0,151894864	**-1,411894864**
chrXIII	806854	806962	108	0,034194727	0,163058261	0,173354671	-0,940991251	-0,799842563	-0,105558627	0,146441373	**-1,113558627**
chrXIII	806855	806962	107	1,615700851	10,48761091	0,133492459	-1,11003274	-0,943527829	-0,224583207	0,034416793	**-1,225583207**
chrXIII	808568	808720	152	0,074088575	0,077823261	0,487707719	-0,030817056	-0,026194498	-0,121675613	-0,177675613	**-1,437675613**
chrXIII	811849	811978	129	3,046180264	4,098691754	0,426344972	-0,185687355	-0,157834252	0,110867304	0,215867304	**-1,044132696**
chrXIII	816433	816597	164	0,162424953	0,26682261	0,378394585	-0,309699913	-0,263244926	-0,03255335	-0,17255335	**-1,43255335**
chrXIII	819869	819975	106	0,250761331	1,111760874	0,184042014	-0,900068066	-0,765057856	-0,079689103	0,186310897	**-1,073689103**
chrXIII	819869	819988	119	0,279256937	1,337818918	0,17269254	-0,943578505	-0,80204173	-0,220795421	-0,045795421	**-1,305795421**
chrXIII	820608	820710	102	0,028495606	0,08894087	0,242646977	-0,69781331	-0,593141313	-0,104326901	0,189673099	**-1,070326901**
chrXIII	820608	820711	103	0,028495606	0,055588044	0,338895921	-0,415478239	-0,353156503	0,157723916	0,444723916	**-0,815276084**
chrXIII	820608	820715	107	0,088336378	1,248878048	0,066059995	-1,50579429	-1,279925146	-0,548099066	-0,289099066	**-1,549099066**
chrXIII	820608	820717	109	0,034194727	0,08894087	0,277699771	-0,589688448	-0,501235181	0,195615598	0,440615598	**-0,819384402**
chrXIII	820608	820733	125	0,028495606	0,114881957	0,198745224	-0,846111675	-0,719194924	-0,259911372	-0,126911372	**-1,386911372**
chrXIII	820608	820737	129	0,034194727	0,074117392	0,315705458	-0,47974192	-0,407780632	-0,115264164	-0,010264164	**-1,270264164**
chrXIII	820608	820738	130	0,042743409	0,129705435	0,247861382	-0,681235065	-0,579049806	-0,357093893	-0,259093893	**-1,519093893**
chrXIII	820608	820740	132	0,059840772	0,044470435	0,573675388	0,185739279	0,157878387	0,261009318	0,345009318	**-0,914990682**
chrXIII	820608	820741	133	0,039893848	0,092646739	0,300993446	-0,521545394	-0,443313585	-0,368555228	-0,291555228	**-1,551555228**
chrXIII	820608	820742	134	0,054141651	0,085235	0,388455674	-0,283346352	-0,2408444	-0,183831624	-0,113831624	**-1,373831624**
chrXIII	820608	820743	135	0,042743409	0,051882174	0,451710916	-0,121339881	-0,103138899	-0,078866383	-0,015866383	**-1,275866383**
chrXIII	820608	820744	136	0,039893848	0,125999566	0,240478794	-0,704763239	-0,599048753	-0,580869815	-0,524869815	**-1,784869815**
chrXIII	820608	820745	137	0,039893848	0,096352609	0,292806499	-0,545204323	-0,463423675	-0,457597069	-0,408597069	**-1,668597069**
chrXIII	820608	820746	138	0,056991212	0,070411522	0,447331152	-0,132407093	-0,112546029	-0,105740327	-0,063740327	**-1,323740327**
chrXIII	820608	820747	139	0,270708255	0,307587175	0,468114118	-0,080011341	-0,06800964	-0,050480863	-0,015480863	**-1,275480863**
chrXIII	820608	820748	140	0,051292091	0,155646522	0,247861382	-0,681235065	-0,579049806	-0,619729879	-0,591729879	**-1,851729879**
chrXIII	820608	820749	141	0,136778908	0,107470218	0,559997534	0,150962962	0,128318518	0,039303923	0,060303923	**-1,199696077**
chrXIII	820608	820750	142	0,091185939	0,292763697	0,237494531	-0,714385133	-0,607227363	-0,705217718	-0,691217718	**-1,951217718**
chrXIII	820608	820751	143	71,30455448	46,2418406	0,606607752	0,270488457	0,229915189	0,087777248	0,094777248	**-1,165222752**
chrXIII	820609	820751	142	0,108283302	0,096352609	0,529151025	0,07313593	0,062165541	-0,044726288	-0,030726288	**-1,290726288**

Chrom	Start	End	Length	Section A	Section B	A/A+B	Z-score	Z * 0.85	Phase correction	Length correction	ΔLKnuc
chrXIII	820610	820751	141	0,14817715	0,118587827	0,555459536	0,139467258	0,118547169	0,032026126	0,053026126	**-1,206973874**
chrXIII	820611	820751	140	0,151026711	0,185293479	0,449056332	-0,12804588	-0,108838998	-0,166790791	-0,138790791	**-1,398790791**
chrXIII	820612	820751	139	0,076938136	0,074117392	0,509336779	0,023405971	0,019895076	0,029395372	0,064395372	**-1,195604628**
chrXIII	820613	820751	138	0,128230226	0,077823261	0,622315244	0,311567137	0,264832067	0,277430372	0,319430372	**-0,940569628**
chrXIII	820614	820751	137	0,028495606	0,055588044	0,338895921	-0,415478239	-0,353156503	-0,335491319	-0,286491319	**-1,546491319**
chrXIII	820615	820751	136	0,04844253	0,092646739	0,343346664	-0,403346515	-0,342844538	-0,336404258	-0,280404258	**-1,540404258**
chrXIII	820616	820751	135	0,037044288	0,066705652	0,357053581	-0,366345661	-0,311393812	-0,298701512	-0,235701512	**-1,495701512**
chrXIII	820617	820751	134	0,045592969	0,055588044	0,450607955	-0,124125498	-0,105506674	-0,050760607	0,019239393	**-1,240760607**
chrXIII	820619	820751	132	0,068389454	0,103764348	0,397257877	-0,260451199	-0,221383519	-0,13005236	-0,04605236	**-1,30605236**
chrXIII	820620	820751	131	0,045592969	0,048176305	0,486225044	-0,034535557	-0,029355224	0,12791438	0,21891438	**-1,04108562**
chrXIII	820621	820751	130	0,034194727	0,037058696	0,479902939	-0,050397187	-0,042837609	0,164655521	0,262655521	**-0,997344479**
chrXIII	820624	820751	127	0,028495606	0,081529131	0,25899272	-0,646453904	-0,549485818	-0,179185462	-0,060185462	**-1,320185462**
chrXIII	820626	820715	89	0,071239015	0,051882174	0,578608892	0,198335964	0,168585569	0,543327095	0,928327095	**-0,331672905**
chrXIII	828572	828720	148	0,188070999	0,151940653	0,553131041	0,13357593	0,113539541	-0,0287041	-0,0567041	**-1,3167041**
chrXIII	835990	836099	109	1,131275552	0,97093783	0,538135454	0,095737455	0,081376837	0,77067512	1,01567512	**-0,24432488**
chrXIII	836333	836463	130	1,102779946	1,371171744	0,445756459	-0,136390076	-0,115931565	0,079995769	0,177995769	**-1,082004231**
chrXIII	842098	842232	134	4,137561967	3,968986319	0,510397499	0,026065615	0,022155773	0,08784021	0,15784021	**-1,10215979**
chrXIII	843431	843583	152	0,270708255	0,181587609	0,598520297	0,249518923	0,212091084	0,117014463	0,061014463	**-1,198985537**
chrXIII	847888	848017	129	0,615505086	1,033937612	0,373159423	-0,323497043	-0,274972487	0,021946515	0,126946515	**-1,133053485**
chrXIII	847897	848050	153	0,971700159	0,426175002	0,695126565	0,510434818	0,433869595	0,346706139	0,283706139	**-0,976293861**
chrXIII	851484	851633	149	0,139628469	0,226058044	0,38182559	-0,300689626	-0,255586182	-0,391947621	-0,426947621	**-1,686947621**
chrXIII	864286	864433	147	0,062690333	0,081529131	0,434687048	-0,164453569	-0,139785534	-0,326539689	-0,347539689	**-1,607539689**
chrXIII	864644	864799	155	0,219416165	0,144528914	0,602882627	0,260815558	0,221693224	0,244585865	0,167585865	**-1,092414135**
chrXIII	864644	864799	155	0,219416165	0,144528914	0,602882627	0,260815558	0,221693224	0,244585865	0,167585865	**-1,092414135**
chrXIII	866005	866155	150	0,151026711	0,048176305	0,758154743	0,700379213	0,595322331	0,469516896	0,427516896	**-0,832483104**
chrXIII	868732	868890	158	0,87766466	0,796761959	0,52415833	0,06059301	0,051504058	0,132647904	0,034647904	**-1,225352096**
chrXIII	871472	871623	151	0,225115286	0,155646522	0,591223387	0,230693111	0,196089145	0,071473628	0,022473628	**-1,237526372**
chrXIII	871472	871623	151	0,225115286	0,155646522	0,591223387	0,230693111	0,196089145	0,071473628	0,022473628	**-1,237526372**
chrXIII	873640	873761	121	0,170973635	0,144528914	0,541908887	0,105243965	0,08945737	0,67489541	0,83589541	**-0,42410459**
chrXIII	873640	873789	149	0,336248149	0,214940436	0,610041932	0,279428325	0,237514076	0,089806041	0,054806041	**-1,205193959**
chrXIII	873640	873790	150	3,063277627	1,552759353	0,663616353	0,422353116	0,359000149	0,224797847	0,182797847	**-1,077202153**
chrXIII	873648	873761	113	0,074088575	0,081529131	0,47609348	-0,059960669	-0,050966568	0,589519266	0,806519266	**-0,453480734**
chrXIII	873648	873789	141	1,90350647	0,96352609	0,663929143	0,423210462	0,359728892	0,273042285	0,294042285	**-0,965957715**
chrXIII	873648	873790	142	0,210867483	0,144528914	0,593330391	0,236120435	0,20070237	0,088354045	0,102354045	**-1,157645955**
chrXIII	878188	878341	153	0,49012442	0,363175219	0,574387235	0,187554968	0,159421723	0,07476291	0,01176291	**-1,24823709**
chrXIII	879147	879302	155	0,262159574	0,277940218	0,485390992	-0,036627541	-0,03113341	-0,003664549	-0,080664549	**-1,340664549**
chrXIII	884345	884496	151	0,068389454	0,074117392	0,479902939	-0,050397187	-0,042837609	-0,15321069	-0,20221069	**-1,46221069**
chrXIII	886126	886284	158	0,105433742	0,074117392	0,58720733	0,220367006	0,187311955	0,273550492	0,175550492	**-1,084449508**
chrXIII	889335	889469	134	0,199469241	0,17788174	0,528604008	0,071761159	0,060996985	0,134776454	0,204776454	**-1,055223546**
chrXIII	890068	890170	102	2,310993633	23,839859	0,088371636	-1,350850672	-1,148223071	-0,638195994	-0,344195994	**-1,604195994**
chrXIII	891024	891149	125	1,994692408	3,33898849	0,373980455	-0,321329227	-0,273129843	0,163974082	0,296974082	**-0,963025918**
chrXIII	891955	892105	150	0,125380666	0,077823261	0,617018911	0,297660652	0,253011555	0,113490538	0,071490538	**-1,188509462**
chrXIII	895348	895499	151	0,447381012	0,259410871	0,632974179	0,339740923	0,288779784	0,184238983	0,135238983	**-1,124761017**
chrXIII	902827	902965	138	0,133929347	0,085235	0,611090941	0,282163534	0,239839004	0,251544474	0,293544474	**-0,966455526**
chrXIII	904201	904335	134	0,076938136	0,111176087	0,408996909	-0,230126057	-0,195607148	-0,136281986	-0,066281986	**-1,326281986**
chrXIII	904428	904582	154	1,684090305	1,252583918	0,573468549	0,185211815	0,157430043	0,093357005	0,023357005	**-1,236642995**
chrXIII	910695	910845	150	0,227964847	0,129705435	0,637360324	0,351411905	0,298700119	0,15789746	0,11589746	**-1,14410254**
chrXIII	911000	911154	154	0,410336724	0,218646305	0,652381233	0,391757318	0,332993721	0,270710975	0,200710975	**-1,059289025**
chrXIII	917204	917355	151	0,091185939	0,103764348	0,467739443	-0,080953558	-0,068810524	-0,168306761	-0,217306761	**-1,477306761**
chrXIII	921093	921253	160	0,450230572	0,326116523	0,579934639	0,201726272	0,171467332	0,242794216	0,130794216	**-1,129205784**
chrXIII	923830	923976	146	1,90350647	0,578115654	0,767041223	0,729137507	0,619766881	0,436375191	0,422375191	**-0,837624809**
chrXIII	924304	924426	122	0,641151131	0,133411305	0,827759134	0,945347037	0,803544981	1,348412087	1,502412087	**0,242412087**
chrXIII	924311	924426	115	0,871965539	0,34835174	0,714540025	0,56669716	0,481692586	1,139325735	1,342325735	**0,082325735**
chrXIII	924321	924426	105	0,310602104	0,092646739	0,770249212	0,739667825	0,628717651	1,243129044	1,516129044	**0,256129044**
chrXIV	614	765	151	0,615505086	0,418763262	0,595111595	0,24071397	0,204606874	0,04785356	-0,00114644	**-1,26114644**
chrXIV	953	1105	152	0,062690333	0,040764565	0,605967759	0,268824834	0,228501109	0,148994944	0,092994944	**-1,167005056**
chrXIV	1779	1930	151	0,455929693	0,300175436	0,602997752	0,26111413	0,22194701	0,07466169	0,02566169	**-1,23433831**
chrXIV	1791	1925	134	0,681044979	0,418763262	0,619239749	0,303484704	0,257961999	0,28675712	0,35675712	**-0,90324288**
chrXIV	1816	1912	96	0,051292091	0,111176087	0,315705458	-0,47974192	-0,407780632	0,014235878	0,350235878	**-0,909764122**
chrXIV	2170	2300	130	0,333398588	0,433586741	0,434687048	-0,164453569	-0,139785534	0,003166341	0,101166341	**-1,158833659**
chrXIV	2449	2601	152	0,239363089	0,100058479	0,705208837	0,539441393	0,458525184	0,382258698	0,326258698	**-0,933741302**
chrXIV	2796	2940	144	1,379187322	0,548468698	0,715473771	0,569447547	0,484030415	0,315162396	0,315162396	**-0,944837604**
chrXIV	2796	2983	187	0,111132863	0,040764565	0,731630971	0,617753167	0,525090192	0,907509045	0,606509045	**-0,653490955**
chrXIV	2848	2940	92	0,142478029	0,155646522	0,477914444	-0,055388588	-0,0470803	0,256539471	0,620539471	**-0,639460529**
chrXIV	2848	2983	135	1,26805446	0,830114786	0,604362333	0,264654886	0,224956653	0,22780873	0,29080873	**-0,96919127**
chrXIV	2936	3080	144	0,125380666	0,040764565	0,754644987	0,68917996	0,585802966	0,419727808	0,419727808	**-0,840272192**
chrXIV	3001	3153	152	0,09973462	0,081529131	0,550218231	0,126212725	0,107280816	0,027135882	-0,028864118	**-1,288864118**
chrXIV	3253	3378	125	0,225115286	0,255705001	0,468190075	-0,079820337	-0,067847286	0,288699987	0,421699987	**-0,838300013**
chrXIV	3283	3426	143	0,376141997	0,466939567	0,446151373	-0,135390993	-0,115082344	-0,35052687	-0,34352687	**-1,60352687**
chrXIV	3584	3721	137	0,182371877	0,125999566	0,591403262	0,231156174	0,196482748	0,089827528	0,138827528	**-1,121172472**
chrXIV	3700	3855	155	0,09688506	0,17417587	0,357429084	-0,365339271	-0,31053838	-0,352110747	-0,429110747	**-1,689110747**
chrXIV	3794	3956	162	0,068389454	0,051882174	0,568624996	0,172874585	0,146943398	0,179118727	0,053118727	**-1,206881273**
chrXIV	3794	3956	162	0,068389454	0,051882174	0,568624996	0,172874585	0,146943398	0,179118727	0,053118727	**-1,206881273**
chrXIV	5019	5160	141	0,156725832	0,081529131	0,657807209	0,406485945	0,345513053	0,13555847	0,15655847	**-1,10344153**
chrXIV	15620	15770	150	0,04844253	0,066705652	0,420697304	-0,200109928	-0,170093439	-0,314250702	-0,356250702	**-1,616250702**
chrXIV	18901	19054	153	0,128230226	0,081529131	0,611320649	0,282762761	0,240348347	0,185485875	0,122485875	**-1,137514125**
chrXIV	22418	22569	151	0,814974327	0,433586741	0,65273085	0,392703751	0,333798188	0,180539646	0,131539646	**-1,128460354**
chrXIV	24850	25019	169	0,270708255	0,292763697	0,48042898	-0,049076965	-0,04171542	0,000251513	-0,174748487	**-1,434748487**
chrXIV	25836	25986	150	0,638301571	0,185293479	0,775018707	0,755477404	0,642155793	0,50357883	0,46157883	**-0,79842117**
chrXIV	26530	26680	150	1,772426683	1,623170875	0,521977841	0,055118174	0,046850448	-0,082838721	-0,124838721	**-1,384838721**
chrXIV	27268	27422	154	0,119681545	0,144528914	0,452978074	-0,118140734	-0,100419624	-0,147611654	-0,217611654	**-1,477611654**
chrXIV	30249	30400	151	0,136778908	0,085235	0,61608261	0,295208275	0,250927034	0,103186702	0,054186702	**-1,205813298**
chrXIV	32059	32210	151	0,339097709	0,296469566	0,533535508	0,084160298	0,071536253	-0,078798737	-0,127798737	**-1,387798737**
chrXIV	33454	33599	145	7,950274028	5,429098933	0,594218731	0,238410741	0,20264913	0,040115665	0,033115665	**-1,226884335**
chrXIV	33454	33606	152	2,29389627	2,041934138	0,52905581	0,072896625	0,061962132	0,002171909	-0,053828091	**-1,313828091**
chrXIV	33459	33599	140	0,045592969	0,037058696	0,551627959	0,129775456	0,110309137	-0,038636694	-0,010636694	**-1,270636694**
chrXIV	33461	33599	138	2,131471316	2,093816312	0,50445591	0,011169543	0,009494111	-0,106245062	-0,064245062	**-1,324245062**
chrXIV	33461	33603	142	0,056991212	0,040764565	0,582995843	0,209563572	0,178129036	-0,065005072	-0,051005072	**-1,311005072**
chrXIV	33461	33606	145	26,10767407	14,02671635	0,650506306	0,386687748	0,328684586	0,176506802	0,169506802	**-1,090493198**
chrXIV	33464	33606	142	0,042743409	0,048176305	0,470122564	-0,074961771	-0,063717505	-0,314819366	-0,300819366	**-1,560819366**
chrXIV	33466	33606	140	0,142478029	0,114881957	0,553613758	0,134796867	0,114577337	-0,033952191	-0,005952191	**-1,265952191**
chrXIV	41403	41548	145	1,336443914	1,026525873	0,565578079	0,165127229	0,140358145	-0,007830872	-0,014830872	**-1,274830872**
chrXIV	45261	45368	107	0,521469587	3,542811317	0,128305498	-1,13443769	-0,964272037	-0,442529939	-0,183529939	**-1,443529939**
chrXIV	45261	45374	113	0,424584527	4,187632624	0,092056491	-1,328197161	-1,128967587	-0,706246433	-0,489246433	**-1,749246433**
chrXIV	45783	45937	154	0,911859387	0,585527393	0,608967168	0,276628128	0,235133909	0,167035336	0,097035336	**-1,162964664**
chrXIV	49049	49199	150	0,567062556	0,392822175	0,590761096	0,229503234	0,195077748	0,077008882	0,035008882	**-1,224991118**

Chrom	Start	End	Length	Section A	Section B	A/A+B	Z-score	Z * 0.85	Phase correction	Length correction	ΔLKnuc
chrXIV	53101	53263	162	0,205168362	0,359469349	0,36336284	-0,349484402	-0,297061742	-0,251356206	-0,377356206	**-1,637356206**
chrXIV	55301	55450	149	0,247911771	0,140823044	0,637740077	0,352424614	0,299560922	0,206835369	0,171835369	**-1,088164631**
chrXIV	58407	58536	129	0,455929693	0,581821524	0,439343925	-0,152632808	-0,129737887	0,049954033	0,154954033	**-1,105045967**
chrXIV	60113	60210	97	0,216566604	1,719523484	0,11185771	-1,216707773	-1,034201607	-0,597967024	-0,268967024	**-1,528967024**
chrXIV	61184	61331	147	0,179522317	0,114881957	0,609781626	0,278749922	0,236937434	0,161243621	0,140243621	**-1,119756379**
chrXIV	63570	63733	163	0,433133209	0,452116089	0,489278229	-0,02687873	-0,02284692	0,056291692	-0,076708308	**-1,336708308**
chrXIV	65407	65535	128	2,151418241	3,498340882	0,380798224	-0,303385036	-0,257877281	-0,065811438	0,046188562	**-1,213811438**
chrXIV	73936	74081	145	0,162424953	0,240881523	0,402733313	-0,246278533	-0,209336753	-0,36042696	-0,36742696	**-1,62742696**
chrXIV	77741	77895	154	0,173823196	0,237175653	0,422928668	-0,194406838	-0,165245812	-0,225767322	-0,295767322	**-1,555767322**
chrXIV	79985	80135	150	0,14532759	0,125999566	0,535617563	0,08939893	0,07598909	-0,057485803	-0,099485803	**-1,359485803**
chrXIV	81420	81583	163	0,304902982	0,466939567	0,395032617	-0,266225903	-0,226292018	-0,144239866	-0,277239866	**-1,537239866**
chrXIV	91093	91242	149	1,382036883	0,726350437	0,65549478	0,400198336	0,340168586	0,247708432	0,212708432	**-1,047291568**
chrXIV	93267	93403	136	0,119681545	0,107470218	0,526879225	0,067427283	0,057313191	0,034733336	0,090733336	**-1,169266664**
chrXIV	97284	97435	151	0,04844253	0,051882174	0,482857442	-0,042983253	-0,036535765	-0,182140008	-0,231140008	**-1,491140008**
chrXIV	97941	98103	162	0,34194727	0,214940436	0,614032715	0,289845327	0,246368528	0,284495878	0,158495878	**-1,101504122**
chrXIV	99408	99557	149	0,108283302	0,059293913	0,646169599	0,37499955	0,318749618	0,239439385	0,204439385	**-1,055560615**
chrXIV	99632	99789	157	0,062690333	0,044470435	0,585011979	0,214732295	0,182522451	0,096231744	0,005231744	**-1,254768256**
chrXIV	99818	99968	150	8,930522869	4,376631972	0,671106858	0,442971589	0,376525851	0,237488158	0,195488158	**-1,064511842**
chrXIV	99822	99968	146	0,071239015	0,051882174	0,578608892	0,198335964	0,168585569	0,029420429	0,015420429	**-1,244579571**
chrXIV	100280	100415	135	0,04844253	0,048176305	0,50137771	0,003453413	0,002935401	-0,004088959	0,058911041	**-1,201088959**
chrXIV	100932	101089	157	0,153876272	0,08894087	0,633712557	0,341702373	0,290447017	0,203750247	0,112750247	**-1,147249753**
chrXIV	102362	102510	148	0,034194727	0,037058696	0,479902939	-0,050397187	-0,042837609	-0,128490541	-0,156490541	**-1,416490541**
chrXIV	103973	104137	164	0,29065518	0,307587175	0,485848548	-0,035479871	-0,030157891	0,044352063	-0,095647937	**-1,355647937**
chrXIV	107707	107834	127	2,216958134	2,749755227	0,446363213	-0,134855118	-0,11462685	0,121218978	0,240218978	**-1,019781022**
chrXIV	110079	110230	151	1,020142689	0,982055438	0,509511359	0,023843701	0,020267146	-0,120310439	-0,169310439	**-1,429310439**
chrXIV	111620	111775	155	0,165274514	0,188999349	0,46651625	-0,084030099	-0,071425584	-0,127656367	-0,204656367	**-1,464656367**
chrXIV	112009	112129	120	0,265009134	0,874585221	0,2325469	-0,730484965	-0,620912221	-0,235286399	-0,067286399	**-1,327286399**
chrXIV	116452	116578	126	0,054141651	0,192705218	0,219332946	-0,774448026	-0,658280822	-0,295161036	-0,169161036	**-1,429161036**
chrXIV	119252	119378	126	0,29350474	0,455821958	0,391691289	-0,274913653	-0,233676605	0,116580392	0,242580392	**-1,017419608**
chrXIV	121333	121464	131	0,065539893	0,044470435	0,595761274	0,242390685	0,206032083	0,331380734	0,422380734	**-0,837619266**
chrXIV	124849	124975	126	0,105433742	0,151940653	0,409651247	-0,228442189	-0,194175861	0,140007787	0,266007787	**-0,993992213**
chrXIV	129691	129843	152	0,042743409	0,066705652	0,390532439	-0,277931585	-0,236241848	-0,293822261	-0,349822261	**-1,609822261**
chrXIV	129719	129870	151	0,350495952	0,192705218	0,645241526	0,372504922	0,316629184	0,179026794	0,130026794	**-1,129973206**
chrXIV	131962	132135	173	0,113982423	0,237175653	0,324590066	-0,454901459	-0,386666241	-0,168223871	-0,371223871	**-1,631223871**
chrXIV	132312	132463	151	0,709540585	0,837526525	0,458635945	-0,103870788	-0,08829017	-0,224553729	-0,273553729	**-1,533553729**
chrXIV	133490	133654	164	0,151026711	0,196411088	0,434687048	-0,164453569	-0,139785534	-0,065443021	-0,205443021	**-1,465443021**
chrXIV	134176	134325	149	0,276407377	0,203822827	0,575572662	0,190579981	0,161992984	0,083704125	0,048704125	**-1,211295875**
chrXIV	135289	135440	151	0,222265726	0,148234783	0,599906668	0,253105531	0,215139702	0,068235154	0,019235154	**-1,240764846**
chrXIV	135829	135979	150	1,154072036	0,715232829	0,61738032	0,29860774	0,253816579	0,108476634	0,066476634	**-1,193523366**
chrXIV	139766	139921	155	0,190920559	0,044470435	0,811078435	0,881877363	0,749595759	0,689633657	0,612633657	**-0,647366343**
chrXIV	140110	140269	159	0,210867483	0,159352392	0,569573644	0,175288799	0,148995479	0,085297053	-0,019702947	**-1,279702947**
chrXIV	141911	142060	149	0,612655525	0,292763697	0,676653986	0,4583625	0,389608125	0,30684807	0,27184807	**-0,98815193**
chrXIV	150518	150667	149	1,248107536	0,711526959	0,636908331	0,350207021	0,297675968	0,21061933	0,17561933	**-1,08438067**
chrXIV	153107	153271	164	0,247911771	0,377998697	0,396081842	-0,263501999	-0,223976699	-0,147672375	-0,287672375	**-1,547672375**
chrXIV	153107	153271	164	0,247911771	0,377998697	0,396081842	-0,263501999	-0,223976699	-0,147672375	-0,287672375	**-1,547672375**
chrXIV	155351	155502	151	0,059840772	0,037058696	0,617555219	0,299066166	0,254206241	0,103664999	0,054664999	**-1,205335001**
chrXIV	163862	164000	138	0,299203861	0,233469783	0,561702018	0,155285859	0,13199298	-0,001547147	0,040452853	**-1,219547147**
chrXIV	163862	164000	138	0,299203861	0,233469783	0,561702018	0,155285859	0,13199298	-0,001547147	0,040452853	**-1,219547147**
chrXIV	165154	165280	126	2,131471316	6,985564156	0,233789955	-0,726422322	-0,617458974	-0,312327677	-0,186327677	**-1,446327677**
chrXIV	167446	167585	139	1,715435471	1,619465006	0,514388805	0,036075209	0,030663927	-0,114871234	-0,079871234	**-1,339871234**
chrXIV	174863	175007	144	0,322000346	0,285351958	0,530170618	0,075698758	0,064343944	-0,094411165	-0,094411165	**-1,354411165**
chrXIV	177654	177807	153	0,49012442	0,541056958	0,475303793	-0,061943802	-0,052652232	-0,105142246	-0,168142246	**-1,428142246**
chrXIV	178329	178461	132	0,669646737	1,182172396	0,361615627	-0,354143619	-0,301022076	-0,234790425	-0,150790425	**-1,410790425**
chrXIV	183769	183934	165	0,316301225	0,370586958	0,460484301	-0,099213694	-0,08433164	-0,061010197	-0,208010197	**-1,468010197**
chrXIV	187776	187960	184	0,071239015	0,17788174	0,285961781	-0,565220842	-0,480437716	-0,15099612	-0,43099612	**-1,69099612**
chrXIV	188930	189078	148	0,051292091	0,037058696	0,58055047	0,203301913	0,172806626	0,078881341	0,050881341	**-1,209118659**
chrXIV	188930	189079	149	0,039893848	0,059293913	0,402205349	-0,247642923	-0,210496484	-0,321432157	-0,356432157	**-1,616432157**
chrXIV	188930	189080	150	0,091185939	0,037058696	0,711031218	0,556399818	0,472939845	0,321819881	0,279819881	**-0,980180119**
chrXIV	188930	189081	151	0,051292091	0,048176305	0,515662191	0,039269381	0,033378974	-0,105159259	-0,154159259	**-1,414159259**
chrXIV	188930	189083	153	28,49275627	21,14569182	0,574005783	0,186581933	0,158594643	0,109623367	0,046623367	**-1,213376633**
chrXIV	188931	189083	152	0,031345166	0,062999783	0,332240005	-0,433736208	-0,368675777	-0,422335508	-0,478335508	**-1,738335508**
chrXIV	188933	189083	150	0,108283302	0,107470218	0,50188429	0,004723232	0,004014747	-0,148303976	-0,190303976	**-1,450303976**
chrXIV	188934	189083	149	0,074088575	0,074117392	0,499902783	-0,000243688	-0,000207135	-0,105315724	-0,140315724	**-1,400315724**
chrXIV	188935	189083	148	0,085486818	0,122293696	0,411428464	-0,223871969	-0,190291174	-0,283354733	-0,311354733	**-1,571354733**
chrXIV	190891	191040	149	0,09973462	0,092646739	0,518421434	0,046192108	0,039263292	-0,056077167	-0,091077167	**-1,351077167**
chrXIV	192308	192455	147	0,287805619	0,277940218	0,508718933	0,021856865	0,018578335	-0,029095422	-0,050095422	**-1,310095422**
chrXIV	192813	192954	141	0,344796831	0,17417587	0,664383368	0,424456031	0,360787627	0,139751378	0,160751378	**-1,099248622**
chrXIV	193504	193657	153	0,04844253	0,144528914	0,251034708	-0,671237229	-0,570551645	-0,626134012	-0,689134012	**-1,949134012**
chrXIV	196760	196910	150	4,160358452	2,720108271	0,604662245	0,265433528	0,225618498	0,065971311	0,023971311	**-1,236028689**
chrXIV	202136	202292	156	0,108283302	0,044470435	0,708874979	0,550101085	0,467585923	0,361169228	0,277169228	**-0,982830772**
chrXIV	203542	203662	120	0,116831984	0,040764565	0,741335927	0,647469471	0,550349051	0,913212574	1,081212574	**-0,178787426**
chrXIV	206857	207023	166	0,042743409	0,077823261	0,354520937	-0,373143192	-0,317171714	-0,287452663	-0,441452663	**-1,701452663**
chrXIV	210139	210288	149	2,379383087	1,84922892	0,562686547	0,157784127	0,134116508	0,035805382	0,000805382	**-1,259194618**
chrXIV	216142	216308	166	0,219416165	0,203822827	0,518421434	0,046192108	0,039263292	0,073692751	-0,080307249	**-1,340307249**
chrXIV	219506	219633	127	0,076938136	0,077823261	0,497140355	-0,007168129	-0,00609291	0,213472079	0,332472079	**-0,927527921**
chrXIV	224826	224979	153	0,04844253	0,055588044	0,465656665	-0,08619258	-0,073263693	-0,113847002	-0,176847002	**-1,436847002**
chrXIV	227320	227476	156	0,039893848	0,037058696	0,518421434	0,046192108	0,039263292	-0,077656323	-0,161656323	**-1,421656323**
chrXIV	235082	235232	150	0,404637603	0,285351958	0,586440181	0,218397212	0,18563763	0,022894934	-0,019105066	**-1,279105066**
chrXIV	238200	238369	169	0,065539893	0,092646739	0,414320049	-0,216446021	-0,183979117	-0,144285701	-0,319285701	**-1,579285701**
chrXIV	246737	246897	160	0,102584181	0,188999349	0,351817475	-0,380418277	-0,323355536	-0,370123121	-0,482123121	**-1,742123121**
chrXIV	247839	247988	149	0,188070999	0,148234783	0,559226183	0,149007602	0,126656462	0,023252355	-0,011747645	**-1,271747645**
chrXIV	254524	254675	151	1,818019652	1,289642613	0,585011979	0,214732295	0,182522451	0,059930533	0,010930533	**-1,249069467**
chrXIV	257454	257590	136	0,250761331	0,196411088	0,560771015	0,152924304	0,129985658	0,097921838	0,153921838	**-1,106078162**
chrXIV	257743	257893	150	0,208017923	0,148234783	0,583905523	0,211894998	0,180110748	0,014233189	-0,027766811	**-1,287766811**
chrXIV	259132	259283	151	0,102584181	0,040764565	0,715626635	0,569898223	0,48441349	0,375331357	0,326331357	**-0,933668643**
chrXIV	259132	259283	151	0,102584181	0,040764565	0,715626635	0,569898223	0,48441349	0,375331357	0,326331357	**-0,933668643**
chrXIV	265572	265700	128	0,131079787	0,125999566	0,509880648	0,024769644	0,021054198	0,209083889	0,321083889	**-0,938916111**
chrXIV	271513	271667	154	0,983098401	0,844938264	0,537789214	0,094865611	0,080635769	0,02724365	-0,04275635	**-1,30275635**
chrXIV	273060	273209	149	5,248890595	3,909692405	0,573111648	0,184301797	0,156656527	0,042149727	0,007149727	**-1,252850273**
chrXIV	273060	273209	149	5,248890595	3,909692405	0,573111648	0,184301797	0,156656527	0,042149727	0,007149727	**-1,252850273**
chrXIV	275916	276056	140	1,971895924	0,937585003	0,677748359	0,461411668	0,392199918	0,239678132	0,267678132	**-0,992321868**
chrXIV	281515	281622	107	0,510071344	1,723229354	0,228393492	-0,744147933	-0,632525743	-0,090421709	0,168578291	**-1,091421709**
chrXIV	281515	281631	116	0,410336724	2,605226314	0,136073005	-1,098133933	-0,933413843	-0,483908334	-0,287908334	**-1,547908334**
chrXIV	281832	281976	144	0,133929347	0,044470435	0,750725957	0,676776004	0,575259604	0,426794196	0,426794196	**-0,833205804**
chrXIV	283804	283989	185	0,037044288	0,066705652	0,357053581	-0,366345661	-0,311393812	0,053634083	-0,233365917	**-1,493365917**
chrXIV	287747	287882	135	0,236513528	0,118587827	0,666045131	0,429018532	0,364665752	0,364786064	0,427786064	**-0,832213936**

Chrom	Start	End	Length	Section A	Section B	A/A+B	Z-score	Z * 0.85	Phase correction	Length correction	ΔLKnuc
chrXIV	291077	291208	131	0,718089267	0,61517435	0,538595112	0,096895004	0,082360754	0,200195339	0,291195339	**-0,968804661**
chrXIV	292461	292633	172	0,108283302	0,155646522	0,410273081	-0,226842569	-0,192816183	-0,010114481	-0,206114481	**-1,466114481**
chrXIV	292477	292630	153	0,313451664	0,248293262	0,557996432	0,14589138	0,124007673	0,084982024	0,021982024	**-1,238017976**
chrXIV	296854	297025	171	0,247911771	0,251999131	0,495911911	-0,010247499	-0,008710374	0,060048161	-0,128951839	**-1,388951839**
chrXIV	297410	297562	152	0,233663968	0,122293696	0,656437525	0,402759782	0,342345815	0,295759636	0,239759636	**-1,020240364**
chrXIV	298390	298549	159	0,284956058	0,255705001	0,527051197	0,06785934	0,057680439	-0,010091251	-0,115091251	**-1,375091251**
chrXIV	302946	303074	128	4,78441222	7,348739374	0,394325595	-0,268062527	-0,227853148	-0,070272057	0,041727943	**-1,218272057**
chrXIV	303880	304030	150	0,079787696	0,085235	0,483495289	-0,041382985	-0,035175537	-0,199278913	-0,241278913	**-1,501278913**
chrXIV	307042	307199	157	0,404637603	0,392822175	0,507408165	0,018570584	0,015784996	-0,062968104	-0,153968104	**-1,413968104**
chrXIV	308271	308446	175	0,108283302	0,181587609	0,37355698	-0,322447164	-0,274080089	-0,019761126	-0,236761126	**-1,496761126**
chrXIV	308271	308446	175	0,108283302	0,181587609	0,37355698	-0,322447164	-0,274080089	-0,019761126	-0,236761126	**-1,496761126**
chrXIV	309709	309860	151	2,00324109	1,801052615	0,52657372	0,066659774	0,056660808	-0,056820068	-0,105820068	**-1,365820068**
chrXIV	310999	311146	147	0,282106498	0,255705001	0,524545307	0,06156483	0,052330105	0,004283084	-0,016716916	**-1,276716916**
chrXIV	314726	314877	151	0,162424953	0,111176087	0,593656197	0,236960278	0,201416237	0,080915047	0,031915047	**-1,228084953**
chrXIV	317936	318050	114	0,037044288	0,604056741	0,057782293	-1,573666432	-1,337616467	-0,915967979	-0,705967979	**-1,965967979**
chrXIV	324620	324765	145	0,515770466	0,426175002	0,547558732	0,11949584	0,101571464	-0,067124924	-0,074124924	**-1,334124924**
chrXIV	324977	325117	140	1,473222822	0,841232394	0,636531142	0,349201932	0,296821642	0,148087413	0,176087413	**-1,083912587**
chrXIV	336864	337018	154	0,119681545	0,037058696	0,763566167	0,717820996	0,610147846	0,55897416	0,48897416	**-0,77102584**
chrXIV	339979	340132	153	0,176672756	0,289057827	0,379345404	-0,307200444	-0,261120377	-0,324791967	-0,387791967	**-1,647791967**
chrXIV	340143	340297	154	0,062690333	0,055588044	0,530023616	0,075329227	0,064029843	0,017904282	-0,052095718	**-1,312095718**
chrXIV	343939	344095	156	0,230814407	0,259410871	0,470833345	-0,073175214	-0,062198932	-0,173207307	-0,257207307	**-1,517207307**
chrXIV	348424	348579	155	0,450230572	0,303881305	0,597034188	0,245677844	0,208826167	0,14669575	0,06969575	**-1,19030425**
chrXIV	349341	349497	156	1,444727216	1,537935875	0,484374927	-0,03917627	-0,033299829	-0,152164237	-0,236164237	**-1,496164237**
chrXIV	357765	357910	145	13,52401453	8,049148725	0,626890659	0,323629327	0,275084928	0,102323921	0,095323921	**-1,164676079**
chrXIV	357767	357910	143	0,054141651	0,037058696	0,593656197	0,236960278	0,201416237	-0,029193499	-0,022193499	**-1,282193499**
chrXIV	358288	358439	151	0,056991212	0,040764565	0,582995843	0,209563572	0,178129036	0,039359196	-0,009640804	**-1,269640804**
chrXIV	360600	360759	159	0,037044288	0,044470435	0,454449042	-0,114428548	-0,097264266	-0,162373813	-0,267373813	**-1,527373813**
chrXIV	360762	360912	150	0,24506221	0,289057827	0,45881486	-0,103419898	-0,087906914	-0,247346224	-0,289346224	**-1,549346224**
chrXIV	361214	361352	138	2,128621756	1,993757833	0,516357533	0,041013751	0,034861688	-0,108493272	-0,066493272	**-1,326493272**
chrXIV	364273	364404	131	0,34194727	0,459527828	0,426647404	-0,184916144	-0,157178722	-0,061929249	0,029070751	**-1,230929249**
chrXIV	365246	365404	158	0,265009134	0,237175653	0,52771239	0,06952062	0,059092527	0,003919977	-0,094080023	**-1,354080023**
chrXIV	366145	366271	126	1,966196803	4,939924148	0,284703499	-0,568925058	-0,4835863	-0,213954433	-0,087954433	**-1,347954433**
chrXIV	368092	368240	148	0,119681545	0,074117392	0,617555219	0,299066166	0,254206241	0,155881285	0,127881285	**-1,132118715**
chrXIV	369049	369214	165	0,094035499	0,17417587	0,350602211	-0,38369513	-0,32614086	-0,308226837	-0,455226837	**-1,715226837**
chrXIV	376927	377089	162	1,547311397	1,356348266	0,532883181	0,082519467	0,070141547	0,11777006	-0,00822994	**-1,26822994**
chrXIV	384387	384546	159	0,909009826	0,678174133	0,572718632	0,183299871	0,15580489	0,097723837	-0,007276163	**-1,267276163**
chrXIV	388430	388576	146	0,056991212	0,066705652	0,460732874	-0,09858756	-0,083799426	-0,215183971	-0,229183971	**-1,489183971**
chrXIV	388719	388880	161	0,159575393	0,140823044	0,531212461	0,078318026	0,066570322	0,046245796	-0,072754204	**-1,332754204**
chrXIV	388719	388880	161	0,159575393	0,140823044	0,531212461	0,078318026	0,066570322	0,046245796	-0,072754204	**-1,332754204**
chrXIV	396710	396865	155	0,265009134	0,196411088	0,574333594	0,187418125	0,159305406	0,076318949	-0,000681051	**-1,260681051**
chrXIV	403433	403582	149	0,63545201	0,726350437	0,466625692	-0,083754801	-0,071191581	-0,191166722	-0,226166722	**-1,486166722**
chrXIV	403995	404145	150	0,088336378	0,051882174	0,629990659	0,331828607	0,282054316	0,129055182	0,087055182	**-1,172944818**
chrXIV	410286	410440	154	0,58415992	0,403939784	0,591195319	0,230620858	0,19602773	0,149937439	0,079937439	**-1,180062561**
chrXIV	410286	410440	154	0,58415992	0,403939784	0,591195319	0,230620858	0,19602773	0,149937439	0,079937439	**-1,180062561**
chrXIV	410317	410470	153	0,279256937	0,196411088	0,587083686	0,220049468	0,187042048	0,112976937	0,049976937	**-1,210023063**
chrXIV	410317	410470	153	0,279256937	0,196411088	0,587083686	0,220049468	0,187042048	0,112976937	0,049976937	**-1,210023063**
chrXIV	410968	411126	158	0,569912117	0,396528045	0,589702435	0,226779597	0,192762657	0,143438401	0,045438401	**-1,214561599**
chrXIV	411008	411145	137	2,131471316	1,778817398	0,545093079	0,113273355	0,096282351	-0,001747297	0,047252703	**-1,212747297**
chrXIV	416136	416292	156	5,704820288	4,302514581	0,570063894	0,176536837	0,150056312	0,031765787	-0,052234213	**-1,312234213**
chrXIV	418446	418610	164	0,039893848	0,08894087	0,309651379	-0,496838761	-0,422312947	-0,355455222	-0,495455222	**-1,755455222**
chrXIV	419242	419381	139	1,068585219	1,048761091	0,504681362	0,011734703	0,009974498	-0,142723137	-0,107723137	**-1,367723137**
chrXIV	420269	420420	151	0,056991212	0,066705652	0,460732874	-0,09858756	-0,083799426	-0,21596795	-0,26496795	**-1,52496795**
chrXIV	421794	421931	137	0,897611538	0,741173916	0,547729757	0,119927619	0,101938476	0,009029625	0,058029625	**-1,201970375**
chrXIV	421911	422067	156	0,222265726	0,233469783	0,487707719	-0,030817056	-0,026194498	-0,12679972	-0,21079972	**-1,47079972**
chrXIV	423473	423612	139	0,051292091	0,044470435	0,535617563	0,08939893	0,07598909	-0,078502101	-0,043502101	**-1,303502101**
chrXIV	423497	423650	153	0,575611238	0,507704132	0,531342261	0,07864439	0,066847732	-0,005487487	-0,068487487	**-1,328487487**
chrXIV	426375	426492	117	0,116831984	0,61146848	0,160417286	-0,992744317	-0,843832669	-0,415910803	-0,226910803	**-1,486910803**
chrXIV	430126	430279	153	0,039893848	0,125999566	0,240478794	-0,704763239	-0,599048753	-0,663508783	-0,726508783	**-1,986508783**
chrXIV	430126	430286	160	8,560079993	6,852152851	0,555408167	0,139337237	0,118436651	0,074584108	-0,037415892	**-1,297415892**
chrXIV	439717	439834	117	0,262159574	0,893114568	0,226924125	-0,749014859	-0,63666263	-0,232695242	-0,043695242	**-1,303695242**
chrXIV	459511	459626	115	0,091185939	0,885702829	0,093343215	-1,32044517	-1,122378395	-0,661924657	-0,458924657	**-1,718924657**
chrXIV	460164	460311	147	0,524319147	0,552174567	0,487061968	-0,032436523	-0,027571045	-0,078910805	-0,099910805	**-1,359910805**
chrXIV	464412	464538	126	2,211259013	4,643454582	0,322589556	-0,460469706	-0,39139925	-0,143312664	-0,017312664	**-1,277312664**
chrXIV	466515	466659	144	1,065735658	0,648527176	0,621687431	0,309915603	0,263428262	0,117888154	0,117888154	**-1,142111846**
chrXIV	467295	467445	150	0,236513528	0,222352175	0,515430826	0,038688995	0,032885645	-0,129666583	-0,171666583	**-1,431666583**
chrXIV	473999	474106	107	0,296354301	2,449579791	0,107924768	-1,237640106	-1,05199409	-0,505815325	-0,246815325	**-1,506815325**
chrXIV	475462	475606	144	0,378991558	0,200116957	0,654439622	0,397334573	0,337734387	0,200882007	0,200882007	**-1,059117993**
chrXIV	477594	477739	145	0,763682236	0,392822175	0,660336639	0,413382053	0,351374745	0,168638388	0,161638388	**-1,098361612**
chrXIV	478141	478275	134	1,008744447	1,304466092	0,436079825	-0,160915886	-0,136778503	-0,107732191	-0,037732191	**-1,297732191**
chrXIV	483192	483305	113	0,065539893	0,43729261	0,130341402	-1,124778745	-0,956061933	-0,533537317	-0,316537317	**-1,576537317**
chrXIV	483272	483422	150	0,065539893	0,070411522	0,4820832	-0,044925865	-0,038186985	-0,194162295	-0,236162295	**-1,496162295**
chrXIV	483529	483666	137	0,037044288	0,100058479	0,270193583	-0,612227627	-0,520393483	-0,623991947	-0,574991947	**-1,834991947**
chrXIV	483529	483666	137	0,037044288	0,100058479	0,270193583	-0,612227627	-0,520393483	-0,623991947	-0,574991947	**-1,834991947**
chrXIV	483752	483901	149	0,159575393	0,107470218	0,597558568	0,24703277	0,209977854	0,093960203	0,058960203	**-1,201039797**
chrXIV	484694	484843	149	1,008744447	0,796761959	0,558704441	0,147685318	0,125532521	0,004009087	-0,030990913	**-1,290990913**
chrXIV	490422	490573	151	0,609805965	0,600350872	0,503906557	0,009792443	0,008323576	-0,116680975	-0,165680975	**-1,425680975**
chrXIV	490584	490699	115	0,042743409	0,044470435	0,490098899	-0,024820929	-0,021097789	0,455546777	0,658546777	**-0,601453223**
chrXIV	490584	490718	134	0,071239015	0,077823261	0,477914444	-0,055388588	-0,0470803	-0,013006182	0,056993818	**-1,203006182**
chrXIV	490584	490722	138	14,74362646	9,32026199	0,612686785	0,286328634	0,243379339	0,095219327	0,137219327	**-1,122780673**
chrXIV	500789	500918	129	0,082637257	0,055588044	0,597844653	0,247772164	0,210606339	0,40210454	0,50710454	**-0,75289546**
chrXIV	502692	502828	136	6,120856133	4,117221102	0,597852115	0,247791452	0,210622734	0,156326381	0,212326381	**-1,047673619**
chrXIV	511107	511261	154	0,142478029	0,096352609	0,596565123	0,24446623	0,207796296	0,163045148	0,093045148	**-1,166954852**
chrXIV	515776	515877	101	1,991842848	19,60405007	0,092232484	-1,327132162	-1,128062338	-0,710420801	-0,409420801	**-1,669420801**
chrXIV	516941	517091	150	0,250761331	0,08894087	0,738179884	0,637744162	0,542082538	0,379774061	0,337774061	**-0,922225939**
chrXIV	517457	517589	132	3,428021382	3,980103927	0,462738039	-0,093538105	-0,079507389	-0,015756178	0,068243822	**-1,191756178**
chrXIV	517457	517589	132	3,428021382	3,980103927	0,462738039	-0,093538105	-0,079507389	-0,015756178	0,068243822	**-1,191756178**
chrXIV	520229	520363	134	0,142478029	0,103764348	0,578608892	0,198335964	0,168585569	0,18908234	0,25908234	**-1,00091766**
chrXIV	520229	520369	140	0,182371877	0,107470218	0,629211148	0,329764776	0,28030006	0,114967032	0,142967032	**-1,117032968**
chrXIV	521272	521407	135	0,076938136	0,062999783	0,549801916	0,125160904	0,106386768	0,093589642	0,156589642	**-1,103410358**
chrXIV	521719	521865	146	0,065539893	0,048176305	0,576346155	0,192554744	0,163671533	0,051689341	0,037689341	**-1,222310659**
chrXIV	521719	521869	150	8,913425505	4,161691537	0,681709041	0,472483219	0,401610736	0,222315472	0,180315472	**-1,079684528**
chrXIV	521898	522055	157	0,068389454	0,059293913	0,535617563	0,08939893	0,07598909	-0,007889802	-0,098889802	**-1,358889802**
chrXIV	524015	524129	114	0,028495606	0,218646305	0,115300581	-1,198811728	-1,018989969	-0,570338374	-0,360338374	**-1,620338374**
chrXIV	524719	524868	149	0,09688506	0,074117392	0,566571175	0,16765125	0,142503563	0,019901778	-0,015098222	**-1,275098222**
chrXIV	524979	525110	131	0,857717736	1,330407179	0,391987555	-0,274142505	-0,233021129	-0,13452413	-0,04352413	**-1,30352413**
chrXIV	526013	526193	180	0,182371877	0,333528262	0,353502284	-0,375882075	-0,319499764	-0,016319167	-0,268319167	**-1,528319167**

Chrom	Start	End	Length	Section A	Section B	A/A+B	Z-score	Z * 0.85	Phase correction	Length correction	ΔLKnuc
chrXIV	526651	526800	149	1,193965884	0,900526308	0,570050291	0,176502204	0,150026874	0,021648155	-0,013351845	**-1,273351845**
chrXIV	527097	527235	138	3,903897999	3,135165663	0,55460473	0,13730396	0,116708366	-0,052138369	-0,010138369	**-1,270138369**
chrXIV	527709	527871	162	0,638301571	0,492880654	0,564278289	0,16182531	0,137551513	0,197370356	0,071370356	**-1,188629644**
chrXIV	529282	529444	162	0,589859041	0,433586741	0,576346155	0,192554744	0,163671533	0,22846521	0,10246521	**-1,15753479**
chrXIV	529934	530079	145	0,894762023	0,689291742	0,564855842	0,163292275	0,138798434	-0,043222683	-0,050222683	**-1,310222683**
chrXIV	530913	531056	143	0,339097709	0,085235	0,799131676	0,83852369	0,712745137	0,486781727	0,493781727	**-0,766218273**
chrXIV	532041	532181	140	2,499064632	1,686170658	0,597114489	0,245885302	0,209002507	0,018558882	0,046558882	**-1,213441118**
chrXIV	532139	532286	147	0,094035499	0,077823261	0,547167331	0,118507771	0,100731606	0,036669403	0,015669403	**-1,244330597**
chrXIV	536984	537144	160	0,054141651	0,059293913	0,477289917	-0,056956516	-0,048413039	-0,088141096	-0,200141096	**-1,460141096**
chrXIV	538071	538217	146	0,079787696	0,051882174	0,605967759	0,268824834	0,228501109	0,11177236	0,09777236	**-1,16222764**
chrXIV	538102	538265	163	0,262159574	0,363175219	0,419230749	-0,203861798	-0,173282528	-0,088478864	-0,221478864	**-1,481478864**
chrXIV	538210	538368	158	0,296354301	0,340940001	0,465019536	-0,087795679	-0,074626327	-0,124036228	-0,222036228	**-1,482036228**
chrXIV	540636	540777	141	0,210867483	0,122293696	0,632929334	0,339621835	0,28867856	0,061928147	0,082928147	**-1,177071853**
chrXIV	541287	541432	145	0,091185939	0,044470435	0,672183225	0,445949741	0,37905728	0,223403711	0,216403711	**-1,043596289**
chrXIV	541780	541943	163	1,875010864	2,078992834	0,474205643	-0,064701982	-0,054996684	0,043282864	-0,089717136	**-1,349717136**
chrXIV	541920	542078	158	0,213717044	0,122293696	0,6360423	0,347899847	0,29571487	0,246037814	0,148037814	**-1,111962186**
chrXIV	542491	542640	149	0,381841118	0,226058044	0,628132331	0,32691082	0,277874197	0,145873261	0,110873261	**-1,149126739**
chrXIV	544884	545027	143	0,116831984	0,096352609	0,548032024	0,120690792	0,102587173	-0,107243496	-0,100243496	**-1,360243496**
chrXIV	544927	545097	170	0,208017923	0,163058261	0,560580095	0,152440128	0,129574109	0,189822255	0,007822255	**-1,252177745**
chrXIV	546338	546446	108	0,250761331	0,570703915	0,305261035	-0,509328378	-0,432929121	0,119522807	0,371522807	**-0,888477193**
chrXIV	546358	546520	162	0,344796831	0,285351958	0,547167331	0,118507771	0,100731606	0,163634961	0,037634961	**-1,222365039**
chrXIV	551772	551902	130	0,085486818	0,144528914	0,371656395	-0,327469522	-0,278349094	-0,115782883	-0,017782883	**-1,277782883**
chrXIV	552571	552722	151	0,094035499	0,085235	0,524545307	0,06156483	0,052330105	-0,066558203	-0,115558203	**-1,375558203**
chrXIV	553534	553684	150	0,276407377	0,200116957	0,580048818	0,202018369	0,171715614	-0,002337208	-0,044337208	**-1,304337208**
chrXIV	554534	554685	151	2,114373953	1,771405659	0,544131208	0,110847113	0,094220046	-0,022815719	-0,071815719	**-1,331815719**
chrXIV	555938	556085	147	0,227964847	0,133411305	0,630824269	0,334037235	0,283931649	0,213904519	0,192904519	**-1,067095481**
chrXIV	557972	558127	155	1,193965884	0,911643916	0,567040429	0,168844262	0,143517622	0,063729903	-0,013270097	**-1,273270097**
chrXIV	560057	560198	141	0,569912117	0,507704132	0,5288637	0,072413804	0,061551733	-0,167557556	-0,146557556	**-1,406557556**
chrXIV	561823	561978	155	0,045592969	0,081529131	0,358654941	-0,362056402	-0,307747942	-0,390683534	-0,467683534	**-1,727683534**
chrXIV	562224	562373	149	0,065539893	0,074117392	0,469290903	-0,077052467	-0,065494597	-0,218251688	-0,253251688	**-1,513251688**
chrXIV	563011	563177	166	0,034194727	0,092646739	0,269586342	-0,614064532	-0,521954852	-0,476085624	-0,630085624	**-1,890085624**
chrXIV	564309	564452	143	0,210867483	0,107470218	0,662401854	0,419027147	0,356173075	0,139623702	0,146623702	**-1,113376298**
chrXIV	565788	565917	129	0,176672756	0,385410436	0,314317807	-0,483648127	-0,411100908	-0,217879427	-0,112879427	**-1,372879427**
chrXIV	567163	567273	110	0,122531105	0,737468046	0,14247817	-1,069251542	-0,90886381	-0,450287582	-0,212287582	**-1,472287582**
chrXIV	573304	573437	133	0,507221784	0,648527176	0,438868475	-0,15383866	-0,130762861	-0,100556835	-0,023556835	**-1,283556835**
chrXIV	575453	575613	160	0,045592969	0,055588044	0,450607955	-0,124125498	-0,105506674	-0,137219608	-0,249219608	**-1,509219608**
chrXIV	575453	575613	160	0,045592969	0,055588044	0,450607955	-0,124125498	-0,105506674	-0,137219608	-0,249219608	**-1,509219608**
chrXIV	576714	576844	130	0,068389454	0,037058696	0,648560019	0,381435717	0,324220359	0,481057975	0,579057975	**-0,680942025**
chrXIV	576714	576864	150	0,983098041	0,718938698	0,577601041	0,195760131	0,166396111	-0,00342841	-0,04542841	**-1,30542841**
chrXIV	578365	578517	152	0,270708255	0,17788174	0,603464764	0,262325541	0,22297671	0,18262252	0,12662252	**-1,13337748**
chrXIV	578706	578859	153	1,629948654	1,193290004	0,577332933	0,195075126	0,165813857	0,111729546	0,048729546	**-1,211270454**
chrXIV	585214	585366	152	0,361894194	0,326116523	0,52600081	0,065220573	0,055437487	0,020630963	-0,035369037	**-1,295369037**
chrXIV	589016	589141	125	0,111132863	0,244587392	0,31241646	-0,489012398	-0,415660538	-0,066717391	0,066282609	**-1,193717391**
chrXIV	589016	589141	125	0,111132863	0,244587392	0,31241646	-0,489012398	-0,415660538	-0,066717391	0,066282609	**-1,193717391**
chrXIV	593245	593393	148	0,170973635	0,125999566	0,575720754	0,190958009	0,162314307	0,064164726	0,036164726	**-1,223835274**
chrXIV	597466	597618	152	0,461628815	0,237175653	0,660597973	0,414095655	0,351981307	0,320194295	0,264194295	**-0,995805705**
chrXIV	598791	598924	133	0,435982769	0,70411522	0,382408156	-0,299162171	-0,254287846	-0,232326611	-0,155326611	**-1,415326611**
chrXIV	602237	602346	109	0,094035499	0,051882174	0,644442151	0,370358087	0,314804374	0,75001835	0,99501835	**-0,26498165**
chrXIV	612323	612470	147	0,282106498	0,203822827	0,58055047	0,203301913	0,172806626	0,101687879	0,080687879	**-1,179312121**
chrXIV	615779	615947	168	0,068389454	0,114881957	0,373159423	-0,323497043	-0,274972487	-0,244435031	-0,412435031	**-1,672435031**
chrXIV	620267	620398	131	0,823523009	1,830699572	0,310269009	-0,495087981	-0,420824784	-0,333545596	-0,242545596	**-1,502545596**
chrXIV	620267	620398	131	0,823523009	1,830699572	0,310269009	-0,495087981	-0,420824784	-0,333545596	-0,242545596	**-1,502545596**
chrXIV	620785	620892	107	0,045592969	0,26311674	0,147688809	-1,046397345	-0,889437743	-0,342314104	-0,083314104	**-1,343314104**
chrXIV	620785	620946	161	9,184133761	8,571676335	0,517246676	0,043244482	0,036757809	0,032259613	-0,086740387	**-1,346740387**
chrXIV	620785	620946	161	9,184133761	8,571676335	0,517246676	0,043244482	0,036757809	0,032259613	-0,086740387	**-1,346740387**
chrXIV	625000	625108	108	0,809275206	2,101228051	0,278053359	-0,588634154	-0,500339031	0,012499131	0,264499131	**-0,995500869**
chrXIV	625453	625609	156	2,937896962	2,490344357	0,541224457	0,103518981	0,087991134	-0,014908464	-0,098908464	**-1,358908464**
chrXIV	630331	630483	152	0,102584181	0,107470218	0,488369592	-0,029157241	-0,024783655	-0,067371137	-0,123371137	**-1,383371137**
chrXIV	630745	630896	151	0,210867483	0,133411305	0,612490489	0,28581604	0,242943634	0,125014332	0,076014332	**-1,183985668**
chrXIV	630745	630896	151	0,210867483	0,133411305	0,612490489	0,28581604	0,242943634	0,125014332	0,076014332	**-1,183985668**
chrXIV	635632	635775	143	0,09688506	0,107470218	0,47410109	-0,064964608	-0,055219916	-0,261614084	-0,254614084	**-1,514614084**
chrXIV	640348	640505	157	0,749434433	0,503998263	0,597905604	0,247929711	0,210740254	0,13419036	0,04319036	**-1,21680964**
chrXIV	640382	640540	158	0,210867483	0,155646522	0,575332675	0,189967434	0,161472318	0,09939658	0,00139658	**-1,25860342**
chrXIV	640382	640540	158	0,210867483	0,155646522	0,575332675	0,189967434	0,161472318	0,09939658	0,00139658	**-1,25860342**
chrXIV	641911	642059	148	0,190920559	0,188999349	0,502528442	0,006337906	0,00538722	-0,085270044	-0,113270044	**-1,373270044**
chrXIV	645323	645472	149	2,291046709	1,649111962	0,58146052	0,205631256	0,174786568	0,023568498	-0,011431502	**-1,271431502**
chrXIV	645323	645472	149	2,291046709	1,649111962	0,58146052	0,205631256	0,174786568	0,023568498	-0,011431502	**-1,271431502**
chrXIV	646222	646370	148	0,29065518	0,222352175	0,566571175	0,16765125	0,142503563	0,046928814	0,018928814	**-1,241071186**
chrXIV	649139	649287	148	0,259310013	0,285351958	0,47609348	-0,059960669	-0,050966568	-0,152489374	-0,180489374	**-1,440489374**
chrXIV	649139	649287	148	0,259310013	0,285351958	0,47609348	-0,059960669	-0,050966568	-0,152489374	-0,180489374	**-1,440489374**
chrXIV	659033	659204	171	0,256460453	0,281646088	0,47659791	-0,058694023	-0,04988992	0,063217926	-0,125782074	**-1,385782074**
chrXIV	660122	660273	151	0,059840772	0,059293913	0,502295129	0,005753068	0,004890107	-0,106174214	-0,155174214	**-1,415174214**
chrXIV	660694	660857	163	4,764465296	4,610101756	0,508233102	0,020638792	0,017542973	0,114612946	-0,018387054	**-1,278387054**
chrXIV	669731	669882	151	0,364743755	0,211234566	0,63325952	0,340498754	0,289423941	0,182739708	0,133739708	**-1,126260292**
chrXIV	673304	673429	125	0,04844253	1,119172613	0,041488439	-1,733668726	-1,473618417	-1,106527638	-0,973527638	**-2,233527638**
chrXIV	673304	673441	137	0,031345166	0,040764565	0,434687048	-0,164453569	-0,139785534	-0,256748695	-0,207748695	**-1,467748695**
chrXIV	673304	673449	145	0,034194727	0,059293913	0,365763444	-0,34309514	-0,291630869	-0,436894619	-0,443894619	**-1,703894619**
chrXIV	673304	673450	146	0,068389454	0,066705652	0,506231912	0,015621722	0,013278464	-0,093126653	-0,107126653	**-1,367126653**
chrXIV	673304	673451	147	0,133929347	0,255705001	0,343730854	-0,402302101	-0,341956786	-0,408340989	-0,429340989	**-1,689340989**
chrXIV	673304	673452	148	0,108283302	0,151940653	0,416115812	-0,211840305	-0,180064259	-0,278576302	-0,306576302	**-1,566576302**
chrXIV	673304	673453	149	0,065539893	0,070411522	0,4820832	-0,044925865	-0,038186985	-0,183425504	-0,218425504	**-1,478425504**
chrXIV	673304	673454	150	0,119681545	0,133411305	0,472876041	-0,06804215	-0,057835827	-0,244023022	-0,286023022	**-1,546023022**
chrXIV	673304	673455	151	50,87035554	51,7487628	0,49572006	-0,010728423	-0,00911916	-0,095266426	-0,144266426	**-1,404266426**
chrXIV	673305	673455	150	0,051292091	0,188999349	0,213457836	-0,794480647	-0,67530855	-0,846858369	-0,888858369	**-2,148858369**
chrXIV	673306	673455	149	0,082637257	0,185293479	0,308427686	-0,500312047	-0,42526524	-0,562060061	-0,597060061	**-1,857060061**
chrXIV	673307	673455	148	0,051292091	0,314998914	0,140030986	-1,080180137	-0,918153116	-1,008895662	-1,036895662	**-2,296895662**
chrXIV	673308	673455	147	0,102584181	0,185293479	0,356346446	-0,368241871	-0,31300559	-0,385660036	-0,406660036	**-1,666660036**
chrXIV	673309	673455	146	0,510071344	0,629997828	0,447403856	-0,132223249	-0,112389761	-0,212343852	-0,226343852	**-1,486343852**
chrXIV	673310	673455	145	0,037044288	0,100058479	0,270193583	-0,612227627	-0,520393483	-0,663751251	-0,670751251	**-1,930751251**
chrXIV	673311	673455	144	0,042743409	0,066705652	0,390532439	-0,277931585	-0,236241848	-0,363067519	-0,363067519	**-1,623067519**
chrXIV	673313	673455	142	0,031345166	0,129705435	0,194629303	-0,860962664	-0,731818264	-0,984529351	-0,970529351	**-2,230529351**
chrXIV	673314	673455	141	0,034194727	0,151940653	0,183708906	-0,901320735	-0,766122625	-0,986408159	-0,965408159	**-2,225408159**
chrXIV	673321	673455	134	0,037044288	0,103764348	0,263082498	-0,633871027	-0,538790373	-0,526359174	-0,456359174	**-1,716359174**
chrXIV	673329	673455	126	0,042743409	0,08894087	0,324590066	-0,454901459	-0,386666241	-0,160813587	-0,034813587	**-1,294813587**
chrXIV	673357	673455	98	0,037044288	0,296469566	0,11107271	-1,22084313	-1,037716661	-0,594138512	-0,272138512	**-1,532138512**
chrXIV	675190	675330	140	0,133929347	0,044470435	0,750725957	0,676776004	0,575259604	0,366676266	0,394676266	**-0,865323734**

Chrom	Start	End	Length	Section A	Section B	A/A+B	Z-score	Z * 0.85	Phase correction	Length correction	ΔLKnuc
chrXIV	678978	679124	146	0,450230572	0,274234349	0,621466353	0,309334231	0,262934096	0,167860777	0,153860777	**-1,106139223**
chrXIV	679561	679717	156	0,852018614	0,904232177	0,485134935	-0,037269817	-0,031679345	-0,11938089	-0,20338089	**-1,46338089**
chrXIV	681968	682096	128	13,28180188	21,15680942	0,385666012	-0,290632993	-0,247038044	-0,072429156	0,039570844	**-1,220429156**
chrXIV	681972	682075	103	0,094035499	0,148234783	0,388142938	-0,284162468	-0,241538098	0,172555612	0,459555612	**-0,800444388**
chrXIV	682856	682998	142	0,125380666	0,140823044	0,470995186	-0,072768458	-0,061853189	-0,298416865	-0,284416865	**-1,544416865**
chrXIV	689540	689646	106	0,028495606	0,655938915	0,041633794	-1,732033557	-1,472228523	-0,95185362	-0,68585362	**-1,94585362**
chrXIV	689540	689701	161	0,074088575	0,085235	0,465019536	-0,087795679	-0,074626327	-0,052265612	-0,171265612	**-1,431265612**
chrXIV	689540	689704	164	0,039893848	0,103764348	0,277699771	-0,589688448	-0,501235181	-0,435952551	-0,575952551	**-1,835952551**
chrXIV	689540	689705	165	23,29800733	18,57011246	0,556461753	0,142004446	0,120703779	0,145191745	-0,001808255	**-1,261808255**
chrXIV	689541	689705	164	0,051292091	0,096352609	0,34740218	-0,39234359	-0,333492051	-0,264238324	-0,404238324	**-1,664238324**
chrXIV	689543	689705	162	0,076938136	0,100058479	0,434687048	-0,164453569	-0,139785534	-0,06765219	-0,19365219	**-1,45365219**
chrXIV	689545	689705	160	0,042743409	0,085235	0,333989218	-0,428924135	-0,364585514	-0,392409185	-0,504409185	**-1,764409185**
chrXIV	689550	689705	155	0,037044288	0,059293913	0,384523349	-0,293622086	-0,249578773	-0,345624926	-0,422624926	**-1,682624926**
chrXIV	689554	689705	151	0,028495606	0,044470435	0,390532439	-0,277931585	-0,236241848	-0,318769115	-0,367769115	**-1,627769115**
chrXIV	694543	694689	146	0,142478029	0,059293913	0,706134002	0,542125583	0,460806745	0,361101543	0,347101543	**-0,912898457**
chrXIV	694549	694699	150	0,14532759	0,044470435	0,765696008	0,724745812	0,61603394	0,440619976	0,398619976	**-0,861380024**
chrXIV	698880	699032	152	1,276603141	0,978349569	0,566132999	0,166537466	0,141556846	0,095273632	0,039273632	**-1,220726368**
chrXIV	704009	704157	148	2,7327286	1,426759788	0,656986712	0,404253146	0,343615174	0,25184921	0,22384921	**-1,03615079**
chrXIV	704660	704785	125	0,088336378	0,055588044	0,613769206	0,28915654	0,245783059	0,634147349	0,767147349	**-0,492852651**
chrXIV	705096	705262	166	0,911859387	1,030231743	0,46952451	-0,076465173	-0,064995397	-0,016998624	-0,170998624	**-1,430998624**
chrXIV	705096	705262	166	0,911859387	1,030231743	0,46952451	-0,076465173	-0,064995397	-0,016998624	-0,170998624	**-1,430998624**
chrXIV	705533	705682	149	0,626903328	0,396528045	0,612550431	0,285972559	0,243076675	0,101234006	0,066234006	**-1,193765994**
chrXIV	705820	705974	154	0,322000346	0,322410653	0,499681642	-0,000798006	-0,000678305	-0,03754358	-0,10754358	**-1,36754358**
chrXIV	706013	706167	154	0,740885752	0,61146848	0,54784888	0,120228376	0,10219412	0,06979743	-0,00020257	**-1,26020257**
chrXIV	706675	706825	150	0,279256937	0,170470001	0,620947766	0,307970909	0,261775272	0,086785026	0,044785026	**-1,215214974**
chrXIV	706675	706825	150	0,279256937	0,170470001	0,620947766	0,307970909	0,261775272	0,086785026	0,044785026	**-1,215214974**
chrXIV	707449	707602	153	0,094035499	0,118587827	0,442263326	-0,145233329	-0,12344833	-0,169744118	-0,232744118	**-1,492744118**
chrXIV	723355	723513	158	0,19661968	0,107470218	0,646584058	0,376114353	0,3196972	0,25085812	0,15285812	**-1,10714188**
chrXIV	724785	724950	165	0,447381012	0,389116306	0,534826595	0,087408503	0,074297228	0,111890638	-0,035109362	**-1,295109362**
chrXIV	727236	727386	150	0,208017923	0,100058479	0,675215374	0,454360676	0,386206574	0,215211041	0,173211041	**-1,086788959**
chrXIV	727633	727771	138	1,461824579	1,360054135	0,518032392	0,045215905	0,038433519	-0,126328093	-0,084328093	**-1,344328093**
chrXIV	731977	732139	162	0,700991904	0,896820438	0,438719795	-0,154215792	-0,131083423	-0,057442498	-0,183442498	**-1,443442498**
chrXIV	735524	735689	165	0,364743755	0,552174567	0,397793071	-0,259063637	-0,220204091	-0,17166689	-0,31866689	**-1,57866689**
chrXIV	735878	736030	152	1,886409106	1,104349134	0,630746103	0,333830067	0,283755557	0,232779739	0,176779739	**-1,083220261**
chrXIV	737786	737917	131	0,598407723	1,019114134	0,369953407	-0,33197675	-0,282180238	-0,207817347	-0,116817347	**-1,376817347**
chrXIV	749149	749259	110	0,319150785	0,859761742	0,270716256	-0,610648191	-0,519050963	-0,09529556	0,14270444	**-1,11729556**
chrXIV	749725	749855	130	0,04844253	0,122293696	0,283727309	-0,571804221	-0,486033588	-0,345726378	-0,247726378	**-1,507726378**
chrXIV	749823	749962	139	0,44168189	0,192705218	0,696234026	0,513599615	0,436559673	0,290810057	0,325810057	**-0,934189943**
chrXIV	750208	750335	127	0,045592969	0,17417587	0,207458753	-0,815270475	-0,692979903	-0,48698518	-0,36798518	**-1,62798518**
chrXIV	753363	753527	164	0,105433742	0,203822827	0,340926442	-0,409936016	-0,348445614	-0,280839786	-0,420839786	**-1,680839786**
chrXIV	754387	754526	139	1,068585219	0,811585438	0,568344802	0,172161713	0,146337456	0,003546114	0,038546114	**-1,221453886**
chrXIV	761541	761643	102	0,190920559	1,964110877	0,088592935	-1,349470563	-1,147049979	-0,758150947	-0,464150947	**-1,724150947**
chrXIV	763360	763525	165	0,062690333	0,062999783	0,498768996	-0,003085674	-0,002622823	0,037983738	-0,109016262	**-1,369016262**
chrXIV	763360	763525	165	0,062690333	0,062999783	0,498768996	-0,003085674	-0,002622823	0,037983738	-0,109016262	**-1,369016262**
chrXIV	767084	767240	156	0,208017923	0,185293479	0,528888615	0,072476421	0,061604958	-0,032752679	-0,116752679	**-1,376752679**
chrXIV	773470	773620	150	0,364743755	0,185293479	0,663125571	0,421008524	0,357857245	0,196066768	0,154066768	**-1,105933232**
chrXIV	778474	778625	151	0,105433742	0,155646522	0,403836506	-0,243429088	-0,206914725	-0,293455578	-0,342455578	**-1,602455578**
chrXIV	779862	780020	158	0,136778908	0,137117174	0,499382491	-0,001547865	-0,001315685	-0,061903906	-0,159903906	**-1,419903906**
chrXIV	781645	781793	148	1,399134247	0,837526525	0,625546021	0,320079643	0,272067696	0,182403555	0,154403555	**-1,105596445**
chrXIV	781935	782064	129	0,19377012	0,333528262	0,367477175	-0,338542574	-0,287761188	-0,103754213	0,001245787	**-1,258754213**
chrXIV	781942	782106	164	0,125380666	0,081529131	0,605967759	0,268824834	0,228501109	0,30102777	0,16102777	**-1,09897223**
chrXIV	783376	783501	125	0,113982423	0,114881957	0,498034789	-0,004926073	-0,004187162	0,36495017	0,49795017	**-0,76204983**
chrXIV	784039	784210	171	2,19986077	0,055588044	0,975353888	1,966055295	1,671147001	1,807247291	1,618247291	**0,358247291**
chrXIV	784040	784199	159	0,071239015	0,155646522	0,313986584	-0,484581602	-0,411894361	-0,470059105	-0,575059105	**-1,835059105**
chrXIV	784044	784183	139	1,36493952	0,559586306	0,709234192	0,551148886	0,468476553	0,32866437	0,36366437	**-0,89633563**
chrXIV	784044	784199	155	0,094035499	0,137117174	0,406811212	-0,235755471	-0,20039215	-0,299685829	-0,376685829	**-1,636685829**
chrXIV	784049	784198	149	1,701187668	0,874585221	0,660457168	0,413711146	0,351654474	0,207740276	0,172740276	**-1,087259724**
chrXIV	784049	784203	154	0,190920559	0,114881957	0,624326319	0,316863258	0,269333769	0,231057712	0,161057712	**-1,098942288**
chrXIV	784063	784183	120	0,111132863	0,122293696	0,47609348	-0,059960669	-0,050966568	0,306770056	0,474770056	**-0,785229944**
chrXIV	784063	784195	132	0,151026711	0,062999783	0,705644933	0,540706158	0,459600234	0,503045964	0,587045964	**-0,672954036**
chrXIV	784063	784199	136	1,977595045	1,660229571	0,543620227	0,109558474	0,093124703	0,018343149	0,074343149	**-1,185656851**
chrXIV	784063	784202	139	0,19661968	0,070411522	0,736317249	0,632032712	0,537227806	0,403542322	0,438542322	**-0,821457678**
chrXIV	784063	784210	147	0,703841464	0,415057393	0,629048336	0,329333894	0,27993381	0,209313897	0,188313897	**-1,071686103**
chrXIV	784063	784233	170	0,173823196	0,218646305	0,44289606	-0,14363067	-0,12208607	-0,044291294	-0,226291294	**-1,486291294**
chrXIV	784063	784239	176	0,071239015	0,040764565	0,6360423	0,347899847	0,29571487	0,591713451	0,367713451	**-0,892286549**
chrXIV	784063	784239	176	0,071239015	0,040764565	0,6360423	0,347899847	0,29571487	0,591713451	0,367713451	**-0,892286549**
chrXIV	784063	784275	212	0,028495606	0,103764348	0,215451503	-0,787647358	-0,669500254	-0,227608692	-0,703608692	**-1,963608692**
chrXIV	784063	784275	212	0,028495606	0,103764348	0,215451503	-0,787647358	-0,669500254	-0,227608692	-0,703608692	**-1,963608692**
chrXIV	784064	784183	119	0,3903898	0,222352175	0,637119401	0,35076961	0,298154169	0,703632911	0,878632911	**-0,381367089**
chrXIV	784064	784199	135	0,082637257	0,048176305	0,631717813	0,336406466	0,285945496	0,278049809	0,341049809	**-0,918950191**
chrXIV	784101	784203	102	0,028495606	0,051882174	0,354520937	-0,373143192	-0,317171714	0,096134907	0,390134907	**-0,869865093**
chrXIV	784101	784210	109	0,185221438	0,062999783	0,746195017	0,662563791	0,563179222	1,013116549	1,258116549	**-0,001883451**
chrXIV	784106	784198	92	0,364743755	1,945581529	0,157875498	-1,003227731	-0,852743571	-0,509753408	-0,145753408	**-1,405753408**
chrXIV	784106	784203	97	0,111132863	0,259410871	0,299918344	-0,524635379	-0,445940072	-0,003219363	0,325780637	**-0,934219363**
chrXIV	784106	784210	104	0,589859041	0,244587392	0,706886647	0,544312122	0,462665304	0,925669973	1,205669973	**-0,054330027**
chrXIV	784106	784238	132	0,384690679	0,122293696	0,75878212	0,702390353	0,5970318	0,629118529	0,713118529	**-0,546881471**
chrXIV	784106	784238	132	0,384690679	0,122293696	0,75878212	0,702390353	0,5970318	0,629118529	0,713118529	**-0,546881471**
chrXIV	784106	784265	159	0,265009134	0,107470218	0,711473355	0,557694096	0,474039982	0,423732317	0,318732317	**-0,941267683**
chrXIV	784106	784265	159	0,265009134	0,107470218	0,711473355	0,557694096	0,474039982	0,423732317	0,318732317	**-0,941267683**
chrXIV	784106	784274	168	2,826764099	0,366881088	0,885121525	1,200985181	1,020837404	1,045628256	0,877628256	**-0,382371744**
chrXIV	784106	784274	168	2,826764099	0,366881088	0,885121525	1,200985181	1,020837404	1,045628256	0,877628256	**-0,382371744**
chrXIV	784106	784275	169	0,079787696	0,077823261	0,506231912	0,015621722	0,013278464	0,045024016	-0,129975984	**-1,389975984**
chrXIV	784106	784275	169	0,079787696	0,077823261	0,506231912	0,015621722	0,013278464	0,045024016	-0,129975984	**-1,389975984**
chrXIV	784106	784277	171	0,19661968	0,081529131	0,706886647	0,544312122	0,462665304	0,62594496	0,43694496	**-0,82305504**
chrXIV	784106	784277	171	0,19661968	0,081529131	0,706886647	0,544312122	0,462665304	0,62594496	0,43694496	**-0,82305504**
chrXIV	784111	784233	122	0,336248149	0,081529131	0,804850252	0,859074354	0,730213201	1,0816173	1,2356173	**-0,0243827**
chrXIV	784111	784286	175	0,131079787	0,059293913	0,688539366	0,491714385	0,417957228	0,721360639	0,504360639	**-0,755639361**
chrXIV	784111	784286	175	0,131079787	0,059293913	0,688539366	0,491714385	0,417957228	0,721360639	0,504360639	**-0,755639361**
chrXIV	784130	784238	108	0,094035499	0,081529131	0,535617563	0,08939893	0,07598909	0,578952915	0,830952915	**-0,429047085**
chrXIV	784130	784274	144	2,188462528	0,478057176	0,820718679	0,918107397	0,780391288	0,648531057	0,648531057	**-0,611468943**
chrXIV	784130	784275	145	0,051292091	0,08894087	0,365763444	-0,34309514	-0,291630869	-0,451014209	-0,458014209	**-1,718014209**
chrXIV	784130	784277	147	0,273557816	0,08894087	0,754644987	0,68917996	0,585802966	0,513154693	0,492154693	**-0,767845307**
chrXIV	784130	784329	199	0,29350474	0,211234566	0,581497689	0,205726418	0,174867455	0,593221173	0,208221173	**-1,051778827**
chrXIV	784132	784275	143	0,954602795	0,214940436	0,816218478	0,901047538	0,765890407	0,582311075	0,589311075	**-0,670688925**
chrXIV	784135	784274	139	0,820673448	0,17788174	0,821860883	0,922480044	0,784108038	0,650652717	0,685652717	**-0,574347283**
chrXIV	784139	784275	136	0,273557816	0,218646305	0,555781238	0,140281573	0,119239337	0,01732953	0,07332953	**-1,18667047**

Chrom	Start	End	Length	Section A	Section B	A/A+B	Z-score	Z * 0.85	Phase correction	Length correction	ΔLKnuc
chrXIV	784139	784296	157	0,111132863	0,037058696	0,74992708	0,674260298	0,573121253	0,513646995	0,422646995	-0,837353005
chrXIV	784140	784275	135	0,028495606	0,092646739	0,235224155	-0,721749813	-0,613487341	-0,621223736	-0,558223736	-1,818223736
chrXIV	784145	784286	141	0,416035845	0,118587827	0,778184482	0,766076079	0,651164667	0,412330189	0,433330189	-0,826669811
chrXIV	784164	784275	111	0,056991212	0,081529131	0,411428464	-0,223871969	-0,190291174	0,225639615	0,456639615	-0,803360385
chrXIV	784169	784274	105	0,279256937	0,285351958	0,49460244	-0,013530088	-0,011500575	0,490042985	0,763042985	-0,496957015
chrXIV	784169	784275	106	0,037044288	0,259410871	0,124957474	-1,150555991	-0,977972592	-0,458247251	-0,192247251	-1,452247251
chrXIV	784169	784277	108	0,034194727	0,040764565	0,456177292	-0,110069087	-0,093558724	0,365206854	0,617206854	-0,642793146
chrXIV	784172	784265	93	0,045592969	0,048176305	0,486225044	-0,034535557	-0,029355224	0,333969233	0,690969233	-0,569030767
chrXIV	784172	784267	95	0,230814407	0,122293696	0,653664997	0,395234266	0,335949126	0,732399665	1,075399665	-0,184600335
chrXIV	784172	784271	99	0,646850252	0,466939567	0,580765097	0,203851168	0,173273493	0,600792914	0,915792914	-0,344207086
chrXIV	784172	784274	102	0,028495606	0,059293913	0,324590066	-0,454901459	-0,386666241	0,03125577	0,32525577	-0,93474423
chrXIV	784172	784275	103	1,795223168	2,764578706	0,393706397	-0,269671752	-0,229220989	0,20248926	0,48948926	-0,77051074
chrXIV	784172	784277	105	0,222265726	0,137117174	0,618464945	0,301451656	0,256233907	0,781715484	1,054715484	-0,205284516
chrXIV	784177	784271	94	0,054141651	0,133411305	0,288673942	-0,557262806	-0,473673385	-0,084111089	0,265888911	-0,994111089
chrXIV	784177	784275	98	0,273557816	2,056757616	0,117390896	-1,188131028	-1,009911374	-0,58051675	-0,25851675	-1,51851675
chrXV	838	992	154	1,51311667	1,452700875	0,510185353	0,025533667	0,021703617	-0,050324046	-0,120324046	-1,380324046
chrXV	6743	6894	151	1,119877309	0,70040935	0,615220302	0,292951275	0,249008584	0,134861164	0,085861164	-1,174138836
chrXV	8892	9048	156	0,039893848	0,055588044	0,417815854	-0,20748423	-0,176361595	-0,154450814	-0,238450814	-1,498450814
chrXV	10875	10982	107	0,165274514	1,297054353	0,11302144	-1,210615295	-1,029023001	-0,04672108	0,21227892	-1,04772108
chrXV	23820	23972	152	0,387540239	0,26311674	0,595613744	0,242009873	0,205708392	0,137145589	0,081145589	-1,178854411
chrXV	29238	29397	159	1,302249187	0,770820872	0,628174229	0,327021609	0,277968368	0,31276023	0,20776023	-1,05223977
chrXV	29238	29397	159	1,302249187	0,770820872	0,628174229	0,327021609	0,277968368	0,31276023	0,20776023	-1,05223977
chrXV	36163	36311	148	0,09973462	0,111176087	0,472876041	-0,06804215	-0,057835827	-0,060033363	-0,088033363	-1,348033363
chrXV	39246	39406	160	0,302053422	0,255705001	0,541548831	0,104336469	0,088685998	0,138516678	0,026516678	-1,233483322
chrXV	43767	43929	162	0,484425299	0,807879568	0,37485373	-0,319025127	-0,271171358	-0,221068033	-0,347068033	-1,607068033
chrXV	44442	44591	149	1,917754273	1,082113917	0,639279512	0,356533624	0,303053581	0,272392175	0,237392175	-1,022607825
chrXV	49181	49338	157	1,70688679	1,163643048	0,594624298	0,239456783	0,203538266	0,244524259	0,153524259	-1,106475741
chrXV	51344	51469	125	0,085486818	0,144528914	0,371656395	-0,327469522	-0,278349094	0,45809714	0,59109714	-0,66890286
chrXV	53593	53699	106	0,324849907	2,338403704	0,12197483	-1,165171301	-0,990395606	0,000651019	0,266651019	-0,993348981
chrXV	53593	53708	115	0,339097709	2,946166315	0,103217795	-1,263427488	-1,073913365	-0,090864622	0,112135378	-1,147864622
chrXV	53860	54011	151	0,222265726	0,26311674	0,457918737	-0,105678457	-0,089826688	-0,194230881	-0,243230881	-1,503230881
chrXV	53996	54153	157	0,763682236	0,663350655	0,535153914	0,08823214	0,074997319	0,115494397	0,024494397	-1,235505603
chrXV	63125	63255	130	0,14817715	0,096352609	0,605967759	0,268824834	0,228501109	0,541492418	0,639492418	-0,620507582
chrXV	64118	64281	163	0,678195419	0,948702612	0,416864122	-0,209922394	-0,178434035	-0,104302814	-0,237302814	-1,497302814
chrXV	66159	66329	170	0,039893848	0,111176087	0,264075364	-0,630831465	-0,536206745	-0,399108517	-0,581108517	-1,841108517
chrXV	72755	72904	149	0,378991558	0,318704784	0,543204164	0,108509339	0,092232938	0,055610787	0,020610787	-1,239389213
chrXV	74613	74750	137	0,700991904	0,311293045	0,692484764	0,502905845	0,427469968	0,591494655	0,640494655	-0,619505345
chrXV	74660	74806	146	1,473222822	0,96352609	0,604585402	0,265234012	0,22544891	0,248387375	0,234387375	-1,025612625
chrXV	74660	74806	146	1,473222822	0,96352609	0,604585402	0,265234012	0,22544891	0,248387375	0,234387375	-1,025612625
chrXV	75646	75807	161	0,108283302	0,196411088	0,355383315	-0,370826679	-0,315202677	-0,257061107	-0,376061107	-1,636061107
chrXV	80807	80978	171	0,447381012	0,667056524	0,401441083	-0,249618789	-0,212175971	-0,061279878	-0,250279878	-1,510279878
chrXV	101110	101258	148	0,549965193	0,418763262	0,567718631	0,170568921	0,144983583	0,141626669	0,113626669	-1,146373331
chrXV	108670	108830	160	0,404637603	0,518821741	0,438175872	-0,155595664	-0,132256314	-0,084753686	-0,196753686	-1,456753686
chrXV	110950	111091	141	0,481575739	0,385410436	0,555459536	0,139467258	0,118547169	0,197202826	0,218202826	-1,041797174
chrXV	112040	112197	157	0,04844253	0,092646739	0,343346664	-0,403346515	-0,342844538	-0,312471504	-0,403471504	-1,663471504
chrXV	113588	113739	151	0,116831984	0,044470435	0,72430398	0,595675482	0,506324159	0,407030525	0,358030525	-0,901969475
chrXV	116654	116808	154	0,028495606	0,040764565	0,411428464	-0,223871969	-0,190291174	-0,25598372	-0,32598372	-1,58598372
chrXV	118023	118177	154	0,484425299	0,370586958	0,566571175	0,16765125	0,142503563	0,07917029	0,00917029	-1,25082971
chrXV	118066	118215	149	0,085486818	0,055588044	0,605967759	0,268824834	0,228501109	0,189276554	0,154276554	-1,105723446
chrXV	118240	118363	123	0,09688506	0,277940218	0,258480592	-0,648036746	-0,550831234	0,196730019	0,343730019	-0,916269981
chrXV	118340	118495	155	0,116831984	0,066705652	0,636556002	0,349268164	0,296877939	0,281221215	0,204221215	-1,055778785
chrXV	118969	119120	151	0,119681545	0,122293696	0,49460244	-0,013530088	-0,011500575	-0,105032477	-0,154032477	-1,414032477
chrXV	118979	119124	145	0,111132863	0,092646739	0,54535813	0,113942039	0,096850733	0,034332884	0,027332884	-1,232667116
chrXV	118997	119148	151	0,056991212	0,037058696	0,605967759	0,268824834	0,228501109	0,134961158	0,085961158	-1,174038842
chrXV	119251	119405	154	0,259310013	0,17417587	0,598197134	0,248683347	0,211380845	0,155347147	0,085347147	-1,174652853
chrXV	119285	119429	144	0,062690333	0,048176305	0,56545715	0,164819952	0,140096959	0,107803736	0,107803736	-1,152196264
chrXV	119816	119951	135	0,065539893	0,074117392	0,469290903	-0,077052467	-0,065494597	0,234993914	0,297993914	-0,962006086
chrXV	120263	120409	146	0,071239015	0,051882174	0,578608892	0,198335964	0,168585569	0,192429279	0,178429279	-1,081570721
chrXV	120263	120413	150	8,708257143	4,120926971	0,678784954	0,464303812	0,394658241	0,30160023	0,25960023	-1,00039977
chrXV	120442	120599	157	0,062690333	0,055588044	0,530023616	0,075329227	0,064029843	0,091757309	0,000757309	-1,259242691
chrXV	120674	120823	149	0,108283302	0,077823261	0,581834946	0,206589951	0,175601459	0,129503135	0,094503135	-1,165496865
chrXV	120933	121062	129	0,056991212	0,103764348	0,354520937	-0,373143192	-0,317171714	0,136287842	0,241287842	-1,018712158
chrXV	121845	121995	150	0,142478029	0,185293479	0,434687048	-0,164453569	-0,139785534	-0,226664224	-0,268664224	-1,528664224
chrXV	122824	122966	142	0,042743409	0,044470435	0,490098899	-0,024820929	-0,021097789	0,030769416	0,044769416	-1,215230584
chrXV	132108	132259	151	0,102584181	0,159352392	0,391637486	-0,275053712	-0,233795655	-0,327024249	-0,376024249	-1,636024249
chrXV	132333	132482	149	0,122531105	0,133411305	0,478744828	-0,053304046	-0,045308439	-0,08000934	-0,11500934	-1,37500934
chrXV	138290	138437	147	0,256460453	0,237175653	0,519533417	0,048982595	0,041635206	0,110909607	0,089909607	-1,170090393
chrXV	141232	141379	147	0,370442876	0,333528262	0,52621884	0,065768269	0,055903029	0,128118531	0,107118531	-1,152881469
chrXV	142140	142290	150	0,062690333	0,037058696	0,628480635	0,327831948	0,278657156	0,19267385	0,15067385	-1,10932615
chrXV	143684	143828	144	3,168711369	2,393991748	0,569635176	0,175445428	0,149128613	0,12123166	0,12123166	-1,13876834
chrXV	147632	147784	152	0,094035499	0,040764565	0,69759239	0,517488464	0,439865194	0,367015728	0,311015728	-0,948984272
chrXV	152975	153131	156	0,29065518	0,277940218	0,511181027	0,028030349	0,023825797	0,048738125	-0,035261875	-1,295261875
chrXV	160615	160744	129	0,04844253	0,048176305	0,50137771	0,003453413	0,002935401	0,427041169	0,532041169	-0,727958831
chrXV	162501	162638	137	0,304902982	0,166764131	0,646436806	0,375718225	0,319360491	0,471037129	0,520037129	-0,739962871
chrXV	163008	163166	158	0,840620372	0,629997828	0,571610206	0,180475116	0,153403848	0,200525949	0,102525949	-1,157474051
chrXV	163425	163564	139	0,692443222	0,459527828	0,601094291	0,256180562	0,217753478	0,280453925	0,315453925	-0,944546075
chrXV	164146	164288	142	1,111328628	0,830114786	0,572423909	0,182548645	0,155166349	0,18415272	0,19815272	-1,06184728
chrXV	169600	169748	148	0,444531451	0,515115871	0,46322377	-0,09231529	-0,078467996	-0,081866628	-0,109866628	-1,369866628
chrXV	171569	171743	174	0,435982769	0,692997611	0,386173911	-0,289305214	-0,245909432	0,022479845	-0,187520155	-1,447520155
chrXV	173781	173933	152	1,168319839	0,874585221	0,571891402	0,18119159	0,154012851	0,072877569	0,016877569	-1,243122431
chrXV	177092	177238	146	0,065539893	0,385410436	0,145337278	-1,056642983	-0,898146535	-0,862246911	-0,876246911	-2,136246911
chrXV	177093	177211	118	0,042743409	0,17788174	0,1937377	-0,864204794	-0,734574075	0,244355211	0,426355211	-0,833644789
chrXV	177093	177214	121	0,045592969	0,125999566	0,265704853	-0,625855528	-0,531977199	0,279754957	0,440754957	-0,819245043
chrXV	177093	177217	124	0,131079787	1,899993485	0,064856524	-1,515234426	-1,287949263	-0,581848134	-0,441848134	-1,701848134
chrXV	177093	177220	127	0,076938136	0,051882174	0,597251597	0,246239543	0,209303612	0,753197378	0,872197378	-0,387802622
chrXV	177093	177221	128	0,111132863	0,144528914	0,434687048	-0,164453569	-0,139785534	0,401544342	0,513544342	-0,746455658
chrXV	177093	177222	129	0,105433742	0,08894087	0,542425478	0,106546147	0,090564225	0,5092585	0,6142585	-0,6457415
chrXV	177093	177223	130	0,168124074	0,092646739	0,644719675	0,371103224	0,315437741	0,620739855	0,718739855	-0,541260145
chrXV	177093	177224	131	0,14817715	0,129705435	0,533236547	0,083408278	0,070897036	0,301815019	0,392815019	-0,867184981
chrXV	177093	177225	132	0,284956058	0,222352175	0,561702018	0,155285859	0,13199298	0,426511838	0,510511838	-0,749488162
chrXV	177093	177226	133	0,09973462	0,274234349	0,266692236	-0,62284791	-0,529420724	-0,247856435	-0,170856435	-1,430856435
chrXV	177093	177227	134	0,202318801	0,148234783	0,577140872	0,194584477	0,165396805	0,477938977	0,547938977	-0,712061023
chrXV	177093	177228	135	0,202318801	0,148234783	0,577140872	0,194584477	0,165396805	0,453877291	0,516877291	-0,743122709
chrXV	177093	177229	136	0,176672756	0,170470001	0,508934013	0,022396121	0,019036703	0,245519253	0,301519253	-0,958480747
chrXV	177093	177230	137	0,208017923	0,240881523	0,463395366	-0,091883336	-0,078100835	0,052783278	0,101783278	-1,158216722
chrXV	177093	177231	138	0,210867483	0,222352175	0,486744956	-0,033231584	-0,028246847	0,117737343	0,159737343	-1,100262657

Chrom	Start	End	Length	Section A	Section B	A/A+B	Z-score	Z * 0.85	Phase correction	Length correction	ΔLKnuc
chrXV	177093	177232	139	0,119681545	0,096352609	0,553993628	0,135757811	0,11539414	0,196254366	0,231254366	**-1,028745634**
chrXV	177093	177233	140	0,250761331	0,285351958	0,467739443	-0,080953558	-0,068810524	0,023305727	0,051305727	**-1,208694273**
chrXV	177093	177234	141	0,250761331	0,222352175	0,530023616	0,075329227	0,064029843	0,131025469	0,152025469	**-1,107974531**
chrXV	177093	177235	142	0,772230918	0,403939784	0,656563641	0,40310264	0,342637244	0,350797087	0,364797087	**-0,895202913**
chrXV	177093	177236	143	0,259310013	0,163058261	0,613942923	0,289610604	0,246169013	0,285196127	0,292196127	**-0,967803873**
chrXV	177093	177237	144	0,322000346	0,296469566	0,520640276	0,051760603	0,043996513	0,016616398	0,016616398	**-1,243383602**
chrXV	177093	177238	145	249,0544446	103,5271725	0,706373879	0,542822178	0,461398851	0,436149214	0,429149214	**-0,830850786**
chrXV	177093	177239	146	0,091185939	0,70411522	0,11465586	-1,202133727	-1,021813668	-0,961173337	-0,975173337	**-2,235173337**
chrXV	177094	177238	144	0,279256937	0,296469566	0,485051384	-0,037479397	-0,031857487	-0,070164434	-0,070164434	**-1,330164434**
chrXV	177095	177238	143	0,370442876	0,311293045	0,543381777	0,108957189	0,092613611	0,13029459	0,13729459	**-1,12270541**
chrXV	177096	177238	142	1,040089613	0,61146848	0,629762657	0,331224804	0,281541083	0,28447824	0,29847824	**-0,96152176**
chrXV	177097	177238	141	0,361894194	0,237175653	0,604093489	0,26395704	0,224363484	0,298607328	0,319607328	**-0,940392672**
chrXV	177098	177238	140	0,58700948	0,426175002	0,579370777	0,200284037	0,170241431	0,264496599	0,292496599	**-0,967503401**
chrXV	177099	177238	139	0,071239015	0,188999349	0,273745245	-0,601524812	-0,51129609	-0,41743343	-0,38243343	**-1,64243343**
chrXV	177100	177238	138	0,091185939	0,107470218	0,459013908	-0,102918298	-0,087480553	0,024360604	0,066360604	**-1,193639396**
chrXV	177101	177238	137	0,071239015	0,185293479	0,277699771	-0,589688448	-0,501235181	-0,343361252	-0,294361252	**-1,554361252**
chrXV	177102	177238	136	0,088336378	0,240881523	0,268321917	-0,617896095	-0,525211681	-0,289321503	-0,233321503	**-1,493321503**
chrXV	177103	177238	135	0,091185939	0,281646088	0,244576464	-0,691656728	-0,587908219	-0,290576382	-0,227576382	**-1,487576382**
chrXV	177104	177238	134	0,068389454	0,326116523	0,173354671	-0,940991251	-0,799842563	-0,503158055	-0,433158055	**-1,693158055**
chrXV	177105	177238	133	0,034194727	0,222352175	0,133288404	-1,11098035	-0,944333298	-0,658043842	-0,581043842	**-1,841043842**
chrXV	177106	177238	132	0,062690333	0,074117392	0,458236793	-0,104876779	-0,089145262	0,188031908	0,272031908	**-0,987968092**
chrXV	177107	177238	131	0,059840772	0,166764131	0,264075364	-0,630831465	-0,536206745	-0,304584125	-0,213584125	**-1,473584125**
chrXV	177109	177238	129	0,094035499	0,326116523	0,223813035	-0,759378669	-0,645471868	-0,24563726	-0,14063726	**-1,40063726**
chrXV	177110	177238	128	0,09688506	0,340940001	0,22128715	-0,767853376	-0,65267537	-0,138237269	-0,026237269	**-1,286237269**
chrXV	177111	177238	127	0,037044288	0,085235	0,302948179	-0,51593994	-0,438548949	0,094569508	0,213569508	**-1,046430492**
chrXV	177112	177238	126	0,051292091	0,233469783	0,180122745	-0,914897409	-0,777662797	-0,17149581	-0,04549581	**-1,30549581**
chrXV	177113	177238	125	0,054141651	0,048176305	0,529151025	0,07313593	0,062165541	0,779487131	0,912487131	**-0,347512869**
chrXV	177115	177238	123	0,031345166	0,118587827	0,209061167	-0,8096831	-0,688230635	0,0474975	0,1944975	**-1,0655025**
chrXV	177117	177238	121	0,028495606	0,062999783	0,311443082	-0,491764035	-0,41799943	0,376491442	0,537491442	**-0,722508558**
chrXV	177118	177238	120	0,054141651	0,048176305	0,529151025	0,07313593	0,062165541	0,961030763	1,129030763	**-0,130969237**
chrXV	177119	177238	119	0,054141651	0,085235	0,388455674	-0,283346352	-0,2408444	0,692666629	0,867666629	**-0,392333371**
chrXV	177120	177238	118	0,045592969	0,103764348	0,305261035	-0,509328378	-0,432929121	0,534930759	0,716930759	**-0,543069241**
chrXV	177121	177238	117	0,039893848	0,114881957	0,257752484	-0,65028992	-0,552746432	0,40924629	0,59824629	**-0,66175371**
chrXV	177124	177238	114	0,085486818	0,185293479	0,315705458	-0,47974192	-0,407780632	0,583965289	0,793965289	**-0,466034711**
chrXV	177125	177238	113	0,054141651	0,203822827	0,209880258	-0,806836798	-0,685811279	0,322763491	0,539763491	**-0,720236509**
chrXV	177128	177238	110	0,039893848	0,163058261	0,196567792	-0,853944782	-0,725853065	0,273640298	0,511640298	**-0,748359702**
chrXV	177130	177238	108	0,028495606	0,059293913	0,324590066	-0,454901459	-0,386666241	0,56013891	0,81213891	**-0,44786109**
chrXV	177132	177217	85	0,125380666	0,163058261	0,434687048	-0,164453569	-0,139785534	0,451462324	0,864462324	**-0,395537676**
chrXV	177132	177238	106	0,031345166	0,570703915	0,052064138	-1,625160916	-1,381386779	-0,375321852	-0,109321852	**-1,369321852**
chrXV	177733	177893	160	0,3903898	0,359469349	0,520617506	0,05170345	0,043947932	0,093220704	-0,018779296	**-1,278779296**
chrXV	178523	178704	181	0,068389454	0,163058261	0,295485544	-0,537429332	-0,456814932	-0,0832499	-0,3422499	**-1,6022499**
chrXV	185749	185889	140	0,199469241	0,226058044	0,468757816	-0,078392761	-0,066633846	-0,004430251	0,023569749	**-1,236430251**
chrXV	187300	187456	156	0,316301225	0,363175219	0,465507271	-0,086568454	-0,073583186	-0,043095032	-0,127095032	**-1,387095032**
chrXV	189704	189862	158	0,056991212	0,070411522	0,447331152	-0,132407093	-0,112546029	-0,076006571	-0,174006571	**-1,434006571**
chrXV	192332	192491	159	0,162424953	0,129705435	0,556001565	0,140839333	0,119713433	0,15996104	0,05496104	**-1,20503896**
chrXV	196562	196707	145	0,09688506	0,096352609	0,50137771	0,003453413	0,002935401	-0,020089093	-0,027089093	**-1,287089093**
chrXV	196562	196707	145	0,09688506	0,096352609	0,50137771	0,003453413	0,002935401	-0,020089093	-0,027089093	**-1,287089093**
chrXV	198924	199072	148	0,983098401	0,548468698	0,64189052	0,363516673	0,308989172	0,284769191	0,256769191	**-1,003230809**
chrXV	202849	203002	153	0,065539893	0,066705652	0,495592445	-0,011048326	-0,009391077	-0,089861553	-0,152861553	**-1,412861553**
chrXV	205781	205911	130	0,621204207	0,767115003	0,447450559	-0,132105155	-0,112289381	0,18829822	0,28629822	**-0,97370178**
chrXV	208753	208900	147	0,621204207	0,455821958	0,576777266	0,193655717	0,16460736	0,243672574	0,222672574	**-1,037327426**
chrXV	209390	209535	145	0,056991212	0,051882174	0,523463207	0,058847485	0,050020363	0,045755242	0,038755242	**-1,221244758**
chrXV	212639	212808	169	0,176672756	0,26682261	0,398364379	-0,25758299	-0,218945542	-0,075529614	-0,250529614	**-1,510529614**
chrXV	214778	214916	138	2,054533181	1,045055221	0,662840647	0,420228267	0,357194027	0,493612706	0,535612706	**-0,724387294**
chrXV	215741	215894	153	0,547115632	0,500292393	0,522351957	0,056057394	0,047648785	-0,030123569	-0,093123569	**-1,353123569**
chrXV	218336	218507	171	0,065539893	0,081529131	0,445640364	-0,13668381	-0,116181238	0,043004388	-0,145995612	**-1,405995612**
chrXV	220428	220557	129	2,627294858	5,080747192	0,340851132	-0,410141347	-0,348620145	0,029893116	0,134893116	**-1,125106884**
chrXV	222406	222534	128	0,188070999	0,522527611	0,264665588	-0,629027313	-0,534673216	-0,039436268	0,072563732	**-1,187436268**
chrXV	225244	225389	145	10,69155131	6,285154805	0,629777722	0,331264697	0,281574992	0,279444375	0,272444375	**-0,987555625**
chrXV	227790	227919	129	0,082637257	0,214940436	0,277699771	-0,589688448	-0,501235181	-0,115952598	-0,010952598	**-1,270952598**
chrXV	229356	229535	179	0,042743409	0,077823261	0,354520937	-0,373143192	-0,317171714	0,042016297	-0,202983703	**-1,462983703**
chrXV	229736	229903	167	0,062690333	0,137117174	0,313753641	-0,48523835	-0,412452597	-0,264697167	-0,425697167	**-1,685697167**
chrXV	233701	233860	159	0,173823196	0,129705435	0,572674792	0,18318812	0,155709902	0,200548692	0,095548692	**-1,164451308**
chrXV	233701	233860	159	0,173823196	0,129705435	0,572674792	0,18318812	0,155709902	0,200548692	0,095548692	**-1,164451308**
chrXV	237293	237436	143	0,133929347	0,155646522	0,462501753	-0,094133	-0,08001305	-0,039865768	-0,032865768	**-1,292865768**
chrXV	242082	242240	158	0,125380666	0,140823044	0,470995186	-0,072768458	-0,061853189	-0,032671127	-0,130671127	**-1,390671127**
chrXV	242082	242240	158	0,125380666	0,140823044	0,470995186	-0,072768458	-0,061853189	-0,032671127	-0,130671127	**-1,390671127**
chrXV	243486	243636	150	0,510071344	0,248293262	0,672593816	0,44708683	0,380023805	0,29006655	0,24806655	**-1,01193345**
chrXV	244606	244758	152	0,108283302	0,137117174	0,441251393	-0,147797242	-0,125627655	-0,219663473	-0,275663473	**-1,535663473**
chrXV	244606	244758	152	0,108283302	0,137117174	0,441251393	-0,147797242	-0,125627655	-0,219663473	-0,275663473	**-1,535663473**
chrXV	248190	248334	144	0,116831984	0,166764131	0,411966095	-0,222490344	-0,189116792	-0,245492756	-0,245492756	**-1,505492756**
chrXV	248556	248722	166	0,883363781	1,263701526	0,411428464	-0,223871969	-0,190291174	-0,061566659	-0,215566659	**-1,475566659**
chrXV	255239	255421	182	0,085486818	0,100058479	0,460732874	-0,09858756	-0,083799426	0,31879579	0,05279579	**-1,20720421**
chrXV	256726	256872	146	1,336443914	1,196995874	0,527521483	0,069040937	0,058684797	0,123412242	0,109412242	**-1,150587758**
chrXV	260668	260810	142	0,059840772	0,092646739	0,392430643	-0,272989502	-0,232041076	-0,228576099	-0,214576099	**-1,474576099**
chrXV	261423	261595	172	0,53856695	0,796761959	0,403321568	-0,244758877	-0,208045045	0,020890716	-0,175109284	**-1,435109284**
chrXV	263580	263731	151	0,14817715	0,048176305	0,754644987	0,68917996	0,585802966	0,488547783	0,439547783	**-0,820452217**
chrXV	265679	265840	161	0,151026711	0,159352392	0,486587884	-0,033625524	-0,028581696	0,040096818	-0,078903182	**-1,338903182**
chrXV	266859	267005	146	0,039893848	0,066705652	0,374240479	-0,320642986	-0,272546538	-0,196278943	-0,210278943	**-1,470278943**
chrXV	268238	268379	141	0,661098055	0,533645219	0,553339005	0,13410191	0,113986624	0,1830404	0,2040404	**-1,0559596**
chrXV	270968	271134	166	0,296354301	0,233469783	0,559344714	0,149308039	0,126911833	0,253677468	0,099677468	**-1,160322532**
chrXV	271150	271308	158	0,079787696	0,085235	0,483495289	-0,041382985	-0,035175537	0,008695488	-0,089304512	**-1,349304512**
chrXV	273050	273212	162	0,076938136	0,118587827	0,393493196	-0,270226	-0,2296921	-0,16451261	-0,29051261	**-1,55051261**
chrXV	276074	276211	137	0,869115978	0,385410436	0,69278412	0,503757552	0,428193919	0,606255311	0,655255311	**-0,604744689**
chrXV	276074	276211	137	0,869115978	0,385410436	0,69278412	0,503757552	0,428193919	0,606255311	0,655255311	**-0,604744689**
chrXV	276839	276983	144	0,051292091	0,062999783	0,448781606	-0,128740216	-0,109429183	-0,159903964	-0,159903964	**-1,419903964**
chrXV	279591	279744	153	0,322000346	0,311293045	0,508453666	0,021191784	0,018013016	-0,0644379	-0,1274379	**-1,3874379**
chrXV	280089	280217	128	0,492973981	0,622586089	0,441907159	-0,146135633	-0,124215288	0,358790379	0,470790379	**-0,789209621**
chrXV	282501	282650	149	0,678195419	0,374292827	0,64437339	0,3701735	0,314647475	0,278839225	0,243839225	**-1,016160775**
chrXV	284484	284582	98	0,555664314	6,737270894	0,076192136	-1,431160321	-1,216486272	-0,304739685	0,017260315	**-1,242739685**
chrXV	284650	284789	139	0,190920559	0,122293696	0,609552586	0,278153109	0,236430142	0,338870367	0,373870367	**-0,886129633**
chrXV	284999	285154	155	0,165274514	0,100058479	0,622894697	0,313092211	0,266128379	0,26726287	0,19026287	**-1,06973713**
chrXV	285195	285326	131	1,544461836	1,704700006	0,475341615	-0,061848815	-0,052571493	0,189635695	0,280635695	**-0,979364305**
chrXV	292850	293005	155	0,63545201	0,489174784	0,565033674	0,163744034	0,139182429	0,137803273	0,060803273	**-1,199196727**
chrXV	292905	293050	145	0,299203861	0,218646305	0,577780757	0,196219347	0,166786445	0,203319709	0,196319709	**-1,063680291**
chrXV	293213	293372	159	0,159575393	0,155646522	0,506231912	0,015621722	0,013278464	0,062555593	-0,042444407	**-1,302444407**

Chrom	Start	End	Length	Section A	Section B	A/A+B	Z-score	Z * 0.85	Phase correction	Length correction	ΔLKnuc
chrXV	294193	294362	169	0,202318801	0,181587609	0,52700032	0,067731518	0,05757179	0,193645257	0,018645257	**-1,241354743**
chrXV	298993	299142	149	0,393239361	0,300175436	0,567105523	0,169009772	0,143658307	0,100227885	0,065227885	**-1,194772115**
chrXV	306450	306602	152	0,225115286	0,192705218	0,538784679	0,097372425	0,082766561	-0,01699903	-0,07299903	**-1,33299903**
chrXV	311787	311943	156	0,598407723	0,692997611	0,46337715	-0,09192919	-0,078139812	-0,046667975	-0,130667975	**-1,390667975**
chrXV	319552	319692	140	3,499260397	1,84922892	0,654252106	0,396825983	0,337302085	0,3905154	0,4185154	**-0,8414846**
chrXV	320812	320963	151	0,14532759	0,077823261	0,65125268	0,388704654	0,330398956	0,242395884	0,193395884	**-1,066604116**
chrXV	321565	321713	148	0,111132863	0,092646739	0,54535813	0,113942039	0,096850733	0,060787195	0,032787195	**-1,227212805**
chrXV	327427	327560	133	1,396284686	1,389701092	0,501181556	0,002961725	0,002517466	0,268839498	0,345839498	**-0,914160502**
chrXV	328438	328583	145	1,290850944	0,78935022	0,620541401	0,306903009	0,260867558	0,294486284	0,287486284	**-0,972513716**
chrXV	328438	328583	145	1,290850944	0,78935022	0,620541401	0,306903009	0,260867558	0,294486284	0,287486284	**-0,972513716**
chrXV	329812	329980	168	0,074088575	0,066705652	0,52621884	0,065768269	0,055903029	0,222895876	0,054895876	**-1,205104124**
chrXV	338001	338161	160	0,549965193	0,589233263	0,482765044	-0,043215075	-0,036732814	0,020786649	-0,091213351	**-1,351213351**
chrXV	341700	341803	103	0,398938482	5,651451107	0,065935999	-1,506760733	-1,280746623	-0,298077672	-0,011077672	**-1,271077672**
chrXV	348865	348972	107	0,68389454	0,696703481	0,49536109	-0,011628286	-0,009884043	0,960124375	1,219124375	**-0,040875625**
chrXV	349258	349411	153	0,287805619	0,229763914	0,556071408	0,14101615	0,119863728	0,03726466	-0,02573534	**-1,28573534**
chrXV	351942	352092	150	0,284956058	0,140823044	0,669257972	0,437865126	0,372185358	0,27339093	0,23139093	**-1,02860907**
chrXV	353769	353922	153	0,028495606	0,040764565	0,411428464	-0,223871969	-0,190291174	-0,287468578	-0,350468578	**-1,610468578**
chrXV	356685	356815	130	2,746976403	2,664520227	0,507618611	0,019098187	0,016233459	0,302677861	0,400677861	**-0,859322139**
chrXV	357881	358029	148	1,114178188	0,941290873	0,542055441	0,105613368	0,089771362	0,06041723	0,03241723	**-1,22758277**
chrXV	359149	359277	128	0,407487163	4,076456536	0,090876958	-1,335374165	-1,13506804	-0,638539428	-0,526539428	**-1,786539428**
chrXV	359963	360110	147	0,031345166	0,074117392	0,297216064	-0,532424343	-0,452560692	-0,371893437	-0,392893437	**-1,652893437**
chrXV	363241	363404	163	0,125380666	0,129705435	0,491522922	-0,021250483	-0,01806291	0,052851659	-0,080148341	**-1,340148341**
chrXV	363647	363809	162	0,179522317	0,188999349	0,487141826	-0,032236246	-0,027400809	0,036861631	-0,089138369	**-1,349138369**
chrXV	365392	365542	150	1,401983807	1,019114134	0,579069431	0,199513431	0,169586416	0,063091774	0,021091774	**-1,238908226**
chrXV	365392	365542	150	1,401983807	1,019114134	0,579069431	0,199513431	0,169586416	0,063091774	0,021091774	**-1,238908226**
chrXV	369266	369425	159	0,287805619	0,214940436	0,572467186	0,182658948	0,155260106	0,208237371	0,103237371	**-1,156762629**
chrXV	369347	369504	157	0,039893848	0,077823261	0,338895921	-0,415478239	-0,353156503	-0,321975616	-0,412975616	**-1,672975616**
chrXV	371267	371398	131	0,068389454	0,040764565	0,626540868	0,322705529	0,274299699	0,48437371	0,57537371	**-0,68462629**
chrXV	371602	371749	147	0,418885406	0,229763914	0,645780999	0,373954724	0,317861515	0,424600295	0,403600295	**-0,856399705**
chrXV	377796	377936	140	0,404637603	0,274234349	0,596044073	0,243120753	0,20665264	0,288888023	0,316888023	**-0,943111977**
chrXV	384219	384368	149	0,983098401	0,830114786	0,542185777	0,105941906	0,09005062	0,050237089	0,015237089	**-1,244762911**
chrXV	387959	388110	151	0,034194727	0,055588044	0,380860679	-0,303221116	-0,257737948	-0,344310674	-0,393310674	**-1,653310674**
chrXV	389816	389942	126	0,128230226	0,226058044	0,361937543	-0,353284604	-0,300291913	0,316521175	0,442521175	**-0,817478825**
chrXV	392262	392373	111	0,609805965	4,421102407	0,1212119	-1,16894994	-0,993607449	0,013359791	0,244359791	**-1,015640209**
chrXV	392576	392737	161	0,122531105	0,17417587	0,412970086	-0,219911382	-0,186924674	-0,114504576	-0,233504576	**-1,493504576**
chrXV	401254	401398	144	0,122531105	0,151940653	0,446425184	-0,134698361	-0,114493607	-0,156581892	-0,156581892	**-1,416581892**
chrXV	403569	403709	140	0,461628815	0,240881523	0,657113198	0,404597219	0,343907636	0,440630931	0,468630931	**-0,791369069**
chrXV	405315	405460	145	0,250761331	0,170470001	0,595305506	0,241214353	0,2050322	0,240115036	0,233115036	**-1,026884964**
chrXV	405735	405887	152	1,798072728	1,426759788	0,557570888	0,144813371	0,123091365	0,021128945	-0,034871055	**-1,294871055**
chrXV	408309	408462	153	0,433133209	0,289057827	0,599748802	0,252696962	0,214792418	0,110242434	0,047242434	**-1,212757566**
chrXV	413175	413325	150	0,755133555	0,670762394	0,529585315	0,074227492	0,063093368	-0,053282683	-0,095282683	**-1,355282683**
chrXV	413525	413650	125	0,749434433	1,311877831	0,36357152	-0,348928431	-0,296589166	0,396959498	0,529959498	**-0,730040502**
chrXV	419166	419317	151	0,168124074	0,122293696	0,578904225	0,199091015	0,169227363	0,075337828	0,026337828	**-1,233662172**
chrXV	420271	420429	158	0,410336724	0,403939784	0,50392799	0,00984617	0,008369244	0,051837202	-0,046162798	**-1,306162798**
chrXV	423240	423391	151	19,55368472	10,35049373	0,653878011	0,395811655	0,336439906	0,248290539	0,199290539	**-1,060709461**
chrXV	423241	423391	150	0,037044288	0,074117392	0,333246923	-0,430964963	-0,366320218	-0,495723725	-0,537723725	**-1,797723725**
chrXV	423242	423391	149	0,039893848	0,092646739	0,300993446	-0,521545394	-0,443313585	-0,500105226	-0,535105226	**-1,795105226**
chrXV	423243	423391	148	0,059840772	0,059293913	0,502295129	0,005753068	0,004890107	-0,03134863	-0,05934863	**-1,31934863**
chrXV	423245	423391	146	0,065539893	0,066705652	0,495592445	-0,011048326	-0,009391077	0,063539942	0,049539942	**-1,210460058**
chrXV	433044	433202	158	0,173823196	0,155646522	0,52758474	0,069199879	0,058819897	0,095857429	-0,002142571	**-1,262142571**
chrXV	433171	433320	149	0,19377012	0,133411305	0,592240589	0,233312412	0,19831555	0,131699167	0,096699167	**-1,163300833**
chrXV	433171	433320	149	0,19377012	0,133411305	0,592240589	0,233312412	0,19831555	0,131699167	0,096699167	**-1,163300833**
chrXV	435682	435849	167	0,128230226	0,125999566	0,504387095	0,010997038	0,009347483	0,166480869	0,005480869	**-1,254519131**
chrXV	441363	441514	151	0,170973635	0,129705435	0,568624996	0,172874585	0,146943398	0,052939266	0,003939266	**-1,256060734**
chrXV	443421	443578	157	0,04844253	0,051882174	0,482857442	-0,042983253	-0,036535765	-0,007522809	-0,098522809	**-1,358522809**
chrXV	445187	445337	150	0,515770466	0,292763697	0,637908068	0,35287272	0,299941812	0,180660015	0,138660015	**-1,121339985**
chrXV	445187	445337	150	0,515770466	0,292763697	0,637908068	0,35287272	0,299941812	0,180660015	0,138660015	**-1,121339985**
chrXV	446874	447012	138	0,188070999	0,092646739	0,669964783	0,439815924	0,373843536	0,519779987	0,561779987	**-0,698220013**
chrXV	450011	450165	154	0,225115286	0,192705218	0,538784679	0,097372425	0,082766561	0,017678533	-0,052321467	**-1,312321467**
chrXV	456168	456329	161	0,102584181	0,062999783	0,619529685	0,304245806	0,258608935	0,33213935	0,21313935	**-1,04686065**
chrXV	457433	457588	155	8,978965399	6,303684153	0,587526748	0,221187421	0,188009308	0,180384098	0,103384098	**-1,156615902**
chrXV	457539	457691	152	0,903310705	0,655938915	0,579323986	0,200164374	0,170139718	0,069901017	0,013901017	**-1,246098983**
chrXV	459473	459604	131	0,262159574	0,563292176	0,317595273	-0,474433869	-0,403268789	-0,193129338	-0,102129338	**-1,362129338**
chrXV	465612	465776	164	0,809275206	1,059878699	0,432963387	-0,168834559	-0,143509375	-0,06017332	-0,20017332	**-1,46017332**
chrXV	469152	469262	110	0,079787696	0,207528696	0,277699771	-0,589688448	-0,501235181	0,486351268	0,724351268	**-0,535648732**
chrXV	469152	469277	125	0,153876272	0,318704784	0,32560821	-0,452072964	-0,384262019	0,291475955	0,424475955	**-0,835524045**
chrXV	480195	480360	165	5,100713444	5,395746106	0,485946087	-0,035235226	-0,029949942	0,050810446	-0,096189554	**-1,356189554**
chrXV	480209	480363	154	0,396088921	0,244587392	0,618235625	0,30085017	0,255722644	0,197348875	0,127348875	**-1,132651125**
chrXV	480628	480755	127	0,045592969	0,96352609	0,045180961	-1,69349165	-1,439467903	-0,906102363	-0,787102363	**-2,047102363**
chrXV	480628	480787	159	0,760832676	0,915349786	0,453908028	-0,115793684	-0,098424632	-0,043184776	-0,148184776	**-1,408184776**
chrXV	480629	480736	107	0,04844253	1,511994788	0,031044201	-1,865663908	-1,585814322	-0,640834775	-0,381834775	**-1,641834775**
chrXV	480629	480737	108	0,051292091	1,708405876	0,029148235	-1,893461897	-1,609442613	-0,644642803	-0,392642803	**-1,652642803**
chrXV	480629	480738	109	0,111132863	1,541641745	0,06724018	-1,496665334	-1,272165534	-0,258939347	-0,013939347	**-1,273939347**
chrXV	480629	480739	110	0,085486818	1,019114134	0,077391584	-1,422837843	-1,209412166	-0,193774507	0,044225493	**-1,215774507**
chrXV	480629	480741	112	0,059840772	2,927636967	0,020030533	-2,053118702	-1,745150897	-0,751101329	-0,527101329	**-1,787101329**
chrXV	480629	480742	113	0,028495606	3,012871967	0,00936934	-2,350689182	-1,998085805	-0,990901057	-0,773901057	**-2,033901057**
chrXV	480629	480743	114	0,042743409	1,560171093	0,026666057	-1,932221982	-1,642388685	-0,652846269	-0,442846269	**-1,702846269**
chrXV	480629	480744	115	0,037044288	1,471230223	0,024560707	-1,96753632	-1,672405872	-0,707861837	-0,504861837	**-1,764861837**
chrXV	480629	480745	116	0,045592969	0,300175436	0,131859848	-1,117642532	-0,949996152	0,005052415	0,201052415	**-1,058947585**
chrXV	480629	480746	117	0,062690333	0,622586089	0,091481818	-1,331685257	-1,131932469	-0,170355473	0,018644527	**-1,241355473**
chrXV	480629	480747	118	0,039893848	0,444704349	0,082323559	-1,389610724	-1,181169116	-0,219879372	-0,037879372	**-1,297879372**
chrXV	480629	480748	119	0,045592969	1,304466092	0,033771092	-1,828049088	-1,553841725	-0,629200179	-0,454200179	**-1,714200179**
chrXV	480629	480749	120	0,128230226	1,274819135	0,091393952	-1,332220006	-1,132387005	-0,255145544	-0,087145544	**-1,347145544**
chrXV	480629	480750	121	0,082637257	1,574994571	0,0498526	-1,646284493	-1,399341819	-0,627655263	-0,466655263	**-1,726655263**
chrXV	480629	480751	122	0,076938136	0,303881305	0,202033109	-0,834381181	-0,709224004	0,050264393	0,204264393	**-1,055735607**
chrXV	480629	480752	123	0,225115286	1,641700223	0,120587859	-1,172053148	-0,996245176	-0,251898698	-0,104898698	**-1,364898698**
chrXV	480629	480753	124	0,208017923	0,415057393	0,333856787	-0,429288104	-0,364894888	0,338346895	0,478346895	**-0,781653105**
chrXV	480629	480754	125	0,227964847	1,000584786	0,185556074	-0,894392081	-0,760233269	-0,092940328	0,040059672	**-1,219940328**
chrXV	480629	480755	126	0,344796831	19,20011029	0,017641262	-2,105100918	-1,78933578	-1,197802401	-1,071802401	**-2,331802401**
chrXV	480629	480756	127	0,14817715	1,14140783	0,114902975	-1,200858875	-1,020730044	-0,50653947	-0,38753947	**-1,64753947**
chrXV	480629	480757	128	0,188070999	0,359469349	0,34348336	-0,402974858	-0,342528629	0,155629347	0,267629347	**-0,992370653**
chrXV	480629	480758	129	0,287805619	0,778232612	0,269976827	-0,612883077	-0,520950615	-0,148404181	-0,043404181	**-1,303404181**
chrXV	480629	480759	130	0,256460453	0,88199696	0,225270133	-0,754514627	-0,641337433	-0,364067322	-0,266067322	**-1,526067322**
chrXV	480629	480760	131	0,270708255	1,274819135	0,175155909	-0,933984635	-0,79388694	-0,556088555	-0,465088555	**-1,725088555**
chrXV	480629	480761	132	0,532867829	1,019114134	0,343346664	-0,403346515	-0,342844538	-0,084374692	-0,000374692	**-1,260374692**
chrXV	480629	480762	133	0,327699467	1,771405659	0,15611389	-1,010558514	-0,858974737	-0,594442909	-0,517442909	**-1,777442909**

Chrom	Start	End	Length	Section A	Section B	A/A+B	Z-score	Z * 0.85	Phase correction	Length correction	ΔLKnuc
chrXV	480629	480763	134	0,367593315	2,734931749	0,118481981	-1,18260946	-1,005218041	-0,702204097	-0,632204097	**-1,892204097**
chrXV	480629	480764	135	0,227964847	1,76028805	0,11465586	-1,202133727	-1,021813668	-0,741855951	-0,678855951	**-1,938855951**
chrXV	480629	480765	136	0,216566604	1,637994354	0,116775134	-1,19126322	-1,012573737	-0,803405819	-0,747405819	**-2,007405819**
chrXV	480629	480766	137	0,176672756	1,300760222	0,119580894	-1,177084483	-1,000521811	-0,823103766	-0,774103766	**-2,034103766**
chrXV	480629	480767	138	0,44168189	0,863467612	0,338414787	-0,416793341	-0,35427434	-0,215521314	-0,173521314	**-1,433521314**
chrXV	480629	480768	139	0,19377012	1,004290656	0,161736469	-0,987346222	-0,839244289	-0,729292923	-0,694292923	**-1,954292923**
chrXV	480629	480769	140	0,44168189	1,952993268	0,184443343	-0,898560728	-0,763776618	-0,675043766	-0,647043766	**-1,907043766**
chrXV	480629	480770	141	0,29065518	0,870879351	0,250233783	-0,673754249	-0,572691112	-0,489509808	-0,468509808	**-1,728509808**
chrXV	480629	480771	142	0,19377012	2,012287181	0,087835488	-1,354205026	-1,151074272	-1,150375054	-1,136375054	**-2,396375054**
chrXV	480629	480772	143	0,24506221	1,137701961	0,177226323	-0,925987172	-0,787089096	-0,750456916	-0,743456916	**-2,003456916**
chrXV	480629	480773	144	0,267858695	1,070996308	0,200065499	-0,841387298	-0,715179204	-0,753460411	-0,753460411	**-2,013460411**
chrXV	480629	480774	145	0,182371877	0,411351523	0,307166396	-0,503898375	-0,428313619	-0,377022048	-0,384022048	**-1,644022048**
chrXV	480629	480775	146	0,202318801	4,613807625	0,042008615	-1,727838236	-1,4686625	-1,397942306	-1,411942306	**-2,671942306**
chrXV	480629	480776	147	0,324849907	0,359469349	0,474705196	-0,063447213	-0,053930131	0,053409465	0,032409465	**-1,227590535**
chrXV	480629	480777	148	0,527168708	1,126584352	0,318771116	-0,471137942	-0,400467251	-0,438506313	-0,466506313	**-1,726506313**
chrXV	480629	480778	149	0,287805619	0,622586089	0,316133832	-0,478537538	-0,406756908	-0,481913413	-0,516913413	**-1,776913413**
chrXV	480629	480779	150	0,624053768	0,518821741	0,546038272	0,115658174	0,098309448	-0,030352265	-0,072352265	**-1,332352265**
chrXV	480629	480780	151	0,544266071	0,796761959	0,405857341	-0,238214562	-0,202482378	-0,29555997	-0,34455997	**-1,60455997**
chrXV	480629	480781	152	0,755133555	2,212404138	0,254464688	-0,660505676	-0,561429825	-0,655562384	-0,711562384	**-1,971562384**
chrXV	480629	480782	153	2,989189052	4,358102624	0,406842301	-0,235675346	-0,200324044	-0,308022204	-0,371022204	**-1,631022204**
chrXV	480629	480783	154	3,07752543	4,120926971	0,427525982	-0,182676363	-0,155274908	-0,207434115	-0,277434115	**-1,537434115**
chrXV	480629	480784	155	2,923649159	3,757751753	0,437580262	-0,157106997	-0,133540948	-0,133269874	-0,210269874	**-1,470269874**
chrXV	480629	480785	156	1,812320531	4,072750667	0,307952184	-0,501663322	-0,426413824	-0,395375556	-0,479375556	**-1,739375556**
chrXV	480629	480786	157	1,843665698	3,009166098	0,379915434	-0,305702896	-0,259847462	-0,23498926	-0,32598926	**-1,58598926**
chrXV	480629	480787	158	2501,498156	1389,156329	0,642950477	0,366356538	0,311403057	0,352430347	0,254430347	**-1,005569653**
chrXV	480629	480788	159	0,051292091	1,167348917	0,042089582	-1,726935968	-1,467895573	-1,414622115	-1,519622115	**-2,779622115**
chrXV	480629	480789	160	0,726637949	1,159937178	0,385162477	-0,291949869	-0,248157388	-0,181781909	-0,293781909	**-1,553781909**
chrXV	480630	480787	157	3,901048439	4,680513278	0,454584907	-0,114085754	-0,096972891	-0,070461779	-0,161461779	**-1,421461779**
chrXV	480631	480787	156	3,778517334	3,279694577	0,53533634	0,088691209	0,075387528	0,103682384	0,019682384	**-1,240317616**
chrXV	480632	480787	155	2,644392221	4,524866755	0,368851541	-0,334896607	-0,284662116	-0,289346486	-0,366346486	**-1,626346486**
chrXV	480633	480787	154	3,456516988	3,920810014	0,468532436	-0,078959457	-0,067115538	-0,116429767	-0,186429767	**-1,446429767**
chrXV	480634	480787	153	3,413773579	2,204992399	0,607566429	0,272981882	0,2320346	0,128926838	0,065926838	**-1,194073162**
chrXV	480635	480787	152	0,735186631	1,852934789	0,284061878	-0,570816915	-0,485194378	-0,582436973	-0,638436973	**-1,898436973**
chrXV	480636	480787	151	1,057186976	1,967816746	0,349482868	-0,386716991	-0,328709443	-0,430648531	-0,479648531	**-1,739648531**
chrXV	480637	480787	150	1,604302609	2,056757616	0,438207107	-0,155516416	-0,132188953	-0,270703578	-0,312703578	**-1,572703578**
chrXV	480638	480787	149	0,772230918	1,341524787	0,365335936	-0,34423193	-0,292597141	-0,367297273	-0,402297273	**-1,662297273**
chrXV	480639	480787	148	0,940354993	0,96352609	0,493914773	-0,015253994	-0,012965895	-0,052924261	-0,080924261	**-1,340924261**
chrXV	480640	480787	147	0,618354647	1,823287833	0,253253559	-0,664286254	-0,564643316	-0,45581727	-0,47681727	**-1,73681727**
chrXV	480641	480787	146	0,547115632	0,337234132	0,618664305	0,301974647	0,25667845	0,32907	0,31507	**-0,94493**
chrXV	480642	480787	145	0,672496298	1,645406093	0,290131414	-0,553000851	-0,470050723	-0,412102069	-0,419102069	**-1,679102069**
chrXV	480643	480787	144	0,495823542	0,96723196	0,338895921	-0,415478239	-0,353156503	-0,399589497	-0,399589497	**-1,659589497**
chrXV	480644	480787	143	0,644000692	1,834405441	0,259844697	-0,643824268	-0,547250628	-0,513861144	-0,506861144	**-1,766861144**
chrXV	480645	480787	142	0,336248149	0,522527611	0,391543596	-0,275298142	-0,234003421	-0,224075214	-0,210075214	**-1,470075214**
chrXV	480646	480787	141	0,336248149	1,200701743	0,21877626	-0,776332786	-0,659882868	-0,576668024	-0,555668024	**-1,815668024**
chrXV	480647	480787	140	0,188070999	0,314998914	0,373846644	-0,321682433	-0,273430068	-0,196032133	-0,168032133	**-1,428032133**
chrXV	480648	480787	139	0,302053422	1,311877831	0,187153834	-0,888433396	-0,755168386	-0,646642097	-0,611642097	**-1,871642097**
chrXV	480649	480787	138	0,304902982	0,61888022	0,330059024	-0,439750188	-0,373787659	-0,227458207	-0,185458207	**-1,445458207**
chrXV	480650	480787	137	0,159575393	0,496586524	0,243195145	-0,69606154	-0,591652309	-0,421534947	-0,372534947	**-1,632534947**
chrXV	480651	480787	136	0,168124074	2,531108922	0,06228587	-1,535864	-1,3054844	-1,104724396	-1,048724396	**-2,308724396**
chrXV	480652	480787	135	0,162424953	0,107470218	0,601807556	0,258028531	0,219324251	0,499161478	0,562161478	**-0,697838522**
chrXV	480653	480787	134	0,333398588	1,045055221	0,241864171	-0,700318625	-0,595270832	-0,301723761	-0,231723761	**-1,491723761**
chrXV	480654	480787	133	0,128230226	2,056757616	0,058686929	-1,565892193	-1,331008364	-1,046488752	-0,969488752	**-2,229488752**
chrXV	480655	480787	132	0,122531105	1,059878699	0,103628289	-1,261145089	-1,071973325	-0,815583892	-0,731583892	**-1,991583892**
chrXV	480656	480787	131	0,359044634	0,896820438	0,285894275	-0,565419375	-0,480606469	-0,23911072	-0,14811072	**-1,40811072**
chrXV	480657	480787	130	0,179522317	0,937585003	0,160702838	-0,991573388	-0,84283738	-0,571972476	-0,473972476	**-1,733972476**
chrXV	480658	480787	129	0,219416165	3,124048055	0,065625396	-1,509187816	-1,282809644	-0,932844739	-0,827844739	**-2,087844739**
chrXV	480659	480787	128	0,173823196	0,822703046	0,17442912	-0,93680623	-0,796285295	-0,300509558	-0,188509558	**-1,448509558**
chrXV	480660	480787	127	0,133929347	1,523112397	0,080824365	-1,399547947	-1,189615755	-0,652477312	-0,533477312	**-1,793477312**
chrXV	480661	480787	126	0,151026711	1,66764131	0,083042484	-1,384893716	-1,177159658	-0,57978471	-0,45378471	**-1,71378471**
chrXV	480662	480787	125	0,316301225	1,789935006	0,15017367	-1,035688819	-0,880335497	-0,219997903	-0,086997903	**-1,346997903**
chrXV	480663	480787	124	0,247911771	0,578115654	0,300125351	-0,524040025	-0,445434021	0,259717681	0,399717681	**-0,860282319**
chrXV	480664	480787	123	0,492973981	2,253168704	0,179515064	-0,917214731	-0,779632522	-0,044819208	0,102180792	**-1,157819208**
chrXV	480665	480787	122	0,208017923	3,531693708	0,055624054	-1,592608219	-1,353716987	-0,588605681	-0,434605681	**-1,694605681**
chrXV	480666	480787	121	0,19661968	2,935048706	0,062784323	-1,53181276	-1,302040846	-0,51184054	-0,35084054	**-1,61084054**
chrXV	480667	480787	120	0,139628469	1,57870044	0,08125829	-1,396657535	-1,187158905	-0,313153232	-0,145153232	**-1,405153232**
chrXV	480668	480787	119	0,182371877	1,185878265	0,133288404	-1,11098035	-0,944333298	-0,004638303	0,170361697	**-1,089638303**
chrXV	480669	480787	118	0,113982423	1,1451137	0,090527182	-1,337515693	-1,136888339	-0,194321239	-0,012321239	**-1,272321239**
chrXV	480670	480787	117	0,125380666	3,16851849	0,038064513	-1,773601889	-1,507561605	-0,524672565	-0,335672565	**-1,595672565**
chrXV	480671	480787	116	0,151026711	0,804173699	0,158109973	-1,002256045	-0,851917638	0,11279301	0,30879301	**-0,95120699**
chrXV	480672	480787	115	0,133929347	0,17417587	0,434687048	-0,164453569	-0,139785534	0,802545376	1,005545376	**-0,254454624**
chrXV	480673	480787	114	0,111132863	0,985761308	0,101315939	-1,274088996	-1,082975647	-0,103879603	0,106120397	**-1,153879603**
chrXV	480674	480787	113	0,131079787	1,997463703	0,061581916	-1,541628761	-1,310384447	-0,299981955	-0,082981955	**-1,342981955**
chrXV	480675	480787	112	0,185221438	1,882581746	0,089574017	-1,343382859	-1,14187543	-0,13743774	0,08656226	**-1,17343774**
chrXV	480676	480787	111	0,153876272	1,015408265	0,131596654	-1,118866003	-0,951036102	0,048771881	0,279771881	**-0,980228119**
chrXV	480677	480787	110	0,108283302	0,185293479	0,368841506	-0,334923211	-0,284684729	0,728996613	0,966996613	**-0,293003387**
chrXV	480678	480787	109	0,094035499	1,419348049	0,062135933	-1,537087569	-1,306524434	-0,286535234	-0,041535234	**-1,301535234**
chrXV	480679	480787	108	0,122531105	0,604056741	0,168639079	-0,959557122	-0,815623554	0,13240042	0,38440042	**-0,87559958**
chrXV	480680	480755	75	0,589859041	0,52623348	0,528503712	0,071509109	0,060782743		0,483	**-0,777**
chrXV	480680	480787	107	0,176672756	5,699627412	0,030065305	-1,879834699	-1,597859494	-0,6530568	-0,3940568	**-1,6540568**
chrXV	480681	480787	106	0,068389454	0,537351089	0,112902223	-1,211237364	-1,02955176	-0,034788184	0,231211816	**-1,028788184**
chrXV	480682	480787	105	0,102584181	0,811585438	0,112215697	-1,2148288	-1,03260448	-0,076120693	0,196879307	**-1,063120693**
chrXV	480683	480787	104	0,059840772	0,470645436	0,112803634	-1,211752154	-1,029989331	-0,04210638	0,23789362	**-1,02210638**
chrXV	480684	480787	103	0,068389454	0,641115437	0,096390391	-1,302396781	-1,107037264	-0,115385469	0,171614531	**-1,088385469**
chrXV	480685	480787	102	0,054141651	0,926467395	0,05521227	-1,59628786	-1,356844681	-0,34604778	-0,05204778	**-1,31204778**
chrXV	480686	480787	101	0,094035499	2,456991531	0,03686182	-1,788324682	-1,520075979	-0,49302532	-0,19202532	**-1,45202532**
chrXV	480687	480787	100	0,062690333	0,663350655	0,086345446	-1,363608257	-1,159067019	-0,153051229	0,154948771	**-1,105051229**
chrXV	480688	480787	99	0,082637257	1,374877614	0,056697368	-1,583117279	-1,345649687	-0,366677074	-0,051677074	**-1,311677074**
chrXV	480689	480787	98	0,045592969	0,118587827	0,277699771	-0,589688448	-0,501235181	0,454528451	0,776528451	**-0,483471549**
chrXV	480690	480787	97	0,051292091	1,278525005	0,038570786	-1,767517673	-1,502390022	-0,604017791	-0,275017791	**-1,535017791**
chrXV	480691	480787	96	0,065539893	0,796761959	0,076005743	-1,432462555	-1,217593172	-0,33716167	-0,00116167	**-1,26116167**
chrXV	480692	480787	95	0,056991212	1,045055221	0,051713984	-1,628457244	-1,384188657	-0,517485007	-0,174485007	**-1,434485007**
chrXV	480693	480787	94	0,071239015	1,252583918	0,053813099	-1,608954925	-1,367611686	-0,527455211	-0,177455211	**-1,437455211**
chrXV	480695	480787	92	0,059840772	4,498925668	0,013126527	-2,22244785	-1,889080673	-1,130573821	-0,766573821	**-2,026573821**
chrXV	480696	480787	91	0,034194727	0,52623348	0,061015357	-1,546305876	-1,314359995	-0,57193622	-0,20093622	**-1,46093622**
chrXV	480697	480787	90	0,028495606	1,437877396	0,019432713	-2,065608871	-1,75576754	-1,036199292	-0,658199292	**-1,918199292**
chrXV	480698	480787	89	0,04844253	0,26311674	0,155484155	-1,013192315	-0,861213468	-0,185230518	0,199769482	**-1,060230518**

Chrom	Start	End	Length	Section A	Section B	A/A+B	Z-score	Z * 0.85	Phase correction	Length correction	ΔLKnuc
chrXV	480700	480787	87	0,045592969	0,626291959	0,0678583	-1,491933418	-1,268143405	-0,643085721	-0,244085721	-1,504085721
chrXV	480701	480787	86	0,037044288	5,592157194	0,006580736	-2,479369952	-2,107464459	-1,516110132	-1,110110132	-2,370110132
chrXV	480703	480787	84	0,034194727	0,333528262	0,092990452	-1,322562523	-1,124178145	-0,548925352	-0,128925352	-1,388925352
chrXV	480704	480787	83	0,028495606	0,496586524	0,054268855	-1,604800529	-1,364080449	-0,827161518	-0,400161518	-1,660161518
chrXV	482098	482249	151	0,19377012	0,270528479	0,417339445	-0,208704547	-0,177398865	-0,280170937	-0,329170937	-1,589170937
chrXV	483506	483656	150	0,322000346	0,185293479	0,634741308	0,344437377	0,29277177	0,173185308	0,131185308	-1,128814692
chrXV	490437	490586	149	0,347646391	0,251999131	0,579753168	0,201262063	0,171072754	0,067558079	0,032558079	-1,227441921
chrXV	492152	492296	144	0,102584181	0,100058479	0,506231912	0,015621722	0,013278464	-0,027616616	-0,027616616	-1,287616616
chrXV	492636	492766	130	2,892303992	4,254338276	0,404708097	-0,241179248	-0,205002361	0,039714474	0,137714474	-1,122285526
chrXV	499981	500137	156	0,108283302	0,037058696	0,745024175	0,658912981	0,560076034	0,591350516	0,507350516	-0,752649484
chrXV	500251	500392	141	0,182371877	0,155646522	0,539532397	0,099255756	0,084367393	0,15874422	0,17974422	-1,08025578
chrXV	500870	501031	161	1,256656217	1,130290221	0,52647022	0,066399762	0,056439798	0,12899923	0,00999923	-1,25000077
chrXV	504656	504829	173	0,29350474	0,418763262	0,412070652	-0,222221698	-0,188888443	0,069843956	-0,133156044	-1,393156044
chrXV	504839	504995	156	0,037044288	0,048176305	0,434687048	-0,164453569	-0,139785534	-0,105906994	-0,189906994	-1,449906994
chrXV	505651	505827	176	0,270708255	0,570703915	0,321730853	-0,462864205	-0,393434574	-0,073242507	-0,297242507	-1,557242507
chrXV	508541	508687	146	0,843469933	0,711526959	0,542425478	0,106546147	0,090564225	0,168571332	0,154571332	-1,105428668
chrXV	512017	512169	152	0,615505086	0,596645002	0,507779599	0,0195018	0,01657653	-0,072824829	-0,128824829	-1,388824829
chrXV	520467	520627	160	0,04844253	0,048176305	0,50137771	0,003453413	0,002935401	0,070129535	-0,041870465	-1,301870465
chrXV	521328	521487	159	8,802292642	6,259213718	0,584423127	0,213222081	0,181238769	0,226841157	0,121841157	-1,138158843
chrXV	524434	524584	150	0,054141651	0,048176305	0,529151025	0,07313593	0,062165541	-0,077271275	-0,119271275	-1,379271275
chrXV	526545	526704	159	0,105433742	0,048176305	0,686372697	0,485594627	0,412755433	0,44990439	0,34490439	-0,91509561
chrXV	527282	527433	151	0,037044288	0,044470435	0,454449042	-0,114428548	-0,097264266	-0,18965849	-0,23865849	-1,49865849
chrXV	528345	528493	148	0,83207169	0,785644351	0,514349657	0,035977015	0,030580463	0,002745817	-0,025254183	-1,285254183
chrXV	529088	529208	120	0,715239706	3,661399144	0,163422144	-0,980490029	-0,833416525	-0,002024708	0,165975292	-1,094024708
chrXV	534111	534263	152	0,094035499	0,044470435	0,678927584	0,464702061	0,394996752	0,297448784	0,241448784	-1,018551216
chrXV	535122	535279	157	0,267858695	0,200116957	0,572377417	0,18243015	0,155065628	0,175223175	0,084223175	-1,175776825
chrXV	539793	539942	149	0,082637257	0,055588044	0,597844653	0,247772164	0,210606339	0,121285076	0,086285076	-1,173714924
chrXV	540123	540277	154	0,133929347	0,188999349	0,414733497	-0,215385217	-0,183077435	-0,227816261	-0,297816261	-1,557816261
chrXV	542672	542801	129	0,085486818	0,459527828	0,156852331	-1,007478964	-0,856357119	-0,530596178	-0,425596178	-1,685596178
chrXV	545231	545391	160	0,421734966	0,478057176	0,468702656	-0,078531454	-0,066751736	-0,001952932	-0,113952932	-1,373952932
chrXV	545588	545715	127	1,914904712	3,839280883	0,332784663	-0,432236787	-0,367401269	0,180010251	0,299010251	-0,960989749
chrXV	550006	550155	149	0,256460453	0,289057827	0,470122564	-0,074961771	-0,063717505	-0,157809124	-0,192809124	-1,452809124
chrXV	550413	550558	145	0,24221265	0,26311674	0,479316372	-0,051869417	-0,044089004	0,003555036	-0,003444964	-1,263444964
chrXV	550604	550752	148	0,142478029	0,100058479	0,587449825	0,220989833	0,187841358	0,156446399	0,128446399	-1,131553601
chrXV	559497	559585	88	0,037044288	0,200116957	0,15619874	-1,010204174	-0,858673548	-0,231368473	0,160631527	-1,099368473
chrXV	559497	559598	101	0,079787696	0,211234566	0,274163549	-0,600268815	-0,510228493	0,501861261	0,802861261	-0,457138739
chrXV	559497	559601	104	9,67140862	46,46419278	0,172286542	-0,94516806	-0,803392851	0,168513814	0,448513814	-0,811486186
chrXV	559497	559654	157	0,262159574	0,26682261	0,495592445	-0,011048326	-0,009391077	0,005934055	-0,085065945	-1,345065945
chrXV	559919	560068	149	0,518620026	0,200116957	0,721571365	0,587515907	0,499388521	0,394811242	0,359811242	-0,900188758
chrXV	559929	560086	157	0,128230226	0,059293913	0,683806504	0,478369846	0,406614369	0,416093258	0,325093258	-0,934906742
chrXV	565053	565199	146	0,031345166	0,100058479	0,238541073	-0,711003374	-0,604352868	-0,523448662	-0,537448662	-1,797448662
chrXV	565053	565201	148	0,037044288	0,333528262	0,099965007	-1,281750982	-1,089488335	-1,106599187	-1,134599187	-2,394599187
chrXV	565053	565202	149	0,644000692	2,141992616	0,231156582	-0,735043233	-0,624786748	-0,719650298	-0,754650298	-2,014650298
chrXV	566775	566926	151	0,09688506	0,077823261	0,554553208	0,137173592	0,116597553	0,036515654	-0,012484346	-1,272484346
chrXV	576392	576555	163	0,139628469	0,196411088	0,415512001	-0,213388432	-0,181380167	-0,107433993	-0,240433993	-1,500433993
chrXV	590114	590263	149	0,384690679	0,385410436	0,499532686	-0,001171382	-0,000995674	-0,102404152	-0,137404152	-1,397404152
chrXV	590721	590873	152	3,051879385	1,952993268	0,609781626	0,278749922	0,236937434	0,148787604	0,092787604	-1,167212396
chrXV	592017	592170	153	1,584355684	1,070996308	0,596665033	0,244724271	0,20801563	0,10876214	0,04576214	-1,21423786
chrXV	592017	592179	162	0,082637257	0,074117392	0,527175798	0,068172392	0,057946533	0,123351908	-0,002648092	-1,262648092
chrXV	592023	592179	156	0,347646391	0,229763914	0,602078605	0,258731013	0,219921361	0,264716078	0,180716078	-1,079283922
chrXV	592044	592126	82	0,056991212	0,040764565	0,582995843	0,209563572	0,178129036		0,434	-0,826
chrXV	595352	595475	123	0,074088575	0,188999349	0,281611463	-0,578061014	-0,491351862	0,209806653	0,356806653	-0,903193347
chrXV	595452	595607	155	0,102584181	0,118587827	0,463820816	-0,090812431	-0,077190567	-0,08636846	-0,16336846	-1,42336846
chrXV	595885	596019	134	0,128230226	0,081529131	0,611320649	0,282762761	0,240348347	0,52373772	0,59373772	-0,66626228
chrXV	595885	596025	140	0,168124074	0,122293696	0,578904225	0,199091015	0,169227363	0,261702883	0,289702883	-0,970297117
chrXV	596928	597063	135	0,09973462	0,081529131	0,550218231	0,126212725	0,107280816	0,387640454	0,450640454	-0,809359546
chrXV	597189	597334	145	0,09973462	0,066705652	0,599221683	0,251333038	0,213633083	0,257559811	0,250559811	-1,009440189
chrXV	597375	597525	150	0,960301917	0,607762611	0,612412244	0,285611739	0,242769978	0,094897274	0,052897274	-1,207102726
chrXV	597554	597711	157	0,068389454	0,051882174	0,568624996	0,172874585	0,146943398	0,14542998	0,05442998	-1,20557002
chrXV	598045	598174	129	0,065539893	0,100058479	0,395776194	-0,264295292	-0,224650998	0,064512057	0,169512057	-1,090487943
chrXV	598957	599107	150	0,48727486	0,415057393	0,540017115	0,100476838	0,085405313	-0,059578722	-0,101578722	-1,361578722
chrXV	600156	600263	107	0,076938136	0,592939133	0,114854078	-1,201110978	-1,020944331	-0,081555398	0,177444602	-1,082555398
chrXV	601179	601328	149	0,091185939	0,055588044	0,621267729	0,308811997	0,262490198	0,164261354	0,129261354	-1,130738646
chrXV	603463	603616	153	0,3903898	0,292763697	0,571452539	0,180073428	0,153062414	0,054444922	-0,008555078	-1,268555078
chrXV	605449	605600	151	2,157117362	1,3155837	0,621164138	0,308539664	0,262258714	0,175177075	0,126177075	-1,133822925
chrXV	606728	606884	156	0,581310359	0,592939133	0,495048423	-0,012412082	-0,010550269	0,036168336	-0,047831664	-1,307831664
chrXV	608095	608251	156	0,088336378	0,129705435	0,405135037	-0,240077633	-0,204065988	-0,168299031	-0,252299031	-1,512299031
chrXV	610131	610285	154	0,988797522	0,748585655	0,569130365	0,174160574	0,148036488	0,113351294	0,043351294	-1,216648706
chrXV	610432	610587	155	0,190920559	0,140823044	0,575506377	0,190410786	0,161849168	0,161335514	0,084335514	-1,175664486
chrXV	610432	610587	155	0,190920559	0,140823044	0,575506377	0,190410786	0,161849168	0,161335514	0,084335514	-1,175664486
chrXV	613403	613549	146	0,113982423	0,200116957	0,362886495	-0,350753893	-0,298140809	-0,210149098	-0,224149098	-1,484149098
chrXV	617632	617793	161	0,541416511	0,43729261	0,553194508	0,133736447	0,11367598	0,189107239	0,070107239	-1,189892761
chrXV	621001	621151	150	0,176672756	0,140823044	0,556456986	0,141992377	0,12069352	-0,017977946	-0,059977946	-1,319977946
chrXV	622311	622441	130	0,219416165	0,26311674	0,454717518	-0,113751185	-0,096688507	0,148036225	0,246036225	-1,013963775
chrXV	624028	624159	131	3,251348626	2,627461531	0,553062361	0,133402235	0,1133919	0,342714108	0,433714108	-0,826285892
chrXV	625834	625959	125	0,185221438	0,455821958	0,28893744	-0,556491534	-0,473017804	0,168653128	0,301653128	-0,958346872
chrXV	626417	626570	153	0,113982423	0,096352609	0,541908887	0,105243965	0,08945737	0,00511256	-0,05788744	-1,31788744
chrXV	632144	632251	107	0,14532759	0,522527611	0,217603441	-0,780312627	-0,663265733	0,267322144	0,526322144	-0,733677856
chrXV	632144	632253	109	0,139628469	0,807879568	0,147363888	-1,047806479	-0,890635507	0,145348727	0,390348727	-0,869651273
chrXV	633485	633638	153	0,88051422	0,663350655	0,570331144	0,177217294	0,1506347	0,071537658	0,008537658	-1,251462342
chrXV	635429	635578	149	0,398938482	0,363175219	0,523463207	0,058847485	0,050020363	-0,05159384	-0,08659384	-1,34659384
chrXV	637512	637669	157	0,071239015	0,066705652	0,516431814	0,041200102	0,035020087	0,031509855	-0,059490145	-1,319490145
chrXV	641886	642041	155	0,19377012	0,207528696	0,482857442	-0,042983253	-0,036535765	-0,038090187	-0,115090187	-1,375090187
chrXV	646176	646338	162	0,547115632	0,61517435	0,470722144	-0,073454704	-0,062436499	0,015497341	-0,110502659	-1,370502659
chrXV	647481	647636	155	0,133929347	0,144528914	0,480967406	-0,04772575	-0,040566888	-0,041866228	-0,118866228	-1,378866228
chrXV	650796	650922	126	1,786674486	2,923931098	0,379287642	-0,30735223	-0,261249395	0,324727741	0,450727741	-0,809272259
chrXV	650923	651080	157	0,09973462	0,159352392	0,38494643	-0,292515043	-0,248637787	-0,235685502	-0,326685502	-1,586685502
chrXV	651588	651740	152	0,119681545	0,070411522	0,629594476	0,330779497	0,281162572	0,190754779	0,134754779	-1,125245221
chrXV	671862	672011	149	0,7807796	0,555880437	0,584127286	0,21246353	0,180594	0,090998247	0,055998247	-1,204001753
chrXV	674697	674849	152	0,236513528	0,107470218	0,687571814	0,488979272	0,415632381	0,324271907	0,268271907	-0,991728093
chrXV	674884	675018	134	0,376141997	0,340940001	0,524545307	0,06156483	0,052330105	0,33347946	0,40347946	-0,85652054
chrXV	677635	677796	161	2,641542661	2,368050661	0,527296827	0,068476474	0,058205003	0,135862002	0,016862002	-1,243137998
chrXV	679372	679525	153	1,829417895	1,482347831	0,552399549	0,13172615	0,111967227	0,048112048	-0,014887952	-1,274887952
chrXV	680555	680689	134	0,139628469	0,166764131	0,455717497	-0,111228699	-0,094544395	0,202435664	0,272435664	-0,987564336
chrXV	682390	682545	155	0,259310013	0,240881523	0,518421434	0,046192108	0,039263292	0,054093852	-0,022906148	-1,282906148
chrXV	682421	682570	149	0,669646737	0,418763262	0,615252283	0,293034955	0,249079711	0,16404296	0,12904296	-1,13095704

Chrom	Start	End	Length	Section A	Section B	A/A+B	Z-score	Z * 0.85	Phase correction	Length correction	ΔLKnuc
chrXV	682600	682746	146	1,376337762	0,904232177	0,603506053	0,262432662	0,223067762	0,292626903	0,278626903	**-0,981373097**
chrXV	683178	683329	151	0,139628469	0,140823044	0,497870264	-0,005338482	-0,00453771	-0,094272096	-0,143272096	**-1,403272096**
chrXV	683178	683329	151	0,139628469	0,140823044	0,497870264	-0,005338482	-0,00453771	-0,094272096	-0,143272096	**-1,403272096**
chrXV	683542	683705	163	0,111132863	0,140823044	0,441080601	-0,14823007	-0,125995559	-0,04824454	-0,18124454	**-1,44124454**
chrXV	698388	698544	156	0,125380666	0,077823261	0,617018911	0,297660652	0,253011555	0,289662398	0,205662398	**-1,054337602**
chrXV	699187	699320	133	0,085486818	0,214940436	0,284550808	-0,569375094	-0,48396883	-0,184216594	-0,107216594	**-1,367216594**
chrXV	704256	704405	149	0,091185939	0,062999783	0,591403262	0,231156174	0,196482748	0,106514664	0,071514664	**-1,188485336**
chrXV	705052	705198	146	0,09973462	0,092646739	0,518421434	0,046192108	0,039263292	0,094649897	0,080649897	**-1,179350103**
chrXV	707863	708011	148	0,108283302	0,074117392	0,593656197	0,236960278	0,201416237	0,180306295	0,152306295	**-1,107693705**
chrXV	709269	709434	165	9,204080685	7,467327201	0,552087787	0,130937909	0,111297222	0,194247599	0,047247599	**-1,212752401**
chrXV	709269	709434	165	9,204080685	7,467327201	0,552087787	0,130937909	0,111297222	0,194247599	0,047247599	**-1,212752401**
chrXV	709269	709462	193	0,706691025	0,79305609	0,471206791	-0,072236647	-0,06140115	0,383538613	0,040538613	**-1,219461387**
chrXV	709269	709462	193	0,706691025	0,79305609	0,471206791	-0,072236647	-0,06140115	0,383538613	0,040538613	**-1,219461387**
chrXV	709332	709434	102	0,113982423	0,148234783	0,434687048	-0,164453569	-0,139785534	0,85856082	1,15256082	**-0,10743918**
chrXV	709332	709462	130	1,621399972	1,630582615	0,498588147	-0,003538998	-0,003008148	0,248608785	0,346608785	**-0,913391215**
chrXV	709396	709550	154	0,185221438	0,140823044	0,568086406	0,171504378	0,145778722	0,113286189	0,043286189	**-1,216713811**
chrXV	709396	709571	175	0,039893848	0,059293913	0,402205349	-0,247642923	-0,210496484	0,077063731	-0,139936269	**-1,399936269**
chrXV	709403	709571	168	0,852018614	0,915349786	0,4820832	-0,044925865	-0,038186985	0,124124654	-0,043875346	**-1,303875346**
chrXV	712148	712278	130	1,70688679	1,856640659	0,478987973	-0,052693716	-0,044789658	0,189801419	0,287801419	**-0,972198581**
chrXV	713754	713908	154	0,059840772	0,059293913	0,502295129	0,005753068	0,004890107	-0,032633769	-0,102633769	**-1,362633769**
chrXV	716082	716234	152	1,037240052	0,79305609	0,566706135	0,167994342	0,142795191	0,048874133	-0,007125867	**-1,267125867**
chrXV	717294	717452	158	1,273753581	1,67505305	0,431955615	-0,171397488	-0,145687865	-0,101867322	-0,199867322	**-1,459867322**
chrXV	717294	717452	158	1,273753581	1,67505305	0,431955615	-0,171397488	-0,145687865	-0,101867322	-0,199867322	**-1,459867322**
chrXV	728130	728275	145	1,641346896	1,411936309	0,537567853	0,094308252	0,080162014	0,11943527	0,11243527	**-1,14756473**
chrXV	728130	728275	145	1,641346896	1,411936309	0,537567853	0,094308252	0,080162014	0,11943527	0,11243527	**-1,14756473**
chrXV	729241	729409	168	0,227964847	0,285351958	0,444101664	-0,140578003	-0,119491302	0,030059749	-0,137940251	**-1,397940251**
chrXV	730369	730527	158	0,059840772	0,074117392	0,446712395	-0,133971905	-0,113876119	-0,072422755	-0,170422755	**-1,430422755**
chrXV	730626	730772	146	0,347646391	0,303881305	0,533586512	0,0842886	0,07164531	0,123685406	0,109685406	**-1,150314594**
chrXV	732904	733026	122	1,567258321	4,602690017	0,254014821	-0,661908847	-0,56262252	0,181697772	0,335697772	**-0,924302228**
chrXV	736114	736267	153	0,119681545	0,196411088	0,378628074	-0,309085948	-0,262723056	-0,323749186	-0,386749186	**-1,646749186**
chrXV	749719	749841	122	0,085486818	0,385410436	0,181540276	-0,909510814	-0,773084192	-0,021719545	0,132280455	**-1,127719545**
chrXV	751696	751803	107	3,137366202	13,92665788	0,183858519	-0,900757933	-0,765644243	0,159742862	0,418742862	**-0,841257138**
chrXV	751696	751804	108	0,074088575	0,277940218	0,210461691	-0,804820306	-0,68409726	0,310384221	0,562384221	**-0,697615779**
chrXV	751696	751805	109	0,065539893	0,400233914	0,140711848	-1,077126632	-0,915557637	0,099161345	0,344161345	**-0,915838655**
chrXV	751696	751808	112	4,131862846	21,73121921	0,159759105	-0,995448438	-0,846131172	0,166089144	0,390089144	**-0,869910856**
chrXV	759323	759474	151	0,869115978	0,689291742	0,557694862	0,145127411	0,1233583	0,049346526	0,000346526	**-1,259653474**
chrXV	759886	760039	153	0,04844253	0,085235	0,36238349	-0,352095051	-0,299280793	-0,36744603	-0,43044603	**-1,69044603**
chrXV	762029	762161	132	0,287805619	0,144528914	0,665701204	0,428073525	0,363862496	0,619472082	0,703472082	**-0,556527918**
chrXV	762283	762425	142	0,404637603	0,233469783	0,634121484	0,342789226	0,291370842	0,311193285	0,325193285	**-0,934806715**
chrXV	763377	763524	147	0,398938482	0,452116089	0,468757816	-0,078392761	-0,066633846	0,02776646	0,00676646	**-1,25323354**
chrXV	765404	765530	126	0,045592969	0,207528696	0,180122745	-0,914897409	-0,777662797	-0,205529827	-0,079529827	**-1,339529827**
chrXV	766078	766228	150	5,049421354	2,923931098	0,633287113	0,340572049	0,289486242	0,163547362	0,121547362	**-1,138452638**
chrXV	767013	767155	142	0,481575739	0,240881523	0,666580246	0,43048963	0,365916185	0,397072049	0,411072049	**-0,848927951**
chrXV	767013	767155	142	0,481575739	0,240881523	0,666580246	0,43048963	0,365916185	0,397072049	0,411072049	**-0,848927951**
chrXV	768735	768888	153	0,102584181	0,062999783	0,619529685	0,304245806	0,258608935	0,187982478	0,124982478	**-1,135017522**
chrXV	770268	770418	150	0,133929347	0,044470435	0,750725957	0,676776004	0,575259604	0,459982688	0,417982688	**-0,842017312**
chrXV	773124	773277	153	0,427434088	0,429880871	0,498572996	-0,003576976	-0,00304043	-0,064038035	-0,127038035	**-1,387038035**
chrXV	775176	775297	121	1,185417203	5,388334367	0,180325829	-0,914124066	-0,777005456	0,001270391	0,162270391	**-1,097729609**
chrXV	777968	778119	151	0,162424953	0,200116957	0,448017039	-0,130672891	-0,111071958	-0,170257799	-0,219257799	**-1,479257799**
chrXV	783192	783285	93	0,216566604	0,374292827	0,366528133	-0,341062852	-0,289903425	0,518100289	0,875100289	**-0,384899711**
chrXV	789013	789147	134	0,34194727	0,429880871	0,443035505	-0,14327752	-0,121785892	0,162260687	0,232260687	**-1,027739313**
chrXV	790833	790987	154	0,039893848	0,051882174	0,434687048	-0,164453569	-0,139785534	-0,172707383	-0,242707383	**-1,502707383**
chrXV	794701	794852	151	0,324849907	0,233469783	0,581834946	0,206589951	0,175601459	0,122226419	0,073226419	**-1,186773581**
chrXV	796032	796168	136	0,900461144	0,674468263	0,571746988	0,180823617	0,153700074	0,341914596	0,397914596	**-0,862085404**
chrXV	801709	801857	148	1,792373607	1,460112614	0,551078002	0,128385385	0,109127577	0,091687355	0,063687355	**-1,196312645**
chrXV	802168	802313	145	1,926302954	1,58240631	0,549006147	0,123150773	0,104678157	0,125739971	0,118739971	**-1,141260029**
chrXV	804681	804839	158	0,042743409	0,040764565	0,51184823	0,029703476	0,025247955	0,06822868	-0,02977132	**-1,28977132**
chrXV	805157	805300	143	1,598603487	1,063584569	0,600484809	0,254602172	0,216411846	0,241943335	0,248943335	**-1,011056665**
chrXV	806800	806942	142	0,051292091	0,081529131	0,386173911	-0,289305214	-0,245909432	-0,230985644	-0,216985644	**-1,476985644**
chrXV	806939	807090	151	0,575611238	0,44099848	0,566206704	0,1667248	0,14171608	0,076220454	0,027220454	**-1,232779546**
chrXV	810091	810222	131	0,353345512	0,322410653	0,522889069	0,057405902	0,048795016	0,303825816	0,394825816	**-0,865174184**
chrXV	815641	815767	126	0,034194727	0,163058261	0,173354671	-0,940991251	-0,799842563	-0,247179464	-0,121179464	**-1,381179464**
chrXV	816418	816570	152	1,199665006	0,533645219	0,692123654	0,501878922	0,426597084	0,325308175	0,269308175	**-0,990691825**
chrXV	816891	817023	132	0,133929347	0,259410871	0,340492381	-0,411119704	-0,349451748	-0,098052286	-0,014052286	**-1,274052286**
chrXV	823212	823372	160	1,282302263	1,634288484	0,439657934	-0,151836531	-0,129061052	-0,059096545	-0,171096545	**-1,431096545**
chrXV	823715	823880	165	0,621204207	0,563292176	0,524445846	0,061315045	0,052117789	0,137108412	-0,009891588	**-1,269891588**
chrXV	824770	824918	148	1,450426337	2,660814357	0,352795286	-0,377784657	-0,321116959	-0,345170593	-0,373170593	**-1,633170593**
chrXV	824898	825045	147	0,074088575	0,074117392	0,499902783	-0,000243688	-0,000207135	0,062238825	0,041238825	**-1,218761175**
chrXV	826750	826899	149	0,210867483	0,277940218	0,431391491	-0,172832638	-0,146907742	-0,245544788	-0,280544788	**-1,540544788**
chrXV	828443	828590	147	0,344796831	0,26682261	0,563744067	0,160468712	0,136398405	0,199537119	0,178537119	**-1,081462881**
chrXV	829595	829749	154	0,253610892	0,200116957	0,558949362	0,148306006	0,126060105	0,10401252	0,03401252	**-1,22598748**
chrXV	832011	832158	147	1,413382049	1,089525656	0,564696032	0,162886327	0,138453378	0,190044655	0,169044655	**-1,090955345**
chrXV	838047	838199	152	1,467523701	0,896820438	0,62068955	0,307292296	0,261198452	0,154390109	0,098390109	**-1,161609891**
chrXV	838047	838199	152	1,467523701	0,896820438	0,62068955	0,307292296	0,261198452	0,154390109	0,098390109	**-1,161609891**
chrXV	838749	838897	148	1,604302609	0,989467177	0,618521589	0,301600243	0,256360206	0,219783427	0,191783427	**-1,068216573**
chrXV	840989	841143	154	0,170973635	0,118587827	0,590457149	0,22872109	0,194412926	0,171693685	0,101693685	**-1,158306315**
chrXV	845931	846091	160	0,225115286	0,285351958	0,440998495	-0,148438156	-0,126172432	-0,06098733	-0,17298733	**-1,43298733**
chrXV	851758	851911	153	0,088336378	0,122293696	0,419391098	-0,203451441	-0,172933725	-0,229122585	-0,292122585	**-1,552122585**
chrXV	855604	855755	151	1,142673794	0,830114786	0,579217563	0,199892222	0,169908389	0,111147431	0,062147431	**-1,197852569**
chrXV	856174	856339	165	2,162816483	2,004875442	0,518948262	0,047514122	0,040387004	0,138878995	-0,008121005	**-1,268121005**
chrXV	856222	856364	142	0,151026711	0,081529131	0,649421274	0,383758496	0,326194722	0,352162238	0,366162238	**-0,893837762**
chrXV	860271	860423	152	0,108283302	0,140823044	0,434687048	-0,164453569	-0,139785534	-0,248365666	-0,304365666	**-1,564365666**
chrXV	863117	863234	117	0,578460798	9,238732859	0,058923234	-1,563876953	-1,32929541	-0,362576111	-0,173576111	**-1,433576111**
chrXV	866318	866474	156	3,750021728	3,272282838	0,53401582	0,085368593	0,072563304	0,114806706	0,030806706	**-1,229193294**
chrXV	866636	866779	143	2,379383087	0,974643699	0,709410878	0,551664489	0,468914816	0,469504403	0,476504403	**-0,783495597**
chrXV	873282	873420	138	3,339685004	2,249462834	0,597530268	0,246959633	0,209915688	0,353757379	0,395757379	**-0,864242621**
chrXV	873426	873558	132	0,094035499	0,100058479	0,484484373	-0,038901718	-0,03306646	0,231910966	0,315910966	**-0,944089034**
chrXV	873491	873643	152	0,136778908	0,163058261	0,456177292	-0,110069087	-0,093558724	-0,191558695	-0,247558695	**-1,507558695**
chrXV	883077	883230	153	0,170973635	0,096352609	0,639569211	0,357307551	0,303711418	0,245885992	0,182885992	**-1,077114008**
chrXV	883077	883230	153	0,170973635	0,096352609	0,639569211	0,357307551	0,303711418	0,245885992	0,182885992	**-1,077114008**
chrXV	884043	884200	157	0,039893848	0,059293913	0,402205349	-0,247642923	-0,210496484	-0,164341449	-0,255341449	**-1,515341449**
chrXV	895782	895933	151	0,350495952	0,318704784	0,523753088	0,059575384	0,050639077	-0,015438459	-0,064438459	**-1,324438459**
chrXV	900646	900802	156	0,065539893	0,137117174	0,323402949	-0,458203983	-0,389473386	-0,347263989	-0,431263989	**-1,691263989**
chrXV	902699	902828	129	0,44168189	0,833820655	0,346280681	-0,395381497	-0,336074272	-0,046014529	0,058985471	**-1,201014529**
chrXV	904931	905079	148	0,14532759	0,137117174	0,51453455	0,036440779	0,030974662	-0,019752677	-0,047752677	**-1,307752677**
chrXV	917190	917343	153	0,729487509	0,459527828	0,613522371	0,288511463	0,245234744	0,192760462	0,129760462	**-1,130239538**

Chrom	Start	End	Length	Section A	Section B	A/A+B	Z-score	Z * 0.85	Phase correction	Length correction	ΔLKnuc
chrXV	920557	920665	108	0,082637257	1,011702395	0,075513353	-1,435914377	-1,220527221	-0,200185328	0,051814672	**-1,208185328**
chrXV	928849	929014	165	0,24221265	0,344645871	0,412727499	-0,220534382	-0,187454224	-0,082350113	-0,229350113	**-1,489350113**
chrXV	932648	932807	159	1,510267109	1,274819135	0,542269422	0,106152755	0,090229842	0,137993179	0,032993179	**-1,227006821**
chrXV	932960	933112	152	0,723788388	0,455821958	0,613582604	0,288668864	0,245368534	0,156429711	0,100429711	**-1,159570289**
chrXV	937286	937429	143	2,313843194	1,752876311	0,568970442	0,173753594	0,147690555	0,129361802	0,136361802	**-1,123638198**
chrXV	941867	942022	155	0,034194727	0,040764565	0,456177292	-0,110069087	-0,093558724	-0,087698493	-0,164698493	**-1,424698493**
chrXV	943142	943285	143	0,119681545	0,048176305	0,712993436	0,562151022	0,477828368	0,450073829	0,457073829	**-0,802926171**
chrXV	956493	956644	151	0,108283302	0,159352392	0,404592155	-0,241478459	-0,20525669	-0,276012508	-0,325012508	**-1,585012508**
chrXV	958715	958860	145	0,803576085	0,452116089	0,639946718	0,358316375	0,304568919	0,31576094	0,30876094	**-0,95123906**
chrXV	961158	961313	155	0,339097709	0,544762828	0,383655221	-0,295894775	-0,251510559	-0,24920315	-0,32620315	**-1,58620315**
chrXV	963324	963474	150	0,173823196	0,233469783	0,426776803	-0,184586205	-0,156898275	-0,264831515	-0,306831515	**-1,566831515**
chrXV	967141	967307	166	0,319150785	0,655938915	0,327304027	-0,447369833	-0,380264358	-0,249405161	-0,403405161	**-1,663405161**
chrXV	967454	967605	151	0,162424953	0,185293479	0,467116317	-0,082520731	-0,070142622	-0,150313211	-0,199313211	**-1,459313211**
chrXV	970738	970906	168	0,863416857	1,078408047	0,444641973	-0,13921033	-0,11832878	0,028296449	-0,139703551	**-1,399703551**
chrXV	970759	970913	154	0,133929347	0,140823044	0,487454711	-0,031451562	-0,026733828	-0,051936832	-0,121936832	**-1,381936832**
chrXV	970847	970977	130	1,738231956	1,815876094	0,489076846	-0,027383708	-0,023276152	0,220124605	0,318124605	**-0,941875395**
chrXV	970875	970977	102	0,09688506	0,163058261	0,372716096	-0,324668217	-0,275967985	0,711065373	1,005065373	**-0,254934627**
chrXV	972298	972446	148	0,074088575	0,051882174	0,588141101	0,222765786	0,189350918	0,15423807	0,12623807	**-1,13376193**
chrXV	975111	975257	146	0,068389454	0,066705652	0,506231912	0,015621722	0,013278464	0,070537133	0,056537133	**-1,203462867**
chrXV	975228	975386	158	0,028495606	0,051882174	0,354520937	-0,373143192	-0,317171714	-0,281351108	-0,379351108	**-1,639351108**
chrXV	975904	976053	149	0,111132863	0,085235	0,565942212	0,166052574	0,141144688	0,050820135	0,015820135	**-1,244179865**
chrXV	975908	976058	150	0,062690333	0,055588044	0,530023616	0,075329227	0,064029843	-0,05049103	-0,09249103	**-1,35249103**
chrXV	977285	977436	151	1,493169746	1,382289353	0,519280468	0,048347795	0,041095625	-0,035094817	-0,084094817	**-1,344094817**
chrXV	977616	977761	145	0,065539893	0,040764565	0,616530051	0,29638	0,251923	0,262049406	0,255049406	**-1,004950594**
chrXV	981987	982146	159	0,108283302	0,203822827	0,346943851	-0,39358467	-0,33454697	-0,28385498	-0,38885498	**-1,64885498**
chrXV	986417	986558	141	0,541416511	0,522527611	0,508876829	0,022252747	0,018914835	0,076816996	0,097816996	**-1,162183004**
chrXV	986699	986864	165	0,031345166	0,051882174	0,376621026	-0,314367347	-0,267212245	-0,154544023	-0,301544023	**-1,561544023**
chrXV	987920	988058	138	0,04844253	0,35205761	0,120955089	-1,17022564	-0,994691794	-0,865581141	-0,823581141	**-2,083581141**
chrXV	988983	989114	131	3,040481143	3,579870013	0,45926282	-0,102291073	-0,086947412	0,201099782	0,292099782	**-0,967900218**
chrXV	989889	990033	144	0,153876272	0,148234783	0,509336779	0,023405971	0,019895076	-0,022460001	-0,022460001	**-1,282460001**
chrXV	996318	996470	152	2,191312089	1,200701743	0,646020977	0,374599901	0,318409916	0,230414771	0,174414771	**-1,085585229**
chrXV	997102	997264	162	0,111132863	0,185293479	0,374908863	-0,318879276	-0,271047759	-0,196654014	-0,322654014	**-1,582654014**
chrXV	1000108	1000248	140	0,133929347	0,144528914	0,480967406	-0,04772575	-0,040566888	0,053099853	0,081099853	**-1,178900147**
chrXV	1000780	1000917	137	0,29065518	0,285351958	0,504603434	0,011539355	0,009808451	0,179954295	0,228954295	**-1,031045705**
chrXV	1001402	1001553	151	0,14532759	0,107470218	0,574876781	0,188803997	0,160483398	0,086022968	0,037022968	**-1,222977032**
chrXV	1002082	1002260	178	0,079787696	0,311293045	0,204018475	-0,827353109	-0,703250143	-0,358832932	-0,596832932	**-1,856832932**
chrXV	1004460	1004618	158	0,039893848	0,062999783	0,387719315	-0,285268256	-0,242478018	-0,20434428	-0,30234428	**-1,56234428**
chrXV	1004460	1004618	158	0,039893848	0,062999783	0,387719315	-0,285268256	-0,242478018	-0,20434428	-0,30234428	**-1,56234428**
chrXV	1005856	1006001	145	0,159575393	0,17417587	0,478126708	-0,054855711	-0,046627354	-0,040118755	-0,047118755	**-1,307118755**
chrXV	1008258	1008393	135	0,213717044	0,26682261	0,444743825	-0,138952542	-0,118109661	0,14570597	0,20870597	**-1,05129403**
chrXV	1019659	1019809	150	0,088336378	0,077823261	0,531635591	0,07938196	0,067474666	-0,050074057	-0,092074057	**-1,352074057**
chrXV	1019667	1019830	163	0,09973462	0,092646739	0,518421434	0,046192108	0,039263292	0,11234849	-0,02065151	**-1,28065151**
chrXV	1023929	1024082	153	0,279256937	0,292763697	0,488193818	-0,029598031	-0,025158326	-0,083759754	-0,146759754	**-1,406759754**
chrXV	1035603	1035747	144	0,985947962	0,670762394	0,59512392	0,240745775	0,204633908	0,158296097	0,158296097	**-1,101703903**
chrXV	1036929	1037060	131	0,105433742	0,144528914	0,421797974	-0,19729595	-0,167701557	0,132420173	0,223420173	**-1,036579827**
chrXV	1041309	1041484	175	0,378991558	0,544762828	0,410273081	-0,226842569	-0,192816183	0,10772199	-0,10927801	**-1,36927801**
chrXV	1042260	1042368	108	0,028495606	0,937585003	0,029496095	-1,888251531	-1,605013801	-0,606496058	-0,354496058	**-1,614496058**
chrXV	1042260	1042387	127	0,056991212	0,08894087	0,390532439	-0,277931585	-0,236241848	0,30982722	0,42882722	**-0,83117278**
chrXV	1042260	1042389	129	0,04844253	0,08894087	0,352608321	-0,378288023	-0,32154482	-0,017543949	0,087456051	**-1,172543949**
chrXV	1042260	1042392	132	23,98760099	27,18255336	0,468781095	-0,078334229	-0,066584095	0,223356769	0,307356769	**-0,952643231**
chrXV	1042265	1042392	127	0,327699467	0,411351523	0,443405761	-0,142339911	-0,120988925	0,435036139	0,554036139	**-0,705963861**
chrXV	1042267	1042368	101	0,113982423	0,144528914	0,440918471	-0,148640972	-0,126344826	0,878280057	1,179280057	**-0,080719943**
chrXV	1043663	1043770	107	2,282498027	11,49190156	0,165705809	-0,971274471	-0,8255833	0,105430677	0,364430677	**-0,895569323**
chrXV	1043663	1043771	108	0,068389454	0,251999131	0,213457836	-0,794480647	-0,67530855	0,336592979	0,588592979	**-0,671407021**
chrXV	1043663	1043772	109	2,707082554	15,49794658	0,14869971	-1,042026441	-0,885722475	0,118211741	0,363211741	**-0,896788259**
chrXV	1047470	1047621	151	0,233663968	0,207528696	0,529618887	0,074311878	0,063165096	-0,014244143	-0,063244143	**-1,323244143**
chrXV	1047470	1047621	151	0,233663968	0,207528696	0,529618887	0,074311878	0,063165096	-0,014244143	-0,063244143	**-1,323244143**
chrXV	1050132	1050280	148	3,681632274	3,009166098	0,550253059	0,126300727	0,107355618	0,075677621	0,047677621	**-1,212322379**
chrXV	1050137	1050280	143	0,042743409	0,037058696	0,535617563	0,08939893	0,07598909	0,048634037	0,055634037	**-1,204365963**
chrXV	1050608	1050773	165	1,818019652	1,556465223	0,538754719	0,097296971	0,082702426	0,202973509	0,055973509	**-1,204026491**
chrXV	1050861	1050999	138	0,111132863	0,107470218	0,508377386	0,021000537	0,017850457	0,116037679	0,158037679	**-1,101962321**
chrXV	1052062	1052210	148	0,142478029	0,048176305	0,747310729	0,66605094	0,566143299	0,531364382	0,503364382	**-0,756635618**
chrXV	1056315	1056470	155	0,139628469	0,133411305	0,511385088	0,028542059	0,02426075	0,030562337	-0,046437663	**-1,306437663**
chrXV	1059723	1059876	153	1,344992595	0,748585655	0,642437222	0,364981045	0,310233888	0,244744473	0,181744473	**-1,078255527**
chrXV	1060927	1061054	127	0,105433742	0,151940653	0,409651247	-0,228442189	-0,194175861	0,362473094	0,481473094	**-0,778526906**
chrXV	1061685	1061838	153	1,393435125	1,000584786	0,582048261	0,207136216	0,176065784	0,108288028	0,045288028	**-1,214711972**
chrXV	1062032	1062164	132	0,170973635	0,303881305	0,360054451	-0,358313251	-0,304566264	-0,018783223	0,065216777	**-1,194783223**
chrXV	1062744	1062896	152	0,165274514	0,129705435	0,560290672	0,151706213	0,128950281	0,046173857	-0,009826143	**-1,269826143**
chrXV	1071888	1072059	171	1,373488201	2,279109791	0,376030487	-0,315922974	-0,268534528	-0,094313612	-0,283313612	**-1,543313612**
chrXV	1073330	1073480	150	0,122531105	0,100058479	0,55047996	0,126874057	0,107842948	0,000274124	-0,041725876	**-1,301725876**
chrXV	1080435	1080579	144	1,310797868	0,670762394	0,661497858	0,416554516	0,354071338	0,306898884	0,306898884	**-0,953101116**
chrXV	1085191	1085346	155	0,416035845	0,466939567	0,471174893	-0,072316812	-0,06146929	-0,053067537	-0,130067537	**-1,390067537**
chrXV	1086206	1086377	171	0,037044288	0,062999783	0,370279692	-0,331112667	-0,281445767	-0,078536511	-0,267536511	**-1,527536511**
chrXV	1086356	1086503	147	1,615700851	1,078408047	0,599716237	0,252612686	0,214720783	0,263393393	0,242393393	**-1,017606607**
chrXV	1086512	1086642	130	0,29065518	0,26311674	0,524864424	0,062366274	0,053011333	0,299502856	0,397502856	**-0,862497144**
chrXV	1086893	1087029	136	0,530018269	0,689291742	0,434687048	-0,164453569	-0,139785534	0,047120071	0,103120071	**-1,156879929**
chrXV	1087274	1087436	162	0,051292091	0,055588044	0,479902939	-0,050397187	-0,042837609	0,031257258	-0,094742742	**-1,354742742**
chrXV	1087375	1087530	155	0,082637257	0,207528696	0,284793085	-0,568661074	-0,483361913	-0,462486779	-0,539486779	**-1,799486779**
chrXV	1087509	1087646	137	0,185221438	0,096352609	0,657807209	0,406485945	0,345513053	0,511361264	0,560361264	**-0,699638736**
chrXV	1087804	1087947	143	0,398938482	0,344645871	0,536507365	0,09163849	0,077892716	0,048759709	0,055759709	**-1,204240291**
chrXV	1087852	1087977	125	0,159575393	0,192705218	0,452978074	-0,118140734	-0,100419624	0,522067118	0,655067118	**-0,604932882**
chrXV	1088077	1088229	152	0,105433742	0,074117392	0,58720733	0,220367006	0,187311955	0,119855504	0,063855504	**-1,196144496**
chrXV	1088150	1088294	144	0,125380666	0,055588044	0,692830634	0,503889924	0,428306435	0,355820531	0,355820531	**-0,904179469**
chrXV	1088247	1088382	135	1,185417203	0,722644568	0,621267729	0,308811997	0,262490198	0,503724222	0,566724222	**-0,693275778**
chrXV	1088247	1088434	187	0,162424953	0,066705652	0,708874979	0,550101085	0,467585923	0,892753984	0,591753984	**-0,668246016**
chrXV	1088290	1088382	92	0,162424953	0,140823044	0,535617563	0,08939893	0,07598909	0,847728761	1,211728761	**-0,048271239**
chrXV	1088290	1088434	144	1,262355338	0,563292176	0,691456225	0,499982287	0,424984944	0,334603135	0,334603135	**-0,925396865**
chrXV	1088629	1088781	152	0,122531105	0,111176087	0,524293257	0,060931471	0,05179207	-0,019406845	-0,075406845	**-1,335406845**
chrXV	1088930	1089060	130	0,416035845	0,466939567	0,471174893	-0,072316812	-0,06146929	0,170550039	0,268550039	**-0,991449961**
chrXV	1089068	1089229	161	0,230814407	0,181587609	0,559683023	0,150165614	0,127640772	0,175280688	0,056280688	**-1,203719312**
chrXV	1090465	1090616	151	0,63545201	0,363175219	0,636325539	0,348654215	0,296356083	0,225516495	0,176516495	**-1,083483505**
chrXVI	523	674	151	0,572761677	0,403939784	0,586424511	0,218356986	0,185603438	0,035128748	-0,013871252	**-1,273871252**
chrXVI	847	1021	174	0,028495606	0,059293913	0,324590066	-0,454901459	-0,386666241	-0,169074792	-0,379074792	**-1,639074792**
chrXVI	1688	1839	151	0,416035845	0,240881523	0,633315339	0,340647026	0,289549972	0,14256344	0,09356344	**-1,16643656**
chrXVI	1700	1834	134	0,703841464	0,478057176	0,595517619	0,241761772	0,205497507	0,263119268	0,333119268	**-0,926880732**
chrXVI	1725	1821	96	0,054141651	0,074117392	0,42212736	-0,196454145	-0,166986023	0,343875265	0,679875265	**-0,580124735**

Chrom	Start	End	Length	Section A	Section B	A/A+B	Z-score	Z * 0.85	Phase correction	Length correction	ΔLKnuc
chrXVI	1725	1834	109	0,042743409	0,051882174	0,451710916	-0,121339881	-0,103138899	0,754581057	0,999581057	**-0,260418943**
chrXVI	2079	2209	130	0,361894194	0,407645654	0,470273495	-0,074582384	-0,063395026	0,141565089	0,239565089	**-1,020434911**
chrXVI	2079	2209	130	0,361894194	0,407645654	0,470273495	-0,074582384	-0,063395026	0,141565089	0,239565089	**-1,020434911**
chrXVI	2358	2510	152	0,168124074	0,125999566	0,571610206	0,180475116	0,153403848	0,059413024	0,003413024	**-1,256586976**
chrXVI	2705	2849	144	1,256656217	0,559586306	0,691898907	0,501240058	0,426054049	0,311657962	0,311657962	**-0,948342038**
chrXVI	2705	2892	187	0,125380666	0,096352609	0,56545715	0,164819952	0,140096959	0,333227232	0,032227232	**-1,227772768**
chrXVI	2757	2849	92	0,136778908	0,111176087	0,551627959	0,129775456	0,110309137	0,579086463	0,943086463	**-0,316913537**
chrXVI	2757	2892	135	1,276603141	0,774526742	0,622390202	0,311764379	0,264999722	0,262389421	0,325389421	**-0,934610579**
chrXVI	2845	2989	144	0,125380666	0,044470435	0,738179884	0,637744162	0,542082538	0,4307497	0,4307497	**-0,8292503**
chrXVI	2910	3062	152	0,088336378	0,066705652	0,569757619	0,175757115	0,149393547	0,059400239	0,003400239	**-1,256599761**
chrXVI	3162	3287	125	0,19377012	0,240881523	0,445805562	-0,136265845	-0,115825968	0,400110375	0,533110375	**-0,726889625**
chrXVI	3192	3335	143	0,350495952	0,400233914	0,466873595	-0,083131234	-0,070661549	-0,16927426	-0,16227426	**-1,42227426**
chrXVI	3192	3343	151	0,045592969	0,040764565	0,527955893	0,070132481	0,059612609	-0,088479633	-0,137479633	**-1,397479633**
chrXVI	3493	3630	137	0,159575393	0,103764348	0,605967759	0,268824834	0,228501109	0,162904284	0,211904284	**-1,048095716**
chrXVI	3609	3764	155	0,079787696	0,17417587	0,314169853	-0,484065048	-0,411455291	-0,5158086	-0,5928086	**-1,8528086**
chrXVI	3703	3865	162	0,059840772	0,055588044	0,518421434	0,046192108	0,039263292	0,104344522	-0,021655478	**-1,281655478**
chrXVI	3703	3865	162	0,059840772	0,055588044	0,518421434	0,046192108	0,039263292	0,104344522	-0,021655478	**-1,281655478**
chrXVI	4928	5069	141	0,125380666	0,096352609	0,56545715	0,164819952	0,140096959	0,030414419	0,051414419	**-1,208585581**
chrXVI	8461	8628	167	0,045592969	0,044470435	0,506231912	0,015621722	0,013278464	0,194044472	0,033044472	**-1,226955528**
chrXVI	10566	10710	144	1,250957096	0,822703046	0,603260424	0,261795443	0,222526126	0,103256195	0,103256195	**-1,156743805**
chrXVI	23283	23434	151	3,735773925	2,116051529	0,638394626	0,354170984	0,301045337	0,149497775	0,100497775	**-1,159502225**
chrXVI	26164	26341	177	0,182371877	0,344645871	0,346045039	-0,396020265	-0,336617225	-0,128423797	-0,359423797	**-1,619423797**
chrXVI	26440	26591	151	0,111132863	0,092646739	0,54535813	0,113942039	0,096850733	-0,054898592	-0,103898592	**-1,363898592**
chrXVI	30175	30330	155	0,039893848	0,055588044	0,417815854	-0,20748423	-0,176361595	-0,280591609	-0,357591609	**-1,617591609**
chrXVI	30411	30566	155	0,313451664	0,244587392	0,561702018	0,155285859	0,13199298	0,029291549	-0,047708451	**-1,307708451**
chrXVI	37895	38053	158	0,199469241	0,122293696	0,619926094	0,305286692	0,259493688	0,218325368	0,120325368	**-1,139674632**
chrXVI	41612	41762	150	0,267858695	0,203822827	0,567880408	0,170980391	0,145333332	-0,025971575	-0,067971575	**-1,327971575**
chrXVI	41938	42057	119	0,039893848	0,170470001	0,189642129	-0,879215839	-0,747333463	-0,104522898	0,070477102	**-1,189522898**
chrXVI	44256	44374	118	0,071239015	0,366881088	0,162601565	-0,983821816	-0,836248543	-0,142080111	0,039919889	**-1,220080111**
chrXVI	54075	54252	177	0,037044288	0,077823261	0,322495674	-0,460731366	-0,391621661	-0,189003736	-0,420003736	**-1,680003736**
chrXVI	55410	55560	150	0,376141997	0,237175653	0,613290677	0,287906064	0,244720154	0,073759223	0,031759223	**-1,228240777**
chrXVI	56814	56963	149	0,094035499	0,051882174	0,644442151	0,370358087	0,314804374	0,158809887	0,123809887	**-1,136190113**
chrXVI	56988	57111	123	0,088336378	0,214940436	0,291273101	-0,549669321	-0,467218923	0,13827172	0,28527172	**-0,97472828**
chrXVI	57088	57243	155	0,116831984	0,096352609	0,548032024	0,120690792	0,102587173	0,00098854	-0,07601146	**-1,33601146**
chrXVI	57521	57655	134	0,111132863	0,122293696	0,47609348	-0,059960669	-0,050966568	-0,005236979	0,064763021	**-1,195236979**
chrXVI	57521	57661	140	0,156725832	0,144528914	0,520243529	0,050764798	0,043150079	-0,044093078	-0,016093078	**-1,276093078**
chrXVI	57717	57868	151	0,116831984	0,081529131	0,588986325	0,224938202	0,191197471	0,041831312	-0,007168688	**-1,267168688**
chrXVI	57727	57872	145	0,091185939	0,08894087	0,506231912	0,015621722	0,013278464	-0,086081866	-0,093081866	**-1,353081866**
chrXVI	57747	57896	149	0,051292091	0,037058696	0,58055047	0,203301913	0,172806626	0,013173628	-0,021826372	**-1,281826372**
chrXVI	57999	58153	154	0,208017923	0,122293696	0,629762657	0,331224804	0,281541083	0,213862576	0,143862576	**-1,116137424**
chrXVI	58564	58699	135	0,068389454	0,077823261	0,467739443	-0,080953558	-0,068810524	-0,068287266	-0,005287266	**-1,265287266**
chrXVI	58718	58879	161	0,076938136	0,085235	0,474419732	-0,064164224	-0,05453959	-0,025572534	-0,144572534	**-1,404572534**
chrXVI	59011	59161	150	8,842186491	4,22098545	0,676878979	0,458989034	0,390140678	0,221078645	0,179078645	**-1,080921355**
chrXVI	59190	59347	157	0,068389454	0,070411522	0,492715945	-0,018259434	-0,015520519	-0,089055979	-0,180055979	**-1,440055979**
chrXVI	59681	59810	129	0,037044288	0,103764348	0,263082498	-0,633871027	-0,538790373	-0,289006599	-0,184006599	**-1,444006599**
chrXVI	60593	60743	150	0,14532759	0,181587609	0,444542163	-0,139462957	-0,118543513	-0,277459688	-0,319459688	**-1,579459688**
chrXVI	61572	61714	142	0,04844253	0,037058696	0,566571175	0,16765125	0,142503563	0,036424799	0,050424799	**-1,209575201**
chrXVI	66888	67040	152	2,242604179	1,656523702	0,575155329	0,189514819	0,161087596	0,074766204	0,018766204	**-1,241233796**
chrXVI	67654	67814	160	0,273557816	0,270528479	0,502783875	0,006978197	0,005931468	0,004210129	-0,107789871	**-1,367789871**
chrXVI	69456	69617	161	0,455929693	0,452116089	0,502099897	0,005263685	0,004474132	0,027062486	-0,091937514	**-1,351937514**
chrXVI	73482	73631	149	0,128230226	0,040764565	0,75878212	0,702390353	0,5970318	0,42884919	0,39384919	**-0,86615081**
chrXVI	74222	74371	149	3,507809078	1,511994788	0,698794051	0,520935222	0,442794938	0,264020507	0,229020507	**-1,030979493**
chrXVI	85235	85390	155	0,071239015	0,077823261	0,477914444	-0,055388588	-0,0470803	-0,153098199	-0,230098199	**-1,490098199**
chrXVI	86229	86396	167	0,113982423	0,166764131	0,405997586	-0,23785292	-0,202174982	-0,016505095	-0,177505095	**-1,437505095**
chrXVI	93799	93933	134	0,213717044	0,218646305	0,494299631	-0,014289193	-0,012145814	0,037767312	0,107767312	**-1,152232688**
chrXVI	97906	98054	148	0,361894194	0,188999349	0,656922193	0,404077658	0,343466009	0,191920125	0,163920125	**-1,096079875**
chrXVI	100090	100196	106	0,398938482	4,146868058	0,08775967	-1,354680605	-1,151478514	-0,562415834	-0,296415834	**-1,556415834**
chrXVI	101988	102144	156	0,376141997	0,17417587	0,683499518	0,477507244	0,405881157	0,329821332	0,245821332	**-1,014178668**
chrXVI	111563	111694	131	1,279452702	1,271113265	0,501634821	0,004097899	0,003483215	0,147795452	0,238795452	**-1,021204548**
chrXVI	111839	111996	157	0,789328282	0,70040935	0,529843823	0,074877279	0,063645687	0,001503818	-0,089496182	**-1,349496182**
chrXVI	112816	112964	148	0,091185939	0,040764565	0,691061693	0,498861988	0,42403269	0,278587346	0,250587346	**-1,009412654**
chrXVI	113851	114002	151	2,675737388	1,634288484	0,620817013	0,307627261	0,261483172	0,11784334	0,06884334	**-1,19115666**
chrXVI	115900	116007	107	1,376337762	9,605613948	0,125327246	-1,148761129	-0,97644696	-0,335787112	-0,076787112	**-1,336787112**
chrXVI	115900	116008	108	0,034194727	0,296469566	0,103412215	-1,262345669	-1,072993819	-0,306082644	-0,054082644	**-1,314082644**
chrXVI	115900	116009	109	0,037044288	0,270528479	0,120440727	-1,172786449	-0,996868482	-0,136531721	0,108468279	**-1,151531721**
chrXVI	115900	116013	113	1,327895232	12,17378157	0,098350394	-1,291008333	-1,097357083	-0,302128504	-0,085128504	**-1,345128504**
chrXVI	117285	117438	153	0,222265726	0,059293913	0,789409045	0,804372445	0,683716578	0,628640195	0,565640195	**-0,694359805**
chrXVI	119500	119650	150	1,102779946	1,007996525	0,522452264	0,056309222	0,047862839	-0,106520542	-0,148520542	**-1,408520542**
chrXVI	119632	119770	138	0,088336378	0,125999566	0,412139824	-0,222043979	-0,188737382	-0,269245546	-0,227245546	**-1,487245546**
chrXVI	124878	125016	138	0,954602795	0,637409568	0,599620215	0,252364199	0,214509569	0,147026653	0,189026653	**-1,070973347**
chrXVI	127061	127209	148	0,455929693	0,26682261	0,630824269	0,334037235	0,283931649	0,139485645	0,111485645	**-1,148514355**
chrXVI	127061	127209	148	0,455929693	0,26682261	0,630824269	0,334037235	0,283931649	0,139485645	0,111485645	**-1,148514355**
chrXVI	127345	127506	161	0,333398588	0,359469349	0,481186342	-0,047176341	-0,04009989	-0,006029955	-0,125029955	**-1,385029955**
chrXVI	128019	128185	166	0,037044288	0,100058479	0,270193583	-0,612227627	-0,520393483	-0,34916196	-0,50316196	**-1,76316196**
chrXVI	128210	128316	106	0,655398934	1,967816746	0,249845615	-0,674975659	-0,573729311	0,038575618	0,304575618	**-0,955424382**
chrXVI	128210	128332	122	0,530018269	2,212404138	0,193266459	-0,865922035	-0,736033729	-0,112193357	0,041806643	**-1,218193357**
chrXVI	130172	130324	152	0,094035499	0,218646305	0,300738636	-0,522277301	-0,443935706	-0,521982522	-0,577982522	**-1,837982522**
chrXVI	131123	131279	156	0,92610719	0,604056741	0,605233969	0,266918306	0,22688056	0,151880866	0,067880866	**-1,192119134**
chrXVI	136763	136914	151	1,188266763	0,667056524	0,640463455	0,359697864	0,305743184	0,170238157	0,121238157	**-1,138761843**
chrXVI	136805	136963	158	0,168124074	0,125999566	0,571610206	0,180475116	0,153403848	0,118564759	0,020564759	**-1,239435241**
chrXVI	139377	139534	157	0,24506221	0,274234349	0,47191187	-0,070464771	-0,059895155	-0,119719293	-0,210719293	**-1,470719293**
chrXVI	142443	142561	118	0,153876272	1,285936744	0,106872399	-1,24333395	-1,056833857	-0,375646725	-0,193646725	**-1,453646725**
chrXVI	144178	144336	158	0,082637257	0,077823261	0,515000562	0,037609696	0,031968242	-0,003994703	-0,101994703	**-1,361994703**
chrXVI	149028	149196	168	0,113982423	0,107470218	0,514703382	0,036864261	0,031334622	0,223188971	0,055188971	**-1,204811029**
chrXVI	152572	152722	150	1,826568334	0,915349786	0,666164435	0,429346432	0,364944468	0,205408307	0,163408307	**-1,096591693**
chrXVI	161247	161414	167	0,871965539	0,915349786	0,487863292	-0,030426909	-0,025862873	0,161367021	0,000367021	**-1,259632979**
chrXVI	164847	164957	110	0,230814407	1,84922892	0,110966154	-1,221406078	-1,038195166	-0,184490994	0,053509006	**-1,206490994**
chrXVI	165348	165502	154	0,262159574	0,377998697	0,40952306	-0,228772014	-0,194456212	-0,264971934	-0,334971934	**-1,594971934**
chrXVI	167030	167184	154	0,304902982	0,137117174	0,689794295	0,495267357	0,420977253	0,349594429	0,279594429	**-0,980405571**
chrXVI	169266	169418	152	0,270708255	0,196411088	0,579526966	0,200683491	0,170580968	0,094184225	0,038184225	**-1,221815775**
chrXVI	173412	173549	137	0,424584527	0,240881523	0,638025828	0,353186878	0,300208846	0,229733126	0,278733126	**-0,981266874**
chrXVI	174194	174363	169	0,752283994	1,041349352	0,41941905	-0,203379911	-0,172872925	0,024995935	-0,150004065	**-1,410004065**
chrXVI	175111	175256	145	0,210867483	0,207528696	0,503989982	0,010001569	0,008501334	-0,090605346	-0,097605346	**-1,357605346**
chrXVI	177466	177621	155	0,259310013	0,211234566	0,551084902	0,128402824	0,1091424	0,0055767	-0,0714233	**-1,3314233**
chrXVI	177625	177797	172	0,039893848	0,066705652	0,374240479	-0,320642986	-0,272546538	-0,05437597	-0,25037597	**-1,51037597**
chrXVI	189167	189337	170	0,037044288	0,062999783	0,370279692	-0,331112667	-0,281445767	-0,085417555	-0,267417555	**-1,527417555**

Chrom	Start	End	Length	Section A	Section B	A/A+B	Z-score	Z * 0.85	Phase correction	Length correction	ΔLKnuc
chrXVI	190409	190560	151	0,447381012	0,415057393	0,518739668	0,04699067	0,039942069	-0,098630912	-0,147630912	**-1,407630912**
chrXVI	191083	191228	145	0,515770466	0,459527828	0,528833557	0,072338048	0,061487341	-0,04132992	-0,04832992	**-1,30832992**
chrXVI	193727	193886	159	0,265009134	0,137117174	0,659019638	0,409789016	0,348320663	0,344312842	0,239312842	**-1,020687158**
chrXVI	194794	194946	152	0,450230572	0,455821958	0,496914425	-0,007734467	-0,006574297	-0,077461999	-0,133461999	**-1,393461999**
chrXVI	198714	198866	152	0,222265726	0,237175653	0,483773852	-0,040684142	-0,03458152	-0,108144256	-0,164144256	**-1,424144256**
chrXVI	200785	200926	141	0,04844253	0,040764565	0,543034495	0,108081541	0,09186931	-0,011227923	0,009772077	**-1,250227923**
chrXVI	203076	203237	161	0,14532759	0,144528914	0,50137771	0,003453413	0,002935401	0,054542265	-0,064457735	**-1,324457735**
chrXVI	203882	204036	154	0,467327936	0,385410436	0,548032024	0,120690792	0,102587173	0,037576264	-0,032423736	**-1,292423736**
chrXVI	205158	205313	155	0,339097709	0,207528696	0,620346375	0,30639062	0,260432027	0,155654951	0,078654951	**-1,181345049**
chrXVI	211347	211529	182	0,210867483	0,329822392	0,389997099	-0,279326594	-0,237427605	-0,031206804	-0,297206804	**-1,557206804**
chrXVI	212567	212715	148	1,427629852	1,011702395	0,585254368	0,215354086	0,183050973	0,033816915	0,005816915	**-1,254183085**
chrXVI	213085	213234	149	0,589859041	0,489174784	0,546654819	0,117214141	0,09963202	-0,068917648	-0,103917648	**-1,363917648**
chrXVI	220298	220444	146	0,435982769	0,270528479	0,617092467	0,297853387	0,253175379	0,148159146	0,134159146	**-1,125840854**
chrXVI	227791	227944	153	3,27414511	2,501461966	0,566891942	0,168466728	0,143196719	0,077127488	0,014127488	**-1,245872512**
chrXVI	228023	228173	150	0,78362916	0,718938698	0,521526636	0,053985485	0,045887663	-0,113878485	-0,155878485	**-1,415878485**
chrXVI	235024	235161	137	0,14817715	0,188999349	0,439464645	-0,152326671	-0,12947767	-0,202746474	-0,153746474	**-1,413746474**
chrXVI	238735	238888	153	0,521469587	0,511410002	0,504869679	0,012206779	0,010375762	-0,057864364	-0,120864364	**-1,380864364**
chrXVI	239149	239288	139	0,227964847	0,270528479	0,457307721	-0,107218749	-0,091135937	-0,170078608	-0,135078608	**-1,395078608**
chrXVI	240071	240209	138	0,156725832	0,114881957	0,577029962	0,194301161	0,165155987	0,097719347	0,139719347	**-1,120280653**
chrXVI	240947	241098	151	0,125380666	0,070411522	0,640376244	0,359464657	0,305544959	0,176259947	0,127259947	**-1,132740053**
chrXVI	242739	242891	152	0,453080133	0,374292827	0,547612932	0,119632674	0,101687773	0,032261237	-0,023738763	**-1,283738763**
chrXVI	242995	243131	136	0,530018269	0,663350655	0,444136141	-0,140490722	-0,119417114	-0,17129551	-0,11529551	**-1,37529551**
chrXVI	244964	245114	150	4,325632966	3,090695229	0,583258029	0,210235413	0,178700101	0,010835994	-0,031164006	**-1,291164006**
chrXVI	246169	246331	162	0,339097709	0,34835174	0,493269301	-0,016872161	-0,014341337	0,052344659	-0,073655341	**-1,333655341**
chrXVI	247500	247655	155	0,612655525	0,44099848	0,581457976	0,205624744	0,174781032	0,073479895	-0,003520105	**-1,263520105**
chrXVI	247870	248025	155	0,142478029	0,155646522	0,477914444	-0,055388588	-0,0470803	-0,152905312	-0,229905312	**-1,489905312**
chrXVI	248518	248667	149	0,353345512	0,333528262	0,514425686	0,036167715	0,030742558	-0,133423709	-0,168423709	**-1,428423709**
chrXVI	251350	251499	149	0,139628469	0,144528914	0,491377234	-0,021615751	-0,018373388	-0,185166043	-0,220166043	**-1,480166043**
chrXVI	251647	251802	155	0,398938482	0,181587609	0,68720164	0,487933817	0,414743744	0,316276752	0,239276752	**-1,020723248**
chrXVI	254749	254901	152	0,031345166	0,059293913	0,345823971	-0,396619676	-0,337126724	-0,419333272	-0,475333272	**-1,735333272**
chrXVI	254749	254901	152	0,031345166	0,059293913	0,345823971	-0,396619676	-0,337126724	-0,419333272	-0,475333272	**-1,735333272**
chrXVI	257303	257455	152	0,250761331	0,203822827	0,551627959	0,129775456	0,110309137	0,024207269	-0,031792731	**-1,291792731**
chrXVI	258470	258614	144	0,24506221	0,237175653	0,508177041	0,020498237	0,017423501	-0,101375157	-0,101375157	**-1,361375157**
chrXVI	269441	269592	151	0,062690333	0,111176087	0,360566076	-0,356946107	-0,303404191	-0,430677473	-0,479677473	**-1,739677473**
chrXVI	272088	272225	137	0,424584527	0,329822392	0,562805717	0,158086591	0,134373602	0,056416884	0,105416884	**-1,154583116**
chrXVI	273849	274002	153	0,028495606	0,08894087	0,242646977	-0,69781331	-0,593141313	-0,66119593	-0,72419593	**-1,98419593**
chrXVI	275518	275683	165	0,039893848	0,125999566	0,240478794	-0,704763239	-0,599048753	-0,45657557	-0,60357557	**-1,86357557**
chrXVI	278767	278916	149	0,404637603	0,277940218	0,592808014	0,234774229	0,199558094	0,036683217	0,001683217	**-1,258316783**
chrXVI	284968	285121	153	1,302249187	0,711526959	0,646670281	0,376346334	0,319894384	0,254644964	0,191644964	**-1,068355036**
chrXVI	287278	287435	157	0,04844253	0,037058696	0,566571175	0,16765125	0,142503563	0,076699869	-0,014300131	**-1,274300131**
chrXVI	287516	287643	127	0,156725832	0,303881305	0,340259235	-0,411755734	-0,349992374	0,079661292	0,198661292	**-1,061338708**
chrXVI	295119	295280	161	0,256460453	0,192705218	0,570970734	0,178846117	0,1520192	0,206771676	0,087771676	**-1,172228324**
chrXVI	296343	296490	147	0,720938828	0,52993935	0,576346155	0,192554744	0,163671533	0,069383491	0,048383491	**-1,211616509**
chrXVI	301254	301382	128	0,034194727	0,114881957	0,229376762	-0,740900911	-0,629765774	-0,287572917	-0,175572917	**-1,435572917**
chrXVI	305851	306015	164	0,359044634	0,559586306	0,39084753	-0,277110761	-0,235544147	-0,11799359	-0,25799359	**-1,51799359**
chrXVI	306387	306542	155	0,507221784	0,585527393	0,464170364	-0,089932658	-0,076442759	-0,167147561	-0,244147561	**-1,504147561**
chrXVI	317763	317907	144	0,082637257	0,066705652	0,553339005	0,13410191	0,113986624	0,003337893	0,003337893	**-1,256662107**
chrXVI	318699	318846	147	0,031345166	0,044470435	0,413439528	-0,218706025	-0,185900121	-0,281030858	-0,302030858	**-1,562030858**
chrXVI	322025	322158	133	0,262159574	0,281646088	0,4820832	-0,044925865	-0,038186985	0,066638742	0,143638742	**-1,116361258**
chrXVI	323166	323321	155	0,208017923	0,218646305	0,487544793	-0,031225648	-0,0265418	-0,117678642	-0,194678642	**-1,454678642**
chrXVI	332594	332745	151	0,467327936	0,170470001	0,732720991	0,621063234	0,527903749	0,402555775	0,353555775	**-0,906444225**
chrXVI	332948	333099	151	0,421734966	0,329822392	0,561148077	0,153880643	0,130798547	0,024875371	-0,024124629	**-1,284124629**
chrXVI	336537	336643	106	0,359044634	2,849813706	0,111891706	-1,216529155	-1,034049782	-0,417968532	-0,151968532	**-1,411968532**
chrXVI	336537	336650	113	0,473027057	3,943045232	0,107114881	-1,242018432	-1,055715667	-0,252826184	-0,035826184	**-1,295826184**
chrXVI	341289	341447	158	0,624053768	0,470645436	0,570068714	0,176549108	0,150066742	0,124506631	0,026506631	**-1,233493369**
chrXVI	344751	344913	162	0,102584181	0,129705435	0,441621897	-0,146858392	-0,124829633	-0,056602772	-0,182602772	**-1,442602772**
chrXVI	344751	344913	162	0,102584181	0,129705435	0,441621897	-0,146858392	-0,124829633	-0,056602772	-0,182602772	**-1,442602772**
chrXVI	350439	350593	154	2,142869559	1,478641962	0,591705852	0,231935261	0,197144972	0,138858384	0,068858384	**-1,191141616**
chrXVI	351311	351471	160	0,558513874	0,770820872	0,420145397	-0,201521534	-0,171293304	-0,17899552	-0,29099552	**-1,55099552**
chrXVI	354968	355128	160	0,703841464	0,61146848	0,535114531	0,088133038	0,074913082	0,071589949	-0,040410051	**-1,300410051**
chrXVI	361977	362073	96	0,062690333	0,166764131	0,273214701	-0,603119188	-0,512651309	0,031229568	0,367229568	**-0,892770432**
chrXVI	364336	364488	152	0,923257629	0,722644568	0,56094319	0,153360973	0,130356827	0,043945004	-0,012054996	**-1,272054996**
chrXVI	366545	366652	107	0,299203861	1,560171093	0,160916366	-0,990698686	-0,842093883	-0,179861549	0,079138451	**-1,180861549**
chrXVI	366545	366653	108	0,364743755	2,364344791	0,133650392	-1,109300001	-0,942905001	-0,157589457	0,094410543	**-1,165589457**
chrXVI	367044	367180	136	0,595558162	0,541056958	0,523975224	0,060133194	0,051113215	-0,004608464	0,051391536	**-1,208608464**
chrXVI	370599	370734	135	6,032519755	4,947335887	0,549417037	0,124188628	0,105560334	0,092826834	0,155826834	**-1,104173166**
chrXVI	370905	371054	149	0,917558508	0,455821958	0,668102198	0,434678778	0,369476962	0,199444908	0,164444908	**-1,095555092**
chrXVI	373474	373616	142	0,19377012	0,118587827	0,620346375	0,30639062	0,260432027	0,153772514	0,167772514	**-1,092227486**
chrXVI	379313	379470	157	1,307948308	1,341524787	0,493663555	-0,015883779	-0,013501212	-0,073126068	-0,164126068	**-1,424126068**
chrXVI	379794	379947	153	1,541612276	0,922761525	0,625559432	0,320115026	0,272097772	0,207517019	0,144517019	**-1,115482981**
chrXVI	379982	380135	153	0,19661968	0,214940436	0,47774231	-0,055820732	-0,047447622	-0,105326443	-0,168326443	**-1,428326443**
chrXVI	379982	380135	153	0,19661968	0,214940436	0,47774231	-0,055820732	-0,047447622	-0,105326443	-0,168326443	**-1,428326443**
chrXVI	383644	383787	143	1,134125112	0,696703481	0,619460017	0,304062908	0,258453472	0,163666316	0,170666316	**-1,089333684**
chrXVI	385233	385386	153	0,723788388	0,463233697	0,609751408	0,278671178	0,236870501	0,17357967	0,11057967	**-1,14942033**
chrXVI	389294	389391	97	0,34194727	1,152525439	0,228807972	-0,742778252	-0,631361514	-0,079099859	0,249900141	**-1,010099859**
chrXVI	390081	390232	151	6,568237145	3,828163275	0,631779932	0,336571244	0,286085557	0,1806827	0,1316827	**-1,1283173**
chrXVI	390081	390232	151	6,568237145	3,828163275	0,631779932	0,336571244	0,286085557	0,1806827	0,1316827	**-1,1283173**
chrXVI	390545	390691	146	0,225115286	0,200116957	0,529393736	0,07374596	0,062684066	-0,047041103	-0,061041103	**-1,321041103**
chrXVI	392582	392731	149	0,045592969	0,048176305	0,486225044	-0,034535557	-0,029355224	-0,210449478	-0,245449478	**-1,505449478**
chrXVI	392582	392736	154	13,41858079	10,65066917	0,557498917	0,144631069	0,122936409	0,070140807	0,000140807	**-1,259859193**
chrXVI	394420	394573	153	0,133929347	0,166764131	0,44540157	-0,137288019	-0,116694816	-0,177244918	-0,240244918	**-1,500244918**
chrXVI	407155	407307	152	11,85417203	7,156034156	0,62356883	0,314867393	0,267637284	0,188118205	0,132118205	**-1,127881795**
chrXVI	407160	407307	147	0,037044288	0,037058696	0,499902783	-0,000243688	-0,000207135	-0,097323552	-0,118323552	**-1,378323552**
chrXVI	411818	411982	164	0,182371877	0,270528479	0,4026755	-0,246427915	-0,209463728	-0,080884868	-0,220884868	**-1,480884868**
chrXVI	411838	411991	153	0,039893848	0,062999783	0,387719315	-0,285268256	-0,242478018	-0,307554274	-0,370554274	**-1,630554274**
chrXVI	412957	413111	154	0,153876272	0,114881957	0,572545341	0,182858153	0,15542943	0,103556768	0,033556768	**-1,226443232**
chrXVI	415881	416027	146	0,062690333	0,051882174	0,547167331	0,118507771	0,100731606	-0,016705482	-0,030705482	**-1,290705482**
chrXVI	417433	417565	132	0,299203861	0,607762611	0,329895173	-0,440202642	-0,374172246	-0,23443892	-0,15043892	**-1,41043892**
chrXVI	418493	418641	148	0,686744101	0,622586089	0,524500318	0,061451844	0,052234068	-0,102537882	-0,130537882	**-1,390537882**
chrXVI	418678	418846	168	0,495823542	0,692997611	0,417071601	-0,209390764	-0,17798215	0,01831689	-0,14968311	**-1,40968311**
chrXVI	418678	418846	168	0,495823542	0,692997611	0,417071601	-0,209390764	-0,17798215	0,01831689	-0,14968311	**-1,40968311**
chrXVI	432250	432440	190	0,712390146	1,289642613	0,355833411	-0,369618423	-0,31417566	-0,113972439	-0,435972439	**-1,695972439**
chrXVI	432594	432737	143	1,929152515	1,059878699	0,645410629	0,372959291	0,317015397	0,219382274	0,226382274	**-1,033617726**
chrXVI	432908	433068	160	0,467327936	0,207528696	0,692484764	0,502905845	0,427469968	0,422488385	0,310488385	**-0,949511615**
chrXVI	433481	433638	157	0,282106498	0,222352175	0,559226183	0,149007602	0,126656462	0,079414991	-0,011585009	**-1,271585009**
chrXVI	437116	437278	162	0,159575393	0,163058261	0,49460244	-0,013530088	-0,011500575	0,07246741	-0,05353259	**-1,31353259**

Chrom	Start	End	Length	Section A	Section B	A/A+B	Z-score	Z * 0.85	Phase correction	Length correction	ΔLKnuc
chrXVI	439535	439688	153	0,128230226	0,370586958	0,257068582	-0,652409303	-0,554547908	-0,620673809	-0,683673809	**-1,943673809**
chrXVI	442027	442174	147	0,111132863	0,133411305	0,454449042	-0,114428548	-0,097264266	-0,199401127	-0,220401127	**-1,480401127**
chrXVI	442326	442474	148	0,256460453	0,259410871	0,497140355	-0,007168129	-0,00609291	-0,159406976	-0,187406976	**-1,447406976**
chrXVI	442340	442502	162	0,059840772	0,062999783	0,487141826	-0,032236246	-0,027400809	0,059165536	-0,066834464	**-1,326834464**
chrXVI	446281	446447	166	0,071239015	0,08894087	0,444743825	-0,138952542	-0,118109661	0,062858032	-0,091141968	**-1,351141968**
chrXVI	448877	449010	133	0,869115978	1,222936961	0,415436895	-0,213581034	-0,181543879	-0,090597369	-0,013597369	**-1,273597369**
chrXVI	454839	454945	106	0,119681545	0,274234349	0,303825122	-0,513430458	-0,436415889	0,182072443	0,448072443	**-0,811927557**
chrXVI	462929	463059	130	0,151026711	0,274234349	0,35513882	-0,371483236	-0,315760751	-0,13599693	-0,03799693	**-1,29799693**
chrXVI	465016	465172	156	0,327699467	0,26311674	0,554655514	0,137432464	0,116817594	0,046743756	-0,037256244	**-1,297256244**
chrXVI	466157	466315	158	1,134125112	1,096937395	0,508334082	0,020891966	0,017758171	0,005085613	-0,092914387	**-1,352914387**
chrXVI	474538	474690	152	0,914708947	0,555880437	0,622001598	0,31074195	0,264130657	0,206252996	0,150252996	**-1,109747004**
chrXVI	484126	484288	162	0,062690333	0,081529131	0,434687048	-0,164453569	-0,139785534	-0,06068286	-0,18668286	**-1,44668286**
chrXVI	484126	484288	162	0,062690333	0,081529131	0,434687048	-0,164453569	-0,139785534	-0,06068286	-0,18668286	**-1,44668286**
chrXVI	484807	484963	156	0,612655525	0,596645002	0,506619745	0,016594003	0,014104902	-0,047719747	-0,131719747	**-1,391719747**
chrXVI	487013	487184	171	0,071239015	0,196411088	0,26616472	-0,624454047	-0,53078594	-0,323409896	-0,512409896	**-1,772409896**
chrXVI	493217	493377	160	0,364743755	0,314998914	0,536590936	0,091848853	0,078071525	0,070304637	-0,041695363	**-1,301695363**
chrXVI	497274	497428	154	1,193965884	0,763409133	0,609983204	0,279275258	0,23738397	0,163330908	0,093330908	**-1,166669092**
chrXVI	501076	501229	153	0,088336378	0,085235	0,508934013	0,022396121	0,019036703	-0,061027529	-0,124027529	**-1,384027529**
chrXVI	501841	501999	158	0,216566604	0,203822827	0,51515711	0,038002385	0,032302027	0,02105665	-0,07694335	**-1,33694335**
chrXVI	505233	505380	147	1,085682582	1,156231309	0,48426596	-0,03944962	-0,033532177	-0,152088424	-0,173088424	**-1,433088424**
chrXVI	506320	506478	158	0,076938136	0,081529131	0,485514374	-0,036318061	-0,030870352	-0,048132268	-0,146132268	**-1,406132268**
chrXVI	506594	506750	156	0,344796831	0,281646088	0,550404227	0,126682692	0,107680288	0,036341015	-0,047658985	**-1,307658985**
chrXVI	509864	510024	160	0,094035499	0,155646522	0,376621026	-0,314367347	-0,267212245	-0,278989859	-0,390989859	**-1,650989859**
chrXVI	511391	511535	144	0,042743409	0,107470218	0,284550808	-0,569375094	-0,48396883	-0,59231565	-0,59231565	**-1,85231565**
chrXVI	517443	517595	152	0,273557816	0,222352175	0,551627959	0,129775456	0,110309137	0,060769391	0,004769391	**-1,255230609**
chrXVI	519049	519173	124	0,133929347	0,566998046	0,191074495	-0,873943563	-0,742852028	-0,173611838	-0,033611838	**-1,293611838**
chrXVI	520082	520209	127	0,165274514	0,296469566	0,357935317	-0,363983093	-0,309385629	0,090349118	0,209349118	**-1,050650882**
chrXVI	522508	522654	146	1,005894886	0,959820221	0,511719568	0,029380828	0,024973704	-0,098160162	-0,112160162	**-1,372160162**
chrXVI	522574	522734	160	3,37103017	3,494635013	0,490998335	-0,022565744	-0,019180883	-0,023786133	-0,135786133	**-1,395786133**
chrXVI	525954	526108	154	0,082637257	0,111176087	0,426375476	-0,185609565	-0,15776813	-0,22572356	-0,29572356	**-1,55572356**
chrXVI	536923	537076	153	0,453080133	0,329822392	0,578718446	0,198616037	0,168823631	0,098347265	0,035347265	**-1,224652735**
chrXVI	538001	538163	162	0,131079787	0,214940436	0,378821174	-0,308578276	-0,262291535	-0,17921883	-0,30521883	**-1,56521883**
chrXVI	541695	541831	136	0,963151477	0,566998046	0,629449255	0,330395039	0,280835783	0,224662772	0,280662772	**-0,979337228**
chrXVI	547021	547160	139	2,866657947	2,056757616	0,582249845	0,207652497	0,176504623	0,086538654	0,121538654	**-1,138461346**
chrXVI	547636	547771	135	0,837770812	0,822703046	0,504537188	0,01137329	0,009667296	-0,013576363	0,049423637	**-1,210576363**
chrXVI	547851	548005	154	0,681044979	0,485468915	0,583829291	0,211699576	0,17994464	0,11914273	0,04914273	**-1,21085727**
chrXVI	550617	550788	171	0,039893848	0,051882174	0,434687048	-0,164453569	-0,139785534	0,071112288	-0,117887712	**-1,377887712**
chrXVI	553457	553564	107	0,854868175	5,8960385	0,126630128	-1,142466277	-0,971096336	-0,303362682	-0,044362682	**-1,304362682**
chrXVI	553457	553565	108	0,983098401	8,61614677	0,102414136	-1,267915097	-1,077727832	-0,268748346	-0,016748346	**-1,276748346**
chrXVI	560771	560926	155	0,413186285	0,237175653	0,635317445	0,345970201	0,294074671	0,208028222	0,131028222	**-1,128971778**
chrXVI	560771	560926	155	0,413186285	0,237175653	0,635317445	0,345970201	0,294074671	0,208028222	0,131028222	**-1,128971778**
chrXVI	564313	564461	148	0,068389454	0,107470218	0,38888651	-0,282222352	-0,239889	-0,394215094	-0,422215094	**-1,682215094**
chrXVI	564317	564438	121	1,082833022	3,583575882	0,232048465	-0,732117375	-0,622299769	0,012372237	0,173372237	**-1,086627763**
chrXVI	566468	566620	152	0,04844253	0,051882174	0,482857442	-0,042983253	-0,036535765	-0,095216924	-0,151216924	**-1,411216924**
chrXVI	566468	566620	152	0,04844253	0,051882174	0,482857442	-0,042983253	-0,036535765	-0,095216924	-0,151216924	**-1,411216924**
chrXVI	568077	568211	134	0,24506221	0,270528479	0,475303793	-0,061943802	-0,052652332	-0,01877957	0,05122043	**-1,20877957**
chrXVI	573444	573609	165	0,079787696	0,155646522	0,338895921	-0,415478239	-0,353156503	-0,19920184	-0,34620184	**-1,60620184**
chrXVI	573688	573839	151	0,532867829	0,318704784	0,625745616	0,320606293	0,272515349	0,164946553	0,115946553	**-1,144053447**
chrXVI	578898	579048	150	0,102584181	0,044470435	0,69759239	0,517488464	0,439865194	0,284575145	0,242575145	**-1,017424855**
chrXVI	580106	580256	150	0,578460798	0,452116089	0,561298051	0,154261063	0,131121903	-0,028128816	-0,070128816	**-1,330128816**
chrXVI	581758	581907	149	0,136778908	0,140823044	0,492715945	-0,018259434	-0,015520519	-0,193594746	-0,228594746	**-1,488594746**
chrXVI	581758	581907	149	0,136778908	0,140823044	0,492715945	-0,018259434	-0,015520519	-0,193594746	-0,228594746	**-1,488594746**
chrXVI	582967	583116	149	0,19377012	0,129705435	0,599025542	0,250825642	0,213201796	0,042621237	0,007621237	**-1,252378763**
chrXVI	584868	585014	146	4,174606255	3,187047838	0,567074492	0,168930872	0,143591241	0,023436187	0,009436187	**-1,250563813**
chrXVI	591560	591713	153	1,655594699	1,122878482	0,59586492	0,24265824	0,206259504	0,143720795	0,080720795	**-1,179279205**
chrXVI	592210	592352	142	0,233663968	0,163058261	0,588986325	0,224938202	0,191197471	0,104031949	0,118031949	**-1,141968051**
chrXVI	593528	593683	155	0,119681545	0,103764348	0,535617563	0,08939893	0,07598909	-0,000265577	-0,077265577	**-1,337265577**
chrXVI	594996	595138	142	0,706691025	0,563292176	0,556456986	0,141992377	0,12069352	0,034375069	0,048375069	**-1,211624931**
chrXVI	599046	599200	154	1,550160957	1,489759571	0,509934698	0,02490517	0,021169395	-0,039215328	-0,109215328	**-1,369215328**
chrXVI	600831	600997	166	0,227964847	0,281646088	0,447331152	-0,132407093	-0,112546029	0,064655484	-0,089344516	**-1,349344516**
chrXVI	600831	600997	166	0,227964847	0,281646088	0,447331152	-0,132407093	-0,112546029	0,064655484	-0,089344516	**-1,349344516**
chrXVI	601108	601231	123	0,159575393	0,444704349	0,264075364	-0,630831465	-0,536206745	0,040828219	0,187828219	**-1,072171781**
chrXVI	603139	603315	176	0,065539893	0,155646522	0,296310663	-0,535041262	-0,454785073	-0,232632526	-0,456632526	**-1,716632526**
chrXVI	604400	604533	133	0,598407723	0,822703046	0,421084503	-0,199119834	-0,169251859	-0,093190771	-0,016190771	**-1,276190771**
chrXVI	611112	611263	151	0,156725832	0,044470435	0,778969881	0,768718839	0,653411013	0,545270986	0,496270986	**-0,763729014**
chrXVI	611769	611929	160	0,256460453	0,300175436	0,460732874	-0,09858756	-0,083799426	-0,081074942	-0,193074942	**-1,453074942**
chrXVI	611769	611929	160	0,256460453	0,300175436	0,460732874	-0,09858756	-0,083799426	-0,081074942	-0,193074942	**-1,453074942**
chrXVI	614264	614371	107	0,056991212	0,407645654	0,122657533	-1,161804056	-0,987533448	-0,319799794	-0,060799794	**-1,320799794**
chrXVI	614264	614381	117	0,039893848	0,500292393	0,073852026	-1,447688987	-1,230535639	-0,505300435	-0,316300435	**-1,576300435**
chrXVI	616962	617122	160	0,464478375	0,570703915	0,448692351	-0,128965812	-0,10962094	-0,099539962	-0,211539962	**-1,471539962**
chrXVI	617750	617867	117	0,92610719	4,39516132	0,174038801	-0,938324639	-0,797575943	-0,084317306	0,104682694	**-1,155317306**
chrXVI	617888	618049	161	0,29065518	0,296469566	0,495048423	-0,012412082	-0,010550269	0,049198185	-0,069801815	**-1,329801815**
chrXVI	623817	623987	170	0,276407377	0,26311674	0,512316999	0,030879046	0,026247189	0,225770625	0,043770625	**-1,216229375**
chrXVI	627166	627316	150	0,068389454	0,062999783	0,520510322	0,051434423	0,043719259	-0,119535304	-0,161535304	**-1,421535304**
chrXVI	630544	630672	128	0,598407723	0,863467612	0,409342513	-0,229236602	-0,194851112	0,129090979	0,241090979	**-1,018909021**
chrXVI	630787	630947	160	1,698338108	1,23405457	0,579164626	0,199756852	0,169793324	0,185555735	0,073555735	**-1,186444265**
chrXVI	639745	639881	136	0,131079787	0,229763914	0,36325918	-0,349760614	-0,297296522	-0,351834519	-0,295834519	**-1,555834519**
chrXVI	644095	644255	160	0,313451664	0,159352392	0,662963145	0,420563695	0,357479141	0,37219519	0,26019519	**-0,99980481**
chrXVI	647938	648089	151	0,410336724	0,489174784	0,456177292	-0,110069087	-0,093558724	-0,210657531	-0,259657531	**-1,519657531**
chrXVI	651006	651170	164	0,339097709	0,407645654	0,454102073	-0,115304027	-0,098008423	0,03365597	-0,10634403	**-1,36634403**
chrXVI	651448	651577	129	1,95479856	1,952993268	0,500230986	0,000578997	0,000492147	0,254196828	0,359196828	**-0,900803172**
chrXVI	651814	651966	152	0,421734966	0,329822392	0,561148077	0,153880643	0,130798547	0,063436251	0,007436251	**-1,252563749**
chrXVI	655669	655818	149	0,059840772	0,037058696	0,617555219	0,299066166	0,254206241	0,088106798	0,053106798	**-1,206893202**
chrXVI	661336	661465	129	6,830396719	10,99531504	0,383176661	-0,297148258	-0,252576019	-0,010643834	0,094356166	**-1,165643834**
chrXVI	664409	664571	162	0,102584181	0,118587827	0,463820816	-0,090812431	-0,077190567	0,008108976	-0,117891024	**-1,377891024**
chrXVI	665020	665144	124	0,273557816	1,000584786	0,214699529	-0,790220412	-0,67168735	-0,128296211	0,011703789	**-1,248296211**
chrXVI	670319	670426	107	0,165274514	1,719523484	0,087688184	-1,35512929	-1,151859897	-0,463598676	-0,204598676	**-1,464598676**
chrXVI	670319	670427	108	0,156725832	2,127169138	0,068622171	-1,486131496	-1,263211772	-0,425003482	-0,173003482	**-1,433003482**
chrXVI	670635	670782	147	0,256460453	0,148234783	0,633712557	0,341702373	0,290447017	0,163258251	0,142258251	**-1,117741749**
chrXVI	672000	672158	158	0,549965193	0,518821741	0,514569532	0,036528524	0,031049245	0,015474477	-0,082525523	**-1,342525523**
chrXVI	678051	678201	150	5,525297971	3,483517404	0,613321257	0,28798596	0,244788066	0,073993351	0,031993351	**-1,228006649**
chrXVI	679094	679205	111	7,468698289	14,20459809	0,344603708	-0,399930896	-0,339941262	0,464294236	0,695294236	**-0,564705764**
chrXVI	679094	679206	112	0,182371877	0,34835174	0,34362872	-0,402579708	-0,342192752	0,461104681	0,685104681	**-0,574895319**
chrXVI	679094	679207	113	0,108283302	0,35205761	0,235224155	-0,721749813	-0,613487341	0,181783152	0,398783152	**-0,861216848**
chrXVI	679098	679205	107	0,165274514	0,870879351	0,159507694	-0,996483277	-0,847010785	-0,143856442	0,115143558	**-1,144856442**
chrXVI	679098	679220	122	0,378991558	1,700994137	0,182208733	-0,906979826	-0,770932852	-0,128831814	0,025168186	**-1,234831814**

Chrom	Start	End	Length	Section A	Section B	A/A+B	Z-score	Z * 0.85	Phase correction	Length correction	ΔLKnuc
chrXVI	681181	681332	151	0,113982423	0,240881523	0,321200349	-0,464344846	-0,394693119	-0,512606949	-0,561606949	**-1,821606949**
chrXVI	685561	685687	126	0,045592969	0,207528696	0,180122745	-0,914897409	-0,777662797	-0,32376229	-0,19776229	**-1,45776229**
chrXVI	685866	686024	158	0,595558162	0,581821524	0,505833563	0,014623096	0,012429632	-0,004667769	-0,102667769	**-1,362667769**
chrXVI	689048	689183	135	0,108283302	0,159352392	0,404592155	-0,241478459	-0,20525669	-0,226976798	-0,163976798	**-1,423976798**
chrXVI	695989	696136	147	1,410532489	0,926467395	0,603565494	0,262586882	0,223198849	0,072951441	0,051951441	**-1,208048559**
chrXVI	697045	697195	150	0,208017923	0,248293262	0,45586856	-0,110847699	-0,094220544	-0,266498098	-0,308498098	**-1,568498098**
chrXVI	702075	702202	127	0,455929693	1,019114134	0,309095693	-0,498415257	-0,423652969	-0,042902945	0,076097055	**-1,183902945**
chrXVI	706433	706576	143	0,421734966	0,270528479	0,609211665	0,277264951	0,235675208	0,129757457	0,136757457	**-1,123242543**
chrXVI	719549	719656	107	0,028495606	0,144528914	0,164691143	-0,975358855	-0,829055027	-0,104596891	0,154403109	**-1,105596891**
chrXVI	719549	719694	145	13,29889924	7,856443507	0,628630762	0,328229063	0,278994703	0,174476475	0,167476475	**-1,092523525**
chrXVI	721766	721909	143	1,535913155	1,608347397	0,488481514	-0,028876575	-0,024545089	-0,139070623	-0,132070623	**-1,392070623**
chrXVI	722565	722701	136	0,082637257	0,118587827	0,410670755	-0,225819886	-0,191946903	-0,250055797	-0,194055797	**-1,454055797**
chrXVI	726383	726544	161	0,113982423	0,107470218	0,514703382	0,036864261	0,031334622	0,093453914	-0,025546086	**-1,285546086**
chrXVI	726865	727012	147	0,344796831	0,211234566	0,620103168	0,305751756	0,259888992	0,10306677	0,08206677	**-1,17793323**
chrXVI	730093	730221	128	0,621204207	0,79305609	0,439243192	-0,152888273	-0,129955032	0,170166285	0,282166285	**-0,977833715**
chrXVI	732571	732734	163	0,792177842	0,770820872	0,50683205	0,017126246	0,014557309	0,129947753	-0,003052247	**-1,263052247**
chrXVI	733908	734067	159	0,056991212	0,055588044	0,506231912	0,015621722	0,013278464	0,008775763	-0,096224237	**-1,356224237**
chrXVI	734318	734467	149	5,872944363	3,194459577	0,647698548	0,379114412	0,32224725	0,165601612	0,130601612	**-1,129398388**
chrXVI	735641	735790	149	0,612655525	0,652233046	0,484355333	-0,039225421	-0,033341608	-0,187353676	-0,222353676	**-1,482353676**
chrXVI	735832	735978	146	1,689789426	1,059878699	0,614543046	0,291179674	0,247502723	0,127043061	0,113043061	**-1,146956939**
chrXVI	736889	737030	141	0,484425299	0,281646088	0,632350075	0,33808405	0,287371443	0,17399213	0,19499213	**-1,06500787**
chrXVI	737626	737736	110	0,87766466	8,834793075	0,090364837	-1,338511746	-1,137734984	-0,316392997	-0,078392997	**-1,338392997**
chrXVI	739761	739921	160	0,170973635	0,188999349	0,474962408	-0,062801193	-0,053381014	-0,035490435	-0,147490435	**-1,407490435**
chrXVI	740972	741124	152	1,635647775	1,600935658	0,505362463	0,013442106	0,01142579	-0,063536127	-0,119536127	**-1,379536127**
chrXVI	742321	742473	152	0,615505086	0,444704349	0,58055047	0,203301913	0,172806626	0,096587429	0,040587429	**-1,219412571**
chrXVI	745613	745766	153	0,136778908	0,196411088	0,41051325	-0,226224907	-0,192291171	-0,259380352	-0,322380352	**-1,582380352**
chrXVI	747443	747595	152	0,43883233	0,633703698	0,409153929	-0,229721923	-0,195263635	-0,262364658	-0,318364658	**-1,578364658**
chrXVI	748166	748325	159	0,062690333	0,114881957	0,353041192	-0,377122752	-0,320554339	-0,321567836	-0,426567836	**-1,686567836**
chrXVI	751513	751665	152	0,165274514	0,17417587	0,486888575	-0,032871387	-0,027940679	-0,074822849	-0,130822849	**-1,390822849**
chrXVI	753264	753415	151	2,430675178	2,757166966	0,468532987	-0,078958069	-0,067114359	-0,183754888	-0,232754888	**-1,492754888**
chrXVI	753264	753415	151	2,430675178	2,757166966	0,468532987	-0,078958069	-0,067114359	-0,183754888	-0,232754888	**-1,492754888**
chrXVI	753454	753608	154	0,484425299	0,337234132	0,589569451	0,226437584	0,192471947	0,129701139	0,059701139	**-1,200298861**
chrXVI	759400	759547	147	0,510071344	0,34835174	0,594195745	0,238351462	0,202598743	0,055028974	0,034028974	**-1,225971026**
chrXVI	761447	761589	142	0,188070999	0,125999566	0,598817654	0,250287928	0,212744739	0,119764611	0,133764611	**-1,126235389**
chrXVI	761447	761589	142	0,188070999	0,125999566	0,598817654	0,250287928	0,212744739	0,119764611	0,133764611	**-1,126235389**
chrXVI	763063	763215	152	0,111132863	0,085235	0,565942212	0,166052574	0,141144688	0,098397692	0,042397692	**-1,217602308**
chrXVI	763430	763578	148	0,230814407	0,170470001	0,575189075	0,18960094	0,161160799	0,011122545	-0,016877455	**-1,276877455**
chrXVI	764397	764544	147	0,04844253	0,059293913	0,449639217	-0,126572917	-0,10758698	-0,254365453	-0,275365453	**-1,535365453**
chrXVI	764502	764659	157	0,29065518	0,429880871	0,403387421	-0,244588792	-0,207900474	-0,255737048	-0,346737048	**-1,606737048**
chrXVI	770323	770455	132	1,07428434	1,007996525	0,515917117	0,039908886	0,033922553	0,168242995	0,252242995	**-1,007757005**
chrXVI	778559	778687	128	0,045592969	0,233469783	0,163378913	-0,980665288	-0,833565495	-0,547727508	-0,435727508	**-1,695727508**
chrXVI	786310	786475	165	0,056991212	0,070411522	0,447331152	-0,132407093	-0,112546029	0,048821568	-0,098178432	**-1,358178432**
chrXVI	788541	788654	113	0,059840772	0,233469783	0,204018475	-0,827353109	-0,703250143	0,085571326	0,302571326	**-0,957428674**
chrXVI	788648	788776	128	1,256656217	1,334113048	0,485051384	-0,037479397	-0,031857487	0,235883302	0,347883302	**-0,912116698**
chrXVI	790762	790920	158	0,045592969	0,08894087	0,338895921	-0,415478239	-0,353156503	-0,359619091	-0,457619091	**-1,717619091**
chrXVI	791267	791414	147	0,062690333	0,062999783	0,498768996	-0,003085674	-0,002622823	-0,154177715	-0,175177715	**-1,435177715**
chrXVI	791438	791595	157	0,105433742	0,070411522	0,599582493	0,252266586	0,214426598	0,176810331	0,085810331	**-1,174189669**
chrXVI	791737	791890	153	1,376337762	1,015408265	0,57545314	0,190274903	0,161733667	0,088585168	0,025585168	**-1,234414832**
chrXVI	792774	792924	150	0,44168189	0,381704567	0,536421126	0,091421413	0,077708201	-0,104549472	-0,146549472	**-1,406549472**
chrXVI	794619	794743	124	0,039893848	0,122293696	0,245973563	-0,687215201	-0,584132921	-0,059240655	0,080759345	**-1,179240655**
chrXVI	796842	796998	156	0,532867829	0,392822175	0,575643927	0,190761892	0,162147608	0,085623064	0,001623064	**-1,258376936**
chrXVI	798394	798554	160	0,142478029	0,103764348	0,578608892	0,198335964	0,168585569	0,185340456	0,073340456	**-1,186659544**
chrXVI	798628	798777	149	0,162424953	0,114881957	0,585722704	0,216555726	0,184072367	0,031006243	-0,003993757	**-1,263993757**
chrXVI	805070	805217	147	0,065539893	0,040764565	0,616530051	0,29638	0,251923	0,092668367	0,071668367	**-1,188331633**
chrXVI	805577	805691	114	0,045592969	0,459527828	0,090261517	-1,339146351	-1,138274398	-0,370834688	-0,160834688	**-1,420834688**
chrXVI	807669	807826	157	0,054141651	0,062999783	0,462190442	-0,094916836	-0,080679311	-0,115924315	-0,206924315	**-1,466924315**
chrXVI	808317	808452	135	0,091185939	0,077823261	0,539532397	0,099255756	0,084367393	0,065012764	0,128012764	**-1,131987236**
chrXVI	809355	809495	140	0,151026711	0,125999566	0,545171067	0,113470102	0,096449587	0,0131766	0,0411766	**-1,2188234**
chrXVI	809361	809495	134	0,122531105	0,092646739	0,569441084	0,174951387	0,148708679	0,176072376	0,246072376	**-1,013927624**
chrXVI	809773	809928	155	0,108283302	0,111176087	0,493409293	-0,016521205	-0,014043024	-0,080064852	-0,157064852	**-1,417064852**
chrXVI	809904	810012	108	0,074088575	0,200116957	0,270193583	-0,612227627	-0,520393483	0,327779192	0,579779192	**-0,680220808**
chrXVI	809934	810052	118	1,595753927	4,713866104	0,252908086	-0,665366397	-0,565561437	0,093085278	0,275085278	**-0,984914722**
chrXVI	810053	810202	149	0,068389454	0,059293913	0,535617563	0,08939893	0,07598909	-0,087210704	-0,122210704	**-1,382210704**
chrXVI	813679	813806	127	1,205364127	1,678758919	0,417930895	-0,207189598	-0,176111158	0,188271248	0,307271248	**-0,952728752**
chrXVI	822438	822593	155	0,558513874	0,629997828	0,469927114	-0,07545308	-0,064135118	-0,140588491	-0,217588491	**-1,477588491**
chrXVI	822870	822974	104	0,082637257	0,592939133	0,122321115	-1,163461699	-0,988942444	-0,418798499	-0,138798499	**-1,398798499**
chrXVI	827045	827170	125	0,527168708	1,100643265	0,323851106	-0,456956639	-0,388413143	0,110990494	0,243990494	**-1,016009506**
chrXVI	827247	827389	142	0,173823196	0,281646088	0,381635385	-0,301188484	-0,256010211	-0,349691954	-0,335691954	**-1,595691954**
chrXVI	827869	828021	152	0,29350474	0,322410653	0,476534185	-0,058854034	-0,050025929	-0,09315842	-0,14915842	**-1,40915842**
chrXVI	830012	830156	144	2,841011902	1,767699789	0,616443833	0,296154188	0,25173106	0,153000132	0,153000132	**-1,106999868**
chrXVI	839224	839390	166	0,330549028	0,515115871	0,390874716	-0,277039947	-0,235483955	-0,06355392	-0,21755392	**-1,47755392**
chrXVI	841656	841805	149	1,256656217	0,767115003	0,620947766	0,307970909	0,261775272	0,09098338	0,05598338	**-1,20401662**
chrXVI	843827	843986	159	0,173823196	0,181587609	0,489076846	-0,027383708	-0,023276152	-0,028427659	-0,133427659	**-1,393427659**
chrXVI	844949	845072	123	0,056991212	0,226058044	0,201347329	-0,836818391	-0,711295633	-0,136444799	0,010555201	**-1,249444799**
chrXVI	845049	845204	155	0,111132863	0,059293913	0,652085695	0,390957556	0,332313923	0,256524069	0,179524069	**-1,080475931**
chrXVI	845482	845616	134	0,156725832	0,155646522	0,501727601	0,004330468	0,003680898	0,010069614	0,080069614	**-1,179930386**
chrXVI	845482	845622	140	0,131079787	0,125999566	0,509880648	0,024769644	0,021054198	-0,068049783	-0,040049783	**-1,300049783**
chrXVI	846525	846660	135	0,082637257	0,066705652	0,553339005	0,13410191	0,113986624	0,080865073	0,143865073	**-1,116134927**
chrXVI	847154	847311	157	0,042743409	0,066705652	0,390532439	-0,277931585	-0,236241848	-0,274023224	-0,365023224	**-1,625023224**
chrXVI	848557	848707	150	0,453080133	0,44099848	0,506756482	0,0169368	0,01439628	-0,157466244	-0,199466244	**-1,459466244**
chrXVI	849289	849403	114	0,031345166	0,259410871	0,107805729	-1,238282217	-1,052539834	-0,300078132	-0,090078132	**-1,350078132**
chrXVI	851012	851164	152	0,028495606	0,037058696	0,434687048	-0,164453569	-0,139785534	-0,184339674	-0,240339674	**-1,500339674**
chrXVI	851565	851679	114	0,062690333	0,400233914	0,135422444	-1,101118957	-0,935951113	-0,20898221	0,00101779	**-1,25898221**
chrXVI	853843	853993	150	8,69115978	4,076456536	0,68071906	0,46971048	0,399253908	0,236425172	0,194425172	**-1,065574828**
chrXVI	853847	853993	146	0,065539893	0,037058696	0,638799168	0,355250862	0,301963233	0,198152147	0,184152147	**-1,075847853**
chrXVI	854031	854169	138	0,424584527	0,515115871	0,451829676	-0,12104	-0,102884	-0,174142968	-0,132142968	**-1,392142968**
chrXVI	854126	854258	132	0,236513528	0,251999131	0,484150254	-0,039739878	-0,033778897	0,07825031	0,16225031	**-1,09774969**
chrXVI	854305	854440	135	0,062690333	0,070411522	0,470995186	-0,072768458	-0,061853189	-0,095205428	-0,032205428	**-1,292205428**
chrXVI	854851	855005	154	0,216566604	0,144528914	0,599748802	0,252696962	0,214792418	0,138957211	0,068957211	**-1,191042789**
chrXVI	855108	855259	151	0,054141651	0,074117392	0,42212736	-0,196454145	-0,166986023	-0,276915168	-0,325915168	**-1,585915168**
chrXVI	855132	855277	145	0,113982423	0,074117392	0,605967759	0,268824834	0,228501109	0,124146142	0,117146142	**-1,142853858**
chrXVI	855136	855287	151	0,102584181	0,081529131	0,557179598	0,143822286	0,122248943	0,013629026	-0,035370974	**-1,295370974**
chrXVI	855343	855483	140	0,216566604	0,107470218	0,66833949	0,435332606	0,370032715	0,287530242	0,315530242	**-0,944469758**
chrXVI	855349	855483	134	0,14817715	0,129705435	0,533236547	0,083408278	0,070897036	0,080534998	0,150534998	**-1,109465002**
chrXVI	855892	856000	108	0,065539893	0,192705218	0,253789483	-0,662612181	-0,563220354	0,290819375	0,542819375	**-0,717180625**
chrXVI	856112	856214	102	0,68389454	5,45504002	0,111402807	-1,219101651	-1,036236403	-0,484300807	-0,190300807	**-1,450300807**

DATA 2

Chrom	Start	End	Length	Section A	Section B	A/A+B	Z-score	Z * 0.85	Phase correction	Length correction	ΔLKnuc
chrXVI	858667	858810	143	1,986143727	2,160521964	0,47897368	-0,052729592	-0,044820154	-0,156500174	-0,149500174	**-1,409500174**
chrXVI	863879	864033	154	0,404637603	0,203822827	0,665018764	0,426199513	0,362269586	0,274700415	0,204700415	**-1,055299585**
chrXVI	863879	864033	154	0,404637603	0,203822827	0,665018764	0,426199513	0,362269586	0,274700415	0,204700415	**-1,055299585**
chrXVI	864255	864385	130	2,416427375	3,909692405	0,381976229	-0,300294593	-0,255250404	-0,090558504	0,007441496	**-1,252558504**
chrXVI	867145	867288	143	0,424584527	0,307587175	0,579897483	0,201631223	0,171386539	0,058266981	0,065266981	**-1,194733019**
chrXVI	868632	868780	148	0,222265726	0,137117174	0,618464945	0,301451656	0,256233907	0,107200352	0,079200352	**-1,180799648**
chrXVI	888008	888137	129	1,473222822	1,838111311	0,4449031	-0,138549438	-0,117767022	0,108987568	0,213987568	**-1,046012432**
chrXVI	891517	891679	162	0,091185939	0,081529131	0,527955893	0,070132481	0,059612609	0,149207772	0,023207772	**-1,236792228**
chrXVI	893449	893599	150	0,430283648	0,381704567	0,529913662	0,075052832	0,063794907	-0,092754161	-0,134754161	**-1,394754161**
chrXVI	897488	897648	160	0,054141651	0,08894087	0,378394585	-0,309699913	-0,263244926	-0,239825204	-0,351825204	**-1,611825204**
chrXVI	897990	898142	152	0,530018269	0,366881088	0,590945087	0,229976764	0,195480249	0,159760579	0,103760579	**-1,156239421**
chrXVI	901200	901349	149	0,319150785	0,214940436	0,597558568	0,24703277	0,209977854	0,038574896	0,003574896	**-1,256425104**
chrXVI	905702	905842	140	0,68389454	0,70411522	0,492715945	-0,018259434	-0,015520519	-0,104380245	-0,076380245	**-1,336380245**
chrXVI	910317	910457	140	0,344796831	0,244587392	0,585011979	0,214732295	0,182522451	0,077125233	0,105125233	**-1,154874767**
chrXVI	910317	910457	140	0,344796831	0,244587392	0,585011979	0,214732295	0,182522451	0,077125233	0,105125233	**-1,154874767**
chrXVI	922519	922676	157	0,045592969	0,062999783	0,419852784	-0,202270107	-0,171929591	-0,213045849	-0,304045849	**-1,564045849**
chrXVI	922519	922681	162	7,246432564	8,656911336	0,455654648	-0,111387218	-0,094679135	-0,001495318	-0,127495318	**-1,387495318**
chrXVI	922523	922681	158	0,045592969	0,059293913	0,434687048	-0,164453569	-0,139785534	-0,145272644	-0,243272644	**-1,503272644**
chrXVI	927439	927574	135	0,088336378	0,055588044	0,613769206	0,28915654	0,245783059	0,209175108	0,272175108	**-0,987824892**
chrXVI	930833	931008	175	0,051292091	0,129705435	0,283385589	-0,572813203	-0,486891222	-0,269019507	-0,486019507	**-1,746019507**
chrXVI	931493	931642	149	0,569912117	0,522527611	0,521687469	0,054389227	0,046230843	-0,126005016	-0,161005016	**-1,421005016**
chrXVI	940363	940515	152	0,569912117	0,485468915	0,540006026	0,100448902	0,085381566	0,034520162	-0,021479838	**-1,281479838**
chrXVI	943076	943229	153	0,495823542	0,607762611	0,449283946	-0,127470662	-0,108350062	-0,175434548	-0,238434548	**-1,498434548**
chrXVI	943085	943229	144	0,034194727	0,085235	0,286316713	-0,564177371	-0,479550765	-0,573880799	-0,573880799	**-1,833880799**
chrXVI	944252	944389	137	0,544266071	0,559586306	0,49306056	-0,017395474	-0,014786153	-0,093987783	-0,044987783	**-1,304987783**
chrXVI	944537	944680	143	0,404637603	0,418763262	0,491422368	-0,021502592	-0,018277203	-0,129609864	-0,122609864	**-1,382609864**
chrXVI	944585	944710	125	0,199469241	0,255705001	0,438226118	-0,155468183	-0,132147955	0,338711742	0,471711742	**-0,788288258**
chrXVI	944810	944962	152	0,102584181	0,077823261	0,568624996	0,172874585	0,146943398	0,103055269	0,047055269	**-1,212944731**
chrXVI	944883	945027	144	0,062690333	0,085235	0,423797138	-0,192188854	-0,163360526	-0,258440935	-0,258440935	**-1,518440935**
chrXVI	944980	945115	135	1,156921597	0,837526525	0,58007104	0,202075221	0,171763938	0,133674957	0,196674957	**-1,063325043**
chrXVI	944980	945167	187	0,111132863	0,070411522	0,612152576	0,284933816	0,242193743	0,438585782	0,137585782	**-1,122414218**
chrXVI	945023	945115	92	0,151026711	0,122293696	0,552562879	0,132139134	0,112318264	0,601709995	0,965709995	**-0,294290005**
chrXVI	945023	945167	144	1,26805446	0,592939133	0,68138572	0,471577263	0,400840674	0,304960231	0,304960231	**-0,955039769**
chrXVI	945362	945514	152	0,188070999	0,144528914	0,56545715	0,164819952	0,140096959	0,089005946	0,033005946	**-1,226994054**
chrXVI	945663	945793	130	0,339097709	0,366881088	0,480322795	-0,049343454	-0,041941936	0,105507633	0,203507633	**-1,056492367**
chrXVI	945663	945793	130	0,339097709	0,366881088	0,480322795	-0,049343454	-0,041941936	0,105507633	0,203507633	**-1,056492367**
chrXVI	945759	945954	195	0,079787696	0,118587827	0,402205349	-0,247642923	-0,210496484	-0,011973299	-0,368973299	**-1,628973299**
chrXVI	945821	945954	133	12,97119978	9,520378947	0,57671362	0,193493162	0,164469188	0,239112924	0,316112924	**-0,943887076**
chrXVI	945821	945969	148	0,666797177	0,555880437	0,54535813	0,113942039	0,096850733	-0,05334048	-0,08134048	**-1,34134048**
chrXVI	946033	946184	151	0,478726178	0,296469566	0,617555219	0,299066166	0,254206241	0,145906245	0,096906245	**-1,163093755**
chrXVI	946038	946172	134	0,581310359	0,429880871	0,574876781	0,188803997	0,160483398	0,160447619	0,230447619	**-1,029552381**
chrXVI	946051	946147	96	0,056991212	0,140823044	0,288104674	-0,558930214	-0,475090682	0,068394864	0,404394864	**-0,855605136**
chrXVI	946159	946281	122	0,125380666	0,070411522	0,640376244	0,359464657	0,305544959	0,923520722	1,077520722	**-0,182479278**
chrXVI	946966	947118	152	0,071239015	0,048176305	0,596565123	0,24446623	0,207796296	0,160702149	0,104702149	**-1,155297851**
chrXVI	947306	947457	151	0,524319147	0,370586958	0,585892916	0,216992528	0,184443648	0,088263992	0,039263992	**-1,220736008**
