# Supplementary material for: Nucleosomal DNA has topological memory
Source: Nat Commun. 2024 May 28;15:4526. doi: 10.1038/s41467-024-49023-4 (PMC11133463; doi:10.1038/s41467-024-49023-4)
Supplement: Supplementary file 6 — Supplementary Data 3 [file 41467_2024_49023_MOESM6_ESM.pdf]

Supplementary Data 3. ΔLk values and genomic context of nucleosomes

The first five columns indicate the chromosomal coordinates, length (bp) and the ΔLk restrained by the nucleosomes of the library. The next four columns are parameters defined in Jiang and Pugh (2009) that indicate the nucleosome IDs, the positional fuzziness, the gene ID, and the allocation within a gene body or intergenic region. Coordinates without a nucleosome ID are those that overlapped by <50 bp with referenced nucleosomes. The next two columns tag the gene terminal nucleosomes and rDNA nucleosomes. The last column tags the nucleosomes of telomeric regions, which are defined from The Saccharomyces Genome Database (SGD) (Cherry and Wong 2012) as those overlapping with chromosomal coordinates that include an X element core sequence, X element combinatorial repeats, short or long Y' elements or a terminal stretch of telomeric repeats. Depending on the chromosomal arm, the length of the telomeric regions spans from 430 to 13896 bp.

CHR	START	END	L(bp)	ΔLknuc	Nuc ID	Fuzziness	Gene ID	Gene body postion or intergene	Terminal	rDNA	Telomeric
chrI	375	485	110	**-1,475273061**	N1:382	21,916		Intergene			TEL LEFT
chrI	1992	2143	151	**-1,145624862**	N1:2081	37,206	+2:YAL068C;	2			
chrI	3900	4046	146	**-1,42891971**				Overlap <50 bp			
chrI	7310	7448	138	**-1,099214006**	N1:7314	32,078	+12:YAL067C;	12			
chrI	19698	19849	151	**-1,170720688**	N1:19743	9,192		Intergene			
chrI	24106	24256	150	**-1,002800143**				Overlap <50 bp			
chrI	24106	24267	161	**-1,371408779**				Overlap <50 bp			
chrI	24156	24264	108	**-1,22036164**				Overlap <50 bp			
chrI	24156	24265	109	**-1,199955994**				Overlap <50 bp			
chrI	24156	24266	110	**-1,664918922**				Overlap <50 bp			
chrI	24156	24267	111	**-1,434214917**				Overlap <50 bp			
chrI	25658	25790	132	**-0,89778306**				Overlap <50 bp			
chrI	25658	25797	139	**-1,018529414**				Overlap <50 bp			
chrI	25671	25797	126	**-0,906695696**				Overlap <50 bp			
chrI	25885	26057	172	**-1,008848565**				Overlap <50 bp			
chrI	26200	26329	129	**-1,574518143**				Overlap <50 bp			
chrI	26200	26330	130	**-1,094609367**				Overlap <50 bp			
chrI	26200	26337	137	**-1,169239802**				Overlap <50 bp			
chrI	26204	26330	126	**-1,046753163**				Overlap <50 bp			
chrI	26211	26330	119	**-0,77735452**				Overlap <50 bp			
chrI	26268	26418	150	**-1,229716689**	N1:26380	32,909	+11:YAL063C;	11			
chrI	26290	26465	175	**-1,827207551**	N1:26380	32,909	+11:YAL063C;	11			
chrI	26425	26573	148	**-1,091924323**	N1:26590	51,619	+10:YAL063C;	10			
chrI	26560	26708	148	**-1,251179066**	N1:26590	51,619	+10:YAL063C;	10			
chrI	26695	26843	148	**-1,090783865**	N1:26787	43,547	+9:YAL063C;	9			
chrI	27347	27495	148	**-1,357301342**	N1:27437	37,487	+5:YAL063C;	5			
chrI	28545	28653	108	**-0,815770683**	N1:28676	33,941	+7:SUT433;	7			
chrI	28558	28705	147	**-1,585279852**	N1:28676	33,941	+7:SUT433;	7			
chrI	28865	28996	131	**-1,004955603**	N1:28942	15,556	+5:SUT433;	5			
chrI	31670	31815	145	**-1,382405277**	N1:31784	18,209	+6:SUT434;	6			
chrI	32825	32968	143	**-1,230034943**	N1:32915	24,186	-1:SUT434;	-1			
chrI	38266	38455	189	**-1,633713958**	N1:38289	16	+6:YAL058W;	6			
chrI	38266	38455	189	**-1,633713958**	N1:38459	37,157	+7:YAL058W;	7			
chrI	39356	39504	148	**-1,28106733**	N1:39417	8,503	+2:YAL056W;	2			
chrI	44014	44169	155	**-1,329113517**	N1:44128	16,158	+6:YAL054C; +5:SUT002;	6			
chrI	48679	48833	154	**-1,364356893**	N1:48747	8,256	+2:YAL051W;	2			
chrI	52107	52211	104	**-0,997609421**	N1:52114	21,119	+4:YAL049C;	4			
chrI	52108	52200	92	**-1,194494905**	N1:52114	21,119	+4:YAL049C;	4			
chrI	52108	52202	94	**-1,115050422**	N1:52114	21,119	+4:YAL049C;	4			
chrI	52108	52203	95	**-1,203469923**	N1:52114	21,119	+4:YAL049C;	4			
chrI	52108	52204	96	**-1,174333328**	N1:52114	21,119	+4:YAL049C;	4			
chrI	52108	52205	97	**-1,008989827**	N1:52114	21,119	+4:YAL049C;	4			
chrI	52108	52206	98	**-1,232433255**	N1:52114	21,119	+4:YAL049C;	4			
chrI	52108	52207	99	**-1,533375011**	N1:52114	21,119	+4:YAL049C;	4			
chrI	52108	52208	100	**-0,882098917**	N1:52114	21,119	+4:YAL049C;	4			
chrI	52108	52209	101	**-1,127128482**	N1:52114	21,119	+4:YAL049C;	4			
chrI	52108	52210	102	**-1,320587152**	N1:52114	21,119	+4:YAL049C;	4			
chrI	52108	52211	103	**-1,00632145**	N1:52114	21,119	+4:YAL049C;	4			
chrI	52112	52211	99	**-1,312463337**	N1:52114	21,119	+4:YAL049C;	4			
chrI	54178	54332	154	**-1,179031036**	N1:54248	6,306	+4:YAL048C;	4			
chrI	56067	56218	151	**-1,391434877**	N1:56143	16,79	+6:YAL047C;	6			
chrI	57573	57723	150	**-1,176476393**	N1:57555	3,899	-1:YAL046C; +1:YAL044W-A;	-1			
chrI	57573	57723	150	**-1,176476393**	N1:57726	5,502	+2:YAL044W-A;	2			
chrI	57985	58142	157	**-1,491896053**	N1:58076	12,422	+3:YAL044C; +4*:YAL044W-A;	3			
chrI	58357	58529	172	**-1,395417578**	N1:58432	12,859	+1:YAL044C;	1			
chrI	59835	59985	150	**-1,525981569**	N1:59903	12,276	+8:YAL043C;	8			
chrI	60956	61114	158	**-1,201260038**	N1:61025	8,618	-1:YAL042W; +1:YAL043C;	-1			
chrI	61139	61253	114	**-1,514954291**				Overlap <50 bp			
chrI	62731	62859	128	**-1,118454861**	N1:62815	15,092	+1:YAL041W;	1			
chrI	73815	73966	151	**-1,456101359**	N1:73878	12,462	AL037C-A; 0:YAL037W; 0:Unit002;	-1			
chrI	75929	76089	160	**-0,635190429**	N1:75990	4,207	+2:YAL036C;	2			
chrI	77820	77969	149	**-1,329929447**	N1:77885	28,324		Intergene			
chrI	78867	79013	146	**-1,476014857**	N1:78863	32,79		Intergene			
chrI	79075	79223	148	**-1,445857802**	N1:79100	26,734		Intergene			
chrI	79820	79919	99	**-0,868345432**	N1:79895	4,29	+2:YAL034W-A;	2			
chrI	80712	80877	165	**-1,102806447**	N1:80770	14,428	+8:YAL034C;	8			
chrI	83877	84031	154	**-1,014732041**	N1:83953	6,494	+4:YAL032C;	4			
chrI	87561	87716	155	**-1,568954094**	N1:87641	9,368		Intergene			
chrI	88852	89013	161	**-1,349929244**	N1:89028	31,911	+22:YAL029C;	22			
chrI	88852	89013	161	**-1,349929244**	N1:88849	19,502	+23:YAL029C;	23			
chrI	88889	89053	164	**-1,71832009**	N1:89028	31,911	+22:YAL029C;	22			
chrI	91036	91130	94	**-0,529061161**	N1:91042	21,515	+10:YAL029C;	10			
chrI	94271	94416	145	**-1,127470224**				Overlap <50 bp			
chrI	97984	98151	167	**-1,439823481**	N1:98096	12,437	+11:YAL026C;	11			
chrI	100299	100401	102	**0,145125764**	N1:100335	20,156	+6:YAL025C;	6			
chrI	100430	100592	162	**-0,972570656**	N1:100502	12,449	+5:YAL025C;	5			
chrI	100744	100901	157	**-1,177782262**	N1:100813	8,781	+3:YAL025C;	3			
chrI	100903	101028	125	**-0,681458043**	N1:100972	4,401	+2:YAL025C;	2			
chrI	101774	101913	139	**-1,268887199**	N1:101784	18,177	+26:YAL024C;	26			
chrI	102759	102918	159	**-1,404046068**	N1:102829	19,565	+20:YAL024C;	20			

CHR	START	END	L(bp)	ΔLknuc	Nuc ID	Fuzziness	Gene ID	Gene body postion or intergene	Terminal	rDNA	Telomeric
chrI	110352	110498	146	**-1,262096736**	N1:110405	10,033	+1:YAL022C;	1			
chrI	110959	111107	148	**-1,365708219**	N1:110988	7,005	+16:YAL021C;	16			
chrI	111829	111987	158	**-2,304517965**	N1:111914	12,91	+10:YAL021C;	10			
chrI	111829	111989	160	**-1,220675432**	N1:111914	12,91	+10:YAL021C;	10			
chrI	111835	111987	152	**-1,321773661**	N1:111914	12,91	+10:YAL021C;	10			
chrI	111835	111989	154	**-1,903586461**	N1:111914	12,91	+10:YAL021C;	10			
chrI	113198	113345	147	**-1,313354286**	N1:113359	19,466	+1:YAL021C;	1			
chrI	113198	113345	147	**-1,313354286**	N1:113192	8,824	+2:YAL021C;	2			
chrI	113272	113431	159	**-1,295849447**	N1:113359	19,466	+1:YAL021C;	1			
chrI	114916	115064	148	**-1,000643596**	N1:115021	8,649	+2:YAL019W;	2			
chrI	116310	116474	164	**-1,186230225**	N1:116396	42,101	+11:YAL019W;	11			
chrI	119258	119408	150	**-0,376293772**	N1:119265	14,519	+3:YAL018C;	3			
chrI	121555	121709	154	**-1,247553434**	N1:121654	14,905	+12:YAL017W;	12			
chrI	128610	128759	149	**-1,338333574**				Overlap <50 bp			
chrI	128749	128913	164	**-1,42014932**	N1:128845	11,978	+2:YAL014C;	2			
chrI	132160	132339	179	**-1,625001105**	N1:132234	8,735	+1:YAL011W;	1			
chrI	132322	132449	127	**-1,12899717**	N1:132398	10,521	+2:YAL011W;	2			
chrI	133311	133456	145	**-1,279779418**	N1:133382	11,127	+8:YAL011W;	8			
chrI	133444	133595	151	**-1,312572395**	N1:133558	12,813	+9:YAL011W;	9			
chrI	134936	135082	146	**-1,233599314**				Overlap <50 bp			
chrI	139478	139611	133	**-0,937522987**	N1:139570	10,426	+13:YAL005C;	13			
chrI	144452	144595	143	**-1,203524654**	N1:144447	8,18	+6:YAL002W;	6			
chrI	144452	144595	143	**-1,203524654**	N1:144596	18,794	+7:YAL002W;	7			
chrI	145995	146131	136	**-0,824624641**	N1:145995	30,089	+15:YAL002W;	15			
chrI	145995	146131	136	**-0,824624641**	N1:146151	24,542	+16:YAL002W;	16			
chrI	157171	157325	154	**-1,420376368**	N1:157317	31,92	+9:YAR007C;	9			
chrI	158225	158386	161	**-1,162030477**	N1:158305	6,164	+3:YAR007C;	3			
chrI	158476	158631	155	**-1,466764872**	N1:158641	1,751	-1:YAR008W; +1:YAR007C;	-1			
chrI	158476	158631	155	**-1,466764872**	N1:158473	6,314	+2:YAR007C;	2			
chrI	159246	159353	107	**-1,32864883**	N1:159237	21,23	+3:YAR008W;	3			
chrI	161173	161287	114	**-1,44116562**	N1:161301	30,254		Intergene			
chrI	163265	163422	157	**-1,360421984**	N1:163329	36,373		Intergene			
chrI	164304	164458	154	**-1,406236959**	N1:164322	46,436		Intergene			
chrI	164304	164458	154	**-1,406236959**	N1:164474	15,524		Intergene			
chrI	164952	165092	140	**-1,239994795**	N1:165025	16,503		Intergene			
chrI	164958	165092	134	**-1,223023256**	N1:165025	16,503		Intergene			
chrI	165370	165525	155	**-1,247413069**	N1:165400	21,385		Intergene			
chrI	165501	165609	108	**-0,974105815**	N1:165556	35,162		Intergene			
chrI	165531	165649	118	**-1,16531138**	N1:165556	35,162		Intergene			
chrI	165650	165799	149	**-1,184904444**	N1:165722	1,414		Intergene			
chrI	170185	170316	131	**-0,991286797**				Overlap <50 bp			
chrI	170746	170892	146	**-1,419404324**	N1:170868	11,895	+3:CUT004; +6:YAR018C;	3			
chrI	173251	173422	171	**-1,247836659**				Overlap <50 bp			
chrI	174403	174559	156	**-1,268429026**	N1:174467	7,257	+5:YAR019C;	5			
chrI	177062	177157	95	**-0,925986621**				Overlap <50 bp			
chrI	178178	178314	136	**-1,157292599**	N1:178222	37,276		Intergene			
chrI	181632	181781	149	**-1,6308092**	N1:181719	10,926		Intergene			
chrI	184868	185010	142	**-1,180277539**	N1:184881	10,053	+1:YAR028W;	1			
chrI	188940	189093	153	**-1,21049533**	N1:189025	15,588	l:anti-YAR033W-2; +6*:YAR033W;	1			
chrI	189954	190061	107	**-0,739135051**	N1:190031	20,354	0:YAR035W; +9:anti006;	0			
chrI	189954	190062	108	**-0,738228073**	N1:190031	20,354	0:YAR035W; +9:anti006;	0			
chrI	189954	190063	109	**-0,850741142**	N1:190031	20,354	0:YAR035W; +9:anti006;	0			
chrI	189954	190077	123	**-1,285620828**	N1:190031	20,354	0:YAR035W; +9:anti006;	0			
chrI	191415	191573	158	**-1,529273336**	N1:191409	39,245	+9:YAR035W; 0:anti006;	9			
chrI	191722	191874	152	**-1,237006303**	N1:191782	11,389	+11:YAR035W; +3:SUT436;	11			
chrI	192889	193041	152	**-1,723783132**	N1:192956	22,963	+3:YAR042W;	3			
chrI	193467	193622	155	**-1,310298541**	N1:193586	11	+7:YAR042W;	7			
chrI	194126	194279	153	**-1,34565981**				Overlap <50 bp			
chrI	194141	194279	138	**-0,95749856**				Overlap <50 bp			
chrI	194290	194442	152	**-1,043901966**	N1:194358	7,396	+12:YAR042W;	12			
chrI	204527	204675	148	**-1,366287583**	N1:204558	21,213	+8:YAR050W;	8			
chrI	204662	204810	148	**-1,204518936**	N1:204754	36,77	+9:YAR050W;	9			
chrI	204797	204945	148	**-1,194263798**				Overlap <50 bp			
chrI	205040	205170	130	**-1,159623338**				Overlap <50 bp			
chrI	205573	205699	126	**-0,727906068**	N1:205618	18,385	+14:YAR050W;	14			
chrI	205573	205705	132	**-1,254685071**	N1:205618	18,385	+14:YAR050W;	14			
chrI	205573	205712	139	**-1,012284504**	N1:205618	18,385	+14:YAR050W;	14			
chrI	205580	205712	132	**-0,99796908**	N1:205618	18,385	+14:YAR050W;	14			
chrI	206390	206543	153	**-1,836820478**	N1:206455	34,269	+19:YAR050W;	19			
chrI	207406	207531	125	**-0,816530428**	N1:207477	12,021	+25:YAR050W;	25			
chrI	215442	215555	113	**-1,736345291**	N1:215495	14,849		Intergene			
chrI	217878	218015	137	**-1,162588108**	N1:217966	3,512		Intergene			
chrI	217916	218076	160	**-1,479307354**	N1:217966	3,512		Intergene			
chrI	218711	218856	145	**-1,136650022**	N1:218780	38,158		Intergene			
chrI	218738	218886	148	**-1,215559937**	N1:218780	38,158		Intergene			
chrI	222387	222554	167	**-1,349608552**	N1:222504	27,109	+2:YAR068W;	2			
chrI	222751	222855	104	**-0,980770953**	N1:222820	21,572	+4:YAR068W;	4			
chrI	223257	223420	163	**-1,483544312**	N1:223335	24,826		Intergene			
chrI	224065	224173	108	**-1,318042122**	N1:224083	3,786		Intergene			
chrI	224877	224997	120	**-1,326308076**	N1:224931	13,892		Intergene			
chrI	226540	226687	147	**-1,204924103**	N1:226556	39,737	+8:YAR071W;	8			
chrI	229861	230005	144	**-1,14650557**	N1:229980	25,565		Intergene			TEL RIGHT
chrI	230119	230218	99	**-0,283250953**	N1:230156	20,664		Intergene			TEL RIGHT
chrI	230124	230218	94	**-0,112407352**	N1:230156	20,664		Intergene			TEL RIGHT
chrII	163	311	148	**-1,339618443**	N2:255	24,749	+17:YBL113C;	17			TEL LEFT
chrII	526	677	151	**-1,122700906**	N2:589	17,214	+15:YBL113C;	15			TEL LEFT
chrII	1089	1240	151	**-1,153642956**	N2:1150	29,783	+11:YBL113C;	11			TEL LEFT
chrII	1727	1858	131	**-0,962631553**	N2:1853	30,854	+7:YBL113C;	7			TEL LEFT
chrII	1727	1870	143	**-1,208984888**	N2:1853	30,854	+7:YBL113C;	7			TEL LEFT
chrII	1727	1914	187	**-1,023851407**	N2:1853	30,854	+7:YBL113C;	7			TEL LEFT
chrII	1931	2076	145	**-1,398920977**				Overlap <50 bp			TEL LEFT
chrII	1936	2043	107	**-0,634852792**				Overlap <50 bp			TEL LEFT
chrII	1937	2034	97	**-0,762519621**				Overlap <50 bp			TEL LEFT
chrII	1937	2042	105	**-1,618967553**				Overlap <50 bp			TEL LEFT
chrII	1937	2046	109	**-0,680066278**				Overlap <50 bp			TEL LEFT
chrII	1937	2051	114	**-1,142580445**				Overlap <50 bp			TEL LEFT

CHR	START	END	L(bp)	ΔLknuc	Nuc ID	Fuzziness	Gene ID	Gene body postion or intergene	Terminal	rDNA	Telomeric
chrII	1937	2054	117	**-1,282598983**				Overlap <50 bp			TEL LEFT
chrII	1937	2057	120	**-1,129549176**				Overlap <50 bp			TEL LEFT
chrII	1937	2062	125	**-1,838064879**				Overlap <50 bp			TEL LEFT
chrII	1937	2071	134	**-1,084959621**				Overlap <50 bp			TEL LEFT
chrII	1937	2072	135	**-1,226959369**				Overlap <50 bp			TEL LEFT
chrII	1937	2073	136	**-1,957126123**				Overlap <50 bp			TEL LEFT
chrII	1937	2075	138	**-1,596758786**				Overlap <50 bp			TEL LEFT
chrII	1937	2076	139	**-1,105255183**				Overlap <50 bp			TEL LEFT
chrII	1937	2082	145	**-2,679580974**				Overlap <50 bp			TEL LEFT
chrII	1937	2086	149	**-1,63174152**				Overlap <50 bp			TEL LEFT
chrII	1938	2076	138	**-1,224450881**				Overlap <50 bp			TEL LEFT
chrII	1939	2076	137	**-1,518387315**				Overlap <50 bp			TEL LEFT
chrII	1940	2076	136	**-1,359600303**				Overlap <50 bp			TEL LEFT
chrII	1941	2076	135	**-1,029142639**				Overlap <50 bp			TEL LEFT
chrII	1942	2076	134	**-1,726837644**				Overlap <50 bp			TEL LEFT
chrII	1944	2076	132	**-1,181981789**				Overlap <50 bp			TEL LEFT
chrII	1947	2076	129	**-1,517897595**				Overlap <50 bp			TEL LEFT
chrII	1948	2076	128	**-1,355921231**				Overlap <50 bp			TEL LEFT
chrII	1952	2076	124	**-0,982107431**				Overlap <50 bp			TEL LEFT
chrII	1953	2076	123	**-0,584200369**				Overlap <50 bp			TEL LEFT
chrII	1964	2076	112	**-0,897829145**				Overlap <50 bp			TEL LEFT
chrII	1966	2076	110	**-0,663844067**				Overlap <50 bp			TEL LEFT
chrII	2124	2286	162	**-1,489886698**	N2:2264	49,166	+4:YBL113C;	4			TEL LEFT
chrII	2124	2286	162	**-1,489886698**	N2:2110	20,033	+5:YBL113C;	5			TEL LEFT
chrII	2191	2286	95	**-0,863684874**	N2:2264	49,166	+4:YBL113C;	4			TEL LEFT
chrII	2267	2374	107	**-0,785948273**	N2:2264	49,166	+4:YBL113C;	4			TEL LEFT
chrII	2267	2414	147	**-1,559119201**	N2:2264	49,166	+4:YBL113C;	4			TEL LEFT
chrII	2522	2620	98	**-0,719252405**				Overlap <50 bp			TEL LEFT
chrII	2522	2673	151	**-1,076920734**				Overlap <50 bp			TEL LEFT
chrII	3354	3498	144	**-0,952337581**	N2:3449	18,475	+11:YBL111C;	11			TEL LEFT
chrII	3419	3571	152	**-1,250718532**	N2:3449	18,475	+11:YBL111C;	11			TEL LEFT
chrII	3701	3844	143	**-1,424193058**	N2:3787	13,868	+9:YBL111C;	9			TEL LEFT
chrII	3992	4129	137	**-1,254485891**	N2:4071	14,731	+7:YBL111C;	7			TEL LEFT
chrII	7980	8135	155	**-1,017882777**				Overlap <50 bp			
chrII	10712	10874	162	**-1,593400301**	N2:10765	10,926	+20:YBL106C; -1:YBL107C;	20			
chrII	20981	21152	171	**-1,715983406**	N2:21084	6,706	+2:YBL104C;	2			
chrII	21442	21596	154	**-1,536203893**	N2:21498	18,577	-1:YBL104C;	-1			
chrII	24248	24399	151	**-1,031056071**	N2:24353	4,583	+3:YBL102W;	3			
chrII	24299	24437	138	**-1,158174315**	N2:24353	4,583	+3:YBL102W;	3			
chrII	25280	25416	136	**-1,143379963**	N2:25382	6,348	+20:YBL101C;	20			
chrII	26126	26261	135	**-1,054825399**	N2:26202	33,963	+15:YBL101C;	15			
chrII	30232	30334	102	**-0,651831833**				Overlap <50 bp			
chrII	31655	31803	148	**-1,092010891**				Overlap <50 bp			
chrII	32970	33121	151	**-1,103793485**	N2:32989	9,866		Intergene			
chrII	35988	36136	148	**-1,244201563**	N2:36067	5,859		Intergene			
chrII	48768	48912	144	**-1,239553472**	N2:48843	7,74	+1:YBL090W; -1:YBL091C;	1			
chrII	54394	54539	145	**-0,986216701**	N2:54546	32,988	+31:YBL088C;	31			
chrII	54394	54539	145	**-0,986216701**	N2:54380	27,379	+32:YBL088C;	32			
chrII	54551	54716	165	**-1,139673053**	N2:54732	19,799	+30:YBL088C;	30			
chrII	54551	54716	165	**-1,139673053**	N2:54546	32,988	+31:YBL088C;	31			
chrII	62389	62546	157	**-1,382861839**	N2:62463	4,875	+2:YBL086C;	2			
chrII	62885	63033	148	**-1,263117382**	N2:62915	14,569	-1:YBL086C;	-1			
chrII	64104	64264	160	**-1,318805016**				Overlap <50 bp			
chrII	64653	64794	141	**-1,398025619**				Overlap <50 bp			
chrII	65899	66050	151	**-1,495715774**	N2:65948	12,404	+14:YBL085W;	14			
chrII	67731	67881	150	**-1,37633213**	N2:67884	32,216		Intergene			
chrII	69118	69263	145	**-1,159153969**	N2:69156	9,333	+3:YBL084C;	3			
chrII	79393	79540	147	**-1,719609071**	N2:79471	11,701	+26:YBL079W;	26			
chrII	81527	81678	151	**-1,374821732**	N2:81676	23,415	+16:YBL076C;	16			
chrII	81527	81678	151	**-1,374821732**	N2:81506	24,489	+17:YBL076C;	17			
chrII	86475	86605	130	**-1,015290796**	N2:86602	9,283	0:YBL075C; +8:YBL074C;	0			
chrII	87052	87211	159	**-1,613149953**	N2:87114	15,611	+5:YBL074C;	5			
chrII	94866	95023	157	**-1,496880406**	N2:94979	10,407	+8:YBL067C;	8			
chrII	97063	97217	154	**-1,273904521**	N2:97213	41,347	+21:YBL066C;	21			
chrII	97108	97247	139	**-0,912827287**	N2:97213	41,347	+21:YBL066C;	21			
chrII	98068	98229	161	**-1,463100968**	N2:98203	16,861	+15:YBL066C;	15			
chrII	98405	98563	158	**-1,400718636**	N2:98532	27,178	+13:YBL066C;	13			
chrII	102188	102336	148	**-1,48455441**	N2:102248	6,686	+3:YBL063W;	3			
chrII	105450	105577	127	**-1,068773785**	N2:105509	25,398	+12:YBL061C;	12			
chrII	105532	105684	152	**-1,44357567**	N2:105680	26,153	+11:YBL061C;	11			
chrII	105532	105684	152	**-1,44357567**	N2:105509	25,398	+12:YBL061C;	12			
chrII	105754	105910	156	**-0,815046163**	N2:105836	34,085	+10:YBL061C;	10			
chrII	106088	106233	145	**-1,111842236**				Overlap <50 bp			
chrII	112631	112737	106	**-1,138361425**	N2:112714	14,148	+5*:YBL057C; +9*:YBL058W;	5	TERM		
chrII	112631	112749	118	**-1,165103148**	N2:112714	14,148	+5*:YBL057C; +9*:YBL058W;	5	TERM		
chrII	117607	117737	130	**-0,896674642**	N2:117637	15,007	+2:YBL054W;	2			
chrII	118185	118336	151	**-1,33033991**	N2:118300	12,57	+6:YBL054W;	6			
chrII	118430	118588	158	**-1,195666979**	N2:118451	26,306	+7:YBL054W;	7			
chrII	118430	118588	158	**-1,195666979**	N2:118611	18,099	+8:YBL054W;	8			
chrII	118604	118737	133	**-0,865913164**	N2:118611	18,099	+8:YBL054W;	8			
chrII	118662	118820	158	**-1,46619699**	N2:118790	26,216	+9:YBL054W;	9			
chrII	120690	120830	140	**-1,11073866**	N2:120678	17,053	+10:YBL052C;	10			
chrII	130330	130481	151	**-1,354184127**	N2:130370	38,561	+11:YBL047C;	11			
chrII	133172	133320	148	**-1,608611205**	N2:133293	14,293	+6:YBL046W;	6			
chrII	134044	134208	164	**-1,343929583**	N2:134212	38,539	-1:Unit009; +10:YBL045C;	-1			
chrII	134044	134208	164	**-1,343929583**	N2:134052	32,455	0:Unit009; +11:YBL045C;	0			
chrII	146223	146376	153	**-1,321233983**	N2:146325	8,017	+2:YBL038W;	2			
chrII	147729	147892	163	**-1,64896304**				Overlap <50 bp			
chrII	157306	157457	151	**-1,34277469**	N2:157445	18,162	+9:YBL034C;	9			
chrII	158794	158975	181	**-1,489809654**	N2:158865	12,624	+6:YBL033C; -1:YBL034C;	6			
chrII	159579	159667	88	**-0,672768137**	N2:159667	4,324	+1:YBL033C;	1			
chrII	159579	159734	155	**-1,41516889**	N2:159667	4,324	+1:YBL033C;	1			
chrII	161150	161286	136	**-1,277479554**	N2:161276	10,559	+8*:YBL032W;	8	TERM		
chrII	161650	161786	136	**-1,18045652**	N2:161723	6,676	+1:YBL031W;	1			
chrII	164812	164963	151	**-1,237920302**	N2:164880	12,501	+1:YBL029C-A; +3*:SUT006;	1			
chrII	169155	169305	150	**-1,30591113**	N2:169306	20,56	+7:YBL027W;	7			

CHR	START	END	L(bp)	ΔLknuc	Nuc ID	Fuzziness	Gene ID	Gene body postion or intergene	Terminal	rDNA	Telomeric
chrII	171571	171720	149	**-1,161946715**	N2:171641	19,308	+2:YBL025W;	2			
chrII	171661	171810	149	**-1,240220877**	N2:171641	19,308	+2:YBL025W;	2			
chrII	171661	171810	149	**-1,240220877**	N2:171803	10,807	+3:YBL025W;	3			
chrII	174305	174447	142	**-1,091529763**	N2:174374	30,192	+13:YBL024W;	13			
chrII	186048	186192	144	**-1,062458065**	N2:186122	13,126		Intergene			
chrII	187191	187351	160	**-1,627421457**	N2:187294	16,294	+28:YBL017C;	28			
chrII	188175	188332	157	**-1,343589318**	N2:188328	34,317	+22:YBL017C;	22			
chrII	189372	189539	167	**-1,520501284**				Overlap <50 bp			
chrII	192490	192638	148	**-1,128087103**	N2:192564	9,283	+2:YBL016W;	2			
chrII	195265	195410	145	**-1,171387466**	N2:195403	43,981	+9:YBL015W;	9			
chrII	203726	203877	151	**-1,250439918**	N2:203822	8,846	+3:YBL011W;	3			
chrII	204265	204408	143	**-1,571212415**	N2:204305	18,267	+6:YBL011W;	6			
chrII	213654	213809	155	**-0,658603789**	N2:213693	21,109	+16:YBL007C;	16			
chrII	214589	214739	150	**-1,489830875**	N2:214660	10,33	+11:YBL007C;	11			
chrII	214869	215017	148	**-1,176619464**	N2:214983	23,267	+9:YBL007C;	9			
chrII	215320	215464	144	**-1,10391934**	N2:215386	24,924	+7:YBL007C;	7			
chrII	216639	216771	132	**-1,723397887**	N2:216655	7,789	+4:YBL006C; -1:YBL007C;	4			
chrII	217546	217694	148	**-1,12663001**	N2:217574	15,84	+2:YBL005W;	2			
chrII	222615	222772	157	**-1,142025999**	N2:222719	20,075		Intergene			
chrII	222619	222772	153	**-1,446041905**	N2:222719	20,075		Intergene			
chrII	223146	223281	135	**-1,214836418**	N2:223185	24,269		Intergene			
chrII	223772	223929	157	**-1,483106003**	N2:223866	14,572		Intergene			
chrII	223872	224016	144	**-1,1357005**	N2:223866	14,572		Intergene			
chrII	223872	224016	144	**-1,1357005**	N2:224033	28,711		Intergene			
chrII	224435	224582	147	**-1,232898347**	N2:224426	19,502		Intergene			
chrII	224435	224582	147	**-1,232898347**	N2:224599	21,92		Intergene			
chrII	225175	225325	150	**-1,410601247**	N2:225274	23,824		Intergene			
chrII	226374	226481	107	**-1,596265069**	N2:226487	21,92		Intergene			
chrII	226832	226946	114	**-1,349868328**	N2:226921	17,037		Intergene			
chrII	228528	228693	165	**-1,754544703**				Overlap <50 bp			
chrII	233542	233703	161	**-1,488641967**	N2:233579	26,011		Intergene			
chrII	234707	234798	91	**-1,399796163**	N2:234705	33,041		Intergene			
chrII	239108	239251	143	**-1,160932322**	N2:239202	30,612	+13:YBR001C;	13			
chrII	249586	249752	166	**-1,460240793**	N2:249650	13,428	+9:YBR007C;	9			
chrII	252919	253070	151	**-1,338288891**				Overlap <50 bp			
chrII	253969	254127	158	**-1,517313475**	N2:254057	17,173	+4:YBR008C;	4			
chrII	255566	255720	154	**-1,430021544**	N2:255613	29,262	+1:YBR009C;	1			
chrII	260109	260232	123	**-1,053192789**				Overlap <50 bp			
chrII	260209	260364	155	**-1,509131457**	N2:260319	31,723		Intergene			
chrII	260642	260776	134	**-0,586070067**	N2:260707	28,618		Intergene			
chrII	260642	260782	140	**-0,963757536**	N2:260707	28,618		Intergene			
chrII	261685	261820	135	**-0,995726897**	N2:261719	2,646		Intergene			
chrII	262132	262282	150	**-1,10978534**	N2:262199	21,197		Intergene			
chrII	262311	262468	157	**-1,462281745**	N2:262403	32,047		Intergene			
chrII	262974	263121	147	**-1,058720824**	N2:262967	21,197		Intergene			
chrII	263717	263867	150	**-1,384266737**	N2:263815	64,023		Intergene			
chrII	264916	265023	107	**-1,458520689**				Overlap <50 bp			
chrII	269730	269883	153	**-1,101384237**	N2:269845	8,62	-1:YBR015C;	-1			
chrII	269730	269885	155	**-2,233425246**	N2:269845	8,62	-1:YBR015C;	-1			
chrII	269781	269930	149	**-1,410364781**	N2:269845	8,62	-1:YBR015C;	-1			
chrII	272917	273071	154	**-1,381071069**	N2:273057	9,695	+5:YBR017C;	5			
chrII	279942	280075	133	**-1,076543114**	N2:280032	11,349	+7:YBR020W;	7			
chrII	282915	283035	120	**-1,09549185**	N2:282943	35,064	+11:YBR021W;	11			
chrII	288899	289053	154	**-1,330754737**	N2:288983	4,899		Intergene			
chrII	289452	289559	107	**-1,216533893**	N2:289558	6,616	+2:YBR024W;	2			
chrII	289452	289573	121	**-1,285147454**	N2:289558	6,616	+2:YBR024W;	2			
chrII	292636	292792	156	**-1,332846524**	N2:292683	7,759		Intergene			
chrII	295986	296116	130	**-0,860940601**	N2:296031	8,479	+1:YBR028C; -1:CUT010;	1			
chrII	296940	297047	107	**-1,335508013**	N2:296987	24,977	+6:YBR029C;	6			
chrII	296940	297056	116	**-1,498188266**	N2:296987	24,977	+6:YBR029C;	6			
chrII	298361	298510	149	**-1,193268767**	N2:298428	10,52	+2:YBR030W;	2			
chrII	298515	298656	141	**-1,059273554**	N2:298593	8,539	+3:YBR030W;	3			
chrII	300341	300475	134	**-0,914362894**	N2:300360	24,322	+2:YBR031W;	2			
chrII	303103	303264	161	**-1,461900746**	N2:303172	49,379	+9:YBR033W; +9:SUT445;	9			
chrII	304167	304319	152	**-1,267210037**	N2:304247	16,721	+15:YBR033W; +3:SUT445;	15			
chrII	306192	306322	130	**-1,280327839**	N2:306304	12,317	+5:YBR035C;	5			
chrII	310844	311002	158	**-1,368252604**	N2:310950	6,408	+4:YBR037C; +3*:CUT013;	4			
chrII	314561	314691	130	**-0,962520912**	N2:314625	45,711	+18*:YBR038W;	18	TERM		
chrII	316179	316338	159	**-1,604946668**	N2:316235	23,964	+5:YBR039W;	5			
chrII	320829	320984	155	**-1,26189574**	N2:320918	9,165	+5:YBR042C;	5			
chrII	324261	324383	122	**-0,975459417**	N2:324329	21,524	R043C; +11*:YBR044C; -1:CUT014;	-1			
chrII	324593	324738	145	**-1,28786413**	N2:324676	5,718	+9:YBR044C; +2:CUT014;	9			
chrII	333410	333578	168	**-1,408097988**	N2:333441	40,576		Intergene			
chrII	334171	334335	164	**-1,639948638**	N2:334243	18,608	+16*:YBR049C;	16	TERM		
chrII	341568	341673	105	**-0,834575191**				Overlap <50 bp			
chrII	344002	344150	148	**-1,201988833**	N2:344104	18,285	+8:YBR054W;	8			
chrII	345574	345727	153	**-1,297378957**	N2:345699	34,213	+11:YBR055C;	11			
chrII	348428	348599	171	**-1,13153481**	N2:348504	6,131	+5:YBR056W;	5			
chrII	348488	348624	136	**-1,132777903**	N2:348504	6,131	+5:YBR056W;	5			
chrII	352601	352731	130	**-1,055310767**	N2:352692	22,232	+5:YBR057C;	5			
chrII	354955	355107	152	**-1,254234777**	N2:355010	14,844	+7:YBR058C;	7			
chrII	360789	360938	149	**-1,474616729**	N2:360839	5,797	+11:YBR060C; +2:CUT018;	11			
chrII	369124	369272	148	**-1,429493757**	N2:369186	29,645	+4:YBR065C;	4			
chrII	369614	369763	149	**-1,063372795**	N2:369688	6,683	+1:YBR065C;	1			
chrII	372782	372926	144	**-1,014742943**	N2:372886	30,028	+11:SUT016; 0:YBR067C;	11			
chrII	379733	379878	145	**-1,112952051**	N2:379800	14,16	+2:YBR070C;	2			
chrII	380438	380593	155	**-1,077365548**	N2:380439	10,198	+1:YBR071W;	1			
chrII	380438	380593	155	**-1,077365548**	N2:380588	15,868	+2:YBR071W;	2			
chrII	383232	383381	149	**-0,996686495**	N2:383330	10,633	+2:YBR073W; -1:YBR072C-A;	2			
chrII	384056	384165	109	**-1,623780409**				Overlap <50 bp			
chrII	384888	385039	151	**-1,319140026**	N2:384899	36,378	+12:YBR073W;	12			
chrII	385956	386120	164	**-1,211693631**	N2:386082	32,347	-1:YBR074W;	-1			
chrII	386724	386859	135	**-1,053143167**	N2:386790	13,644	+4:YBR074W;	4			
chrII	402057	402202	145	**-1,276781246**	N2:402105	40,195	+21:YBR081C;	21			
chrII	405294	405447	153	**-1,159552264**	N2:405333	7,083	+1:YBR081C;	1			
chrII	411086	411218	132	**-0,877040498**	N2:411221	7,092	+2:YBR084W;	2			

CHR	START	END	L(bp)	ΔLknuc	Nuc ID	Fuzziness	Gene ID	Gene body postion or intergene	Terminal	rDNA	Telomeric
chrII	412922	413067	145	**-1,266292301**	N2:413068	10,368	+13:YBR084W;	13			
chrII	417276	417442	166	**-1,2587924**	N2:417375	10,21	+4:Unit017;	4			
chrII	418844	418980	136	**-2,768708743**	N2:418877	4,021	+3:YBR085C-A;	3			
chrII	420492	420630	138	**-1,109448642**	N2:420545	31,738	+17:YBR086C;	17			
chrII	432763	432911	148	**-1,831373047**	N2:432818	16,227	+6:YBR094W;	6			
chrII	435614	435764	150	**-1,369178642**	N2:435690	11,118	+1:YBR095C; -1:YBR096W;	1			
chrII	441246	441413	167	**-1,504828399**	N2:441290	29,543	+29:YBR097W; -1:YBR098W;	29			
chrII	442313	442462	149	**-1,369837877**				Overlap <50 bp			
chrII	447274	447421	147	**-1,002159996**	N2:447332	3,656	-1:YBR103W; +1:YBR102C;	-1			
chrII	448183	448311	128	**-1,092945784**	N2:448253	12,973	+5:YBR103W;	5			
chrII	448687	448853	166	**-1,509032771**				Overlap <50 bp			
chrII	450522	450683	161	**-1,314301324**	N2:450575	13,726	+7*:YBR104W;	7	TERM		
chrII	456967	457110	143	**-1,532475687**	N2:456997	9,602	+14:YBR108W;	14			
chrII	459507	459619	112	**-1,579094464**	N2:459525	13,172	+5:YBR110W;	5			
chrII	463510	463659	149	**-1,404145294**	N2:463671	35,929		Intergene			
chrII	464402	464557	155	**-1,127412307**	N2:464478	22,113	+10:YBR112C;	10			
chrII	467973	468117	144	**-1,032238428**	N2:468080	9,236	+6:YBR114W;	6			
chrII	469937	470099	162	**-1,37154338**	N2:469962	17,234	+27:YBR115C;	27			
chrII	478544	478678	134	**-0,815841234**	N2:478563	46,083	+8:YBR118W;	8			
chrII	479993	480154	161	**-1,259329534**	N2:480057	49,497		Intergene			
chrII	480073	480226	153	**-1,23061109**	N2:480057	49,497		Intergene			
chrII	483614	483775	161	**-1,214965849**	N2:483752	12,416	+1:CUT026; -1:YBR121C;	1			
chrII	490237	490391	154	**-0,981533398**	N2:490316	21,747	+2:YBR126C;	2			
chrII	496459	496596	137	**-1,246572939**	N2:496548	5,805	+3:YBR130C;	3			
chrII	498698	498864	166	**-1,379409744**	N2:498780	27,474	+11:YBR131W;	11			
chrII	499848	499990	142	**-0,927358262**	N2:499949	23,845	+12:YBR132C;	12			
chrII	501137	501288	151	**-1,080504278**	N2:501227	9,888	+4:YBR132C;	4			
chrII	503704	503871	167	**-1,452303465**	N2:503875	21,622	+4:YBR133C;	4			
chrII	503704	503871	167	**-1,452303465**	N2:503696	25,832	+5:YBR133C;	5			
chrII	505974	506148	174	**-1,394268654**	N2:506017	6,87	+4:YBR136W;	4			
chrII	506025	506148	123	**-0,62939195**	N2:506017	6,87	+4:YBR136W;	4			
chrII	506025	506179	154	**-1,241093561**	N2:506017	6,87	+4:YBR136W;	4			
chrII	506025	506179	154	**-1,241093561**	N2:506177	7,225	+5:YBR136W;	5			
chrII	509772	509912	140	**-1,082668098**	N2:509886	33,421	+28:YBR136W;	28			
chrII	511646	511795	149	**-1,434658486**	N2:511651	27,644	+38:YBR136W;	38			
chrII	512273	512423	150	**-1,44706331**	N2:512342	8,937	+42:YBR136W;	42			
chrII	519148	519306	158	**-1,271055536**	N2:519220	10,668	+46:YBR140C; +54:YBR141C;	46			
chrII	523260	523407	147	**-1,337333998**	N2:523344	0	+21:YBR140C; +29:YBR141C;	21			
chrII	523774	523925	151	**-1,316681204**				Overlap <50 bp			
chrII	525378	525528	150	**-1,287368088**	N2:525423	30,425	+9:YBR140C; +17:YBR141C;	9			
chrII	527770	527902	132	**-1,066277836**	N2:527832	3,204	+2:YBR141C;	2			
chrII	528914	529061	147	**-1,099649381**	N2:528946	34,173	+5:YBR142W;	5			
chrII	529231	529384	153	**-1,273423221**	N2:529279	12,75	+7:YBR142W;	7			
chrII	533755	533907	152	**-0,989226591**	N2:533826	29,992	+1:YBR145W;	1			
chrII	543934	544097	163	**-1,34536352**	N2:543989	13,867	+4:YBR150C;	4			
chrII	545512	545645	133	**-1,088763431**				Overlap <50 bp			
chrII	550054	550200	146	**-0,857880812**	N2:550101	17,509	+3:YBR155W;	3			
chrII	551769	551916	147	**-1,300364602**	N2:551862	37,91	+9:YBR156C;	9			
chrII	551821	551946	125	**-1,326670932**	N2:551862	37,91	+9:YBR156C;	9			
chrII	552584	552756	172	**-1,440625385**	N2:552697	13,633	+4:YBR156C;	4			
chrII	560316	560442	126	**-1,221672782**	N2:560343	13,038	+3:YBR160W;	3			
chrII	568857	569009	152	**-1,31346641**	N2:568953	8,085	+2:YBR165W;	2			
chrII	570513	570675	162	**-1,289589675**	N2:570686	8,733	+4:YBR166C;	4			
chrII	570513	570675	162	**-1,289589675**	N2:570530	24,785	+5:YBR166C;	5			
chrII	575360	575489	129	**-0,977519812**	N2:575420	12,49	+4:YBR169C;	4			
chrII	575363	575489	126	**-1,175867983**	N2:575420	12,49	+4:YBR169C;	4			
chrII	575635	575789	154	**-1,754362726**	N2:575736	6,775	+2:YBR169C;	2			
chrII	577662	577794	132	**-1,636094452**	N2:577744	7,321	+3:YBR170C;	3			
chrII	581371	581525	154	**-1,645286481**	N2:581425	9,182	+1:YBR172C;	1			
chrII	583028	583186	158	**-1,289905811**	N2:583091	13,088	+5:YBR175W;	5			
chrII	583277	583426	149	**-1,159587992**				Overlap <50 bp			
chrII	589093	589249	156	**-1,56010912**	N2:589146	13,038	+1:YBR179C;	1			
chrII	598510	598657	147	**-1,232680898**	N2:598568	32,623	+8:YBR184W;	8			
chrII	599865	600024	159	**-1,182357063**	N2:599938	9,094	+1:YBR185C;	1			
chrII	601519	601677	158	**-1,511310659**	N2:601539	8,526	+5*:anti012; +7:YBR186W;	5	TERM		
chrII	601519	601677	158	**-1,511310659**	N2:601690	18,091	+4:anti012; +8:YBR186W;	4			
chrII	608166	608308	142	**-1,05398561**	N2:608176	2,191	+5:YBR192W;	5			
chrII	608166	608308	142	**-1,05398561**	N2:608330	8,025	+6:YBR192W;	6			
chrII	610440	610604	164	**-1,484272313**	N2:610482	28,991	+4*:YBR194W;	4	TERM		
chrII	615980	616137	157	**-1,063248295**	N2:616096	25,129	-1:YBR197C;	-1			
chrII	618802	618957	155	**-1,229307997**	N2:618851	6,772	+1:YBR199W; -1:YBR198C;	1			
chrII	621368	621503	135	**-1,129265196**	N2:621487	21,166	+5:YBR200W;	5			
chrII	624538	624687	149	**-1,249099736**	N2:624638	10,756	+2:YBR201C-A;	2			
chrII	626717	626881	164	**-1,361430048**	N2:626851	25,153	+8:YBR202W;	8			
chrII	633744	633889	145	**-1,035454002**	N2:633802	9,98	+2:YBR205W;	2			
chrII	633747	633889	142	**-1,135180111**	N2:633802	9,98	+2:YBR205W;	2			
chrII	634590	634750	160	**-1,296665043**	N2:634645	27,668	+7:YBR205W;	7			
chrII	635204	635350	146	**-1,938915442**	N2:635289	8,289	+2:YBR207W;	2			
chrII	636694	636853	159	**-1,375705026**	N2:636766	22,941		Intergene			
chrII	636695	636853	158	**-1,364353674**	N2:636766	22,941		Intergene			
chrII	636699	636853	154	**-1,515038997**	N2:636766	22,941		Intergene			
chrII	641881	642011	130	**-1,209245845**	N2:641909	5,037	+3:YBR208C;	3			
chrII	643000	643147	147	**-1,329472018**	N2:643066	11,003		Intergene			
chrII	643625	643773	148	**-1,155154848**	N2:643703	20,516		Intergene			
chrII	645958	646066	108	**-0,417230945**	N2:646057	9,452	+5*:YBR210W;	5	TERM		
chrII	652842	653006	164	**-1,075597988**				Overlap <50 bp			
chrII	667311	667462	151	**-2,246118016**	N2:667376	25,608	+7:YBR222C;	7			
chrII	670468	670600	132	**-1,364029643**	N2:670509	22,176	-1:YBR223C; +1:YBR225W;	-1			
chrII	672951	673112	161	**-1,184502263**	N2:673046	8,585	+16:YBR225W;	16			
chrII	673886	673964	78	**-0,798**	N2:673878	30,222	+8:YBR227C;	8			
chrII	673886	673989	103	**-0,672906555**	N2:673878	30,222	+8:YBR227C;	8			
chrII	674406	674512	106	**-1,582178995**	N2:674454	9,668	+5:YBR227C;	5			
chrII	674406	674523	117	**-1,454474226**	N2:674454	9,668	+5:YBR227C;	5			
chrII	678764	678912	148	**-1,175676074**	N2:678851	8,477	+3:YBR229C;	3			
chrII	682036	682166	130	**-1,045846273**	N2:682093	41,012	-1:CUT034;	-1			
chrII	683947	684082	135	**-1,093585499**	N2:684039	9,867	+5:YBR233W;	5			

CHR	START	END	L(bp)	ΔLknuc	Nuc ID	Fuzziness	Gene ID	Gene body postion or intergene	Terminal	rDNA	Telomeric
chrII	684506	684665	159	-1,400190924	N2:684541	13,867	+8:YBR233W;	8			
chrII	685114	685267	153	-1,143652323	N2:685155	9,201	+2:YBR233W-A;	2			
chrII	688568	688714	146	-1,313717319	N2:688658	27,969	+12:YBR235W;	12			
chrII	693669	693820	151	-1,624186433	N2:693836	6,429	+13*:YBR237W;	13	TERM		
chrII	696303	696437	134	-0,984598953				Overlap <50 bp			
chrII	698169	698308	139	-0,934238114	N2:698202	28,19	+3:anti016;	3			
chrII	698728	698854	126	-0,996001992	N2:698800	13,206	+8:YBR239C;	8			
chrII	698730	698836	106	-0,715778305	N2:698800	13,206	+8:YBR239C;	8			
chrII	699474	699629	155	-1,3447482	N2:699534	13,05	+4:YBR239C;	4			
chrII	699802	699945	143	-1,679432646	N2:699870	11,622	+2:YBR239C;	2			
chrII	699802	699953	151	-1,700062035	N2:699870	11,622	+2:YBR239C;	2			
chrII	699834	699945	111	-1,313347211	N2:699870	11,622	+2:YBR239C;	2			
chrII	699834	699953	119	-0,934322568	N2:699870	11,622	+2:YBR239C;	2			
chrII	702899	703031	132	-1,534616715	N2:702974	14,422	+7*:YBR241C;	7	TERM		
chrII	706525	706696	171	-1,242849783	N2:706600	3,125	+2:YBR243C;	2			
chrII	708493	708657	164	-1,040369298	N2:708624	31,291	+18:YBR245C;	18			
chrII	708881	709054	173	-1,481569108	N2:708943	34,96	+16:YBR245C;	16			
chrII	716866	717023	157	-1,369908789	N2:716955	10,727	+8:YBR249C;	8			
chrII	716874	717023	149	-1,108888063	N2:716955	10,727	+8:YBR249C;	8			
chrII	718549	718695	146	-1,483357466	N2:718618	16,956	+2:Unit021;	2			
chrII	721490	721639	149	-1,114106296	N2:721551	12,073	+2:YBR251W;	2			
chrII	723357	723507	150	-1,24577868	N2:723426	6,221	+2:YBR253W;	2			
chrII	727858	728011	153	-1,111040747	N2:727936	4,135	+2:YBR256C;	2			
chrII	733512	733676	164	-1,628574356	N2:733639	30,94	+8:YBR260C;	8			
chrII	735258	735391	133	-0,601625644	N2:735332	8,118	+2:YBR261C;	2			
chrII	736781	736927	146	-1,194053632	N2:736768	5,989	+4:YBR263W;	4			
chrII	736912	737033	121	-1,076385223				Overlap <50 bp			
chrII	740252	740411	159	-1,308668504	N2:740348	11,413	+4:YBR267W;	4			
chrII	743678	743781	103	-1,597747713	N2:743723	18,469	+6:YBR270C;	6			
chrII	746216	746373	157	-1,423993514	N2:746234	22,405	+9*:YBR271W; +10*:YBR272C;	9	TERM		
chrII	747192	747321	129	-1,046172535	N2:747277	11,185	+4:YBR272C;	4			
chrII	748930	749090	160	-1,367845746	N2:749008	9,935	+3:YBR273C;	3			
chrII	749254	749401	147	-1,108331229	N2:749334	7,026	-1:YBR274W; +1:YBR273C;	-1			
chrII	750045	750206	161	-1,100825585	N2:750083	17,143	+4:YBR274W;	4			
chrII	760513	760620	107	-0,945882361				Overlap <50 bp			
chrII	760513	760634	121	-1,219239247				Overlap <50 bp			
chrII	771359	771518	159	-1,437711441				Overlap <50 bp			
chrII	771799	771953	154	-1,298920275				Overlap <50 bp			
chrII	774249	774405	156	-1,411326104	N2:774294	5,404	+3:YBR285W;	3			
chrII	775701	775861	160	-0,929463514				Overlap <50 bp			
chrII	777331	777484	153	-1,339755057	N2:777349	9,607	+5:YBR287W;	5			
chrII	779629	779798	169	-1,50707337	N2:779707	7,899	+1:YBR289W; -1:YBR288C;	1			
chrII	780145	780251	106	-1,053022222	N2:780192	4,676	+4:YBR289W;	4			
chrII	780145	780260	115	-0,918611905	N2:780192	4,676	+4:YBR289W;	4			
chrII	780582	780757	175	-1,500281704	N2:780666	31,575		Intergene			
chrII	781915	782083	168	-1,198476355	N2:782014	4		Intergene			
chrII	786436	786542	106	-0,801755722	N2:786464	5,128	Init023; +3*:SUT451; +7*:SUT023;	-1			
chrII	786436	786560	124	-1,062389934	N2:786464	5,128	Init023; +3*:SUT451; +7*:SUT023;	-1			
chrII	788442	788582	140	-1,010280463	N2:788514	10,759	+10*:YBR293W;	10	TERM		
chrII	788604	788759	155	-1,447389067	N2:788689	4,848		Intergene			
chrII	789546	789709	163	-1,586585326	N2:789650	8,66	+16:anti017; +4:YBR294W;	16			
chrII	791029	791155	126	-1,301735875	N2:791088	39,659	+7:anti017; +13:YBR294W;	7			
chrII	791029	791156	127	-1,036811816	N2:791088	39,659	+7:anti017; +13:YBR294W;	7			
chrII	791031	791136	105	-0,713070249	N2:791088	39,659	+7:anti017; +13:YBR294W;	7			
chrII	793238	793400	162	-1,179922456	N2:793321	24,028	+4:YBR295W;	4			
chrII	795412	795559	147	-1,168072292	N2:795519	41,841	+18:YBR295W;	18			
chrII	809074	809203	129	-1,3505723	N2:809118	18,385	+2:YBR301W;	2			
chrII	812955	813098	143	-1,217374679	N2:812953	6,083		Intergene			TEL RIGHT
chrIII	2	145	143	-0,954679452				Overlap <50 bp			TEL LEFT
chrIII	2	176	174	-0,229084052				Overlap <50 bp			TEL LEFT
chrIII	4	145	141	-1,406102945				Overlap <50 bp			TEL LEFT
chrIII	10	145	135	-1,31929281				Overlap <50 bp			TEL LEFT
chrIII	10	146	136	-0,995025744				Overlap <50 bp			TEL LEFT
chrIII	14	145	131	-0,89276556				Overlap <50 bp			TEL LEFT
chrIII	19	145	126	-0,676104031				Overlap <50 bp			TEL LEFT
chrIII	26	145	119	-1,106431779				Overlap <50 bp			TEL LEFT
chrIII	37	145	108	-0,911015253				Overlap <50 bp			TEL LEFT
chrIII	41	145	104	-1,016883833				Overlap <50 bp			TEL LEFT
chrIII	50	145	95	-0,840231361				Overlap <50 bp			TEL LEFT
chrIII	50	176	126	0,156676152				Overlap <50 bp			TEL LEFT
chrIII	50	185	135	-0,746822302				Overlap <50 bp			TEL LEFT
chrIII	50	201	151	-0,678938322				Overlap <50 bp			TEL LEFT
chrIII	50	220	170	-0,703926889				Overlap <50 bp			TEL LEFT
chrIII	73	263	190	-1,312793189				Overlap <50 bp			TEL LEFT
chrIII	88	231	143	-1,267464587				Overlap <50 bp			TEL LEFT
chrIII	97	212	115	-0,931331371				Overlap <50 bp			TEL LEFT
chrIII	110	263	153	-1,269630523				Overlap <50 bp			TEL LEFT
chrIII	110	280	170	-0,88860386	N3:298	23,979		Intergene			TEL LEFT
chrIII	110	283	173	-1,116390842	N3:298	23,979		Intergene			TEL LEFT
chrIII	115	280	165	-0,923278483	N3:298	23,979		Intergene			TEL LEFT
chrIII	115	283	168	-1,147224261	N3:298	23,979		Intergene			TEL LEFT
chrIII	118	284	166	-0,929152627	N3:298	23,979		Intergene			TEL LEFT
chrIII	129	252	123	-0,919105825				Overlap <50 bp			TEL LEFT
chrIII	129	260	131	-0,782812905				Overlap <50 bp			TEL LEFT
chrIII	129	263	134	-0,72076581				Overlap <50 bp			TEL LEFT
chrIII	129	280	151	-0,943894971	N3:298	23,979		Intergene			TEL LEFT
chrIII	129	283	154	-0,955521888	N3:298	23,979		Intergene			TEL LEFT
chrIII	129	284	155	-1,723242825	N3:298	23,979		Intergene			TEL LEFT
chrIII	129	301	172	-1,695454046	N3:298	23,979		Intergene			TEL LEFT
chrIII	129	309	180	-1,188465012	N3:298	23,979		Intergene			TEL LEFT
chrIII	129	326	197	-1,004285517	N3:298	23,979		Intergene			TEL LEFT
chrIII	143	284	141	-1,573492119	N3:298	23,979		Intergene			TEL LEFT
chrIII	164	284	120	-0,828521588	N3:298	23,979		Intergene			TEL LEFT
chrIII	164	341	177	-0,676782129	N3:298	23,979		Intergene			TEL LEFT
chrIII	186	284	98	-0,74917568	N3:298	23,979		Intergene			TEL LEFT
chrIII	186	320	134	-1,551770143	N3:298	23,979		Intergene			TEL LEFT

CHR	START	END	L(bp)	ΔLknuc	Nuc ID	Fuzziness	Gene ID	Gene body postion or intergene	Terminal	rDNA	Telomeric
chrIII	186	337	151	**-1,671725041**	N3:298	23,979		Intergene			TEL LEFT
chrIII	186	341	155	**-1,327448223**	N3:298	23,979		Intergene			TEL LEFT
chrIII	186	352	166	**-1,426052459**	N3:298	23,979		Intergene			TEL LEFT
chrIII	186	353	167	**-1,532908001**	N3:298	23,979		Intergene			TEL LEFT
chrIII	186	355	169	**-0,772229989**	N3:298	23,979		Intergene			TEL LEFT
chrIII	192	329	137	**-0,926236863**	N3:298	23,979		Intergene			TEL LEFT
chrIII	246	341	95	**-0,900552583**	N3:298	23,979		Intergene			TEL LEFT
chrIII	246	352	106	**-0,70570749**	N3:298	23,979		Intergene			TEL LEFT
chrIII	5166	5314	148	**-1,232000214**	N3:5206	29,597		Intergene			
chrIII	5882	5994	112	**-1,835019833**	N3:5973	10,599		Intergene			
chrIII	7560	7714	154	**-1,589499231**	N3:7557	35,473	+6:YCL073C;	6			
chrIII	9830	9940	110	**-0,920543576**	N3:9914	23,714	+2:YCL069W;	2			
chrIII	9831	9940	109	**-1,031392141**	N3:9914	23,714	+2:YCL069W;	2			
chrIII	9831	9941	110	**-0,834303001**	N3:9914	23,714	+2:YCL069W;	2			
chrIII	9832	9955	123	**-1,865931237**	N3:9914	23,714	+2:YCL069W;	2			
chrIII	11462	11613	151	**-1,358056487**	N3:11616	29,676	+5:YCL068C;	5			
chrIII	17946	18093	147	**-1,381815646**	N3:18008	16,096	+5:YCL063W;	5			
chrIII	23277	23427	150	**-1,304688533**	N3:23351	10,386	+1:YCL059C; -1:YCL058W-A;	1			
chrIII	27235	27381	146	**-1,287126281**	N3:27325	26,748	+2:YCL056C;	2			
chrIII	32929	33083	154	**-1,205673076**	N3:33061	29,942	+10:YCL054W;	10			
chrIII	34797	34957	160	**-1,554230317**	N3:34875	5,68	+4:YCL052C;	4			
chrIII	36971	37125	154	**-1,313352521**				Overlap <50 bp			
chrIII	37274	37421	147	**-1,087073864**	N3:37360	22,565	+12:YCL051W;	12			
chrIII	41105	41261	156	**-1,375512355**	N3:41160	18,78	-1:YCL048W-A;	-1			
chrIII	42270	42419	149	**-1,120656719**	N3:42308	15,28	+2:YCL048W;	2			
chrIII	56437	56565	128	**-1,162300779**	N3:56555	12,423	+1:YCL038C;	1			
chrIII	62417	62568	151	**-1,338661321**	N3:62518	44,392	+6:YCL034W;	6			
chrIII	62420	62568	148	**-1,395111568**	N3:62518	44,392	+6:YCL034W;	6			
chrIII	62779	62946	167	**-1,52612384**	N3:62871	14,007	+3*:YCL033C;	3	TERM		
chrIII	62988	63143	155	**-1,460107083**	N3:63052	10,71	+2:YCL033C;	2			
chrIII	68315	68492	177	**-1,726689326**	N3:68474	39,598	+10*:YCL029C; 0:YCL030C;	10	TERM		
chrIII	68580	68746	166	**-1,413775684**	N3:68637	37,978	:YCL029C; -1:CUT041; -1:YCL030C;	9			
chrIII	74254	74383	129	**-1,009646792**				Overlap <50 bp			
chrIII	75106	75269	163	**-1,425221364**	N3:75209	19,463	+2:YCL026C-A;	2			
chrIII	82256	82435	179	**-2,027682426**	N3:82326	31,277	+21*:YCL024W;	21	TERM		
chrIII	86659	86788	129	**-1,251349554**				Overlap <50 bp			
chrIII	86884	87013	129	**-1,331965765**	N3:86885	15,588		Intergene			
chrIII	88349	88492	143	**-1,005872508**	N3:88396	13,748		Intergene			
chrIII	89624	89790	166	**-1,771992996**				Overlap <50 bp			
chrIII	90428	90577	149	**-1,065351661**	N3:90522	24,846		Intergene			
chrIII	90432	90582	150	**-1,320665557**	N3:90522	24,846		Intergene			
chrIII	92227	92379	152	**-1,112431138**	N3:92268	8,485	+7:YCL018W;	7			
chrIII	93544	93697	153	**-1,293327448**	N3:93669	7,463	+5:YCL017C;	5			
chrIII	94513	94671	158	**-1,465807665**	N3:94604	12,106	+8*:YCL016C; -1:YCL017C;	8	TERM		
chrIII	95867	96028	161	**-1,710905784**	N3:95947	9,065	-1:YCL014W; 0:YCL016C;	-1			
chrIII	95874	96028	154	**-1,447836567**	N3:95947	9,065	-1:YCL014W; 0:YCL016C;	-1			
chrIII	97469	97602	133	**-1,495463613**	N3:97480	31,252	+9:YCL014W;	9			
chrIII	100878	101025	147	**-1,28986259**	N3:100936	15,083	+29:YCL014W;	29			
chrIII	100878	101048	170	**-1,335822729**	N3:100936	15,083	+29:YCL014W;	29			
chrIII	101513	101652	139	**-1,045398788**	N3:101599	8,55	+2:YCL012C;	2			
chrIII	101547	101689	142	**-1,14446132**	N3:101599	8,55	+2:YCL012C;	2			
chrIII	101696	101853	157	**-1,118911908**	N3:101768	5,854	+1:YCL012C;	1			
chrIII	103033	103165	132	**-0,774247191**	N3:103166	25,719	+2:YCL011C;	2			
chrIII	103103	103249	146	**-1,156783737**	N3:103166	25,719	+2:YCL011C;	2			
chrIII	103103	103251	148	**-1,102522501**	N3:103166	25,719	+2:YCL011C;	2			
chrIII	104725	104877	152	**-1,120089765**	N3:104791	9,066	+6:YCL009C;	6			
chrIII	106067	106230	163	**-1,314909405**	N3:106142	4,967	+5:YCL008C; -1:SUT027;	5			
chrIII	116514	116644	130	**-1,218378718**	N3:116605	17,682	+5:SUT456; +2:Unit067;	5			
chrIII	117818	117976	158	**-1,308629601**	N3:117863	14,865	+4:YCR002C;	4			
chrIII	132816	132923	107	**-1,552858369**				Overlap <50 bp			
chrIII	132817	132923	106	**-2,005595077**				Overlap <50 bp			
chrIII	133268	133417	149	**-1,198946699**	N3:133250	24,654	0:YCR010C;	0			
chrIII	134878	135010	132	**-1,602285663**	N3:134986	45,665	+13:YCR011C;	13			
chrIII	136592	136744	152	**-1,025019285**	N3:136655	25,667	+3:YCR011C;	3			
chrIII	137946	138097	151	**-1,251124188**	N3:138059	33,63	+3:YCR012W;	3			
chrIII	138189	138339	150	**-1,222217245**	N3:138298	30,452	+4:YCR012W;	4			
chrIII	146706	146857	151	**-1,617003251**	N3:146686	11,149	+7:YCR017C;	7			
chrIII	154254	154394	140	**-1,164384349**	N3:154383	21,554		Intergene			
chrIII	154256	154394	138	**-1,381052849**	N3:154383	21,554		Intergene			
chrIII	156400	156550	150	**-1,246299697**	N3:156457	28,745	+5:YCR021C;	5			
chrIII	156437	156550	113	**-1,042650876**	N3:156457	28,745	+5:YCR021C;	5			
chrIII	156484	156645	161	**-1,554770183**	N3:156660	32,7	+4:YCR021C;	4			
chrIII	158189	158338	149	**-1,564439488**				Overlap <50 bp			
chrIII	159911	160084	173	**-1,769715977**	N3:160082	7,148	+3:YCR023C;	3			
chrIII	159911	160084	173	**-1,769715977**	N3:159912	18,454	+4:YCR023C;	4			
chrIII	161801	161938	137	**-1,845675426**	N3:161850	16,664	+4:YCR024C;	4			
chrIII	175934	176084	150	**-1,159976807**	N3:176001	18,243	+4:YCR030C;	4			
chrIII	186417	186569	152	**-1,345774075**	N3:186495	7,483	+2:YCR033W;	2			
chrIII	188100	188260	160	**-1,669387205**	N3:188165	17,349	+13:YCR033W;	13			
chrIII	191522	191646	124	**-1,031499942**	N3:191559	45,337	+7:YCR034W;	7			
chrIII	195429	195578	149	**-1,20943358**				Overlap <50 bp			
chrIII	201078	201230	152	**-1,18881041**				Overlap <50 bp			
chrIII	208738	208879	141	**-1,882906559**	N3:208806	20,744	+6:YCR045C;	6			
chrIII	209585	209734	149	**-1,10906871**	N3:209637	10,635	+1:YCR045C;	1			
chrIII	210916	211072	156	**-1,244734556**	N3:210951	13,817	+5:YCR047C;	5			
chrIII	213468	213602	134	**-0,954607696**	N3:213471	16,678	+3:YCR050C; +10:YCR048W;	3			
chrIII	215653	215790	137	**-0,865616105**	N3:215679	16,303	+6:YCR052W;	6			
chrIII	217960	218102	142	**-1,208113529**	N3:218083	30,45	+10*:YCR053W;	10	TERM		
chrIII	228591	228741	150	**-1,284766155**	N3:228733	34,94	+3:YCR063W;	3			
chrIII	231011	231166	155	**-1,659060501**	N3:231100	18,298	+12*:YCR065W;	12	TERM		
chrIII	234251	234402	151	**-1,518111731**	N3:234286	23,258	+13:YCR067C;	13			
chrIII	234921	235052	131	**-1,76544927**				Overlap <50 bp			
chrIII	236307	236469	162	**-1,389862065**	N3:236392	4,412	+1:YCR067C;	1			
chrIII	238120	238291	171	**-1,962163652**	N3:238307	2,646	+3:CUT467; +8:YCR068W;	3			
chrIII	238120	238291	171	**-1,962163652**	N3:238147	55,567	+4:CUT467; +7:YCR068W;	4			
chrIII	240298	240452	154	**-1,67725888**	N3:240369	4,622	+2:YCR071C;	2			

CHR	START	END	L(bp)	ΔLknuc	Nuc ID	Fuzziness	Gene ID	Gene body postion or intergene	Terminal	rDNA	Telomeric
chrIII	240452	240614	162	**-1,282702067**	N3:240532	4,792	+1:YCR071C;	1			
chrIII	242569	242719	150	**-1,059230457**	N3:242631	10,431	YCR073C; +1:SUT038; -1:YCR072C;	25			
chrIII	246007	246188	181	**-1,929933178**	N3:246094	9,786	+4:YCR073C;	4			
chrIII	246926	247057	131	**-1,007454773**	N3:246993	9,368	-1:YCR073C; +1:YCR073W-A;	-1			
chrIII	246926	247058	132	**-0,897760429**	N3:246993	9,368	-1:YCR073C; +1:YCR073W-A;	-1			
chrIII	248934	249085	151	**-1,480690621**	N3:249001	8,01	-1:YCR075C; +1:YCR075W-A;	-1			
chrIII	251728	251875	147	**-0,985710543**				Overlap <50 bp			
chrIII	252213	252361	148	**-1,181121786**	N3:252282	2,608	+3:YCR077C;	3			
chrIII	252238	252397	159	**-1,370693883**	N3:252282	2,608	+3:YCR077C;	3			
chrIII	254663	254821	158	**-1,293109683**	N3:254734	3,937	+4:YCR081W;	4			
chrIII	257083	257228	145	**-1,463182799**	N3:257179	22,247	+18:YCR081W;	18			
chrIII	257806	257969	163	**-1,40977351**	N3:257802	27,308	+22:YCR081W;	22			
chrIII	258183	258325	142	**-1,03873871**	N3:258264	20,248	+25:YCR081W;	25			
chrIII	259144	259292	148	**-1,33061948**	N3:259206	9,131	+3:YCR082W;	3			
chrIII	262204	262356	152	**-1,170638304**	N3:262264	7,778	+3:YCR084C;	3			
chrIII	269289	269448	159	**-1,511255299**	N3:269317	29,513	+13:YCR089W;	13			
chrIII	272784	272929	145	**-1,636330826**	N3:272830	30,64	+1:YCR090C;	1			
chrIII	276672	276825	153	**-1,525113886**	N3:276740	25,57	+15:YCR091W; +21*:YCR092C;	15			
chrIII	280552	280680	128	**-1,388452975**	N3:280622	24,214	+4:YCR093W;	4			
chrIII	281034	281170	136	**-1,077118391**	N3:281080	15,057	+7:YCR093W;	7			
chrIII	286830	286938	108	**-1,112362447**	N3:286909	6,882	+2:YCR094W;	2			
chrIII	286830	286939	109	**-1,124724778**	N3:286909	6,882	+2:YCR094W;	2			
chrIII	286830	286940	110	**-1,309761628**	N3:286909	6,882	+2:YCR094W;	2			
chrIII	286830	286941	111	**-1,155714204**	N3:286909	6,882	+2:YCR094W;	2			
chrIII	286832	286938	106	**-1,580583781**	N3:286909	6,882	+2:YCR094W;	2			
chrIII	286832	286939	107	**-1,643068654**	N3:286909	6,882	+2:YCR094W;	2			
chrIII	286832	286955	123	**-1,721702946**	N3:286909	6,882	+2:YCR094W;	2			
chrIII	288517	288666	149	**-1,741922271**	N3:288593	13,989	+5:YCR095C;	5			
chrIII	303804	303963	159	**-1,37380065**	N3:303873	17,951	+1:anti-YCR102C;	1			
chrIII	307204	307345	141	**-1,196540658**	N3:307293	5,354		Intergene			
chrIII	307633	307788	155	**-0,89410963**				Overlap <50 bp			
chrIII	308042	308191	149	**-1,370040044**	N3:308087	19,807	+3:YCR104W;	3			
chrIII	310321	310472	151	**-1,747404911**	N3:310383	14,711		Intergene			
chrIII	310654	310803	149	**-1,380621571**	N3:310728	17,914	-1:YCR106W;	-1			
chrIII	311798	311947	149	**-1,34814441**	N3:311793	20,917	+6:YCR106W;	6			
chrIII	314554	314707	153	**-1,398051656**	N3:314702	50,43	+6:YCR107W;	6			
chrIV	0	126	126	**-1,10383509**				Overlap <50 bp			TEL LEFT
chrIV	0	145	145	**-1,757917866**				Overlap <50 bp			TEL LEFT
chrIV	0	155	155	**-0,929815149**				Overlap <50 bp			TEL LEFT
chrIV	5	126	121	**-0,65036107**				Overlap <50 bp			TEL LEFT
chrIV	14	126	112	**-0,341478537**				Overlap <50 bp			TEL LEFT
chrIV	14	134	120	**0,009244849**				Overlap <50 bp			TEL LEFT
chrIV	14	155	141	**-0,320519223**				Overlap <50 bp			TEL LEFT
chrIV	44	126	82	**-1,797422563**				Overlap <50 bp			TEL LEFT
chrIV	44	134	90	**-0,866532098**				Overlap <50 bp			TEL LEFT
chrIV	44	145	101	**-1,335249574**				Overlap <50 bp			TEL LEFT
chrIV	44	155	111	**-0,627750502**				Overlap <50 bp			TEL LEFT
chrIV	57	155	98	**-1,175481081**				Overlap <50 bp			TEL LEFT
chrIV	1765	1923	158	**-1,35502516**	N4:1847	27,354	+2:YDL248W;	2			
chrIV	6558	6652	94	**-1,414337508**	N4:6650	21,932	+5:YDL247W;	5			
chrIV	8786	8917	131	**-1,245132351**	N4:8900	7,071	+6:YDL246C;	6			
chrIV	10468	10622	154	**-1,473432049**	N4:10539	3,464		Intergene			
chrIV	11706	11865	159	**-1,150143293**	N4:11735	6,348	+11:YDL245C;	11			
chrIV	12342	12498	156	**-1,614260576**	N4:12435	26,163	+7:YDL245C;	7			
chrIV	12922	13062	140	**-1,097755446**				Overlap <50 bp			
chrIV	13187	13340	153	**-1,067679421**	N4:13269	4,583	+2:YDL245C;	2			
chrIV	15846	16000	154	**-1,342552837**	N4:15932	4,359	-1:YDL244W;	-1			
chrIV	16215	16372	157	**-1,069107003**	N4:16282	7,024	+2:YDL244W;	2			
chrIV	16517	16676	159	**-1,192054713**	N4:16612	14,519	+4:YDL244W;	4			
chrIV	22332	22485	153	**-1,343674134**	N4:22409	3,882	-1:YDL240W; +1:CUT468;	-1			
chrIV	22780	22921	141	**-1,016114724**	N4:22851	8,112	+2:YDL240W;	2			
chrIV	24904	25025	121	**-1,389808288**	N4:25029	29,024	+15:YDL240W;	15			
chrIV	26118	26260	142	**-1,314219095**	N4:26130	27,577		Intergene			
chrIV	28132	28284	152	**-1,460268241**	N4:28244	31,106	+15:YDL238C; +5:YDL239C;	15			
chrIV	28565	28649	84	**-0,345479309**				Overlap <50 bp			
chrIV	28606	28762	156	**-1,05599875**	N4:28689	9,659	+12:YDL238C; +2:YDL239C;	12			
chrIV	29713	29864	151	**-1,459773062**	N4:29784	20,5	+5:YDL238C;	5			
chrIV	30032	30191	159	**-1,422734494**	N4:30113	8,159	+3:YDL238C;	3			
chrIV	31789	31954	165	**-1,593088039**	N4:31846	6,76	+2:CUT469; +8*:YDL237W;	2			
chrIV	32229	32385	156	**-1,61034737**	N4:32309	10,218	+1:YDL236W;	1			
chrIV	33584	33743	159	**-1,391758771**	N4:33663	4,858	+3:YDL235C;	3			
chrIV	34942	35098	156	**-1,390390195**	N4:35008	6,602	+11:YDL234C;	11			
chrIV	43249	43399	150	**-1,213538559**	N4:43358	10,483	+5:YDL230W;	5			
chrIV	43986	44132	146	**-1,385102157**	N4:44075	13,952	+1:YDL229W;	1			
chrIV	51938	52044	106	**-1,216172823**	N4:51987	7,12	+2:YDL226C;	2			
chrIV	55412	55541	129	**-1,196002235**				Overlap <50 bp			
chrIV	56586	56757	171	**-1,227866412**	N4:56663	7,23	+1:YDL224C;	1			
chrIV	60333	60467	134	**-1,097455173**	N4:60396	7,339	+1:YDL223C; +8:SUT041;	1			
chrIV	61937	62077	140	**-1,028434641**	N4:62069	6,221	+18:YDL220C; -1:YDL222C;	18			
chrIV	77124	77282	158	**-1,551926047**	N4:77237	42,658	+5*:YDL213C;	5	TERM		
chrIV	78906	79062	156	**-1,232193114**	N4:78931	10,635	+4:YDL212W;	4			
chrIV	86969	87132	163	**-1,158265081**	N4:87040	6,047	+2:YDL209C;	2			
chrIV	90699	90847	148	**-1,410873582**	N4:90745	8,989	+5:YDL206W;	5			
chrIV	94580	94722	142	**-1,42529725**	N4:94705	27,009	+4:YDL204W;	4			
chrIV	97197	97345	148	**-0,911843753**	N4:97289	4,32	+6:YDL203C;	6			
chrIV	101729	101892	163	**-1,168445221**	N4:101825	20,245	+12:YDL199C;	12			
chrIV	103149	103279	130	**-0,529212448**	N4:103192	9,585	+4:YDL199C;	4			
chrIV	109515	109652	137	**-1,443348703**	N4:109593	10,599		Intergene			
chrIV	109515	109656	141	**-1,213831778**	N4:109593	10,599		Intergene			
chrIV	109515	109666	151	**-1,015005865**	N4:109593	10,599		Intergene			
chrIV	109518	109656	138	**-1,422156087**	N4:109593	10,599		Intergene			
chrIV	110479	110614	135	**-1,188914917**	N4:110476	39,939		Intergene			
chrIV	113056	113240	184	**-1,547311362**	N4:113034	35,62	+10:YDL194W;	10			
chrIV	118783	118936	153	**-1,291131377**	N4:118787	40,577	+19:YDL190C;	19			
chrIV	121491	121639	148	**-1,219188809**	N4:121562	26,283	+2:YDL190C;	2			
chrIV	122619	122777	158	**-1,438248176**	N4:122683	13,914	+4:YDL189W;	4			

CHR	START	END	L(bp)	ΔLknuc	Nuc ID	Fuzziness	Gene ID	Gene body postion or intergene	Terminal	rDNA	Telomeric
chrIV	124114	124238	124	**-1,024991831**	N4:124182	10,017	+7:YDL188C;	7			
chrIV	125012	125174	162	**-1,492641519**	N4:125123	7,95	+1:YDL188C;	1			
chrIV	126907	127064	157	**-1,161292061**	N4:126899	4,506	+2:YDL185W;	2			
chrIV	126907	127064	157	**-1,161292061**	N4:127056	1,826	+3:YDL185W;	3			
chrIV	127133	127254	121	**-1,338539227**	N4:127204	2,95	+4:YDL185W;	4			
chrIV	130391	130540	149	**-1,371628326**	N4:130459	10,834	+1:YDL184C;	1			
chrIV	134454	134627	173	**-1,223835209**	N4:134518	7,906	+8:YDL182W;	8			
chrIV	137744	137906	162	**-1,398042036**	N4:137857	32,549		Intergene			
chrIV	147424	147558	134	**-0,705629157**				Overlap <50 bp			
chrIV	148138	148259	121	**-0,813745752**	N4:148212	3,082	+1:YDL173W;	1			
chrIV	148138	148287	149	**-1,208846281**	N4:148212	3,082	+1:YDL173W;	1			
chrIV	148147	148256	109	**-1,122393819**	N4:148212	3,082	+1:YDL173W;	1			
chrIV	148147	148259	112	**-1,141336115**	N4:148212	3,082	+1:YDL173W;	1			
chrIV	148147	148287	140	**-1,565186748**	N4:148212	3,082	+1:YDL173W;	1			
chrIV	148150	148259	109	**-1,024588753**	N4:148212	3,082	+1:YDL173W;	1			
chrIV	148662	148839	177	**-1,286223853**	N4:148802	33,352	+4:YDL173W;	4			
chrIV	150319	150438	119	**-1,353528407**				Overlap <50 bp			
chrIV	150791	150954	163	**-1,538821231**	N4:150914	16,008	+33:YDL171C;	33			
chrIV	151146	151295	149	**-1,149644659**	N4:151233	24,993	+31:YDL171C;	31			
chrIV	154011	154153	142	**-1,749524939**	N4:154052	36,679	+13:YDL171C;	13			
chrIV	156456	156616	160	**-1,472583844**	N4:156533	4,622	+2:YDL170W;	2			
chrIV	156781	156930	149	**-1,360907042**				Overlap <50 bp			
chrIV	158096	158254	158	**-1,343130283**	N4:158248	11,576	+4:YDL169C;	4			
chrIV	158096	158254	158	**-1,343130283**	N4:158093	50,153	+5:YDL169C;	5			
chrIV	158496	158646	150	**-1,106203647**	N4:158568	5,601	+2:YDL169C;	2			
chrIV	162837	162965	128	**-1,135418386**	N4:162831	10,188	+3:YDL167C;	3			
chrIV	163802	163934	132	**-1,034309912**	N4:163873	11,718	+2:YDL166C;	2			
chrIV	163887	164037	150	**-1,029478734**	N4:164037	7,211	-1:YDL165W; +1:YDL166C;	-1			
chrIV	163887	164037	150	**-1,029478734**	N4:163873	11,718	+2:YDL166C;	2			
chrIV	164671	164820	149	**-1,207463713**	N4:164814	27,338	+4*:YDL165W;	4	TERM		
chrIV	168971	169122	151	**-1,005288111**	N4:169057	14,738	+9*:YDL161W;	9	TERM		
chrIV	180947	181107	160	**-1,293493543**	N4:181019	33,306	+18:YDL154W;	18			
chrIV	185030	185179	149	**-1,263427469**	N4:185100	8,456	+3:YDL149W;	3			
chrIV	185042	185156	114	**-1,280167402**	N4:185100	8,456	+3:YDL149W;	3			
chrIV	196778	196946	168	**-1,063367532**	N4:196824	40,782	+11:YDL145C;	11			
chrIV	198969	199115	146	**-1,081610066**	N4:199010	15,975	+5:YDL144C;	5			
chrIV	200831	200925	94	**-1,401682972**	N4:200946	30,748	+7:YDL143W;	7			
chrIV	202938	203097	159	**-1,561079247**	N4:203012	6,686	+2:YDL141W;	2			
chrIV	204891	205041	150	**-1,164834503**	N4:204992	19,395	+14:YDL141W;	14			
chrIV	205789	205917	128	**-0,914710537**	N4:205848	37,034	+31:YDL140C;	31			
chrIV	214844	215003	159	**-1,426304769**				Overlap <50 bp			
chrIV	224519	224676	157	**-1,291868774**	N4:224539	22,057	+4:YDL132W;	4			
chrIV	224519	224676	157	**-1,291868774**	N4:224699	13,672	+5:YDL132W;	5			
chrIV	228430	228579	149	**-1,442284036**	N4:228507	10,922	+7:YDL131W;	7			
chrIV	230300	230458	158	**-1,403873142**	N4:230409	29,012		Intergene			
chrIV	235048	235188	140	**-1,084110797**	N4:235136	30,684	+2:YDL127W;	2			
chrIV	235556	235704	148	**-1,282020667**	N4:235550	29,368	+4:YDL127W;	4			
chrIV	235556	235704	148	**-1,282020667**	N4:235716	16,932	+5:YDL127W;	5			
chrIV	240743	240895	152	**-0,728390045**	N4:240819	28,978	+4:YDL124W;	4			
chrIV	241083	241236	153	**-1,508388003**	N4:241147	7,25	+6*:YDL124W; -1:YDL123W;	6	TERM		
chrIV	243252	243404	152	**-1,635774479**	N4:243309	7,906	+7:YDL122W;	7			
chrIV	247954	248116	162	**-1,729018586**	N4:247969	5,428	-1:YDL117W; -1:YDL119C;	-1			
chrIV	249148	249302	154	**-1,325363328**				Overlap <50 bp			
chrIV	249297	249444	147	**-1,283329213**				Overlap <50 bp			
chrIV	253799	253940	141	**-1,113168052**				Overlap <50 bp			
chrIV	262881	263029	148	**-1,41641368**	N4:262904	24,803	+25:YDL112W;	25			
chrIV	268349	268491	142	**-0,997311227**	N4:268409	5,02	+5:YDL108W;	5			
chrIV	269754	269886	132	**-0,999288012**	N4:269794	15,672	+6:YDL107W;	6			
chrIV	275399	275562	163	**-1,422105141**	N4:275425	13,206	+8:YDL103C;	8			
chrIV	277085	277203	118	**-1,542351584**	N4:277148	6,091	+3:YDL102W;	3			
chrIV	281092	281244	152	**-1,473127037**	N4:281168	12,95	+5:YDL101C;	5			
chrIV	282751	282862	111	**-1,589919947**	N4:282817	7,301	+3:YDL100C;	3			
chrIV	287334	287498	164	**-1,373556499**	N4:287398	14,029	+3:YDL095W;	3			
chrIV	290503	290642	139	**-1,09999004**	N4:290580	6,025	+5:YDL093W;	5			
chrIV	291728	291875	147	**-1,023422687**	N4:291860	9,218	+13:YDL093W;	13			
chrIV	294716	294818	102	**-1,340369689**	N4:294818	7,376	+1:YDL091C; +10:YDL090C;	1			
chrIV	303242	303392	150	**-1,275637142**	N4:303310	15,604	+2:YDL085W;	2			
chrIV	305394	305543	149	**-1,570453476**	N4:305450	33,906	+3:YDL084W;	3			
chrIV	307871	308025	154	**-1,438235216**	N4:307996	0,577	-1:YDL083C;	-1			
chrIV	308997	309152	155	**-1,359995753**	N4:309056	8,183		Intergene			
chrIV	311358	311498	140	**-1,324086032**	N4:311442	8,808	+7:YDL080C;	7			
chrIV	313115	313272	157	**-1,18734136**	N4:313232	21,674	+10:YDL079C; +3:SUT043;	10			
chrIV	315132	315251	119	**-0,750437952**	N4:315205	6,186	+2:SUT044;	2			
chrIV	316257	316381	124	**-0,957325176**	N4:316392	11,454	+1:YDL078C; +9:SUT044;	1			
chrIV	317932	318025	93	**-1,214586684**				Overlap <50 bp			
chrIV	320773	320934	161	**-1,36708013**	N4:320798	13,517	+3:anti030; +6:YDL076C;	3			
chrIV	324041	324187	146	**-1,285155009**	N4:324118	13,545	+14*:YDL074C; +3:SUT046;	14	TERM		
chrIV	326087	326232	145	**-1,305473147**	N4:326162	12,156	+1:YDL074C;	1			
chrIV	331066	331232	166	**-1,435411356**	N4:331089	11,127	+1:YDL070W; +20*:YDL069C;	1			
chrIV	331066	331232	166	**-1,435411356**	N4:331253	13,964	+2:YDL070W; +19:YDL069C;	2			
chrIV	336500	336644	144	**-1,269088495**	N4:336586	11,776	+5:YDL065C;	5			
chrIV	339003	339155	152	**-1,429959585**	N4:339069	33,411	+8:YDL063C;	8			
chrIV	339626	339796	170	**-1,457911274**	N4:339709	7,855	+4:YDL063C;	4			
chrIV	343754	343879	125	**-1,263432816**	N4:343821	19,281		Intergene			
chrIV	344625	344793	168	**-1,353642831**	N4:344682	3,67	+3:YDL059C;	3			
chrIV	344904	345057	153	**-1,109370367**	N4:345036	4,844	+1:YDL059C;	1			
chrIV	346054	346184	130	**-1,156138877**	N4:346140	9,834	+4:YDL058W;	4			
chrIV	346695	346849	154	**-1,403788246**				Overlap <50 bp			
chrIV	354236	354370	134	**-1,106681292**	N4:354255	24,166	+10:YDL056W;	10			
chrIV	357373	357527	154	**-1,181851262**	N4:357441	26,552		Intergene			
chrIV	357519	357616	97	**-1,596323564**	N4:357598	23,627		Intergene			
chrIV	367261	367414	153	**-1,338313528**	N4:367365	15,497	+6:YDL048C;	6			
chrIV	369734	369884	150	**-1,559769134**	N4:369786	19,343	+2:YDL047W;	2			
chrIV	372733	372859	126	**-1,591174564**	N4:372737	18,759	+7:YDL045C;	7			
chrIV	377302	377457	155	**-1,602995297**	N4:377352	9,138	+8:YDL042C;	8			
chrIV	377961	378102	141	**-1,236383197**	N4:378096	13,382	+3:YDL042C;	3			

CHR	START	END	L(bp)	ΔLknuc	Nuc ID	Fuzziness	Gene ID	Gene body postion or intergene	Terminal	rDNA	Telomeric
chrIV	379551	379707	156	-1,350401121	N4:379708	30,649	+12:YDL040C;	12			
chrIV	380490	380611	121	-1,262833261	N4:380618	5,357	+6:YDL040C;	6			
chrIV	380895	381031	136	-1,146987929	N4:380935	6,593	+4:YDL040C;	4			
chrIV	383984	384136	152	-1,011197975	N4:384003	46,392	DL037C; +2:YDL038C; +15:SUT048;	11			
chrIV	384406	384547	141	-1,383858974				Overlap <50 bp			
chrIV	386414	386562	148	-0,981478283	N4:386473	30,287	it123; +4*:Unit079; +29*:SUT048;	2			
chrIV	389563	389664	101	-1,503448267	N4:389614	24,214	+17:YDL035C;	17			
chrIV	394898	395059	161	-1,400132814	N4:395016	28,599	+6:YDL031W;	6			
chrIV	395659	395798	139	-1,232800588	N4:395816	41,967	+11:YDL031W;	11			
chrIV	405838	405992	154	-1,281385099	N4:405924	18,349	+10:YDL025C;	10			
chrIV	406138	406284	146	-0,952758036				Overlap <50 bp			
chrIV	407097	407258	161	-1,119711242	N4:407198	16,597	+2:YDL025C;	2			
chrIV	407639	407774	135	-1,133144203				Overlap <50 bp			
chrIV	410001	410147	146	-1,557684068	N4:410035	14,72	0:YDL024C;	0			
chrIV	412510	412666	156	-1,689284093	N4:412554	21,014	+5:YDL022W;	5			
chrIV	414505	414611	106	-1,1389347				Overlap <50 bp			
chrIV	414505	414627	122	-1,309091331				Overlap <50 bp			
chrIV	417857	418015	158	-1,412643102	N4:418030	16,739		Intergene			
chrIV	418210	418363	153	-1,321576422	N4:418242	30,003		Intergene			
chrIV	418328	418478	150	-1,541852845				Overlap <50 bp			
chrIV	419977	420127	150	-1,450435292				Overlap <50 bp			
chrIV	430775	430925	150	-1,305814201				Overlap <50 bp			
chrIV	431218	431366	148	-1,35536047	N4:431307	9,579	+2:YDL012C;	2			
chrIV	433624	433778	154	-1,292421838	N4:433704	20,216	+2:YDL008W; +3:SUT464;	2			
chrIV	444592	444742	150	-1,160523841	N4:444681	7,19	+1:YDL003W;	1			
chrIV	447153	447303	150	-1,171812575	N4:447172	10,905	+4:YDL002C;	4			
chrIV	452119	452270	151	-1,427081433	N4:452201	6,419	+4:YDR001C;	4			
chrIV	456027	456181	154	-1,165000882	N4:456128	24,884	+6:YDR004W;	6			
chrIV	464196	464345	149	-1,335150598	N4:464243	15,055	+6:YDR009W;	6			
chrIV	465523	465668	145	-1,238221467	N4:465537	27,213	0:YDR011W;	0			
chrIV	472028	472162	134	-1,005416049	N4:472039	17,914	+2:YDR012W;	2			
chrIV	480768	480918	150	-1,282328158	N4:480858	16,51	+10:YDR017C;	10			
chrIV	481714	481864	150	-1,094149607	N4:481812	29,575	+4:YDR017C; -1:anti-YDR017C;	4			
chrIV	481903	482061	158	-1,306316079	N4:481994	19,073	+3:YDR017C; +1:anti-YDR017C;	3			
chrIV	487395	487546	151	-1,30830358	N4:487410	13,372	+5:YDR021W;	5			
chrIV	488401	488552	151	-1,165496622	N4:488452	17,773	+2:YDR022C;	2			
chrIV	488508	488615	107	-0,944019618	N4:488621	22,914	+1:YDR022C;	1			
chrIV	488508	488632	124	-1,403800432	N4:488621	22,914	+1:YDR022C;	1			
chrIV	491144	491308	164	-1,422663446	N4:491316	12,897	-1:YDR025W;	-1			
chrIV	491144	491308	164	-1,422663446	N4:491134	19,092		Intergene			
chrIV	492176	492332	156	-1,189090425	N4:492275	22,819		Intergene			
chrIV	494125	494275	150	-1,088166097	N4:494221	17,648	+2:YDR026C;	2			
chrIV	499197	499355	158	-0,962310372	N4:499232	49,303	+14:YDR028C;	14			
chrIV	499814	499967	153	-1,465156544	N4:499892	31,785	+10:YDR028C;	10			
chrIV	503596	503736	140	-1,067984168	N4:503693	10,206	+2:YDR031W;	2			
chrIV	503768	503924	156	-1,386951835				Overlap <50 bp			
chrIV	508653	508795	142	-1,078587534	N4:508667	3,899	+6:YDR033W;	6			
chrIV	513880	514029	149	-1,153428135	N4:513900	18,175		Intergene			
chrIV	517444	517573	129	-1,18276644				Overlap <50 bp			
chrIV	518819	518929	110	-1,241534086	N4:518890	20,075		Intergene			
chrIV	521357	521482	125	-1,239691432	N4:521409	13,667	+2:YDR034W-B;	2			
chrIV	522483	522574	91	-1,034891693	N4:522532	38,701	+6:YDR035W;	6			
chrIV	522691	522836	145	-1,03146379	N4:522707	26,26	+7:YDR035W;	7			
chrIV	524988	525151	163	-1,10856945	N4:525064	20,449	-1:YDR037W;	-1			
chrIV	525488	525645	157	-1,396523345				Overlap <50 bp			
chrIV	527030	527163	133	-1,059952544	N4:527071	27,833	+12:YDR037W;	12			
chrIV	527878	528014	136	-1,240376702	N4:527977	28,355	+18:YDR038C;	18			
chrIV	528421	528544	123	-0,683155097	N4:528503	3,536	+15:YDR038C;	15			
chrIV	528421	528548	127	-0,977995532	N4:528503	3,536	+15:YDR038C;	15			
chrIV	529656	529804	148	-1,303964923				Overlap <50 bp			
chrIV	529731	529886	155	-1,419662276				Overlap <50 bp			
chrIV	530359	530519	160	-1,183591246	N4:530430	7,81	+3:YDR038C;	3			
chrIV	531763	531899	136	-1,1859211	N4:531865	26,102	+19:YDR039C;	19			
chrIV	532306	532429	123	-0,846804145	N4:532381	19,732	+15:YDR039C;	15			
chrIV	532306	532433	127	-0,974854934	N4:532381	19,732	+15:YDR039C;	15			
chrIV	533541	533689	148	-1,428567679				Overlap <50 bp			
chrIV	533616	533771	155	-1,111462625				Overlap <50 bp			
chrIV	534244	534404	160	-1,270103301	N4:534319	7,572	+3:YDR039C;	3			
chrIV	535648	535784	136	-1,096550797	N4:535758	21,502	+17:YDR040C;	17			
chrIV	536109	536229	120	-1,433892143				Overlap <50 bp			
chrIV	537426	537574	148	-1,247459289	N4:537413	3,536	+8:YDR040C;	8			
chrIV	537501	537656	155	-1,159342627				Overlap <50 bp			
chrIV	540906	541052	146	-1,611192237	N4:541006	6,723	+5:YDR042C;	5			
chrIV	541731	541872	141	-0,993125626	N4:541799	28,657	-1:YDR042C;	-1			
chrIV	545183	545329	146	-1,111914643	N4:545271	9,352	+5:SUT469; +5:Unit130;	5			
chrIV	549706	549856	150	-1,484719952	N4:549849	34,637	+6:YDR046C;	6			
chrIV	549706	549856	150	-1,484719952	N4:549694	0	+7:YDR046C;	7			
chrIV	552005	552154	149	-1,297012773	N4:552080	20,322	+4:YDR047W;	4			
chrIV	557304	557456	152	-1,126539365	N4:557386	11,635	+5:YDR051C;	5			
chrIV	560465	560600	135	-1,108353986	N4:560548	9,004	+2:YDR052C;	2			
chrIV	570638	570778	140	-1,212675905	N4:570621	8,335	+1:YDR060W;	1			
chrIV	570638	570778	140	-1,212675905	N4:570783	6,998	+2:YDR060W;	2			
chrIV	574209	574359	150	-1,13324516	N4:574313	43,29	+2:YDR061W;	2			
chrIV	575376	575525	149	-1,397404726	N4:575491	11,652	+9:YDR061W; +3:SUT470;	9			
chrIV	585684	585843	159	-1,00622343	N4:585698	22,72	+13:YDR069C;	13			
chrIV	587983	588137	154	-1,245538413	N4:588040	21,649	R069C; +4*:YDR070C; +1:CUT067;	-1			
chrIV	601658	601795	137	-0,750910641	N4:601720	42,687	R077W; +8:YDR078C; -1:Unit131;	5			
chrIV	607967	608120	153	-1,535272632	N4:608009	45,5	+14:YDR081C;	14			
chrIV	615631	615766	135	-1,767681315	N4:615640	15,162	+4:YDR085C; +9:SUT057;	4			
chrIV	618044	618203	159	-1,321763579	N4:618139	5,413	+2:YDR087C;	2			
chrIV	625810	625961	151	-1,393621247	N4:625892	15,947	+2:YDR090C;	2			
chrIV	640754	640908	154	-1,159351216	N4:640848	33,065	+19:YDR097C;	19			
chrIV	643481	643635	154	-1,29941771	N4:643559	13,777	+3:YDR097C;	3			
chrIV	645695	645844	149	-1,199548165	N4:645718	10,693		Intergene			
chrIV	646491	646637	146	-1,376936315	N4:646571	14,468		Intergene			
chrIV	647130	647280	150	-1,328296514	N4:647179	16,803		Intergene			

CHR	START	END	L(bp)	ΔLknuc	Nuc ID	Fuzziness	Gene ID	Gene body postion or intergene	Terminal	rDNA	Telomeric
chrIV	648526	648683	157	**-0,957285711**	N4:648585	34,646		Intergene			
chrIV	648994	649155	161	**-1,397407214**	N4:649119	45,962		Intergene			
chrIV	649174	649309	135	**-1,004035614**	N4:649272	23,065		Intergene			
chrIV	650212	650352	140	**-1,196188496**	N4:650289	52,532		Intergene			
chrIV	650218	650352	134	**-1,145547098**	N4:650289	52,532		Intergene			
chrIV	650630	650785	155	**-1,222824444**	N4:650651	15,044		Intergene			
chrIV	650761	650869	108	**-0,746233357**	N4:650859	51,118		Intergene			
chrIV	654067	654214	147	**-1,095592234**	N4:654056	31,161	+4:YDR099W;	4			
chrIV	654067	654214	147	**-1,095592234**	N4:654214	32,997	+5:YDR099W;	5			
chrIV	656839	656998	159	**-1,177322567**	N4:656949	6,419	+4:YDR101C;	4			
chrIV	658487	658641	154	**-1,539803086**	N4:658664	14,177	+3:YDR103W;	3			
chrIV	658600	658747	147	**-1,321267512**	N4:658664	14,177	+3:YDR103W;	3			
chrIV	662245	662401	156	**-1,201168959**	N4:662311	41,701	+17:YDR104C;	17			
chrIV	665084	665248	164	**-1,637505983**	N4:665151	29,838	-1:YDR104C;	-1			
chrIV	665597	665732	135	**-1,26201557**	N4:665678	14,886	+8:YDR105C;	8			
chrIV	666941	667094	153	**-1,344306476**	N4:667026	8,532	-1:YDR105C; +1:YDR106W;	-1			
chrIV	666948	667109	161	**-1,589752762**	N4:667026	8,532	-1:YDR105C; +1:YDR106W;	-1			
chrIV	669497	669656	159	**-1,393388218**	N4:669574	13,11	+9:YDR107C;	9			
chrIV	669741	669906	165	**-1,663900623**	N4:669781	43,632	+8:YDR107C;	8			
chrIV	677555	677687	132	**-1,440701538**	N4:677564	10,672	+10:YDR110W;	10			
chrIV	682440	682595	155	**-1,099609367**	N4:682517	11,446	-1:YDR114C; +3:YDR115W;	-1			
chrIV	682928	683076	148	**-1,299709395**	N4:683045	23,424	+4:YDR116C;	4			
chrIV	684905	685053	148	**-1,37643622**	N4:684976	22,003	+5:YDR117C;	5			
chrIV	685367	685521	154	**-1,07353604**	N4:685462	7,659	+2:YDR117C;	2			
chrIV	685409	685539	130	**-1,174834812**	N4:685462	7,659	+2:YDR117C;	2			
chrIV	692976	693125	149	**-1,161819403**	N4:693039	17,201	+2:YDR120C;	2			
chrIV	699427	699543	116	**-1,206881546**	N4:699492	6,919	+1:YDR123C;	1			
chrIV	699429	699572	143	**-1,017853798**	N4:699492	6,919	+1:YDR123C;	1			
chrIV	701924	702080	156	**-1,445347585**	N4:702005	22,546	+5:YDR125C;	5			
chrIV	705337	705438	101	**-1,494147377**	N4:705421	25,314	+7:YDR127W;	7			
chrIV	705533	705696	163	**-1,269779883**				Overlap <50 bp			
chrIV	709396	709543	147	**-1,414813573**	N4:709492	29,663	0:YDR128W;	0			
chrIV	710394	710553	159	**-1,370386623**	N4:710486	41,504	+7:YDR128W;	7			
chrIV	710648	710758	110	**-0,487758835**	N4:710780	38,698	+9:YDR128W;	9			
chrIV	711680	711827	147	**-1,435110026**	N4:711712	33,139		Intergene			
chrIV	712887	713032	145	**-1,198220012**	N4:712928	16,376		Intergene			
chrIV	717311	717466	155	**-1,593478262**	N4:717376	7,19	+8:YDR131C;	8			
chrIV	723685	723835	150	**-1,267074106**				Overlap <50 bp			
chrIV	724692	724830	138	**-1,357829463**	N4:724752	43,68	+18:YDR135C; +16:SUT060;	18			
chrIV	727870	728018	148	**-1,344269525**	N4:727949	17,366	-1:YDR137W;	-1			
chrIV	729848	729970	122	**-1,29837542**	N4:729980	23,761	+12:YDR137W;	12			
chrIV	729848	729954	106	**-1,221131484**				Overlap <50 bp			
chrIV	730903	730997	94	**-1,177649955**	N4:730925	5,686	+3:YDR138W;	3			
chrIV	734743	734893	150	**-1,703921558**	N4:734827	15,305	+5*:YDR140W;	5	TERM		
chrIV	736895	737045	150	**-1,28468377**				Overlap <50 bp			
chrIV	737420	737548	128	**-1,112255558**	N4:737479	34,438	+16:YDR141C;	16			
chrIV	737638	737793	155	**-1,594408687**	N4:737705	21,498	+15:YDR141C;	15			
chrIV	745330	745443	113	**-1,569221438**	N4:745385	10,747	+6:YDR144C;	6			
chrIV	747582	747734	152	**-1,463098449**	N4:747675	7,174	+7:YDR145W;	7			
chrIV	750682	750850	168	**-1,58050291**	N4:750748	27,362	+2:YDR146C;	2			
chrIV	760658	760811	153	**-1,397555805**	N4:760826	15,436		Intergene			
chrIV	761833	761982	149	**-1,218845409**	N4:761970	22,612		Intergene			
chrIV	762005	762157	152	**-1,130067186**	N4:762143	11,314		Intergene			
chrIV	764493	764644	151	**-1,455314455**	N4:764585	8,06	+5:YDR151C;	5			
chrIV	766598	766749	151	**-1,203212325**	N4:766690	16,514	+10*:YDR153C;	10	TERM		
chrIV	768253	768411	158	**-1,607347271**	N4:768405	14,08	+4*:YDR155C;	4	TERM		
chrIV	771149	771299	150	**-1,178227072**				Overlap <50 bp			
chrIV	773973	774101	128	**-1,345804847**				Overlap <50 bp			
chrIV	779473	779647	174	**-1,849471933**	N4:779537	10,237	+4:YDR161W;	4			
chrIV	779988	780110	122	**-1,285242314**	N4:779996	18,284	+7:YDR161W;	7			
chrIV	785291	785441	150	**-1,696944994**	N4:785366	21,213	+4:YDR165W;	4			
chrIV	787217	787366	149	**-1,247100679**	N4:787358	22,528	+13:YDR166C;	13			
chrIV	787217	787366	149	**-1,247100679**	N4:787195	23,788	+14:YDR166C;	14			
chrIV	789145	789305	160	**-1,3336118**	N4:789230	19,692	+1:YDR166C; -1:YDR167W;	1			
chrIV	800500	800661	161	**-1,445438319**	N4:800519	14,064		Intergene			
chrIV	803067	803219	152	**-1,554898307**	N4:803114	40,951		Intergene			
chrIV	805417	805524	107	**-0,979370718**	N4:805529	17,01		Intergene			
chrIV	805417	805530	113	**-1,29302957**	N4:805529	17,01		Intergene			
chrIV	810559	810694	135	**-1,36235258**	N4:810661	7,668	+7*:YDR173C;	7	TERM		
chrIV	815236	815393	157	**-1,298327366**	N4:815317	26,988	+6:YDR176W;	6			
chrIV	815520	815678	158	**-1,659962576**	N4:815624	44,294	+8:YDR176W;	8			
chrIV	826418	826583	165	**-1,43380278**	N4:826491	11,286	+6:YDR181C;	6			
chrIV	827506	827652	146	**-0,991530057**	N4:827588	9,411	-1:YDR181C; +1:YDR182W;	-1			
chrIV	835382	835525	143	**-1,292362561**	N4:835504	19,705	+1:YDR186C;	1			
chrIV	835382	835528	146	**-1,2799835**	N4:835504	19,705	+1:YDR186C;	1			
chrIV	835383	835528	145	**-1,400809352**	N4:835504	19,705	+1:YDR186C;	1			
chrIV	835384	835528	144	**-1,235082832**	N4:835504	19,705	+1:YDR186C;	1			
chrIV	835385	835528	143	**-1,5864253**	N4:835504	19,705	+1:YDR186C;	1			
chrIV	835386	835528	142	**-1,880812245**	N4:835504	19,705	+1:YDR186C;	1			
chrIV	835932	836065	133	**-0,908233636**	N4:835972	14,114		Intergene			
chrIV	836520	836671	151	**-0,865853371**	N4:836563	5,541	+2:YDR188W;	2			
chrIV	837322	837477	155	**-1,25043489**	N4:837482	29,372	+8:YDR188W;	8			
chrIV	838477	838608	131	**-2,471460103**	N4:838553	7,195	+2:YDR189W;	2			
chrIV	843960	844136	176	**-1,617694818**	N4:844047	23,356	+6:YDR192C;	6			
chrIV	844482	844623	141	**-1,13846221**	N4:844544	2,63	+3:YDR192C;	3			
chrIV	844482	844649	167	**-1,52255302**	N4:844544	2,63	+3:YDR192C;	3			
chrIV	850466	850631	165	**-1,293353945**	N4:850636	8,841	+3:YDR196C;	3			
chrIV	850466	850631	165	**-1,293353945**	N4:850477	5,745	+4:YDR196C;	4			
chrIV	857181	857346	165	**-1,260794076**				Overlap <50 bp			
chrIV	861657	861796	139	**-1,25951816**	N4:861762	4,879	+1:YDR206W;	1			
chrIV	863651	863788	137	**-1,147617344**	N4:863721	31,07	+13:YDR206W;	13			
chrIV	866143	866295	152	**-1,023217713**	N4:866178	6,841	+9:YDR207C;	9			
chrIV	867460	867598	138	**-1,643394345**	N4:867531	9,783	+1:YDR207C;	1			
chrIV	870210	870347	137	**-1,276218809**	N4:870239	20,312	+15:YDR208W;	15			
chrIV	872409	872511	102	**-0,588837978**	N4:872520	29,28		Intergene			
chrIV	873832	873980	148	**-1,516638041**				Overlap <50 bp			

CHR	START	END	L(bp)	ΔLknuc	Nuc ID	Fuzziness	Gene ID	Gene body postion or intergene	Terminal	rDNA	Telomeric
chrIV	874621	874762	141	**-1,439702297**				Overlap <50 bp			
chrIV	875359	875502	143	**-1,120581077**	N4:875420	19,858		Intergene			
chrIV	876645	876791	146	**-1,307156374**	N4:876701	12,728		Intergene			
chrIV	877438	877587	149	**-1,397636453**	N4:877571	0,707		Intergene			
chrIV	878298	878451	153	**-1,651776053**	N4:878280	33,279		Intergene			
chrIV	878496	878645	149	**-1,675764605**	N4:878510	27,429		Intergene			
chrIV	879292	879438	146	**-1,15315166**	N4:879370	5,686		Intergene			
chrIV	881327	881484	157	**-1,475286737**	N4:881391	30,551		Intergene			
chrIV	881795	881956	161	**-1,365654221**				Overlap <50 bp			
chrIV	881975	882110	135	**-1,14359206**	N4:882066	27,301		Intergene			
chrIV	883013	883153	140	**-1,046501802**	N4:883091	39,887		Intergene			
chrIV	883019	883153	134	**-1,086239038**	N4:883091	39,887		Intergene			
chrIV	883431	883586	155	**-1,41285025**	N4:883463	24,132		Intergene			
chrIV	883562	883670	108	**-0,702786672**				Overlap <50 bp			
chrIV	883696	883848	152	**-1,374578115**	N4:883720	47,606		Intergene			
chrIV	884819	884969	150	**-1,242860796**	N4:884882	7,861	+2:YDR211W;	2			
chrIV	888390	888567	177	**-1,328871178**	N4:888453	33,49	+8:YDR212W;	8			
chrIV	889668	889827	159	**-1,669720174**	N4:889734	12,903	+2:YDR213W;	2			
chrIV	890834	890988	154	**-0,791781583**	N4:890967	31,753	+10:YDR213W;	10			
chrIV	891389	891533	144	**-0,835485106**	N4:891510	38,291	+13:YDR213W;	13			
chrIV	891986	892153	167	**-1,608359805**	N4:892055	27,537	+16:YDR213W; +3*:CUT515;	16			
chrIV	896334	896482	148	**-0,88067986**	N4:896422	15,438	+10:YDR216W;	10			
chrIV	897629	897776	147	**-1,286044597**	N4:897659	36,527	+17:YDR216W;	17			
chrIV	899028	899177	149	**-1,592654491**	N4:899049	18,159	+25*:YDR216W;	25	TERM		
chrIV	906466	906593	127	**-1,354777757**	N4:906545	17,068	+4:YDR219C;	4			
chrIV	907937	908075	138	**-1,70983835**				Overlap <50 bp			
chrIV	908538	908667	129	**-1,775390989**	N4:908599	35,786	+6*:SUT474; +9:YDR221W;	6	TERM		
chrIV	909620	909773	153	**-1,686271496**	N4:909630	14,849	-1:SUT474; +15*:YDR221W;	-1			
chrIV	915796	915980	184	**-1,486066447**	N4:915793	10,472	+3:YDR225W;	3			
chrIV	917617	917765	148	**-1,21107431**	N4:917653	10,498	+2:YDR227W;	2			
chrIV	924934	925100	166	**-0,917021194**	N4:925073	25,319	+3:YDR229W;	3			
chrIV	926425	926570	145	**-1,17948512**	N4:926519	11,437	+3:YDR231C;	3			
chrIV	927161	927289	128	**-0,472034977**	N4:927242	9,326	+1:YDR232W;	1			
chrIV	928961	929115	154	**-1,400149499**	N4:929074	12,458	+11:YDR232W;	11			
chrIV	930238	930383	145	**-1,300991281**	N4:930295	36,425	+1:YDR233C;	1			
chrIV	931111	931260	149	**-1,081827997**	N4:931170	10,602	+2:YDR234W;	2			
chrIV	933440	933600	160	**-1,726468871**	N4:933517	5,225	+1:YDR235W;	1			
chrIV	944365	944524	159	**-1,513022674**	N4:944467	19,672	+5:YDR240C;	5			
chrIV	944403	944557	154	**-1,379890931**	N4:944467	19,672	+5:YDR240C;	5			
chrIV	948002	948171	169	**-1,814066687**	N4:948157	42,823	+10:YDR242W;	10			
chrIV	949233	949387	154	**-0,819364007**	N4:949302	16,661	+7:YDR243C;	7			
chrIV	955015	955163	148	**-1,199178502**	N4:955072	27,826	+5*:YDR246W; +1:YDR246W-A;	5	TERM		
chrIV	957192	957317	125	**-0,854749929**	N4:957237	22,485	+10:YDR247W;	10			
chrIV	957192	957321	129	**-0,942615234**	N4:957237	22,485	+10:YDR247W;	10			
chrIV	957196	957298	102	**-0,395654796**	N4:957237	22,485	+10:YDR247W;	10			
chrIV	962120	962274	154	**-1,503690862**	N4:962102	30,697	+12:YDR251W;	12			
chrIV	962406	962555	149	**-1,216378209**	N4:962480	29,614	+14:YDR251W;	14			
chrIV	965842	965991	149	**-1,297042977**	N4:965942	7,211	+6:YDR254W;	6			
chrIV	968475	968604	129	**-1,217294172**	N4:968597	21,904	+4:anti039; +8:YDR256C;	4			
chrIV	971192	971329	137	**-1,153472476**	N4:971253	10,583	+2:YDR257C;	2			
chrIV	975387	975508	121	**-1,211154132**	N4:975430	23,039	+3:YDR259C;	3			
chrIV	979606	979759	153	**-1,361501555**	N4:979673	31,32	+2:Unit141;	2			
chrIV	980738	980889	151	**-1,314028889**	N4:980809	11,437	+1:SUT479;	1			
chrIV	981759	981861	102	**-0,556377537**	N4:981871	42,414		Intergene			
chrIV	983182	983330	148	**-1,489235912**				Overlap <50 bp			
chrIV	984709	984852	143	**-0,882634791**	N4:984756	11,504		Intergene			
chrIV	985995	986141	146	**-0,981612181**				Overlap <50 bp			
chrIV	986788	986937	149	**-1,071508726**	N4:986912	25,456		Intergene			
chrIV	987343	987492	149	**-1,362452466**	N4:987353	12,503		Intergene			
chrIV	987657	987771	114	**-1,285773884**	N4:987788	2,517		Intergene			
chrIV	988353	988503	150	**-1,793175299**	N4:988403	10,017		Intergene			
chrIV	989662	989806	144	**-1,367248348**	N4:989808	37		Intergene			
chrIV	989749	989906	157	**-1,386005362**	N4:989808	37		Intergene			
chrIV	990217	990378	161	**-1,623091229**	N4:990348	50,912		Intergene			
chrIV	990397	990532	135	**-1,327630588**	N4:990499	32,593		Intergene			
chrIV	991435	991575	140	**-1,145213344**	N4:991512	35,553		Intergene			
chrIV	991441	991575	134	**-0,962274359**	N4:991512	35,553		Intergene			
chrIV	991853	992008	155	**-1,29763554**	N4:991879	21,221		Intergene			
chrIV	991984	992092	108	**-0,766194109**	N4:992082	47,648		Intergene			
chrIV	992133	992282	149	**-1,478913425**				Overlap <50 bp			
chrIV	994735	994883	148	**-1,274640739**	N4:994783	34,313	+7:YDR263C;	7			
chrIV	1001383	1001529	146	**-1,410974783**	N4:1001542	5,315	+5:YDR266C;	5			
chrIV	1001383	1001529	146	**-1,410974783**	N4:1001379	8,802	+6:YDR266C;	6			
chrIV	1005517	1005664	147	**-1,183038915**	N4:1005589	6,994	+1:YDR270W;	1			
chrIV	1005876	1006022	146	**-1,206796155**	N4:1005930	12,922	+3:YDR270W;	3			
chrIV	1012282	1012417	135	**-1,078895653**	N4:1012368	27,109	+2:YDR275W;	2			
chrIV	1016514	1016662	148	**-0,967388928**	N4:1016582	30,801	+14:SUT065;	14			
chrIV	1019255	1019404	149	**-1,625001612**	N4:1019326	11,828	+1:YDR279W; -1:SUT480;	1			
chrIV	1021134	1021284	150	**-1,398208887**	N4:1021147	6,95	+4:YDR280W;	4			
chrIV	1021134	1021284	150	**-1,398208887**	N4:1021302	4,933	+5:YDR280W;	5			
chrIV	1025226	1025374	148	**-1,281731333**	N4:1025251	7,411		Intergene			
chrIV	1028806	1028950	144	**-1,207551937**	N4:1028803	22,345		Intergene			
chrIV	1039896	1040063	167	**-1,660125295**	N4:1039922	6,186	+2:YDR291W; -1:YDR289C;	2			
chrIV	1042109	1042252	143	**-1,249609016**	N4:1042173	23,619	+16:YDR291W;	16			
chrIV	1052004	1052154	150	**-1,010694186**	N4:1052080	8,55	+2:YDR294C;	2			
chrIV	1055603	1055756	153	**-1,279731933**	N4:1055666	5,707	+4*:YDR296W;	4	TERM		
chrIV	1058574	1058755	181	**-1,368076919**	N4:1058759	6,861	+2:YDR298C;	2			
chrIV	1058574	1058755	181	**-1,368076919**	N4:1058587	19,194	+3:YDR298C;	3			
chrIV	1063264	1063420	156	**-0,865698397**	N4:1063318	5,215	+1:YDR301W;	1			
chrIV	1066695	1066823	128	**-0,822100309**	N4:1066763	26,306	+21:YDR301W;	21			
chrIV	1075797	1075953	156	**-1,58549808**	N4:1075869	5,269	+1:YDR307W;	1			
chrIV	1079066	1079219	153	**-1,125259776**	N4:1079155	10,607	+7:YDR309C;	7			
chrIV	1079249	1079403	154	**-1,206042957**	N4:1079306	6,025	+6:YDR309C;	6			
chrIV	1080074	1080246	172	**-1,499505391**	N4:1080115	14,195	+1:YDR309C;	1			
chrIV	1090868	1091040	172	**-1,245002011**	N4:1090903	21,687	+12:YDR314C; +4:CUT079;	12			
chrIV	1091309	1091450	141	**-1,48421628**				Overlap <50 bp			

CHR	START	END	L(bp)	ΔLknuc	Nuc ID	Fuzziness	Gene ID	Gene body postion or intergene	Terminal	rDNA	Telomeric
chrIV	1096304	1096427	123	-1,242179707				Overlap <50 bp			
chrIV	1096404	1096559	155	-1,380357536	N4:1096525	34,962		Intergene			
chrIV	1096837	1096971	134	-1,253225439	N4:1096900	24,214		Intergene			
chrIV	1096837	1096977	140	-1,222702462	N4:1096900	24,214		Intergene			
chrIV	1097880	1098015	135	-1,221031054	N4:1097914	19,502		Intergene			
chrIV	1098034	1098195	161	-1,598648121	N4:1098134	26,458		Intergene			
chrIV	1098506	1098663	157	-1,105015426	N4:1098582	22,053		Intergene			
chrIV	1098997	1099126	129	-1,280463803				Overlap <50 bp			
chrIV	1099909	1100059	150	-1,237679791	N4:1100005	8,622		Intergene			
chrIV	1100669	1100827	158	-1,30434629	N4:1100752	28,219		Intergene			
chrIV	1102545	1102694	149	-1,230737537	N4:1102665	32,019	+4:YDR317W; +6:SUT481;	4			
chrIV	1102953	1103100	147	-1,841385305	N4:1102998	35,444	+6:YDR317W; +4:SUT481;	6			
chrIV	1107156	1107303	147	-1,404751627	N4:1107223	31,356	+7:YDR320C;	7			
chrIV	1109551	1109723	172	-1,390447929	N4:1109646	15,865	+7:YDR321W;	7			
chrIV	1112644	1112796	152	-1,382598612	N4:1112718	6,066	+9:YDR323C;	9			
chrIV	1117237	1117387	150	-1,013796475	N4:1117306	8,809	+2:YDR325W;	2			
chrIV	1121058	1121205	147	-1,357747123	N4:1121186	12,971		Intergene			
chrIV	1121801	1121948	147	-1,320931937	N4:1121812	30,534	+21*:YDR326C;	21	TERM		
chrIV	1124473	1124641	168	-1,637973632	N4:1124564	25,882	+4:YDR326C;	4			
chrIV	1128248	1128395	147	-1,283399164	N4:1128331	15,042	+4:YDR330W;	4			
chrIV	1137826	1137974	148	-1,343574184	N4:1137842	30,178	+13:YDR334W;	13			
chrIV	1140412	1140528	116	-1,458879409	N4:1140541	12,189	35W; +31*:YDR334W; -1:SUT067;	-1			
chrIV	1144636	1144783	147	-1,323722881	N4:1144674	21,217	-1:YDR336W;	-1			
chrIV	1154940	1155065	125	-1,129208036	N4:1155020	40,204	+7:YDR342C;	7			
chrIV	1155027	1155183	156	-1,155254882	N4:1155020	40,204	+7:YDR342C;	7			
chrIV	1155195	1155331	136	-0,998969773	N4:1155235	0,707	+6:YDR342C;	6			
chrIV	1158507	1158654	147	-1,140734364	N4:1158572	30,138	+8:SUT068;	8			
chrIV	1160332	1160457	125	-1,214566306	N4:1160332	0	+8:YDR343C;	8			
chrIV	1160419	1160575	156	-1,093249186	N4:1160485	38,371	+7:YDR343C;	7			
chrIV	1160587	1160723	136	-0,946640958	N4:1160638	19,092	+6:YDR343C;	6			
chrIV	1164215	1164366	151	-1,234746417	N4:1164258	25,502	+3:YDR345C; +11:SUT069;	3			
chrIV	1166734	1166868	134	-1,230065171	N4:1166854	16,183	+2:Unit103; +2:SUT070;	2			
chrIV	1169216	1169370	154	-1,359826604	N4:1169282	11,397	+2:YDR347W;	2			
chrIV	1170479	1170639	160	-1,172665911	N4:1170579	27,306	+10:YDR348C;	10			
chrIV	1170874	1171037	163	-1,530421618	N4:1171049	28,358	+7:YDR348C;	7			
chrIV	1170874	1171037	163	-1,530421618	N4:1170900	37,528	+8:YDR348C;	8			
chrIV	1171736	1171886	150	-1,409341001	N4:1171844	18,902	+2:YDR348C;	2			
chrIV	1175589	1175742	153	-1,466943524	N4:1175671	10,085		Intergene			
chrIV	1176500	1176647	147	-1,313180512	N4:1176576	19,271	+11:YDR350C;	11			
chrIV	1179339	1179509	170	-1,598893454	N4:1179429	15,384	+7:YDR351W;	7			
chrIV	1185766	1185913	147	-1,255694347	N4:1185835	4,93	+8*:YDR354W; +1:YDR356W;	8	TERM		
chrIV	1186635	1186765	130	-1,516892523	N4:1186749	15,681	+7:YDR356W;	7			
chrIV	1187842	1187997	155	-1,60281164	N4:1187892	47,06	+14:YDR356W;	14			
chrIV	1199637	1199781	144	-1,407776687	N4:1199716	15,283	+5:YDR363W;	5			
chrIV	1199946	1200093	147	-1,454907531	N4:1200030	33,208	+7:YDR363W;	7			
chrIV	1202429	1202587	158	-1,75263873	N4:1202473	7	+11*:YDR364C; +3*:YDR363W-A;	11	TERM		
chrIV	1205211	1205359	148	-1,269646068	N4:1205237	15,962	+8:YDR365C;	8			
chrIV	1207063	1207212	149	-1,093876745	N4:1207202	34,598		Intergene			
chrIV	1207237	1207360	123	-1,385145384				Overlap <50 bp			
chrIV	1207337	1207492	155	-1,446858693	N4:1207459	34,646		Intergene			
chrIV	1207770	1207904	134	-1,179991693	N4:1207827	23,065		Intergene			
chrIV	1207770	1207910	140	-1,084824366	N4:1207827	23,065		Intergene			
chrIV	1207966	1208117	151	-1,502669143	N4:1208017	24,062		Intergene			
chrIV	1207976	1208121	145	-1,421192634	N4:1208017	24,062		Intergene			
chrIV	1207994	1208145	151	-1,484614536	N4:1208017	24,062		Intergene			
chrIV	1207996	1208145	149	-1,388787762	N4:1208017	24,062		Intergene			
chrIV	1208248	1208402	154	-1,146588717	N4:1208380	21,502		Intergene			
chrIV	1208282	1208426	144	-1,066842954	N4:1208380	21,502		Intergene			
chrIV	1208813	1208948	135	-1,211399633	N4:1208846	3,786		Intergene			
chrIV	1208967	1209128	161	-1,362519076	N4:1209000	34,646		Intergene			
chrIV	1209439	1209596	157	-1,177219376	N4:1209520	25,58		Intergene			
chrIV	1209930	1210059	129	-1,478192145	N4:1210072	10,408		Intergene			
chrIV	1210842	1210992	150	-1,649101236	N4:1210937	4,243		Intergene			
chrIV	1211574	1211688	114	-1,385958414	N4:1211673	34,933		Intergene			
chrIV	1215599	1215745	146	-1,268995414	N4:1215661	31,31	+13:YDR369C;	13			
chrIV	1216176	1216327	151	-1,652756105				Overlap <50 bp			
chrIV	1217346	1217492	146	-1,412513411	N4:1217374	18,661	+3:YDR369C;	3			
chrIV	1218508	1218653	145	-1,288764769	N4:1218588	5,727	+4:YDR370C;	4			
chrIV	1221088	1221239	151	-1,570973339	N4:1221154	32,036	+9:YDR372C;	9			
chrIV	1225710	1225871	161	-1,481847672	N4:1225704	18,619	+6:YDR375C;	6			
chrIV	1225924	1226071	147	-0,905292488	N4:1225999	7,95	+4:YDR375C;	4			
chrIV	1227651	1227806	155	-1,663778754	N4:1227756	6,099	+7:YDR376W;	7			
chrIV	1238397	1238557	160	-1,37784321	N4:1238463	11,535	+4:YDR381C-A;	4			
chrIV	1240257	1240406	149	-1,314931113	N4:1240306	6,121	+2:CUT088; +5:YDR383C;	2			
chrIV	1240385	1240526	141	-1,308831282	N4:1240462	2,805	+3:CUT088; +4:YDR383C;	3			
chrIV	1244524	1244687	163	-1,30013175	N4:1244612	39,4	+9:YDR385W;	9			
chrIV	1245862	1245987	125	-1,097051488	N4:1245846	27,677	0:YDR386W; +17*:YDR385W;	0			
chrIV	1251539	1251695	156	-1,137182002	N4:1251612	11,384	+10*:YDR388W; -1:SUT486;	10	TERM		
chrIV	1259546	1259668	122	-1,279201745	N4:1259621	5,857	-1:YDR393W; +7:YDR392W;	-1			
chrIV	1265859	1266020	161	-1,377089085	N4:1265947	32,749	+17*:YDR395W;	17	TERM		
chrIV	1266398	1266553	155	-1,42106368	N4:1266476	8,524		Intergene			
chrIV	1267550	1267699	149	-1,314929408	N4:1267615	6,314	+2:YDR398W;	2			
chrIV	1269091	1269198	107	-1,086760565	N4:1269188	36,028	+12:YDR398W; +4:CUT528;	12			
chrIV	1269091	1269209	118	-1,217623014	N4:1269188	36,028	+12:YDR398W; +4:CUT528;	12			
chrIV	1272800	1272981	181	-1,726560067	N4:1272869	10,488	+6:YDR402C;	6			
chrIV	1277856	1277982	126	-1,465556501	N4:1277949	5,683	+3:YDR405W;	3			
chrIV	1278160	1278320	160	-1,303550916	N4:1278264	14,096	+5:YDR405W;	5			
chrIV	1287754	1287911	157	-1,001161007	N4:1287812	7,396	+2:YDR407C;	2			
chrIV	1288372	1288504	132	-0,84042479				Overlap <50 bp			
chrIV	1291651	1291799	148	-1,278329605	N4:1291819	23,549	+3:SUT490; +15:YDR409W;	3			
chrIV	1293635	1293774	139	-1,279514777	N4:1293718	2,658	+5:YDR411C;	5			
chrIV	1298059	1298223	164	-1,165141825	N4:1298152	26,529	+1:YDR415C; -1:YDR416W;	1			
chrIV	1300809	1300966	157	-1,394797029	N4:1300924	10,907	+17*:YDR416W; +1:CUT532;	17	TERM		
chrIV	1300809	1300970	161	-1,40182097	N4:1300924	10,907	+17*:YDR416W; +1:CUT532;	17	TERM		
chrIV	1300843	1301002	159	-1,128882862	N4:1300924	10,907	+17*:YDR416W; +1:CUT532;	17	TERM		
chrIV	1300844	1300970	126	-1,186655185	N4:1300924	10,907	+17*:YDR416W; +1:CUT532;	17	TERM		

CHR	START	END	L(bp)	**ΔLknuc**	Nuc ID	Fuzziness	Gene ID	Gene body postion or intergene	Terminal	rDNA	Telomeric
chrIV	1300844	1300987	143	**-1,546729534**	N4:1300924	10,907	+17*:YDR416W; +1:CUT532;	17	TERM		
chrIV	1302267	1302373	106	**-1,303502499**	N4:1302245	32,813	+5*:YDR418W;	5	TERM		
chrIV	1302267	1302383	116	**-1,412978415**	N4:1302245	32,813	+5*:YDR418W;	5	TERM		
chrIV	1308150	1308260	110	**-1,121372724**	N4:1308222	30,406		Intergene			
chrIV	1308150	1308309	159	**-1,263627541**	N4:1308222	30,406		Intergene			
chrIV	1308153	1308309	156	**-2,407150106**	N4:1308222	30,406		Intergene			
chrIV	1308185	1308314	129	**-1,34375978**	N4:1308222	30,406		Intergene			
chrIV	1308191	1308309	118	**-0,573116402**	N4:1308222	30,406		Intergene			
chrIV	1308196	1308309	113	**-0,990823111**	N4:1308222	30,406		Intergene			
chrIV	1308234	1308344	110	**-0,940517056**	N4:1308222	30,406		Intergene			
chrIV	1308280	1308442	162	**-1,034589053**	N4:1308370	24,749		Intergene			
chrIV	1308280	1308477	197	**-1,433198597**	N4:1308370	24,749		Intergene			
chrIV	1308478	1308605	127	**-0,334029733**	N4:1308546	31,711		Intergene			
chrIV	1308858	1308982	124	**-1,341140054**	N4:1308860	40,536		Intergene			
chrIV	1312653	1312780	127	**-1,321916089**	N4:1312676	20,705	+5:YDR421W;	5			
chrIV	1312778	1312924	146	**-1,339573388**	N4:1312823	27,048	+6:YDR421W;	6			
chrIV	1324502	1324680	178	**-1,257165089**	N4:1324612	12,518	0:YDR428C; +5*:YDR429C;	0			
chrIV	1324730	1324882	152	**-0,397433523**	N4:1324791	12,74	-1:YDR428C; +4:YDR429C;	-1			
chrIV	1329033	1329189	156	**-1,267920286**				Overlap <50 bp			
chrIV	1331269	1331431	162	**-1,026872837**	N4:1331328	17,002	+2:YDR434W;	2			
chrIV	1332261	1332409	148	**-1,396982393**	N4:1332317	20,971	+8:YDR434W;	8			
chrIV	1334150	1334293	143	**-1,129181834**	N4:1334177	16,61	-1:YDR435C;	-1			
chrIV	1334858	1335004	146	**-0,936310044**	N4:1334915	8,472	+3:YDR436W;	3			
chrIV	1335220	1335376	156	**-1,426561302**	N4:1335248	33,548	+5:YDR436W;	5			
chrIV	1335220	1335376	156	**-1,426561302**	N4:1335399	7,638	+6:YDR436W;	6			
chrIV	1337771	1337882	111	**0,107701928**	N4:1337823	5,913	+4:YDR437W;	4			
chrIV	1341919	1342069	150	**-1,128627307**	N4:1342003	3,688	+2:SUT492;	2			
chrIV	1354730	1354858	128	**-1,075013376**	N4:1354868	44,223		Intergene			
chrIV	1356736	1356889	153	**-1,668536338**				Overlap <50 bp			
chrIV	1359819	1359977	158	**-1,306578954**				Overlap <50 bp			
chrIV	1360674	1360822	148	**-1,150210204**	N4:1360796	29,792	-1:CUT096;	-1			
chrIV	1361305	1361420	115	**-1,001790655**	N4:1361443	11,927	+5:YDR451C;	5			
chrIV	1361305	1361412	107	**-0,978890425**	N4:1361296	13,609	+6:YDR451C;	6			
chrIV	1361305	1361420	115	**-1,001790655**	N4:1361296	13,609	+6:YDR451C;	6			
chrIV	1364336	1364488	152	**-1,153204869**	N4:1364372	15,073	+11:YDR452W;	11			
chrIV	1364512	1364671	159	**-1,374921696**	N4:1364657	14,818	+13*:YDR452W;	13	TERM		
chrIV	1365728	1365875	147	**-1,605996813**	N4:1365799	9,607	0:YDR453C;	0			
chrIV	1365746	1365909	163	**-1,169500437**	N4:1365799	9,607	0:YDR453C;	0			
chrIV	1367419	1367567	148	**-1,018619058**	N4:1367482	12,133	+1:YDR456W;	1			
chrIV	1367927	1368075	148	**-1,427036708**	N4:1367994	21,942	+4:YDR456W;	4			
chrIV	1372245	1372378	133	**-1,04382859**	N4:1372326	22,7		Intergene			
chrIV	1376748	1376898	150	**-1,676420309**	N4:1376802	22,55		Intergene			
chrIV	1382369	1382521	152	**-1,373429188**	N4:1382446	13,501	-1:YDR458C; +7:YDR459C;	-1			
chrIV	1387683	1387833	150	**-1,181751444**	N4:1387776	33,429	+7:YDR463W;	7			
chrIV	1390839	1391009	170	**-1,613104723**	N4:1390850	23,902	+14*:YDR464W;	14	TERM		
chrIV	1391435	1391580	145	**-1,2655942**	N4:1391591	9,68		Intergene			
chrIV	1391940	1392060	120	**-1,358456605**				Overlap <50 bp			
chrIV	1397500	1397655	155	**-1,247298266**				Overlap <50 bp			
chrIV	1398087	1398256	169	**-1,306912439**	N4:1398124	27,844	+5:YDR468C;	5			
chrIV	1406033	1406179	146	**-1,297647896**	N4:1406110	15,237	-1:YDR473C; +1:SUT075;	-1			
chrIV	1407803	1407943	140	**-1,273460181**	N4:1407944	22,59	+13:YDR475C; +12:SUT075;	13			
chrIV	1407803	1407943	140	**-1,273460181**	N4:1407780	16,765	+14:YDR475C; +11:SUT075;	14			
chrIV	1408060	1408202	142	**-1,276788755**	N4:1408111	21,788	+12:YDR475C; +13*:SUT075;	12			
chrIV	1408065	1408226	161	**-1,364439977**	N4:1408111	21,788	+12:YDR475C; +13*:SUT075;	12			
chrIV	1408340	1408489	149	**-1,235952361**				Overlap <50 bp			
chrIV	1409678	1409825	147	**-1,30147182**	N4:1409695	22,722	+4:YDR475C;	4			
chrIV	1413171	1413310	139	**-1,277423238**	N4:1413232	4,95	+8:YDR477W;	8			
chrIV	1420451	1420608	157	**-2,07211598**	N4:1420526	28,932	-1:YDR481C; +3:YDR482C;	-1			
chrIV	1420630	1420804	174	**-1,310192133**	N4:1420827	38,771	+1:YDR482C;	1			
chrIV	1420630	1420804	174	**-1,310192133**	N4:1420679	4,967	+2:YDR482C;	2			
chrIV	1420634	1420804	170	**-1,615403162**	N4:1420827	38,771	+1:YDR482C;	1			
chrIV	1420634	1420804	170	**-1,615403162**	N4:1420679	4,967	+2:YDR482C;	2			
chrIV	1424831	1424973	142	**-1,027281053**	N4:1424916	22,382	+17:YDR485C;	17			
chrIV	1428254	1428414	160	**-1,543886236**	N4:1428344	13,05	+5*:YDR487C; -1:YDR486C;	5	TERM		
chrIV	1430994	1431130	136	**-1,590789679**	N4:1431036	3,559	+1:YDR489W; -1:YDR488C;	1			
chrIV	1433662	1433813	151	**-1,562571203**	N4:1433787	23,266	+4:YDR490C;	4			
chrIV	1447757	1447907	150	**-1,173107294**	N4:1447835	10,405	+1:YDR499W;	1			
chrIV	1454001	1454153	152	**-1,299958983**	N4:1454073	16,281	+3:YDR502C;	3			
chrIV	1460987	1461146	159	**-1,241074773**	N4:1461050	8,39	+4:YDR506C;	4			
chrIV	1461681	1461828	147	**-1,264455085**	N4:1461801	44,636	-1:YDR506C;	-1			
chrIV	1462856	1462994	138	**-1,219373496**				Overlap <50 bp			
chrIV	1462967	1463131	164	**-1,178123503**	N4:1463035	24,28	+18:YDR507C;	18			
chrIV	1464532	1464669	137	**-1,298784607**	N4:1464622	35,517	+9:YDR507C;	9			
chrIV	1465805	1465959	154	**-1,28869406**	N4:1465878	10,237	+1:YDR507C;	1			
chrIV	1466131	1466277	146	**-1,340047467**	N4:1466195	16,068	0:SUT080;	0			
chrIV	1473970	1474117	147	**-1,394674278**	N4:1474065	31,316	+6:YDR515W;	6			
chrIV	1477488	1477647	159	**-1,281157312**	N4:1477526	8,981	+3:YDR517W;	3			
chrIV	1478568	1478697	129	**-1,364258552**	N4:1478612	15,796	+1:YDR518W;	1			
chrIV	1479228	1479388	160	**-1,226678961**	N4:1479283	15,192	+5:YDR518W;	5			
chrIV	1479727	1479884	157	**-1,243067692**	N4:1479790	8,746	+8*:YDR518W;	8	TERM		
chrIV	1481305	1481467	162	**-1,491295083**	N4:1481450	14,184	+13:YDR520C;	13			
chrIV	1489675	1489832	157	**-1,37933781**	N4:1489795	32,828	+1:YDR524C-B;	1			
chrIV	1495628	1495753	125	**-1,108187242**	N4:1495663	31,925	+10:YDR528W;	10			
chrIV	1496435	1496575	140	**-1,351387013**	N4:1496536	27,154	+9*:YDR530C; +1:YDR529C;	9	TERM		
chrIV	1497627	1497776	149	**-1,313031642**	N4:1497727	15,807	+2:YDR530C;	2			
chrIV	1498743	1498886	143	**-1,428867749**	N4:1498864	18,389	+5:YDR531W;	5			
chrIV	1499148	1499307	159	**-1,459238528**	N4:1499221	45,768	+7*:YDR531W;	7	TERM		
chrIV	1503439	1503593	154	**-1,256177469**	N4:1503589	15,156	+9:YDR534C;	9			
chrIV	1505128	1505252	124	**-0,663450371**	N4:1505200	17,186	-1:YDR534C;	-1			
chrIV	1507157	1507304	147	**-1,318227142**	N4:1507240	34,464		Intergene			
chrIV	1512418	1512571	153	**-1,511941655**	N4:1512551	9,899	+4:YDR539W;	4			
chrIV	1512829	1512960	131	**-1,076663242**	N4:1512870	31,852	+6:YDR539W;	6			
chrIV	1526039	1526194	155	**-1,360461567**	N4:1526079	28,919	-1:YDR545W;	-1			TEL RIGHT
chrIV	1527054	1527225	171	**-1,769058663**	N4:1527142	3,536	+6:YDR545W;	6			TEL RIGHT
chrIV	1527204	1527351	147	**-1,219791967**				Overlap <50 bp			TEL RIGHT
chrIV	1527360	1527490	130	**-1,068814589**	N4:1527407	23,065	+8:YDR545W;	8			TEL RIGHT

CHR	START	END	L(bp)	ΔLknuc	Nuc ID	Fuzziness	Gene ID	Gene body postion or intergene	Terminal	rDNA	Telomeric
chrIV	1527741	1527877	136	**-1,307047804**	N4:1527764	0	+10:YDR545W;	10			TEL RIGHT
chrIV	1528122	1528284	162	**-1,393786404**	N4:1528239	10,97	+13:YDR545W;	13			TEL RIGHT
chrIV	1528223	1528378	155	**-1,87269017**	N4:1528239	10,97	+13:YDR545W;	13			TEL RIGHT
chrIV	1528357	1528494	137	**-1,121803696**	N4:1528410	10,693	+14:YDR545W;	14			TEL RIGHT
chrIV	1528652	1528795	143	**-1,551961505**	N4:1528689	11,358	+16:YDR545W;	16			TEL RIGHT
chrIV	1528700	1528825	125	**-0,803983472**	N4:1528689	11,358	+16:YDR545W;	16			TEL RIGHT
chrIV	1528925	1529077	152	**-1,353584297**	N4:1529021	12,423	+18:YDR545W;	18			TEL RIGHT
chrIV	1528998	1529142	144	**-1,152820753**	N4:1529021	12,423	+18:YDR545W;	18			TEL RIGHT
chrIV	1529095	1529230	135	**-1,010144152**	N4:1529177	23,388	+19:YDR545W;	19			TEL RIGHT
chrIV	1529095	1529282	187	**-0,855284919**	N4:1529177	23,388	+19:YDR545W;	19			TEL RIGHT
chrIV	1529138	1529230	92	**-0,439262005**	N4:1529177	23,388	+19:YDR545W;	19			TEL RIGHT
chrIV	1529138	1529282	144	**-0,977153589**	N4:1529177	23,388	+19:YDR545W;	19			TEL RIGHT
chrIV	1529477	1529629	152	**-1,311269828**	N4:1529532	30,172	+21:YDR545W;	21			TEL RIGHT
chrIV	1529778	1529908	130	**-1,260262391**	N4:1529917	35,341	+23:YDR545W;	23			TEL RIGHT
chrIV	1529916	1530077	161	**-1,426708642**	N4:1529917	35,341	+23:YDR545W;	23			TEL RIGHT
chrIV	1530973	1531125	152	**-1,537106359**	N4:1531041	9,899	+30:YDR545W;	30			TEL RIGHT
chrIV	1531313	1531464	151	**-1,227070303**	N4:1531365	42,454	+32:YDR545W;	32			TEL RIGHT
chrIV	1531313	1531484	171	**-1,547343973**	N4:1531365	42,454	+32:YDR545W;	32			TEL RIGHT
chrIX	1597	1749	152	**-1,19285005**	N9:1629	28,006	+29:YIL177C;	29			TEL LEFT
chrIX	1686	1802	116	**-0,514595173**	N9:1790	44,523	+28:YIL177C;	28			TEL LEFT
chrIX	1686	1821	135	**-0,780811757**	N9:1790	44,523	+28:YIL177C;	28			TEL LEFT
chrIX	1686	1838	152	**-1,270768931**	N9:1790	44,523	+28:YIL177C;	28			TEL LEFT
chrIX	1710	1802	92	**-0,511637481**	N9:1790	44,523	+28:YIL177C;	28			TEL LEFT
chrIX	1710	1811	101	**-0,829794123**	N9:1790	44,523	+28:YIL177C;	28			TEL LEFT
chrIX	1710	1821	111	**-0,472629278**	N9:1790	44,523	+28:YIL177C;	28			TEL LEFT
chrIX	1710	1838	128	**-0,833855878**	N9:1790	44,523	+28:YIL177C;	28			TEL LEFT
chrIX	1710	1857	147	**-1,203879749**	N9:1790	44,523	+28:YIL177C;	28			TEL LEFT
chrIX	1746	1838	92	**-0,982758461**	N9:1790	44,523	+28:YIL177C;	28			TEL LEFT
chrIX	2215	2352	137	**-1,09552453**	N9:2320	18,385	+24:YIL177C;	24			TEL LEFT
chrIX	2382	2529	147	**-1,219056028**	N9:2535	41,012	+23:YIL177C;	23			TEL LEFT
chrIX	2382	2530	148	**-1,234116981**	N9:2535	41,012	+23:YIL177C;	23			TEL LEFT
chrIX	2382	2592	210	**-1,504650127**	N9:2535	41,012	+23:YIL177C;	23			TEL LEFT
chrIX	2383	2530	147	**-1,744892777**	N9:2535	41,012	+23:YIL177C;	23			TEL LEFT
chrIX	2385	2530	145	**-1,552370664**	N9:2535	41,012	+23:YIL177C;	23			TEL LEFT
chrIX	2397	2530	133	**-0,974052269**	N9:2535	41,012	+23:YIL177C;	23			TEL LEFT
chrIX	2437	2530	93	**-0,756768789**	N9:2535	41,012	+23:YIL177C;	23			TEL LEFT
chrIX	2437	2592	155	**-1,254118621**	N9:2535	41,012	+23:YIL177C;	23			TEL LEFT
chrIX	2558	2688	130	**-0,952862903**	N9:2535	41,012	+23:YIL177C;	23			TEL LEFT
chrIX	2879	3032	153	**-1,293194708**	N9:2921	22,301	+21:YIL177C;	21			TEL LEFT
chrIX	3324	3468	144	**-1,166378597**	N9:3423	11,533	+18:YIL177C;	18			TEL LEFT
chrIX	3531	3658	127	**-1,10511893**	N9:3591	6,028	+17:YIL177C;	17			TEL LEFT
chrIX	4088	4243	155	**-1,788390823**	N9:4203	6,807	+13:YIL177C;	13			TEL LEFT
chrIX	4805	4968	163	**-1,551739927**	N9:4855	10,504	+9:YIL177C;	9			TEL LEFT
chrIX	5216	5367	151	**-1,170904377**				Overlap <50 bp			TEL LEFT
chrIX	5429	5588	159	**-1,276265315**	N9:5453	17,243	+5:YIL177C;	5			TEL LEFT
chrIX	9290	9441	151	**-1,340792373**	N9:9376	3,055	-1:YIL176C;	-1			
chrIX	10228	10371	143	**-1,0729291**	N9:10309	5,292		Intergene			
chrIX	11460	11611	151	**-1,40406829**	N9:11554	17,898	+2:YIL173W;	2			
chrIX	12345	12490	145	**-1,126933457**				Overlap <50 bp			
chrIX	12910	13059	149	**-1,360544233**				Overlap <50 bp			
chrIX	14208	14357	149	**-1,045742171**	N9:14236	56,964	+19:YIL173W;	19			
chrIX	16078	16185	107	**-1,555620992**	N9:16064	4,041	+30:YIL173W;	30			
chrIX	25656	25806	150	**-1,434893897**	N9:25676	26,163	+19:anti079; +3:YIL169C;	19			
chrIX	32330	32492	162	**-1,150972996**	N9:32393	15,629	+2:YIL166C; +8:anti080;	2			
chrIX	33763	33917	154	**-1,447263707**	N9:33762	35,155	+8*:YIL164C; +3:YIL165C;	8	TERM		
chrIX	35803	35935	132	**-0,854739376**	N9:35809	22,95	+2:SUT602; +5:anti081;	2			
chrIX	41284	41431	147	**-1,243953704**	N9:41356	6,94	+2:YIL160C; +7:SUT177;	2			
chrIX	48410	48570	160	**-1,015165438**	N9:48477	19,201	+3:YIL156W;	3			
chrIX	55336	55486	150	**-0,94436784**	N9:55401	7,95	+2:YIL153W;	2			
chrIX	55497	55655	158	**-1,306580637**	N9:55554	14,592	+3:YIL153W;	3			
chrIX	55498	55655	157	**-1,42961383**	N9:55554	14,592	+3:YIL153W;	3			
chrIX	55500	55655	155	**-1,689362206**	N9:55554	14,592	+3:YIL153W;	3			
chrIX	56537	56665	128	**-1,458850015**	N9:56598	12,734	+1:YIL152W;	1			
chrIX	56786	56893	107	**-1,081763297**	N9:56789	33,126	+2:YIL152W;	2			
chrIX	56786	56905	119	**-1,283552102**	N9:56789	33,126	+2:YIL152W;	2			
chrIX	56791	56905	114	**-1,007944781**	N9:56789	33,126	+2:YIL152W;	2			
chrIX	59003	59166	163	**-1,490248005**	N9:59158	26,885	+11:YIL151C;	11			
chrIX	59003	59166	163	**-1,490248005**	N9:58990	17,349	+12:YIL151C;	12			
chrIX	59591	59741	150	**-1,20306081**	N9:59671	26,95	+8:YIL151C;	8			
chrIX	60731	60881	150	**-1,431779436**	N9:60802	2,229	+1:YIL151C;	1			
chrIX	62677	62823	146	**-1,277875339**	N9:62723	10,226	+1:YIL150C;	1			
chrIX	70968	71118	150	**-1,494228197**	N9:71141	30,666	+16:YIL147C;	16			
chrIX	77046	77204	158	**-1,300750076**	N9:77127	9,75	+2:YIL145C;	2			
chrIX	78123	78268	145	**-1,175037582**	N9:78189	4,69	+2:YIL144W;	2			
chrIX	80413	80548	135	**-1,149998527**	N9:80482	19,858		Intergene			
chrIX	89142	89281	139	**-1,314422241**	N9:89221	22,687	+4:YIL138C;	4			
chrIX	91580	91733	153	**-1,209591733**	N9:91642	18,061	+8:YIL137C;	8			
chrIX	93328	93477	149	**-1,010740899**	N9:93388	13,92	-1:YIL136W; -1:Unit327;	-1			
chrIX	96377	96489	112	**-1,60214521**	N9:96391	20,798	+1:YIL135C; +4*:YIL134C-A;	1			
chrIX	97100	97255	155	**-1,657090971**	N9:97083	46,769	-1:YIL134C-A;	-1			
chrIX	101159	101314	155	**-1,496745126**	N9:101310	9,055	+7:YIL131C;	7			
chrIX	101159	101314	155	**-1,496745126**	N9:101155	12,3	+8:YIL131C;	8			
chrIX	109382	109534	152	**-1,272202779**	N9:109439	6,5	+23:YIL129C;	23			
chrIX	122918	123072	154	**-1,512403055**	N9:122984	29,558	+4:YIL125W;	4			
chrIX	123420	123580	160	**-1,469104963**	N9:123402	18,464	+7:YIL125W;	7			
chrIX	123420	123580	160	**-1,469104963**	N9:123567	8,963	+8:YIL125W;	8			
chrIX	123668	123818	150	**-1,422071678**	N9:123750	15,526	+9:YIL125W;	9			
chrIX	127761	127867	106	**-1,181987067**	N9:127744	12,66		Intergene			
chrIX	127761	127880	119	**-1,283622363**	N9:127744	12,66		Intergene			
chrIX	129250	129396	146	**-1,023593811**	N9:129359	30,164	+8:YIL123W;	8			
chrIX	132115	132265	150	**-1,337879065**	N9:132195	23,529	0:YIL121W;	0			
chrIX	140470	140605	135	**-1,48675792**	N9:140507	18,886	+6*:YIL118W;	6	TERM		
chrIX	140794	140945	151	**-1,445811521**	N9:140849	18,093	+6*:YIL117C;	6	TERM		
chrIX	145476	145609	133	**-0,878135101**				Overlap <50 bp			
chrIX	145755	145902	147	**-1,435251811**	N9:145791	22,42	+19:YIL115C;	19			
chrIX	149396	149568	172	**-1,480252043**	N9:149494	5,701	+4:YIL114C;	4			

CHR	START	END	L(bp)	ΔLknuc	Nuc ID	Fuzziness	Gene ID	Gene body postion or intergene	Terminal	rDNA	Telomeric
chrIX	150158	150305	147	**-1,343166142**	N9:150234	4,733	-1:YIL114C; -1:YIL113W;	-1			
chrIX	154400	154561	161	**-1,363998438**	N9:154551	18,708	+3:anti083;	3			
chrIX	157942	158056	114	**-1,088522929**	N9:157996	21,695		Intergene			
chrIX	158500	158651	151	**-1,251719636**				Overlap <50 bp			
chrIX	159528	159680	152	**-1,138727302**	N9:159539	7,127	+5:YIL109C;	5			
chrIX	160370	160515	145	**-1,217825291**	N9:160446	9,899	-1:YIL109C; -1:YIL108W;	-1			
chrIX	164945	165092	147	**-1,182457529**	N9:165040	31,488	+6:YIL107C;	6			
chrIX	167654	167783	129	**-1,064205949**				Overlap <50 bp			
chrIX	170117	170264	147	**-1,199911999**	N9:170186	8,017	+9:YIL104C;	9			
chrIX	175448	175623	175	**-1,18692502**	N9:175501	8,562	+13:YIL101C; +2:anti085;	13			
chrIX	175932	176081	149	**-1,245524268**	N9:175976	6,656	+10:YIL101C; +5:anti085;	10			
chrIX	176147	176286	139	**-1,160200596**	N9:176155	18,281	+9:YIL101C; +6:anti085;	9			
chrIX	178557	178730	173	**-1,111746071**	N9:178691	35,197	+5:YIL099W;	5			
chrIX	182864	183017	153	**-1,206324586**	N9:182946	10,559	+2:YIL096C;	2			
chrIX	190132	190312	180	**-1,63757736**	N9:190192	25,517	+8:YIL092W;	8			
chrIX	190978	191126	148	**-1,269345296**	N9:191107	40,441	+13*:YIL092W; +14*:YIL091C;	13	TERM		
chrIX	192456	192591	135	**-1,123866091**	N9:192542	16,546	+5:YIL091C;	5			
chrIX	192983	193089	106	**-1,407425886**	N9:193029	7,026	+2:YIL091C;	2			
chrIX	192983	193098	115	**-1,379278188**	N9:193029	7,026	+2:YIL091C;	2			
chrIX	193702	193858	156	**-1,287039113**	N9:193815	11,619	+3:YIL090W;	3			
chrIX	196790	196938	148	**-1,126062896**	N9:196869	32,578	+8*:YIL089W;	8	TERM		
chrIX	200251	200397	146	**-1,553507125**	N9:200295	31,754	0:YIL087C;	0			
chrIX	205441	205561	120	**-1,228780623**				Overlap <50 bp			
chrIX	208655	208802	147	**-1,146288011**	N9:208742	9,394		Intergene			
chrIX	212863	213023	160	**-1,269621762**	N9:212982	26,996	+4:YIL078W;	4			
chrIX	213578	213684	106	**-0,850217553**				Overlap <50 bp			
chrIX	213578	213686	108	**-0,800870433**				Overlap <50 bp			
chrIX	213578	213701	123	**-1,191552165**				Overlap <50 bp			
chrIX	213866	214017	151	**-1,642303779**	N9:213871	32,025	+9:YIL078W;	9			
chrIX	213866	214017	151	**-1,642303779**	N9:214029	15,819	+10:YIL078W;	10			
chrIX	217876	218011	135	**-0,888586327**	N9:217888	17,138	+18*:YIL075C;	18	TERM		
chrIX	218740	218900	160	**-1,346679022**	N9:218850	30,111	+12:YIL075C;	12			
chrIX	223366	223481	115	**-1,505521161**	N9:223420	12,287	+4:SUT183; +17:YIL073C;	4			
chrIX	224421	224561	140	**-1,345669602**	N9:224566	36,238	+10:YIL073C;	10			
chrIX	236168	236319	151	**-1,008775557**	N9:236230	10,784	+10:YIL067C;	10			
chrIX	240288	240448	160	**-1,287572037**	N9:240358	6,633	+3:YIL066C;	3			
chrIX	241031	241216	185	**-1,062089726**	N9:241153	13,773		Intergene			
chrIX	241349	241481	132	**-0,897409324**				Overlap <50 bp			
chrIX	244655	244803	148	**-1,264391587**	N9:244726	6,861	-1:YIL062C; +6:YIL061C;	-1			
chrIX	244820	244976	156	**-1,255243575**	N9:244898	3,05	+5:YIL061C;	5			
chrIX	244822	244983	161	**-1,224404438**	N9:244898	3,05	+5:YIL061C;	5			
chrIX	247146	247297	151	**-1,908432857**	N9:247208	15,362		Intergene			
chrIX	249643	249773	130	**-1,023043686**	N9:249728	29,438	+1:Unit338; +1:YIL056W;	1			
chrIX	254780	254919	139	**-1,536526198**	N9:254874	41,004	-1:YIL053W;	-1			
chrIX	255472	255626	154	**-1,218539025**	N9:255553	51,733		Intergene			
chrIX	255629	255772	143	**-1,277251977**	N9:255778	22,427	-1:Unit339;	-1			
chrIX	255629	255776	147	**-1,10792192**	N9:255778	22,427	-1:Unit339;	-1			
chrIX	258137	258291	154	**-1,870358384**	N9:258298	11,077	+1:YIL051C;	1			
chrIX	258137	258291	154	**-1,870358384**	N9:258138	25,962	+2:YIL051C;	2			
chrIX	263024	263196	172	**-0,786258258**	N9:263062	25,173		Intergene			
chrIX	265122	265273	151	**-1,111649668**	N9:265235	27,545	+17:YIL047C;	17			
chrIX	265238	265355	117	**-1,936674668**	N9:265235	27,545	+17:YIL047C;	17			
chrIX	270073	270226	153	**-1,240937102**	N9:270153	16,633	+11:YIL046W;	11			
chrIX	271691	271837	146	**-1,385880416**	N9:271766	24,031	+5:YIL045W;	5			
chrIX	280995	281156	161	**-1,133865779**	N9:281073	36,927	+12:YIL038C; 0:anti088;	12			
chrIX	282582	282731	149	**-1,390150929**	N9:282678	15,398	+2:YIL038C;	2			
chrIX	283375	283539	164	**-1,610878615**	N9:283458	4,179	+3:SUT186; +10:YIL037C;	3			
chrIX	285840	285993	153	**-1,35765103**	N9:285843	8,198	+3:YIL036W;	3			
chrIX	285840	285993	153	**-1,35765103**	N9:286010	2,927	+4:YIL036W;	4			
chrIX	286233	286373	140	**-0,663978152**				Overlap <50 bp			
chrIX	287987	288121	134	**-1,395324736**	N9:288045	4,561	+6:YIL035C;	6			
chrIX	289697	289831	134	**-1,000710493**	N9:289737	11,367	+4:SUT187; +3:YIL034C;	4			
chrIX	289822	289971	149	**-1,353811109**	N9:289899	4,435	+5:SUT187; +2:YIL034C;	5			
chrIX	292476	292604	128	**-0,939002874**	N9:292512	11,983	+1:YIL031W;	1			
chrIX	296896	297052	156	**-1,36382984**	N9:297004	41,557	+19:YIL030C;	19			
chrIX	299139	299300	161	**-1,203494257**	N9:299210	11,236	+6:YIL030C;	6			
chrIX	301522	301672	150	**-1,30602044**	N9:301595	3,869	-1:YIL029C;	-1			
chrIX	301692	301851	159	**-1,409464246**	N9:301782	5,888		Intergene			
chrIX	301709	301857	148	**-1,219807588**	N9:301782	5,888		Intergene			
chrIX	306823	306944	121	**-1,25645718**	N9:306927	20,499	+7:YIL026C;	7			
chrIX	309339	309491	152	**-1,452785614**	N9:309417	6,261	+7*:YIL023C; 0:YIL024C;	7	TERM		
chrIX	309860	310002	142	**-1,1666166**	N9:309904	8,556	+4:YIL023C;	4			
chrIX	314424	314578	154	**-1,126397982**				Overlap <50 bp			
chrIX	320445	320583	138	**-1,352205055**	N9:320481	17,63	+4:YIL017C;	4			
chrIX	331176	331345	169	**-1,427806791**	N9:331307	44,998	+20:SUT189; +8:YIL013C;	20			
chrIX	332149	332296	147	**-1,279913126**	N9:332195	9,559	+3:YIL013C;	3			
chrIX	333705	333871	166	**-1,644484936**	N9:333780	13,301	+6:SUT613; +1:YIL011W;	6			
chrIX	338938	339077	139	**-0,895412862**	N9:338970	23,377	-1:Unit331; -1:YIL009W;	-1			
chrIX	339456	339607	151	**-1,091095368**	N9:339523	8,112	+2:YIL009W;	2			
chrIX	343521	343669	148	**-1,234215454**	N9:343647	10,922	+1:YIL007C;	1			
chrIX	345865	346018	153	**-0,63832697**	N9:345939	13,426	+3:YIL005W;	3			
chrIX	346400	346566	166	**-1,029617311**	N9:346455	20,253	+6:YIL005W;	6			
chrIX	352082	352233	151	**-1,647800673**	N9:352150	27,725	+9:YIL002C;	9			
chrIX	360987	361138	151	**-1,210703454**	N9:361056	4,438	+2:YIR003W;	2			
chrIX	361485	361638	153	**-1,042543361**	N9:361563	10,515	+5:YIR003W;	5			
chrIX	364237	364386	149	**-1,216916821**	N9:364329	13,916	+8:YIR004W;	8			
chrIX	365450	365589	139	**-1,217638974**	N9:365504	8,687	+28*:YIR006C;	28	TERM		
chrIX	371073	371217	144	**-1,179461302**	N9:371134	4,262	+3:YIR007W;	3			
chrIX	377621	377774	153	**-1,444311502**	N9:377728	9,663	+4:YIR011C;	4			
chrIX	378406	378536	130	**-1,10479358**	N9:378506	4,561	+1:YIR012W; -1:YIR011C;	1			
chrIX	380201	380307	106	**-1,441369505**	N9:380285	34,457	+2:YIR013C;	2			
chrIX	380201	380310	109	**-1,462718401**	N9:380285	34,457	+2:YIR013C;	2			
chrIX	381638	381781	143	**-1,490053572**	N9:381710	16,293	UT669; 0:YIR015W; +5*:YIR014W;	4			
chrIX	382286	382451	165	**-1,70319655**	N9:382299	17,578	:UT669; +4:YIR015W; -1:YIR016W;	-1			
chrIX	386066	386219	153	**-1,163326126**	N9:386093	32,897		Intergene			
chrIX	386767	386910	143	**-1,348305049**	N9:386861	26,153		Intergene			

CHR	START	END	L(bp)	ΔLknuc	Nuc ID	Fuzziness	Gene ID	Gene body postion or intergene	Terminal	rDNA	Telomeric
chrIX	387904	388014	110	-1,331240035	N9:387935	7,047	+1:Unit342;	1			
chrIX	388900	389051	151	-1,589890837	N9:389018	7,448	-1:SUT194;	-1			
chrIX	390177	390342	165	-1,351704484	N9:390256	34,664	+7:SUT194;	7			
chrIX	390386	390532	146	-1,277423677	N9:390414	30,376	+8:SUT194;	8			
chrIX	392788	392939	151	-1,079274103	N9:392855	31,658	+23:SUT194; +6*:YIR019C;	23			
chrIX	396135	396302	167	-1,350706304	N9:396197	15,385		Intergene			
chrIX	396795	396922	127	-1,239545508	N9:396865	11,996		Intergene			
chrIX	405278	405390	112	-1,510251564	N9:405412	16,415	+4:YIR026C;	4			
chrIX	407378	407518	140	-1,116357612	N9:407408	5,958	+3:YIR027C; +5:SUT195;	3			
chrIX	407520	407679	159	-1,472422306	N9:407581	5,02	+2:YIR027C; +6:SUT195;	2			
chrIX	409036	409181	145	-1,184197753	N9:409099	16,245	+7:SUT614; +5:YIR028W;	7			
chrIX	420519	420625	106	-1,493833426	N9:420590	6,863	+2:YIR034C;	2			
chrIX	425922	426069	147	-1,405452334	N9:426001	7,441		Intergene			
chrIX	433552	433722	170	-1,450340168	N9:433692	21,749	-1:YIR041W;	-1			
chrIX	438504	438651	147	-1,433835083	N9:438544	28,569		Intergene			
chrIX	438570	438711	141	-1,262801477				Overlap <50 bp			
chrIX	439579	439688	109	-1,10793233	N9:439641	36,222		Intergene			TEL RIGHT
chrIX	439796	439887	91	-1,086286073	N9:439845	9,539		Intergene			TEL RIGHT
chrV	1165	1320	155	-1,405729016				Overlap <50 bp			TEL LEFT
chrV	1854	1981	127	-0,494765301	N5:1841	19,33	+16:YEL077C;	16			TEL LEFT
chrV	3000	3144	144	-0,959470146	N5:3094	3,055	+8:YEL077C;	8			TEL LEFT
chrV	3648	3785	137	-1,296180049	N5:3709	8,66	+4:YEL077C;	4			TEL LEFT
chrV	3764	3919	155	-2,003651267	N5:3872	9,849	+3:YEL077C;	3			TEL LEFT
chrV	4024	4184	160	-1,282456513	N5:4031	6,557	+8*:YEL076C-A; +2:YEL077C;	8	TERM		TEL LEFT
chrV	4831	4982	151	-1,210534144	N5:4948	15,885	+2:YEL076C; +2:YEL076C-A;	2			TEL LEFT
chrV	7115	7272	157	-1,565806674	N5:7242	34,646	+4:YEL073C;	4			
chrV	9760	9930	170	-0,698113174				Overlap <50 bp			
chrV	11061	11170	109	-1,55023065	N5:11123	2,858	+2:SUT501;	2			
chrV	23046	23205	159	-1,22357103	N5:23195	30,03	+1*:YEL069C;	1	TERM		
chrV	26697	26869	172	-1,149432758	N5:26684	26,838	+1:YEL066W; -1:YEL067C;	1			
chrV	26697	26869	172	-1,149432758	N5:26878	19,396	+2:YEL066W;	2			
chrV	27053	27184	131	-0,905165587	N5:27040	24,698	+3*:YEL066W;	3	TERM		
chrV	28903	29024	121	-1,082490146	N5:29008	21,777	+9:YEL065W;	9			
chrV	32832	32992	160	-1,574941414	N5:32875	40,033	+5:YEL063C;	5			
chrV	41648	41795	147	-1,507225129	N5:41813	32,66	+2:YEL060C;	2			
chrV	41648	41797	149	-1,1411875	N5:41813	32,66	+2:YEL060C;	2			
chrV	41648	41798	150	-1,205541708	N5:41813	32,66	+2:YEL060C;	2			
chrV	41648	41799	151	-1,129014846	N5:41813	32,66	+2:YEL060C;	2			
chrV	41648	41800	152	-1,644917889	N5:41813	32,66	+2:YEL060C;	2			
chrV	41648	41801	153	-1,645272097	N5:41813	32,66	+2:YEL060C;	2			
chrV	41648	41802	154	-1,156441591	N5:41813	32,66	+2:YEL060C;	2			
chrV	41648	41754	106	-1,780909181	N5:41658	22,906	+3:YEL060C;	3			
chrV	41648	41774	126	-1,047649136	N5:41658	22,906	+3:YEL060C;	3			
chrV	41648	41782	134	-2,087017223	N5:41658	22,906	+3:YEL060C;	3			
chrV	41648	41785	137	-1,362538346	N5:41658	22,906	+3:YEL060C;	3			
chrV	41648	41795	147	-1,507225129	N5:41658	22,906	+3:YEL060C;	3			
chrV	41648	41797	149	-1,1411875	N5:41658	22,906	+3:YEL060C;	3			
chrV	41648	41798	150	-1,205541708	N5:41658	22,906	+3:YEL060C;	3			
chrV	41648	41799	151	-1,129014846	N5:41658	22,906	+3:YEL060C;	3			
chrV	41648	41800	152	-1,644917889	N5:41658	22,906	+3:YEL060C;	3			
chrV	41648	41801	153	-1,645272097	N5:41658	22,906	+3:YEL060C;	3			
chrV	41648	41802	154	-1,156441591	N5:41658	22,906	+3:YEL060C;	3			
chrV	41649	41802	153	-1,713590505	N5:41813	32,66	+2:YEL060C;	2			
chrV	41649	41802	153	-1,713590505	N5:41658	22,906	+3:YEL060C;	3			
chrV	41650	41802	152	-1,781225545	N5:41813	32,66	+2:YEL060C;	2			
chrV	41650	41802	152	-1,781225545	N5:41658	22,906	+3:YEL060C;	3			
chrV	41651	41802	151	-1,518929443	N5:41813	32,66	+2:YEL060C;	2			
chrV	41651	41802	151	-1,518929443	N5:41658	22,906	+3:YEL060C;	3			
chrV	41652	41802	150	-1,309938999	N5:41813	32,66	+2:YEL060C;	2			
chrV	41652	41802	150	-1,309938999	N5:41658	22,906	+3:YEL060C;	3			
chrV	41653	41802	149	-1,557833551	N5:41813	32,66	+2:YEL060C;	2			
chrV	41653	41802	149	-1,557833551	N5:41658	22,906	+3:YEL060C;	3			
chrV	41654	41802	148	-1,615267201	N5:41813	32,66	+2:YEL060C;	2			
chrV	41654	41802	148	-1,615267201	N5:41658	22,906	+3:YEL060C;	3			
chrV	41658	41802	144	-1,857621742	N5:41813	32,66	+2:YEL060C;	2			
chrV	41658	41802	144	-1,857621742	N5:41658	22,906	+3:YEL060C;	3			
chrV	42773	42909	136	-1,165453148	N5:42882	7,448	-1:YEL059C-A;	-1			
chrV	44013	44174	161	-1,187848156	N5:44097	21,735	+6:YEL058W;	6			
chrV	51922	52070	148	-1,096922028	N5:52074	19,837	0:CUT543;	0			
chrV	56549	56680	131	-1,058590095	N5:56569	10,147	+1:YEL052W;	1			
chrV	58655	58795	140	-1,068121843	N5:58725	20,849	+3:YEL051W;	3			
chrV	63504	63668	164	-1,378907037	N5:63566	28,732	0:YEL049W; +2:CUT546;	0			
chrV	64024	64155	131	-1,33630923	N5:64053	24,166	+3:YEL049W;	3			
chrV	65475	65628	153	-1,237339626	N5:65581	5,119	+8:YEL047C;	8			
chrV	69437	69601	164	-1,159649181	N5:69487	41,142	-1:YEL044W;	-1			
chrV	71549	71648	99	-1,379388459	N5:71615	9,968	+9:YEL043W;	9			
chrV	72028	72188	160	-1,409697122	N5:72094	8,562		Intergene			
chrV	78159	78308	149	-1,104142081	N5:78154	26,666	+1:YEL040W;	1			
chrV	78159	78308	149	-1,104142081	N5:78324	39,084	+2:YEL040W;	2			
chrV	78447	78544	97	-1,153851563	N5:78484	28,766	+3:YEL040W;	3			
chrV	78782	78922	140	-1,107250967	N5:78847	12,641	+5:YEL040W;	5			
chrV	79095	79249	154	-1,176006052	N5:79182	9,43	+7:YEL040W;	7			
chrV	83616	83770	154	-1,169693679	N5:83673	9,737	+7:YEL036C;	7			
chrV	84397	84539	142	-1,04400915	N5:84467	1,472	+2:YEL036C;	2			
chrV	87005	87158	153	-1,002422033	N5:87089	11,777	+2:YEL032W;	2			
chrV	92533	92664	131	-1,161891156	N5:92614	23,554	+16:YEL031W;	16			
chrV	94783	94926	143	-1,091911591	N5:94888	17,871	+3:YEL030W;	3			
chrV	96167	96341	174	-0,965810655	N5:96189	45,96	+11:YEL030W;	11			
chrV	105359	105516	157	-1,676054623	N5:105459	20,025	+5:YEL025C;	5			
chrV	105504	105653	149	-0,844524669	N5:105613	9,011	+4:YEL025C;	4			
chrV	106502	106638	136	-1,339012408	N5:106562	16,709		Intergene			
chrV	112707	112854	147	-1,16041213	N5:112767	39,414	+10:YEL022W;	10			
chrV	114004	114167	163	-1,380709464	N5:114084	25,367	+18:YEL022W;	18			
chrV	116119	116272	153	-1,303927027	N5:116132	21,032	+1:YEL021W;	1			
chrV	116657	116785	128	-1,344624347	N5:116788	20,551	+5:YEL021W;	5			
chrV	119860	120019	159	-1,365154346	N5:119994	9,849	+3:YEL020C; +9:YEL019C;	3			

CHR	START	END	L(bp)	ΔLknuc	Nuc ID	Fuzziness	Gene ID	Gene body postion or intergene	Terminal	rDNA	Telomeric
chrV	121418	121572	154	**-1,211805922**	N5:121505	5,231	+1:YEL018W; -1:YEL019C;	1			
chrV	127589	127741	152	**-1,195535731**	N5:127639	34,888	+7:YEL015W;	7			
chrV	129771	129919	148	**-1,19688081**	N5:129920	19,722	+9:YEL013W;	9			
chrV	132213	132348	135	**-1,355209347**	N5:132209	5,086		Intergene			
chrV	134881	135033	152	**-1,498693151**	N5:134908	8,562	+4:SUT504; +12*:YEL011W;	4			
chrV	137057	137219	162	**-1,311213349**	N5:137135	14,152		Intergene			
chrV	137099	137245	146	**-1,237627772**	N5:137135	14,152		Intergene			
chrV	143216	143369	153	**-0,922303719**	N5:143330	30,179	+12:YEL007W;	12			
chrV	146039	146192	153	**-1,202559763**	N5:146118	13,967	+4:YEL005C;	4			
chrV	147368	147525	157	**-1,452076532**	N5:147443	6,113	+4:YEL004W;	4			
chrV	147862	148011	149	**-1,3070106**	N5:147935	7,259	+7:YEL004W; -1:YEL003W;	7			
chrV	150905	151028	123	**-1,479539424**	N5:150967	7,468	+5:CUT106; +1:YEL001C;	5			
chrV	154668	154823	155	**-1,529104692**	N5:154737	8,756	+8:YER001W;	8			
chrV	154964	155109	145	**-1,605098809**	N5:155071	18,679	+10:YER001W;	10			
chrV	156735	156886	151	**-1,693406271**	N5:156804	11,274	+1:YER002W;	1			
chrV	157741	157900	159	**-0,820126426**	N5:157806	25,965		Intergene			
chrV	158361	158517	156	**-1,457691927**	N5:158435	16,216		Intergene			
chrV	158999	159169	170	**-1,433168259**	N5:159105	7,396	+1*:YER003C;	1	TERM		
chrV	161114	161267	153	**-1,34370096**	N5:161151	18,777	+4:YER005W;	4			
chrV	162828	162978	150	**-1,387541174**	N5:162902	13,027	+2:YER006W;	2			
chrV	162990	163135	145	**-1,132688929**	N5:163066	4,461	+3:YER006W;	3			
chrV	163162	163326	164	**-1,209782358**	N5:163230	22,107	+4:YER006W;	4			
chrV	164105	164236	131	**-0,778678852**	N5:164174	27,019	-1:YER007W; +10:YER006W;	-1			
chrV	165644	165751	107	**-0,820742287**	N5:165694	6,38	+9:YER007W;	9			
chrV	165645	165751	106	**-1,131996043**	N5:165694	6,38	+9:YER007W;	9			
chrV	165645	165753	108	**-1,381562961**	N5:165694	6,38	+9:YER007W;	9			
chrV	165645	165770	125	**-1,148063114**	N5:165694	6,38	+9:YER007W;	9			
chrV	169801	169967	166	**-1,79464542**	N5:169892	22,692	+13:YER008C;	13			
chrV	170735	170894	159	**-1,456763496**	N5:170840	12,383	+7:YER008C;	7			
chrV	175783	175962	179	**-1,516190621**	N5:175893	20,008	+6:YER011W;	6			
chrV	177369	177538	169	**-1,406530292**	N5:177535	9,311	+1:SUT508; -1:YER012W;	1			
chrV	177369	177538	169	**-1,406530292**	N5:177381	24,234	+2:SUT508;	2			
chrV	177766	177914	148	**-1,482333537**	N5:177821	12,858	-1:SUT508; +1:YER012W;	-1			
chrV	179498	179607	109	**-2,015610091**	N5:179488	12,644	+5:YER013W;	5			
chrV	179498	179665	167	**-2,004173751**	N5:179488	12,644	+5:YER013W;	5			
chrV	179498	179666	168	**-1,63857675**	N5:179488	12,644	+5:YER013W;	5			
chrV	179498	179667	169	**-1,680425085**	N5:179488	12,644	+5:YER013W;	5			
chrV	179498	179669	171	**-1,75983774**	N5:179488	12,644	+5:YER013W;	5			
chrV	179498	179670	172	**-1,342618679**	N5:179488	12,644	+5:YER013W;	5			
chrV	179498	179665	167	**-2,004173751**	N5:179670	27,842	+6:YER013W;	6			
chrV	179498	179666	168	**-1,63857675**	N5:179670	27,842	+6:YER013W;	6			
chrV	179498	179667	169	**-1,680425085**	N5:179670	27,842	+6:YER013W;	6			
chrV	179498	179669	171	**-1,75983774**	N5:179670	27,842	+6:YER013W;	6			
chrV	179498	179670	172	**-1,342618679**	N5:179670	27,842	+6:YER013W;	6			
chrV	179499	179670	171	**-1,445092566**	N5:179488	12,644	+5:YER013W;	5			
chrV	179499	179670	171	**-1,445092566**	N5:179670	27,842	+6:YER013W;	6			
chrV	179502	179670	168	**-1,841093399**	N5:179488	12,644	+5:YER013W;	5			
chrV	179502	179670	168	**-1,841093399**	N5:179670	27,842	+6:YER013W;	6			
chrV	180478	180630	152	**-1,289395641**	N5:180475	42,543	+11:YER013W;	11			
chrV	180478	180630	152	**-1,289395641**	N5:180645	23,945	+12:YER013W;	12			
chrV	182865	183029	164	**-1,509791068**	N5:182948	7,554	+3:YER014W;	3			
chrV	186698	186836	138	**-1,050741078**	N5:186791	10,083	+13:YER015W;	13			
chrV	189568	189720	152	**-1,047244802**	N5:189634	26,397	+15:YER017C;	15			
chrV	190625	190773	148	**-1,321681522**	N5:190729	28,404	+8:YER017C;	8			
chrV	193660	193792	132	**-0,552565504**	N5:193731	8,106	+3:SUT510;	3			
chrV	194009	194135	126	**-0,885772981**				Overlap <50 bp			
chrV	194030	194163	133	**-1,094744788**	N5:194161	6,156	0:SUT510;	0			
chrV	195885	196040	155	**-1,10388581**	N5:195969	9,798	+6:YER020W;	6			
chrV	198265	198403	138	**-0,890763225**	N5:198244	16,025	+9:YER021W;	9			
chrV	201379	201531	152	**-1,31106074**	N5:201422	5,385	+3:YER023W;	3			
chrV	201380	201531	151	**-1,365803429**	N5:201422	5,385	+3:YER023W;	3			
chrV	201382	201531	149	**-1,291548225**	N5:201422	5,385	+3:YER023W;	3			
chrV	201383	201531	148	**-1,402498629**	N5:201422	5,385	+3:YER023W;	3			
chrV	206462	206572	110	**-1,438113353**				Overlap <50 bp			
chrV	207496	207646	150	**-1,51306086**	N5:207609	14,821	+7*:YER026C;	7	TERM		
chrV	212628	212767	139	**-1,137426022**				Overlap <50 bp			
chrV	213547	213709	162	**-1,41726739**	N5:213639	13,761	+2:YER030W;	2			
chrV	215300	215450	150	**-1,464001951**	N5:215374	8,961	+3:YER032W;	3			
chrV	220704	220853	149	**-1,619077269**	N5:220767	15,329	+4:YER033C;	4			
chrV	226712	226861	149	**-1,257169902**	N5:226775	12,078	038C; +7:YER037W; +18:YER039C;	10	TERM		
chrV	227455	227599	144	**-1,249784308**	N5:227578	17,421	+5:YER038C; +13:YER039C;	5			
chrV	234231	234385	154	**-1,111371683**	N5:234301	5,574		Intergene			
chrV	238011	238157	146	**-1,23343657**	N5:238087	10,45	+1:YER044C; +8*:YER044C-A;	1			
chrV	239144	239298	154	**-1,247910581**	N5:239207	7,887	+1:YER044C-A; +4*:CUT109;	1			
chrV	250567	250727	160	**-1,163035273**	N5:250698	7,688	+1:YER048W-A;	1			
chrV	250628	250757	129	**-1,072003738**	N5:250698	7,688	+1:YER048W-A;	1			
chrV	253010	253142	132	**-1,100009951**	N5:253162	32,519	+10:YER049W;	10			
chrV	255896	256035	139	**-1,352552539**	N5:256029	34,914	+9:YER051W;	9			
chrV	258267	258412	145	**-1,225289908**	N5:258341	18,228	-1:YER052C;	-1			
chrV	263170	263306	136	**-1,292138703**				Overlap <50 bp			
chrV	268138	268266	128	**-1,170559319**	N5:268214	8,386	0:YER056C;	0			
chrV	271754	271916	162	**-1,271015519**	N5:271765	7,679	+1:YER058W;	1			
chrV	273268	273395	127	**-1,050756748**	N5:273323	27,749	+6:YER059W;	6			
chrV	274395	274545	150	**-1,165681084**	N5:274471	20,294	+22:SUT514; +1:YER060W;	22			
chrV	279045	279197	152	**-1,465899569**	N5:279105	8,515	+4:YER061C;	4			
chrV	282333	282495	162	**-1,512285142**	N5:282372	25,461	+5*:YER063W; +1:CUT557;	5	TERM		
chrV	286305	286436	131	**-0,825699181**	N5:286328	14,009	+8:anti052; +6:YER065C;	8			
chrV	293237	293387	150	**-1,479895789**	N5:293318	9,973	+3:YER068W;	3			
chrV	293855	294005	150	**-1,228169228**	N5:293929	9,292	+7:YER068W;	7			
chrV	298879	299018	139	**-1,268345527**	N5:298928	15,218	+1:YER070W;	1			
chrV	299205	299328	123	**-1,748545952**	N5:299273	9,183	+3:YER070W;	3			
chrV	299678	299831	153	**-1,293653467**	N5:299784	13,809	+6:YER070W;	6			
chrV	301505	301647	142	**-1,224899935**	N5:301602	18,777	+17:YER070W; +1:CUT559;	17			
chrV	314667	314817	150	**-0,975536208**	N5:314812	27,626		Intergene			
chrV	315905	316063	158	**-1,237697138**	N5:315980	9,67	+4:YER077C;	4			
chrV	320077	320234	157	**-0,957392236**	N5:320131	14,134	+2:YER080W;	2			

CHR	START	END	L(bp)	ΔLknuc	Nuc ID	Fuzziness	Gene ID	Gene body postion or intergene	Terminal	rDNA	Telomeric
chrV	321656	321815	159	**-1,595969993**	N5:321662	22,068	+2*:CUT563; +12:YER080W;	2	TERM		
chrV	321656	321815	159	**-1,595969993**	N5:321825	38,767	+1:CUT563; +13*:YER080W;	1			
chrV	323513	323661	148	**-1,075349918**	N5:323606	6,557	+7:YER081W;	7			
chrV	324743	324895	152	**-1,410358804**	N5:324823	32,406	+8:YER082C;	8			
chrV	324904	325052	148	**-1,196926183**	N5:324975	31,489	+7:YER082C;	7			
chrV	325348	325499	151	**-1,445431373**	N5:325472	17,501	+4:YER082C;	4			
chrV	328966	329128	162	**-1,273102331**	N5:329104	18,91	+5:YER086W;	5			
chrV	331009	331174	165	**-1,348813496**	N5:331055	6,156	+4:YER087W;	4			
chrV	333088	333236	148	**-1,622644224**	N5:333122	14,975	-1:YER087C-B; +1*:Unit175;	-1			
chrV	340240	340377	137	**-1,159098468**	N5:340278	28,039	+12:YER091C;	12			
chrV	340798	340946	148	**-1,413410293**	N5:340808	15,143	+9:YER091C;	9			
chrV	341934	342080	146	**-1,148432865**				Overlap <50 bp			
chrV	349486	349586	100	**-1,040302446**				Overlap <50 bp			
chrV	351515	351672	157	**-1,552705746**	N5:351572	9,783	0:YER096W;	0			
chrV	352323	352478	155	**-1,476046162**	N5:352405	18,501	+6:YER096W;	6			
chrV	360556	360703	147	**-1,446553627**	N5:360635	27,381	+8*:YER101C;	8	TERM		
chrV	360556	360709	153	**-1,277684752**	N5:360635	27,381	+8*:YER101C;	8	TERM		
chrV	360596	360757	161	**-1,308263094**	N5:360635	27,381	+8*:YER101C;	8	TERM		
chrV	361101	361246	145	**-1,458577418**	N5:361124	17,146	+5:YER101C;	5			
chrV	364550	364707	157	**-1,422709887**	N5:364627	11,269	+2:YER103W;	2			
chrV	369752	369900	148	**-0,982879854**				Overlap <50 bp			
chrV	370967	371076	109	**-1,332652675**	N5:371022	7,234	+7:YER105C;	7			
chrV	372915	373052	137	**-1,472221282**	N5:373029	29,3	+5:YER106W;	5			
chrV	373805	373958	153	**-1,302935443**	N5:373887	10,967	+6:YER107C;	6			
chrV	376017	376164	147	**-1,290673883**	N5:376149	19,149	+11:YER109C;	11			
chrV	376371	376519	148	**-1,375841574**				Overlap <50 bp			
chrV	376832	376976	144	**-1,113571873**	N5:376938	5,252	+6:YER109C;	6			
chrV	377517	377651	134	**-1,204259516**	N5:377578	5,854	+2:YER109C;	2			
chrV	378722	378880	158	**-1,338800433**	N5:378804	19,408	+22:YER110C;	22			
chrV	389589	389739	150	**-1,39479508**	N5:389641	11,967	+4:YER113C;	4			
chrV	392068	392208	140	**-1,602776899**	N5:392146	36,065	+12:YER114C;	12			
chrV	408769	408926	157	**-1,205256794**	N5:408837	22,819	+2:YER124C;	2			
chrV	411385	411539	154	**-1,21231688**	N5:411472	26,414	+9:YER125W;	9			
chrV	421211	421362	151	**-1,177834881**	N5:421329	21,066	+8:YER130C;	8			
chrV	426798	426949	151	**-1,457116882**				Overlap <50 bp			
chrV	426821	426975	154	**-1,446654544**				Overlap <50 bp			
chrV	428076	428230	154	**-1,45927449**	N5:428133	20,599	+17:YER132C;	17			
chrV	429069	429212	143	**-0,946082725**				Overlap <50 bp			
chrV	434847	434995	148	**-1,292879224**	N5:434827	11,15		Intergene			
chrV	440738	440898	160	**-1,24324523**	N5:440906	46,309	+9:YER136W; +6:YER137C;	9			
chrV	444332	444446	114	**-1,484456338**	N5:444468	28,688		Intergene			
chrV	445028	445178	150	**-1,646320469**	N5:445075	14,503		Intergene			
chrV	446200	446349	149	**-0,953826865**	N5:446313	30,348		Intergene			
chrV	446424	446581	157	**-1,304288189**	N5:446484	31,66		Intergene			
chrV	446610	446760	150	**-1,031728129**	N5:446692	15,1		Intergene			
chrV	446614	446760	146	**-1,193634273**	N5:446692	15,1		Intergene			
chrV	447072	447207	135	**-0,909776363**	N5:447121	27,025		Intergene			
chrV	447594	447738	144	**-1,303305836**	N5:447622	21,733		Intergene			
chrV	447618	447772	154	**-1,156913567**	N5:447622	21,733		Intergene			
chrV	447875	448026	151	**-1,276266079**	N5:447970	17,098		Intergene			
chrV	447899	448044	145	**-1,062120308**	N5:447970	17,098		Intergene			
chrV	447903	448054	151	**-1,160645126**	N5:447970	17,098		Intergene			
chrV	448110	448250	140	**-1,187091515**	N5:448190	53,181		Intergene			
chrV	448116	448250	134	**-1,042788351**	N5:448190	53,181		Intergene			
chrV	448528	448683	155	**-0,800265026**	N5:448518	26,539		Intergene			
chrV	448659	448767	108	**-0,836065226**	N5:448761	30,86		Intergene			
chrV	448808	448957	149	**-1,291995728**				Overlap <50 bp			
chrV	450553	450702	149	**-1,498929923**	N5:450631	11,149	+5:YER139C;	5			
chrV	451474	451643	169	**-1,305773283**	N5:451553	3,777	+1:YER140W; -1:YER139C;	1			
chrV	456731	456859	128	**-1,582477679**	N5:456821	6,346	+4:YER143W;	4			
chrV	458282	458446	164	**-1,16316004**				Overlap <50 bp			
chrV	458920	459063	143	**-1,037636199**	N5:458935	35,138		Intergene			
chrV	458920	459063	143	**-1,037636199**	N5:459085	25,589		Intergene			
chrV	460202	460309	107	**-1,97004916**	N5:460198	15,639	+2*:YER144C;	2	TERM		
chrV	460202	460330	128	**-1,075262103**	N5:460198	15,639	+2*:YER144C;	2	TERM		
chrV	460206	460309	103	**-0,853250857**	N5:460198	15,639	+2*:YER144C;	2	TERM		
chrV	465104	465256	152	**-1,407236171**	N5:465150	10,521	+1:YER148W; -1:YER147C;	1			
chrV	466895	467059	164	**-1,349266676**	N5:466980	8,421	+4:YER149C;	4			
chrV	471982	472140	158	**-1,460037468**	N5:472119	11,718	+12:YER152C; +3:YER151C;	12			
chrV	472402	472551	149	**-1,365135805**	N5:472474	17,601	+10:YER152C; +1:YER151C;	10			
chrV	472672	472832	160	**-1,303675239**	N5:472741	8,085	+8:YER152C; -1:YER151C;	8			
chrV	472989	473145	156	**-1,115916989**	N5:473075	7,047	+6:YER152C;	6			
chrV	473919	474069	150	**-1,146365534**	N5:473967	10,572	+1:YER152C;	1			
chrV	481969	482118	149	**-1,306220774**				Overlap <50 bp			
chrV	488812	488981	169	**-1,233366554**	N5:488949	4,278		Intergene			
chrV	489802	489965	163	**-1,24163244**	N5:489855	28,19	0:SUT097; +6:YER158C;	0			
chrV	493537	493686	149	**-1,370183782**	N5:493561	30,616		Intergene			
chrV	495710	495860	150	**-1,01270983**	N5:495765	28,054		Intergene			
chrV	496172	496307	135	**-1,482903475**	N5:496222	62,83		Intergene			
chrV	497210	497350	140	**-1,222169136**	N5:497289	53,072		Intergene			
chrV	497216	497350	134	**-1,122360157**	N5:497289	53,072		Intergene			
chrV	497628	497783	155	**-1,235145567**	N5:497650	19,348		Intergene			
chrV	497759	497867	108	**-1,079909369**	N5:497866	50,013		Intergene			
chrV	497893	498045	152	**-1,592611579**				Overlap <50 bp			
chrV	501813	501975	162	**-1,228523236**	N5:501881	22,788	+9:YER162C;	9			
chrV	502108	502246	138	**-1,547012948**	N5:502208	10,183	+7:YER162C;	7			
chrV	503589	503721	132	**-1,089692801**	N5:503607	12,728	+2:YER163C;	2			
chrV	504504	504656	152	**-1,143423463**	N5:504574	15,604	+4:SUT098;	4			
chrV	506128	506280	152	**-1,292878026**	N5:506233	19,502	+8:YER164W;	8			
chrV	508434	508572	138	**-1,305140811**				Overlap <50 bp			
chrV	508812	508941	129	**-1,20027805**	N5:508869	22,167	+24:YER164W;	24			
chrV	517240	517402	162	**-1,33571515**	N5:517367	9,813		Intergene			
chrV	522575	522735	160	**-1,627618347**	N5:522650	3,983	+1:YER168C;	1			
chrV	525612	525757	145	**-1,293545217**	N5:525693	10,443	+17:YER169W; -1:YER170W;	17			
chrV	528789	528933	144	**-1,391505336**	N5:528845	21,927	+12:YER171W; +37:YER169W;	12			
chrV	536553	536715	162	**-1,369931942**	N5:536626	4,792	+3:YER173W;	3			

CHR	START	END	L(bp)	ΔLknuc	Nuc ID	Fuzziness	Gene ID	Gene body postion or intergene	Terminal	rDNA	Telomeric
chrV	538015	538173	158	**-1,422613978**	N5:538060	16,486	+12:YER173W;	12			
chrV	540818	540968	150	**-1,556260148**	N5:540902	24,345	+3*:YER175W-A; +4:anti054;	3	TERM		
chrV	541050	541210	160	**-1,264164375**	N5:541230	5,568	+2:anti054;	2			
chrV	541050	541210	160	**-1,264164375**	N5:541076	6,427	+3:anti054;	3			
chrV	541616	541757	141	**-1,213651428**	N5:541694	3,869	-1:anti054; +1:YER176W;	-1			
chrV	541626	541757	131	**-0,759951749**	N5:541694	3,869	-1:anti054; +1:YER176W;	-1			
chrV	541626	541777	151	**-1,09133325**	N5:541694	3,869	-1:anti054; +1:YER176W;	-1			
chrV	542716	542874	158	**-1,825210392**	N5:542824	35,576	+8:YER176W;	8			
chrV	544584	544725	141	**-1,210300792**	N5:544632	19,98	+19:YER176W; +3*:CUT571;	19			
chrV	560594	560752	158	**-1,31177382**	N5:560681	13,292	+1:Unit200; +3:SUT522;	1			
chrV	560787	560934	147	**-1,125255844**	N5:560849	6,911	+2:Unit200; +2:SUT522;	2			
chrV	561033	561174	141	**-1,16344903**	N5:561031	2,563	+3*:Unit200; +1:SUT522;	3	TERM		
chrV	561033	561174	141	**-1,16344903**	N5:561188	9,879	0:SUT522;	0			
chrV	571735	571876	141	**-1,143998579**	N5:571819	39,305	+3:YER190W;	3			TEL RIGHT
chrV	572939	573101	162	**-1,236180843**	N5:573065	8,718	+11:YER190W;	11			TEL RIGHT
chrV	573040	573195	155	**-1,923495307**	N5:573065	8,718	+11:YER190W;	11			TEL RIGHT
chrV	573174	573311	137	**-1,171630664**	N5:573237	10,817	+12:YER190W;	12			TEL RIGHT
chrV	573461	573612	151	**-1,040248665**	N5:573516	22,368	+14:YER190W;	14			TEL RIGHT
chrV	573469	573612	143	**-1,502217954**	N5:573516	22,368	+14:YER190W;	14			TEL RIGHT
chrV	573517	573642	125	**-0,821271071**	N5:573516	22,368	+14:YER190W;	14			TEL RIGHT
chrV	573742	573894	152	**-1,236127845**	N5:573853	12,014	+16:YER190W;	16			TEL RIGHT
chrV	573815	573959	144	**-1,00007447**	N5:573853	12,014	+16:YER190W;	16			TEL RIGHT
chrV	573912	574047	135	**-0,99409991**	N5:574037	55,842	+17:YER190W;	17			TEL RIGHT
chrV	573912	574099	187	**-0,784612356**	N5:574037	55,842	+17:YER190W;	17			TEL RIGHT
chrV	573955	574047	92	**-0,484806051**	N5:574037	55,842	+17:YER190W;	17			TEL RIGHT
chrV	573955	574099	144	**-1,034482195**	N5:574037	55,842	+17:YER190W;	17			TEL RIGHT
chrV	574294	574446	152	**-1,398100759**	N5:574369	12,53	+19:YER190W;	19			TEL RIGHT
chrV	574595	574725	130	**-1,002021939**	N5:574672	41,677	+21:YER190W;	21			TEL RIGHT
chrV	574965	575116	151	**-1,044563714**	N5:575085	20,306	+24:YER190W;	24			TEL RIGHT
chrV	574970	575104	134	**-0,973015657**	N5:575085	20,306	+24:YER190W;	24			TEL RIGHT
chrV	574983	575079	96	**-0,768229719**	N5:575085	20,306	+24:YER190W;	24			TEL RIGHT
chrV	575037	575152	115	**-0,565347997**	N5:575085	20,306	+24:YER190W;	24			TEL RIGHT
chrV	576130	576281	151	**-1,187276244**	N5:576205	0	+31:YER190W; +1:YER190C-B;	31			TEL RIGHT
chrVI	257	431	174	**-1,200114346**	N6:337	43,267	+3:YFL068W;	3			TEL LEFT
chrVI	377	525	148	**-1,48073657**				Overlap <50 bp			TEL LEFT
chrVI	2507	2632	125	**-0,758061132**	N6:2616	10,066	+1:YFL066C;	1			TEL LEFT
chrVI	2537	2680	143	**-1,438728731**	N6:2616	10,066	+1:YFL066C;	1			TEL LEFT
chrVI	2537	2688	151	**-1,386433233**	N6:2616	10,066	+1:YFL066C;	1			TEL LEFT
chrVI	2828	2965	137	**-1,010188926**	N6:2902	5,196	-1:YFL066C; +4*:YFL065C;	-1			TEL LEFT
chrVI	3919	4067	148	**-1,098782974**	N6:4059	27,074	-1:YFL064C;	-1			TEL LEFT
chrVI	4714	4814	100	**0,062255146**	N6:4769	17,678		Intergene			TEL LEFT
chrVI	6022	6182	160	**-1,209614041**	N6:6092	34,131	-1:YFL062W;	-1			
chrVI	13717	13867	150	**-1,250256886**	N6:13829	5,657	+7:YFL058W;	7			
chrVI	15894	16044	150	**-1,12433456**	N6:15963	11,994		Intergene			
chrVI	24066	24172	106	**-1,332111344**	N6:24173	13,416	+6:YFL053W;	6			
chrVI	24067	24172	105	**-1,73228138**	N6:24173	13,416	+6:YFL053W;	6			
chrVI	29981	30130	149	**-1,512408731**	N6:30055	18,228	+4:YFL051C;	4			
chrVI	36134	36286	152	**-1,414020516**	N6:36241	4,764		Intergene			
chrVI	36777	36932	155	**-0,969227534**	N6:36827	6,314	+1:YFL049W;	1			
chrVI	41762	41909	147	**-0,951451902**	N6:41884	23,166	+10:YFL047W;	10			
chrVI	48029	48199	170	**-1,331649524**	N6:48100	14,542		Intergene			
chrVI	49950	50105	155	**-0,716641098**	N6:50008	9,579	+6:YFL041W;	6			
chrVI	55917	56077	160	**-1,413934322**	N6:55979	7,174	+1:YFL038C; -1:YFL037W;	1			
chrVI	60687	60850	163	**-1,414413713**	N6:60777	39,259	+14:YFL036W;	14			
chrVI	65236	65388	152	**-1,092265165**	N6:65325	8,232	+1:YFL034W;	1			
chrVI	65445	65606	161	**-1,138623168**	N6:65510	26,489	+2:YFL034W;	2			
chrVI	68129	68276	147	**-1,347365696**	N6:68162	46,167	+19:YFL034W;	19			
chrVI	68203	68367	164	**-1,258627703**				Overlap <50 bp			
chrVI	71963	72118	155	**-1,240194561**	N6:72001	12,021		Intergene			
chrVI	72978	73128	150	**-1,291792381**				Overlap <50 bp			
chrVI	76566	76713	147	**-0,988080596**	N6:76715	22,509	0:YFL030W; +7*:anti055;	0			
chrVI	77258	77379	121	**-1,498582826**				Overlap <50 bp			
chrVI	79934	80100	166	**-1,96983285**	N6:80017	11,635	+2:YFL028C;	2			
chrVI	81318	81445	127	**-1,234917645**	N6:81386	12,042	+4:YFL027C;	4			
chrVI	82718	82885	167	**-1,330963051**				Overlap <50 bp			
chrVI	84294	84451	157	**-1,448113915**	N6:84401	43,535	+18:YFL025C;	18			
chrVI	91095	91248	153	**-1,383248162**	N6:91173	6,356	+2:YFL023W;	2			
chrVI	94045	94152	107	**-1,173735745**	N6:94050	19,305	+7*:YFL022C;	7	TERM		
chrVI	94173	94319	146	**-1,263006617**	N6:94222	13,892	+6:YFL022C;	6			
chrVI	94286	94435	149	**-1,240856035**	N6:94398	14,348	+5:YFL022C;	5			
chrVI	97720	97865	145	**-1,194853985**	N6:97797	30,798	+2:Unit204;	2			
chrVI	97725	97874	149	**-2,07190461**	N6:97797	30,798	+2:Unit204;	2			
chrVI	100985	101138	153	**-1,341002939**	N6:101049	8,361		Intergene			
chrVI	108232	108382	150	**-1,171328526**	N6:108293	20,809	+11:YFL013C;	11			
chrVI	108406	108531	125	**-1,256091324**	N6:108481	12,832	+10:YFL013C;	10			
chrVI	110912	111064	152	**-1,449062316**	N6:110937	25,521	+3:YFL012W;	3			
chrVI	110912	111064	152	**-1,449062316**	N6:111087	19,217	+4:YFL012W;	4			
chrVI	111983	112114	131	**-1,175445116**	N6:112065	15,026	YFL011W; +1:Unit217; -1:Unit205;	-1			
chrVI	115465	115634	169	**-1,136239443**	N6:115552	11,76	+2:YFL010C;	2			
chrVI	123008	123146	138	**-1,03810545**	N6:123085	13,52	-1:YFL007W;	-1			
chrVI	130462	130613	151	**-1,23955614**	N6:130530	3,098	+3:YFL005W;	3			
chrVI	131462	131615	153	**-1,278392274**	N6:131556	23,69	-1:Unit206; 0:YFL004W;	-1			
chrVI	139986	140115	129	**-1,304365929**	N6:139988	18,248		Intergene			
chrVI	140063	140227	164	**-1,310806718**	N6:140160	24,269		Intergene			
chrVI	141451	141594	143	**-0,985920775**	N6:141487	30,006		Intergene			
chrVI	142726	142892	166	**-1,301314702**	N6:142759	24,434		Intergene			
chrVI	143530	143679	149	**-1,254139061**	N6:143622	24,194		Intergene			
chrVI	148326	148498	172	**-1,326220649**	N6:148376	9,288	+8*:YFL001W;	8	TERM		
chrVI	151570	151741	171	**-1,507539224**	N6:151682	32,887	+11:YFR002W;	11			
chrVI	151602	151755	153	**-1,28941835**	N6:151682	32,887	+11:YFR002W;	11			
chrVI	151830	151995	165	**-1,473101052**	N6:151925	16,258	+12:YFR002W;	12			
chrVI	152009	152167	158	**-1,429043515**	N6:152083	17,855	+13:YFR002W;	13			
chrVI	152552	152696	144	**-0,953524862**	N6:152640	4,708	+4*:YFR003C;	4	TERM		
chrVI	155423	155586	163	**-1,497475844**	N6:155517	10,71	+3:YFR005C;	3			
chrVI	162535	162694	159	**-1,290762297**	N6:162635	6,309	+2:YFR009W;	2			
chrVI	174443	174598	155	**-1,434724964**	N6:174527	14,046	+12:YFR015C;	12			

CHR	START	END	L(bp)	ΔLknuc	Nuc ID	Fuzziness	Gene ID	Gene body postion or intergene	Terminal	rDNA	Telomeric
chrVI	175285	175426	141	**-1,098600541**	N6:175340	22,993	+7:YFR015C;	7			
chrVI	176188	176314	126	**-0,460691877**	N6:176274	18,228	+2:YFR015C;	2			
chrVI	177968	178139	171	**-1,502795237**	N6:178030	38,026		Intergene			
chrVI	180563	180715	152	**-1,292676894**	N6:180613	25,528	+2:YFR016C;	2			
chrVI	182163	182304	141	**-1,016809972**	N6:182222	6,058	+5*:YFR017C;	5	TERM		
chrVI	183274	183405	131	**-2,130995957**				Overlap <50 bp			
chrVI	183755	183882	127	**-0,753083521**	N6:183852	9,96	+3:YFR018C;	3			
chrVI	185980	186131	151	**-1,30572199**	N6:186127	16,489	+10:YFR019W;	10			
chrVI	186486	186602	116	**-1,500241078**	N6:186517	20,777	+12:YFR019W;	12			
chrVI	192368	192528	160	**-1,479828325**	N6:192442	6,535		Intergene			
chrVI	193031	193186	155	**-1,422354477**	N6:193102	1,915		Intergene			
chrVI	195001	195152	151	**-1,306456249**	N6:195090	9,394	+3:YFR021W;	3			
chrVI	197175	197337	162	**-1,448677025**	N6:197229	24,205	+4:YFR022W;	4			
chrVI	197468	197595	127	**-1,262299419**				Overlap <50 bp			
chrVI	198399	198562	163	**-1,142073798**	N6:198429	7,789	+11:YFR022W;	11			
chrVI	202836	202999	163	**-1,356147388**	N6:202902	9,98		Intergene			
chrVI	204046	204202	156	**-1,297534777**	N6:204111	1,643	+5:YFR025C;	5			
chrVI	208087	208238	151	**-1,036805945**	N6:208144	24,403	+5:YFR027W;	5			
chrVI	208444	208571	127	**-1,268102919**	N6:208478	17,871	+11*:YFR028C; +7:YFR027W;	11	TERM		
chrVI	208746	208898	152	**-1,272290667**	N6:208819	26,853	+9:YFR028C; +9:YFR027W;	9			
chrVI	210302	210398	96	**-1,565444235**	N6:210340	4,98	-1:YFR028C;	-1			
chrVI	210302	210400	98	**-1,414462921**	N6:210340	4,98	-1:YFR028C;	-1			
chrVI	210302	210402	100	**-1,419983737**	N6:210340	4,98	-1:YFR028C;	-1			
chrVI	210302	210403	101	**-1,436289577**	N6:210340	4,98	-1:YFR028C;	-1			
chrVI	210302	210404	102	**-1,343411677**	N6:210340	4,98	-1:YFR028C;	-1			
chrVI	210302	210405	103	**-1,617392982**	N6:210340	4,98	-1:YFR028C;	-1			
chrVI	210302	210406	104	**-1,46114542**	N6:210340	4,98	-1:YFR028C;	-1			
chrVI	210302	210407	105	**-1,4880736**	N6:210340	4,98	-1:YFR028C;	-1			
chrVI	210302	210409	107	**-1,367556663**	N6:210340	4,98	-1:YFR028C;	-1			
chrVI	214215	214366	151	**-1,306344638**	N6:214204	32,337	+7:YFR030W;	7			
chrVI	214215	214366	151	**-1,306344638**	N6:214374	16,008	+8:YFR030W;	8			
chrVI	224528	224665	137	**-1,055195735**	N6:224600	41,62	+2:YFR033C;	2			
chrVI	225246	225383	137	**-0,758999867**				Overlap <50 bp			
chrVI	225640	225800	160	**-1,44169213**	N6:225764	5,441	+2:YFR034C;	2			
chrVI	228483	228624	141	**-0,995094292**	N6:228495	14,751	+5:YFR037C;	5			
chrVI	232870	233015	145	**-1,061602468**	N6:232930	12,637	+5:YFR039C;	5			
chrVI	236135	236288	153	**-1,569244981**	N6:236220	42,965	+15:YFR040W;	15			
chrVI	236706	236866	160	**-1,351420267**	N6:236786	35,865	+18:YFR040W;	18			
chrVI	237607	237756	149	**-1,184381531**	N6:237708	6,504	+4:YFR041C;	4			
chrVI	237976	238116	140	**-1,090784436**	N6:238042	8,379	+2:YFR041C;	2			
chrVI	239069	239219	150	**-1,342599476**	N6:239120	13,831	+5*:YFR042W;	5	TERM		
chrVI	242117	242275	158	**-1,131817428**	N6:242181	10,159	+2:YFR045W;	2			
chrVI	242831	242985	154	**-1,37019621**	N6:242929	45,045	+6*:YFR045W; +9*:YFR046C;	6	TERM		
chrVI	242895	243045	150	**-1,523153594**	N6:242929	45,045	+6*:YFR045W; +9*:YFR046C;	6	TERM		
chrVI	244023	244165	142	**-1,160516421**	N6:244096	10,309	+2:YFR046C;	2			
chrVI	244610	244760	150	**-1,228527098**	N6:244637	0	+4:YFR047C;	4			
chrVI	247013	247149	136	**-0,705578496**	N6:247082	23,398	+7:YFR048W;	7			
chrVI	258453	258604	151	**-1,671543592**	N6:258521	27,206		Intergene			
chrVI	260977	261105	128	**-1,011655971**	N6:261029	34,994		Intergene			
chrVI	264647	264774	127	**-1,073368727**				Overlap <50 bp			
chrVI	265600	265745	145	**-1,034138493**	N6:265749	18,434		Intergene			
chrVI	267454	267607	153	**-1,534590669**	N6:267517	27,951		Intergene			
chrVI	269103	269252	149	**-1,329072761**	N6:269131	22,098	+2:YFR057W;	2			
chrVI	270018	270158	140	**-1,200338748**	N6:270163	33,941		Intergene			TEL RIGHT
chrVII	6501	6604	103	**-1,344732916**	N7:6569	11,504	-1:YGL260W; +2:YGL261C;	-1			
chrVII	7179	7332	153	**-1,542523593**	N7:7319	7,805	+4*:YGL260W;	4	TERM		
chrVII	7179	7332	153	**-1,542523593**	N7:7156	4,272	+3:YGL260W;	3			
chrVII	8743	8898	155	**-1,287471763**	N7:8851	28,687	+4:YGL259W; -1:YGL258W-A;	4			
chrVII	8747	8898	151	**-1,325453083**	N7:8851	28,687	+4:YGL259W; -1:YGL258W-A;	4			
chrVII	9683	9793	110	**-1,196384333**	N7:9667	21,339	+9:YGL259W;	9			
chrVII	15630	15761	131	**-0,942814163**	N7:15706	47,019	+7:YGL256W;	7			
chrVII	15633	15761	128	**-1,07464229**	N7:15706	47,019	+7:YGL256W;	7			
chrVII	15791	15935	144	**-1,499304451**				Overlap <50 bp			
chrVII	20349	20487	138	**-1,195206169**	N7:20424	5,899		Intergene			
chrVII	21325	21484	159	**-1,395544131**	N7:21457	24,473	+5:YGL255W;	5			
chrVII	22410	22546	136	**-1,05037092**	N7:22504	5,879	+5:CUT583; +2:YGL254W;	5			
chrVII	23209	23365	156	**-1,426702114**	N7:23217	14,748	+1:CUT583; +6:YGL254W;	1			
chrVII	32814	32961	147	**-1,456570695**	N7:32833	24,46	GL250W; +1:SUT533; -1:YGL249W;	6	TERM		
chrVII	33222	33355	133	**-1,306549984**	N7:33236	10,496	+11:SUT534; +2:YGL249W;	11			
chrVII	36941	37083	142	**-1,259313104**	N7:36962	8,687	+1:YGL247W;	1			
chrVII	41750	41885	135	**-1,574749961**	N7:41812	5,785	+3:YGL244W;	3			
chrVII	52996	53149	153	**-1,228202349**	N7:53078	32,752	+4:YGL237C;	4			
chrVII	53375	53486	111	**-1,364857664**	N7:53412	3,502	+2:YGL237C;	2			
chrVII	62645	62823	178	**-1,761732769**	N7:62782	30,625		Intergene			
chrVII	69139	69303	164	**-1,330589542**	N7:69217	9,757	+11:YGL228W; -1:YGL227W;	11			
chrVII	72210	72368	158	**-1,404539502**	N7:72277	8,042		Intergene			
chrVII	73049	73197	148	**-1,393409235**	N7:73145	16,622	+1*:YGL226C-A; -1:YGL226W;	1	TERM		
chrVII	78222	78356	134	**-1,04970544**	N7:78264	9,585	+14:YGL223C; +5:YGL224C;	14			
chrVII	83125	83232	107	**-1,093800065**	N7:83164	17,63	+8:YGL219C;	8			
chrVII	83125	83247	122	**-1,314671883**	N7:83164	17,63	+8:YGL219C;	8			
chrVII	84927	85059	132	**-1,070576812**	N7:84990	14,085	+3:YGL216W;	3			
chrVII	88176	88368	192	**-1,325156433**	N7:88188	9,988	+5:YGL215W;	5			
chrVII	88303	88443	140	**-1,092830011**				Overlap <50 bp			
chrVII	88402	88555	153	**-1,119596801**	N7:88473	12,759	+7:YGL215W;	7			
chrVII	89045	89177	132	**-1,121197653**				Overlap <50 bp			
chrVII	90038	90161	123	**-1,175779921**	N7:90075	15,222	+8*:YGL213C;	8	TERM		
chrVII	91402	91556	154	**-1,156406361**	N7:91484	22,807	+1:YGL212W; -1:YGL213C;	1			
chrVII	92970	93138	168	**-1,266217493**	N7:93036	24,978	+4:YGL211W;	4			
chrVII	97437	97599	162	**-1,427513424**	N7:97514	14,549	+2:YGL208W;	2			
chrVII	102150	102299	149	**-1,405788269**	N7:102228	18,52		Intergene			
chrVII	102520	102673	153	**-1,603581125**	N7:102600	27,313	+32:YGL206C;	32			
chrVII	103288	103440	152	**-1,136028401**	N7:103447	49,516	+27:YGL206C;	27			
chrVII	103852	103984	132	**-1,149755245**	N7:103952	22,777	+24:YGL206C;	24			
chrVII	108983	109147	164	**-1,410889484**	N7:109010	40,278	+8:SUT537; +6:YGL205W;	8			
chrVII	116027	116173	146	**-1,120644916**	N7:116126	14,519	+2:YGL202W;	2			
chrVII	119268	119414	146	**-1,16952184**	N7:119279	40,009	+11:YGL201C;	11			

CHR	START	END	L(bp)	ΔLknuc	Nuc ID	Fuzziness	Gene ID	Gene body postion or intergene	Terminal	rDNA	Telomeric
chrVII	125888	126050	162	**-1,384429889**	N7:125970	26,986	+9:YGL197W;	9			
chrVII	138978	139125	147	**-1,328937718**	N7:139099	25,612		Intergene			
chrVII	147769	147920	151	**-1,395131641**	N7:147908	6,535	0:Unit261;	0			
chrVII	152057	152208	151	**-1,591722759**	N7:152137	19,449	+5:YGL186C;	5			
chrVII	156139	156292	153	**-1,624930912**	N7:156241	20,868	-1:YGL184C;	-1			
chrVII	167824	167963	139	**-1,01828221**	N7:167816	7,935		Intergene			
chrVII	167824	167963	139	**-1,01828221**	N7:167983	28,836		Intergene			
chrVII	168448	168554	106	**-1,596342667**	N7:168455	9,368		Intergene			
chrVII	168635	168789	154	**-1,686079628**	N7:168761	37,278		Intergene			
chrVII	171388	171548	160	**-1,329231893**	N7:171452	17,19	+10*:YGL176C;	10	TERM		
chrVII	174752	174849	97	**-1,325810284**	N7:174743	2,422	+2:YGL174W;	2			
chrVII	174828	174980	152	**-1,176151799**	N7:174904	5,989	+3:YGL174W;	3			
chrVII	178188	178320	132	**-1,004723465**	N7:178266	27,142	+15:YGL173C;	15			
chrVII	183143	183279	136	**-1,372240676**				Overlap <50 bp			
chrVII	184850	184994	144	**-1,051215364**	N7:184898	19,765	+4:YGL170C;	4			
chrVII	196178	196334	156	**-1,302984775**	N7:196334	11,032	+2:YGL163C;	2			
chrVII	196178	196334	156	**-1,302984775**	N7:196162	11,619	+3:YGL163C;	3			
chrVII	204030	204183	153	**-1,233350122**	N7:204104	5,468	+9*:YGL159W;	9	TERM		
chrVII	204995	205129	134	**-1,020663457**	N7:205048	8,021		Intergene			
chrVII	209064	209201	137	**-1,299980566**	N7:209110	12,687	+2:YGL157W;	2			
chrVII	214611	214774	163	**-1,40460981**	N7:214691	10,944	+5:YGL155W;	5			
chrVII	217469	217614	145	**-1,144533382**	N7:217533	8,542	+1:YGL151W;	1			
chrVII	221203	221378	175	**-1,098196641**	N7:221318	17,398	+28:YGL150C;	28			
chrVII	223475	223638	163	**-1,344556629**	N7:223563	16,468	+15:YGL150C;	15			
chrVII	226620	226777	157	**-0,70175811**	N7:226722	16,053	+3:YGL148W;	3			
chrVII	226621	226770	149	**-1,260000477**	N7:226722	16,053	+3:YGL148W;	3			
chrVII	233772	233924	152	**-1,341728485**	N7:233838	7,47	+6:YGL144C;	6			
chrVII	238049	238197	148	**-1,272556421**	N7:238126	20,169	-1:YGL141W; +1:YGL142C;	-1			
chrVII	243163	243328	165	**-1,205011668**	N7:243257	26,011	+13:YGL140C;	13			
chrVII	249506	249649	143	**-1,785763635**	N7:249567	4,933	+1:YGL138C;	1			
chrVII	251482	251633	151	**-1,02841495**				Overlap <50 bp			
chrVII	266552	266693	141	**-1,137114217**	N7:266631	15,972	+4:YGL130W;	4			
chrVII	267377	267528	151	**-1,171384347**	N7:267455	27,294	+9:YGL130W;	9			
chrVII	268853	268959	106	**-0,842375882**	N7:268920	11,524	+2:YGL129C;	2			
chrVII	268853	268977	124	**-1,237224761**	N7:268920	11,524	+2:YGL129C;	2			
chrVII	270004	270159	155	**-1,36956371**	N7:270127	17,201	+1:YGL128C;	1			
chrVII	273536	273656	120	**-1,121368652**	N7:273600	25,616		Intergene			
chrVII	275567	275723	156	**-1,285337291**	N7:275555	16,263		Intergene			
chrVII	275567	275723	156	**-1,285337291**	N7:275720	26,82		Intergene			
chrVII	279938	280099	161	**-1,125338512**	N7:280010	22,5	+4:YGL122C;	4			
chrVII	281760	281908	148	**-1,253125879**	N7:281898	21,64	+14:YGL120C;	14			
chrVII	281764	281908	144	**-1,388605836**	N7:281898	21,64	+14:YGL120C;	14			
chrVII	284331	284485	154	**-1,062502872**	N7:284426	7,899	+1:YGL119W;	1			
chrVII	289603	289760	157	**-1,462679938**	N7:289668	37,131	+1:YGL116W;	1			
chrVII	293723	293871	148	**-1,041166926**	N7:293806	25,272	+4:YGL114W;	4			
chrVII	294019	294183	164	**-1,780793194**	N7:294135	15,498	+6:YGL114W;	6			
chrVII	297177	297313	136	**-1,15074275**	N7:297182	35,303	+9:YGL113W;	9			
chrVII	299926	300073	147	**-1,170676717**	N7:300004	6,735	-1:YGL112C; +1:YGL111W;	-1			
chrVII	300091	300244	153	**-1,190705321**	N7:300166	6,465	+2:YGL111W;	2			
chrVII	300595	300755	160	**-1,451002598**				Overlap <50 bp			
chrVII	313487	313646	159	**-1,487474576**	N7:313555	8,204	+3:YGL100W;	3			
chrVII	314575	314719	144	**-1,412057745**	N7:314647	5,01	+1:YGL099W; -1:CUT595;	1			
chrVII	320068	320221	153	**-1,159280053**	N7:320063	7,859	+1:SUT114;	1			
chrVII	320068	320221	153	**-1,159280053**	N7:320242	21,417	+2:SUT114;	2			
chrVII	323178	323326	148	**-1,490382885**				Overlap <50 bp			
chrVII	324915	325051	136	**-1,411869503**	N7:325012	11,082	-1:YGL096W;	-1			
chrVII	327547	327717	170	**-1,247979841**	N7:327548	34,723		Intergene			
chrVII	327547	327717	170	**-1,247979841**	N7:327719	24,372		Intergene			
chrVII	328882	329034	152	**-1,447105949**	N7:329029	16,562	+11:YGL095C;	11			
chrVII	329271	329388	117	**-1,778571749**	N7:329338	20,498	+9:YGL095C;	9			
chrVII	330372	330517	145	**-1,167160957**	N7:330463	11,79	+2:YGL095C;	2			
chrVII	331632	331780	148	**-1,275417619**	N7:331795	31,694	+18:YGL094C;	18			
chrVII	331632	331780	148	**-1,275417619**	N7:331642	32,828	+19:YGL094C;	19			
chrVII	341547	341699	152	**-1,528194936**	N7:341572	4,546	+24:YGL092W;	24			
chrVII	342190	342339	149	**-1,265549255**				Overlap <50 bp			
chrVII	342310	342453	143	**-1,110882225**	N7:342424	27,427	+5:YGL091C;	5			
chrVII	343265	343403	138	**-1,169386757**	N7:343315	4,844	+1:YGL090W; -1:YGL091C;	1			
chrVII	352278	352440	162	**-1,466163356**	N7:352305	6,439	+1:YGL084C;	1			
chrVII	354451	354624	173	**-1,461552527**	N7:354512	51,432	+10:YGL083W;	10			
chrVII	356531	356676	145	**-1,262464981**	N7:356589	11,61	+6:YGL082W;	6			
chrVII	364639	364801	162	**-1,404407366**	N7:364708	1,414		Intergene			
chrVII	367967	368120	153	**-1,011372664**	N7:368054	8,802	+2:YGL075C;	2			
chrVII	370607	370760	153	**-1,491822377**	N7:370672	4,97	+12:YGL073W;	12			
chrVII	382677	382828	151	**-1,428412347**	N7:382840	6,042	+3:YGL064C;	3			
chrVII	382677	382828	151	**-1,428412347**	N7:382673	17,697	+4:YGL064C;	4			
chrVII	383386	383517	131	**-0,874142509**	N7:383447	21,685	+1:YGL063W; -1:YGL064C;	1			
chrVII	383588	383739	151	**-0,78952824**	N7:383638	24,47	+2:YGL063W;	2			
chrVII	384835	384978	143	**-1,827958092**	N7:384900	12,22	+9:YGL063W; -1:YGL062W;	9			
chrVII	384841	384978	137	**-1,501809551**	N7:384900	12,22	+9:YGL063W; -1:YGL062W;	9			
chrVII	386231	386378	147	**-1,082886696**	N7:386322	34,798	+8:YGL062W;	8			
chrVII	386588	386752	164	**-1,534194436**	N7:386632	45,997	+10:YGL062W;	10			
chrVII	398118	398266	148	**-1,16567702**				Overlap <50 bp			
chrVII	398740	398893	153	**-1,282158266**	N7:398821	14,799	+2:YGL055W;	2			
chrVII	399113	399273	160	**-1,29757531**	N7:399182	11,256	+4:YGL055W;	4			
chrVII	402184	402330	146	**-1,402219053**	N7:402298	26,92	-1:YGL053W;	-1			
chrVII	406230	406375	145	**-1,061600504**	N7:406270	19,026	+4:YGL050W;	4			
chrVII	412759	412909	150	**-1,373005527**	N7:412835	6,745	+3:CUT600;	3			
chrVII	413027	413197	170	**-1,880692813**	N7:413154	24,499	+1:CUT600;	1			
chrVII	416964	417114	150	**-1,385567264**	N7:417040	5,086	+2:YGL044C;	2			
chrVII	419105	419256	151	**-1,471173429**	N7:419189	16,861	-1:YGL041C-B; +3:YGL041W-A;	-1			
chrVII	422885	423015	130	**-1,162825096**	N7:422958	7,071	+5*:SUT118;	5	TERM		
chrVII	424432	424587	155	**-1,459672603**	N7:424573	47,995	+5:YGL039W;	5			
chrVII	426688	426828	140	**-1,241124711**	N7:426744	16,661	+2:YGL038C;	2			
chrVII	430629	430785	156	**-1,370031438**	N7:430704	34,387	+14:YGL036W;	14			
chrVII	438337	438504	167	**-1,225368127**	N7:438393	27,83		Intergene			
chrVII	445398	445550	152	**-1,33024666**	N7:445477	5,762	+5:YGL027C;	5			

CHR	START	END	L(bp)	ΔLknuc	Nuc ID	Fuzziness	Gene ID	Gene body postion or intergene	Terminal	rDNA	Telomeric
chrVII	445405	445562	157	**-1,066353576**	N7:445477	5,762	+5:YGL027C;	5			
chrVII	448295	448446	151	**-1,14277151**	N7:448359	5,612	+2:YGL026C;	2			
chrVII	449063	449214	151	**-1,372267789**	N7:449131	13,626	+6:YGL025C;	6			
chrVII	449866	450017	151	**-1,349950154**	N7:449940	9,933	+1:YGL025C;	1			
chrVII	450289	450475	186	**-1,118411944**	N7:450357	12,05	+12*:YGL023C;	12	TERM		
chrVII	450564	450727	163	**-1,370390337**				Overlap <50 bp			
chrVII	451657	451811	154	**-1,196634111**	N7:451765	9,301	+3:YGL023C;	3			
chrVII	452015	452160	145	**-1,458813534**	N7:452093	5,788	+1:YGL023C; -1:YGL022W;	1			
chrVII	454120	454220	100	**-1,563903427**	N7:454191	22,142	+12:YGL022W;	12			
chrVII	457180	457330	150	**-1,539786381**	N7:457208	7,868	+5*:YGL020C;	5	TERM		
chrVII	457820	457954	134	**-1,197197554**	N7:457886	3,983	-1:YGL019W; +1:YGL020C;	-1			
chrVII	458030	458181	151	**-1,188453173**	N7:458116	14,538	+1:YGL019W; -1:YGL020C;	1			
chrVII	464168	464326	158	**-1,563938962**	N7:464210	35,227	+16:YGL016W;	16			
chrVII	468550	468701	151	**-1,297502165**	N7:468622	12,5	+19:YGL014W;	19			
chrVII	470833	470993	160	**-1,289167805**				Overlap <50 bp			
chrVII	471018	471094	76	**-0,784**	N7:471038	27,175	+9:YGL013C;	9			
chrVII	471018	471123	105	**-0,340437773**	N7:471038	27,175	+9:YGL013C;	9			
chrVII	471173	471322	149	**-1,139935366**	N7:471209	46,098	+8:YGL013C;	8			
chrVII	475774	475931	157	**-1,373988807**	N7:475867	10,889	+3:YGL010W;	3			
chrVII	476346	476454	108	**-1,305185286**				Overlap <50 bp			
chrVII	476346	476465	119	**-1,620063938**				Overlap <50 bp			
chrVII	478282	478437	155	**-0,897100408**	N7:478357	17,783	+3:YGL009C;	3			
chrVII	482579	482701	122	**-1,241825791**	N7:482600	20,659	+3:YGL008C;	3			
chrVII	483653	483754	101	**-0,823890476**	N7:483643	36,062	+2:SUT548;	2			
chrVII	495041	495196	155	**-1,058623414**	N7:495024	24,326	+4:YGL002W;	4			
chrVII	501090	501258	168	**-1,499675317**	N7:501148	9,873	+7:YGR003W;	7			
chrVII	502391	502522	131	**-1,209325931**	N7:502483	13,866	+15*:YGR003W;	15	TERM		
chrVII	502870	503027	157	**-1,406405847**	N7:502941	6,976	+1:YGR004W;	1			
chrVII	504629	504778	149	**-1,361011428**	N7:504726	14,919	+8:YGR005C;	8			
chrVII	505104	505255	151	**-1,424476987**	N7:505190	8,62	+5:YGR005C;	5			
chrVII	509103	509251	148	**-1,244391604**	N7:509169	19,167		Intergene			
chrVII	509261	509410	149	**-1,523709292**	N7:509325	12,936	+12*:YGR009C;	12	TERM		
chrVII	510438	510590	152	**-1,426014425**	N7:510527	7,765	+4:YGR009C;	4			
chrVII	512325	512500	175	**-1,283216756**	N7:512399	15,62	+6:YGR010W;	6			
chrVII	515194	515319	125	**-1,02938617**	N7:515214	14,283	+5:YGR013W;	5			
chrVII	516112	516262	150	**-1,459946635**	N7:516278	20,731	0:CUT604; +12:YGR013W;	0			
chrVII	516112	516262	150	**-1,459946635**	N7:516125	8,77	+2:CUT604; +11:YGR013W;	2			
chrVII	517734	517882	148	**-1,265985578**	N7:517806	10,64	+8:YGR014W;	8			
chrVII	517736	517882	146	**-1,773840711**	N7:517806	10,64	+8:YGR014W;	8			
chrVII	518337	518484	147	**-1,34590264**	N7:518402	6,058	+12:YGR014W;	12			
chrVII	519407	519563	156	**-1,275596467**	N7:519436	17,328	+18:YGR014W;	18			
chrVII	519674	519834	160	**-1,284890704**				Overlap <50 bp			
chrVII	519935	520102	167	**-1,32908629**	N7:520057	32,474	+21:YGR014W;	21			
chrVII	520743	520902	159	**-1,252213009**	N7:520845	32,141	+25*:YGR014W;	25	TERM		
chrVII	520956	521107	151	**-1,722404401**	N7:521078	28,902	+7*:YGR015C;	7	TERM		
chrVII	526220	526384	164	**-1,708989585**	N7:526309	8,06		Intergene			
chrVII	529947	530056	109	**-0,349196698**	N7:529978	13,7	+6:YGR023W;	6			
chrVII	535422	535536	114	**-1,389281652**	N7:535504	49,636	-1:CUT606;	-1			
chrVII	536123	536272	149	**-1,239770123**	N7:536209	2,828		Intergene			
chrVII	536273	536379	106	**-0,892230054**				Overlap <50 bp			
chrVII	536273	536391	118	**-1,063166576**				Overlap <50 bp			
chrVII	536297	536420	123	**-0,988501489**				Overlap <50 bp			
chrVII	536397	536552	155	**-1,226783257**	N7:536510	31,048		Intergene			
chrVII	536830	536964	134	**-1,175275641**	N7:536902	26,577		Intergene			
chrVII	536830	536970	140	**-1,179556935**	N7:536902	26,577		Intergene			
chrVII	537026	537177	151	**-1,153255118**	N7:537109	26,458		Intergene			
chrVII	537036	537181	145	**-1,115608884**	N7:537109	26,458		Intergene			
chrVII	537308	537462	154	**-1,230613727**	N7:537445	10		Intergene			
chrVII	537342	537486	144	**-1,114514704**	N7:537445	10		Intergene			
chrVII	537873	538008	135	**-1,200992722**	N7:537917	2,887		Intergene			
chrVII	538055	538187	132	**-1,239847503**				Overlap <50 bp			
chrVII	538144	538282	138	**-1,27156563**	N7:538222	17,678		Intergene			
chrVII	538499	538656	157	**-1,465812911**	N7:538585	30		Intergene			
chrVII	538599	538743	144	**-1,39422548**	N7:538585	30		Intergene			
chrVII	538990	539119	129	**-1,233675531**				Overlap <50 bp			
chrVII	539470	539601	131	**-1,454414983**	N7:539492	52,747		Intergene			
chrVII	539902	540052	150	**-1,671066088**	N7:539986	21,502		Intergene			
chrVII	540853	541004	151	**-1,259961489**	N7:540903	16,971		Intergene			
chrVII	542644	542795	151	**-1,316033793**	N7:542763	21,005	+5:YGR028W;	5			
chrVII	544012	544140	128	**-1,015211528**	N7:544085	10,257	+3:SUT550;	3			
chrVII	545414	545580	166	**-0,946329183**	N7:545553	40,847		Intergene			
chrVII	546693	546840	147	**-0,658631856**	N7:546767	5,565	+3:YGR031W;	3			
chrVII	548258	548409	151	**-1,229340682**	N7:548239	22,448	+1:YGR032W;	1			
chrVII	554460	554622	162	**-1,325179451**	N7:554535	15,349	+4:YGR033C;	4			
chrVII	554946	555089	143	**-1,472131007**	N7:555041	5,845	+1:YGR033C;	1			
chrVII	562268	562415	147	**-1,288448153**	N7:562318	11,79		Intergene			
chrVII	562775	562889	114	**-1,425104861**				Overlap <50 bp			
chrVII	563471	563621	150	**-1,316115291**	N7:563527	36,665		Intergene			
chrVII	564404	564533	129	**-0,943711102**				Overlap <50 bp			
chrVII	564867	565024	157	**-1,34156453**	N7:564949	19,975		Intergene			
chrVII	565335	565496	161	**-1,356854588**	N7:565396	7,778		Intergene			
chrVII	565515	565650	135	**-1,101787127**	N7:565587	55,126		Intergene			
chrVII	566553	566693	140	**-1,209913358**	N7:566644	34,598		Intergene			
chrVII	566559	566693	134	**-0,825968925**	N7:566644	34,598		Intergene			
chrVII	566971	567126	155	**-1,307082354**	N7:567004	20,809		Intergene			
chrVII	567102	567210	108	**-0,925011704**	N7:567215	49,003		Intergene			
chrVII	568106	568272	166	**-1,317516187**	N7:568185	17,729		Intergene			
chrVII	568932	569081	149	**-1,276465994**	N7:568958	7,55		Intergene			
chrVII	569719	569885	166	**-1,923964984**	N7:569812	18,148		Intergene			
chrVII	571017	571160	143	**-1,103078113**	N7:571098	15,885		Intergene			
chrVII	572385	572549	164	**-1,281663843**	N7:572444	21,455		Intergene			
chrVII	572497	572626	129	**-1,174394484**	N7:572607	26,577		Intergene			
chrVII	573872	573982	110	**-1,342307143**	N7:573948	17,214		Intergene			
chrVII	575134	575270	136	**-1,298923694**	N7:575205	10,187	+2:YGR040W;	2			
chrVII	575584	575763	179	**-1,441272738**	N7:575570	24,546		Intergene			
chrVII	575584	575763	179	**-1,441272738**	N7:575745	8,905		Intergene			

CHR	START	END	L(bp)	ΔLknuc	Nuc ID	Fuzziness	Gene ID	Gene body postion or intergene	Terminal	rDNA	Telomeric
chrVII	578432	578606	174	-1,570721487	N7:578577	30,004	+5:SUT551; +9:YGR041W;	5			
chrVII	578432	578606	174	-1,570721487	N7:578412	51,237	+6:SUT551; +8:YGR041W;	6			
chrVII	578979	579129	150	-1,222984445	N7:579094	11,234	+2:SUT551; +12*:YGR041W;	2			
chrVII	579144	579294	150	-1,460838059	N7:579259	11,786	+1:SUT551; -1:YGR042W;	1			
chrVII	583828	583997	169	-1,089287822	N7:583861	10,759	+1:YGR044C;	1			
chrVII	584107	584258	151	-1,271826777	N7:584192	28,369	-1:YGR044C;	-1			
chrVII	584200	584336	136	-0,674520287	N7:584192	28,369	-1:YGR044C;	-1			
chrVII	584954	585099	145	-1,497111235	N7:584996	7,859	+2:YGR046W;	2			
chrVII	594298	594445	147	-1,036821297	N7:594404	21,664	+6:YGR052W;	6			
chrVII	595048	595213	165	-1,648510807	N7:595207	9,416	+5:YGR053C;	5			
chrVII	595048	595213	165	-1,648510807	N7:595043	17,394	+6:YGR053C;	6			
chrVII	595281	595432	151	-1,249140419	N7:595369	5,955	+4:YGR053C;	4			
chrVII	610980	611160	180	-1,254965427	N7:610959	31,098	+4:YGR060W;	4			
chrVII	610980	611160	180	-1,254965427	N7:611131	11,845	+5:YGR060W;	5			
chrVII	613266	613414	148	-1,326462944	N7:613372	40,623	+17:YGR061C;	17			
chrVII	637713	637869	156	-1,297848873	N7:637824	8,14	+11:YGR077C; -1:YGR076C;	11			
chrVII	640000	640148	148	-1,327519137	N7:640070	9,883	+3:YGR078C;	3			
chrVII	640002	640148	146	-1,68990033	N7:640070	9,883	+3:YGR078C;	3			
chrVII	640719	640865	146	-1,713622115	N7:640822	24,427	+1:YGR079W;	1			
chrVII	652407	652570	163	-1,538933903	N7:652507	3,371	+4:YGR087C;	4			
chrVII	652780	652925	145	-1,116862565	N7:652859	7,563	+2:YGR087C;	2			
chrVII	654255	654401	146	-1,258719467	N7:654398	16,94	-1:YGR088W;	-1			
chrVII	654255	654401	146	-1,258719467	N7:654248	17,349		Intergene			
chrVII	668831	668991	160	-1,485326114	N7:668917	30,091	+6:YGR092W;	6			
chrVII	671142	671294	152	-1,53570138				Overlap <50 bp			
chrVII	671676	671849	173	-1,063294291	N7:671743	11,76	+9:YGR093W; +2:CUT614;	9			
chrVII	674557	674713	156	-1,354891259	N7:674628	18,992	+16:YGR094W;	16			
chrVII	676654	676817	163	-1,050555283	N7:676774	11,432	+2:YGR096W;	2			
chrVII	680899	681051	152	-1,136803514	N7:680980	14,61	+16:YGR097W;	16			
chrVII	681312	681478	166	-1,599713766	N7:681411	21,461		Intergene			
chrVII	682534	682696	162	-1,434187556	N7:682605	12,75	+32:YGR098C;	32			
chrVII	687608	687758	150	-1,400455956	N7:687651	5,01	-1:YGR099W; +1:YGR098C;	-1			
chrVII	689536	689689	153	-1,219952645	N7:689603	31,394	+11:YGR099W; -1:YGR100W;	11			
chrVII	692091	692197	106	-1,411156342	N7:692094	15,549	+15:YGR100W;	15			
chrVII	692091	692218	127	-0,896234228	N7:692094	15,549	+15:YGR100W;	15			
chrVII	692091	692219	128	-1,075965493	N7:692094	15,549	+15:YGR100W;	15			
chrVII	692091	692220	129	-1,191786782	N7:692094	15,549	+15:YGR100W;	15			
chrVII	692091	692221	130	-1,533516088	N7:692094	15,549	+15:YGR100W;	15			
chrVII	692091	692222	131	-1,358763356	N7:692094	15,549	+15:YGR100W;	15			
chrVII	692091	692223	132	-1,051681441	N7:692094	15,549	+15:YGR100W;	15			
chrVII	692092	692223	131	-1,161381983	N7:692094	15,549	+15:YGR100W;	15			
chrVII	692095	692223	128	-1,01414832	N7:692094	15,549	+15:YGR100W;	15			
chrVII	692097	692197	100	-0,605408406	N7:692094	15,549	+15:YGR100W;	15			
chrVII	693772	693921	149	-0,85173129	N7:693854	9,116	+4:YGR101W;	4			
chrVII	697506	697652	146	-1,277158175	N7:697541	26,334	+6*:YGR104C;	6	TERM		
chrVII	697827	697977	150	-1,228775726	N7:697877	27,396	+4:YGR104C;	4			
chrVII	701841	701992	151	-1,139744544				Overlap <50 bp			
chrVII	707416	707536	120	-1,196073719	N7:707505	13,204		Intergene			
chrVII	709744	709890	146	-1,336973352	N7:709832	3,055		Intergene			
chrVII	712418	712569	151	-1,305648025	N7:712512	26,569		Intergene			
chrVII	714004	714140	136	-1,16641641	N7:714042	30,224	+3:YGR110W;	3			
chrVII	716031	716189	158	-1,678539305				Overlap <50 bp			
chrVII	716536	716689	153	-1,722950029	N7:716629	4,924	+7:YGR111W;	7			
chrVII	726352	726496	144	-1,135990245	N7:726431	19,356	+1:YGR117C;	1			
chrVII	731531	731680	149	-1,297052363	N7:731570	10,178	+2:SUT128; +8:YGR121C;	2			
chrVII	740022	740127	105	-1,464218768	N7:740078	19,476	+2:YGR124W;	2			
chrVII	746153	746303	150	-1,589410555	N7:746206	16,724	R126W; +3:CUT618; -1:YGR127W;	5			
chrVII	747978	748102	124	-1,260440085	N7:748002	23,392	+13:YGR128C;	13			
chrVII	748361	748501	140	-0,946734144	N7:748439	49,872	+11:YGR128C;	11			
chrVII	751512	751624	112	-1,270813929	N7:751606	17,302	+14:YGR130C; +2:SUT130;	14			
chrVII	758738	758894	156	-1,041544541	N7:758814	16,395	+8:YGR134W; +13:YGR133W;	8			
chrVII	760806	760962	156	-1,257734376	N7:760938	6,351	134W; +2:CUT619; +25:YGR133W;	20			
chrVII	760828	760978	150	-1,212257977	N7:760938	6,351	134W; +2:CUT619; +25:YGR133W;	20			
chrVII	762415	762565	150	-1,222057706	N7:762428	16,741	-1:CUT620; +1:YGR136W;	-1			
chrVII	766213	766368	155	-1,058979339	N7:766304	36,17	+4:Unit239;	4			
chrVII	769383	769497	114	-1,049630355	N7:769444	31,04	+13:YGR140W;	13			
chrVII	773391	773557	166	-1,867772188	N7:773500	35,612	+7:YGR142W;	7			
chrVII	776840	777001	161	-1,204658885	N7:777019	36,146	+14:YGR143W; +5:SUT562;	14			
chrVII	776982	777149	167	-1,355650916	N7:777019	36,146	+14:YGR143W; +5:SUT562;	14			
chrVII	780856	781004	148	-1,045575989	N7:780944	21,173	+4:YGR144W;	4			
chrVII	781219	781380	161	-1,300784208	N7:781318	18,534	+6:YGR144W;	6			
chrVII	782373	782522	149	-1,302708936				Overlap <50 bp			
chrVII	782985	783136	151	-1,493305693	N7:783055	19,553	+9:YGR145W;	9			
chrVII	811114	811251	137	-1,648645565	N7:811183	20,461		Intergene			
chrVII	813520	813649	129	-1,268882154	N7:813530	18,175		Intergene			
chrVII	813597	813761	164	-1,300315013	N7:813691	22,502		Intergene			
chrVII	814985	815128	143	-0,979070106	N7:815042	14,189		Intergene			
chrVII	816260	816426	166	-1,608770339	N7:816238	45,181		Intergene			
chrVII	817064	817213	149	-1,449977044	N7:817170	21,572		Intergene			
chrVII	817068	817218	150	-1,278634937	N7:817170	21,572		Intergene			
chrVII	817863	817968	105	-1,791400316	N7:817860	38,837		Intergene			
chrVII	817863	817970	107	-1,525364634	N7:817860	38,837		Intergene			
chrVII	819019	819169	150	-1,313251878	N7:819103	24,007		Intergene			
chrVII	819762	819909	147	-1,366069831	N7:819930	29,698		Intergene			
chrVII	820415	820572	157	-1,557311394	N7:820493	15,948		Intergene			
chrVII	820601	820751	150	-1,132202479	N7:820670	23,352		Intergene			
chrVII	820792	820937	145	-1,019939731				Overlap <50 bp			
chrVII	821063	821198	135	-1,041413445	N7:821136	20,841		Intergene			
chrVII	821345	821513	168	-1,220367417	N7:821422	24,96		Intergene			
chrVII	822101	822241	140	-1,157950276	N7:822182	22,338		Intergene			
chrVII	822107	822241	134	-1,10336759	N7:822182	22,338		Intergene			
chrVII	822519	822674	155	-1,621234488	N7:822552	24,502		Intergene			
chrVII	822650	822758	108	-0,829525841	N7:822752	38,083		Intergene			
chrVII	825754	825897	143	-1,137284697	N7:825833	17,326	+11:YGR162W;	11			
chrVII	831865	832015	150	-1,317760078	N7:831995	7,176	+10:YGR166W; +2:SUT565;	10			
chrVII	841785	841939	154	-1,362164835	N7:841891	16,415	+5:YGR171C;	5			

CHR	START	END	L(bp)	ΔLknuc	Nuc ID	Fuzziness	Gene ID	Gene body postion or intergene	Terminal	rDNA	Telomeric
chrVII	842909	843085	176	**-1,36868009**	N7:843080	3,886	+4:YGR172C;	4			
chrVII	842909	843085	176	**-1,36868009**	N7:842921	10,381	+5:YGR172C;	5			
chrVII	843924	844090	166	**-1,013010432**	N7:844011	8,477	+2:YGR173W;	2			
chrVII	844983	845124	141	**-0,92933734**	N7:845017	21,939		Intergene			
chrVII	847489	847638	149	**-1,084795454**				Overlap <50 bp			
chrVII	853632	853775	143	**-1,054244501**	N7:853718	6,473	+8*:YGR179C;	8	TERM		
chrVII	855743	855883	140	**-1,18475975**	N7:855791	22,301	+5:YGR180C;	5			
chrVII	855860	856013	153	**-1,468646796**	N7:855962	20,579	+4:YGR180C;	4			
chrVII	861234	861372	138	**-1,352465861**	N7:861384	22,728	+28:YGR184C;	28			
chrVII	869483	869631	148	**-1,450254733**	N7:869530	23,106	+12:YGR186W;	12			
chrVII	873303	873436	133	**-1,408824646**	N7:873338	16,958		Intergene			
chrVII	873927	874079	152	**-1,125799159**	N7:873992	17,844		Intergene			
chrVII	878924	879054	130	**-1,137395017**	N7:878962	4,69		Intergene			
chrVII	879023	879172	149	**-1,157572469**				Overlap <50 bp			
chrVII	879984	880088	104	**-1,39452278**				Overlap <50 bp			
chrVII	882915	883031	116	**-0,070106345**	N7:883049	13,478	+5:YGR192C;	5			
chrVII	882915	883023	108	**0,034251293**				Overlap <50 bp			
chrVII	887398	887557	159	**-1,41258926**				Overlap <50 bp			
chrVII	889980	890139	159	**-1,57606307**	N7:890158	32,249	+13:YGR196C;	13			
chrVII	889980	890139	159	**-1,57606307**	N7:890006	29,841	+14:YGR196C;	14			
chrVII	892462	892609	147	**-1,3504593**	N7:892520	10,991		Intergene			
chrVII	895680	895828	148	**-1,577695109**	N7:895781	23,114	+8:YGR198W;	8			
chrVII	911520	911682	162	**-1,215343234**	N7:911624	11,489	+1:YGR207C; -1:YGR208W;	1			
chrVII	914384	914518	134	**-0,761231872**	N7:914490	8,246	+3:YGR210C;	3			
chrVII	920349	920433	84	**-0,892818035**	N7:920408	36,509	-1:YGR214W;	-1			
chrVII	922436	922538	102	**-1,670878864**	N7:922490	5,345	+3*:YGR215W;	3	TERM		
chrVII	925873	926024	151	**-1,457316922**				Overlap <50 bp			
chrVII	928464	928607	143	**-1,544581278**				Overlap <50 bp			
chrVII	928560	928710	150	**-1,32055031**	N7:928631	26,21	+25:YGR217W;	25			
chrVII	929745	929899	154	**-1,729924862**	N7:929875	25,703		Intergene			
chrVII	938659	938812	153	**-1,649277095**	N7:938652	16,879	+4:YGR221C;	4			
chrVII	943256	943409	153	**-1,21334625**	N7:943358	25,948	+10:SUT569; +5:YGR224W;	10			
chrVII	943702	943854	152	**-1,270994004**	N7:943837	20,389	+7:SUT569; +8:YGR224W;	7			
chrVII	948590	948746	156	**-1,555273123**	N7:948665	11,219	+9:YGR227W;	9			
chrVII	949733	949881	148	**-1,34873511**	N7:949820	22,248	+8:YGR229C;	8			
chrVII	960612	960754	142	**-1,072037737**	N7:960724	28,562	+6:YGR234W;	6			
chrVII	961680	961814	134	**-1,742766225**	N7:961727	7,403	+3:YGR235C;	3			
chrVII	961681	961788	107	**-1,28236431**	N7:961727	7,403	+3:YGR235C;	3			
chrVII	961681	961791	110	**-0,777638249**	N7:961727	7,403	+3:YGR235C;	3			
chrVII	961681	961794	113	**-0,7995937**	N7:961727	7,403	+3:YGR235C;	3			
chrVII	961681	961795	114	**-0,46740488**	N7:961727	7,403	+3:YGR235C;	3			
chrVII	961681	961796	115	**-0,860428875**	N7:961727	7,403	+3:YGR235C;	3			
chrVII	961681	961797	116	**-1,290380421**	N7:961727	7,403	+3:YGR235C;	3			
chrVII	961681	961798	117	**-0,700419951**	N7:961727	7,403	+3:YGR235C;	3			
chrVII	961681	961799	118	**-1,180469031**	N7:961727	7,403	+3:YGR235C;	3			
chrVII	961681	961800	119	**-0,385064969**	N7:961727	7,403	+3:YGR235C;	3			
chrVII	961681	961801	120	**-0,791752291**	N7:961727	7,403	+3:YGR235C;	3			
chrVII	961681	961802	121	**-1,422433316**	N7:961727	7,403	+3:YGR235C;	3			
chrVII	961681	961803	122	**-1,218893163**	N7:961727	7,403	+3:YGR235C;	3			
chrVII	961681	961804	123	**-1,342559562**	N7:961727	7,403	+3:YGR235C;	3			
chrVII	961681	961805	124	**-1,234138058**	N7:961727	7,403	+3:YGR235C;	3			
chrVII	961681	961806	125	**-1,975143821**	N7:961727	7,403	+3:YGR235C;	3			
chrVII	961681	961807	126	**-1,109819015**	N7:961727	7,403	+3:YGR235C;	3			
chrVII	961681	961808	127	**-0,684855061**	N7:961727	7,403	+3:YGR235C;	3			
chrVII	961681	961809	128	**-1,068102773**	N7:961727	7,403	+3:YGR235C;	3			
chrVII	961681	961810	129	**-1,2768176**	N7:961727	7,403	+3:YGR235C;	3			
chrVII	961681	961811	130	**-1,232362305**	N7:961727	7,403	+3:YGR235C;	3			
chrVII	961681	961812	131	**-1,036456889**	N7:961727	7,403	+3:YGR235C;	3			
chrVII	961681	961813	132	**-1,433047964**	N7:961727	7,403	+3:YGR235C;	3			
chrVII	961681	961814	133	**-1,05489492**	N7:961727	7,403	+3:YGR235C;	3			
chrVII	961682	961814	132	**-1,195670018**	N7:961727	7,403	+3:YGR235C;	3			
chrVII	961683	961814	131	**-1,251596042**	N7:961727	7,403	+3:YGR235C;	3			
chrVII	961684	961814	130	**-1,588790809**	N7:961727	7,403	+3:YGR235C;	3			
chrVII	961685	961814	129	**-1,096625562**	N7:961727	7,403	+3:YGR235C;	3			
chrVII	961686	961814	128	**-0,79349347**	N7:961727	7,403	+3:YGR235C;	3			
chrVII	961687	961814	127	**-0,949634893**	N7:961727	7,403	+3:YGR235C;	3			
chrVII	961694	961814	120	**-1,163899238**	N7:961727	7,403	+3:YGR235C;	3			
chrVII	961695	961814	119	**-0,780301144**	N7:961727	7,403	+3:YGR235C;	3			
chrVII	961696	961814	118	**-1,099381997**	N7:961727	7,403	+3:YGR235C;	3			
chrVII	961708	961806	98	**-0,949572954**	N7:961727	7,403	+3:YGR235C;	3			
chrVII	962925	963072	147	**-1,341990433**	N7:963033	25,365	0:YGR236C;	0			
chrVII	966527	966637	110	**-1,359676012**	N7:966590	31,713	+14:YGR238C;	14			
chrVII	974170	974327	157	**-1,419815787**	N7:974249	28,729	-1:YGR240C; 0:SUT141;	-1			
chrVII	975022	975129	107	**-0,96158172**	N7:975032	21,194	164; -1:YGR240C-A; +12:YGR241C;	1			
chrVII	975022	975130	108	**-1,018475442**	N7:975032	21,194	164; -1:YGR240C-A; +12:YGR241C;	1			
chrVII	975022	975131	109	**-1,252938788**	N7:975032	21,194	164; -1:YGR240C-A; +12:YGR241C;	1			
chrVII	975022	975142	120	**-1,209455307**	N7:975032	21,194	164; -1:YGR240C-A; +12:YGR241C;	1			
chrVII	979943	980105	162	**-1,192441561**	N7:980040	9,68	+14:YGR245C;	14			
chrVII	982482	982630	148	**-1,079859202**	N7:982592	16,598	+11:YGR246C;	11			
chrVII	987451	987604	153	**-1,60399418**	N7:987557	29,569	-1:YGR249W;	-1			
chrVII	987451	987606	155	**-1,673811507**	N7:987557	29,569	-1:YGR249W;	-1			
chrVII	987451	987608	157	**-1,674417946**	N7:987557	29,569	-1:YGR249W;	-1			
chrVII	987451	987609	158	**-1,429625702**	N7:987557	29,569	-1:YGR249W;	-1			
chrVII	988814	988967	153	**-1,24153461**	N7:988944	20,516	+8:YGR249W;	8			
chrVII	988881	989019	138	**-0,803715516**	N7:988944	20,516	+8:YGR249W;	8			
chrVII	989319	989475	156	**-1,197266065**	N7:989398	12,219	+11:YGR249W;	11			
chrVII	989319	989477	158	**-1,328625083**	N7:989398	12,219	+11:YGR249W;	11			
chrVII	991949	992103	154	**-1,189031495**	N7:992011	23,264	+12:YGR250C;	12			
chrVII	999326	999473	147	**-1,464530646**	N7:999420	27,779	-1:YGR253C;	-1			
chrVII	1001929	1002078	149	**-1,127199798**	N7:1001976	38,888	+7:YGR254W;	7			
chrVII	1012079	1012238	159	**-1,601341534**	N7:1012073	20,617	-1:YGR260W; +2:CUT634;	-1			
chrVII	1012603	1012726	123	**-1,500318607**	N7:1012698	5,523		Intergene			
chrVII	1016668	1016789	121	**-0,969040897**	N7:1016735	11,409	+1:YGR261C;	1			
chrVII	1018529	1018686	157	**-1,091452275**	N7:1018544	6,325	+4:CUT167; +5:YGR263C;	4			
chrVII	1018639	1018770	131	**-1,028881385**	N7:1018717	13,049	+5:CUT167; +4:YGR263C;	5			
chrVII	1024687	1024847	160	**-0,984500662**				Overlap <50 bp			

CHR	START	END	L(bp)	ΔLknuc	Nuc ID	Fuzziness	Gene ID	Gene body postion or intergene	Terminal	rDNA	Telomeric
chrVII	1030700	1030865	165	-1,26404173	N7:1030777	23,824	+22:YGR270W;	22			
chrVII	1030899	1031060	161	-1,492972672	N7:1030983	49,859	+23:YGR270W;	23			
chrVII	1037731	1037891	160	-1,60394904	N7:1037843	23,104	+2:YGR271C-A;	2			
chrVII	1039928	1040095	167	-1,353507373	N7:1040010	30,636	+20*:YGR274C;	20	TERM		
chrVII	1043906	1044058	152	-1,109871585	N7:1044003	17,964		Intergene			
chrVII	1044184	1044344	160	-1,342215261	N7:1044171	23,02		Intergene			
chrVII	1045755	1045901	146	-1,234862619	N7:1045865	12,75	+5:YGR277C; -1:YGR276C;	5			
chrVII	1049209	1049360	151	-1,38506989	N7:1049248	27,636	-1:SUT144; +6:YGR279C;	-1			
chrVII	1051642	1051789	147	-1,049867551	N7:1051693	14,588	+1:YGR280C;	1			
chrVII	1052012	1052165	153	-1,257690487	N7:1052015	11,021	0:Unit253; -1:YGR280C;	0			
chrVII	1065087	1065242	155	-1,323574383	N7:1065159	8,976	0:YGR286C;	0			
chrVII	1065864	1066016	152	-1,117659017	N7:1065932	11,345	+3*:CUT170;	3	TERM		
chrVII	1065866	1066016	150	-1,387352075	N7:1065932	11,345	+3*:CUT170;	3	TERM		
chrVII	1065867	1066016	149	-1,215335159	N7:1065932	11,345	+3*:CUT170;	3	TERM		
chrVII	1067431	1067584	153	-1,321335082	N7:1067518	36,979	+11:YGR287C;	11			
chrVII	1071145	1071303	158	-1,235928613	N7:1071240	35,836	+7:YGR288W;	7			
chrVII	1079049	1079187	138	-1,661784877	N7:1079141	4,792	+4:Unit255; +4:Unit287;	4			
chrVII	1079684	1079846	162	-1,349728491	N7:1079686	11,345	+1:Unit255;	1			
chrVII	1080323	1080452	129	-1,313721496	N7:1080409	41,191	+2:YGR294W;	2			
chrVII	1085801	1085942	141	-1,211450567	N7:1085920	22,113	+7:YGR296W;	7			TEL RIGHT
chrVII	1087005	1087167	162	-1,320183316	N7:1086993	15,567	+13:YGR296W;	13			TEL RIGHT
chrVII	1087005	1087167	162	-1,320183316	N7:1087148	18	+14:YGR296W;	14			TEL RIGHT
chrVII	1087106	1087261	155	-1,844548914	N7:1087148	18	+14:YGR296W;	14			TEL RIGHT
chrVII	1087240	1087377	137	-1,199143123	N7:1087315	9,815	+15:YGR296W;	15			TEL RIGHT
chrVII	1087527	1087678	151	-1,23742729	N7:1087597	17	+17:YGR296W;	17			TEL RIGHT
chrVII	1087535	1087678	143	-1,572093981	N7:1087597	17	+17:YGR296W;	17			TEL RIGHT
chrVII	1087583	1087708	125	-0,693715414	N7:1087597	17	+17:YGR296W;	17			TEL RIGHT
chrVII	1087808	1087960	152	-1,219283862	N7:1087922	14,224	+19:YGR296W;	19			TEL RIGHT
chrVII	1087881	1088025	144	-1,023063265	N7:1087922	14,224	+19:YGR296W;	19			TEL RIGHT
chrVII	1087978	1088113	135	-0,993309501	N7:1088072	37,041	+20:YGR296W;	20			TEL RIGHT
chrVII	1087978	1088165	187	-0,838896224	N7:1088072	37,041	+20:YGR296W;	20			TEL RIGHT
chrVII	1088021	1088113	92	-0,478563188	N7:1088072	37,041	+20:YGR296W;	20			TEL RIGHT
chrVII	1088021	1088165	144	-0,955510176	N7:1088072	37,041	+20:YGR296W;	20			TEL RIGHT
chrVII	1088360	1088512	152	-1,377737626	N7:1088436	17,673	+22:YGR296W;	22			TEL RIGHT
chrVII	1088661	1088791	130	-0,959485957	N7:1088758	37,242	+24:YGR296W;	24			TEL RIGHT
chrVII	1089031	1089182	151	-1,256789845	N7:1089163	38,734	+26:YGR296W;	26			TEL RIGHT
chrVII	1089036	1089170	134	-0,957153105	N7:1089163	38,734	+26:YGR296W;	26			TEL RIGHT
chrVII	1089049	1089145	96	-0,702783422	N7:1089163	38,734	+26:YGR296W;	26			TEL RIGHT
chrVII	1090196	1090347	151	-1,276105724	N7:1090283	36,77	+32:YGR296W;	32			TEL RIGHT
chrVIII	691	842	151	-1,214555062	N8:754	11,136	+18:YHL050C;	18			TEL LEFT
chrVIII	1289	1405	116	-0,794251441	N8:1300	28,74	+14:YHL050C;	14			TEL LEFT
chrVIII	1346	1501	155	-1,310772488				Overlap <50 bp			TEL LEFT
chrVIII	1712	1899	187	-1,508362362	N8:1845	18,083	+10:YHL050C;	10			TEL LEFT
chrVIII	1733	1899	166	-2,25101612	N8:1845	18,083	+10:YHL050C;	10			TEL LEFT
chrVIII	1748	1846	98	-0,827137784	N8:1845	18,083	+10:YHL050C;	10			TEL LEFT
chrVIII	1748	1855	107	-1,084236509	N8:1845	18,083	+10:YHL050C;	10			TEL LEFT
chrVIII	1748	1878	130	-1,568753508	N8:1845	18,083	+10:YHL050C;	10			TEL LEFT
chrVIII	1748	1879	131	-1,015364392	N8:1845	18,083	+10:YHL050C;	10			TEL LEFT
chrVIII	1748	1880	132	-1,185239046	N8:1845	18,083	+10:YHL050C;	10			TEL LEFT
chrVIII	1748	1889	141	-1,416915625	N8:1845	18,083	+10:YHL050C;	10			TEL LEFT
chrVIII	1748	1891	143	-1,351785569	N8:1845	18,083	+10:YHL050C;	10			TEL LEFT
chrVIII	1748	1894	146	-1,027556964	N8:1845	18,083	+10:YHL050C;	10			TEL LEFT
chrVIII	1748	1895	147	-1,391704254	N8:1845	18,083	+10:YHL050C;	10			TEL LEFT
chrVIII	1748	1896	148	-1,128648232	N8:1845	18,083	+10:YHL050C;	10			TEL LEFT
chrVIII	1748	1897	149	-1,415258873	N8:1845	18,083	+10:YHL050C;	10			TEL LEFT
chrVIII	1748	1898	150	-1,719025954	N8:1845	18,083	+10:YHL050C;	10			TEL LEFT
chrVIII	1748	1899	151	-1,15736106	N8:1845	18,083	+10:YHL050C;	10			TEL LEFT
chrVIII	1748	1909	161	-2,11615602	N8:1845	18,083	+10:YHL050C;	10			TEL LEFT
chrVIII	1748	1932	184	-1,904963662	N8:1845	18,083	+10:YHL050C;	10			TEL LEFT
chrVIII	1749	1899	150	-1,575487647	N8:1845	18,083	+10:YHL050C;	10			TEL LEFT
chrVIII	1750	1899	149	-1,574888675	N8:1845	18,083	+10:YHL050C;	10			TEL LEFT
chrVIII	1751	1899	148	-1,634679602	N8:1845	18,083	+10:YHL050C;	10			TEL LEFT
chrVIII	1752	1899	147	-1,263349294	N8:1845	18,083	+10:YHL050C;	10			TEL LEFT
chrVIII	1753	1899	146	-1,162816376	N8:1845	18,083	+10:YHL050C;	10			TEL LEFT
chrVIII	1754	1899	145	-1,309440533	N8:1845	18,083	+10:YHL050C;	10			TEL LEFT
chrVIII	1755	1899	144	-1,877474038	N8:1845	18,083	+10:YHL050C;	10			TEL LEFT
chrVIII	1756	1899	143	-1,666952	N8:1845	18,083	+10:YHL050C;	10			TEL LEFT
chrVIII	1757	1899	142	-1,308406001	N8:1845	18,083	+10:YHL050C;	10			TEL LEFT
chrVIII	1758	1899	141	-1,439200269	N8:1845	18,083	+10:YHL050C;	10			TEL LEFT
chrVIII	1759	1899	140	-1,236253829	N8:1845	18,083	+10:YHL050C;	10			TEL LEFT
chrVIII	1760	1899	139	-1,438682121	N8:1845	18,083	+10:YHL050C;	10			TEL LEFT
chrVIII	1761	1899	138	-1,885350855	N8:1845	18,083	+10:YHL050C;	10			TEL LEFT
chrVIII	1762	1899	137	-1,738108503	N8:1845	18,083	+10:YHL050C;	10			TEL LEFT
chrVIII	1764	1899	135	-1,2406654	N8:1845	18,083	+10:YHL050C;	10			TEL LEFT
chrVIII	1765	1899	134	-1,546702362	N8:1845	18,083	+10:YHL050C;	10			TEL LEFT
chrVIII	1766	1899	133	-0,976808376	N8:1845	18,083	+10:YHL050C;	10			TEL LEFT
chrVIII	1769	1899	130	-0,800299475	N8:1845	18,083	+10:YHL050C;	10			TEL LEFT
chrVIII	1771	1899	128	-1,549747623	N8:1845	18,083	+10:YHL050C;	10			TEL LEFT
chrVIII	1773	1899	126	-1,401502018	N8:1845	18,083	+10:YHL050C;	10			TEL LEFT
chrVIII	1784	1899	115	-0,689805015	N8:1845	18,083	+10:YHL050C;	10			TEL LEFT
chrVIII	1821	1968	147	-0,636263276	N8:1845	18,083	+10:YHL050C;	10			TEL LEFT
chrVIII	2051	2222	171	-0,903431168				Overlap <50 bp			TEL LEFT
chrVIII	2441	2594	153	-1,141610292	N8:2474	10,408	+6:YHL050C;	6			TEL LEFT
chrVIII	2951	3103	152	-1,323567952	N8:2994	19,192	+3:YHL050C;	3			TEL LEFT
chrVIII	3093	3220	127	-0,996200907	N8:3153	6,325	+2:YHL050C;	2			TEL LEFT
chrVIII	3233	3376	143	-1,196774043	N8:3313	10,786	+1:YHL050C;	1			TEL LEFT
chrVIII	3524	3661	137	-1,151491737	N8:3599	7,095	-1:YHL050C; +7*:YHL049C;	-1			TEL LEFT
chrVIII	4667	4822	155	-1,561009173	N8:4741	23,335	0:YHL049C;	0			TEL LEFT
chrVIII	9299	9453	154	-1,423690734	N8:9352	24,533	+6:YHL047C;	6			
chrVIII	10577	10683	106	-1,310720016	N8:10600	5,292	0:SUT147;	0			
chrVIII	10578	10683	105	-1,637540864	N8:10600	5,292	0:SUT147;	0			
chrVIII	10864	11003	139	-0,908851512	N8:10940	16,869	+2:SUT147;	2			
chrVIII	12729	12877	148	-1,602577493	N8:12794	9,311		Intergene			
chrVIII	14994	15154	160	-1,234168136	N8:15064	43,65	+2:YHL043W; +6:SUT573;	2			
chrVIII	15294	15466	172	-1,497801652	N8:15381	9,839	+4:YHL043W; +4:SUT573;	4			
chrVIII	17876	18017	141	-1,242839156	N8:18009	25,395		Intergene			

CHR	START	END	L(bp)	ΔLknuc	Nuc ID	Fuzziness	Gene ID	Gene body postion or intergene	Terminal	rDNA	Telomeric
chrVIII	18350	18522	172	**-1,469435581**	N8:18473	28,374		Intergene			
chrVIII	23920	24090	170	**-1,702762371**	N8:24051	18,708	+10:YHL038C;	10			
chrVIII	26981	27134	153	**-1,132259381**	N8:27018	21,775	+6:YHL036W;	6			
chrVIII	32520	32666	146	**-1,238756396**	N8:32599	26,876	+2:YHL035C;	2			
chrVIII	33329	33481	152	**-1,253260673**	N8:33481	35,088	+5:YHL034C;	5			
chrVIII	33329	33481	152	**-1,253260673**	N8:33328	25,521	+6:YHL034C;	6			
chrVIII	33632	33782	150	**-1,172043085**	N8:33647	7,765	+4:YHL034C;	4			
chrVIII	39986	40150	164	**-1,43506912**	N8:40093	7,202	+1:YHL030W;	1			
chrVIII	46044	46208	164	**-1,237177152**	N8:46074	8,335	+13:YHL029C;	13			
chrVIII	48223	48365	142	**-0,909004197**	N8:48283	3,536	-1:YHL029C;	-1			
chrVIII	49771	49922	151	**-1,230784042**	N8:49832	12,816	+7:YHL028W;	7			
chrVIII	50265	50415	150	**-0,795046436**	N8:50342	18,779	+2:CUT637; +10:YHL028W;	2			
chrVIII	51501	51665	164	**-1,200528029**	N8:51521	23,53	+4:YHL027W;	4			
chrVIII	52096	52204	108	**-1,255071045**	N8:52145	35,726	+8:YHL027W;	8			
chrVIII	52096	52213	117	**-1,411502374**	N8:52145	35,726	+8:YHL027W;	8			
chrVIII	52629	52803	174	**-1,418183622**	N8:52786	5,468	+11:YHL027W;	11			
chrVIII	53713	53863	150	**-1,148618579**	N8:53785	11,374	+3:YHL026C;	3			
chrVIII	58605	58733	128	**-0,514590521**	N8:58676	27,193	+13:YHL024W; +2:SUT574;	13			
chrVIII	59349	59502	153	**-1,245938153**	N8:59376	25,918	+2:SUT148;	2			
chrVIII	61087	61247	160	**-1,87221545**	N8:61111	11,354	+10:YHL023C;	10			
chrVIII	62149	62261	112	**-1,290683303**	N8:62222	5,404	+3:YHL023C;	3			
chrVIII	65254	65423	169	**-1,214235674**	N8:65391	17,011	+4:YHL021C;	4			
chrVIII	66426	66576	150	**-0,977488551**	N8:66523	12,069	+7:YHL020C;	7			
chrVIII	66660	66810	150	**-1,326633142**	N8:66683	8,044	+6:YHL020C;	6			
chrVIII	66812	66936	124	**-1,43438553**	N8:66852	3	+5:YHL020C;	5			
chrVIII	66813	66902	89	**-0,484570609**	N8:66852	3	+5:YHL020C;	5			
chrVIII	66813	66904	91	**-0,975525697**	N8:66852	3	+5:YHL020C;	5			
chrVIII	66813	66911	98	**-0,951864323**	N8:66852	3	+5:YHL020C;	5			
chrVIII	66813	66912	99	**-0,839639659**	N8:66852	3	+5:YHL020C;	5			
chrVIII	66813	66924	111	**-0,464190944**	N8:66852	3	+5:YHL020C;	5			
chrVIII	66813	66925	112	**-0,628096934**	N8:66852	3	+5:YHL020C;	5			
chrVIII	66813	66926	113	**-1,053373951**	N8:66852	3	+5:YHL020C;	5			
chrVIII	66813	66928	115	**-1,035733416**	N8:66852	3	+5:YHL020C;	5			
chrVIII	66813	66929	116	**-0,920060753**	N8:66852	3	+5:YHL020C;	5			
chrVIII	66813	66930	117	**-1,22746726**	N8:66852	3	+5:YHL020C;	5			
chrVIII	66813	66931	118	**-1,157170762**	N8:66852	3	+5:YHL020C;	5			
chrVIII	66813	66932	119	**-0,975825326**	N8:66852	3	+5:YHL020C;	5			
chrVIII	66813	66933	120	**-0,939460335**	N8:66852	3	+5:YHL020C;	5			
chrVIII	66813	66934	121	**-0,640567728**	N8:66852	3	+5:YHL020C;	5			
chrVIII	66813	66935	122	**-1,07838856**	N8:66852	3	+5:YHL020C;	5			
chrVIII	66813	66936	123	**-0,870093444**	N8:66852	3	+5:YHL020C;	5			
chrVIII	66815	66936	121	**-1,250420064**	N8:66852	3	+5:YHL020C;	5			
chrVIII	66816	66936	120	**-1,405470966**	N8:66852	3	+5:YHL020C;	5			
chrVIII	66817	66936	119	**-1,13002513**	N8:66852	3	+5:YHL020C;	5			
chrVIII	66818	66936	118	**-0,738377312**	N8:66852	3	+5:YHL020C;	5			
chrVIII	66820	66936	116	**-1,280546637**	N8:66852	3	+5:YHL020C;	5			
chrVIII	66821	66936	115	**-0,546113539**	N8:66852	3	+5:YHL020C;	5			
chrVIII	66822	66936	114	**-0,733222464**	N8:66852	3	+5:YHL020C;	5			
chrVIII	66823	66936	113	**-1,106581507**	N8:66852	3	+5:YHL020C;	5			
chrVIII	66830	66936	106	**-0,869579508**	N8:66852	3	+5:YHL020C;	5			
chrVIII	68261	68399	138	**-0,93731092**	N8:68264	38,448		Intergene			
chrVIII	69975	70096	121	**-0,397908483**	N8:70043	7,603	-1:YHL017W; +3:YHL018W;	-1			
chrVIII	69975	70122	147	**-1,005752188**	N8:70043	7,603	-1:YHL017W; +3:YHL018W;	-1			
chrVIII	70402	70553	151	**-1,312551993**	N8:70464	9,475	+2:YHL017W;	2			
chrVIII	72065	72199	134	**-1,136126209**	N8:72067	24,511	+14:YHL016C;	14			
chrVIII	77481	77612	131	**-0,643425909**	N8:77523	20,918	-1:YHL014C;	-1			
chrVIII	86148	86310	162	**-1,684499431**	N8:86173	7,024		Intergene			
chrVIII	86176	86324	148	**-1,115642165**	N8:86173	7,024		Intergene			
chrVIII	86476	86623	147	**-1,411195094**	N8:86566	12,858		Intergene			
chrVIII	88963	89116	153	**-1,926932052**	N8:89119	23,847		Intergene			
chrVIII	91372	91534	162	**-1,593772132**	N8:91357	31,113		Intergene			
chrVIII	91372	91534	162	**-1,593772132**	N8:91547	35,557		Intergene			
chrVIII	94804	94953	149	**-1,065940992**	N8:94867	15,145	-1:YHL008C;	-1			
chrVIII	94985	95141	156	**-1,397346819**	N8:95070	19,956	+19*:YHL007C;	19	TERM		
chrVIII	96353	96506	153	**-1,136074458**	N8:96448	34,452	+11:YHL007C; +2*:SUT150;	11			
chrVIII	100385	100534	149	**-1,798941269**	N8:100544	36,981	+10*:YHL003C; +9*:YHL004W;	10	TERM		
chrVIII	107252	107412	160	**-1,518043361**	N8:107297	1,949	+9*:YHR001W;	9	TERM		
chrVIII	108345	108493	148	**-1,562329229**	N8:108419	9,99	-1:YHR002W;	-1			
chrVIII	109527	109701	174	**-1,566944703**	N8:109636	27,373	+7*:YHR002W;	7	TERM		
chrVIII	114779	114932	153	**-1,16204338**	N8:114850	11,147	+2:YHR005C;	2			
chrVIII	115531	115709	178	**-1,454019066**	N8:115711	7,868	+2*:CUT173; +2:YHR005C-A;	2	TERM		
chrVIII	121476	121637	161	**-1,100409818**	N8:121495	19,604	+3:YHR007C;	3			
chrVIII	122292	122451	159	**-0,791064114**	N8:122379	11,269	+4*:YHR007C-A; +4:SUT153;	4	TERM		
chrVIII	123820	123978	158	**-1,410352592**	N8:123911	7,653	-1:YHR008C; +14*:SUT153;	-1			
chrVIII	125638	125758	120	**-0,777011762**	N8:125733	19,601	+2:YHR009C;	2			
chrVIII	130844	131002	158	**-1,275527539**	N8:130922	7,731	+4:YHR013C;	4			
chrVIII	131196	131345	149	**-1,042345871**	N8:131260	6,802	+2:YHR013C;	2			
chrVIII	142630	142776	146	**-1,134598201**	N8:142760	8,438	+6:YHR019C;	6			
chrVIII	144193	144334	141	**-0,912713065**	N8:144264	7,085	+3:YHR020W;	3			
chrVIII	152201	152353	152	**-1,462538567**	N8:152258	6,274	+5:YHR023W;	5			
chrVIII	153827	153949	122	**-1,416468334**	N8:153924	14,572	+15:YHR023W;	15			
chrVIII	157804	157963	159	**-1,039803567**	N8:157803	35,25		Intergene			
chrVIII	158390	158542	152	**-1,143965281**	N8:158513	30	+5:YHR024C;	5			
chrVIII	162439	162593	154	**-1,122715696**	N8:162524	24,161	+14:YHR027C;	14			
chrVIII	162949	163087	138	**-0,772142831**				Overlap <50 bp			
chrVIII	165932	166076	144	**-1,089462702**	N8:165989	4,868	+10:YHR028C; +7:SUT155;	10			
chrVIII	167959	168110	151	**-1,376459633**	N8:168035	6,47	+4:YHR029C;	4			
chrVIII	171020	171171	151	**-1,064260495**	N8:171084	8,214	+3:CUT175; +13:YHR031C;	3			
chrVIII	171703	171855	152	**-1,279124868**	N8:171826	49,31	+8:YHR031C;	8			
chrVIII	174446	174586	140	**-2,483796618**				Overlap <50 bp			
chrVIII	174447	174533	86	**-1,757935898**				Overlap <50 bp			
chrVIII	174447	174535	88	**-1,403734994**				Overlap <50 bp			
chrVIII	174447	174538	91	**-1,135103149**				Overlap <50 bp			
chrVIII	174447	174540	93	**-1,334705523**				Overlap <50 bp			
chrVIII	174447	174541	94	**-1,688759971**				Overlap <50 bp			
chrVIII	174447	174544	97	**-1,848430106**				Overlap <50 bp			

CHR	START	END	L(bp)	ΔLknuc	Nuc ID	Fuzziness	Gene ID	Gene body postion or intergene	Terminal	rDNA	Telomeric
chrVIII	174447	174545	98	**-1,886644163**				Overlap <50 bp			
chrVIII	174447	174548	101	**-1,367309311**				Overlap <50 bp			
chrVIII	174447	174550	103	**-1,337354548**				Overlap <50 bp			
chrVIII	174447	174551	104	**-1,429594893**				Overlap <50 bp			
chrVIII	174447	174552	105	**-1,33918531**				Overlap <50 bp			
chrVIII	174447	174553	106	**-2,212746809**				Overlap <50 bp			
chrVIII	174447	174555	108	**-1,393899438**				Overlap <50 bp			
chrVIII	174447	174561	114	**-1,750334161**				Overlap <50 bp			
chrVIII	174447	174565	118	**-1,755634194**				Overlap <50 bp			
chrVIII	174447	174566	119	**-1,583491617**				Overlap <50 bp			
chrVIII	174447	174567	120	**-1,519140194**				Overlap <50 bp			
chrVIII	174447	174568	121	**-1,50479122**				Overlap <50 bp			
chrVIII	174447	174569	122	**-1,172773356**				Overlap <50 bp			
chrVIII	174447	174570	123	**-1,305586702**				Overlap <50 bp			
chrVIII	174447	174571	124	**-1,086245943**				Overlap <50 bp			
chrVIII	174447	174572	125	**-1,516021409**				Overlap <50 bp			
chrVIII	174447	174573	126	**-1,339498373**				Overlap <50 bp			
chrVIII	174447	174574	127	**-1,54327693**				Overlap <50 bp			
chrVIII	174447	174575	128	**-1,429042701**				Overlap <50 bp			
chrVIII	174447	174576	129	**-1,233539464**				Overlap <50 bp			
chrVIII	174447	174577	130	**-1,196052957**				Overlap <50 bp			
chrVIII	174447	174578	131	**-1,258911953**				Overlap <50 bp			
chrVIII	174447	174579	132	**-1,242932972**				Overlap <50 bp			
chrVIII	174447	174580	133	**-1,372127814**				Overlap <50 bp			
chrVIII	174447	174581	134	**-1,386338066**				Overlap <50 bp			
chrVIII	174447	174582	135	**-1,238892414**				Overlap <50 bp			
chrVIII	174447	174583	136	**-1,378172993**				Overlap <50 bp			
chrVIII	174447	174584	137	**-1,574889836**				Overlap <50 bp			
chrVIII	174447	174585	138	**-1,747809058**				Overlap <50 bp			
chrVIII	174447	174586	139	**-1,21136349**				Overlap <50 bp			
chrVIII	174447	174587	140	**-1,793791499**				Overlap <50 bp			
chrVIII	174448	174586	138	**-1,652348415**				Overlap <50 bp			
chrVIII	174449	174586	137	**-1,599178604**				Overlap <50 bp			
chrVIII	174450	174586	136	**-1,587297859**				Overlap <50 bp			
chrVIII	174451	174586	135	**-1,358499898**				Overlap <50 bp			
chrVIII	174452	174586	134	**-1,486654464**				Overlap <50 bp			
chrVIII	174453	174586	133	**-1,698119925**				Overlap <50 bp			
chrVIII	174454	174586	132	**-1,85214847**				Overlap <50 bp			
chrVIII	174455	174586	131	**-1,244822515**				Overlap <50 bp			
chrVIII	174456	174586	130	**-1,414295965**				Overlap <50 bp			
chrVIII	174457	174586	129	**-1,458023269**				Overlap <50 bp			
chrVIII	174458	174586	128	**-1,15552345**				Overlap <50 bp			
chrVIII	174459	174586	127	**-1,500069553**				Overlap <50 bp			
chrVIII	174460	174586	126	**-1,440462389**				Overlap <50 bp			
chrVIII	174461	174586	125	**-1,935941559**				Overlap <50 bp			
chrVIII	174462	174553	91	**-0,297957357**				Overlap <50 bp			
chrVIII	174467	174586	119	**-2,091322845**				Overlap <50 bp			
chrVIII	174928	175084	156	**-1,725251745**	N8:175011	18,389	+11:YHR032W;	11			
chrVIII	176211	176366	155	**-0,922480506**	N8:176283	9,287	+6:YHR033W;	6			
chrVIII	177719	177867	148	**-1,16595669**	N8:177804	4,875	+2:YHR034C;	2			
chrVIII	177797	177947	150	**-1,134243544**	N8:177804	4,875	+2:YHR034C;	2			
chrVIII	178837	178985	148	**-1,015099819**	N8:178874	14,152	+5:YHR035W;	5			
chrVIII	180375	180509	134	**-0,755025009**	N8:180443	11,415	+2:YHR036W;	2			
chrVIII	190177	190319	142	**-1,248374651**	N8:190199	11,93	+7*:SUT156;	7	TERM		
chrVIII	194784	194941	157	**-1,705812859**				Overlap <50 bp			
chrVIII	194987	195122	135	**-1,013025373**	N8:195028	18,082	-1:YHR044C;	-1			
chrVIII	205654	205804	150	**-1,30716269**	N8:205739	10,012	+8:YHR048W;	8			
chrVIII	207083	207225	142	**-0,925799823**	N8:207213	3,83	-1:YHR050W; +6*:YHR049W;	-1			
chrVIII	212526	212683	157	**-1,277206009**	N8:212615	13,503	+2:YHR053C;	2			
chrVIII	212729	212874	145	**-1,300209905**	N8:212883	12,728	0:YHR053C;	0			
chrVIII	212963	213069	106	**-1,410910171**	N8:213049	35,355	+9*:YHR054C; -1:YHR053C;	9	TERM		
chrVIII	212963	213076	113	**-1,647861438**	N8:213049	35,355	+9*:YHR054C; -1:YHR053C;	9	TERM		
chrVIII	213025	213187	162	**-1,304875911**	N8:213049	35,355	+9*:YHR054C; -1:YHR053C;	9	TERM		
chrVIII	214079	214248	169	**-1,33370417**	N8:214186	30,414	+2:YHR054C;	2			
chrVIII	214176	214329	153	**-1,56138711**	N8:214186	30,414	+2:YHR054C;	2			
chrVIII	214524	214681	157	**-1,280571047**	N8:214628	15,631	-1:YHR054C; +2:YHR055C;	-1			
chrVIII	214727	214872	145	**-1,0610766**	N8:214799	4,95	+1:YHR055C;	1			
chrVIII	214961	215067	106	**-1,413393278**	N8:215040	24,749	-1:YHR055C;	-1			
chrVIII	214961	215074	113	**-1,638190833**	N8:215040	24,749	-1:YHR055C;	-1			
chrVIII	215023	215185	162	**-1,275500244**	N8:215040	24,749	-1:YHR055C;	-1			
chrVIII	216077	216246	169	**-1,611889619**	N8:216180	23,866	+10*:YHR056C;	10	TERM		
chrVIII	217808	217962	154	**-1,252549277**	N8:217835	7,394	+1:YHR056C; -1:anti-YHR057C;	1			
chrVIII	218430	218569	139	**-1,519807445**	N8:218465	31,519	+3:YHR057C;	3			
chrVIII	219279	219430	151	**-1,245124527**	N8:219365	5,345	+3:CUT179; +4:YHR058C;	3			
chrVIII	223655	223760	105	**-1,429807162**	N8:223731	8,612	+5*:SUT159; +1:YHR062C;	5	TERM		
chrVIII	226108	226256	148	**-0,489329589**	N8:226185	7,81	+7:YHR064C;	7			
chrVIII	227838	227999	161	**-1,515272845**	N8:228012	53,866	+7:YHR065C;	7			
chrVIII	227838	227999	161	**-1,515272845**	N8:227836	26,621	+8:YHR065C;	8			
chrVIII	228803	228946	143	**-1,059057729**	N8:228877	11,788	+2:YHR065C;	2			
chrVIII	234383	234536	153	**-1,334466065**	N8:234466	3,619	+2:YHR069C;	2			
chrVIII	234544	234693	149	**-1,239096513**	N8:234634	7,969	-1:YHR070W; +1:YHR069C;	-1			
chrVIII	240775	240923	148	**-1,197731409**	N8:240835	30,507	+12:YHR072W;	12			
chrVIII	250570	250724	154	**-0,987295623**	N8:250736	14,77	+2:SUT581;	2			
chrVIII	256594	256759	165	**-1,627989876**	N8:256675	19,235	+3:YHR078W;	3			
chrVIII	258714	258856	142	**-0,991625696**	N8:258740	30,827	+19:YHR079C;	19			
chrVIII	261335	261482	147	**-1,269476242**	N8:261454	6,463	+3:YHR079C;	3			
chrVIII	264809	264956	147	**-1,11333577**	N8:264814	27,568	+14:YHR080C;	14			
chrVIII	270516	270670	154	**-1,40332622**	N8:270646	24,98	+7:YHR082C;	7			
chrVIII	272692	272812	120	**-0,965491674**	N8:272823	2,345	+2:YHR083W;	2			
chrVIII	275159	275317	158	**-1,166809161**				Overlap <50 bp			
chrVIII	281596	281745	149	**-1,22580215**	N8:281674	6,512	+2:YHR088W;	2			
chrVIII	288944	289050	106	**-0,932917537**	N8:288960	4,163	0:YHR092C;	0			
chrVIII	291607	291756	149	**-1,032527556**	N8:291704	10	+6:YHR094C; +5:SUT163;	6			
chrVIII	291938	292090	152	**-1,176345208**	N8:292062	41,088	+4:YHR094C; +7:SUT163;	4			
chrVIII	294718	294863	145	**-1,085619824**	N8:294786	16,078	+11:YHR096C; +5:SUT164;	11			
chrVIII	297474	297608	134	**-1,022219934**				Overlap <50 bp			

CHR	START	END	L(bp)	ΔLknuc	Nuc ID	Fuzziness	Gene ID	Gene body postion or intergene	Terminal	rDNA	Telomeric
chrVIII	297952	298079	127	**-0,792051085**				Overlap <50 bp			
chrVIII	299058	299223	165	**-1,335808728**	N8:299169	16,753	+19*:YHR098C;	19	TERM		
chrVIII	299773	299910	137	**-0,961894503**	N8:299791	50,553	+15:YHR098C;	15			
chrVIII	310121	310260	139	**-0,659400977**	N8:310259	29,744	+47:YHR099W;	47			
chrVIII	310839	310998	159	**-1,404089165**	N8:310900	14,96	+51:YHR099W;	51			
chrVIII	311969	312130	161	**-1,306490135**	N8:312117	34,174	+58:YHR099W;	58			
chrVIII	333005	333157	152	**-1,170985126**	N8:333100	13,467	+1:YHR111W;	1			
chrVIII	334511	334659	148	**-1,238584528**	N8:334641	12,39	+7:YHR112C; +2:SUT166;	7			
chrVIII	341021	341173	152	**-1,644478449**	N8:341133	6,058	+3:YHR115C;	3			
chrVIII	341021	341178	157	**-1,312389427**	N8:341133	6,058	+3:YHR115C;	3			
chrVIII	342001	342158	157	**-1,208299946**	N8:342074	21,135	+3:YHR116W; -1:YHR117W;	3			
chrVIII	342217	342368	151	**-1,480422533**	N8:342346	17,487	+5:YHR116W; +1:YHR117W;	5			
chrVIII	343838	343999	161	**-1,330576912**	N8:344006	22,521	+15:YHR116W; +11:YHR117W;	15			
chrVIII	345951	346111	160	**-1,564221048**	N8:346037	30,244	+2:YHR119W;	2			
chrVIII	346434	346582	148	**-1,662165903**	N8:346511	21,836	+5:YHR119W;	5			
chrVIII	350191	350346	155	**-1,386031371**	N8:350266	8,612	+5:YHR120W;	5			
chrVIII	353263	353415	152	**-1,522945572**				Overlap <50 bp			
chrVIII	354917	355076	159	**-1,548652808**	N8:355010	8,542		Intergene			
chrVIII	355941	356089	148	**-1,20876205**	N8:356016	8,914		Intergene			
chrVIII	357549	357703	154	**-1,039656568**	N8:357581	28,314	+7:YHR124W; +4:anti074;	7			
chrVIII	360834	360982	148	**-1,458143089**	N8:360921	13,197	+1:YHR127W;	1			
chrVIII	364130	364290	160	**-1,271346267**	N8:364200	8,612	-1:SUT167; +1:YHR129C;	-1			
chrVIII	370648	370829	181	**-1,423306518**	N8:370765	23,128	+6:YHR133C;	6			
chrVIII	371736	371883	147	**-1,504276641**	N8:371818	5,727	-1:YHR133C; +1:YHR134W;	-1			
chrVIII	374061	374221	160	**-1,224398193**	N8:374205	4,658	+2:YHR135C;	2			
chrVIII	374061	374255	194	**-1,372099878**	N8:374205	4,658	+2:YHR135C;	2			
chrVIII	374061	374221	160	**-1,224398193**	N8:374049	6,033	+3:YHR135C;	3			
chrVIII	374061	374255	194	**-1,372099878**	N8:374049	6,033	+3:YHR135C;	3			
chrVIII	374122	374209	87	**-0,188959209**	N8:374205	4,658	+2:YHR135C;	2			
chrVIII	374122	374221	99	**-0,26867702**	N8:374205	4,658	+2:YHR135C;	2			
chrVIII	374122	374255	133	**-0,954386934**	N8:374205	4,658	+2:YHR135C;	2			
chrVIII	376459	376622	163	**-1,367194803**	N8:376456	32,253	+6:YHR137W;	6			
chrVIII	376459	376622	163	**-1,367194803**	N8:376622	10,63	+7:YHR137W;	7			
chrVIII	381490	381641	151	**-1,591911886**	N8:381642	25,373	-1:CUT650;	-1			
chrVIII	382542	382702	160	**-1,436106299**	N8:382584	5,292	+2:YHR141C;	2			
chrVIII	382976	383151	175	**-1,246339989**	N8:383155	42,505	-1:YHR142W;	-1			
chrVIII	399609	399757	148	**-1,119313118**	N8:399693	19,343	+9:YHR151C;	9			
chrVIII	402529	402689	160	**-1,318842936**	N8:402659	11,15	+1:YHR153C; -1:YHR154W;	1			
chrVIII	402529	402689	160	**-1,318842936**	N8:402507	13,837	+2:YHR153C;	2			
chrVIII	403584	403737	153	**-1,325040433**	N8:403661	23,944	+5:YHR154W;	5			
chrVIII	404097	404230	133	**-0,98936652**	N8:404184	31,659	+8:YHR154W;	8			
chrVIII	414157	414333	176	**-1,559454807**	N8:414190	28,902		Intergene			
chrVIII	414157	414333	176	**-1,559454807**	N8:414356	19,07		Intergene			
chrVIII	416537	416675	138	**-0,828491242**	N8:416611	6,38	+5:YHR158C;	5			
chrVIII	424973	425127	154	**-1,170937868**	N8:425133	16,664	+27:YHR164C;	27			
chrVIII	424973	425127	154	**-1,170937868**	N8:424954	27,398	+28:YHR164C;	28			
chrVIII	427961	428122	161	**-1,297013317**	N8:428089	14,29	+8:YHR164C;	8			
chrVIII	431042	431168	126	**-0,711998868**	N8:431037	19,435	+38:YHR165C;	38			
chrVIII	433338	433488	150	**-1,328091826**	N8:433377	10,626	+23:YHR165C;	23			
chrVIII	439467	439624	157	**-1,119690507**	N8:439505	10,962	+2:YHR167W;	2			
chrVIII	444830	444988	158	**-1,419465552**	N8:444909	11,502	+8:YHR170W;	8			
chrVIII	447873	448000	127	**-1,173966999**	N8:448007	29,228	-1:YHR172W;	-1			
chrVIII	456058	456162	104	**-1,559025913**	N8:456183	19,411		Intergene			
chrVIII	465568	465728	160	**-1,241678085**	N8:465665	14,935	-1:SUT593; 0:Unit319;	-1			
chrVIII	469779	469934	155	**-1,461060373**	N8:469909	7,188		Intergene			
chrVIII	469928	470081	153	**-1,125689026**	N8:469909	7,188		Intergene			
chrVIII	469928	470081	153	**-1,125689026**	N8:470061	25,066		Intergene			
chrVIII	470995	471124	129	**-0,72567365**	N8:471041	21,059	-1:SUT594; +2:YHR183W;	-1			
chrVIII	482780	482943	163	**-1,151572456**	N8:482852	30,892	+7:YHR188C;	7			
chrVIII	485160	485318	158	**-1,031534018**	N8:485166	15,71	+3:YHR190W;	3			
chrVIII	485160	485318	158	**-1,031534018**	N8:485325	34,672	+4:YHR190W;	4			
chrVIII	495830	495995	165	**-1,321977575**	N8:495933	28,957	+13:YHR197W;	13			
chrVIII	497750	497909	159	**-1,287334229**	N8:497804	8,456	+6:YHR199C;	6			
chrVIII	501432	501582	150	**-1,583211409**	N8:501512	22,95	+2:CUT183;	2			
chrVIII	503721	503876	155	**-1,03079082**	N8:503793	30,696	+9:YHR202W;	9			
chrVIII	510274	510423	149	**-1,264522799**	N8:510320	41,677	+9:YHR205W;	9			
chrVIII	518475	518638	163	**-1,174338137**	N8:518487	33,259	+7:YHR208W;	7			
chrVIII	518475	518638	163	**-1,174338137**	N8:518641	31,729	+8:YHR208W;	8			
chrVIII	521779	521913	134	**-1,151231827**	N8:521848	15,9	0:YHR210C;	0			
chrVIII	522442	522595	153	**-1,319513397**	N8:522525	4,992	+3*:SUT174;	3	TERM		
chrVIII	523030	523182	152	**-1,106894614**	N8:523103	10,265	+15:SUT600;	15			
chrVIII	523197	523325	128	**-0,939017474**	N8:523263	12,987	+14:SUT600;	14			
chrVIII	525186	525342	156	**-1,290952622**	N8:525241	18,638	+3:YHR211W; +2:SUT600;	3			
chrVIII	526030	526198	168	**-1,085711028**	N8:526058	12,288	+8:YHR211W;	8			
chrVIII	526169	526320	151	**-1,060668784**	N8:526224	23,742	+9*:YHR211W;	9	TERM		
chrVIII	526293	526441	148	**-1,201515461**	N8:526381	27,465		Intergene			
chrVIII	526293	526481	188	**-1,254711863**	N8:526381	27,465		Intergene			
chrVIII	526600	526706	106	**-0,637645674**	N8:526683	30,347		Intergene			
chrVIII	526600	526743	143	**-1,295753797**	N8:526683	30,347		Intergene			
chrVIII	526600	526749	149	**-1,205808589**	N8:526683	30,347		Intergene			
chrVIII	526600	526751	151	**-1,153146903**	N8:526683	30,347		Intergene			
chrVIII	526624	526751	127	**-0,645420904**	N8:526683	30,347		Intergene			
chrVIII	526651	526751	100	**-0,127679689**	N8:526683	30,347		Intergene			
chrVIII	527864	528038	174	**-1,518700927**	N8:527985	32,393		Intergene			
chrVIII	536044	536157	113	**-1,96902132**	N8:536107	8,888		Intergene			
chrVIII	538480	538617	137	**-0,852417988**	N8:538580	7	0:YHR212W-A;	0			
chrVIII	539313	539458	145	**-0,98901669**	N8:539402	29,687	+3:YHR213W;	3			
chrVIII	539340	539488	148	**-1,055729843**	N8:539402	29,687	+3:YHR213W;	3			
chrVIII	542782	542926	144	**-1,023092029**	N8:542760	3,512		Intergene			
chrVIII	542782	542926	144	**-1,023092029**	N8:542922	7,136		Intergene			
chrVIII	542989	543156	167	**-1,354595086**				Overlap <50 bp			
chrVIII	543353	543457	104	**-1,112766739**	N8:543426	31,749		Intergene			
chrVIII	543780	543928	148	**-1,641852386**	N8:543820	4,726		Intergene			
chrVIII	544540	544654	114	**-1,584603356**				Overlap <50 bp			
chrVIII	546632	546789	157	**-1,36763172**	N8:546708	28		Intergene			
chrVIII	546818	546968	150	**-0,989375953**	N8:546888	18,52		Intergene			

CHR	START	END	L(bp)	ΔLknuc	Nuc ID	Fuzziness	Gene ID	Gene body postion or intergene	Terminal	rDNA	Telomeric
chrVIII	546822	546968	146	**-1,181618747**	N8:546888	18,52		Intergene			
chrVIII	547280	547415	135	**-0,769316725**	N8:547333	41,016		Intergene			
chrVIII	548318	548458	140	**-1,07056189**	N8:548393	32,97		Intergene			
chrVIII	548324	548458	134	**-0,897969286**	N8:548393	32,97		Intergene			
chrVIII	548850	549005	155	**-1,193138205**	N8:548872	26,23		Intergene			
chrVIII	548981	549089	108	**-0,90835103**	N8:549081	34,675		Intergene			
chrVIII	549893	550056	163	**-1,279316305**	N8:549980	23,798		Intergene			
chrVIII	550702	550810	108	**-0,911356535**	N8:550729	5,859	+3:YHR214C-D;	3			
chrVIII	551514	551634	120	**-1,113007121**	N8:551574	15,62	+1:YHR214C-E;	1			
chrVIII	553179	553326	147	**-1,016007985**	N8:553215	37,358	+8:YHR215W;	8			
chrVIII	553630	553778	148	**-1,332517702**	N8:553708	12,222	+11*:YHR215W;	11	TERM		
chrVIII	556401	556536	135	**-0,912232065**	N8:556476	25,401		Intergene			TEL RIGHT
chrVIII	556826	556854	28	**-0,448**				Overlap <50 bp			TEL RIGHT
chrVIII	556826	556859	33	**-0,483**				Overlap <50 bp			TEL RIGHT
chrVIII	556826	556865	39	**-0,525**				Overlap <50 bp			TEL RIGHT
chrVIII	556826	556871	45	**-0,567**				Overlap <50 bp			TEL RIGHT
chrVIII	556826	556873	47	**-0,581**				Overlap <50 bp			TEL RIGHT
chrVIII	556826	556876	50	**-0,602**				Overlap <50 bp			TEL RIGHT
chrVIII	556826	556884	58	**-0,658**	N8:556907	34,186		Intergene			TEL RIGHT
chrVIII	556826	556910	84	**-1,226387911**	N8:556907	34,186		Intergene			TEL RIGHT
chrVIII	556826	556914	88	**-0,669511342**	N8:556907	34,186		Intergene			TEL RIGHT
chrVIII	556826	556918	92	**-1,179094785**	N8:556907	34,186		Intergene			TEL RIGHT
chrVIII	556826	556919	93	**-0,554900173**	N8:556907	34,186		Intergene			TEL RIGHT
chrVIII	556826	556921	95	**-0,802117372**	N8:556907	34,186		Intergene			TEL RIGHT
chrVIII	556826	556923	97	**-0,652536804**	N8:556907	34,186		Intergene			TEL RIGHT
chrVIII	556826	556926	100	**-0,631980636**	N8:556907	34,186		Intergene			TEL RIGHT
chrVIII	556826	556930	104	**-1,059931527**	N8:556907	34,186		Intergene			TEL RIGHT
chrVIII	556826	556935	109	**-0,496516074**	N8:556907	34,186		Intergene			TEL RIGHT
chrVIII	556826	556936	110	**-0,27061736**	N8:556907	34,186		Intergene			TEL RIGHT
chrVIII	556826	556939	113	**-0,685664606**	N8:556907	34,186		Intergene			TEL RIGHT
chrVIII	556826	556942	116	**-1,039251849**	N8:556907	34,186		Intergene			TEL RIGHT
chrVIII	556826	556944	118	**-1,280593646**	N8:556907	34,186		Intergene			TEL RIGHT
chrVIII	556826	556950	124	**-0,636932973**	N8:556907	34,186		Intergene			TEL RIGHT
chrVIII	556826	556954	128	**-1,560902706**	N8:556907	34,186		Intergene			TEL RIGHT
chrVIII	556826	556956	130	**-1,702452052**	N8:556907	34,186		Intergene			TEL RIGHT
chrVIII	556826	556961	135	**-1,352952049**	N8:556907	34,186		Intergene			TEL RIGHT
chrVIII	556826	556963	137	**-1,060107198**	N8:556907	34,186		Intergene			TEL RIGHT
chrVIII	556826	556965	139	**-1,440694378**	N8:556907	34,186		Intergene			TEL RIGHT
chrVIII	556826	556967	141	**-1,423598416**	N8:556907	34,186		Intergene			TEL RIGHT
chrVIII	556826	556969	143	**-1,208665072**	N8:556907	34,186		Intergene			TEL RIGHT
chrVIII	556826	556970	144	**-2,01641571**	N8:556907	34,186		Intergene			TEL RIGHT
chrVIII	556826	556971	145	**-1,735863071**	N8:556907	34,186		Intergene			TEL RIGHT
chrVIII	556826	556972	146	**-2,136743975**	N8:556907	34,186		Intergene			TEL RIGHT
chrVIII	556826	556973	147	**-1,559662379**	N8:556907	34,186		Intergene			TEL RIGHT
chrVIII	556826	556975	149	**-1,152335566**	N8:556907	34,186		Intergene			TEL RIGHT
chrVIII	556826	556976	150	**-2,405678773**	N8:556907	34,186		Intergene			TEL RIGHT
chrVIII	556826	556977	151	**-1,898001685**	N8:556907	34,186		Intergene			TEL RIGHT
chrVIII	556826	556979	153	**-1,604003431**	N8:556907	34,186		Intergene			TEL RIGHT
chrVIII	556826	556981	155	**-2,143811282**	N8:556907	34,186		Intergene			TEL RIGHT
chrVIII	556826	556982	156	**-1,483601099**	N8:556907	34,186		Intergene			TEL RIGHT
chrVIII	556826	556983	157	**-1,679147984**	N8:556907	34,186		Intergene			TEL RIGHT
chrVIII	556826	556984	158	**-1,26789513**	N8:556907	34,186		Intergene			TEL RIGHT
chrVIII	556826	556985	159	**-1,582692098**	N8:556907	34,186		Intergene			TEL RIGHT
chrVIII	556826	556986	160	**-1,724828859**	N8:556907	34,186		Intergene			TEL RIGHT
chrVIII	556826	556987	161	**-1,230308377**	N8:556907	34,186		Intergene			TEL RIGHT
chrVIII	556826	556989	163	**-0,954907273**	N8:556907	34,186		Intergene			TEL RIGHT
chrVIII	556833	556986	153	**-1,753257145**	N8:556907	34,186		Intergene			TEL RIGHT
chrVIII	556840	556986	146	**-0,968458255**	N8:556907	34,186		Intergene			TEL RIGHT
chrVIII	556843	556989	146	**-1,562075941**	N8:556907	34,186		Intergene			TEL RIGHT
chrVIII	556853	556986	133	**-1,142041105**	N8:556907	34,186		Intergene			TEL RIGHT
chrVIII	556853	556989	136	**-0,874601035**	N8:556907	34,186		Intergene			TEL RIGHT
chrVIII	556858	556975	117	**-0,408248201**	N8:556907	34,186		Intergene			TEL RIGHT
chrVIII	556858	556989	131	**-1,004330007**	N8:556907	34,186		Intergene			TEL RIGHT
chrVIII	556859	556963	104	**-0,067348136**	N8:556907	34,186		Intergene			TEL RIGHT
chrVIII	556859	556989	130	**-0,792983028**	N8:556907	34,186		Intergene			TEL RIGHT
chrVIII	556863	556986	123	**-1,266982049**	N8:556907	34,186		Intergene			TEL RIGHT
chrVIII	556869	556986	117	**-1,048771995**	N8:556907	34,186		Intergene			TEL RIGHT
chrVIII	556878	556973	95	**-1,171114788**	N8:556907	34,186		Intergene			TEL RIGHT
chrVIII	556878	556975	97	**-1,002943975**	N8:556907	34,186		Intergene			TEL RIGHT
chrVIII	556878	556983	105	**-0,952897343**	N8:556907	34,186		Intergene			TEL RIGHT
chrVIII	556878	556989	111	**-0,356202184**	N8:556907	34,186		Intergene			TEL RIGHT
chrVIII	556883	556989	106	**-0,275450427**	N8:556907	34,186		Intergene			TEL RIGHT
chrVIII	556887	556986	99	**-0,587029762**	N8:556907	34,186		Intergene			TEL RIGHT
chrVIII	556893	556975	82	**-0,339628128**	N8:556907	34,186		Intergene			TEL RIGHT
chrVIII	556893	556989	96	**-0,212940564**	N8:556907	34,186		Intergene			TEL RIGHT
chrVIII	556901	556986	85	**-1,15658262**	N8:556907	34,186		Intergene			TEL RIGHT
chrVIII	556904	556989	85	**-0,177766469**	N8:556907	34,186		Intergene			TEL RIGHT
chrVIII	558701	558838	137	**-1,012345963**	N8:558769	8,185	+5:YHR218W;	5			TEL RIGHT
chrVIII	558986	559129	143	**-1,062129617**	N8:559040	9,165	+7:YHR218W;	7			TEL RIGHT
chrVIII	559259	559411	152	**-1,07763092**	N8:559370	13,317	+9:YHR218W;	9			TEL RIGHT
chrVIII	559332	559476	144	**-0,863222414**	N8:559370	13,317	+9:YHR218W;	9			TEL RIGHT
chrVIII	560157	560308	151	**-1,176163456**				Overlap <50 bp			TEL RIGHT
chrVIII	560416	560563	147	**-1,357717533**	N8:560523	23,245	+4:YHR219W;	4			TEL RIGHT
chrVIII	560448	560563	115	**-0,530914926**	N8:560523	23,245	+4:YHR219W;	4			TEL RIGHT
chrVIII	560502	560671	169	**-2,016890925**	N8:560523	23,245	+4:YHR219W;	4			TEL RIGHT
chrVIII	560544	560634	90	**-0,495364542**	N8:560523	23,245	+4:YHR219W;	4			TEL RIGHT
chrVIII	560544	560639	95	**-0,554475013**	N8:560523	23,245	+4:YHR219W;	4			TEL RIGHT
chrVIII	560784	560911	127	**-0,890469386**	N8:560813	40,104	+6:YHR219W;	6			TEL RIGHT
chrVIII	561208	561356	148	**-1,395654132**				Overlap <50 bp			TEL RIGHT
chrVIII	561302	561476	174	**-1,545644246**	N8:561386	33,234	+9:YHR219W;	9			TEL RIGHT
chrVIII	561649	561800	151	**-1,150978012**	N8:561716	37,643	+11:YHR219W;	11			TEL RIGHT
chrVIII	562455	562594	139	**0,024984111**	N8:562514	24,98		Intergene			TEL RIGHT
chrVIII	562455	562640	185	**-0,616816244**	N8:562514	24,98		Intergene			TEL RIGHT
chrVIII	562469	562594	125	**-0,482565486**	N8:562514	24,98		Intergene			TEL RIGHT
chrVIII	562469	562614	145	**-1,401378939**	N8:562514	24,98		Intergene			TEL RIGHT
chrVIII	562469	562632	163	**-1,305930156**	N8:562514	24,98		Intergene			TEL RIGHT

CHR	START	END	L(bp)	ΔLknuc	Nuc ID	Fuzziness	Gene ID	Gene body postion or intergene	Terminal	rDNA	Telomeric
chrVIII	562469	562636	167	**-1,717037227**	N8:562514	24,98		Intergene			TEL RIGHT
chrVIII	562469	562640	171	**-1,193022156**	N8:562514	24,98		Intergene			TEL RIGHT
chrVIII	562500	562636	136	**-0,767455159**	N8:562514	24,98		Intergene			TEL RIGHT
chrVIII	562506	562632	126	**-0,636794581**	N8:562514	24,98		Intergene			TEL RIGHT
chrVIII	562506	562636	130	**-1,324128897**	N8:562514	24,98		Intergene			TEL RIGHT
chrVIII	562506	562640	134	**-0,456973707**	N8:562514	24,98		Intergene			TEL RIGHT
chrVIII	562520	562621	101	**0,370846173**	N8:562514	24,98		Intergene			TEL RIGHT
chrVIII	562520	562640	120	**-0,974758759**	N8:562514	24,98		Intergene			TEL RIGHT
chrVIII	562523	562640	117	**0,771860303**	N8:562514	24,98		Intergene			TEL RIGHT
chrVIII	562552	562640	88	**-0,426356096**				Overlap <50 bp			TEL RIGHT
chrX	1669	1785	116	**-0,087124876**	N10:1770	35,355	+29:YJL225C;	29			TEL LEFT
chrX	1669	1804	135	**-0,835787938**	N10:1770	35,355	+29:YJL225C;	29			TEL LEFT
chrX	1669	1821	152	**-1,389767415**	N10:1770	35,355	+29:YJL225C;	29			TEL LEFT
chrX	1693	1785	92	**-0,516507022**	N10:1770	35,355	+29:YJL225C;	29			TEL LEFT
chrX	1693	1794	101	**-0,849956774**	N10:1770	35,355	+29:YJL225C;	29			TEL LEFT
chrX	1693	1804	111	**-0,180335421**	N10:1770	35,355	+29:YJL225C;	29			TEL LEFT
chrX	1693	1821	128	**-0,786742979**	N10:1770	35,355	+29:YJL225C;	29			TEL LEFT
chrX	1698	1821	123	**-0,662951956**	N10:1770	35,355	+29:YJL225C;	29			TEL LEFT
chrX	1980	2120	140	**-1,100483265**	N10:2021	28,284	+27:YJL225C;	27			TEL LEFT
chrX	2198	2335	137	**-1,10664366**	N10:2294	49,87	+25:YJL225C;	25			TEL LEFT
chrX	2365	2509	144	**-1,206861675**	N10:2503	42,123	+24:YJL225C;	24			TEL LEFT
chrX	2365	2512	147	**-1,310235743**	N10:2503	42,123	+24:YJL225C;	24			TEL LEFT
chrX	2365	2513	148	**-1,149851639**	N10:2503	42,123	+24:YJL225C;	24			TEL LEFT
chrX	2365	2575	210	**-1,448835178**	N10:2503	42,123	+24:YJL225C;	24			TEL LEFT
chrX	2370	2513	143	**-1,327709072**	N10:2503	42,123	+24:YJL225C;	24			TEL LEFT
chrX	2380	2513	133	**-1,171343452**	N10:2503	42,123	+24:YJL225C;	24			TEL LEFT
chrX	2420	2513	93	**-0,628850472**	N10:2503	42,123	+24:YJL225C;	24			TEL LEFT
chrX	2420	2575	155	**-1,332863382**	N10:2503	42,123	+24:YJL225C;	24			TEL LEFT
chrX	2862	3015	153	**-1,228937881**	N10:2906	19,858	+22:YJL225C;	22			TEL LEFT
chrX	3307	3451	144	**-1,13439162**	N10:3406	9,609	+19:YJL225C;	19			TEL LEFT
chrX	3518	3609	91	**-1,230099332**	N10:3573	3,512	+18:YJL225C;	18			TEL LEFT
chrX	3518	3627	109	**-0,828325748**	N10:3573	3,512	+18:YJL225C;	18			TEL LEFT
chrX	3518	3628	110	**-0,87589654**	N10:3573	3,512	+18:YJL225C;	18			TEL LEFT
chrX	3518	3629	111	**-1,105302234**	N10:3573	3,512	+18:YJL225C;	18			TEL LEFT
chrX	3518	3644	126	**-2,457596047**	N10:3573	3,512	+18:YJL225C;	18			TEL LEFT
chrX	3520	3627	107	**-1,415407206**	N10:3573	3,512	+18:YJL225C;	18			TEL LEFT
chrX	3520	3644	124	**-1,719221196**	N10:3573	3,512	+18:YJL225C;	18			TEL LEFT
chrX	4071	4226	155	**-1,910277336**	N10:4185	5,508	+14:YJL225C;	14			TEL LEFT
chrX	4165	4327	162	**-1,450790461**	N10:4343	6,429	+13:YJL225C;	13			TEL LEFT
chrX	4165	4327	162	**-1,450790461**	N10:4185	5,508	+14:YJL225C;	14			TEL LEFT
chrX	4788	4951	163	**-1,443979978**	N10:4836	12,055	+10:YJL225C;	10			TEL LEFT
chrX	5199	5350	151	**-1,122297434**				Overlap <50 bp			TEL LEFT
chrX	5412	5571	159	**-1,321430513**	N10:5402	32,563	+6:YJL225C;	6			TEL LEFT
chrX	9273	9424	151	**-1,399793187**	N10:9358	3,606	-1:YJL223C;	-1			
chrX	10211	10354	143	**-1,10018093**	N10:10296	5,132		Intergene			
chrX	11443	11594	151	**-1,251838268**	N10:11535	2	+2:YJL222W;	2			
chrX	12328	12473	145	**-1,051660823**	N10:12354	30,139	+7:YJL222W;	7			
chrX	12893	13042	149	**-1,497918187**				Overlap <50 bp			
chrX	14191	14340	149	**-0,926196058**	N10:14274	40,447	+20:YJL222W;	20			
chrX	16003	16132	129	**-0,737658224**	N10:16047	20,091	+31:YJL222W;	31			
chrX	16061	16168	107	**-1,534038136**	N10:16047	20,091	+31:YJL222W;	31			
chrX	21416	21535	119	**-1,460940079**	N10:21469	5,167		Intergene			
chrX	28854	29005	151	**-1,358456578**	N10:28867	7,861		Intergene			
chrX	28854	29005	151	**-1,358456578**	N10:29020	40,305		Intergene			
chrX	30398	30549	151	**-1,336830824**	N10:30469	24,095	+5:Unit347;	5			
chrX	31626	31734	108	**-0,836092408**	N10:31614	28,374	-1:YJL213W;	-1			
chrX	32694	32839	145	**-1,290824729**	N10:32766	15,684	+6:YJL213W;	6			
chrX	33933	34077	144	**-1,255625659**	N10:33990	7,294	+15:YJL212C;	15			
chrX	37483	37615	132	**-1,400493138**	N10:37503	40,631	+5*:YJL210W;	5	TERM		
chrX	38720	38876	156	**-1,25364164**	N10:38782	8,167	+6:YJL209W;	6			
chrX	40633	40803	170	**-1,706630918**	N10:40707	8,573	+4:YJL208C;	4			
chrX	40783	40953	170	**-1,537780227**	N10:40870	5,138	+3:YJL208C;	3			
chrX	46039	46190	151	**-1,280367817**	N10:46072	47,91		Intergene			
chrX	53460	53598	138	**-1,230023216**	N10:53518	4,472	+2:YJL203W;	2			
chrX	55658	55819	161	**-1,298669196**	N10:55711	12,723	+9:YJL201W;	9			
chrX	59858	59998	140	**-1,190265656**	N10:59955	7,668		Intergene			
chrX	64405	64545	140	**-1,285034458**				Overlap <50 bp			
chrX	86256	86397	141	**-1,173704167**	N10:86335	2,875	+5:YJL181W;	5			
chrX	88120	88277	157	**-1,3813057**	N10:88190	2,401	+3:YJL180C;	3			
chrX	90912	91092	180	**-1,719804921**	N10:91046	29,945	+2*:YJL177W;	2	TERM		
chrX	94290	94441	151	**-0,717480091**	N10:94365	4,98	+2:YJL176C;	2			
chrX	94366	94450	84	**-0,781400672**	N10:94365	4,98	+2:YJL176C;	2			
chrX	94724	94862	138	**-1,352699576**	N10:94769	7,148	-1:YJL174W; -1:YJL176C;	-1			
chrX	100058	100217	159	**-1,229272908**	N10:100203	6,058	+5:YJL171C;	5			
chrX	100062	100217	155	**-1,586128037**	N10:100203	6,058	+5:YJL171C;	5			
chrX	100066	100217	151	**-1,422028956**	N10:100203	6,058	+5:YJL171C;	5			
chrX	105547	105698	151	**-1,11964408**	N10:105607	28,499	+5:YJL167W;	5			
chrX	106971	107136	165	**-1,288219538**				Overlap <50 bp			
chrX	108096	108187	91	**-1,155196189**	N10:108130	32,496	+10:YJL165C;	10			
chrX	108096	108204	108	**-0,954118304**	N10:108130	32,496	+10:YJL165C;	10			
chrX	108096	108205	109	**-0,865699674**	N10:108130	32,496	+10:YJL165C;	10			
chrX	108096	108206	110	**-0,944887381**	N10:108130	32,496	+10:YJL165C;	10			
chrX	108096	108207	111	**-1,184729181**	N10:108130	32,496	+10:YJL165C;	10			
chrX	108096	108208	112	**-1,153019389**	N10:108130	32,496	+10:YJL165C;	10			
chrX	108096	108220	124	**-2,626057229**	N10:108130	32,496	+10:YJL165C;	10			
chrX	108097	108205	108	**-0,879269643**	N10:108130	32,496	+10:YJL165C;	10			
chrX	108098	108200	102	**-1,359329665**	N10:108130	32,496	+10:YJL165C;	10			
chrX	108098	108205	107	**-1,443181936**	N10:108130	32,496	+10:YJL165C;	10			
chrX	108098	108206	108	**-1,36077316**	N10:108130	32,496	+10:YJL165C;	10			
chrX	108098	108220	122	**-1,559361863**	N10:108130	32,496	+10:YJL165C;	10			
chrX	108135	108220	85	**-1,511526842**	N10:108130	32,496	+10:YJL165C;	10			
chrX	117567	117726	159	**-1,242489198**				Overlap <50 bp			
chrX	118042	118180	138	**-1,067853198**	N10:118106	19,442	+1:CUT199; +6:YJL160C;	1			
chrX	118045	118180	135	**-0,941288422**	N10:118106	19,442	+1:CUT199; +6:YJL160C;	1			
chrX	118063	118213	150	**-1,195724196**	N10:118106	19,442	+1:CUT199; +6:YJL160C;	1			
chrX	118208	118364	156	**-1,523681402**	N10:118386	11,769	+3:CUT199; +4:YJL160C;	3			

CHR	START	END	L(bp)	ΔLknuc	Nuc ID	Fuzziness	Gene ID	Gene body postion or intergene	Terminal	rDNA	Telomeric
chrX	122371	122478	107	**-1,299979864**	N10:122443	42,087	+3:YJL158C;	3			
chrX	126969	127111	142	**-1,31984166**	N10:126996	12,661	+13:YJL156C;	13			
chrX	127866	128026	160	**-1,248126691**	N10:127919	32,764	+7:YJL156C;	7			
chrX	131244	131407	163	**-1,525964617**	N10:131345	42,669	+17:YJL154C;	17			
chrX	139844	139996	152	**-1,512267132**	N10:139830	39,009		Intergene			
chrX	141168	141318	150	**-1,296773228**	N10:141239	26,038	+6*:YJL148W; +9:YJL147C;	6	TERM		
chrX	142696	142846	150	**-1,293556673**	N10:142702	38,514	-1:YJL147C;	-1			
chrX	145224	145374	150	**-1,477802941**	N10:145222	49,666	+2:YJL145W;	2			
chrX	145224	145374	150	**-1,477802941**	N10:145384	34,029	+3:YJL145W;	3			
chrX	148124	148271	147	**-1,174888629**	N10:148257	26,077	+14:YJL141C;	14			
chrX	149558	149726	168	**-1,617893786**	N10:149585	44,825	+6:YJL141C;	6			
chrX	154966	155110	144	**-1,539786272**	N10:155017	51,915	+7:YJL137C; +1:CUT204;	7			
chrX	162602	162770	168	**-1,47941696**	N10:162588	28,464		Intergene			
chrX	163009	163162	153	**-1,366731271**	N10:163000	32,042		Intergene			
chrX	169091	169251	160	**-1,56410159**	N10:169161	33,306	+19:YJL130C;	19			
chrX	173679	173830	151	**-1,562499413**	N10:173775	23,452		Intergene			
chrX	173874	174016	142	**-1,272576999**	N10:173949	3,055		Intergene			
chrX	178426	178580	154	**-1,244160335**	N10:178551	28,717	+12:YJL128C;	12			
chrX	182961	183077	116	**-0,933371074**	N10:183051	43,298	+5:CUT206; +8:YJL127C;	5			
chrX	182964	183077	113	**-1,083078441**	N10:183051	43,298	+5:CUT206; +8:YJL127C;	5			
chrX	182966	183077	111	**-0,692674699**	N10:183051	43,298	+5:CUT206; +8:YJL127C;	5			
chrX	185927	186084	157	**-1,283875464**	N10:186100	34,903	+4:YJL125C;	4			
chrX	185927	186084	157	**-1,283875464**	N10:185938	24,838	+5:YJL125C;	5			
chrX	189658	189821	163	**-1,154444421**	N10:189737	28,077	+1:YJL122W;	1			
chrX	190658	190805	147	**-1,145166371**	N10:190730	48,552	+3:YJL121C;	3			
chrX	191225	191359	134	**-1,196156856**	N10:191216	36,482	-1:YJL121C;	-1			
chrX	192006	192112	106	**-0,941621829**				Overlap <50 bp			
chrX	192006	192128	122	**-1,282933223**				Overlap <50 bp			
chrX	192132	192293	161	**-1,264457106**	N10:192220	1	-1:YJL117W; +5*:YJL118W;	-1			
chrX	192599	192736	137	**-1,834692411**	N10:192656	36,725	+2:YJL117W;	2			
chrX	194181	194318	137	**-1,139031062**	N10:194236	34,21	+4:YJL116C; +3:SUT206;	4			
chrX	197629	197777	148	**-0,956472212**	N10:197770	6,364		Intergene			
chrX	198154	198316	162	**-1,695549094**	N10:198291	45,177		Intergene			
chrX	198182	198330	148	**-1,258065444**	N10:198291	45,177		Intergene			
chrX	199707	199858	151	**-1,592069346**	N10:199867	44,529		Intergene			
chrX	203379	203541	162	**-1,480224784**	N10:203536	13,435		Intergene			
chrX	203482	203630	148	**-1,160378042**	N10:203536	13,435		Intergene			
chrX	206401	206560	159	**-1,675180542**	N10:206482	30,659	+9:YJL112W;	9			
chrX	209002	209147	145	**-1,29703576**	N10:209025	33,537	+9:YJL111W;	9			
chrX	210689	210857	168	**-1,493451383**	N10:210876	33,935	+6:YJL110C;	6			
chrX	210689	210857	168	**-1,493451383**	N10:210724	33,777	+7:YJL110C;	7			
chrX	212042	212183	141	**-1,147084734**	N10:212201	36,483	+30:YJL109C;	30			
chrX	212042	212183	141	**-1,147084734**	N10:212021	34,303	+31:YJL109C;	31			
chrX	215649	215789	140	**-1,22446354**	N10:215728	45,002	+10:YJL109C;	10			
chrX	218927	219076	149	**-1,608594929**				Overlap <50 bp			
chrX	219865	220016	151	**-1,199576177**	N10:220031	40,698	0:YJL107C; +4*:Unit351;	0			
chrX	219865	220016	151	**-1,199576177**	N10:219873	26,588	+1:YJL107C;	1			
chrX	220580	220715	135	**-1,585892948**	N10:220634	37,474	+2:Unit372; 0:Unit351;	2			
chrX	221358	221508	150	**-1,309063329**	N10:221437	47,182	+2:YJL106W;	2			
chrX	225766	225921	155	**-1,276208315**	N10:225854	32,578	+6:YJL105W;	6			
chrX	226052	226204	152	**-1,488928758**	N10:226103	51,122	+8:YJL105W;	8			
chrX	226472	226633	161	**-1,294524921**	N10:226484	33,16	+10:YJL105W; +4:SUT628;	10			
chrX	227406	227571	165	**-1,410733447**	N10:227419	41,205	+2:YJL104W;	2			
chrX	227406	227571	165	**-1,410733447**	N10:227588	34,326	+3:YJL104W;	3			
chrX	229005	229137	132	**-1,328275249**	N10:229146	23,742	+11*:YJL103C;	11	TERM		
chrX	229384	229535	151	**-1,419721107**	N10:229378	30,578	+10:YJL103C;	10			
chrX	233516	233665	149	**-1,583696633**				Overlap <50 bp			
chrX	235494	235644	150	**-1,417236663**	N10:235627	35,36	+6:YJL101C;	6			
chrX	237835	237975	140	**-1,147203109**	N10:237857	8,185	+8:YJL100W;	8			
chrX	240265	240414	149	**-1,096552212**	N10:240350	34,189		Intergene			
chrX	240554	240707	153	**-1,315187587**	N10:240699	27,785		Intergene			
chrX	240601	240747	146	**-1,300899591**	N10:240699	27,785		Intergene			
chrX	242702	242868	166	**-1,578505634**				Overlap <50 bp			
chrX	243519	243619	100	**-0,666567693**	N10:243533	34,634	+12:YJL098W;	12			
chrX	245287	245435	148	**-1,358801988**	N10:245271	29,24	-1:YJL097W;	-1			
chrX	247930	248081	151	**-1,567695434**	N10:247910	35,901	+8:YJL095W;	8			
chrX	248270	248435	165	**-1,467112674**	N10:248400	28,162	+11:YJL095W;	11			
chrX	251069	251224	155	**-1,372474**	N10:251097	33,323	+28:YJL095W;	28			
chrX	251916	252080	164	**-1,379793165**	N10:251970	34,616	+15:YJL094C;	15			
chrX	253721	253885	164	**-1,426559303**	N10:253709	45,908	+4:YJL094C;	4			
chrX	254829	255000	171	**-1,46592918**	N10:254961	5,568	+11:YJL093C;	11			
chrX	255572	255735	163	**-1,690054238**	N10:255685	33,927	+6:YJL093C;	6			
chrX	271510	271677	167	**-1,598903556**	N10:271629	42,484	+6:YJL087C;	6			
chrX	271664	271795	131	**-0,798483465**				Overlap <50 bp			
chrX	272130	272276	146	**-1,274905833**	N10:272201	34,004	+2:YJL087C;	2			
chrX	273881	274032	151	**-1,317405679**	N10:273955	31,329	+9:YJL085W;	9			
chrX	275971	276080	109	**-1,372479318**	N10:276085	33,62	+13:YJL084C;	13			
chrX	276361	276455	94	**-0,404812783**				Overlap <50 bp			
chrX	276361	276463	102	**-0,46933392**				Overlap <50 bp			
chrX	276481	276614	133	**-1,521107296**	N10:276566	35,204	+10:YJL084C;	10			
chrX	278918	279067	149	**-1,408743397**	N10:278918	2,517	+2:YJL083W;	2			
chrX	283812	283977	165	**-1,233456467**	N10:283889	37,704	+9:YJL081C;	9			
chrX	287746	287904	158	**-1,080879283**	N10:287749	6,557	+10:YJL080C;	10			
chrX	292191	292322	131	**-0,712240112**	N10:292221	37,136	+12:YJL078C;	12			
chrX	292971	293126	155	**-1,064407892**				Overlap <50 bp			
chrX	293774	293933	159	**-1,121600903**	N10:293870	26,577	+1:YJL078C; +1:YJL077W-B;	1			
chrX	301109	301275	166	**-1,28946474**	N10:301192	25,305	+10:YJL074C;	10			
chrX	303727	303887	160	**-1,273586686**	N10:303776	36,009	+6*:YJL073W;	6	TERM		
chrX	309182	309321	139	**-1,391568691**	N10:309163	36,678	+8:YJL070C;	8			
chrX	310987	311146	159	**-1,233853829**	N10:310995	48,895		Intergene			
chrX	310987	311146	159	**-1,233853829**	N10:311155	12,014		Intergene			
chrX	321769	321919	150	**-1,417862454**	N10:321795	49,992	+12:YJL061W;	12			
chrX	322337	322492	155	**-1,461691398**	N10:322482	48,304		Intergene			
chrX	325234	325383	149	**-1,123861123**	N10:325322	46,647	+4:YJL059W;	4			
chrX	326985	327142	157	**-1,308513442**	N10:327050	19,772	+5:YJL058C;	5			
chrX	327633	327785	152	**-1,405140593**	N10:327706	35,566	+1:YJL058C;	1			

CHR	START	END	L(bp)	ΔLknuc	Nuc ID	Fuzziness	Gene ID	Gene body postion or intergene	Terminal	rDNA	Telomeric
chrX	328569	328730	161	**-1,51163706**				Overlap <50 bp			
chrX	329259	329407	148	**-1,596777234**	N10:329251	33,381	+6*:YJL057C;	6	TERM		
chrX	329259	329407	148	**-1,596777234**	N10:329425	36,325	+5:YJL057C;	5			
chrX	331899	332052	153	**-1,482096683**				Overlap <50 bp			
chrX	333305	333466	161	**-1,410095529**	N10:333312	35,767	+1:YJL055W; -1:YJL056C;	1			
chrX	333305	333466	161	**-1,410095529**	N10:333478	3,215	+2:YJL055W;	2			
chrX	333361	333513	152	**-1,032268301**	N10:333478	3,215	+2:YJL055W;	2			
chrX	337451	337612	161	**-1,154894181**				Overlap <50 bp			
chrX	338776	338907	131	**-1,252496263**	N10:338772	2,828	+5:YJL052W;	5			
chrX	343385	343533	148	**-1,203966575**	N10:343380	8,327	+7:YJL050W;	7			
chrX	344740	344900	160	**-1,477610994**				Overlap <50 bp			
chrX	350347	350517	170	**-1,488234514**	N10:350502	31,67	+11:YJL047C;	11			
chrX	350347	350517	170	**-1,488234514**	N10:350327	49,044	+12:YJL047C;	12			
chrX	357774	357934	160	**-1,424926248**	N10:357775	40,351	+13:YJL045W;	13			
chrX	358769	358924	155	**-1,323058654**	N10:358940	39,357	+5:YJL044C;	5			
chrX	359603	359757	154	**-1,163835496**	N10:359674	3,786	0:YJL044C; -1:YJL043W;	0			
chrX	362162	362320	158	**-1,344427311**	N10:362260	26,5	+8:YJL042W;	8			
chrX	365711	365851	140	**-1,385159**	N10:365757	3,215		Intergene			
chrX	366070	366219	149	**-1,325857401**	N10:366182	14,107		Intergene			
chrX	366353	366502	149	**-1,017253777**	N10:366336	30,237		Intergene			
chrX	366353	366502	149	**-1,017253777**	N10:366486	48,083		Intergene			
chrX	366520	366671	151	**-1,05145062**	N10:366641	32,083		Intergene			
chrX	367696	367845	149	**-1,321155084**	N10:367708	34,691		Intergene			
chrX	367696	367845	149	**-1,321155084**	N10:367868	47,107		Intergene			
chrX	369411	369539	128	**-1,198777534**	N10:369398	27,3	+28:YJL039C;	28			
chrX	369776	369927	151	**-1,202669624**	N10:369846	34,392	+26:YJL039C;	26			
chrX	374096	374246	150	**-1,685702638**	N10:374118	34,861	+2*:Unit353; -1:YJL039C;	2	TERM		
chrX	380580	380750	170	**-0,95644113**	N10:380752	32,65	+2:YJL035C;	2			
chrX	380580	380750	170	**-0,95644113**	N10:380571	6,11	+3:YJL035C;	3			
chrX	380923	381086	163	**-1,175209665**	N10:380993	31,549	-1:YJL034W; 0:YJL035C;	-1			
chrX	385357	385485	128	**-1,261501152**				Overlap <50 bp			
chrX	386997	387161	164	**-1,586430376**	N10:387073	39,461	+3:YJL031C;	3			
chrX	392484	392621	137	**-1,276479645**	N10:392516	34,593	+2:YJL026W; -1:YJL027C;	2			
chrX	392991	393143	152	**-1,143021383**	N10:393070	46,144	+5:YJL026W;	5			
chrX	394180	394326	146	**-1,249649075**	N10:394214	24,228	+3:YJL025W;	3			
chrX	394276	394372	96	**-1,071782309**	N10:394374	36,682	+4:YJL025W;	4			
chrX	398245	398394	149	**-1,389163967**	N10:398328	34,519	-1:Unit375; +1:YJL023C;	-1			
chrX	399541	399701	160	**-1,64390185**	N10:399582	51,041	+17:YJL020C;	17			
chrX	407672	407824	152	**-1,218462318**	N10:407809	32,908	+3:YJL014W;	3			
chrX	410284	410430	146	**-1,243962475**	N10:410379	44,132	+4:YJL013C;	4			
chrX	410723	410889	166	**-1,477927134**	N10:410731	56,691	+2:YJL013C;	2			
chrX	412052	412213	161	**-1,263714498**				Overlap <50 bp			
chrX	425378	425524	146	**-1,176392357**				Overlap <50 bp			
chrX	429176	429332	156	**-1,149147595**	N10:429178	47,831	+26:YJL005W;	26			
chrX	429194	429332	138	**-1,251419524**	N10:429178	47,831	+26:YJL005W;	26			
chrX	429194	429339	145	**-1,240375697**	N10:429178	47,831	+26:YJL005W;	26			
chrX	435614	435721	107	**-1,102078135**	N10:435670	37,917		Intergene			
chrX	435614	435731	117	**-1,103064954**	N10:435670	37,917		Intergene			
chrX	439795	439950	155	**-1,219028458**	N10:439773	35,566	+6:YJR002W;	6			
chrX	447409	447538	129	**-1,060616923**				Overlap <50 bp			
chrX	448364	448517	153	**-1,175115514**	N10:448344	23,242	+4:YJR005C-A;	4			
chrX	456180	456328	148	**-1,559992869**	N10:456302	36,148	+3:YJR010W;	3			
chrX	459326	459480	154	**-0,996928954**	N10:459411	29,08	0:YJR011C; 0:SUT214;	0			
chrX	465469	465620	151	**-1,279018597**	N10:465481	49,761	+4:YJR016C;	4			
chrX	466504	466659	155	**-1,050892906**	N10:466505	18,751	+4:YJR017C;	4			
chrX	467197	467335	138	**-1,099641469**	N10:467195	36,606	+7*:YJR019C; -1:YJR017C;	7	TERM		
chrX	467559	467705	146	**-2,040973872**	N10:467683	46,753	+4:YJR019C;	4			
chrX	470836	470984	148	**-1,224507849**	N10:470847	7	+1:YJR024C;	1			
chrX	472163	472315	152	**-1,352345881**	N10:472226	51,389		Intergene			
chrX	472826	472975	149	**-1,120039169**	N10:472972	43,981		Intergene			
chrX	473000	473123	123	**-1,045392998**	N10:473146	67,089		Intergene			
chrX	473100	473255	155	**-1,325222881**	N10:473146	67,089		Intergene			
chrX	473533	473667	134	**-1,037384491**	N10:473586	11,314		Intergene			
chrX	473533	473673	140	**-1,087593171**	N10:473586	11,314		Intergene			
chrX	473729	473880	151	**-1,197159524**	N10:473881	1,414		Intergene			
chrX	473739	473884	145	**-1,362180335**	N10:473881	1,414		Intergene			
chrX	473757	473908	151	**-1,64397204**	N10:473881	1,414		Intergene			
chrX	474011	474165	154	**-1,315507124**	N10:474051	12,728		Intergene			
chrX	474045	474189	144	**-1,179614863**	N10:474051	12,728		Intergene			
chrX	474045	474189	144	**-1,179614863**	N10:474202	38,891		Intergene			
chrX	474576	474711	135	**-1,292907621**				Overlap <50 bp			
chrX	475023	475169	146	**-1,110101161**				Overlap <50 bp			
chrX	475023	475173	150	**-1,060358114**				Overlap <50 bp			
chrX	475693	475822	129	**-1,006382044**	N10:475768	41,356		Intergene			
chrX	477337	477451	114	**-1,167420193**				Overlap <50 bp			
chrX	477657	477804	147	**-1,43726861**	N10:477753	33,941		Intergene			
chrX	478238	478390	152	**-1,41848238**	N10:478237	52,918		Intergene			
chrX	478410	478559	149	**-1,299871786**	N10:478482	41,885		Intergene			
chrX	478560	478666	106	**-0,923119576**				Overlap <50 bp			
chrX	478560	478678	118	**-0,96178181**				Overlap <50 bp			
chrX	478584	478707	123	**-0,961213981**	N10:478712	31,113		Intergene			
chrX	478684	478839	155	**-1,270297824**	N10:478712	31,113		Intergene			
chrX	479117	479251	134	**-0,997850123**				Overlap <50 bp			
chrX	479117	479257	140	**-1,209276341**	N10:479275	21,213		Intergene			
chrX	480160	480295	135	**-1,043351806**	N10:480180	39,247		Intergene			
chrX	480607	480757	150	**-1,037428655**	N10:480589	19,092		Intergene			
chrX	480786	480943	157	**-1,715180794**	N10:480816	49,541		Intergene			
chrX	481277	481406	129	**-1,014070226**	N10:481374	34,044		Intergene			
chrX	481757	481888	131	**-1,067587683**	N10:481781	7,071		Intergene			
chrX	482189	482339	150	**-1,483185188**	N10:482287	43,097		Intergene			
chrX	483140	483291	151	**-1,67233072**	N10:483274	17,616		Intergene			
chrX	495866	496005	139	**-1,145658399**	N10:495880	36,396	+4:YJR033C;	4			
chrX	496369	496517	148	**-1,19197368**	N10:496351	3,215	+1:YJR033C; -1:YJR034W;	1			
chrX	498437	498584	147	**-1,262264386**	N10:498554	27,129	+11:YJR035W;	11			
chrX	503072	503238	166	**-1,441143302**	N10:503242	34,881	+3:YJR036C;	3			
chrX	503072	503238	166	**-1,441143302**	N10:503073	34,49	+4:YJR036C;	4			

CHR	START	END	L(bp)	ΔLknuc	Nuc ID	Fuzziness	Gene ID	Gene body postion or intergene	Terminal	rDNA	Telomeric
chrX	503922	504069	147	-1,293096882	N10:504008	42,922	+2:YJR039W;	2			
chrX	512701	512853	152	-0,889908597	N10:512703	34,322	+6:YJR041C;	6			
chrX	516708	516897	189	-1,445909132	N10:516725	28,289	+6:YJR043C;	6			
chrX	519161	519304	143	-1,279845374	N10:519222	47,376	+1:YJR044C;	1			
chrX	519732	519838	106	-1,351632717	N10:519795	34,961	+12:YJR045C;	12			
chrX	519732	519858	126	-1,167615602	N10:519795	34,961	+12:YJR045C;	12			
chrX	523978	524108	130	-0,990012305	N10:523988	36,396		Intergene			
chrX	526346	526498	152	-0,881388541	N10:526454	29,134	+2:SUT641; +2:YJR048W;	2			
chrX	527259	527387	128	-1,393180911	N10:527394	37,757	+6:YJR049C;	6			
chrX	530647	530795	148	-1,141729479	N10:530763	18,771	+7:YJR051W;	7			
chrX	531614	531753	139	-1,077069317	N10:531763	9,539	-1:YJR052W;	-1			
chrX	533693	533845	152	-1,375582729	N10:533743	6,245	+2:YJR053W;	2			
chrX	537746	537899	153	-1,726252063	N10:537764	48,32		Intergene			
chrX	542575	542723	148	-2,394643628				Overlap <50 bp			
chrX	544311	544443	132	-1,258662035	N10:544344	10,708	+3:YJR057W;	3			
chrX	547133	547290	157	-1,473099822	N10:547288	17,671	+11:YJR059W;	11			
chrX	554602	554737	135	-1,267845257	N10:554644	7,371	+1:YJR062C;	1			
chrX	557805	557945	140	-1,172590938	N10:557963	22,716	+8:YJR065C;	8			
chrX	557805	557945	140	-1,172590938	N10:557804	31,858	+9:YJR065C;	9			
chrX	557934	558083	149	-0,835953679	N10:557963	22,716	+8:YJR065C;	8			
chrX	558445	558574	129	-0,96728835	N10:558562	35,584	+4:YJR065C;	4			
chrX	561483	561587	104	-1,583504592				Overlap <50 bp			
chrX	561700	561836	136	-1,176344522	N10:561697	40,28	+15:YJR066W;	15			
chrX	563670	563822	152	-1,335483118	N10:563737	34,256	+28:YJR066W;	28			
chrX	567134	567269	135	-1,085491083	N10:567226	34,364	+2:YJR067C; -1:YJR068W;	2			
chrX	569137	569291	154	-1,062542371	N10:569134	34,974	+2:YJR069C;	2			
chrX	572775	572903	128	-0,673208568				Overlap <50 bp			
chrX	573799	573959	160	-1,371202031	N10:573951	37,712	+1:YJR075W;	1			
chrX	582808	582958	150	-1,002744758				Overlap <50 bp			
chrX	586031	586180	149	-1,326640475	N10:586162	37,954	+2*:YJR086W;	2	TERM		
chrX	590874	591029	155	-1,379331855	N10:591040	38,188		Intergene			
chrX	591957	592113	156	-1,264491637	N10:592071	23,195		Intergene			
chrX	594961	595112	151	-1,191112781	N10:594945	37,249	+18:YJR091C;	18			
chrX	595496	595660	164	-0,869054055	N10:595504	47,178	+15:YJR091C;	15			
chrX	598239	598403	164	-1,292927307	N10:598323	36,065	0:YJR091C; -1:YJR092W;	0			
chrX	598789	598947	158	-1,369018598	N10:598769	34,496	+2:YJR092W;	2			
chrX	600553	600699	146	-0,958173686	N10:600632	32,685	+14:YJR092W;	14			
chrX	606604	606728	124	-1,215552723	N10:606660	14,107	+5:SUT643;	5			
chrX	608387	608544	157	-1,383765238	N10:608402	48,976	+2*:YJR094W-A;	2	TERM		
chrX	608709	608811	102	-1,016717165	N10:608690	45,318		Intergene			
chrX	610999	611149	150	-1,357078591	N10:610987	31,919	+1:YJR096W; +7*:SUT644;	1			
chrX	623512	623664	152	-1,564751633	N10:623582	26,986	+2:YJR105W;	2			
chrX	624211	624342	131	-1,214013879	N10:624345	23,796	+7:YJR105W;	7			
chrX	624949	625089	140	-1,141512165	N10:625080	32,388	+3:YJR106W;	3			
chrX	632590	632738	148	-1,271270927	N10:632669	44,076	+2:YJR109C;	2			
chrX	635827	635966	139	-1,165099263	N10:635846	26,87	+6*:YJR111C;	6	TERM		
chrX	636181	636337	156	-1,484780806	N10:636350	33,776	+3:YJR111C;	3			
chrX	636181	636337	156	-1,484780806	N10:636186	30,397	+4:YJR111C;	4			
chrX	636714	636864	150	-1,359844187	N10:636770	35,584	0:YJR111C; -1:YJR112W;	0			
chrX	637154	637305	151	-1,540868913	N10:637320	48,003	-1:YJR112W-A; +3:YJR112W;	-1			
chrX	637154	637305	151	-1,540868913	N10:637147	3,215	+2:YJR112W;	2			
chrX	638977	639126	149	-1,159844519	N10:638974	18,903	0:YJR113C;	0			
chrX	653069	653214	145	-1,178306589	N10:653126	33,955	+7:YJR124C;	7			
chrX	661255	661420	165	-1,399719386	N10:661267	27,789	+9:YJR127C;	9			
chrX	662029	662161	132	-0,915804221	N10:662110	43,279	+4:YJR127C;	4			
chrX	662034	662161	127	-0,884119286	N10:662110	43,279	+4:YJR127C;	4			
chrX	662037	662135	98	-0,578244156	N10:662110	43,279	+4:YJR127C;	4			
chrX	671104	671223	119	-1,227155771				Overlap <50 bp			
chrX	671901	672050	149	-1,585638614				Overlap <50 bp			
chrX	673361	673549	188	-1,608961129				Overlap <50 bp			
chrX	673385	673539	154	-1,304641944				Overlap <50 bp			
chrX	674527	674676	149	-1,202480408	N10:674605	35,449	+12:YJR135C; +9:YJR134C;	12			
chrX	684490	684642	152	-1,326344291	N10:684478	38,073	+1:YJR138W;	1			
chrX	684490	684642	152	-1,326344291	N10:684641	38,118	+2:YJR138W;	2			
chrX	685942	686069	127	-1,023331107	N10:686043	45,181	+11:YJR138W;	11			
chrX	687031	687188	157	-1,522408057	N10:687180	29,092	+18:YJR138W;	18			
chrX	687681	687842	161	-1,503074974	N10:687764	6,856	+22*:YJR138W;	22	TERM		
chrX	689548	689698	150	-1,139736761	N10:689678	19,883	+6:YJR139C;	6			
chrX	695637	695789	152	-1,200633399	N10:695637	7,55	+1:YJR140C; -1:YJR141W;	1			
chrX	696260	696391	131	-1,089926385	N10:696342	35,763	+4:YJR141W;	4			
chrX	697538	697692	154	-1,423417057	N10:697604	34,568	+4:YJR142W;	4			
chrX	699421	699528	107	-1,322540912	N10:699480	35,259	+8:YJR143C;	8			
chrX	699421	699559	138	-1,238454783	N10:699480	35,259	+8:YJR143C;	8			
chrX	699421	699560	139	-1,427267091	N10:699480	35,259	+8:YJR143C;	8			
chrX	699421	699561	140	-1,133150691	N10:699480	35,259	+8:YJR143C;	8			
chrX	699421	699563	142	-1,523311187	N10:699480	35,259	+8:YJR143C;	8			
chrX	699421	699564	143	-1,198565158	N10:699480	35,259	+8:YJR143C;	8			
chrX	700845	700991	146	-1,1199237	N10:700894	36,581	-1:YJR143C; +2:YJR144W;	-1			
chrX	701322	701470	148	-1,259819895	N10:701322	32,342	+5:YJR144W;	5			
chrX	705757	705926	169	-1,672219511	N10:705760	32,609	+2:YJR148W;	2			
chrX	705757	705926	169	-1,672219511	N10:705934	32,725	+3:YJR148W;	3			
chrX	706905	707066	161	-1,489130344	N10:707000	42,289	+2*:YJR149W;	2	TERM		
chrX	712931	713070	139	-1,229911679	N10:712913	47,376	+17:YJR151C;	17			
chrX	714988	715121	133	-0,98982772	N10:715105	7,572	+5:YJR151C;	5			
chrX	720830	720969	139	-0,888870258	N10:720969	46,456	+9:YJR152W;	9			
chrX	721017	721124	107	-1,577883772				Overlap <50 bp			
chrX	721017	721148	131	-1,134228498				Overlap <50 bp			
chrX	721017	721149	132	-1,315438328				Overlap <50 bp			
chrX	721017	721153	136	-1,177066163				Overlap <50 bp			
chrX	721018	721153	135	-0,923998916				Overlap <50 bp			
chrX	721019	721153	134	-1,379371996				Overlap <50 bp			
chrX	721020	721153	133	-1,416510415				Overlap <50 bp			
chrX	723086	723238	152	-1,424966951	N10:723143	40,454	+3:YJR153W; +7:anti102;	3			
chrX	723301	723431	130	-0,425733754	N10:723416	25,487	+5:YJR153W; +5:anti102;	5			
chrX	723442	723594	152	-1,106831378	N10:723587	8,505	+6:YJR153W; +4:anti102;	6			
chrX	725588	725741	153	-1,466226462	N10:725606	7,371	+5*:Unit382; +2:YJR154W;	5	TERM		

CHR	START	END	L(bp)	ΔLknuc	Nuc ID	Fuzziness	Gene ID	Gene body postion or intergene	Terminal	rDNA	Telomeric
chrX	729427	729584	157	**-1,151697126**	N10:729606	0,707	+1:YJR156C;	1			
chrX	729427	729584	157	**-1,151697126**	N10:729424	8,485	+2:YJR156C;	2			
chrX	729799	729953	154	**-1,19958189**				Overlap <50 bp			
chrX	732737	732877	140	**-1,265381153**	N10:732736	20,664	+4:YJR158W;	4			
chrX	735177	735331	154	**-1,755704053**	N10:735172	9,192		Intergene			
chrX	735177	735331	154	**-1,755704053**	N10:735346	0,707		Intergene			
chrX	736882	737013	131	**-1,39783147**	N10:736967	3,536	+7:YJR159W;	7			
chrX	739148	739242	94	**-1,286313551**	N10:739259	35,355	+4:YJR160C;	4			
chrX	745644	745739	95	**-1,048020398**				Overlap <50 bp			TEL RIGHT
chrX	745644	745742	98	**-0,55235635**				Overlap <50 bp			TEL RIGHT
chrXI	6938	7102	164	**-1,277801213**	N11:6990	9,888	+6:YKL221W; +5:SUT652;	6			
chrXI	10271	10420	149	**-1,087930626**	N11:10428	25,377	+7:YKL220C;	7			
chrXI	17984	18132	148	**-1,143517674**	N11:18117	31,079	+3:YKL218C;	3			
chrXI	19266	19389	123	**-1,087703789**	N11:19336	3,724	+4:Unit402;	4			
chrXI	19267	19389	122	**-1,077864938**	N11:19336	3,724	+4:Unit402;	4			
chrXI	19267	19414	147	**-1,446101711**	N11:19336	3,724	+4:Unit402;	4			
chrXI	20703	20861	158	**-1,425894316**	N11:20761	35,26	+3:SUT656;	3			
chrXI	31510	31641	131	**-1,245794249**				Overlap <50 bp			
chrXI	32617	32778	161	**-1,502199352**	N11:32717	20,549	+10:YKL213C;	10			
chrXI	38219	38379	160	**-1,032578271**				Overlap <50 bp			
chrXI	39078	39236	158	**-1,568674681**	N11:39170	9,099	+1:YKL210W;	1			
chrXI	41766	41916	150	**-1,284555618**				Overlap <50 bp			
chrXI	42476	42618	142	**-1,143447387**	N11:42545	29,925	+25:YKL209C;	25			
chrXI	42479	42618	139	**-1,367993666**	N11:42545	29,925	+25:YKL209C;	25			
chrXI	44129	44285	156	**-1,523919203**	N11:44262	39,703	+14:YKL209C;	14			
chrXI	45474	45591	117	**-1,231710626**				Overlap <50 bp			
chrXI	52845	53010	165	**-1,440049486**	N11:52908	27,244	+18:YKL205W;	18			
chrXI	53086	53185	99	**-1,730695943**	N11:53095	29,811	+19:YKL205W;	19			
chrXI	55429	55585	156	**-1,246833511**	N11:55522	19,892	+12*:YKL204W;	12	TERM		
chrXI	55463	55623	160	**-1,303570973**	N11:55522	19,892	+12*:YKL204W;	12	TERM		
chrXI	55924	56074	150	**-1,38574672**	N11:55980	9,894		Intergene			
chrXI	55924	56080	156	**-1,354459686**	N11:55980	9,894		Intergene			
chrXI	56846	56978	132	**-1,039575521**	N11:56861	21,047		Intergene			
chrXI	61848	62032	184	**-1,528614544**	N11:61896	24,205		Intergene			
chrXI	63877	64030	153	**-1,645514067**	N11:63947	7,823		Intergene			
chrXI	65154	65316	162	**-1,216569883**	N11:65282	34,252		Intergene			
chrXI	65672	65838	166	**-1,180368197**	N11:65746	17,971		Intergene			
chrXI	68614	68763	149	**-1,293394349**	N11:68734	35,242	+33:YKL197C; +11:YKL198C;	33			
chrXI	73102	73261	159	**-1,354809962**	N11:73185	9,317	+5:YKL197C;	5			
chrXI	86463	86611	148	**-1,294537371**	N11:86556	25,561	+13:YKL188C;	13			
chrXI	86483	86645	162	**-1,351649744**	N11:86556	25,561	+13:YKL188C;	13			
chrXI	88336	88484	148	**-1,360316734**	N11:88371	8,75	+2:YKL188C;	2			
chrXI	89789	89957	168	**-1,179881379**	N11:89946	7,815	+8:YKL187C; +8:SUT223;	8			
chrXI	89789	89957	168	**-1,179881379**	N11:89782	11,075	+9:YKL187C; +7:SUT223;	9			
chrXI	91032	91184	152	**-1,315963927**	N11:91137	10,652	+1:YKL187C; +15:SUT223;	1			
chrXI	97596	97707	111	**-1,407182241**	N11:97729	17,103	+8:YKL184W;	8			
chrXI	97601	97707	106	**-1,435094211**	N11:97729	17,103	+8:YKL184W;	8			
chrXI	99816	99946	130	**-0,634674032**	N11:99812	0		Intergene			
chrXI	99816	99946	130	**-0,634674032**	N11:99961	26,7		Intergene			
chrXI	103049	103197	148	**-1,282889892**	N11:103034	27,998	+15:YKL182W;	15			
chrXI	103049	103197	148	**-1,282889892**	N11:103200	36,661	+16:YKL182W;	16			
chrXI	103372	103526	154	**-1,156374647**	N11:103384	31,835	+17:YKL182W;	17			
chrXI	105256	105405	149	**-1,109838074**	N11:105284	18,267	+29:YKL182W;	29			
chrXI	105988	106134	146	**-0,832272706**	N11:106069	15,457	+34:YKL182W;	34			
chrXI	110658	110811	153	**-1,398077383**	N11:110802	28,341	+12:YKL179C;	12			
chrXI	115225	115382	157	**-1,318169762**	N11:115291	36,875	+2:anti-YKL176C-2;	2			
chrXI	116126	116262	136	**-1,524932821**	N11:116268	37,211	+14:YKL176C;	14			
chrXI	116126	116262	136	**-1,524932821**	N11:116112	30,053	+15:YKL176C;	15			
chrXI	116231	116308	77	**-0,791**	N11:116268	37,211	+14:YKL176C;	14			
chrXI	116232	116308	76	**-0,784**	N11:116268	37,211	+14:YKL176C;	14			
chrXI	116232	116357	125	**-0,554372798**	N11:116268	37,211	+14:YKL176C;	14			
chrXI	117579	117716	137	**-1,047346837**	N11:117661	14,618	+5:YKL176C;	5			
chrXI	120342	120499	157	**-1,253643673**	N11:120460	22,418	+12:YKL174C;	12			
chrXI	120705	120857	152	**-1,373594098**	N11:120781	22,241	+10:YKL174C;	10			
chrXI	121556	121713	157	**-1,840848996**	N11:121624	28,284	+5:YKL174C;	5			
chrXI	130706	130855	149	**-1,294789974**	N11:130776	9,564	+2:YKL170W;	2			
chrXI	130929	131079	150	**-1,243841213**	N11:130940	7,333	+3:YKL170W;	3			
chrXI	132985	133151	166	**-1,265520167**	N11:133074	6,573	+3:YKL168C;	3			
chrXI	134033	134163	130	**-1,413200418**	N11:134102	15,895	+1:YKL167C;	1			
chrXI	136517	136676	159	**-1,640354503**				Overlap <50 bp			
chrXI	140618	140768	150	**-1,365274442**	N11:140692	25,563	+1:YKL165C;	1			
chrXI	142672	142804	132	**-0,873870395**	N11:142756	11,5	+9*:SUT227; +2:YKL164C;	9	TERM		
chrXI	145250	145428	178	**-1,218550033**	N11:145382	18,7	+7:YKL163W;	7			
chrXI	146568	146719	151	**-1,207028851**	N11:146644	7,448		Intergene			
chrXI	147356	147520	164	**-1,525104196**	N11:147421	6,274		Intergene			
chrXI	149408	149568	160	**-1,434937275**	N11:149508	6,892	+9:YKL161C;	9			
chrXI	149964	150108	144	**-1,305308002**	N11:149984	38,811	+6:YKL161C;	6			
chrXI	151755	151890	135	**-1,56837928**	N11:151768	26,098	+2:Unit408;	2			
chrXI	161235	161384	149	**-1,235547801**	N11:161325	6,573	+1:YKL155C; -1:YKL154W;	1			
chrXI	161799	161928	129	**-1,042344321**	N11:161887	12,317	+3:YKL154W;	3			
chrXI	162802	162952	150	**-1,449849305**	N11:162881	9,708		Intergene			
chrXI	170837	170984	147	**-1,956666349**	N11:170954	14,012	+3:YKL148C;	3			
chrXI	171758	171891	133	**-1,210196837**	N11:171815	21,408	+1:YKL146W;	1			
chrXI	172320	172456	136	**-1,128780039**	N11:172446	19,442	+5:YKL146W;	5			
chrXI	178039	178227	188	**-1,620970865**	N11:178152	9,365	+9*:YKL143W;	9	TERM		
chrXI	178982	179138	156	**-1,41986694**	N11:179070	28,487	+4:YKL142W;	4			
chrXI	179058	179209	151	**-1,801591915**	N11:179232	11,314	+1*:Unit387; +5*:YKL142W;	1	TERM		
chrXI	179058	179209	151	**-1,801591915**	N11:179070	28,487	+4:YKL142W;	4			
chrXI	182120	182298	178	**-1,142131712**	N11:182189	5,128	+11:YKL140W;	11			
chrXI	183536	183690	154	**-1,383518065**	N11:183621	15,501	+6:YKL139W;	6			
chrXI	183964	184081	117	**-1,173982643**	N11:183955	25,587	+8:YKL139W;	8			
chrXI	187208	187352	144	**-1,142518053**	N11:187276	5,231	+10:YKL135C;	10			
chrXI	188029	188179	150	**-1,254409008**	N11:188071	21,144	+5:YKL135C;	5			
chrXI	190331	190479	148	**-1,342896657**	N11:190473	13,732	+7:YKL134C;	7			
chrXI	191024	191171	147	**-1,16542173**	N11:191119	8,448	+3:YKL134C;	3			
chrXI	195322	195449	127	**-1,307366987**	N11:195406	13,648	+2:SUT231;	2			

CHR	START	END	L(bp)	ΔLknuc	Nuc ID	Fuzziness	Gene ID	Gene body postion or intergene	Terminal	rDNA	Telomeric
chrXI	196316	196481	165	**-1,342314753**	N11:196493	8,585	+25:YKL129C;	25			
chrXI	196620	196752	132	**-1,172248545**	N11:196660	22,379	+24:YKL129C;	24			
chrXI	201937	202099	162	**-1,657529265**	N11:202016	9,317	+3:anti105;	3			
chrXI	204817	204983	166	**-1,485847362**	N11:204909	7,439	KL127W; -1:YKL126W; +2:SUT663;	12	TERM		
chrXI	207618	207770	152	**-1,135945232**				Overlap <50 bp			
chrXI	209750	209876	126	**-1,257680546**	N11:209839	15,742	-1:YKL124W;	-1			
chrXI	214868	215018	150	**-1,857026937**	N11:214868	21,197	+9:YKL121W;	9			
chrXI	214868	215019	151	**-1,349297145**	N11:214868	21,197	+9:YKL121W;	9			
chrXI	214886	215018	132	**-1,287451285**	N11:214868	21,197	+9:YKL121W;	9			
chrXI	214886	215019	133	**-1,145250632**	N11:214868	21,197	+9:YKL121W;	9			
chrXI	216300	216452	152	**-1,351795978**	N11:216342	6,387	+18*:YKL121W;	18	TERM		
chrXI	216511	216657	146	**-1,055433005**	N11:216610	41,801	-1:YKL120W;	-1			
chrXI	226833	226983	150	**-1,396144118**	N11:226966	16,021	+6:YKL112W;	6			
chrXI	226930	227062	132	**-1,114905315**	N11:226966	16,021	+6:YKL112W;	6			
chrXI	227206	227326	120	**-1,307397937**				Overlap <50 bp			
chrXI	229813	229973	160	**-1,355229093**	N11:229834	45,727	-1:YKL110C;	-1			
chrXI	233634	233784	150	**-1,313879396**	N11:233802	9,899	-1:YKL108W;	-1			
chrXI	233634	233784	150	**-1,313879396**	N11:233638	27,845		Intergene			
chrXI	236594	236764	170	**-1,468111303**	N11:236682	12,661	+2*:YKL106C-A; +7:YKL107W;	2	TERM		
chrXI	241460	241610	150	**-1,128458802**	N11:241499	30,545	+6:YKL105C;	6			
chrXI	245174	245347	173	**-1,534240295**	N11:245177	23,08	+1:YKL104C;	1			
chrXI	245189	245339	150	**-1,28827703**	N11:245177	23,08	+1:YKL104C;	1			
chrXI	245551	245725	174	**-1,516690766**	N11:245572	13,378	-1:CUT229; +12*:YKL103C;	-1			
chrXI	247204	247369	165	**-1,415912496**	N11:247366	10,69	+1:YKL103C;	1			
chrXI	247204	247369	165	**-1,415912496**	N11:247197	5,715	+2:YKL103C;	2			
chrXI	249638	249773	135	**-1,454078261**	N11:249715	14,782	+9*:YKL101W;	9	TERM		
chrXI	251126	251263	137	**-1,669708437**	N11:251198	23,215		Intergene			
chrXI	253186	253299	113	**-1,304056574**	N11:253297	14,096	+12:YKL100C;	12			
chrXI	253541	253688	147	**-1,520689262**	N11:253657	9,529	+10:YKL100C;	10			
chrXI	257352	257460	108	**-0,441043313**				Overlap <50 bp			
chrXI	257353	257478	125	**-1,055238517**	N11:257490	10,685	+4*:CUT708; +8*:YKL098W;	4	TERM		
chrXI	257353	257460	107	**-0,873985153**				Overlap <50 bp			
chrXI	257617	257767	150	**-1,165734102**	N11:257744	10,895	+2:CUT708;	2			
chrXI	258177	258336	159	**-1,332134099**	N11:258256	40,596	-1:CUT708;	-1			
chrXI	261407	261543	136	**-0,960315721**	N11:261504	9,674	-1:YKL095W; +5*:YKL096W;	-1			
chrXI	269387	269539	152	**-1,384663016**	N11:269374	12,178	+6*:YKL091C; -1:YKL092C;	6	TERM		
chrXI	269387	269539	152	**-1,384663016**	N11:269548	11,255	+5:YKL091C;	5			
chrXI	270529	270668	139	**-1,141787867**	N11:270660	5,776	+1:Unit413; +7:SUT666;	1			
chrXI	275707	275869	162	**-1,429772106**	N11:275750	17,297	+6:YKL088W;	6			
chrXI	275877	276030	153	**-1,117352052**	N11:276025	28,839	+8:YKL088W;	8			
chrXI	277324	277474	150	**-1,444413256**	N11:277389	10,132	+2:YKL087C;	2			
chrXI	278497	278637	140	**-1,088358319**	N11:278487	22,78	it414; +5*:YKL086W; -1:YKL085W;	1			
chrXI	278499	278637	138	**-1,071049835**	N11:278487	22,78	it414; +5*:YKL086W; -1:YKL085W;	1			
chrXI	278501	278637	136	**-1,08346536**	N11:278487	22,78	it414; +5*:YKL086W; -1:YKL085W;	1			
chrXI	278502	278637	135	**-0,856760414**	N11:278487	22,78	it414; +5*:YKL086W; -1:YKL085W;	1			
chrXI	282993	283156	163	**-1,441195826**	N11:283075	28,618		Intergene			
chrXI	286651	286824	173	**-1,396885971**	N11:286733	14,89	+5:YKL079W;	5			
chrXI	287603	287693	90	**-0,992563336**	N11:287704	46,226	+11:YKL079W;	11			
chrXI	288326	288474	148	**-1,383497141**				Overlap <50 bp			
chrXI	290076	290228	152	**-1,390011365**	N11:290108	19,357	+11:YKL078W;	11			
chrXI	292965	293112	147	**-1,244305627**	N11:293063	26,326	+7:YKL075C;	7			
chrXI	297006	297125	119	**-1,157503768**	N11:297036	15,007	+7:YKL073W;	7			
chrXI	297006	297156	150	**-1,024765712**	N11:297036	15,007	+7:YKL073W;	7			
chrXI	298702	298832	130	**-1,151423589**	N11:298779	28,827	‹L073W; +1:CUT713; -1:YKL072W;	18	TERM		
chrXI	298875	299007	132	**-1,501964606**				Overlap <50 bp			
chrXI	300199	300305	106	**-1,122868376**	N11:300189	14,849	+11:SUT669; +8:YKL072W;	11			
chrXI	300199	300317	118	**-1,289205404**	N11:300189	14,849	+11:SUT669; +8:YKL072W;	11			
chrXI	303921	304068	147	**-1,420259895**	N11:304040	12,728	+3:Unit394;	3			
chrXI	305787	305949	162	**-1,130742803**				Overlap <50 bp			
chrXI	318927	319072	145	**-1,547433426**				Overlap <50 bp			
chrXI	319054	319202	148	**-1,42625176**	N11:319121	18,148	+13:YKL064W;	13			
chrXI	322500	322670	170	**-1,76912977**	N11:322551	13,736	-1:YKL062W;	-1			
chrXI	329617	329749	132	**-1,0887366**				Overlap <50 bp			
chrXI	329629	329749	120	**-0,701973648**				Overlap <50 bp			
chrXI	329629	329775	146	**-1,416694403**				Overlap <50 bp			
chrXI	337370	337525	155	**-1,385232608**	N11:337362	13,686	+8:YKL054C;	8			
chrXI	337447	337600	153	**-1,287507965**				Overlap <50 bp			
chrXI	337450	337600	150	**-1,429669448**				Overlap <50 bp			
chrXI	337452	337600	148	**-1,484611305**				Overlap <50 bp			
chrXI	337462	337600	138	**-1,181901582**				Overlap <50 bp			
chrXI	337968	338118	150	**-1,313410235**				Overlap <50 bp			
chrXI	340261	340401	140	**-1,16170491**	N11:340305	20,506	0:YKL052C; -1:YKL051W;	0			
chrXI	341684	341830	146	**-1,222004622**	N11:341720	15,777	+7:YKL051W; +1:anti108;	7			
chrXI	347279	347450	171	**-1,410199378**	N11:347369	11,283	+10:YKL048C;	10			
chrXI	347320	347467	147	**-1,5137025**	N11:347369	11,283	+10:YKL048C;	10			
chrXI	347479	347608	129	**-1,104127238**	N11:347544	6,95	+9:YKL048C;	9			
chrXI	351244	351421	177	**-1,54469598**				Overlap <50 bp			
chrXI	351885	352030	145	**-1,135151385**	N11:351957	9,68	+4:YKL046C;	4			
chrXI	352745	352882	137	**-1,083066438**	N11:352864	15,553	-1:YKL045W;	-1			
chrXI	353147	353287	140	**-1,150448513**	N11:353269	12,7	+2:YKL045W;	2			
chrXI	355288	355448	160	**-1,273033904**				Overlap <50 bp			
chrXI	357460	357614	154	**-1,382746132**	N11:357564	11,325	+9*:YKL043W;	9	TERM		
chrXI	357462	357614	152	**-1,692727786**	N11:357564	11,325	+9*:YKL043W;	9	TERM		
chrXI	362917	363070	153	**-1,47423929**				Overlap <50 bp			
chrXI	364062	364210	148	**-1,52575083**				Overlap <50 bp			
chrXI	364602	364723	121	**-0,846473789**	N11:364623	6,535	+2:anti109;	2			
chrXI	368064	368220	156	**-1,459457895**				Overlap <50 bp			
chrXI	368389	368522	133	**-1,205237908**	N11:368440	13,136	+22:YKL038W;	22			
chrXI	368971	369078	107	**-0,970769711**	N11:368963	29,344	+1:YKL037W; +1:anti-YKL037W;	1			
chrXI	368971	369121	150	**-1,34870179**	N11:368963	29,344	+1:YKL037W; +1:anti-YKL037W;	1			
chrXI	368971	369122	151	**-1,331130205**	N11:368963	29,344	+1:YKL037W; +1:anti-YKL037W;	1			
chrXI	372978	373087	109	**-1,430283221**	N11:372980	13,682	+10:YKL034W;	10			
chrXI	373360	373511	151	**-1,228841272**	N11:373499	11,514	+13*:YKL034W;	13	TERM		
chrXI	377591	377751	160	**-1,423960104**	N11:377588	14,078	+16:YKL033W;	16			
chrXI	379254	379407	153	**-1,480716649**	N11:379329	40,278		Intergene			
chrXI	385681	385837	156	**-1,370355021**	N11:385749	5,431	+3:YKL028W;	3			

CHR	START	END	L(bp)	ΔLknuc	Nuc ID	Fuzziness	Gene ID	Gene body postion or intergene	Terminal	rDNA	Telomeric
chrXI	394646	394790	144	**-1,187442759**	N11:394794	41,012	+13:YKL022C;	13			
chrXI	397867	398011	144	**-0,938006967**	N11:398025	2,066	+2:YKL021C;	2			
chrXI	397867	398011	144	**-0,938006967**	N11:397862	6,969	+3:YKL021C;	3			
chrXI	399301	399460	159	**-1,349659052**				Overlap <50 bp			
chrXI	400364	400506	142	**-1,246531614**	N11:400496	32,782	+9:YKL020C;	9			
chrXI	401469	401625	156	**-1,293918031**	N11:401550	1,817	+2:YKL020C;	2			
chrXI	401694	401852	158	**-1,297923275**	N11:401711	12,34	+1:YKL020C;	1			
chrXI	403288	403463	175	**-1,546859788**	N11:403356	7,627	+2:YKL018C-A;	2			
chrXI	406796	406955	159	**-1,345582711**	N11:406832	9,411	+1:YKL017C;	1			
chrXI	407916	408065	149	**-1,286718438**				Overlap <50 bp			
chrXI	416183	416327	144	**-0,953664975**	N11:416221	9,566	+3:YKL014C;	3			
chrXI	417925	418084	159	**-1,336156511**	N11:417916	9,772	+1:YKL012W; -1:YKL013C;	1			
chrXI	417925	418084	159	**-1,336156511**	N11:418102	12,287	+2:YKL012W;	2			
chrXI	434342	434496	154	**-1,350534294**	N11:434396	8,329	+2:YKL005C;	2			
chrXI	434529	434684	155	**-0,686696579**	N11:434609	3,114	-1:YKL004W; +1:YKL005C;	-1			
chrXI	434561	434692	131	**-0,640462858**	N11:434609	3,114	-1:YKL004W; +1:YKL005C;	-1			
chrXI	434662	434792	130	**-1,088301757**				Overlap <50 bp			
chrXI	439672	439806	134	**-1,001442981**	N11:439814	7,767		Intergene			
chrXI	439682	439780	98	**-0,446824277**				Overlap <50 bp			
chrXI	447937	448094	157	**-1,538607083**	N11:447938	9,247	R005C; -1:YKR004C; +2*:Unit396;	10	TERM		
chrXI	448453	448602	149	**-1,313032092**	N11:448490	17,581	+7:YKR005C;	7			
chrXI	450755	450913	158	**-1,022292696**	N11:450835	17,815	-1:YKR007W; +1:YKR006C;	-1			
chrXI	454604	454760	156	**-1,366766752**	N11:454730	21,986	+13:YKR009C;	13			
chrXI	455031	455148	117	**-1,137213638**	N11:455162	46,231	+10:YKR009C; -1:anti110;	10			
chrXI	455031	455138	107	**-1,013180878**				Overlap <50 bp			
chrXI	456736	456888	152	**-1,494743672**	N11:456824	11,843	0:YKR009C;	0			
chrXI	459229	459336	107	**-1,656623344**	N11:459206	18,877	+12:YKR010C;	12			
chrXI	459229	459341	112	**-1,383589223**	N11:459206	18,877	+12:YKR010C;	12			
chrXI	462337	462501	164	**-1,135339798**	N11:462401	7,607	+1:YKR011C; +8*:SUT237;	1			
chrXI	468677	468825	148	**-1,467080651**				Overlap <50 bp			
chrXI	470132	470274	142	**-1,420480946**				Overlap <50 bp			
chrXI	473335	473488	153	**-1,176038381**	N11:473478	15,401	-1:YKR017C; +14:YKR018C;	-1			
chrXI	479959	480086	127	**-1,544628482**	N11:479984	23,099	+8:YKR021W;	8			
chrXI	483328	483476	148	**-1,159816275**	N11:483388	19,159	+3:YKR023W;	3			
chrXI	484474	484623	149	**-1,078249487**	N11:484589	4,472	+10*:YKR023W;	10	TERM		
chrXI	488055	488207	152	**-1,919810319**	N11:488122	34,518	+5*:YKR025W;	5	TERM		
chrXI	488418	488598	180	**-1,676439491**	N11:488497	42,997	+6:YKR026C;	6			
chrXI	496967	497107	140	**-1,532335667**	N11:497088	24,042	+23*:YKR028W; +16*:YKR029C;	23	TERM		
chrXI	501192	501346	154	**-0,931030758**	N11:501317	13,229	+31:YKR031C;	31			
chrXI	506644	506790	146	**-1,204656566**	N11:506685	8,775	+2:YKR034W;	2			
chrXI	509826	509992	166	**-1,433164296**	N11:509894	14,798	+3:YKR036C;	3			
chrXI	514096	514200	104	**-1,941858161**				Overlap <50 bp			
chrXI	516175	516336	161	**-1,456654672**	N11:516248	10,289	+10:YKR039W; +2*:CUT722;	10			
chrXI	517302	517453	151	**-1,283868679**	N11:517465	7,312	+1*:CUT243;	1	TERM		
chrXI	517302	517453	151	**-1,283868679**	N11:517303	5,859	0:CUT243;	0			
chrXI	517458	517572	114	**-1,071799122**	N11:517465	7,312	+1*:CUT243;	1	TERM		
chrXI	519553	519695	142	**-1,954964418**	N11:519598	24,595	-1:SUT680; +3:YKR042W;	-1			
chrXI	527505	527640	135	**-1,776897392**	N11:527634	3,421	+2:YKR050W;	2			
chrXI	527506	527630	124	**-1,728056172**	N11:527634	3,421	+2:YKR050W;	2			
chrXI	527506	527632	126	**-0,942646825**	N11:527634	3,421	+2:YKR050W;	2			
chrXI	527506	527635	129	**-1,067958772**	N11:527634	3,421	+2:YKR050W;	2			
chrXI	527506	527636	130	**-1,311221893**	N11:527634	3,421	+2:YKR050W;	2			
chrXI	527506	527637	131	**-1,220788904**	N11:527634	3,421	+2:YKR050W;	2			
chrXI	527506	527638	132	**-1,434465716**	N11:527634	3,421	+2:YKR050W;	2			
chrXI	527506	527639	133	**-1,63929572**	N11:527634	3,421	+2:YKR050W;	2			
chrXI	527506	527640	134	**-1,074074951**	N11:527634	3,421	+2:YKR050W;	2			
chrXI	527506	527641	135	**-1,725193084**	N11:527634	3,421	+2:YKR050W;	2			
chrXI	527506	527600	94	**-0,781739611**				Overlap <50 bp			
chrXI	527507	527640	133	**-1,65119413**	N11:527634	3,421	+2:YKR050W;	2			
chrXI	527508	527640	132	**-1,408614796**	N11:527634	3,421	+2:YKR050W;	2			
chrXI	527509	527640	131	**-0,731289278**	N11:527634	3,421	+2:YKR050W;	2			
chrXI	527510	527640	130	**-1,107015988**	N11:527634	3,421	+2:YKR050W;	2			
chrXI	527511	527640	129	**-1,291822961**	N11:527634	3,421	+2:YKR050W;	2			
chrXI	527513	527640	127	**-0,997766103**	N11:527634	3,421	+2:YKR050W;	2			
chrXI	527515	527640	125	**-1,085926311**	N11:527634	3,421	+2:YKR050W;	2			
chrXI	527516	527640	124	**-0,580555537**	N11:527634	3,421	+2:YKR050W;	2			
chrXI	529083	529220	137	**-1,014028983**	N11:529204	40,962	+12:YKR050W;	12			
chrXI	529403	529549	146	**-1,047215862**	N11:529570	19,88	+14:YKR050W;	14			
chrXI	532781	532933	152	**-0,973794087**	N11:532891	2,582	+2:YKR052C;	2			
chrXI	533171	533320	149	**-1,230147776**	N11:533271	26,626	0:YKR052C;	0			
chrXI	540415	540570	155	**-1,545139632**	N11:540451	10,801		Intergene			
chrXI	540630	540787	157	**-1,456999207**	N11:540698	31,054		Intergene			
chrXI	540646	540797	151	**-1,303932263**	N11:540698	31,054		Intergene			
chrXI	541723	541874	151	**-1,044781952**	N11:541853	6,238		Intergene			
chrXI	542290	542416	126	**-1,256873632**	N11:542354	21,299		Intergene			
chrXI	542290	542440	150	**-1,063540739**	N11:542354	21,299		Intergene			
chrXI	544676	544823	147	**-0,939931276**				Overlap <50 bp			
chrXI	545722	545853	131	**-0,972122009**	N11:545860	29,126	+12:YKR054C;	12			
chrXI	547817	547975	158	**-1,378496788**	N11:547848	12,42	-1:YKR054C; +1:YKR055W;	-1			
chrXI	548333	548481	148	**-1,188062781**	N11:548344	9,094	+4:YKR055W;	4			
chrXI	548769	548931	162	**-1,068846234**	N11:548953	10,607	-1:YKR056W; +8*:YKR055W;	-1			
chrXI	548769	548931	162	**-1,068846234**	N11:548799	12,814	+7:YKR055W;	7			
chrXI	549757	549907	150	**-1,477039257**	N11:549905	7,815	+5:YKR056W;	5			
chrXI	550284	550422	138	**-1,355780273**	N11:550349	33,534	+8:YKR056W;	8			
chrXI	554400	554551	151	**-1,261101912**	N11:554437	9,899	0:CUT723; 0:YKR059W;	0			
chrXI	558933	559085	152	**-1,440657893**	N11:558954	20,841		Intergene			
chrXI	562000	562173	173	**-1,414134405**	N11:562186	10,108	+1:YKR064W; -1:YKR063C;	1			
chrXI	571010	571152	142	**-1,426668893**	N11:571147	7,234	+1:YKR069W;	1			
chrXI	573165	573306	141	**-1,03733852**	N11:573269	4,457	+1:YKR070W;	1			
chrXI	574030	574178	148	**-1,176840884**	N11:574099	7,893		Intergene			
chrXI	574402	574555	153	**-1,158885339**	N11:574493	31,675	+8*:YKR071C;	8	TERM		
chrXI	575707	575877	170	**-1,814578963**				Overlap <50 bp			
chrXI	577254	577388	134	**-1,08489176**	N11:577250	28,862	+5:YKR072C;	5			
chrXI	577351	577483	132	**-1,248560245**				Overlap <50 bp			
chrXI	577690	577817	127	**-2,232113062**	N11:577711	8,571	+2:YKR072C;	2			
chrXI	578476	578613	137	**-1,053063029**	N11:578514	43,317		Intergene			

CHR	START	END	L(bp)	ΔLknuc	Nuc ID	Fuzziness	Gene ID	Gene body postion or intergene	Terminal	rDNA	Telomeric
chrXI	579137	579295	158	-1,266759603	N11:579194	5,514	+3*:YKR074W;	3	TERM		
chrXI	590716	590822	106	-1,015979271	N11:590776	16,754		Intergene			
chrXI	590716	590835	119	-0,928565403	N11:590776	16,754		Intergene			
chrXI	600361	600514	153	-1,308757421	N11:600370	5,115	+6:YKR086W;	6			
chrXI	600361	600514	153	-1,308757421	N11:600524	8,544	+7:YKR086W;	7			
chrXI	601868	602039	171	-1,646568101	N11:601908	24,105	+15:YKR086W;	15			
chrXI	602067	602222	155	-1,318641727	N11:602124	10,496	+16:YKR086W;	16			
chrXI	603803	603944	141	-1,105775151	N11:603837	21,977	+1:YKR087C; -1:CUT245;	1			
chrXI	604749	604856	107	-1,207103495				Overlap <50 bp			
chrXI	604749	604865	116	-1,400489492				Overlap <50 bp			
chrXI	607498	607646	148	-1,006721509	N11:607543	11,017	+15:SUT241; +4:YKR089C;	15			
chrXI	607998	608143	145	-1,060848765	N11:608050	7,635	+18*:SUT241; +1:YKR089C;	18	TERM		
chrXI	613167	613294	127	-1,292647269	N11:613186	6,348	+3:YKR092C;	3			
chrXI	614331	614461	130	-0,962289354	N11:614435	21,213	+1:Unit424;	1			
chrXI	618644	618800	156	-1,313314927	N11:618766	22,095	+2:SUT242;	2			
chrXI	621479	621632	153	-1,417530231	N11:621620	21,733	+16:YKR095W;	16			
chrXI	624990	625141	151	-0,65999762	N11:625071	10,095		Intergene			
chrXI	632085	632240	155	-1,182367077	N11:632159	7,874	+9:YKR097W;	9			
chrXI	632617	632753	136	-1,149859204	N11:632714	21,925	+15*:YKR098C;	15	TERM		
chrXI	638182	638313	131	-0,862741226	N11:638195	7,629	0:Unit399;	0			
chrXI	638182	638316	134	-0,987126256	N11:638195	7,629	0:Unit399;	0			
chrXI	643087	643244	157	-1,507185576	N11:643162	17,502	+4:Unit426;	4			
chrXI	646189	646315	126	-1,131510651	N11:646259	24,505	+3:YKR102W;	3			
chrXI	647330	647482	152	-1,160321071	N11:647430	61,175	+10:YKR102W;	10			
chrXI	648395	648512	117	-1,79810144				Overlap <50 bp			
chrXI	650461	650612	151	-1,268612453	N11:650494	16,068		Intergene			
chrXI	651142	651292	150	-1,210417098	N11:651252	57,57		Intergene			
chrXI	654048	654197	149	-1,227711402				Overlap <50 bp			
chrXI	655704	655870	166	-1,455669477	N11:655848	10,909	+20:YKR103W;	20			
chrXI	655763	655920	157	-1,248381432	N11:655848	10,909	+20:YKR103W;	20			
chrXI	657176	657328	152	-1,245411891	N11:657284	18,889	+6:YKR104W;	6			
chrXI	658054	658203	149	-1,263381555	N11:658207	19,992	+13*:YKR105C;	13	TERM		
chrXI	659842	659965	123	-1,039751102	N11:659867	6,083	+3:YKR105C;	3			
chrXI	661850	662006	156	-1,523923982	N11:662001	30,265	+6:YKR106W;	6			
chrXI	663739	663846	107	-1,591157694	N11:663807	18,484		Intergene			
chrXI	663739	663851	112	-1,496994452	N11:663807	18,484		Intergene			
chrXI	664373	664519	146	-1,219839868	N11:664394	30,288		Intergene			
chrXI	666612	666748	136	-0,170790474				Overlap <50 bp			TEL RIGHT
chrXI	666628	666815	187	-1,317524387				Overlap <50 bp			TEL RIGHT
chrXI	666656	666815	159	-1,276379071				Overlap <50 bp			TEL RIGHT
chrXI	666681	666771	90	-0,637675508				Overlap <50 bp			TEL RIGHT
chrXI	666681	666810	129	-0,226776015				Overlap <50 bp			TEL RIGHT
chrXI	666693	666810	117	-0,181331826				Overlap <50 bp			TEL RIGHT
chrXI	666703	666728	25	-0,455				Overlap <50 bp			TEL RIGHT
chrXI	666703	666733	30	-0,462				Overlap <50 bp			TEL RIGHT
chrXI	666703	666737	34	-0,49				Overlap <50 bp			TEL RIGHT
chrXI	666703	666739	36	-0,504				Overlap <50 bp			TEL RIGHT
chrXI	666709	666792	83	-1,278737534				Overlap <50 bp			TEL RIGHT
chrXI	666709	666810	101	-0,727775771				Overlap <50 bp			TEL RIGHT
chrXI	666709	666815	106	-0,579146041				Overlap <50 bp			TEL RIGHT
chrXI	666714	666815	101	-0,877945081				Overlap <50 bp			TEL RIGHT
chrXII	723	874	151	-1,215029429	N12:805	36,056	+22:YLL067C;	22			TEL LEFT
chrXII	1167	1315	148	-1,450874716				Overlap <50 bp			TEL LEFT
chrXII	1996	2147	151	-1,114284137	N12:2014	33,886	+15:YLL067C;	15			TEL LEFT
chrXII	2008	2142	134	-1,018833784	N12:2014	33,886	+15:YLL067C;	15			TEL LEFT
chrXII	2033	2193	160	-1,478624355	N12:2177	0	+14:YLL067C;	14			TEL LEFT
chrXII	2033	2129	96	-0,764202285	N12:2014	33,886	+15:YLL067C;	15			TEL LEFT
chrXII	2033	2193	160	-1,478624355	N12:2014	33,886	+15:YLL067C;	15			TEL LEFT
chrXII	2211	2359	148	-1,173163436	N12:2350	42,884	+13:YLL067C;	13			TEL LEFT
chrXII	2226	2355	129	-1,203857305	N12:2350	42,884	+13:YLL067C;	13			TEL LEFT
chrXII	2226	2359	133	-0,950778968	N12:2350	42,884	+13:YLL067C;	13			TEL LEFT
chrXII	2226	2421	195	-1,467525801	N12:2350	42,884	+13:YLL067C;	13			TEL LEFT
chrXII	2387	2517	130	-1,180494239				Overlap <50 bp			TEL LEFT
chrXII	2666	2818	152	-1,257051464	N12:2754	19,655	+11:YLL067C;	11			TEL LEFT
chrXII	3013	3157	144	-0,979723026	N12:3070	21,794	+9:YLL067C;	9			TEL LEFT
chrXII	3013	3200	187	-1,119063938	N12:3070	21,794	+9:YLL067C;	9			TEL LEFT
chrXII	3065	3157	92	-0,362412574	N12:3070	21,794	+9:YLL067C;	9			TEL LEFT
chrXII	3065	3200	135	-1,017112954	N12:3070	21,794	+9:YLL067C;	9			TEL LEFT
chrXII	3153	3297	144	-0,849559593	N12:3257	10,97	+8:YLL067C;	8			TEL LEFT
chrXII	3218	3370	152	-1,283866477	N12:3257	10,97	+8:YLL067C;	8			TEL LEFT
chrXII	3470	3595	125	-0,741908549	N12:3583	6,807	+6:YLL067C;	6			TEL LEFT
chrXII	3500	3643	143	-1,417970922	N12:3583	6,807	+6:YLL067C;	6			TEL LEFT
chrXII	3500	3651	151	-1,327111307	N12:3583	6,807	+6:YLL067C;	6			TEL LEFT
chrXII	3791	3928	137	-1,23138484	N12:3868	7,371	+4:YLL067C;	4			TEL LEFT
chrXII	6346	6517	171	-1,58589495	N12:6439	52,275	+23:YLL066C;	23			TEL LEFT
chrXII	6366	6517	151	-1,240022875	N12:6439	52,275	+23:YLL066C;	23			TEL LEFT
chrXII	6690	6864	174	-1,542909836	N12:6762	16,258	+21:YLL066C;	21			TEL LEFT
chrXII	6705	6857	152	-1,661251081	N12:6762	16,258	+21:YLL066C;	21			TEL LEFT
chrXII	7495	7682	187	-1,207477855	N12:7546	19,313	+16:YLL066C;	16			TEL LEFT
chrXII	7531	7682	151	-1,036472979	N12:7546	19,313	+16:YLL066C;	16			TEL LEFT
chrXII	7543	7677	134	-0,979266891	N12:7546	19,313	+16:YLL066C;	16			TEL LEFT
chrXII	7568	7664	96	-0,897952495	N12:7546	19,313	+16:YLL066C;	16			TEL LEFT
chrXII	7746	7894	148	-1,380037904	N12:7816	1,414	+14:YLL066C;	14			TEL LEFT
chrXII	7761	7894	133	-0,967320245	N12:7816	1,414	+14:YLL066C;	14			TEL LEFT
chrXII	7761	7956	195	-1,537952513	N12:7816	1,414	+14:YLL066C;	14			TEL LEFT
chrXII	7922	8052	130	-1,180881073				Overlap <50 bp			TEL LEFT
chrXII	8201	8353	152	-1,093006861	N12:8284	26,502	+11:YLL066C;	11			TEL LEFT
chrXII	8548	8692	144	-0,964894372	N12:8607	17,786	+9:YLL066C;	9			TEL LEFT
chrXII	8548	8735	187	-1,383346343	N12:8607	17,786	+9:YLL066C;	9			TEL LEFT
chrXII	8600	8692	92	-0,712992886	N12:8607	17,786	+9:YLL066C;	9			TEL LEFT
chrXII	8600	8735	135	-0,984965139	N12:8607	17,786	+9:YLL066C;	9			TEL LEFT
chrXII	8688	8832	144	-1,18201862	N12:8786	7,024	+8:YLL066C;	8			TEL LEFT
chrXII	8753	8905	152	-1,311576448	N12:8786	7,024	+8:YLL066C;	8			TEL LEFT
chrXII	9005	9130	125	-1,016326141	N12:9112	11,533	+6:YLL066C;	6			TEL LEFT
chrXII	9035	9178	143	-1,525598667	N12:9112	11,533	+6:YLL066C;	6			TEL LEFT
chrXII	9326	9463	137	-1,097412697	N12:9402	8,021	+4:YLL066C;	4			TEL LEFT

CHR	START	END	L(bp)	ΔLknuc	Nuc ID	Fuzziness	Gene ID	Gene body postion or intergene	Terminal	rDNA	Telomeric
chrXII	11195	11357	162	-1,172257359	N12:11275	20,614		Intergene			TEL LEFT
chrXII	11198	11354	156	-0,882613757	N12:11275	20,614		Intergene			TEL LEFT
chrXII	11205	11354	149	-1,648300921	N12:11275	20,614		Intergene			TEL LEFT
chrXII	11205	11357	152	-0,788488036	N12:11275	20,614		Intergene			TEL LEFT
chrXII	11207	11357	150	-1,170195429	N12:11275	20,614		Intergene			TEL LEFT
chrXII	11208	11354	146	-1,088604166	N12:11275	20,614		Intergene			TEL LEFT
chrXII	11211	11357	146	-1,137888129	N12:11275	20,614		Intergene			TEL LEFT
chrXII	11213	11310	97	-0,275265869	N12:11275	20,614		Intergene			TEL LEFT
chrXII	11213	11346	133	-0,558080116	N12:11275	20,614		Intergene			TEL LEFT
chrXII	11213	11352	139	-1,815797997	N12:11275	20,614		Intergene			TEL LEFT
chrXII	11213	11355	142	-1,903383612	N12:11275	20,614		Intergene			TEL LEFT
chrXII	11213	11357	144	-1,638455941	N12:11275	20,614		Intergene			TEL LEFT
chrXII	11213	11358	145	-2,074981651	N12:11275	20,614		Intergene			TEL LEFT
chrXII	11218	11354	136	-1,059025851	N12:11275	20,614		Intergene			TEL LEFT
chrXII	11222	11354	132	-1,029743305	N12:11275	20,614		Intergene			TEL LEFT
chrXII	11222	11357	135	-1,331619259	N12:11275	20,614		Intergene			TEL LEFT
chrXII	11226	11354	128	-0,649215873	N12:11275	20,614		Intergene			TEL LEFT
chrXII	11226	11357	131	-0,896213419	N12:11275	20,614		Intergene			TEL LEFT
chrXII	11226	11358	132	-1,02941682	N12:11275	20,614		Intergene			TEL LEFT
chrXII	11227	11357	130	-0,959847532	N12:11275	20,614		Intergene			TEL LEFT
chrXII	11228	11357	129	-1,495122644	N12:11275	20,614		Intergene			TEL LEFT
chrXII	11229	11357	128	-0,88002734	N12:11275	20,614		Intergene			TEL LEFT
chrXII	11230	11357	127	-1,43034951	N12:11275	20,614		Intergene			TEL LEFT
chrXII	11231	11354	123	-0,419679387	N12:11275	20,614		Intergene			TEL LEFT
chrXII	11231	11357	126	-0,411269424	N12:11275	20,614		Intergene			TEL LEFT
chrXII	11231	11358	127	-0,421440385	N12:11275	20,614		Intergene			TEL LEFT
chrXII	11233	11357	124	-0,430225541	N12:11275	20,614		Intergene			TEL LEFT
chrXII	11235	11357	122	-0,812901387	N12:11275	20,614		Intergene			TEL LEFT
chrXII	11252	11354	102	-0,396421488	N12:11275	20,614		Intergene			TEL LEFT
chrXII	11252	11357	105	-0,831026036	N12:11275	20,614		Intergene			TEL LEFT
chrXII	11259	11357	98	-0,620313337	N12:11275	20,614		Intergene			TEL LEFT
chrXII	11262	11357	95	-1,070259517	N12:11275	20,614		Intergene			TEL LEFT
chrXII	11273	11357	84	-1,168520136	N12:11275	20,614		Intergene			TEL LEFT
chrXII	13256	13420	164	-0,944229987	N12:13360	13,115	+2:YLL064C;	2			
chrXII	13298	13427	129	-1,394486236	N12:13360	13,115	+2:YLL064C;	2			
chrXII	13457	13612	155	-0,981379292	N12:13509	0,707	+1:YLL064C;	1			
chrXII	15085	15257	172	-1,376752866	N12:15154	16,622	+6:YLL063C;	6			
chrXII	15201	15361	160	-1,305305707	N12:15316	26,994	+5:YLL063C;	5			
chrXII	16724	16865	141	-1,229086565	N12:16731	5,128	+7*:YLL062C;	7	TERM		
chrXII	18847	18996	149	-1,205823412	N12:18970	11,546	+7:YLL061W;	7			
chrXII	25117	25257	140	-1,021632727	N12:25169	33,344	+11*:YLL058W;	11	TERM		
chrXII	27714	27865	151	-1,098276376	N12:27788	23,692	+5:YLL056C;	5			
chrXII	35557	35688	131	-0,859584828	N12:35640	5,505	+3:YLL053C;	3			
chrXII	39250	39399	149	-1,599332552	N12:39315	14,152	+2:YLL051C;	2			
chrXII	48577	48723	146	-1,426204565	N12:48603	33,129	+1:YLL045C;	1			
chrXII	51782	51932	150	-1,286591913	N12:51881	32,34		Intergene			
chrXII	56756	56881	125	-1,027874236	N12:56813	23,372	+43*:YLL040C;	43	TERM		
chrXII	57933	58079	146	-1,16846774	N12:58069	15,659	+35:YLL040C;	35			
chrXII	60326	60478	152	-1,3612987	N12:60341	25,056	+21:YLL040C;	21			
chrXII	62223	62381	158	-1,404856717				Overlap <50 bp			
chrXII	67309	67460	151	-1,775579442				Overlap <50 bp			
chrXII	70032	70180	148	-1,262270144	N12:70102	20,315	+11:YLL035W;	11			
chrXII	70780	70917	137	-1,07679287	N12:70939	17,869	+15:YLL034C;	15			
chrXII	70780	70917	137	-1,07679287	N12:70773	35,665	+16:YLL034C;	16			
chrXII	71343	71501	158	-1,591948363	N12:71413	23,137	+12:YLL034C;	12			
chrXII	73489	73643	154	-1,451911524	N12:73587	11,161	+3:anti-YLL033W; +2:YLL033W;	3			
chrXII	79801	79962	161	-1,176457144				Overlap <50 bp			
chrXII	99031	99175	144	-1,136021263	N12:99103	10,373	CUT246; +7*:YLL022C; -1:YLL023C;	8	TERM		
chrXII	104748	104900	152	-1,158723201	N12:104799	23,028		Intergene			
chrXII	104994	105164	170	-1,284073315	N12:104983	15,052		Intergene			
chrXII	106767	106921	154	-1,629492884	N12:106904	35,972	+8:YLL019C;	8			
chrXII	107154	107242	88	-1,177940147	N12:107195	27,508	+6:YLL019C;	6			
chrXII	107154	107250	96	-1,147943904	N12:107195	27,508	+6:YLL019C;	6			
chrXII	107154	107252	98	-0,829810076	N12:107195	27,508	+6:YLL019C;	6			
chrXII	107154	107253	99	-0,896035148	N12:107195	27,508	+6:YLL019C;	6			
chrXII	107154	107255	101	-1,040529761	N12:107195	27,508	+6:YLL019C;	6			
chrXII	107154	107257	103	-0,637750857	N12:107195	27,508	+6:YLL019C;	6			
chrXII	107154	107258	104	-0,79579632	N12:107195	27,508	+6:YLL019C;	6			
chrXII	107154	107259	105	-0,959044453	N12:107195	27,508	+6:YLL019C;	6			
chrXII	107154	107260	106	-0,909917987	N12:107195	27,508	+6:YLL019C;	6			
chrXII	107154	107261	107	-0,890953559	N12:107195	27,508	+6:YLL019C;	6			
chrXII	107154	107262	108	-1,012194712	N12:107195	27,508	+6:YLL019C;	6			
chrXII	107154	107265	111	-1,121650654	N12:107195	27,508	+6:YLL019C;	6			
chrXII	107154	107267	113	-1,262695093	N12:107195	27,508	+6:YLL019C;	6			
chrXII	107154	107269	115	-1,186868891	N12:107195	27,508	+6:YLL019C;	6			
chrXII	107154	107270	116	-0,945675724	N12:107195	27,508	+6:YLL019C;	6			
chrXII	107154	107271	117	-1,585444505	N12:107195	27,508	+6:YLL019C;	6			
chrXII	107154	107273	119	-1,305611231	N12:107195	27,508	+6:YLL019C;	6			
chrXII	107154	107274	120	-1,180463625	N12:107195	27,508	+6:YLL019C;	6			
chrXII	107154	107285	131	-1,112987873	N12:107195	27,508	+6:YLL019C;	6			
chrXII	107271	107423	152	-1,134848349	N12:107365	32,347	+5:YLL019C;	5			
chrXII	110633	110799	166	-1,622628817	N12:110737	24,954	+6:YLL018C;	6			
chrXII	112506	112634	128	-1,589031641	N12:112551	10,998	+11:anti115;	11			
chrXII	113051	113206	155	-1,440164869	N12:113139	42,716	+8:anti115;	8			
chrXII	118248	118400	152	-1,399982792	N12:118377	9,504	+14:YLL015W;	14			
chrXII	119597	119738	141	-1,356877866	N12:119672	27,683	+21:YLL015W;	21			
chrXII	121717	121889	172	-1,646744401	N12:121848	32,485		Intergene			
chrXII	126647	126799	152	-1,352649175	N12:126629	5,657	+8:YLL012W;	8			
chrXII	132281	132417	136	-1,162843256				Overlap <50 bp			
chrXII	132310	132466	156	-1,370869368				Overlap <50 bp			
chrXII	133301	133451	150	-1,322944539	N12:133308	20,688	+11:YLL008W;	11			
chrXII	133762	133946	184	-1,786922939	N12:133881	17,521	+15:YLL008W;	15			
chrXII	136653	136779	126	-1,380410878	N12:136749	12,656	+2:YLL006W; +3*:YLL006W-A;	2			
chrXII	138426	138582	156	-1,445659153	N12:138554	20,664	+13:YLL005C; +12*:YLL006W;	13			
chrXII	142345	142492	147	-1,455631662	N12:142429	11,673	+9:YLL004W;	9			
chrXII	143973	144111	138	-1,373961249	N12:144010	17,904	+6:YLL003W;	6			

CHR	START	END	L(bp)	ΔLknuc	Nuc ID	Fuzziness	Gene ID	Gene body postion or intergene	Terminal	rDNA	Telomeric
chrXII	144765	144914	149	-1,220921696	N12:144832	22,013	+11:YLL003W;	11			
chrXII	145430	145576	146	-1,327704722	N12:145533	19,524	+15:YLL003W;	15			
chrXII	155097	155220	123	-0,928358937	N12:155205	20,149	+8:YLR002C;	8			
chrXII	160945	161106	161	-1,329141498	N12:161017	21,909	+6:YLR005W;	6			
chrXII	163470	163588	118	-1,509741427	N12:163522	13,565	+4:YLR006C;	4			
chrXII	171732	171883	151	-1,548097036	N12:171897	23,169	+4*:YLR013W;	4	TERM		
chrXII	177830	177964	134	-1,08819054	N12:177819	6,542	+2:YLR017W;	2			
chrXII	177830	177964	134	-1,08819054	N12:177986	6,686	+3:YLR017W;	3			
chrXII	178588	178741	153	-1,170853701				Overlap <50 bp			
chrXII	179076	179226	150	-1,817080258	N12:179114	7,808	+4:YLR018C;	4			
chrXII	180676	180836	160	-1,272923005	N12:180728	11,726	+5:YLR019W;	5			
chrXII	183315	183461	146	-1,089593376	N12:183389	4,037	+1:YLR020C; -1:YLR021W;	1			
chrXII	192705	192836	131	-1,196488888	N12:192762	22,418	+5:YLR024C;	5			
chrXII	193019	193125	106	-0,868440921	N12:193086	6,646	+3:YLR024C;	3			
chrXII	193019	193144	125	-1,138759005	N12:193086	6,646	+3:YLR024C;	3			
chrXII	194389	194550	161	-1,550441442	N12:194429	16,669	+1:YLR025W;	1			
chrXII	197350	197482	132	-2,001958585	N12:197391	23,618	+4*:CUT252; +5:YLR027C;	4	TERM		
chrXII	197802	197965	163	-1,362741621				Overlap <50 bp			
chrXII	199062	199223	161	-1,429848524	N12:199130	20,257		Intergene			
chrXII	199271	199406	135	-1,082122182	N12:199299	18,673		Intergene			
chrXII	199721	199871	150	-1,383895841	N12:199806	27,862	+10:YLR028C;	10			
chrXII	207917	208073	156	-1,708440981	N12:207961	16,362	+19:YLR032W;	19			
chrXII	208414	208561	147	-1,372723134	N12:208481	8,746	+22*:YLR032W; -1:YLR033W;	22	TERM		
chrXII	209818	209960	142	-0,934799937	N12:209848	25,883	+8:YLR033W;	8			
chrXII	211059	211185	126	-0,731668939	N12:211093	24,468	+7:YLR034C;	7			
chrXII	213217	213369	152	-1,163751071	N12:213269	5,269	+8:YLR035C;	8			
chrXII	214356	214519	163	-1,550549705	N12:214440	10,328	+1:YLR035C;	1			
chrXII	216121	216270	149	-1,158542274	N12:216153	39,703		Intergene			
chrXII	217884	218033	149	-0,865537787	N12:218006	19,732		Intergene			
chrXII	218108	218265	157	-1,545823073	N12:218186	18,877		Intergene			
chrXII	218294	218444	150	-0,982792643	N12:218356	27,099		Intergene			
chrXII	218298	218444	146	-1,080093025	N12:218356	27,099		Intergene			
chrXII	218756	218891	135	-1,18255401	N12:218862	33,645		Intergene			
chrXII	219794	219934	140	-1,098200789	N12:219876	37,634		Intergene			
chrXII	219800	219934	134	-1,111348084	N12:219876	37,634		Intergene			
chrXII	220212	220367	155	-0,8618629	N12:220242	19,925		Intergene			
chrXII	220343	220451	108	-1,124492107	N12:220455	49,003		Intergene			
chrXII	223444	223638	194	-2,232023883	N12:223443	6,524	-1:YLR037C; +7:SUT688;	-1			
chrXII	223444	223638	194	-2,232023883	N12:223614	15,123	+6:SUT688;	6			
chrXII	230481	230653	172	-1,761350347	N12:230555	17,166	-1:SUT251; 0:YLR042C;	-1			
chrXII	230739	230894	155	-1,291902736	N12:230860	10,967	+1:SUT251;	1			
chrXII	232813	232957	144	-1,376459762	N12:232966	14,154	+7:YLR044C;	7			
chrXII	232813	232957	144	-1,376459762	N12:232814	22,311	+8:YLR044C;	8			
chrXII	235469	235619	150	-0,881454953	N12:235506	16,538	+14:YLR045C; +6:SUT252;	14			
chrXII	235483	235631	148	-1,571333857	N12:235506	16,538	+14:YLR045C; +6:SUT252;	14			
chrXII	237484	237634	150	-1,414712146	N12:237551	10,87	+2:YLR045C;	2			
chrXII	238295	238446	151	-1,419905244	N12:238404	24,977	+21*:YLR047C; +4:YLR046C;	21	TERM		
chrXII	239615	239753	138	-1,219336787	N12:239689	6,921	+13:YLR047C;	13			
chrXII	255888	256021	133	-1,055837785				Overlap <50 bp			
chrXII	256324	256484	160	-1,11953004	N12:256416	10,284	+8:YLR057W;	8			
chrXII	259071	259235	164	-1,445486947	N12:259139	18,69	+3:YLR058C;	3			
chrXII	260642	260797	155	-1,061392298	N12:260719	3,312	0:YLR059C; -1:YLR060W;	0			
chrXII	262571	262718	147	-1,15601585	N12:262667	32,722		Intergene			
chrXII	270658	270826	168	-1,526070813	N12:270763	5,465	+1:YLR067C; -1:YLR068W;	1			
chrXII	270673	270826	153	-0,772546666	N12:270763	5,465	+1:YLR067C; -1:YLR068W;	1			
chrXII	271752	271896	144	-1,236893198	N12:271899	25,981	+13:YLR069C;	13			
chrXII	273309	273417	108	-1,281740562	N12:273383	18,217	+4:YLR069C;	4			
chrXII	273309	273418	109	-1,124731612	N12:273383	18,217	+4:YLR069C;	4			
chrXII	273309	273419	110	-1,420347604	N12:273383	18,217	+4:YLR069C;	4			
chrXII	273309	273420	111	-1,42419264	N12:273383	18,217	+4:YLR069C;	4			
chrXII	274415	274568	153	-1,311784924	N12:274467	16,86	+3:anti119; +5:YLR070C;	3			
chrXII	275945	276112	167	-1,475374505	N12:276130	34,15	+16:YLR071C;	16			
chrXII	275945	276112	167	-1,475374505	N12:275964	18,771	+17:YLR071C;	17			
chrXII	280170	280332	162	-1,090622611	N12:280290	22,143	+9:YLR072W;	9			
chrXII	280378	280532	154	-1,23215883	N12:280450	34,574	+10:YLR072W;	10			
chrXII	281608	281761	153	-1,746857777	N12:281710	25,034	+1:YLR073C; +4:SUT257;	1			
chrXII	285125	285277	152	-1,149068845	N12:285193	27,083	+9:YLR077W;	9			
chrXII	285128	285277	149	-1,418503979	N12:285193	27,083	+9:YLR077W;	9			
chrXII	286283	286443	160	-1,331590165	N12:286398	3,724	+1:YLR078C;	1			
chrXII	292173	292332	159	-1,504775814	N12:292269	6,245	+9*:YLR082C;	9	TERM		
chrXII	293330	293472	142	-1,364315349	N12:293400	19,892	+2:YLR082C;	2			
chrXII	295117	295270	153	-1,404531963	N12:295096	27,545	+8:YLR083C;	8			
chrXII	295472	295620	148	-0,965011755	N12:295545	10,863	+5:YLR083C;	5			
chrXII	296601	296740	139	-1,022373239	N12:296667	9,45	+24*:YLR084C;	24	TERM		
chrXII	300804	300959	155	-1,054261819	N12:300827	33,018	+8:YLR085C;	8			
chrXII	301895	302037	142	-1,469906682	N12:301967	8,773	-1:YLR086W; +1:YLR085C;	-1			
chrXII	302498	302646	148	-1,183312288	N12:302567	3,71	+3:YLR086W;	3			
chrXII	304308	304455	147	-1,210972085	N12:304426	22,884	+14:YLR086W;	14			
chrXII	304635	304774	139	-1,018120678	N12:304748	16,754	+16:YLR086W;	16			
chrXII	310453	310583	130	-1,253342475	N12:310571	35,549	+32:YLR087C;	32			
chrXII	311744	311903	159	-1,706893012				Overlap <50 bp			
chrXII	312700	312835	135	-1,418469889	N12:312689	30,859	+19:YLR087C;	19			
chrXII	314498	314655	157	-1,439540057	N12:314563	26,84	+8:YLR087C;	8			
chrXII	319179	319325	146	-1,49771803	N12:319307	38,105	+6:YLR089C;	6			
chrXII	320329	320487	158	-1,591880671	N12:320461	19,14	R089C; -1:YLR090W; +2*:Unit433;	-1			
chrXII	320335	320487	152	-1,35133557	N12:320461	19,14	R089C; -1:YLR090W; +2*:Unit433;	-1			
chrXII	320336	320487	151	-1,615866232	N12:320461	19,14	R089C; -1:YLR090W; +2*:Unit433;	-1			
chrXII	321520	321655	135	-1,260570288	N12:321554	31,642	+6:YLR090W;	6			
chrXII	322116	322262	146	-1,243086272				Overlap <50 bp			
chrXII	327876	328044	168	-1,360443226	N12:327934	21,847	+9:YLR094C;	9			
chrXII	338236	338387	151	-1,298905447	N12:338317	25,519	+8:YLR098C;	8			
chrXII	340076	340209	133	-1,104252333	N12:340159	23,969	+5:SUT261; +5:YLR099C;	5			
chrXII	340597	340761	164	-1,579950386	N12:340639	18,276	+8:SUT261; +2:YLR099C;	8			
chrXII	340875	341034	159	-1,188794746	N12:340984	11,389	JT261; -1:YLR099W-A; 0:YLR099C;	10			
chrXII	347768	347916	148	-1,231399673	N12:347855	31,324	+3:YLR105C;	3			
chrXII	347831	348011	180	-1,779904971	N12:348007	34,01	+2:YLR105C;	2			

CHR	START	END	L(bp)	ΔLknuc	Nuc ID	Fuzziness	Gene ID	Gene body postion or intergene	Terminal	rDNA	Telomeric
chrXII	347831	348011	180	-1,779904971	N12:347855	31,324	+3:YLR105C;	3			
chrXII	348735	348938	203	-1,825869978	N12:348883	16,971	+1*:Unit434;	1	TERM		
chrXII	348736	348938	202	-1,743917651	N12:348883	16,971	+1*:Unit434;	1	TERM		
chrXII	350679	350830	151	-1,173055678				Overlap <50 bp			
chrXII	355881	356007	126	-1,525486704	N12:355867	7,211		Intergene			
chrXII	355881	356007	126	-1,525486704	N12:356021	30,128		Intergene			
chrXII	358183	358323	140	-1,185859295	N12:358217	18,166		Intergene			
chrXII	359562	359714	152	-1,337600487	N12:359697	24,334		Intergene			
chrXII	360814	360966	152	-0,997567375				Overlap <50 bp			
chrXII	367954	368092	138	-1,486814766	N12:368026	16,087	+2:YLR108C;	2			
chrXII	372826	372968	142	-1,254508295	N12:372913	22,665	+10*:YLR113W;	10	TERM		
chrXII	375291	375434	143	-1,0007626	N12:375295	9,539	+13:YLR114C;	13			
chrXII	375584	375750	166	-1,78042701	N12:375684	28,702	+11:YLR114C;	11			
chrXII	388796	388957	161	-1,35102331	N12:388850	23,676	1:Unit436; +8:YLR121C; 0:anti122;	-1			
chrXII	388880	389024	144	-1,111894857	N12:389034	42,293	+7:YLR121C; +2:anti122;	7			
chrXII	391687	391826	139	-0,996018838				Overlap <50 bp			
chrXII	395857	396006	149	-0,959966178	N12:395841	5,225	LR126C; +1:SUT263; +17:YLR127C;	-1			
chrXII	395857	396006	149	-0,959966178	N12:396018	7,685	+2:SUT263; +16:YLR127C;	2			
chrXII	397064	397220	156	-1,208131262	N12:397207	7,396	+8:YLR127C;	8			
chrXII	398081	398231	150	-1,087937152	N12:398173	2,757	+2:YLR127C;	2			
chrXII	400245	400397	152	-1,327418445				Overlap <50 bp			
chrXII	401669	401819	150	-1,261426889	N12:401676	20,371	+13:YLR129W;	13			
chrXII	401669	401819	150	-1,261426889	N12:401833	28,733	+14:YLR129W;	14			
chrXII	405414	405520	106	-1,20271227	N12:405442	26,857	+18:YLR132C; +10:YLR131C;	18			
chrXII	405414	405538	124	-1,530378564	N12:405442	26,857	+18:YLR132C; +10:YLR131C;	18			
chrXII	406427	406580	153	-1,4667777	N12:406529	22,876	+11:YLR132C; +3:YLR131C;	11			
chrXII	406621	406768	147	-1,295054924	N12:406710	17,175	+10:YLR132C; +2:YLR131C;	10			
chrXII	408412	408522	110	-0,856810074	N12:408439	9,564	+1:YLR133W; -1:YLR132C;	1			
chrXII	408412	408523	111	-0,773911104	N12:408439	9,564	+1:YLR133W; -1:YLR132C;	1			
chrXII	408415	408537	122	-1,871925096	N12:408439	9,564	+1:YLR133W; -1:YLR132C;	1			
chrXII	411908	412040	132	-1,246599746	N12:411988	27,15		Intergene			
chrXII	412316	412427	111	-1,377712832	N12:412343	15,55		Intergene			
chrXII	412319	412427	108	-1,363948696	N12:412343	15,55		Intergene			
chrXII	412320	412427	107	-1,410672298	N12:412343	15,55		Intergene			
chrXII	419068	419220	152	-1,831397902	N12:419117	3,564	+6:YLR138W;	6			
chrXII	419734	419893	159	-1,197104405	N12:419779	16,709		Intergene			
chrXII	421131	421283	152	-1,397536735	N12:421250	7,603		Intergene			
chrXII	423648	423793	145	-0,679647373	N12:423723	9,888	-1:YLR139C; +1:YLR141W;	-1			
chrXII	424263	424441	178	-1,27029736	N12:424247	2,828	+4:YLR141W;	4			
chrXII	425377	425494	117	-1,564343258	N12:425416	10,685	+2:YLR142W;	2			
chrXII	428829	428982	153	-1,127774284	N12:428985	31,284	+10:YLR143W;	10			
chrXII	433448	433586	138	-0,949881576	N12:433530	10,431	+2:YLR146C; -1:YLR146W-A;	2			
chrXII	433602	433749	147	-1,728650857	N12:433706	8,802	+1:YLR146C; 0:YLR146W-A;	1			
chrXII	434337	434490	153	-1,222846256	N12:434430	3,559	-1:YLR148W; +1:YLR147C;	-1			
chrXII	443464	443629	165	-1,440060345	N12:443445	27,112	+8*:YLR152C;	8	TERM		
chrXII	446788	446962	174	-1,512566972	N12:446975	12,675	+5:YLR153C;	5			
chrXII	446788	446962	174	-1,512566972	N12:446824	24,183	+6:YLR153C;	6			
chrXII	447291	447454	163	-1,544928781	N12:447462	18,694	+2:YLR153C;	2			
chrXII	447291	447454	163	-1,544928781	N12:447286	3,271	+3:YLR153C;	3			
chrXII	450323	450477	154	-1,163487034	N12:450413	20,919	+5:Unit457;	5			
chrXII	451465	451626	161	-1,639740675	N12:451492	34,463				rDNA	
chrXII	451590	451738	148	-1,494170043				Overlap <50 bp		rDNA	
chrXII	451599	451733	134	-1,164068075				Overlap <50 bp		rDNA	
chrXII	451709	451842	133	-1,433511471	N12:451807	35,838				rDNA	
chrXII	451726	451885	159	-1,722956651	N12:451807	35,838				rDNA	
chrXII	451734	451869	135	-1,569225321	N12:451807	35,838				rDNA	
chrXII	451757	451842	85	-0,457327461	N12:451807	35,838				rDNA	
chrXII	451757	451908	151	-1,462446946	N12:451807	35,838				rDNA	
chrXII	451769	451932	163	-1,565028581	N12:451807	35,838				rDNA	
chrXII	451790	451908	118	-1,165323576	N12:451807	35,838				rDNA	
chrXII	451790	451932	142	-1,291093773	N12:451807	35,838				rDNA	
chrXII	451790	451960	170	-1,302580348	N12:451807	35,838				rDNA	
chrXII	451790	451960	170	-1,302580348	N12:451976	40,079				rDNA	
chrXII	451800	451934	134	-1,064520309	N12:451807	35,838				rDNA	
chrXII	451800	451961	161	-1,548953909	N12:451807	35,838				rDNA	
chrXII	451800	451961	161	-1,548953909	N12:451976	40,079				rDNA	
chrXII	451851	451934	83	-0,778815603				Overlap <50 bp		rDNA	
chrXII	451851	451958	107	-1,329802255	N12:451976	40,079				rDNA	
chrXII	451851	451959	108	-1,256939933	N12:451976	40,079				rDNA	
chrXII	451851	451960	109	-1,510613919	N12:451976	40,079				rDNA	
chrXII	451851	451961	110	-1,285875966	N12:451976	40,079				rDNA	
chrXII	451876	451960	84	-0,791582711	N12:451976	40,079				rDNA	
chrXII	451876	452009	133	-1,193600939	N12:451976	40,079				rDNA	
chrXII	451919	452075	156	-1,420369664	N12:451976	40,079				rDNA	
chrXII	451919	452077	158	-1,221589763	N12:451976	40,079				rDNA	
chrXII	451919	452078	159	-1,243546966	N12:451976	40,079				rDNA	
chrXII	451919	452092	173	-1,30589316	N12:451976	40,079				rDNA	
chrXII	451923	452075	152	-1,157762497	N12:451976	40,079				rDNA	
chrXII	451923	452077	154	-1,639654166	N12:451976	40,079				rDNA	
chrXII	451923	452092	169	-1,235507847	N12:451976	40,079				rDNA	
chrXII	451931	452062	131	-1,254948784	N12:451976	40,079				rDNA	
chrXII	451931	452077	146	-1,193787476	N12:451976	40,079				rDNA	
chrXII	451931	452109	178	-1,437264692	N12:451976	40,079				rDNA	
chrXII	451932	452062	130	-1,124495991	N12:451976	40,079				rDNA	
chrXII	451932	452075	143	-1,282064137	N12:451976	40,079				rDNA	
chrXII	451932	452077	145	-1,605633967	N12:451976	40,079				rDNA	
chrXII	451932	452109	177	-1,331310592	N12:451976	40,079				rDNA	
chrXII	451945	452051	106	-0,736154396	N12:451976	40,079				rDNA	
chrXII	451945	452062	117	-0,868281387	N12:451976	40,079				rDNA	
chrXII	451945	452075	130	-0,753814651	N12:451976	40,079				rDNA	
chrXII	451945	452077	132	-0,982406328	N12:451976	40,079				rDNA	
chrXII	451945	452078	133	-1,027384552	N12:451976	40,079				rDNA	
chrXII	451945	452092	147	-1,039323428	N12:451976	40,079				rDNA	
chrXII	451945	452109	164	-1,306733111	N12:451976	40,079				rDNA	
chrXII	451958	452109	151	-1,088061784	N12:451976	40,079				rDNA	
chrXII	452041	452196	155	-1,080362528	N12:452173	28,583				rDNA	

CHR	START	END	L(bp)	ΔLknuc	Nuc ID	Fuzziness	Gene ID	Gene body postion or intergene	Terminal	rDNA	Telomeric
chrXII	452059	452215	156	**-1,294197441**	N12:452173	28,583				rDNA	
chrXII	452081	452227	146	**-1,105546451**	N12:452173	28,583				rDNA	
chrXII	452081	452236	155	**-1,439692328**	N12:452173	28,583				rDNA	
chrXII	452081	452247	166	**-1,195652769**	N12:452173	28,583				rDNA	
chrXII	452081	452251	170	**-1,363803369**	N12:452173	28,583				rDNA	
chrXII	452104	452247	143	**-0,977867166**	N12:452173	28,583				rDNA	
chrXII	452104	452251	147	**-1,07513005**	N12:452173	28,583				rDNA	
chrXII	452115	452271	156	**-1,218995954**	N12:452173	28,583				rDNA	
chrXII	452190	452344	154	**-1,31290605**	N12:452173	28,583				rDNA	
chrXII	452190	452344	154	**-1,31290605**	N12:452361	15,716				rDNA	
chrXII	452260	452414	154	**-1,354485193**	N12:452361	15,716				rDNA	
chrXII	452293	452400	107	**-1,479635313**	N12:452361	15,716				rDNA	
chrXII	452294	452455	161	**-1,343251014**	N12:452361	15,716				rDNA	
chrXII	452295	452455	160	**-1,395266066**	N12:452361	15,716				rDNA	
chrXII	452299	452455	156	**-1,471744026**	N12:452361	15,716				rDNA	
chrXII	452323	452455	132	**-1,197849248**	N12:452361	15,716				rDNA	
chrXII	452323	452485	162	**-1,287017701**	N12:452361	15,716				rDNA	
chrXII	452323	452486	163	**-1,152817435**	N12:452361	15,716				rDNA	
chrXII	452334	452441	107	**-0,896778421**	N12:452361	15,716				rDNA	
chrXII	452334	452455	121	**-1,044382945**	N12:452361	15,716				rDNA	
chrXII	452334	452485	151	**-1,199898478**	N12:452361	15,716				rDNA	
chrXII	452334	452486	152	**-1,596333429**	N12:452361	15,716				rDNA	
chrXII	452346	452496	150	**-1,169144695**	N12:452361	15,716				rDNA	
chrXII	452423	452584	161	**-1,534443647**	N12:452551	34,395				rDNA	
chrXII	452469	452584	115	**-1,234893602**	N12:452551	34,395				rDNA	
chrXII	452469	452620	151	**-1,51561518**	N12:452551	34,395				rDNA	
chrXII	452469	452621	152	**-1,552052632**	N12:452551	34,395				rDNA	
chrXII	452469	452629	160	**-1,25107865**	N12:452551	34,395				rDNA	
chrXII	452469	452630	161	**-1,470757401**	N12:452551	34,395				rDNA	
chrXII	452491	452621	130	**-1,237878987**	N12:452551	34,395				rDNA	
chrXII	452491	452629	138	**-1,313026015**	N12:452551	34,395				rDNA	
chrXII	452491	452663	172	**-1,450320305**	N12:452551	34,395				rDNA	
chrXII	452492	452584	92	**-1,138468787**	N12:452551	34,395				rDNA	
chrXII	452492	452620	128	**-1,410493358**	N12:452551	34,395				rDNA	
chrXII	452492	452621	129	**-1,428985011**	N12:452551	34,395				rDNA	
chrXII	452492	452629	137	**-1,365106135**	N12:452551	34,395				rDNA	
chrXII	452492	452663	171	**-1,916598169**	N12:452551	34,395				rDNA	
chrXII	452494	452621	127	**-1,234550926**	N12:452551	34,395				rDNA	
chrXII	452525	452695	170	**-1,574892129**	N12:452551	34,395				rDNA	
chrXII	452539	452673	134	**-1,34971257**	N12:452551	34,395				rDNA	
chrXII	452550	452672	122	**-1,405173722**	N12:452551	34,395				rDNA	
chrXII	452550	452702	152	**-1,453196622**	N12:452551	34,395				rDNA	
chrXII	452668	452796	128	**-1,195558592**	N12:452761	20,502				rDNA	
chrXII	452684	452796	112	**-0,684394069**	N12:452761	20,502				rDNA	
chrXII	452684	452835	151	**-1,133040361**	N12:452761	20,502				rDNA	
chrXII	452686	452814	128	**-1,188401963**	N12:452761	20,502				rDNA	
chrXII	452686	452884	198	**-1,778198455**	N12:452761	20,502				rDNA	
chrXII	452709	452864	155	**-1,340921092**	N12:452761	20,502				rDNA	
chrXII	452709	452884	175	**-1,234116436**	N12:452761	20,502				rDNA	
chrXII	452709	452900	191	**-1,333922725**	N12:452761	20,502				rDNA	
chrXII	452723	452814	91	**-0,78772414**	N12:452761	20,502				rDNA	
chrXII	452723	452864	141	**-1,637532571**	N12:452761	20,502				rDNA	
chrXII	452723	452884	161	**-1,267867521**	N12:452761	20,502				rDNA	
chrXII	452723	452900	177	**-1,327310445**	N12:452761	20,502				rDNA	
chrXII	452765	452864	99	**-0,504356379**	N12:452761	20,502				rDNA	
chrXII	452765	452871	106	**-0,900966829**	N12:452761	20,502				rDNA	
chrXII	452765	452884	119	**-0,779531249**	N12:452761	20,502				rDNA	
chrXII	452765	452900	135	**-1,094331164**	N12:452761	20,502				rDNA	
chrXII	452765	452925	160	**-1,509037703**	N12:452761	20,502				rDNA	
chrXII	452765	452925	160	**-1,509037703**	N12:452927	2,887				rDNA	
chrXII	452773	452864	91	**-0,708056173**	N12:452761	20,502				rDNA	
chrXII	452773	452879	106	**-1,09608273**	N12:452761	20,502				rDNA	
chrXII	452773	452884	111	**-0,961006774**	N12:452761	20,502				rDNA	
chrXII	452773	452900	127	**-1,132682414**	N12:452761	20,502				rDNA	
chrXII	452773	452925	152	**-1,370232757**	N12:452761	20,502				rDNA	
chrXII	452773	452925	152	**-1,370232757**	N12:452927	2,887				rDNA	
chrXII	452783	452962	179	**-1,674350294**	N12:452761	20,502				rDNA	
chrXII	452783	452962	179	**-1,674350294**	N12:452927	2,887				rDNA	
chrXII	452788	452923	135	**-1,052630765**	N12:452927	2,887				rDNA	
chrXII	452788	452953	165	**-1,104200658**	N12:452927	2,887				rDNA	
chrXII	452801	452923	122	**-0,664961388**	N12:452927	2,887				rDNA	
chrXII	452801	452953	152	**-1,268040098**	N12:452927	2,887				rDNA	
chrXII	452801	452999	198	**-1,624506722**	N12:452927	2,887				rDNA	
chrXII	452840	452953	113	**-0,691840125**	N12:452927	2,887				rDNA	
chrXII	452840	452999	159	**-1,254730292**	N12:452927	2,887				rDNA	
chrXII	452840	453023	183	**-1,558460647**	N12:452927	2,887				rDNA	
chrXII	452847	452953	106	**-1,031668425**	N12:452927	2,887				rDNA	
chrXII	452847	452996	149	**-1,527444518**	N12:452927	2,887				rDNA	
chrXII	452876	453034	158	**-1,351089933**	N12:452927	2,887				rDNA	
chrXII	452885	453034	149	**-1,017007914**	N12:452927	2,887				rDNA	
chrXII	452893	452999	106	**-0,809381927**	N12:452927	2,887				rDNA	
chrXII	452895	453023	128	**-1,006362814**	N12:452927	2,887				rDNA	
chrXII	452922	453029	107	**-1,421338671**	N12:452927	2,887				rDNA	
chrXII	452922	453031	109	**-1,225115667**	N12:452927	2,887				rDNA	
chrXII	452922	453034	112	**-1,472328186**	N12:452927	2,887				rDNA	
chrXII	452922	453056	134	**-1,441546027**	N12:452927	2,887				rDNA	
chrXII	452922	453056	134	**-1,441546027**	N12:453077	11,314				rDNA	
chrXII	452922	453075	153	**-1,918531986**	N12:452927	2,887				rDNA	
chrXII	452922	453075	153	**-1,918531986**	N12:453077	11,314				rDNA	
chrXII	452926	452997	71	**-0,749**	N12:452927	2,887				rDNA	
chrXII	452926	453033	107	**-0,777986396**	N12:452927	2,887				rDNA	
chrXII	452926	453034	108	**-0,910518129**	N12:452927	2,887				rDNA	
chrXII	452926	453056	130	**-0,940671026**	N12:452927	2,887				rDNA	
chrXII	452926	453056	130	**-0,940671026**	N12:453077	11,314				rDNA	
chrXII	452926	453070	144	**-1,105249305**	N12:452927	2,887				rDNA	
chrXII	452926	453070	144	**-1,105249305**	N12:453077	11,314				rDNA	

CHR	START	END	L(bp)	ΔLknuc	Nuc ID	Fuzziness	Gene ID	Gene body postion or intergene	Terminal	rDNA	Telomeric
chrXII	452926	453075	149	**-1,223425464**	N12:452927	2,887				rDNA	
chrXII	452926	453075	149	**-1,223425464**	N12:453077	11,314				rDNA	
chrXII	452976	453138	162	**-1,590667628**	N12:453077	11,314				rDNA	
chrXII	452976	453163	187	**-1,520120757**	N12:453077	11,314				rDNA	
chrXII	452991	453141	150	**-1,323610662**	N12:453077	11,314				rDNA	
chrXII	453033	453163	130	**-1,183362186**	N12:453077	11,314				rDNA	
chrXII	453033	453187	154	**-1,411350238**	N12:453077	11,314				rDNA	
chrXII	453033	453193	160	**-1,530822309**	N12:453077	11,314				rDNA	
chrXII	453039	453163	124	**-0,840193248**	N12:453077	11,314				rDNA	
chrXII	453039	453187	148	**-1,180033573**	N12:453077	11,314				rDNA	
chrXII	453039	453193	154	**-1,503427977**	N12:453077	11,314				rDNA	
chrXII	453042	453141	99	**-0,975505566**	N12:453077	11,314				rDNA	
chrXII	453042	453212	170	**-1,576007874**	N12:453077	11,314				rDNA	
chrXII	453042	453218	176	**-1,231480914**	N12:453077	11,314				rDNA	
chrXII	453043	453163	120	**-1,025952909**	N12:453077	11,314				rDNA	
chrXII	453043	453187	144	**-1,23166489**	N12:453077	11,314				rDNA	
chrXII	453043	453190	147	**-1,359773731**	N12:453077	11,314				rDNA	
chrXII	453043	453193	150	**-1,227583328**	N12:453077	11,314				rDNA	
chrXII	453091	453199	108	**-0,785979772**	N12:453077	11,314				rDNA	
chrXII	453092	453199	107	**-0,729687003**	N12:453077	11,314				rDNA	
chrXII	453092	453212	120	**-1,156455905**	N12:453077	11,314				rDNA	
chrXII	453092	453236	144	**-1,560901931**	N12:453077	11,314				rDNA	
chrXII	453092	453245	153	**-1,51192762**	N12:453077	11,314				rDNA	
chrXII	453092	453270	178	**-1,520025594**	N12:453077	11,314				rDNA	
chrXII	453092	453282	190	**-2,097441483**	N12:453077	11,314				rDNA	
chrXII	453092	453286	194	**-1,759000832**	N12:453077	11,314				rDNA	
chrXII	453092	453288	196	**-1,60564391**	N12:453077	11,314				rDNA	
chrXII	453094	453236	142	**-1,850329181**	N12:453077	11,314				rDNA	
chrXII	453095	453201	106	**-0,941158569**	N12:453077	11,314				rDNA	
chrXII	453095	453212	117	**-0,679329034**	N12:453077	11,314				rDNA	
chrXII	453095	453218	123	**-0,458069409**	N12:453077	11,314				rDNA	
chrXII	453095	453236	141	**-1,867564354**	N12:453077	11,314				rDNA	
chrXII	453095	453245	150	**-1,124660699**	N12:453077	11,314				rDNA	
chrXII	453095	453270	175	**-1,451932329**	N12:453077	11,314				rDNA	
chrXII	453095	453286	191	**-1,562953822**	N12:453077	11,314				rDNA	
chrXII	453095	453288	193	**-1,561438801**	N12:453077	11,314				rDNA	
chrXII	453099	453236	137	**-0,706369277**	N12:453077	11,314				rDNA	
chrXII	453099	453287	188	**-1,448905869**	N12:453077	11,314				rDNA	
chrXII	453099	453288	189	**-1,529320066**	N12:453077	11,314				rDNA	
chrXII	453100	453238	138	**-1,248023589**	N12:453077	11,314				rDNA	
chrXII	453100	453245	145	**-0,701166354**	N12:453077	11,314				rDNA	
chrXII	453113	453270	157	**-1,432507267**				Overlap <50 bp		rDNA	
chrXII	453113	453286	173	**-1,592096458**				Overlap <50 bp		rDNA	
chrXII	453113	453288	175	**-1,451933519**				Overlap <50 bp		rDNA	
chrXII	453114	453236	122	**-0,914496184**				Overlap <50 bp		rDNA	
chrXII	453114	453269	155	**-1,508105897**				Overlap <50 bp		rDNA	
chrXII	453114	453270	156	**-1,049493545**				Overlap <50 bp		rDNA	
chrXII	453114	453286	172	**-1,388726154**				Overlap <50 bp		rDNA	
chrXII	453114	453287	173	**-1,390645671**				Overlap <50 bp		rDNA	
chrXII	453114	453288	174	**-1,38636794**				Overlap <50 bp		rDNA	
chrXII	453114	453302	188	**-1,522686895**				Overlap <50 bp		rDNA	
chrXII	453114	453309	195	**-1,861987022**				Overlap <50 bp		rDNA	
chrXII	453116	453269	153	**-0,869780898**				Overlap <50 bp		rDNA	
chrXII	453116	453270	154	**-0,843237345**				Overlap <50 bp		rDNA	
chrXII	453116	453288	172	**-1,292091959**				Overlap <50 bp		rDNA	
chrXII	453119	453236	117	**-0,827954707**				Overlap <50 bp		rDNA	
chrXII	453119	453245	126	**-1,068037658**				Overlap <50 bp		rDNA	
chrXII	453119	453269	150	**-1,319742249**				Overlap <50 bp		rDNA	
chrXII	453119	453270	151	**-1,335573212**				Overlap <50 bp		rDNA	
chrXII	453119	453282	163	**-2,472034165**				Overlap <50 bp		rDNA	
chrXII	453119	453286	167	**-1,374914415**				Overlap <50 bp		rDNA	
chrXII	453119	453287	168	**-1,220847453**				Overlap <50 bp		rDNA	
chrXII	453119	453288	169	**-1,306159688**				Overlap <50 bp		rDNA	
chrXII	453119	453302	183	**-1,233563504**				Overlap <50 bp		rDNA	
chrXII	453119	453309	190	**-1,957136229**				Overlap <50 bp		rDNA	
chrXII	453120	453228	108	**-0,489887225**				Overlap <50 bp		rDNA	
chrXII	453121	453270	149	**-1,134219118**				Overlap <50 bp		rDNA	
chrXII	453121	453282	161	**-1,629300245**				Overlap <50 bp		rDNA	
chrXII	453121	453286	165	**-1,446224619**				Overlap <50 bp		rDNA	
chrXII	453121	453288	167	**-1,527854968**				Overlap <50 bp		rDNA	
chrXII	453125	453231	106	**-1,083676986**				Overlap <50 bp		rDNA	
chrXII	453125	453236	111	**-1,300868661**				Overlap <50 bp		rDNA	
chrXII	453125	453270	145	**-1,389673767**				Overlap <50 bp		rDNA	
chrXII	453125	453286	161	**-1,459005225**				Overlap <50 bp		rDNA	
chrXII	453125	453287	162	**-1,23961474**				Overlap <50 bp		rDNA	
chrXII	453125	453288	163	**-1,378966264**				Overlap <50 bp		rDNA	
chrXII	453125	453302	177	**-1,347453643**				Overlap <50 bp		rDNA	
chrXII	453126	453248	122	**-1,594624393**				Overlap <50 bp		rDNA	
chrXII	453126	453270	144	**-1,370868156**				Overlap <50 bp		rDNA	
chrXII	453126	453286	160	**-1,414253349**				Overlap <50 bp		rDNA	
chrXII	453126	453287	161	**-1,334940746**				Overlap <50 bp		rDNA	
chrXII	453126	453288	162	**-1,612408164**				Overlap <50 bp		rDNA	
chrXII	453135	453270	135	**-1,288874124**				Overlap <50 bp		rDNA	
chrXII	453135	453282	147	**-1,328952715**				Overlap <50 bp		rDNA	
chrXII	453135	453286	151	**-1,615071406**				Overlap <50 bp		rDNA	
chrXII	453135	453288	153	**-1,620874875**				Overlap <50 bp		rDNA	
chrXII	453138	453286	148	**-1,274369061**				Overlap <50 bp		rDNA	
chrXII	453138	453288	150	**-1,754737165**				Overlap <50 bp		rDNA	
chrXII	453140	453245	105	**-0,238606443**				Overlap <50 bp		rDNA	
chrXII	453140	453246	106	**-0,915232661**				Overlap <50 bp		rDNA	
chrXII	453140	453251	111	**-1,413838737**				Overlap <50 bp		rDNA	
chrXII	453140	453270	130	**-1,084174987**				Overlap <50 bp		rDNA	
chrXII	453140	453282	142	**-1,478152831**				Overlap <50 bp		rDNA	
chrXII	453140	453286	146	**-1,358675116**				Overlap <50 bp		rDNA	
chrXII	453140	453287	147	**-1,380617363**				Overlap <50 bp		rDNA	
chrXII	453140	453288	148	**-1,194319694**				Overlap <50 bp		rDNA	

CHR	START	END	L(bp)	ΔLknuc	Nuc ID	Fuzziness	Gene ID	Gene body postion or intergene	Terminal	rDNA	Telomeric
chrXII	453140	453302	162	**-1,666411185**				Overlap <50 bp		rDNA	
chrXII	453140	453309	169	**-1,843043158**				Overlap <50 bp		rDNA	
chrXII	453140	453323	183	**-1,408711684**				Overlap <50 bp		rDNA	
chrXII	453141	453236	95	**-0,643867953**				Overlap <50 bp		rDNA	
chrXII	453141	453245	104	**-0,427593631**				Overlap <50 bp		rDNA	
chrXII	453141	453251	110	**-0,931901947**				Overlap <50 bp		rDNA	
chrXII	453141	453269	128	**-0,8723526**				Overlap <50 bp		rDNA	
chrXII	453141	453270	129	**-0,945885167**				Overlap <50 bp		rDNA	
chrXII	453141	453281	140	**-1,27534751**				Overlap <50 bp		rDNA	
chrXII	453141	453282	141	**-1,382524872**				Overlap <50 bp		rDNA	
chrXII	453141	453286	145	**-1,234318114**				Overlap <50 bp		rDNA	
chrXII	453141	453287	146	**-1,398220625**				Overlap <50 bp		rDNA	
chrXII	453141	453288	147	**-1,271435943**				Overlap <50 bp		rDNA	
chrXII	453141	453302	161	**-1,311822625**				Overlap <50 bp		rDNA	
chrXII	453141	453309	168	**-1,371141228**				Overlap <50 bp		rDNA	
chrXII	453141	453323	182	**-1,459025922**				Overlap <50 bp		rDNA	
chrXII	453141	453324	183	**-1,445749047**				Overlap <50 bp		rDNA	
chrXII	453144	453288	144	**-1,869085848**				Overlap <50 bp		rDNA	
chrXII	453150	453286	136	**-1,160093829**				Overlap <50 bp		rDNA	
chrXII	453150	453287	137	**-1,288786282**				Overlap <50 bp		rDNA	
chrXII	453150	453288	138	**-1,284642731**				Overlap <50 bp		rDNA	
chrXII	453150	453295	145	**-1,062988513**				Overlap <50 bp		rDNA	
chrXII	453150	453302	152	**-1,84951621**				Overlap <50 bp		rDNA	
chrXII	453152	453240	88	**-0,593715668**				Overlap <50 bp		rDNA	
chrXII	453152	453270	118	**-0,564489561**				Overlap <50 bp		rDNA	
chrXII	453152	453286	134	**-1,113267061**				Overlap <50 bp		rDNA	
chrXII	453152	453287	135	**-1,210451589**				Overlap <50 bp		rDNA	
chrXII	453152	453288	136	**-1,287494979**				Overlap <50 bp		rDNA	
chrXII	453152	453302	150	**-1,458554267**				Overlap <50 bp		rDNA	
chrXII	453152	453309	157	**-1,403027613**				Overlap <50 bp		rDNA	
chrXII	453153	453286	133	**-1,51487596**				Overlap <50 bp		rDNA	
chrXII	453153	453288	135	**-1,129917945**				Overlap <50 bp		rDNA	
chrXII	453159	453270	111	**-0,647734571**				Overlap <50 bp		rDNA	
chrXII	453159	453286	127	**-1,070865061**				Overlap <50 bp		rDNA	
chrXII	453159	453287	128	**-1,031334415**				Overlap <50 bp		rDNA	
chrXII	453159	453288	129	**-1,025873518**				Overlap <50 bp		rDNA	
chrXII	453159	453302	143	**-1,124678362**				Overlap <50 bp		rDNA	
chrXII	453159	453309	150	**-1,561989909**				Overlap <50 bp		rDNA	
chrXII	453159	453335	176	**-1,688153227**				Overlap <50 bp		rDNA	
chrXII	453159	453342	183	**-1,666339773**				Overlap <50 bp		rDNA	
chrXII	453161	453245	84	**-0,39863806**				Overlap <50 bp		rDNA	
chrXII	453161	453268	107	**-0,854039433**				Overlap <50 bp		rDNA	
chrXII	453161	453270	109	**-0,827805551**				Overlap <50 bp		rDNA	
chrXII	453161	453282	121	**-0,82493292**				Overlap <50 bp		rDNA	
chrXII	453161	453286	125	**-0,942457735**				Overlap <50 bp		rDNA	
chrXII	453161	453288	127	**-1,113344395**				Overlap <50 bp		rDNA	
chrXII	453161	453309	148	**-1,427578397**				Overlap <50 bp		rDNA	
chrXII	453161	453323	162	**-1,105452985**				Overlap <50 bp		rDNA	
chrXII	453161	453324	163	**-1,101547436**				Overlap <50 bp		rDNA	
chrXII	453162	453269	107	**-0,594054974**				Overlap <50 bp		rDNA	
chrXII	453162	453286	124	**-0,95735413**				Overlap <50 bp		rDNA	
chrXII	453162	453288	126	**-1,290809458**				Overlap <50 bp		rDNA	
chrXII	453162	453323	161	**-1,356721729**				Overlap <50 bp		rDNA	
chrXII	453162	453324	162	**-1,342367579**				Overlap <50 bp		rDNA	
chrXII	453165	453286	121	**-1,010079882**				Overlap <50 bp		rDNA	
chrXII	453165	453287	122	**-1,020999093**				Overlap <50 bp		rDNA	
chrXII	453165	453288	123	**-1,001823635**				Overlap <50 bp		rDNA	
chrXII	453165	453302	137	**-1,282037093**				Overlap <50 bp		rDNA	
chrXII	453165	453342	177	**-1,287950972**				Overlap <50 bp		rDNA	
chrXII	453180	453286	106	**-1,400973842**				Overlap <50 bp		rDNA	
chrXII	453180	453288	108	**-0,994346844**				Overlap <50 bp		rDNA	
chrXII	453180	453340	160	**-1,440407218**				Overlap <50 bp		rDNA	
chrXII	453181	453270	89	**-1,337550529**				Overlap <50 bp		rDNA	
chrXII	453181	453282	101	**-1,854233757**				Overlap <50 bp		rDNA	
chrXII	453181	453286	105	**-1,117474245**				Overlap <50 bp		rDNA	
chrXII	453181	453288	107	**-1,192141437**				Overlap <50 bp		rDNA	
chrXII	453181	453323	142	**-1,266947266**				Overlap <50 bp		rDNA	
chrXII	453181	453324	143	**-1,722976**				Overlap <50 bp		rDNA	
chrXII	453286	453432	146	**-0,944000478**	N12:453378	19,858				rDNA	
chrXII	453299	453425	126	**-1,515497291**	N12:453378	19,858				rDNA	
chrXII	453299	453440	141	**-1,067509288**	N12:453378	19,858				rDNA	
chrXII	453299	453460	161	**-1,180011587**	N12:453378	19,858				rDNA	
chrXII	453300	453410	110	**-1,365285834**	N12:453378	19,858				rDNA	
chrXII	453300	453414	114	**-1,499902166**	N12:453378	19,858				rDNA	
chrXII	453300	453440	140	**-1,978581283**	N12:453378	19,858				rDNA	
chrXII	453302	453440	138	**-1,164256023**	N12:453378	19,858				rDNA	
chrXII	453310	453440	130	**-0,817772089**	N12:453378	19,858				rDNA	
chrXII	453310	453460	150	**-1,236884475**	N12:453378	19,858				rDNA	
chrXII	453322	453481	159	**-1,310065467**	N12:453378	19,858				rDNA	
chrXII	453458	453595	137	**-1,633667366**	N12:453535	22,121				rDNA	
chrXII	453458	453610	152	**-1,354015096**	N12:453535	22,121				rDNA	
chrXII	453458	453612	154	**-1,558180698**	N12:453535	22,121				rDNA	
chrXII	453460	453612	152	**-1,423694186**	N12:453535	22,121				rDNA	
chrXII	453487	453595	108	**-1,471679521**	N12:453535	22,121				rDNA	
chrXII	453487	453610	123	**-1,377331296**	N12:453535	22,121				rDNA	
chrXII	453487	453612	125	**-1,11113785**	N12:453535	22,121				rDNA	
chrXII	453500	453652	152	**-1,282975984**	N12:453535	22,121				rDNA	
chrXII	453574	453727	153	**-0,958674505**	N12:453693	25,423				rDNA	
chrXII	453587	453737	150	**-1,664370448**	N12:453693	25,423				rDNA	
chrXII	453606	453774	168	**-1,114323848**	N12:453693	25,423				rDNA	
chrXII	453627	453776	149	**-1,742013422**	N12:453693	25,423				rDNA	
chrXII	453627	453779	152	**-1,069797779**	N12:453693	25,423				rDNA	
chrXII	453657	453814	157	**-0,944340368**	N12:453693	25,423				rDNA	
chrXII	453659	453776	117	**-1,408011648**	N12:453693	25,423				rDNA	
chrXII	453659	453779	120	**-1,157080791**	N12:453693	25,423				rDNA	
chrXII	453734	453853	119	**-1,053841366**	N12:453841	6,364				rDNA	

CHR	START	END	L(bp)	ΔLknuc	Nuc ID	Fuzziness	Gene ID	Gene body postion or intergene	Terminal	rDNA	Telomeric
chrXII	453748	453910	162	**-1,876176937**	N12:453841	6,364				rDNA	
chrXII	453748	453913	165	**-1,367106647**	N12:453841	6,364				rDNA	
chrXII	453748	453919	171	**-1,379956812**	N12:453841	6,364				rDNA	
chrXII	453748	453922	174	**-1,718968275**	N12:453841	6,364				rDNA	
chrXII	453748	453962	214	**-1,731085258**	N12:453841	6,364				rDNA	
chrXII	453752	453853	101	**-1,025030532**	N12:453841	6,364				rDNA	
chrXII	453752	453859	107	**-0,53510933**	N12:453841	6,364				rDNA	
chrXII	453752	453908	156	**-1,522404847**	N12:453841	6,364				rDNA	
chrXII	453752	453911	159	**-1,427007064**	N12:453841	6,364				rDNA	
chrXII	453752	453914	162	**-1,341100402**	N12:453841	6,364				rDNA	
chrXII	453752	453916	164	**-1,388930983**	N12:453841	6,364				rDNA	
chrXII	453752	453922	170	**-1,387833823**	N12:453841	6,364				rDNA	
chrXII	453752	453924	172	**-1,349546569**	N12:453841	6,364				rDNA	
chrXII	453753	453913	160	**-1,645741933**	N12:453841	6,364				rDNA	
chrXII	453753	453919	166	**-1,134946401**	N12:453841	6,364				rDNA	
chrXII	453753	453922	169	**-1,413770668**	N12:453841	6,364				rDNA	
chrXII	453754	453910	156	**-1,650313296**	N12:453841	6,364				rDNA	
chrXII	453754	453924	170	**-1,673342306**	N12:453841	6,364				rDNA	
chrXII	453774	453910	136	**-1,12920419**	N12:453841	6,364				rDNA	
chrXII	453774	453913	139	**-1,220142143**	N12:453841	6,364				rDNA	
chrXII	453774	453914	140	**-1,542051489**	N12:453841	6,364				rDNA	
chrXII	453774	453916	142	**-1,297713418**	N12:453841	6,364				rDNA	
chrXII	453774	453919	145	**-1,139587321**	N12:453841	6,364				rDNA	
chrXII	453774	453922	148	**-1,994673623**	N12:453841	6,364				rDNA	
chrXII	453774	453925	151	**-1,408783162**	N12:453841	6,364				rDNA	
chrXII	453774	453962	188	**-1,316041416**	N12:453841	6,364				rDNA	
chrXII	453775	453908	133	**-1,303308731**	N12:453841	6,364				rDNA	
chrXII	453775	453911	136	**-1,169773296**	N12:453841	6,364				rDNA	
chrXII	453775	453914	139	**-1,05645459**	N12:453841	6,364				rDNA	
chrXII	453775	453916	141	**-1,437427921**	N12:453841	6,364				rDNA	
chrXII	453775	453919	144	**-2,178844761**	N12:453841	6,364				rDNA	
chrXII	453775	453922	147	**-1,501005416**	N12:453841	6,364				rDNA	
chrXII	453781	453908	127	**-0,872289474**	N12:453841	6,364				rDNA	
chrXII	453781	453911	130	**-1,000628502**	N12:453841	6,364				rDNA	
chrXII	453781	453914	133	**-1,18210055**	N12:453841	6,364				rDNA	
chrXII	453781	453916	135	**-1,087186833**	N12:453841	6,364				rDNA	
chrXII	453781	453922	141	**-1,345406678**	N12:453841	6,364				rDNA	
chrXII	453782	453908	126	**-0,961066656**	N12:453841	6,364				rDNA	
chrXII	453782	453911	129	**-1,312732098**	N12:453841	6,364				rDNA	
chrXII	453782	453913	131	**-0,838520311**	N12:453841	6,364				rDNA	
chrXII	453782	453914	132	**-1,090012453**	N12:453841	6,364				rDNA	
chrXII	453782	453919	137	**-1,217429412**	N12:453841	6,364				rDNA	
chrXII	453782	453922	140	**-1,146997168**	N12:453841	6,364				rDNA	
chrXII	453782	453925	143	**-1,132929801**	N12:453841	6,364				rDNA	
chrXII	453782	453929	147	**-1,310697855**	N12:453841	6,364				rDNA	
chrXII	453782	453937	155	**-1,54162973**	N12:453841	6,364				rDNA	
chrXII	453782	453962	180	**-1,462803965**	N12:453841	6,364				rDNA	
chrXII	453783	453911	128	**-1,026686018**	N12:453841	6,364				rDNA	
chrXII	453783	453913	130	**-1,024370582**	N12:453841	6,364				rDNA	
chrXII	453783	453914	131	**-1,85582847**	N12:453841	6,364				rDNA	
chrXII	453812	453913	101	**-0,672409103**	N12:453841	6,364				rDNA	
chrXII	453812	453919	107	**-0,460245968**	N12:453841	6,364				rDNA	
chrXII	453812	453962	150	**-1,147287853**	N12:453841	6,364				rDNA	
chrXII	453812	454001	189	**-1,473452888**	N12:453841	6,364				rDNA	
chrXII	453812	454001	189	**-1,473452888**	N12:454007	8,505				rDNA	
chrXII	453851	453958	107	**-0,559635716**	N12:453841	6,364				rDNA	
chrXII	453851	453961	110	**-1,140559031**	N12:453841	6,364				rDNA	
chrXII	453851	453962	111	**-0,65850533**	N12:453841	6,364				rDNA	
chrXII	453851	454001	150	**-1,099442387**	N12:453841	6,364				rDNA	
chrXII	453851	454001	150	**-1,099442387**	N12:454007	8,505				rDNA	
chrXII	453909	454072	163	**-1,500128358**	N12:454007	8,505				rDNA	
chrXII	453918	454080	162	**-1,234988308**	N12:454007	8,505				rDNA	
chrXII	453922	454079	157	**-1,346571791**	N12:454007	8,505				rDNA	
chrXII	453929	454079	150	**-1,833161087**	N12:454007	8,505				rDNA	
chrXII	453929	454097	168	**-1,587762446**	N12:454007	8,505				rDNA	
chrXII	453929	454108	179	**-1,527671331**	N12:454007	8,505				rDNA	
chrXII	453959	454097	138	**-1,202488253**	N12:454007	8,505				rDNA	
chrXII	453959	454108	149	**-1,176974072**	N12:454007	8,505				rDNA	
chrXII	453959	454112	153	**-1,084044682**	N12:454007	8,505				rDNA	
chrXII	453960	454116	156	**-1,212103047**	N12:454007	8,505				rDNA	
chrXII	453964	454097	133	**-1,343610196**	N12:454007	8,505				rDNA	
chrXII	453964	454108	144	**-1,112521418**	N12:454007	8,505				rDNA	
chrXII	453971	454123	152	**-1,280386921**	N12:454007	8,505				rDNA	
chrXII	454059	454216	157	**-1,397576609**	N12:454198	24,269				rDNA	
chrXII	454111	454268	157	**-1,531530374**	N12:454198	24,269				rDNA	
chrXII	454120	454267	147	**-1,590901998**	N12:454198	24,269				rDNA	
chrXII	454120	454277	157	**-1,424361653**	N12:454198	24,269				rDNA	
chrXII	454124	454277	153	**-1,399493395**	N12:454198	24,269				rDNA	
chrXII	454131	454277	146	**-1,425230991**	N12:454198	24,269				rDNA	
chrXII	454141	454277	136	**-1,280053521**	N12:454198	24,269				rDNA	
chrXII	454196	454346	150	**-0,881708481**	N12:454367	64,01	-1:YLR154W-C;	-1		rDNA	
chrXII	454196	454346	150	**-0,881708481**	N12:454198	24,269				rDNA	
chrXII	454221	454392	171	**-1,296471933**	N12:454367	64,01	-1:YLR154W-C;	-1		rDNA	
chrXII	454221	454392	171	**-1,296471933**	N12:454198	24,269				rDNA	
chrXII	454244	454392	148	**-1,56618022**	N12:454367	64,01	-1:YLR154W-C;	-1		rDNA	
chrXII	454244	454402	158	**-1,120982349**	N12:454367	64,01	-1:YLR154W-C;	-1		rDNA	
chrXII	454248	454374	126	**-1,078312309**	N12:454367	64,01	-1:YLR154W-C;	-1		rDNA	
chrXII	454248	454392	144	**-1,398153008**	N12:454367	64,01	-1:YLR154W-C;	-1		rDNA	
chrXII	454248	454398	150	**-1,24796621**	N12:454367	64,01	-1:YLR154W-C;	-1		rDNA	
chrXII	454273	454436	163	**-1,245692034**	N12:454367	64,01	-1:YLR154W-C;	-1		rDNA	
chrXII	454277	454398	121	**-0,634273441**	N12:454367	64,01	-1:YLR154W-C;	-1		rDNA	
chrXII	454277	454402	125	**-1,422699207**	N12:454367	64,01	-1:YLR154W-C;	-1		rDNA	
chrXII	454277	454438	161	**-1,148371885**	N12:454367	64,01	-1:YLR154W-C;	-1		rDNA	
chrXII	454277	454446	169	**-1,586449775**	N12:454367	64,01	-1:YLR154W-C;	-1		rDNA	
chrXII	454398	454551	153	**-1,422643255**				Overlap <50 bp		rDNA	
chrXII	454398	454552	154	**-1,181586119**				Overlap <50 bp		rDNA	

CHR	START	END	L(bp)	ΔLknuc	Nuc ID	Fuzziness	Gene ID	Gene body postion or intergene	Terminal	rDNA	Telomeric
chrXII	454398	454555	157	-1,215492537				Overlap <50 bp		rDNA	
chrXII	454398	454567	169	-1,137203077				Overlap <50 bp		rDNA	
chrXII	454398	454573	175	-1,463178836				Overlap <50 bp		rDNA	
chrXII	454398	454575	177	-1,051247577				Overlap <50 bp		rDNA	
chrXII	454404	454552	148	-1,144430038				Overlap <50 bp		rDNA	
chrXII	454404	454555	151	-1,100057884				Overlap <50 bp		rDNA	
chrXII	454422	454552	130	-0,920526742				Overlap <50 bp		rDNA	
chrXII	454422	454555	133	-0,79819432				Overlap <50 bp		rDNA	
chrXII	454422	454567	145	-1,13871689				Overlap <50 bp		rDNA	
chrXII	454422	454573	151	-1,161154563				Overlap <50 bp		rDNA	
chrXII	454422	454575	153	-1,210931753				Overlap <50 bp		rDNA	
chrXII	454521	454678	157	-1,426562991	N12:454654	30,199	+1:YLR154W-C;	1		rDNA	
chrXII	454553	454712	159	-1,231917398	N12:454654	30,199	+1:YLR154W-C;	1		rDNA	
chrXII	454553	454715	162	-1,197068638	N12:454654	30,199	+1:YLR154W-C;	1		rDNA	
chrXII	454553	454733	180	-1,632580235	N12:454654	30,199	+1:YLR154W-C;	1		rDNA	
chrXII	454555	454703	148	-1,175552155	N12:454654	30,199	+1:YLR154W-C;	1		rDNA	
chrXII	454560	454678	118	-0,919035871	N12:454654	30,199	+1:YLR154W-C;	1		rDNA	
chrXII	454560	454712	152	-1,438878957	N12:454654	30,199	+1:YLR154W-C;	1		rDNA	
chrXII	454560	454715	155	-1,344898696	N12:454654	30,199	+1:YLR154W-C;	1		rDNA	
chrXII	454560	454724	164	-1,068596116	N12:454654	30,199	+1:YLR154W-C;	1		rDNA	
chrXII	454560	454733	173	-1,37305148	N12:454654	30,199	+1:YLR154W-C;	1		rDNA	
chrXII	454562	454712	150	-1,089673703	N12:454654	30,199	+1:YLR154W-C;	1		rDNA	
chrXII	454570	454712	142	-1,011819202	N12:454654	30,199	+1:YLR154W-C;	1		rDNA	
chrXII	454570	454715	145	-1,333321638	N12:454654	30,199	+1:YLR154W-C;	1		rDNA	
chrXII	454570	454724	154	-1,173953046	N12:454654	30,199	+1:YLR154W-C;	1		rDNA	
chrXII	454570	454733	163	-1,406490175	N12:454654	30,199	+1:YLR154W-C;	1		rDNA	
chrXII	454576	454712	136	-1,476847827	N12:454654	30,199	+1:YLR154W-C;	1		rDNA	
chrXII	454576	454715	139	-1,291344565	N12:454654	30,199	+1:YLR154W-C;	1		rDNA	
chrXII	454576	454724	148	-1,37148199	N12:454654	30,199	+1:YLR154W-C;	1		rDNA	
chrXII	454576	454733	157	-1,412750071	N12:454654	30,199	+1:YLR154W-C;	1		rDNA	
chrXII	454576	454772	196	-1,493923074	N12:454654	30,199	+1:YLR154W-C;	1		rDNA	
chrXII	454587	454746	159	-1,395125026	N12:454654	30,199	+1:YLR154W-C;	1		rDNA	
chrXII	454592	454742	150	-1,282190339	N12:454654	30,199	+1:YLR154W-C;	1		rDNA	
chrXII	454626	454724	98	-0,356137251	N12:454654	30,199	+1:YLR154W-C;	1		rDNA	
chrXII	454626	454733	107	-0,575614643	N12:454654	30,199	+1:YLR154W-C;	1		rDNA	
chrXII	454626	454772	146	-1,167294426	N12:454654	30,199	+1:YLR154W-C;	1		rDNA	
chrXII	454649	454741	92	-0,618965613	N12:454654	30,199	+1:YLR154W-C;	1		rDNA	
chrXII	454677	454828	151	-1,429219436	N12:454654	30,199	+1:YLR154W-C;	1		rDNA	
chrXII	454677	454828	151	-1,429219436	N12:454834	31,88	+2:YLR154W-C;	2		rDNA	
chrXII	454723	454854	131	-1,117114606	N12:454834	31,88	+2:YLR154W-C;	2		rDNA	
chrXII	454725	454863	138	-1,082110205	N12:454834	31,88	+2:YLR154W-C;	2		rDNA	
chrXII	454725	454886	161	-1,297765154	N12:454834	31,88	+2:YLR154W-C;	2		rDNA	
chrXII	454733	454863	130	-1,199206879	N12:454834	31,88	+2:YLR154W-C;	2		rDNA	
chrXII	454733	454886	153	-1,673674328	N12:454834	31,88	+2:YLR154W-C;	2		rDNA	
chrXII	454741	454896	155	-1,505428713	N12:454834	31,88	+2:YLR154W-C;	2		rDNA	
chrXII	454747	454853	106	-0,443622589	N12:454834	31,88	+2:YLR154W-C;	2		rDNA	
chrXII	454747	454886	139	-1,077833095	N12:454834	31,88	+2:YLR154W-C;	2		rDNA	
chrXII	454825	454972	147	-1,680443806	N12:454834	31,88	+2:YLR154W-C;	2		rDNA	
chrXII	454825	454972	147	-1,680443806	N12:454994	15,556	+3:YLR154W-C;	3		rDNA	
chrXII	454837	454991	154	-0,958204752	N12:454834	31,88	+2:YLR154W-C;	2		rDNA	
chrXII	454837	454991	154	-0,958204752	N12:454994	15,556	+3:YLR154W-C;	3		rDNA	
chrXII	454864	455021	157	-1,054009343	N12:454994	15,556	+3:YLR154W-C;	3		rDNA	
chrXII	454876	455023	147	-1,065648787	N12:454994	15,556	+3:YLR154W-C;	3		rDNA	
chrXII	454876	455029	153	-1,616796046	N12:454994	15,556	+3:YLR154W-C;	3		rDNA	
chrXII	454901	455029	128	-1,451662519	N12:454994	15,556	+3:YLR154W-C;	3		rDNA	
chrXII	454901	455074	173	-1,422004281	N12:454994	15,556	+3:YLR154W-C;	3		rDNA	
chrXII	454920	455087	167	-1,273990201	N12:454994	15,556	+3:YLR154W-C;	3		rDNA	
chrXII	454932	455073	141	-0,978355727	N12:454994	15,556	+3:YLR154W-C;	3		rDNA	
chrXII	454933	455023	90	-0,847714769	N12:454994	15,556	+3:YLR154W-C;	3		rDNA	
chrXII	454933	455024	91	-1,265537125	N12:454994	15,556	+3:YLR154W-C;	3		rDNA	
chrXII	454933	455025	92	-0,69161037	N12:454994	15,556	+3:YLR154W-C;	3		rDNA	
chrXII	454933	455027	94	-0,994638715	N12:454994	15,556	+3:YLR154W-C;	3		rDNA	
chrXII	454933	455029	96	-1,181792976	N12:454994	15,556	+3:YLR154W-C;	3		rDNA	
chrXII	454933	455073	140	-0,830362198	N12:454994	15,556	+3:YLR154W-C;	3		rDNA	
chrXII	454933	455074	141	-1,326709506	N12:454994	15,556	+3:YLR154W-C;	3		rDNA	
chrXII	454933	455076	143	-1,145400143	N12:454994	15,556	+3:YLR154W-C;	3		rDNA	
chrXII	454933	455132	199	-2,180476381	N12:454994	15,556	+3:YLR154W-C;	3		rDNA	
chrXII	454964	455132	168	-1,313679037	N12:454994	15,556	+3:YLR154W-C;	3		rDNA	
chrXII	454995	455164	169	-1,697455115	N12:454994	15,556	+3:YLR154W-C;	3		rDNA	
chrXII	455000	455132	132	-1,389881427	N12:454994	15,556	+3:YLR154W-C;	3		rDNA	
chrXII	455015	455166	151	-1,215953421	N12:454994	15,556	+3:YLR154W-C;	3		rDNA	
chrXII	455058	455208	150	-1,439311083				Overlap <50 bp		rDNA	
chrXII	455068	455223	155	-1,281133884				Overlap <50 bp		rDNA	
chrXII	455095	455269	174	-1,447474451				Overlap <50 bp		rDNA	
chrXII	455096	455223	127	-1,189315108				Overlap <50 bp		rDNA	
chrXII	455096	455240	144	-1,276225788				Overlap <50 bp		rDNA	
chrXII	455096	455243	147	-1,257096994				Overlap <50 bp		rDNA	
chrXII	455096	455245	149	-1,199283538				Overlap <50 bp		rDNA	
chrXII	455096	455249	153	-1,211227729				Overlap <50 bp		rDNA	
chrXII	455097	455240	143	-1,286935047				Overlap <50 bp		rDNA	
chrXII	455097	455243	146	-1,142746925				Overlap <50 bp		rDNA	
chrXII	455098	455243	145	-1,326139823				Overlap <50 bp		rDNA	
chrXII	455098	455249	151	-1,429443897				Overlap <50 bp		rDNA	
chrXII	455129	455293	164	-1,279321555	N12:455294	19,975	+5*:YLR154W-C;	5	TERM	rDNA	
chrXII	455138	455289	151	-1,363275748	N12:455294	19,975	+5*:YLR154W-C;	5	TERM	rDNA	
chrXII	455182	455338	156	-1,456895455	N12:455294	19,975	+5*:YLR154W-C;	5	TERM	rDNA	
chrXII	455217	455366	149	-1,371046834	N12:455294	19,975	+5*:YLR154W-C;	5	TERM	rDNA	
chrXII	455221	455395	174	-1,957052919	N12:455294	19,975	+5*:YLR154W-C;	5	TERM	rDNA	
chrXII	455247	455381	134	-1,223894193	N12:455294	19,975	+5*:YLR154W-C;	5	TERM	rDNA	
chrXII	455254	455401	147	-1,265113843	N12:455294	19,975	+5*:YLR154W-C;	5	TERM	rDNA	
chrXII	455255	455400	145	-1,267794559	N12:455294	19,975	+5*:YLR154W-C;	5	TERM	rDNA	
chrXII	455278	455423	145	-1,573385181	N12:455294	19,975	+5*:YLR154W-C;	5	TERM	rDNA	
chrXII	455297	455447	150	-1,425154852	N12:455294	19,975	+5*:YLR154W-C;	5	TERM	rDNA	
chrXII	455367	455512	145	-1,111708771	N12:455488	36,77				rDNA	
chrXII	455367	455531	164	-1,555598298	N12:455488	36,77				rDNA	
chrXII	455367	455549	182	-1,359658714	N12:455488	36,77				rDNA	

CHR	START	END	L(bp)	ΔLknuc	Nuc ID	Fuzziness	Gene ID	Gene body postion or intergene	Terminal	rDNA	Telomeric
chrXII	455377	455512	135	**-0,913926326**	N12:455488	36,77				rDNA	
chrXII	455377	455531	154	**-1,005930195**	N12:455488	36,77				rDNA	
chrXII	455377	455549	172	**-1,208723702**	N12:455488	36,77				rDNA	
chrXII	455397	455503	106	**-0,676561875**	N12:455488	36,77				rDNA	
chrXII	455397	455512	115	**-0,408277799**	N12:455488	36,77				rDNA	
chrXII	455397	455531	134	**-1,175529786**	N12:455488	36,77				rDNA	
chrXII	455397	455549	152	**-1,294722163**	N12:455488	36,77				rDNA	
chrXII	455397	455568	171	**-1,581457993**	N12:455488	36,77				rDNA	
chrXII	455410	455512	102	**-0,461780418**	N12:455488	36,77				rDNA	
chrXII	455410	455549	139	**-1,495215213**	N12:455488	36,77				rDNA	
chrXII	455410	455560	150	**-0,677159378**	N12:455488	36,77				rDNA	
chrXII	455410	455568	158	**-1,158707772**	N12:455488	36,77				rDNA	
chrXII	455410	455569	159	**-1,281606414**	N12:455488	36,77				rDNA	
chrXII	455416	455571	155	**-1,607926045**	N12:455488	36,77				rDNA	
chrXII	455422	455569	147	**-1,062262769**	N12:455488	36,77				rDNA	
chrXII	455591	455751	160	**-1,645431743**	N12:455656	22,627				rDNA	
chrXII	455701	455870	169	**-1,595963139**	N12:455829	32,808				rDNA	
chrXII	455747	455895	148	**-1,087612301**	N12:455829	32,808				rDNA	
chrXII	455749	455889	140	**-1,075128679**	N12:455829	32,808				rDNA	
chrXII	455783	455958	175	**-1,704204164**	N12:455829	32,808				rDNA	
chrXII	455869	456021	152	**-1,377701939**	N12:456042	22,48				rDNA	
chrXII	455903	456067	164	**-1,389719575**	N12:456042	22,48				rDNA	
chrXII	455918	456039	121	**-1,280252299**	N12:456042	22,48				rDNA	
chrXII	455926	456086	160	**-1,364669614**	N12:456042	22,48				rDNA	
chrXII	456022	456173	151	**-1,150091008**	N12:456042	22,48				rDNA	
chrXII	456022	456205	183	**-1,527096809**	N12:456042	22,48				rDNA	
chrXII	456022	456205	183	**-1,527096809**	N12:456220	28				rDNA	
chrXII	456022	456211	189	**-1,283453682**	N12:456042	22,48				rDNA	
chrXII	456022	456211	189	**-1,283453682**	N12:456220	28				rDNA	
chrXII	456022	456220	198	**-1,519940886**	N12:456042	22,48				rDNA	
chrXII	456022	456220	198	**-1,519940886**	N12:456220	28				rDNA	
chrXII	456025	456173	148	**-0,981641815**	N12:456042	22,48				rDNA	
chrXII	456025	456182	157	**-1,121232648**	N12:456042	22,48				rDNA	
chrXII	456042	456173	131	**-0,562287906**	N12:456042	22,48				rDNA	
chrXII	456045	456173	128	**-0,893016349**	N12:456042	22,48				rDNA	
chrXII	456045	456175	130	**-1,027711176**	N12:456042	22,48				rDNA	
chrXII	456045	456205	160	**-1,336056619**	N12:456042	22,48				rDNA	
chrXII	456045	456205	160	**-1,336056619**	N12:456220	28				rDNA	
chrXII	456056	456173	117	**-0,738642625**	N12:456042	22,48				rDNA	
chrXII	456056	456175	119	**-0,781885292**	N12:456042	22,48				rDNA	
chrXII	456056	456205	149	**-1,261128826**	N12:456042	22,48				rDNA	
chrXII	456056	456205	149	**-1,261128826**	N12:456220	28				rDNA	
chrXII	456056	456211	155	**-1,477494129**	N12:456042	22,48				rDNA	
chrXII	456056	456211	155	**-1,477494129**	N12:456220	28				rDNA	
chrXII	456056	456220	164	**-1,410412803**	N12:456042	22,48				rDNA	
chrXII	456056	456220	164	**-1,410412803**	N12:456220	28				rDNA	
chrXII	456063	456215	152	**-1,046412445**	N12:456042	22,48				rDNA	
chrXII	456063	456215	152	**-1,046412445**	N12:456220	28				rDNA	
chrXII	456064	456214	150	**-1,125587004**	N12:456042	22,48				rDNA	
chrXII	456064	456214	150	**-1,125587004**	N12:456220	28				rDNA	
chrXII	456066	456173	107	**-0,415858579**				Overlap <50 bp		rDNA	
chrXII	456067	456205	138	**-1,137025389**	N12:456220	28				rDNA	
chrXII	456067	456211	144	**-0,9710934**	N12:456220	28				rDNA	
chrXII	456067	456215	148	**-1,213205785**	N12:456220	28				rDNA	
chrXII	456067	456220	153	**-1,081665104**	N12:456220	28				rDNA	
chrXII	456067	456266	199	**-1,719861336**	N12:456220	28				rDNA	
chrXII	456080	456173	93	**-0,480139128**				Overlap <50 bp		rDNA	
chrXII	456080	456205	125	**-0,634883535**	N12:456220	28				rDNA	
chrXII	456080	456211	131	**-0,72285898**	N12:456220	28				rDNA	
chrXII	456080	456220	140	**-0,956255938**	N12:456220	28				rDNA	
chrXII	456084	456215	131	**-1,138923383**	N12:456220	28				rDNA	
chrXII	456084	456243	159	**-1,309482124**	N12:456220	28				rDNA	
chrXII	456084	456266	182	**-1,472844182**	N12:456220	28				rDNA	
chrXII	456115	456215	100	**-0,693989257**	N12:456220	28				rDNA	
chrXII	456115	456243	128	**-1,215431524**	N12:456220	28				rDNA	
chrXII	456115	456266	151	**-1,14951293**	N12:456220	28				rDNA	
chrXII	456115	456281	166	**-1,497622718**	N12:456220	28				rDNA	
chrXII	456120	456290	170	**-1,281636057**	N12:456220	28				rDNA	
chrXII	456124	456215	91	**-0,785201802**	N12:456220	28				rDNA	
chrXII	456124	456266	142	**-1,279910624**	N12:456220	28				rDNA	
chrXII	456124	456281	157	**-1,429016872**	N12:456220	28				rDNA	
chrXII	456124	456333	209	**-1,632864062**	N12:456220	28				rDNA	
chrXII	456181	456266	85	**-0,590663226**	N12:456220	28				rDNA	
chrXII	456181	456281	100	**-0,729809544**	N12:456220	28				rDNA	
chrXII	456181	456287	106	**-1,202175501**	N12:456220	28				rDNA	
chrXII	456181	456306	125	**-0,813543047**	N12:456220	28				rDNA	
chrXII	456181	456310	129	**-0,96834544**	N12:456220	28				rDNA	
chrXII	456181	456329	148	**-1,34981526**	N12:456220	28				rDNA	
chrXII	456181	456332	151	**-1,840742954**	N12:456220	28				rDNA	
chrXII	456181	456333	152	**-1,297586545**	N12:456220	28				rDNA	
chrXII	456181	456336	155	**-1,44791953**	N12:456220	28				rDNA	
chrXII	456181	456349	168	**-1,076720451**	N12:456220	28				rDNA	
chrXII	456181	456387	206	**-1,413400921**	N12:456220	28				rDNA	
chrXII	456186	456333	147	**-1,627256815**	N12:456220	28				rDNA	
chrXII	456187	456333	146	**-1,59771293**	N12:456220	28				rDNA	
chrXII	456187	456349	162	**-1,316137336**	N12:456220	28				rDNA	
chrXII	456194	456333	139	**-1,25889814**	N12:456220	28				rDNA	
chrXII	456194	456336	142	**-1,013500075**	N12:456220	28				rDNA	
chrXII	456213	456319	106	**-0,969153116**	N12:456220	28				rDNA	
chrXII	456213	456320	107	**-0,815628147**	N12:456220	28				rDNA	
chrXII	456213	456321	108	**-0,984425382**	N12:456220	28				rDNA	
chrXII	456213	456333	120	**-1,147941248**	N12:456220	28				rDNA	
chrXII	456213	456336	123	**-1,009917497**	N12:456220	28				rDNA	
chrXII	456213	456349	136	**-1,569253966**	N12:456220	28				rDNA	
chrXII	456213	456352	139	**-1,254217269**	N12:456220	28				rDNA	
chrXII	456213	456387	174	**-1,309745443**	N12:456220	28				rDNA	

CHR	START	END	L(bp)	ΔLknuc	Nuc ID	Fuzziness	Gene ID	Gene body postion or intergene	Terminal	rDNA	Telomeric
chrXII	456213	456419	206	**-1,7028479**	N12:456220	28				rDNA	
chrXII	456213	456419	206	**-1,7028479**	N12:456421	19,858				rDNA	
chrXII	456221	456386	165	**-1,085235348**	N12:456220	28				rDNA	
chrXII	456240	456333	93	**-0,57545793**	N12:456220	28				rDNA	
chrXII	456240	456386	146	**-0,987663683**	N12:456220	28				rDNA	
chrXII	456240	456387	147	**-1,181208107**	N12:456220	28				rDNA	
chrXII	456240	456419	179	**-1,388089483**	N12:456220	28				rDNA	
chrXII	456240	456419	179	**-1,388089483**	N12:456421	19,858				rDNA	
chrXII	456240	456447	207	**-1,439992588**	N12:456220	28				rDNA	
chrXII	456240	456447	207	**-1,439992588**	N12:456421	19,858				rDNA	
chrXII	456288	456387	99	**-0,589925161**				Overlap <50 bp		rDNA	
chrXII	456288	456395	107	**-1,355476424**				Overlap <50 bp		rDNA	
chrXII	456288	456419	131	**-1,127127751**	N12:456421	19,858				rDNA	
chrXII	456288	456447	159	**-1,296588691**	N12:456421	19,858				rDNA	
chrXII	456288	456457	169	**-1,368998002**	N12:456421	19,858				rDNA	
chrXII	456288	456471	183	**-1,902593388**	N12:456421	19,858				rDNA	
chrXII	456290	456419	129	**-1,235364681**	N12:456421	19,858				rDNA	
chrXII	456290	456447	157	**-1,663206413**	N12:456421	19,858				rDNA	
chrXII	456290	456471	181	**-1,776796652**	N12:456421	19,858				rDNA	
chrXII	456298	456447	149	**-1,193647556**	N12:456421	19,858				rDNA	
chrXII	456310	456417	107	**-0,36959189**	N12:456421	19,858				rDNA	
chrXII	456310	456419	109	**-0,70786376**	N12:456421	19,858				rDNA	
chrXII	456310	456447	137	**-1,347510747**	N12:456421	19,858				rDNA	
chrXII	456310	456471	161	**-1,354361979**	N12:456421	19,858				rDNA	
chrXII	456361	456447	86	**-1,328979226**	N12:456421	19,858				rDNA	
chrXII	456361	456466	105	**-1,716721808**	N12:456421	19,858				rDNA	
chrXII	456361	456468	107	**-1,522622032**	N12:456421	19,858				rDNA	
chrXII	456361	456470	109	**-1,421743856**	N12:456421	19,858				rDNA	
chrXII	456361	456471	110	**-1,53194537**	N12:456421	19,858				rDNA	
chrXII	456388	456471	83	**-1,076338967**	N12:456421	19,858				rDNA	
chrXII	456388	456551	163	**-1,271009504**	N12:456421	19,858				rDNA	
chrXII	456388	456551	163	**-1,271009504**	N12:456568	7,071				rDNA	
chrXII	456444	456594	150	**-1,137932227**	N12:456421	19,858				rDNA	
chrXII	456444	456594	150	**-1,137932227**	N12:456568	7,071				rDNA	
chrXII	456444	456595	151	**-1,404318252**	N12:456421	19,858				rDNA	
chrXII	456444	456595	151	**-1,404318252**	N12:456568	7,071				rDNA	
chrXII	456444	456619	175	**-1,667758771**	N12:456421	19,858				rDNA	
chrXII	456444	456619	175	**-1,667758771**	N12:456568	7,071				rDNA	
chrXII	456445	456595	150	**-1,618067859**	N12:456568	7,071				rDNA	
chrXII	456447	456594	147	**-1,282503292**	N12:456568	7,071				rDNA	
chrXII	456447	456595	148	**-1,564730455**	N12:456568	7,071				rDNA	
chrXII	456447	456608	161	**-1,845538213**	N12:456568	7,071				rDNA	
chrXII	456447	456619	172	**-1,602807876**	N12:456568	7,071				rDNA	
chrXII	456479	456585	106	**-0,814204026**	N12:456568	7,071				rDNA	
chrXII	456479	456594	115	**-0,736398515**	N12:456568	7,071				rDNA	
chrXII	456479	456608	129	**-0,934613757**	N12:456568	7,071				rDNA	
chrXII	456479	456609	130	**-1,110704052**	N12:456568	7,071				rDNA	
chrXII	456479	456653	174	**-1,273201422**	N12:456568	7,071				rDNA	
chrXII	456483	456594	111	**-1,171021328**	N12:456568	7,071				rDNA	
chrXII	456483	456608	125	**-1,114631272**	N12:456568	7,071				rDNA	
chrXII	456483	456609	126	**-1,131675499**	N12:456568	7,071				rDNA	
chrXII	456493	456599	106	**-0,569145552**	N12:456568	7,071				rDNA	
chrXII	456493	456608	115	**-0,727160737**	N12:456568	7,071				rDNA	
chrXII	456493	456653	160	**-1,482686853**	N12:456568	7,071				rDNA	
chrXII	456508	456594	86	**-0,633894362**	N12:456568	7,071				rDNA	
chrXII	456508	456608	100	**-0,592340289**	N12:456568	7,071				rDNA	
chrXII	456508	456642	134	**-1,281826631**	N12:456568	7,071				rDNA	
chrXII	456508	456653	145	**-1,14597173**	N12:456568	7,071				rDNA	
chrXII	456508	456670	162	**-1,267302287**	N12:456568	7,071				rDNA	
chrXII	456524	456695	171	**-1,609925187**	N12:456568	7,071				rDNA	
chrXII	456587	456738	151	**-1,770478345**	N12:456568	7,071				rDNA	
chrXII	456587	456738	151	**-1,770478345**	N12:456730	37,899				rDNA	
chrXII	456588	456751	163	**-1,017805662**	N12:456568	7,071				rDNA	
chrXII	456588	456751	163	**-1,017805662**	N12:456730	37,899				rDNA	
chrXII	456594	456751	157	**-1,541495057**	N12:456730	37,899				rDNA	
chrXII	456616	456772	156	**-1,132985005**	N12:456730	37,899				rDNA	
chrXII	456634	456751	117	**-0,78510286**	N12:456730	37,899				rDNA	
chrXII	456634	456762	128	**-1,123108749**	N12:456730	37,899				rDNA	
chrXII	456634	456781	147	**-1,300825975**	N12:456730	37,899				rDNA	
chrXII	456649	456803	154	**-1,366873488**	N12:456730	37,899				rDNA	
chrXII	456653	456762	109	**-0,546662435**	N12:456730	37,899				rDNA	
chrXII	456653	456803	150	**-0,965012152**	N12:456730	37,899				rDNA	
chrXII	456733	456869	136	**-1,137813736**	N12:456730	37,899				rDNA	
chrXII	456733	456869	136	**-1,137813736**	N12:456891	32,655				rDNA	
chrXII	456766	456920	154	**-1,33740753**	N12:456891	32,655				rDNA	
chrXII	456780	456887	107	**-1,745208602**	N12:456891	32,655				rDNA	
chrXII	456780	456890	110	**-1,601302824**	N12:456891	32,655				rDNA	
chrXII	456780	456920	140	**-2,173661016**	N12:456891	32,655				rDNA	
chrXII	456794	456903	109	**-0,85538798**	N12:456891	32,655				rDNA	
chrXII	456795	456903	108	**-0,9215189**	N12:456891	32,655				rDNA	
chrXII	456795	456904	109	**-1,015424336**	N12:456891	32,655				rDNA	
chrXII	456795	456905	110	**-1,234605321**	N12:456891	32,655				rDNA	
chrXII	456797	456890	93	**-1,551908122**	N12:456891	32,655				rDNA	
chrXII	456797	456920	123	**-1,885943385**	N12:456891	32,655				rDNA	
chrXII	456803	456959	156	**-1,418588796**	N12:456891	32,655				rDNA	
chrXII	456804	456911	107	**-1,188188098**	N12:456891	32,655				rDNA	
chrXII	456804	456920	116	**-1,299414435**	N12:456891	32,655				rDNA	
chrXII	456804	456960	156	**-1,79560905**	N12:456891	32,655				rDNA	
chrXII	456817	456920	103	**-1,073304825**	N12:456891	32,655				rDNA	
chrXII	456817	456971	154	**-1,414587486**	N12:456891	32,655				rDNA	
chrXII	456938	457104	166	**-1,394260883**	N12:457054	34,078				rDNA	
chrXII	456984	457118	134	**-1,046364252**	N12:457054	34,078				rDNA	
chrXII	456984	457127	143	**-1,407129479**	N12:457054	34,078				rDNA	
chrXII	456984	457130	146	**-1,627737478**	N12:457054	34,078				rDNA	
chrXII	456984	457140	156	**-1,627988076**	N12:457054	34,078				rDNA	
chrXII	456990	457127	137	**-1,357231234**	N12:457054	34,078				rDNA	

CHR	START	END	L(bp)	ΔLknuc	Nuc ID	Fuzziness	Gene ID	Gene body postion or intergene	Terminal	rDNA	Telomeric
chrXII	456990	457140	150	**-1,265434703**	N12:457054	34,078				rDNA	
chrXII	457005	457166	161	**-1,707329845**	N12:457054	34,078				rDNA	
chrXII	457021	457127	106	**-1,729888188**	N12:457054	34,078				rDNA	
chrXII	457022	457205	183	**-2,094253224**	N12:457054	34,078				rDNA	
chrXII	457022	457205	183	**-2,094253224**	N12:457213	27,713				rDNA	
chrXII	457047	457207	160	**-1,440694378**	N12:457054	34,078				rDNA	
chrXII	457047	457207	160	**-1,440694378**	N12:457213	27,713				rDNA	
chrXII	457070	457200	130	**-1,415691373**	N12:457054	34,078				rDNA	
chrXII	457070	457200	130	**-1,415691373**	N12:457213	27,713				rDNA	
chrXII	457083	457217	134	**-1,461019882**	N12:457213	27,713				rDNA	
chrXII	457115	457248	133	**-1,091597452**	N12:457213	27,713				rDNA	
chrXII	457140	457305	165	**-1,283712949**	N12:457213	27,713				rDNA	
chrXII	457140	457312	172	**-1,660956141**	N12:457213	27,713				rDNA	
chrXII	457143	457248	105	**-0,897134712**	N12:457213	27,713				rDNA	
chrXII	457143	457279	136	**-1,106421363**	N12:457213	27,713				rDNA	
chrXII	457143	457316	173	**-1,428495619**	N12:457213	27,713				rDNA	
chrXII	457152	457305	153	**-1,125429647**	N12:457213	27,713				rDNA	
chrXII	457152	457312	160	**-1,471274216**	N12:457213	27,713				rDNA	
chrXII	457153	457248	95	**-0,822754884**	N12:457213	27,713				rDNA	
chrXII	457153	457279	126	**-0,982601006**	N12:457213	27,713				rDNA	
chrXII	457153	457312	159	**-1,482818599**	N12:457213	27,713				rDNA	
chrXII	457153	457316	163	**-1,300547407**	N12:457213	27,713				rDNA	
chrXII	457153	457317	164	**-1,858981961**	N12:457213	27,713				rDNA	
chrXII	457154	457312	158	**-1,432625836**	N12:457213	27,713				rDNA	
chrXII	457154	457316	162	**-1,370748074**	N12:457213	27,713				rDNA	
chrXII	457187	457316	129	**-1,034260647**	N12:457213	27,713				rDNA	
chrXII	457187	457317	130	**-1,002170958**	N12:457213	27,713				rDNA	
chrXII	457198	457346	148	**-1,157632343**	N12:457213	27,713				rDNA	
chrXII	457198	457365	167	**-1,060785429**	N12:457213	27,713				rDNA	
chrXII	457212	457365	153	**-1,073949231**	N12:457213	27,713				rDNA	
chrXII	457214	457363	149	**-0,974284083**	N12:457213	27,713				rDNA	
chrXII	457219	457312	93	**-0,970833668**	N12:457213	27,713				rDNA	
chrXII	457219	457346	127	**-1,139096961**	N12:457213	27,713				rDNA	
chrXII	457219	457363	144	**-1,378376444**	N12:457213	27,713				rDNA	
chrXII	457219	457365	146	**-1,286451175**	N12:457213	27,713				rDNA	
chrXII	457219	457422	203	**-1,915949793**	N12:457213	27,713				rDNA	
chrXII	457227	457316	89	**-0,341855947**	N12:457213	27,713				rDNA	
chrXII	457227	457363	136	**-1,014263317**	N12:457213	27,713				rDNA	
chrXII	457227	457395	168	**-1,770082218**	N12:457213	27,713				rDNA	
chrXII	457230	457395	165	**-1,996545303**	N12:457213	27,713				rDNA	
chrXII	457230	457401	171	**-1,268737505**	N12:457213	27,713				rDNA	
chrXII	457248	457395	147	**-1,659630334**				Overlap <50 bp		rDNA	
chrXII	457248	457401	153	**-0,981301511**				Overlap <50 bp		rDNA	
chrXII	457255	457365	110	**-0,294778099**				Overlap <50 bp		rDNA	
chrXII	457255	457407	152	**-1,102372052**				Overlap <50 bp		rDNA	
chrXII	457255	457422	167	**-1,454556303**				Overlap <50 bp		rDNA	
chrXII	457266	457365	99	**-0,544665328**				Overlap <50 bp		rDNA	
chrXII	457266	457407	141	**-1,016402178**				Overlap <50 bp		rDNA	
chrXII	457266	457422	156	**-1,129833572**				Overlap <50 bp		rDNA	
chrXII	457295	457378	83	**-1,337960809**				Overlap <50 bp		rDNA	
chrXII	457295	457384	89	**-1,01934915**				Overlap <50 bp		rDNA	
chrXII	457295	457390	95	**-1,293055282**				Overlap <50 bp		rDNA	
chrXII	457295	457391	96	**-1,479094384**				Overlap <50 bp		rDNA	
chrXII	457295	457393	98	**-1,498740471**				Overlap <50 bp		rDNA	
chrXII	457295	457394	99	**-1,705260463**				Overlap <50 bp		rDNA	
chrXII	457295	457395	100	**-1,361307624**				Overlap <50 bp		rDNA	
chrXII	457295	457397	102	**-1,699109196**				Overlap <50 bp		rDNA	
chrXII	457295	457401	106	**-1,040004191**				Overlap <50 bp		rDNA	
chrXII	457295	457402	107	**-0,991810719**				Overlap <50 bp		rDNA	
chrXII	457337	457476	139	**-1,375582036**	N12:457494	9,899				rDNA	
chrXII	457337	457481	144	**-0,959935821**	N12:457494	9,899				rDNA	
chrXII	457360	457476	116	**-1,174417981**	N12:457494	9,899				rDNA	
chrXII	457360	457481	121	**-0,639633385**	N12:457494	9,899				rDNA	
chrXII	457360	457530	170	**-1,461578819**	N12:457494	9,899				rDNA	
chrXII	457370	457476	106	**-1,496843022**	N12:457494	9,899				rDNA	
chrXII	457372	457476	104	**-1,8046605**	N12:457494	9,899				rDNA	
chrXII	457420	457563	143	**-1,278611526**	N12:457494	9,899				rDNA	
chrXII	457420	457567	147	**-1,24833696**	N12:457494	9,899				rDNA	
chrXII	457420	457571	151	**-1,17514218**	N12:457494	9,899				rDNA	
chrXII	457420	457572	152	**-0,896145818**	N12:457494	9,899				rDNA	
chrXII	457437	457592	155	**-1,349422117**	N12:457494	9,899				rDNA	
chrXII	457440	457593	153	**-1,405659838**	N12:457494	9,899				rDNA	
chrXII	457540	457654	114	**-1,509183439**				Overlap <50 bp		rDNA	
chrXII	457553	457706	153	**-1,328591869**	N12:457682	13,229				rDNA	
chrXII	457569	457736	167	**-1,619046263**	N12:457682	13,229				rDNA	
chrXII	457569	457741	172	**-1,59854435**	N12:457682	13,229				rDNA	
chrXII	457600	457736	136	**-1,229052112**	N12:457682	13,229				rDNA	
chrXII	457600	457739	139	**-0,572599256**	N12:457682	13,229				rDNA	
chrXII	457600	457741	141	**-1,596682821**	N12:457682	13,229				rDNA	
chrXII	457606	457763	157	**-0,923770464**	N12:457682	13,229				rDNA	
chrXII	457620	457773	153	**-1,384555884**	N12:457682	13,229				rDNA	
chrXII	457620	457787	167	**-1,525940035**	N12:457682	13,229				rDNA	
chrXII	457622	457771	149	**-1,521147421**	N12:457682	13,229				rDNA	
chrXII	457634	457757	123	**-1,203859799**	N12:457682	13,229				rDNA	
chrXII	457650	457805	155	**-1,786824643**	N12:457682	13,229				rDNA	
chrXII	457667	457819	152	**-1,541599981**	N12:457682	13,229				rDNA	
chrXII	457803	457959	156	**-1,116720891**	N12:457885	36,199				rDNA	
chrXII	457816	457974	158	**-1,120690586**	N12:457885	36,199				rDNA	
chrXII	457820	457953	133	**-0,906236552**	N12:457885	36,199				rDNA	
chrXII	457872	458023	151	**-1,308748766**	N12:457885	36,199				rDNA	
chrXII	457945	458104	159	**-1,43931108**				Overlap <50 bp		rDNA	
chrXII	457949	458059	110	**-0,740069368**				Overlap <50 bp		rDNA	
chrXII	457949	458101	152	**-1,210234772**				Overlap <50 bp		rDNA	
chrXII	457950	458101	151	**-1,138303345**				Overlap <50 bp		rDNA	
chrXII	457950	458103	153	**-1,223267332**				Overlap <50 bp		rDNA	
chrXII	457959	458110	151	**-1,18700606**				Overlap <50 bp		rDNA	

CHR	START	END	L(bp)	ΔLknuc	Nuc ID	Fuzziness	Gene ID	Gene body postion or intergene	Terminal	rDNA	Telomeric
chrXII	457976	458059	83	**-0,691302863**				Overlap <50 bp		rDNA	
chrXII	457976	458119	143	**-1,016475699**				Overlap <50 bp		rDNA	
chrXII	457983	458089	106	**-0,576092235**				Overlap <50 bp		rDNA	
chrXII	457983	458101	118	**-0,778624695**				Overlap <50 bp		rDNA	
chrXII	457983	458103	120	**-1,002230921**				Overlap <50 bp		rDNA	
chrXII	457983	458137	154	**-1,451691684**	N12:458145	37,162				rDNA	
chrXII	457986	458117	131	**-0,693143423**				Overlap <50 bp		rDNA	
chrXII	458025	458167	142	**-1,328913323**	N12:458145	37,162				rDNA	
chrXII	458055	458137	82	**-0,826**	N12:458145	37,162				rDNA	
chrXII	458055	458206	151	**-1,144107649**	N12:458145	37,162				rDNA	
chrXII	458079	458227	148	**-1,159596627**	N12:458145	37,162				rDNA	
chrXII	458087	458231	144	**-1,054807619**	N12:458145	37,162				rDNA	
chrXII	458127	458277	150	**-1,437385509**	N12:458145	37,162				rDNA	
chrXII	458130	458293	163	**-1,259887156**	N12:458145	37,162				rDNA	
chrXII	458134	458272	138	**-0,964316105**	N12:458145	37,162				rDNA	
chrXII	458134	458277	143	**-1,166514995**	N12:458145	37,162				rDNA	
chrXII	458136	458295	159	**-1,300142728**	N12:458145	37,162				rDNA	
chrXII	458143	458291	148	**-1,118032353**	N12:458145	37,162				rDNA	
chrXII	458166	458321	155	**-1,476247261**	N12:458145	37,162				rDNA	
chrXII	458166	458321	155	**-1,476247261**	N12:458325	64,042				rDNA	
chrXII	458166	458322	156	**-1,264833973**	N12:458145	37,162				rDNA	
chrXII	458166	458322	156	**-1,264833973**	N12:458325	64,042				rDNA	
chrXII	458167	458322	155	**-1,180999212**	N12:458145	37,162				rDNA	
chrXII	458167	458322	155	**-1,180999212**	N12:458325	64,042				rDNA	
chrXII	458177	458322	145	**-1,227979635**	N12:458325	64,042				rDNA	
chrXII	458177	458327	150	**-1,511203035**	N12:458325	64,042				rDNA	
chrXII	458177	458350	173	**-1,48923011**	N12:458325	64,042				rDNA	
chrXII	458179	458327	148	**-0,929336692**	N12:458325	64,042				rDNA	
chrXII	458191	458321	130	**-0,988652948**	N12:458325	64,042				rDNA	
chrXII	458191	458352	161	**-1,162492706**	N12:458325	64,042				rDNA	
chrXII	458195	458322	127	**-1,174457517**	N12:458325	64,042				rDNA	
chrXII	458195	458327	132	**-1,126380519**	N12:458325	64,042				rDNA	
chrXII	458195	458350	155	**-1,125510608**	N12:458325	64,042				rDNA	
chrXII	458204	458350	146	**-1,445133047**	N12:458325	64,042				rDNA	
chrXII	458204	458380	176	**-1,477894642**	N12:458325	64,042				rDNA	
chrXII	458291	458451	160	**-1,104411188**	N12:458325	64,042				rDNA	
chrXII	458297	458451	154	**-1,213084623**	N12:458325	64,042				rDNA	
chrXII	458298	458451	153	**-1,206093527**	N12:458325	64,042				rDNA	
chrXII	458319	458472	153	**-1,082883441**	N12:458325	64,042				rDNA	
chrXII	458338	458492	154	**-1,240288389**	N12:458325	64,042				rDNA	
chrXII	458356	458493	137	**-1,15964863**				Overlap <50 bp		rDNA	
chrXII	458356	458508	152	**-1,113959374**	N12:458524	15,556				rDNA	
chrXII	458359	458523	164	**-1,559855743**	N12:458524	15,556				rDNA	
chrXII	458365	458493	128	**-1,127147441**				Overlap <50 bp		rDNA	
chrXII	458373	458493	120	**-0,871994003**				Overlap <50 bp		rDNA	
chrXII	458373	458530	157	**-1,336124749**	N12:458524	15,556				rDNA	
chrXII	458374	458532	158	**-1,49667944**	N12:458524	15,556				rDNA	
chrXII	458395	458501	106	**-0,613623019**	N12:458524	15,556				rDNA	
chrXII	458395	458519	124	**-0,922640841**	N12:458524	15,556				rDNA	
chrXII	458419	458587	168	**-1,446254399**	N12:458524	15,556				rDNA	
chrXII	458508	458658	150	**-1,393642725**	N12:458524	15,556				rDNA	
chrXII	458508	458658	150	**-1,393642725**	N12:458681	0				rDNA	
chrXII	458508	458659	151	**-1,250711736**	N12:458524	15,556				rDNA	
chrXII	458508	458659	151	**-1,250711736**	N12:458681	0				rDNA	
chrXII	458746	458901	155	**-1,356935675**	N12:458830	17,205				rDNA	
chrXII	458746	458912	166	**-1,170788193**	N12:458830	17,205				rDNA	
chrXII	458750	458912	162	**-1,574860532**	N12:458830	17,205				rDNA	
chrXII	458768	458900	132	**-0,983896285**	N12:458830	17,205				rDNA	
chrXII	458768	458901	133	**-1,009927836**	N12:458830	17,205				rDNA	
chrXII	458768	458912	144	**-1,16596879**	N12:458830	17,205				rDNA	
chrXII	458769	458900	131	**-1,185463585**	N12:458830	17,205				rDNA	
chrXII	458769	458901	132	**-0,990437932**	N12:458830	17,205				rDNA	
chrXII	458769	458912	143	**-1,225566231**	N12:458830	17,205				rDNA	
chrXII	458769	458913	144	**-1,167909471**	N12:458830	17,205				rDNA	
chrXII	458770	458900	130	**-1,435229287**	N12:458830	17,205				rDNA	
chrXII	458770	458901	131	**-1,379998286**	N12:458830	17,205				rDNA	
chrXII	458770	458912	142	**-1,149949557**	N12:458830	17,205				rDNA	
chrXII	458846	459002	156	**-0,707015271**	N12:458830	17,205				rDNA	
chrXII	458846	459002	156	**-0,707015271**	N12:458985	0				rDNA	
chrXII	458902	459048	146	**-1,360067074**	N12:458985	0				rDNA	
chrXII	458908	459044	136	**-0,964023298**	N12:458985	0				rDNA	
chrXII	458908	459048	140	**-1,221372978**	N12:458985	0				rDNA	
chrXII	458910	459043	133	**-1,197184511**	N12:458985	0				rDNA	
chrXII	458910	459066	156	**-1,557282324**	N12:458985	0				rDNA	
chrXII	458913	459002	89	**-0,761766034**	N12:458985	0				rDNA	
chrXII	458913	459038	125	**-1,090949826**	N12:458985	0				rDNA	
chrXII	458913	459043	130	**-1,191761209**	N12:458985	0				rDNA	
chrXII	458913	459044	131	**-1,280686726**	N12:458985	0				rDNA	
chrXII	458913	459047	134	**-1,223438986**	N12:458985	0				rDNA	
chrXII	458913	459048	135	**-1,18570979**	N12:458985	0				rDNA	
chrXII	458913	459066	153	**-1,321006572**	N12:458985	0				rDNA	
chrXII	458932	459038	106	**-0,623031435**	N12:458985	0				rDNA	
chrXII	458933	459044	111	**-0,77060004**	N12:458985	0				rDNA	
chrXII	458933	459047	114	**-0,670650593**	N12:458985	0				rDNA	
chrXII	458933	459066	133	**-1,067063485**	N12:458985	0				rDNA	
chrXII	458957	459044	87	**-0,736117665**	N12:458985	0				rDNA	
chrXII	459050	459146	96	**-1,229102172**				Overlap <50 bp		rDNA	
chrXII	459089	459262	173	**-1,586410505**	N12:459173	25,456				rDNA	
chrXII	459089	459265	176	**-1,978183737**	N12:459173	25,456				rDNA	
chrXII	459095	459262	167	**-1,845112943**	N12:459173	25,456				rDNA	
chrXII	459095	459265	170	**-1,553855544**	N12:459173	25,456				rDNA	
chrXII	459098	459233	135	**-1,074327545**	N12:459173	25,456				rDNA	
chrXII	459098	459262	164	**-1,637625524**	N12:459173	25,456				rDNA	
chrXII	459166	459328	162	**-1,31323683**	N12:459173	25,456				rDNA	
chrXII	459185	459328	143	**-1,126970673**	N12:459173	25,456				rDNA	
chrXII	459204	459353	149	**-1,083754062**				Overlap <50 bp		rDNA	

CHR	START	END	L(bp)	ΔLknuc	Nuc ID	Fuzziness	Gene ID	Gene body postion or intergene	Terminal	rDNA	Telomeric
chrXII	459204	459417	213	**-1,794668548**				Overlap <50 bp		rDNA	
chrXII	459204	459419	215	**-1,508440292**				Overlap <50 bp		rDNA	
chrXII	459227	459353	126	**-1,083946406**				Overlap <50 bp		rDNA	
chrXII	459227	459376	149	**-1,327600072**				Overlap <50 bp		rDNA	
chrXII	459227	459419	192	**-1,679369252**				Overlap <50 bp		rDNA	
chrXII	459227	459432	205	**-1,926623666**				Overlap <50 bp		rDNA	
chrXII	459235	459395	160	**-1,0075863**				Overlap <50 bp		rDNA	
chrXII	459236	459328	92	**-0,82580406**				Overlap <50 bp		rDNA	
chrXII	459236	459393	157	**-1,408383138**				Overlap <50 bp		rDNA	
chrXII	459236	459417	181	**-1,239900366**				Overlap <50 bp		rDNA	
chrXII	459236	459419	183	**-1,583105474**				Overlap <50 bp		rDNA	
chrXII	459264	459353	89	**-0,453448029**				Overlap <50 bp		rDNA	
chrXII	459264	459376	112	**-0,935159529**				Overlap <50 bp		rDNA	
chrXII	459264	459417	153	**-1,266843002**				Overlap <50 bp		rDNA	
chrXII	459264	459419	155	**-1,30296611**				Overlap <50 bp		rDNA	
chrXII	459264	459432	168	**-1,222785504**				Overlap <50 bp		rDNA	
chrXII	459264	459441	177	**-1,509443008**				Overlap <50 bp		rDNA	
chrXII	459265	459376	111	**-0,66271253**				Overlap <50 bp		rDNA	
chrXII	459265	459393	128	**-1,037598419**				Overlap <50 bp		rDNA	
chrXII	459265	459416	151	**-1,33305579**				Overlap <50 bp		rDNA	
chrXII	459265	459417	152	**-1,296757275**				Overlap <50 bp		rDNA	
chrXII	459265	459419	154	**-1,259303986**				Overlap <50 bp		rDNA	
chrXII	459265	459422	157	**-1,413848697**				Overlap <50 bp		rDNA	
chrXII	459265	459432	167	**-1,536426888**				Overlap <50 bp		rDNA	
chrXII	459265	459441	176	**-1,963536502**				Overlap <50 bp		rDNA	
chrXII	459265	459467	202	**-1,499202467**	N12:459485	42,477				rDNA	
chrXII	459265	459469	204	**-1,645283042**	N12:459485	42,477				rDNA	
chrXII	459267	459393	126	**-1,216318047**				Overlap <50 bp		rDNA	
chrXII	459267	459417	150	**-1,273390539**				Overlap <50 bp		rDNA	
chrXII	459267	459419	152	**-1,283371939**				Overlap <50 bp		rDNA	
chrXII	459267	459422	155	**-1,098347277**				Overlap <50 bp		rDNA	
chrXII	459267	459432	165	**-1,455804089**				Overlap <50 bp		rDNA	
chrXII	459267	459441	174	**-1,125823556**				Overlap <50 bp		rDNA	
chrXII	459267	459469	202	**-1,847406659**	N12:459485	42,477				rDNA	
chrXII	459268	459374	106	**-1,405548963**				Overlap <50 bp		rDNA	
chrXII	459268	459376	108	**-1,083596376**				Overlap <50 bp		rDNA	
chrXII	459268	459393	125	**-0,698453731**				Overlap <50 bp		rDNA	
chrXII	459268	459416	148	**-1,441000592**				Overlap <50 bp		rDNA	
chrXII	459268	459417	149	**-1,191426265**				Overlap <50 bp		rDNA	
chrXII	459268	459419	151	**-1,115525881**				Overlap <50 bp		rDNA	
chrXII	459268	459422	154	**-1,322544518**				Overlap <50 bp		rDNA	
chrXII	459268	459431	163	**-1,438496428**				Overlap <50 bp		rDNA	
chrXII	459268	459432	164	**-1,319347296**				Overlap <50 bp		rDNA	
chrXII	459268	459441	173	**-1,900103265**				Overlap <50 bp		rDNA	
chrXII	459268	459467	199	**-1,376971002**	N12:459485	42,477				rDNA	
chrXII	459268	459469	201	**-1,568375129**	N12:459485	42,477				rDNA	
chrXII	459269	459376	107	**-1,288326132**				Overlap <50 bp		rDNA	
chrXII	459269	459393	124	**-1,215014891**				Overlap <50 bp		rDNA	
chrXII	459269	459413	144	**-1,214560938**				Overlap <50 bp		rDNA	
chrXII	459269	459417	148	**-1,073595191**				Overlap <50 bp		rDNA	
chrXII	459269	459419	150	**-1,186090912**				Overlap <50 bp		rDNA	
chrXII	459269	459422	153	**-1,586776623**				Overlap <50 bp		rDNA	
chrXII	459269	459432	163	**-1,942235017**				Overlap <50 bp		rDNA	
chrXII	459269	459467	198	**-1,491325267**	N12:459485	42,477				rDNA	
chrXII	459269	459469	200	**-1,617723496**	N12:459485	42,477				rDNA	
chrXII	459271	459419	148	**-1,368597729**				Overlap <50 bp		rDNA	
chrXII	459308	459414	106	**-0,814520133**				Overlap <50 bp		rDNA	
chrXII	459308	459419	111	**-0,699559805**				Overlap <50 bp		rDNA	
chrXII	459308	459467	159	**-1,318756425**	N12:459485	42,477				rDNA	
chrXII	459308	459469	161	**-1,063821381**	N12:459485	42,477				rDNA	
chrXII	459325	459466	141	**-0,854807418**	N12:459485	42,477				rDNA	
chrXII	459326	459417	91	**-0,827464423**				Overlap <50 bp		rDNA	
chrXII	459326	459419	93	**-0,450589866**				Overlap <50 bp		rDNA	
chrXII	459326	459467	141	**-0,943208308**	N12:459485	42,477				rDNA	
chrXII	459326	459469	143	**-1,06792038**	N12:459485	42,477				rDNA	
chrXII	459408	459559	151	**-0,966752195**	N12:459485	42,477				rDNA	
chrXII	459429	459558	129	**-1,066094198**	N12:459485	42,477				rDNA	
chrXII	459429	459559	130	**-1,201653976**	N12:459485	42,477				rDNA	
chrXII	459429	459579	150	**-1,504459762**	N12:459485	42,477				rDNA	
chrXII	459430	459558	128	**-1,119961871**	N12:459485	42,477				rDNA	
chrXII	459430	459559	129	**-0,738801499**	N12:459485	42,477				rDNA	
chrXII	459430	459573	143	**-1,132256481**	N12:459485	42,477				rDNA	
chrXII	459430	459579	149	**-1,279957362**	N12:459485	42,477				rDNA	
chrXII	459519	459673	154	**-1,297894152**				Overlap <50 bp		rDNA	
chrXII	459521	459673	152	**-1,264163764**				Overlap <50 bp		rDNA	
chrXII	459602	459756	154	**-1,280103315**	N12:459720	38,118				rDNA	
chrXII	459602	459759	157	**-1,235683514**	N12:459720	38,118				rDNA	
chrXII	459604	459722	118	**-1,043424818**	N12:459720	38,118				rDNA	
chrXII	459604	459756	152	**-1,224011959**	N12:459720	38,118				rDNA	
chrXII	459604	459759	155	**-0,9716152**	N12:459720	38,118				rDNA	
chrXII	459604	459774	170	**-1,381253365**	N12:459720	38,118				rDNA	
chrXII	459604	459796	192	**-1,568180903**	N12:459720	38,118				rDNA	
chrXII	459604	459798	194	**-1,76635304**	N12:459720	38,118				rDNA	
chrXII	459604	459804	200	**-1,385778878**	N12:459720	38,118				rDNA	
chrXII	459604	459806	202	**-1,905480729**	N12:459720	38,118				rDNA	
chrXII	459618	459756	138	**-1,152443788**	N12:459720	38,118				rDNA	
chrXII	459618	459774	156	**-1,112623004**	N12:459720	38,118				rDNA	
chrXII	459618	459796	178	**-1,267103397**	N12:459720	38,118				rDNA	
chrXII	459621	459756	135	**-1,228611434**	N12:459720	38,118				rDNA	
chrXII	459621	459774	153	**-1,346246386**	N12:459720	38,118				rDNA	
chrXII	459625	459756	131	**-1,521083941**	N12:459720	38,118				rDNA	
chrXII	459625	459774	149	**-1,464308181**	N12:459720	38,118				rDNA	
chrXII	459625	459796	171	**-1,632843104**	N12:459720	38,118				rDNA	
chrXII	459625	459798	173	**-1,350708007**	N12:459720	38,118				rDNA	
chrXII	459625	459804	179	**-1,542441342**	N12:459720	38,118				rDNA	
chrXII	459625	459806	181	**-1,744677107**	N12:459720	38,118				rDNA	

CHR	START	END	L(bp)	ΔLknuc	Nuc ID	Fuzziness	Gene ID	Gene body postion or intergene	Terminal	rDNA	Telomeric
chrXII	459628	459756	128	**-0,922787136**	N12:459720	38,118				rDNA	
chrXII	459628	459774	146	**-1,453679542**	N12:459720	38,118				rDNA	
chrXII	459628	459796	168	**-1,23257791**	N12:459720	38,118				rDNA	
chrXII	459628	459798	170	**-1,49570819**	N12:459720	38,118				rDNA	
chrXII	459628	459804	176	**-1,151975833**	N12:459720	38,118				rDNA	
chrXII	459628	459806	178	**-1,76817145**	N12:459720	38,118				rDNA	
chrXII	459633	459756	123	**-0,408312065**	N12:459720	38,118				rDNA	
chrXII	459633	459774	141	**-0,905858266**	N12:459720	38,118				rDNA	
chrXII	459633	459796	163	**-1,317824323**	N12:459720	38,118				rDNA	
chrXII	459633	459798	165	**-1,492815425**	N12:459720	38,118				rDNA	
chrXII	459633	459804	171	**-1,035811662**	N12:459720	38,118				rDNA	
chrXII	459633	459806	173	**-1,434037811**	N12:459720	38,118				rDNA	
chrXII	459648	459796	148	**-1,213895557**	N12:459720	38,118				rDNA	
chrXII	459673	459804	131	**-0,965210962**	N12:459720	38,118				rDNA	
chrXII	459673	459806	133	**-1,361716608**	N12:459720	38,118				rDNA	
chrXII	459686	459793	107	**-0,754851428**	N12:459720	38,118				rDNA	
chrXII	459686	459796	110	**-0,978483143**	N12:459720	38,118				rDNA	
chrXII	459686	459798	112	**-1,123389994**	N12:459720	38,118				rDNA	
chrXII	459686	459799	113	**-0,792643338**	N12:459720	38,118				rDNA	
chrXII	459686	459801	115	**-1,069719075**	N12:459720	38,118				rDNA	
chrXII	459686	459804	118	**-1,019418416**	N12:459720	38,118				rDNA	
chrXII	459686	459806	120	**-1,375134601**	N12:459720	38,118				rDNA	
chrXII	459686	459856	170	**-1,763960877**	N12:459720	38,118				rDNA	
chrXII	459688	459793	105	**-1,378871398**	N12:459720	38,118				rDNA	
chrXII	459688	459851	163	**-1,325042528**	N12:459720	38,118				rDNA	
chrXII	459698	459804	106	**-1,242933518**	N12:459720	38,118				rDNA	
chrXII	459698	459806	108	**-1,065499049**	N12:459720	38,118				rDNA	
chrXII	459698	459851	153	**-1,574371076**	N12:459720	38,118				rDNA	
chrXII	459698	459869	171	**-1,497642081**	N12:459720	38,118				rDNA	
chrXII	459818	459980	162	**-1,610125296**	N12:459899	7,43				rDNA	
chrXII	459834	459969	135	**-1,334389082**	N12:459899	7,43				rDNA	
chrXII	459846	459969	123	**-1,056817996**	N12:459899	7,43				rDNA	
chrXII	459846	460002	156	**-1,583955114**	N12:459899	7,43				rDNA	
chrXII	459899	460068	169	**-1,572256926**	N12:459899	7,43				rDNA	
chrXII	459899	460068	169	**-1,572256926**	N12:460071	11,206				rDNA	
chrXII	459930	460057	127	**-1,411320036**	N12:460071	11,206				rDNA	
chrXII	460017	460128	111	**-0,692692996**	N12:460071	11,206				rDNA	
chrXII	460017	460159	142	**-1,04580746**	N12:460071	11,206				rDNA	
chrXII	460032	460159	127	**-1,219835518**	N12:460071	11,206				rDNA	
chrXII	460102	460251	149	**-1,141959205**				Overlap <50 bp		rDNA	
chrXII	460102	460266	164	**-1,214136836**				Overlap <50 bp		rDNA	
chrXII	460112	460251	139	**-1,183958859**				Overlap <50 bp		rDNA	
chrXII	460112	460266	154	**-1,419879802**				Overlap <50 bp		rDNA	
chrXII	460188	460366	178	**-1,63267678**	N12:460347	14,446				rDNA	
chrXII	460243	460384	141	**-1,045238909**	N12:460347	14,446				rDNA	
chrXII	460256	460415	159	**-1,658160485**	N12:460347	14,446				rDNA	
chrXII	460262	460436	174	**-1,860156586**	N12:460347	14,446				rDNA	
chrXII	460302	460477	175	**-1,724575025**	N12:460347	14,446				rDNA	
chrXII	460314	460446	132	**-1,42920196**	N12:460347	14,446				rDNA	
chrXII	460402	460571	169	**-1,693998877**				Overlap <50 bp		rDNA	
chrXII	460461	460595	134	**-1,069910373**	N12:460603	33,387				rDNA	
chrXII	460487	460616	129	**-1,017611253**	N12:460603	33,387				rDNA	
chrXII	460493	460595	102	**-1,556469984**	N12:460603	33,387				rDNA	
chrXII	460493	460659	166	**-2,008001199**	N12:460603	33,387				rDNA	
chrXII	460512	460595	83	**-1,310340248**	N12:460603	33,387				rDNA	
chrXII	460512	460639	127	**-1,229662665**	N12:460603	33,387				rDNA	
chrXII	460521	460659	138	**-1,275402451**	N12:460603	33,387				rDNA	
chrXII	460602	460763	161	**-1,590887061**	N12:460603	33,387				rDNA	
chrXII	460846	460979	133	**-1,479111747**	N12:460826	15,556				rDNA	
chrXII	460863	461022	159	**-1,295823817**				Overlap <50 bp		rDNA	
chrXII	460871	461006	135	**-1,483873741**				Overlap <50 bp		rDNA	
chrXII	460894	460979	85	**-0,759025328**				Overlap <50 bp		rDNA	
chrXII	460894	461045	151	**-1,523354561**				Overlap <50 bp		rDNA	
chrXII	460894	461097	203	**-1,765695745**				Overlap <50 bp		rDNA	
chrXII	460906	461069	163	**-1,399714976**				Overlap <50 bp		rDNA	
chrXII	460927	461045	118	**-1,223043342**				Overlap <50 bp		rDNA	
chrXII	460927	461097	170	**-1,377093935**				Overlap <50 bp		rDNA	
chrXII	460937	461071	134	**-0,977204797**				Overlap <50 bp		rDNA	
chrXII	460937	461098	161	**-1,542901964**				Overlap <50 bp		rDNA	
chrXII	460988	461071	83	**-0,85669586**				Overlap <50 bp		rDNA	
chrXII	460988	461095	107	**-1,43876598**				Overlap <50 bp		rDNA	
chrXII	460988	461096	108	**-0,945073079**				Overlap <50 bp		rDNA	
chrXII	460988	461098	110	**-1,299774635**				Overlap <50 bp		rDNA	
chrXII	461013	461146	133	**-1,267856788**	N12:461125	34,641				rDNA	
chrXII	461056	461146	90	**-0,738013277**	N12:461125	34,641				rDNA	
chrXII	461056	461148	92	**-1,447851605**	N12:461125	34,641				rDNA	
chrXII	461056	461199	143	**-1,61588963**	N12:461125	34,641				rDNA	
chrXII	461056	461212	156	**-1,177381364**	N12:461125	34,641				rDNA	
chrXII	461056	461214	158	**-1,382805765**	N12:461125	34,641				rDNA	
chrXII	461056	461215	159	**-1,217202941**	N12:461125	34,641				rDNA	
chrXII	461056	461229	173	**-1,252693267**	N12:461125	34,641				rDNA	
chrXII	461060	461146	86	**-0,635572156**	N12:461125	34,641				rDNA	
chrXII	461060	461212	152	**-1,275856664**	N12:461125	34,641				rDNA	
chrXII	461060	461214	154	**-1,748558927**	N12:461125	34,641				rDNA	
chrXII	461060	461215	155	**-1,419763076**	N12:461125	34,641				rDNA	
chrXII	461060	461229	169	**-0,906330119**	N12:461125	34,641				rDNA	
chrXII	461068	461199	131	**-1,208852873**	N12:461125	34,641				rDNA	
chrXII	461068	461214	146	**-1,240158825**	N12:461125	34,641				rDNA	
chrXII	461068	461246	178	**-1,322613742**	N12:461125	34,641				rDNA	
chrXII	461069	461199	130	**-1,119193943**	N12:461125	34,641				rDNA	
chrXII	461069	461214	145	**-1,554933122**	N12:461125	34,641				rDNA	
chrXII	461069	461246	177	**-1,388781136**	N12:461125	34,641				rDNA	
chrXII	461082	461188	106	**-0,983832423**	N12:461125	34,641				rDNA	
chrXII	461082	461199	117	**-0,809951429**	N12:461125	34,641				rDNA	
chrXII	461082	461212	130	**-1,00202511**	N12:461125	34,641				rDNA	
chrXII	461082	461214	132	**-1,021239956**	N12:461125	34,641				rDNA	

CHR	START	END	L(bp)	ΔLknuc	Nuc ID	Fuzziness	Gene ID	Gene body postion or intergene	Terminal	rDNA	Telomeric
chrXII	461082	461215	133	-1,150598564	N12:461125	34,641				rDNA	
chrXII	461082	461229	147	-1,017966106	N12:461125	34,641				rDNA	
chrXII	461082	461246	164	-1,395751218	N12:461125	34,641				rDNA	
chrXII	461095	461246	151	-1,025970322	N12:461125	34,641				rDNA	
chrXII	461178	461333	155	-1,389661098	N12:461308	61,052				rDNA	
chrXII	461196	461352	156	-1,390413046	N12:461308	61,052				rDNA	
chrXII	461218	461364	146	-1,213886286	N12:461308	61,052				rDNA	
chrXII	461218	461384	166	-1,301897258	N12:461308	61,052				rDNA	
chrXII	461218	461388	170	-1,106705622	N12:461308	61,052				rDNA	
chrXII	461241	461384	143	-1,173358607	N12:461308	61,052				rDNA	
chrXII	461241	461388	147	-0,823477867	N12:461308	61,052				rDNA	
chrXII	461252	461408	156	-1,168312379	N12:461308	61,052				rDNA	
chrXII	461260	461367	107	-1,258905485	N12:461308	61,052				rDNA	
chrXII	461327	461481	154	-1,343883463	N12:461308	61,052				rDNA	
chrXII	461397	461551	154	-1,394557994	N12:461507	14,422				rDNA	
chrXII	461430	461537	107	-1,593911389	N12:461507	14,422				rDNA	
chrXII	461431	461592	161	-1,304230891	N12:461507	14,422				rDNA	
chrXII	461432	461592	160	-1,239111892	N12:461507	14,422				rDNA	
chrXII	461436	461592	156	-1,186791263	N12:461507	14,422				rDNA	
chrXII	461460	461622	162	-1,419288148	N12:461507	14,422				rDNA	
chrXII	461460	461623	163	-1,333641154	N12:461507	14,422				rDNA	
chrXII	461471	461578	107	-0,730947057	N12:461507	14,422				rDNA	
chrXII	461471	461592	121	-0,922646777	N12:461507	14,422				rDNA	
chrXII	461471	461622	151	-1,209718649	N12:461507	14,422				rDNA	
chrXII	461471	461623	152	-1,409184694	N12:461507	14,422				rDNA	
chrXII	461483	461633	150	-1,248029841	N12:461507	14,422				rDNA	
chrXII	461536	461685	149	-1,373995135	N12:461692	5,774				rDNA	
chrXII	461560	461721	161	-1,502916154	N12:461692	5,774				rDNA	
chrXII	461606	461757	151	-1,617421207	N12:461692	5,774				rDNA	
chrXII	461606	461758	152	-1,623816206	N12:461692	5,774				rDNA	
chrXII	461606	461766	160	-1,476822976	N12:461692	5,774				rDNA	
chrXII	461606	461767	161	-1,334150821	N12:461692	5,774				rDNA	
chrXII	461628	461758	130	-1,708770553	N12:461692	5,774				rDNA	
chrXII	461628	461766	138	-1,6251081	N12:461692	5,774				rDNA	
chrXII	461628	461800	172	-1,545659216	N12:461692	5,774				rDNA	
chrXII	461629	461721	92	-1,178017207	N12:461692	5,774				rDNA	
chrXII	461629	461757	128	-1,554312406	N12:461692	5,774				rDNA	
chrXII	461629	461758	129	-1,421107881	N12:461692	5,774				rDNA	
chrXII	461629	461766	137	-1,546194707	N12:461692	5,774				rDNA	
chrXII	461629	461800	171	-1,980364364	N12:461692	5,774				rDNA	
chrXII	461662	461758	96	-0,726193226	N12:461692	5,774				rDNA	
chrXII	461662	461832	170	-1,546249051	N12:461692	5,774				rDNA	
chrXII	461676	461810	134	-1,542380032	N12:461692	5,774				rDNA	
chrXII	461687	461800	113	-0,980876838	N12:461692	5,774				rDNA	
chrXII	461687	461809	122	-1,602595426	N12:461692	5,774				rDNA	
chrXII	461687	461839	152	-1,319174046	N12:461692	5,774				rDNA	
chrXII	461805	461933	128	-1,368454211	N12:461891	27,622				rDNA	
chrXII	461805	461951	146	-1,533184236	N12:461891	27,622				rDNA	
chrXII	461821	461933	112	-0,87384802	N12:461891	27,622				rDNA	
chrXII	461821	461972	151	-1,185196443	N12:461891	27,622				rDNA	
chrXII	461823	461951	128	-1,099271338	N12:461891	27,622				rDNA	
chrXII	461823	462001	178	-1,589564566	N12:461891	27,622				rDNA	
chrXII	461846	461952	106	-0,675483877	N12:461891	27,622				rDNA	
chrXII	461846	462001	155	-1,367892209	N12:461891	27,622				rDNA	
chrXII	461846	462021	175	-1,224499161	N12:461891	27,622				rDNA	
chrXII	461846	462037	191	-1,502568982	N12:461891	27,622				rDNA	
chrXII	461860	461951	91	-0,636169527	N12:461891	27,622				rDNA	
chrXII	461860	462001	141	-1,419207735	N12:461891	27,622				rDNA	
chrXII	461860	462021	161	-1,238731069	N12:461891	27,622				rDNA	
chrXII	461860	462037	177	-1,333089514	N12:461891	27,622				rDNA	
chrXII	461902	462001	99	-0,708383238	N12:461891	27,622				rDNA	
chrXII	461902	462008	106	-0,915563165	N12:461891	27,622				rDNA	
chrXII	461902	462021	119	-0,755709778	N12:461891	27,622				rDNA	
chrXII	461902	462037	135	-1,125045892	N12:461891	27,622				rDNA	
chrXII	461902	462062	160	-0,926331205	N12:461891	27,622				rDNA	
chrXII	461902	462062	160	-0,926331205	N12:462072	28,042				rDNA	
chrXII	461910	462001	91	-0,868969365	N12:461891	27,622				rDNA	
chrXII	461910	462016	106	-1,002198979	N12:461891	27,622				rDNA	
chrXII	461910	462021	111	-0,818868114	N12:461891	27,622				rDNA	
chrXII	461910	462037	127	-0,993582129	N12:461891	27,622				rDNA	
chrXII	461910	462062	152	-1,355538681	N12:461891	27,622				rDNA	
chrXII	461910	462062	152	-1,355538681	N12:462072	28,042				rDNA	
chrXII	461920	462099	179	-1,795874783	N12:462072	28,042				rDNA	
chrXII	461925	462060	135	-1,102158522	N12:462072	28,042				rDNA	
chrXII	461925	462090	165	-1,345084215	N12:462072	28,042				rDNA	
chrXII	461938	462060	122	-0,885108621	N12:462072	28,042				rDNA	
chrXII	461938	462090	152	-1,278177534	N12:462072	28,042				rDNA	
chrXII	461938	462136	198	-1,670009443	N12:462072	28,042				rDNA	
chrXII	461977	462090	113	-0,703973579	N12:462072	28,042				rDNA	
chrXII	461977	462136	159	-1,283025192	N12:462072	28,042				rDNA	
chrXII	461977	462160	183	-1,464920009	N12:462072	28,042				rDNA	
chrXII	461984	462090	106	-0,826732631	N12:462072	28,042				rDNA	
chrXII	461984	462133	149	-1,472566543	N12:462072	28,042				rDNA	
chrXII	462013	462171	158	-1,290787868	N12:462072	28,042				rDNA	
chrXII	462022	462171	149	-1,180034919	N12:462072	28,042				rDNA	
chrXII	462030	462136	106	-0,739653604	N12:462072	28,042				rDNA	
chrXII	462032	462160	128	-0,98105994	N12:462072	28,042				rDNA	
chrXII	462059	462166	107	-1,464163252	N12:462072	28,042				rDNA	
chrXII	462059	462167	108	-1,324770286	N12:462072	28,042				rDNA	
chrXII	462059	462171	112	-1,434563087	N12:462072	28,042				rDNA	
chrXII	462059	462193	134	-1,313044451	N12:462072	28,042				rDNA	
chrXII	462059	462212	153	-1,706287945	N12:462072	28,042				rDNA	
chrXII	462063	462134	71	-0,749	N12:462072	28,042				rDNA	
chrXII	462063	462170	107	-0,979522693	N12:462072	28,042				rDNA	
chrXII	462063	462171	108	-0,605029641	N12:462072	28,042				rDNA	
chrXII	462063	462193	130	-1,042789847	N12:462072	28,042				rDNA	

CHR	START	END	L(bp)	ΔLknuc	Nuc ID	Fuzziness	Gene ID	Gene body postion or intergene	Terminal	rDNA	Telomeric
chrXII	462063	462207	144	**-1,062640054**	N12:462072	28,042				rDNA	
chrXII	462063	462212	149	**-1,384053019**	N12:462072	28,042				rDNA	
chrXII	462113	462275	162	**-1,539597824**	N12:462253	25,456				rDNA	
chrXII	462128	462278	150	**-1,242559948**	N12:462253	25,456				rDNA	
chrXII	462168	462275	107	**-0,993415835**	N12:462253	25,456				rDNA	
chrXII	462170	462300	130	**-1,173539009**	N12:462253	25,456				rDNA	
chrXII	462170	462324	154	**-1,402908952**	N12:462253	25,456				rDNA	
chrXII	462170	462330	160	**-1,504258326**	N12:462253	25,456				rDNA	
chrXII	462176	462300	124	**-0,791852804**	N12:462253	25,456				rDNA	
chrXII	462176	462324	148	**-1,105808041**	N12:462253	25,456				rDNA	
chrXII	462176	462330	154	**-1,222273098**	N12:462253	25,456				rDNA	
chrXII	462179	462349	170	**-1,656822088**	N12:462253	25,456				rDNA	
chrXII	462179	462355	176	**-1,310916345**	N12:462253	25,456				rDNA	
chrXII	462180	462300	120	**-1,007527494**	N12:462253	25,456				rDNA	
chrXII	462180	462324	144	**-1,166792665**	N12:462253	25,456				rDNA	
chrXII	462180	462327	147	**-1,156929363**	N12:462253	25,456				rDNA	
chrXII	462180	462330	150	**-1,281108512**	N12:462253	25,456				rDNA	
chrXII	462228	462336	108	**-0,483761136**	N12:462253	25,456				rDNA	
chrXII	462229	462336	107	**-0,841602115**	N12:462253	25,456				rDNA	
chrXII	462229	462337	108	**-0,770439775**	N12:462253	25,456				rDNA	
chrXII	462229	462349	120	**-1,148835858**	N12:462253	25,456				rDNA	
chrXII	462229	462373	144	**-1,59916081**	N12:462253	25,456				rDNA	
chrXII	462229	462382	153	**-1,384740384**	N12:462253	25,456				rDNA	
chrXII	462229	462388	159	**-1,325279425**	N12:462253	25,456				rDNA	
chrXII	462229	462407	178	**-1,711292995**	N12:462253	25,456				rDNA	
chrXII	462229	462423	194	**-1,71060805**	N12:462253	25,456				rDNA	
chrXII	462229	462425	196	**-1,897176877**	N12:462253	25,456				rDNA	
chrXII	462232	462338	106	**-1,010167448**	N12:462253	25,456				rDNA	
chrXII	462232	462349	117	**-0,867537202**	N12:462253	25,456				rDNA	
chrXII	462232	462355	123	**-0,763248727**	N12:462253	25,456				rDNA	
chrXII	462232	462373	141	**-1,583144002**	N12:462253	25,456				rDNA	
chrXII	462232	462382	150	**-1,242722246**	N12:462253	25,456				rDNA	
chrXII	462232	462407	175	**-1,234454735**	N12:462253	25,456				rDNA	
chrXII	462232	462419	187	**-1,536254509**	N12:462253	25,456				rDNA	
chrXII	462232	462423	191	**-1,539395061**	N12:462253	25,456				rDNA	
chrXII	462232	462425	193	**-1,693854267**	N12:462253	25,456				rDNA	
chrXII	462236	462373	137	**-0,855473563**	N12:462253	25,456				rDNA	
chrXII	462236	462425	189	**-1,347544847**	N12:462253	25,456				rDNA	
chrXII	462237	462375	138	**-1,222113025**	N12:462253	25,456				rDNA	
chrXII	462237	462382	145	**-1,029391304**	N12:462253	25,456				rDNA	
chrXII	462250	462382	132	**-1,258446751**	N12:462253	25,456				rDNA	
chrXII	462250	462407	157	**-1,471150145**	N12:462253	25,456				rDNA	
chrXII	462250	462423	173	**-1,637996903**	N12:462253	25,456				rDNA	
chrXII	462250	462425	175	**-1,588528981**	N12:462253	25,456				rDNA	
chrXII	462251	462373	122	**-0,583575677**	N12:462253	25,456				rDNA	
chrXII	462251	462406	155	**-1,284997819**	N12:462253	25,456				rDNA	
chrXII	462251	462407	156	**-1,163408296**	N12:462253	25,456				rDNA	
chrXII	462251	462423	172	**-1,243606312**	N12:462253	25,456				rDNA	
chrXII	462251	462424	173	**-1,345366309**	N12:462253	25,456				rDNA	
chrXII	462251	462425	174	**-1,325462079**	N12:462253	25,456				rDNA	
chrXII	462251	462439	188	**-1,643109934**	N12:462253	25,456				rDNA	
chrXII	462253	462406	153	**-0,941129587**	N12:462253	25,456				rDNA	
chrXII	462253	462407	154	**-1,283870006**	N12:462253	25,456				rDNA	
chrXII	462253	462424	171	**-1,252664851**	N12:462253	25,456				rDNA	
chrXII	462253	462425	172	**-1,084616153**	N12:462253	25,456				rDNA	
chrXII	462256	462373	117	**-0,688064683**	N12:462253	25,456				rDNA	
chrXII	462256	462388	132	**-1,506836606**	N12:462253	25,456				rDNA	
chrXII	462256	462406	150	**-1,288585557**	N12:462253	25,456				rDNA	
chrXII	462256	462407	151	**-1,429895149**	N12:462253	25,456				rDNA	
chrXII	462256	462419	163	**-2,167397761**	N12:462253	25,456				rDNA	
chrXII	462256	462423	167	**-1,435482441**	N12:462253	25,456				rDNA	
chrXII	462256	462424	168	**-1,400714873**	N12:462253	25,456				rDNA	
chrXII	462256	462425	169	**-1,3114823**	N12:462253	25,456				rDNA	
chrXII	462256	462439	183	**-1,507268189**	N12:462253	25,456				rDNA	
chrXII	462256	462446	190	**-1,788095378**	N12:462253	25,456				rDNA	
chrXII	462257	462365	108	**-0,336153445**	N12:462253	25,456				rDNA	
chrXII	462258	462407	149	**-1,237324861**	N12:462253	25,456				rDNA	
chrXII	462258	462423	165	**-1,37820741**	N12:462253	25,456				rDNA	
chrXII	462258	462425	167	**-1,491431317**	N12:462253	25,456				rDNA	
chrXII	462262	462368	106	**-1,13043658**	N12:462253	25,456				rDNA	
chrXII	462262	462373	111	**-1,28856716**	N12:462253	25,456				rDNA	
chrXII	462262	462407	145	**-1,420251734**	N12:462253	25,456				rDNA	
chrXII	462262	462419	157	**-2,076701185**	N12:462253	25,456				rDNA	
chrXII	462262	462423	161	**-1,404982031**	N12:462253	25,456				rDNA	
chrXII	462262	462424	162	**-1,217048808**	N12:462253	25,456				rDNA	
chrXII	462262	462425	163	**-1,355209827**	N12:462253	25,456				rDNA	
chrXII	462262	462439	177	**-1,404331055**	N12:462253	25,456				rDNA	
chrXII	462263	462385	122	**-1,465249066**	N12:462253	25,456				rDNA	
chrXII	462263	462407	144	**-1,432089506**	N12:462253	25,456				rDNA	
chrXII	462263	462423	160	**-1,322477134**	N12:462253	25,456				rDNA	
chrXII	462263	462424	161	**-1,445678272**	N12:462253	25,456				rDNA	
chrXII	462263	462425	162	**-1,567583167**	N12:462253	25,456				rDNA	
chrXII	462272	462407	135	**-1,488203503**	N12:462253	25,456				rDNA	
chrXII	462272	462419	147	**-1,292397848**	N12:462253	25,456				rDNA	
chrXII	462272	462423	151	**-1,579990156**	N12:462253	25,456				rDNA	
chrXII	462272	462425	153	**-1,736374966**	N12:462253	25,456				rDNA	
chrXII	462275	462423	148	**-1,370781459**	N12:462253	25,456				rDNA	
chrXII	462275	462425	150	**-1,915876347**	N12:462253	25,456				rDNA	
chrXII	462277	462373	96	**-0,567121191**				Overlap <50 bp		rDNA	
chrXII	462277	462382	105	**-0,302258508**				Overlap <50 bp		rDNA	
chrXII	462277	462383	106	**-0,970455414**				Overlap <50 bp		rDNA	
chrXII	462277	462388	111	**-1,40254736**				Overlap <50 bp		rDNA	
chrXII	462277	462407	130	**-1,050457647**				Overlap <50 bp		rDNA	
chrXII	462277	462419	142	**-1,260675382**				Overlap <50 bp		rDNA	
chrXII	462277	462423	146	**-1,242909695**				Overlap <50 bp		rDNA	
chrXII	462277	462424	147	**-1,351397919**				Overlap <50 bp		rDNA	

CHR	START	END	L(bp)	ΔLknuc	Nuc ID	Fuzziness	Gene ID	Gene body postion or intergene	Terminal	rDNA	Telomeric
chrXII	462277	462425	148	**-1,271145951**				Overlap <50 bp		rDNA	
chrXII	462277	462439	162	**-1,650712858**				Overlap <50 bp		rDNA	
chrXII	462277	462460	183	**-1,291285531**				Overlap <50 bp		rDNA	
chrXII	462278	462373	95	**-0,389088976**				Overlap <50 bp		rDNA	
chrXII	462278	462382	104	**-0,449366181**				Overlap <50 bp		rDNA	
chrXII	462278	462406	128	**-0,513846831**				Overlap <50 bp		rDNA	
chrXII	462278	462407	129	**-0,858033522**				Overlap <50 bp		rDNA	
chrXII	462278	462418	140	**-1,312192965**				Overlap <50 bp		rDNA	
chrXII	462278	462419	141	**-1,330812732**				Overlap <50 bp		rDNA	
chrXII	462278	462420	142	**-1,493351734**				Overlap <50 bp		rDNA	
chrXII	462278	462423	145	**-1,171651451**				Overlap <50 bp		rDNA	
chrXII	462278	462424	146	**-1,460693145**				Overlap <50 bp		rDNA	
chrXII	462278	462425	147	**-1,234977821**				Overlap <50 bp		rDNA	
chrXII	462278	462439	161	**-1,155210563**				Overlap <50 bp		rDNA	
chrXII	462278	462446	168	**-1,340070299**				Overlap <50 bp		rDNA	
chrXII	462278	462460	182	**-1,424874251**				Overlap <50 bp		rDNA	
chrXII	462278	462461	183	**-1,540534897**				Overlap <50 bp		rDNA	
chrXII	462278	462479	201	**-1,673067841**				Overlap <50 bp		rDNA	
chrXII	462281	462425	144	**-1,927457252**				Overlap <50 bp		rDNA	
chrXII	462287	462423	136	**-1,123977213**				Overlap <50 bp		rDNA	
chrXII	462287	462425	138	**-1,452089606**				Overlap <50 bp		rDNA	
chrXII	462287	462432	145	**-1,259254691**				Overlap <50 bp		rDNA	
chrXII	462287	462439	152	**-1,969162681**				Overlap <50 bp		rDNA	
chrXII	462289	462377	88	**-0,771557704**				Overlap <50 bp		rDNA	
chrXII	462289	462407	118	**-0,911764917**				Overlap <50 bp		rDNA	
chrXII	462289	462423	134	**-1,157213041**				Overlap <50 bp		rDNA	
chrXII	462289	462424	135	**-1,376744813**				Overlap <50 bp		rDNA	
chrXII	462289	462425	136	**-1,239319998**				Overlap <50 bp		rDNA	
chrXII	462289	462439	150	**-1,026972379**				Overlap <50 bp		rDNA	
chrXII	462290	462423	133	**-1,122164485**				Overlap <50 bp		rDNA	
chrXII	462290	462425	135	**-0,971792625**				Overlap <50 bp		rDNA	
chrXII	462296	462407	111	**-0,751666002**				Overlap <50 bp		rDNA	
chrXII	462296	462423	127	**-1,138218147**				Overlap <50 bp		rDNA	
chrXII	462296	462424	128	**-0,987759496**				Overlap <50 bp		rDNA	
chrXII	462296	462425	129	**-0,946623822**				Overlap <50 bp		rDNA	
chrXII	462296	462439	143	**-1,148745233**				Overlap <50 bp		rDNA	
chrXII	462296	462446	150	**-1,511981275**				Overlap <50 bp		rDNA	
chrXII	462296	462472	176	**-1,584458547**				Overlap <50 bp		rDNA	
chrXII	462296	462479	183	**-1,831632027**				Overlap <50 bp		rDNA	
chrXII	462298	462382	84	**-0,422770002**				Overlap <50 bp		rDNA	
chrXII	462298	462405	107	**-0,951692148**				Overlap <50 bp		rDNA	
chrXII	462298	462407	109	**-0,704815923**				Overlap <50 bp		rDNA	
chrXII	462298	462419	121	**-1,057561331**				Overlap <50 bp		rDNA	
chrXII	462298	462423	125	**-1,009155207**				Overlap <50 bp		rDNA	
chrXII	462298	462425	127	**-1,080826114**				Overlap <50 bp		rDNA	
chrXII	462298	462446	148	**-1,549906994**				Overlap <50 bp		rDNA	
chrXII	462298	462460	162	**-1,372380122**				Overlap <50 bp		rDNA	
chrXII	462298	462461	163	**-1,478055751**				Overlap <50 bp		rDNA	
chrXII	462299	462406	107	**-0,650650614**				Overlap <50 bp		rDNA	
chrXII	462299	462423	124	**-1,048727808**				Overlap <50 bp		rDNA	
chrXII	462299	462425	126	**-1,235184027**				Overlap <50 bp		rDNA	
chrXII	462299	462460	161	**-1,442117698**				Overlap <50 bp		rDNA	
chrXII	462299	462461	162	-1,28246185				Overlap <50 bp		rDNA	
chrXII	462302	462424	122	**-1,007377917**				Overlap <50 bp		rDNA	
chrXII	462302	462425	123	**-1,042169499**				Overlap <50 bp		rDNA	
chrXII	462302	462439	137	**-1,492231006**				Overlap <50 bp		rDNA	
chrXII	462302	462479	177	**-1,336233641**				Overlap <50 bp		rDNA	
chrXII	462308	462479	171	**-1,410751805**				Overlap <50 bp		rDNA	
chrXII	462317	462425	108	**-1,160751066**				Overlap <50 bp		rDNA	
chrXII	462317	462477	160	**-1,752379822**				Overlap <50 bp		rDNA	
chrXII	462318	462407	89	**-1,218961854**				Overlap <50 bp		rDNA	
chrXII	462318	462419	101	**-1,706901076**				Overlap <50 bp		rDNA	
chrXII	462318	462423	105	**-1,133388482**				Overlap <50 bp		rDNA	
chrXII	462318	462425	107	**-1,248235465**				Overlap <50 bp		rDNA	
chrXII	462318	462460	142	**-1,335013962**				Overlap <50 bp		rDNA	
chrXII	462318	462461	143	**-1,790923092**				Overlap <50 bp		rDNA	
chrXII	462423	462569	146	**-0,872423658**	N12:462515	21,517	+2:YLR154C-G;	2		rDNA	
chrXII	462436	462577	141	**-1,028616253**	N12:462515	21,517	+2:YLR154C-G;	2		rDNA	
chrXII	462436	462597	161	**-1,509804872**	N12:462515	21,517	+2:YLR154C-G;	2		rDNA	
chrXII	462437	462547	110	**-1,316457738**	N12:462515	21,517	+2:YLR154C-G;	2		rDNA	
chrXII	462437	462577	140	**-1,512851225**	N12:462515	21,517	+2:YLR154C-G;	2		rDNA	
chrXII	462439	462577	138	**-1,428489747**	N12:462515	21,517	+2:YLR154C-G;	2		rDNA	
chrXII	462440	462577	137	**-1,412360047**	N12:462515	21,517	+2:YLR154C-G;	2		rDNA	
chrXII	462447	462577	130	**-0,924465004**	N12:462515	21,517	+2:YLR154C-G;	2		rDNA	
chrXII	462447	462597	150	**-1,318042782**	N12:462515	21,517	+2:YLR154C-G;	2		rDNA	
chrXII	462459	462618	159	**-1,356019689**	N12:462515	21,517	+2:YLR154C-G;	2		rDNA	
chrXII	462595	462732	137	**-1,651212947**	N12:462669	19,296	+1:YLR154C-G;	1		rDNA	
chrXII	462595	462747	152	**-1,439696431**	N12:462669	19,296	+1:YLR154C-G;	1		rDNA	
chrXII	462595	462749	154	**-1,415710209**	N12:462669	19,296	+1:YLR154C-G;	1		rDNA	
chrXII	462597	462749	152	**-1,200723581**	N12:462669	19,296	+1:YLR154C-G;	1		rDNA	
chrXII	462624	462732	108	**-1,470986452**	N12:462669	19,296	+1:YLR154C-G;	1		rDNA	
chrXII	462624	462747	123	**-1,307820989**	N12:462669	19,296	+1:YLR154C-G;	1		rDNA	
chrXII	462624	462749	125	**-1,429388607**	N12:462669	19,296	+1:YLR154C-G;	1		rDNA	
chrXII	462637	462789	152	**-0,960058724**	N12:462669	19,296	+1:YLR154C-G;	1		rDNA	
chrXII	462711	462864	153	**-1,113263202**	N12:462833	22,942	0:YLR154C-G;	0		rDNA	
chrXII	462724	462874	150	**-1,432615997**	N12:462833	22,942	0:YLR154C-G;	0		rDNA	
chrXII	462764	462913	149	**-1,807936083**	N12:462833	22,942	0:YLR154C-G;	0		rDNA	
chrXII	462764	462916	152	**-1,092942978**	N12:462833	22,942	0:YLR154C-G;	0		rDNA	
chrXII	462794	462951	157	**-0,876778185**	N12:462833	22,942	0:YLR154C-G;	0		rDNA	
chrXII	462796	462913	117	**-1,32578172**	N12:462833	22,942	0:YLR154C-G;	0		rDNA	
chrXII	462796	462916	120	**-1,412727582**	N12:462833	22,942	0:YLR154C-G;	0		rDNA	
chrXII	462871	462990	119	**-1,159231333**	N12:462990	9,899	-1:YLR154C-G;	-1		rDNA	
chrXII	462885	463047	162	**-1,907815123**	N12:462990	9,899	-1:YLR154C-G;	-1		rDNA	
chrXII	462885	463050	165	**-1,360044289**	N12:462990	9,899	-1:YLR154C-G;	-1		rDNA	
chrXII	462885	463056	171	**-1,402708028**	N12:462990	9,899	-1:YLR154C-G;	-1		rDNA	
chrXII	462885	463059	174	**-1,345505709**	N12:462990	9,899	-1:YLR154C-G;	-1		rDNA	

CHR	START	END	L(bp)	ΔLknuc	Nuc ID	Fuzziness	Gene ID	Gene body postion or intergene	Terminal	rDNA	Telomeric
chrXII	462885	463099	214	**-1,388139775**	N12:462990	9,899	-1:YLR154C-G;	-1		rDNA	
chrXII	462889	462996	107	**-0,420298127**	N12:462990	9,899	-1:YLR154C-G;	-1		rDNA	
chrXII	462889	463045	156	**-1,512219097**	N12:462990	9,899	-1:YLR154C-G;	-1		rDNA	
chrXII	462889	463048	159	**-1,415230859**	N12:462990	9,899	-1:YLR154C-G;	-1		rDNA	
chrXII	462889	463051	162	**-1,352677981**	N12:462990	9,899	-1:YLR154C-G;	-1		rDNA	
chrXII	462889	463053	164	**-1,348525169**	N12:462990	9,899	-1:YLR154C-G;	-1		rDNA	
chrXII	462889	463059	170	**-1,381941782**	N12:462990	9,899	-1:YLR154C-G;	-1		rDNA	
chrXII	462889	463061	172	**-1,255925174**	N12:462990	9,899	-1:YLR154C-G;	-1		rDNA	
chrXII	462889	463066	177	**-1,480355991**	N12:462990	9,899	-1:YLR154C-G;	-1		rDNA	
chrXII	462890	463050	160	**-1,094599057**	N12:462990	9,899	-1:YLR154C-G;	-1		rDNA	
chrXII	462890	463059	169	**-1,318562791**	N12:462990	9,899	-1:YLR154C-G;	-1		rDNA	
chrXII	462891	463047	156	**-1,715156071**	N12:462990	9,899	-1:YLR154C-G;	-1		rDNA	
chrXII	462891	463056	165	**-1,730680222**	N12:462990	9,899	-1:YLR154C-G;	-1		rDNA	
chrXII	462891	463061	170	**-1,496767484**	N12:462990	9,899	-1:YLR154C-G;	-1		rDNA	
chrXII	462911	463047	136	**-1,288132838**	N12:462990	9,899	-1:YLR154C-G;	-1		rDNA	
chrXII	462911	463050	139	**-1,273277334**	N12:462990	9,899	-1:YLR154C-G;	-1		rDNA	
chrXII	462911	463051	140	**-1,747682671**	N12:462990	9,899	-1:YLR154C-G;	-1		rDNA	
chrXII	462911	463053	142	**-1,326359236**	N12:462990	9,899	-1:YLR154C-G;	-1		rDNA	
chrXII	462911	463056	145	**-1,149894996**	N12:462990	9,899	-1:YLR154C-G;	-1		rDNA	
chrXII	462911	463062	151	**-1,33135686**	N12:462990	9,899	-1:YLR154C-G;	-1		rDNA	
chrXII	462911	463099	188	**-1,397153673**	N12:462990	9,899	-1:YLR154C-G;	-1		rDNA	
chrXII	462912	463045	133	**-1,27733178**	N12:462990	9,899	-1:YLR154C-G;	-1		rDNA	
chrXII	462912	463048	136	**-1,366103625**	N12:462990	9,899	-1:YLR154C-G;	-1		rDNA	
chrXII	462912	463051	139	**-1,067048979**	N12:462990	9,899	-1:YLR154C-G;	-1		rDNA	
chrXII	462912	463053	141	**-1,198634693**	N12:462990	9,899	-1:YLR154C-G;	-1		rDNA	
chrXII	462912	463059	147	**-1,420418388**	N12:462990	9,899	-1:YLR154C-G;	-1		rDNA	
chrXII	462918	463045	127	**-0,928699495**	N12:462990	9,899	-1:YLR154C-G;	-1		rDNA	
chrXII	462918	463048	130	**-1,189890733**	N12:462990	9,899	-1:YLR154C-G;	-1		rDNA	
chrXII	462918	463051	133	**-1,129095014**	N12:462990	9,899	-1:YLR154C-G;	-1		rDNA	
chrXII	462918	463053	135	**-1,200000469**	N12:462990	9,899	-1:YLR154C-G;	-1		rDNA	
chrXII	462918	463059	141	**-1,332478689**	N12:462990	9,899	-1:YLR154C-G;	-1		rDNA	
chrXII	462919	463048	129	**-1,151413803**	N12:462990	9,899	-1:YLR154C-G;	-1		rDNA	
chrXII	462919	463050	131	**-1,021000708**	N12:462990	9,899	-1:YLR154C-G;	-1		rDNA	
chrXII	462919	463051	132	**-1,303583622**	N12:462990	9,899	-1:YLR154C-G;	-1		rDNA	
chrXII	462919	463056	137	**-1,133186046**	N12:462990	9,899	-1:YLR154C-G;	-1		rDNA	
chrXII	462919	463059	140	**-1,469927096**	N12:462990	9,899	-1:YLR154C-G;	-1		rDNA	
chrXII	462919	463062	143	**-1,069947045**	N12:462990	9,899	-1:YLR154C-G;	-1		rDNA	
chrXII	462919	463066	147	**-1,348886903**	N12:462990	9,899	-1:YLR154C-G;	-1		rDNA	
chrXII	462919	463074	155	**-1,636297045**	N12:462990	9,899	-1:YLR154C-G;	-1		rDNA	
chrXII	462919	463099	180	**-1,172780269**	N12:462990	9,899	-1:YLR154C-G;	-1		rDNA	
chrXII	462920	463045	125	**-0,978291226**	N12:462990	9,899	-1:YLR154C-G;	-1		rDNA	
chrXII	462920	463048	128	**-1,237160695**	N12:462990	9,899	-1:YLR154C-G;	-1		rDNA	
chrXII	462920	463050	130	**-1,274710878**	N12:462990	9,899	-1:YLR154C-G;	-1		rDNA	
chrXII	462920	463051	131	**-1,47007321**	N12:462990	9,899	-1:YLR154C-G;	-1		rDNA	
chrXII	462949	463050	101	**-0,74920353**	N12:462990	9,899	-1:YLR154C-G;	-1		rDNA	
chrXII	462949	463056	107	**-0,426795306**	N12:462990	9,899	-1:YLR154C-G;	-1		rDNA	
chrXII	462949	463099	150	**-1,10618243**	N12:462990	9,899	-1:YLR154C-G;	-1		rDNA	
chrXII	462949	463138	189	**-1,338758548**	N12:462990	9,899	-1:YLR154C-G;	-1		rDNA	
chrXII	462949	463138	189	**-1,338758548**	N12:463152	4,041				rDNA	
chrXII	462988	463095	107	**-0,54216178**	N12:462990	9,899	-1:YLR154C-G;	-1		rDNA	
chrXII	462988	463099	111	**-0,713358026**	N12:462990	9,899	-1:YLR154C-G;	-1		rDNA	
chrXII	462988	463138	150	**-1,131567353**	N12:462990	9,899	-1:YLR154C-G;	-1		rDNA	
chrXII	462988	463138	150	**-1,131567353**	N12:463152	4,041				rDNA	
chrXII	463046	463209	163	**-1,622772572**	N12:463152	4,041				rDNA	
chrXII	463055	463217	162	**-1,379019452**	N12:463152	4,041				rDNA	
chrXII	463059	463216	157	**-1,265069485**	N12:463152	4,041				rDNA	
chrXII	463059	463234	175	**-1,415362578**	N12:463152	4,041				rDNA	
chrXII	463066	463216	150	**-1,953700408**	N12:463152	4,041				rDNA	
chrXII	463066	463234	168	**-1,492294796**	N12:463152	4,041				rDNA	
chrXII	463066	463245	179	**-1,621560895**	N12:463152	4,041				rDNA	
chrXII	463096	463234	138	**-1,544826372**	N12:463152	4,041				rDNA	
chrXII	463096	463245	149	**-1,189996991**	N12:463152	4,041				rDNA	
chrXII	463096	463249	153	**-1,149130461**	N12:463152	4,041				rDNA	
chrXII	463097	463253	156	**-1,238066263**	N12:463152	4,041				rDNA	
chrXII	463101	463234	133	**-1,033142839**	N12:463152	4,041				rDNA	
chrXII	463101	463245	144	**-1,249158536**	N12:463152	4,041				rDNA	
chrXII	463108	463245	137	**-0,986648848**	N12:463152	4,041				rDNA	
chrXII	463108	463260	152	**-1,202182707**	N12:463152	4,041				rDNA	
chrXII	463196	463353	157	**-1,246245367**	N12:463325	17,474				rDNA	
chrXII	463248	463405	157	**-1,49545714**	N12:463325	17,474				rDNA	
chrXII	463257	463404	147	**-1,356702689**	N12:463325	17,474				rDNA	
chrXII	463257	463414	157	**-1,423163998**	N12:463325	17,474				rDNA	
chrXII	463261	463414	153	**-1,628062193**	N12:463325	17,474				rDNA	
chrXII	463268	463414	146	**-1,229070119**	N12:463325	17,474				rDNA	
chrXII	463278	463414	136	**-1,315356249**	N12:463325	17,474				rDNA	
chrXII	463333	463483	150	**-1,184926423**	N12:463325	17,474				rDNA	
chrXII	463333	463483	150	**-1,184926423**	N12:463504	37,477				rDNA	
chrXII	463358	463511	153	**-1,844061972**	N12:463504	37,477				rDNA	
chrXII	463358	463529	171	**-1,254982253**	N12:463504	37,477				rDNA	
chrXII	463381	463529	148	**-1,165241011**	N12:463504	37,477				rDNA	
chrXII	463381	463539	158	**-1,303742568**	N12:463504	37,477				rDNA	
chrXII	463385	463511	126	**-1,085079341**	N12:463504	37,477				rDNA	
chrXII	463385	463529	144	**-1,382706278**	N12:463504	37,477				rDNA	
chrXII	463385	463535	150	**-1,284090886**	N12:463504	37,477				rDNA	
chrXII	463385	463539	154	**-1,981963002**	N12:463504	37,477				rDNA	
chrXII	463410	463573	163	**-1,346463084**	N12:463504	37,477				rDNA	
chrXII	463414	463521	107	**-0,639576809**	N12:463504	37,477				rDNA	
chrXII	463414	463535	121	**-0,716134641**	N12:463504	37,477				rDNA	
chrXII	463414	463539	125	**-1,466607688**	N12:463504	37,477				rDNA	
chrXII	463414	463575	161	**-1,137122627**	N12:463504	37,477				rDNA	
chrXII	463414	463583	169	**-1,499229188**	N12:463504	37,477				rDNA	
chrXII	463463	463603	140	**-1,225107835**	N12:463504	37,477				rDNA	
chrXII	463471	463608	137	**-1,562700146**	N12:463504	37,477				rDNA	
chrXII	463535	463688	153	**-1,555538451**				Overlap <50 bp		rDNA	
chrXII	463535	463689	154	**-1,224153616**				Overlap <50 bp		rDNA	
chrXII	463535	463690	155	**-1,512024304**				Overlap <50 bp		rDNA	

CHR	START	END	L(bp)	ΔLknuc	Nuc ID	Fuzziness	Gene ID	Gene body postion or intergene	Terminal	rDNA	Telomeric
chrXII	463535	463692	157	**-1,214113789**				Overlap <50 bp		rDNA	
chrXII	463535	463704	169	**-1,243470838**				Overlap <50 bp		rDNA	
chrXII	463535	463710	175	**-1,551768098**				Overlap <50 bp		rDNA	
chrXII	463535	463712	177	**-1,323898552**				Overlap <50 bp		rDNA	
chrXII	463541	463689	148	**-1,227753448**				Overlap <50 bp		rDNA	
chrXII	463541	463692	151	**-1,097905797**				Overlap <50 bp		rDNA	
chrXII	463559	463689	130	**-0,777913181**				Overlap <50 bp		rDNA	
chrXII	463559	463704	145	**-1,106538451**				Overlap <50 bp		rDNA	
chrXII	463559	463710	151	**-1,478491197**				Overlap <50 bp		rDNA	
chrXII	463559	463712	153	**-1,158334765**				Overlap <50 bp		rDNA	
chrXII	463658	463815	157	**-1,339081123**	N12:463786	6,658				rDNA	
chrXII	463658	463852	194	**-1,731037165**	N12:463786	6,658				rDNA	
chrXII	463658	463870	212	**-1,52721217**	N12:463786	6,658				rDNA	
chrXII	463690	463849	159	**-1,284883711**	N12:463786	6,658				rDNA	
chrXII	463690	463870	180	**-1,522182911**	N12:463786	6,658				rDNA	
chrXII	463692	463840	148	**-1,205370905**	N12:463786	6,658				rDNA	
chrXII	463697	463815	118	**-0,831407861**	N12:463786	6,658				rDNA	
chrXII	463697	463849	152	**-1,387924403**	N12:463786	6,658				rDNA	
chrXII	463697	463852	155	**-1,318906556**	N12:463786	6,658				rDNA	
chrXII	463697	463861	164	**-1,259434206**	N12:463786	6,658				rDNA	
chrXII	463697	463870	173	**-1,334837966**	N12:463786	6,658				rDNA	
chrXII	463699	463849	150	**-1,088250816**	N12:463786	6,658				rDNA	
chrXII	463707	463849	142	**-1,071534672**	N12:463786	6,658				rDNA	
chrXII	463707	463852	145	**-1,527546399**	N12:463786	6,658				rDNA	
chrXII	463707	463861	154	**-1,223691861**	N12:463786	6,658				rDNA	
chrXII	463707	463870	163	**-1,25052542**	N12:463786	6,658				rDNA	
chrXII	463713	463849	136	**-1,233972975**	N12:463786	6,658				rDNA	
chrXII	463713	463852	139	**-1,465489107**	N12:463786	6,658				rDNA	
chrXII	463713	463861	148	**-1,367426781**	N12:463786	6,658				rDNA	
chrXII	463713	463870	157	**-1,30642325**	N12:463786	6,658				rDNA	
chrXII	463713	463909	196	**-1,482773162**	N12:463786	6,658				rDNA	
chrXII	463724	463883	159	**-1,093610181**	N12:463786	6,658				rDNA	
chrXII	463729	463879	150	**-1,357274644**	N12:463786	6,658				rDNA	
chrXII	463763	463849	86	**-0,441157983**	N12:463786	6,658				rDNA	
chrXII	463763	463870	107	**-0,576000587**	N12:463786	6,658				rDNA	
chrXII	463763	463909	146	**-1,198370929**	N12:463786	6,658				rDNA	
chrXII	463860	463991	131	**-1,146460514**	N12:463954	7,071				rDNA	
chrXII	463862	464000	138	**-1,064057189**	N12:463954	7,071				rDNA	
chrXII	463862	464023	161	**-1,151862236**	N12:463954	7,071				rDNA	
chrXII	463870	464023	153	**-1,481560325**	N12:463954	7,071				rDNA	
chrXII	463878	464033	155	**-1,319954406**	N12:463954	7,071				rDNA	
chrXII	463884	464023	139	**-1,054586869**	N12:463954	7,071				rDNA	
chrXII	463974	464128	154	**-0,837325318**	N12:463954	7,071				rDNA	
chrXII	463974	464128	154	**-0,837325318**	N12:464114	7,778				rDNA	
chrXII	464001	464158	157	**-1,164822956**	N12:464114	7,778				rDNA	
chrXII	464013	464160	147	**-1,138127981**	N12:464114	7,778				rDNA	
chrXII	464013	464166	153	**-1,678143929**	N12:464114	7,778				rDNA	
chrXII	464038	464166	128	**-1,291466261**	N12:464114	7,778				rDNA	
chrXII	464038	464211	173	**-1,459496678**	N12:464114	7,778				rDNA	
chrXII	464057	464224	167	**-1,471343759**	N12:464114	7,778				rDNA	
chrXII	464069	464210	141	**-1,048842111**	N12:464114	7,778				rDNA	
chrXII	464070	464160	90	**-0,785887954**	N12:464114	7,778				rDNA	
chrXII	464070	464166	96	**-1,158013946**	N12:464114	7,778				rDNA	
chrXII	464070	464210	140	**-1,143743918**	N12:464114	7,778				rDNA	
chrXII	464070	464211	141	**-1,302584909**	N12:464114	7,778				rDNA	
chrXII	464070	464213	143	**-1,226673522**	N12:464114	7,778				rDNA	
chrXII	464101	464269	168	**-1,251300944**	N12:464114	7,778				rDNA	
chrXII	464101	464269	168	**-1,251300944**	N12:464279	14,142				rDNA	
chrXII	464132	464301	169	**-1,649425781**	N12:464114	7,778				rDNA	
chrXII	464132	464301	169	**-1,649425781**	N12:464279	14,142				rDNA	
chrXII	464137	464269	132	**-1,067927668**	N12:464114	7,778				rDNA	
chrXII	464137	464269	132	**-1,067927668**	N12:464279	14,142				rDNA	
chrXII	464152	464303	151	**-1,217171292**	N12:464279	14,142				rDNA	
chrXII	464195	464345	150	**-1,671626346**	N12:464279	14,142				rDNA	
chrXII	464205	464360	155	**-1,247577908**	N12:464279	14,142				rDNA	
chrXII	464232	464406	174	**-1,418381264**	N12:464279	14,142				rDNA	
chrXII	464232	464406	174	**-1,418381264**	N12:464427	22,679				rDNA	
chrXII	464233	464360	127	**-1,299187093**	N12:464279	14,142				rDNA	
chrXII	464233	464377	144	**-1,119894183**	N12:464279	14,142				rDNA	
chrXII	464233	464380	147	**-1,119849088**	N12:464279	14,142				rDNA	
chrXII	464233	464382	149	**-1,093330695**	N12:464279	14,142				rDNA	
chrXII	464233	464386	153	**-1,240075175**	N12:464279	14,142				rDNA	
chrXII	464234	464377	143	**-1,582792225**	N12:464279	14,142				rDNA	
chrXII	464234	464380	146	**-1,27963756**	N12:464279	14,142				rDNA	
chrXII	464234	464382	148	**-1,251369002**	N12:464279	14,142				rDNA	
chrXII	464235	464377	142	**-1,225883176**	N12:464279	14,142				rDNA	
chrXII	464235	464380	145	**-1,403622677**	N12:464279	14,142				rDNA	
chrXII	464235	464382	147	**-1,50302983**	N12:464279	14,142				rDNA	
chrXII	464235	464386	151	**-1,136642671**	N12:464279	14,142				rDNA	
chrXII	464266	464430	164	**-1,268995931**	N12:464279	14,142				rDNA	
chrXII	464266	464430	164	**-1,268995931**	N12:464427	22,679				rDNA	
chrXII	464275	464426	151	**-1,323866282**	N12:464279	14,142				rDNA	
chrXII	464275	464426	151	**-1,323866282**	N12:464427	22,679				rDNA	
chrXII	464319	464475	156	**-1,689774551**	N12:464427	22,679				rDNA	
chrXII	464354	464503	149	**-1,459627491**	N12:464427	22,679				rDNA	
chrXII	464358	464532	174	**-1,774508688**	N12:464427	22,679				rDNA	
chrXII	464384	464518	134	**-1,635147555**	N12:464427	22,679				rDNA	
chrXII	464391	464538	147	**-1,402020385**	N12:464427	22,679				rDNA	
chrXII	464392	464537	145	**-1,630022925**	N12:464427	22,679				rDNA	
chrXII	464415	464560	145	**-1,360717059**	N12:464427	22,679				rDNA	
chrXII	464434	464584	150	**-1,280217243**	N12:464427	22,679				rDNA	
chrXII	464504	464649	145	**-1,101904191**	N12:464616	19				rDNA	
chrXII	464504	464668	164	**-1,130510575**	N12:464616	19				rDNA	
chrXII	464504	464686	182	**-1,314977046**	N12:464616	19				rDNA	
chrXII	464514	464649	135	**-1,150238815**	N12:464616	19				rDNA	
chrXII	464514	464668	154	**-1,175799933**	N12:464616	19				rDNA	

CHR	START	END	L(bp)	ΔLknuc	Nuc ID	Fuzziness	Gene ID	Gene body postion or intergene	Terminal	rDNA	Telomeric
chrXII	464514	464686	172	**-1,202026112**	N12:464616	19				rDNA	
chrXII	464534	464640	106	**-0,710188086**	N12:464616	19				rDNA	
chrXII	464534	464649	115	**-0,452673507**	N12:464616	19				rDNA	
chrXII	464534	464668	134	**-0,633933501**	N12:464616	19				rDNA	
chrXII	464534	464686	152	**-1,339918936**	N12:464616	19				rDNA	
chrXII	464547	464649	102	**-0,475639868**	N12:464616	19				rDNA	
chrXII	464547	464686	139	**-1,411086837**	N12:464616	19				rDNA	
chrXII	464547	464697	150	**-0,648494907**	N12:464616	19				rDNA	
chrXII	464547	464705	158	**-1,188623716**	N12:464616	19				rDNA	
chrXII	464547	464706	159	**-1,479829738**	N12:464616	19				rDNA	
chrXII	464553	464708	155	**-1,267859493**	N12:464616	19				rDNA	
chrXII	464559	464706	147	**-1,026528444**	N12:464616	19				rDNA	
chrXII	464728	464888	160	**-1,757799016**	N12:464796	30,199				rDNA	
chrXII	464838	465007	169	**-1,64829069**	N12:464962	52,647				rDNA	
chrXII	464884	465032	148	**-1,358902718**	N12:464962	52,647				rDNA	
chrXII	464886	465026	140	**-1,06336186**	N12:464962	52,647				rDNA	
chrXII	464920	465095	175	**-1,79108638**	N12:464962	52,647				rDNA	
chrXII	465006	465158	152	**-1,399748791**	N12:465169	29,501				rDNA	
chrXII	465040	465204	164	**-1,688477357**	N12:465169	29,501				rDNA	
chrXII	465055	465176	121	**-1,350886964**	N12:465169	29,501				rDNA	
chrXII	465063	465223	160	**-1,34237679**	N12:465169	29,501				rDNA	
chrXII	465159	465310	151	**-1,212476488**	N12:465169	29,501				rDNA	
chrXII	465159	465342	183	**-1,319695984**	N12:465169	29,501				rDNA	
chrXII	465159	465342	183	**-1,319695984**	N12:465356	20,664				rDNA	
chrXII	465159	465348	189	**-1,395346351**	N12:465169	29,501				rDNA	
chrXII	465159	465348	189	**-1,395346351**	N12:465356	20,664				rDNA	
chrXII	465159	465357	198	**-1,265115529**	N12:465169	29,501				rDNA	
chrXII	465159	465357	198	**-1,265115529**	N12:465356	20,664				rDNA	
chrXII	465179	465310	131	**-0,906452825**	N12:465169	29,501				rDNA	
chrXII	465182	465312	130	**-1,089315353**	N12:465169	29,501				rDNA	
chrXII	465193	465310	117	**-0,64626942**				Overlap <50 bp		rDNA	
chrXII	465193	465312	119	**-0,556159197**				Overlap <50 bp		rDNA	
chrXII	465193	465342	149	**-1,273839188**	N12:465356	20,664				rDNA	
chrXII	465193	465348	155	**-1,253516586**	N12:465356	20,664				rDNA	
chrXII	465193	465357	164	**-1,248167912**	N12:465356	20,664				rDNA	
chrXII	465200	465352	152	**-1,266798765**	N12:465356	20,664				rDNA	
chrXII	465201	465342	141	**-0,813038446**	N12:465356	20,664				rDNA	
chrXII	465201	465345	144	**-0,950333857**	N12:465356	20,664				rDNA	
chrXII	465201	465351	150	**-1,035174987**	N12:465356	20,664				rDNA	
chrXII	465203	465310	107	**-0,313755689**				Overlap <50 bp		rDNA	
chrXII	465204	465342	138	**-1,206626675**	N12:465356	20,664				rDNA	
chrXII	465204	465348	144	**-1,010079394**	N12:465356	20,664				rDNA	
chrXII	465204	465352	148	**-1,261860582**	N12:465356	20,664				rDNA	
chrXII	465204	465357	153	**-1,036499032**	N12:465356	20,664				rDNA	
chrXII	465204	465403	199	**-1,68084809**	N12:465356	20,664				rDNA	
chrXII	465204	465418	214	**-1,77160931**	N12:465356	20,664				rDNA	
chrXII	465217	465310	93	**-0,34965455**				Overlap <50 bp		rDNA	
chrXII	465217	465342	125	**-0,75856945**	N12:465356	20,664				rDNA	
chrXII	465217	465348	131	**-0,909228921**	N12:465356	20,664				rDNA	
chrXII	465217	465357	140	**-0,887234027**	N12:465356	20,664				rDNA	
chrXII	465221	465352	131	**-0,76609552**	N12:465356	20,664				rDNA	
chrXII	465221	465375	154	**-1,350621696**	N12:465356	20,664				rDNA	
chrXII	465221	465380	159	**-1,353591856**	N12:465356	20,664				rDNA	
chrXII	465221	465403	182	**-1,70769705**	N12:465356	20,664				rDNA	
chrXII	465252	465352	100	**-0,578241955**	N12:465356	20,664				rDNA	
chrXII	465252	465380	128	**-0,888432155**	N12:465356	20,664				rDNA	
chrXII	465252	465403	151	**-1,219558956**	N12:465356	20,664				rDNA	
chrXII	465252	465418	166	**-1,441285825**	N12:465356	20,664				rDNA	
chrXII	465257	465427	170	**-1,316955745**	N12:465356	20,664				rDNA	
chrXII	465261	465352	91	**-0,718600986**	N12:465356	20,664				rDNA	
chrXII	465261	465403	142	**-1,381703957**	N12:465356	20,664				rDNA	
chrXII	465261	465418	157	**-1,360370725**	N12:465356	20,664				rDNA	
chrXII	465261	465470	209	**-1,522324322**	N12:465356	20,664				rDNA	
chrXII	465318	465403	85	**-0,607218614**	N12:465356	20,664				rDNA	
chrXII	465318	465418	100	**-0,692373553**	N12:465356	20,664				rDNA	
chrXII	465318	465447	129	**-1,066165389**	N12:465356	20,664				rDNA	
chrXII	465318	465469	151	**-1,745366324**	N12:465356	20,664				rDNA	
chrXII	465318	465470	152	**-1,304533325**	N12:465356	20,664				rDNA	
chrXII	465318	465473	155	**-1,213890461**	N12:465356	20,664				rDNA	
chrXII	465318	465486	168	**-1,301002233**	N12:465356	20,664				rDNA	
chrXII	465318	465524	206	**-1,599876434**	N12:465356	20,664				rDNA	
chrXII	465318	465524	206	**-1,599876434**	N12:465541	35,303				rDNA	
chrXII	465323	465470	147	**-1,614559164**	N12:465356	20,664				rDNA	
chrXII	465324	465470	146	**-1,61665492**	N12:465356	20,664				rDNA	
chrXII	465324	465486	162	**-1,216629237**	N12:465356	20,664				rDNA	
chrXII	465331	465470	139	**-1,311337704**	N12:465356	20,664				rDNA	
chrXII	465331	465473	142	**-1,189451823**	N12:465356	20,664				rDNA	
chrXII	465350	465456	106	**-0,83709612**	N12:465356	20,664				rDNA	
chrXII	465350	465457	107	**-0,852792116**	N12:465356	20,664				rDNA	
chrXII	465350	465458	108	**-1,050623833**	N12:465356	20,664				rDNA	
chrXII	465350	465470	120	**-1,229149517**	N12:465356	20,664				rDNA	
chrXII	465350	465473	123	**-1,193061194**	N12:465356	20,664				rDNA	
chrXII	465350	465486	136	**-1,402680295**	N12:465356	20,664				rDNA	
chrXII	465350	465524	174	**-1,460260563**	N12:465356	20,664				rDNA	
chrXII	465350	465524	174	**-1,460260563**	N12:465541	35,303				rDNA	
chrXII	465350	465556	206	**-1,805916871**	N12:465356	20,664				rDNA	
chrXII	465350	465556	206	**-1,805916871**	N12:465541	35,303				rDNA	
chrXII	465358	465523	165	**-1,007146733**	N12:465356	20,664				rDNA	
chrXII	465358	465523	165	**-1,007146733**	N12:465541	35,303				rDNA	
chrXII	465377	465470	93	**-0,569053498**	N12:465356	20,664				rDNA	
chrXII	465377	465484	107	**-0,878121394**	N12:465356	20,664				rDNA	
chrXII	465377	465523	146	**-1,039561969**	N12:465356	20,664				rDNA	
chrXII	465377	465523	146	**-1,039561969**	N12:465541	35,303				rDNA	
chrXII	465377	465524	147	**-1,210166714**	N12:465356	20,664				rDNA	
chrXII	465377	465524	147	**-1,210166714**	N12:465541	35,303				rDNA	
chrXII	465377	465556	179	**-1,340352287**	N12:465356	20,664				rDNA	

CHR	START	END	L(bp)	ΔLknuc	Nuc ID	Fuzziness	Gene ID	Gene body postion or intergene	Terminal	rDNA	Telomeric
chrXII	465377	465556	179	-1,340352287	N12:465541	35,303				rDNA	
chrXII	465377	465584	207	-1,712269214	N12:465356	20,664				rDNA	
chrXII	465377	465584	207	-1,712269214	N12:465541	35,303				rDNA	
chrXII	465425	465524	99	-0,650000163	N12:465541	35,303				rDNA	
chrXII	465425	465532	107	-1,238452803	N12:465541	35,303				rDNA	
chrXII	465425	465556	131	-1,075907886	N12:465541	35,303				rDNA	
chrXII	465425	465584	159	-1,1945625	N12:465541	35,303				rDNA	
chrXII	465425	465594	169	-1,371021688	N12:465541	35,303				rDNA	
chrXII	465425	465608	183	-1,877009901	N12:465541	35,303				rDNA	
chrXII	465427	465524	97	-0,73042467	N12:465541	35,303				rDNA	
chrXII	465427	465556	129	-1,243939783	N12:465541	35,303				rDNA	
chrXII	465427	465584	157	-1,640094	N12:465541	35,303				rDNA	
chrXII	465427	465608	181	-2,029628688	N12:465541	35,303				rDNA	
chrXII	465435	465584	149	-1,283628907	N12:465541	35,303				rDNA	
chrXII	465447	465556	109	-0,725427933	N12:465541	35,303				rDNA	
chrXII	465447	465584	137	-1,106094081	N12:465541	35,303				rDNA	
chrXII	465447	465608	161	-1,419102613	N12:465541	35,303				rDNA	
chrXII	465498	465584	86	-1,141204061	N12:465541	35,303				rDNA	
chrXII	465498	465603	105	-1,626306636	N12:465541	35,303				rDNA	
chrXII	465498	465605	107	-1,44440594	N12:465541	35,303				rDNA	
chrXII	465498	465606	108	-1,282178349	N12:465541	35,303				rDNA	
chrXII	465498	465607	109	-1,32173857	N12:465541	35,303				rDNA	
chrXII	465498	465608	110	-1,510691085	N12:465541	35,303				rDNA	
chrXII	465525	465608	83	-1,049290733	N12:465541	35,303				rDNA	
chrXII	465525	465688	163	-1,448472887	N12:465541	35,303				rDNA	
chrXII	465525	465688	163	-1,448472887	N12:465711	28,919				rDNA	
chrXII	465562	465739	177	-1,54386528	N12:465541	35,303				rDNA	
chrXII	465562	465739	177	-1,54386528	N12:465711	28,919				rDNA	
chrXII	465581	465731	150	-1,281580632	N12:465711	28,919				rDNA	
chrXII	465581	465732	151	-1,328742751	N12:465711	28,919				rDNA	
chrXII	465581	465756	175	-1,610495994	N12:465711	28,919				rDNA	
chrXII	465582	465732	150	-1,351958361	N12:465711	28,919				rDNA	
chrXII	465584	465731	147	-1,356467928	N12:465711	28,919				rDNA	
chrXII	465584	465732	148	-1,516457509	N12:465711	28,919				rDNA	
chrXII	465584	465745	161	-1,695353124	N12:465711	28,919				rDNA	
chrXII	465584	465756	172	-1,396825526	N12:465711	28,919				rDNA	
chrXII	465616	465722	106	-0,82411353	N12:465711	28,919				rDNA	
chrXII	465616	465731	115	-0,761810369	N12:465711	28,919				rDNA	
chrXII	465616	465745	129	-1,02369807	N12:465711	28,919				rDNA	
chrXII	465616	465746	130	-1,567618533	N12:465711	28,919				rDNA	
chrXII	465616	465790	174	-1,026305713	N12:465711	28,919				rDNA	
chrXII	465620	465731	111	-0,737739116	N12:465711	28,919				rDNA	
chrXII	465620	465745	125	-1,003039587	N12:465711	28,919				rDNA	
chrXII	465620	465746	126	-0,988387854	N12:465711	28,919				rDNA	
chrXII	465630	465736	106	-0,550861011	N12:465711	28,919				rDNA	
chrXII	465630	465745	115	-0,728664547	N12:465711	28,919				rDNA	
chrXII	465630	465790	160	-1,234164439	N12:465711	28,919				rDNA	
chrXII	465645	465731	86	-0,574702254	N12:465711	28,919				rDNA	
chrXII	465645	465745	100	-0,410819086	N12:465711	28,919				rDNA	
chrXII	465645	465779	134	-1,195662104	N12:465711	28,919				rDNA	
chrXII	465645	465790	145	-1,333502136	N12:465711	28,919				rDNA	
chrXII	465645	465807	162	-0,951768945	N12:465711	28,919				rDNA	
chrXII	465661	465832	171	-1,428454789	N12:465711	28,919				rDNA	
chrXII	465724	465875	151	-1,511976185	N12:465711	28,919				rDNA	
chrXII	465724	465875	151	-1,511976185	N12:465875	14,142				rDNA	
chrXII	465725	465888	163	-1,212443271	N12:465711	28,919				rDNA	
chrXII	465725	465888	163	-1,212443271	N12:465875	14,142				rDNA	
chrXII	465731	465888	157	-1,364958294	N12:465711	28,919				rDNA	
chrXII	465731	465888	157	-1,364958294	N12:465875	14,142				rDNA	
chrXII	465753	465909	156	-1,255174649	N12:465875	14,142				rDNA	
chrXII	465771	465899	128	-1,070703	N12:465875	14,142				rDNA	
chrXII	465771	465918	147	-1,218083451	N12:465875	14,142				rDNA	
chrXII	465786	465940	154	-1,203024075	N12:465875	14,142				rDNA	
chrXII	465790	465899	109	-0,795337195	N12:465875	14,142				rDNA	
chrXII	465790	465940	150	-0,860308771	N12:465875	14,142				rDNA	
chrXII	465903	466057	154	-1,111125655	N12:466028	33,501				rDNA	
chrXII	465917	466024	107	-1,714368733	N12:466028	33,501				rDNA	
chrXII	465917	466027	110	-1,599447913	N12:466028	33,501				rDNA	
chrXII	465917	466057	140	-2,249467514	N12:466028	33,501				rDNA	
chrXII	465931	466040	109	-0,771118229	N12:466028	33,501				rDNA	
chrXII	465932	466040	108	-1,003861473	N12:466028	33,501				rDNA	
chrXII	465932	466041	109	-1,116046151	N12:466028	33,501				rDNA	
chrXII	465934	466027	93	-1,405374122	N12:466028	33,501				rDNA	
chrXII	465934	466057	123	-2,044151178	N12:466028	33,501				rDNA	
chrXII	465940	466096	156	-1,50789203	N12:466028	33,501				rDNA	
chrXII	465941	466048	107	-1,064341363	N12:466028	33,501				rDNA	
chrXII	465941	466057	116	-1,233141412	N12:466028	33,501				rDNA	
chrXII	465941	466097	156	-1,615176487	N12:466028	33,501				rDNA	
chrXII	465954	466057	103	-0,777525185	N12:466028	33,501				rDNA	
chrXII	465954	466108	154	-1,493198002	N12:466028	33,501				rDNA	
chrXII	466075	466241	166	-1,516575912	N12:466190	42,899				rDNA	
chrXII	466105	466258	153	-1,528878287	N12:466190	42,899				rDNA	
chrXII	466121	466255	134	-1,402794148	N12:466190	42,899				rDNA	
chrXII	466121	466264	143	-1,429706159	N12:466190	42,899				rDNA	
chrXII	466121	466267	146	-1,255345988	N12:466190	42,899				rDNA	
chrXII	466121	466277	156	-1,627858752	N12:466190	42,899				rDNA	
chrXII	466127	466264	137	-1,273071282	N12:466190	42,899				rDNA	
chrXII	466127	466277	150	-1,4800357	N12:466190	42,899				rDNA	
chrXII	466142	466303	161	-1,812771488	N12:466190	42,899				rDNA	
chrXII	466158	466264	106	-1,822864991	N12:466190	42,899				rDNA	
chrXII	466159	466264	105	-1,14603945	N12:466190	42,899				rDNA	
chrXII	466159	466342	183	-2,339348407	N12:466190	42,899				rDNA	
chrXII	466159	466342	183	-2,339348407	N12:466349	34,356				rDNA	
chrXII	466184	466344	160	-1,438213629	N12:466190	42,899				rDNA	
chrXII	466184	466344	160	-1,438213629	N12:466349	34,356				rDNA	
chrXII	466207	466337	130	-1,530726957	N12:466190	42,899				rDNA	

CHR	START	END	L(bp)	ΔLknuc	Nuc ID	Fuzziness	Gene ID	Gene body postion or intergene	Terminal	rDNA	Telomeric
chrXII	466207	466337	130	**-1,530726957**	N12:466349	34,356				rDNA	
chrXII	466220	466354	134	**-0,91864188**	N12:466349	34,356				rDNA	
chrXII	466252	466385	133	**-1,029702167**	N12:466349	34,356				rDNA	
chrXII	466277	466442	165	**-1,50174518**	N12:466349	34,356				rDNA	
chrXII	466277	466449	172	**-1,676271405**	N12:466349	34,356				rDNA	
chrXII	466280	466385	105	**-0,599588757**	N12:466349	34,356				rDNA	
chrXII	466280	466416	136	**-1,051930252**	N12:466349	34,356				rDNA	
chrXII	466280	466453	173	**-1,418004259**	N12:466349	34,356				rDNA	
chrXII	466289	466442	153	**-1,237834909**	N12:466349	34,356				rDNA	
chrXII	466289	466449	160	**-1,551531932**	N12:466349	34,356				rDNA	
chrXII	466290	466385	95	**-0,739626473**	N12:466349	34,356				rDNA	
chrXII	466290	466416	126	**-0,900497292**	N12:466349	34,356				rDNA	
chrXII	466290	466449	159	**-1,615122146**	N12:466349	34,356				rDNA	
chrXII	466290	466453	163	**-1,342215939**	N12:466349	34,356				rDNA	
chrXII	466290	466454	164	**-1,529692835**	N12:466349	34,356				rDNA	
chrXII	466291	466449	158	**-1,359058459**	N12:466349	34,356				rDNA	
chrXII	466291	466453	162	**-1,516868428**	N12:466349	34,356				rDNA	
chrXII	466324	466453	129	**-0,973504824**	N12:466349	34,356				rDNA	
chrXII	466324	466454	130	**-0,902645381**	N12:466349	34,356				rDNA	
chrXII	466335	466483	148	**-1,162806442**	N12:466349	34,356				rDNA	
chrXII	466349	466502	153	**-1,131025452**	N12:466349	34,356				rDNA	
chrXII	466351	466500	149	**-1,20776272**	N12:466349	34,356				rDNA	
chrXII	466356	466449	93	**-0,925541919**	N12:466349	34,356				rDNA	
chrXII	466356	466483	127	**-0,921694478**	N12:466349	34,356				rDNA	
chrXII	466356	466500	144	**-1,191233807**	N12:466349	34,356				rDNA	
chrXII	466356	466502	146	**-1,211866682**	N12:466349	34,356				rDNA	
chrXII	466364	466500	136	**-1,111816631**	N12:466349	34,356				rDNA	
chrXII	466367	466532	165	**-1,504521289**	N12:466349	34,356				rDNA	
chrXII	466367	466538	171	**-1,284897755**	N12:466349	34,356				rDNA	
chrXII	466385	466532	147	**-1,268181282**				Overlap <50 bp		rDNA	
chrXII	466385	466538	153	**-0,727811556**				Overlap <50 bp		rDNA	
chrXII	466392	466502	110	**-0,774761812**				Overlap <50 bp		rDNA	
chrXII	466392	466544	152	**-1,160492306**				Overlap <50 bp		rDNA	
chrXII	466403	466500	97	**-0,644377544**				Overlap <50 bp		rDNA	
chrXII	466403	466502	99	**0,002228858**				Overlap <50 bp		rDNA	
chrXII	466403	466544	141	**-0,859246023**				Overlap <50 bp		rDNA	
chrXII	466403	466559	156	**-1,279564773**				Overlap <50 bp		rDNA	
chrXII	466432	466521	89	**-1,434349753**				Overlap <50 bp		rDNA	
chrXII	466432	466523	91	**-1,305883336**				Overlap <50 bp		rDNA	
chrXII	466432	466527	95	**-1,68279282**				Overlap <50 bp		rDNA	
chrXII	466432	466528	96	**-1,402346336**				Overlap <50 bp		rDNA	
chrXII	466432	466530	98	**-1,596597643**				Overlap <50 bp		rDNA	
chrXII	466432	466531	99	**-1,587053606**				Overlap <50 bp		rDNA	
chrXII	466432	466532	100	**-1,381212083**				Overlap <50 bp		rDNA	
chrXII	466432	466534	102	**-1,649766327**				Overlap <50 bp		rDNA	
chrXII	466432	466538	106	**-1,022076878**				Overlap <50 bp		rDNA	
chrXII	466432	466539	107	**-0,701497635**				Overlap <50 bp		rDNA	
chrXII	466474	466613	139	**-1,367357428**	N12:466630	9,192				rDNA	
chrXII	466474	466618	144	**-1,183456413**	N12:466630	9,192				rDNA	
chrXII	466497	466613	116	**-1,050074927**	N12:466630	9,192				rDNA	
chrXII	466497	466618	121	**-0,66238937**	N12:466630	9,192				rDNA	
chrXII	466497	466667	170	**-1,445557855**	N12:466630	9,192				rDNA	
chrXII	466507	466613	106	**-1,419414891**	N12:466630	9,192				rDNA	
chrXII	466509	466613	104	**-1,758094708**	N12:466630	9,192				rDNA	
chrXII	466509	466618	109	**-0,736531972**	N12:466630	9,192				rDNA	
chrXII	466557	466700	143	**-0,989660603**	N12:466630	9,192				rDNA	
chrXII	466557	466704	147	**-1,304748707**	N12:466630	9,192				rDNA	
chrXII	466557	466708	151	**-1,183837733**	N12:466630	9,192				rDNA	
chrXII	466557	466709	152	**-0,978047549**	N12:466630	9,192				rDNA	
chrXII	466574	466729	155	**-1,754345084**	N12:466630	9,192				rDNA	
chrXII	466577	466730	153	**-1,200108241**	N12:466630	9,192				rDNA	
chrXII	466611	466736	125	**-1,525849683**	N12:466630	9,192				rDNA	
chrXII	466690	466843	153	**-1,093769123**	N12:466825	26				rDNA	
chrXII	466706	466873	167	**-1,763604263**	N12:466825	26				rDNA	
chrXII	466706	466878	172	**-1,690582062**	N12:466825	26				rDNA	
chrXII	466737	466873	136	**-1,064523378**	N12:466825	26				rDNA	
chrXII	466737	466876	139	**-0,748869281**	N12:466825	26				rDNA	
chrXII	466737	466878	141	**-1,458186302**	N12:466825	26				rDNA	
chrXII	466743	466900	157	**-0,946379516**	N12:466825	26				rDNA	
chrXII	466757	466910	153	**-1,354696362**	N12:466825	26				rDNA	
chrXII	466757	466924	167	**-1,635540173**	N12:466825	26				rDNA	
chrXII	466771	466894	123	**-1,245787505**	N12:466825	26				rDNA	
chrXII	466804	466956	152	**-1,352999552**	N12:466825	26				rDNA	
chrXII	466940	467096	156	**-1,171158002**	N12:467036	20,033				rDNA	
chrXII	466947	467098	151	**-1,463342238**	N12:467036	20,033				rDNA	
chrXII	466953	467111	158	**-1,002309502**	N12:467036	20,033				rDNA	
chrXII	466957	467090	133	**-0,806778547**	N12:467036	20,033				rDNA	
chrXII	467009	467160	151	**-1,278850459**	N12:467036	20,033				rDNA	
chrXII	467063	467189	126	**-0,863552457**				Overlap <50 bp		rDNA	
chrXII	467082	467240	158	**-1,362117349**				Overlap <50 bp		rDNA	
chrXII	467082	467241	159	**-1,443762402**				Overlap <50 bp		rDNA	
chrXII	467086	467196	110	**-0,574687176**				Overlap <50 bp		rDNA	
chrXII	467086	467238	152	**-1,236715493**				Overlap <50 bp		rDNA	
chrXII	467087	467238	151	**-1,199358749**				Overlap <50 bp		rDNA	
chrXII	467087	467240	153	**-1,284611933**				Overlap <50 bp		rDNA	
chrXII	467096	467196	100	**-0,689144471**				Overlap <50 bp		rDNA	
chrXII	467096	467247	151	**-1,014330747**				Overlap <50 bp		rDNA	
chrXII	467113	467196	83	**-0,871964081**				Overlap <50 bp		rDNA	
chrXII	467113	467256	143	**-1,202291577**				Overlap <50 bp		rDNA	
chrXII	467120	467226	106	**-0,474446778**				Overlap <50 bp		rDNA	
chrXII	467120	467240	120	**-1,267839735**				Overlap <50 bp		rDNA	
chrXII	467120	467274	154	**-1,453196088**				Overlap <50 bp		rDNA	
chrXII	467123	467254	131	**-0,968564182**				Overlap <50 bp		rDNA	
chrXII	467162	467304	142	**-1,511607052**	N12:467327	20,207				rDNA	
chrXII	467192	467274	82	**-0,616547545**				Overlap <50 bp		rDNA	
chrXII	467192	467343	151	**-1,223140886**	N12:467327	20,207				rDNA	

CHR	START	END	L(bp)	ΔLknuc	Nuc ID	Fuzziness	Gene ID	Gene body postion or intergene	Terminal	rDNA	Telomeric
chrXII	467216	467364	148	**-1,373866419**	N12:467327	20,207				rDNA	
chrXII	467224	467368	144	**-1,120404641**	N12:467327	20,207				rDNA	
chrXII	467264	467414	150	**-1,202163882**	N12:467327	20,207				rDNA	
chrXII	467267	467430	163	**-1,116152514**	N12:467327	20,207				rDNA	
chrXII	467271	467409	138	**-0,790704365**	N12:467327	20,207				rDNA	
chrXII	467271	467414	143	**-1,207333239**	N12:467327	20,207				rDNA	
chrXII	467273	467432	159	**-1,25419807**	N12:467327	20,207				rDNA	
chrXII	467280	467428	148	**-1,356606866**	N12:467327	20,207				rDNA	
chrXII	467303	467458	155	**-1,376416837**	N12:467327	20,207				rDNA	
chrXII	467303	467459	156	**-1,409747579**	N12:467327	20,207				rDNA	
chrXII	467304	467459	155	**-1,1902023**	N12:467327	20,207				rDNA	
chrXII	467314	467414	100	**-0,709148306**	N12:467327	20,207				rDNA	
chrXII	467314	467459	145	**-1,219511077**	N12:467327	20,207				rDNA	
chrXII	467314	467464	150	**-1,42553872**	N12:467327	20,207				rDNA	
chrXII	467314	467487	173	**-1,463968457**	N12:467327	20,207				rDNA	
chrXII	467316	467464	148	**-1,124212653**	N12:467327	20,207				rDNA	
chrXII	467328	467458	130	**-1,048002428**	N12:467327	20,207				rDNA	
chrXII	467328	467489	161	**-1,166966948**	N12:467327	20,207				rDNA	
chrXII	467332	467459	127	**-0,817150859**	N12:467327	20,207				rDNA	
chrXII	467332	467487	155	**-1,005391883**	N12:467327	20,207				rDNA	
chrXII	467341	467487	146	**-1,422135006**	N12:467327	20,207				rDNA	
chrXII	467341	467517	176	**-1,415628495**	N12:467327	20,207				rDNA	
chrXII	467428	467588	160	**-1,024847051**	N12:467582	21,213				rDNA	
chrXII	467434	467588	154	**-1,246197816**	N12:467582	21,213				rDNA	
chrXII	467435	467598	163	**-1,043102213**	N12:467582	21,213				rDNA	
chrXII	467456	467609	153	**-1,406761565**	N12:467582	21,213				rDNA	
chrXII	467475	467629	154	**-1,420864087**	N12:467582	21,213				rDNA	
chrXII	467493	467630	137	**-0,946015819**	N12:467582	21,213				rDNA	
chrXII	467493	467645	152	**-1,214933813**	N12:467582	21,213				rDNA	
chrXII	467496	467660	164	**-1,293725572**	N12:467582	21,213				rDNA	
chrXII	467502	467630	128	**-1,023597402**	N12:467582	21,213				rDNA	
chrXII	467510	467630	120	**-0,956374307**	N12:467582	21,213				rDNA	
chrXII	467510	467645	135	**-1,544213338**	N12:467582	21,213				rDNA	
chrXII	467510	467667	157	**-1,535756594**	N12:467582	21,213				rDNA	
chrXII	467511	467669	158	**-1,43830926**	N12:467582	21,213				rDNA	
chrXII	467515	467649	134	**-0,869686564**	N12:467582	21,213				rDNA	
chrXII	467532	467638	106	**-0,647616736**	N12:467582	21,213				rDNA	
chrXII	467556	467724	168	**-1,466857542**	N12:467582	21,213				rDNA	
chrXII	467645	467795	150	**-1,76462558**				Overlap <50 bp		rDNA	
chrXII	467645	467796	151	**-1,535740651**				Overlap <50 bp		rDNA	
chrXII	467883	468037	154	**-1,509928375**	N12:467959	20,928				rDNA	
chrXII	467883	468038	155	**-1,433762196**	N12:467959	20,928				rDNA	
chrXII	467883	468049	166	**-1,071629874**	N12:467959	20,928				rDNA	
chrXII	467887	468049	162	**-1,359411744**	N12:467959	20,928				rDNA	
chrXII	467905	468037	132	**-1,242353329**	N12:467959	20,928				rDNA	
chrXII	467905	468038	133	**-1,314191197**	N12:467959	20,928				rDNA	
chrXII	467905	468049	144	**-1,251132614**	N12:467959	20,928				rDNA	
chrXII	467906	468037	131	**-1,101002414**	N12:467959	20,928				rDNA	
chrXII	467906	468038	132	**-1,018304977**	N12:467959	20,928				rDNA	
chrXII	467906	468049	143	**-1,234558674**	N12:467959	20,928				rDNA	
chrXII	467906	468050	144	**-0,869977729**	N12:467959	20,928				rDNA	
chrXII	467907	468037	130	**-1,148690398**	N12:467959	20,928				rDNA	
chrXII	467907	468038	131	**-1,309637707**	N12:467959	20,928				rDNA	
chrXII	467907	468049	142	**-1,185390092**	N12:467959	20,928				rDNA	
chrXII	467983	468139	156	**-0,818398013**	N12:468113	0				rDNA	
chrXII	468039	468185	146	**-1,36906816**	N12:468113	0				rDNA	
chrXII	468045	468181	136	**-1,282772836**	N12:468113	0				rDNA	
chrXII	468045	468185	140	**-0,80249428**	N12:468113	0				rDNA	
chrXII	468047	468203	156	**-1,708220607**	N12:468113	0				rDNA	
chrXII	468050	468139	89	**-0,565841931**	N12:468113	0				rDNA	
chrXII	468050	468175	125	**-1,094304398**	N12:468113	0				rDNA	
chrXII	468050	468176	126	**-1,04361809**	N12:468113	0				rDNA	
chrXII	468050	468180	130	**-1,185329644**	N12:468113	0				rDNA	
chrXII	468050	468181	131	**-1,240440179**	N12:468113	0				rDNA	
chrXII	468050	468184	134	**-1,2395425**	N12:468113	0				rDNA	
chrXII	468050	468185	135	**-1,208465711**	N12:468113	0				rDNA	
chrXII	468050	468203	153	**-1,40053062**	N12:468113	0				rDNA	
chrXII	468070	468181	111	**-0,893178776**	N12:468113	0				rDNA	
chrXII	468070	468184	114	**-0,724066852**	N12:468113	0				rDNA	
chrXII	468070	468203	133	**-1,099181137**	N12:468113	0				rDNA	
chrXII	468187	468283	96	**-1,300706257**	N12:468275	25,423				rDNA	
chrXII	468226	468399	173	**-1,501027388**	N12:468275	25,423				rDNA	
chrXII	468226	468402	176	**-1,563694439**	N12:468275	25,423				rDNA	
chrXII	468232	468399	167	**-1,465487332**	N12:468275	25,423				rDNA	
chrXII	468232	468402	170	**-1,570627036**	N12:468275	25,423				rDNA	
chrXII	468235	468370	135	**-1,071895468**	N12:468275	25,423				rDNA	
chrXII	468235	468399	164	**-1,428588157**	N12:468275	25,423				rDNA	
chrXII	468303	468465	162	**-1,255920819**	N12:468484	36,77				rDNA	
chrXII	468322	468465	143	**-1,202072398**	N12:468484	36,77				rDNA	
chrXII	468341	468490	149	**-1,078225171**	N12:468484	36,77				rDNA	
chrXII	468341	468513	172	**-1,302597169**	N12:468484	36,77				rDNA	
chrXII	468341	468556	215	**-1,571146876**	N12:468484	36,77				rDNA	
chrXII	468364	468513	149	**-1,360222575**	N12:468484	36,77				rDNA	
chrXII	468364	468554	190	**-1,866584018**	N12:468484	36,77				rDNA	
chrXII	468364	468556	192	**-1,727382058**	N12:468484	36,77				rDNA	
chrXII	468364	468569	205	**-1,800549754**	N12:468484	36,77				rDNA	
chrXII	468372	468532	160	**-0,869370592**	N12:468484	36,77				rDNA	
chrXII	468373	468530	157	**-1,405697754**	N12:468484	36,77				rDNA	
chrXII	468373	468554	181	**-1,198605701**	N12:468484	36,77				rDNA	
chrXII	468373	468556	183	**-1,645116746**	N12:468484	36,77				rDNA	
chrXII	468401	468490	89	**-0,320836144**	N12:468484	36,77				rDNA	
chrXII	468401	468513	112	**-0,714540302**	N12:468484	36,77				rDNA	
chrXII	468401	468532	131	**-1,073124839**	N12:468484	36,77				rDNA	
chrXII	468401	468554	153	**-1,236944389**	N12:468484	36,77				rDNA	
chrXII	468401	468556	155	**-1,282179175**	N12:468484	36,77				rDNA	
chrXII	468401	468569	168	**-1,265690437**	N12:468484	36,77				rDNA	

CHR	START	END	L(bp)	ΔLknuc	Nuc ID	Fuzziness	Gene ID	Gene body postion or intergene	Terminal	rDNA	Telomeric
chrXII	468401	468578	177	**-1,440639164**	N12:468484	36,77				rDNA	
chrXII	468402	468513	111	**-0,664532219**	N12:468484	36,77				rDNA	
chrXII	468402	468530	128	**-0,844276685**	N12:468484	36,77				rDNA	
chrXII	468402	468550	148	**-1,233234729**	N12:468484	36,77				rDNA	
chrXII	468402	468553	151	**-1,729648055**	N12:468484	36,77				rDNA	
chrXII	468402	468554	152	**-1,34783442**	N12:468484	36,77				rDNA	
chrXII	468402	468556	154	**-1,241023194**	N12:468484	36,77				rDNA	
chrXII	468402	468559	157	**-1,270244821**	N12:468484	36,77				rDNA	
chrXII	468402	468569	167	**-1,520945279**	N12:468484	36,77				rDNA	
chrXII	468402	468578	176	**-1,93501063**	N12:468484	36,77				rDNA	
chrXII	468402	468604	202	**-1,39920955**	N12:468484	36,77				rDNA	
chrXII	468402	468606	204	**-1,628984296**	N12:468484	36,77				rDNA	
chrXII	468404	468530	126	**-1,071233395**	N12:468484	36,77				rDNA	
chrXII	468404	468554	150	**-1,233858937**	N12:468484	36,77				rDNA	
chrXII	468404	468556	152	**-1,176810342**	N12:468484	36,77				rDNA	
chrXII	468404	468559	155	**-1,087112055**	N12:468484	36,77				rDNA	
chrXII	468404	468569	165	**-1,17923553**	N12:468484	36,77				rDNA	
chrXII	468404	468578	174	**-1,544182889**	N12:468484	36,77				rDNA	
chrXII	468404	468606	202	**-1,509033435**	N12:468484	36,77				rDNA	
chrXII	468405	468513	108	**-1,021063141**	N12:468484	36,77				rDNA	
chrXII	468405	468530	125	**-0,999843874**	N12:468484	36,77				rDNA	
chrXII	468405	468532	127	**-1,188020613**	N12:468484	36,77				rDNA	
chrXII	468405	468550	145	**-1,149280641**	N12:468484	36,77				rDNA	
chrXII	468405	468553	148	**-1,480884123**	N12:468484	36,77				rDNA	
chrXII	468405	468554	149	**-1,167947921**	N12:468484	36,77				rDNA	
chrXII	468405	468556	151	**-1,118964829**	N12:468484	36,77				rDNA	
chrXII	468405	468559	154	**-1,208320294**	N12:468484	36,77				rDNA	
chrXII	468405	468568	163	**-1,386682838**	N12:468484	36,77				rDNA	
chrXII	468405	468569	164	**-1,251510383**	N12:468484	36,77				rDNA	
chrXII	468405	468578	173	**-1,631934013**	N12:468484	36,77				rDNA	
chrXII	468405	468604	199	**-1,112319253**	N12:468484	36,77				rDNA	
chrXII	468405	468606	201	**-1,533035749**	N12:468484	36,77				rDNA	
chrXII	468406	468513	107	**-1,318675714**	N12:468484	36,77				rDNA	
chrXII	468406	468530	124	**-1,188335156**	N12:468484	36,77				rDNA	
chrXII	468406	468550	144	**-1,211509896**	N12:468484	36,77				rDNA	
chrXII	468406	468554	148	**-1,087112126**	N12:468484	36,77				rDNA	
chrXII	468406	468556	150	**-1,246006796**	N12:468484	36,77				rDNA	
chrXII	468406	468559	153	**-1,308781689**	N12:468484	36,77				rDNA	
chrXII	468406	468568	162	**-1,426619073**	N12:468484	36,77				rDNA	
chrXII	468406	468604	198	**-1,305863656**	N12:468484	36,77				rDNA	
chrXII	468406	468606	200	**-1,754377046**	N12:468484	36,77				rDNA	
chrXII	468445	468551	106	**-0,659464735**	N12:468484	36,77				rDNA	
chrXII	468445	468554	109	**-0,38648491**	N12:468484	36,77				rDNA	
chrXII	468445	468556	111	**-0,864271568**	N12:468484	36,77				rDNA	
chrXII	468445	468604	159	**-1,093614882**	N12:468484	36,77				rDNA	
chrXII	468445	468606	161	**-1,249276035**	N12:468484	36,77				rDNA	
chrXII	468462	468603	141	**-1,058112547**	N12:468484	36,77				rDNA	
chrXII	468463	468554	91	**-0,665772876**	N12:468484	36,77				rDNA	
chrXII	468463	468556	93	**-0,424489235**	N12:468484	36,77				rDNA	
chrXII	468463	468604	141	**-0,888867406**	N12:468484	36,77				rDNA	
chrXII	468463	468606	143	**-1,107891164**	N12:468484	36,77				rDNA	
chrXII	468545	468696	151	**-0,870498471**	N12:468638	64,023	+3*:YLR154C-H;	3	TERM	rDNA	
chrXII	468566	468665	99	**-1,340735727**	N12:468638	64,023	+3*:YLR154C-H;	3	TERM	rDNA	
chrXII	468566	468695	129	**-1,173024667**	N12:468638	64,023	+3*:YLR154C-H;	3	TERM	rDNA	
chrXII	468566	468710	144	**-1,392045818**	N12:468638	64,023	+3*:YLR154C-H;	3	TERM	rDNA	
chrXII	468566	468716	150	**-1,703042994**	N12:468638	64,023	+3*:YLR154C-H;	3	TERM	rDNA	
chrXII	468567	468695	128	**-0,913565629**	N12:468638	64,023	+3*:YLR154C-H;	3	TERM	rDNA	
chrXII	468567	468710	143	**-1,054112339**	N12:468638	64,023	+3*:YLR154C-H;	3	TERM	rDNA	
chrXII	468567	468716	149	**-1,346445114**	N12:468638	64,023	+3*:YLR154C-H;	3	TERM	rDNA	
chrXII	468656	468810	154	**-1,260621642**	N12:468638	64,023	+3*:YLR154C-H;	3	TERM	rDNA	
chrXII	468658	468810	152	**-1,482197731**	N12:468638	64,023	+3*:YLR154C-H;	3	TERM	rDNA	
chrXII	468703	468859	156	**-1,312964843**	N12:468850	34,655	+2:YLR154C-H;	2		rDNA	
chrXII	468739	468893	154	**-1,46375081**	N12:468850	34,655	+2:YLR154C-H;	2		rDNA	
chrXII	468739	468896	157	**-1,230694242**	N12:468850	34,655	+2:YLR154C-H;	2		rDNA	
chrXII	468741	468859	118	**-0,803441936**	N12:468850	34,655	+2:YLR154C-H;	2		rDNA	
chrXII	468741	468893	152	**-1,236921858**	N12:468850	34,655	+2:YLR154C-H;	2		rDNA	
chrXII	468741	468896	155	**-0,779062839**	N12:468850	34,655	+2:YLR154C-H;	2		rDNA	
chrXII	468741	468911	170	**-1,082507045**	N12:468850	34,655	+2:YLR154C-H;	2		rDNA	
chrXII	468755	468893	138	**-1,299623807**	N12:468850	34,655	+2:YLR154C-H;	2		rDNA	
chrXII	468755	468911	156	**-1,126481285**	N12:468850	34,655	+2:YLR154C-H;	2		rDNA	
chrXII	468758	468893	135	**-1,101079734**	N12:468850	34,655	+2:YLR154C-H;	2		rDNA	
chrXII	468762	468893	131	**-1,506212546**	N12:468850	34,655	+2:YLR154C-H;	2		rDNA	
chrXII	468762	468911	149	**-1,511021843**	N12:468850	34,655	+2:YLR154C-H;	2		rDNA	
chrXII	468765	468893	128	**-1,135031882**	N12:468850	34,655	+2:YLR154C-H;	2		rDNA	
chrXII	468765	468911	146	**-1,463343424**	N12:468850	34,655	+2:YLR154C-H;	2		rDNA	
chrXII	468770	468893	123	**-0,526333235**	N12:468850	34,655	+2:YLR154C-H;	2		rDNA	
chrXII	468770	468911	141	**-0,962413117**	N12:468850	34,655	+2:YLR154C-H;	2		rDNA	
chrXII	472060	472213	153	**-1,149884765**	N12:472134	7,81	+1:YLR156W;	1			
chrXII	472461	472545	84	**-0,388965263**	N12:472515	24,637	+2:YLR156C-A; +3*:YLR156W;	2			
chrXII	472461	472563	102	**-0,468873801**	N12:472515	24,637	+2:YLR156C-A; +3*:YLR156W;	2			
chrXII	475711	475864	153	**-1,31405863**	N12:475777	13,077	+1:YLR157W-D;	1			
chrXII	476909	477023	114	**-1,424773089**				Overlap <50 bp			
chrXII	477605	477755	150	**-1,531104029**	N12:477669	18,23		Intergene			
chrXII	478538	478667	129	**-1,20339556**	N12:478520	6,364		Intergene			
chrXII	479001	479158	157	**-1,465621638**	N12:479064	36,828		Intergene			
chrXII	479187	479337	150	**-1,049483026**	N12:479289	19,975		Intergene			
chrXII	479469	479630	161	**-1,566850609**	N12:479454	1,414		Intergene			
chrXII	480195	480349	154	**-1,330920335**	N12:480208	13		Intergene			
chrXII	480476	480621	145	**-1,45511423**	N12:480542	14,107		Intergene			
chrXII	480480	480631	151	**-1,223892436**	N12:480542	14,107		Intergene			
chrXII	480687	480827	140	**-0,948759724**	N12:480768	39,311		Intergene			
chrXII	480693	480827	134	**-1,08812727**	N12:480768	39,311		Intergene			
chrXII	481105	481260	155	**-1,374396656**	N12:481138	19,858		Intergene			
chrXII	481236	481344	108	**-0,818781704**				Overlap <50 bp			
chrXII	481385	481534	149	**-1,052169201**	N12:481399	46,09		Intergene			
chrXII	482041	482125	84	**-0,3979398884**	N12:482076	14,572	+2:YLR157C-C; +3:YLR157W-E;	2			

CHR	START	END	L(bp)	ΔLknuc	Nuc ID	Fuzziness	Gene ID	Gene body postion or intergene	Terminal	rDNA	Telomeric
chrXII	482041	482143	102	-0,555590478	N12:482076	14,572	+2:YLR157C-C; +3:YLR157W-E;	2			
chrXII	482885	483037	152	-1,566952739	N12:482962	13,229	+6:YLR158C;	6			
chrXII	485292	485445	153	-1,196738413	N12:485368	9,292	+1:YLR159W;	1			
chrXII	485693	485777	84	-0,452332423	N12:485732	28,827	+3*:YLR159W; +2:YLR159C-A;	3	TERM		
chrXII	488944	489097	153	-1,030790079	N12:489014	10,116	+1:YLR161W;	1			
chrXII	489345	489429	84	-0,578720288	N12:489399	19,975	+3*:YLR161W; 0:YLR162W;	3	TERM		
chrXII	489345	489447	102	-0,460759231	N12:489399	19,975	+3*:YLR161W; 0:YLR162W;	3	TERM		
chrXII	489345	489466	121	-0,925624305	N12:489399	19,975	+3*:YLR161W; 0:YLR162W;	3	TERM		
chrXII	489345	489469	124	-0,772964675	N12:489399	19,975	+3*:YLR161W; 0:YLR162W;	3	TERM		
chrXII	489345	489471	126	-1,002823668	N12:489399	19,975	+3*:YLR161W; 0:YLR162W;	3	TERM		
chrXII	489359	489469	110	-0,993882911	N12:489399	19,975	+3*:YLR161W; 0:YLR162W;	3	TERM		
chrXII	489359	489471	112	-1,177352657	N12:489399	19,975	+3*:YLR161W; 0:YLR162W;	3	TERM		
chrXII	489361	489466	105	-1,241056183	N12:489399	19,975	+3*:YLR161W; 0:YLR162W;	3	TERM		
chrXII	489556	489702	146	-1,241581141	N12:489601	15,011	+1:YLR162W;	1			
chrXII	489926	490077	151	-1,090907147	N12:489932	48,407	+3:YLR162W;	3			
chrXII	489933	490080	147	-1,352555735	N12:489932	48,407	+3:YLR162W;	3			
chrXII	490009	490164	155	-1,21027846	N12:490150	25,865	-1:YLR162W-A; +4*:YLR162W;	-1			
chrXII	490027	490183	156	-1,259777408	N12:490150	25,865	-1:YLR162W-A; +4*:YLR162W;	-1			
chrXII	490049	490195	146	-1,136889749	N12:490150	25,865	-1:YLR162W-A; +4*:YLR162W;	-1			
chrXII	490049	490215	166	-1,276140013	N12:490150	25,865	-1:YLR162W-A; +4*:YLR162W;	-1			
chrXII	490049	490219	170	-0,956575636	N12:490150	25,865	-1:YLR162W-A; +4*:YLR162W;	-1			
chrXII	490072	490215	143	-1,268629292	N12:490150	25,865	-1:YLR162W-A; +4*:YLR162W;	-1			
chrXII	490072	490219	147	-0,988491923	N12:490150	25,865	-1:YLR162W-A; +4*:YLR162W;	-1			
chrXII	490083	490239	156	-1,212383367	N12:490150	25,865	-1:YLR162W-A; +4*:YLR162W;	-1			
chrXII	490158	490312	154	-1,359112771	N12:490150	25,865	-1:YLR162W-A; +4*:YLR162W;	-1			
chrXII	490158	490312	154	-1,359112771	N12:490324	25,146	+1:YLR162W-A;	1			
chrXII	490228	490334	106	-1,44929239	N12:490324	25,146	+1:YLR162W-A;	1			
chrXII	490228	490337	109	-1,35960868	N12:490324	25,146	+1:YLR162W-A;	1			
chrXII	490228	490382	154	-1,234252158	N12:490324	25,146	+1:YLR162W-A;	1			
chrXII	490261	490368	107	-1,444551773	N12:490324	25,146	+1:YLR162W-A;	1			
chrXII	490262	490423	161	-1,356906522	N12:490324	25,146	+1:YLR162W-A;	1			
chrXII	490263	490423	160	-1,300623985	N12:490324	25,146	+1:YLR162W-A;	1			
chrXII	490267	490423	156	-1,291185827	N12:490324	25,146	+1:YLR162W-A;	1			
chrXII	490291	490423	132	-1,010558675	N12:490324	25,146	+1:YLR162W-A;	1			
chrXII	490302	490409	107	-0,946282916	N12:490324	25,146	+1:YLR162W-A;	1			
chrXII	490302	490423	121	-0,989517242	N12:490324	25,146	+1:YLR162W-A;	1			
chrXII	494931	495079	148	-1,188021641	N12:495031	11,669	+2:YLR165C;	2			
chrXII	496357	496507	150	-0,950725566				Overlap <50 bp			
chrXII	508585	508725	140	-1,159232027	N12:508668	26,883	+11:YLR176C;	11			
chrXII	514529	514679	150	-1,044012982	N12:514599	7,376	+2:YLR179C;	2			
chrXII	518369	518501	132	-1,391154497	N12:518444	10,237	+4:YLR182W;	4			
chrXII	522434	522581	147	-1,146343903	N12:522432	4,243	-1:YLR185W;	-1			
chrXII	528715	528875	160	-1,427233078	N12:528770	19,139	+4:YLR188W;	4			
chrXII	529249	529419	170	-1,662679256	N12:529380	8,185	+8:YLR188W;	8			
chrXII	537872	537976	104	-1,252899303	N12:537943	7,136	+5:YLR191W;	5			
chrXII	542120	542273	153	-1,628624905	N12:542188	12,738	+8:YLR195C; +2:CUT265;	8			
chrXII	544133	544261	128	-1,234353244	N12:544269	4,32	+3:YLR196W;	3			
chrXII	545204	545351	147	-1,161817649	N12:545241	12,755	+9:YLR196W;	9			
chrXII	546037	546189	152	-0,989916049	N12:546112	8,981	+1:YLR197W;	1			
chrXII	546038	546189	151	-1,433649953	N12:546112	8,981	+1:YLR197W;	1			
chrXII	546040	546189	149	-1,241394288	N12:546112	8,981	+1:YLR197W;	1			
chrXII	547890	548039	149	-1,181620294	N12:547946	15,655		Intergene			
chrXII	553542	553689	147	-1,107622526	N12:553631	7,642	+2:YLR205C;	2			
chrXII	555694	555847	153	-1,373030693	N12:555721	8,963	+9:YLR206W;	9			
chrXII	559139	559286	147	-1,209683481	N12:559216	3,189	+15:YLR207W; -1:YLR208W;	15			
chrXII	559606	559755	149	-0,919625008	N12:559682	6,242	+2:YLR208W;	2			
chrXII	560049	560194	145	-1,240110231	N12:560179	9,68	+5:YLR208W;	5			
chrXII	570252	570403	151	-1,195045578	N12:570424	17,024	+14:YLR214W;	14			
chrXII	573533	573669	136	-0,984279315	N12:573595	15,659	+2:YLR218C;	2			
chrXII	576803	576966	163	-1,439396479	N12:576902	26,235	+2:YLR220W;	2			
chrXII	586445	586571	126	-1,503237124	N12:586538	12,565	+2:YLR224W;	2			
chrXII	587477	587583	106	-1,378130062	N12:587489	9,381	+8*:YLR224W;	8	TERM		
chrXII	588088	588237	149	-1,200938211	N12:588110	13,435	+6:YLR225C;	6			
chrXII	593332	593484	152	-1,285444315	N12:593448	24,021		Intergene			
chrXII	593504	593653	149	-1,093835678	N12:593653	9,899		Intergene			
chrXII	593678	593801	123	-1,152554228				Overlap <50 bp			
chrXII	593778	593933	155	-1,349428874	N12:593897	31,48		Intergene			
chrXII	594211	594345	134	-1,160388708	N12:594281	32,624		Intergene			
chrXII	594211	594351	140	-1,072096703	N12:594281	32,624		Intergene			
chrXII	594407	594558	151	-1,071849284	N12:594490	27,062		Intergene			
chrXII	594417	594562	145	-1,111925036	N12:594490	27,062		Intergene			
chrXII	594435	594586	151	-1,620286771	N12:594490	27,062		Intergene			
chrXII	594689	594843	154	-1,335690177	N12:594825	8,505		Intergene			
chrXII	594723	594867	144	-1,266666298	N12:594825	8,505		Intergene			
chrXII	595254	595389	135	-1,241025624	N12:595293	18,717		Intergene			
chrXII	595436	595568	132	-1,188026385	N12:595587	3,536		Intergene			
chrXII	595525	595663	138	-1,14712218	N12:595587	3,536		Intergene			
chrXII	598015	598129	114	-1,461548698	N12:598127	33,181		Intergene			
chrXII	598489	598636	147	-1,043544883	N12:598658	29,771		Intergene			
chrXII	603237	603400	163	-1,089035045	N12:603419	20,203	:Unit459; +3*:SUT698; 0:Unit440;	0			
chrXII	607039	607198	159	-1,540087145	N12:607110	2,658	YLR233C; +1:YLR231C; -1:SUT276;	16	TERM		
chrXII	608189	608339	150	-1,970000913	N12:608276	38,05	+9:YLR233C; +6:SUT276;	9			
chrXII	610208	610357	149	-1,179280944	N12:610297	9,121	+4:YLR234W;	4			
chrXII	611103	611232	129	-0,762168679	N12:611216	20,535	+10:YLR234W;	10			
chrXII	614828	614977	149	-1,874652591	N12:614901	6,998	+2:YLR238W;	2			
chrXII	614828	614997	169	-1,6675472	N12:614901	6,998	+2:YLR238W;	2			
chrXII	614830	614977	147	-1,378254727	N12:614901	6,998	+2:YLR238W;	2			
chrXII	625233	625381	148	-1,202323984	N12:625336	34,788		Intergene			
chrXII	628912	629085	173	-1,768049895	N12:629078	27,506	+25:YLR247C;	25			
chrXII	631988	632127	139	-1,20290166	N12:632062	13,809	+9:YLR247C;	9			
chrXII	635940	636108	168	-1,543986029	N12:635980	10,498	+12:YLR248W;	12			
chrXII	640543	640689	146	-1,233114775	N12:640632	11	+3:YLR250W;	3			
chrXII	649877	650024	147	-1,570065353	N12:649854	31,564	+23:YLR256W;	23			
chrXII	649877	650024	147	-1,570065353	N12:650003	40,336	+24:YLR256W;	24			
chrXII	651183	651332	149	-1,389171765	N12:651337	43,684		Intergene			
chrXII	651357	651480	123	-1,090420043	N12:651337	43,684		Intergene			

CHR	START	END	L(bp)	ΔLknuc	Nuc ID	Fuzziness	Gene ID	Gene body postion or intergene	Terminal	rDNA	Telomeric
chrXII	651457	651612	155	**-0,978854574**	N12:651585	34,641		Intergene			
chrXII	651890	652024	134	**-1,110433573**	N12:651960	52,412		Intergene			
chrXII	651890	652030	140	**-0,882039969**	N12:651960	52,412		Intergene			
chrXII	652086	652237	151	**-1,179257004**	N12:652161	24,269		Intergene			
chrXII	652096	652241	145	**-0,985471169**	N12:652161	24,269		Intergene			
chrXII	652114	652265	151	**-1,435100285**	N12:652161	24,269		Intergene			
chrXII	652116	652265	149	**-1,327548019**	N12:652161	24,269		Intergene			
chrXII	652368	652522	154	**-1,541774625**	N12:652504	16,653		Intergene			
chrXII	652618	652786	168	**-1,306519664**	N12:652671	19,348		Intergene			
chrXII	652933	653068	135	**-1,077645629**	N12:652970	7,572		Intergene			
chrXII	653087	653248	161	**-1,369015978**	N12:653246	53,119		Intergene			
chrXII	653194	653339	145	**-1,246072008**	N12:653246	53,119		Intergene			
chrXII	653561	653718	157	**-1,37350691**	N12:653642	21,633		Intergene			
chrXII	654052	654181	129	**-1,281861347**				Overlap <50 bp			
chrXII	654964	655114	150	**-1,46602872**	N12:655066	6,506		Intergene			
chrXII	656400	656549	149	**-1,294664736**	N12:656524	53,569		Intergene			
chrXII	657729	657887	158	**-1,592834728**	N12:657828	21,638	+1:anti126;	1			
chrXII	664370	664478	108	**-1,112143121**	N12:664374	19,891	+5:YLR259C;	5			
chrXII	664370	664482	112	**-1,365450233**	N12:664374	19,891	+5:YLR259C;	5			
chrXII	665850	666010	160	**-1,433175291**	N12:665933	10,445	+2:YLR260W;	2			
chrXII	675650	675803	153	**-1,612298274**	N12:675721	5,508	R265C; +1:CUT268; +13*:YLR266C;	-1			
chrXII	678419	678585	166	**-1,339341513**	N12:678473	28,407	+3:YLR267W;	3			
chrXII	684089	684224	135	**-1,029221001**	N12:684178	29,296	+19:YLR272C;	19			
chrXII	686656	686810	154	**-1,421944125**	N12:686728	6,346	+4:YLR272C;	4			
chrXII	686971	687118	147	**-1,259381872**	N12:687063	8,704	+2:YLR272C;	2			
chrXII	691050	691199	149	**-1,543316174**	N12:691100	8,86	+2:YLR273C;	2			
chrXII	695440	695604	164	**-1,299008189**	N12:695602	37,758	+9:YLR276C;	9			
chrXII	698814	698965	151	**-1,25229095**	N12:698805	4,868	+6:YLR277C;	6			
chrXII	700732	700860	128	**-2,105816082**	N12:700798	27,391	+21:YLR278C; +7:anti128;	21			
chrXII	700732	700861	129	**-2,204084361**	N12:700798	27,391	+21:YLR278C; +7:anti128;	21			
chrXII	704633	704781	148	**-1,113566424**	N12:704801	35,242	+2:YLR281C;	2			
chrXII	704633	704785	152	**-1,220321279**	N12:704801	35,242	+2:YLR281C;	2			
chrXII	704633	704781	148	**-1,113566424**	N12:704613	0	+3:YLR281C;	3			
chrXII	704633	704785	152	**-1,220321279**	N12:704613	0	+3:YLR281C;	3			
chrXII	704637	704785	148	**-1,496555695**	N12:704801	35,242	+2:YLR281C;	2			
chrXII	704738	704878	140	**-0,93576076**	N12:704801	35,242	+2:YLR281C;	2			
chrXII	707321	707471	150	**-1,302650556**	N12:707390	3,742	+1:YLR285W; -1:YLR284C;	1			
chrXII	707855	708017	162	**-1,300801497**	N12:707899	24,295	+4:YLR285W;	4			
chrXII	710806	710956	150	**-1,125379508**	N12:710883	25,079	+8*:YLR287C;	8	TERM		
chrXII	713802	713939	137	**-0,915756343**	N12:713872	34,268	+7:YLR288C;	7			
chrXII	714204	714311	107	**-1,327109314**	N12:714214	7,083	+5:YLR288C;	5			
chrXII	714204	714312	108	**-1,266675365**	N12:714214	7,083	+5:YLR288C;	5			
chrXII	714794	714945	151	**-1,614690376**	N12:714880	17,952	-1:YLR289W; +1:YLR288C;	-1			
chrXII	721307	721447	140	**-1,179253806**	N12:721455	23,215	+1:YLR293C;	1			
chrXII	723297	723432	135	**-1,152113397**	N12:723451	34,025		Intergene			
chrXII	726306	726432	126	**-0,899340527**	N12:726432	8,548	+3:YLR299W;	3			
chrXII	727018	727148	130	**-1,792398558**	N12:727116	23	+7:YLR299W;	7			
chrXII	731448	731612	164	**-1,398383819**	N12:731633	17,01	+6*:YLR301W;	6	TERM		
chrXII	731448	731612	164	**-1,398383819**	N12:731474	4,619	+5:YLR301W;	5			
chrXII	733723	733874	151	**-1,352314842**	N12:733875	29,092	+9*:YLR303W;	9	TERM		
chrXII	733723	733874	151	**-1,352314842**	N12:733718	4,243	+8:YLR303W;	8			
chrXII	735532	735635	103	**-2,071022605**	N12:735568	9,395	+13:YLR304C;	13			
chrXII	736027	736189	162	**-1,257376238**	N12:736034	34,677	+10:YLR304C;	10			
chrXII	738785	738957	172	**-1,487648485**	N12:738797	4,792	+32:YLR305C;	32			
chrXII	740597	740703	106	**-1,558174977**	N12:740592	23,78	+21:YLR305C;	21			
chrXII	745846	745998	152	**-1,702485895**	N12:745926	16,947	+3:YLR307W;	3			
chrXII	751027	751192	165	**-1,19160281**	N12:751096	26,661	+5:YLR309C;	5			
chrXII	752922	753058	136	**-0,801637738**	N12:753013	14,67		Intergene			
chrXII	753617	753790	173	**-1,30239763**	N12:753609	33,231		Intergene			
chrXII	753617	753790	173	**-1,30239763**	N12:753760	30,729		Intergene			
chrXII	759427	759579	152	**-1,290340323**	N12:759504	11,143	+2:YLR312W-A;	2			
chrXII	761633	761798	165	**-1,673856365**				Overlap <50 bp			
chrXII	762105	762269	164	**-1,605670916**	N12:762185	8,01	+2:YLR313C;	2			
chrXII	770786	770938	152	**-1,648730318**	N12:770870	8,886	+6:YLR319C;	6			
chrXII	779526	779669	143	**-1,331404865**	N12:779529	6,025	+3:YLR324W;	3			
chrXII	779740	779903	163	**-1,645898663**	N12:779869	8,28	+5:YLR324W;	5			
chrXII	781130	781282	152	**-1,629690566**	N12:781211	16,145	+2:YLR325C;	2			
chrXII	782565	782713	148	**-1,181935995**	N12:782593	19,449	+4:YLR326W;	4			
chrXII	783914	784060	146	**-1,252694093**	N12:783921	31,634	-1:YLR327C; 0:Unit466;	-1			
chrXII	783914	784060	146	**-1,252694093**	N12:784073	31,129	+1:Unit466;	1			
chrXII	785535	785685	150	**-1,167413271**	N12:785589	36,322	+6:YLR328W;	6			
chrXII	786502	786666	164	**-1,622967527**	N12:786585	5,419	+2:YLR329W; +6:CUT746;	2			
chrXII	792530	792669	139	**-0,985672355**	N12:792622	18,052	-1:Unit447; +3*:Unit448;	-1			
chrXII	797667	797817	150	**-0,81750493**	N12:797755	9,574	+3:YLR335W;	3			
chrXII	799286	799439	153	**-1,502847957**	N12:799357	5,273	+13:YLR335W;	13			
chrXII	803374	803523	149	**-1,367805467**	N12:803394	37,532	+12:YLR337C;	12			
chrXII	804062	804197	135	**-1,272608929**	N12:804104	25,936	+7:YLR337C;	7			
chrXII	804515	804661	146	**-0,520779775**	N12:804614	5,03	+4:YLR337C;	4			
chrXII	806849	807003	154	**-1,506225373**	N12:806937	21,954	+12*:SUT707;	12	TERM		
chrXII	807887	808014	127	**-1,307191765**	N12:807864	17,291	+4:YLR341W; +7:SUT707;	4			
chrXII	807887	808014	127	**-1,307191765**	N12:808034	40,873	+5:YLR341W; +6:SUT707;	5			
chrXII	827684	827838	154	**-1,332999705**	N12:827706	9,708	+2:YLR348C;	2			
chrXII	830101	830255	154	**-1,37999663**	N12:830181	13,243	+2:YLR351C;	2			
chrXII	830655	830774	119	**-1,37050083**	N12:830722	5,845	-1:YLR352W;	-1			
chrXII	830666	830794	128	**-0,998965775**	N12:830722	5,845	-1:YLR352W;	-1			
chrXII	831008	831187	179	**-1,613248547**	N12:831140	8,841	+2:YLR352W;	2			
chrXII	831542	831700	158	**-1,285080462**	N12:831639	25,066	+5:YLR352W;	5			
chrXII	839642	839772	130	**-1,780652983**	N12:839733	26,666		Intergene			
chrXII	840366	840517	151	**-1,253677951**				Overlap <50 bp			
chrXII	841812	841967	155	**-1,547434673**	N12:841835	12,073	+4:YLR357W;	4			
chrXII	842219	842368	149	**-1,380688311**	N12:842300	15,834	+7:YLR357W;	7			
chrXII	846202	846357	155	**-1,236857121**	N12:846233	7,457	+2:YLR360W;	2			
chrXII	864284	864432	148	**-1,057629113**	N12:864280	9,912	+11:YLR371W;	11			
chrXII	868597	868734	137	**-1,279072435**	N12:868681	17,493	+17*:YLR373C;	17	TERM		
chrXII	871320	871444	124	**-1,871475206**	N12:871354	10,035	+1:YLR373C;	1			
chrXII	871320	871449	129	**-1,041860957**	N12:871354	10,035	+1:YLR373C;	1			

CHR	START	END	L(bp)	ΔLknuc	Nuc ID	Fuzziness	Gene ID	Gene body postion or intergene	Terminal	rDNA	Telomeric
chrXII	871322	871449	127	-1,227968948	N12:871354	10,035	+1:YLR373C;	1			
chrXII	874679	874837	158	-1,890568156	N12:874753	6,055	+1:YLR377C; +7:SUT281;	1			
chrXII	878567	878705	138	-0,713345953	N12:878691	35,912	+4:YLR380W;	4			
chrXII	884555	884713	158	-1,361433238	N12:884629	14,796	+2:YLR382C;	2			
chrXII	889517	889681	164	-1,661222833	N12:889511	15,355	+22:YLR384C;	22			
chrXII	893166	893327	161	-1,249270363	N12:893245	1,708	-1:YLR384C; +2:YLR385C;	-1			
chrXII	896277	896446	169	-1,433396506	N12:896387	20,801	+18*:YLR386W; +9*:YLR387C;	18	TERM		
chrXII	896668	896822	154	-1,574832143	N12:896735	18,488	+7:YLR387C;	7			
chrXII	896811	896958	147	-1,181704098	N12:896895	4,719	+6:YLR387C;	6			
chrXII	903630	903761	131	-1,078568887	N12:903719	11,432	+1:YLR390W-A; +18*:YLR392C;	1			
chrXII	903784	903945	161	-1,33171439	N12:903890	5,076	+2:YLR390W-A; +17:YLR392C;	2			
chrXII	903961	904117	156	-1,215793365	N12:904050	10,035	+3:YLR390W-A; +16:YLR392C;	3			
chrXII	905180	905335	155	-1,697798567				Overlap <50 bp			
chrXII	908649	908820	171	-1,7539698	N12:908748	20,169	+6:YLR394W; +11:YLR393W;	6			
chrXII	912499	912653	154	-1,256421667	N12:912578	15,845	-1:YLR396C; +14*:YLR397C;	-1			
chrXII	913527	913695	168	-1,589973244	N12:913636	55,94	+8:YLR397C;	8			
chrXII	922406	922556	150	-1,604816867	N12:922523	20,657	+12*:YLR401C;	12	TERM		
chrXII	922509	922654	145	-1,185258968	N12:922523	20,657	+12*:YLR401C;	12	TERM		
chrXII	924375	924524	149	-1,23336691	N12:924433	6,338	+1:YLR401C;	1			
chrXII	926149	926303	154	-1,367460252	N12:926312	33,277	+5:YLR403W;	5			
chrXII	926358	926515	157	-1,343691735	N12:926512	47,794	+6:YLR403W;	6			
chrXII	929019	929172	153	-1,378424745	N12:929097	9,538	+3:YLR404W; +4:CUT753;	3			
chrXII	931573	931698	125	-1,353750099				Overlap <50 bp			
chrXII	937960	938108	148	-1,291993363	N12:938021	7,033	+4:YLR410W;	4			
chrXII	938740	938880	140	-1,246618974	N12:938790	9,535	+8:YLR410W;	8			
chrXII	939726	939894	168	-1,420088907	N12:939769	15,955		Intergene			
chrXII	940022	940161	139	-0,922168637	N12:940088	15,176		Intergene			
chrXII	941909	942015	106	-1,224131786	N12:941949	36,042		Intergene			
chrXII	941909	942019	110	-1,395497851	N12:941949	36,042		Intergene			
chrXII	943265	943394	129	-1,428878182	N12:943276	16,263		Intergene			
chrXII	944730	944873	143	-0,885989839	N12:944794	21,656		Intergene			
chrXII	946105	946219	114	-1,386011908	N12:946210	33,292		Intergene			
chrXII	946809	946958	149	-1,063448881	N12:946920	31,507	-1:YLR411W;	-1			
chrXII	947096	947245	149	-1,661922292	N12:947106	31,07	0:YLR411W;	0			
chrXII	947588	947739	151	-1,618389137	N12:947709	32,591	+4:YLR411W;	4			
chrXII	952012	952159	147	-0,87917015	N12:952105	30,279	+7:YLR413W;	7			
chrXII	955881	956039	158	-1,204435279	N12:955940	15,369	+7:YLR417W;	7			
chrXII	956586	956713	127	-1,425935455	N12:956629	44,319	+10*:YLR418C; +12:YLR417W;	10	TERM		
chrXII	962105	962271	166	-1,321875338	N12:962110	20,942	+23:YLR419W;	23			
chrXII	962105	962271	166	-1,321875338	N12:962293	8,864	+24:YLR419W;	24			
chrXII	966042	966188	146	-1,158152817	N12:966156	18,446	+3:YLR422W;	3			
chrXII	967180	967328	148	-1,481968846	N12:967232	33,921	+10:YLR422W;	10			
chrXII	967209	967341	132	-0,887760468	N12:967232	33,921	+10:YLR422W;	10			
chrXII	974145	974297	152	-1,521288488	N12:974223	4,243	+6:YLR424W;	6			
chrXII	975018	975184	166	-1,423018085	N12:975031	34,852	+11:YLR424W;	11			
chrXII	976449	976598	149	-1,232510533	N12:976465	29,866		Intergene			
chrXII	978018	978161	143	-0,837629057	N12:978103	25,697		Intergene			
chrXII	978601	978756	155	-1,360556813	N12:978687	47,658		Intergene			
chrXII	979540	979688	148	-1,485456149				Overlap <50 bp			
chrXII	981009	981111	102	-0,465985516				Overlap <50 bp			
chrXII	981009	981139	130	-1,130117322				Overlap <50 bp			
chrXII	981073	981227	154	-1,323320528	N12:981187	32,868		Intergene			
chrXII	981080	981248	168	-1,415991227	N12:981187	32,868		Intergene			
chrXII	982426	982590	164	-1,298551797	N12:982511	15,007	-1:YLR425W;	-1			
chrXII	987388	987536	148	-1,1864529	N12:987440	6,178	+3:YLR426W;	3			
chrXII	988804	988961	157	-1,387466686	N12:988850	10,114	+4:YLR427W;	4			
chrXII	988850	988961	111	-0,895631229	N12:988850	10,114	+4:YLR427W;	4			
chrXII	988957	989104	147	-1,070461326	N12:989040	19,887	+5:YLR427W;	5			
chrXII	990093	990242	149	-1,18180111	N12:990189	11,541	+12:YLR427W;	12			
chrXII	990292	990442	150	-1,075066551	N12:990360	11,978	+13*:YLR427W;	13	TERM		
chrXII	997620	997777	157	-1,480616283	N12:997691	37,877		Intergene			
chrXII	998925	999073	148	-1,828157734	N12:999015	28,169		Intergene			
chrXII	1001567	1001716	149	-1,494810195	N12:1001641	7,731	+3:YLR431C;	3			
chrXII	1005509	1005668	159	-1,765200535	N12:1005574	10,474	+4:YLR433C;	4			
chrXII	1005994	1006142	148	-1,176876468	N12:1006065	4,761	+1:YLR433C; -1:YLR435W;	1			
chrXII	1012419	1012577	158	-1,292574676	N12:1012484	16,355	+1:YLR438W;	1			
chrXII	1012549	1012696	147	-2,60724131	N12:1012647	29,704	+2:YLR438W;	2			
chrXII	1012550	1012632	82	-1,703987772	N12:1012647	29,704	+2:YLR438W;	2			
chrXII	1012550	1012633	83	-1,555418571	N12:1012647	29,704	+2:YLR438W;	2			
chrXII	1012550	1012634	84	-1,229970034	N12:1012647	29,704	+2:YLR438W;	2			
chrXII	1012550	1012635	85	-1,050611192	N12:1012647	29,704	+2:YLR438W;	2			
chrXII	1012550	1012636	86	-0,594355953	N12:1012647	29,704	+2:YLR438W;	2			
chrXII	1012550	1012637	87	-1,816592235	N12:1012647	29,704	+2:YLR438W;	2			
chrXII	1012550	1012638	88	-1,28809307	N12:1012647	29,704	+2:YLR438W;	2			
chrXII	1012550	1012639	89	-1,249917554	N12:1012647	29,704	+2:YLR438W;	2			
chrXII	1012550	1012640	90	-1,432514376	N12:1012647	29,704	+2:YLR438W;	2			
chrXII	1012550	1012641	91	-1,39232261	N12:1012647	29,704	+2:YLR438W;	2			
chrXII	1012550	1012642	92	-1,013001793	N12:1012647	29,704	+2:YLR438W;	2			
chrXII	1012550	1012643	93	-1,268010483	N12:1012647	29,704	+2:YLR438W;	2			
chrXII	1012550	1012644	94	-1,234401513	N12:1012647	29,704	+2:YLR438W;	2			
chrXII	1012550	1012645	95	-1,559710548	N12:1012647	29,704	+2:YLR438W;	2			
chrXII	1012550	1012646	96	-1,132476066	N12:1012647	29,704	+2:YLR438W;	2			
chrXII	1012550	1012648	98	-1,340709012	N12:1012647	29,704	+2:YLR438W;	2			
chrXII	1012550	1012649	99	-1,168547102	N12:1012647	29,704	+2:YLR438W;	2			
chrXII	1012550	1012650	100	-0,978978077	N12:1012647	29,704	+2:YLR438W;	2			
chrXII	1012550	1012651	101	-0,601324136	N12:1012647	29,704	+2:YLR438W;	2			
chrXII	1012550	1012652	102	-1,732895924	N12:1012647	29,704	+2:YLR438W;	2			
chrXII	1012550	1012653	103	-1,17360965	N12:1012647	29,704	+2:YLR438W;	2			
chrXII	1012550	1012654	104	-1,154786672	N12:1012647	29,704	+2:YLR438W;	2			
chrXII	1012550	1012655	105	-0,5863446	N12:1012647	29,704	+2:YLR438W;	2			
chrXII	1012550	1012656	106	-1,63626122	N12:1012647	29,704	+2:YLR438W;	2			
chrXII	1012550	1012657	107	-1,141816806	N12:1012647	29,704	+2:YLR438W;	2			
chrXII	1012550	1012659	109	-0,985866094	N12:1012647	29,704	+2:YLR438W;	2			
chrXII	1012550	1012660	110	-0,753558564	N12:1012647	29,704	+2:YLR438W;	2			
chrXII	1012550	1012661	111	-1,144849445	N12:1012647	29,704	+2:YLR438W;	2			
chrXII	1012550	1012662	112	-0,867200904	N12:1012647	29,704	+2:YLR438W;	2			

CHR	START	END	L(bp)	ΔLknuc	Nuc ID	Fuzziness	Gene ID	Gene body postion or intergene	Terminal	rDNA	Telomeric
chrXII	1012550	1012663	113	**-1,104667559**	N12:1012647	29,704	+2:YLR438W;	2			
chrXII	1012550	1012664	114	**-1,446563429**	N12:1012647	29,704	+2:YLR438W;	2			
chrXII	1012550	1012665	115	**-1,222845338**	N12:1012647	29,704	+2:YLR438W;	2			
chrXII	1012550	1012666	116	**-1,274760103**	N12:1012647	29,704	+2:YLR438W;	2			
chrXII	1012550	1012667	117	**-0,960653632**	N12:1012647	29,704	+2:YLR438W;	2			
chrXII	1012550	1012668	118	**-1,288907071**	N12:1012647	29,704	+2:YLR438W;	2			
chrXII	1012550	1012669	119	**-0,980708146**	N12:1012647	29,704	+2:YLR438W;	2			
chrXII	1012550	1012670	120	**-1,147419989**	N12:1012647	29,704	+2:YLR438W;	2			
chrXII	1012550	1012671	121	**-1,491951922**	N12:1012647	29,704	+2:YLR438W;	2			
chrXII	1012550	1012672	122	**-1,358985062**	N12:1012647	29,704	+2:YLR438W;	2			
chrXII	1012550	1012673	123	**-1,301851291**	N12:1012647	29,704	+2:YLR438W;	2			
chrXII	1012550	1012674	124	**-2,23299362**	N12:1012647	29,704	+2:YLR438W;	2			
chrXII	1012550	1012675	125	**-1,369276971**	N12:1012647	29,704	+2:YLR438W;	2			
chrXII	1012550	1012676	126	**-1,390116407**	N12:1012647	29,704	+2:YLR438W;	2			
chrXII	1012550	1012677	127	**-1,688013805**	N12:1012647	29,704	+2:YLR438W;	2			
chrXII	1012550	1012678	128	**-1,053736296**	N12:1012647	29,704	+2:YLR438W;	2			
chrXII	1012550	1012679	129	**-0,815601844**	N12:1012647	29,704	+2:YLR438W;	2			
chrXII	1012550	1012680	130	**-1,174603453**	N12:1012647	29,704	+2:YLR438W;	2			
chrXII	1012550	1012681	131	**-1,389263522**	N12:1012647	29,704	+2:YLR438W;	2			
chrXII	1012550	1012682	132	**-1,096373597**	N12:1012647	29,704	+2:YLR438W;	2			
chrXII	1012550	1012683	133	**-1,601493863**	N12:1012647	29,704	+2:YLR438W;	2			
chrXII	1012550	1012684	134	**-1,027464857**	N12:1012647	29,704	+2:YLR438W;	2			
chrXII	1012550	1012685	135	**-1,105396275**	N12:1012647	29,704	+2:YLR438W;	2			
chrXII	1012550	1012686	136	**-1,262704946**	N12:1012647	29,704	+2:YLR438W;	2			
chrXII	1012550	1012687	137	**-1,40186849**	N12:1012647	29,704	+2:YLR438W;	2			
chrXII	1012550	1012688	138	**-1,129146826**	N12:1012647	29,704	+2:YLR438W;	2			
chrXII	1012550	1012689	139	**-1,063228137**	N12:1012647	29,704	+2:YLR438W;	2			
chrXII	1012550	1012690	140	**-1,096232623**	N12:1012647	29,704	+2:YLR438W;	2			
chrXII	1012550	1012691	141	**-1,395176989**	N12:1012647	29,704	+2:YLR438W;	2			
chrXII	1012550	1012692	142	**-1,113868199**	N12:1012647	29,704	+2:YLR438W;	2			
chrXII	1012550	1012693	143	**-1,096978822**	N12:1012647	29,704	+2:YLR438W;	2			
chrXII	1012550	1012694	144	**-1,431926774**	N12:1012647	29,704	+2:YLR438W;	2			
chrXII	1012550	1012695	145	**-1,413781992**	N12:1012647	29,704	+2:YLR438W;	2			
chrXII	1012550	1012696	146	**-1,070588043**	N12:1012647	29,704	+2:YLR438W;	2			
chrXII	1012550	1012698	148	**-1,525375093**	N12:1012647	29,704	+2:YLR438W;	2			
chrXII	1012551	1012693	142	**-1,489015829**	N12:1012647	29,704	+2:YLR438W;	2			
chrXII	1012551	1012696	145	**-1,826416557**	N12:1012647	29,704	+2:YLR438W;	2			
chrXII	1012552	1012696	144	**-1,640072941**	N12:1012647	29,704	+2:YLR438W;	2			
chrXII	1012553	1012693	140	**-0,847102545**	N12:1012647	29,704	+2:YLR438W;	2			
chrXII	1012553	1012696	143	**-1,565814544**	N12:1012647	29,704	+2:YLR438W;	2			
chrXII	1012554	1012696	142	**-0,940696314**	N12:1012647	29,704	+2:YLR438W;	2			
chrXII	1012555	1012696	141	**-1,23411604**	N12:1012647	29,704	+2:YLR438W;	2			
chrXII	1012556	1012696	140	**-1,299232068**	N12:1012647	29,704	+2:YLR438W;	2			
chrXII	1012557	1012696	139	**-1,762170494**	N12:1012647	29,704	+2:YLR438W;	2			
chrXII	1012558	1012696	138	**-1,583648443**	N12:1012647	29,704	+2:YLR438W;	2			
chrXII	1012559	1012696	137	**-0,946648921**	N12:1012647	29,704	+2:YLR438W;	2			
chrXII	1012560	1012696	136	**-1,36337568**	N12:1012647	29,704	+2:YLR438W;	2			
chrXII	1012561	1012696	135	**-1,295297759**	N12:1012647	29,704	+2:YLR438W;	2			
chrXII	1012562	1012696	134	**-1,938357556**	N12:1012647	29,704	+2:YLR438W;	2			
chrXII	1012563	1012696	133	**-1,432966431**	N12:1012647	29,704	+2:YLR438W;	2			
chrXII	1012564	1012696	132	**-2,259462671**	N12:1012647	29,704	+2:YLR438W;	2			
chrXII	1012565	1012696	131	**-1,785833694**	N12:1012647	29,704	+2:YLR438W;	2			
chrXII	1012566	1012696	130	**-1,583583607**	N12:1012647	29,704	+2:YLR438W;	2			
chrXII	1012567	1012696	129	**-2,158811696**	N12:1012647	29,704	+2:YLR438W;	2			
chrXII	1012568	1012696	128	**-1,949151486**	N12:1012647	29,704	+2:YLR438W;	2			
chrXII	1012569	1012696	127	**-2,052981344**	N12:1012647	29,704	+2:YLR438W;	2			
chrXII	1012570	1012696	126	**-1,916861355**	N12:1012647	29,704	+2:YLR438W;	2			
chrXII	1012571	1012696	125	**-2,217038942**	N12:1012647	29,704	+2:YLR438W;	2			
chrXII	1012572	1012696	124	**-1,487194401**	N12:1012647	29,704	+2:YLR438W;	2			
chrXII	1012573	1012696	123	**-1,871224647**	N12:1012647	29,704	+2:YLR438W;	2			
chrXII	1012574	1012696	122	**-1,851573068**	N12:1012647	29,704	+2:YLR438W;	2			
chrXII	1012575	1012696	121	**-0,951586593**	N12:1012647	29,704	+2:YLR438W;	2			
chrXII	1012576	1012696	120	**-2,097664905**	N12:1012647	29,704	+2:YLR438W;	2			
chrXII	1012578	1012696	118	**-2,282261357**	N12:1012647	29,704	+2:YLR438W;	2			
chrXII	1012579	1012696	117	**-1,436830513**	N12:1012647	29,704	+2:YLR438W;	2			
chrXII	1012580	1012696	116	**-1,947610656**	N12:1012647	29,704	+2:YLR438W;	2			
chrXII	1012581	1012696	115	**-1,736134278**	N12:1012647	29,704	+2:YLR438W;	2			
chrXII	1012583	1012696	113	**-0,962909448**	N12:1012647	29,704	+2:YLR438W;	2			
chrXII	1012584	1012696	112	**-2,008328572**	N12:1012647	29,704	+2:YLR438W;	2			
chrXII	1012586	1012696	110	**-0,706380039**	N12:1012647	29,704	+2:YLR438W;	2			
chrXII	1012590	1012674	84	**-0,850573336**	N12:1012647	29,704	+2:YLR438W;	2			
chrXII	1012610	1012696	86	**-1,6800582**	N12:1012647	29,704	+2:YLR438W;	2			
chrXII	1013868	1014024	156	**-1,130354816**	N12:1013902	23,483	+3:YLR438C-A;	3			
chrXII	1017667	1017816	149	**-1,478527849**	N12:1017757	36,682	+1:YLR440C;	1			
chrXII	1023621	1023767	146	**-1,197893854**	N12:1023663	7,563	+8:YLR443W;	8			
chrXII	1024132	1024289	157	**-1,522310166**	N12:1024222	10,035	+1:YLR445W;	1			
chrXII	1031294	1031445	151	**-1,315034471**	N12:1031333	26,332	+4:YLR449W;	4			
chrXII	1032438	1032586	148	**-1,41857051**	N12:1032481	9,508	+1:YLR450W;	1			
chrXII	1050400	1050528	128	**-1,402878192**	N12:1050503	12,161		Intergene			
chrXII	1050732	1050875	143	**-1,634285902**	N12:1050868	32,723		Intergene			
chrXII	1059679	1059820	141	**-1,397733471**	N12:1059812	9,854	+8*:YLR460C;	8	TERM		
chrXII	1065029	1065154	125	**-0,350752953**	N12:1065098	32,501		Intergene			TEL RIGHT
chrXII	1065029	1065156	127	**-0,303005082**	N12:1065098	32,501		Intergene			TEL RIGHT
chrXII	1066703	1066854	151	**-1,393222444**				Overlap <50 bp			TEL RIGHT
chrXII	1067646	1067808	162	**-1,186246987**	N12:1067769	9,539	+5:YLR466W;	5			TEL RIGHT
chrXII	1067747	1067902	155	**-1,852097459**	N12:1067769	9,539	+5:YLR466W;	5			TEL RIGHT
chrXII	1067881	1068018	137	**-1,092789151**	N12:1067950	18,583	+6:YLR466W;	6			TEL RIGHT
chrXII	1068176	1068319	143	**-1,302361921**	N12:1068230	14,503	+8:YLR466W;	8			TEL RIGHT
chrXII	1068224	1068349	125	**-1,021672271**	N12:1068230	14,503	+8:YLR466W;	8			TEL RIGHT
chrXII	1068449	1068601	152	**-1,213184443**				Overlap <50 bp			TEL RIGHT
chrXII	1068522	1068666	144	**-1,231008052**				Overlap <50 bp			TEL RIGHT
chrXII	1068619	1068754	135	**-1,049580135**	N12:1068716	23,302	+11:YLR466W;	11			TEL RIGHT
chrXII	1068619	1068806	187	**-1,083121784**	N12:1068716	23,302	+11:YLR466W;	11			TEL RIGHT
chrXII	1068662	1068754	92	**-0,366049365**	N12:1068716	23,302	+11:YLR466W;	11			TEL RIGHT
chrXII	1068662	1068806	144	**-0,948731851**	N12:1068716	23,302	+11:YLR466W;	11			TEL RIGHT
chrXII	1069001	1069153	152	**-1,351882452**	N12:1069071	40,377	+13:YLR466W;	13			TEL RIGHT

CHR	START	END	L(bp)	ΔLknuc	Nuc ID	Fuzziness	Gene ID	Gene body postion or intergene	Terminal	rDNA	Telomeric
chrXII	1069302	1069432	130	**-1,117771804**	N12:1069283	42,438	+14:YLR466W;	14			TEL RIGHT
chrXII	1069440	1069601	161	**-1,173760425**				Overlap <50 bp			TEL RIGHT
chrXII	1070837	1070988	151	**-1,274029915**	N12:1070915	64,003	+23:YLR466W;	23			TEL RIGHT
chrXII	1070837	1071008	171	**-1,278700138**	N12:1070915	64,003	+23:YLR466W;	23			TEL RIGHT
chrXII	1072226	1072381	155	**-1,508760617**	N12:1072335	30,271	0:YLR467W;	0			TEL RIGHT
chrXII	1073391	1073538	147	**-1,161982674**				Overlap <50 bp			TEL RIGHT
chrXII	1073547	1073677	130	**-1,049429275**	N12:1073613	19,63	+8:YLR467W;	8			TEL RIGHT
chrXII	1073928	1074064	136	**-1,300139293**	N12:1073964	0	+10:YLR467W;	10			TEL RIGHT
chrXII	1074309	1074471	162	**-1,216368113**	N12:1074439	10,97	+13:YLR467W;	13			TEL RIGHT
chrXII	1074410	1074565	155	**-1,929018038**	N12:1074439	10,97	+13:YLR467W;	13			TEL RIGHT
chrXII	1074544	1074681	137	**-1,099427163**	N12:1074612	9,713	+14:YLR467W;	14			TEL RIGHT
chrXII	1074831	1074982	151	**-1,520081729**	N12:1074890	4,359	+16:YLR467W;	16			TEL RIGHT
chrXII	1074839	1074982	143	**-1,387980516**	N12:1074890	4,359	+16:YLR467W;	16			TEL RIGHT
chrXII	1074887	1075012	125	**-0,951224242**	N12:1074890	4,359	+16:YLR467W;	16			TEL RIGHT
chrXII	1075112	1075264	152	**-1,299676744**				Overlap <50 bp			TEL RIGHT
chrXII	1075185	1075329	144	**-1,393480009**				Overlap <50 bp			TEL RIGHT
chrXII	1075282	1075417	135	**-0,986164431**	N12:1075369	30,199	+19:YLR467W;	19			TEL RIGHT
chrXII	1075282	1075469	187	**-1,088191836**	N12:1075369	30,199	+19:YLR467W;	19			TEL RIGHT
chrXII	1075325	1075417	92	**-0,325215863**	N12:1075369	30,199	+19:YLR467W;	19			TEL RIGHT
chrXII	1075325	1075469	144	**-1,028038225**	N12:1075369	30,199	+19:YLR467W;	19			TEL RIGHT
chrXII	1075664	1075816	152	**-1,181249815**	N12:1075734	7,778	+21:YLR467W;	21			TEL RIGHT
chrXII	1075965	1076095	130	**-1,086125662**	N12:1076061	36,638	+23:YLR467W;	23			TEL RIGHT
chrXII	1076103	1076264	161	**-1,289945398**				Overlap <50 bp			TEL RIGHT
chrXII	1077160	1077312	152	**-1,399093427**	N12:1077229	52,624	+30:YLR467W;	30			TEL RIGHT
chrXII	1077500	1077651	151	**-1,265868927**	N12:1077576	34,962	+32:YLR467W;	32			TEL RIGHT
chrXIII	687	858	171	**-1,183022396**	N13:774	35,791	+25:YML133C;	25			TEL LEFT
chrXIII	707	858	151	**-1,165709288**	N13:774	35,791	+25:YML133C;	25			TEL LEFT
chrXIII	1046	1198	152	**-1,480095132**	N13:1107	43,92	+23:YML133C;	23			TEL LEFT
chrXIII	1151	1299	148	**-1,439779997**				Overlap <50 bp			TEL LEFT
chrXIII	1836	2023	187	**-0,910571306**	N13:1892	9,452	+18:YML133C;	18			TEL LEFT
chrXIII	1872	2023	151	**-1,18877236**	N13:1892	9,452	+18:YML133C;	18			TEL LEFT
chrXIII	1884	2018	134	**-1,03886823**	N13:1892	9,452	+18:YML133C;	18			TEL LEFT
chrXIII	1909	2005	96	**-0,957176331**	N13:1892	9,452	+18:YML133C;	18			TEL LEFT
chrXIII	2542	2694	152	**-1,176021394**	N13:2630	14,978	+14:YML133C;	14			TEL LEFT
chrXIII	2889	3033	144	**-1,003528128**	N13:2945	16,462	+12:YML133C;	12			TEL LEFT
chrXIII	2889	3076	187	**-1,003940139**	N13:2945	16,462	+12:YML133C;	12			TEL LEFT
chrXIII	2941	3033	92	**-0,271296725**	N13:2945	16,462	+12:YML133C;	12			TEL LEFT
chrXIII	2941	3076	135	**-0,859328525**	N13:2945	16,462	+12:YML133C;	12			TEL LEFT
chrXIII	3029	3173	144	**-1,294580427**	N13:3125	4,509	+11:YML133C;	11			TEL LEFT
chrXIII	3094	3246	152	**-1,19440057**	N13:3125	4,509	+11:YML133C;	11			TEL LEFT
chrXIII	3346	3471	125	**-0,827654523**	N13:3450	12,124	+9:YML133C;	9			TEL LEFT
chrXIII	3376	3519	143	**-1,449081815**	N13:3450	12,124	+9:YML133C;	9			TEL LEFT
chrXIII	3376	3527	151	**-1,556775214**	N13:3450	12,124	+9:YML133C;	9			TEL LEFT
chrXIII	5624	5767	143	**-1,19399343**	N13:5763	5,686		Intergene			TEL LEFT
chrXIII	9070	9207	137	**-1,136301165**	N13:9142	17,711	0:Unit471;	0			
chrXIII	13123	13267	144	**-1,258306945**	N13:13226	17,268	-1:SUT285; +1:YML130C;	-1			
chrXIII	14333	14505	172	**-1,644543528**	N13:14398	2,881	+3*:YML129C;	3	TERM		
chrXIII	16025	16179	154	**-1,283756977**	N13:16099	9,452	+5:YML128C;	5			
chrXIII	18573	18744	171	**-1,484644976**	N13:18675	25,185	+11:YML127W;	11			
chrXIII	19858	19999	141	**-1,117666276**	N13:19950	6,768	+4:YML126C;	4			
chrXIII	20186	20333	147	**-1,320687818**	N13:20274	13,808	+2:YML126C;	2			
chrXIII	21593	21740	147	**-1,238327004**	N13:21676	8,718	+1:YML125C; -1:anti-YML124C;	1			
chrXIII	23095	23242	147	**-1,221766854**	N13:23173	7,662	+4:YML124C;	4			
chrXIII	32413	32558	145	**-1,275997143**	N13:32440	18,802	+2:YML118W;	2			
chrXIII	33232	33384	152	**-1,243536568**	N13:33387	20,253	+8:YML118W;	8			
chrXIII	38992	39143	151	**-1,328742332**	N13:39050	7,071	+7:YML116W;	7			
chrXIII	40472	40624	152	**-1,343492743**	N13:40521	24,185	+9:YML115C;	9			
chrXIII	42972	43133	161	**-1,309797338**	N13:43054	4,848	+4:YML114C;	4			
chrXIII	47095	47254	159	**-1,127314419**	N13:47157	13,074	+3:YML111W;	3			
chrXIII	54675	54757	82	**-0,753764522**				Overlap <50 bp			
chrXIII	54913	55020	107	**-1,173871325**	N13:54961	12,817	+2:YML108W; +2:CUT759;	2			
chrXIII	54913	55032	119	**-1,324745926**	N13:54961	12,817	+2:YML108W; +2:CUT759;	2			
chrXIII	59231	59396	165	**-1,143192055**	N13:59272	19,191	+20:YML104C; +3*:CUT283;	20			
chrXIII	63883	64021	138	**-1,059913134**	N13:63895	39,306		Intergene			
chrXIII	64235	64393	158	**-1,705670368**	N13:64363	38,475		Intergene			
chrXIII	64871	65018	147	**-1,308217953**	N13:64980	24,9		Intergene			
chrXIII	66681	66829	148	**-1,217484632**	N13:66758	26,701	+6:YML103C;	6			
chrXIII	75788	75939	151	**-1,317183113**	N13:75859	21,265	+7:YML099C;	7			
chrXIII	76732	76885	153	**-1,391942299**	N13:76800	13,794	+2:YML099C;	2			
chrXIII	77591	77728	137	**-1,20762293**	N13:77626	5,568	+3:YML098W;	3			
chrXIII	83978	84137	159	**-1,215911279**				Overlap <50 bp			
chrXIII	93745	93871	126	**-1,282405246**	N13:93877	21,352	+11:YML088W;	11			
chrXIII	98827	98976	149	**-0,863674115**	N13:98894	8,385		Intergene			
chrXIII	102493	102629	136	**-1,00378864**	N13:102572	20,94	+5:YML082W;	5			
chrXIII	104088	104231	143	**-1,149830956**	N13:104162	13,029	+1:YML081C-A;	1			
chrXIII	104601	104708	107	**-1,191806582**	N13:104679	5,007	+1:YML081W;	1			
chrXIII	104601	104720	119	**-1,262640637**	N13:104679	5,007	+1:YML081W;	1			
chrXIII	107860	108019	159	**-1,171594288**	N13:107958	44,191	+22:YML081W;	22			
chrXIII	109020	109176	156	**-1,253875206**	N13:109082	7,007	+3:YML080W;	3			
chrXIII	112712	112852	140	**-1,244036558**	N13:112775	5,167	+17:YML076C;	17			
chrXIII	113210	113365	155	**-1,172115839**	N13:113351	22,543	+13:YML076C;	13			
chrXIII	113210	113365	155	**-1,172115839**	N13:113196	37,179	+14:YML076C;	14			
chrXIII	116680	116830	150	**-1,676344701**	N13:116697	12,723	+16:YML075C;	16			
chrXIII	116680	116833	153	**-1,29154697**	N13:116697	12,723	+16:YML075C;	16			
chrXIII	116680	116835	155	**-1,141570437**	N13:116697	12,723	+16:YML075C;	16			
chrXIII	116684	116835	151	**-1,394129796**	N13:116697	12,723	+16:YML075C;	16			
chrXIII	116783	116928	145	**-1,094623581**				Overlap <50 bp			
chrXIII	117349	117491	142	**-1,172366766**	N13:117410	14,328	+11:YML075C;	11			
chrXIII	117514	117618	104	**-1,532456473**	N13:117572	7,594	+10:YML075C;	10			
chrXIII	119216	119393	177	**-1,45800678**	N13:119273	28,583	-1:SUT288; -1:YML075C;	-1			
chrXIII	120074	120243	169	**-1,553893484**	N13:120063	19,092	+4*:SUT288;	4	TERM		
chrXIII	120074	120243	169	**-1,553893484**	N13:120213	12,925		Intergene			
chrXIII	123583	123746	163	**-1,407894304**	N13:123684	9,065		Intergene			
chrXIII	124934	125083	149	**-1,264136325**	N13:125011	35,084	+29:YML072C;	29			
chrXIII	125456	125612	156	**-1,201897911**	N13:125539	48,813	+26:YML072C;	26			
chrXIII	126339	126468	129	**-0,702747337**				Overlap <50 bp			

CHR	START	END	L(bp)	ΔLknuc	Nuc ID	Fuzziness	Gene ID	Gene body postion or intergene	Terminal	rDNA	Telomeric
chrXIII	129825	129930	105	-1,476714441	N13:129823	11,396	-1:YML072C; +1:CUT285;	-1			
chrXIII	129825	129932	107	-1,28624102	N13:129823	11,396	-1:YML072C; +1:CUT285;	-1			
chrXIII	129843	130004	161	-1,162787455	N13:129987	10,863	+2*:CUT285;	2	TERM		
chrXIII	129843	129930	87	-1,166659195	N13:129823	11,396	-1:YML072C; +1:CUT285;	-1			
chrXIII	129843	130004	161	-1,162787455	N13:129823	11,396	-1:YML072C; +1:CUT285;	-1			
chrXIII	130886	131027	141	-1,035647797	N13:130956	24,214	+5:YML071C;	5			
chrXIII	131028	131190	162	-1,052845016	N13:131120	9,973	+4:YML071C;	4			
chrXIII	131602	131755	153	-1,370976245	N13:131595	39,185	+1:YML071C;	1			
chrXIII	133367	133536	169	-1,418589705	N13:133467	19,94	+1:YML070W;	1			
chrXIII	134348	134447	99	-1,429663408	N13:134415	29,538	+7:YML070W;	7			
chrXIII	135575	135725	150	-1,381659658	N13:135649	9,158	+2:YML069W;	2			
chrXIII	139632	139738	106	-1,444777998	N13:139678	7,439		Intergene			
chrXIII	139632	139741	109	-1,648734967	N13:139678	7,439		Intergene			
chrXIII	142450	142583	133	-2,215482142	N13:142508	7,43	+3:YML065W;	3			
chrXIII	147977	148129	152	-1,254179292				Overlap <50 bp			
chrXIII	148322	148434	112	-1,381900204				Overlap <50 bp			
chrXIII	154231	154388	157	-1,4887113				Overlap <50 bp			
chrXIII	155621	155784	163	-1,235086737				Overlap <50 bp			
chrXIII	163164	163259	95	-1,197794011				Overlap <50 bp			
chrXIII	169237	169345	108	-1,466604463	N13:169314	1,5	+4:YML053C;	4			
chrXIII	170327	170483	156	-1,469530991	N13:170398	9,6	+1:YML052W;	1			
chrXIII	170574	170680	106	-0,479397498	N13:170557	5,167	+2:YML052W;	2			
chrXIII	170574	170696	122	-0,638800202	N13:170557	5,167	+2:YML052W;	2			
chrXIII	170574	170696	122	-0,638800202	N13:170707	9,192	+3:YML052W;	3			
chrXIII	171072	171220	148	-1,347544467	N13:171141	18,841	+6:YML052W;	6			
chrXIII	175762	175915	153	-1,289261468	N13:175792	10,04		Intergene			
chrXIII	179143	179321	178	-1,485660979				Overlap <50 bp			
chrXIII	180747	180905	158	-1,015989932	N13:180820	9,048	+3:YML047C;	3			
chrXIII	181711	181851	140	-1,07003881	N13:181781	7,789	+3:YML046W;	3			
chrXIII	181848	181977	129	-1,124786751				Overlap <50 bp			
chrXIII	184182	184330	148	-1,340931298	N13:184276	24,173		Intergene			
chrXIII	184701	184824	123	-0,987566196				Overlap <50 bp			
chrXIII	184801	184956	155	-1,538963216	N13:184876	30,238		Intergene			
chrXIII	185234	185368	134	-0,785998027	N13:185305	24,007		Intergene			
chrXIII	185234	185374	140	-1,084495687	N13:185305	24,007		Intergene			
chrXIII	186277	186412	135	-0,91523725	N13:186311	22,942		Intergene			
chrXIII	186724	186874	150	-1,061757367	N13:186799	16,643		Intergene			
chrXIII	186903	187060	157	-1,527474579	N13:186999	35,119		Intergene			
chrXIII	187135	187284	149	-1,237363695	N13:187179	14,849		Intergene			
chrXIII	187394	187523	129	-1,146332821				Overlap <50 bp			
chrXIII	188306	188456	150	-1,373263247	N13:188399	8,185		Intergene			
chrXIII	195171	195314	143	-1,206340075	N13:195288	11,554		Intergene			
chrXIII	196534	196661	127	-1,349408121	N13:196631	7,767		Intergene			
chrXIII	196694	196843	149	-1,436177973	N13:196842	59,152		Intergene			
chrXIII	196868	196991	123	-1,167746496				Overlap <50 bp			
chrXIII	196968	197123	155	-1,608269175	N13:197081	27,221		Intergene			
chrXIII	197401	197535	134	-0,866397868	N13:197469	22,03		Intergene			
chrXIII	197401	197541	140	-0,829586298	N13:197469	22,03		Intergene			
chrXIII	197597	197748	151	-1,16191336	N13:197630	25,146		Intergene			
chrXIII	197607	197752	145	-1,250707401	N13:197630	25,146		Intergene			
chrXIII	197625	197776	151	-1,447382794	N13:197630	25,146		Intergene			
chrXIII	197879	198033	154	-1,234426289	N13:198018	3,606		Intergene			
chrXIII	197913	198057	144	-1,273608373	N13:198018	3,606		Intergene			
chrXIII	198891	199037	146	-1,065200705	N13:198960	14,048		Intergene			
chrXIII	198891	199041	150	-1,068014789	N13:198960	14,048		Intergene			
chrXIII	199070	199227	157	-1,155116532	N13:199146	32,005		Intergene			
chrXIII	199302	199451	149	-1,093849413	N13:199440	39,038		Intergene			
chrXIII	199561	199690	129	-1,116261561	N13:199711	11,93		Intergene			
chrXIII	200473	200623	150	-1,537549481	N13:200564	22,605		Intergene			
chrXIII	203778	203929	151	-1,786825043	N13:203861	11,197	+3:YML038C;	3			
chrXIII	204102	204256	154	-1,267077789	N13:204195	15,664	IL038C; +9*:YML037C; -1:CUT288;	1			
chrXIII	208554	208676	122	-0,385407748	N13:208664	11,295	+3:YML035C;	3			
chrXIII	211138	211313	175	-1,063784224	N13:211187	46,082	+11:YML034W;	11			
chrXIII	218290	218401	111	-1,347884584	N13:218320	22,821	+7:YML029W;	7			
chrXIII	220837	220983	146	-1,036238715	N13:220846	28,247	+5*:YML028W;	5	TERM		
chrXIII	228866	229000	134	-1,100913902	N13:228934	16,112	+1:YML022W; -1:YML023C;	1			
chrXIII	230938	231091	153	-1,401564521	N13:231007	13,186	+1:YML020W; -1:YML021C;	1			
chrXIII	231257	231408	151	-1,406040535	N13:231330	5,909	+3:YML020W;	3			
chrXIII	233569	233719	150	-1,224469638	N13:233649	10,354	+2:YML019W;	2			
chrXIII	240347	240506	159	-1,775706464	N13:240446	17,877		Intergene			
chrXIII	242945	243091	146	-1,223632645	N13:243011	11,771	+1:YML015C; -1:YML014W;	1			
chrXIII	243502	243662	160	-1,511479903	N13:243575	1,924	+3:YML014W;	3			
chrXIII	243825	243980	155	-1,272443629	N13:243892	4,856	0:YML013W; +5*:YML014W;	0			
chrXIII	244965	245130	165	-1,419863435	N13:244990	8,066	+7:YML013W;	7			
chrXIII	244965	245130	165	-1,419863435	N13:245141	2,608	+8:YML013W;	8			
chrXIII	245559	245710	151	-1,060040689	N13:245618	9,543	+11:YML013W;	11			
chrXIII	246032	246179	147	-1,581607109	N13:246115	24,238	+14*:YML013W; +1:YML012W;	14	TERM		
chrXIII	246042	246200	158	-1,263232657	N13:246115	24,238	+14*:YML013W; +1:YML012W;	14	TERM		
chrXIII	246354	246509	155	-0,81597938	N13:246437	11,356	+3:YML012W;	3			
chrXIII	246617	246737	120	-2,014758173	N13:246744	9,745	+5:YML012W;	5			
chrXIII	250435	250548	113	-0,414256154	N13:250485	35,796	+19:YML010W;	19			
chrXIII	250507	250631	124	-2,184232096	N13:250485	35,796	+19:YML010W;	19			
chrXIII	250507	250662	155	-1,922632252	N13:250485	35,796	+19:YML010W;	19			
chrXIII	250507	250664	157	-2,378686658	N13:250485	35,796	+19:YML010W;	19			
chrXIII	250507	250665	158	-2,029478431	N13:250485	35,796	+19:YML010W;	19			
chrXIII	250507	250666	159	-2,20065512	N13:250485	35,796	+19:YML010W;	19			
chrXIII	250507	250667	160	-1,652154651	N13:250485	35,796	+19:YML010W;	19			
chrXIII	250507	250668	161	-1,946263119	N13:250485	35,796	+19:YML010W;	19			
chrXIII	250507	250669	162	-1,739441696	N13:250485	35,796	+19:YML010W;	19			
chrXIII	250507	250670	163	-2,208768783	N13:250485	35,796	+19:YML010W;	19			
chrXIII	250507	250671	164	-1,972065587	N13:250485	35,796	+19:YML010W;	19			
chrXIII	250507	250673	166	-1,910430155	N13:250485	35,796	+19:YML010W;	19			
chrXIII	250507	250674	167	-1,482753129	N13:250485	35,796	+19:YML010W;	19			
chrXIII	250507	250675	168	-1,774771304	N13:250485	35,796	+19:YML010W;	19			
chrXIII	250507	250676	169	-1,932912245	N13:250485	35,796	+19:YML010W;	19			
chrXIII	250507	250677	170	-2,148264149	N13:250485	35,796	+19:YML010W;	19			

CHR	START	END	L(bp)	ΔLknuc	Nuc ID	Fuzziness	Gene ID	Gene body postion or intergene	Terminal	rDNA	Telomeric
chrXIII	250507	250678	171	**-1,335858588**	N13:250485	35,796	+19:YML010W;	19			
chrXIII	250507	250718	211	**-2,721214476**	N13:250485	35,796	+19:YML010W;	19			
chrXIII	250507	250718	211	**-2,721214476**	N13:250740	40,82	+21:YML010W;	21			
chrXIII	250508	250678	170	**-1,036986671**	N13:250485	35,796	+19:YML010W;	19			
chrXIII	250509	250678	169	**-1,76837862**				Overlap <50 bp			
chrXIII	250510	250678	168	**-1,547810362**				Overlap <50 bp			
chrXIII	250511	250678	167	**-1,737106909**				Overlap <50 bp			
chrXIII	250514	250678	164	**-1,583573622**				Overlap <50 bp			
chrXIII	250515	250678	163	**-1,62230085**				Overlap <50 bp			
chrXIII	250566	250718	152	**-1,148424337**	N13:250740	40,82	+21:YML010W;	21			
chrXIII	253372	253532	160	**-0,895652325**	N13:253539	55,194	-1:YML007C-A; -1:YML007W;	-1			
chrXIII	256009	256161	152	**-1,459542201**	N13:256158	23,422	+15:YML006C;	15			
chrXIII	260961	261102	141	**-1,071497549**	N13:261093	16,056	+6*:YML005W;	6	TERM		
chrXIII	261845	261977	132	**-1,466420039**	N13:261963	32,192	+5:YML004C;	5			
chrXIII	262094	262251	157	**-1,153168844**	N13:262151	9,888	+4:YML004C;	4			
chrXIII	266226	266377	151	**-1,429313389**	N13:266295	10,932	003W; +12:YML002W; +8:SUT724;	18			
chrXIII	271419	271566	147	**-1,108145805**	N13:271513	12,629	C-A; +1:YMR001C; +2:YMR001C-A;	1			
chrXIII	273047	273197	150	**-1,428081353**	N13:273120	6,285	+1:YMR003W;	1			
chrXIII	276628	276785	157	**-1,451674262**	N13:276642	8,155	+5:YMR005W;	5			
chrXIII	276628	276785	157	**-1,451674262**	N13:276801	18,019	+6:YMR005W;	6			
chrXIII	277879	278014	135	**-0,94715038**	N13:277912	47,171	+11:YMR006C;	11			
chrXIII	279195	279369	174	**-1,223115394**	N13:279270	29,816	+3:YMR006C;	3			
chrXIII	283987	284078	91	**-1,015905132**	N13:284101	37,356	+1:YMR009W;	1			
chrXIII	283987	284079	92	**-1,028006025**	N13:284101	37,356	+1:YMR009W;	1			
chrXIII	289854	289961	107	**-0,895972097**	N13:289883	27,96	-1:SUT727; 0:SUT297;	-1			
chrXIII	289854	289977	123	**-1,291630713**	N13:289883	27,96	-1:SUT727; 0:SUT297;	-1			
chrXIII	303791	303918	127	**-1,156799892**	N13:303847	4,231	+13:YMR016C;	13			
chrXIII	304476	304622	146	**-1,341575043**	N13:304602	8,385	+8:YMR016C;	8			
chrXIII	310201	310351	150	**-1,388518271**	N13:310222	13,471	+1:YMR018W;	1			
chrXIII	313026	313180	154	**-1,237752648**				Overlap <50 bp			
chrXIII	324504	324669	165	**-1,456600636**	N13:324559	20,268	+7:YMR026C;	7			
chrXIII	325807	325958	151	**-1,253213543**	N13:325840	12,145	-1:YMR026C; +1:YMR027W;	-1			
chrXIII	333017	333169	152	**-1,069852276**	N13:333098	27,704	+12:YMR031C;	12			
chrXIII	335127	335272	145	**-1,282986666**	N13:335202	14,516	+1:YMR032W;	1			
chrXIII	335472	335613	141	**-1,346087722**	N13:335545	8,287	+3:YMR032W;	3			
chrXIII	336952	337113	161	**-1,190040157**	N13:337009	16,583	-1:YMR030W-A; +12:YMR032W;	-1			
chrXIII	340438	340585	147	**-1,510910328**	N13:340517	13,823	+3:YMR034C;	3			
chrXIII	342150	342302	152	**-1,188058539**	N13:342208	18,435	+9:YMR036C;	9			
chrXIII	348582	348731	149	**-1,277457065**	N13:348736	26,216	+6*:YMR039C; +1*:CUT294;	6	TERM		
chrXIII	351853	352022	169	**-1,18721648**	N13:351943	10,685	+1:YMR041C;	1			
chrXIII	351863	352007	144	**-1,423567584**	N13:351943	10,685	+1:YMR041C;	1			
chrXIII	357010	357124	114	**-1,438113404**				Overlap <50 bp			
chrXIII	357475	357580	105	**-1,637157197**	N13:357470	40,501		Intergene			
chrXIII	358631	358781	150	**-1,302298543**	N13:358713	20,232		Intergene			
chrXIII	358907	359055	148	**-1,346182852**	N13:358983	27,635		Intergene			
chrXIII	360027	360184	157	**-1,535050948**	N13:360111	27,622		Intergene			
chrXIII	360404	360549	145	**-1,064731222**	N13:360557	0		Intergene			
chrXIII	360675	360810	135	**-0,848823071**	N13:360775	51,856		Intergene			
chrXIII	362078	362238	160	**-1,269737615**	N13:362147	10,714		Intergene			
chrXIII	362592	362706	114	**-1,416220804**	N13:362628	0,707		Intergene			
chrXIII	374780	374911	131	**-1,121933137**	N13:374845	40		Intergene			
chrXIII	375262	375391	129	**-1,31965933**				Overlap <50 bp			
chrXIII	375501	375650	149	**-1,076227568**	N13:375616	40,129		Intergene			
chrXIII	375725	375882	157	**-1,63927456**	N13:375785	37,749		Intergene			
chrXIII	375911	376061	150	**-0,952371414**	N13:376003	16,643		Intergene			
chrXIII	376373	376508	135	**-0,951968185**	N13:376424	32,532		Intergene			
chrXIII	376763	376917	154	**-1,51158548**	N13:376925	25,534		Intergene			
chrXIII	377411	377551	140	**-1,270495708**	N13:377486	52,468		Intergene			
chrXIII	377417	377551	134	**-0,981920607**	N13:377486	52,468		Intergene			
chrXIII	377829	377984	155	**-1,194261494**	N13:377857	20,232		Intergene			
chrXIII	377960	378068	108	**-0,70606957**				Overlap <50 bp			
chrXIII	379716	379822	106	**-0,864531541**	N13:379764	20,096	+2:YMR052W;	2			
chrXIII	379716	379838	122	**-1,356189495**	N13:379764	20,096	+2:YMR052W;	2			
chrXIII	381135	381283	148	**-1,124631794**	N13:381244	27,959	+11:YMR053C;	11			
chrXIII	383859	384012	153	**-1,363344256**	N13:383963	25,28	+6:YMR054W;	6			
chrXIII	384143	384283	140	**-1,256686057**	N13:384129	22,14	+7:YMR054W;	7			
chrXIII	384143	384283	140	**-1,256686057**	N13:384300	9,933	+8:YMR054W;	8			
chrXIII	384959	385107	148	**-1,236476284**				Overlap <50 bp			
chrXIII	386345	386477	132	**-0,965623439**	N13:386478	11,76	+4:YMR055C;	4			
chrXIII	386345	386477	132	**-0,965623439**	N13:386323	7,937	+5:YMR055C;	5			
chrXIII	387854	388024	170	**-1,313352513**	N13:387911	15,308	+2:YMR056C;	2			
chrXIII	395268	395412	144	**-1,281700963**	N13:395361	16,979	+7:YMR062C;	7			
chrXIII	400158	400292	134	**-1,632416398**	N13:400231	13,293	+3:YMR065W;	3			
chrXIII	402458	402607	149	**-1,1222067**	N13:402522	14,17	+7:YMR066W;	7			
chrXIII	406280	406447	167	**-1,368368444**	N13:406339	11,709	+1:YMR068W;	1			
chrXIII	408429	408536	107	**-0,04025224**	N13:408500	7,071	+6:YMR069W;	6			
chrXIII	411105	411252	147	**-1,066499899**	N13:411102	3,061	+2:YMR071C;	2			
chrXIII	412475	412581	106	**-1,357510065**	N13:412535	12,153	+5:YMR073C;	5			
chrXIII	412475	412589	114	**-1,634297997**	N13:412535	12,153	+5:YMR073C;	5			
chrXIII	413837	414003	166	**-1,032786013**	N13:413996	41,884	+1:YMR075W;	1			
chrXIII	422824	422985	161	**-1,145543381**	N13:422941	8,764	+12:YMR078C;	12			
chrXIII	431294	431455	161	**-1,39738954**	N13:431429	30,875	-1:YMR081C;	-1			
chrXIII	431358	431515	157	**-1,607674928**	N13:431429	30,875	-1:YMR081C;	-1			
chrXIII	435010	435159	149	**-1,112774013**	N13:435081	17,972	+3:YMR083W;	3			
chrXIII	436980	437142	162	**-1,161931876**	N13:437057	3,5	+4:YMR084W;	4			
chrXIII	439283	439457	174	**-1,728658448**	N13:439388	11,149	+2:YMR086W;	2			
chrXIII	443760	443869	109	**-0,94193934**	N13:443884	7,024	+9:YMR088C;	9			
chrXIII	445137	445296	159	**-1,638215942**	N13:445171	29,416	+1:YMR088C;	1			
chrXIII	448884	449018	134	**-1,12528915**	N13:448865	8,222	-1:YMR090W;	-1			
chrXIII	448987	449140	153	**-0,973944237**	N13:449042	17,251	0:YMR090W;	0			
chrXIII	452811	452953	142	**-1,224030692**	N13:452872	8,276	+5:YMR092C;	5			
chrXIII	455777	455925	148	**-1,375167806**	N13:455844	17,963	+1:YMR094W;	1			
chrXIII	456607	456760	153	**-1,49553899**	N13:456671	16,196	+6:YMR094W;	6			
chrXIII	458537	458683	146	**-1,032910028**				Overlap <50 bp			
chrXIII	460130	460281	151	**-1,482220402**	N13:460189	8,583	+6:YMR097C;	6			
chrXIII	460228	460362	134	**-0,801576707**	N13:460349	11,389	+5:YMR097C;	5			

CHR	START	END	L(bp)	ΔLknuc	Nuc ID	Fuzziness	Gene ID	Gene body postion or intergene	Terminal	rDNA	Telomeric
chrXIII	460442	460567	125	-0,919323994	N13:460520	10,813	+4:YMR097C;	4			
chrXIII	460442	460595	153	-1,062350709	N13:460520	10,813	+4:YMR097C;	4			
chrXIII	460442	460598	156	-1,698620617	N13:460520	10,813	+4:YMR097C;	4			
chrXIII	460442	460600	158	-1,061570918	N13:460520	10,813	+4:YMR097C;	4			
chrXIII	461194	461345	151	-1,262757852	N13:461301	12,021	+9:YMR098C;	9			
chrXIII	464378	464520	142	-1,24412839	N13:464473	7,501	+3:YMR099C;	3			
chrXIII	467950	468100	150	-1,51287914				Overlap <50 bp			
chrXIII	470902	471051	149	-1,539207987	N13:470916	10,017	+10:YMR102C;	10			
chrXIII	473040	473146	106	-1,055787622	N13:473098	31,984	+13:YMR104C;	13			
chrXIII	473040	473162	122	-1,240734	N13:473098	31,984	+13:YMR104C;	13			
chrXIII	473911	474081	170	-1,061923504	N13:474025	7,925	+8:YMR104C;	8			
chrXIII	484473	484603	130	-2,197076681	N13:484551	12,657	+5:YMR108W;	5			
chrXIII	488956	489063	107	-1,422093102	N13:489016	37,901	+15:YMR109W;	15			
chrXIII	488956	489073	117	-1,619386548	N13:489016	37,901	+15:YMR109W;	15			
chrXIII	491261	491409	148	-1,327601077	N13:491313	8,886	+5:YMR110C;	5			
chrXIII	492970	493101	131	-1,110228497	N13:492992	35,329	+8:YMR111C;	8			
chrXIII	495547	495700	153	-1,138855301	N13:495639	20,75	+5:YMR113W;	5			
chrXIII	496801	496973	172	-1,311892877	N13:496786	17,487	+5*:YMR114C;	5	TERM		
chrXIII	496801	496973	172	-1,311892877	N13:496955	23,034	+4:YMR114C;	4			
chrXIII	499783	499912	129	-1,022804316	N13:499808	15,947		Intergene			
chrXIII	503855	504003	148	-1,099190138	N13:503924	27,185		Intergene			
chrXIII	505412	505581	169	-1,427235611	N13:505521	21,658	+3:YMR119W;	3			
chrXIII	512141	512286	145	-1,41928132	N13:512212	22,879	+8:CUT775;	8			
chrXIII	514521	514669	148	-1,161641121	N13:514629	8,424	+3:YMR124W;	3			
chrXIII	514553	514703	150	-1,58806141	N13:514629	8,424	+3:YMR124W;	3			
chrXIII	515323	515459	136	-1,36689729	N13:515420	23,756	+8:YMR124W;	8			
chrXIII	520223	520378	155	-1,17460798	N13:520207	21,349		Intergene			
chrXIII	520223	520378	155	-1,17460798	N13:520395	28,077		Intergene			
chrXIII	520376	520508	132	-0,654714499	N13:520395	28,077		Intergene			
chrXIII	521730	521901	171	-1,617508894	N13:521801	10,685	+2:YMR126C;	2			
chrXIII	522712	522817	105	-1,338817696	N13:522725	7,789	+5:YMR127C; +4*:anti141;	5			
chrXIII	528716	528857	141	-1,11894495	N13:528767	2,828	+7:YMR129W;	7			
chrXIII	533451	533610	159	-1,064703565	N13:533576	31,143	+8:YMR131C;	8			
chrXIII	535081	535246	165	-1,519159054	N13:535146	4,803	+2:CUT301; +4:YMR132C;	2			
chrXIII	536826	536976	150	-1,260422671	N13:536905	10,445	+5:YMR133W; +2:SUT732;	5			
chrXIII	537368	537507	139	-1,218431053	N13:537514	25,573	+8:YMR133W; -1:YMR134W;	8			
chrXIII	539637	539760	123	-1,244669314	N13:539719	8,937	+3:YMR135C;	3			
chrXIII	540611	540759	148	-1,347502544	N13:540749	18,556		Intergene			
chrXIII	541727	541880	153	-1,375830119	N13:541835	29,208	+4:YMR136W;	4			
chrXIII	549153	549302	149	-1,500657959	N13:549269	21,03	+11:YMR140W;	11			
chrXIII	549525	549679	154	-1,407554174	N13:549554	28,294	+13*:YMR140W;	13	TERM		
chrXIII	549713	549857	144	-1,30361384	N13:549747	27,727		Intergene			
chrXIII	555791	555929	138	-1,199100746	N13:555792	26,291	+6:YMR145C;	6			
chrXIII	562267	562429	162	-1,217407946	N13:562366	29,778	+2:YMR150C;	2			
chrXIII	562901	563062	161	-1,627779898	N13:562920	22,133	0:YMR152W;	0			
chrXIII	571585	571738	153	-1,411203072	N13:571681	19,23	-1:YMR156C; +3:YMR157C;	-1			
chrXIII	571590	571738	148	-1,27501725	N13:571681	19,23	-1:YMR156C; +3:YMR157C;	-1			
chrXIII	574736	574904	168	-0,917721721	N13:574834	9,867	-1:YMR160W; +1:YMR159C;	-1			
chrXIII	580311	580467	156	-1,109786367	N13:580401	13,467	+23:YMR162C;	23			
chrXIII	580644	580772	128	-1,15437328	N13:580704	28,693	+21:YMR162C;	21			
chrXIII	581583	581719	136	-1,320981759	N13:581638	19,344	+16:YMR162C;	16			
chrXIII	582011	582174	163	-1,160146188	N13:582099	17,761	+13:YMR162C;	13			
chrXIII	582013	582174	161	-1,263855346	N13:582099	17,761	+13:YMR162C;	13			
chrXIII	583110	583268	158	-1,168676087				Overlap <50 bp			
chrXIII	588471	588586	115	-1,351804913				Overlap <50 bp			
chrXIII	595003	595166	163	-1,31144452	N13:595084	4,183	+2:YMR167W;	2			
chrXIII	596303	596450	147	-1,087121469	N13:596386	17,111	+10:YMR167W;	10			
chrXIII	598087	598214	127	-1,686991525	N13:598137	11,929	+7:YMR168C;	7			
chrXIII	598887	599027	140	-1,226031277	N13:598952	3,435	+2:YMR168C;	2			
chrXIII	600432	600562	130	-1,018931095	N13:600483	40,294	+3:YMR169C;	3			
chrXIII	600662	600821	159	-1,376411267	N13:600688	6,658	+2:YMR169C;	2			
chrXIII	602872	603031	159	-1,369766695	N13:602904	37,477	+2:YMR170C;	2			
chrXIII	608199	608341	142	-1,229130495	N13:608209	24,222	+14*:YMR172W;	14	TERM		
chrXIII	614091	614242	151	-1,277209225	N13:614214	20,03	+17:YMR176W;	17			
chrXIII	617621	617773	152	-1,109055473	N13:617702	23,192	+8:YMR177W;	8			
chrXIII	617962	618111	149	-1,518148784	N13:618058	15,485	+10:YMR177W;	10			
chrXIII	622293	622443	150	-1,221823506	N13:622398	23,499	+8:YMR180C;	8			
chrXIII	627780	627908	128	-1,045984368	N13:627847	7,733	+1:YMR183C;	1			
chrXIII	627788	627919	131	-1,03335309	N13:627847	7,733	+1:YMR183C;	1			
chrXIII	634568	634713	145	-1,376256504	N13:634666	37,098	+9*:YMR187C;	9	TERM		
chrXIII	637620	637765	145	-1,462292351	N13:637702	7,287	+3:YMR189W;	3			
chrXIII	641481	641611	130	-0,245231112	N13:641533	33,088	+24:YMR190C;	24			
chrXIII	642381	642525	144	-1,089336123	N13:642458	32,517	+18:YMR190C;	18			
chrXIII	642389	642541	152	-1,243230254	N13:642458	32,517	+18:YMR190C;	18			
chrXIII	649168	649320	152	-1,725134006	N13:649263	12,554		Intergene			
chrXIII	652798	652958	160	-1,483894571	N13:652939	37,727	+1:YMR194C-B;	1			
chrXIII	657660	657789	129	-1,161476411	N13:657675	16,832	+5:CUT784;	5			
chrXIII	658250	658404	154	-1,429230454	N13:658346	11,692	+1:CUT784;	1			
chrXIII	658827	658990	163	-1,16555984	N13:658863	7,967	+3:YMR197C;	3			
chrXIII	658970	659105	135	-1,108034287	N13:659025	9,606	+2:YMR197C;	2			
chrXIII	660778	660927	149	-1,305258508	N13:660944	33,887	+10:YMR198W;	10			
chrXIII	662900	663052	152	-1,852514575	N13:662956	20,659	+4:YMR199W;	4			
chrXIII	663803	663949	146	-1,231901438	N13:663870	9,762	+9:YMR199W;	9			
chrXIII	663837	663944	107	-1,310336811	N13:663870	9,762	+9:YMR199W;	9			
chrXIII	663837	663953	116	-1,463453801	N13:663870	9,762	+9:YMR199W;	9			
chrXIII	667634	667789	155	-1,135562628	N13:667703	8,124	+2:YMR202W;	2			
chrXIII	671983	672128	145	-1,311176996	N13:672090	21,407	+17:YMR205C;	17			
chrXIII	675888	676037	149	-1,173041663	N13:675911	24,481	+1:YMR206W;	1			
chrXIII	675888	676037	149	-1,173041663	N13:676060	8,145	+2:YMR206W;	2			
chrXIII	675950	676037	87	-0,706323604	N13:676060	8,145	+2:YMR206W;	2			
chrXIII	675950	676105	155	-1,34728913	N13:676060	8,145	+2:YMR206W;	2			
chrXIII	677204	677344	140	-1,466891716	N13:677258	15,741	+40:YMR207C;	40			
chrXIII	677920	678066	146	-1,422725477	N13:677969	32,254	+36:YMR207C;	36			
chrXIII	678554	678723	169	-1,46359263	N13:678626	13,527	+32:YMR207C;	32			
chrXIII	679422	679570	148	-1,535292214				Overlap <50 bp			
chrXIII	680831	680955	124	-0,944302573	N13:680907	26	+18:YMR207C;	18			

CHR	START	END	L(bp)	ΔLknuc	Nuc ID	Fuzziness	Gene ID	Gene body postion or intergene	Terminal	rDNA	Telomeric
chrXIII	684362	684526	164	**-1,563087733**	N13:684432	13,442	0:YMR208W;	0			
chrXIII	686491	686656	165	**-1,24842551**	N13:686622	3,715	+5:YMR209C;	5			
chrXIII	692898	693052	154	**-1,560026231**	N13:692964	12,972	+2:YMR212C;	2			
chrXIII	693627	693771	144	**-1,15466149**	N13:693719	4,561	+3:YMR213W;	3			
chrXIII	693831	693958	127	**-1,130676747**	N13:693886	3,271	+4:YMR213W;	4			
chrXIII	694160	694314	154	**-1,176653897**	N13:694234	5,07	+6:YMR213W;	6			
chrXIII	694285	694446	161	**-1,388551281**				Overlap <50 bp			
chrXIII	694710	694842	132	**-1,049615381**	N13:694758	28,877	+9:YMR213W;	9			
chrXIII	711631	711787	156	**-1,116457697**	N13:711674	23,923		Intergene			
chrXIII	713556	713705	149	**-1,197033137**	N13:713622	32,896	+9:YMR220W;	9			
chrXIII	714522	714675	153	**-1,743643898**	N13:714616	14,48	+6:YMR221C;	6			
chrXIII	715186	715332	146	**-1,102109835**	N13:715254	5,727	+2:YMR221C;	2			
chrXIII	718847	718995	148	**-1,394105952**	N13:719006	38,532	+11:YMR224C;	11			
chrXIII	718847	718995	148	**-1,394105952**	N13:718849	32,887	+12:YMR224C;	12			
chrXIII	719890	720048	158	**-1,209884701**	N13:719976	9,453	+5:YMR224C;	5			
chrXIII	723055	723205	150	**-1,246705755**				Overlap <50 bp			
chrXIII	723972	724126	154	**-1,425963828**	N13:724061	7,23	+3:YMR227C;	3			
chrXIII	730943	731091	148	**-1,243015513**	N13:731008	3,92	+2:YMR229C;	2			
chrXIII	737903	738069	166	**-1,67434349**	N13:737964	20,992	+8:YMR232W;	8			
chrXIII	748521	748674	153	**-1,325026035**	N13:748606	19,524	+8:YMR239C;	8			
chrXIII	750522	750675	153	**-1,116784236**	N13:750581	5,177	+6:YMR240C;	6			
chrXIII	757851	758017	166	**-1,180891504**	N13:757933	6,76	+6:YMR244W;	6			
chrXIII	758839	758989	150	**-1,238349559**	N13:759009	35,487	+1:YMR244C-A;	1			
chrXIII	758839	758989	150	**-1,238349559**	N13:758860	52,169	+2:YMR244C-A;	2			
chrXIII	759376	759557	181	**-1,593404033**	N13:759498	23,295	0:YMR246W;	0			
chrXIII	765908	766066	158	**-1,130909835**	N13:766027	28,492	+14:YMR247C;	14			
chrXIII	772941	773097	156	**-1,22924665**	N13:773019	9,783	+2:YMR251W; +4:SUT738;	2			
chrXIII	773665	773803	138	**-1,254895417**	N13:773793	7,842	+7:YMR251W; -1:SUT738;	7			
chrXIII	773665	773807	142	**-1,235607642**	N13:773793	7,842	+7:YMR251W; -1:SUT738;	7			
chrXIII	774153	774312	159	**-0,896784979**	N13:774158	12,361	+9*:YMR251W;	9	TERM		
chrXIII	774153	774312	159	**-0,896784979**	N13:774317	12,276		Intergene			
chrXIII	778663	778795	132	**-1,435094604**	N13:778773	23,569	+6*:YMR255W; +3*:YMR256C;	6	TERM		
chrXIII	779035	779188	153	**-1,720841578**	N13:779109	3,033	+1:YMR256C;	1			
chrXIII	780072	780218	146	**-1,800675782**	N13:780137	27,495	+14:YMR257C;	14			
chrXIII	780801	780937	136	**-1,369242476**	N13:780898	64,519	+10:YMR257C;	10			
chrXIII	790225	790394	169	**-1,146081977**	N13:790301	17,725	+20:YMR261C;	20			
chrXIII	790368	790515	147	**-1,348560293**	N13:790486	14,46	+19:YMR261C;	19			
chrXIII	790640	790805	165	**-1,167242662**	N13:790824	29,813	+17:YMR261C;	17			
chrXIII	790640	790805	165	**-1,167242662**	N13:790646	7,228	+18:YMR261C;	18			
chrXIII	791775	791919	144	**-1,28525158**	N13:791864	4,573	+11:YMR261C;	11			
chrXIII	792265	792408	143	**-1,351420283**	N13:792303	22,621	+8:YMR261C;	8			
chrXIII	792607	792777	170	**-1,501527541**	N13:792794	10,661	+5:YMR261C;	5			
chrXIII	792607	792777	170	**-1,501527541**	N13:792641	13,077	+6:YMR261C;	6			
chrXIII	792935	793098	163	**-1,296089687**	N13:793117	6,481	+3:YMR261C;	3			
chrXIII	792935	793098	163	**-1,296089687**	N13:792956	12,872	+4:YMR261C;	4			
chrXIII	793020	793160	140	**-0,985563371**	N13:793117	6,481	+3:YMR261C;	3			
chrXIII	793713	793841	128	**-1,0337298**	N13:793740	8,847	-1:YMR261C; +1:YMR262W;	-1			
chrXIII	795911	796064	153	**-1,246971525**	N13:795966	4,336	+2:YMR264W;	2			
chrXIII	797737	797897	160	**-1,315848977**	N13:797874	16,582	+1:YMR265C;	1			
chrXIII	797737	797940	203	**-1,400772218**	N13:797874	16,582	+1:YMR265C;	1			
chrXIII	797778	797885	107	**-0,405682677**	N13:797874	16,582	+1:YMR265C;	1			
chrXIII	797778	797897	119	**-0,778151205**	N13:797874	16,582	+1:YMR265C;	1			
chrXIII	797778	797940	162	**-1,144257575**	N13:797874	16,582	+1:YMR265C;	1			
chrXIII	799674	799836	162	**-0,927437545**				Overlap <50 bp			
chrXIII	800670	800819	149	**-1,560584081**				Overlap <50 bp			
chrXIII	801962	802127	165	**-1,528671835**	N13:802061	7,791	+3:YMR267W;	3			
chrXIII	804104	804264	160	**-1,411894864**	N13:804203	7,055	+1:YMR268C; -1:YMR269W;	1			
chrXIII	806854	806962	108	**-1,113558627**	N13:806905	9,555	+6*:YMR271C;	6	TERM		
chrXIII	806855	806962	107	**-1,225583207**	N13:806905	9,555	+6*:YMR271C;	6	TERM		
chrXIII	808568	808720	152	**-1,437675613**				Overlap <50 bp			
chrXIII	811849	811978	129	**-1,044132696**	N13:811868	21,483	+15:YMR273C;	15			
chrXIII	816433	816597	164	**-1,43255335**	N13:816517	21,716	+14:YMR275C;	14			
chrXIII	819869	819975	106	**-1,073689103**	N13:819933	11,431	+8*:YMR276W; -1:YMR277W;	8	TERM		
chrXIII	819869	819988	119	**-1,305795421**	N13:819933	11,431	+8*:YMR276W; -1:YMR277W;	8	TERM		
chrXIII	820608	820710	102	**-1,070326901**	N13:820698	7,314	+4:YMR277W;	4			
chrXIII	820608	820711	103	**-0,815276084**	N13:820698	7,314	+4:YMR277W;	4			
chrXIII	820608	820715	107	**-1,549099066**	N13:820698	7,314	+4:YMR277W;	4			
chrXIII	820608	820717	109	**-0,819384402**	N13:820698	7,314	+4:YMR277W;	4			
chrXIII	820608	820733	125	**-1,386911372**	N13:820698	7,314	+4:YMR277W;	4			
chrXIII	820608	820737	129	**-1,270264164**	N13:820698	7,314	+4:YMR277W;	4			
chrXIII	820608	820738	130	**-1,519093893**	N13:820698	7,314	+4:YMR277W;	4			
chrXIII	820608	820740	132	**-0,914990682**	N13:820698	7,314	+4:YMR277W;	4			
chrXIII	820608	820741	133	**-1,551555228**	N13:820698	7,314	+4:YMR277W;	4			
chrXIII	820608	820742	134	**-1,373831624**	N13:820698	7,314	+4:YMR277W;	4			
chrXIII	820608	820743	135	**-1,275866383**	N13:820698	7,314	+4:YMR277W;	4			
chrXIII	820608	820744	136	**-1,784869815**	N13:820698	7,314	+4:YMR277W;	4			
chrXIII	820608	820745	137	**-1,668597069**	N13:820698	7,314	+4:YMR277W;	4			
chrXIII	820608	820746	138	**-1,323740327**	N13:820698	7,314	+4:YMR277W;	4			
chrXIII	820608	820747	139	**-1,275480863**	N13:820698	7,314	+4:YMR277W;	4			
chrXIII	820608	820748	140	**-1,851729879**	N13:820698	7,314	+4:YMR277W;	4			
chrXIII	820608	820749	141	**-1,199696077**	N13:820698	7,314	+4:YMR277W;	4			
chrXIII	820608	820750	142	**-1,951217718**	N13:820698	7,314	+4:YMR277W;	4			
chrXIII	820608	820751	143	**-1,165222752**	N13:820698	7,314	+4:YMR277W;	4			
chrXIII	820609	820751	142	**-1,290726288**	N13:820698	7,314	+4:YMR277W;	4			
chrXIII	820610	820751	141	**-1,206973874**	N13:820698	7,314	+4:YMR277W;	4			
chrXIII	820611	820751	140	**-1,398790791**	N13:820698	7,314	+4:YMR277W;	4			
chrXIII	820612	820751	139	**-1,195604628**	N13:820698	7,314	+4:YMR277W;	4			
chrXIII	820613	820751	138	**-0,940569628**	N13:820698	7,314	+4:YMR277W;	4			
chrXIII	820614	820751	137	**-1,546491319**	N13:820698	7,314	+4:YMR277W;	4			
chrXIII	820615	820751	136	**-1,540404258**	N13:820698	7,314	+4:YMR277W;	4			
chrXIII	820616	820751	135	**-1,495701512**	N13:820698	7,314	+4:YMR277W;	4			
chrXIII	820617	820751	134	**-1,240760607**	N13:820698	7,314	+4:YMR277W;	4			
chrXIII	820619	820751	132	**-1,30605236**	N13:820698	7,314	+4:YMR277W;	4			
chrXIII	820620	820751	131	**-1,04108562**	N13:820698	7,314	+4:YMR277W;	4			
chrXIII	820621	820751	130	**-0,997344479**	N13:820698	7,314	+4:YMR277W;	4			
chrXIII	820624	820751	127	**-1,320185462**	N13:820698	7,314	+4:YMR277W;	4			

CHR	START	END	L(bp)	ΔLknuc	Nuc ID	Fuzziness	Gene ID	Gene body postion or intergene	Terminal	rDNA	Telomeric
chrXIII	820626	820715	89	**-0,331672905**	N13:820698	7,314	+4:YMR277W;	4			
chrXIII	828572	828720	148	**-1,3167041**	N13:828670	12,4	+17:YMR280C;	17			
chrXIII	835990	836099	109	**-0,24432488**				Overlap <50 bp			
chrXIII	836333	836463	130	**-1,082004231**	N13:836461	20,877	+3:YMR283C;	3			
chrXIII	842098	842232	134	**-1,10215979**	N13:842139	11,743	+2:YMR286W;	2			
chrXIII	843431	843583	152	**-1,198985537**	N13:843529	44,584		Intergene			
chrXIII	847888	848017	129	**-1,133053485**	N13:847969	12,983	+15:YMR288W;	15			
chrXIII	847897	848050	153	**-0,976293861**	N13:847969	12,983	+15:YMR288W;	15			
chrXIII	851484	851633	149	**-1,686947621**	N13:851582	14,261	+1:YMR290C;	1			
chrXIII	864286	864433	147	**-1,607539689**	N13:864337	12,285	+7*:YMR299C;	7	TERM		
chrXIII	864644	864799	155	**-1,092414135**	N13:864820	7,548	+4:YMR299C;	4			
chrXIII	864644	864799	155	**-1,092414135**	N13:864653	29,305	+5:YMR299C;	5			
chrXIII	866005	866155	150	**-0,832483104**				Overlap <50 bp			
chrXIII	868732	868890	158	**-1,225352096**	N13:868810	10,644	+6:YMR301C;	6			
chrXIII	871472	871623	151	**-1,237526372**	N13:871631	10,741	+7:YMR302C;	7			
chrXIII	871472	871623	151	**-1,237526372**	N13:871471	13,502	+8:YMR302C;	8			
chrXIII	873640	873761	121	**-0,42410459**	N13:873682	27,883		Intergene			
chrXIII	873640	873789	149	**-1,205193959**	N13:873682	27,883		Intergene			
chrXIII	873640	873790	150	**-1,077202153**	N13:873682	27,883		Intergene			
chrXIII	873648	873761	113	**-0,453480734**	N13:873682	27,883		Intergene			
chrXIII	873648	873789	141	**-0,965957715**	N13:873682	27,883		Intergene			
chrXIII	873648	873790	142	**-1,157645955**	N13:873682	27,883		Intergene			
chrXIII	878188	878341	153	**-1,24823709**	N13:878290	25,795		Intergene			
chrXIII	879147	879302	155	**-1,340664549**	N13:879224	28,107	+8:YMR305C;	8			
chrXIII	884345	884496	151	**-1,46221069**	N13:884479	35,302	+7:CUT792;	7			
chrXIII	886126	886284	158	**-1,084449508**				Overlap <50 bp			
chrXIII	889335	889469	134	**-1,055223546**	N13:889367	19,917	+19:YMR308C;	19			
chrXIII	890068	890170	102	**-1,604195994**	N13:890188	29,579	+14:YMR308C;	14			
chrXIII	891024	891149	125	**-0,963025918**	N13:891052	13,096	+9:YMR308C;	9			
chrXIII	891955	892105	150	**-1,188509462**	N13:892008	3,782	+3:YMR308C;	3			
chrXIII	895348	895499	151	**-1,124761017**	N13:895429	10,616	+1:YMR309C;	1			
chrXIII	902827	902965	138	**-0,966455526**	N13:902909	40,773	+2:YMR315W;	2			
chrXIII	904201	904335	134	**-1,326281986**	N13:904278	13,693	+1:YMR315W-A;	1			
chrXIII	904428	904582	154	**-1,236642995**	N13:904475	14,106	+2*:YMR315W-A; -1:YMR316W;	2	TERM		
chrXIII	910695	910845	150	**-1,14410254**	N13:910757	14,404	+22:YMR317W; -1:SUT321;	22			
chrXIII	911000	911154	154	**-1,059289025**	N13:911157	31,729	+7*:YMR318C; +2:SUT321;	7	TERM		
chrXIII	917204	917355	151	**-1,477306761**	N13:917298	10,572		Intergene			
chrXIII	921093	921253	160	**-1,129205784**	N13:921174	15,275	+8:YMR323W;	8			
chrXIII	923830	923976	146	**-0,837624809**	N13:923958	13,026		Intergene			TEL RIGHT
chrXIII	924304	924426	122	**0,242412087**	N13:924361	19,218		Intergene			TEL RIGHT
chrXIII	924311	924426	115	**0,082325735**	N13:924361	19,218		Intergene			TEL RIGHT
chrXIII	924321	924426	105	**0,256129044**	N13:924361	19,218		Intergene			TEL RIGHT
chrXIV	614	765	151	**-1,26114644**	N14:689	23,756	+34:YNL339C;	34			TEL LEFT
chrXIV	953	1105	152	**-1,167005056**	N14:1005	17,039	+32:YNL339C;	32			TEL LEFT
chrXIV	1779	1930	151	**-1,23433831**	N14:1793	10,97	+28:YNL339C;	28			TEL LEFT
chrXIV	1791	1925	134	**-0,90324288**	N14:1793	10,97	+28:YNL339C;	28			TEL LEFT
chrXIV	1816	1912	96	**-0,909764122**	N14:1793	10,97	+28:YNL339C;	28			TEL LEFT
chrXIV	2170	2300	130	**-1,158833659**	N14:2318	20,075	+24:YNL339C;	24			TEL LEFT
chrXIV	2449	2601	152	**-0,933741302**	N14:2530	42,442	+23:YNL339C;	23			TEL LEFT
chrXIV	2796	2940	144	**-0,944837604**	N14:2849	32,347	+21:YNL339C;	21			TEL LEFT
chrXIV	2796	2983	187	**-0,653490955**	N14:2849	32,347	+21:YNL339C;	21			TEL LEFT
chrXIV	2848	2940	92	**-0,639460529**	N14:2849	32,347	+21:YNL339C;	21			TEL LEFT
chrXIV	2848	2983	135	**-0,96919127**	N14:2849	32,347	+21:YNL339C;	21			TEL LEFT
chrXIV	2936	3080	144	**-0,840272192**	N14:3031	21,127	+20:YNL339C;	20			TEL LEFT
chrXIV	3001	3153	152	**-1,288864118**	N14:3031	21,127	+20:YNL339C;	20			TEL LEFT
chrXIV	3253	3378	125	**-0,838300013**	N14:3371	17,616	+18:YNL339C;	18			TEL LEFT
chrXIV	3283	3426	143	**-1,60352687**	N14:3371	17,616	+18:YNL339C;	18			TEL LEFT
chrXIV	3584	3721	137	**-1,121172472**	N14:3651	8,718	+16:YNL339C;	16			TEL LEFT
chrXIV	3700	3855	155	**-1,689110747**	N14:3808	10,017	+15:YNL339C;	15			TEL LEFT
chrXIV	3794	3956	162	**-1,206881273**	N14:3967	7,211	+14:YNL339C;	14			TEL LEFT
chrXIV	3794	3956	162	**-1,206881273**	N14:3808	10,017	+15:YNL339C;	15			TEL LEFT
chrXIV	5019	5160	141	**-1,10344153**	N14:5067	19,672	+7:YNL339C;	7			TEL LEFT
chrXIV	15620	15770	150	**-1,616250702**	N14:15736	19,425	+7:YNL332W;	7			
chrXIV	18901	19054	153	**-1,137514125**	N14:18957	7,223	+3:YNL330C;	3			
chrXIV	22418	22569	151	**-1,128460354**	N14:22459	29,615	+6:YNL328C; +2:YNL329C;	6			
chrXIV	24850	25019	169	**-1,434748487**	N14:24952	26,405	+6:YNL327W;	6			
chrXIV	25836	25986	150	**-0,79842117**	N14:25940	33,589	+12:YNL327W;	12			
chrXIV	26530	26680	150	**-1,384838721**	N14:26673	28,253	+16:YNL327W;	16			
chrXIV	27268	27422	154	**-1,477611654**				Overlap <50 bp			
chrXIV	30249	30400	151	**-1,205813298**	N14:30360	10,854	+7:YNL325C;	7			
chrXIV	32059	32210	151	**-1,387798737**	N14:32102	7,649	+2:YNL323W;	2			
chrXIV	33454	33599	145	**-1,226884335**	N14:33540	30,632	+5:YNL322C;	5			
chrXIV	33454	33606	152	**-1,313828091**	N14:33540	30,632	+5:YNL322C;	5			
chrXIV	33459	33599	140	**-1,270636694**	N14:33540	30,632	+5:YNL322C;	5			
chrXIV	33461	33599	138	**-1,324245062**	N14:33540	30,632	+5:YNL322C;	5			
chrXIV	33461	33603	142	**-1,311005072**	N14:33540	30,632	+5:YNL322C;	5			
chrXIV	33461	33606	145	**-1,090493198**	N14:33540	30,632	+5:YNL322C;	5			
chrXIV	33464	33606	142	**-1,560819366**	N14:33540	30,632	+5:YNL322C;	5			
chrXIV	33466	33606	140	**-1,265952191**	N14:33540	30,632	+5:YNL322C;	5			
chrXIV	41403	41548	145	**-1,274830872**	N14:41462	15,123	+6:YNL317W;	6			
chrXIV	45261	45368	107	**-1,443529939**	N14:45343	13,171	+17*:YNL313C;	17	TERM		
chrXIV	45261	45374	113	**-1,749246433**	N14:45343	13,171	+17*:YNL313C;	17	TERM		
chrXIV	45783	45937	154	**-1,162964664**	N14:45851	24,025	+14:YNL313C;	14			
chrXIV	49049	49199	150	**-1,224991118**	N14:49135	9,96	+6:YNL312W;	6			
chrXIV	53101	53263	162	**-1,637356206**	N14:53189	11,462	+4:YNL309W;	4			
chrXIV	55301	55450	149	**-1,088164631**	N14:55396	16,82	+4:YNL308C;	4			
chrXIV	58407	58536	129	**-1,105045967**	N14:58525	21,102	+3:YNL306W;	3			
chrXIV	60113	60210	97	**-1,528967024**				Overlap <50 bp			
chrXIV	61184	61331	147	**-1,119756379**	N14:61254	14,37	+7:YNL304W;	7			
chrXIV	63570	63733	163	**-1,336708308**	N14:63662	16,41		Intergene			
chrXIV	65407	65535	128	**-1,213811438**	N14:65439	5,981	-1:Unit516; -1:YNL300W;	-1			
chrXIV	73936	74081	145	**-1,62742696**	N14:73929	21,37	+17:YNL297C;	17			
chrXIV	77741	77895	154	**-1,555767322**	N14:77801	39,107	+7:YNL295W;	7			
chrXIV	79985	80135	150	**-1,359485803**	N14:80056	4,367	+3:YNL294C;	3			
chrXIV	81420	81583	163	**-1,537239866**	N14:81459	11,295	+6:YNL293W;	6			
chrXIV	91093	91242	149	**-1,047291568**	N14:91178	3,435	+7:YNL288W;	7			

CHR	START	END	L(bp)	ΔLknuc	Nuc ID	Fuzziness	Gene ID	Gene body postion or intergene	Terminal	rDNA	Telomeric
chrXIV	93267	93403	136	**-1,169266664**	N14:93374	29,579	+11:YNL287W;	11			
chrXIV	97284	97435	151	**-1,491140008**	N14:97317	31,82		Intergene			
chrXIV	97941	98103	162	**-1,101504122**	N14:98029	22,332		Intergene			
chrXIV	99408	99557	149	**-1,055560615**	N14:99525	52,354		Intergene			
chrXIV	99632	99789	157	**-1,254768256**	N14:99713	23,643		Intergene			
chrXIV	99818	99968	150	**-1,064511842**	N14:99892	16,166		Intergene			
chrXIV	99822	99968	146	**-1,244579571**	N14:99892	16,166		Intergene			
chrXIV	100280	100415	135	**-1,201088959**	N14:100384	42,618		Intergene			
chrXIV	100932	101089	157	**-1,147249753**	N14:101020	32,14		Intergene			
chrXIV	102362	102510	148	**-1,416490541**	N14:102397	6,658		Intergene			
chrXIV	103973	104137	164	**-1,355647937**	N14:104026	5,167	+1:YNL284C; -1:CUT315;	1			
chrXIV	107707	107834	127	**-1,019781022**	N14:107700	12,287	+1:YNL282W;	1			
chrXIV	110079	110230	151	**-1,429310439**	N14:110172	5,465	+2:YNL280C;	2			
chrXIV	111620	111775	155	**-1,464656367**				Overlap <50 bp			
chrXIV	112009	112129	120	**-1,327286399**	N14:112032	11,846	+7:YNL279W; +6:SUT743;	7			
chrXIV	116452	116578	126	**-1,429161036**	N14:116519	22,941	278W; +1:CUT802; 0:YNL277W-A;	22	TERM		
chrXIV	119252	119378	126	**-1,017419608**	N14:119284	9,973	+13*:YNL277W; +1:YNL275W;	13	TERM		
chrXIV	121333	121464	131	**-0,837619266**	N14:121346	34,219	+6:YNL274C;	6			
chrXIV	124849	124975	126	**-0,993992213**	N14:124938	13,279	+14:YNL273W;	14			
chrXIV	129691	129843	152	**-1,609822261**	N14:129772	22,828		Intergene			
chrXIV	129719	129870	151	**-1,129973206**	N14:129772	22,828		Intergene			
chrXIV	131962	132135	173	**-1,631223871**	N14:131992	36,936	+22:YNL271C;	22			
chrXIV	132312	132463	151	**-1,533553729**	N14:132390	22,05	+20:YNL271C;	20			
chrXIV	133490	133654	164	**-1,465443021**	N14:133593	30,406	+13:YNL271C;	13			
chrXIV	134176	134325	149	**-1,211295875**	N14:134291	25,981	+9:YNL271C;	9			
chrXIV	135289	135440	151	**-1,240764846**	N14:135423	10,304	+2:YNL271C;	2			
chrXIV	135829	135979	150	**-1,193523366**	N14:135901	10,551	-1:YNL271C; +11:YNL270C;	-1			
chrXIV	139766	139921	155	**-0,647366343**	N14:139942	37,76	+10:YNL268W;	10			
chrXIV	140110	140269	159	**-1,279702947**	N14:140187	32,047	+11:YNL268W;	11			
chrXIV	141911	142060	149	**-0,98815193**	N14:142074	10,905	+9:YNL267W;	9			
chrXIV	150518	150667	149	**-1,08438067**	N14:150605	14,011	+14:YNL262W;	14			
chrXIV	153107	153271	164	**-1,547672375**	N14:153137	34,072	+29:YNL262W;	29			
chrXIV	153107	153271	164	**-1,547672375**	N14:153294	12,565	+30:YNL262W;	30			
chrXIV	155351	155502	151	**-1,205335001**	N14:155389	10,616	+3:YNL261W;	3			
chrXIV	163862	164000	138	**-1,219547147**	N14:164012	26,711	+3:YNL257C;	3			
chrXIV	163862	164000	138	**-1,219547147**	N14:163855	2,582	+4:YNL257C;	4			
chrXIV	165154	165280	126	**-1,446327677**	N14:165235	7,956	+5:YNL256W;	5			
chrXIV	167446	167585	139	**-1,339871234**	N14:167470	13,15	+3:YNL255C;	3			
chrXIV	174863	175007	144	**-1,354411165**	N14:174931	7,414	+1:CUT320;	1			
chrXIV	177654	177807	153	**-1,428142246**	N14:177732	36,891	+15:YNL250W;	15			
chrXIV	178329	178461	132	**-1,410790425**	N14:178391	25,861	+19:YNL250W;	19			
chrXIV	183769	183934	165	**-1,468010197**	N14:183818	8,963	+7:YNL247W;	7			
chrXIV	187776	187960	184	**-1,69099612**	N14:187755	32,449	-1:YNL243W; -1:YNL244C;	-1			
chrXIV	188930	189078	148	**-1,209118659**				Overlap <50 bp			
chrXIV	188930	189079	149	**-1,616432157**				Overlap <50 bp			
chrXIV	188930	189080	150	**-0,980180119**				Overlap <50 bp			
chrXIV	188930	189081	151	**-1,414159259**				Overlap <50 bp			
chrXIV	188930	189083	153	**-1,213376633**				Overlap <50 bp			
chrXIV	188931	189083	152	**-1,738335508**				Overlap <50 bp			
chrXIV	188933	189083	150	**-1,450303976**				Overlap <50 bp			
chrXIV	188934	189083	149	**-1,400315724**				Overlap <50 bp			
chrXIV	188935	189083	148	**-1,571354733**				Overlap <50 bp			
chrXIV	190891	191040	149	**-1,351077167**	N14:190930	12,014	+18*:YNL243W; 0:YNL242W;	18	TERM		
chrXIV	192308	192455	147	**-1,310095422**	N14:192383	27,389	+9:YNL242W;	9			
chrXIV	192813	192954	141	**-1,099248622**	N14:192825	36,813	+11:YNL242W;	11			
chrXIV	193504	193657	153	**-1,949134012**	N14:193627	36,56	+16:YNL242W;	16			
chrXIV	196760	196910	150	**-1,236028689**	N14:196922	9,678	+7:YNL241C; +36:YNL242W;	7			
chrXIV	202136	202292	156	**-0,982830772**	N14:202215	4,528	+1:YNL238W; -1:SUT744;	1			
chrXIV	203542	203662	120	**-0,178787426**	N14:203649	41,856	+10:YNL238W;	10			
chrXIV	206857	207023	166	**-1,701452663**	N14:206949	4,844	-1:anti149; +1:YNL236W;	-1			
chrXIV	210139	210288	149	**-1,259194618**	N14:210220	10,597	+1:YNL234W;	1			
chrXIV	216142	216308	166	**-1,340307249**	N14:216232	10,113	+7:YNL231C;	7			
chrXIV	219506	219633	127	**-0,927527921**	N14:219552	28,527	+6:YNL229C;	6			
chrXIV	224826	224979	153	**-1,436847002**	N14:224898	10,342	+15*:YNL224C;	15	TERM		
chrXIV	227320	227476	156	**-1,421656323**	N14:227397	6,229	-1:YNL224C; +1:YNL223W;	-1			
chrXIV	235082	235232	150	**-1,279105066**				Overlap <50 bp			
chrXIV	238200	238369	169	**-1,579285701**	N14:238254	4,546	+1:YNL218W;	1			
chrXIV	246737	246897	160	**-1,742123121**	N14:246814	5,888	+3:YNL213C;	3			
chrXIV	247839	247988	149	**-1,271747645**	N14:247931	11,584	+4:YNL212W;	4			
chrXIV	254524	254675	151	**-1,249069467**	N14:254603	21,237	+2:YNL208W;	2			
chrXIV	257454	257590	136	**-1,106078162**	N14:257486	20,957	+5:YNL206C;	5			
chrXIV	257743	257893	150	**-1,287766811**	N14:257796	10,281	+3:YNL206C;	3			
chrXIV	259132	259283	151	**-0,933668643**	N14:259278	34,57	-1:YNL202W; +1:YNL204C;	-1			
chrXIV	259132	259283	151	**-0,933668643**	N14:259110	26,195	+2:YNL204C;	2			
chrXIV	265572	265700	128	**-0,938916111**	N14:265642	18,97	+7:YNL199C;	7			
chrXIV	271513	271667	154	**-1,30275635**	N14:271563	21,615	+3*:YNL195C;	3	TERM		
chrXIV	273060	273209	149	**-1,252850273**	N14:273068	42,111	+6:SUT326; +5:YNL194C;	6			
chrXIV	273060	273209	149	**-1,252850273**	N14:273226	15,642	+7:SUT326; +4:YNL194C;	7			
chrXIV	275916	276056	140	**-0,992321868**	N14:276038	27,532	+11*:YNL193W; +1:CUT806;	11	TERM		
chrXIV	281515	281622	107	**-1,091421709**	N14:281537	13,653	+8*:YNL191W;	8	TERM		
chrXIV	281515	281631	116	**-1,547908334**	N14:281537	13,653	+8*:YNL191W;	8	TERM		
chrXIV	281832	281976	144	**-0,833205804**	N14:281889	14,048	-1:YNL190W;	-1			
chrXIV	283804	283989	185	**-1,493365917**	N14:283836	11,916	-1:YNL189W;	-1			
chrXIV	287747	287882	135	**-0,832213936**	N14:287841	8,264	+1:YNL187W;	1			
chrXIV	291077	291208	131	**-0,968804661**	N14:291175	15,438	+12:YNL186W;	12			
chrXIV	292461	292633	172	**-1,466114481**	N14:292551	7,468	+2:YNL185C;	2			
chrXIV	292477	292630	153	**-1,238017976**	N14:292551	7,468	+2:YNL185C;	2			
chrXIV	296854	297025	171	**-1,388951839**	N14:296864	42,64	+6:YNL182C;	6			
chrXIV	297410	297562	152	**-1,020240364**	N14:297470	12,422	+2:YNL182C;	2			
chrXIV	298390	298549	159	**-1,375091251**	N14:298469	10,677	+2:YNL181W;	2			
chrXIV	302946	303074	128	**-1,218272057**	N14:302995	12,05	+3:YNL178W;	3			
chrXIV	303880	304030	150	**-1,501278913**	N14:303933	10,068	+5:YNL177C;	5			
chrXIV	307042	307199	157	**-1,413968104**	N14:307110	8,998	+1:YNL176C;	1			
chrXIV	308271	308446	175	**-1,496761126**	N14:308444	10,52	+2:YNL175C;	2			
chrXIV	308271	308446	175	**-1,496761126**	N14:308284	6,325	+3:YNL175C;	3			
chrXIV	309709	309860	151	**-1,365820068**	N14:309803	2,872	+2:YNL173C;	2			

CHR	START	END	L(bp)	ΔLknuc	Nuc ID	Fuzziness	Gene ID	Gene body postion or intergene	Terminal	rDNA	Telomeric
chrXIV	310999	311146	147	**-1,276716916**	N14:311066	8,302	+3:YNL172W;	3			
chrXIV	314726	314877	151	**-1,228084953**	N14:314793	16,072	+27:YNL172W;	27			
chrXIV	317936	318050	114	**-1,965967979**	N14:318021	5,241	-1:YNL169C; +6:YNL168C;	-1			
chrXIV	324620	324765	145	**-1,334124924**	N14:324681	26,049	+6:YNL165W;	6			
chrXIV	324977	325117	140	**-1,083912587**	N14:325028	13,091	+8*:YNL165W;	8	TERM		
chrXIV	336864	337018	154	**-0,77102584**	N14:336949	6,626	+5:SUT747; +4:YNL160W;	5			
chrXIV	339979	340132	153	**-1,647791967**	N14:340063	7,369	+4:YNL158W; -1:YNL157W;	4			
chrXIV	340143	340297	154	**-1,312095718**				Overlap <50 bp			
chrXIV	343939	344095	156	**-1,517207307**	N14:344007	20,224	+9:YNL154C; +14:YNL153C;	9			
chrXIV	348424	348579	155	**-1,19030425**	N14:348518	11,514	+1:YNL151C; -1:CUT332;	1			
chrXIV	349341	349497	156	**-1,496164237**	N14:349432	8,622	+3*:YNL149C;	3	TERM		
chrXIV	357765	357910	145	**-1,164676079**	N14:357862	10,7	+5:YNL142W;	5			
chrXIV	357767	357910	143	**-1,282193499**	N14:357862	10,7	+5:YNL142W;	5			
chrXIV	358288	358439	151	**-1,269640804**	N14:358379	41,494	+8:YNL142W;	8			
chrXIV	360600	360759	159	**-1,527373813**	N14:360625	33,342		Intergene			
chrXIV	360762	360912	150	**-1,549346224**	N14:360803	10,21	+33*:YNL139C;	33	TERM		
chrXIV	361214	361352	138	**-1,326493272**	N14:361265	29,938	+30:YNL139C;	30			
chrXIV	364273	364404	131	**-1,230929249**	N14:364350	8,386	+10:YNL139C;	10			
chrXIV	365246	365404	158	**-1,354080023**	N14:365272	14,66	+4:YNL139C;	4			
chrXIV	366145	366271	126	**-1,347954433**				Overlap <50 bp			
chrXIV	368092	368240	148	**-1,132118715**	N14:368205	22,678	+10:YNL138W;	10			
chrXIV	369049	369214	165	**-1,715226837**	N14:369138	22,052	+7:YNL137C;	7			
chrXIV	376927	377089	162	**-1,26822994**	N14:377019	12,755	+12:YNL132W;	12			
chrXIV	384387	384546	159	**-1,267276163**	N14:384456	9,642	+4:YNL127W;	4			
chrXIV	388430	388576	146	**-1,489183971**	N14:388511	8	+9:YNL126W;	9			
chrXIV	388719	388880	161	**-1,332754204**	N14:388701	22,067	+10:YNL126W;	10			
chrXIV	388719	388880	161	**-1,332754204**	N14:388900	32,085	+11:YNL126W;	11			
chrXIV	396710	396865	155	**-1,260681051**	N14:396752	40,296	+14:YNL123W;	14			
chrXIV	403433	403582	149	**-1,486166722**	N14:403451	27,415	+14:YNL118C;	14			
chrXIV	403995	404145	150	**-1,172944818**				Overlap <50 bp			
chrXIV	410286	410440	154	**-1,180062561**	N14:410457	49,662	+10:YNL115C;	10			
chrXIV	410286	410440	154	**-1,180062561**	N14:410304	41,071	+11:YNL115C;	11			
chrXIV	410317	410470	153	**-1,210023063**	N14:410457	49,662	+10:YNL115C;	10			
chrXIV	410317	410470	153	**-1,210023063**	N14:410304	41,071	+11:YNL115C;	11			
chrXIV	410968	411126	158	**-1,214561599**	N14:411083	29,918	+7:YNL115C;	7			
chrXIV	411008	411145	137	**-1,212747297**	N14:411083	29,918	+7:YNL115C;	7			
chrXIV	416136	416292	156	**-1,312234213**	N14:416212	15,789		Intergene			
chrXIV	418446	418610	164	**-1,755455222**	N14:418509	14,55	+1:YNL110C;	1			
chrXIV	419242	419381	139	**-1,367723137**	N14:419334	12,612	+4:YNL108C;	4			
chrXIV	420269	420420	151	**-1,52496795**	N14:420290	10,257	+2:YNL107W;	2			
chrXIV	421794	421931	137	**-1,201970375**	N14:421859	28,993	+18:YNL106C;	18			
chrXIV	421911	422067	156	**-1,47079972**	N14:422017	26,329	+17:YNL106C;	17			
chrXIV	423473	423612	139	**-1,303502101**	N14:423543	28,726	+8:YNL106C;	8			
chrXIV	423497	423650	153	**-1,328487487**	N14:423543	28,726	+8:YNL106C;	8			
chrXIV	426375	426492	117	**-1,486910803**	N14:426395	26,397	+4:YNL104C;	4			
chrXIV	430126	430279	153	**-1,986508783**	N14:430203	18,608	+2:YNL102W;	2			
chrXIV	430126	430286	160	**-1,297415892**	N14:430203	18,608	+2:YNL102W;	2			
chrXIV	439717	439834	117	**-1,303695242**	N14:439718	28,219	+6:YNL098C;	6			
chrXIV	459511	459626	115	**-1,718924657**	N14:459588	4,359	-1:CUT813; +15:YNL088W;	-1			
chrXIV	460164	460311	147	**-1,359910805**	N14:460238	28,077	+19:YNL088W;	19			
chrXIV	464412	464538	126	**-1,277312664**	N14:464472	47,776	+14:YNL087W;	14			
chrXIV	466515	466659	144	**-1,142111846**	N14:466599	7,537	JT748; -1:YNL085W; +4:YNL086W;	1			
chrXIV	467295	467445	150	**-1,431666583**				Overlap <50 bp			
chrXIV	473999	474106	107	**-1,506815325**	N14:474082	8,989	+18:YNL083W; +5:YNL082W;	18			
chrXIV	475462	475606	144	**-1,059117993**	N14:475517	36,679	+26:YNL083W; +13:YNL082W;	26			
chrXIV	477594	477739	145	**-1,098361612**	N14:477658	22,863	+4:YNL080C;	4			
chrXIV	478141	478275	134	**-1,297732191**	N14:478165	17,994	+1:YNL080C;	1			
chrXIV	483192	483305	113	**-1,576537317**				Overlap <50 bp			
chrXIV	483272	483422	150	**-1,496162295**	N14:483343	6,463	+1:YNL076W;	1			
chrXIV	483529	483666	137	**-1,834991947**	N14:483509	3,189	+2:YNL076W;	2			
chrXIV	483529	483666	137	**-1,834991947**	N14:483683	5,82	+3:YNL076W; +11*:CUT815;	3			
chrXIV	483752	483901	149	**-1,201039797**	N14:483855	18,801	+4:YNL076W; +10:CUT815;	4			
chrXIV	484694	484843	149	**-1,290990913**				Overlap <50 bp			
chrXIV	490422	490573	151	**-1,425680975**	N14:490508	4,708	+2:YNL072W;	2			
chrXIV	490584	490699	115	**-0,601453223**	N14:490670	9,039	+3:YNL072W;	3			
chrXIV	490584	490718	134	**-1,203006182**	N14:490670	9,039	+3:YNL072W;	3			
chrXIV	490584	490722	138	**-1,122780673**	N14:490670	9,039	+3:YNL072W;	3			
chrXIV	500789	500918	129	**-0,75289546**	N14:500830	31,204		Intergene			
chrXIV	502692	502828	136	**-1,047673619**	N14:502692	35,576	+7:YNL066W;	7			
chrXIV	511107	511261	154	**-1,166954852**				Overlap <50 bp			
chrXIV	515776	515877	101	**-1,669420801**	N14:515831	8,689		Intergene			
chrXIV	516941	517091	150	**-0,922225939**	N14:517027	28,81	'NL058C; 0:YNL056W; +1:Unit525;	-1			
chrXIV	517457	517589	132	**-1,191756178**	N14:517452	9,67	+2:YNL056W;	2			
chrXIV	517457	517589	132	**-1,191756178**	N14:517609	6,812	+3:YNL056W;	3			
chrXIV	520229	520363	134	**-1,00091766**	N14:520294	52,37		Intergene			
chrXIV	520229	520369	140	**-1,117032968**	N14:520294	52,37		Intergene			
chrXIV	521272	521407	135	**-1,103410358**	N14:521305	5,508		Intergene			
chrXIV	521719	521865	146	**-1,222310659**	N14:521781	16,371		Intergene			
chrXIV	521719	521869	150	**-1,079684528**	N14:521781	16,371		Intergene			
chrXIV	521898	522055	157	**-1,358889802**	N14:521995	35,171		Intergene			
chrXIV	524015	524129	114	**-1,620338374**				Overlap <50 bp			
chrXIV	524719	524868	149	**-1,275098222**	N14:524853	25,482		Intergene			
chrXIV	524979	525110	131	**-1,30352413**	N14:525025	32,441		Intergene			
chrXIV	526013	526193	180	**-1,528319167**	N14:526078	8,931	-1:CUT816; +1:YNL054W;	-1			
chrXIV	526651	526800	149	**-1,273351845**	N14:526736	8,983	+5:YNL054W;	5			
chrXIV	527097	527235	138	**-1,270138369**	N14:527172	40,714	+8:YNL054W;	8			
chrXIV	527709	527871	162	**-1,188629644**	N14:527797	30,415	+12:YNL054W;	12			
chrXIV	529282	529444	162	**-1,15753479**	N14:529327	11,692	+20:YNL054W; +2:CUT817;	20			
chrXIV	529934	530079	145	**-1,310222683**	N14:530020	33,33	L054W; +2:YNL053W; +9:SUT749;	24			
chrXIV	530913	531056	143	**-0,766218273**				Overlap <50 bp			
chrXIV	532041	532181	140	**-1,213441118**	N14:532069	19,07	+3:YNL052W;	3			
chrXIV	532139	532286	147	**-1,244330597**	N14:532293	15,769	+4*:YNL052W; -1:YNL051W;	4	TERM		
chrXIV	536984	537144	160	**-1,460141096**	N14:537118	6,238	+6:YNL049C;	6			
chrXIV	538071	538217	146	**-1,16222764**	N14:538181	6,919	+1:YNL048W; -1:YNL049C;	1			
chrXIV	538102	538265	163	**-1,481478864**	N14:538181	6,919	+1:YNL048W; -1:YNL049C;	1			
chrXIV	538210	538368	158	**-1,482036228**	N14:538353	9,503	+2:YNL048W;	2			

CHR	START	END	L(bp)	ΔLknuc	Nuc ID	Fuzziness	Gene ID	Gene body postion or intergene	Terminal	rDNA	Telomeric
chrXIV	540636	540777	141	**-1,177071853**	N14:540797	32,015	+8:YNL047C;	8			
chrXIV	541287	541432	145	**-1,043596289**	N14:541365	16,092	+4:YNL047C;	4			
chrXIV	541780	541943	163	**-1,349717136**	N14:541870	13,258	+1:YNL047C;	1			
chrXIV	541920	542078	158	**-1,111962186**				Overlap <50 bp			
chrXIV	542491	542640	149	**-1,149126739**	N14:542475	11,547	NL046W; -1:YNL045W; +4:SUT750;	2	TERM		
chrXIV	544884	545027	143	**-1,360243496**	N14:544952	4,082	-1:YNL044W;	-1			
chrXIV	544927	545097	170	**-1,252177745**	N14:544952	4,082	-1:YNL044W;	-1			
chrXIV	546338	546446	108	**-0,888477193**	N14:546423	10,727		Intergene			
chrXIV	546358	546520	162	**-1,222365039**	N14:546423	10,727		Intergene			
chrXIV	551772	551902	130	**-1,277782883**	N14:551848	6,797	+2:YNL041C;	2			
chrXIV	552571	552722	151	**-1,375558203**	N14:552590	10,708		Intergene			
chrXIV	553534	553684	150	**-1,304337208**	N14:553556	9,18	+2:YNL040W;	2			
chrXIV	554534	554685	151	**-1,331815719**	N14:554609	11,149	+9:YNL040W;	9			
chrXIV	555938	556085	147	**-1,067095481**	N14:555999	12,58	+18:YNL040W; +7:YNL039W;	18			
chrXIV	557972	558127	155	**-1,273270097**	N14:558022	18,912	+7:YNL037C;	7			
chrXIV	560057	560198	141	**-1,406557556**	N14:560145	19,856	+4*:YNL036W;	4	TERM		
chrXIV	561823	561978	155	**-1,727683534**	N14:561825	41,073		Intergene			
chrXIV	562224	562373	149	**-1,513251688**	N14:562251	14,64		Intergene			
chrXIV	563011	563177	166	**-1,890085624**	N14:563098	21,939		Intergene			
chrXIV	564309	564452	143	**-1,113376298**	N14:564390	19,502		Intergene			
chrXIV	565788	565917	129	**-1,372879427**	N14:565899	24,664		Intergene			
chrXIV	567163	567273	110	**-1,472287582**	N14:567239	17,301		Intergene			
chrXIV	573304	573437	133	**-1,283556835**	N14:573369	27,074	+4:YNL033W;	4			
chrXIV	575453	575613	160	**-1,509219608**	N14:575487	22,93	+4*:YNL031C;	4	TERM		
chrXIV	575453	575613	160	**-1,509219608**	N14:575635	37,233	+3:YNL031C;	3			
chrXIV	576714	576844	130	**-0,680942025**	N14:576799	11,846	+1:YNL030W;	1			
chrXIV	576714	576864	150	**-1,30542841**	N14:576799	11,846	+1:YNL030W;	1			
chrXIV	578365	578517	152	**-1,13337748**	N14:578459	6,022	+3:YNL029C;	3			
chrXIV	578706	578859	153	**-1,211270454**	N14:578787	6,979	+1:YNL029C;	1			
chrXIV	585214	585366	152	**-1,295369037**	N14:585310	8,258	+1:YNL025C;	1			
chrXIV	589016	589141	125	**-1,193717391**	N14:589151	10,986	+13:YNL023C;	13			
chrXIV	589016	589141	125	**-1,193717391**	N14:589003	31,456	+14:YNL023C;	14			
chrXIV	593245	593393	148	**-1,223835274**	N14:593327	8,343	+2:YNL021W;	2			
chrXIV	597466	597618	152	**-0,995805705**	N14:597483	10,815	+2:YNL020C;	2			
chrXIV	598791	598924	133	**-1,415326611**	N14:598861	23,352	+3:YNL019C;	3			
chrXIV	602237	602346	109	**-0,26498165**	N14:602324	11		Intergene			
chrXIV	612323	612470	147	**-1,179312121**	N14:612426	45,358	+6:YNL011C;	6			
chrXIV	615779	615947	168	**-1,672435031**	N14:615899	17,367	+7:YNL009W;	7			
chrXIV	620267	620398	131	**-1,502545596**	N14:620245	11,389	+2:YNL006W;	2			
chrXIV	620267	620398	131	**-1,502545596**	N14:620397	6,501	+3:YNL006W;	3			
chrXIV	620785	620892	107	**-1,343314104**	N14:620770	28,87	+5:YNL006W;	5			
chrXIV	620785	620946	161	**-1,346740387**	N14:620770	28,87	+5:YNL006W;	5			
chrXIV	620785	620946	161	**-1,346740387**	N14:620937	39,069	+6:YNL006W;	6			
chrXIV	625000	625108	108	**-0,995500869**	N14:625110	27,682	+5*:YNL003C;	5	TERM		
chrXIV	625453	625609	156	**-1,358908464**	N14:625501	15,253	+3:YNL003C;	3			
chrXIV	630331	630483	152	**-1,383371137**	N14:630418	30,1	+6:YNR001C;	6			
chrXIV	630745	630896	151	**-1,183985668**	N14:630914	23,664	+3:YNR001C;	3			
chrXIV	630745	630896	151	**-1,183985668**	N14:630752	21,175	+4:YNR001C;	4			
chrXIV	635632	635775	143	**-1,514614084**	N14:635681	12,708	Init535; +2:YNR004W; +8:SUT753;	2			
chrXIV	640348	640505	157	**-1,21680964**	N14:640377	24,696	+1:YNR008W; -1:YNR007C;	1			
chrXIV	640382	640540	158	**-1,25860342**	N14:640377	24,696	+1:YNR008W; -1:YNR007C;	1			
chrXIV	640382	640540	158	**-1,25860342**	N14:640553	22,991	+2:YNR008W;	2			
chrXIV	641911	642059	148	**-1,373270044**	N14:641943	22,14	+10:YNR008W;	10			
chrXIV	645323	645472	149	**-1,271431502**	N14:645480	38,301	+10:YNR011C;	10			
chrXIV	645323	645472	149	**-1,271431502**	N14:645332	11,923	+11:YNR011C;	11			
chrXIV	646222	646370	148	**-1,241071186**	N14:646249	13,442	+5:YNR011C;	5			
chrXIV	649139	649287	148	**-1,440489374**	N14:649302	9,309	+16:YNR013C;	16			
chrXIV	649139	649287	148	**-1,440489374**	N14:649141	26,986	+17:YNR013C;	17			
chrXIV	659033	659204	171	**-1,385782074**	N14:659107	12,871	+15:YNR016C;	15			
chrXIV	660122	660273	151	**-1,415174214**				Overlap <50 bp			
chrXIV	660694	660857	163	**-1,278387054**	N14:660797	11,719	+5:YNR016C;	5			
chrXIV	669731	669882	151	**-1,126260292**	N14:669825	8,28	+3*:YNR022C;	3	TERM		
chrXIV	673304	673429	125	**-2,233527638**	N14:673428	50,012	+9:YNR026C;	9			
chrXIV	673304	673441	137	**-1,467748695**	N14:673428	50,012	+9:YNR026C;	9			
chrXIV	673304	673449	145	**-1,703894619**	N14:673428	50,012	+9:YNR026C;	9			
chrXIV	673304	673450	146	**-1,367126653**	N14:673428	50,012	+9:YNR026C;	9			
chrXIV	673304	673451	147	**-1,689340989**	N14:673428	50,012	+9:YNR026C;	9			
chrXIV	673304	673452	148	**-1,566576302**	N14:673428	50,012	+9:YNR026C;	9			
chrXIV	673304	673453	149	**-1,478425504**	N14:673428	50,012	+9:YNR026C;	9			
chrXIV	673304	673454	150	**-1,546023022**	N14:673428	50,012	+9:YNR026C;	9			
chrXIV	673304	673455	151	**-1,404266426**	N14:673428	50,012	+9:YNR026C;	9			
chrXIV	673305	673455	150	**-2,148858369**	N14:673428	50,012	+9:YNR026C;	9			
chrXIV	673306	673455	149	**-1,857060061**	N14:673428	50,012	+9:YNR026C;	9			
chrXIV	673307	673455	148	**-2,296895662**	N14:673428	50,012	+9:YNR026C;	9			
chrXIV	673308	673455	147	**-1,666660036**	N14:673428	50,012	+9:YNR026C;	9			
chrXIV	673309	673455	146	**-1,486343852**	N14:673428	50,012	+9:YNR026C;	9			
chrXIV	673310	673455	145	**-1,930751251**	N14:673428	50,012	+9:YNR026C;	9			
chrXIV	673311	673455	144	**-1,623067519**	N14:673428	50,012	+9:YNR026C;	9			
chrXIV	673313	673455	142	**-2,230529351**	N14:673428	50,012	+9:YNR026C;	9			
chrXIV	673314	673455	141	**-2,225408159**	N14:673428	50,012	+9:YNR026C;	9			
chrXIV	673321	673455	134	**-1,716359174**	N14:673428	50,012	+9:YNR026C;	9			
chrXIV	673329	673455	126	**-1,294813587**	N14:673428	50,012	+9:YNR026C;	9			
chrXIV	673357	673455	98	**-1,532138512**	N14:673428	50,012	+9:YNR026C;	9			
chrXIV	675190	675330	140	**-0,865323734**	N14:675271	10,289	+3:YNR027W;	3			
chrXIV	678978	679124	146	**-1,106139223**	N14:679095	14,675	+3:YNR030W;	3			
chrXIV	679561	679717	156	**-1,46338089**	N14:679584	16,502	+6:YNR030W; +36*:YNR031C;	6			
chrXIV	681968	682096	128	**-1,220429156**	N14:682040	42,039	+22:YNR031C;	22			
chrXIV	681972	682075	103	**-0,800444388**	N14:682040	42,039	+22:YNR031C;	22			
chrXIV	682856	682998	142	**-1,544416865**	N14:682935	12,55	+17:YNR031C;	17			
chrXIV	689540	689646	106	**-1,94585362**	N14:689607	12,133	+12:YNR033W;	12			
chrXIV	689540	689701	161	**-1,431265612**	N14:689607	12,133	+12:YNR033W;	12			
chrXIV	689540	689704	164	**-1,835952551**	N14:689607	12,133	+12:YNR033W;	12			
chrXIV	689540	689705	165	**-1,261808255**	N14:689607	12,133	+12:YNR033W;	12			
chrXIV	689541	689705	164	**-1,664238324**	N14:689607	12,133	+12:YNR033W;	12			
chrXIV	689543	689705	162	**-1,45365219**	N14:689607	12,133	+12:YNR033W;	12			
chrXIV	689545	689705	160	**-1,764409185**	N14:689607	12,133	+12:YNR033W;	12			

CHR	START	END	L(bp)	ΔLknuc	Nuc ID	Fuzziness	Gene ID	Gene body postion or intergene	Terminal	rDNA	Telomeric
chrXIV	689550	689705	155	**-1,682624926**	N14:689607	12,133	+12:YNR033W;	12			
chrXIV	689554	689705	151	**-1,627769115**	N14:689607	12,133	+12:YNR033W;	12			
chrXIV	694543	694689	146	**-0,912898457**	N14:694619	4,528	+2:YNR036C;	2			
chrXIV	694549	694699	150	**-0,861380024**	N14:694619	4,528	+2:YNR036C;	2			
chrXIV	698880	699032	152	**-1,220726368**	N14:698920	23,309	+4:YNR039C;	4			
chrXIV	704009	704157	148	**-1,03615079**	N14:704120	18,735	+3:YNR044W;	3			
chrXIV	704660	704785	125	**-0,492852651**	N14:704768	7,396	+7:YNR044W;	7			
chrXIV	705096	705262	166	**-1,430998624**	N14:705073	15,822	+9:YNR044W;	9			
chrXIV	705096	705262	166	**-1,430998624**	N14:705244	36,116	+10:YNR044W;	10			
chrXIV	705533	705682	149	**-1,193765994**	N14:705572	14,825	+12*:YNR044W; +3:SUT755;	12	TERM		
chrXIV	705820	705974	154	**-1,36754358**	N14:705901	36,918	-1:YNR045W; +1:SUT755;	-1			
chrXIV	706013	706167	154	**-1,26020257**	N14:706150	8,524	+1:YNR045W; -1:SUT755;	1			
chrXIV	706675	706825	150	**-1,215214974**	N14:706652	7,815	+4:YNR045W;	4			
chrXIV	706675	706825	150	**-1,215214974**	N14:706826	11,431	+5:YNR045W;	5			
chrXIV	707449	707602	153	**-1,492744118**	N14:707511	10,262	R045W; +1:CUT819; -1:YNR046W;	9			
chrXIV	723355	723513	158	**-1,10714188**	N14:723439	9,011	+6:YNR054C; +1:CUT351;	6			
chrXIV	724785	724950	165	**-1,295109362**	N14:724864	13,759	+3:SUT339;	3			
chrXIV	727236	727386	150	**-1,086788959**	N14:727324	23,881		Intergene			
chrXIV	727633	727771	138	**-1,344328093**	N14:727696	16,802	-1:SUT340;	-1			
chrXIV	731977	732139	162	**-1,443442498**	N14:732086	8,287	+8:YNR056C; +5:SUT342;	8			
chrXIV	735524	735689	165	**-1,57866689**	N14:735604	19,149	+8*:YNR058W;	8	TERM		
chrXIV	735878	736030	152	**-1,083220261**	N14:735951	20,216	+2*:SUT756;	2	TERM		
chrXIV	737786	737917	131	**-1,376817347**	N14:737841	21,267	+8:YNR059W;	8			
chrXIV	749149	749259	110	**-1,11729556**	N14:749152	22,888	+15:YNR063W; +6:YNR064C;	15			
chrXIV	749725	749855	130	**-1,507726378**	N14:749877	26,846	+2:YNR064C;	2			
chrXIV	749823	749962	139	**-0,934189943**	N14:749877	26,846	+2:YNR064C;	2			
chrXIV	750208	750335	127	**-1,62798518**				Overlap <50 bp			
chrXIV	753363	753527	164	**-1,680839786**	N14:753511	24,417	+10:YNR066C; +3:YNR065C;	10			
chrXIV	754387	754526	139	**-1,221453886**	N14:754466	21,424	+4:YNR066C;	4			
chrXIV	761541	761643	102	**-1,724150947**	N14:761639	45,249	+9:YNR069C;	9			
chrXIV	763360	763525	165	**-1,369016262**	N14:763380	19,259	+3:CUT353;	3			
chrXIV	763360	763525	165	**-1,369016262**	N14:763542	13,737	+4:CUT353;	4			
chrXIV	767084	767240	156	**-1,376752679**	N14:767184	24,921	+11:YNR070W; +14:SUT760;	11			
chrXIV	773470	773620	150	**-1,105933232**	N14:773461	25,007	+6:YNR072W; +7:anti162;	6			
chrXIV	778474	778625	151	**-1,602455578**	N14:778523	7,5	+3:YNR074C;	3			
chrXIV	779862	780020	158	**-1,419903906**	N14:779923	12,127	+1:YNR075W;	1			
chrXIV	781645	781793	148	**-1,105596445**	N14:781689	14,224	+1:YNR075C-A; -1:YNR076W;	1			
chrXIV	781935	782064	129	**-1,258754213**	N14:781999	13,856	+2:YNR076W;	2			
chrXIV	781942	782106	164	**-1,09897223**	N14:781999	13,856	+2:YNR076W;	2			
chrXIV	783376	783501	125	**-0,76204983**	N14:783457	20,599		Intergene			TEL RIGHT
chrXIV	784039	784210	171	**0,358247291**	N14:784097	20,984		Intergene			TEL RIGHT
chrXIV	784040	784199	159	**-1,835059105**	N14:784097	20,984		Intergene			TEL RIGHT
chrXIV	784044	784183	139	**-0,89633563**	N14:784097	20,984		Intergene			TEL RIGHT
chrXIV	784044	784199	155	**-1,636685829**	N14:784097	20,984		Intergene			TEL RIGHT
chrXIV	784049	784198	149	**-1,087259724**	N14:784097	20,984		Intergene			TEL RIGHT
chrXIV	784049	784203	154	**-1,098942288**	N14:784097	20,984		Intergene			TEL RIGHT
chrXIV	784063	784183	120	**-0,785229944**	N14:784097	20,984		Intergene			TEL RIGHT
chrXIV	784063	784195	132	**-0,672954036**	N14:784097	20,984		Intergene			TEL RIGHT
chrXIV	784063	784199	136	**-1,185656851**	N14:784097	20,984		Intergene			TEL RIGHT
chrXIV	784063	784202	139	**-0,821457678**	N14:784097	20,984		Intergene			TEL RIGHT
chrXIV	784063	784210	147	**-1,071686103**	N14:784097	20,984		Intergene			TEL RIGHT
chrXIV	784063	784233	170	**-1,486291294**	N14:784097	20,984		Intergene			TEL RIGHT
chrXIV	784063	784239	176	**-0,892286549**	N14:784097	20,984		Intergene			TEL RIGHT
chrXIV	784063	784239	176	**-0,892286549**	N14:784261	54,976		Intergene			TEL RIGHT
chrXIV	784063	784275	212	**-1,963608692**	N14:784097	20,984		Intergene			TEL RIGHT
chrXIV	784063	784275	212	**-1,963608692**	N14:784261	54,976		Intergene			TEL RIGHT
chrXIV	784064	784183	119	**-0,381367089**	N14:784097	20,984		Intergene			TEL RIGHT
chrXIV	784064	784199	135	**-0,918950191**	N14:784097	20,984		Intergene			TEL RIGHT
chrXIV	784101	784203	102	**-0,869865093**	N14:784097	20,984		Intergene			TEL RIGHT
chrXIV	784101	784210	109	**-0,001883451**	N14:784097	20,984		Intergene			TEL RIGHT
chrXIV	784106	784198	92	**-1,405753408**	N14:784097	20,984		Intergene			TEL RIGHT
chrXIV	784106	784203	97	**-0,934219363**	N14:784097	20,984		Intergene			TEL RIGHT
chrXIV	784106	784210	104	**-0,054330027**	N14:784097	20,984		Intergene			TEL RIGHT
chrXIV	784106	784238	132	**-0,546881471**	N14:784097	20,984		Intergene			TEL RIGHT
chrXIV	784106	784238	132	**-0,546881471**	N14:784261	54,976		Intergene			TEL RIGHT
chrXIV	784106	784265	159	**-0,941267683**	N14:784097	20,984		Intergene			TEL RIGHT
chrXIV	784106	784265	159	**-0,941267683**	N14:784261	54,976		Intergene			TEL RIGHT
chrXIV	784106	784274	168	**-0,382371744**	N14:784097	20,984		Intergene			TEL RIGHT
chrXIV	784106	784274	168	**-0,382371744**	N14:784261	54,976		Intergene			TEL RIGHT
chrXIV	784106	784275	169	**-1,389975984**	N14:784097	20,984		Intergene			TEL RIGHT
chrXIV	784106	784275	169	**-1,389975984**	N14:784261	54,976		Intergene			TEL RIGHT
chrXIV	784106	784277	171	**-0,82305504**	N14:784097	20,984		Intergene			TEL RIGHT
chrXIV	784106	784277	171	**-0,82305504**	N14:784261	54,976		Intergene			TEL RIGHT
chrXIV	784111	784233	122	**-0,0243827**	N14:784097	20,984		Intergene			TEL RIGHT
chrXIV	784111	784286	175	**-0,755639361**	N14:784097	20,984		Intergene			TEL RIGHT
chrXIV	784111	784286	175	**-0,755639361**	N14:784261	54,976		Intergene			TEL RIGHT
chrXIV	784130	784238	108	**-0,429047085**	N14:784261	54,976		Intergene			TEL RIGHT
chrXIV	784130	784274	144	**-0,611468943**	N14:784261	54,976		Intergene			TEL RIGHT
chrXIV	784130	784275	145	**-1,718014209**	N14:784261	54,976		Intergene			TEL RIGHT
chrXIV	784130	784277	147	**-0,767845307**	N14:784261	54,976		Intergene			TEL RIGHT
chrXIV	784130	784329	199	**-1,051778827**	N14:784261	54,976		Intergene			TEL RIGHT
chrXIV	784132	784275	143	**-0,670688925**	N14:784261	54,976		Intergene			TEL RIGHT
chrXIV	784135	784274	139	**-0,574347283**	N14:784261	54,976		Intergene			TEL RIGHT
chrXIV	784139	784275	136	**-1,18667047**	N14:784261	54,976		Intergene			TEL RIGHT
chrXIV	784139	784296	157	**-0,837353005**	N14:784261	54,976		Intergene			TEL RIGHT
chrXIV	784140	784275	135	**-1,818223736**	N14:784261	54,976		Intergene			TEL RIGHT
chrXIV	784145	784286	141	**-0,826669811**	N14:784261	54,976		Intergene			TEL RIGHT
chrXIV	784164	784275	111	**-0,803360385**	N14:784261	54,976		Intergene			TEL RIGHT
chrXIV	784169	784274	105	**-0,496957015**	N14:784261	54,976		Intergene			TEL RIGHT
chrXIV	784169	784275	106	**-1,452247251**	N14:784261	54,976		Intergene			TEL RIGHT
chrXIV	784169	784277	108	**-0,642793146**	N14:784261	54,976		Intergene			TEL RIGHT
chrXIV	784172	784265	93	**-0,569030767**	N14:784261	54,976		Intergene			TEL RIGHT
chrXIV	784172	784267	95	**-0,184600335**	N14:784261	54,976		Intergene			TEL RIGHT
chrXIV	784172	784271	99	**-0,344207086**	N14:784261	54,976		Intergene			TEL RIGHT
chrXIV	784172	784274	102	**-0,93474423**	N14:784261	54,976		Intergene			TEL RIGHT
chrXIV	784172	784275	103	**-0,77051074**	N14:784261	54,976		Intergene			TEL RIGHT

CHR	START	END	L(bp)	ΔLknuc	Nuc ID	Fuzziness	Gene ID	Gene body postion or intergene	Terminal	rDNA	Telomeric
chrXIV	784172	784277	105	**-0,205284516**	N14:784261	54,976		Intergene			TEL RIGHT
chrXIV	784177	784271	94	**-0,994111089**	N14:784261	54,976		Intergene			TEL RIGHT
chrXIV	784177	784275	98	**-1,51851675**	N14:784261	54,976		Intergene			TEL RIGHT
chrXV	838	992	154	**-1,380324046**	N15:915	15,086	+3*:YOL166W-A;	3	TERM		TEL LEFT
chrXV	6743	6894	151	**-1,174138836**	N15:6849	19,188	+7:YOL164W; +1:SUT761;	7			
chrXV	8892	9048	156	**-1,498450814**	N15:8985	11,411	+3*:SUT347;	3	TERM		
chrXV	10875	10982	107	**-1,04772108**	N15:10900	18,446	+6*:YOL162W;	6	TERM		
chrXV	23820	23972	152	**-1,178854411**	N15:23919	14,142	+3:YOL157C;	3			
chrXV	29238	29397	159	**-1,05223977**	N15:29410	42,658	+15:YOL155C; +7:anti164;	15			
chrXV	29238	29397	159	**-1,05223977**	N15:29253	42,426	+16:YOL155C; +6:anti164;	16			
chrXV	36163	36311	148	**-1,348033363**	N15:36242	12,069	+3:SUT352;	3			
chrXV	39246	39406	160	**-1,233483322**	N15:39349	23,908	+4:Unit583; +6:SUT764;	4			
chrXV	43767	43929	162	**-1,607068033**	N15:43844	3,869	+2:YOL151W;	2			
chrXV	44442	44591	149	**-1,022607825**	N15:44533	7,521		Intergene			
chrXV	49181	49338	157	**-1,106475741**	N15:49259	27,242	+3:YOL146W;	3			
chrXV	51344	51469	125	**-0,66890286**				Overlap <50 bp			
chrXV	53593	53699	106	**-0,993348981**				Overlap <50 bp			
chrXV	53593	53708	115	**-1,147864622**				Overlap <50 bp			
chrXV	53860	54011	151	**-1,503230881**	N15:53913	9,743	+6*:YOL144W;	6	TERM		
chrXV	53996	54153	157	**-1,235505603**	N15:54073	31,044		Intergene			
chrXV	63125	63255	130	**-0,620507582**	N15:63178	14,886	+15:YOL138C;	15			
chrXV	64118	64281	163	**-1,497302814**	N15:64218	28,312	+8:YOL138C;	8			
chrXV	66159	66329	170	**-1,841108517**	N15:66265	21,442	+5:YOL137W;	5			
chrXV	72755	72904	149	**-1,239389213**	N15:72830	16,477	0:YOL131W; +10*:YOL132W;	0			
chrXV	74613	74750	137	**-0,619505345**	N15:74669	4,309	+4:YOL130W;	4			
chrXV	74660	74806	146	**-1,025612625**	N15:74669	4,309	+4:YOL130W;	4			
chrXV	74660	74806	146	**-1,025612625**	N15:74825	13,401	+5:YOL130W;	5			
chrXV	75646	75807	161	**-1,636061107**	N15:75770	20,057	+11:YOL130W;	11			
chrXV	80807	80978	171	**-1,510279878**	N15:80842	39,144		Intergene			
chrXV	101110	101258	148	**-1,146373331**	N15:101210	10,443	0:YOL115W; +1:SUT767;	0			
chrXV	108670	108830	160	**-1,456753686**	N15:108728	3,189	+2:YOL111C;	2			
chrXV	110950	111091	141	**-1,041797174**	N15:111017	31,321		Intergene			
chrXV	112040	112197	157	**-1,663471504**	N15:112099	3,033	-1:YOL108C; +1:YOL107W;	-1			
chrXV	113588	113739	151	**-0,901969475**	N15:113650	9,368		Intergene			
chrXV	116654	116808	154	**-1,58598372**	N15:116777	43,911	+6:YOL104C;	6			
chrXV	118023	118177	154	**-1,25082971**	N15:118099	16,442		Intergene			
chrXV	118066	118215	149	**-1,105723446**	N15:118099	16,442		Intergene			
chrXV	118240	118363	123	**-0,916269981**	N15:118282	47,376		Intergene			
chrXV	118340	118495	155	**-1,055778785**	N15:118453	30,73		Intergene			
chrXV	118969	119120	151	**-1,414032477**	N15:119046	27,301		Intergene			
chrXV	118979	119124	145	**-1,232667116**	N15:119046	27,301		Intergene			
chrXV	118997	119148	151	**-1,174038842**	N15:119046	27,301		Intergene			
chrXV	119251	119405	154	**-1,174652853**	N15:119384	12,124		Intergene			
chrXV	119285	119429	144	**-1,152196264**	N15:119384	12,124		Intergene			
chrXV	119816	119951	135	**-0,962006086**	N15:119853	2,646		Intergene			
chrXV	120263	120409	146	**-1,081570721**	N15:120330	17,559		Intergene			
chrXV	120263	120413	150	**-1,00039977**	N15:120330	17,559		Intergene			
chrXV	120442	120599	157	**-1,259242691**	N15:120533	32,512		Intergene			
chrXV	120674	120823	149	**-1,165496865**	N15:120708	41,012		Intergene			
chrXV	120933	121062	129	**-1,018712158**				Overlap <50 bp			
chrXV	121845	121995	150	**-1,528664224**	N15:121935	24,007		Intergene			
chrXV	122824	122966	142	**-1,215230584**	N15:122896	37,581		Intergene			
chrXV	132108	132259	151	**-1,636024249**	N15:132177	18,73	+20:YOL100W;	20			
chrXV	132333	132482	149	**-1,37500934**	N15:132411	23,803	+21*:YOL100W;	21	TERM		
chrXV	138290	138437	147	**-1,170090393**	N15:138366	8,206	+3*:CUT358; +5:YOL096C;	3	TERM		
chrXV	141232	141379	147	**-1,152881469**	N15:141343	14,096	+1:YOL095C;	1			
chrXV	142140	142290	150	**-1,10932615**	N15:142219	4,05	+3:YOL094C;	3			
chrXV	143684	143828	144	**-1,13876834**	N15:143766	17,672	+7*:YOL093W;	7	TERM		
chrXV	147632	147784	152	**-0,948984272**	N15:147706	6,401	+3:YOL090W;	3			
chrXV	152975	153131	156	**-1,295261875**	N15:153029	5,132	+4:YOL089C;	4			
chrXV	160615	160744	129	**-0,727958831**	N15:160677	39,887	0:YOL086C;	0			
chrXV	162501	162638	137	**-0,739962871**	N15:162550	6,14	+2:YOL084W;	2			
chrXV	163008	163166	158	**-1,157474051**	N15:163097	9,288	+5:YOL084W;	5			
chrXV	163425	163564	139	**-0,944546075**	N15:163509	7,967	+7:YOL084W;	7			
chrXV	164146	164288	142	**-1,06184728**				Overlap <50 bp			
chrXV	169600	169748	148	**-1,369866628**	N15:169700	15,65	+8:YOL082W;	8			
chrXV	171569	171743	174	**-1,447520155**	N15:171764	24,647	+7:YOL081W;	7			
chrXV	173781	173933	152	**-1,243122431**				Overlap <50 bp			
chrXV	177092	177238	146	**-2,136246911**	N15:177172	13,52	+41:YOL081W;	41			
chrXV	177093	177211	118	**-0,833644789**	N15:177172	13,52	+41:YOL081W;	41			
chrXV	177093	177214	121	**-0,819245043**	N15:177172	13,52	+41:YOL081W;	41			
chrXV	177093	177217	124	**-1,701848134**	N15:177172	13,52	+41:YOL081W;	41			
chrXV	177093	177220	127	**-0,387802622**	N15:177172	13,52	+41:YOL081W;	41			
chrXV	177093	177221	128	**-0,746455658**	N15:177172	13,52	+41:YOL081W;	41			
chrXV	177093	177222	129	**-0,6457415**	N15:177172	13,52	+41:YOL081W;	41			
chrXV	177093	177223	130	**-0,541260145**	N15:177172	13,52	+41:YOL081W;	41			
chrXV	177093	177224	131	**-0,867184981**	N15:177172	13,52	+41:YOL081W;	41			
chrXV	177093	177225	132	**-0,749488162**	N15:177172	13,52	+41:YOL081W;	41			
chrXV	177093	177226	133	**-1,430856435**	N15:177172	13,52	+41:YOL081W;	41			
chrXV	177093	177227	134	**-0,712061023**	N15:177172	13,52	+41:YOL081W;	41			
chrXV	177093	177228	135	**-0,743122709**	N15:177172	13,52	+41:YOL081W;	41			
chrXV	177093	177229	136	**-0,958480747**	N15:177172	13,52	+41:YOL081W;	41			
chrXV	177093	177230	137	**-1,158216722**	N15:177172	13,52	+41:YOL081W;	41			
chrXV	177093	177231	138	**-1,100262657**	N15:177172	13,52	+41:YOL081W;	41			
chrXV	177093	177232	139	**-1,028745634**	N15:177172	13,52	+41:YOL081W;	41			
chrXV	177093	177233	140	**-1,208694273**	N15:177172	13,52	+41:YOL081W;	41			
chrXV	177093	177234	141	**-1,107974531**	N15:177172	13,52	+41:YOL081W;	41			
chrXV	177093	177235	142	**-0,895202913**	N15:177172	13,52	+41:YOL081W;	41			
chrXV	177093	177236	143	**-0,967803873**	N15:177172	13,52	+41:YOL081W;	41			
chrXV	177093	177237	144	**-1,243383602**	N15:177172	13,52	+41:YOL081W;	41			
chrXV	177093	177238	145	**-0,830850786**	N15:177172	13,52	+41:YOL081W;	41			
chrXV	177093	177239	146	**-2,235173337**	N15:177172	13,52	+41:YOL081W;	41			
chrXV	177094	177238	144	**-1,330164434**	N15:177172	13,52	+41:YOL081W;	41			
chrXV	177095	177238	143	**-1,12270541**	N15:177172	13,52	+41:YOL081W;	41			
chrXV	177096	177238	142	**-0,96152176**	N15:177172	13,52	+41:YOL081W;	41			
chrXV	177097	177238	141	**-0,940392672**	N15:177172	13,52	+41:YOL081W;	41			

CHR	START	END	L(bp)	ΔLknuc	Nuc ID	Fuzziness	Gene ID	Gene body postion or intergene	Terminal	rDNA	Telomeric
chrXV	177098	177238	140	**-0,967503401**	N15:177172	13,52	+41:YOL081W;	41			
chrXV	177099	177238	139	**-1,64243343**	N15:177172	13,52	+41:YOL081W;	41			
chrXV	177100	177238	138	**-1,193639396**	N15:177172	13,52	+41:YOL081W;	41			
chrXV	177101	177238	137	**-1,554361252**	N15:177172	13,52	+41:YOL081W;	41			
chrXV	177102	177238	136	**-1,493321503**	N15:177172	13,52	+41:YOL081W;	41			
chrXV	177103	177238	135	**-1,487576382**	N15:177172	13,52	+41:YOL081W;	41			
chrXV	177104	177238	134	**-1,693158055**	N15:177172	13,52	+41:YOL081W;	41			
chrXV	177105	177238	133	**-1,841043842**	N15:177172	13,52	+41:YOL081W;	41			
chrXV	177106	177238	132	**-0,987968092**	N15:177172	13,52	+41:YOL081W;	41			
chrXV	177107	177238	131	**-1,473584125**	N15:177172	13,52	+41:YOL081W;	41			
chrXV	177109	177238	129	**-1,40063726**	N15:177172	13,52	+41:YOL081W;	41			
chrXV	177110	177238	128	**-1,286237269**	N15:177172	13,52	+41:YOL081W;	41			
chrXV	177111	177238	127	**-1,046430492**	N15:177172	13,52	+41:YOL081W;	41			
chrXV	177112	177238	126	**-1,30549581**	N15:177172	13,52	+41:YOL081W;	41			
chrXV	177113	177238	125	**-0,347512869**	N15:177172	13,52	+41:YOL081W;	41			
chrXV	177115	177238	123	**-1,0655025**	N15:177172	13,52	+41:YOL081W;	41			
chrXV	177117	177238	121	**-0,722508558**	N15:177172	13,52	+41:YOL081W;	41			
chrXV	177118	177238	120	**-0,130969237**	N15:177172	13,52	+41:YOL081W;	41			
chrXV	177119	177238	119	**-0,392333371**	N15:177172	13,52	+41:YOL081W;	41			
chrXV	177120	177238	118	**-0,543069241**	N15:177172	13,52	+41:YOL081W;	41			
chrXV	177121	177238	117	**-0,66175371**	N15:177172	13,52	+41:YOL081W;	41			
chrXV	177124	177238	114	**-0,466034711**	N15:177172	13,52	+41:YOL081W;	41			
chrXV	177125	177238	113	**-0,720236509**	N15:177172	13,52	+41:YOL081W;	41			
chrXV	177128	177238	110	**-0,748359702**	N15:177172	13,52	+41:YOL081W;	41			
chrXV	177130	177238	108	**-0,44786109**	N15:177172	13,52	+41:YOL081W;	41			
chrXV	177132	177217	85	**-0,395537676**	N15:177172	13,52	+41:YOL081W;	41			
chrXV	177132	177238	106	**-1,369321852**	N15:177172	13,52	+41:YOL081W;	41			
chrXV	177733	177893	160	**-1,278779296**	N15:177858	30,517	+45:YOL081W;	45			
chrXV	178523	178704	181	**-1,6022499**	N15:178709	11,015	+51:YOL081W;	51			
chrXV	185749	185889	140	**-1,236430251**	N15:185774	30,288	+3*:YOL077W-A; +7*:YOL077C;	3	TERM		
chrXV	187300	187456	156	**-1,387095032**	N15:187379	9,813	+3:YOL076W;	3			
chrXV	189704	189862	158	**-1,434006571**	N15:189795	17,838	+23:YOL075C;	23			
chrXV	192332	192491	159	**-1,20503896**	N15:192321	30,993	+9:YOL075C;	9			
chrXV	196562	196707	145	**-1,287089093**	N15:196546	19,26	+10*:YOL072W; +1:YOL071W;	10	TERM		
chrXV	196562	196707	145	**-1,287089093**	N15:196728	15,172	+2:YOL071W;	2			
chrXV	198924	199072	148	**-1,003230809**	N15:198961	11,219	-1:YOL070C; +1:YOL069W;	-1			
chrXV	202849	203002	153	**-1,412861553**	N15:202912	19,627	+10:YOL066C; +18:YOL065C;	10			
chrXV	205781	205911	130	**-0,97370178**	N15:205866	13,943	+1:YOL065C;	1			
chrXV	208753	208900	147	**-1,037327426**	N15:208837	10,61	+10:YOL063C;	10			
chrXV	209390	209535	145	**-1,221244758**	N15:209458	29,068	+7:YOL063C;	7			
chrXV	212639	212808	169	**-1,510529614**	N15:212754	23,37	+4:YOL061W;	4			
chrXV	214778	214916	138	**-0,724387294**	N15:214872	17,056	+9:YOL060C;	9			
chrXV	215741	215894	153	**-1,353123569**	N15:215837	20,917	+3:YOL060C;	3			
chrXV	218336	218507	171	**-1,405995612**	N15:218350	26,499	+9:YOL059W;	9			
chrXV	220428	220557	129	**-1,125106884**	N15:220513	10,61	OL058W; -1:YOL057W; +1:anti167;	8	TERM		
chrXV	222406	222534	128	**-1,187436268**	N15:222459	11,161	+11:YOL057W;	11			
chrXV	225244	225389	145	**-0,987555625**	N15:225300	6,419		Intergene			
chrXV	227790	227919	129	**-1,270952598**	N15:227846	36,592		Intergene			
chrXV	229356	229535	179	**-1,462983703**	N15:229431	34,896	+6:YOL054W;	6			
chrXV	229736	229903	167	**-1,685697167**	N15:229797	15,293	+8*:YOL054W;	8	TERM		
chrXV	233701	233860	159	**-1,164451308**	N15:233870	7,778	-1:YOL052C;	-1			
chrXV	233701	233860	159	**-1,164451308**	N15:233712	24,612	0:YOL052C;	0			
chrXV	237293	237436	143	**-1,292865768**	N15:237339	5,802	+16:YOL051W;	16			
chrXV	242082	242240	158	**-1,390671127**	N15:242059	10,747	+3:SUT357; +5:YOL047C;	3			
chrXV	242082	242240	158	**-1,390671127**	N15:242224	12,971	+4:SUT357; +4:YOL047C;	4			
chrXV	243486	243636	150	**-1,01193345**	N15:243571	6,285	+4:YOL045W;	4			
chrXV	244606	244758	152	**-1,535663473**	N15:244626	13,846	+11:YOL045W;	11			
chrXV	244606	244758	152	**-1,535663473**	N15:244776	39,569	+12:YOL045W;	12			
chrXV	248190	248334	144	**-1,505492756**	N15:248279	17,462	+8*:YOL044W;	8	TERM		
chrXV	248556	248722	166	**-1,475566659**				Overlap <50 bp			
chrXV	255239	255421	182	**-1,20720421**	N15:255344	8,618	-1:YOL038C-A; +1:YOL038W;	-1			
chrXV	256726	256872	146	**-1,150587758**	N15:256740	13,546	+3:YOL036W;	3			
chrXV	260668	260810	142	**-1,474576099**				Overlap <50 bp			
chrXV	261423	261595	172	**-1,435109284**	N15:261549	31,264	+11:YOL034W;	11			
chrXV	263580	263731	151	**-0,820452217**	N15:263648	8,497	0:anti-YOL033W; +2:YOL033W;	0			
chrXV	265679	265840	161	**-1,338903182**	N15:265718	8,727	+3:YOL032W;	3			
chrXV	266859	267005	146	**-1,470278943**	N15:266903	10,559	+5:YOL031C;	5			
chrXV	268238	268379	141	**-1,0559596**	N15:268361	4,98	+2:YOL030W;	2			
chrXV	270968	271134	166	**-1,160322532**	N15:271050	9,948	+4:YOL028C;	4			
chrXV	271150	271308	158	**-1,349304512**	N15:271220	6,723	+3:YOL028C;	3			
chrXV	273050	273212	162	**-1,55051261**	N15:273212	30,589	+4:YOL027C;	4			
chrXV	276074	276211	137	**-0,604744689**	N15:276066	24,854	+8:YOL025W;	8			
chrXV	276074	276211	137	**-0,604744689**	N15:276231	22,633	+9:YOL025W;	9			
chrXV	276839	276983	144	**-1,419903964**	N15:276892	10,614	+13*:YOL025W; 0:YOL024W;	13	TERM		
chrXV	279591	279744	153	**-1,3874379**	N15:279667	22,007	+11:YOL023W;	11			
chrXV	280089	280217	128	**-0,789209621**				Overlap <50 bp			
chrXV	282501	282650	149	**-1,016160775**	N15:282573	19,856	+19:YOL021C;	19			
chrXV	284484	284582	98	**-1,242739685**				Overlap <50 bp			
chrXV	284650	284789	139	**-0,886129633**	N15:284768	27,722	+5:YOL021C;	5			
chrXV	284999	285154	155	**-1,06973713**	N15:285080	11,548	+3:YOL021C;	3			
chrXV	285195	285326	131	**-0,979364305**	N15:285245	12,071	+2:YOL021C;	2			
chrXV	292850	293005	155	**-1,199196727**	N15:292974	3,651	+4:YOL017W;	4			
chrXV	292905	293050	145	**-1,063680291**	N15:292974	3,651	+4:YOL017W;	4			
chrXV	293213	293372	159	**-1,302444407**	N15:293294	6,907	+6:YOL017W;	6			
chrXV	294193	294362	169	**-1,241354743**	N15:294360	12,576	+12:YOL017W;	12			
chrXV	298993	299142	149	**-1,194772115**	N15:299031	18,702	+13*:YOL015W; -1:Unit592;	13	TERM		
chrXV	306450	306602	152	**-1,33299903**	N15:306552	11,59	+8:YOL011W;	8			
chrXV	311787	311943	156	**-1,390667975**	N15:311928	17,315	+4:YOL007C;	4			
chrXV	319552	319692	140	**-0,8414846**	N15:319612	19,675	+19:YOL004W;	19			
chrXV	320812	320963	151	**-1,066604116**	N15:320792	45,768	+26:YOL004W;	26			
chrXV	321565	321713	148	**-1,227212805**	N15:321561	10,149	+30*:YOL004W;	30	TERM		
chrXV	327427	327560	133	**-0,914160502**	N15:327498	11,537	+5:YOR001W;	5			
chrXV	328438	328583	145	**-0,972513716**	N15:328423	33,596	+10:YOR001W;	10			
chrXV	328438	328583	145	**-0,972513716**	N15:328597	24,648	+11:YOR001W;	11			
chrXV	329812	329980	168	**-1,205104124**	N15:329910	8,424	+4:YOR002W;	4			
chrXV	338001	338161	160	**-1,351213351**	N15:338135	10,053	+4:YOR006C; +4:SUT360;	4			

CHR	START	END	L(bp)	ΔLknuc	Nuc ID	Fuzziness	Gene ID	Gene body postion or intergene	Terminal	rDNA	Telomeric
chrXV	341700	341803	103	**-1,271077672**	N15:341798	15,84	+6:YOR008C;	6			
chrXV	348865	348972	107	**-0,040875625**	N15:348916	16,293	+2*:SUT361;	2	TERM		
chrXV	349258	349411	153	**-1,28573534**	N15:349279	15,864	+4:SUT770;	4			
chrXV	351942	352092	150	**-1,02860907**	N15:351997	40,035	+13:YOR011W;	13			
chrXV	353769	353922	153	**-1,610468578**	N15:353843	3,536	+24*:YOR011W;	24	TERM		
chrXV	356685	356815	130	**-0,859322139**	N15:356719	8,585	+2:YOR012W; +4:SUT772;	2			
chrXV	357881	358029	148	**-1,22758277**	N15:357967	9,209	+4:YOR014W;	4			
chrXV	359149	359277	128	**-1,786539428**				Overlap <50 bp			
chrXV	359963	360110	147	**-1,652893437**	N15:360012	28,378	+17*:YOR014W;	17	TERM		
chrXV	363241	363404	163	**-1,340148341**	N15:363313	23,231	+13:YOR017W;	13			
chrXV	363647	363809	162	**-1,349138369**	N15:363630	9,503	+4*:CUT834; +15:YOR017W;	4	TERM		
chrXV	365392	365542	150	**-1,238908226**	N15:365381	33,966	+7:YOR018W;	7			
chrXV	365392	365542	150	**-1,238908226**	N15:365558	50,406	+8:YOR018W;	8			
chrXV	369266	369425	159	**-1,156762629**	N15:369250	26,615	+7:YOR019W; +2:anti170;	7			
chrXV	369347	369504	157	**-1,672975616**	N15:369518	12,775	+9:YOR019W; 0:anti170;	9			
chrXV	371267	371398	131	**-0,68462629**	N15:371332	16,558	+1:SUT774; -1:YOR020W-A;	1			
chrXV	371602	371749	147	**-0,856399705**	N15:371676	16,485	+5*:SUT775; +1:YOR020W-A;	5	TERM		
chrXV	377796	377936	140	**-0,943111977**	N15:377801	3,775	+1:YOR023C;	1			
chrXV	384219	384368	149	**-1,244762911**	N15:384312	13,906	+3:YOR028C;	3			
chrXV	387959	388110	151	**-1,653310674**	N15:388058	9,95	+9:YOR030W;	9			
chrXV	389816	389942	126	**-0,817478825**	N15:389861	7,234	+8:YOR032C;	8			
chrXV	392262	392373	111	**-1,015640209**	N15:392325	26,655	033C; +2:YOR032W-A; -1:CUT371;	14	TERM		
chrXV	392576	392737	161	**-1,493504576**	N15:392661	28,648	R033C; +29:YOR034C; +1:CUT371;	12			
chrXV	401254	401398	144	**-1,416581892**	N15:401349	6,348	-1:YOR037W;	-1			
chrXV	403569	403709	140	**-0,791369069**				Overlap <50 bp			
chrXV	405315	405460	145	**-1,026884964**	N15:405385	4,412	-1:YOR039W; +1:YOR038C;	-1			
chrXV	405735	405887	152	**-1,294871055**	N15:405829	6,033	+2:YOR039W;	2			
chrXV	408309	408462	153	**-1,212757566**	N15:408405	5,565	+1:YOR042W;	1			
chrXV	413175	413325	150	**-1,355282683**	N15:413244	9,576	+4:YOR044W;	4			
chrXV	413525	413650	125	**-0,730040502**	N15:413578	9,136	+6*:YOR044W; -1:YOR045W;	6	TERM		
chrXV	419166	419317	151	**-1,233662172**	N15:419244	21,394	+16:YOR048C;	16			
chrXV	420271	420429	158	**-1,306162798**	N15:420382	6,397	+9:YOR048C;	9			
chrXV	423240	423391	151	**-1,060709461**	N15:423319	4,278	+6:anti172; +5:YOR049C;	6			
chrXV	423241	423391	150	**-1,797723725**	N15:423319	4,278	+6:anti172; +5:YOR049C;	6			
chrXV	423242	423391	149	**-1,795105226**	N15:423319	4,278	+6:anti172; +5:YOR049C;	6			
chrXV	423243	423391	148	**-1,31934863**	N15:423319	4,278	+6:anti172; +5:YOR049C;	6			
chrXV	423245	423391	146	**-1,210460058**	N15:423319	4,278	+6:anti172; +5:YOR049C;	6			
chrXV	433044	433202	158	**-1,262142571**	N15:433178	22,196	+7:YOR057W;	7			
chrXV	433171	433320	149	**-1,163300833**	N15:433331	22,264	+8*:YOR057W;	8	TERM		
chrXV	433171	433320	149	**-1,163300833**	N15:433178	22,196	+7:YOR057W;	7			
chrXV	435682	435849	167	**-1,254519131**	N15:435774	9,543	+5:YOR058C;	5			
chrXV	441363	441514	151	**-1,256060734**	N15:441436	4,494	+1:YOR061W; -1:YOR060C;	1			
chrXV	443421	443578	157	**-1,358522809**	N15:443539	18,119	+2:YOR062C;	2			
chrXV	445187	445337	150	**-1,121339985**	N15:445171	30,447	+3:YOR063W;	3			
chrXV	445187	445337	150	**-1,121339985**	N15:445329	15,953	+4:YOR063W;	4			
chrXV	446874	447012	138	**-0,698220013**	N15:446940	5	-1:YOR064C; -1:YOR065W;	-1			
chrXV	450011	450165	154	**-1,312321467**	N15:450081	16,563	+5:YOR066W;	5			
chrXV	456168	456329	161	**-1,04686065**	N15:456318	17,474	+11:YOR070C;	11			
chrXV	457433	457588	155	**-1,156615902**	N15:457449	2,708	+4:YOR070C;	4			
chrXV	457539	457691	152	**-1,246098983**	N15:457612	2,317	+3:YOR070C;	3			
chrXV	459473	459604	131	**-1,362129338**				Overlap <50 bp			
chrXV	465612	465776	164	**-1,46017332**	N15:465716	10,577	+7:YOR073W;	7			
chrXV	469152	469262	110	**-0,535648732**	N15:469219	14,637	+7*:YOR075W;	7	TERM		
chrXV	469152	469277	125	**-0,835524045**	N15:469219	14,637	+7*:YOR075W;	7	TERM		
chrXV	480195	480360	165	**-1,356189554**	N15:480344	20,72	-1:YOR084W; +6:YOR083W;	-1			
chrXV	480209	480363	154	**-1,132651125**	N15:480344	20,72	-1:YOR084W; +6:YOR083W;	-1			
chrXV	480628	480755	127	**-2,047102363**	N15:480718	26,92	+2:YOR084W; +8:YOR083W;	2			
chrXV	480628	480787	159	**-1,408184776**	N15:480718	26,92	+2:YOR084W; +8:YOR083W;	2			
chrXV	480629	480736	107	**-1,641834775**	N15:480718	26,92	+2:YOR084W; +8:YOR083W;	2			
chrXV	480629	480737	108	**-1,652642803**	N15:480718	26,92	+2:YOR084W; +8:YOR083W;	2			
chrXV	480629	480738	109	**-1,273939347**	N15:480718	26,92	+2:YOR084W; +8:YOR083W;	2			
chrXV	480629	480739	110	**-1,215774507**	N15:480718	26,92	+2:YOR084W; +8:YOR083W;	2			
chrXV	480629	480741	112	**-1,787101329**	N15:480718	26,92	+2:YOR084W; +8:YOR083W;	2			
chrXV	480629	480742	113	**-2,033901057**	N15:480718	26,92	+2:YOR084W; +8:YOR083W;	2			
chrXV	480629	480743	114	**-1,702846269**	N15:480718	26,92	+2:YOR084W; +8:YOR083W;	2			
chrXV	480629	480744	115	**-1,764861837**	N15:480718	26,92	+2:YOR084W; +8:YOR083W;	2			
chrXV	480629	480745	116	**-1,058947585**	N15:480718	26,92	+2:YOR084W; +8:YOR083W;	2			
chrXV	480629	480746	117	**-1,241355473**	N15:480718	26,92	+2:YOR084W; +8:YOR083W;	2			
chrXV	480629	480747	118	**-1,297879372**	N15:480718	26,92	+2:YOR084W; +8:YOR083W;	2			
chrXV	480629	480748	119	**-1,714200179**	N15:480718	26,92	+2:YOR084W; +8:YOR083W;	2			
chrXV	480629	480749	120	**-1,347145544**	N15:480718	26,92	+2:YOR084W; +8:YOR083W;	2			
chrXV	480629	480750	121	**-1,726655263**	N15:480718	26,92	+2:YOR084W; +8:YOR083W;	2			
chrXV	480629	480751	122	**-1,055735607**	N15:480718	26,92	+2:YOR084W; +8:YOR083W;	2			
chrXV	480629	480752	123	**-1,364898698**	N15:480718	26,92	+2:YOR084W; +8:YOR083W;	2			
chrXV	480629	480753	124	**-0,781653105**	N15:480718	26,92	+2:YOR084W; +8:YOR083W;	2			
chrXV	480629	480754	125	**-1,219940328**	N15:480718	26,92	+2:YOR084W; +8:YOR083W;	2			
chrXV	480629	480755	126	**-2,331802401**	N15:480718	26,92	+2:YOR084W; +8:YOR083W;	2			
chrXV	480629	480756	127	**-1,64753947**	N15:480718	26,92	+2:YOR084W; +8:YOR083W;	2			
chrXV	480629	480757	128	**-0,992370653**	N15:480718	26,92	+2:YOR084W; +8:YOR083W;	2			
chrXV	480629	480758	129	**-1,303404181**	N15:480718	26,92	+2:YOR084W; +8:YOR083W;	2			
chrXV	480629	480759	130	**-1,526067322**	N15:480718	26,92	+2:YOR084W; +8:YOR083W;	2			
chrXV	480629	480760	131	**-1,725088555**	N15:480718	26,92	+2:YOR084W; +8:YOR083W;	2			
chrXV	480629	480761	132	**-1,260374692**	N15:480718	26,92	+2:YOR084W; +8:YOR083W;	2			
chrXV	480629	480762	133	**-1,777442909**	N15:480718	26,92	+2:YOR084W; +8:YOR083W;	2			
chrXV	480629	480763	134	**-1,892204097**	N15:480718	26,92	+2:YOR084W; +8:YOR083W;	2			
chrXV	480629	480764	135	**-1,938855951**	N15:480718	26,92	+2:YOR084W; +8:YOR083W;	2			
chrXV	480629	480765	136	**-2,007405819**	N15:480718	26,92	+2:YOR084W; +8:YOR083W;	2			
chrXV	480629	480766	137	**-2,034103766**	N15:480718	26,92	+2:YOR084W; +8:YOR083W;	2			
chrXV	480629	480767	138	**-1,433521314**	N15:480718	26,92	+2:YOR084W; +8:YOR083W;	2			
chrXV	480629	480768	139	**-1,954292923**	N15:480718	26,92	+2:YOR084W; +8:YOR083W;	2			
chrXV	480629	480769	140	**-1,907043766**	N15:480718	26,92	+2:YOR084W; +8:YOR083W;	2			
chrXV	480629	480770	141	**-1,728509808**	N15:480718	26,92	+2:YOR084W; +8:YOR083W;	2			
chrXV	480629	480771	142	**-2,396375054**	N15:480718	26,92	+2:YOR084W; +8:YOR083W;	2			
chrXV	480629	480772	143	**-2,003456916**	N15:480718	26,92	+2:YOR084W; +8:YOR083W;	2			
chrXV	480629	480773	144	**-2,013460411**	N15:480718	26,92	+2:YOR084W; +8:YOR083W;	2			
chrXV	480629	480774	145	**-1,644022048**	N15:480718	26,92	+2:YOR084W; +8:YOR083W;	2			

CHR	START	END	L(bp)	ΔLknuc	Nuc ID	Fuzziness	Gene ID	Gene body postion or intergene	Terminal	rDNA	Telomeric
chrXV	480629	480775	146	**-2,671942306**	N15:480718	26,92	+2:YOR084W; +8:YOR083W;	2			
chrXV	480629	480776	147	**-1,227590535**	N15:480718	26,92	+2:YOR084W; +8:YOR083W;	2			
chrXV	480629	480777	148	**-1,726506313**	N15:480718	26,92	+2:YOR084W; +8:YOR083W;	2			
chrXV	480629	480778	149	**-1,776913413**	N15:480718	26,92	+2:YOR084W; +8:YOR083W;	2			
chrXV	480629	480779	150	**-1,332352265**	N15:480718	26,92	+2:YOR084W; +8:YOR083W;	2			
chrXV	480629	480780	151	**-1,60455997**	N15:480718	26,92	+2:YOR084W; +8:YOR083W;	2			
chrXV	480629	480781	152	**-1,971562384**	N15:480718	26,92	+2:YOR084W; +8:YOR083W;	2			
chrXV	480629	480782	153	**-1,631022204**	N15:480718	26,92	+2:YOR084W; +8:YOR083W;	2			
chrXV	480629	480783	154	**-1,537434115**	N15:480718	26,92	+2:YOR084W; +8:YOR083W;	2			
chrXV	480629	480784	155	**-1,470269874**	N15:480718	26,92	+2:YOR084W; +8:YOR083W;	2			
chrXV	480629	480785	156	**-1,739375556**	N15:480718	26,92	+2:YOR084W; +8:YOR083W;	2			
chrXV	480629	480786	157	**-1,58598926**	N15:480718	26,92	+2:YOR084W; +8:YOR083W;	2			
chrXV	480629	480787	158	**-1,005569653**	N15:480718	26,92	+2:YOR084W; +8:YOR083W;	2			
chrXV	480629	480788	159	**-2,779622115**	N15:480718	26,92	+2:YOR084W; +8:YOR083W;	2			
chrXV	480629	480789	160	**-1,553781909**	N15:480718	26,92	+2:YOR084W; +8:YOR083W;	2			
chrXV	480630	480787	157	**-1,421461779**	N15:480718	26,92	+2:YOR084W; +8:YOR083W;	2			
chrXV	480631	480787	156	**-1,240317616**	N15:480718	26,92	+2:YOR084W; +8:YOR083W;	2			
chrXV	480632	480787	155	**-1,626346486**	N15:480718	26,92	+2:YOR084W; +8:YOR083W;	2			
chrXV	480633	480787	154	**-1,446429767**	N15:480718	26,92	+2:YOR084W; +8:YOR083W;	2			
chrXV	480634	480787	153	**-1,194073162**	N15:480718	26,92	+2:YOR084W; +8:YOR083W;	2			
chrXV	480635	480787	152	**-1,898436973**	N15:480718	26,92	+2:YOR084W; +8:YOR083W;	2			
chrXV	480636	480787	151	**-1,739648531**	N15:480718	26,92	+2:YOR084W; +8:YOR083W;	2			
chrXV	480637	480787	150	**-1,572703578**	N15:480718	26,92	+2:YOR084W; +8:YOR083W;	2			
chrXV	480638	480787	149	**-1,662297273**	N15:480718	26,92	+2:YOR084W; +8:YOR083W;	2			
chrXV	480639	480787	148	**-1,340924261**	N15:480718	26,92	+2:YOR084W; +8:YOR083W;	2			
chrXV	480640	480787	147	**-1,73681727**	N15:480718	26,92	+2:YOR084W; +8:YOR083W;	2			
chrXV	480641	480787	146	**-0,94493**	N15:480718	26,92	+2:YOR084W; +8:YOR083W;	2			
chrXV	480642	480787	145	**-1,679102069**	N15:480718	26,92	+2:YOR084W; +8:YOR083W;	2			
chrXV	480643	480787	144	**-1,659589497**	N15:480718	26,92	+2:YOR084W; +8:YOR083W;	2			
chrXV	480644	480787	143	**-1,766861144**	N15:480718	26,92	+2:YOR084W; +8:YOR083W;	2			
chrXV	480645	480787	142	**-1,470075214**	N15:480718	26,92	+2:YOR084W; +8:YOR083W;	2			
chrXV	480646	480787	141	**-1,815668024**	N15:480718	26,92	+2:YOR084W; +8:YOR083W;	2			
chrXV	480647	480787	140	**-1,428032133**	N15:480718	26,92	+2:YOR084W; +8:YOR083W;	2			
chrXV	480648	480787	139	**-1,871642097**	N15:480718	26,92	+2:YOR084W; +8:YOR083W;	2			
chrXV	480649	480787	138	**-1,445458207**	N15:480718	26,92	+2:YOR084W; +8:YOR083W;	2			
chrXV	480650	480787	137	**-1,632534947**	N15:480718	26,92	+2:YOR084W; +8:YOR083W;	2			
chrXV	480651	480787	136	**-2,308724396**	N15:480718	26,92	+2:YOR084W; +8:YOR083W;	2			
chrXV	480652	480787	135	**-0,697838522**	N15:480718	26,92	+2:YOR084W; +8:YOR083W;	2			
chrXV	480653	480787	134	**-1,491723761**	N15:480718	26,92	+2:YOR084W; +8:YOR083W;	2			
chrXV	480654	480787	133	**-2,229488752**	N15:480718	26,92	+2:YOR084W; +8:YOR083W;	2			
chrXV	480655	480787	132	**-1,991583892**	N15:480718	26,92	+2:YOR084W; +8:YOR083W;	2			
chrXV	480656	480787	131	**-1,40811072**	N15:480718	26,92	+2:YOR084W; +8:YOR083W;	2			
chrXV	480657	480787	130	**-1,733972476**	N15:480718	26,92	+2:YOR084W; +8:YOR083W;	2			
chrXV	480658	480787	129	**-2,087844739**	N15:480718	26,92	+2:YOR084W; +8:YOR083W;	2			
chrXV	480659	480787	128	**-1,448509558**	N15:480718	26,92	+2:YOR084W; +8:YOR083W;	2			
chrXV	480660	480787	127	**-1,793477312**	N15:480718	26,92	+2:YOR084W; +8:YOR083W;	2			
chrXV	480661	480787	126	**-1,71378471**	N15:480718	26,92	+2:YOR084W; +8:YOR083W;	2			
chrXV	480662	480787	125	**-1,346997903**	N15:480718	26,92	+2:YOR084W; +8:YOR083W;	2			
chrXV	480663	480787	124	**-0,860282319**	N15:480718	26,92	+2:YOR084W; +8:YOR083W;	2			
chrXV	480664	480787	123	**-1,157819208**	N15:480718	26,92	+2:YOR084W; +8:YOR083W;	2			
chrXV	480665	480787	122	**-1,694605681**	N15:480718	26,92	+2:YOR084W; +8:YOR083W;	2			
chrXV	480666	480787	121	**-1,61084054**	N15:480718	26,92	+2:YOR084W; +8:YOR083W;	2			
chrXV	480667	480787	120	**-1,405153232**	N15:480718	26,92	+2:YOR084W; +8:YOR083W;	2			
chrXV	480668	480787	119	**-1,089638303**	N15:480718	26,92	+2:YOR084W; +8:YOR083W;	2			
chrXV	480669	480787	118	**-1,272321239**	N15:480718	26,92	+2:YOR084W; +8:YOR083W;	2			
chrXV	480670	480787	117	**-1,595672565**	N15:480718	26,92	+2:YOR084W; +8:YOR083W;	2			
chrXV	480671	480787	116	**-0,95120699**	N15:480718	26,92	+2:YOR084W; +8:YOR083W;	2			
chrXV	480672	480787	115	**-0,254454624**	N15:480718	26,92	+2:YOR084W; +8:YOR083W;	2			
chrXV	480673	480787	114	**-1,153879603**	N15:480718	26,92	+2:YOR084W; +8:YOR083W;	2			
chrXV	480674	480787	113	**-1,342981955**	N15:480718	26,92	+2:YOR084W; +8:YOR083W;	2			
chrXV	480675	480787	112	**-1,17343774**	N15:480718	26,92	+2:YOR084W; +8:YOR083W;	2			
chrXV	480676	480787	111	**-0,980228119**	N15:480718	26,92	+2:YOR084W; +8:YOR083W;	2			
chrXV	480677	480787	110	**-0,293003387**	N15:480718	26,92	+2:YOR084W; +8:YOR083W;	2			
chrXV	480678	480787	109	**-1,301535234**	N15:480718	26,92	+2:YOR084W; +8:YOR083W;	2			
chrXV	480679	480787	108	**-0,87559958**	N15:480718	26,92	+2:YOR084W; +8:YOR083W;	2			
chrXV	480680	480755	75	**-0,777**	N15:480718	26,92	+2:YOR084W; +8:YOR083W;	2			
chrXV	480680	480787	107	**-1,6540568**	N15:480718	26,92	+2:YOR084W; +8:YOR083W;	2			
chrXV	480681	480787	106	**-1,028788184**	N15:480718	26,92	+2:YOR084W; +8:YOR083W;	2			
chrXV	480682	480787	105	**-1,063120693**	N15:480718	26,92	+2:YOR084W; +8:YOR083W;	2			
chrXV	480683	480787	104	**-1,02210638**	N15:480718	26,92	+2:YOR084W; +8:YOR083W;	2			
chrXV	480684	480787	103	**-1,088385469**	N15:480718	26,92	+2:YOR084W; +8:YOR083W;	2			
chrXV	480685	480787	102	**-1,31204778**	N15:480718	26,92	+2:YOR084W; +8:YOR083W;	2			
chrXV	480686	480787	101	**-1,45202532**	N15:480718	26,92	+2:YOR084W; +8:YOR083W;	2			
chrXV	480687	480787	100	**-1,105051229**	N15:480718	26,92	+2:YOR084W; +8:YOR083W;	2			
chrXV	480688	480787	99	**-1,311677074**	N15:480718	26,92	+2:YOR084W; +8:YOR083W;	2			
chrXV	480689	480787	98	**-0,483471549**	N15:480718	26,92	+2:YOR084W; +8:YOR083W;	2			
chrXV	480690	480787	97	**-1,535017791**	N15:480718	26,92	+2:YOR084W; +8:YOR083W;	2			
chrXV	480691	480787	96	**-1,26116167**	N15:480718	26,92	+2:YOR084W; +8:YOR083W;	2			
chrXV	480692	480787	95	**-1,434485007**	N15:480718	26,92	+2:YOR084W; +8:YOR083W;	2			
chrXV	480693	480787	94	**-1,437455211**	N15:480718	26,92	+2:YOR084W; +8:YOR083W;	2			
chrXV	480695	480787	92	**-2,026573821**	N15:480718	26,92	+2:YOR084W; +8:YOR083W;	2			
chrXV	480696	480787	91	**-1,46093622**	N15:480718	26,92	+2:YOR084W; +8:YOR083W;	2			
chrXV	480697	480787	90	**-1,918199292**	N15:480718	26,92	+2:YOR084W; +8:YOR083W;	2			
chrXV	480698	480787	89	**-1,060230518**	N15:480718	26,92	+2:YOR084W; +8:YOR083W;	2			
chrXV	480700	480787	87	**-1,504085721**	N15:480718	26,92	+2:YOR084W; +8:YOR083W;	2			
chrXV	480701	480787	86	**-2,370110132**	N15:480718	26,92	+2:YOR084W; +8:YOR083W;	2			
chrXV	480703	480787	84	**-1,388925352**	N15:480718	26,92	+2:YOR084W; +8:YOR083W;	2			
chrXV	480704	480787	83	**-1,660161518**	N15:480718	26,92	+2:YOR084W; +8:YOR083W;	2			
chrXV	482098	482249	151	**-1,589170937**	N15:482205	8,05	+2:YOR085W;	2			
chrXV	483506	483656	150	**-1,128814692**	N15:483559	37,401	+22:YOR086C;	22			
chrXV	490437	490586	149	**-1,227441921**				Overlap <50 bp			
chrXV	492152	492296	144	**-1,287616616**				Overlap <50 bp			
chrXV	492636	492766	130	**-1,122285526**	N15:492727	11,535	+2:YOR090C;	2			
chrXV	499981	500137	156	**-0,752649484**	N15:499985	29,69		Intergene			
chrXV	500251	500392	141	**-1,08025578**	N15:500266	37,264		Intergene			
chrXV	500870	501031	161	**-1,25000077**	N15:500992	38,972		Intergene			

CHR	START	END	L(bp)	ΔLknuc	Nuc ID	Fuzziness	Gene ID	Gene body postion or intergene	Terminal	rDNA	Telomeric
chrXV	504656	504829	173	**-1,393156044**	N15:504757	7,985	-1:YOR095C;	-1			
chrXV	504839	504995	156	**-1,449906994**	N15:504924	6,573		Intergene			
chrXV	505651	505827	176	**-1,557242507**	N15:505793	30,433	+1:YOR096W;	1			
chrXV	508541	508687	146	**-1,105428668**	N15:508522	35,529	+18:YOR098C;	18			
chrXV	512017	512169	152	**-1,388824829**	N15:512077	11,221	+3:YOR099W;	3			
chrXV	520467	520627	160	**-1,301870465**	N15:520577	0,707	-1:CUT843;	-1			
chrXV	521328	521487	159	**-1,138158843**	N15:521406	17,512	+3:YOR107W;	3			
chrXV	524434	524584	150	**-1,379271275**	N15:524552	11,303	+11:YOR108W;	11			
chrXV	526545	526704	159	**-0,91509561**	N15:526589	22,811	+10:YOR109W;	10			
chrXV	527282	527433	151	**-1,49865849**	N15:527364	13,18	+15:YOR109W;	15			
chrXV	528345	528493	148	**-1,285254183**	N15:528465	16,461	+22:YOR109W;	22			
chrXV	529088	529208	120	**-1,094024708**				Overlap <50 bp			
chrXV	534111	534263	152	**-1,018551216**	N15:534188	4,494	+2:YOR113W;	2			
chrXV	535122	535279	157	**-1,175776825**				Overlap <50 bp			
chrXV	539793	539942	149	**-1,173714924**	N15:539889	19,429		Intergene			
chrXV	540123	540277	154	**-1,557816261**	N15:540228	21,037	+26:YOR116C;	26			
chrXV	542672	542801	129	**-1,685596178**	N15:542768	19,242	+11:YOR116C;	11			
chrXV	545231	545391	160	**-1,373952932**	N15:545321	11,589	+3:YOR117W;	3			
chrXV	545588	545715	127	**-0,960989749**	N15:545625	8,302	+5:YOR117W;	5			
chrXV	550006	550155	149	**-1,452809124**	N15:550070	5,776	+2:YOR119C;	2			
chrXV	550413	550558	145	**-1,263444964**	N15:550502	8,472	-1:YOR119C;	-1			
chrXV	550604	550752	148	**-1,131553601**	N15:550670	15,946		Intergene			
chrXV	559497	559585	88	**-1,099368473**	N15:559589	8,764	+2:YOR125C;	2			
chrXV	559497	559598	101	**-0,457138739**	N15:559589	8,764	+2:YOR125C;	2			
chrXV	559497	559601	104	**-0,811486186**	N15:559589	8,764	+2:YOR125C;	2			
chrXV	559497	559654	157	**-1,345065945**	N15:559589	8,764	+2:YOR125C;	2			
chrXV	559919	560068	149	**-0,900188758**	N15:559990	9,136	+5*:YOR126C; -1:YOR125C;	5	TERM		
chrXV	559929	560086	157	**-0,934906742**	N15:559990	9,136	+5*:YOR126C; -1:YOR125C;	5	TERM		
chrXV	565053	565199	146	**-1,797448662**	N15:565222	25,393	+7*:YOR128C;	7	TERM		
chrXV	565053	565201	148	**-2,394599187**	N15:565222	25,393	+7*:YOR128C;	7	TERM		
chrXV	565053	565202	149	**-2,014650298**	N15:565222	25,393	+7*:YOR128C;	7	TERM		
chrXV	566775	566926	151	**-1,272484346**	N15:566870	23,51	+18*:YOR129C;	18	TERM		
chrXV	576392	576555	163	**-1,500433993**	N15:576489	38,592	+9:YOR133W;	9			
chrXV	590114	590263	149	**-1,397404152**	N15:590176	16,104	+16:YOR141C;	16			
chrXV	590721	590873	152	**-1,167212396**	N15:590856	36,321	+11:YOR141C;	11			
chrXV	592017	592170	153	**-1,21423786**	N15:592096	28,268	+4:YOR141C;	4			
chrXV	592017	592179	162	**-1,262648092**	N15:592096	28,268	+4:YOR141C;	4			
chrXV	592023	592179	156	**-1,079283922**	N15:592096	28,268	+4:YOR141C;	4			
chrXV	592044	592126	82	**-0,826**	N15:592096	28,268	+4:YOR141C;	4			
chrXV	595352	595475	123	**-0,903193347**				Overlap <50 bp			
chrXV	595452	595607	155	**-1,42336846**	N15:595551	22,068		Intergene			
chrXV	595885	596019	134	**-0,66626228**	N15:595950	30,643		Intergene			
chrXV	595885	596025	140	**-0,970297117**	N15:595950	30,643		Intergene			
chrXV	596928	597063	135	**-0,809359546**	N15:596968	29,195		Intergene			
chrXV	597189	597334	145	**-1,009440189**	N15:597178	5,657		Intergene			
chrXV	597375	597525	150	**-1,207102726**	N15:597445	14,048		Intergene			
chrXV	597554	597711	157	**-1,20557002**	N15:597645	29,513		Intergene			
chrXV	598045	598174	129	**-1,090487943**				Overlap <50 bp			
chrXV	598957	599107	150	**-1,361578722**	N15:599055	14,572		Intergene			
chrXV	600156	600263	107	**-1,082555398**				Overlap <50 bp			
chrXV	601179	601328	149	**-1,130738646**	N15:601265	12,561		Intergene			
chrXV	603463	603616	153	**-1,268555078**	N15:603547	12,629	+10:YOR144C;	10			
chrXV	605449	605600	151	**-1,133822925**	N15:605543	14,944	+2*:CUT384; +5:YOR145C;	2	TERM		
chrXV	606728	606884	156	**-1,307831664**	N15:606775	5,128	+2:YOR147W;	2			
chrXV	608095	608251	156	**-1,512299031**	N15:608159	32,269	+11:YOR147W;	11			
chrXV	610131	610285	154	**-1,216648706**	N15:610149	21,151	+9:YOR149C;	9			
chrXV	610432	610587	155	**-1,175664486**	N15:610582	10,45	+6:YOR149C;	6			
chrXV	610432	610587	155	**-1,175664486**	N15:610423	18,327	+7:YOR149C;	7			
chrXV	613403	613549	146	**-1,484149098**	N15:613529	27,017	+21:YOR151C;	21			
chrXV	617632	617793	161	**-1,189892761**	N15:617725	15,758	+4:YOR152C;	4			
chrXV	621001	621151	150	**-1,319977946**	N15:621077	32,654	+10:YOR153W;	10			
chrXV	622311	622441	130	**-1,013963775**				Overlap <50 bp			
chrXV	624028	624159	131	**-0,826285892**	N15:624066	28,715	+26:YOR153W;	26			
chrXV	625834	625959	125	**-0,958346872**	N15:625885	12,285	+8:YOR154W;	8			
chrXV	626417	626570	153	**-1,31788744**	N15:626508	32,039	+12*:YOR154W;	12	TERM		
chrXV	632144	632251	107	**-0,733677856**	N15:632173	3,619	+1:YOR158W;	1			
chrXV	632144	632253	109	**-0,869651273**	N15:632173	3,619	+1:YOR158W;	1			
chrXV	633485	633638	153	**-1,251462342**	N15:633575	8,468	-1:YOR160W; +1:YOR159C;	-1			
chrXV	635429	635578	149	**-1,34659384**	N15:635550	24,098	+11:YOR160W;	11			
chrXV	637512	637669	157	**-1,319490145**	N15:637581	32,836	+7:YOR161C;	7			
chrXV	641886	642041	155	**-1,375090187**	N15:641956	15,201	+2:YOR162C;	2			
chrXV	646176	646338	162	**-1,370502659**	N15:646189	23,384	+11:YOR165W;	11			
chrXV	647481	647636	155	**-1,378866228**	N15:647630	14,061	+6:YOR166C;	6			
chrXV	650796	650922	126	**-0,809272259**				Overlap <50 bp			
chrXV	650923	651080	157	**-1,586685502**	N15:650997	13,704	+12:YOR168W;	12			
chrXV	651588	651740	152	**-1,125245221**	N15:651687	17,587		Intergene			
chrXV	671862	672011	149	**-1,204001753**	N15:671896	6,408	+4:YOR179C;	4			
chrXV	674697	674849	152	**-0,991728093**	N15:674786	33,65	+3:YOR180C;	3			
chrXV	674884	675018	134	**-0,85652054**	N15:674983	36,793	+2:YOR180C;	2			
chrXV	677635	677796	161	**-1,243137998**	N15:677669	31,321	-1:anti176; +15:YOR181W;	-1			
chrXV	679372	679525	153	**-1,274887952**	N15:679447	6,314	+2:YOR183W; +2:YOR184W;	2			
chrXV	680555	680689	134	**-0,987564336**	N15:680616	11,06	+9*:YOR183W; +9*:YOR184W;	9	TERM		
chrXV	682390	682545	155	**-1,282906148**	N15:682487	7,106		Intergene			
chrXV	682421	682570	149	**-1,13095704**	N15:682487	7,106		Intergene			
chrXV	682600	682746	146	**-0,981373097**	N15:682669	23,768		Intergene			
chrXV	683178	683329	151	**-1,403272096**	N15:683179	19,274	+2:YOR186W;	2			
chrXV	683178	683329	151	**-1,403272096**	N15:683348	39,191	+3:YOR186W;	3			
chrXV	683542	683705	163	**-1,44124454**	N15:683602	15,188	+5:YOR186W;	5			
chrXV	698388	698544	156	**-1,054337602**	N15:698448	9,02		Intergene			
chrXV	699187	699320	133	**-1,367216594**	N15:699226	40,166	+9:YOR192C;	9			
chrXV	704256	704405	149	**-1,188485336**	N15:704277	28,583	+1:YOR192C-C;	1			
chrXV	705052	705198	146	**-1,179350103**	N15:705134	6,429		Intergene			
chrXV	707863	708011	148	**-1,107693705**				Overlap <50 bp			
chrXV	709269	709434	165	**-1,212752401**	N15:709269	19,95		Intergene			
chrXV	709269	709434	165	**-1,212752401**	N15:709445	21,213		Intergene			
chrXV	709269	709462	193	**-1,219461387**	N15:709269	19,95		Intergene			

CHR	START	END	L(bp)	ΔLknuc	Nuc ID	Fuzziness	Gene ID	Gene body postion or intergene	Terminal	rDNA	Telomeric
chrXV	709269	709462	193	**-1,219461387**	N15:709445	21,213		Intergene			
chrXV	709332	709434	102	**-0,10743918**	N15:709445	21,213		Intergene			
chrXV	709332	709462	130	**-0,913391215**	N15:709445	21,213		Intergene			
chrXV	709396	709550	154	**-1,216713811**	N15:709445	21,213		Intergene			
chrXV	709396	709571	175	**-1,399936269**	N15:709445	21,213		Intergene			
chrXV	709403	709571	168	**-1,303875346**	N15:709445	21,213		Intergene			
chrXV	712148	712278	130	**-0,972198581**	N15:712206	4,916	+3:YOR194C;	3			
chrXV	713754	713908	154	**-1,362633769**				Overlap <50 bp			
chrXV	716082	716234	152	**-1,267125867**	N15:716164	13,59	+5:YOR196C;	5			
chrXV	717294	717452	158	**-1,459867322**	N15:717288	5,391	+2:YOR197W;	2			
chrXV	717294	717452	158	**-1,459867322**	N15:717452	4,412	+3:YOR197W;	3			
chrXV	728130	728275	145	**-1,14756473**	N15:728119	15,822	+5:YOR206W;	5			
chrXV	728130	728275	145	**-1,14756473**	N15:728288	8,735	+6:YOR206W;	6			
chrXV	729241	729409	168	**-1,397940251**	N15:729229	32,517	+12:YOR206W;	12			
chrXV	730369	730527	158	**-1,430422755**	N15:730492	43,783	+18:YOR207C;	18			
chrXV	730626	730772	146	**-1,150314594**	N15:730666	35,066	+17:YOR207C;	17			
chrXV	732904	733026	122	**-0,924302228**	N15:732962	10,61	+4:YOR207C;	4			
chrXV	736114	736267	153	**-1,646749186**	N15:736157	26,475	+15*:YOR208W;	15	TERM		
chrXV	749719	749841	122	**-1,127719545**	N15:749778	6,555	+4:YOR217W;	4			
chrXV	751696	751803	107	**-0,841257138**	N15:751682	34,112	+16:YOR217W;	16			
chrXV	751696	751804	108	**-0,697615779**	N15:751682	34,112	+16:YOR217W;	16			
chrXV	751696	751805	109	**-0,915838655**	N15:751682	34,112	+16:YOR217W;	16			
chrXV	751696	751808	112	**-0,869910856**	N15:751682	34,112	+16:YOR217W;	16			
chrXV	759323	759474	151	**-1,259653474**	N15:759342	12,219		Intergene			
chrXV	759886	760039	153	**-1,69044603**	N15:759976	11,29	+2:YOR223W;	2			
chrXV	762029	762161	132	**-0,556527918**	N15:762082	8,462	+1:YOR226C;	1			
chrXV	762283	762425	142	**-0,934806715**	N15:762373	18,815	-1:YOR227W; -1:YOR226C;	-1			
chrXV	763377	763524	147	**-1,25323354**	N15:763477	13,164	+6:YOR227W;	6			
chrXV	765404	765530	126	**-1,339529827**	N15:765450	31,757	+17:YOR227W;	17			
chrXV	766078	766228	150	**-1,138452638**	N15:766247	28,768		Intergene			
chrXV	767013	767155	142	**-0,848927951**	N15:767164	18,501	+6:YOR228C;	6			
chrXV	767013	767155	142	**-0,848927951**	N15:767010	10,689	+7:YOR228C;	7			
chrXV	768735	768888	153	**-1,135017522**	N15:768713	6,066	+3:YOR229W;	3			
chrXV	770268	770418	150	**-0,842017312**				Overlap <50 bp			
chrXV	773124	773277	153	**-1,387038035**	N15:773165	8,86	+5:YOR231W;	5			
chrXV	775176	775297	121	**-1,097729609**	N15:775265	23,329	+6*:YOR232W; +2:CUT860;	6	TERM		
chrXV	777968	778119	151	**-1,479257799**	N15:778110	24,499	+16*:YOR233W;	16	TERM		
chrXV	783192	783285	93	**-0,384899711**	N15:783252	18,833	+10:YOR237W;	10			
chrXV	789013	789147	134	**-1,027739313**	N15:789066	4,796	+6:YOR242C;	6			
chrXV	790833	790987	154	**-1,502707383**	N15:790822	41,429	+10:YOR243C;	10			
chrXV	794701	794852	151	**-1,186773581**				Overlap <50 bp			
chrXV	796032	796168	136	**-0,862085404**	N15:796058	27,477	+6:YOR246C;	6			
chrXV	801709	801857	148	**-1,196312645**	N15:801792	9,854	+4:YOR250C;	4			
chrXV	802168	802313	145	**-1,141260029**	N15:802287	9,121	-1:anti-YOR251C; +1:YOR250C;	-1			
chrXV	804681	804839	158	**-1,28977132**	N15:804764	16,916	+1:SUT788; +3*:YOR253W;	1			
chrXV	805157	805300	143	**-1,011056665**	N15:805287	18,794	+12:YOR254C;	12			
chrXV	806800	806942	142	**-1,476985644**	N15:806847	6,309	+2:YOR254C;	2			
chrXV	806939	807090	151	**-1,232779546**	N15:807004	13,971	-1:YOR255W; +1:YOR254C;	-1			
chrXV	810091	810222	131	**-0,865174184**	N15:810121	29,58	+5:YOR256C;	5			
chrXV	815641	815767	126	**-1,381179464**	N15:815726	31,945	+12*:YOR260W;	12	TERM		
chrXV	816418	816570	152	**-0,990691825**	N15:816506	14,905	+4:YOR261C;	4			
chrXV	816891	817023	132	**-1,274052286**	N15:817009	12,738	+1:YOR261C; -1:YOR262W;	1			
chrXV	823212	823372	160	**-1,431096545**	N15:823302	8,699	+13:YOR267C;	13			
chrXV	823715	823880	165	**-1,269891588**	N15:823900	30,616	+9:YOR267C;	9			
chrXV	824770	824918	148	**-1,633170593**	N15:824827	14,414	+3:YOR267C;	3			
chrXV	824898	825045	147	**-1,218761175**	N15:824984	11,454	+2:YOR267C;	2			
chrXV	826750	826899	149	**-1,540544788**	N15:826822	8,819	+4:YOR269W;	4			
chrXV	828443	828590	147	**-1,081462881**	N15:828514	14,39	+14:YOR270C;	14			
chrXV	829595	829749	154	**-1,22598748**	N15:829756	17,773	+7:YOR270C;	7			
chrXV	832011	832158	147	**-1,090955345**	N15:832060	7,139	+2*:SUT790; +1:YOR271C;	2	TERM		
chrXV	838047	838199	152	**-1,161609891**	N15:838035	12,703	+4:YOR274W;	4			
chrXV	838047	838199	152	**-1,161609891**	N15:838195	8,468	+5:YOR274W;	5			
chrXV	838749	838897	148	**-1,068216573**	N15:838866	26,12	+9:YOR274W;	9			
chrXV	840989	841143	154	**-1,158306315**	N15:841051	10,147	-1:YOR276W; +1:YOR275C;	-1			
chrXV	845931	846091	160	**-1,43298733**	N15:845986	13,292	-1:YOR280C;	-1			
chrXV	851758	851911	153	**-1,552122585**	N15:851815	9,524	+1:YOR287C;	1			
chrXV	855604	855755	151	**-1,197852569**	N15:855699	29,815	+29:YOR290C;	29			
chrXV	856174	856339	165	**-1,268121005**	N15:856207	14,44	+26:YOR290C;	26			
chrXV	856222	856364	142	**-0,893837762**	N15:856207	14,44	+26:YOR290C;	26			
chrXV	860271	860423	152	**-1,564365666**	N15:860352	4,561	+1:YOR290C;	1			
chrXV	863117	863234	117	**-1,433576111**	N15:863177	11,759	+15:YOR291W;	15			
chrXV	866318	866474	156	**-1,229193294**	N15:866417	12,28	+2:YOR292C;	2			
chrXV	866636	866779	143	**-0,783495597**				Overlap <50 bp			
chrXV	873282	873420	138	**-0,864242621**	N15:873312	10,858	+20:YOR296W;	20			
chrXV	873426	873558	132	**-0,944089034**	N15:873537	26,853	+21:YOR296W;	21			
chrXV	873491	873643	152	**-1,507558695**	N15:873537	26,853	+21:YOR296W;	21			
chrXV	883077	883230	153	**-1,077114008**	N15:883079	18,005	+3:YOR303W; +3:YOR302W;	3			
chrXV	883077	883230	153	**-1,077114008**	N15:883239	22,73	+4:YOR303W; +4:YOR302W;	4			
chrXV	884043	884200	157	**-1,515341449**	N15:884168	36,873	3W; +9*:YOR302W; -1:YOR304W;	9	TERM		
chrXV	895782	895933	151	**-1,324438459**	N15:895865	5,086	+4:YOR308C;	4			
chrXV	900646	900802	156	**-1,691263989**	N15:900717	12,137	+4*:YOR312C;	4	TERM		
chrXV	902699	902828	129	**-1,201014529**	N15:902765	15,329	+2:YOR313C;	2			
chrXV	904931	905079	148	**-1,307752677**	N15:904922	20,404	+2:YOR315W;	2			
chrXV	917190	917343	153	**-1,130239538**	N15:917323	14,184	+9:YOR321W;	9			
chrXV	920557	920665	108	**-1,208185328**	N15:920598	9,731	+5:YOR322C;	5			
chrXV	928849	929014	165	**-1,489350113**	N15:928971	4,655	+21:YOR326W;	21			
chrXV	932648	932807	159	**-1,227006821**	N15:932684	31,149	+7:YOR328W;	7			
chrXV	932960	933112	152	**-1,159570289**	N15:933085	17,672	+10:YOR328W;	10			
chrXV	937286	937429	143	**-1,123638198**	N15:937309	27,898	+14:YOR329C; +4:SUT383;	14			
chrXV	941867	942022	155	**-1,424698493**	N15:941903	38,923	+10:YOR330C;	10			
chrXV	943142	943285	143	**-0,802926171**	N15:943207	14,3	+2:YOR330C;	2			
chrXV	956493	956644	151	**-1,585012508**	N15:956543	16,171	+14:YOR337W; -1:YOR338W;	14			
chrXV	958715	958860	145	**-0,95123906**	N15:958840	31,909	SUT384; +1:YOR339C; -1:Unit611;	4	TERM		
chrXV	961158	961313	155	**-1,58620315**	N15:961224	14,445	+3:YOR341W;	3			
chrXV	963324	963474	150	**-1,566831515**	N15:963309	7,455	+16:YOR341W;	16			
chrXV	967141	967307	166	**-1,663405161**	N15:967161	27,577	+4:YOR342C;	4			

CHR	START	END	L(bp)	ΔLknuc	Nuc ID	Fuzziness	Gene ID	Gene body postion or intergene	Terminal	rDNA	Telomeric
chrXV	967454	967605	151	**-1,459313211**	N15:967556	20,229	+2:YOR342C;	2			
chrXV	970738	970906	168	**-1,399703551**	N15:970789	9,713		Intergene			
chrXV	970759	970913	154	**-1,381936832**	N15:970789	9,713		Intergene			
chrXV	970847	970977	130	**-0,941875395**				Overlap <50 bp			
chrXV	970875	970977	102	**-0,254934627**				Overlap <50 bp			
chrXV	972298	972446	148	**-1,13376193**				Overlap <50 bp			
chrXV	975111	975257	146	**-1,203462867**				Overlap <50 bp			
chrXV	975228	975386	158	**-1,639351108**	N15:975305	32,512		Intergene			
chrXV	975904	976053	149	**-1,244179865**	N15:975989	28,042		Intergene			
chrXV	975908	976058	150	**-1,35249103**	N15:975989	28,042		Intergene			
chrXV	977285	977436	151	**-1,344094817**	N15:977376	19,483	+6:YOR344C;	6			
chrXV	977616	977761	145	**-1,004950594**				Overlap <50 bp			
chrXV	981987	982146	159	**-1,64885498**	N15:982019	4,393	+3:YOR346W;	3			
chrXV	986417	986558	141	**-1,162183004**	N15:986505	5,076	+1:YOR347C;	1			
chrXV	986699	986864	165	**-1,561544023**	N15:986780	28,922	-1:YOR347C; +13*:YOR348C;	-1			
chrXV	987920	988058	138	**-2,083581141**	N15:987920	30,887	+6:YOR348C;	6			
chrXV	988983	989114	131	**-0,967900218**	N15:989106	25,548	-1:YOR348C; +3:SUT798;	-1			
chrXV	989889	990033	144	**-1,282460001**	N15:989965	12,545	+2:YOR349W;	2			
chrXV	996318	996470	152	**-1,085585229**	N15:996407	7,574	+2:YOR351C;	2			
chrXV	997102	997264	162	**-1,582654014**	N15:997152	7,797	+1:YOR352W;	1			
chrXV	1000108	1000248	140	**-1,178900147**				Overlap <50 bp			
chrXV	1000780	1000917	137	**-1,031045705**	N15:1000816	4,167	+1:YOR353C;	1			
chrXV	1001402	1001553	151	**-1,222977032**	N15:1001454	10,083	+3*:CUT396; +11*:YOR354C;	3	TERM		
chrXV	1002082	1002260	178	**-1,856832932**	N15:1002190	48,149	+7:YOR354C;	7			
chrXV	1004460	1004618	158	**-1,56234428**	N15:1004454	50,205	+4*:SUT388;	4	TERM		
chrXV	1004460	1004618	158	**-1,56234428**	N15:1004637	17,349		Intergene			
chrXV	1005856	1006001	145	**-1,307118755**				Overlap <50 bp			
chrXV	1008258	1008393	135	**-1,05129403**	N15:1008325	5,508	+8:YOR356W;	8			
chrXV	1019659	1019809	150	**-1,352074057**	N15:1019734	9,883		Intergene			
chrXV	1019667	1019830	163	**-1,28065151**	N15:1019734	9,883		Intergene			
chrXV	1023929	1024082	153	**-1,406759754**	N15:1024013	16,86	+10:YOR365C;	10			
chrXV	1035603	1035747	144	**-1,101703903**	N15:1035709	23,887	+6:YOR372C;	6			
chrXV	1036929	1037060	131	**-1,036579827**	N15:1036972	5,565	+2:YOR373W;	2			
chrXV	1041309	1041484	175	**-1,36927801**	N15:1041351	34,665	+11*:YOR374W;	11	TERM		
chrXV	1042260	1042368	108	**-1,614496058**	N15:1042335	3,962	+5:YOR375C;	5			
chrXV	1042260	1042387	127	**-0,83117278**	N15:1042335	3,962	+5:YOR375C;	5			
chrXV	1042260	1042389	129	**-1,172543949**	N15:1042335	3,962	+5:YOR375C;	5			
chrXV	1042260	1042392	132	**-0,952643231**	N15:1042335	3,962	+5:YOR375C;	5			
chrXV	1042265	1042392	127	**-0,705963861**	N15:1042335	3,962	+5:YOR375C;	5			
chrXV	1042267	1042368	101	**-0,080719943**	N15:1042335	3,962	+5:YOR375C;	5			
chrXV	1043663	1043770	107	**-0,895569323**				Overlap <50 bp			
chrXV	1043663	1043771	108	**-0,671407021**				Overlap <50 bp			
chrXV	1043663	1043772	109	**-0,896788259**				Overlap <50 bp			
chrXV	1047470	1047621	151	**-1,323244143**	N15:1047478	18,606	+9:YOR377W;	9			
chrXV	1047470	1047621	151	**-1,323244143**	N15:1047635	27,803	+10:YOR377W;	10			
chrXV	1050132	1050280	148	**-1,212322379**	N15:1050219	11,683	+6:YOR378W; +4:anti179;	6			
chrXV	1050137	1050280	143	**-1,204365963**	N15:1050219	11,683	+6:YOR378W; +4:anti179;	6			
chrXV	1050608	1050773	165	**-1,204026491**	N15:1050703	11,077	+9:YOR378W; +1:anti179;	9			
chrXV	1050861	1050999	138	**-1,101962321**	N15:1050887	27,595	)R378W; -1:anti179; -1:YOR380W;	10			
chrXV	1052062	1052210	148	**-0,756635618**	N15:1052087	22,623	+7:YOR380W;	7			
chrXV	1056315	1056470	155	**-1,306437663**	N15:1056388	5,263	+6:YOR381W;	6			
chrXV	1059723	1059876	153	**-1,078255527**	N15:1059763	9,158	+4:YOR382W;	4			
chrXV	1060927	1061054	127	**-0,778526906**	N15:1060979	35,816	+2:YOR383C;	2			
chrXV	1061685	1061838	153	**-1,214711972**	N15:1061757	11,136	+2:YOR384W;	2			
chrXV	1062032	1062164	132	**-1,194783223**	N15:1062167	32,908	+4:YOR384W;	4			
chrXV	1062744	1062896	152	**-1,269826143**	N15:1062785	34,965	+8:YOR384W;	8			
chrXV	1071888	1072059	171	**-1,543313612**	N15:1071953	16,442	+7:YOR388C;	7			
chrXV	1073330	1073480	150	**-1,301725876**	N15:1073396	14,261		Intergene			
chrXV	1080435	1080579	144	**-0,953101116**	N15:1080493	2,646	+3:YOR393W;	3			
chrXV	1085191	1085346	155	**-1,390067537**	N15:1085246	25,325	-1:YOR396W;	-1			TEL RIGHT
chrXV	1086206	1086377	171	**-1,527536511**	N15:1086303	16,971	+6:YOR396W;	6			TEL RIGHT
chrXV	1086356	1086503	147	**-1,017606607**				Overlap <50 bp			TEL RIGHT
chrXV	1086512	1086642	130	**-0,862497144**	N15:1086572	24,194	+8:YOR396W;	8			TEL RIGHT
chrXV	1086893	1087029	136	**-1,156879929**	N15:1086930	1,414	+10:YOR396W;	10			TEL RIGHT
chrXV	1087274	1087436	162	**-1,354742742**	N15:1087405	11,59	+13:YOR396W;	13			TEL RIGHT
chrXV	1087375	1087530	155	**-1,799486779**	N15:1087405	11,59	+13:YOR396W;	13			TEL RIGHT
chrXV	1087509	1087646	137	**-0,699638736**	N15:1087575	10,116	+14:YOR396W;	14			TEL RIGHT
chrXV	1087804	1087947	143	**-1,204240291**	N15:1087853	10,693	+16:YOR396W;	16			TEL RIGHT
chrXV	1087852	1087977	125	**-0,604932882**	N15:1087853	10,693	+16:YOR396W;	16			TEL RIGHT
chrXV	1088077	1088229	152	**-1,196144496**	N15:1088185	13	+18:YOR396W;	18			TEL RIGHT
chrXV	1088150	1088294	144	**-0,904179469**	N15:1088185	13	+18:YOR396W;	18			TEL RIGHT
chrXV	1088247	1088382	135	**-0,693275778**	N15:1088344	22,716	+19:YOR396W;	19			TEL RIGHT
chrXV	1088247	1088434	187	**-0,668246016**	N15:1088344	22,716	+19:YOR396W;	19			TEL RIGHT
chrXV	1088290	1088382	92	**-0,048271239**	N15:1088344	22,716	+19:YOR396W;	19			TEL RIGHT
chrXV	1088290	1088434	144	**-0,925396865**	N15:1088344	22,716	+19:YOR396W;	19			TEL RIGHT
chrXV	1088629	1088781	152	**-1,335406845**	N15:1088692	20,502	+21:YOR396W;	21			TEL RIGHT
chrXV	1088930	1089060	130	**-0,991449961**	N15:1088919	25,456	+22:YOR396W;	22			TEL RIGHT
chrXV	1089068	1089229	161	**-1,203719312**	N15:1089094	17,678	+23:YOR396W;	23			TEL RIGHT
chrXV	1090465	1090616	151	**-1,083483505**	N15:1090543	42,426	+32:YOR396W;	32			TEL RIGHT
chrXVI	523	674	151	**-1,273871252**	N16:600	36,715	+34:YPL283C;	34			TEL LEFT
chrXVI	847	1021	174	**-1,639074792**	N16:925	37,82	+32:YPL283C;	32			TEL LEFT
chrXVI	1688	1839	151	**-1,16643656**	N16:1728	11,24	+27:YPL283C;	27			TEL LEFT
chrXVI	1700	1834	134	**-0,926880732**	N16:1728	11,24	+27:YPL283C;	27			TEL LEFT
chrXVI	1725	1821	96	**-0,580124735**	N16:1728	11,24	+27:YPL283C;	27			TEL LEFT
chrXVI	1725	1834	109	**-0,260418943**	N16:1728	11,24	+27:YPL283C;	27			TEL LEFT
chrXVI	2079	2209	130	**-1,020434911**	N16:2222	27,006	+24:YPL283C;	24			TEL LEFT
chrXVI	2079	2209	130	**-1,020434911**	N16:2056	0,707	+25:YPL283C;	25			TEL LEFT
chrXVI	2358	2510	152	**-1,256586976**	N16:2443	19,858	+23:YPL283C;	23			TEL LEFT
chrXVI	2705	2849	144	**-0,948342038**	N16:2758	21,385	+21:YPL283C;	21			TEL LEFT
chrXVI	2705	2892	187	**-1,227772768**	N16:2758	21,385	+21:YPL283C;	21			TEL LEFT
chrXVI	2757	2849	92	**-0,316913537**	N16:2758	21,385	+21:YPL283C;	21			TEL LEFT
chrXVI	2757	2892	135	**-0,934610579**	N16:2758	21,385	+21:YPL283C;	21			TEL LEFT
chrXVI	2845	2989	144	**-0,8292503**	N16:2943	8,888	+20:YPL283C;	20			TEL LEFT
chrXVI	2910	3062	152	**-1,256599761**	N16:2943	8,888	+20:YPL283C;	20			TEL LEFT
chrXVI	3162	3287	125	**-0,726889625**	N16:3271	12,897	+18:YPL283C;	18			TEL LEFT
chrXVI	3192	3335	143	**-1,42227426**	N16:3271	12,897	+18:YPL283C;	18			TEL LEFT

CHR	START	END	L(bp)	ΔLknuc	Nuc ID	Fuzziness	Gene ID	Gene body postion or intergene	Terminal	rDNA	Telomeric
chrXVI	3192	3343	151	**-1,397479633**	N16:3271	12,897	+18:YPL283C;	18			TEL LEFT
chrXVI	3493	3630	137	**-1,048095716**	N16:3559	7,767	+16:YPL283C;	16			TEL LEFT
chrXVI	3609	3764	155	**-1,8528086**	N16:3722	7,024	+15:YPL283C;	15			TEL LEFT
chrXVI	3703	3865	162	**-1,281655478**	N16:3880	4,583	+14:YPL283C;	14			TEL LEFT
chrXVI	3703	3865	162	**-1,281655478**	N16:3722	7,024	+15:YPL283C;	15			TEL LEFT
chrXVI	4928	5069	141	**-1,208585581**	N16:4975	20,075	+7:YPL283C;	7			TEL LEFT
chrXVI	8461	8628	167	**-1,226955528**	N16:8544	10,97	0:YPL282C;	0			
chrXVI	10566	10710	144	**-1,156743805**	N16:10653	6,083	+3:YPL281C;	3			
chrXVI	23283	23434	151	**-1,159502225**	N16:23371	6,573	+4:YPL274W;	4			
chrXVI	26164	26341	177	**-1,619423797**	N16:26333	2,483	+1:anti-YPL272C;	1			
chrXVI	26440	26591	151	**-1,363898592**				Overlap <50 bp			
chrXVI	30175	30330	155	**-1,617591609**	N16:30253	10,629	-1:YPL270W; +2*:YPL271W;	-1			
chrXVI	30411	30566	155	**-1,307708451**	N16:30492	11,147	+1:YPL270W;	1			
chrXVI	37895	38053	158	**-1,139674632**	N16:37928	29,233	+17*:YPL268W; -1:YPL267W;	17	TERM		
chrXVI	41612	41762	150	**-1,327971575**	N16:41620	14,747	+5:YPL265W; +18:YPL264C;	5			
chrXVI	41938	42057	119	**-1,189522898**				Overlap <50 bp			
chrXVI	44256	44374	118	**-1,220080111**	N16:44326	28,272	+3:YPL264C;	3			
chrXVI	54075	54252	177	**-1,680003736**	N16:54126	22,106	+9:YPL258C;	9			
chrXVI	55410	55560	150	**-1,228240777**	N16:55405	35,216	+1:YPL258C;	1			
chrXVI	56814	56963	149	**-1,136190113**	N16:56959	36,501		Intergene			
chrXVI	56988	57111	123	**-0,97472828**				Overlap <50 bp			
chrXVI	57088	57243	155	**-1,33601146**	N16:57189	29,501		Intergene			
chrXVI	57521	57655	134	**-1,195236979**	N16:57591	17,436		Intergene			
chrXVI	57521	57661	140	**-1,276093078**	N16:57591	17,436		Intergene			
chrXVI	57717	57868	151	**-1,267168688**	N16:57746	27,135		Intergene			
chrXVI	57727	57872	145	**-1,353081866**	N16:57746	27,135		Intergene			
chrXVI	57747	57896	149	**-1,281826372**	N16:57746	27,135		Intergene			
chrXVI	57999	58153	154	**-1,116137424**	N16:58141	5,132		Intergene			
chrXVI	58564	58699	135	**-1,265287266**	N16:58596	13,892		Intergene			
chrXVI	58718	58879	161	**-1,404572534**	N16:58834	41,405		Intergene			
chrXVI	59011	59161	150	**-1,080921355**	N16:59079	42,771		Intergene			
chrXVI	59190	59347	157	**-1,440055979**	N16:59296	28,501		Intergene			
chrXVI	59681	59810	129	**-1,444006599**	N16:59829	14,731		Intergene			
chrXVI	60593	60743	150	**-1,579459688**	N16:60686	10,786		Intergene			
chrXVI	61572	61714	142	**-1,209575201**	N16:61694	32,254		Intergene			
chrXVI	66888	67040	152	**-1,241233796**	N16:66990	12,156	-1:YPL256C;	-1			
chrXVI	67654	67814	160	**-1,367789871**	N16:67736	6,899	+1:YPL255W;	1			
chrXVI	69456	69617	161	**-1,351937514**	N16:69502	6,824	+2:YPL254W;	2			
chrXVI	73482	73631	149	**-0,86615081**	N16:73588	12,92	+3:YPL252C;	3			
chrXVI	74222	74371	149	**-1,030979493**	N16:74321	17,044	+2:CUT401; +3:YPL250C;	2			
chrXVI	85235	85390	155	**-1,490098199**	N16:85298	8,456	+1:YPL246C; -1:YPL245W;	1			
chrXVI	86229	86396	167	**-1,437505095**	N16:86219	13,029	+5:YPL245W;	5			
chrXVI	93799	93933	134	**-1,152232688**	N16:93856	30,448	+9:YPL242C;	9			
chrXVI	97906	98054	148	**-1,096079875**	N16:97910	33,147	+6:YPL240C;	6			
chrXVI	100090	100196	106	**-1,556415834**	N16:100168	6,907	-1:YPL237W; +5*:YPL239W;	-1			
chrXVI	101988	102144	156	**-1,014178668**	N16:102072	5,814	+5:YPL236C;	5			
chrXVI	111563	111694	131	**-1,021204548**	N16:111614	32,322	+20:YPL231W;	20			
chrXVI	111839	111996	157	**-1,349496182**	N16:111919	37,173		Intergene			
chrXVI	112816	112964	148	**-1,009412654**	N16:112902	31,405		Intergene			
chrXVI	113851	114002	151	**-1,19115666**	N16:113884	13,142		Intergene			
chrXVI	115900	116007	107	**-1,336787112**	N16:115995	32,411	+3:SUT807; +2:YPL230W;	3			
chrXVI	115900	116008	108	**-1,314082644**	N16:115995	32,411	+3:SUT807; +2:YPL230W;	3			
chrXVI	115900	116009	109	**-1,151531721**	N16:115995	32,411	+3:SUT807; +2:YPL230W;	3			
chrXVI	115900	116013	113	**-1,345128504**	N16:115995	32,411	+3:SUT807; +2:YPL230W;	3			
chrXVI	117285	117438	153	**-0,694359805**				Overlap <50 bp			
chrXVI	119500	119650	150	**-1,408520542**	N16:119550	20,761	+9:YPL228W;	9			
chrXVI	119632	119770	138	**-1,487245546**	N16:119717	8,325	+10:YPL228W;	10			
chrXVI	124878	125016	138	**-1,070973347**	N16:124969	11,777	+19:YPL226W;	19			
chrXVI	127061	127209	148	**-1,148514355**	N16:127222	6,713	+7:YPL224C;	7			
chrXVI	127061	127209	148	**-1,148514355**	N16:127054	5,419	+8:YPL224C;	8			
chrXVI	127345	127506	161	**-1,385029955**	N16:127392	16,881	+6:YPL224C;	6			
chrXVI	128019	128185	166	**-1,76316196**	N16:128056	8,741	-1:Unit649; +2:YPL224C;	-1			
chrXVI	128210	128316	106	**-0,955424382**	N16:128260	23,322	+1:Unit649; +1:YPL224C;	1			
chrXVI	128210	128332	122	**-1,218193357**	N16:128260	23,322	+1:Unit649; +1:YPL224C;	1			
chrXVI	130172	130324	152	**-1,837982522**	N16:130248	18,302	+2:YPL222W;	2			
chrXVI	131123	131279	156	**-1,192119134**	N16:131253	37,125	+8:YPL222W;	8			
chrXVI	136763	136914	151	**-1,138761843**	N16:136878	7,563	+2:YPL219W;	2			
chrXVI	136805	136963	158	**-1,239435241**	N16:136878	7,563	+2:YPL219W;	2			
chrXVI	139377	139534	157	**-1,470719293**	N16:139490	11,446		Intergene			
chrXVI	142443	142561	118	**-1,453646725**	N16:142521	10,046	+5:YPL217C;	5			
chrXVI	144178	144336	158	**-1,361994703**	N16:144285	18,687	+5:YPL216W;	5			
chrXVI	149028	149196	168	**-1,204811029**	N16:149123	8,408	+8:YPL214C;	8			
chrXVI	152572	152722	150	**-1,096591693**	N16:152635	5,857	+4:YPL212C;	4			
chrXVI	161247	161414	167	**-1,259632979**	N16:161357	51,735	+10:YPL207W;	10			
chrXVI	164847	164957	110	**-1,206490994**	N16:164935	8,958	+6:YPL204W;	6			
chrXVI	165348	165502	154	**-1,594971934**	N16:165511	34,953	+10:YPL204W; +1:SUT811;	10			
chrXVI	167030	167184	154	**-0,980405571**	N16:167113	32,5	+7:YPL203W;	7			
chrXVI	169266	169418	152	**-1,221815775**	N16:169376	17,029	+1:YPL202C;	1			
chrXVI	173412	173549	137	**-0,981266874**	N16:173465	6,364		Intergene			
chrXVI	174194	174363	169	**-1,410004065**	N16:174232	28,827		Intergene			
chrXVI	175111	175256	145	**-1,357605346**	N16:175175	6,834	+2:YPL196W;	2			
chrXVI	177466	177621	155	**-1,3314233**	N16:177552	31,17	+9:YPL195W;	9			
chrXVI	177625	177797	172	**-1,51037597**	N16:177780	21,378	+10:YPL195W;	10			
chrXVI	189167	189337	170	**-1,527417555**	N16:189180	28,27	+1:YPL189W;	1			
chrXVI	190409	190560	151	**-1,407630912**	N16:190473	22,412	+9:YPL189W;	9			
chrXVI	191083	191228	145	**-1,30832992**	N16:191166	17,058	PL189W; +1:CUT888; -1:YPL188W;	13	TERM		
chrXVI	193727	193886	159	**-1,020687158**	N16:193851	10,402	+2:YPL187W;	2			
chrXVI	194794	194946	152	**-1,393461999**	N16:194885	7,823	+3:YPL186C;	3			
chrXVI	198714	198866	152	**-1,424144256**	N16:198810	5,203	-1:YPL183W-A;	-1			
chrXVI	200785	200926	141	**-1,250227923**				Overlap <50 bp			
chrXVI	203076	203237	161	**-1,324457735**	N16:203255	19,502	+1:YPL181W;	1			
chrXVI	203882	204036	154	**-1,292423736**	N16:203940	13,722	+5:YPL181W;	5			
chrXVI	205158	205313	155	**-1,181345049**	N16:205222	6,293	0:YPL180W; -1:CUT890;	0			
chrXVI	211347	211529	182	**-1,557206804**	N16:211407	47,917		Intergene			
chrXVI	212567	212715	148	**-1,254183085**	N16:212620	11,769	+4:YPL178W;	4			
chrXVI	213085	213234	149	**-1,363917648**	N16:213180	30,105	+7*:YPL177C;	7	TERM		

CHR	START	END	L(bp)	ΔLknuc	Nuc ID	Fuzziness	Gene ID	Gene body postion or intergene	Terminal	rDNA	Telomeric
chrXVI	220298	220444	146	**-1,125840854**	N16:220437	26,974	+16:YPL174C;	16			
chrXVI	227791	227944	153	**-1,245872512**				Overlap <50 bp			
chrXVI	228023	228173	150	**-1,415878485**	N16:228073	4,95	-1:YPL170W;	-1			
chrXVI	235024	235161	137	**-1,413746474**	N16:235068	23,858	+14:YPL167C;	14			
chrXVI	238735	238888	153	**-1,380864364**	N16:238814	17,992	+9:YPL166W; +3:YPL165C;	9			
chrXVI	239149	239288	139	**-1,395078608**	N16:239177	17,627	.66W; +1:YPL165C; +15*:YPL164C;	11	TERM		
chrXVI	240071	240209	138	**-1,120280653**	N16:240084	21,307	+10:YPL164C;	10			
chrXVI	240947	241098	151	**-1,132740053**	N16:241005	8,826	+4:YPL164C;	4			
chrXVI	242739	242891	152	**-1,283738763**	N16:242817	22,634	+9*:YPL162C; 0:YPL163C;	9	TERM		
chrXVI	242995	243131	136	**-1,37529551**	N16:243075	19,263	+7:YPL162C; -1:anti182;	7			
chrXVI	244964	245114	150	**-1,291164006**	N16:245006	15,132	+9:YPL161C;	9			
chrXVI	246169	246331	162	**-1,333655341**	N16:246246	14,621	+1:YPL161C;	1			
chrXVI	247500	247655	155	**-1,263520105**				Overlap <50 bp			
chrXVI	247870	248025	155	**-1,489905312**	N16:248033	42,382	+8:YPL160W;	8			
chrXVI	248518	248667	149	**-1,428423709**	N16:248596	31,886	+11:YPL160W;	11			
chrXVI	251350	251499	149	**-1,480166043**	N16:251383	22,257	+3:YPL159C;	3			
chrXVI	251647	251802	155	**-1,020723248**	N16:251733	5,797	+1:YPL159C;	1			
chrXVI	254749	254901	152	**-1,735333272**	N16:254752	5,046	+1:YPL157W;	1			
chrXVI	254749	254901	152	**-1,735333272**	N16:254920	8,813	+2:YPL157W;	2			
chrXVI	257303	257455	152	**-1,291792731**	N16:257371	12,319	+13:YPL155C;	13			
chrXVI	258470	258614	144	**-1,361375157**	N16:258522	9,933	+6:YPL155C;	6			
chrXVI	269441	269592	151	**-1,739677473**	N16:269499	29,331	+10:YPL150W;	10			
chrXVI	272088	272225	137	**-1,154583116**	N16:272113	33,171	+6*:YPL148C;	6	TERM		
chrXVI	273849	274002	153	**-1,98419593**	N16:273925	33,991	+5:YPL147W;	5			
chrXVI	275518	275683	165	**-1,86357557**	N16:275553	22,26	+14:YPL147W;	14			
chrXVI	278767	278916	149	**-1,258316783**	N16:278752	21,479	+8:YPL145C;	8			
chrXVI	284968	285121	153	**-1,068355036**	N16:285013	29,392	+8:YPL141C;	8			
chrXVI	287278	287435	157	**-1,274300131**	N16:287376	11,3	+12*:YPL140C;	12	TERM		
chrXVI	287516	287643	127	**-1,061338708**	N16:287547	21,811	+11:YPL140C;	11			
chrXVI	295119	295280	161	**-1,172228324**	N16:295214	31,102	+11:YPL137C;	11			
chrXVI	296343	296490	147	**-1,211616509**	N16:296416	17,992	+4:YPL137C;	4			
chrXVI	301254	301382	128	**-1,435572917**	N16:301333	19	-1:YPL132W; +1:YPL133C;	-1			
chrXVI	305851	306015	164	**-1,51799359**	N16:305962	6,807		Intergene			
chrXVI	306387	306542	155	**-1,504147561**	N16:306463	42,894	+12*:YPL128C;	12	TERM		
chrXVI	317763	317907	144	**-1,256662107**	N16:317793	33,037		Intergene			
chrXVI	318699	318846	147	**-1,562030858**				Overlap <50 bp			
chrXVI	322025	322158	133	**-1,116361258**	N16:322082	3,808	+1:YPL120W;	1			
chrXVI	323166	323321	155	**-1,454678642**	N16:323256	23,173	+8:YPL120W;	8			
chrXVI	332594	332745	151	**-0,906444225**				Overlap <50 bp			
chrXVI	332948	333099	151	**-1,284124629**	N16:333032	23,522	+17:YPL115C;	17			
chrXVI	336537	336643	106	**-1,411968532**	N16:336620	3,987	+5:YPL113C;	5			
chrXVI	336537	336650	113	**-1,295826184**	N16:336620	3,987	+5:YPL113C;	5			
chrXVI	341289	341447	158	**-1,233493369**	N16:341297	29,523	+22*:YPL110C;	22	TERM		
chrXVI	344751	344913	162	**-1,442602772**	N16:344907	35,117	-1:SUT402; 0:YPL110C;	-1			
chrXVI	344751	344913	162	**-1,442602772**	N16:344736	12,754	+2:YPL110C;	2			
chrXVI	350439	350593	154	**-1,191141616**	N16:350549	31,706	+12:YPL106C;	12			
chrXVI	351311	351471	160	**-1,55099552**	N16:351332	7,05	+7:YPL106C;	7			
chrXVI	354968	355128	160	**-1,300410051**	N16:355047	5,857	+3:YPL105C;	3			
chrXVI	361977	362073	96	**-0,892770432**	N16:362047	4,55	+2:YPL100W;	2			
chrXVI	364336	364488	152	**-1,272054996**	N16:364416	8,916	+3:YPL098C;	3			
chrXVI	366545	366652	107	**-1,180861549**	N16:366569	3,601	+2:YPL096C-A;	2			
chrXVI	366545	366653	108	**-1,165589457**	N16:366569	3,601	+2:YPL096C-A;	2			
chrXVI	367044	367180	136	**-1,208608464**	N16:367128	9,928	+2:YPL096W;	2			
chrXVI	370599	370734	135	**-1,104173166**	N16:370630	8,361	+1:YPL094C; -1:YPL093W;	1			
chrXVI	370905	371054	149	**-1,095555092**	N16:370964	15,531	-1:YPL094C; +1:YPL093W;	-1			
chrXVI	373474	373616	142	**-1,092227486**	N16:373635	17,473	+1:YPL092W; -1:CUT895;	1			
chrXVI	379313	379470	157	**-1,424126068**	N16:379337	56,889	+2:SUT406;	2			
chrXVI	379794	379947	153	**-1,115482981**				Overlap <50 bp			
chrXVI	379982	380135	153	**-1,428326443**	N16:379982	17,445	+6:SUT406;	6			
chrXVI	379982	380135	153	**-1,428326443**	N16:380145	14,986	+7:SUT406;	7			
chrXVI	383644	383787	143	**-1,089333684**	N16:383784	18,111	+4:YPL087W;	4			
chrXVI	385233	385386	153	**-1,14942033**	N16:385330	17,297	+8:YPL086C;	8			
chrXVI	389294	389391	97	**-1,010099859**	N16:389336	40,087	+16:YPL085W;	16			
chrXVI	390081	390232	151	**-1,1283173**	N16:390091	25,843	+21:YPL085W;	21			
chrXVI	390081	390232	151	**-1,1283173**	N16:390239	0	+22:YPL085W;	22			
chrXVI	390545	390691	146	**-1,321041103**	N16:390526	34,385	+24:YPL085W;	24			
chrXVI	392582	392731	149	**-1,505449478**	N16:392651	35,2		Intergene			
chrXVI	392582	392736	154	**-1,259859193**	N16:392651	35,2		Intergene			
chrXVI	394420	394573	153	**-1,500244918**	N16:394533	3,962	+4:YPL084W;	4			
chrXVI	407155	407307	152	**-1,127881795**	N16:407273	15,834		Intergene			
chrXVI	407160	407307	147	**-1,378323552**	N16:407273	15,834		Intergene			
chrXVI	411818	411982	164	**-1,480884868**	N16:411890	10,252	-1:YPL075W; -1:CUT897;	-1			
chrXVI	411838	411991	153	**-1,630554274**	N16:411890	10,252	-1:YPL075W; -1:CUT897;	-1			
chrXVI	412957	413111	154	**-1,226443232**				Overlap <50 bp			
chrXVI	415881	416027	146	**-1,290705482**	N16:415956	10,991	+2:YPL074W;	2			
chrXVI	417433	417565	132	**-1,41043892**	N16:417568	26,063	+12:YPL074W;	12			
chrXVI	418493	418641	148	**-1,390537882**				Overlap <50 bp			
chrXVI	418678	418846	168	**-1,40968311**	N16:418685	22,013	+3:YPL072W;	3			
chrXVI	418678	418846	168	**-1,40968311**	N16:418866	7,301	+4:YPL072W;	4			
chrXVI	432250	432440	190	**-1,695972439**	N16:432391	28,898	+1:Unit653; -1:YPL061W;	1			
chrXVI	432594	432737	143	**-1,033617726**	N16:432613	20,408	+1:YPL061W;	1			
chrXVI	432908	433068	160	**-0,949511615**	N16:433087	53,51	+4:YPL061W;	4			
chrXVI	433481	433638	157	**-1,271585009**	N16:433601	26,912	+7:YPL061W;	7			
chrXVI	437116	437278	162	**-1,31353259**	N16:437280	12,702		Intergene			
chrXVI	439535	439688	153	**-1,943673809**				Overlap <50 bp			
chrXVI	442027	442174	147	**-1,480401127**	N16:442080	10,017		Intergene			
chrXVI	442326	442474	148	**-1,447406976**	N16:442468	19,553		Intergene			
chrXVI	442340	442502	162	**-1,326834464**	N16:442468	19,553		Intergene			
chrXVI	446281	446447	166	**-1,351141968**	N16:446378	7,182	+25:YPL058C;	25			
chrXVI	448877	449010	133	**-1,273597369**	N16:448925	17,257	+11:YPL058C;	11			
chrXVI	454839	454945	106	**-0,811927557**	N16:454945	4,604	+1:YPL055C;	1			
chrXVI	462929	463059	130	**-1,29799693**	N16:462975	23,302	+7:YPL049C;	7			
chrXVI	465016	465172	156	**-1,297256244**	N16:465100	13,985	+6:YPL048W;	6			
chrXVI	466157	466315	158	**-1,352914387**	N16:466243	4,926	+3:YPL047W;	3			
chrXVI	474538	474690	152	**-1,109747004**	N16:474587	12,49	+3:YPL042C;	3			
chrXVI	484126	484288	162	**-1,44668286**	N16:484123	4,722	+9:YPL036W;	9			

CHR	START	END	L(bp)	ΔLknuc	Nuc ID	Fuzziness	Gene ID	Gene body postion or intergene	Terminal	rDNA	Telomeric
chrXVI	484126	484288	162	-1,44668286	N16:484296	18,118	+10:YPL036W;	10			
chrXVI	484807	484963	156	-1,391719747				Overlap <50 bp			
chrXVI	487013	487184	171	-1,772409896	N16:487139	30,232	+5*:YPL034W; +7*:YPL033C;	5	TERM		
chrXVI	493217	493377	160	-1,301695363	N16:493271	33,361	-1:YPL031C; -1:YPL030W;	-1			
chrXVI	497274	497428	154	-1,166669092	N16:497348	7,662	+12:YPL029W; +3*:CUT903;	12			
chrXVI	501076	501229	153	-1,384027529	N16:501190	23,255	+7*:YPL026C;	7	TERM		
chrXVI	501841	501999	158	-1,33694335	N16:501884	19,243	+3:YPL026C;	3			
chrXVI	505233	505380	147	-1,433088424	N16:505303	36,928	+8:YPL023C;	8			
chrXVI	506320	506478	158	-1,406132268	N16:506395	2,881	+1:YPL023C; -1:YPL022W;	1			
chrXVI	506594	506750	156	-1,307658985	N16:506676	24,906	-1:YPL023C; +1:YPL022W;	-1			
chrXVI	509864	510024	160	-1,650989859	N16:509922	26,571	+21:YPL022W;	21			
chrXVI	511391	511535	144	-1,85231565	N16:511439	13,342	+3:YPL021W;	3			
chrXVI	517443	517595	152	-1,255230609	N16:517508	19,453	0:YPL018W;	0			
chrXVI	519049	519173	124	-1,293611838	N16:519058	35,379	+9:YPL018W; +9:YPL017C;	9			
chrXVI	520082	520209	127	-1,050650882	N16:520178	33,32	+2:YPL017C;	2			
chrXVI	522508	522654	146	-1,372160162	N16:522677	10,832	+14:YPL016W; +1*:anti186;	14			
chrXVI	522574	522734	160	-1,395786133	N16:522677	10,832	+14:YPL016W; +1*:anti186;	14			
chrXVI	525954	526108	154	-1,55572356	N16:526036	10,69	+6:YPL015C;	6			
chrXVI	536923	537076	153	-1,224652735	N16:537021	31,915	+13:YPL009C;	13			
chrXVI	538001	538163	162	-1,56521883	N16:538105	11,874	+6:YPL009C;	6			
chrXVI	541695	541831	136	-0,979337228	N16:541839	46,217	+18:YPL008W;	18			
chrXVI	547021	547160	139	-1,138461346	N16:547005	28,754	+16:YPL006W;	16			
chrXVI	547636	547771	135	-1,210576363	N16:547723	5,601	+20:YPL006W;	20			
chrXVI	547851	548005	154	-1,21085727	N16:547889	12,608	+21*:YPL006W;	21	TERM		
chrXVI	550617	550788	171	-1,377887712	N16:550762	34,631	+6*:YPL004C;	6	TERM		
chrXVI	553457	553564	107	-1,304362682	N16:553491	32,716	+6:YPL002C;	6			
chrXVI	553457	553565	108	-1,276748346	N16:553491	32,716	+6:YPL002C;	6			
chrXVI	560771	560926	155	-1,128971778	N16:560761	20,182		Intergene			
chrXVI	560771	560926	155	-1,128971778	N16:560933	8,042		Intergene			
chrXVI	564313	564461	148	-1,682215094	N16:564375	11,256	+5:YPR004C;	5			
chrXVI	564317	564438	121	-1,086627763	N16:564375	11,256	+5:YPR004C;	5			
chrXVI	566468	566620	152	-1,411216924	N16:566631	20,839	+1:YPR005C;	1			
chrXVI	566468	566620	152	-1,411216924	N16:566462	44,641	+2:YPR005C;	2			
chrXVI	568077	568211	134	-1,20877957	N16:568157	11,349	+6:YPR006C;	6			
chrXVI	573444	573609	165	-1,60620184	N16:573552	11,533	+7:YPR008W;	7			
chrXVI	573688	573839	151	-1,144053447	N16:573733	13,408	+8:YPR008W;	8			
chrXVI	578898	579048	150	-1,017424855	N16:579014	14,839	+13:YPR010C;	13			
chrXVI	580106	580256	150	-1,330128816	N16:580137	29,606	+7:YPR010C;	7			
chrXVI	581758	581907	149	-1,488594746	N16:581900	7,521	+3*:CUT424;	3	TERM		
chrXVI	581758	581907	149	-1,488594746	N16:581737	3,899	+2:CUT424;	2			
chrXVI	582967	583116	149	-1,252378763	N16:583058	11,125	UT416; -1:Unit633; -1:YPR010C-A;	1			
chrXVI	584868	585014	146	-1,250563813	N16:584868	19,99	+8:YPR013C;	8			
chrXVI	591560	591713	153	-1,179279205	N16:591636	21,79		Intergene			
chrXVI	592210	592352	142	-1,141968051	N16:592274	34,238	+6*:YPR016C;	6	TERM		
chrXVI	593528	593683	155	-1,337265577	N16:593589	7,767	+3:YPR017C;	3			
chrXVI	594996	595138	142	-1,211624931	N16:595089	43,597	+5:YPR018W;	5			
chrXVI	599046	599200	154	-1,369215328	N16:599073	8,562	+16:YPR019W;	16			
chrXVI	600831	600997	166	-1,349344516	N16:600995	6,928	+17:YPR021C;	17			
chrXVI	600831	600997	166	-1,349344516	N16:600844	4,879	+18:YPR021C;	18			
chrXVI	601108	601231	123	-1,072171781	N16:601151	14,846	+16:YPR021C;	16			
chrXVI	603139	603315	176	-1,716632526	N16:603211	4,59	+3:YPR021C;	3			
chrXVI	604400	604533	133	-1,276190771	N16:604513	17,952		Intergene			
chrXVI	611112	611263	151	-0,763729014				Overlap <50 bp			
chrXVI	611769	611929	160	-1,453074942	N16:611782	8,655	+10:YPR024W;	10			
chrXVI	611769	611929	160	-1,453074942	N16:611938	33,548	+11:YPR024W;	11			
chrXVI	614264	614371	107	-1,320799794	N16:614344	5,958	+2:YPR025C;	2			
chrXVI	614264	614381	117	-1,576300435	N16:614344	5,958	+2:YPR025C;	2			
chrXVI	616962	617122	160	-1,471539962	N16:617008	22,426	+11:YPR026W;	11			
chrXVI	617750	617867	117	-1,155317306	N16:617790	18,584	+16:YPR026W;	16			
chrXVI	617888	618049	161	-1,329801815	N16:617954	26,435	+17:YPR026W;	17			
chrXVI	623817	623987	170	-1,216229375	N16:623864	10,677		Intergene			
chrXVI	627166	627316	150	-1,421535304	N16:627249	13,008	-1:YPR029C;	-1			
chrXVI	630544	630672	128	-1,018909021				Overlap <50 bp			
chrXVI	630787	630947	160	-1,186444265	N16:630889	9,311	+19:YPR030W;	19			
chrXVI	639745	639881	136	-1,555834519	N16:639847	6,156	+3:YPR034W;	3			
chrXVI	644095	644255	160	-0,99980481	N16:644162	26,136	+6:SUT829; +3:YPR036W;	6			
chrXVI	647938	648089	151	-1,519657531	N16:647982	8,548	+5:YPR040W;	5			
chrXVI	651006	651170	164	-1,36634403	N16:651112	4,243	+16:YPR042C;	16			
chrXVI	651448	651577	129	-0,900803172	N16:651501	45,675	+14:YPR042C;	14			
chrXVI	651814	651966	152	-1,252563749				Overlap <50 bp			
chrXVI	655669	655818	149	-1,206893202	N16:655729	25,381	+6:YPR045C;	6			
chrXVI	661336	661465	129	-1,165643834	N16:661468	46,144	+21:YPR049C;	21			
chrXVI	664409	664571	162	-1,377891024	N16:664522	7,057	+2:YPR049C;	2			
chrXVI	665020	665144	124	-1,248296211	N16:665145	12,656	+2:YPR051W;	2			
chrXVI	670319	670426	107	-1,464598676	N16:670368	6,758	+18:YPR055W;	18			
chrXVI	670319	670427	108	-1,433003482	N16:670368	6,758	+18:YPR055W;	18			
chrXVI	670635	670782	147	-1,117741749	N16:670711	15,362	+20:YPR055W;	20			
chrXVI	672000	672158	158	-1,342525523	N16:672118	25,399	+1:YPR057W; +9:CUT908;	1			
chrXVI	678051	678201	150	-1,228006649	N16:678147	4,535		Intergene			
chrXVI	679094	679205	111	-0,564705764	N16:679146	16,523		Intergene			
chrXVI	679094	679206	112	-0,574895319	N16:679146	16,523		Intergene			
chrXVI	679094	679207	113	-0,861216848	N16:679146	16,523		Intergene			
chrXVI	679098	679205	107	-1,144856442	N16:679146	16,523		Intergene			
chrXVI	679098	679220	122	-1,234831814	N16:679146	16,523		Intergene			
chrXVI	681181	681332	151	-1,821606949	N16:681246	15,293	+2:YPR066W; +5*:SUT833;	2			
chrXVI	685561	685687	126	-1,45776229	N16:685673	39,469	-1:YPR070W; -1:YPR069C;	-1			
chrXVI	685866	686024	158	-1,362667769	N16:685893	14,109	+1:YPR070W;	1			
chrXVI	689048	689183	135	-1,423976798	N16:689086	28,7	+7*:YPR071W;	7	TERM		
chrXVI	695989	696136	147	-1,208048559	N16:696027	10,445	+6:YPR075C;	6			
chrXVI	697045	697195	150	-1,568498098	N16:697144	20,341	+8:YPR078C; +1:CUT431;	8			
chrXVI	702075	702202	127	-1,183902945	N16:702218	18,022		Intergene			
chrXVI	706433	706576	143	-1,123242543	N16:706496	2,16	+2:CUT911; +11:YPR083W;	2			
chrXVI	719549	719656	107	-1,105596891	N16:719618	6,831	+6:YPR093C;	6			
chrXVI	719549	719694	145	-1,092523525	N16:719618	6,831	+6:YPR093C;	6			
chrXVI	721766	721909	143	-1,392070623	N16:721871	22,506	+20:YPR095C; +20:YPR096C;	20			
chrXVI	722565	722701	136	-1,454055797	N16:722569	35,379	+16:YPR095C; +16:YPR096C;	16			

CHR	START	END	L(bp)	ΔLknuc	Nuc ID	Fuzziness	Gene ID	Gene body postion or intergene	Terminal	rDNA	Telomeric
chrXVI	726383	726544	161	**-1,285546086**	N16:726563	37,57	+10:YPR097W;	10			
chrXVI	726865	727012	147	**-1,17793323**	N16:726909	24,315	+12:YPR097W;	12			
chrXVI	730093	730221	128	**-0,977833715**	N16:730129	46,934	+3*:YPR100W;	3	TERM		
chrXVI	732571	732734	163	**-1,263052247**	N16:732631	8,846	+3:YPR103W;	3			
chrXVI	733908	734067	159	**-1,356224237**	N16:733903	31,934	+18:YPR104C;	18			
chrXVI	734318	734467	149	**-1,129398388**	N16:734451	40,054	+15:YPR104C;	15			
chrXVI	735641	735790	149	**-1,482353676**	N16:735692	19,149	+7:YPR104C;	7			
chrXVI	735832	735978	146	**-1,146956939**	N16:735865	9,072	+6:YPR104C;	6			
chrXVI	736889	737030	141	**-1,06500787**	N16:736965	7,925	-1:YPR104C; +18*:YPR105C;	-1			
chrXVI	737626	737736	110	**-1,338392997**	N16:737632	27,399	+14:YPR105C;	14			
chrXVI	739761	739921	160	**-1,407490435**	N16:739810	18,995	-1:YPR106W; 0:YPR105C;	-1			
chrXVI	740972	741124	152	**-1,379536127**	N16:741035	19,352	+7:YPR106W;	7			
chrXVI	742321	742473	152	**-1,219412571**	N16:742396	8,526	+1:YPR108W; -1:YPR107C;	1			
chrXVI	745613	745766	153	**-1,582380352**	N16:745699	19,502	+8*:YPR110C;	8	TERM		
chrXVI	747443	747595	152	**-1,578364658**	N16:747452	9,854	+2:YPR111W;	2			
chrXVI	748166	748325	159	**-1,686567836**	N16:748291	22,076	+7:YPR111W;	7			
chrXVI	751513	751665	152	**-1,390822849**	N16:751585	9,988	+3:YPR112C;	3			
chrXVI	753264	753415	151	**-1,492754888**	N16:753263	10,895	-1:CUT912; +3:YPR114W;	-1			
chrXVI	753264	753415	151	**-1,492754888**	N16:753426	8,701	+4:YPR114W;	4			
chrXVI	753454	753608	154	**-1,200298861**	N16:753612	19,753	+5:YPR114W;	5			
chrXVI	759400	759547	147	**-1,225971026**	N16:759472	10,284	0:CUT913; +6:YPR116W;	0			
chrXVI	761447	761589	142	**-1,126235389**	N16:761432	27,999	+17:YPR116W; +9:YPR117W;	17			
chrXVI	761447	761589	142	**-1,126235389**	N16:761585	22,142	+18:YPR116W; +10:YPR117W;	18			
chrXVI	763063	763215	152	**-1,217602308**	N16:763092	14,986	+27:YPR116W; +19:YPR117W;	27			
chrXVI	763430	763578	148	**-1,276877455**	N16:763564	27,998	+30:YPR116W; +22:YPR117W;	30			
chrXVI	764397	764544	147	**-1,535365453**	N16:764439	30,391	+35:YPR116W; +27:YPR117W;	35			
chrXVI	764502	764659	157	**-1,606737048**	N16:764652	30,882	+36:YPR116W; +28:YPR117W;	36			
chrXVI	770323	770455	132	**-1,007757005**	N16:770351	5,357	-1:Unit663; +3:SUT834;	-1			
chrXVI	778559	778687	128	**-1,695727508**	N16:778565	9,397	+1:YPR121W;	1			
chrXVI	786310	786475	165	**-1,358178432**				Overlap <50 bp			
chrXVI	788541	788654	113	**-0,957428674**				Overlap <50 bp			
chrXVI	788648	788776	128	**-0,912116698**	N16:788711	27,346	+6:YPR125W;	6			
chrXVI	790762	790920	158	**-1,717619091**	N16:790928	22,465	-1:SUT839; +6:YPR127W;	-1			
chrXVI	791267	791414	147	**-1,435177715**	N16:791367	20,597	+6:YPR128C;	6			
chrXVI	791438	791595	157	**-1,174189669**	N16:791540	15,057	+5:YPR128C;	5			
chrXVI	791737	791890	153	**-1,234414832**	N16:791867	11,384	+3:YPR128C;	3			
chrXVI	792774	792924	150	**-1,406549472**	N16:792865	9,535	+2:YPR129W;	2			
chrXVI	794619	794743	124	**-1,179240655**	N16:794744	0	-1:YPR132W; -1:YPR131C;	-1			
chrXVI	796842	796998	156	**-1,258376936**	N16:796924	5,167	+3:YPR133C;	3			
chrXVI	798394	798554	160	**-1,186659544**	N16:798384	26,696	+5:SUT840; +3:YPR134W;	5			
chrXVI	798628	798777	149	**-1,263993757**	N16:798655	37,61	+3:SUT840; +5:YPR134W;	3			
chrXVI	805070	805217	147	**-1,188331633**	N16:805107	12,021		Intergene			
chrXVI	805577	805691	114	**-1,420834688**				Overlap <50 bp			
chrXVI	807669	807826	157	**-1,466924315**	N16:807711	23,065		Intergene			
chrXVI	808317	808452	135	**-1,131987236**	N16:808366	33,843		Intergene			
chrXVI	809355	809495	140	**-1,2188234**	N16:809434	35,921		Intergene			
chrXVI	809361	809495	134	**-1,013927624**	N16:809434	35,921		Intergene			
chrXVI	809773	809928	155	**-1,417064852**	N16:809806	26,539		Intergene			
chrXVI	809904	810012	108	**-0,680220808**	N16:809957	38,136		Intergene			
chrXVI	809934	810052	118	**-0,984914722**	N16:809957	38,136		Intergene			
chrXVI	810053	810202	149	**-1,382210704**	N16:810107	16,971		Intergene			
chrXVI	813679	813806	127	**-0,952728752**	N16:813742	6,369	+4*:SUT424; +3:YPR139C;	4	TERM		
chrXVI	822438	822593	155	**-1,477588491**				Overlap <50 bp			
chrXVI	822870	822974	104	**-1,398798499**	N16:822924	21,198	+3:YPR145W;	3			
chrXVI	827045	827170	125	**-1,016009506**	N16:827139	12,522	+7:YPR148C;	7			
chrXVI	827247	827389	142	**-1,595691954**	N16:827298	19,325	+6:YPR148C;	6			
chrXVI	827869	828021	152	**-1,40915842**	N16:827957	7,57	+2:YPR148C;	2			
chrXVI	830012	830156	144	**-1,106999868**	N16:830081	24,766	+3:YPR149W;	3			
chrXVI	839224	839390	166	**-1,47755392**	N16:839304	25,12	+6*:YPR156C;	6	TERM		
chrXVI	841656	841805	149	**-1,20401662**				Overlap <50 bp			
chrXVI	843827	843986	159	**-1,393427659**	N16:843902	35,884	+5*:YPR158W;	5	TERM		
chrXVI	844949	845072	123	**-1,249444799**				Overlap <50 bp			
chrXVI	845049	845204	155	**-1,080475931**	N16:845158	30,73		Intergene			
chrXVI	845482	845616	134	**-1,179930386**	N16:845543	30,172		Intergene			
chrXVI	845482	845622	140	**-1,300049783**	N16:845543	30,172		Intergene			
chrXVI	846525	846660	135	**-1,116134927**	N16:846560	5,033		Intergene			
chrXVI	847154	847311	157	**-1,625023224**	N16:847244	32,604		Intergene			
chrXVI	848557	848707	150	**-1,459466244**	N16:848653	13,503		Intergene			
chrXVI	849289	849403	114	**-1,350078132**	N16:849394	31,565		Intergene			
chrXVI	851012	851164	152	**-1,500339674**	N16:851100	18,009		Intergene			
chrXVI	851565	851679	114	**-1,25898221**	N16:851699	5,132		Intergene			
chrXVI	853843	853993	150	**-1,065574828**	N16:853920	16,258		Intergene			
chrXVI	853847	853993	146	**-1,075847853**	N16:853920	16,258		Intergene			
chrXVI	854031	854169	138	**-1,392142968**	N16:854079	0		Intergene			
chrXVI	854126	854258	132	**-1,09774969**	N16:854239	38,891		Intergene			
chrXVI	854305	854440	135	**-1,292205428**	N16:854397	22,517		Intergene			
chrXVI	854851	855005	154	**-1,191042789**	N16:854861	22,03		Intergene			
chrXVI	855108	855259	151	**-1,585915168**	N16:855198	12,055		Intergene			
chrXVI	855132	855277	145	**-1,142853858**	N16:855198	12,055		Intergene			
chrXVI	855136	855287	151	**-1,295370974**	N16:855198	12,055		Intergene			
chrXVI	855343	855483	140	**-0,944469758**	N16:855418	34,962		Intergene			
chrXVI	855349	855483	134	**-1,109465002**	N16:855418	34,962		Intergene			
chrXVI	855892	856000	108	**-0,717180625**	N16:855993	49,602		Intergene			
chrXVI	856112	856214	102	**-1,450300807**				Overlap <50 bp			
chrXVI	858667	858810	143	**-1,409500174**	N16:858736	38,96	+10:YPR159W;	10			
chrXVI	863879	864033	154	**-1,055299585**	N16:864033	22,755	+17*:YPR160W;	17	TERM		
chrXVI	863879	864033	154	**-1,055299585**	N16:863878	41,094	+16:YPR160W;	16			
chrXVI	864255	864385	130	**-1,252558504**	N16:864244	23,845		Intergene			
chrXVI	867145	867288	143	**-1,194733019**				Overlap <50 bp			
chrXVI	868632	868780	148	**-1,180799648**	N16:868711	11,606	+9*:YPR163C;	9	TERM		
chrXVI	888008	888137	129	**-1,046012432**	N16:888074	10,323	:YPR174C; 0:SUT427; -1:YPR173C;	5	TERM		
chrXVI	891517	891679	162	**-1,236792228**	N16:891562	6,501	+4:YPR176C;	4			
chrXVI	893449	893599	150	**-1,394754161**	N16:893574	34,979	+8:YPR178W;	8			
chrXVI	897488	897648	160	**-1,611825204**	N16:897619	22,599	+14:YPR181C;	14			
chrXVI	897990	898142	152	**-1,156239421**	N16:898110	26,338	+11:YPR181C;	11			
chrXVI	901200	901349	149	**-1,256425104**	N16:901238	6,397	+4:YPR183W; +3:CUT923;	4			

CHR	START	END	L(bp)	ΔLknuc	Nuc ID	Fuzziness	Gene ID	Gene body postion or intergene	Terminal	rDNA	Telomeric
chrXVI	905702	905842	140	**-1,336380245**	N16:905808	23,973		Intergene			
chrXVI	910317	910457	140	**-1,154874767**	N16:910477	3,017	+4:YPR186C;	4			
chrXVI	910317	910457	140	**-1,154874767**	N16:910305	13,424	+5:YPR186C;	5			
chrXVI	922519	922676	157	**-1,564045849**	N16:922581	2,828	+2:anti192; +5:YPR192W;	2			
chrXVI	922519	922681	162	**-1,387495318**	N16:922581	2,828	+2:anti192; +5:YPR192W;	2			
chrXVI	922523	922681	158	**-1,503272644**	N16:922581	2,828	+2:anti192; +5:YPR192W;	2			
chrXVI	927439	927574	135	**-0,987824892**	N16:927563	36,449	0:SUT429;	0			
chrXVI	930833	931008	175	**-1,746019507**	N16:930869	24,595	+3:SUT846; +14:SUT430;	3			
chrXVI	931493	931642	149	**-1,421005016**	N16:931566	13,692	+2:YPR196W;	2			
chrXVI	940363	940515	152	**-1,281479838**	N16:940386	7,036	+4:YPR201W;	4			
chrXVI	943076	943229	153	**-1,498434548**	N16:943200	29,905	+2:YPR202W;	2			TEL RIGHT
chrXVI	943085	943229	144	**-1,833880799**	N16:943200	29,905	+2:YPR202W;	2			TEL RIGHT
chrXVI	944252	944389	137	**-1,304987783**	N16:944314	4,041	-1:YPR204W; +4*:YPR203W;	-1			TEL RIGHT
chrXVI	944537	944680	143	**-1,382609864**	N16:944589	14	+1:YPR204W;	1			TEL RIGHT
chrXVI	944585	944710	125	**-0,788288258**	N16:944589	14	+1:YPR204W;	1			TEL RIGHT
chrXVI	944810	944962	152	**-1,212944731**	N16:944922	11,533	+3:YPR204W;	3			TEL RIGHT
chrXVI	944883	945027	144	**-1,518440935**	N16:944922	11,533	+3:YPR204W;	3			TEL RIGHT
chrXVI	944980	945115	135	**-1,063325043**	N16:945080	21,502	+4:YPR204W;	4			TEL RIGHT
chrXVI	944980	945167	187	**-1,122414218**	N16:945080	21,502	+4:YPR204W;	4			TEL RIGHT
chrXVI	945023	945115	92	**-0,294290005**	N16:945080	21,502	+4:YPR204W;	4			TEL RIGHT
chrXVI	945023	945167	144	**-0,955039769**	N16:945080	21,502	+4:YPR204W;	4			TEL RIGHT
chrXVI	945362	945514	152	**-1,226994054**	N16:945422	14,364	+6:YPR204W;	6			TEL RIGHT
chrXVI	945663	945793	130	**-1,056492367**	N16:945658	23,335	+7:YPR204W;	7			TEL RIGHT
chrXVI	945663	945793	130	**-1,056492367**	N16:945816	41,102	+8:YPR204W;	8			TEL RIGHT
chrXVI	945759	945954	195	**-1,628973299**	N16:945816	41,102	+8:YPR204W;	8			TEL RIGHT
chrXVI	945821	945954	133	**-0,943887076**	N16:945816	41,102	+8:YPR204W;	8			TEL RIGHT
chrXVI	945821	945969	148	**-1,34134048**	N16:945816	41,102	+8:YPR204W;	8			TEL RIGHT
chrXVI	946033	946184	151	**-1,163093755**	N16:946078	43,134	+10:YPR204W;	10			TEL RIGHT
chrXVI	946038	946172	134	**-1,029552381**	N16:946078	43,134	+10:YPR204W;	10			TEL RIGHT
chrXVI	946051	946147	96	**-0,855605136**	N16:946078	43,134	+10:YPR204W;	10			TEL RIGHT
chrXVI	946159	946281	122	**-0,182479278**				Overlap <50 bp			TEL RIGHT
chrXVI	946966	947118	152	**-1,155297851**	N16:947053	20,506	+16:YPR204W;	16			TEL RIGHT
chrXVI	947306	947457	151	**-1,220736008**	N16:947384	3,536	+18:YPR204W;	18			TEL RIGHT
